# An *In Silico* Infrared Spectral Library of Molecular Ions for Metabolite Identification

**DOI:** 10.1021/acs.analchem.3c01078

**Published:** 2023-06-01

**Authors:** Kas J. Houthuijs, Giel Berden, Udo F. H. Engelke, Vasuk Gautam, David S. Wishart, Ron A. Wevers, Jonathan Martens, Jos Oomens

**Affiliations:** † Institute for Molecules and Materials, FELIX Laboratory, Radboud University, Nijmegen 6525 ED, The Netherlands; ‡ Department of Genetics, Translational Metabolic Laboratory, Radboud University Medical Center, Nijmegen 6525 GA, The Netherlands; § Department of Biological Sciences, University of Alberta, Edmonton AB T6G 2E9, Canada; ∥ Department of Computing Science, University of Alberta, Edmonton, AB T6G 2E8, Canada; ⊥ Department of Laboratory Medicine and Pathology, University of Alberta, Edmonton, AB T6G 2B7, Canada; # Faculty of Pharmacy and Pharmaceutical Sciences, University of Alberta, Edmonton, AB T6G 2H7, Canada; g van ’t Hoff Institute for Molecular Sciences, University of Amsterdam, Amsterdam 1098 XH, The Netherlands

## Abstract

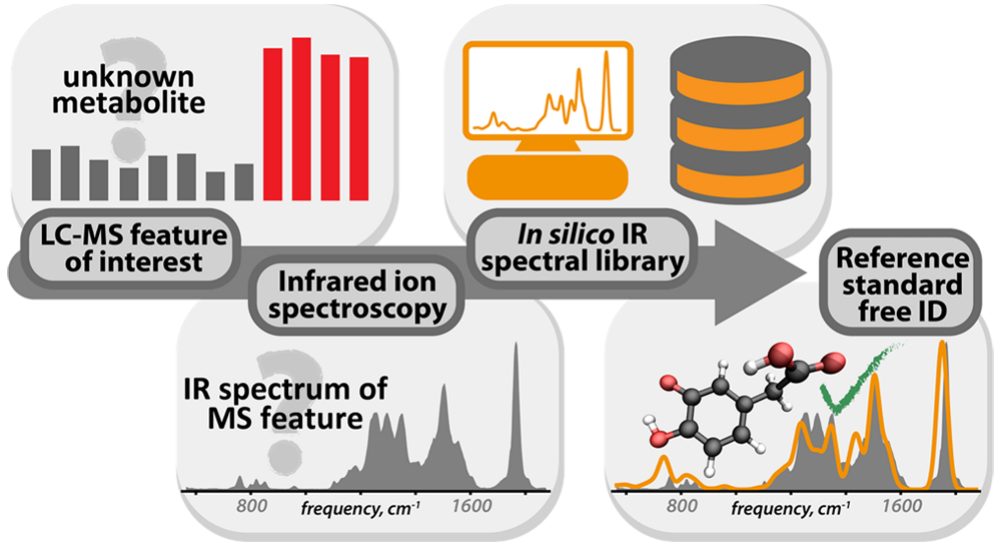

Infrared ion spectroscopy (IRIS) continues to see increasing use as an analytical tool for small-molecule identification in conjunction with mass spectrometry (MS). The IR spectrum of an *m*/*z* selected population of ions constitutes a unique fingerprint that is specific to the molecular structure. However, direct translation of an IR spectrum to a molecular structure remains challenging, as reference libraries of IR spectra of molecular ions largely do not exist. Quantum-chemically computed spectra can reliably be used as reference, but the challenge of selecting the candidate structures remains. Here, we introduce an *in silico* library of vibrational spectra of common MS adducts of over 4500 compounds found in the human metabolome database. In total, the library currently contains more than 75,000 spectra computed at the DFT level that can be queried with an experimental IR spectrum. Moreover, we introduce a database of 189 experimental IRIS spectra, which is employed to validate the automated spectral matching routines. This demonstrates that 75% of the metabolites in the experimental data set are correctly identified, based solely on their exact *m*/*z* and IRIS spectrum. Additionally, we demonstrate an approach for specifically identifying substructures by performing a search without *m*/*z* constraints to find structural analogues. Such an unsupervised search paves the way toward the *de novo* identification of unknowns that are absent in spectral libraries. We apply the *in silico* spectral library to identify an unknown in a plasma sample as 3-hydroxyhexanoic acid, highlighting the potential of the method.

## Introduction

Mass spectrometry (MS) is the primary analytical approach in untargeted metabolomics due to its high sensitivity, selectivity, and throughput. Routine analyses of complex biological samples yield large numbers of detected features—numbers that are unrivalled by other analytical techniques.^1^ Subsequent (biological) interpretation requires the precise molecular identification of the *m*/*z* features of interest.^2^ Herein lies the major challenge of MS-based metabolomics, as little structural information is contained in an *m*/*z* value alone. To distinguish between structural isomers, MS is often hyphenated with gas or liquid chromatography (GC or LC), tandem MS (MS/MS), or ion mobility spectrometry (IMS).^3,4^ Annotation is then achieved by comparison against libraries with reference MS/MS spectra,^5,6^ retention times, or CCS values.^6,7^ Yet, despite the many reference libraries, detailed molecular identification remains the main bottleneck in MS-based metabolomics.

Infrared ion spectroscopy (IRIS) provides an alternative approach to molecular structure determination in MS. IRIS measures the IR spectrum of an *m*/*z*-selected population of ions in the MS instrument, providing detailed structural information.^8^ Being rooted in MS, IRIS can be appended to LC-MS workflows,^9,10^ which has enabled its analytical implementation in, for instance, the identification of biomarkers for metabolic diseases^11−13^ and other small-molecule isomerism questions.^14−16^ Spectrum-to-structure translation is usually performed by formulating (a small number of) candidate structures based on prior knowledge of the underlying (bio)chemistry. Structural confirmation of these candidates is then achieved through comparison of the experimental IRIS spectrum to a reference spectrum from a physical standard and/or from a quantum-chemical calculation.^17^ The latter enables identification without physical reference standards. Density functional theory (DFT) calculations typically give accurate predictions of a compound’s vibrational spectrum and have been employed in numerous fundamental ion chemistry studies utilizing IRIS.^18−20^ DFT-predictions are therefore useful for (preliminary) metabolite identification and can at least reduce the number of candidate structures substantially.

This approach to spectrum-to-structure conversion is efficient if detailed prior knowledge on the detected feature is available, especially if the candidate structures are well defined and limited in number. However, such information is often not available or not sufficiently detailed. Ideally, one would use a large IRIS spectral library, similar to those available for MS/MS spectra, to facilitate quick and high-throughput screening. Large IR spectral libraries are available for neutral (gaseous) molecules, but their ions formed in MS (e.g., [M+H]^+^, [M–H]^−^, [M+Na]^+^) possess drastically different IR spectra.^21^ An experimental IRIS library sufficiently large for identification purposes is currently unavailable (although efforts are underway to compile small libraries for specific compound classes, such as glycans^22,23^). Moreover, in light of the vastness of small-molecule chemical space,^24,25^ experimental reference libraries are intrinsically limited, as is also the case for MS/MS and IMS data. Therefore, the focus in the metabolomics community has shifted toward expanding MS/MS and IMS libraries with computational data.^5,26−28^ In addition to their cost effectiveness, *in silico* libraries have the advantage of a greatly increased coverage, since only a small fraction of molecules are available as physical reference standards.

Using quantum-chemical methods, vibrational spectra can be routinely and accurately calculated (somewhat in contrast to MS/MS spectra). For example, Karunaratne et al. pursued identification of neutral gaseous molecules detected with GC-FTIR spectroscopy by employing DFT-calculated reference spectra of compounds extracted from the PubChem database.^29^ This approach yields identification rates comparable to those achieved with MS/MS. However, GC-FTIR is not compatible with typical sample matrices in metabolomics, where LC-MS is the method of choice.

Here, we present an *in silico* IR spectral library of molecular ions, compatible with IRIS and hence LC-MS workflows. This library was compiled by coupling a standardized workflow for calculating vibrational spectra at the DFT level to the human metabolome database (HMDB).^6^ This workflow requires only a simple chemical identifier as input to automatically generate relevant IR spectra and was applied to HMDB entries with molecular weights <210 Da. We present benchmark tests of this *in silico* library using a new, extensive set of 189 experimental IRIS spectra, derived from 87 unique metabolites. We demonstrate that the specificity of the IR fingerprint not only allows for the identification of library metabolites, but also enables *de novo* structural elucidation of unknowns not contained in our library.

## Methods

Experimental and computational methods are briefly described here; further details are provided in the Supporting Information.

### Infrared Ion Spectroscopy

Experiments were performed in an electrospray ionization (ESI) ion trap mass spectrometer coupled to the tunable IR laser FELIX.^30^ Solutions of reference standards were directly infused and protonated ([M+H]^+^) or deprotonated ([M–H]^−^), and sodiated ([M+Na]^+^) ions, generically referred to as “adducts”, were mass isolated. IRIS spectra were obtained by irradiating the *m*/*z*-selected ions with FELIX, monitoring the IR frequency-dependent multiple-photon dissociation (IRMPD) of the precursor ion. Although, the IRMPD yield depends nonlinearly on laser pulse energy, a first-order linear correction is applied to account for frequency-dependent power variations.^31^

### Computational Workflow

The automated workflow to compute vibrational spectra for HMDB entries derives from methods described in ref (17). As further detailed in the SI, protonated, deprotonated, and sodiated ions were constructed by considering all nitrogen, oxygen, and sulfur atoms as sites for H^+^/Na^+^ addition/removal.^32^ After a conformational search, ion geometries are optimized at the PM6 level. Unfavorable geometries were filtered by their relative energies (>40 kJ mol^–1^). Remaining structures are reoptimized using B3LYP/6-311+G(d,p), followed by a frequency calculation. Accurate electronic energies are calculated at the MP2/6-311+G(d,p) level using the B3LYP geometry and combined with the B3LYP thermal energy. Calculated frequencies are used to generate reference IR spectra and populate the spectral library. Harmonic frequencies are scaled by a factor of 0.975, and the stick spectrum is convolved with a Gaussian profile of 45 cm^–1^ full width at half-maximum (fwhm).

### Scoring of Spectral Similarity

A search of the library with an experimental IR spectrum retrieves computed IR spectra sorted by their spectral similarity *S*_*spec*_, based on the cosine similarity score.^17^ Several similarity metrics—including one corresponding to the square of the cosine similarity—were previously evaluated, demonstrating similar performance.^33^ Earlier studies used a log transformation of the normalized spectral intensities to make *S*_*spec*_ less sensitive to intensity deviations, which are common in IRMPD-based IRIS, and hence more sensitive to frequency overlap.^17^ A similar effect is achieved by taking the square root,^34^ which was found to give optimal retrieval of correct spectra (Table S1). The absolute *S*_*spec*_ values should be interpreted with care since the scoring is solely optimized on the retrieval rate.

## Results and Discussion

### Library and Validation Set

IR spectra were calculated for the protonated ([M+H]^+^), deprotonated ([M–H]^−^), and sodiated ([M+Na]^+^) ions of all entries in HMDB 4.0 with a molecular weight lower than 210 Da.^35^ This amounts to a total of 11,823 ions generated from 4640 metabolites. Since several tautomers and conformers are included for each ion, the library contains a total of 75,941 computed IR spectra. The experimental validation set consists of 12 IRIS spectra reported previously^8,14,17,36^ and 177 newly measured IRIS spectra derived from 87 metabolites (Table S3 and Scheme S1). Chemical class information for the library and validation set as categorized by ClassyFire^37^ (Figure S2) indicates that all major classes in the library are represented by at least six reference metabolites; both the library and the validation set cover similar distributions in mass and adduct type (Figure S3). Experimental and computational IR spectra are available through the HMDB website (https://hmdb.ca) and are interactively viewable (via JSpectraView) in the “spectrum” field of the *MetaboCard* of the corresponding metabolite.^38^

### Identifying 3,4-Dihydroxyphenylacetic Acid

**Figure 1:**
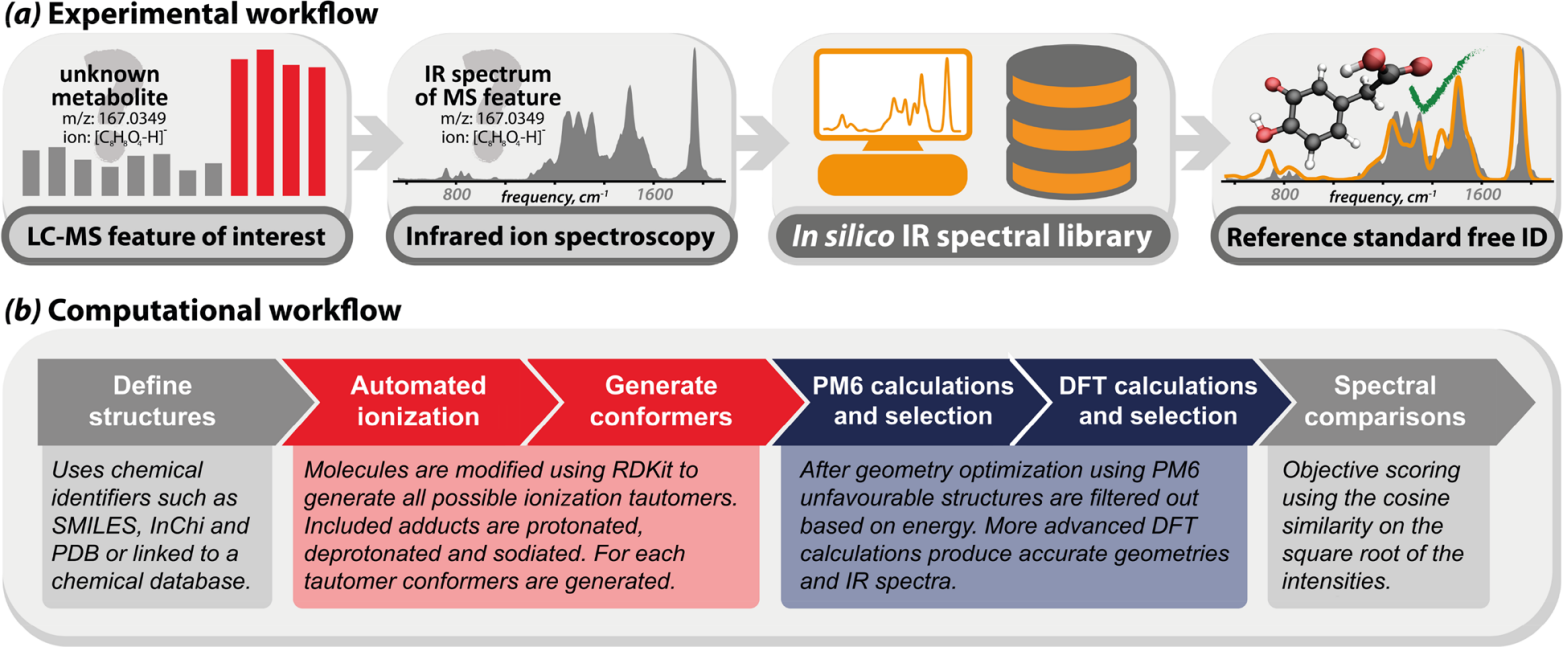
(a) Experimental workflow for reference standard free metabolite identification. A metabolite of unknown structure, encountered in an untargeted LC-MS screening, is characterized by its IRIS spectrum, which is compared against computed IR spectra in the library. The ion is annotated with the structure of the best matching library spectrum. (b) The computational workflow used to populate the in silico IR spectra library.

Figure 1 presents a schematic overview of the proposed experimental and computational workflows, where an LC-MS feature with known accurate mass but unknown molecular structure is characterized by recording its IRIS spectrum. This spectrum is compared to computed spectra of candidate isomers in the *in silico* library of IR ion spectra. The unknown was annotated with the structure that provides the best IR spectral match in terms of the cosine similarity. As a proof-of-concept method, we demonstrate these workflows using the IRIS spectrum of deprotonated 3,4-dihydroxyphenylacetic acid (DOPAC), a metabolite of dopamine. As previously shown, DOPAC is spectroscopically distinguishable from its structural isomer homogentisic acid (HGA), a biomarker for the genetic disorder alkaptonuria.^8^ However, the HMDB contains a total of 18 metabolites that share the elemental composition of DOPAC (C_8_H_8_O_4_), 16 of which can be deprotonated; their computed IR spectra are present in the *in silico* IR spectral library.

**Figure 2:**
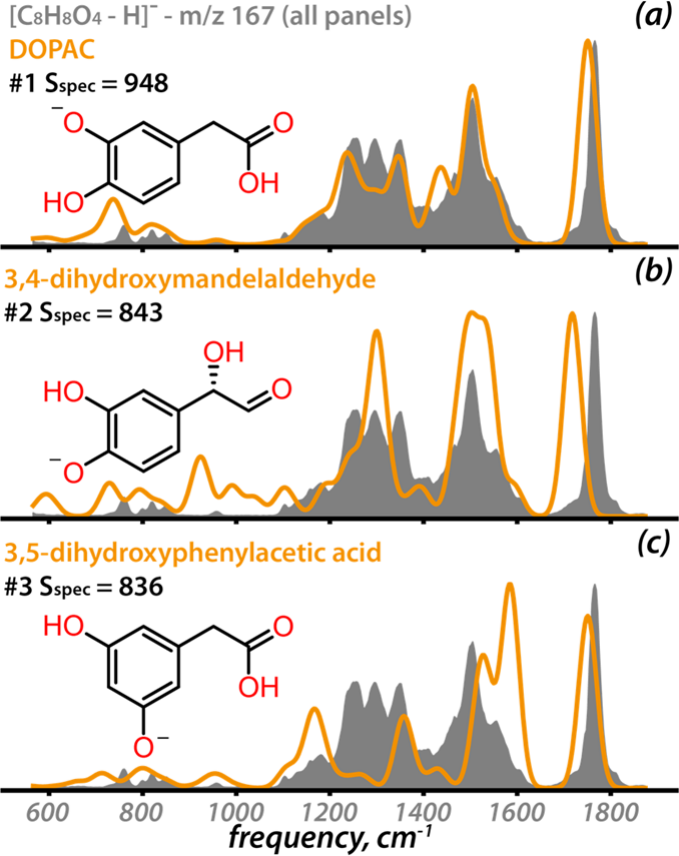
(a–c) The three computed vibrational spectra (orange) for [C_8_H_8_O_4_–H]^−^ ions in the library giving the best spectral match (S_spec_) to the experimental spectrum of [DOPAC–H]^−^ (gray).

Figure 2 shows the IR spectrum of [DOPAC–H]^−^ along with the three best matching spectra in the *in silico* library and their corresponding strucures (see Results and Discussion for all 16 isomers in Figure S4). The computed spectrum of [DOPAC–H]^−^ indeed returns the highest spectral similarity. Interestingly, metabolites that are structurally similar to DOPAC give the second and third highest scores, 3,4-dihydroxymandelaldehyde and 3,5-dihydroxyphenylacetic acid, respectively. These metabolites are the only 1,2-positional isomers of DOPAC in the set of 16 isomers (Figure S4) and have the highest structural similarity with DOPAC (0.56 and 0.78, see details about structural similarity in the SI).

The use of *in silico* IR spectra as a reference enables us to attribute normal mode vibrations of specific functional groups to each band in the spectrum. Spectral mismatches can thus be correlated with structural mismatches. For example, in Figure 2b, the computed bands in the 900–1050 cm^–1^ range originate from CH/OH bending vibrations of the aldehyde and the α-hydroxide. Likewise, the band at 1600 cm^–1^ in Figure 2c corresponds to the C–O^–^ stretch of the phenoxide. In cases where the actual metabolite is not included in the library, this may suggest new structures based on correctly and incorrectly matching substructure(s). Computing IR spectra for these newly conceived structures is then a route toward *de novo* structure identification of compounds that are not in the library.

### Unsupervised Searches for Structural analogues

The example in Figure 2 demonstrates the potential of IRIS-based identification of library metabolites, based on the inherent sensitivity of an IR spectrum to chemical structure. Not only is the correct metabolite ranked best, but the search also assigns high scores to structural analogues. This inherent link between structure and spectroscopy provides venues toward identification of compounds outside the library. Searching the entire library without constraint on chemical formula (i.e., on exact mass) should assign high spectral similarity scores to compounds with high structural similarity to the unknown, even if the elemental composition is not the same.

**Figure 3:**
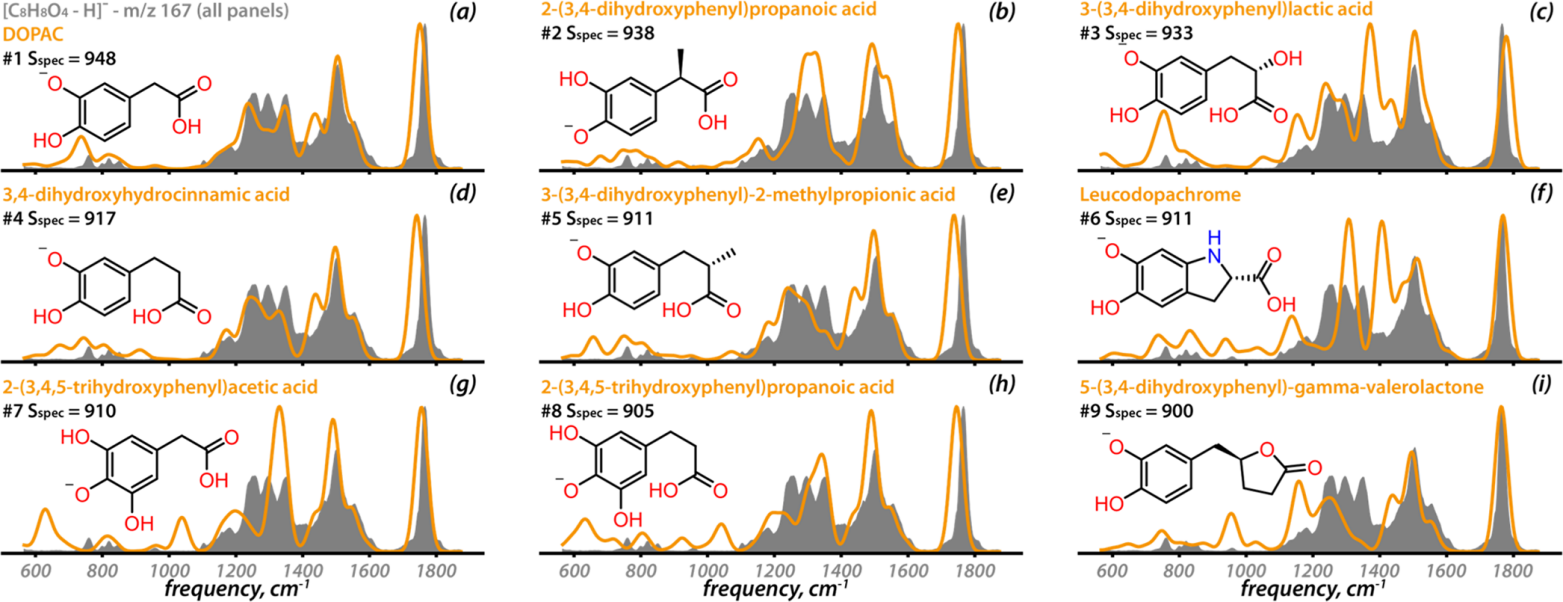
(a–i) The nine best matching computed vibrational spectra (orange) in the DFT library with the experimental IR spectrum of [DOPAC–H]^−^ (gray), ranked by S_spec_. No *m*/*z* or molecular formula constraint was imposed (unsupervised).

For this proof-of-concept method, the experimental IRIS spectrum of [DOPAC–H]^−^ is again taken as the hypothetical unknown. Figure 3 presents the nine highest-ranked entries upon matching this IRIS spectrum against the entire library, without constraint on chemical formula (but retaining the adduct constraint). The fact that DOPAC itself is ranked #1 out of 2707 deprotonated entries demonstrates both the uniqueness of the experimental IR spectrum, as well as its accurate prediction by DFT calculations. Inspecting the compounds ranked #2–#9 reveals the potential for *de novo* structural elucidation via this unsupervised search: the top-9 metabolites are close structural analogues of DOPAC, differing mostly in the length of the alkyl chain, addition of a methyl or hydroxyl group, or a combination thereof. The strong correlation between structural and spectral similarity is quantified for all 2707 deprotonated entries in Figure S5 and forms the basis for this identification strategy as well as for recently developed scoring approaches in metabolite identification by tandem MS.^39^

### General Performance

**Figure 4:**
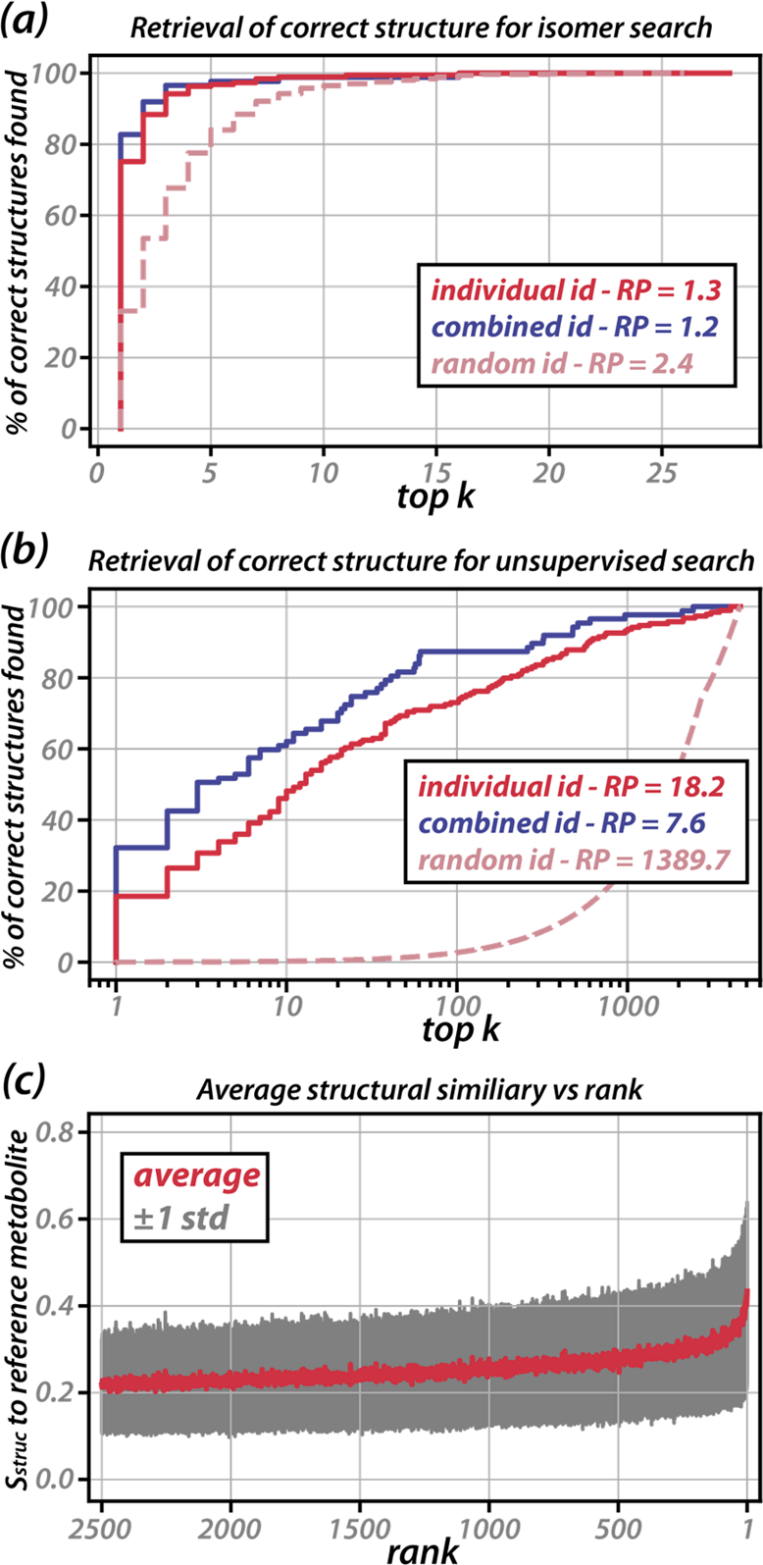
Percentage of correct structures found in the top k hits when performing an isomer search (a) or an unsupervised search (b). Results for individual adducts are in red (*N* = 189) and for adducts combined in purple (*N* = 87); random annotation results are indicated with the dashed line. Panel (c) shows the average structural similarity to the correct metabolite per rank after an unsupervised search.

To verify how the results for [DOPAC–H]^−^ generalize, a validation set of 189 experimental IRIS spectra was subjected to spectral matching against the *in silico* IR library spectra, analogous to the analysis for DOPAC. The ranking of each IR spectrum match as well as spectral plots similar to those for DOPAC are available in the Supporting Information. Figure 4a shows how well the metabolites are identified based on their IRIS spectrum and exact *m*/*z*, plotting the percentage of correctly identified metabolites in the top *k* for increasing *k*. A total of 142 (75%) correct metabolite identifications is achieved when a single experimental IR spectrum is used (red solid trace); 97% of the metabolites are among the top-5 ranked structures. Annotating the experimental IR spectrum to a random isomeric entry from the spectral library correctly identifies 33% of the metabolites (dashed trace), which is relatively high due to entries in the database with only one or a few isomeric structures.

The overall performance can be captured in a single number by taking the geometric mean of the rank of each of the 189 reference metabolites.^40^ This rank product (RP, details in the SI) is equal to 1.3 here. When the IR spectra from different adducts ([M+H]^+^, [M–H]^−^, [M+Na]^+^) of the same metabolite are combined (blue solid trace) by ranking on the product of individual spectral similarity scores, the correct identification rate increases to 83% (72 out of 87 metabolites at rank #1) and the RP improves to 1.2. Overall, this performance is higher than typical retrieval rates when employing tandem MS libraries, which typically are 45%–70%,^41^ although true comparison is hampered by the much smaller validation set used here. What the present results do clearly demonstrate is the general applicability of the *in silico* IR spectral library in identifying compounds across a large data set.

A similar plot can be constructed to assess how well metabolites are retrieved in an unsupervised search, i.e., releasing the *m*/*z* constraint (Figure 4b) and matching the IRIS spectrum against all adduct-constrained computed spectra in the IR library. In this case, 19% of the identifications are correct, while 48% are scored in the top 10. The specificity improves significantly when IR spectra of different adducts ([M+H]^+^, [M–H]^−^, [M+Na]^+^) are combined, boosting the top 1 and top 10 percentages to 32% and 62%, respectively. The RPs follow a similar trend, improving from 18.2 to 7.6. For the unsupervised search, the contrast with randomly annotating the experimental spectra is pronounced, with just 0.03% correct identification, as the number of adduct-constrained candidates increases to 3892 on average.

The effectiveness of combining spectra of different adducts is best illustrated with acetylglycine (HMDB0000532), for which the individual adducts rank 10th ([M–H]^−^), 20th ([M+H]^+^), and sixth ([M+Na]^+^) in an unsupervised search, but combined they rank first (Table S4). Decomposition of Figure 4b into the individual adducts shows that the [M+H]^+^ adducts are retrieved best from the library, followed by [M+Na]^+^ and [M–H]^−^ (RPs are 12.4, 15.8 and 30.0, respectively). A discussion of this trend is presented in the SI along with Figure S6.

The correlation between spectral similarity and structural similarity for the whole validation set is visualized by plotting the average structural similarity between each reference metabolite and the ranked library structures, where the reference metabolites themselves are removed from the data (Figure 4c). On average, a clear increase in structural similarity is observed when approaching rank 1, with a steep increase for the ∼50 best matches. Inspection of the individual structure vs spectral similarity plots (see SI) suggests that the correlation between structural similarity and spectral similarity for some metabolites is limited by the number of structural analogues present in the library (e.g., Figure S7). An expansion of the library to include more metabolites would therefore not only allow for the direct identification of more metabolites, but also make unsupervised searches more sensitive to substructure, as more structural analogues would be included.

### Higher-Energy Geometries

To assess where the scoring can be further improved, we manually inspected the computed geometries and vibrational spectra for each reference metabolite and compared these with the experimental IR spectra. It appears that about 10% of ions adopt a higher-energy conformational or tautomeric geometry, with a significantly different computed IR spectrum. These higher-energy geometries lie up to 68.8 kJ mol^–1^ above the lowest-energy geometry (Figures S8 and S9). The presence of higher-energy geometries is not uncommon and may be due to inaccuracies in the calculated energies, incomplete conformer ensembles, or kinetic trapping of solution-phase conformers or tautomers that are transferred to the gas phase.^42^

To account for these higher-energy geometries, the spectral library can be searched with an energy tolerance, where the ranking is based on the best matching computed spectrum per entry. For a tolerance of 10 kJ mol^–1^, this results in an overall better performance as derived from the RP, which improves from 18.2 to 17.0 in the unsupervised search. However, the 10 kJ mol^–1^ tolerance increases the number of considered spectra about 3-fold (to 10811 on average), possibly leading to a worse ranking for metabolites that already performed well. This effect becomes pronounced when all adduct-constrained spectra in the *in silico* library are searched, i.e., without energy constraints (24,865 spectra on average, Table S4), yielding a poorer RP of 19.0.

For some ions, a mix of conformers or tautomers may be present, each with distinct IR spectra (Figures S10 and S11), which may be addressed by optimizing linear combinations of computed vibrational spectra.^43^ This is not pursued here, as it would severely slow down the spectral matching and likely give poorer performance for metabolites that exhibit a “pure” population. The benefits are likely small as mixtures are not frequently observed (<15% based on manual inspection) and minimally affect the IR spectrum when the contribution of the minor population is small (Figure S12). Methods that experimentally deconvolute the mixtures, e.g., by separating conformers or tautomers with ion mobility spectrometry^23,42^ or by employing two-color laser experiments,^44^ could improve their identification analogous to using spectra of multiple adducts.

### Broader Spectra

Severe spectral broadening observed in some IRIS spectra can limit the performance of our method. Extensive broadening is often caused by strong ionic hydrogen bonds that induce shared-proton motifs.^45,46^ Such problematic spectra may be avoided by selecting different adducts. For instance, for l-aspartic acid (HMDB0000191), the [M–H]^−^ ion shows severe broadening,^47^ while both [M+H]^+^ and [M+Na]^+^ ions do not (Figure S13).^48^ We also note that spectral broadening in such systems is often reduced on cryogenic tandem mass spectrometry platforms.^15,49−51^ Alternatively, IRIS identification may be applied to MS/MS fragments, which typically exhibit better resolved spectra,^17,36^ in combination with a bottom-up approach for structure elucidation. This also extends the applicability of the IR spectral library toward molecules beyond its upper mass limit.^22,23^

### Identification from a Human Plasma Sample

**Figure 5:**
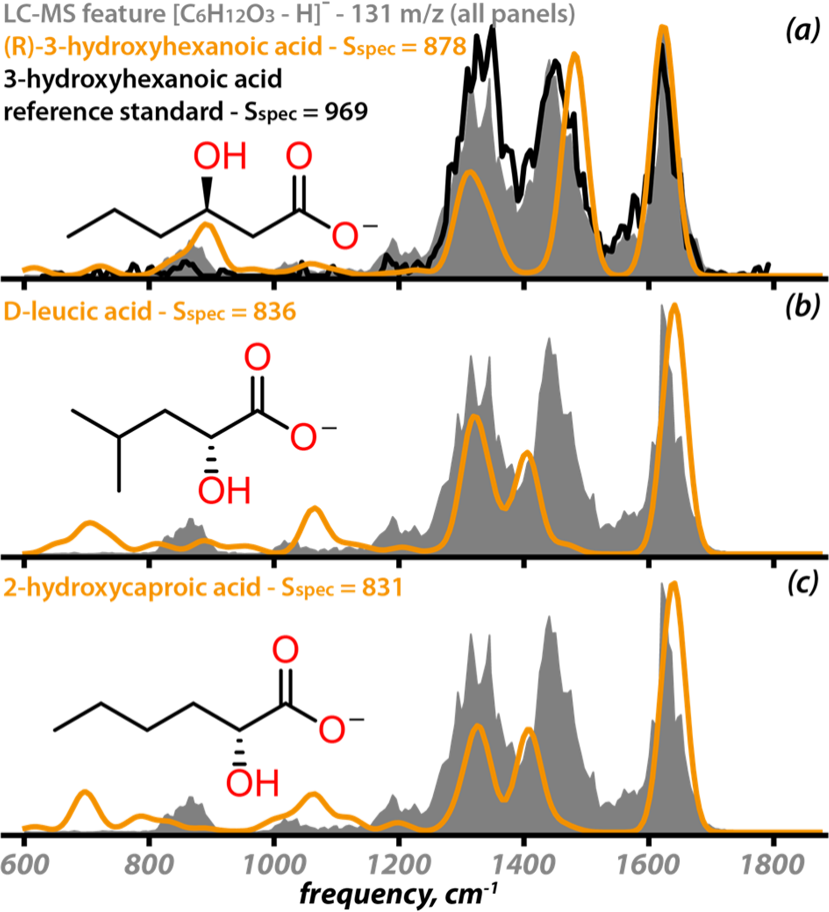
(a–c) The three best matching computed vibrational spectra (orange) obtained in a library query with the IRIS spectrum of an unknown LC-MS feature (gray) with chemical formula [C_6_H_12_O_3_–H]^−^. (a) The experimental spectrum of the 3-hydroxyhexanoate ([M–H]^−^) reference standard (black).

To demonstrate the applicability of the *in silico* IR spectral library to actual biofluids, we elucidated the molecular structure of an LC-MS feature (−ESI, *m*/*z* 131.0713, RT = 6.27 min) elevated in a patient’s plasma sample (see SI for experimental details). The measured *m*/*z* suggests a chemical formula of [C_6_H_12_O_3_–H]^−^ (Δ*m* = +7.6 ppm), which corresponds with 10 entries in our spectral library. Figure 5 shows the experimental IR spectrum of the LC-MS feature and the three best matching computed IR spectra. All 10 spectral comparisons are shown in Figure S14. The three top-ranked structures all possess a carboxylate moiety and a nearby hydroxyl group, giving rise to three main spectral bands between 1200 and 1700 cm^–1^ that are also observed in the IRIS spectrum. Specifically, these bands originate from symmetric and antisymmetric carboxylate O–C–O stretchings (1300 and 1650 cm^–1^) and O–H bending (1450 cm^–1^). The 3-hydroxyhexanoate anion is ranked highest, as the experimental band at 850 cm^–1^ is also reproduced, as the O–H out-of-plane bending vibration. For the 2-hydroxy isomers, this O–H bending vibration is computed at 700 cm^–1^. This assignment is further corroborated by inspecting the top-25 matches of an unsupervised search, which yields the 3-hydroxycarboxylate motif in 13 out of 25 structures, while the 2-hydroxycarboxylate substructure occurs only once (see Figure S15).

The annotation of the LC-MS feature as 3-hydroxyhexanoic acid was confirmed by measuring an IRIS spectrum for a reference standard of this molecule (Figure 5a), which indeed gives high spectral similarity (*S*_*spec*_ = 969) with the spectrum of the LC-MS feature. The stereochemistry of the hydroxide cannot be determined directly from an IR measurement. In general, 3-hydroxycarboxylic acids are biomarkers for fatty acid oxidative disorders of both long- and short-chain 3-hydroxyacyl-CoA dehydrogenases.^52^ Specifically, 3-hydroxyhexanoic acid is increased in the serum of diabetic ketoacidotic patients^53^ and has been observed in body fluids of patients with 3-hydroxy-3-methylglutaryl-CoA synthase deficiency^54^ and metastatic melanoma.^55^ The patient in this case was in ketosis as exemplified by the high body fluid concentrations of acetoacetate and 3-hydroxybutyric acid. After the identification of this *m*/*z* 131.0713 feature, we observed 3-hydroxyhexanoic acid in many plasma samples of ketotic patients. As such, 3-hydroxyhexanoic acid may serve as ketosis biomarker.

## Conclusions and Outlook

The compilation and utilization of an *in silico* IR spectral library of ionized molecules for the identification of unknown metabolites using MS-based IRIS experiments have been demonstrated. An automated workflow to produce IR spectra of molecular ions generated over 75,000 DFT-calculated vibrational spectra for 4640 metabolites taken from the HMDB. A scoring algorithm based on cosine similarity was employed to identify the molecular structures that match favorably with a user supplied experimental IR ion spectrum. By collecting a set of 189 experimental IRIS spectra, we evaluated the performance of the *in silico* IR spectral library in the identification of metabolites. With a known accurate mass value and working within the boundaries of our data set, 75% of the metabolites were correctly identified, which further improves to 83% by simultaneous identification of multiple adducts of the same metabolite. In an *unsupervised* search, where an experimental IRIS spectrum is compared against the entire library, without *m*/*z* constraints, molecular substructures in an unknown molecule are identified, deriving from the strong spectrum–structure correlation of vibrational spectroscopy. This is especially valuable for *de novo* identification of metabolites not included in the library.

The data sets presented here are larger than any previously reported set of experimental or computational IR ion spectra and provide further opportunities for improvement of the metabolite identification workflow. For instance, adapting the scoring method to better capture structural similarity,^39^ applying chemical element-dependent frequency scaling to better correct for anharmonicity,^33^ integrating IRIS scoring with MS/MS scoring algorithms,^56,57^ or tackling the spectrum-to-structure conversion directly^56,57^ may be evaluated with this data set. The experimental and computational spectra are available through the HMDB website and will be added to the Spectra Search interface in its next release (HMDB 6.0). Moreover, we will continue to expand both the experimental and *in silico* IR spectral library. A promising prospect in this regard is the development of machine-learned density functionals, which should significantly speed up DFT calculations.^58^ This could extend the feasibility of our approach to molecules of increased size and with a much larger coverage of chemical space, thereby further establishing infrared ion spectroscopy as an appealing route for small-molecule identification far beyond metabolomics alone.

## Data Availability

Data sets are available at the human metabolome database (https://hmdb.ca) and Zenodo (10.5281/zenodo.7706021).

## Supporting Information Available

Detailed methods, LC-MS procedure, optimization of spectral similarity scoring, evaluation of dispersion correction, sample information, and tables/figures in support of the results (PDF)
Spectral comparisons with all computed conformers and tautomers for all experimental spectra (PDF)
Spectral comparisons for unsupervised searching of the library (PDF)
Spectral comparisons for isomer searching of the library (PDF)
Spectral comparisons for B3LYP vs B3LYP-D3(BJ) vibrational spectra (PDF)
Excel versions of tables in the Supporting Information (XLSX)
