# Supplementary material for: An In Silico Infrared Spectral Library of Molecular Ions for Metabolite Identification
Source: Anal Chem. 2023 Jun 1;95(23):8998–9005. doi: 10.1021/acs.analchem.3c01078 (PMC10267894; doi:10.1021/acs.analchem.3c01078)
Supplement: Supplementary file 1 — ac3c01078_si_001.pdf [file ac3c01078_si_001.pdf]

Supporting Information

An *in silico* infrared spectral library of molecular ions for metabolite identification

Kas J. Houthuijs,[a] Giel Berden,[a] Udo F.H. Engelke,[b] Vasuk Gautam,[c] David S. Wishart,[c,d,e,f] Ron A. Wevers,[b] Jonathan Martens,[a] Jos Oomens[a,g,*]

[a] Institute for Molecules and Materials, FELIX Laboratory, Radboud University, Nijmegen, 6525 ED, The Netherlands
[b] Department of Genetics, Translational Metabolic Laboratory, Radboud University Medical Center, Nijmegen, 6525 GA, The Netherlands
[c] Department of Biological Sciences, University of Alberta, Edmonton, AB T6G 2E9, Canada
[d] Department of Computing Science, University of Alberta, Edmonton, AB T6G 2E8, Canada
[e] Department of Laboratory Medicine and Pathology, University of Alberta, Edmonton, AB T6G 2B7, Canada
[f] Faculty of Pharmacy and Pharmaceutical Sciences, University of Alberta, Edmonton, AB T6G 2H7, Canada
[g] van 't Hoff Institute for Molecular Sciences, University of Amsterdam, Amsterdam, 1098 XH, The Netherlands
* corresponding author email address: jos.oomens@ru.nl

Contents

Chemicals ... 2
Infrared ion spectroscopy ... 2
Computational workflow ... 2
Scoring of spectral similarity ... 3
Scoring of structural similarity ... 3
Sample preparation ... 3
Untargeted LC-MS workflow ... 4
Online LC-IRIS ... 4
Optimization of spectral similarity scoring ... 4
Effect of dispersion correction on computed IR spectra ... 5
Validation metabolites ... 7
Extended results of the library searching ... 12
Correlation of structural and spectral similarity ... 13
Discussion on different adducts ... 18
Observation of higher-energy geometries ... 20
Observation of isomeric mixtures ... 21
Effect of adduct on spectral broadening ... 23
Unknown identification from a patient sample ... 24
References ... 26

Chemicals

Methanol and water (HPLC grade) were obtained from Sigma-Aldrich (St. Louis, USA). The metabolite reference compounds originate from vendors indicated in Table S2. The 3-hydroxyhexanoic acid reference metabolite was obtained from Enamine Ltd. (Kiev, Ukraine).

Infrared ion spectroscopy

IR fingerprint spectra (600-1900 cm^{-1}) of the reference compounds were measured using an ion trap mass spectrometer (Bruker AmaZon Speed ETD, Bremen, Germany) coupled to the beamline of the Free-Electron Laser for Infrared eXperiments, FELIX.[1,2] Solutions of the reference compounds (approx. 10^{-5} M in 1:1 methanol:water) were directly infused into the electrospray ionization (ESI) source, after which adducts of interest ($[M+H]^+$, $[M-H]^-$, and/or $[M+Na]^+$) were mass-isolated and subjected to IR measurements. LC-IRIS measurements were performed in an online fashion using a single 80 µL sample loop installed between a 6-port switching valve and the ESI source.[3,4] The isolated ions were irradiated with 1 to 10 FELIX macropulses of 10 µs; the pulse energy ranges from 20 to 180 mJ depending on wavelength and the laser bandwidth amounts to 0.5% of the center frequency. The internal energy of the ions increases by frequency-dependent absorption of multiple IR photons until the ions undergo fragmentation. Each IR spectrum was reconstructed from a series of mass spectra by plotting the fragmentation yield, $-\ln[\,I_{precursor}/\sum(I_{all})]$, as a function of IR frequency (scanned in 3-5 cm^{-1} steps). When no IR-induced fragment ions were observed, the depletion of the precursor ion was plotted instead, $-\ln[\,I_{precursor}/I_{precursor}^{no\ IR}]$. Although, the IRMPD yield depends non-linearly on laser pulse energy, a first-order linear correction is applied to account for frequency-dependent power variations [5]. A grating spectrometer was used to calibrate the laser frequency.

Computational workflow

The SMILES chemical identifier associated with each HMDB entry was used to generate the starting 2D-structure for the workflow. Using the cheminformatics toolbox RDKit,[6] protonated, deprotonated and sodiated adduct ions were constructed by considering all nitrogen, oxygen and sulfur atoms as sites for H^+ or Na^+ addition or removal. This was done for all possible tautomers, after which resonance structures were filtered out. Some HMDB entries contained unspecified stereochemistry and were omitted, unless their stereochemistry would not affect the IR spectrum (enantiomers), in which case the stereochemistry was randomly assigned. For each ionized isomer a conformational search using RDKit's distance geometry algorithm was performed to produce 500 random 3D-conformations. After minimization using the MMFF94 classical force field, ten conformations were selected after clustering, or fewer if conformations were too similar (rms-deviation of atom positions <1.4 Å). The selected 3D-geometries were then submitted to Gaussian16 for geometry optimization and frequency calculation at the semi-empirical PM6 level.[7] Unfavorable ionization sites and unfavorable conformations were filtered by their relative energies (electronic + thermal) using a threshold of +40 kJ/mol from the global minimum. Additionally, geometries that converged to the same local minimum in the optimization were filtered based on their (nearly) identical vibrational spectrum. The remaining geometries were reoptimized using the B3LYP density functional and 6-311+G(d,p) basis set, followed by a frequency calculation. More accurate electronic energies at the MP2/6-311+G(d,p) level were calculated using the B3LYP geometry and combined with the thermal energy from the B3LYP frequency calculation.

The B3LYP/6-311+G(d,p) frequency calculation was used to generate the reference IR spectra to populate the spectral library. The computed frequencies were scaled by a factor of 0.975 to correct for the harmonic approximation used. The stick spectrum was convolved with a Gaussian profile of 45 cm^{-1} full width at half maximum (FWHM), which gives an optimal spectral scoring performance (see Table S1).

Scoring of spectral similarity

A search of the library with experimental IR spectra retrieves computed IR spectra sorted by their spectral similarity S_{spec}, derived from the cosine similarity score, *i.e.* the normalized Euclidean dot product of two spectra with a common x-axis of *n* points, represented as vectors ***a*** and ***b***

$$S_{spec} = 1000 \cdot \frac{\boldsymbol{a} \cdot \boldsymbol{b}}{|\boldsymbol{a}||\boldsymbol{b}|} = 1000 \cdot \frac{\sum_{i=1}^{n} a_i b_i}{\sqrt{\sum_{i=1}^{n}(a_i)^2}\sqrt{\sum_{i=1}^{n}(b_i)^2}}$$

such that $0 \leq S_{spec} \leq 1000$, with a score closer to 1000 indicating greater similarity.[8] Earlier work evaluated several similarity metrics, one of them defined as the square of the cosine similarity, i.e. providing an identical sorting order; similar performance was established across the different metrics.[8] To expedite spectral comparisons, the convolved computed spectra were binned once at 3 cm^{-1} intervals (minimum experimental step size) and saved into the library. Spectral comparisons are then performed by evaluating the experimental intensities at these wavenumber points through linear interpolation, which ensures a common x-axis.

Earlier studies used a log transformation of the normalized spectral intensities I_i to make S_{spec} less sensitive to intensity deviations (which are typical for IRMPD-based IRIS) and hence more sensitive to frequency overlap.[9] A similar effect is achieved with a power function,[10]

$$I_i^{transformed} = (I_i)^{0.5}$$

Both the values of the exponent (here 0.5) and the Gaussian line broadening (here 45 cm^{-1}) were optimized to provide the best retrieval of the correct spectra (Table S1). The absolute S_{spec} values should be interpreted with care since the scoring is solely optimized on the retrieval rate.

Scoring of structural similarity

To define the structural similarity S_{struc} between two molecules, the ionized metabolites were represented by a Morgan2 bit-vector (based on ECFP4) using RDKit.[11] Conceptually, each bit in this 2048-bit vector represents the presence (1) or absence (0) of a specific chemical substructure in the molecule. Molecules with many substructures in common have similar bit vectors and therefore yield a Dice similarity score closer to 1. The combination of Morgan2 and Dice similarity scores was chosen as this gave a more uniform spread across structural similarities compared to other fingerprints (*e.g.* Morgan3) and similarity measures (*e.g.* Tanimoto).[12]

Sample preparation

Plasma samples were stored at −80 °C and thawed at 4 °C just before sample preparation. A total of 100 µL of the sample was combined with 400 µL of ice-cold methanol/ethanol 50:50 [v/v] and mixed for 15 s using a vortex mixer. For the samples used in metabolic profiling, the methanol/ethanol mixture contained five internal standards [caffeine-d3 0.88 µmol/L, hippuric-d5 acid 0.22 µmol/L, nicotinic-d4 acid 0.88 µmol/L, octanoyl-l-carnitine-d3 0.22 µmol/L, and l-phenyl-d5-alanine 0.44 µmol/L (all from C/D/N Isotopes, Pointe-Claire, Canada)]. The resulting mixture was incubated for 20 min at 4 °C and centrifuged for 15 min at 4 °C (18,600 g). The supernatant (350 µL) was dried in a centrifugal vacuum evaporator (Eppendorf). The dried sample was reconstituted in 100 mL of deionized water/methanol 90:10 [v/v] with 0.1% formic acid, mixed for 15 s with a vortex mixer at room temperature and centrifuged for 15 min at 18600 g. The supernatant (90 µL) was used for LC–MS analysis.

Untargeted LC-MS workflow

A full description of the untargeted metabolic screening procedure and statistical analysis leading to the detection of the biomarker discussed in this work is given elsewhere.[13] In short, LC–MS runs were performed using an Agilent 1290 UHPLC system coupled to an Agilent 6545 QTOF mass spectrometer. Data acquisition and analysis was done using Agilent Mass Hunter (version B.08.00). Separations were performed using a flow rate of 0.4 mL/min on a Waters Acquity HSS T3 C18 column (100 × 2.1 mm i.d., 1.8 µm particles, 100 Å pore size) held at 40 °C. The mobile phase consisted of 0.1% (v/v) formic acid in water and 0.1% (v/v) formic acid in 99:1 (v/v) methanol: water (mobile phase B). After a hold at 99% A for 1 min, a gradient of 15 min was run to 100% B, followed by a hold of 4 min at 100% B and a return to 99% A of 1 min. An equilibration time of 4 min was used, leading to a total analysis time of 25 min. Injection volumes of 2 µL were used for all samples. Alignment and feature extraction were performed using the open access software package XCMS,[14] and two-sided t-tests were performed to identify significantly altered features between patients and controls.

Online LC-IRIS

The online LC-IRIS experiments were performed with a Bruker Elute SP HPLC system (Bremen, Germany) and a two-position six-port switching valve controlled by the quadrupole ion trap mass spectrometer [4]. The separations were performed using the same separation procedure as described above, but with a mobile phase consisting of 10 mM acetic acid in water (mobile phase A) and 10 mM acetic acid in methanol (mobile phase B). The LC and IRIS experiments were combined by installing an 80 µL sample loop between the switching valve and the ESI ion source. As soon as the LC peak of interest arrived at the mass spectrometer, the valve was switched, such that the remainder of the feature material was infused by a syringe pump at a flow rate of 120 µL/h for IRIS experiments.

Optimization of spectral similarity scoring

The extensive set of computational spectra allows for optimization of the FWHM of the Gaussian convolution that is applied to the computational stick spectra as well as of the intensity transformation that is applied prior to the spectral similarity scoring of computational and experimental spectra. To avoid optimizing the scoring algorithm on spectra that have an intrinsically poor match (*e.g.,* several low-energy tautomers/conformers or poor spectral quality), not all IR spectra were included. We selected the IR spectra that ranked within the top-50 of an unsupervised search at 0 kJ/mol energy tolerance, using no intensity scaling and convolution with a FWHM of 25 cm^{-1} (standard parameters for visual inspection). This yielded 125 IR spectra as indicated in Table S4.

To assess the performance of the search to retrieve the correct structures the geometric mean of the ranks r_i of k experimental spectra was minimized. The geometric mean of a set of ranks, also known as the rank product, *RP*, is given by

$$RP = \left(\prod_{i=1}^{k} r_i\right)^{1/k}$$

The *RP* was employed to give equal weight to improvements from 100 → 10 and 10 → 1, whereas an arithmetic mean would favor the former.[15] For the intensity transformation we considered 3 approaches: no transformation, log transformation ($I_{transf.} = \log(I + c)$, based on Ref. [9]) and exponential transformation ($I_{transf.} = I^p$, based on Ref. [10]). The variables *c* and *p* were optimized together with the FWHM using a grid search. From this search, we found that the lowest *RP* is obtained when the power transformation with exponent $p = 0.5$ was combined with a broadening of 45 cm^{-1} FWHM, as is shown in Table S1.

Table S1. *Rank product values of a grid search for the different broadening and intensity transformations. For each type of intensity transformation, the lowest RP is in bold and underlined.*

	$I_{transf.} = I$	$I_{transf.} = I^p$, $p =$							$I_{transf.} = \log(I + c)$, $c =$							
FWHM (cm^{-1})		0.40	0.45	0.50	0.55	0.60	0.65	0.70	-1.2	-1.1	-1.0	-0.9	-0.8	-0.7	-0.6	-0.5
15	11.06	8.04	7.33	7.12	6.92	7.05	7.28	7.54	6.27	5.79	5.84	5.70	5.81	5.98	6.26	6.48
20	9.32	6.33	6.04	5.85	5.84	5.90	6.08	6.32	6.07	5.67	5.61	5.39	5.40	5.45	5.54	5.78
25	8.30	5.58	5.26	5.27	5.26	5.38	5.54	5.75	6.19	5.68	5.55	5.24	5.27	5.31	5.39	5.45
30	7.47	4.94	4.81	4.87	4.93	4.99	5.13	5.36	6.40	5.80	5.64	5.14	5.13	5.19	5.26	5.42
35	7.06	4.77	4.66	4.67	4.64	4.76	4.78	5.07	6.38	5.84	5.64	5.15	**<u>5.10</u>**	5.18	5.12	5.26
40	6.73	4.82	4.56	4.56	4.65	4.57	4.65	4.85	6.72	6.19	6.04	5.53	5.42	5.32	5.34	5.46
45	6.38	4.80	4.56	**<u>4.55</u>**	4.60	4.60	4.59	4.78	7.40	6.66	6.50	5.98	5.78	5.71	5.74	5.88
50	6.27	4.91	4.80	4.71	4.64	4.63	4.61	4.81	8.00	7.17	6.91	6.37	6.20	6.11	6.12	6.44
55	6.21	5.22	5.01	5.00	4.76	4.59	4.65	4.80	8.78	7.96	7.60	7.05	6.92	6.82	6.98	7.16
60	6.20	5.55	5.22	5.10	5.04	4.76	4.72	4.79	9.93	8.97	8.69	7.94	7.89	7.93	8.09	8.33
65	6.08	5.97	5.55	5.47	5.26	5.06	4.85	4.86	11.67	10.45	10.02	9.24	9.10	9.20	9.49	9.97
70	**<u>6.02</u>**	6.64	6.20	5.80	5.54	5.40	5.11	5.02	13.64	12.33	11.87	11.12	10.92	11.00	11.49	12.00
75	6.06	7.27	6.72	6.10	5.69	5.61	5.51	5.26	16.13	14.48	13.99	13.01	12.91	12.98	13.51	14.52

Effect of dispersion correction on computed IR spectra

Upon suggestion of one of the reviewers, we performed a quick evaluation of the inclusion of dispersion correction into the Density functional theory (DFT) computations. Efficient dispersion correction are now available in many computational software suites, such as the D3(BJ) method of Grimme *et al.*[16] The effect of such a correction on the computed vibrational spectra was evaluated using the same validation set as used above (shown in Table S4). The lowest-energy geometry at the MP2/6-311+G(d,p)//B3LYP/6-311+G(d,p) level of theory was used as a starting point for a geometry optimization and frequency analysis at the B3LYP-D3(BJ)/6-311+G(d,p) level of theory using Gaussian16. Spectral similarity scores S_{spec} were calculated of the dispersion-corrected computed spectra, and the average similarity score and standard deviation are shown in Table S2, together with the original score obtained with B3LYP. A two-sided t-test indicates that the spectral similarity scores obtained with the two methods do not differ significantly. The individual spectral comparisons for all 125 compounds are shown in a separate SI file.

Table S2. *Mean spectral similarity score, standard deviation, and two-sided t-test when experimental IR spectra (n = 125) are compared to spectra computed with the B3LYP and B3LYP-D3(BJ) methods.*

	mean S_{spec}	std dev S_{spec}
B3LYP	896.5	±46.1
B3LYP-D3(BJ)	895.4	±51.4
two-sided t-test:	p-value = 0.86	

This insignificant difference appears to originate from the fact that the dispersion-corrected geometries, and thus computed vibrational spectra, remain largely unchanged. This is also seen in Figure S1, where the spectral similarity scores between the B3LYP and B3LYP-D3(BJ) computed vibrational spectra are shown. We speculate that for the ionic systems investigated here, the dispersion interactions are much weaker than electrostatic interactions, which then determine the conformations adopted by these systems. For 115 out of 125 spectra, the spectral similarity between B3LYP and B3LYP-D3(BJ) is 994 or higher, demonstrating that most spectra (and thus geometries) are virtually identical, irrespective of a dispersion correction. Of the 10 ions where the spectra are more distinct, the B3LYP spectrum matches better with the experiment in 4 cases (red sticks in Figure S1),

and the B3LYP-D3(BJ) spectrum matches better with the experiment in 6 cases (blue sticks). Certainly, further evaluation is warranted, for instance including different dispersion corrections and including these corrections already at the conformational search stage.

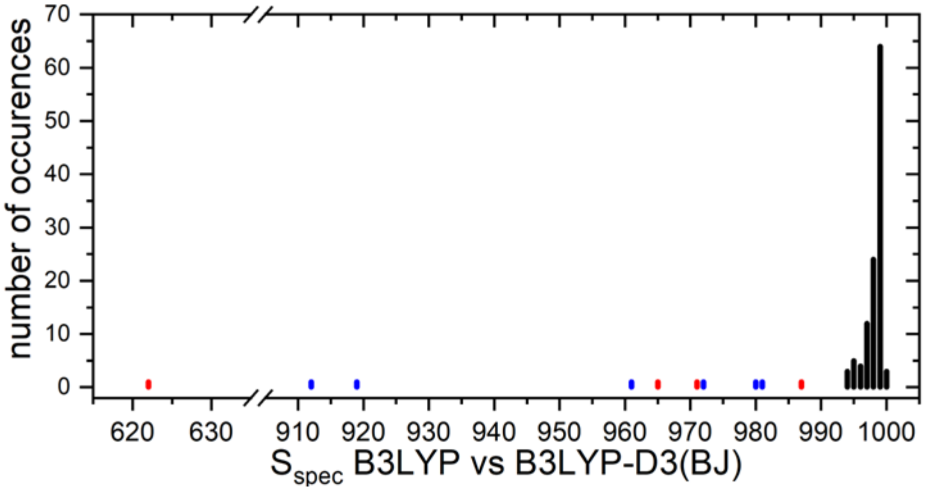

Figure S1. *Spectral similarity between the B3LYP and B3LYP-D3(BJ) computed vibrational spectra. For the 10 computed spectra that deviate more (S_{spec} < 994), it is indicated whether the B3LYP-computed vibrational spectrum (red) or B3LYP-D3(BJ)-computed vibrational spectrum (blue) matches best with the experimental IR spectrum.*

Validation metabolites

Table S3. *HMDB ID, name, vendor, formula and accurate mass of the 87 metabolites in the validation set. Full names of the vendors are: MetaSci (Toronto, Canada), Merck Life sciences N.V. (Amsterdam, The Netherlands), TCI Europe N.V. (Zwijndrecht, Belgium), Amsterdam Police Laboratory (Amsterdam, The Netherlands), Enamine Ltd. (Kiev, Ukraine), Biomatik (Kitchener, Canada).*

#	HMDB ID	Name	Vendor	Formula	Accurate mass
1	HMDB0000062	L-Carnitine	MetaSci	C7H15NO3	161.1052
2	HMDB0000070	Pipecolic acid	MetaSci	C6H11NO2	129.0790
3	HMDB0000073	Dopamine	MetaSci	C8H11NO2	153.0790
4	HMDB0000107	Galactitol	MetaSci	C6H14O6	182.0790
5	HMDB0000130	Homogentisic acid	Merck	C8H8O4	168.0423
6	HMDB0000132	Guanine	MetaSci	C5H5N5O	151.0494
7	HMDB0000143	D-Galactose	Merck	C6H12O6	180.0634
8	HMDB0000148	L-Glutamic acid	MetaSci	C5H9NO4	147.0532
9	HMDB0000159	L-Phenylalanine	MetaSci	C9H11NO2	165.0790
10	HMDB0000172	L-Isoleucine	Merck	C6H13NO2	131.0946
11	HMDB0000182	L-Lysine	Merck	C6H14N2O2	146.1055
12	HMDB0000191	L-Aspartic acid	MetaSci	C4H7NO4	133.0375
13	HMDB0000201	L-Acetylcarnitine	MetaSci	C9H17NO4	203.1158
14	HMDB0000202	Methylmalonic acid	MetaSci	C4H6O4	118.0266
15	HMDB0000208	Oxoglutaric acid	MetaSci	C5H6O5	146.0215
16	HMDB0000211	myo-Inositol	Merck	C6H12O6	180.0634
17	HMDB0000214	Ornithine	MetaSci	C5H12N2O2	132.0899
18	HMDB0000247	Sorbitol	Merck	C6H14O6	182.0790
19	HMDB0000251	Taurine	MetaSci	C2H7NO3S	125.0147
20	HMDB0000254	Succinic acid	MetaSci	C4H6O4	118.0266
21	HMDB0000263	Phosphoenolpyruvic acid	MetaSci	C3H5O6P	167.9824
22	HMDB0000272	Phosphoserine	MetaSci	C3H8NO6P	185.0089
23	HMDB0000292	Xanthine	MetaSci	C5H4N4O2	152.0334
24	HMDB0000300	Uracil	MetaSci	C4H4N2O2	112.0273
25	HMDB0000301	Urocanic acid	Merck	C6H6N2O2	138.0429
26	HMDB0000306	Tyramine	MetaSci	C8H11NO	137.0841
27	HMDB0000323	3-Amino-2-piperidone	Merck	C5H10N2O	114.0793
28	HMDB0000434	Homoveratric acid	MetaSci	C10H12O4	196.0736
29	HMDB0000446	N-Alpha-acetyllysine	MetaSci	C8H16N2O3	188.1161
30	HMDB0000448	Adipic acid	MetaSci	C6H10O4	146.0579
31	HMDB0000500	4-Hydroxybenzoic acid	MetaSci	C7H6O3	138.0317
32	HMDB0000510	Aminoadipic acid	MetaSci	C6H11NO4	161.0688
33	HMDB0000512	N-Acetyl-L-phenylalanine	Merck	C11H13NO3	207.0895
34	HMDB0000517	L-Arginine	MetaSci	C6H14N4O2	174.1117
35	HMDB0000522	3-Methylglutaconic acid	Merck	C6H8O4	144.0423
36	HMDB0000532	Acetylglycine	MetaSci	C4H7NO3	117.0426
37	HMDB0000622	Ethylmalonic acid	Merck	C5H8O4	132.0423
38	HMDB0000630	Cytosine	MetaSci	C4H5N3O	111.0433
39	HMDB0000641	L-Glutamine	MetaSci	C5H10N2O3	146.0691
40	HMDB0000660	D-Fructose	Merck	C6H12O6	180.0634
41	HMDB0000661	Glutaric acid	Merck	C5H8O4	132.0423
42	HMDB0000679	Homocitrulline	MetaSci	C7H15N3O3	189.1113
43	HMDB0000687	L-Leucine	MetaSci	C6H13NO2	131.0946
44	HMDB0000715	Kynurenic acid	MetaSci	C10H7NO3	189.0426
45	HMDB0000759	Glycylleucine	MetaSci	C8H16N2O3	188.1161
46	HMDB0000765	Mannitol	Merck	C6H14O6	182.0790
47	HMDB0000821	Phenylacetylglycine	MetaSci	C10H11NO3	193.0739
48	HMDB0000822	p-Hydroxymandelic acid	MetaSci	C8H8O4	168.0423
49	HMDB0000828	Ureidosuccinic acid	MetaSci	C5H8N2O5	176.0433
50	HMDB0000842	Quinaldic acid	MetaSci	C10H7NO2	173.0477
51	HMDB0000860	Phenylpropionylglycine	Merck	C11H13NO3	207.0895
52	HMDB0000873	4-Methylcatechol	MetaSci	C7H8O2	124.0524

Table S3 (continued). *HMDB ID, name, vendor, formula and accurate mass of the 87 metabolites in the validation set. Full names of the vendors are: MetaSci (Toronto, Canada), Merck Life sciences N.V. (Amsterdam, The Netherlands), TCI Europe N.V. (Zwijndrecht, Belgium), Amsterdam Police Laboratory (Amsterdam, The Netherlands), Enamine Ltd. (Kiev, Ukraine), Biomatik (Kitchener, Canada).*

#	HMDB ID	Name	Vendor	Formula	Accurate mass
53	HMDB0000904	Citrulline	MetaSci	C6H13N3O3	175.0957
54	HMDB0000929	L-Tryptophan	MetaSci	C11H12N2O2	204.0899
55	HMDB0000930	trans-Cinnamic acid	MetaSci	C9H8O2	148.0524
56	HMDB0000959	Tiglylglycine	TCI Europe	C7H11NO3	157.0739
57	HMDB0001149	5-Aminolevulinic acid	MetaSci	C5H9NO3	131.0582
58	HMDB0001232	4-Nitrophenol	MetaSci	C6H5NO3	139.0269
59	HMDB0001336	3,4-Dihydroxybenzeneacetic acid	MetaSci	C8H8O4	168.0423
60	HMDB0001859	Acetaminophen	MetaSci	C8H9NO2	151.0633
61	HMDB0001886	3-Methylxanthine	MetaSci	C6H6N4O2	166.0491
62	HMDB0001890	Acetylcysteine	MetaSci	C5H9NO3S	163.0303
63	HMDB0001964	Caffeic acid	MetaSci	C9H8O4	180.0423
64	HMDB0002266	(E)-2-Methylglutaconic acid	Merck	C6H8O4	144.0423
65	HMDB0002285	2-Indolecarboxylic acid	MetaSci	C9H7NO2	161.0477
66	HMDB0002302	Indole-3-propionic acid	MetaSci	C11H11NO2	189.0790
67	HMDB0002432	Sumiki's acid	MetaSci	C6H6O4	142.0266
68	HMDB0002825	Theobromine	MetaSci	C7H8N4O2	180.0647
69	HMDB0003152	N-Methylnicotinamide	MetaSci	C7H8N2O	136.0637
70	HMDB0003320	Indole-3-carboxylic acid	MetaSci	C9H7NO2	161.0477
71	HMDB0003633	N-Methyltyramine	MetaSci	C9H13NO	151.0997
72	HMDB0004095	5-Methoxytryptamine	MetaSci	C11H14N2O	190.1106
73	HMDB0005807	Gallic acid	MetaSci	C7H6O5	170.0215
74	HMDB0012128	(R)-Amphetamine	Amsterdam Police	C9H13N	135.1048
75	HMDB0012140	(R) 2,3-Dihydroxy-3-methylvalerate	Enamine	C6H12O4	148.0736
76	HMDB0013318	Tryptophanamide	MetaSci	C11H13N3O	203.1059
77	HMDB0014389	Mesalazine	MetaSci	C7H7NO3	153.0426
78	HMDB0015517	Methamphetamine	Amsterdam Police	C10H15N	149.1204
79	HMDB0028850	Glycyl-Serine	Biomatik	C5H10N2O4	162.0641
80	HMDB0031861	2-Acetylpyrazine	MetaSci	C6H6N2O	122.0480
81	HMDB0036458	1-Aminocyclopropanecarboxylic acid	MetaSci	C4H7NO2	101.0477
82	HMDB0041923	Mephedrone	Amsterdam Police	C11H15NO	177.1154
83	HMDB0041931	3,4-Methylenedioxyamphetamine	Amsterdam Police	C10H13NO2	179.0946
84	HMDB0059720	Meta-Tyrosine	MetaSci	C9H11NO3	181.0739
85	HMDB0060608	N-isopropylterephthalamic acid	Enamine	C11H13NO3	207.0895
86	HMDB0061705	6-Oxopiperidine-2-carboxylic acid	Merck	C6H9NO3	143.0582
87	HMDB0094701	N-Acetylproline	Merck	C7H11NO3	157.0739

Scheme S1. *HMDB ID, name and neutral chemical structure of the 87 metabolites in the validation set.*

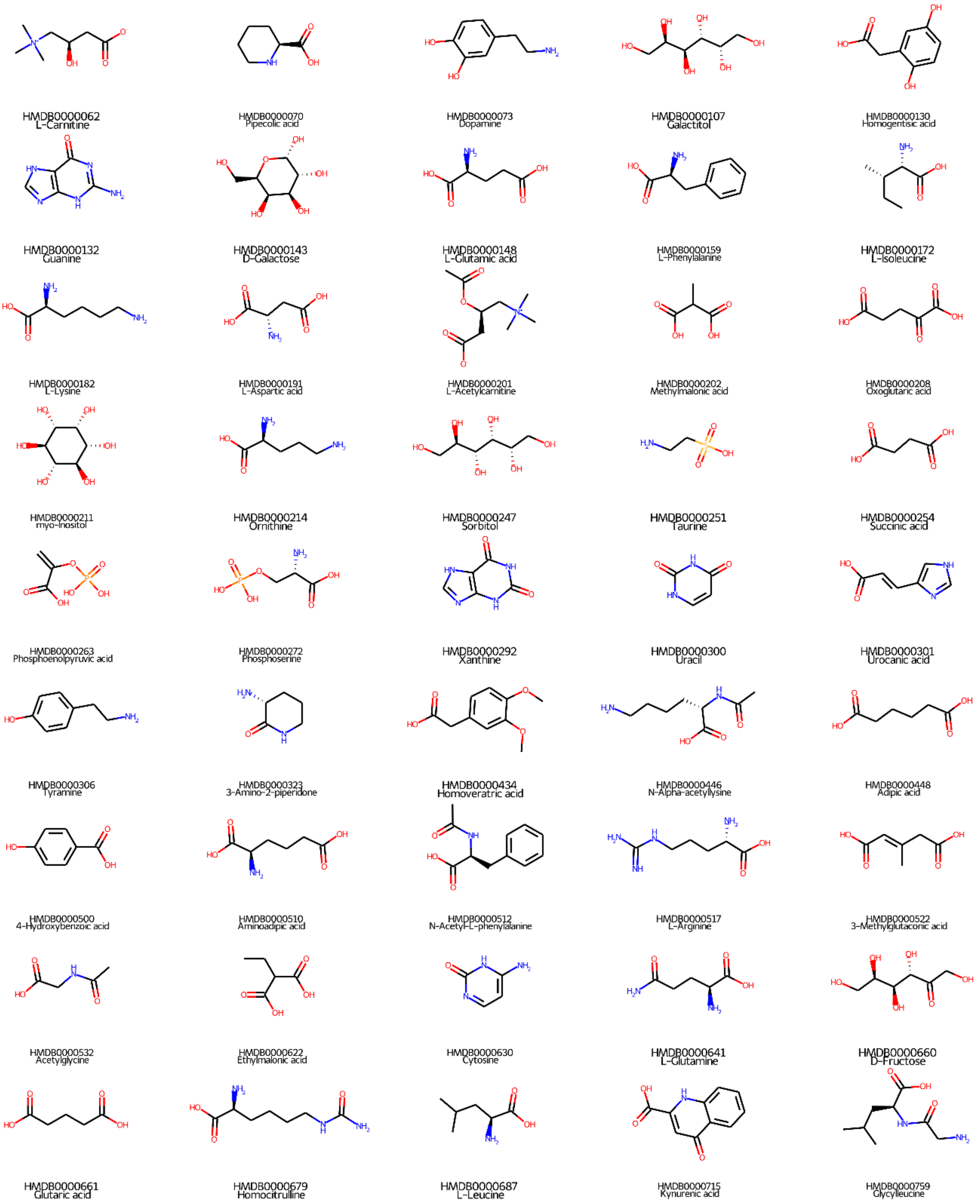

Scheme S1 (continued). *HMDB ID, name and neutral chemical structure of the 87 metabolites in the validation set.*

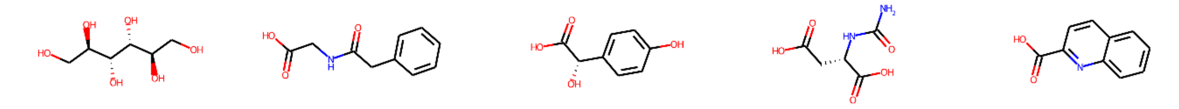

HMDB0000765 Mannitol | HMDB0000821 Phenylacetylglycine | HMDB0000822 p-Hydroxymandelic acid | HMDB0000828 Ureidosuccinic acid | HMDB0000842 Quinaldic acid

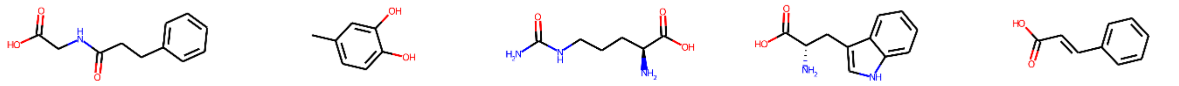

HMDB0000860 Phenylpropionylglycine | HMDB0000873 4-Methylcatechol | HMDB0000904 Citrulline | HMDB0000929 L-Tryptophan | HMDB0000930 trans-Cinnamic acid

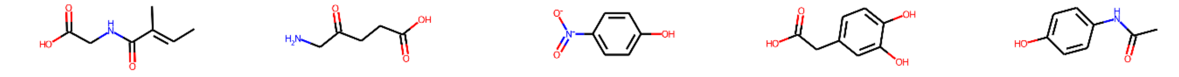

HMDB0000959 Tiglylglycine | HMDB0001149 5-Aminolevulinic acid | HMDB0001232 4-Nitrophenol | HMDB0001336 3,4-Dihydroxybenzeneacetic acid | HMDB0001859 Acetaminophen

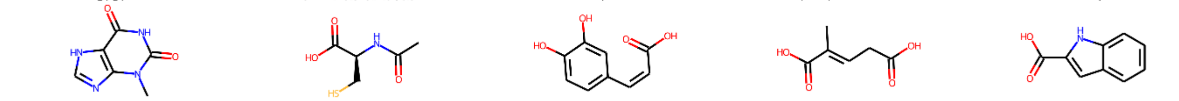

HMDB0001886 3-Methylxanthine | HMDB0001890 Acetylcysteine | HMDB0001964 Caffeic acid | HMDB0002266 (E)-2-Methylglutaconic acid | HMDB0002285 2-Indolecarboxylic acid

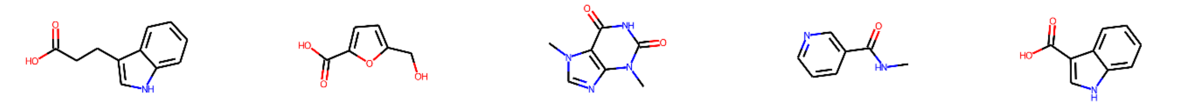

HMDB0002302 Indole-3-propionic acid | HMDB0002432 Sumiki's acid | HMDB0002825 Theobromine | HMDB0003152 N-Methylnicotinamide | HMDB0003320 Indole-3-carboxylic acid

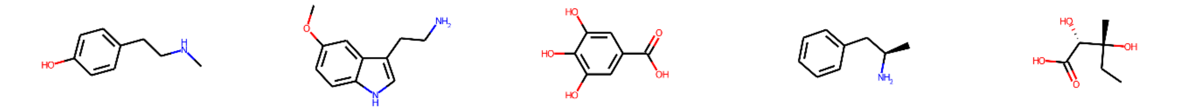

HMDB0003633 N-Methyltyramine | HMDB0004095 5-Methoxytryptamine | HMDB0005807 Gallic acid | HMDB0012128 (R)-Amphetamine | HMDB0012140 (R) 2,3-Dihydroxy-3-methylvalerate

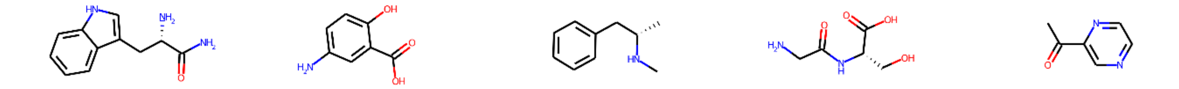

HMDB0013318 Tryptophanamide | HMDB0014389 Mesalazine | HMDB0015517 Methamphetamine | HMDB0028850 Glycyl-Serine | HMDB0031861 2-Acetylpyrazine

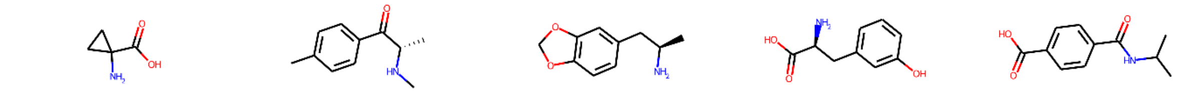

HMDB0036458 1-Aminocyclopropanecarboxylic acid | HMDB0041923 Mephedrone | HMDB0041931 3,4-Methylenedioxyamphetamine | HMDB0059720 Meta-Tyrosine | HMDB0060608 N-isopropylterephthalamic acid

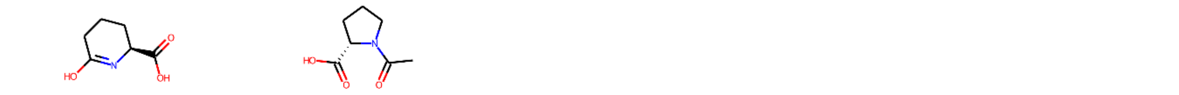

HMDB0061705 6-Oxopiperidine-2-carboxylic acid | HMDB0094701 N-Acetylproline

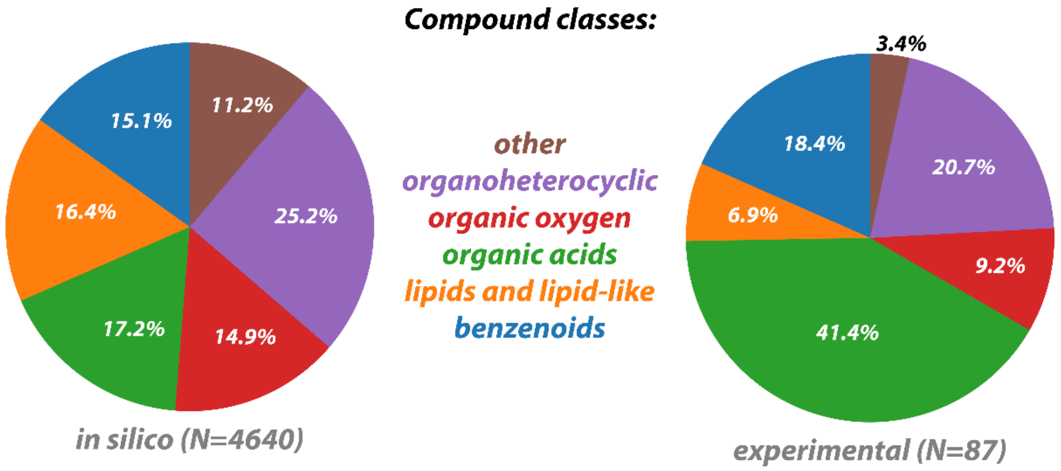

Figure S2. *Compound classes as determined with ClassyFire [17] for the metabolites included in the in silico library (left) and the experimental validation set (right).*

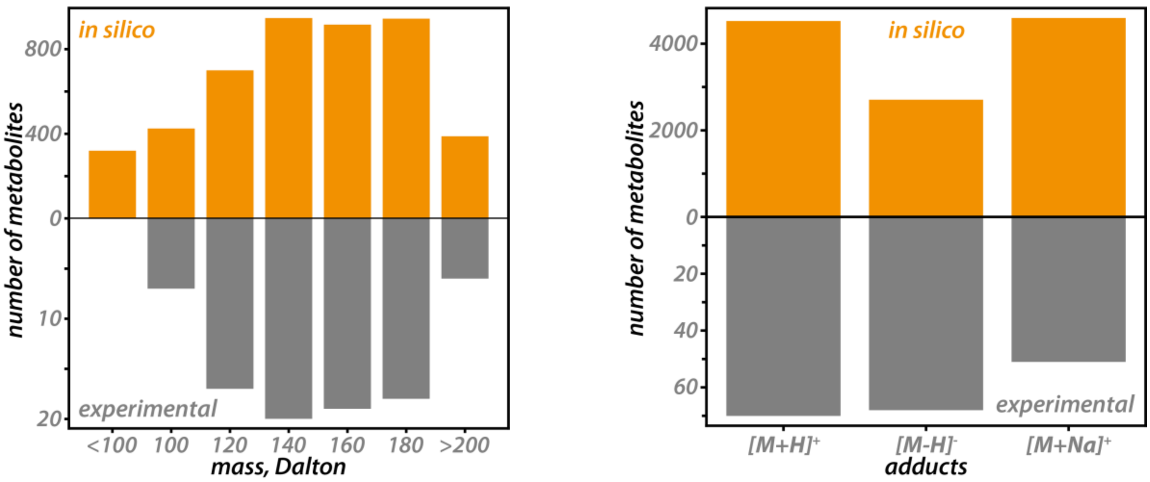

Figure S3. *Mass distribution (left) and adduct distribution (right) of the metabolites included in the in silico library (orange) and experimental validation set (gray).*

Extended results of the library searching

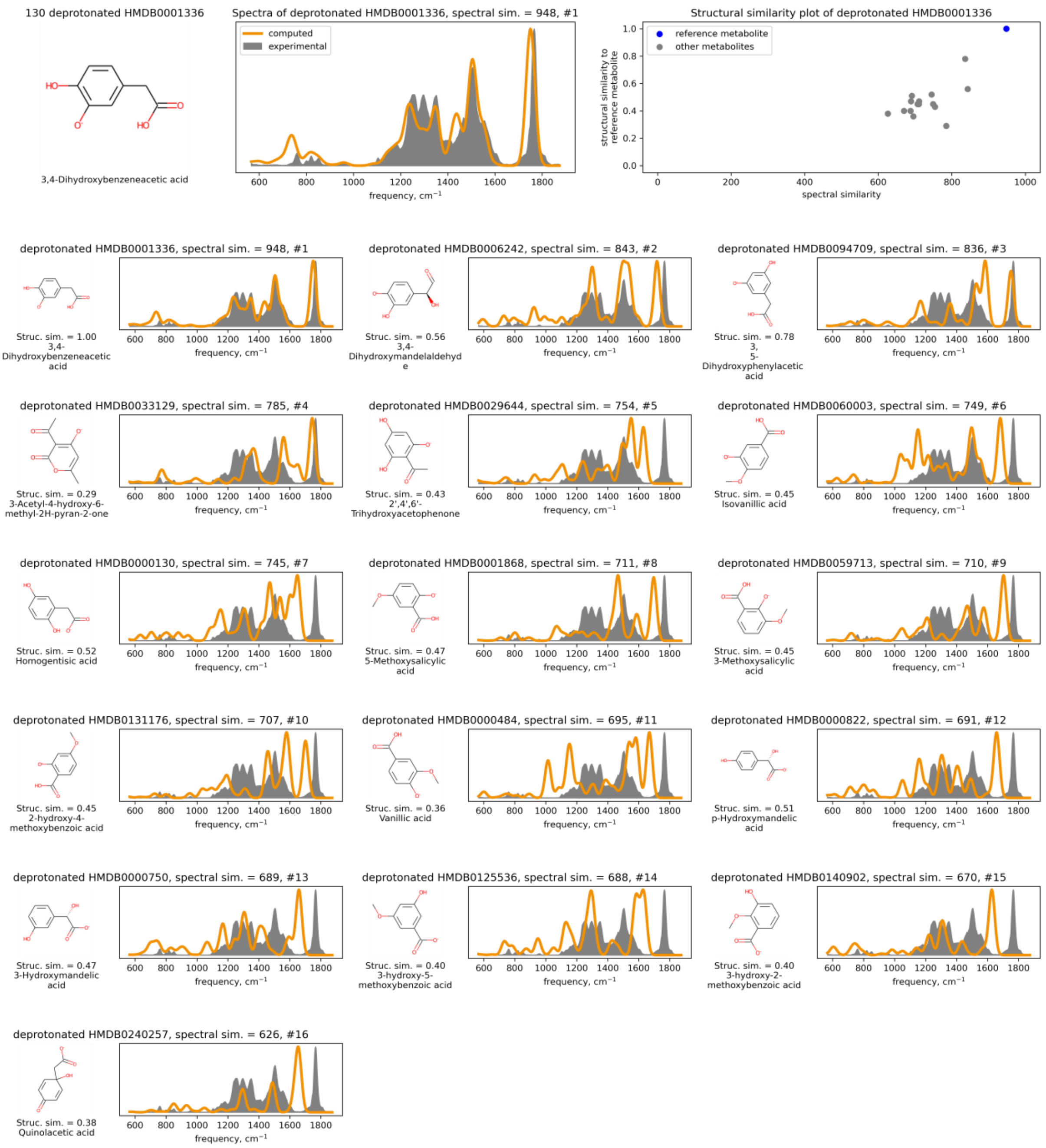

Figure S4. *Spectral comparisons of the computed vibrational spectra (orange) of 16 deprotonated entries in the in silico library with formula [$C_8H_8O_4$-H]$^-$ with the experimental IR spectrum (gray) of deprotonated 3,4-dihydroxybenzeneacetic acid ([DOPAC-H]$^-$). For each library entry, the spectral similarity with the experiment is given, as well as the structural similarity of the deprotonated ion with the structure of [DOPAC-H]$^-$. The top right shows the relation between spectral and structural similarity for all 16 entries.*

Correlation of structural and spectral similarity

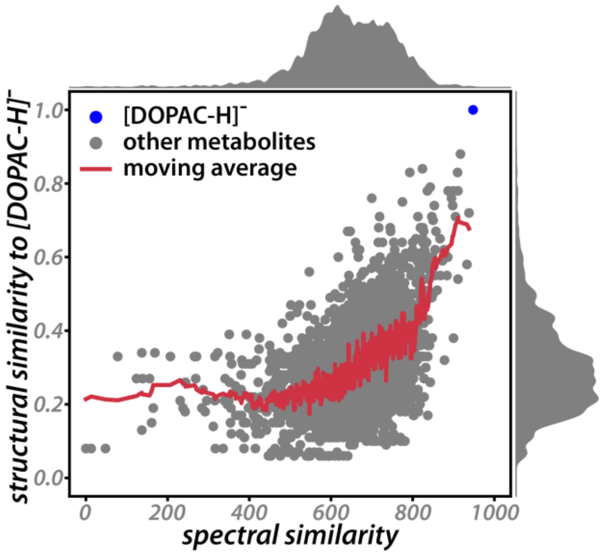

Figure S5. *For each entry in the in silico library, the structural similarity and spectral similarity to [DOPAC-H]$^-$ are plotted as the grey dots. [DOPAC-H]$^-$ itself is the blue dot. The trend is visualized with a 15-point moving average (red line), excluding the blue data point. Distributions of the structural and spectral similarity are presented on the right and top axis, respectively.*

To further quantify the ability of the unsupervised IRIS spectral search to find structurally similar metabolites, the structural similarity between each deprotonated metabolite and [DOPAC-H]$^-$ was calculated using the Dice similarity score of their bit vectors (see details above). For each metabolite in our library, the grey dots in Figure S5 mark the structural similarity to [DOPAC-H]$^-$ plotted versus the spectral similarity to its IRIS spectrum. For a large majority of structures with intermediate spectral similarity to [DOPAC-H]$^-$, the structural similarity fluctuates greatly. This arises from the fact that coincidental overlap between absorption bands due to different functional groups is common in vibrational spectra. However, the moving average (bin size of 15) shows a steep increase as the spectral score approaches its maximum value of 1000. This indicates that good spectral similarity indeed correlates well with similarity in the type, number and relative positioning of functional groups within a molecule. This is clearly reflected in the top-9 retrieved structures all being very similar to DOPAC (Figure 3 in the main manuscript).

Table S4. *Rank of the spectrum and number of queried spectra for each search (type and energy tolerance). The last 2 columns show whether the spectrum was used in the grid search (to optimize the parameters for the spectral similarity scoring) and the literature source (if any).*

#	HMDB ID	ion form	isomer search						unsupervised search						combined		grid search?	lit.
			0 kJ/mol		10 kJ/mol		no energy		0 kJ/mol		10 kJ/mol		no energy		0 kJ/mol			
1	HMDB0000062	pro	1	1	1	2	1	10	3	4529	4	12133	8	30842	1	4536	y	
2		sod	1	1	1	2	1	5	3	4599	3	14415	3	30950			y	
3		dep	2	7	2	15	1	26	85	2707	95	6748	39	14149				
4	HMDB0000070	pro	1	7	1	16	1	47	4	4529	4	12133	5	30842	61	2656	y	[18]
5		sod	7	7	7	19	7	50	3466	4599	3584	14415	3174	30950				
6	HMDB0000073	dep	1	3	1	6	1	8	21	2707	26	6748	34	14149	3	2675	y	
7		pro	1	3	1	7	1	10	2	4529	1	12133	1	30842			y	
8	HMDB0000107	dep	1	4	2	16	3	136	56	2707	6	6748	9	14149	56	2707	y	
9	HMDB0000130	dep	2	16	3	34	4	54	38	2707	64	6748	99	14149	2	2733		[19]
10		sod	1	18	1	42	1	98	9	4599	13	14415	16	30950				
11		dep	1	3	1	6	2	17	678	2707	836	6748	652	14149				
12	HMDB0000132	pro	1	3	1	8	1	13	1	4529	1	12133	2	30842	4	2656	y	
13		sod	1	3	1	7	1	19	21	4599	26	14415	63	30950			y	
14	HMDB0000143	dep	16	17	17	46	12	177	604	2707	672	6748	241	14149	604	2707		
15	HMDB0000148	dep	4	7	4	15	5	49	1171	2707	1342	6748	1140	14149	273	2733		
16		sod	4	7	5	20	2	83	337	4599	443	14415	10	30950				
17		dep	1	9	1	20	1	28	36	2707	5	6748	11	14149			y	
18	HMDB0000159	pro	1	9	1	17	1	42	6	4529	8	12133	8	30842	6	2656	y	
19		sod	1	9	1	21	1	51	126	4599	182	14415	300	30950				
20	HMDB0000172	pro	2	10	3	33	3	97	16	4529	20	12133	22	30842	16	4529	y	
21		dep	3	3	2	23	2	36	404	2707	356	6748	462	14149				
22	HMDB0000182	pro	3	3	3	7	3	44	107	4529	51	12133	125	30842	13	2656		[18]
23		sod	1	3	2	6	3	30	12	4599	17	14415	60	30950			y	
24		dep	1	2	1	3	1	4	2135	2707	2206	6748	2010	14149				
25	HMDB0000191	pro	1	2	1	6	1	14	1	4529	1	12133	1	30842	35	2656	y	
26		sod	1	2	1	4	1	11	13	4599	22	14415	40	30950			y	
27	HMDB0000201	pro	1	2	1	5	1	32	1	4529	1	12133	1	30842	1	4536	y	
28		sod	1	2	1	5	1	41	1	4599	1	14415	1	30950			y	
29	HMDB0000202	pro	2	7	2	8	3	31	275	4529	383	12133	685	30842	275	4599		[20]
30	HMDB0000208	dep	1	2	1	3	1	12	289	2707	403	6748	552	14149	24	2733		
31		sod	1	2	1	5	1	8	12	4599	14	14415	17	30950			y	
32	HMDB0000211	dep	3	17	6	46	12	177	38	2707	69	6748	84	14149	38	2707	y	
33		dep	1	1	1	9	1	10	154	2707	62	6748	83	14149				
34	HMDB0000214	pro	1	1	1	1	1	15	2	4529	5	12133	4	30842	6	2656	y	
35		sod	1	1	1	3	1	9	2120	4599	2375	14415	2276	30950				
36	HMDB0000247	dep	2	4	3	16	3	136	36	2707	14	6748	5	14149	36	2707	y	
37	HMDB0000251	dep	1	1	1	2	1	2	626	2707	757	6748	911	14149	485	2733		
38		sod	1	1	1	1	1	11	577	4599	767	14415	1066	30950				
39	HMDB0000254	dep	3	7	4	11	4	13	1727	2707	1840	6748	1937	14149	2146	2675		
40		pro	6	7	6	8	7	31	4028	4529	4123	12133	3673	30842				[20]
41	HMDB0000263	dep	1	1	1	4	1	9	320	2707	182	6748	300	14149	320	2707		
42		dep	1	1	1	2	1	19	16	2707	36	6748	36	14149				
43	HMDB0000272	pro	1	1	1	1	1	7	7	4529	20	12133	12	30842	3	2656	y	
44		sod	1	1	1	2	1	16	38	4599	60	14415	6	30950				
45	HMDB0000292	dep	2	3	2	4	2	8	18	2707	29	6748	27	14149	2	2675	y	
46		pro	1	3	1	4	1	15	2	4529	3	12133	3	30842			y	
47		dep	1	2	1	3	1	4	2	2707	2	6748	2	14149			y	
48	HMDB0000300	pro	1	2	1	3	2	9	38	4529	61	12133	130	30842	1	2656	y	
49		sod	1	2	1	3	1	9	1	4599	1	14415	1	30950			y	
50	HMDB0000301	dep	1	3	1	6	1	10	3	2707	3	6748	4	14149	60	2675	y	
51		pro	3	4	1	7	1	20	555	4529	1	12133	1	30842				
52		dep	1	6	1	9	1	10	38	2707	46	6748	59	14149			y	
53	HMDB0000306	pro	1	8	1	13	1	17	2	4529	2	12133	2	30842	1	2656	y	
54		sod	1	8	1	16	1	30	1	4599	1	14415	1	30950			y	
55	HMDB0000323	pro	1	2	1	4	1	5	967	4529	1255	12133	1808	30842	967	4529		
56	HMDB0000434	dep	1	25	1	86	1	182	1	2707	1	6748	1	14149	1	2733	y	
57		sod	1	27	1	87	3	264	1	4599	1	14415	3	30950			y	
58		dep	1	5	1	18	1	83	24	2707	32	6748	33	14149			y	
59	HMDB0000446	pro	2	4	1	12	2	64	2	4529	1	12133	2	30842	3	2656	y	
60		sod	2	5	3	17	4	97	103	4599	146	14415	101	30950				
61	HMDB0000448	dep	2	7	2	20	2	24	438	2707	550	6748	714	14149	22	2733		
62		sod	2	8	2	20	4	75	44	4599	27	14415	67	30950			y	

Table S4 (continued). *Rank of the spectrum and number of queried spectra for each search (type and energy tolerance). The last 2 columns show whether the spectrum was used in the grid search (to optimize the parameters for the spectral similarity scoring) and the literature source (if any).*

		ion	isomer search						unsupervised search						combined		grid	
#	HMDB ID	form	0 kJ/mol		10 kJ/mol		no energy		0 kJ/mol		10 kJ/mol		no energy		0 kJ/mol		search?	lit.
63	HMDB0000500	dep	1	7	1	11	1	16	1	2707	1	6748	1	14149	1	2675	y	
64		pro	1	9	1	14	1	18	1	4529	2	12133	2	30842			y	
65		dep	1	5	1	24	1	48	177	2707	229	6748	252	14149				
66	HMDB0000510	pro	2	5	2	9	2	63	9	4529	13	12133	25	30842	3	2656	y	
67		sod	1	5	2	16	2	58	43	4599	78	14415	91	30950			y	
68		dep	1	5	1	12	1	36	11	2707	13	6748	14	14149			y	
69	HMDB0000512	pro	1	5	1	16	1	75	8	4529	6	12133	17	30842	2	2656	y	
70		sod	2	5	2	10	2	29	9	4599	9	14415	9	30950			y	
71	HMDB0000517	pro	1	1	1	1	1	22	9	4529	13	12133	9	30842	9	4529	y	
72	HMDB0000522	dep	1	7	1	13	1	26	734	2707	935	6748	1110	14149	69	2675		
73		pro	1	8	1	16	1	44	8	4529	7	12133	24	30842			y	
74		dep	1	3	1	8	1	10	10	2707	16	6748	19	14149			y	
75	HMDB0000532	pro	1	3	1	8	1	28	20	4529	28	12133	52	30842	1	2656	y	
76		sod	1	3	1	3	1	15	6	4599	14	14415	23	30950			y	
77	HMDB0000622	dep	1	8	2	15	2	16	1059	2707	1192	6748	1319	14149	542	2675		20
78		pro	2	8	2	16	5	59	367	4529	484	12133	775	30842				20
79		dep	1	1	1	1	1	3	17	2707	25	6748	41	14149			y	
80	HMDB0000630	pro	1	1	1	1	1	1	229	4529	301	12133	486	30842	7	2656		
81		sod	1	1	1	1	1	2	3	4599	3	14415	3	30950			y	
82		dep	1	3	1	11	1	22	51	2707	104	6748	33	14149				
83	HMDB0000641	pro	1	4	2	7	2	42	16	4529	24	12133	44	30842	10	2656	y	
84		sod	1	3	1	11	1	42	152	4599	2	14415	3	30950				
85	HMDB0000660	dep	1	17	1	46	1	177	2	2707	5	6748	2	14149	2	2707	y	
86	HMDB0000661	dep	8	8	8	15	8	16	2495	2707	2525	6748	2174	14149	2481	2675		20
87		pro	7	8	7	16	4	59	4031	4529	4002	12133	3086	30842				20
88		dep	1	1	1	6	1	12	4	2707	1	6748	1	14149			y	
89	HMDB0000679	pro	1	1	1	1	1	13	1	4529	1	12133	1	30842	1	2656	y	
90		sod	1	1	1	6	1	16	5	4599	1	14415	1	30950			y	
91	HMDB0000687	pro	1	10	1	33	1	97	7	4529	9	12133	11	30842	11	4536	y	
92		sod	3	9	2	49	4	121	242	4599	153	14415	296	30950				
93		dep	1	1	1	1	1	2	1	2707	1	6748	2	14149			y	
94	HMDB0000715	pro	1	3	1	4	1	4	6	4529	8	12133	18	30842	1	2656	y	
95		sod	1	3	1	3	1	4	239	4599	392	14415	312	30950				
96	HMDB0000759	dep	2	5	3	18	3	83	27	2707	33	6748	36	14149	29	2675	y	
97		pro	3	4	1	12	1	64	208	4529	16	12133	31	30842				
98	HMDB0000765	dep	2	4	2	16	4	136	41	2707	6	6748	8	14149	41	2707	y	
99		dep	2	8	2	15	2	30	8	2707	10	6748	11	14149			y	
100	HMDB0000821	pro	5	8	1	26	1	124	585	4529	3	12133	6	30842	20	2656		
101		sod	1	8	1	14	1	68	1	4599	1	14415	1	30950			y	
102	HMDB0000822	dep	1	16	1	34	1	54	1	2707	2	6748	3	14149	1	2733	y	
103		sod	2	18	2	42	3	98	21	4599	33	14415	64	30950				
104		dep	1	3	1	9	1	42	258	2707	101	6748	146	14149				
105	HMDB0000828	pro	1	3	1	5	1	46	1	4529	1	12133	1	30842	1	2656	y	
106		sod	2	3	2	8	1	41	13	4599	18	14415	1	30950			y	
107		dep	1	1	1	1	1	1	1	2707	1	6748	3	14149			y	
108	HMDB0000842	pro	1	2	1	2	1	3	1	4529	1	12133	1	30842	1	2656	y	
109		sod	1	2	1	2	1	4	161	4599	250	14415	441	30950				
110		dep	1	5	1	12	1	36	18	2707	18	6748	18	14149			y	
111	HMDB0000860	pro	1	5	2	16	2	75	3	4529	4	12133	10	30842	1	2656	y	
112		sod	1	5	1	10	1	29	1	4599	1	14415	1	30950			y	
113		dep	1	8	1	10	1	14	2	2707	2	6748	2	14149			Y	
114	HMDB0000873	pro	11	11	11	20	11	26	3094	4529	3246	12133	3595	30842	48	2656		
115		sod	1	12	1	19	1	29	13	4599	8	14415	12	30950			y	
116		dep	1	2	1	7	1	47	22	2707	57	6748	25	14149			y	
117	HMDB0000904	pro	1	2	1	8	1	39	13	4529	13	12133	9	30842	2	2656	y	
118		sod	1	2	1	3	1	40	4	4599	4	14415	7	30950			y	
119		dep	2	5	2	12	1	29	1352	2707	976	6748	10	14149				
120	HMDB0000929	pro	1	6	1	19	1	62	1	4529	1	12133	2	30842	21	2656	y	
121		sod	1	5	1	13	2	40	9	4599	9	14415	25	30950			y	
122	HMDB0000930	dep	1	6	1	6	1	6	51	2707	78	6748	113	14149	60	2733	y	
123		sod	4	8	2	13	2	19	1001	4599	227	14415	369	30950				
124	HMDB0000959	pro	3	4	1	10	1	56	323	4529	9	12133	9	30842	323	4529		

Table S4 (continued). *Rank of the spectrum and number of queried spectra for each search (type and energy tolerance). The last 2 columns show whether the spectrum was used in the grid search (to optimize the parameters for the spectral similarity scoring) and the literature source (if any).*

		ion	isomer search						unsupervised search						combined		grid	
#	HMDB ID	form	0 kJ/mol		10 kJ/mol		no energy		0 kJ/mol		10 kJ/mol		no energy		0 kJ/mol		search?	lit.
125		dep	3	9	1	27	1	38	46	2707	1	6748	1	14149				
126	HMDB0001149	pro	1	9	1	18	1	69	1	4529	2	12133	7	30842	1	2656	y	
127		sod	2	9	1	21	1	76	120	4599	15	14415	30	30950				
128	HMDB0001232	dep	1	4	1	6	1	10	10	2707	11	6748	15	14149	3	2675		
129		pro	1	4	1	4	1	9	32	4529	50	12133	74	30842				
130	HMDB0001336	dep	1	16	1	34	1	54	1	2707	1	6748	2	14149	1	2733	y	19
131		sod	1	18	1	42	1	98	2	4599	3	14415	7	30950			y	
132	HMDB0001859	pro	1	9	1	12	1	32	6	4529	7	12133	6	30842	1	4536	y	
133		sod	1	9	1	17	1	36	1	4599	1	14415	1	30950			y	
134		dep	1	4	1	5	1	8	4	2707	7	6748	9	14149			y	
135	HMDB0001886	pro	3	4	3	4	3	7	749	4529	1020	12133	1494	30842	4	2656		
136		sod	2	4	2	5	1	14	9	4599	12	14415	1	30950			y	
137		dep	1	1	1	3	1	12	4	2707	5	6748	6	14149			y	
138	HMDB0001890	pro	1	1	1	5	1	16	413	4529	168	12133	312	30842	7	2656		
139		sod	1	1	1	4	1	12	10	4599	9	14415	11	30950			y	
140		dep	2	11	4	36	4	67	113	2707	88	6748	151	14149				
141	HMDB0001964	pro	1	11	1	19	2	50	187	4529	292	12133	541	30842	25	2656		
142		sod	1	11	1	38	1	67	10	4599	14	14415	18	30950			y	
143	HMDB0002266	pro	1	8	1	16	1	44	1	4529	1	12133	4	30842	1	4529	y	
144	HMDB0002285	dep	1	5	1	7	1	14	69	2707	122	6748	13	14149	5	2675	y	
145		pro	1	5	1	6	1	10	24	4529	34	12133	80	30842			y	
146		dep	1	2	1	4	1	9	562	2707	674	6748	52	14149				
147	HMDB0002302	pro	2	3	2	3	2	14	2901	4529	3254	12133	994	30842	489	2656		
148		sod	1	3	1	3	1	6	28	4599	52	14415	112	30950			y	
149		dep	1	7	1	8	1	15	3	2707	3	6748	3	14149			y	
150	HMDB0002432	pro	1	7	1	12	1	20	1	4529	1	12133	1	30842	1	2656	y	
151		sod	1	7	1	10	1	26	70	4599	112	14415	16	30950				
152	HMDB0002825	pro	1	3	1	4	1	4	102	4529	143	12133	302	30842	1	4536		
153		sod	1	3	1	3	1	10	1	4599	1	14415	1	30950			y	
154		dep	1	4	1	4	1	8	4	2707	5	6748	8	14149			y	
155	HMDB0003152	pro	1	6	1	8	1	26	1	4529	1	12133	2	30842	1	2656	y	
156		sod	1	6	1	6	1	12	2	4599	3	14415	5	30950			y	
157	HMDB0003320	dep	1	5	1	7	1	14	2	2707	5	6748	9	14149	2	2675	y	
158		pro	2	5	2	6	2	10	92	4529	127	12133	232	30842				
159	HMDB0003633	dep	1	9	1	17	1	21	5	2707	7	6748	7	14149	2	2675	y	
160		pro	1	12	1	28	1	35	2	4529	1	12133	1	30842			y	
161		dep	1	2	1	4	1	11	1	2707	1	6748	1	14149			y	
162	HMDB0004095	pro	1	2	1	4	1	8	1	4529	1	12133	1	30842	1	2656	y	
163		sod	1	2	1	7	1	16	1	4599	1	14415	1	30950			y	
164	HMDB0005807	dep	1	5	1	7	1	22	5	2707	5	6748	1	14149	1	2675	y	
165		pro	1	5	1	6	1	12	1	4529	1	12133	1	30842			y	
166	HMDB0012128	pro	1	5	1	16	1	17	11	4529	17	12133	32	30842	11	4529	y	
167	HMDB0012140	dep	1	4	1	13	1	29	6	2707	9	6748	19	14149	6	2707	y	
168	HMDB0013318	dep	1	2	1	11	1	14	13	2707	1	6748	1	14149	1	2675	y	
169		pro	1	2	1	4	1	17	1	4529	1	12133	2	30842			y	
170		dep	4	6	4	7	4	11	440	2707	622	6748	754	14149				
171	HMDB0014389	pro	1	6	1	7	1	16	7	4529	12	12133	5	30842	2	2656	y	
172		sod	1	6	1	12	1	38	2	4599	1	14415	4	30950			y	
173	HMDB0015517	pro	1	6	1	25	1	32	16	4529	22	12133	33	30842	16	4529	y	
174	HMDB0028850	pro	1	2	2	5	1	35	6	4529	8	12133	2	30842	6	4529	y	
175	HMDB0031861	pro	1	2	1	3	1	4	3	4529	5	12133	10	30842	3	4529	y	
176	HMDB0036458	pro	1	3	1	4	1	6	2	4529	3	12133	3	30842	1	4536	y	
177		sod	1	3	1	4	1	9	14	4599	23	14415	65	30950				
178	HMDB0041923	pro	1	9	1	20	1	31	1	4529	2	12133	5	30842	1	4529	y	21
179	HMDB0041931	pro	1	9	1	15	1	34	1	4529	1	12133	1	30842	1	4529	y	21
180		dep	1	6	3	24	3	60	5	2707	14	6748	12	14149			y	
181	HMDB0059720	pro	1	6	1	14	2	43	3	4529	3	12133	4	30842	1	2656	y	
182		sod	1	6	1	23	1	57	14	4599	25	14415	26	30950			y	

Table S4 (continued). *Rank of the spectrum and number of queried spectra for each search (type and energy tolerance). The last 2 columns show whether the spectrum was used in the grid search (to optimize the parameters for the spectral similarity scoring) and the literature source (if any).*

		ion	isomer search						unsupervised search						combined		grid	
**#**	**HMDB ID**	**form**	**0 kJ/mol**		**10 kJ/mol**		**no energy**		**0 kJ/mol**		**10 kJ/mol**		**no energy**		**0 kJ/mol**		**search?**	**lit.**
183		dep	3	5	3	12	3	36	38	2707	44	6748	49	14149			y	
184	HMDB0060608	pro	1	5	1	16	1	75	1	4529	1	12133	1	30842	1	2656	y	
185		sod	1	5	1	10	1	29	1	4599	1	14415	1	30950			y	
186		dep	1	3	1	7	1	11	9	2707	25	6748	55	14149			y	
187	HMDB0061705	pro	2	4	1	6	1	38	170	4529	11	12133	30	30842	21	2656		
188		sod	1	4	1	11	2	32	183	4599	263	14415	299	30950				
189	HMDB0094701	pro	1	4	1	10	1	56	2	4529	3	12133	7	30842	2	4529	y	
	**rank product (RP)**		**1.30**		**1.30**		**1.35**		**18.2**		**17.0**		**19.0**		**7.6**			
	**% ranked first**		**75%**		**75%**		**74%**		**19%**		**21%**		**17%**		**32%**			

Discussion on different adducts

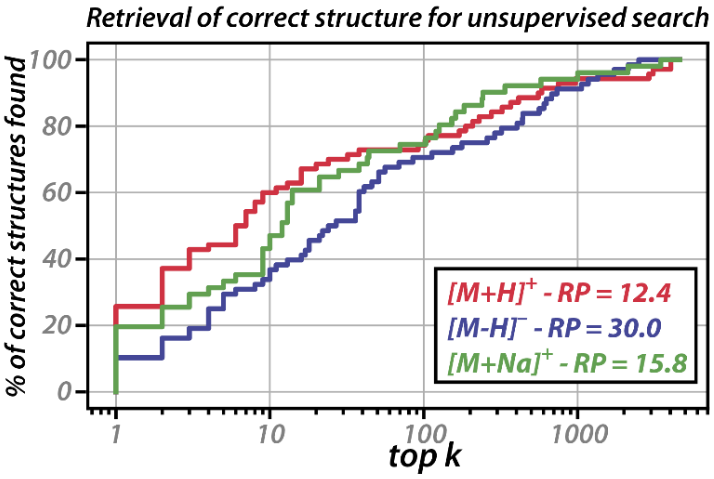

Figure S6. *Percentage of correct structures found in the top k output when performing an unsupervised search. Results for protonated ions are in red (N=70), for deprotonated ions in blue (N=68) and for sodiated ions in green (N=51).*

It is observed from Figure S6 that $[M+H]^+$ ions are best retrieved from the spectral library, followed by $[M+Na]^+$ and then $[M-H]^-$. A major contribution to this trend is the quality of the experimental IR spectra for the different adducts. Particularly, the $[M-H]^-$ ions appear to show more spectral broadening than the other two adducts (*e.g.*, see Figure S13 below). The molecular classes that are typically measured as $[M-H]^-$ ions, such as organic oxygen compounds (mostly carbohydrates here) and organic acids, have more dynamic structures at room temperature and therefore display broader spectral features. Shared protons are an extreme example of these dynamics, which may result in a completely different IR spectrum than the computed harmonic spectrum, as can be seen for deprotonated L-aspartic acid in Figure S13 below.

One explanation for the slightly poorer performance of sodiated ions is that some metabolites appear to assume a zwitterionic form when complexed with a sodium cation (L-pipecolic acid, ornithine and 6-oxopiperidine-2-carboxylic acid). These zwitterionic forms are not explicitly considered in the computational workflow, although they may be generated during the geometry optimizations in Gaussian16 (see ornithine). The resulting spectra therefore match poorly to the computed spectra, because zwitterionic geometries are not considered.

From a practical point of view, we recommend measuring protonated ions where possible. Sodiated ions, although they are retrieved reasonably as well, are experimentally more challenging, because they often detach the sodium cation upon IRMPD. Since the *m/z* of Na^+ lies below the low-mass cut-off of the ion trap, this requires one to measure these spectra as depletion spectra, which typically have lower quality. Ideally, spectra of multiple ions forms are acquired to improve specificity (see Figure 4 and discussion in main text). Obviously, the observed ion intensities are a strong factor in selecting the preferred adduct to be measured, and the choice is often limited.

Limited presence of structural analogues

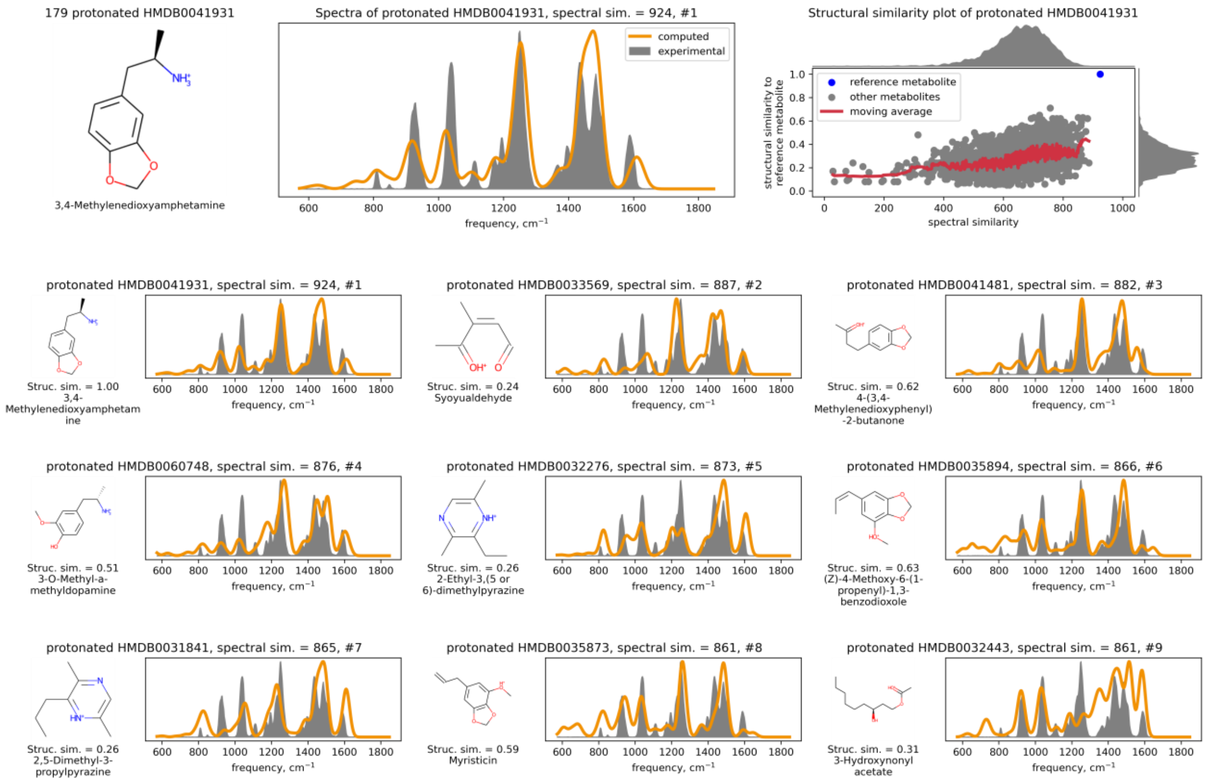

Figure S7. *Spectral comparisons of the computed vibrational spectra (orange) of 9 protonated entries in the in silico library with the experimental IR spectrum (gray) of protonated 3,4-methylenedioxyamphetamine ([MDA+H]$^+$). For each library entry the spectral similarity with the experiment is given, as well as the structural similarity of the protonated ion with the structure of [MDA+H]$^+$. The top right shows the relation between spectral and structural similarity for all entries in the library.*

Observation of higher-energy geometries

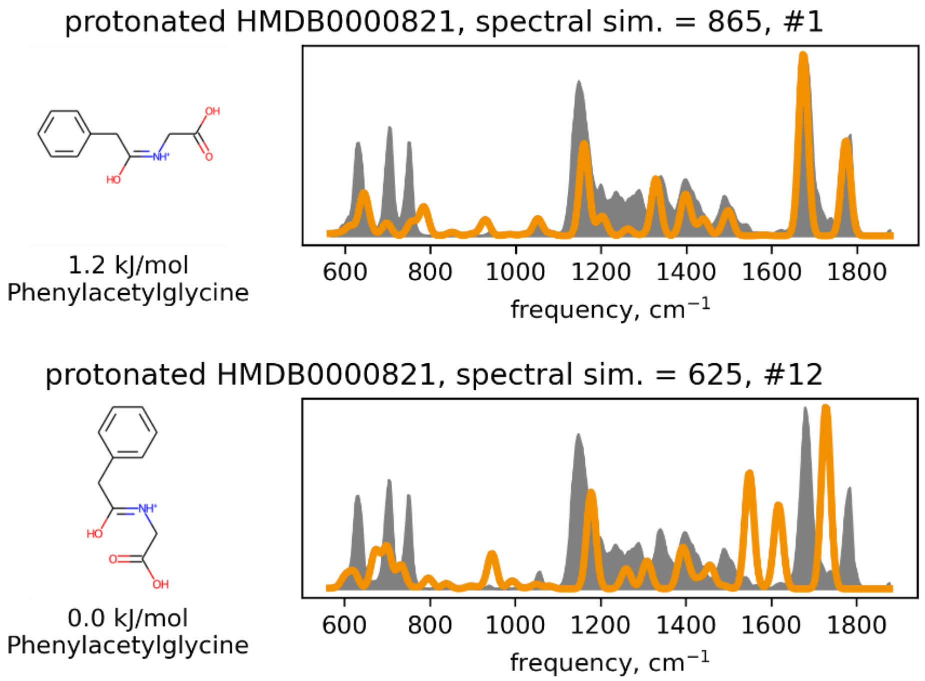

Figure S8. *Spectral comparison of computed vibrational spectra (orange) of two conformers of [Phenylacetylglgycine+H]$^+$ with the experimental IR spectrum (gray) of [Phenylacetylglgycine+H]$^+$. The ion population mostly consists of the conformer with a higher computed energy of 1.2 kJ/mol (top).*

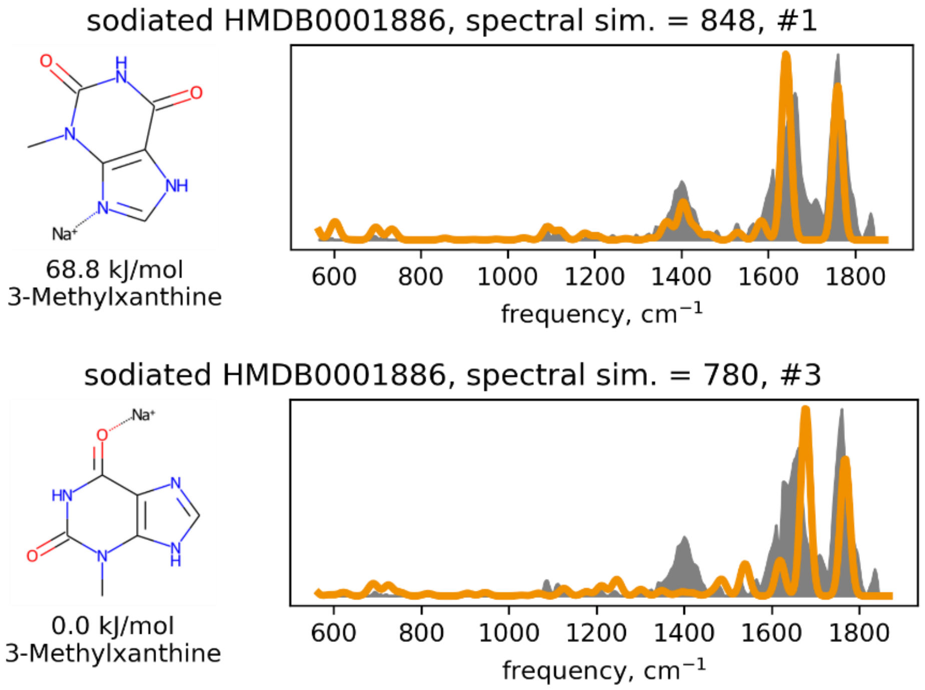

Figure S9. *Spectral comparison of computed vibrational spectra (orange) of two tautomers of [3-methylxanthine+Na]$^+$ with the experimental IR spectrum (gray) of [3-methylxanthine+Na]$^+$. The ion population mostly consists of the tautomer with a higher computed energy of 68.8 kJ/mol (top).*

Observation of isomeric mixtures

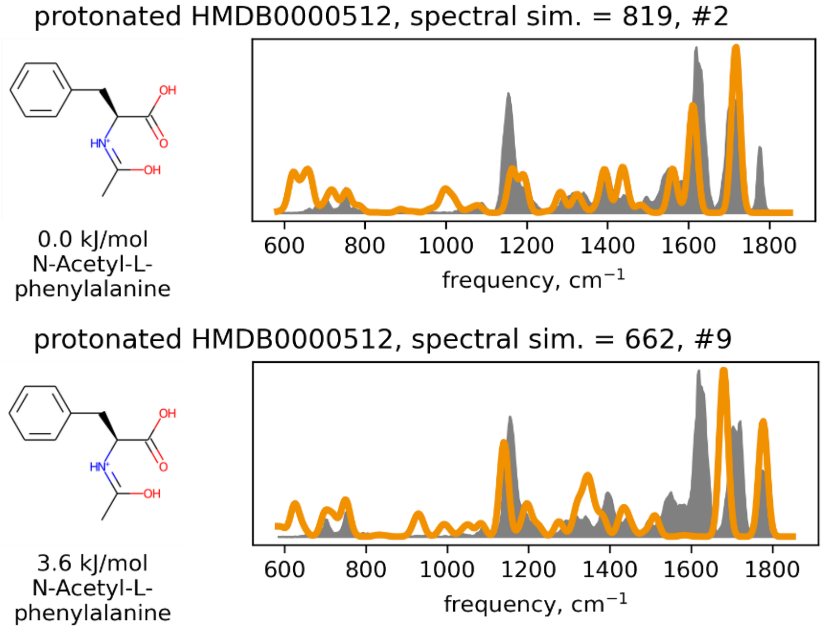

Figure S10. *Spectral comparison of computed vibrational spectra (orange) of two conformers of [N-acetyl-L-phenylalanine+H]$^+$ with the experimental IR spectrum (grey) of [N-acetyl-L-phenylalanine+H]$^+$. Based on the IR spectrum the ion population is a mix of the lowest-energy and a higher-energy conformer at 3.6 kJ/mol.*

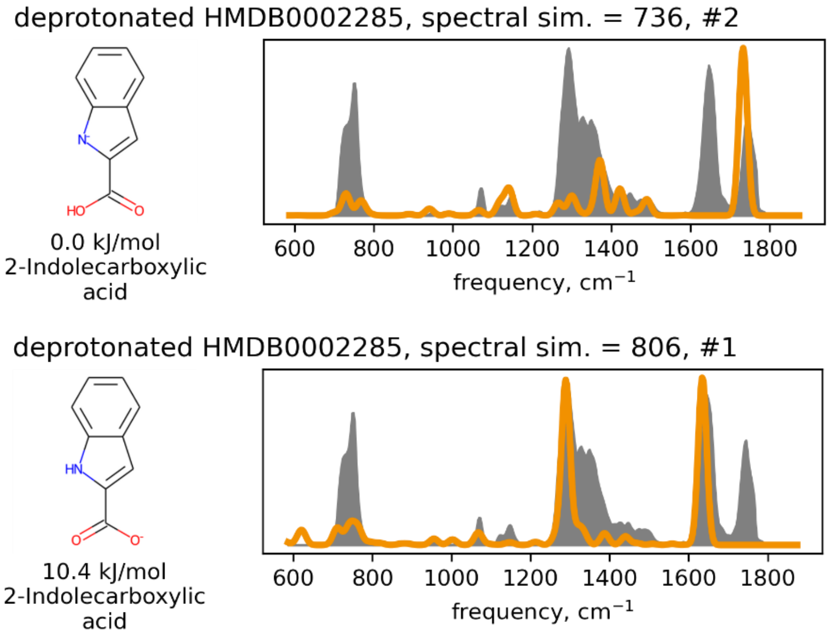

Figure S11. *Spectral comparison of computed vibrational spectra (orange) of two conformers of [2-indolecarboxylic acid-H]$^-$ with the experimental IR spectrum (grey) of [2-indolecarboxylic acid-H]$^-$. Based on the IR spectrum the ion population is a mix of the lowest-energy and a higher-energy conformer at 10.4 kJ/mol.*

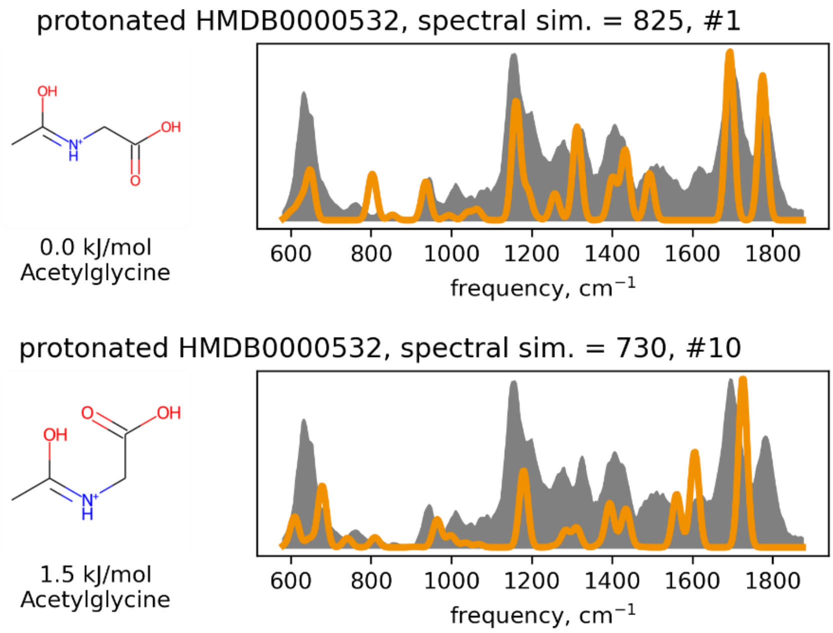

Figure S12. *Spectral comparison of computed vibrational spectra (orange) of two conformers of [Acetylglycine+H]$^{+}$ with the experimental IR spectrum (grey) of [Acetylglycine+H]$^{+}$. Based on the IR spectrum the ion population is a mix of the lowest-energy and a higher-energy conformer at 1.5 kJ/mol.*

Effect of adduct on spectral broadening

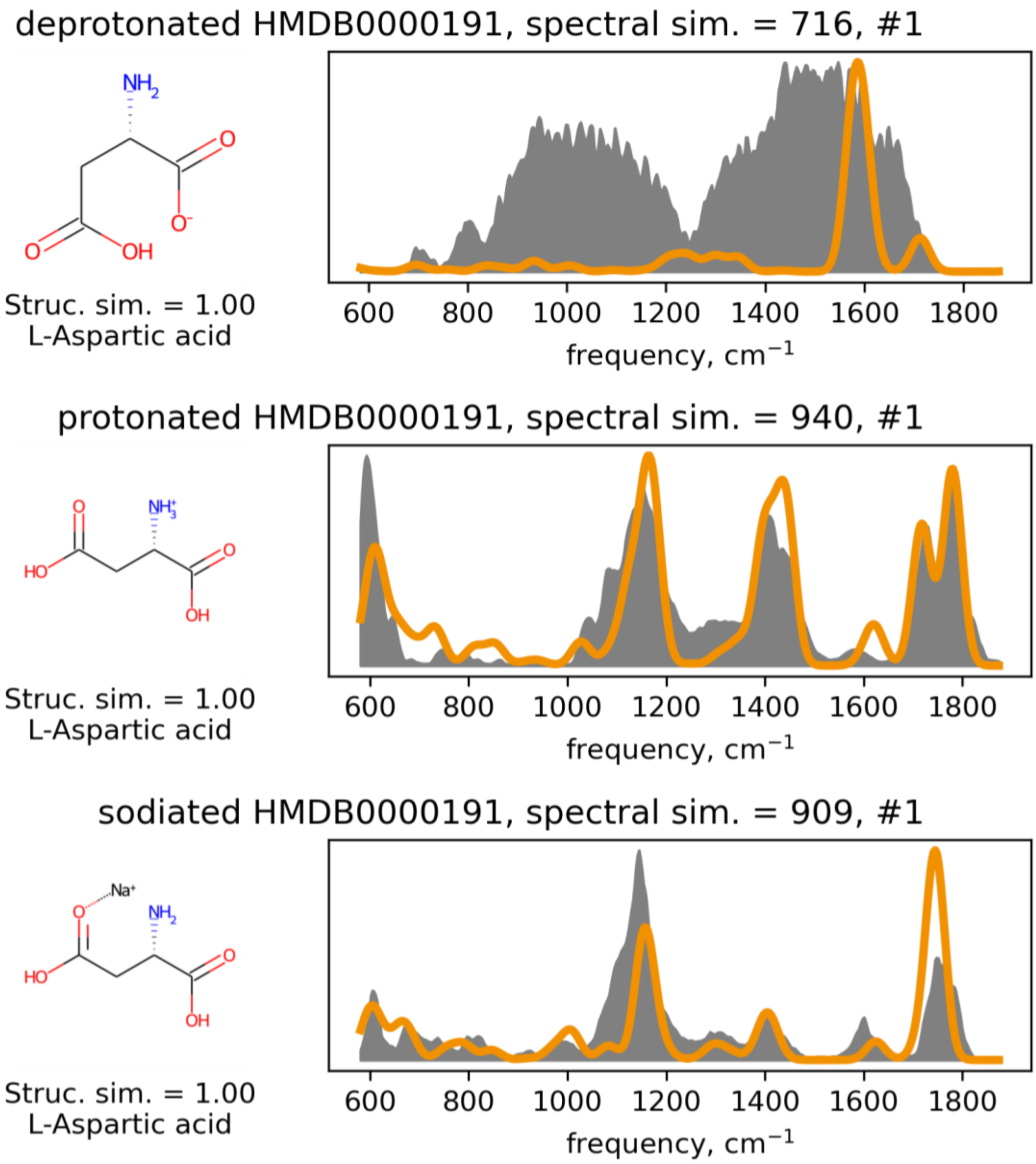

Figure S13. *Spectral comparison of computed vibrational spectrum (orange) and experimental IR spectrum (grey) of [L-aspartic acid-H]⁻ (top), [L-aspartic acid+H]⁺ (middle) and [L-aspartic acid+Na]⁺ (bottom). The deprotonated ion shows severe spectral broadening as a consequence of a strongly delocalized proton; molecular-dynamics modeling of the IR spectra of such shared-proton systems at room temperature rationalize the observation of increased bandwidths, which is not captured in static zero-Kelvin frequency calculations.[22,23] The protonated and sodiated spectra show well resolved bands.*

Unknown identification from a patient sample

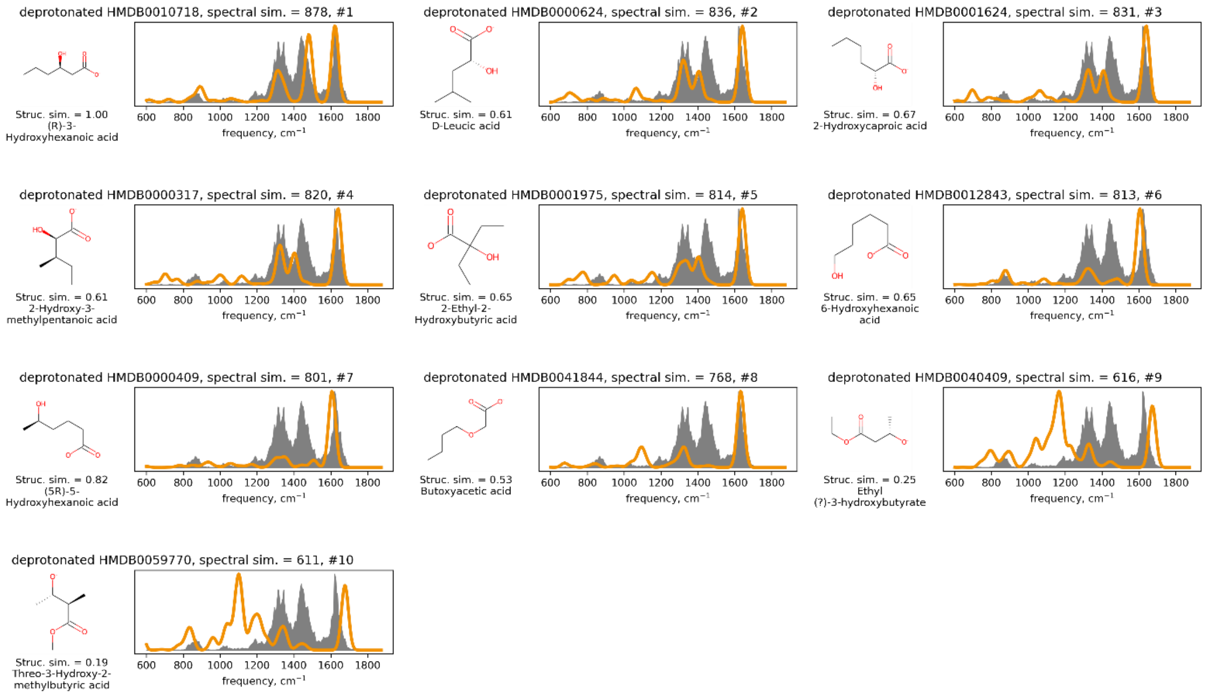

Figure S14. *Spectral comparisons of the computed vibrational spectra of 10 deprotonated entries in the in silico library with formula* $[C_6H_{12}O_3\text{-}H]^-$ *against the experimental IR spectrum of the unknown LC-MS feature (grey). For each library entry, the spectral similarity with the experiment is given, as well as the structural similarity of the deprotonated structure with the structure of the assigned (R)-3-hydroxyhexanoic acid.*

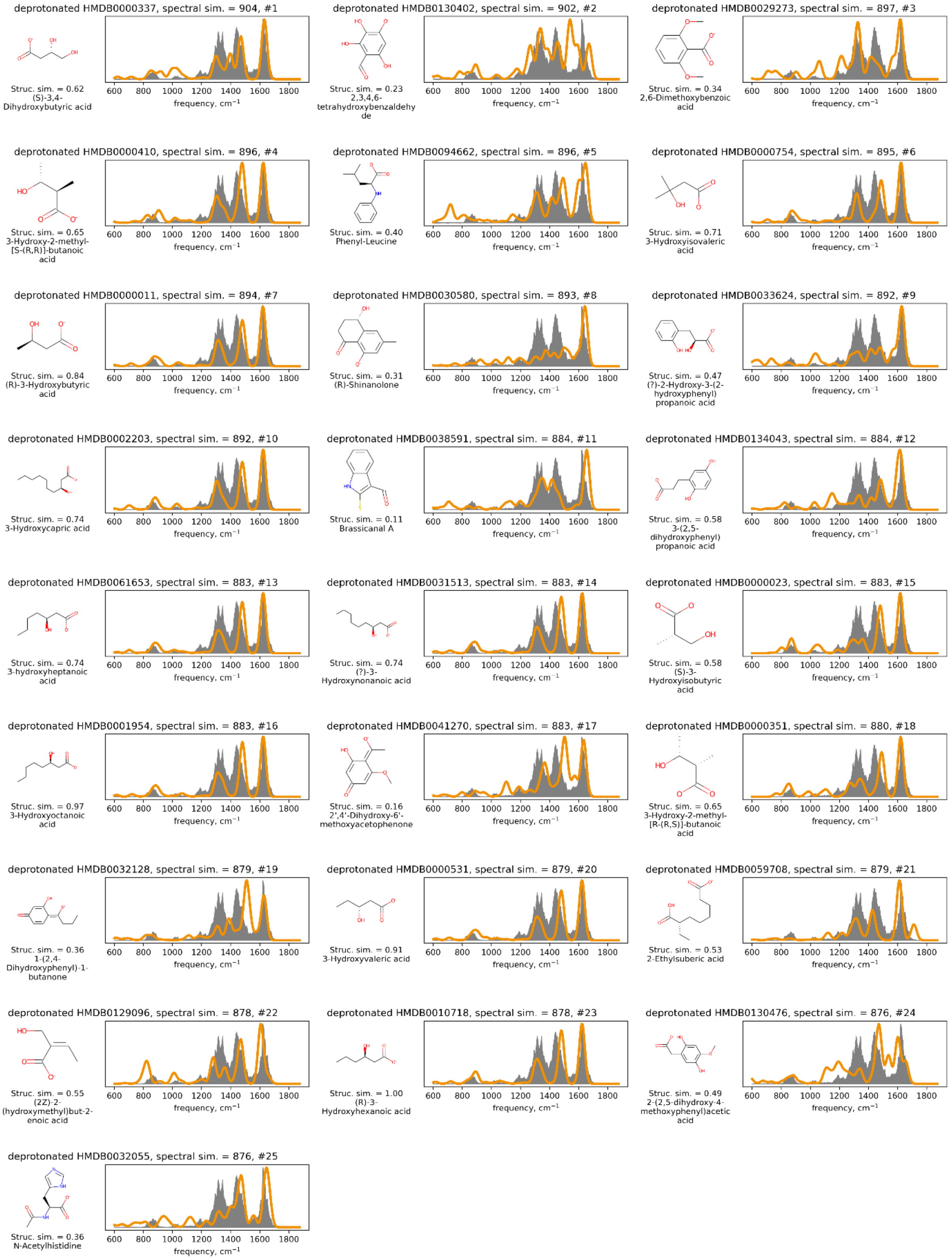

Figure S15. *Spectral comparisons of the computed vibrational spectra of 25 deprotonated entries in the in silico library (without m/z constraint) that match best with the experimental IR spectrum of the unknown LC-MS feature (grey). For each library entry the spectral similarity with the experiment is given, as well as the structural similarity of the deprotonated structure with the structure of the assigned (R)-3-hydroxyhexanoic acid.*

References

1. Martens, J.; Berden, G.; Gebhardt, C. R.; Oomens, J., Infrared ion spectroscopy in a modified quadrupole ion trap mass spectrometer at the FELIX free electron laser laboratory. *Rev. Sci. Instrum.* **2016,** *87*, 103108.
2. Oepts, D.; Van der Meer, A. F. G.; Van Amersfoort, P. W., The free-electron-laser user facility FELIX. *Infrared Phys. Technol.* **1995,** *36*, 297-308.
3. van Outersterp, R. E.; Engelke, U. F. H.; Merx, J.; Berden, G.; Paul, M.; Thomulka, T.; Berkessel, A.; Huigen, M. C. D. G.; Kluijtmans, L. A.; Mecinović, J.; Rutjes, F. P.; Van Karnebeek, C. D. M.; Wevers, R. A.; Boltje, T. J.; Coene, K. L. M.; Martens, J.; Oomens, J., Metabolite Identification Using Infrared Ion Spectroscopy– Novel Biomarkers for Pyridoxine-Dependent Epilepsy. *Anal. Chem.* **2021,** *93*, 15340-15348.
4. van Outersterp, R. E.; Oosterhout, J.; Gebhardt, C. R.; Berden, G.; Engelke, U. F.; Wevers, R. A.; Cuyckens, F.; Oomens, J.; Martens, J., Targeted small molecule identification using heartcutting liquid chromatography–infrared ion spectroscopy. *Anal. Chem.* **2023,** *95*, 3406-3413.
5. Berden, G.; Derksen, M.; Houthuijs, K. J.; Martens, J.; Oomens, J., An automatic variable laser attenuator for IRMPD spectroscopy and analysis of power-dependence in fragmentation spectra. *Int. J. Mass spectrom.* **2019,** *443*, 1-8.
6. Landrum, G., RDKit: Open-source cheminformatics. **2006**.
7. Frisch, M. J.; Trucks, G. W.; Schlegel, H. B.; Scuseria, G. E.; Robb, M. A.; Cheeseman, J. R.; Scalmani, G.; Barone, V.; Petersson, G. A.; Nakatsuji, H.; Li, X.; Caricato, M.; Marenich, A. V.; Bloino, J.; Janesko, B. G.; Gomperts, R.; Mennucci, B.; Hratchian, H. P.; Ortiz, J. V.; Izmaylov, A. F.; Sonnenberg, J. L.; Williams; Ding, F.; Lipparini, F.; Egidi, F.; Goings, J.; Peng, B.; Petrone, A.; Henderson, T.; Ranasinghe, D.; Zakrzewski, V. G.; Gao, J.; Rega, N.; Zheng, G.; Liang, W.; Hada, M.; Ehara, M.; Toyota, K.; Fukuda, R.; Hasegawa, J.; Ishida, M.; Nakajima, T.; Honda, Y.; Kitao, O.; Nakai, H.; Vreven, T.; Throssell, K.; Montgomery Jr., J. A.; Peralta, J. E.; Ogliaro, F.; Bearpark, M. J.; Heyd, J. J.; Brothers, E. N.; Kudin, K. N.; Staroverov, V. N.; Keith, T. A.; Kobayashi, R.; Normand, J.; Raghavachari, K.; Rendell, A. P.; Burant, J. C.; Iyengar, S. S.; Tomasi, J.; Cossi, M.; Millam, J. M.; Klene, M.; Adamo, C.; Cammi, R.; Ochterski, J. W.; Martin, R. L.; Morokuma, K.; Farkas, O.; Foresman, J. B.; Fox, D. J. *Gaussian 16 Rev. C.01*, Wallingford, CT, 2016.
8. Pracht, P.; Grant, D. F.; Grimme, S., Comprehensive assessment of GFN tight-binding and composite density functional theory methods for calculating gas-phase infrared spectra. *J. Chem. Theory Comput.* **2020,** *16*, 7044-7060.
9. Kempkes, L. J. M.; Martens, J.; Berden, G.; Houthuijs, K. J.; Oomens, J., Investigation of the position of the radical in z 3-ions resulting from electron transfer dissociation using infrared ion spectroscopy. *Faraday Discuss.* **2019,** *217*, 434-452.
10. Stein, S. E.; Scott, D. R., Optimization and testing of mass spectral library search algorithms for compound identification. *J. Am. Soc. Mass. Spectrom.* **1994,** *5*, 859-866.
11. Rogers, D.; Hahn, M., Extended-connectivity fingerprints. *J. Chem. Inf. Model.* **2010,** *50*, 742-754.
12. Huber, F.; Ridder, L.; Verhoeven, S.; Spaaks, J. H.; Diblen, F.; Rogers, S.; van der Hooft, J. J. J., Spec2Vec: Improved mass spectral similarity scoring through learning of structural relationships. *PLoS Comput. Biol.* **2021,** *17*, e1008724.
13. Coene, K. L. M.; Kluijtmans, L. A. J.; van der Heeft, E.; Engelke, U. F. H.; de Boer, S.; Hoegen, B.; Kwast, H. J. T.; van de Vorst, M.; Huigen, M. C. D. G.; Keularts, I. M. L. W.; Schreuder, M. F.; Van Karnebeek, C. D. M.; Wortmann, S. B.; de Vries, M. C.; Janssen, M. C. H.; Gilissen, C.; Engel, J.; Wevers, R. A., Next-generation metabolic screening: targeted and untargeted metabolomics for the diagnosis of inborn errors of metabolism in individual patients. *J. Inherit. Metab. Dis.* **2018,** *41*, 337-353.
14. Tautenhahn, R.; Patti, G. J.; Rinehart, D.; Siuzdak, G., XCMS Online: a web-based platform to process untargeted metabolomic data. *Anal. Chem.* **2012,** *84*, 5035-5039.

15. Breitling, R.; Armengaud, P.; Amtmann, A.; Herzyk, P., Rank products: a simple, yet powerful, new method to detect differentially regulated genes in replicated microarray experiments. *FEBS Lett.* **2004,** *573*, 83-92.
16. Grimme, S.; Ehrlich, S.; Goerigk, L., Effect of the damping function in dispersion corrected density functional theory. *J. Comput. Chem.* **2011,** *32*, 1456-1465.
17. Djoumbou Feunang, Y.; Eisner, R.; Knox, C.; Chepelev, L.; Hastings, J.; Owen, G.; Fahy, E.; Steinbeck, C.; Subramanian, S.; Bolton, E.; Wishart, D. S.; Greiner, R., ClassyFire: automated chemical classification with a comprehensive, computable taxonomy. *J. Cheminform.* **2016,** *8*, 1-20.
18. van Outersterp, R. E.; Houthuijs, K. J.; Berden, G.; Engelke, U. F.; Kluijtmans, L. A. J.; Wevers, R. A.; Coene, K. L. M.; Oomens, J.; Martens, J., Reference-standard free metabolite identification using infrared ion spectroscopy. *Int. J. Mass spectrom.* **2019,** *443*, 77-85.
19. Martens, J.; van Outersterp, R. E.; Vreeken, R. J.; Cuyckens, F.; Coene, K. L. M.; Engelke, U. F.; Kluijtmans, L. A. J.; Wevers, R. A.; Buydens, L. M. C.; Redlich, B.; Berden, G.; Oomens, J., Infrared ion spectroscopy: New opportunities for small-molecule identification in mass spectrometry-A tutorial perspective. *Anal. Chim. Acta* **2020,** *1093*, 1-15.
20. Martens, J.; Berden, G.; Bentlage, H.; Coene, K. L. M.; Engelke, U. F. H.; Wishart, D. S.; van Scherpenzeel, M.; Kluijtmans, L. A. J.; Wevers, R. A.; Oomens, J., Unraveling the unknown areas of the human metabolome: the role of infrared ion spectroscopy. *J. Inherit. Metab. Dis.* **2018,** *41*, 367-377.
21. Kranenburg, R. F.; van Geenen, F. A. M. G.; Berden, G.; Oomens, J.; Martens, J.; van Asten, A. C., Mass-spectrometry-based identification of synthetic drug isomers using infrared ion spectroscopy. *Anal. Chem.* **2020,** *92*, 7282-7288.
22. Li, X.; Moore, D. T.; Iyengar, S. S., Insights from first principles molecular dynamics studies toward infrared multiple-photon and single-photon action spectroscopy: Case study of the proton-bound dimethyl ether dimer. *J. Chem. Phys.* **2008,** *128*, 184308.
23. Martínez-Haya, B.; Avilés-Moreno, J. R.; Gámez, F.; Martens, J.; Oomens, J.; Berden, G., A Dynamic Proton Bond: $MH^{+}{\cdot}H_2O \rightleftharpoons M{\cdot}H_3O^{+}$ Interconversion in Loosely Coordinated Environments. *J. Phys. Chem. Lett.* **2023,** *14*, 1294-1300.
