# Supplementary material for: An In Silico Infrared Spectral Library of Molecular Ions for Metabolite Identification
Source: Anal Chem. 2023 Jun 1;95(23):8998–9005. doi: 10.1021/acs.analchem.3c01078 (PMC10267894; doi:10.1021/acs.analchem.3c01078)
Supplement: Supplementary file 2 — ac3c01078_si_002.pdf [file ac3c01078_si_002.pdf]

1 protonated HMDB0000062

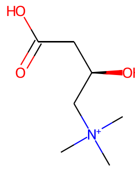


L-Carnitine

Spectra of protonated HMDB0000062, spectral sim. = 779, #1

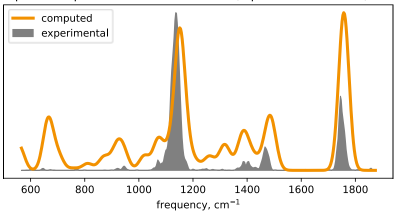


Structural similarity plot of protonated HMDB0000062

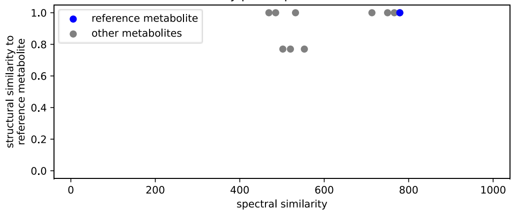


protonated HMDB0000062, spectral sim. = 779, #1

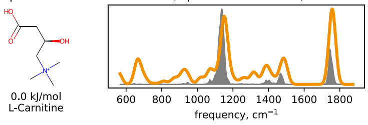


protonated HMDB0000062, spectral sim. = 766, #2

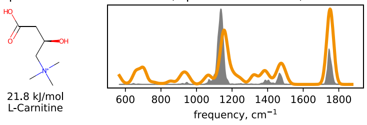


protonated HMDB0000062, spectral sim. = 750, #3

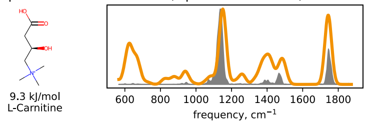


protonated HMDB0000062, spectral sim. = 713, #4

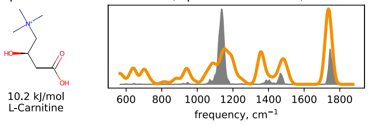


protonated HMDB0000062, spectral sim. = 553, #5

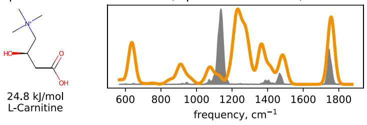


protonated HMDB0000062, spectral sim. = 532, #6

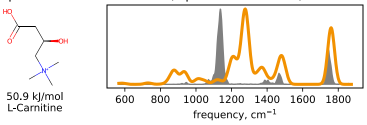


protonated HMDB0000062, spectral sim. = 520, #7

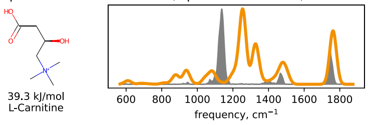


protonated HMDB0000062, spectral sim. = 502, #8

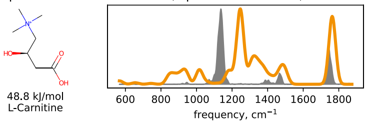


protonated HMDB0000062, spectral sim. = 485, #9

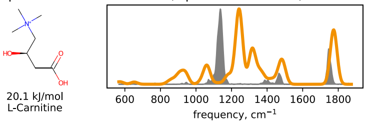


protonated HMDB0000062, spectral sim. = 469, #10

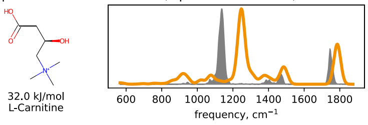


2 sodiated HMDB0000062

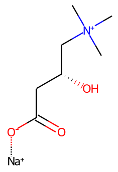


L-Carnitine

Spectra of sodiated HMDB0000062, spectral sim. = 914, #1

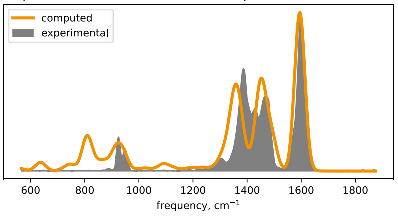


Structural similarity plot of sodiated HMDB0000062

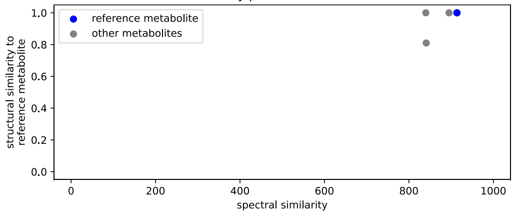


sodiated HMDB0000062, spectral sim. = 914, #1

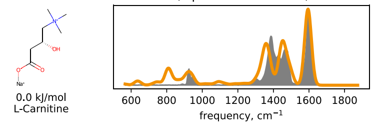


sodiated HMDB0000062, spectral sim. = 913, #2

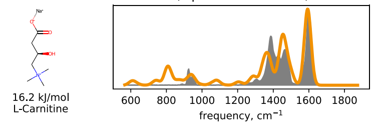


sodiated HMDB0000062, spectral sim. = 895, #3

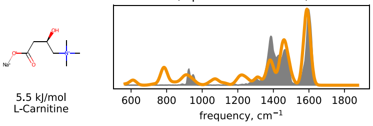


sodiated HMDB0000062, spectral sim. = 841, #4

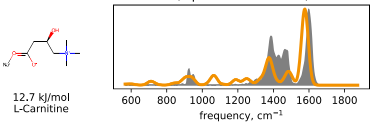


sodiated HMDB0000062, spectral sim. = 840, #5

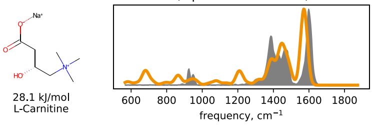

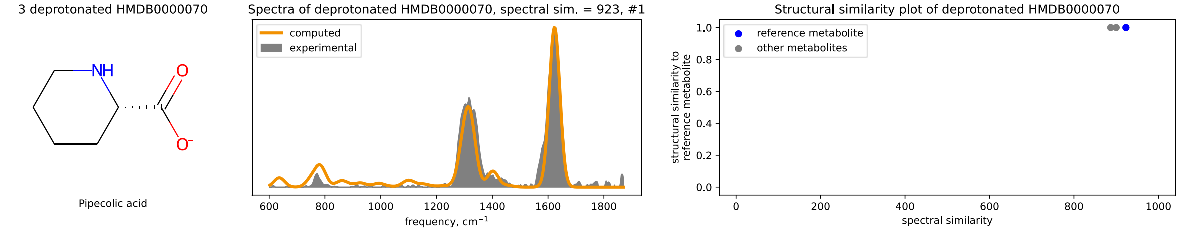
3 deprotonated HMDB0000070
Pipecolic acid
Spectra of deprotonated HMDB0000070, spectral sim. = 923, #1
computed
experimental
frequency, cm⁻¹
Structural similarity plot of deprotonated HMDB0000070
reference metabolite
other metabolites
structural similarity to reference metabolite
spectral similarity

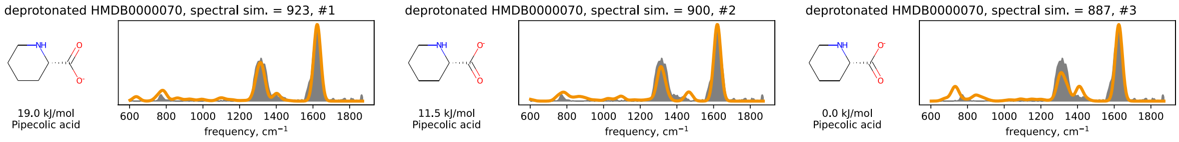
deprotonated HMDB0000070, spectral sim. = 923, #1
19.0 kJ/mol
Pipecolic acid
deprotonated HMDB0000070, spectral sim. = 900, #2
11.5 kJ/mol
Pipecolic acid
deprotonated HMDB0000070, spectral sim. = 887, #3
0.0 kJ/mol
Pipecolic acid
frequency, cm⁻¹

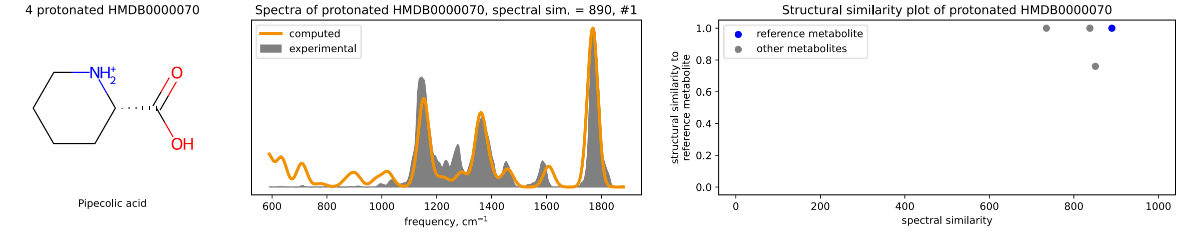
4 protonated HMDB0000070
Pipecolic acid
Spectra of protonated HMDB0000070, spectral sim. = 890, #1
computed
experimental
frequency, cm⁻¹
Structural similarity plot of protonated HMDB0000070
reference metabolite
other metabolites
structural similarity to reference metabolite
spectral similarity

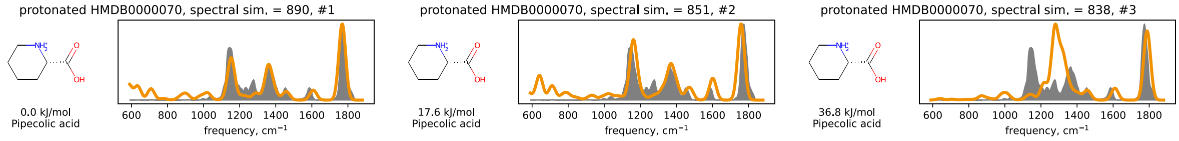
protonated HMDB0000070, spectral sim. = 890, #1
0.0 kJ/mol
Pipecolic acid
protonated HMDB0000070, spectral sim. = 851, #2
17.6 kJ/mol
Pipecolic acid
protonated HMDB0000070, spectral sim. = 838, #3
36.8 kJ/mol
Pipecolic acid
frequency, cm⁻¹

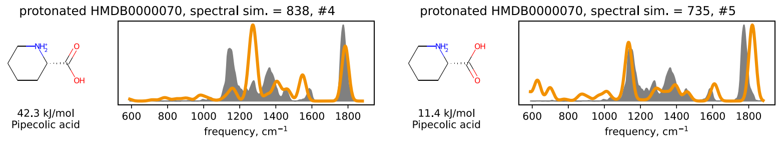
protonated HMDB0000070, spectral sim. = 838, #4
42.3 kJ/mol
Pipecolic acid
protonated HMDB0000070, spectral sim. = 735, #5
11.4 kJ/mol
Pipecolic acid
frequency, cm⁻¹

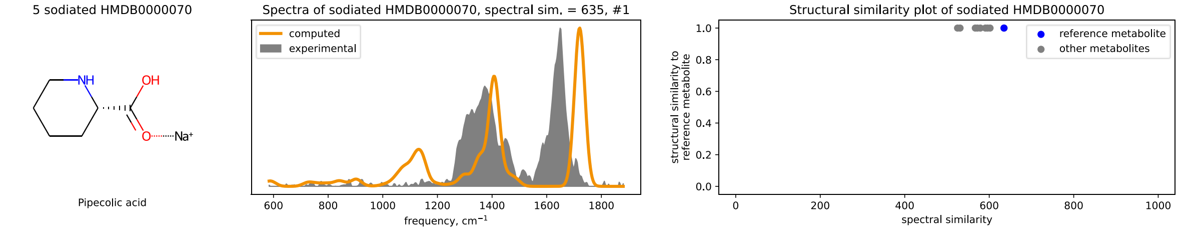
5 sodiated HMDB0000070
Pipecolic acid
Spectra of sodiated HMDB0000070, spectral sim. = 635, #1
computed
experimental
frequency, cm⁻¹
Structural similarity plot of sodiated HMDB0000070
reference metabolite
other metabolites
structural similarity to reference metabolite
spectral similarity

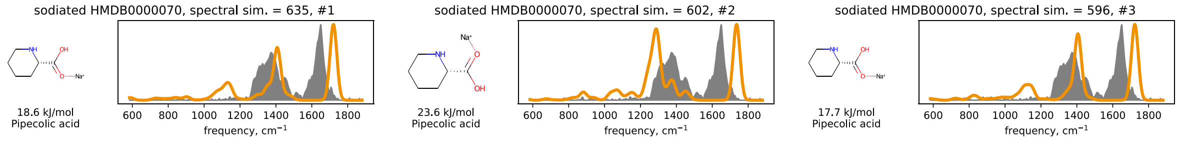
sodiated HMDB0000070, spectral sim. = 635, #1
18.6 kJ/mol
Pipecolic acid
sodiated HMDB0000070, spectral sim. = 602, #2
23.6 kJ/mol
Pipecolic acid
sodiated HMDB0000070, spectral sim. = 596, #3
17.7 kJ/mol
Pipecolic acid
frequency, cm⁻¹

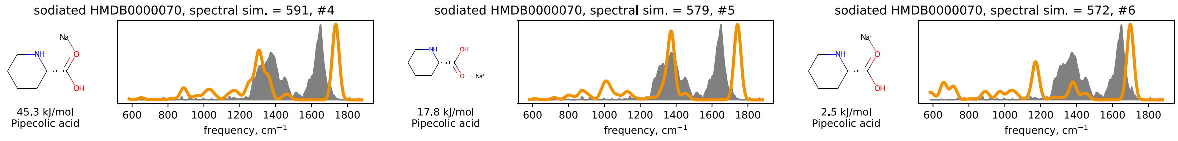
sodiated HMDB0000070, spectral sim. = 591, #4
45.3 kJ/mol
Pipecolic acid
sodiated HMDB0000070, spectral sim. = 579, #5
17.8 kJ/mol
Pipecolic acid
sodiated HMDB0000070, spectral sim. = 572, #6
2.5 kJ/mol
Pipecolic acid
frequency, cm⁻¹

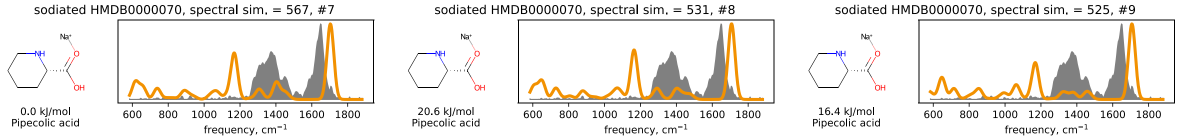
sodiated HMDB0000070, spectral sim. = 567, #7
0.0 kJ/mol
Pipecolic acid
sodiated HMDB0000070, spectral sim. = 531, #8
20.6 kJ/mol
Pipecolic acid
sodiated HMDB0000070, spectral sim. = 525, #9
16.4 kJ/mol
Pipecolic acid
frequency, cm⁻¹

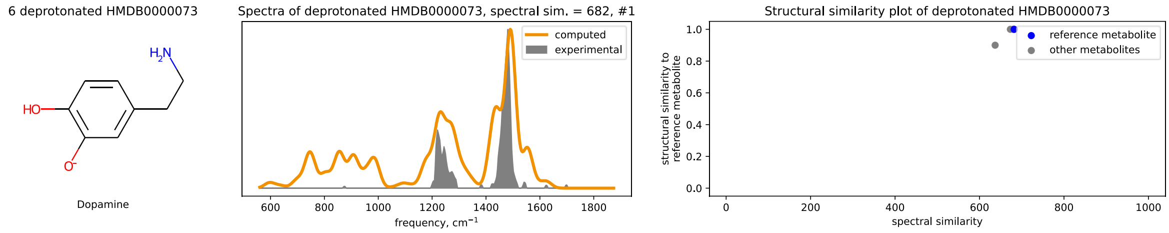
6 deprotonated HMDB0000073
Dopamine
Spectra of deprotonated HMDB0000073, spectral sim. = 682, #1
computed
experimental
frequency, cm⁻¹
Structural similarity plot of deprotonated HMDB0000073
reference metabolite
other metabolites
structural similarity to reference metabolite
spectral similarity

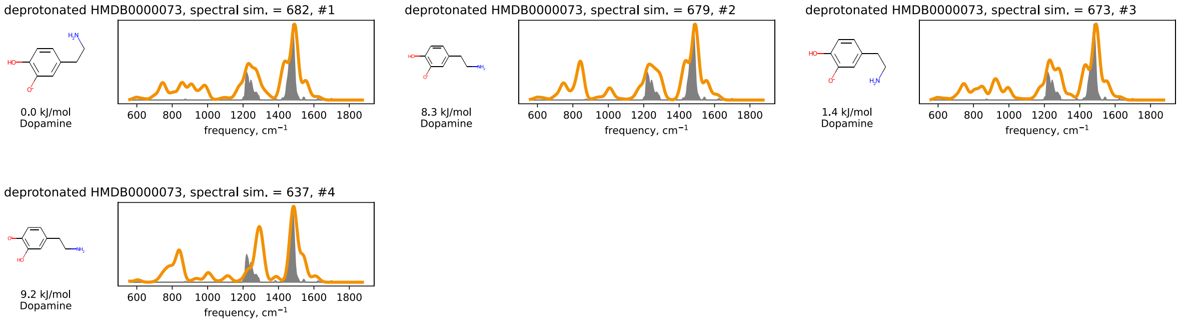
deprotonated HMDB0000073, spectral sim. = 682, #1
0.0 kJ/mol
Dopamine
deprotonated HMDB0000073, spectral sim. = 679, #2
8.3 kJ/mol
Dopamine
deprotonated HMDB0000073, spectral sim. = 673, #3
1.4 kJ/mol
Dopamine
deprotonated HMDB0000073, spectral sim. = 637, #4
9.2 kJ/mol
Dopamine
frequency, cm⁻¹

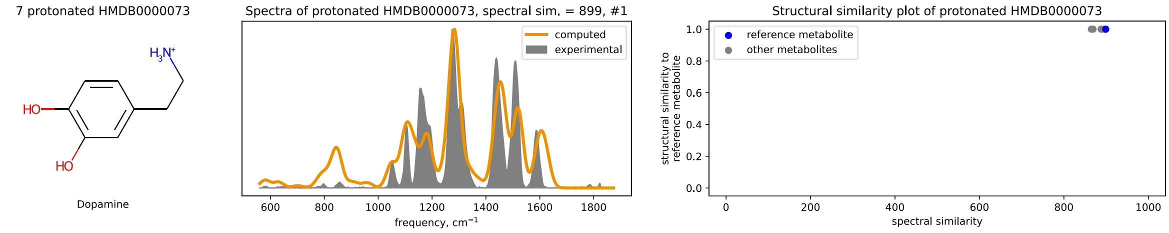
7 protonated HMDB0000073
Dopamine
Spectra of protonated HMDB0000073, spectral sim. = 899, #1
computed
experimental
frequency, cm⁻¹
Structural similarity plot of protonated HMDB0000073
reference metabolite
other metabolites
structural similarity to reference metabolite
spectral similarity

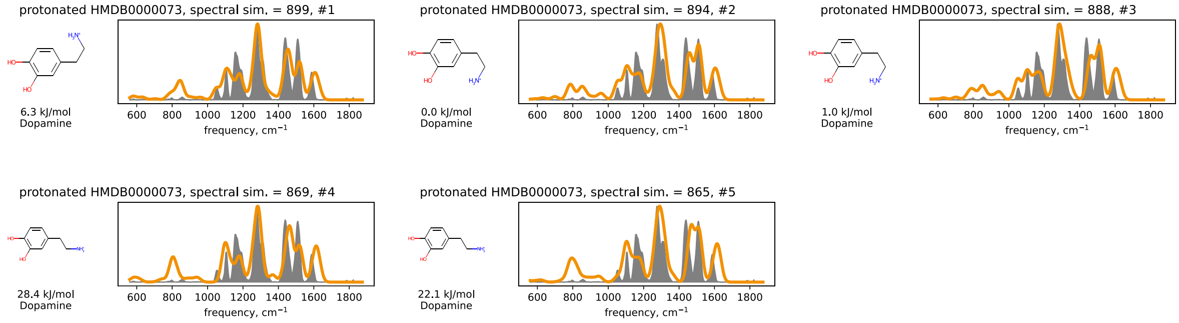
protonated HMDB0000073, spectral sim. = 899, #1
6.3 kJ/mol
Dopamine
protonated HMDB0000073, spectral sim. = 894, #2
0.0 kJ/mol
Dopamine
protonated HMDB0000073, spectral sim. = 888, #3
1.0 kJ/mol
Dopamine
protonated HMDB0000073, spectral sim. = 869, #4
28.4 kJ/mol
Dopamine
protonated HMDB0000073, spectral sim. = 865, #5
22.1 kJ/mol
Dopamine
frequency, cm⁻¹

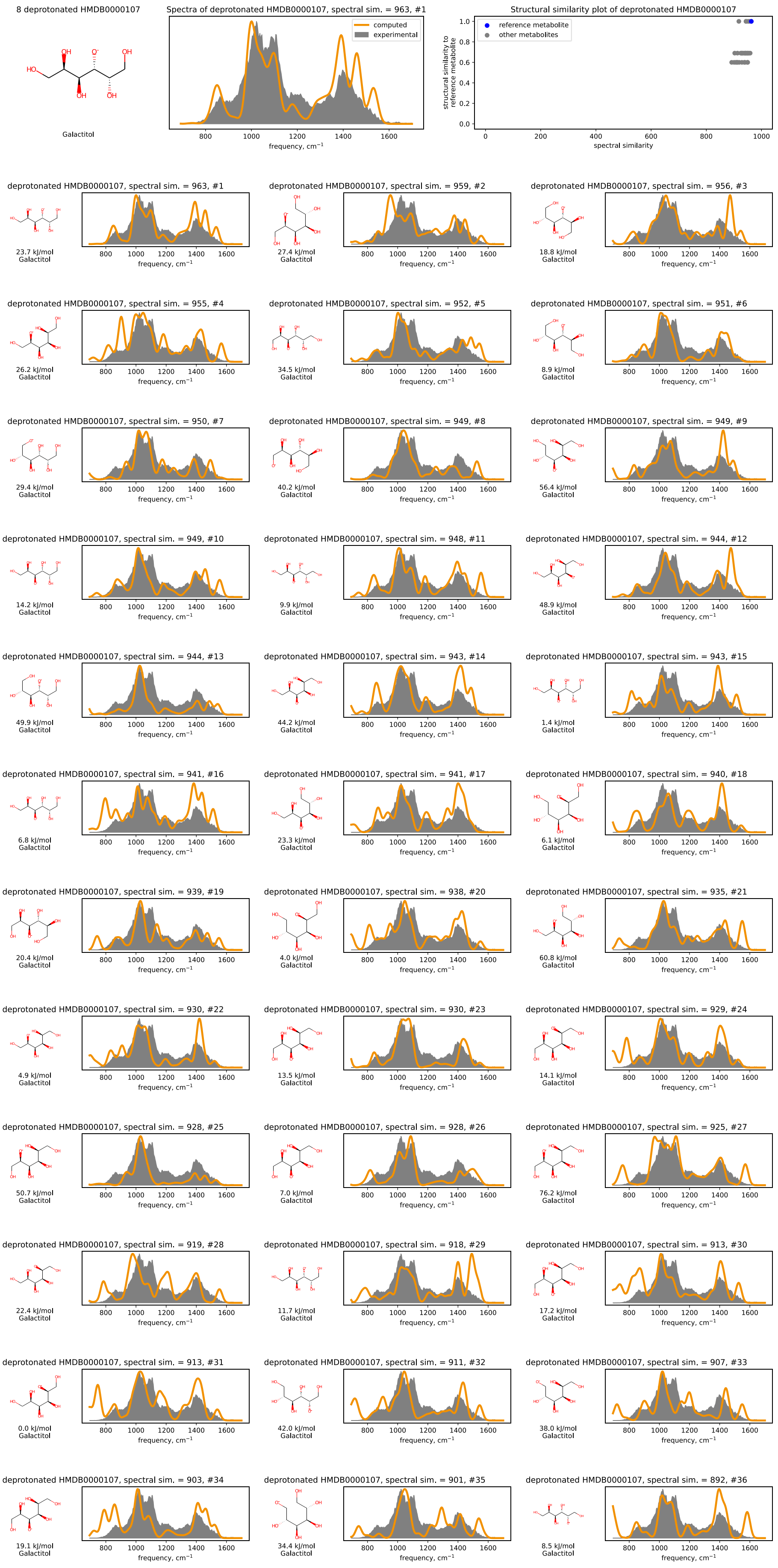
8 deprotonated HMDB0000107
Galactitol
Spectra of deprotonated HMDB0000107, spectral sim. = 963, #1
computed
experimental
frequency, cm−1
Structural similarity plot of deprotonated HMDB0000107
reference metabolite
other metabolites
structural similarity to reference metabolite
spectral similarity
deprotonated HMDB0000107, spectral sim. = 963, #1
23.7 kJ/mol Galactitol
deprotonated HMDB0000107, spectral sim. = 959, #2
27.4 kJ/mol Galactitol
deprotonated HMDB0000107, spectral sim. = 956, #3
18.8 kJ/mol Galactitol
deprotonated HMDB0000107, spectral sim. = 955, #4
26.2 kJ/mol Galactitol
deprotonated HMDB0000107, spectral sim. = 952, #5
34.5 kJ/mol Galactitol
deprotonated HMDB0000107, spectral sim. = 951, #6
8.9 kJ/mol Galactitol
deprotonated HMDB0000107, spectral sim. = 950, #7
29.4 kJ/mol Galactitol
deprotonated HMDB0000107, spectral sim. = 949, #8
40.2 kJ/mol Galactitol
deprotonated HMDB0000107, spectral sim. = 949, #9
56.4 kJ/mol Galactitol
deprotonated HMDB0000107, spectral sim. = 949, #10
14.2 kJ/mol Galactitol
deprotonated HMDB0000107, spectral sim. = 948, #11
9.9 kJ/mol Galactitol
deprotonated HMDB0000107, spectral sim. = 944, #12
48.9 kJ/mol Galactitol
deprotonated HMDB0000107, spectral sim. = 944, #13
49.9 kJ/mol Galactitol
deprotonated HMDB0000107, spectral sim. = 943, #14
44.2 kJ/mol Galactitol
deprotonated HMDB0000107, spectral sim. = 943, #15
1.4 kJ/mol Galactitol
deprotonated HMDB0000107, spectral sim. = 941, #16
6.8 kJ/mol Galactitol
deprotonated HMDB0000107, spectral sim. = 941, #17
23.3 kJ/mol Galactitol
deprotonated HMDB0000107, spectral sim. = 940, #18
6.1 kJ/mol Galactitol
deprotonated HMDB0000107, spectral sim. = 939, #19
20.4 kJ/mol Galactitol
deprotonated HMDB0000107, spectral sim. = 938, #20
4.0 kJ/mol Galactitol
deprotonated HMDB0000107, spectral sim. = 935, #21
60.8 kJ/mol Galactitol
deprotonated HMDB0000107, spectral sim. = 930, #22
4.9 kJ/mol Galactitol
deprotonated HMDB0000107, spectral sim. = 930, #23
13.5 kJ/mol Galactitol
deprotonated HMDB0000107, spectral sim. = 929, #24
14.1 kJ/mol Galactitol
deprotonated HMDB0000107, spectral sim. = 928, #25
50.7 kJ/mol Galactitol
deprotonated HMDB0000107, spectral sim. = 928, #26
7.0 kJ/mol Galactitol
deprotonated HMDB0000107, spectral sim. = 925, #27
76.2 kJ/mol Galactitol
deprotonated HMDB0000107, spectral sim. = 919, #28
22.4 kJ/mol Galactitol
deprotonated HMDB0000107, spectral sim. = 918, #29
11.7 kJ/mol Galactitol
deprotonated HMDB0000107, spectral sim. = 913, #30
17.2 kJ/mol Galactitol
deprotonated HMDB0000107, spectral sim. = 913, #31
0.0 kJ/mol Galactitol
deprotonated HMDB0000107, spectral sim. = 911, #32
42.0 kJ/mol Galactitol
deprotonated HMDB0000107, spectral sim. = 907, #33
38.0 kJ/mol Galactitol
deprotonated HMDB0000107, spectral sim. = 903, #34
19.1 kJ/mol Galactitol
deprotonated HMDB0000107, spectral sim. = 901, #35
34.4 kJ/mol Galactitol
deprotonated HMDB0000107, spectral sim. = 892, #36
8.5 kJ/mol Galactitol

9 deprotonated HMDB0000130

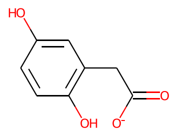


Homogentisic acid

Spectra of deprotonated HMDB0000130, spectral sim. = 895, #1

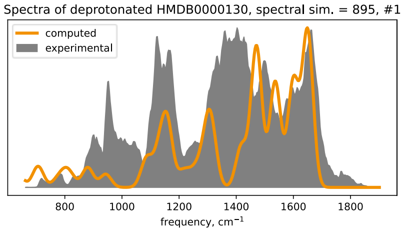


Structural similarity plot of deprotonated HMDB0000130

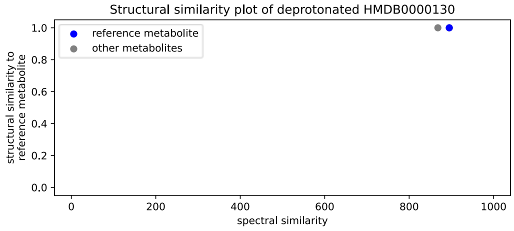


deprotonated HMDB0000130, spectral sim. = 895, #1

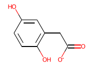

0.0 kJ/mol
Homogentisic acid

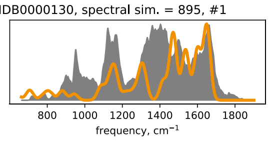


deprotonated HMDB0000130, spectral sim. = 868, #2

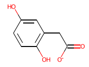

2.6 kJ/mol
Homogentisic acid

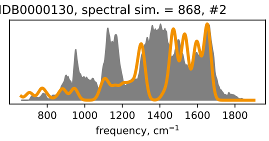


10 sodiated HMDB0000130

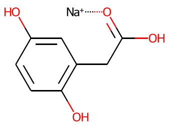


Homogentisic acid

Spectra of sodiated HMDB0000130, spectral sim. = 932, #1

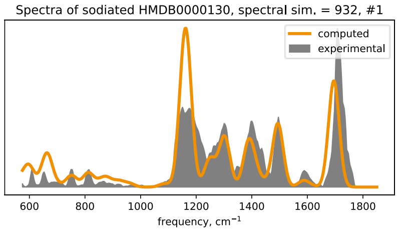


Structural similarity plot of sodiated HMDB0000130

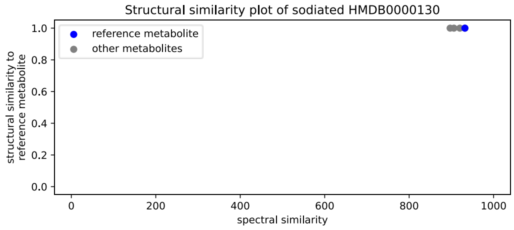


sodiated HMDB0000130, spectral sim. = 932, #1

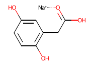

0.0 kJ/mol
Homogentisic acid

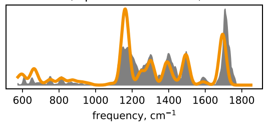


sodiated HMDB0000130, spectral sim. = 920, #2

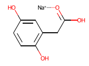

1.9 kJ/mol
Homogentisic acid

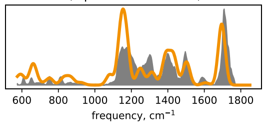


sodiated HMDB0000130, spectral sim. = 906, #3

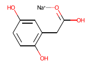

20.8 kJ/mol
Homogentisic acid

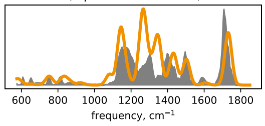


sodiated HMDB0000130, spectral sim. = 897, #4

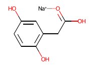

18.3 kJ/mol
Homogentisic acid

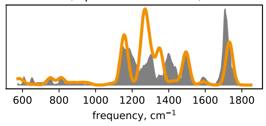


11 deprotonated HMDB0000132

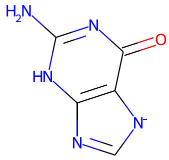


Guanine

Spectra of deprotonated HMDB0000132, spectral sim. = 868, #1

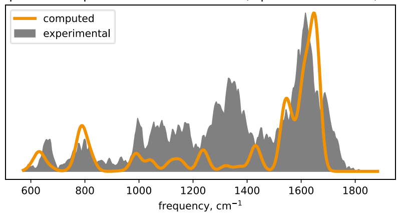


Structural similarity plot of deprotonated HMDB0000132

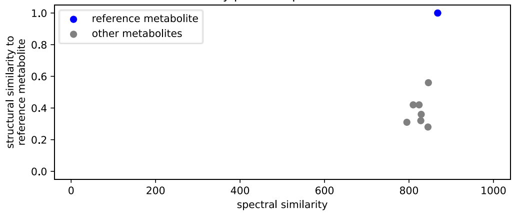


deprotonated HMDB0000132, spectral sim. = 868, #1

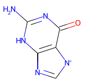

28.8 kJ/mol
Guanine

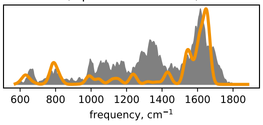


deprotonated HMDB0000132, spectral sim. = 846, #2

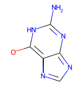

0.0 kJ/mol
Guanine

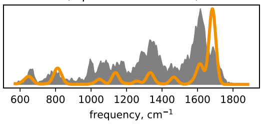


deprotonated HMDB0000132, spectral sim. = 845, #3

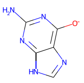

18.9 kJ/mol
Guanine

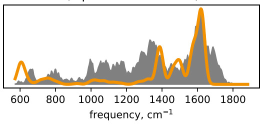


deprotonated HMDB0000132, spectral sim. = 829, #4

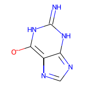

8.4 kJ/mol
Guanine

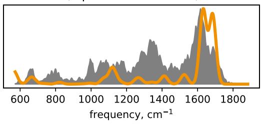


deprotonated HMDB0000132, spectral sim. = 828, #5

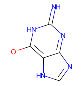

44.2 kJ/mol
Guanine

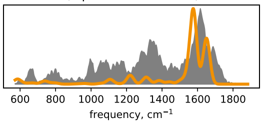


deprotonated HMDB0000132, spectral sim. = 824, #6

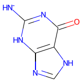

60.2 kJ/mol
Guanine

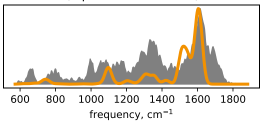


deprotonated HMDB0000132, spectral sim. = 810, #7

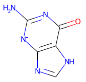

5.9 kJ/mol
Guanine

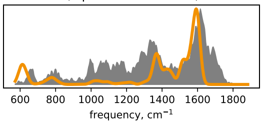


deprotonated HMDB0000132, spectral sim. = 795, #8

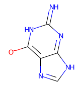

29.6 kJ/mol
Guanine

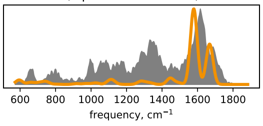

12 protonated HMDB0000132

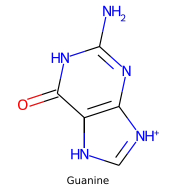


Spectra of protonated HMDB0000132, spectral sim. = 932, #1

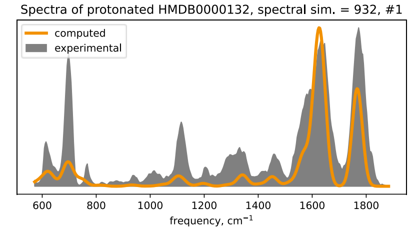


Structural similarity plot of protonated HMDB0000132

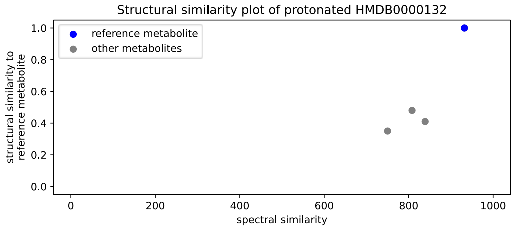


protonated HMDB0000132, spectral sim. = 932, #1

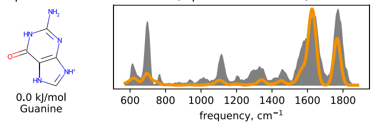


protonated HMDB0000132, spectral sim. = 839, #2

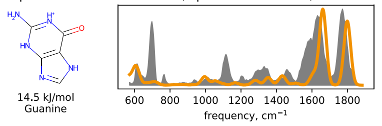


protonated HMDB0000132, spectral sim. = 808, #3

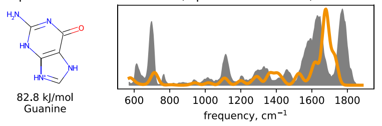


protonated HMDB0000132, spectral sim. = 750, #4

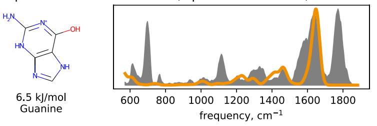


13 sodiated HMDB0000132

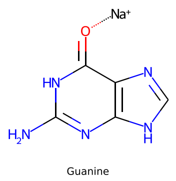


Spectra of sodiated HMDB0000132, spectral sim. = 910, #1

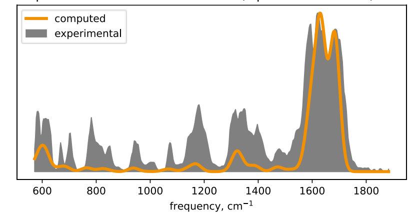


Structural similarity plot of sodiated HMDB0000132

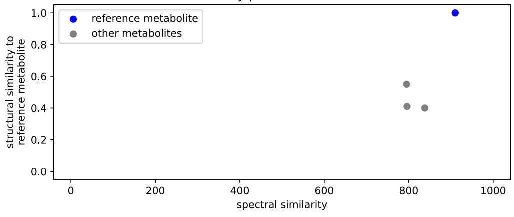


sodiated HMDB0000132, spectral sim. = 910, #1

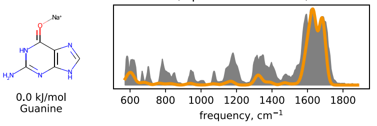


sodiated HMDB0000132, spectral sim. = 838, #2

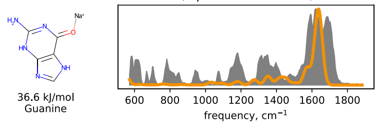


sodiated HMDB0000132, spectral sim. = 796, #3

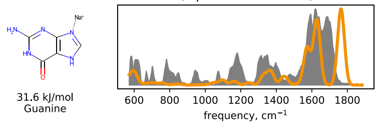


sodiated HMDB0000132, spectral sim. = 795, #4

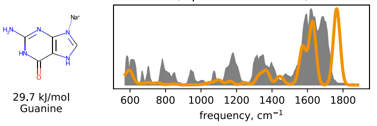

14 deprotonated HMDB0000143

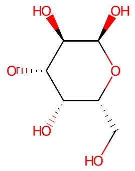


D-Galactose

Spectra of deprotonated HMDB0000143, spectral sim. = 875, #1

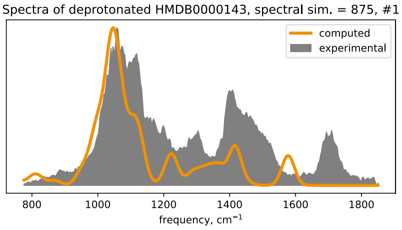


Structural similarity plot of deprotonated HMDB0000143

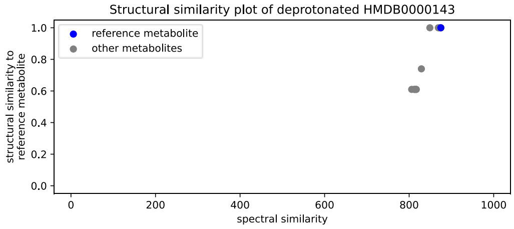


deprotonated HMDB0000143, spectral sim. = 875, #1

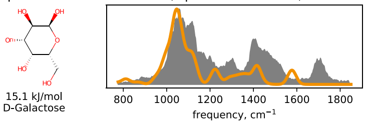


deprotonated HMDB0000143, spectral sim. = 872, #2

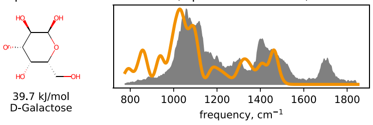


deprotonated HMDB0000143, spectral sim. = 869, #3

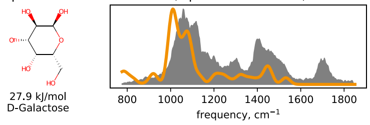


deprotonated HMDB0000143, spectral sim. = 849, #4

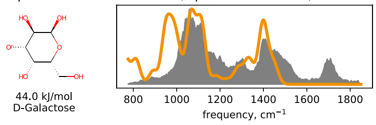


deprotonated HMDB0000143, spectral sim. = 829, #5

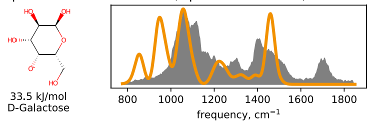


deprotonated HMDB0000143, spectral sim. = 817, #6

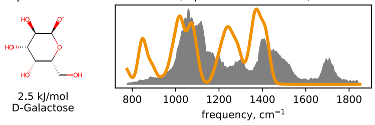


deprotonated HMDB0000143, spectral sim. = 816, #7

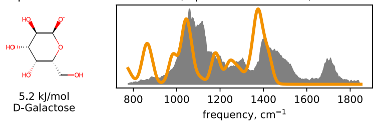


deprotonated HMDB0000143, spectral sim. = 813, #8

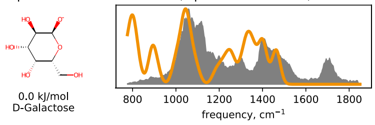


deprotonated HMDB0000143, spectral sim. = 812, #9

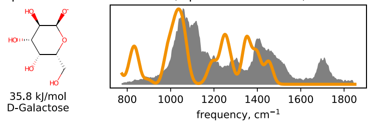


deprotonated HMDB0000143, spectral sim. = 806, #10

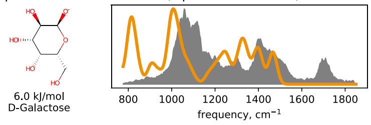


15 deprotonated HMDB0000148

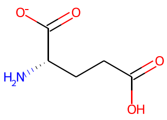


L-Glutamic acid

Spectra of deprotonated HMDB0000148, spectral sim. = 816, #1

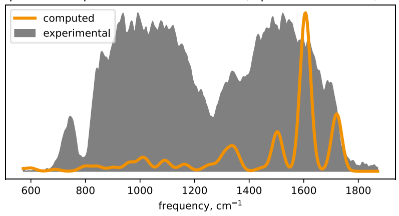


Structural similarity plot of deprotonated HMDB0000148

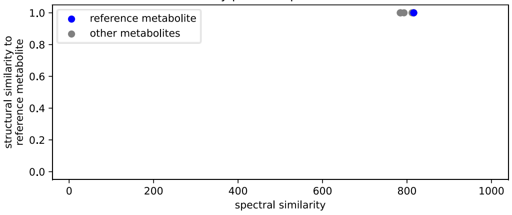


deprotonated HMDB0000148, spectral sim. = 816, #1

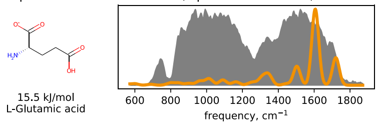


deprotonated HMDB0000148, spectral sim. = 816, #2

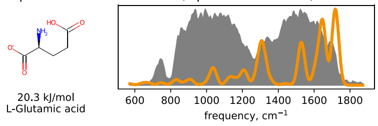


deprotonated HMDB0000148, spectral sim. = 812, #3

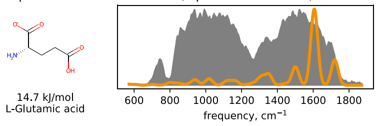


deprotonated HMDB0000148, spectral sim. = 793, #4

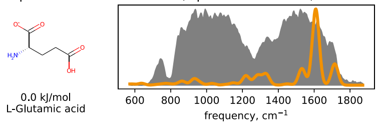


deprotonated HMDB0000148, spectral sim. = 785, #5

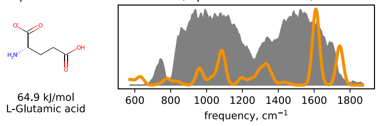


deprotonated HMDB0000148, spectral sim. = 784, #6

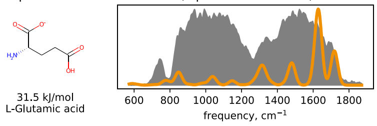

16 sodiated HMDB0000148

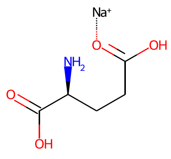


L-Glutamic acid

Spectra of sodiated HMDB0000148, spectral sim. = 931, #1

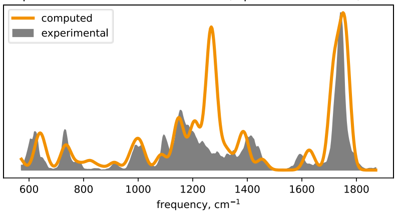


Structural similarity plot of sodiated HMDB0000148

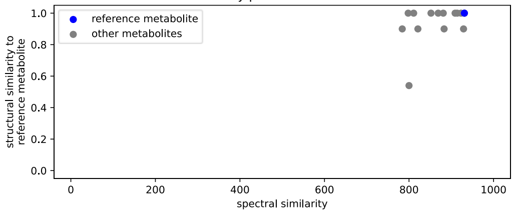


sodiated HMDB0000148, spectral sim. = 931, #1

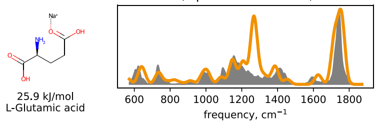


sodiated HMDB0000148, spectral sim. = 929, #2

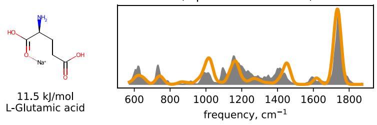


sodiated HMDB0000148, spectral sim. = 923, #3

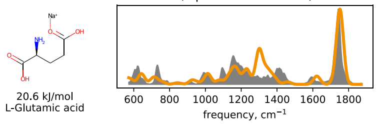


sodiated HMDB0000148, spectral sim. = 914, #4

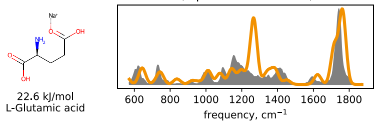


sodiated HMDB0000148, spectral sim. = 909, #5

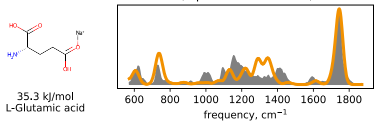


sodiated HMDB0000148, spectral sim. = 883, #6

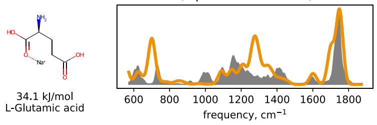


sodiated HMDB0000148, spectral sim. = 881, #7

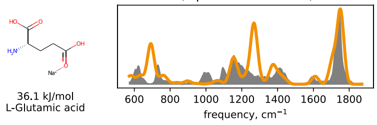


sodiated HMDB0000148, spectral sim. = 869, #8

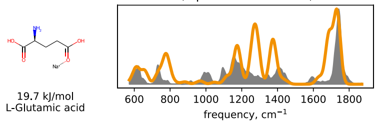


sodiated HMDB0000148, spectral sim. = 852, #9

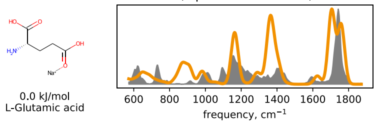


sodiated HMDB0000148, spectral sim. = 821, #10

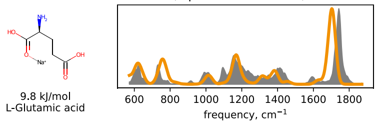


sodiated HMDB0000148, spectral sim. = 811, #11

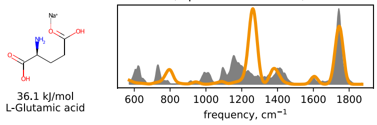


sodiated HMDB0000148, spectral sim. = 800, #12

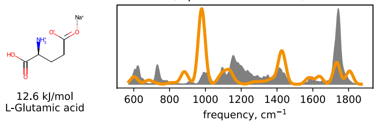


sodiated HMDB0000148, spectral sim. = 798, #13

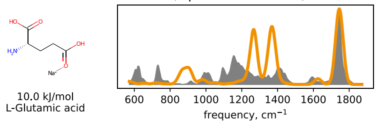


sodiated HMDB0000148, spectral sim. = 784, #14

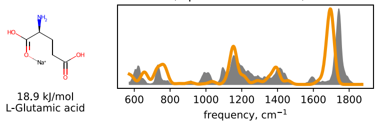


17 deprotonated HMDB0000159

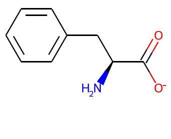


L-Phenylalanine

Spectra of deprotonated HMDB0000159, spectral sim. = 940, #1

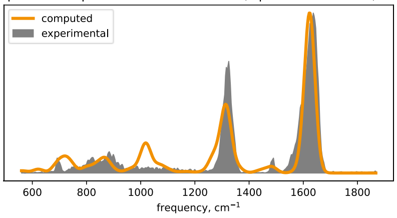


Structural similarity plot of deprotonated HMDB0000159

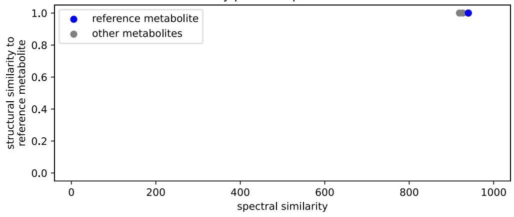


deprotonated HMDB0000159, spectral sim. = 940, #1

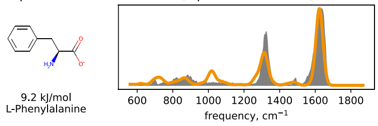


deprotonated HMDB0000159, spectral sim. = 927, #2

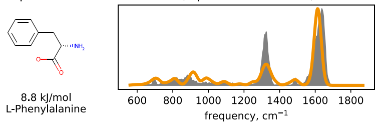


deprotonated HMDB0000159, spectral sim. = 919, #3

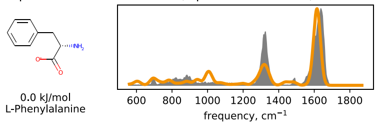

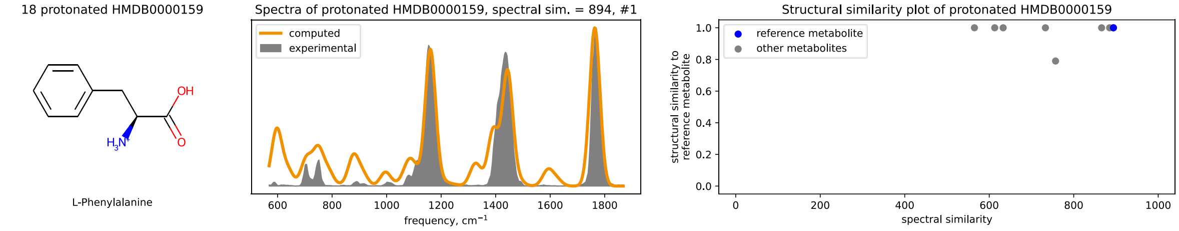
18 protonated HMDB0000159
L-Phenylalanine
Spectra of protonated HMDB0000159, spectral sim. = 894, #1
computed
experimental
frequency, cm$^{-1}$
Structural similarity plot of protonated HMDB0000159
reference metabolite
other metabolites
structural similarity to reference metabolite
spectral similarity

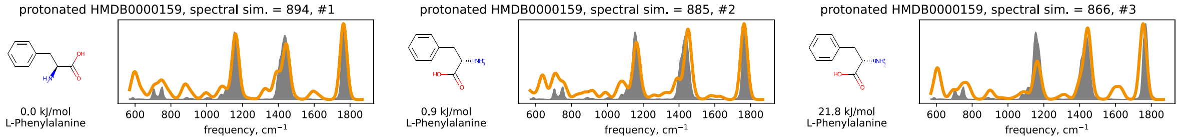
protonated HMDB0000159, spectral sim. = 894, #1
0.0 kJ/mol
L-Phenylalanine
protonated HMDB0000159, spectral sim. = 885, #2
0.9 kJ/mol
L-Phenylalanine
protonated HMDB0000159, spectral sim. = 866, #3
21.8 kJ/mol
L-Phenylalanine
frequency, cm$^{-1}$

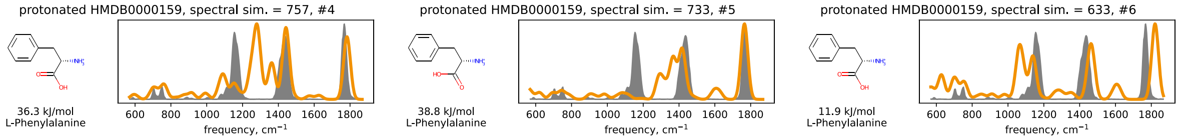
protonated HMDB0000159, spectral sim. = 757, #4
36.3 kJ/mol
L-Phenylalanine
protonated HMDB0000159, spectral sim. = 733, #5
38.8 kJ/mol
L-Phenylalanine
protonated HMDB0000159, spectral sim. = 633, #6
11.9 kJ/mol
L-Phenylalanine
frequency, cm$^{-1}$

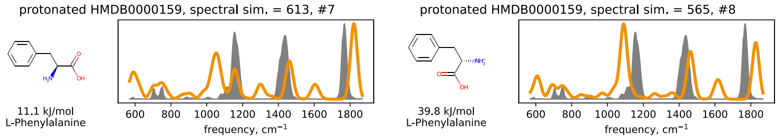
protonated HMDB0000159, spectral sim. = 613, #7
11.1 kJ/mol
L-Phenylalanine
protonated HMDB0000159, spectral sim. = 565, #8
39.8 kJ/mol
L-Phenylalanine
frequency, cm$^{-1}$

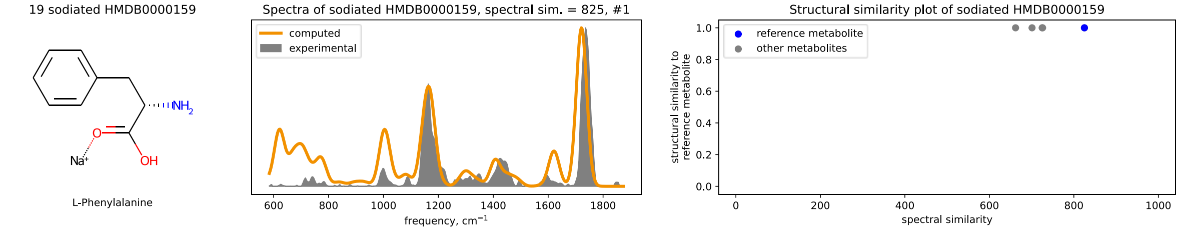
19 sodiated HMDB0000159
L-Phenylalanine
Spectra of sodiated HMDB0000159, spectral sim. = 825, #1
computed
experimental
frequency, cm$^{-1}$
Structural similarity plot of sodiated HMDB0000159
reference metabolite
other metabolites
structural similarity to reference metabolite
spectral similarity

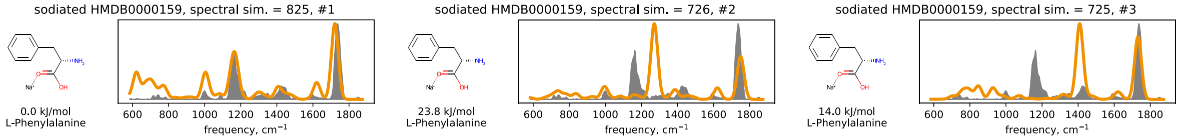
sodiated HMDB0000159, spectral sim. = 825, #1
0.0 kJ/mol
L-Phenylalanine
sodiated HMDB0000159, spectral sim. = 726, #2
23.8 kJ/mol
L-Phenylalanine
sodiated HMDB0000159, spectral sim. = 725, #3
14.0 kJ/mol
L-Phenylalanine
frequency, cm$^{-1}$

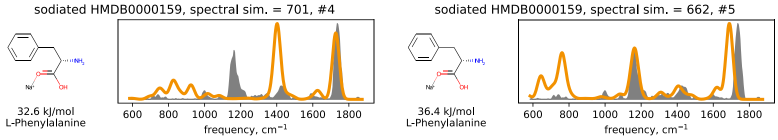
sodiated HMDB0000159, spectral sim. = 701, #4
32.6 kJ/mol
L-Phenylalanine
sodiated HMDB0000159, spectral sim. = 662, #5
36.4 kJ/mol
L-Phenylalanine
frequency, cm$^{-1}$

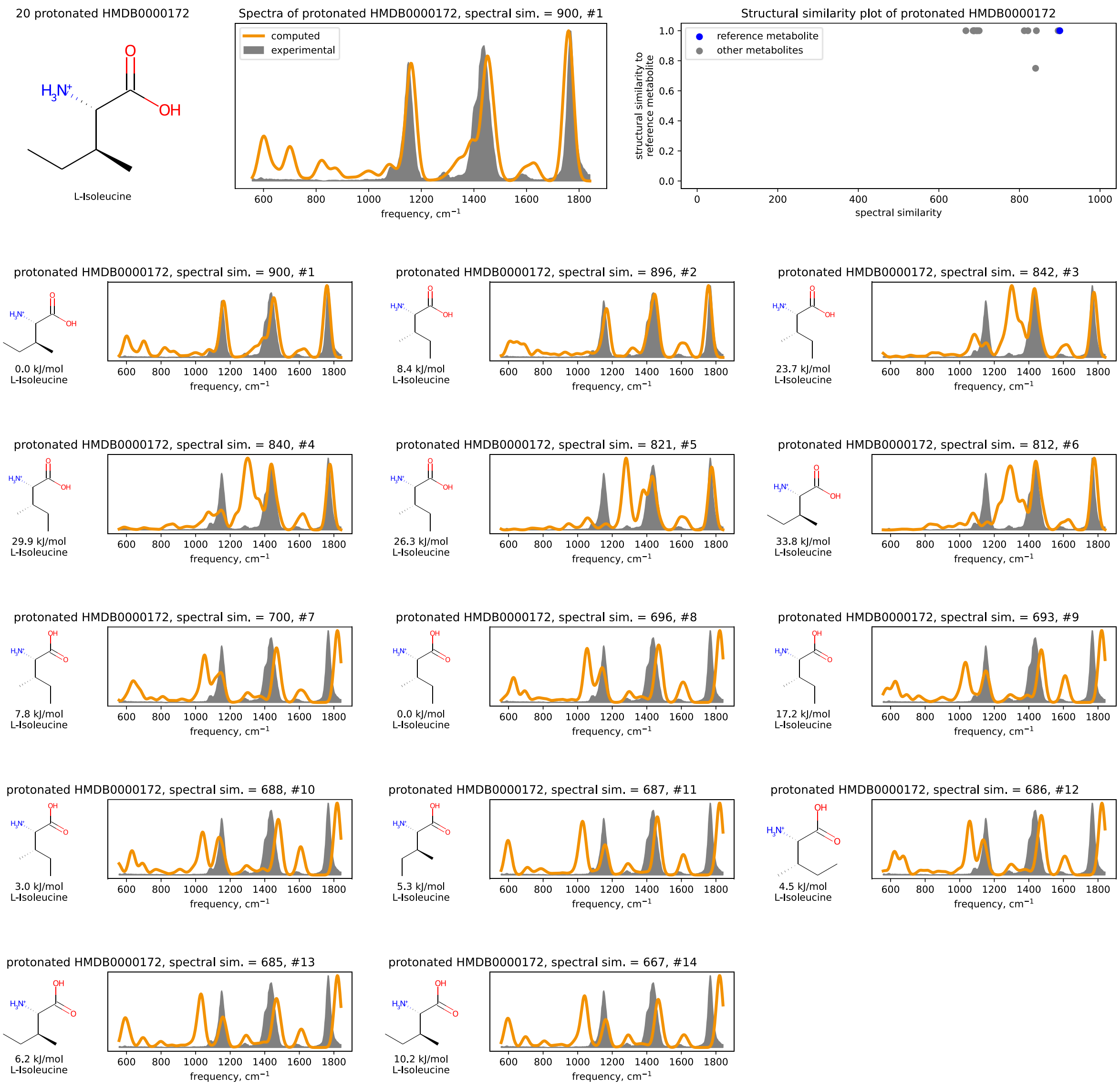

20 protonated HMDB0000172
L-Isoleucine
Spectra of protonated HMDB0000172, spectral sim. = 900, #1
computed
experimental
frequency, cm⁻¹
Structural similarity plot of protonated HMDB0000172
reference metabolite
other metabolites
structural similarity to reference metabolite
spectral similarity
protonated HMDB0000172, spectral sim. = 900, #1
0.0 kJ/mol
L-Isoleucine
protonated HMDB0000172, spectral sim. = 896, #2
8.4 kJ/mol
L-Isoleucine
protonated HMDB0000172, spectral sim. = 842, #3
23.7 kJ/mol
L-Isoleucine
protonated HMDB0000172, spectral sim. = 840, #4
29.9 kJ/mol
L-Isoleucine
protonated HMDB0000172, spectral sim. = 821, #5
26.3 kJ/mol
L-Isoleucine
protonated HMDB0000172, spectral sim. = 812, #6
33.8 kJ/mol
L-Isoleucine
protonated HMDB0000172, spectral sim. = 700, #7
7.8 kJ/mol
L-Isoleucine
protonated HMDB0000172, spectral sim. = 696, #8
0.0 kJ/mol
L-Isoleucine
protonated HMDB0000172, spectral sim. = 693, #9
17.2 kJ/mol
L-Isoleucine
protonated HMDB0000172, spectral sim. = 688, #10
3.0 kJ/mol
L-Isoleucine
protonated HMDB0000172, spectral sim. = 687, #11
5.3 kJ/mol
L-Isoleucine
protonated HMDB0000172, spectral sim. = 686, #12
4.5 kJ/mol
L-Isoleucine
protonated HMDB0000172, spectral sim. = 685, #13
6.2 kJ/mol
L-Isoleucine
protonated HMDB0000172, spectral sim. = 667, #14
10.2 kJ/mol
L-Isoleucine

21 deprotonated HMDB0000182

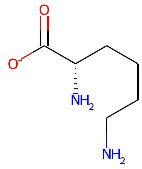


L-Lysine

Spectra of deprotonated HMDB0000182, spectral sim. = 864, #1

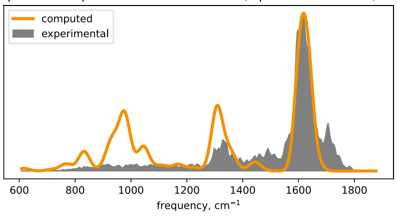


Structural similarity plot of deprotonated HMDB0000182

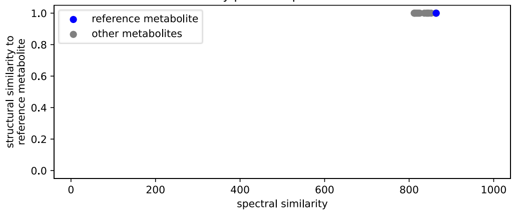


deprotonated HMDB0000182, spectral sim. = 864, #1

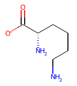

8.5 kJ/mol
L-Lysine

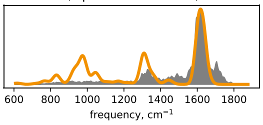

deprotonated HMDB0000182, spectral sim. = 863, #2

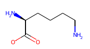

2.0 kJ/mol
L-Lysine

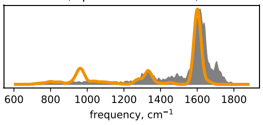

deprotonated HMDB0000182, spectral sim. = 852, #3

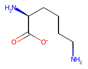

0.0 kJ/mol
L-Lysine

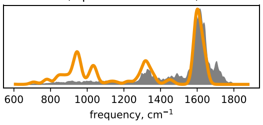

deprotonated HMDB0000182, spectral sim. = 847, #4

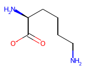

8.7 kJ/mol
L-Lysine

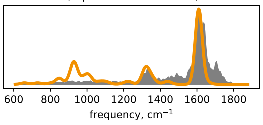

deprotonated HMDB0000182, spectral sim. = 844, #5

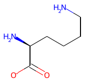

5.3 kJ/mol
L-Lysine

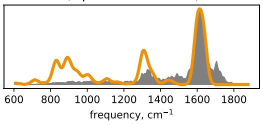

deprotonated HMDB0000182, spectral sim. = 841, #6

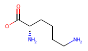

6.0 kJ/mol
L-Lysine

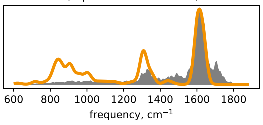

deprotonated HMDB0000182, spectral sim. = 836, #7

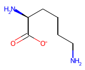

0.9 kJ/mol
L-Lysine

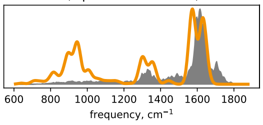

deprotonated HMDB0000182, spectral sim. = 825, #8

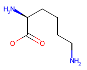

7.6 kJ/mol
L-Lysine

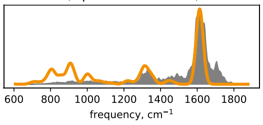

deprotonated HMDB0000182, spectral sim. = 822, #9

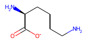

4.2 kJ/mol
L-Lysine

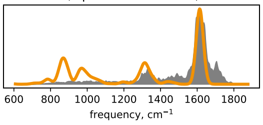

deprotonated HMDB0000182, spectral sim. = 819, #10

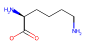

7.7 kJ/mol
L-Lysine

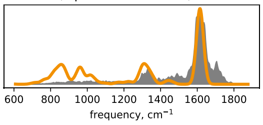

deprotonated HMDB0000182, spectral sim. = 816, #11

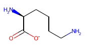

5.5 kJ/mol
L-Lysine

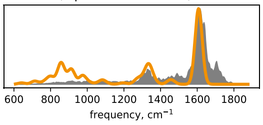

deprotonated HMDB0000182, spectral sim. = 813, #12

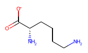

13.0 kJ/mol
L-Lysine

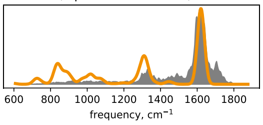

deprotonated HMDB0000182, spectral sim. = 812, #13

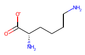

6.6 kJ/mol
L-Lysine

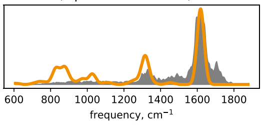

22 protonated HMDB0000182

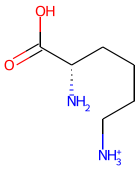


L-Lysine

Spectra of protonated HMDB0000182, spectral sim. = 895, #1

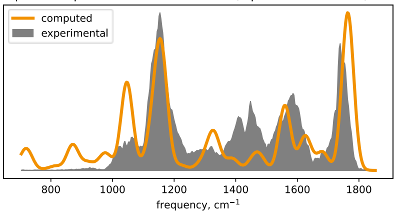


Structural similarity plot of protonated HMDB0000182

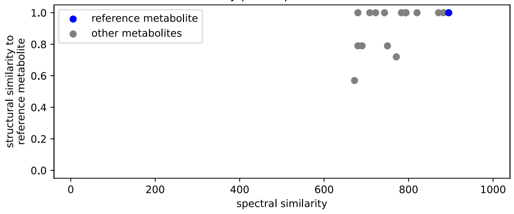


protonated HMDB0000182, spectral sim. = 895, #1

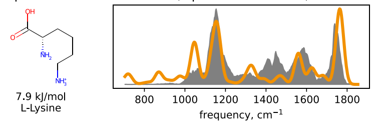


protonated HMDB0000182, spectral sim. = 883, #2

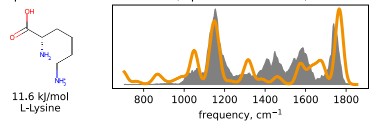


protonated HMDB0000182, spectral sim. = 871, #3

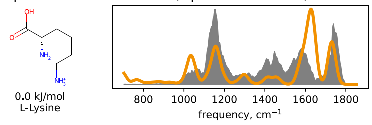


protonated HMDB0000182, spectral sim. = 820, #4

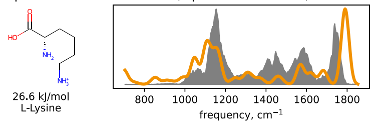


protonated HMDB0000182, spectral sim. = 794, #5

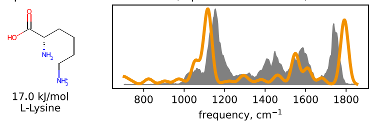


protonated HMDB0000182, spectral sim. = 793, #6

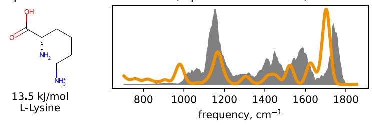


protonated HMDB0000182, spectral sim. = 783, #7

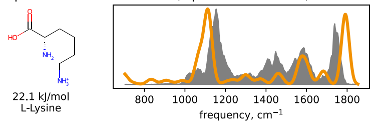


protonated HMDB0000182, spectral sim. = 771, #8

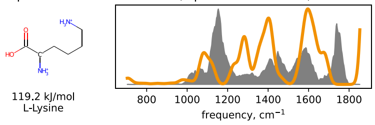


protonated HMDB0000182, spectral sim. = 750, #9

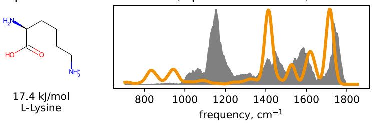


protonated HMDB0000182, spectral sim. = 743, #10

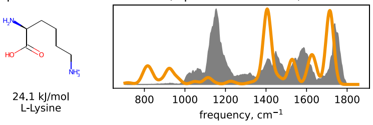


protonated HMDB0000182, spectral sim. = 722, #11

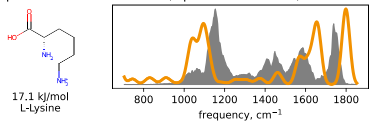


protonated HMDB0000182, spectral sim. = 708, #12

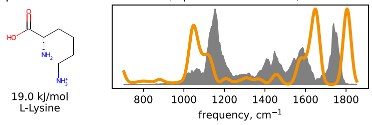


protonated HMDB0000182, spectral sim. = 690, #13

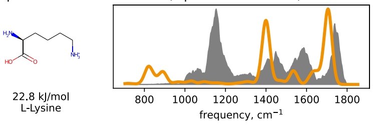


protonated HMDB0000182, spectral sim. = 680, #14

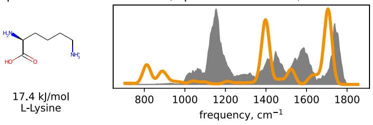


protonated HMDB0000182, spectral sim. = 680, #15

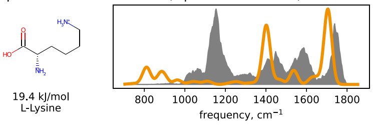


protonated HMDB0000182, spectral sim. = 672, #16

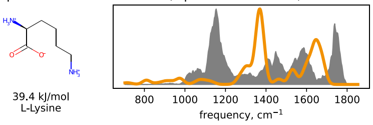

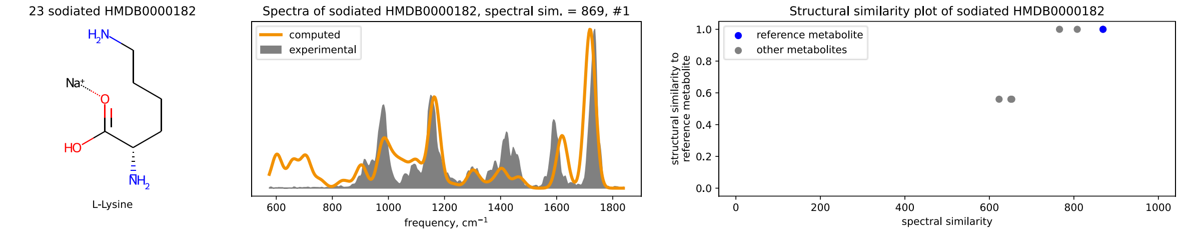
23 sodiated HMDB0000182
L-Lysine
Spectra of sodiated HMDB0000182, spectral sim. = 869, #1
computed
experimental
frequency, cm$^{-1}$
Structural similarity plot of sodiated HMDB0000182
reference metabolite
other metabolites
structural similarity to reference metabolite
spectral similarity

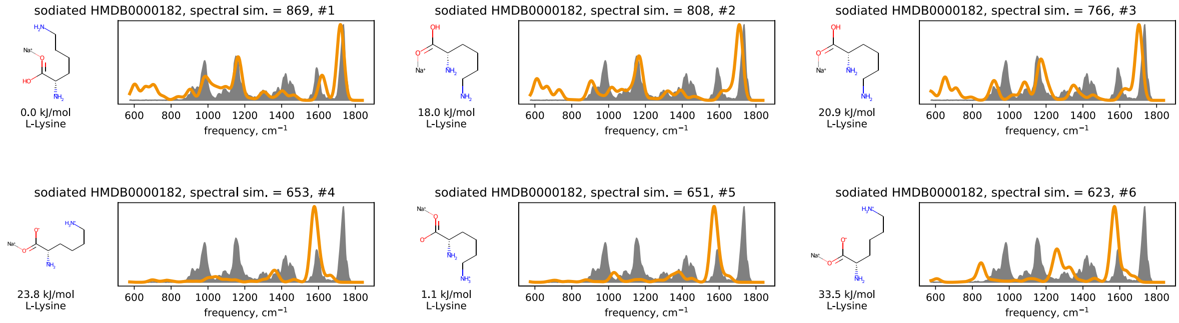
sodiated HMDB0000182, spectral sim. = 869, #1
0.0 kJ/mol
L-Lysine
sodiated HMDB0000182, spectral sim. = 808, #2
18.0 kJ/mol
L-Lysine
sodiated HMDB0000182, spectral sim. = 766, #3
20.9 kJ/mol
L-Lysine
sodiated HMDB0000182, spectral sim. = 653, #4
23.8 kJ/mol
L-Lysine
sodiated HMDB0000182, spectral sim. = 651, #5
1.1 kJ/mol
L-Lysine
sodiated HMDB0000182, spectral sim. = 623, #6
33.5 kJ/mol
L-Lysine
frequency, cm$^{-1}$

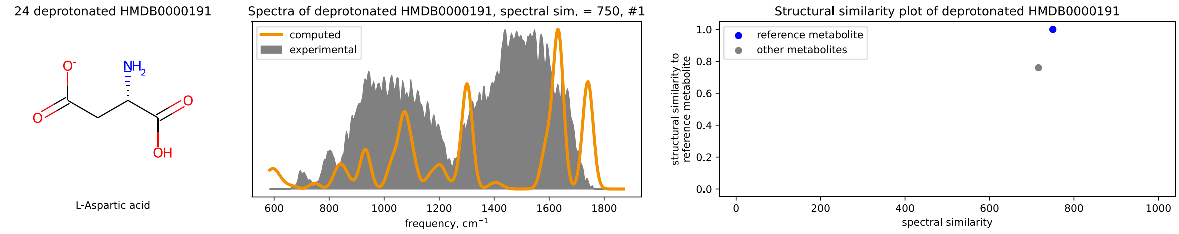
24 deprotonated HMDB0000191
L-Aspartic acid
Spectra of deprotonated HMDB0000191, spectral sim. = 750, #1
computed
experimental
frequency, cm$^{-1}$
Structural similarity plot of deprotonated HMDB0000191
reference metabolite
other metabolites
structural similarity to reference metabolite
spectral similarity

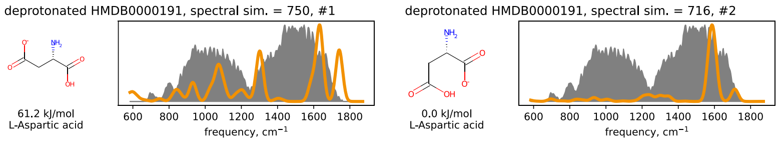
deprotonated HMDB0000191, spectral sim. = 750, #1
61.2 kJ/mol
L-Aspartic acid
deprotonated HMDB0000191, spectral sim. = 716, #2
0.0 kJ/mol
L-Aspartic acid
frequency, cm$^{-1}$

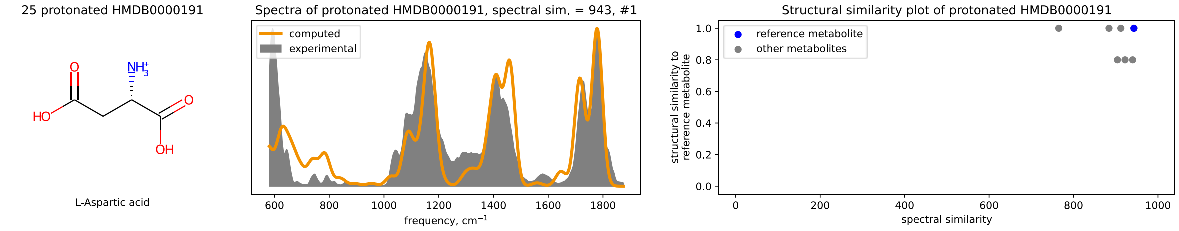
25 protonated HMDB0000191
L-Aspartic acid
Spectra of protonated HMDB0000191, spectral sim. = 943, #1
computed
experimental
frequency, cm$^{-1}$
Structural similarity plot of protonated HMDB0000191
reference metabolite
other metabolites
structural similarity to reference metabolite
spectral similarity

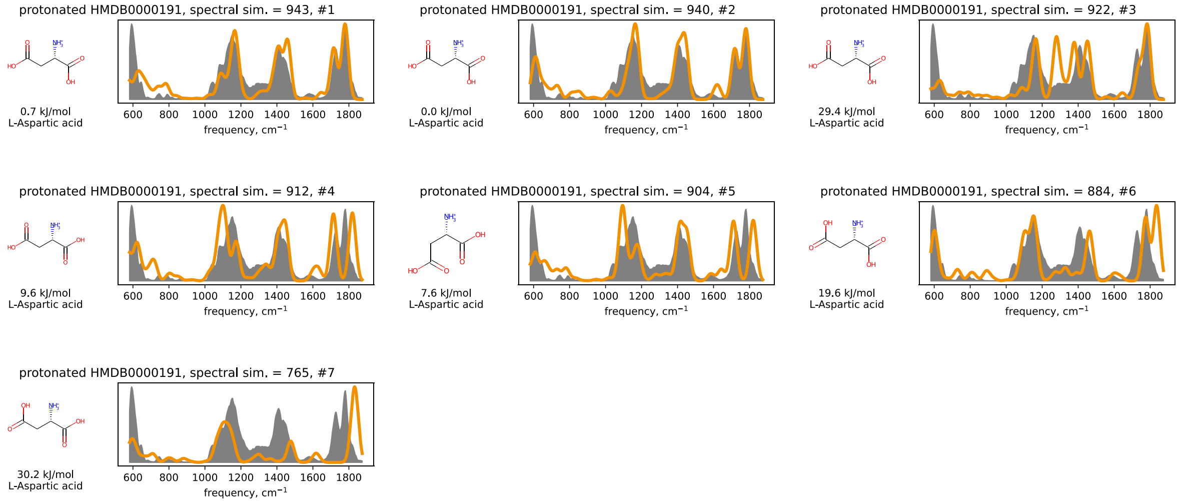
protonated HMDB0000191, spectral sim. = 943, #1
0.7 kJ/mol
L-Aspartic acid
protonated HMDB0000191, spectral sim. = 940, #2
0.0 kJ/mol
L-Aspartic acid
protonated HMDB0000191, spectral sim. = 922, #3
29.4 kJ/mol
L-Aspartic acid
protonated HMDB0000191, spectral sim. = 912, #4
9.6 kJ/mol
L-Aspartic acid
protonated HMDB0000191, spectral sim. = 904, #5
7.6 kJ/mol
L-Aspartic acid
protonated HMDB0000191, spectral sim. = 884, #6
19.6 kJ/mol
L-Aspartic acid
protonated HMDB0000191, spectral sim. = 765, #7
30.2 kJ/mol
L-Aspartic acid
frequency, cm$^{-1}$

26 sodiated HMDB0000191

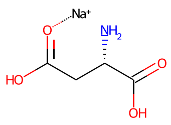


L-Aspartic acid

Spectra of sodiated HMDB0000191, spectral sim. = 909, #1

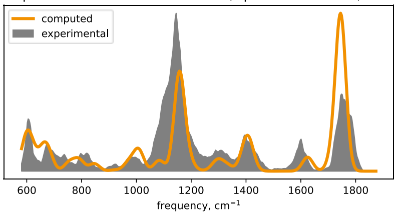


Structural similarity plot of sodiated HMDB0000191

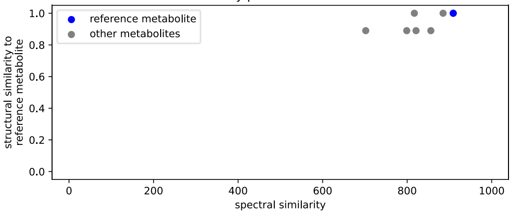


sodiated HMDB0000191, spectral sim. = 909, #1

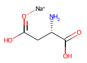

0.0 kJ/mol
L-Aspartic acid

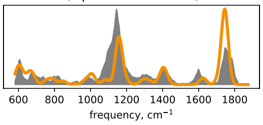


sodiated HMDB0000191, spectral sim. = 885, #2

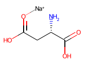

23.3 kJ/mol
L-Aspartic acid

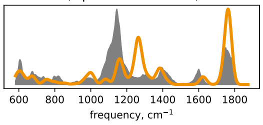


sodiated HMDB0000191, spectral sim. = 856, #3

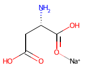

11.5 kJ/mol
L-Aspartic acid

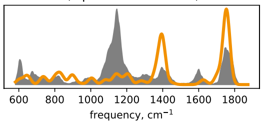


sodiated HMDB0000191, spectral sim. = 821, #4

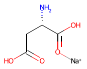

9.4 kJ/mol
L-Aspartic acid

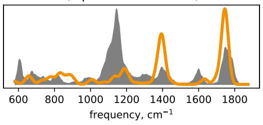


sodiated HMDB0000191, spectral sim. = 817, #5

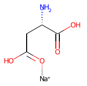

18.5 kJ/mol
L-Aspartic acid

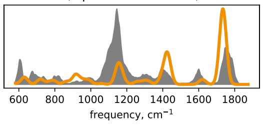


sodiated HMDB0000191, spectral sim. = 799, #6

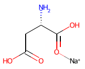

28.8 kJ/mol
L-Aspartic acid

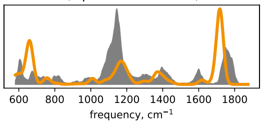


sodiated HMDB0000191, spectral sim. = 702, #7

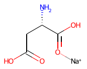

32.7 kJ/mol
L-Aspartic acid

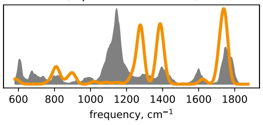


27 protonated HMDB0000201

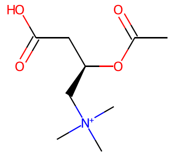


L-Acetylcarnitine

Spectra of protonated HMDB0000201, spectral sim. = 857, #1

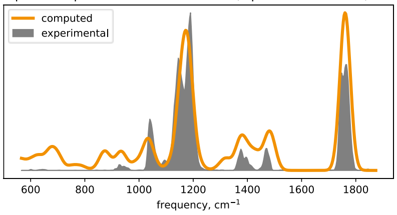


Structural similarity plot of protonated HMDB0000201

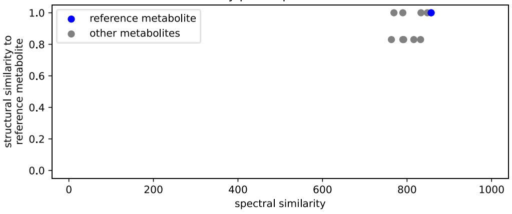


protonated HMDB0000201, spectral sim. = 857, #1

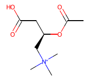

25.4 kJ/mol
L-Acetylcarnitine

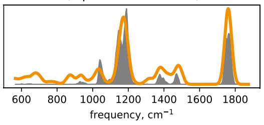


protonated HMDB0000201, spectral sim. = 848, #2

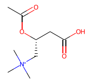

0.0 kJ/mol
L-Acetylcarnitine

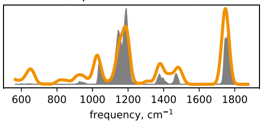


protonated HMDB0000201, spectral sim. = 833, #3

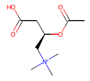

9.4 kJ/mol
L-Acetylcarnitine

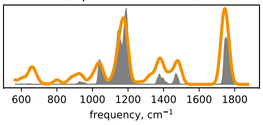


protonated HMDB0000201, spectral sim. = 832, #4

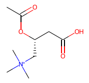

21.1 kJ/mol
L-Acetylcarnitine

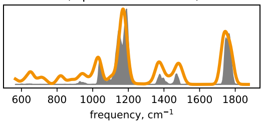


protonated HMDB0000201, spectral sim. = 816, #5

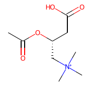

30.6 kJ/mol
L-Acetylcarnitine

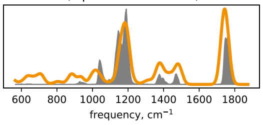


protonated HMDB0000201, spectral sim. = 792, #6

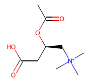

16.7 kJ/mol
L-Acetylcarnitine

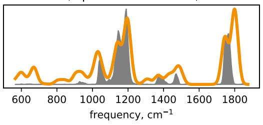


protonated HMDB0000201, spectral sim. = 790, #7

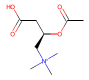

20.0 kJ/mol
L-Acetylcarnitine

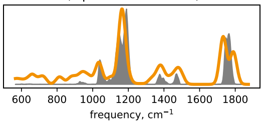


protonated HMDB0000201, spectral sim. = 790, #8

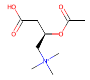

39.5 kJ/mol
L-Acetylcarnitine

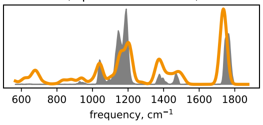


protonated HMDB0000201, spectral sim. = 769, #9

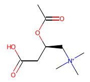

22.0 kJ/mol
L-Acetylcarnitine

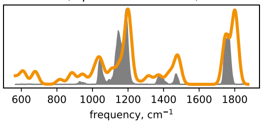


protonated HMDB0000201, spectral sim. = 763, #10

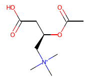

26.5 kJ/mol
L-Acetylcarnitine

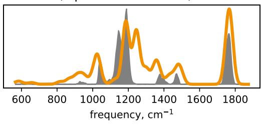

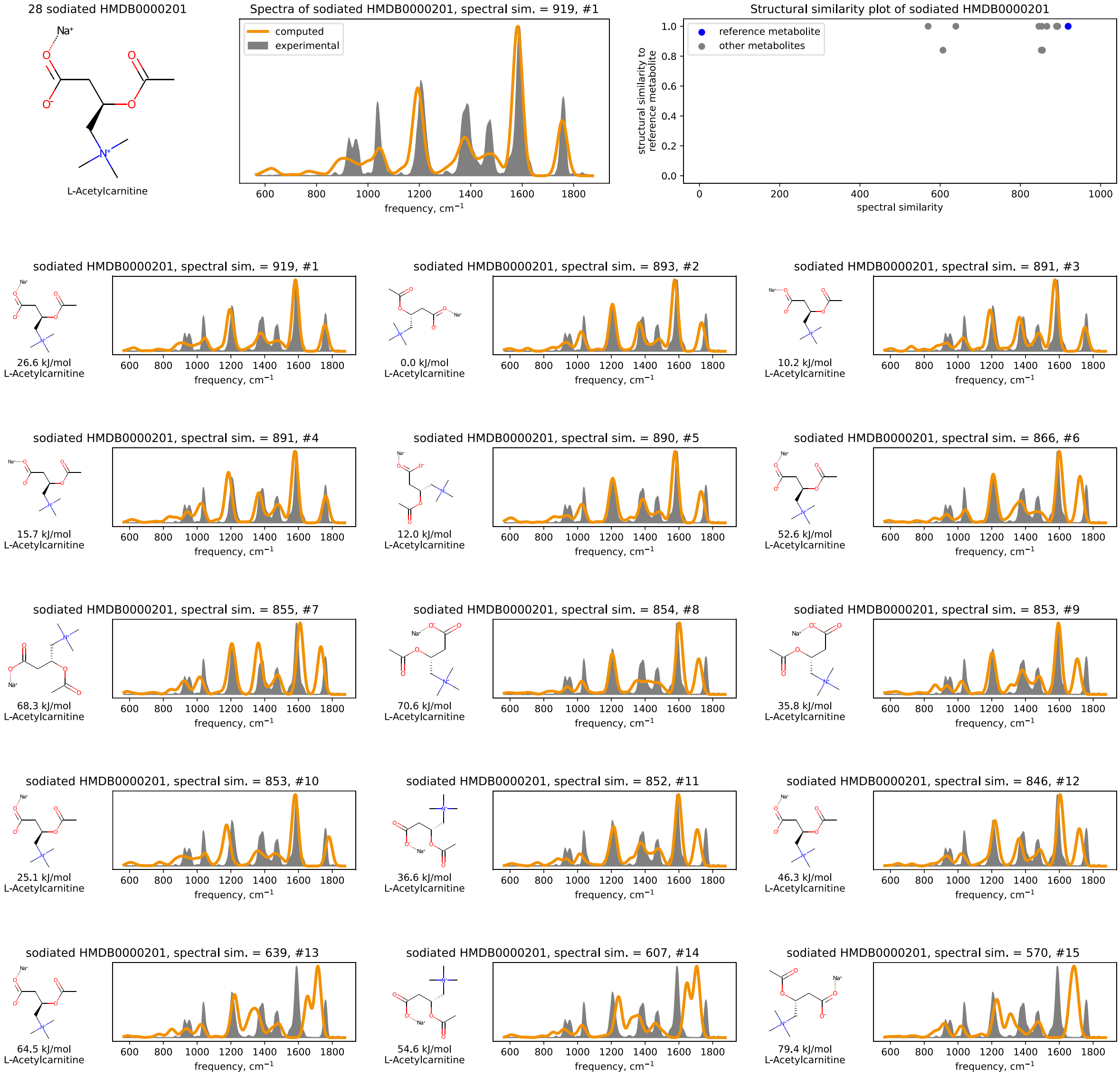
28 sodiated HMDB0000201
L-Acetylcarnitine
Spectra of sodiated HMDB0000201, spectral sim. = 919, #1
computed
experimental
frequency, cm$^{-1}$
Structural similarity plot of sodiated HMDB0000201
reference metabolite
other metabolites
structural similarity to reference metabolite
spectral similarity
sodiated HMDB0000201, spectral sim. = 919, #1
26.6 kJ/mol
L-Acetylcarnitine
sodiated HMDB0000201, spectral sim. = 893, #2
0.0 kJ/mol
L-Acetylcarnitine
sodiated HMDB0000201, spectral sim. = 891, #3
10.2 kJ/mol
L-Acetylcarnitine
sodiated HMDB0000201, spectral sim. = 891, #4
15.7 kJ/mol
L-Acetylcarnitine
sodiated HMDB0000201, spectral sim. = 890, #5
12.0 kJ/mol
L-Acetylcarnitine
sodiated HMDB0000201, spectral sim. = 866, #6
52.6 kJ/mol
L-Acetylcarnitine
sodiated HMDB0000201, spectral sim. = 855, #7
68.3 kJ/mol
L-Acetylcarnitine
sodiated HMDB0000201, spectral sim. = 854, #8
70.6 kJ/mol
L-Acetylcarnitine
sodiated HMDB0000201, spectral sim. = 853, #9
35.8 kJ/mol
L-Acetylcarnitine
sodiated HMDB0000201, spectral sim. = 853, #10
25.1 kJ/mol
L-Acetylcarnitine
sodiated HMDB0000201, spectral sim. = 852, #11
36.6 kJ/mol
L-Acetylcarnitine
sodiated HMDB0000201, spectral sim. = 846, #12
46.3 kJ/mol
L-Acetylcarnitine
sodiated HMDB0000201, spectral sim. = 639, #13
64.5 kJ/mol
L-Acetylcarnitine
sodiated HMDB0000201, spectral sim. = 607, #14
54.6 kJ/mol
L-Acetylcarnitine
sodiated HMDB0000201, spectral sim. = 570, #15
79.4 kJ/mol
L-Acetylcarnitine

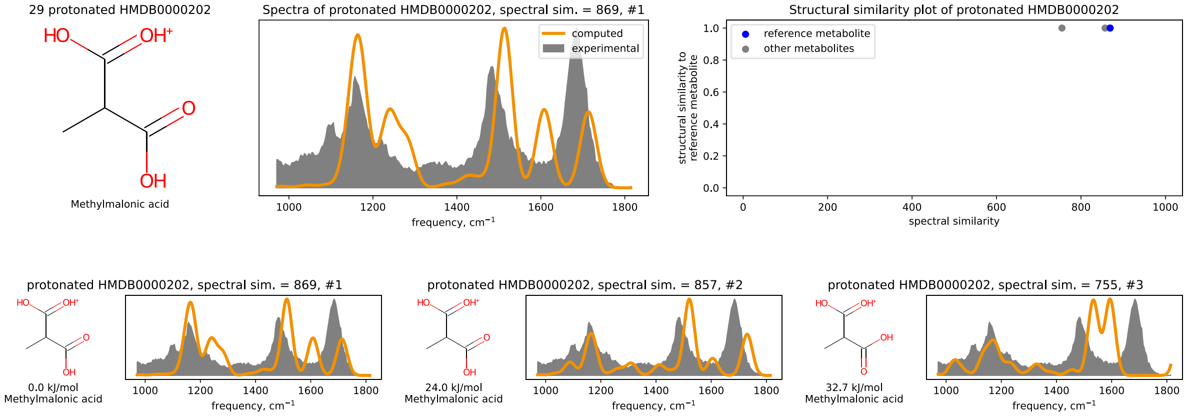
29 protonated HMDB0000202
Methylmalonic acid
Spectra of protonated HMDB0000202, spectral sim. = 869, #1
computed
experimental
frequency, cm$^{-1}$
Structural similarity plot of protonated HMDB0000202
reference metabolite
other metabolites
structural similarity to reference metabolite
spectral similarity
protonated HMDB0000202, spectral sim. = 869, #1
0.0 kJ/mol
Methylmalonic acid
protonated HMDB0000202, spectral sim. = 857, #2
24.0 kJ/mol
Methylmalonic acid
protonated HMDB0000202, spectral sim. = 755, #3
32.7 kJ/mol
Methylmalonic acid

30 deprotonated HMDB0000208

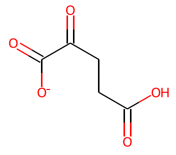


Oxoglutaric acid

Spectra of deprotonated HMDB0000208, spectral sim. = 853, #1

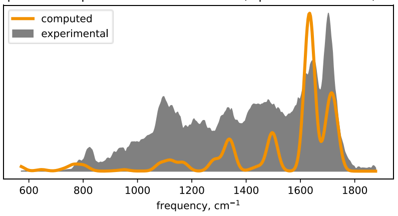


Structural similarity plot of deprotonated HMDB0000208

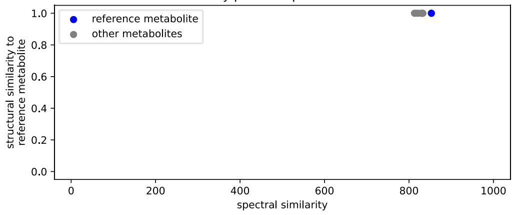


deprotonated HMDB0000208, spectral sim. = 853, #1

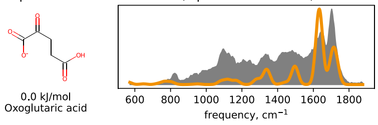


deprotonated HMDB0000208, spectral sim. = 833, #2

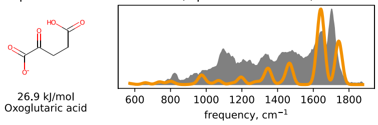


deprotonated HMDB0000208, spectral sim. = 831, #3

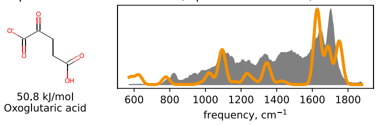


deprotonated HMDB0000208, spectral sim. = 830, #4

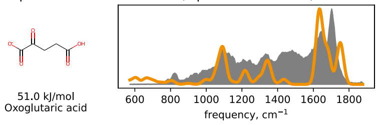


deprotonated HMDB0000208, spectral sim. = 823, #5

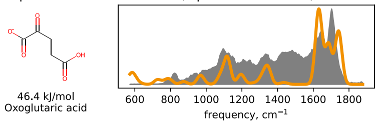


deprotonated HMDB0000208, spectral sim. = 818, #6

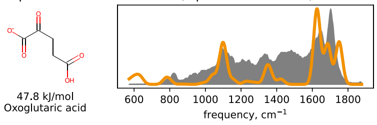


deprotonated HMDB0000208, spectral sim. = 813, #7

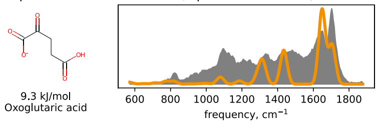


31 sodiated HMDB0000208

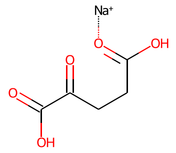


Oxoglutaric acid

Spectra of sodiated HMDB0000208, spectral sim. = 919, #1

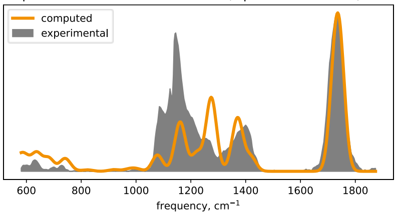


Structural similarity plot of sodiated HMDB0000208

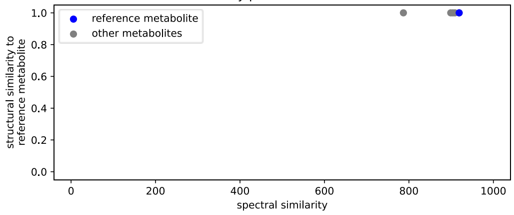


sodiated HMDB0000208, spectral sim. = 919, #1

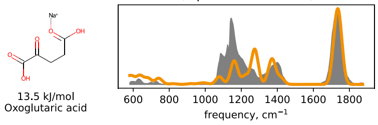


sodiated HMDB0000208, spectral sim. = 909, #2

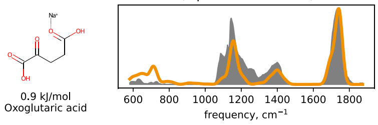


sodiated HMDB0000208, spectral sim. = 904, #3

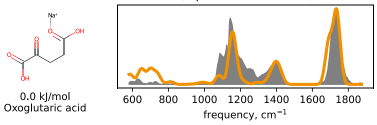


sodiated HMDB0000208, spectral sim. = 899, #4

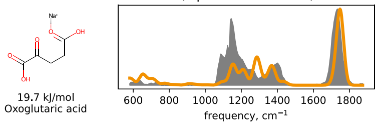


sodiated HMDB0000208, spectral sim. = 787, #5

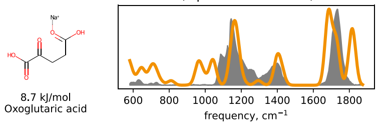

32 deprotonated HMDB0000211

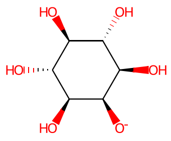


myo-Inositol

Spectra of deprotonated HMDB0000211, spectral sim. = 845, #1

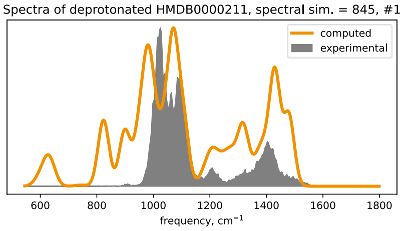


Structural similarity plot of deprotonated HMDB0000211

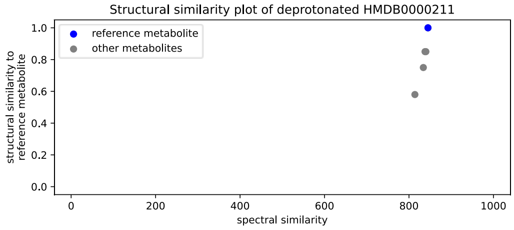


deprotonated HMDB0000211, spectral sim. = 845, #1

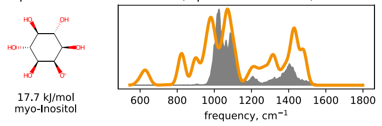


deprotonated HMDB0000211, spectral sim. = 840, #2

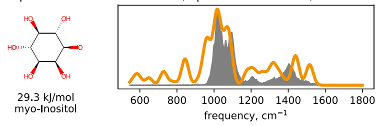


deprotonated HMDB0000211, spectral sim. = 838, #3

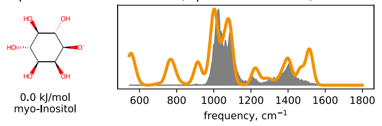


deprotonated HMDB0000211, spectral sim. = 834, #4

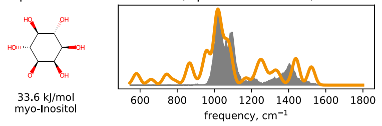


deprotonated HMDB0000211, spectral sim. = 814, #5

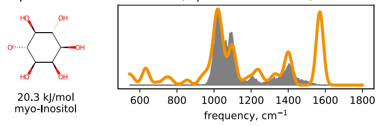


33 deprotonated HMDB0000214

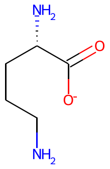


Ornithine

Spectra of deprotonated HMDB0000214, spectral sim. = 918, #1

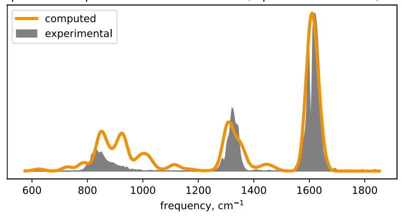


Structural similarity plot of deprotonated HMDB0000214

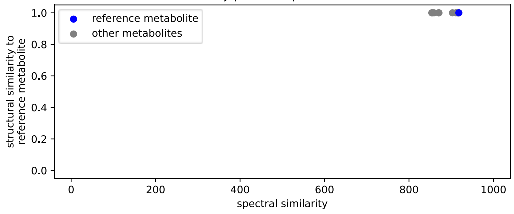


deprotonated HMDB0000214, spectral sim. = 918, #1

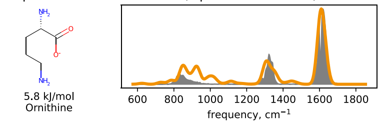


deprotonated HMDB0000214, spectral sim. = 916, #2

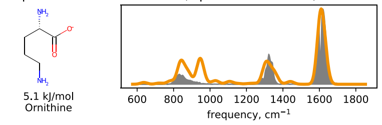


deprotonated HMDB0000214, spectral sim. = 915, #3

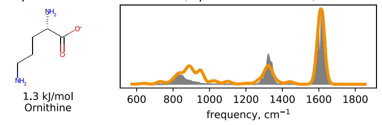


deprotonated HMDB0000214, spectral sim. = 911, #4

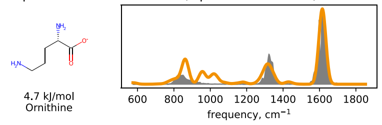


deprotonated HMDB0000214, spectral sim. = 903, #5

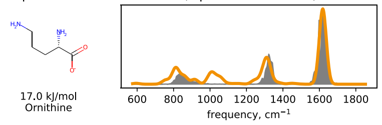


deprotonated HMDB0000214, spectral sim. = 871, #6

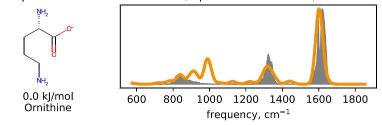


deprotonated HMDB0000214, spectral sim. = 870, #7

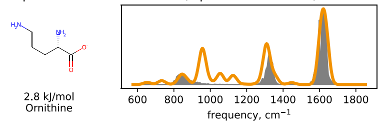


deprotonated HMDB0000214, spectral sim. = 859, #8

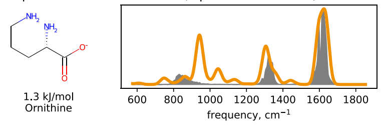


deprotonated HMDB0000214, spectral sim. = 855, #9

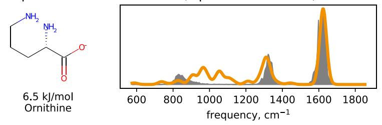


deprotonated HMDB0000214, spectral sim. = 854, #10

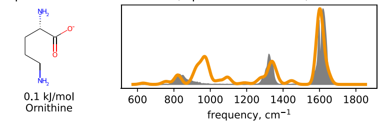

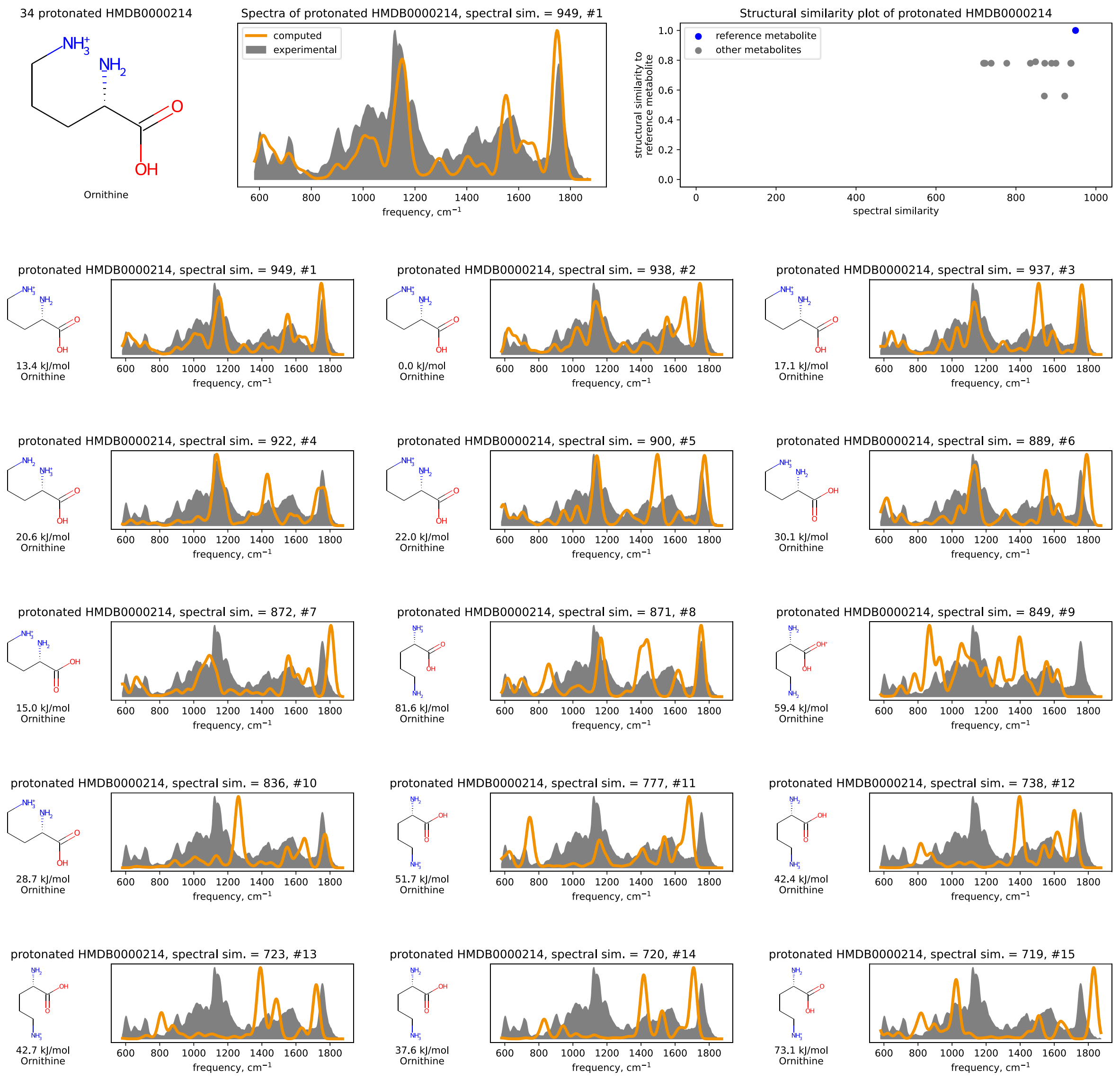
34 protonated HMDB0000214
Ornithine
Spectra of protonated HMDB0000214, spectral sim. = 949, #1
computed
experimental
frequency, cm⁻¹
Structural similarity plot of protonated HMDB0000214
reference metabolite
other metabolites
structural similarity to reference metabolite
spectral similarity
protonated HMDB0000214, spectral sim. = 949, #1
13.4 kJ/mol
Ornithine
protonated HMDB0000214, spectral sim. = 938, #2
0.0 kJ/mol
Ornithine
protonated HMDB0000214, spectral sim. = 937, #3
17.1 kJ/mol
Ornithine
protonated HMDB0000214, spectral sim. = 922, #4
20.6 kJ/mol
Ornithine
protonated HMDB0000214, spectral sim. = 900, #5
22.0 kJ/mol
Ornithine
protonated HMDB0000214, spectral sim. = 889, #6
30.1 kJ/mol
Ornithine
protonated HMDB0000214, spectral sim. = 872, #7
15.0 kJ/mol
Ornithine
protonated HMDB0000214, spectral sim. = 871, #8
81.6 kJ/mol
Ornithine
protonated HMDB0000214, spectral sim. = 849, #9
59.4 kJ/mol
Ornithine
protonated HMDB0000214, spectral sim. = 836, #10
28.7 kJ/mol
Ornithine
protonated HMDB0000214, spectral sim. = 777, #11
51.7 kJ/mol
Ornithine
protonated HMDB0000214, spectral sim. = 738, #12
42.4 kJ/mol
Ornithine
protonated HMDB0000214, spectral sim. = 723, #13
42.7 kJ/mol
Ornithine
protonated HMDB0000214, spectral sim. = 720, #14
37.6 kJ/mol
Ornithine
protonated HMDB0000214, spectral sim. = 719, #15
73.1 kJ/mol
Ornithine
frequency, cm⁻¹

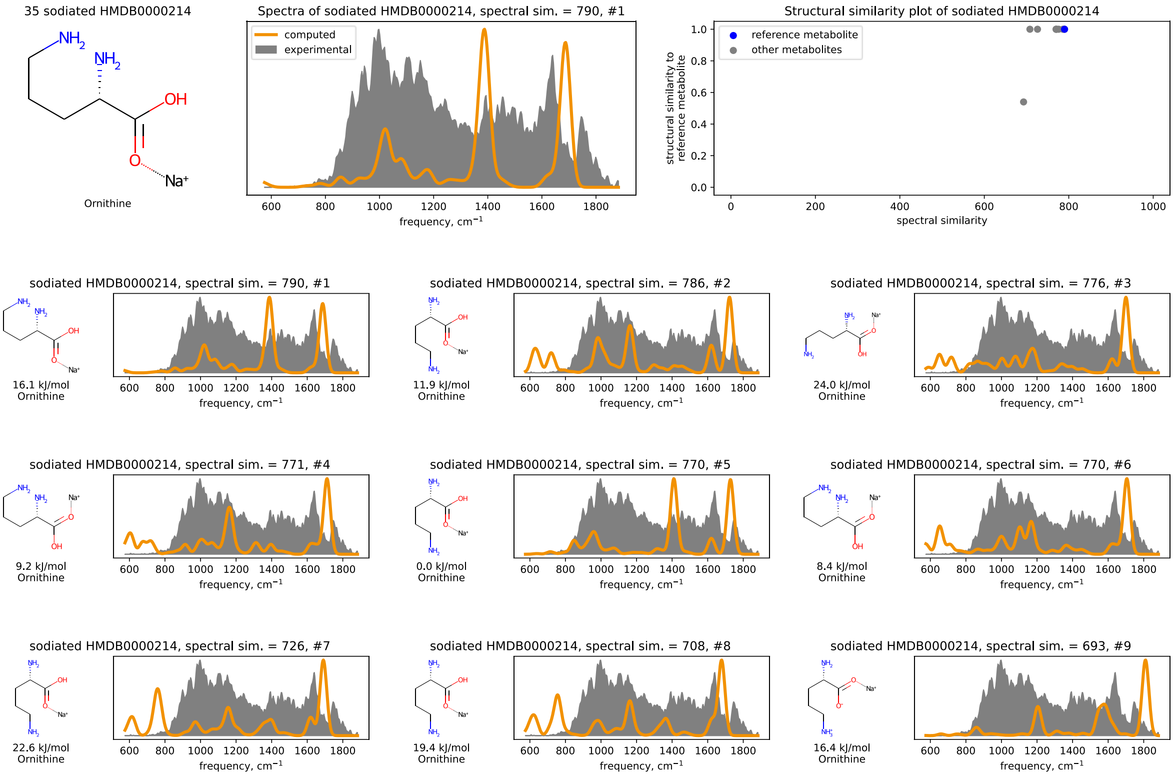
35 sodiated HMDB0000214
Ornithine
Spectra of sodiated HMDB0000214, spectral sim. = 790, #1
computed
experimental
frequency, cm$^{-1}$
Structural similarity plot of sodiated HMDB0000214
reference metabolite
other metabolites
structural similarity to reference metabolite
spectral similarity
sodiated HMDB0000214, spectral sim. = 790, #1
16.1 kJ/mol
Ornithine
sodiated HMDB0000214, spectral sim. = 786, #2
11.9 kJ/mol
Ornithine
sodiated HMDB0000214, spectral sim. = 776, #3
24.0 kJ/mol
Ornithine
sodiated HMDB0000214, spectral sim. = 771, #4
9.2 kJ/mol
Ornithine
sodiated HMDB0000214, spectral sim. = 770, #5
0.0 kJ/mol
Ornithine
sodiated HMDB0000214, spectral sim. = 770, #6
8.4 kJ/mol
Ornithine
sodiated HMDB0000214, spectral sim. = 726, #7
22.6 kJ/mol
Ornithine
sodiated HMDB0000214, spectral sim. = 708, #8
19.4 kJ/mol
Ornithine
sodiated HMDB0000214, spectral sim. = 693, #9
16.4 kJ/mol
Ornithine
frequency, cm$^{-1}$

36 deprotonated HMDB0000247

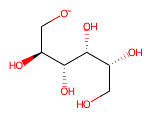


Sorbitol

Spectra of deprotonated HMDB0000247, spectral sim. = 958, #1

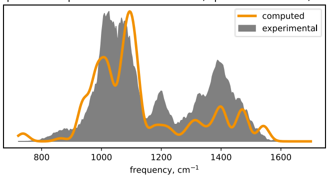


Structural similarity plot of deprotonated HMDB0000247

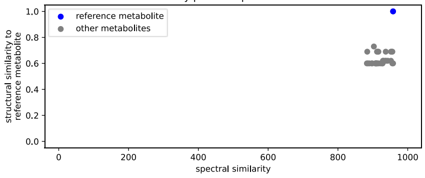


deprotonated HMDB0000247, spectral sim. = 958, #1

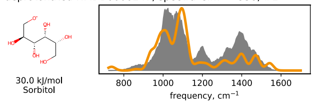

30.0 kJ/mol
Sorbitol

deprotonated HMDB0000247, spectral sim. = 958, #2

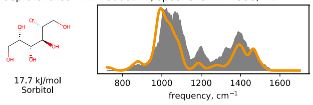

17.7 kJ/mol
Sorbitol

deprotonated HMDB0000247, spectral sim. = 956, #3

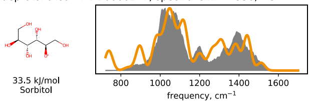

33.5 kJ/mol
Sorbitol

deprotonated HMDB0000247, spectral sim. = 956, #4

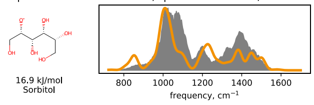

16.9 kJ/mol
Sorbitol

deprotonated HMDB0000247, spectral sim. = 954, #5

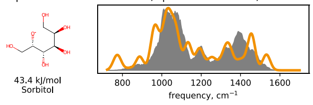

43.4 kJ/mol
Sorbitol

deprotonated HMDB0000247, spectral sim. = 952, #6

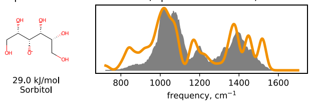

29.0 kJ/mol
Sorbitol

deprotonated HMDB0000247, spectral sim. = 952, #7

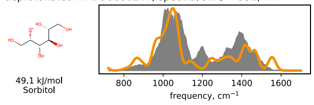

49.1 kJ/mol
Sorbitol

deprotonated HMDB0000247, spectral sim. = 943, #8

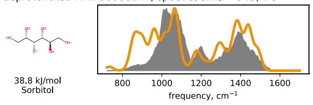

38.8 kJ/mol
Sorbitol

deprotonated HMDB0000247, spectral sim. = 938, #9

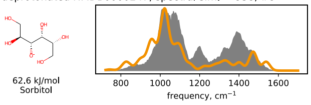

62.6 kJ/mol
Sorbitol

deprotonated HMDB0000247, spectral sim. = 937, #10

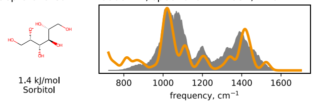

1.4 kJ/mol
Sorbitol

deprotonated HMDB0000247, spectral sim. = 935, #11

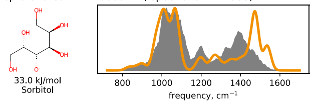

33.0 kJ/mol
Sorbitol

deprotonated HMDB0000247, spectral sim. = 930, #12

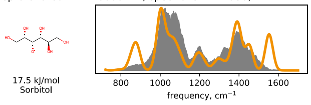

17.5 kJ/mol
Sorbitol

deprotonated HMDB0000247, spectral sim. = 929, #13

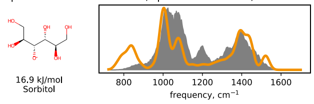

16.9 kJ/mol
Sorbitol

deprotonated HMDB0000247, spectral sim. = 928, #14

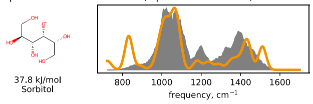

37.8 kJ/mol
Sorbitol

deprotonated HMDB0000247, spectral sim. = 927, #15

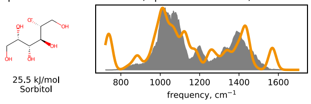

25.5 kJ/mol
Sorbitol

deprotonated HMDB0000247, spectral sim. = 923, #16

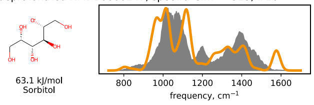

63.1 kJ/mol
Sorbitol

deprotonated HMDB0000247, spectral sim. = 917, #17

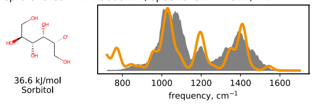

36.6 kJ/mol
Sorbitol

deprotonated HMDB0000247, spectral sim. = 916, #18

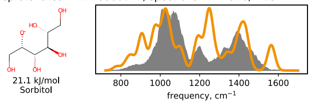

21.1 kJ/mol
Sorbitol

deprotonated HMDB0000247, spectral sim. = 914, #19

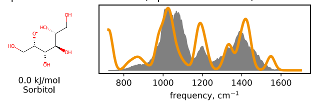

0.0 kJ/mol
Sorbitol

deprotonated HMDB0000247, spectral sim. = 913, #20

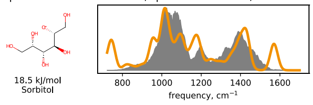

18.5 kJ/mol
Sorbitol

deprotonated HMDB0000247, spectral sim. = 912, #21

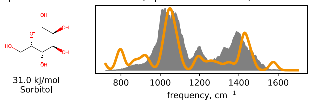

31.0 kJ/mol
Sorbitol

deprotonated HMDB0000247, spectral sim. = 912, #22

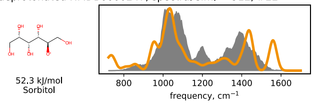

52.3 kJ/mol
Sorbitol

deprotonated HMDB0000247, spectral sim. = 910, #23

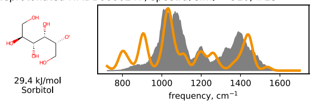

29.4 kJ/mol
Sorbitol

deprotonated HMDB0000247, spectral sim. = 909, #24

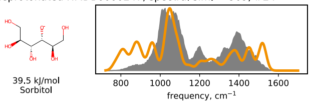

39.5 kJ/mol
Sorbitol

deprotonated HMDB0000247, spectral sim. = 908, #25

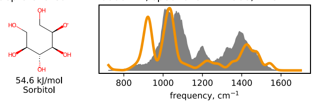

54.6 kJ/mol
Sorbitol

deprotonated HMDB0000247, spectral sim. = 907, #26

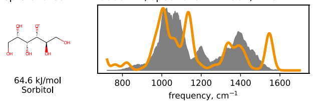

64.6 kJ/mol
Sorbitol

deprotonated HMDB0000247, spectral sim. = 903, #27

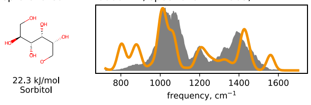

22.3 kJ/mol
Sorbitol

deprotonated HMDB0000247, spectral sim. = 898, #28

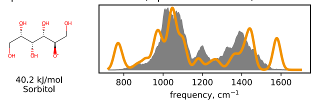

40.2 kJ/mol
Sorbitol

deprotonated HMDB0000247, spectral sim. = 897, #29

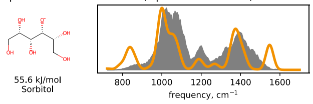

55.6 kJ/mol
Sorbitol

deprotonated HMDB0000247, spectral sim. = 890, #30

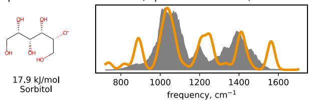

17.9 kJ/mol
Sorbitol

deprotonated HMDB0000247, spectral sim. = 886, #31

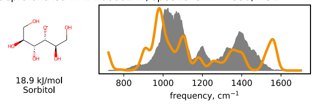

18.9 kJ/mol
Sorbitol

deprotonated HMDB0000247, spectral sim. = 884, #32

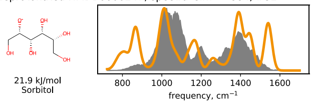

21.9 kJ/mol
Sorbitol

deprotonated HMDB0000247, spectral sim. = 883, #33

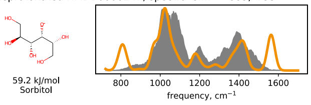

59.2 kJ/mol
Sorbitol

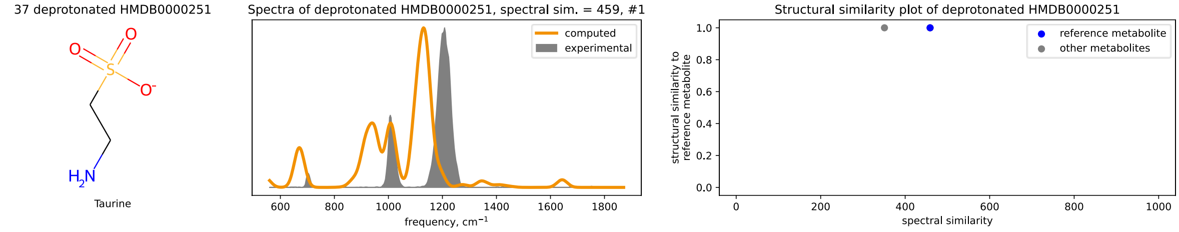
37 deprotonated HMDB0000251
Taurine
Spectra of deprotonated HMDB0000251, spectral sim. = 459, #1
computed
experimental
frequency, cm$^{-1}$
Structural similarity plot of deprotonated HMDB0000251
reference metabolite
other metabolites
structural similarity to reference metabolite
spectral similarity

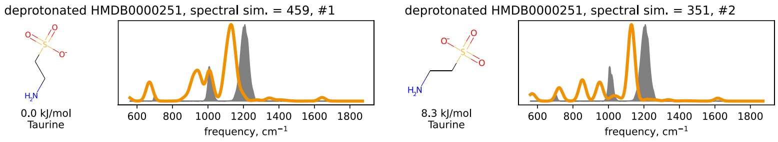
deprotonated HMDB0000251, spectral sim. = 459, #1
0.0 kJ/mol
Taurine
frequency, cm$^{-1}$
deprotonated HMDB0000251, spectral sim. = 351, #2
8.3 kJ/mol
Taurine
frequency, cm$^{-1}$

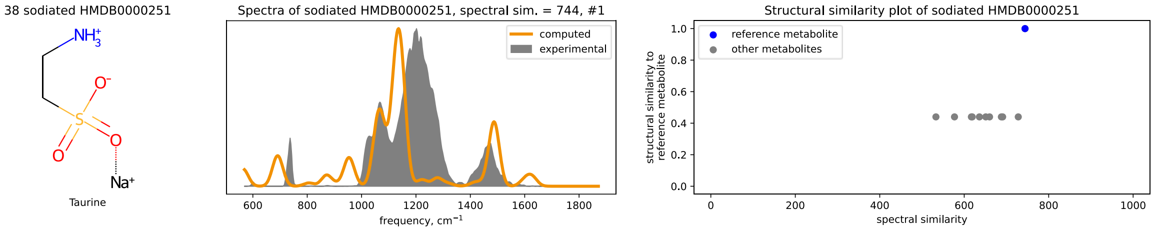
38 sodiated HMDB0000251
Taurine
Spectra of sodiated HMDB0000251, spectral sim. = 744, #1
computed
experimental
frequency, cm$^{-1}$
Structural similarity plot of sodiated HMDB0000251
reference metabolite
other metabolites
structural similarity to reference metabolite
spectral similarity

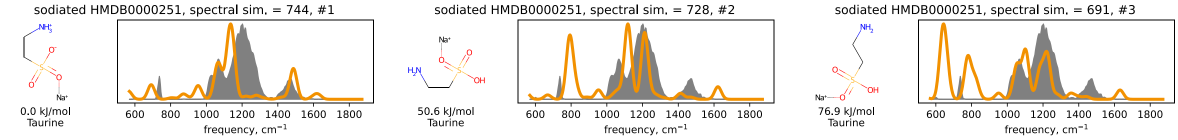
sodiated HMDB0000251, spectral sim. = 744, #1
0.0 kJ/mol
Taurine
sodiated HMDB0000251, spectral sim. = 728, #2
50.6 kJ/mol
Taurine
sodiated HMDB0000251, spectral sim. = 691, #3
76.9 kJ/mol
Taurine
frequency, cm$^{-1}$

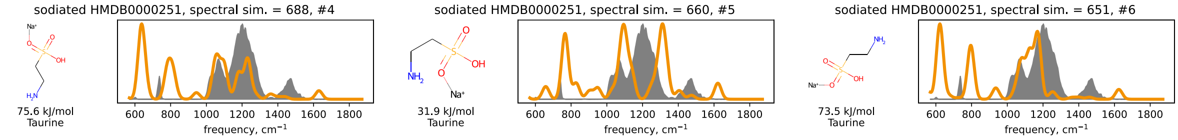
sodiated HMDB0000251, spectral sim. = 688, #4
75.6 kJ/mol
Taurine
sodiated HMDB0000251, spectral sim. = 660, #5
31.9 kJ/mol
Taurine
sodiated HMDB0000251, spectral sim. = 651, #6
73.5 kJ/mol
Taurine
frequency, cm$^{-1}$

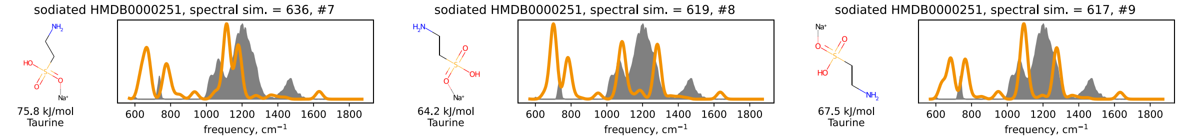
sodiated HMDB0000251, spectral sim. = 636, #7
75.8 kJ/mol
Taurine
sodiated HMDB0000251, spectral sim. = 619, #8
64.2 kJ/mol
Taurine
sodiated HMDB0000251, spectral sim. = 617, #9
67.5 kJ/mol
Taurine
frequency, cm$^{-1}$

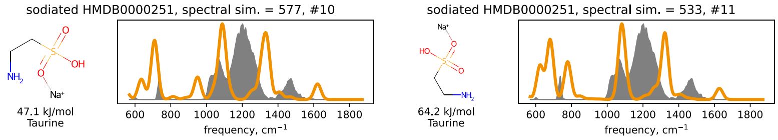
sodiated HMDB0000251, spectral sim. = 577, #10
47.1 kJ/mol
Taurine
sodiated HMDB0000251, spectral sim. = 533, #11
64.2 kJ/mol
Taurine
frequency, cm$^{-1}$

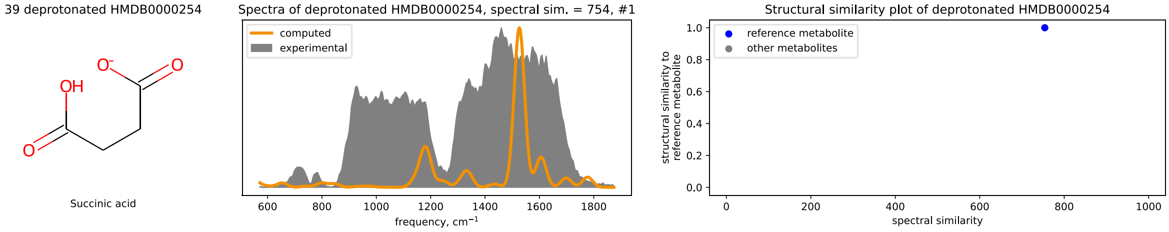
39 deprotonated HMDB0000254
Succinic acid
Spectra of deprotonated HMDB0000254, spectral sim. = 754, #1
computed
experimental
frequency, cm$^{-1}$
Structural similarity plot of deprotonated HMDB0000254
reference metabolite
other metabolites
structural similarity to reference metabolite
spectral similarity

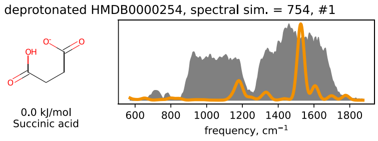
deprotonated HMDB0000254, spectral sim. = 754, #1
0.0 kJ/mol
Succinic acid
frequency, cm$^{-1}$

40 protonated HMDB0000254

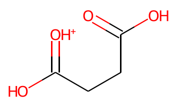

Succinic acid

Spectra of protonated HMDB0000254, spectral sim. = 772, #1

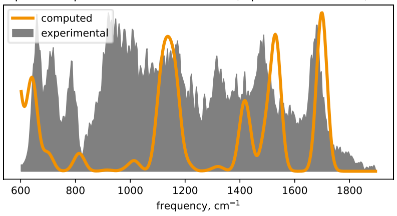


Structural similarity plot of protonated HMDB0000254

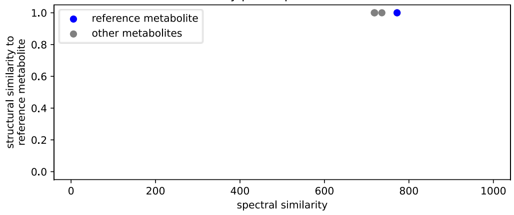


protonated HMDB0000254, spectral sim. = 772, #1

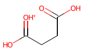

65.9 kJ/mol
Succinic acid

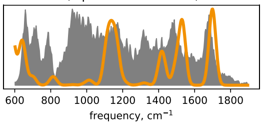

protonated HMDB0000254, spectral sim. = 736, #2

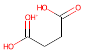

47.1 kJ/mol
Succinic acid

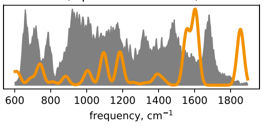

protonated HMDB0000254, spectral sim. = 719, #3

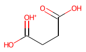

29.5 kJ/mol
Succinic acid

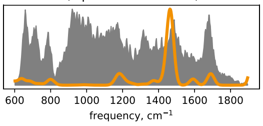

protonated HMDB0000254, spectral sim. = 718, #4

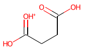

0.0 kJ/mol
Succinic acid

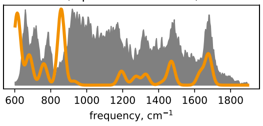

41 deprotonated HMDB0000263

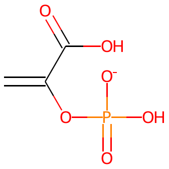

Phosphoenolpyruvic acid

Spectra of deprotonated HMDB0000263, spectral sim. = 841, #1

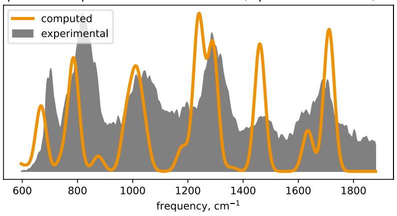


Structural similarity plot of deprotonated HMDB0000263

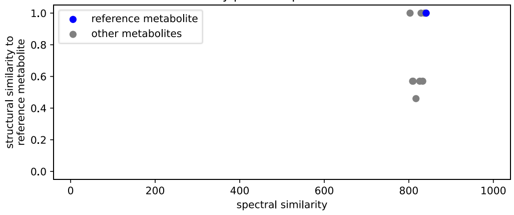


deprotonated HMDB0000263, spectral sim. = 841, #1

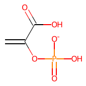

2.0 kJ/mol
Phosphoenolpyruvic acid

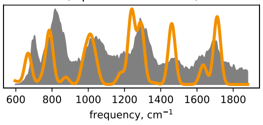

deprotonated HMDB0000263, spectral sim. = 837, #2

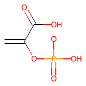

1.7 kJ/mol
Phosphoenolpyruvic acid

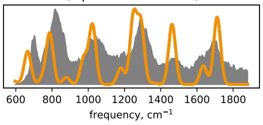

deprotonated HMDB0000263, spectral sim. = 833, #3

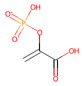

14.2 kJ/mol
Phosphoenolpyruvic acid

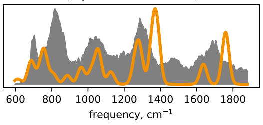

deprotonated HMDB0000263, spectral sim. = 829, #4

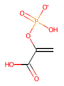

13.6 kJ/mol
Phosphoenolpyruvic acid

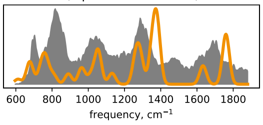

deprotonated HMDB0000263, spectral sim. = 826, #5

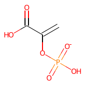

17.0 kJ/mol
Phosphoenolpyruvic acid

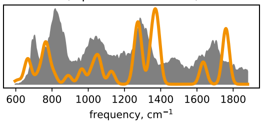

deprotonated HMDB0000263, spectral sim. = 817, #6

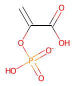

0.0 kJ/mol
Phosphoenolpyruvic acid

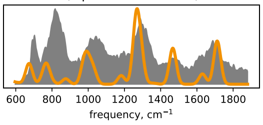

deprotonated HMDB0000263, spectral sim. = 810, #7

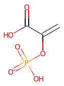

1.3 kJ/mol
Phosphoenolpyruvic acid

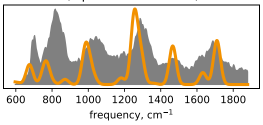

deprotonated HMDB0000263, spectral sim. = 809, #8

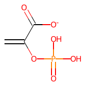

11.0 kJ/mol
Phosphoenolpyruvic acid

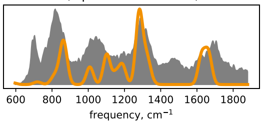

deprotonated HMDB0000263, spectral sim. = 803, #9

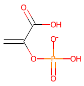

49.0 kJ/mol
Phosphoenolpyruvic acid

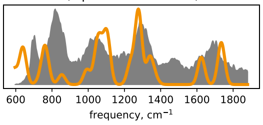

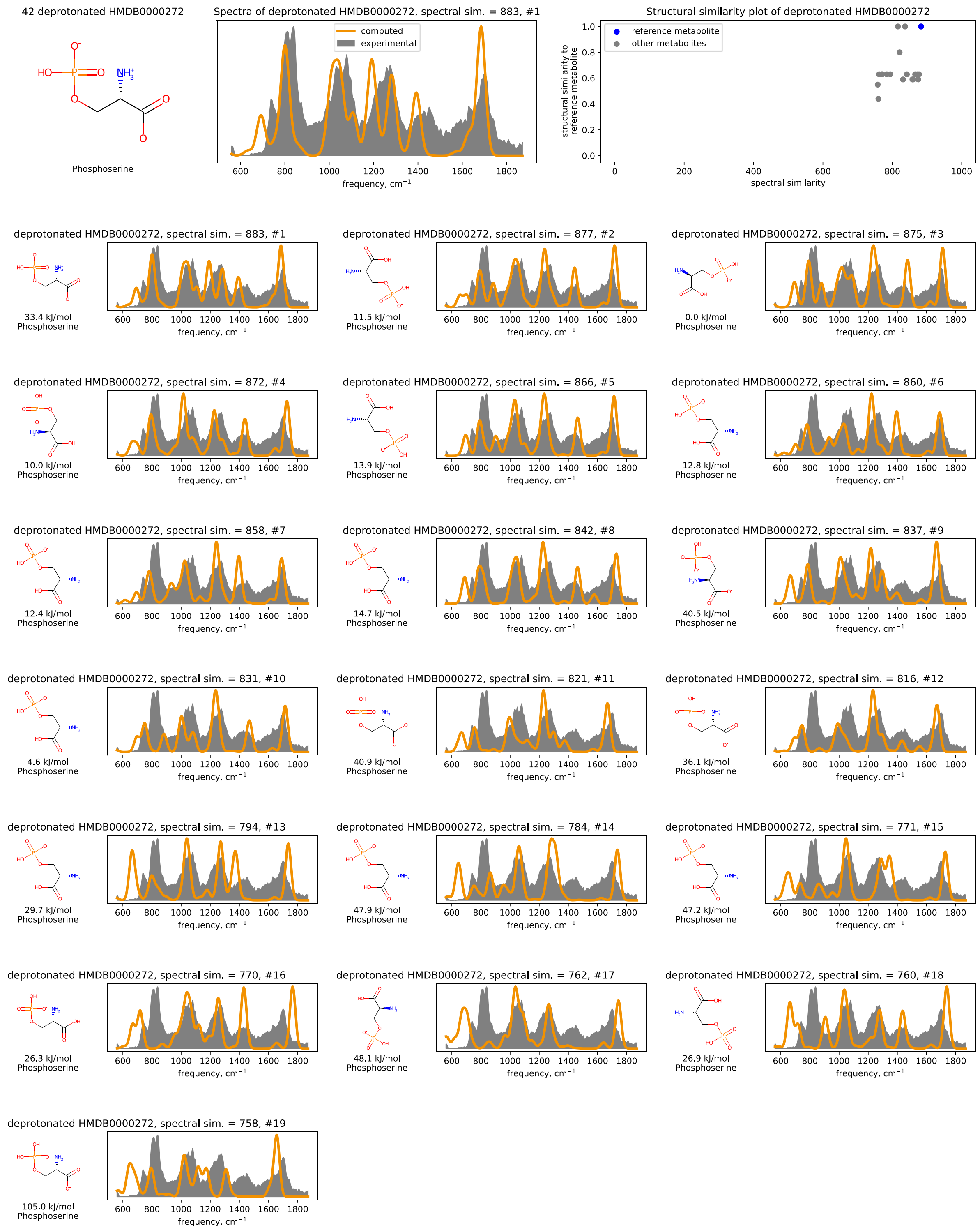

42 deprotonated HMDB0000272
Phosphoserine
Spectra of deprotonated HMDB0000272, spectral sim. = 883, #1
computed
experimental
frequency, cm⁻¹
Structural similarity plot of deprotonated HMDB0000272
reference metabolite
other metabolites
structural similarity to reference metabolite
spectral similarity
deprotonated HMDB0000272, spectral sim. = 883, #1
33.4 kJ/mol
Phosphoserine
deprotonated HMDB0000272, spectral sim. = 877, #2
11.5 kJ/mol
Phosphoserine
deprotonated HMDB0000272, spectral sim. = 875, #3
0.0 kJ/mol
Phosphoserine
deprotonated HMDB0000272, spectral sim. = 872, #4
10.0 kJ/mol
Phosphoserine
deprotonated HMDB0000272, spectral sim. = 866, #5
13.9 kJ/mol
Phosphoserine
deprotonated HMDB0000272, spectral sim. = 860, #6
12.8 kJ/mol
Phosphoserine
deprotonated HMDB0000272, spectral sim. = 858, #7
12.4 kJ/mol
Phosphoserine
deprotonated HMDB0000272, spectral sim. = 842, #8
14.7 kJ/mol
Phosphoserine
deprotonated HMDB0000272, spectral sim. = 837, #9
40.5 kJ/mol
Phosphoserine
deprotonated HMDB0000272, spectral sim. = 831, #10
4.6 kJ/mol
Phosphoserine
deprotonated HMDB0000272, spectral sim. = 821, #11
40.9 kJ/mol
Phosphoserine
deprotonated HMDB0000272, spectral sim. = 816, #12
36.1 kJ/mol
Phosphoserine
deprotonated HMDB0000272, spectral sim. = 794, #13
29.7 kJ/mol
Phosphoserine
deprotonated HMDB0000272, spectral sim. = 784, #14
47.9 kJ/mol
Phosphoserine
deprotonated HMDB0000272, spectral sim. = 771, #15
47.2 kJ/mol
Phosphoserine
deprotonated HMDB0000272, spectral sim. = 770, #16
26.3 kJ/mol
Phosphoserine
deprotonated HMDB0000272, spectral sim. = 762, #17
48.1 kJ/mol
Phosphoserine
deprotonated HMDB0000272, spectral sim. = 760, #18
26.9 kJ/mol
Phosphoserine
deprotonated HMDB0000272, spectral sim. = 758, #19
105.0 kJ/mol
Phosphoserine

43 protonated HMDB0000272

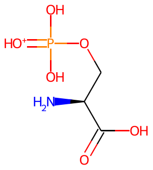


Phosphoserine

Spectra of protonated HMDB0000272, spectral sim. = 894, #1

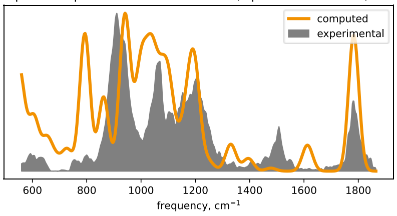


Structural similarity plot of protonated HMDB0000272

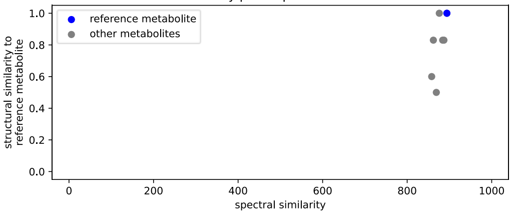


protonated HMDB0000272, spectral sim. = 894, #1

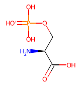

34.0 kJ/mol
Phosphoserine

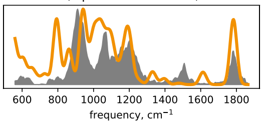


protonated HMDB0000272, spectral sim. = 887, #2

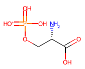

36.2 kJ/mol
Phosphoserine

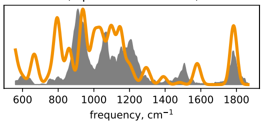


protonated HMDB0000272, spectral sim. = 884, #3

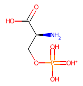

72.8 kJ/mol
Phosphoserine

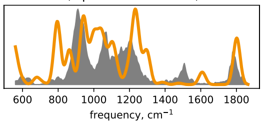


protonated HMDB0000272, spectral sim. = 876, #4

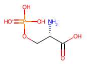

42.0 kJ/mol
Phosphoserine

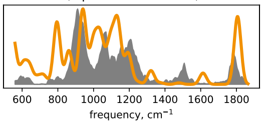


protonated HMDB0000272, spectral sim. = 869, #5

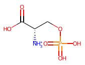

0.0 kJ/mol
Phosphoserine

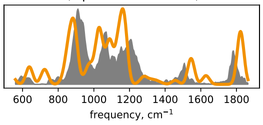


protonated HMDB0000272, spectral sim. = 862, #6

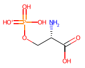

42.2 kJ/mol
Phosphoserine

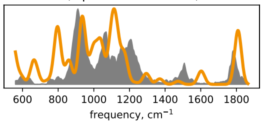


protonated HMDB0000272, spectral sim. = 858, #7

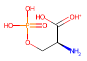

84.5 kJ/mol
Phosphoserine

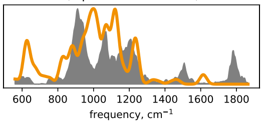

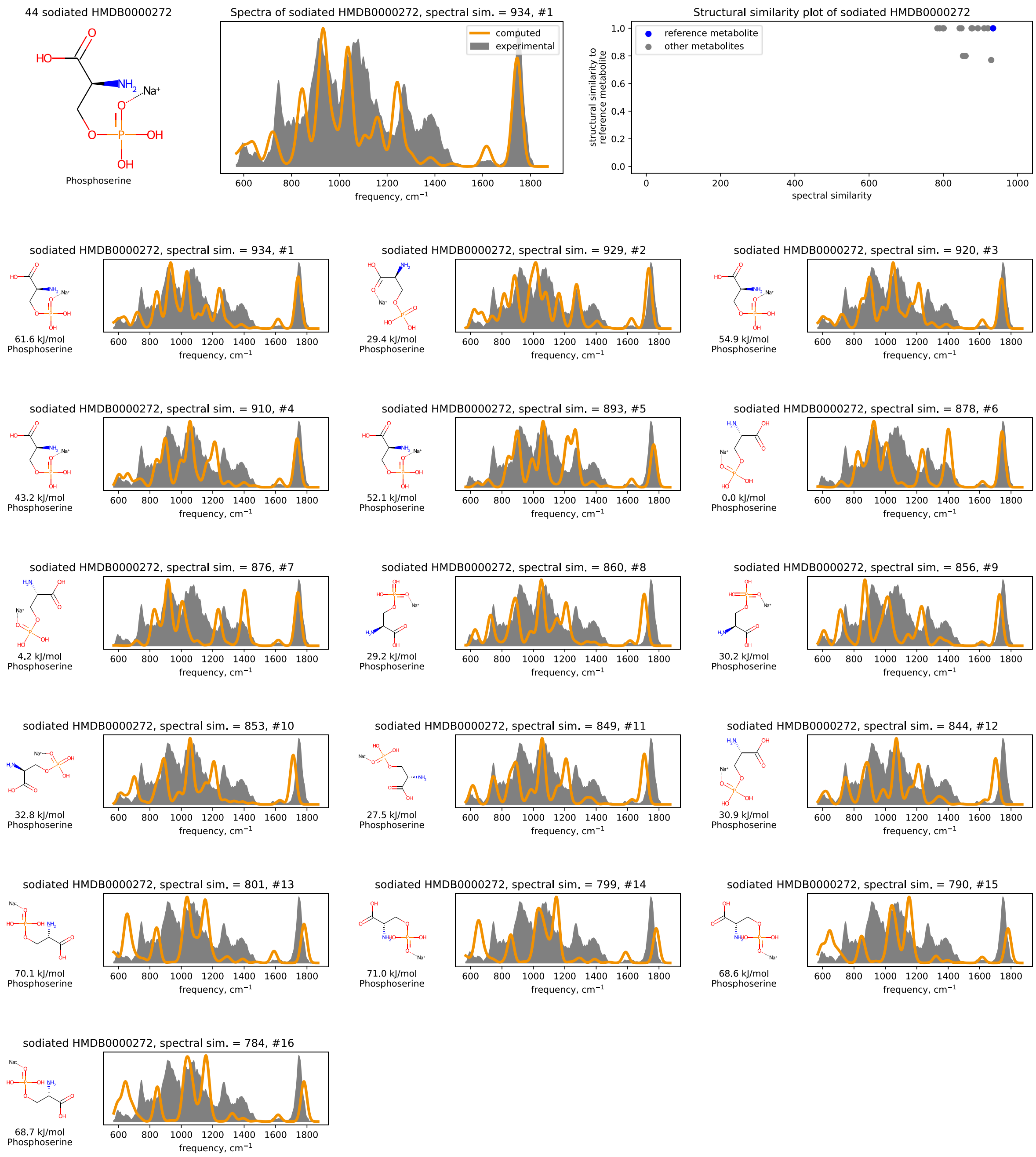
44 sodiated HMDB0000272
Phosphoserine
Spectra of sodiated HMDB0000272, spectral sim. = 934, #1
computed
experimental
frequency, cm⁻¹
Structural similarity plot of sodiated HMDB0000272
reference metabolite
other metabolites
structural similarity to reference metabolite
spectral similarity
sodiated HMDB0000272, spectral sim. = 934, #1
61.6 kJ/mol Phosphoserine
sodiated HMDB0000272, spectral sim. = 929, #2
29.4 kJ/mol Phosphoserine
sodiated HMDB0000272, spectral sim. = 920, #3
54.9 kJ/mol Phosphoserine
sodiated HMDB0000272, spectral sim. = 910, #4
43.2 kJ/mol Phosphoserine
sodiated HMDB0000272, spectral sim. = 893, #5
52.1 kJ/mol Phosphoserine
sodiated HMDB0000272, spectral sim. = 878, #6
0.0 kJ/mol Phosphoserine
sodiated HMDB0000272, spectral sim. = 876, #7
4.2 kJ/mol Phosphoserine
sodiated HMDB0000272, spectral sim. = 860, #8
29.2 kJ/mol Phosphoserine
sodiated HMDB0000272, spectral sim. = 856, #9
30.2 kJ/mol Phosphoserine
sodiated HMDB0000272, spectral sim. = 853, #10
32.8 kJ/mol Phosphoserine
sodiated HMDB0000272, spectral sim. = 849, #11
27.5 kJ/mol Phosphoserine
sodiated HMDB0000272, spectral sim. = 844, #12
30.9 kJ/mol Phosphoserine
sodiated HMDB0000272, spectral sim. = 801, #13
70.1 kJ/mol Phosphoserine
sodiated HMDB0000272, spectral sim. = 799, #14
71.0 kJ/mol Phosphoserine
sodiated HMDB0000272, spectral sim. = 790, #15
68.6 kJ/mol Phosphoserine
sodiated HMDB0000272, spectral sim. = 784, #16
68.7 kJ/mol Phosphoserine

45 deprotonated HMDB0000292

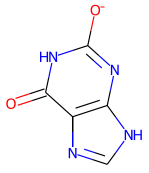

Xanthine

Spectra of deprotonated HMDB0000292, spectral sim. = 789, #1

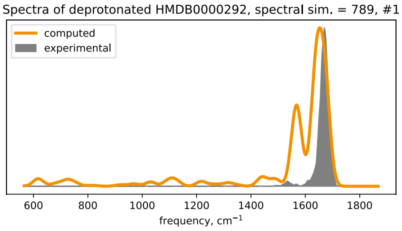


Structural similarity plot of deprotonated HMDB0000292

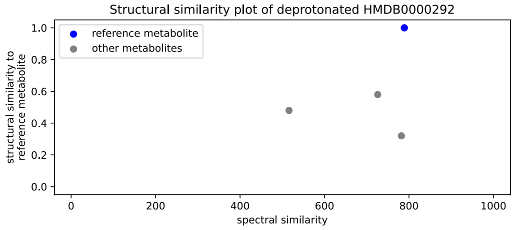


deprotonated HMDB0000292, spectral sim. = 789, #1

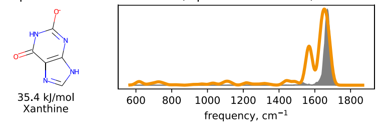


deprotonated HMDB0000292, spectral sim. = 782, #2

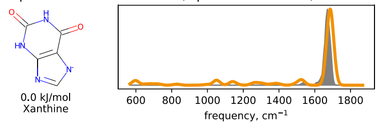


deprotonated HMDB0000292, spectral sim. = 726, #3

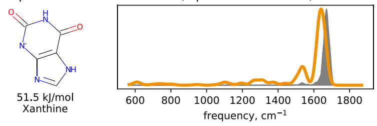


deprotonated HMDB0000292, spectral sim. = 516, #4

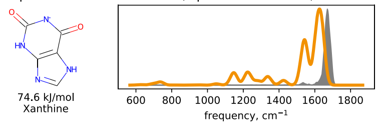


46 protonated HMDB0000292

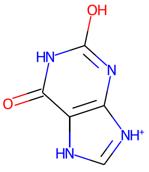

Xanthine

Spectra of protonated HMDB0000292, spectral sim. = 894, #1

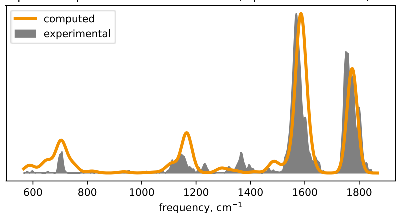


Structural similarity plot of protonated HMDB0000292

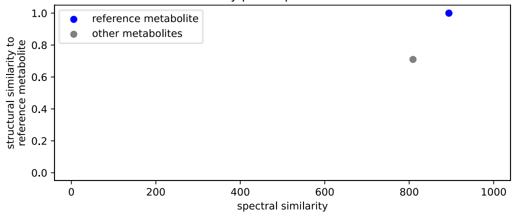


protonated HMDB0000292, spectral sim. = 894, #1

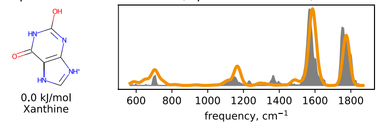


protonated HMDB0000292, spectral sim. = 809, #2

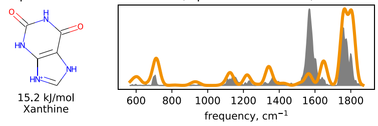


47 deprotonated HMDB0000300

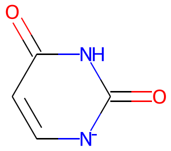

Uracil

Spectra of deprotonated HMDB0000300, spectral sim. = 928, #1

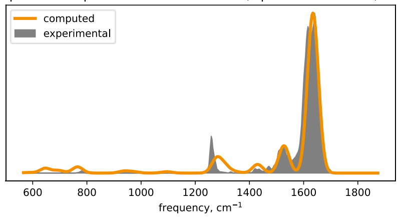


Structural similarity plot of deprotonated HMDB0000300

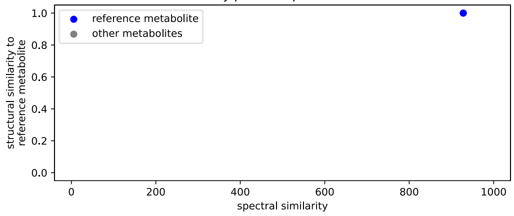


deprotonated HMDB0000300, spectral sim. = 928, #1

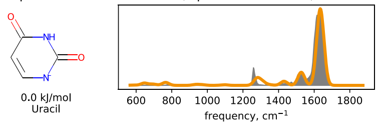

48 protonated HMDB0000300

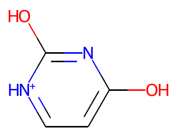


Uracil

Spectra of protonated HMDB0000300, spectral sim. = 816, #1

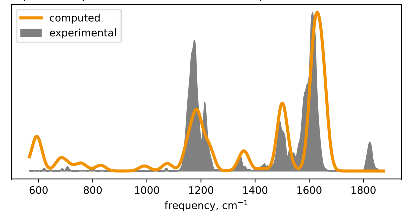


Structural similarity plot of protonated HMDB0000300

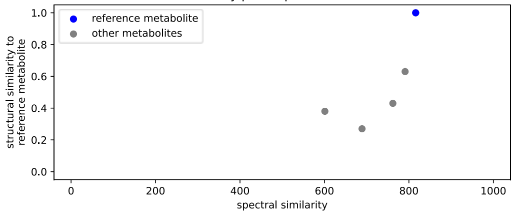


protonated HMDB0000300, spectral sim. = 816, #1

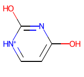

0.0 kJ/mol
Uracil

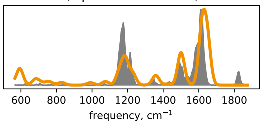

protonated HMDB0000300, spectral sim. = 791, #2

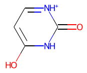

8.9 kJ/mol
Uracil

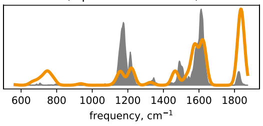

protonated HMDB0000300, spectral sim. = 762, #3

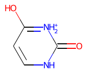

20.8 kJ/mol
Uracil

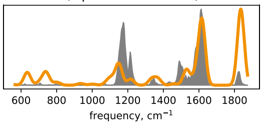

protonated HMDB0000300, spectral sim. = 689, #4

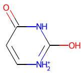

40.3 kJ/mol
Uracil

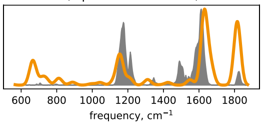

protonated HMDB0000300, spectral sim. = 601, #5

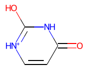

45.5 kJ/mol
Uracil

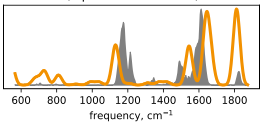

49 sodiated HMDB0000300

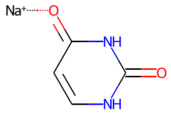


Uracil

Spectra of sodiated HMDB0000300, spectral sim. = 937, #1

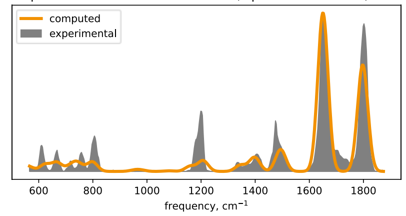


Structural similarity plot of sodiated HMDB0000300

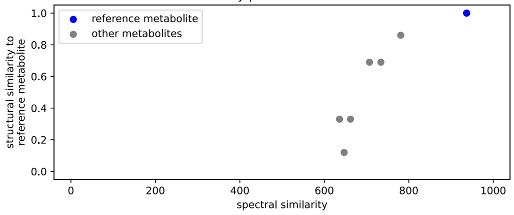


sodiated HMDB0000300, spectral sim. = 937, #1

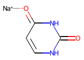

0.0 kJ/mol
Uracil

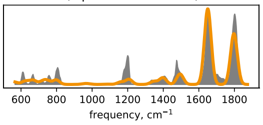

sodiated HMDB0000300, spectral sim. = 781, #2

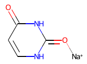

12.3 kJ/mol
Uracil

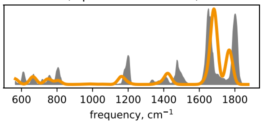

sodiated HMDB0000300, spectral sim. = 734, #3

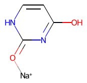

12.8 kJ/mol
Uracil

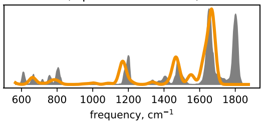

sodiated HMDB0000300, spectral sim. = 707, #4

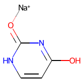

16.0 kJ/mol
Uracil

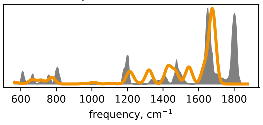

sodiated HMDB0000300, spectral sim. = 662, #5

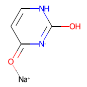

47.9 kJ/mol
Uracil

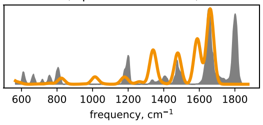

sodiated HMDB0000300, spectral sim. = 647, #6

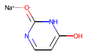

31.6 kJ/mol
Uracil

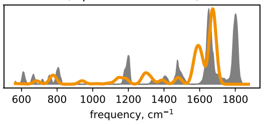

sodiated HMDB0000300, spectral sim. = 636, #7

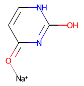

29.3 kJ/mol
Uracil

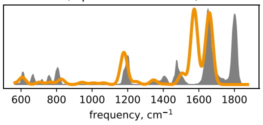

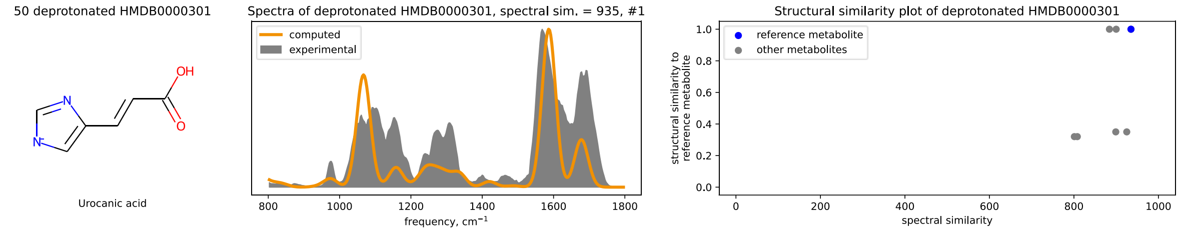
50 deprotonated HMDB0000301
Urocanic acid
Spectra of deprotonated HMDB0000301, spectral sim. = 935, #1
computed
experimental
frequency, cm⁻¹
Structural similarity plot of deprotonated HMDB0000301
reference metabolite
other metabolites
structural similarity to reference metabolite
spectral similarity

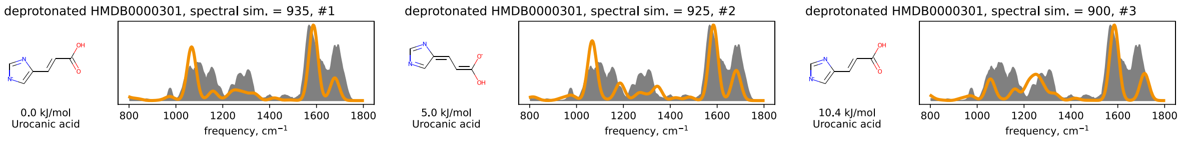
deprotonated HMDB0000301, spectral sim. = 935, #1
0.0 kJ/mol
Urocanic acid
deprotonated HMDB0000301, spectral sim. = 925, #2
5.0 kJ/mol
Urocanic acid
deprotonated HMDB0000301, spectral sim. = 900, #3
10.4 kJ/mol
Urocanic acid
frequency, cm⁻¹

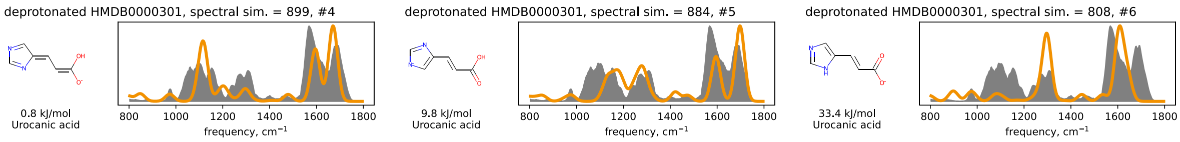
deprotonated HMDB0000301, spectral sim. = 899, #4
0.8 kJ/mol
Urocanic acid
deprotonated HMDB0000301, spectral sim. = 884, #5
9.8 kJ/mol
Urocanic acid
deprotonated HMDB0000301, spectral sim. = 808, #6
33.4 kJ/mol
Urocanic acid
frequency, cm⁻¹

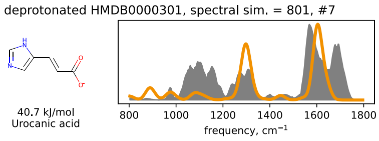
deprotonated HMDB0000301, spectral sim. = 801, #7
40.7 kJ/mol
Urocanic acid
frequency, cm⁻¹

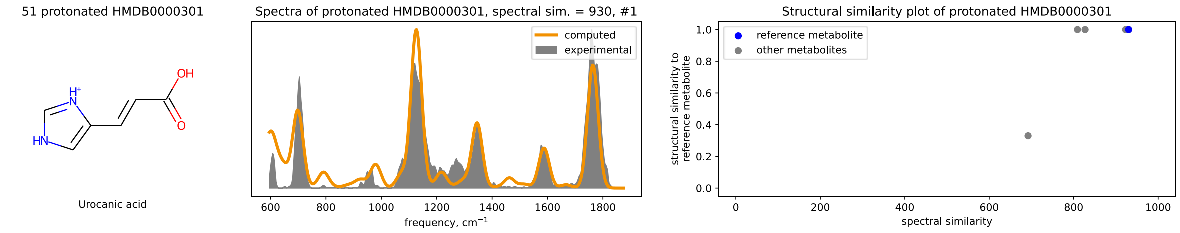
51 protonated HMDB0000301
Urocanic acid
Spectra of protonated HMDB0000301, spectral sim. = 930, #1
computed
experimental
frequency, cm⁻¹
Structural similarity plot of protonated HMDB0000301
reference metabolite
other metabolites
structural similarity to reference metabolite
spectral similarity

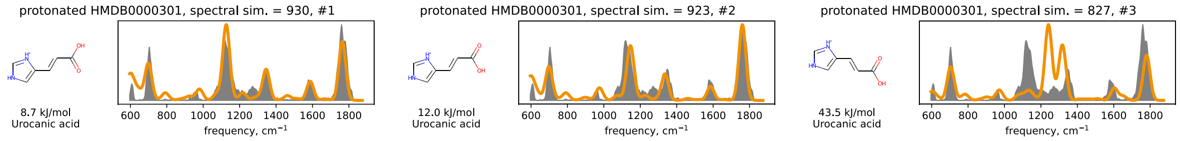
protonated HMDB0000301, spectral sim. = 930, #1
8.7 kJ/mol
Urocanic acid
protonated HMDB0000301, spectral sim. = 923, #2
12.0 kJ/mol
Urocanic acid
protonated HMDB0000301, spectral sim. = 827, #3
43.5 kJ/mol
Urocanic acid
frequency, cm⁻¹

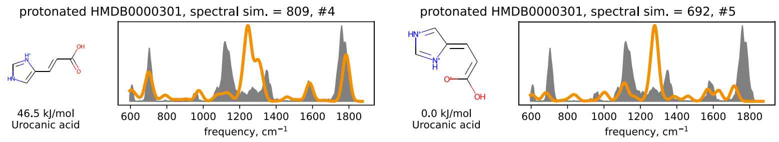
protonated HMDB0000301, spectral sim. = 809, #4
46.5 kJ/mol
Urocanic acid
protonated HMDB0000301, spectral sim. = 692, #5
0.0 kJ/mol
Urocanic acid
frequency, cm⁻¹

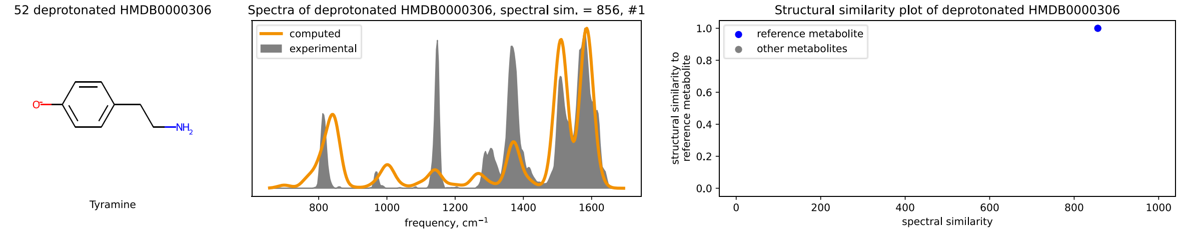
52 deprotonated HMDB0000306
Tyramine
Spectra of deprotonated HMDB0000306, spectral sim. = 856, #1
computed
experimental
frequency, cm⁻¹
Structural similarity plot of deprotonated HMDB0000306
reference metabolite
other metabolites
structural similarity to reference metabolite
spectral similarity

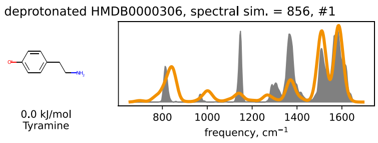
deprotonated HMDB0000306, spectral sim. = 856, #1
0.0 kJ/mol
Tyramine
frequency, cm⁻¹

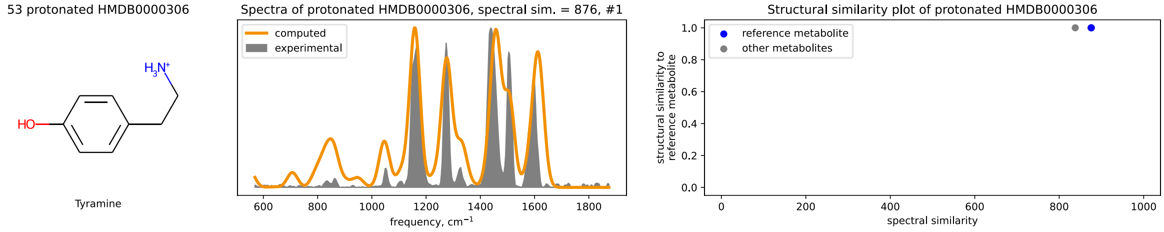
53 protonated HMDB0000306
Tyramine
Spectra of protonated HMDB0000306, spectral sim. = 876, #1
computed
experimental
frequency, cm⁻¹
Structural similarity plot of protonated HMDB0000306
reference metabolite
other metabolites
structural similarity to reference metabolite
spectral similarity

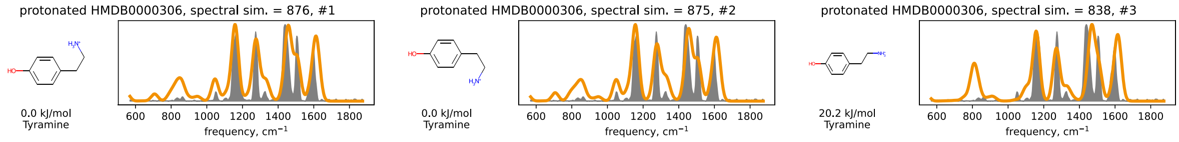
protonated HMDB0000306, spectral sim. = 876, #1
0.0 kJ/mol
Tyramine
protonated HMDB0000306, spectral sim. = 875, #2
0.0 kJ/mol
Tyramine
protonated HMDB0000306, spectral sim. = 838, #3
20.2 kJ/mol
Tyramine
frequency, cm⁻¹

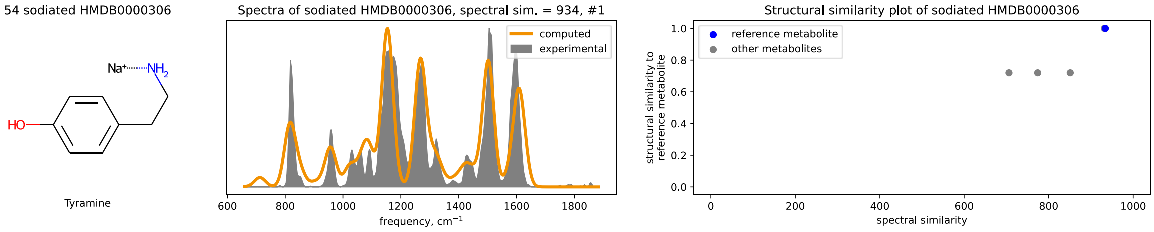
54 sodiated HMDB0000306
Tyramine
Spectra of sodiated HMDB0000306, spectral sim. = 934, #1
computed
experimental
frequency, cm⁻¹
Structural similarity plot of sodiated HMDB0000306
reference metabolite
other metabolites
structural similarity to reference metabolite
spectral similarity

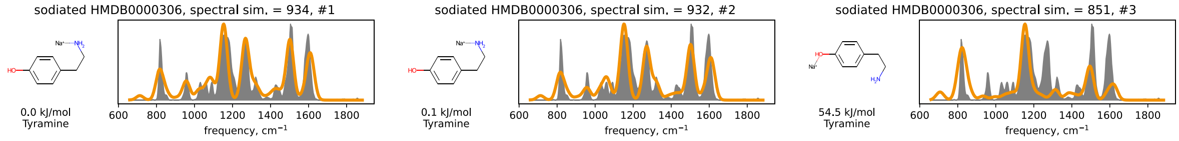
sodiated HMDB0000306, spectral sim. = 934, #1
0.0 kJ/mol
Tyramine
sodiated HMDB0000306, spectral sim. = 932, #2
0.1 kJ/mol
Tyramine
sodiated HMDB0000306, spectral sim. = 851, #3
54.5 kJ/mol
Tyramine
frequency, cm⁻¹

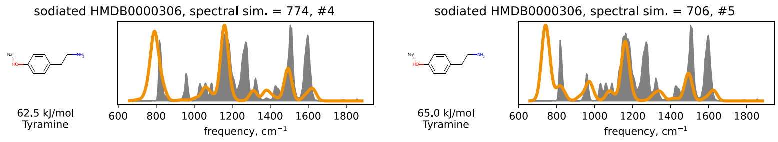
sodiated HMDB0000306, spectral sim. = 774, #4
62.5 kJ/mol
Tyramine
sodiated HMDB0000306, spectral sim. = 706, #5
65.0 kJ/mol
Tyramine
frequency, cm⁻¹

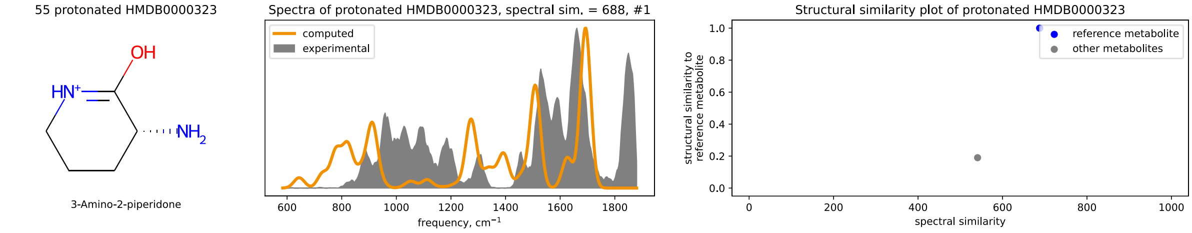
55 protonated HMDB0000323
3-Amino-2-piperidone
Spectra of protonated HMDB0000323, spectral sim. = 688, #1
computed
experimental
frequency, cm⁻¹
Structural similarity plot of protonated HMDB0000323
reference metabolite
other metabolites
structural similarity to reference metabolite
spectral similarity

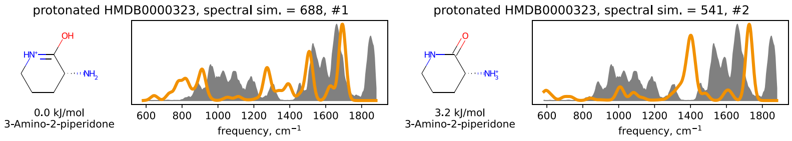
protonated HMDB0000323, spectral sim. = 688, #1
0.0 kJ/mol
3-Amino-2-piperidone
protonated HMDB0000323, spectral sim. = 541, #2
3.2 kJ/mol
3-Amino-2-piperidone
frequency, cm⁻¹

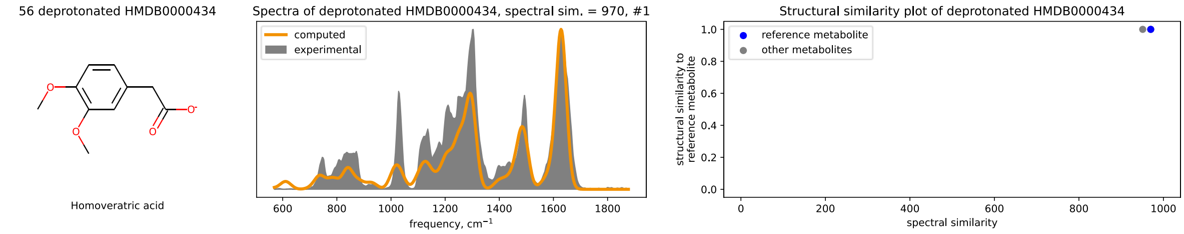
56 deprotonated HMDB0000434
Homoveratric acid
Spectra of deprotonated HMDB0000434, spectral sim. = 970, #1
computed
experimental
frequency, cm⁻¹
Structural similarity plot of deprotonated HMDB0000434
reference metabolite
other metabolites
structural similarity to reference metabolite
spectral similarity

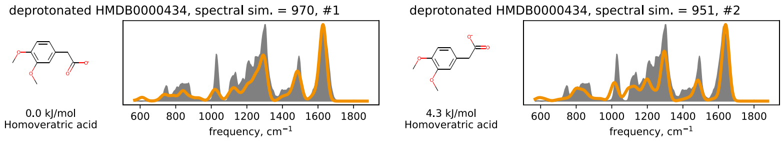
deprotonated HMDB0000434, spectral sim. = 970, #1
0.0 kJ/mol
Homoveratric acid
frequency, cm⁻¹
deprotonated HMDB0000434, spectral sim. = 951, #2
4.3 kJ/mol
Homoveratric acid
frequency, cm⁻¹

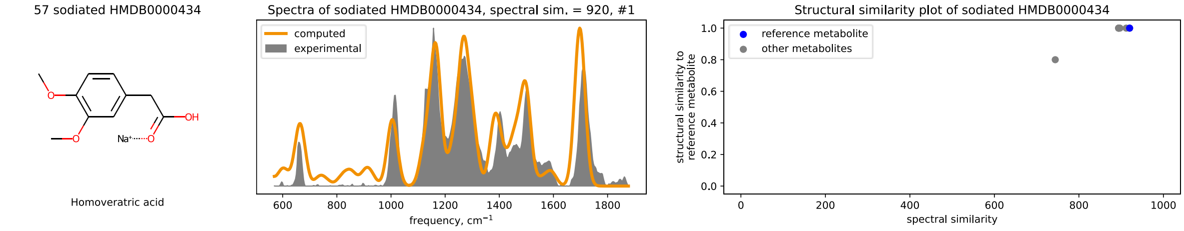
57 sodiated HMDB0000434
Homoveratric acid
Spectra of sodiated HMDB0000434, spectral sim. = 920, #1
computed
experimental
frequency, cm⁻¹
Structural similarity plot of sodiated HMDB0000434
reference metabolite
other metabolites
structural similarity to reference metabolite
spectral similarity

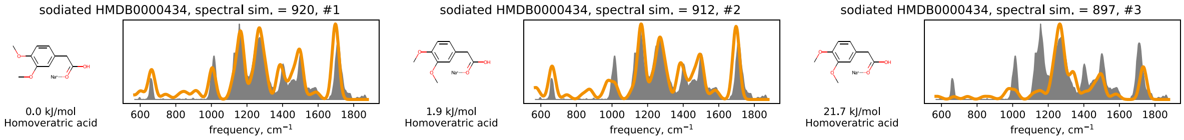
sodiated HMDB0000434, spectral sim. = 920, #1
0.0 kJ/mol
Homoveratric acid
frequency, cm⁻¹
sodiated HMDB0000434, spectral sim. = 912, #2
1.9 kJ/mol
Homoveratric acid
frequency, cm⁻¹
sodiated HMDB0000434, spectral sim. = 897, #3
21.7 kJ/mol
Homoveratric acid
frequency, cm⁻¹

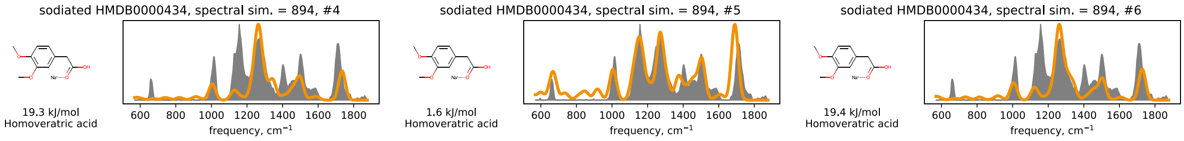
sodiated HMDB0000434, spectral sim. = 894, #4
19.3 kJ/mol
Homoveratric acid
frequency, cm⁻¹
sodiated HMDB0000434, spectral sim. = 894, #5
1.6 kJ/mol
Homoveratric acid
frequency, cm⁻¹
sodiated HMDB0000434, spectral sim. = 894, #6
19.4 kJ/mol
Homoveratric acid
frequency, cm⁻¹

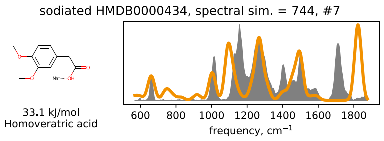
sodiated HMDB0000434, spectral sim. = 744, #7
33.1 kJ/mol
Homoveratric acid
frequency, cm⁻¹

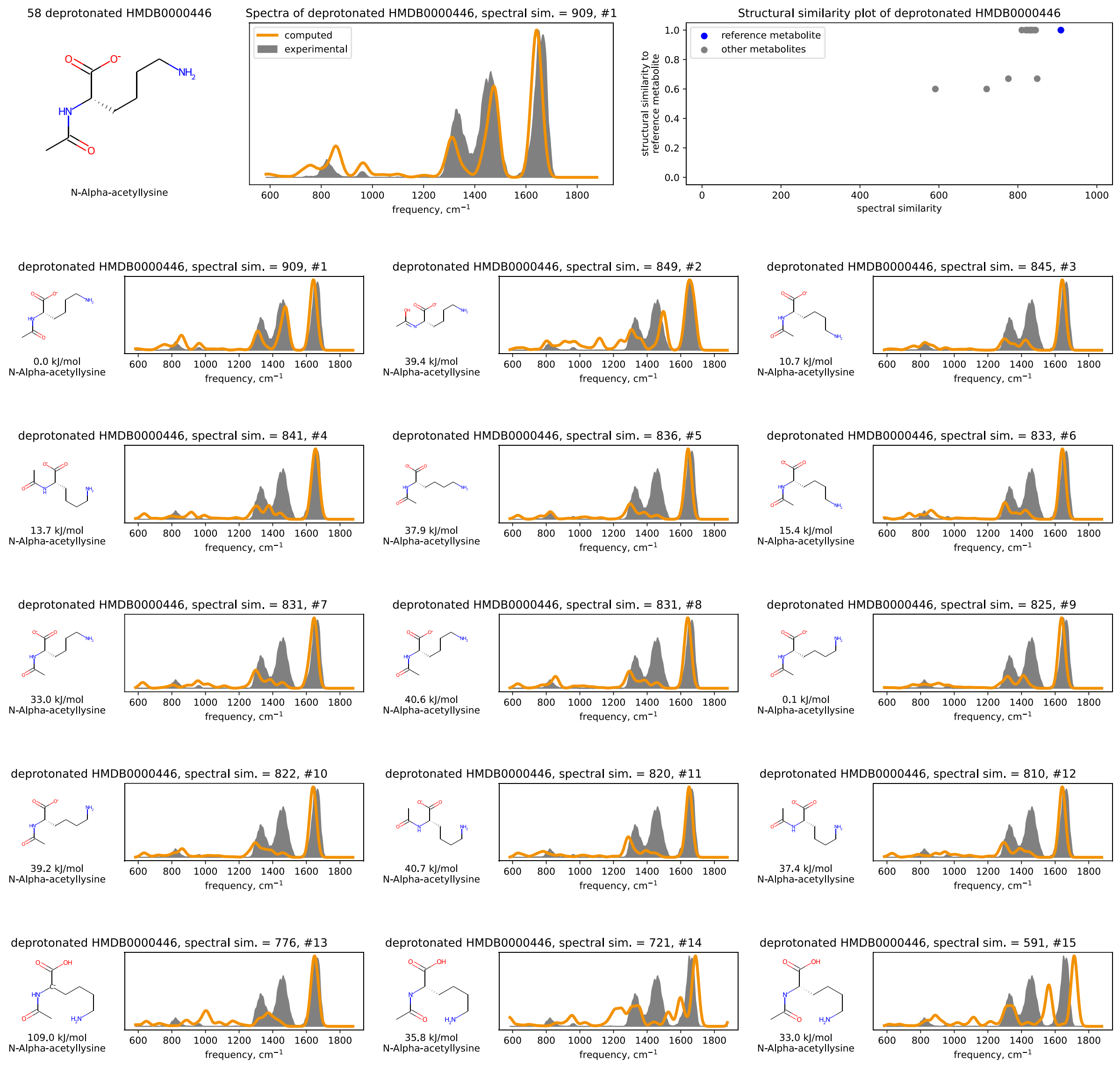
58 deprotonated HMDB0000446
N-Alpha-acetyllysine
Spectra of deprotonated HMDB0000446, spectral sim. = 909, #1
computed
experimental
frequency, cm−1
Structural similarity plot of deprotonated HMDB0000446
reference metabolite
other metabolites
structural similarity to reference metabolite
spectral similarity
deprotonated HMDB0000446, spectral sim. = 909, #1
0.0 kJ/mol
N-Alpha-acetyllysine
deprotonated HMDB0000446, spectral sim. = 849, #2
39.4 kJ/mol
N-Alpha-acetyllysine
deprotonated HMDB0000446, spectral sim. = 845, #3
10.7 kJ/mol
N-Alpha-acetyllysine
deprotonated HMDB0000446, spectral sim. = 841, #4
13.7 kJ/mol
N-Alpha-acetyllysine
deprotonated HMDB0000446, spectral sim. = 836, #5
37.9 kJ/mol
N-Alpha-acetyllysine
deprotonated HMDB0000446, spectral sim. = 833, #6
15.4 kJ/mol
N-Alpha-acetyllysine
deprotonated HMDB0000446, spectral sim. = 831, #7
33.0 kJ/mol
N-Alpha-acetyllysine
deprotonated HMDB0000446, spectral sim. = 831, #8
40.6 kJ/mol
N-Alpha-acetyllysine
deprotonated HMDB0000446, spectral sim. = 825, #9
0.1 kJ/mol
N-Alpha-acetyllysine
deprotonated HMDB0000446, spectral sim. = 822, #10
39.2 kJ/mol
N-Alpha-acetyllysine
deprotonated HMDB0000446, spectral sim. = 820, #11
40.7 kJ/mol
N-Alpha-acetyllysine
deprotonated HMDB0000446, spectral sim. = 810, #12
37.4 kJ/mol
N-Alpha-acetyllysine
deprotonated HMDB0000446, spectral sim. = 776, #13
109.0 kJ/mol
N-Alpha-acetyllysine
deprotonated HMDB0000446, spectral sim. = 721, #14
35.8 kJ/mol
N-Alpha-acetyllysine
deprotonated HMDB0000446, spectral sim. = 591, #15
33.0 kJ/mol
N-Alpha-acetyllysine
frequency, cm−1

59 protonated HMDB0000446

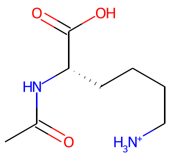


N-Alpha-acetyllysine

Spectra of protonated HMDB0000446, spectral sim. = 928, #1

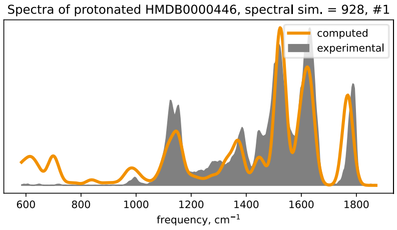


Structural similarity plot of protonated HMDB0000446

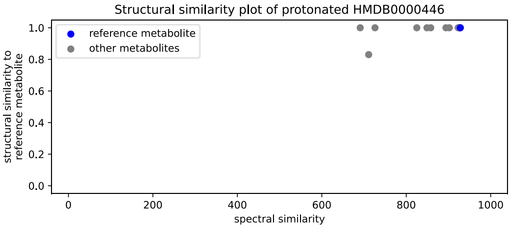


protonated HMDB0000446, spectral sim. = 928, #1

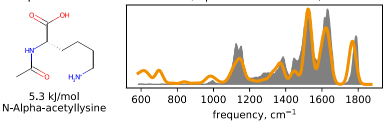

5.3 kJ/mol
N-Alpha-acetyllysine

protonated HMDB0000446, spectral sim. = 925, #2

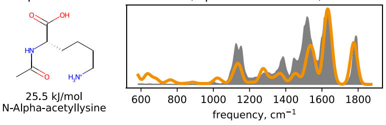

25.5 kJ/mol
N-Alpha-acetyllysine

protonated HMDB0000446, spectral sim. = 923, #3

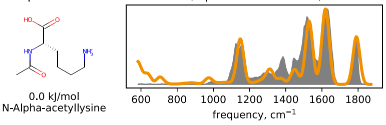

0.0 kJ/mol
N-Alpha-acetyllysine

protonated HMDB0000446, spectral sim. = 902, #4

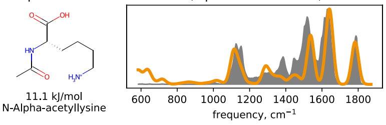

11.1 kJ/mol
N-Alpha-acetyllysine

protonated HMDB0000446, spectral sim. = 894, #5

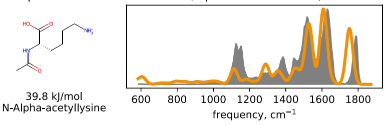

39.8 kJ/mol
N-Alpha-acetyllysine

protonated HMDB0000446, spectral sim. = 858, #6

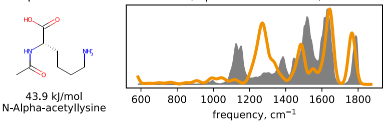

43.9 kJ/mol
N-Alpha-acetyllysine

protonated HMDB0000446, spectral sim. = 849, #7

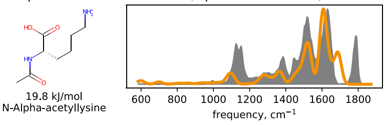

19.8 kJ/mol
N-Alpha-acetyllysine

protonated HMDB0000446, spectral sim. = 825, #8

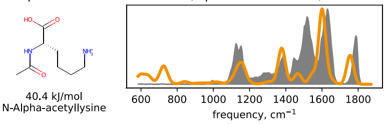

40.4 kJ/mol
N-Alpha-acetyllysine

protonated HMDB0000446, spectral sim. = 726, #9

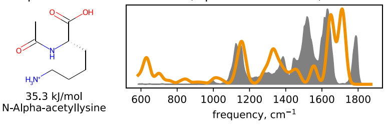

35.3 kJ/mol
N-Alpha-acetyllysine

protonated HMDB0000446, spectral sim. = 711, #10

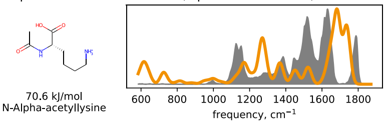

70.6 kJ/mol
N-Alpha-acetyllysine

protonated HMDB0000446, spectral sim. = 691, #11

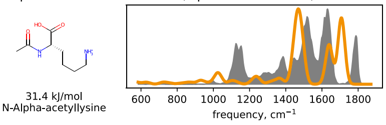

31.4 kJ/mol
N-Alpha-acetyllysine

60 sodiated HMDB0000446

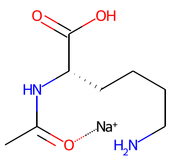

N-Alpha-acetyllysine

Spectra of sodiated HMDB0000446, spectral sim. = 877, #1

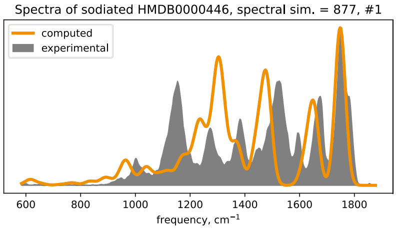


Structural similarity plot of sodiated HMDB0000446

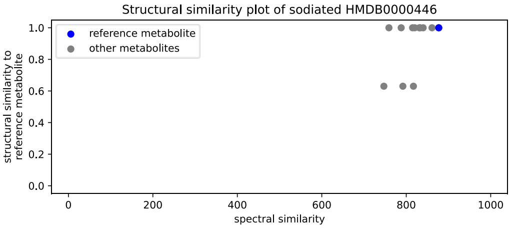


sodiated HMDB0000446, spectral sim. = 877, #1

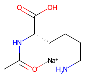

45.3 kJ/mol
N-Alpha-acetyllysine

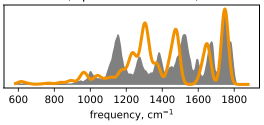

sodiated HMDB0000446, spectral sim. = 861, #2

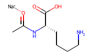

12.9 kJ/mol
N-Alpha-acetyllysine

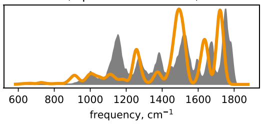

sodiated HMDB0000446, spectral sim. = 840, #3

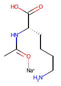

0.0 kJ/mol
N-Alpha-acetyllysine

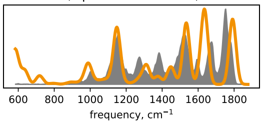

sodiated HMDB0000446, spectral sim. = 833, #4

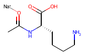

31.4 kJ/mol
N-Alpha-acetyllysine

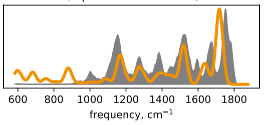

sodiated HMDB0000446, spectral sim. = 831, #5

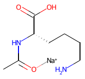

16.3 kJ/mol
N-Alpha-acetyllysine

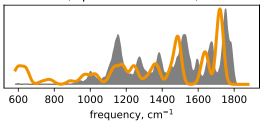

sodiated HMDB0000446, spectral sim. = 820, #6

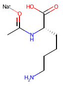

20.4 kJ/mol
N-Alpha-acetyllysine

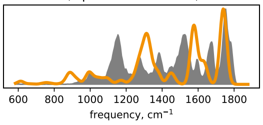

sodiated HMDB0000446, spectral sim. = 817, #7

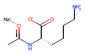

3.8 kJ/mol
N-Alpha-acetyllysine

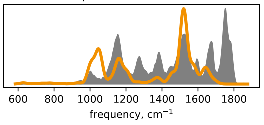

sodiated HMDB0000446, spectral sim. = 815, #8

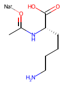

17.6 kJ/mol
N-Alpha-acetyllysine

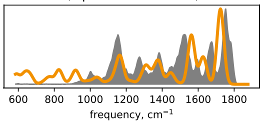

sodiated HMDB0000446, spectral sim. = 792, #9

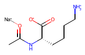

17.3 kJ/mol
N-Alpha-acetyllysine

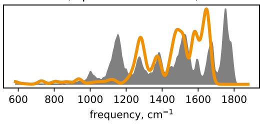

sodiated HMDB0000446, spectral sim. = 788, #10

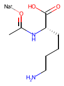

26.6 kJ/mol
N-Alpha-acetyllysine

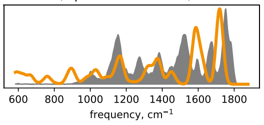

sodiated HMDB0000446, spectral sim. = 759, #11

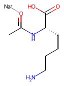

4.7 kJ/mol
N-Alpha-acetyllysine

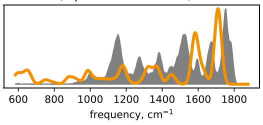

sodiated HMDB0000446, spectral sim. = 747, #12

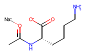

23.2 kJ/mol
N-Alpha-acetyllysine

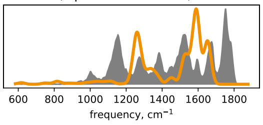

61 deprotonated HMDB0000448

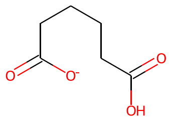

Adipic acid

Spectra of deprotonated HMDB0000448, spectral sim. = 847, #1

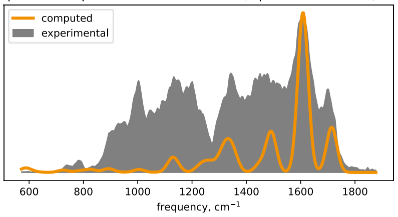


Structural similarity plot of deprotonated HMDB0000448

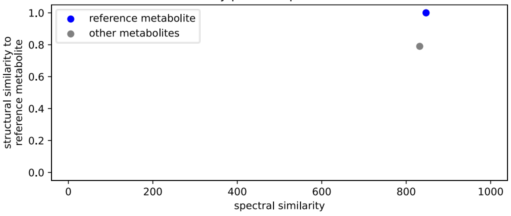


deprotonated HMDB0000448, spectral sim. = 847, #1

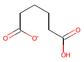

0.0 kJ/mol
Adipic acid

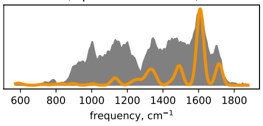

deprotonated HMDB0000448, spectral sim. = 832, #2

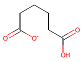

7.3 kJ/mol
Adipic acid

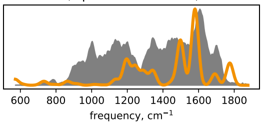

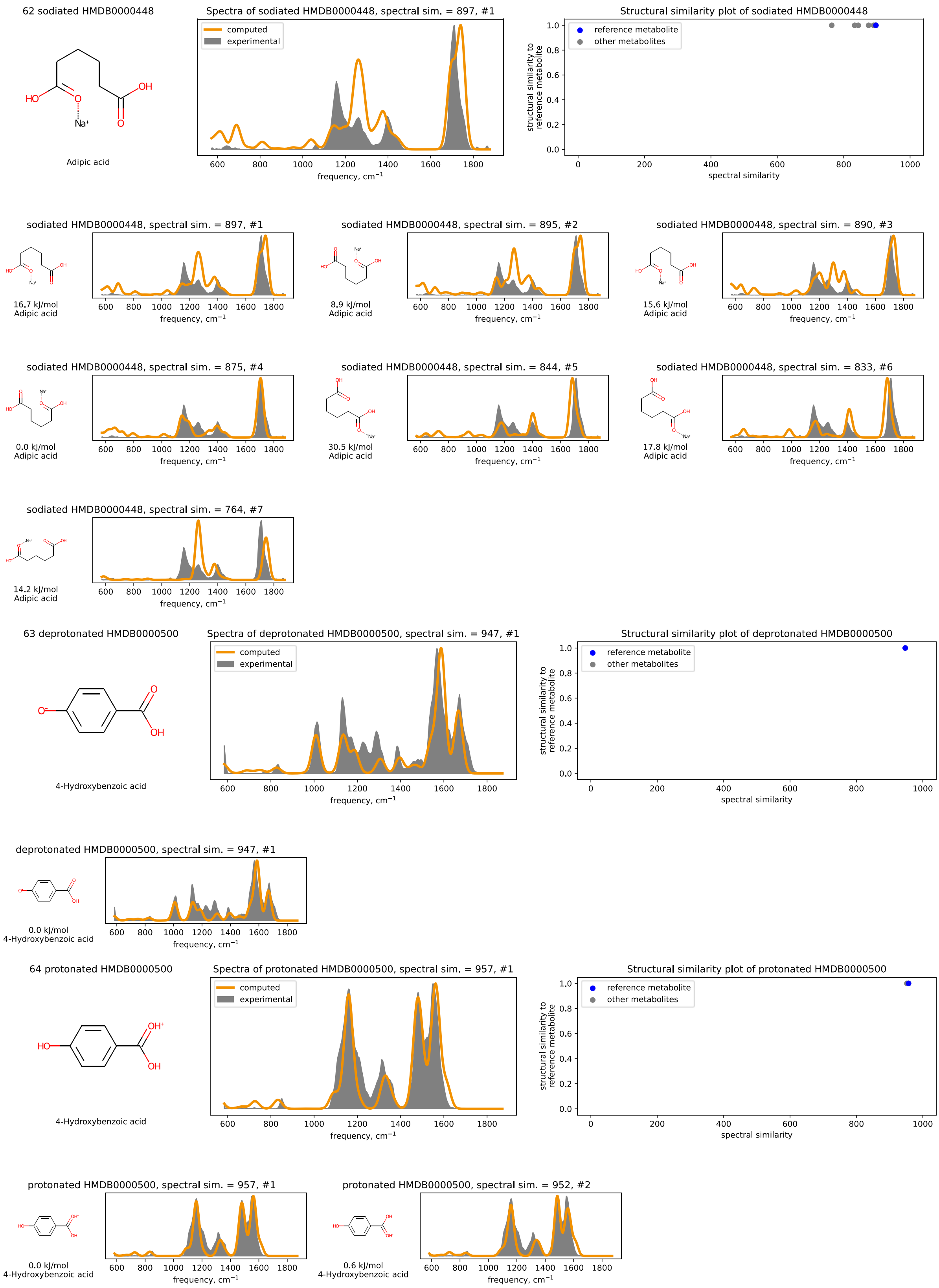
62 sodiated HMDB0000448
Adipic acid
Spectra of sodiated HMDB0000448, spectral sim. = 897, #1
computed
experimental
frequency, cm⁻¹
Structural similarity plot of sodiated HMDB0000448
reference metabolite
other metabolites
structural similarity to reference metabolite
spectral similarity
sodiated HMDB0000448, spectral sim. = 897, #1
16.7 kJ/mol
Adipic acid
sodiated HMDB0000448, spectral sim. = 895, #2
8.9 kJ/mol
Adipic acid
sodiated HMDB0000448, spectral sim. = 890, #3
15.6 kJ/mol
Adipic acid
sodiated HMDB0000448, spectral sim. = 875, #4
0.0 kJ/mol
Adipic acid
sodiated HMDB0000448, spectral sim. = 844, #5
30.5 kJ/mol
Adipic acid
sodiated HMDB0000448, spectral sim. = 833, #6
17.8 kJ/mol
Adipic acid
sodiated HMDB0000448, spectral sim. = 764, #7
14.2 kJ/mol
Adipic acid
63 deprotonated HMDB0000500
4-Hydroxybenzoic acid
Spectra of deprotonated HMDB0000500, spectral sim. = 947, #1
Structural similarity plot of deprotonated HMDB0000500
deprotonated HMDB0000500, spectral sim. = 947, #1
0.0 kJ/mol
4-Hydroxybenzoic acid
64 protonated HMDB0000500
4-Hydroxybenzoic acid
Spectra of protonated HMDB0000500, spectral sim. = 957, #1
Structural similarity plot of protonated HMDB0000500
protonated HMDB0000500, spectral sim. = 957, #1
0.0 kJ/mol
4-Hydroxybenzoic acid
protonated HMDB0000500, spectral sim. = 952, #2
0.6 kJ/mol
4-Hydroxybenzoic acid

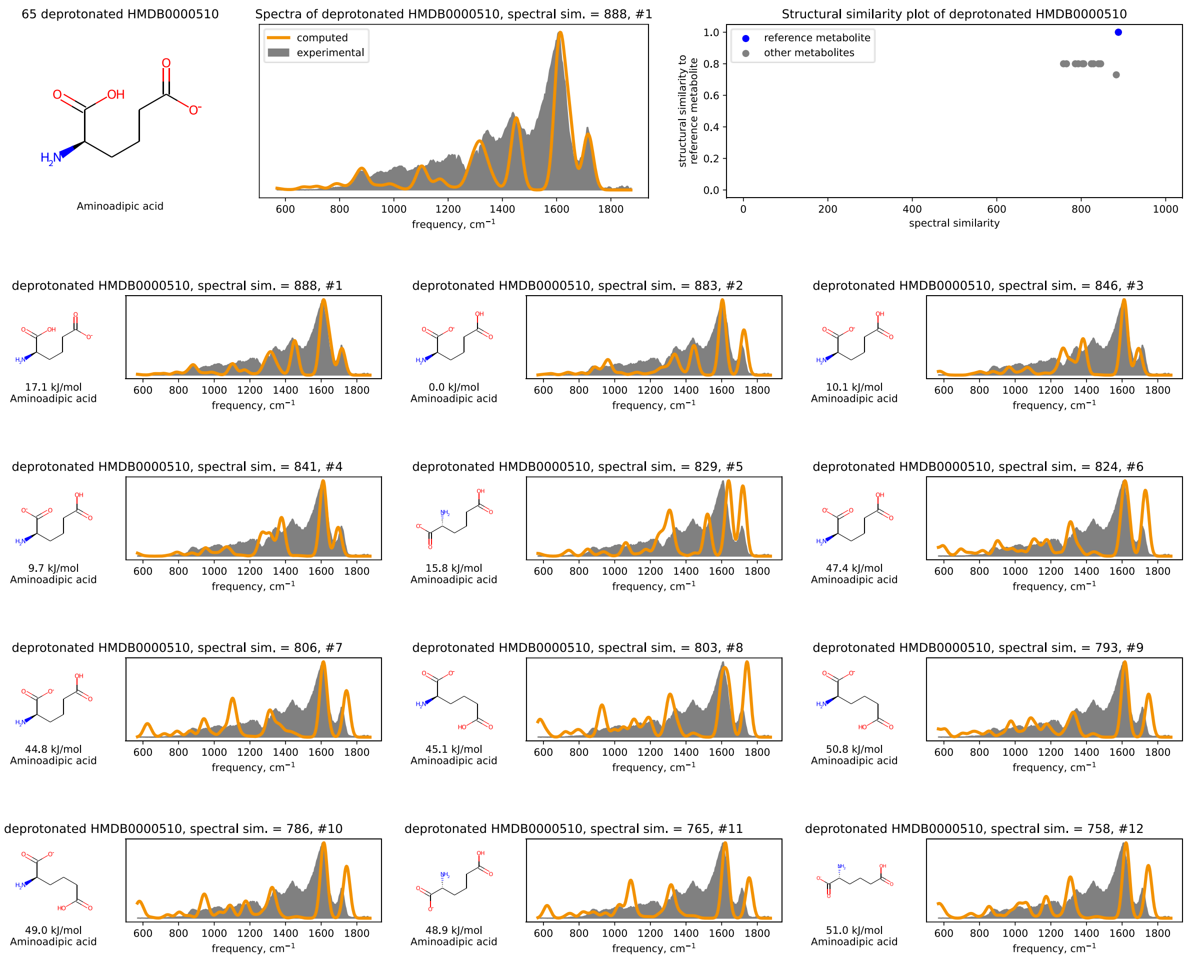
65 deprotonated HMDB0000510
Aminoadipic acid
Spectra of deprotonated HMDB0000510, spectral sim. = 888, #1
computed
experimental
frequency, cm⁻¹
Structural similarity plot of deprotonated HMDB0000510
reference metabolite
other metabolites
structural similarity to reference metabolite
spectral similarity
deprotonated HMDB0000510, spectral sim. = 888, #1
17.1 kJ/mol
Aminoadipic acid
deprotonated HMDB0000510, spectral sim. = 883, #2
0.0 kJ/mol
Aminoadipic acid
deprotonated HMDB0000510, spectral sim. = 846, #3
10.1 kJ/mol
Aminoadipic acid
deprotonated HMDB0000510, spectral sim. = 841, #4
9.7 kJ/mol
Aminoadipic acid
deprotonated HMDB0000510, spectral sim. = 829, #5
15.8 kJ/mol
Aminoadipic acid
deprotonated HMDB0000510, spectral sim. = 824, #6
47.4 kJ/mol
Aminoadipic acid
deprotonated HMDB0000510, spectral sim. = 806, #7
44.8 kJ/mol
Aminoadipic acid
deprotonated HMDB0000510, spectral sim. = 803, #8
45.1 kJ/mol
Aminoadipic acid
deprotonated HMDB0000510, spectral sim. = 793, #9
50.8 kJ/mol
Aminoadipic acid
deprotonated HMDB0000510, spectral sim. = 786, #10
49.0 kJ/mol
Aminoadipic acid
deprotonated HMDB0000510, spectral sim. = 765, #11
48.9 kJ/mol
Aminoadipic acid
deprotonated HMDB0000510, spectral sim. = 758, #12
51.0 kJ/mol
Aminoadipic acid

66 protonated HMDB0000510

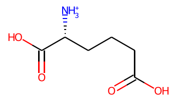

Aminoadipic acid

Spectra of protonated HMDB0000510, spectral sim. = 876, #1

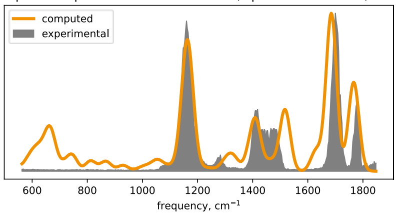


Structural similarity plot of protonated HMDB0000510

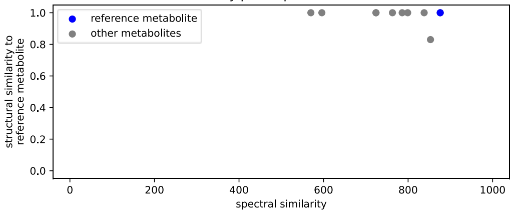


protonated HMDB0000510, spectral sim. = 876, #1

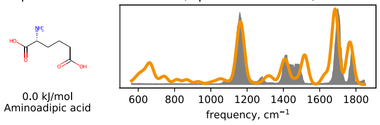

0.0 kJ/mol
Aminoadipic acid

protonated HMDB0000510, spectral sim. = 853, #2

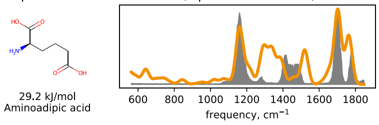

29.2 kJ/mol
Aminoadipic acid

protonated HMDB0000510, spectral sim. = 838, #3

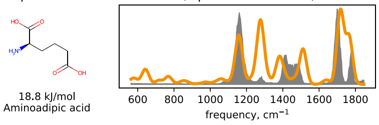

18.8 kJ/mol
Aminoadipic acid

protonated HMDB0000510, spectral sim. = 799, #4

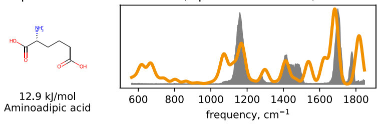

12.9 kJ/mol
Aminoadipic acid

protonated HMDB0000510, spectral sim. = 786, #5

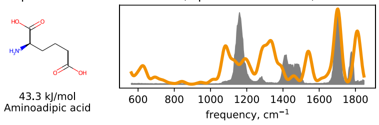

43.3 kJ/mol
Aminoadipic acid

protonated HMDB0000510, spectral sim. = 763, #6

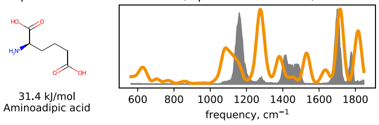

31.4 kJ/mol
Aminoadipic acid

protonated HMDB0000510, spectral sim. = 724, #7

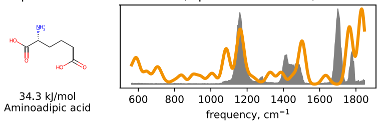

34.3 kJ/mol
Aminoadipic acid

protonated HMDB0000510, spectral sim. = 724, #8

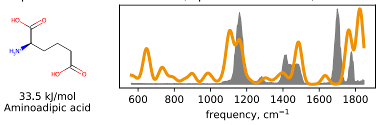

33.5 kJ/mol
Aminoadipic acid

protonated HMDB0000510, spectral sim. = 596, #9

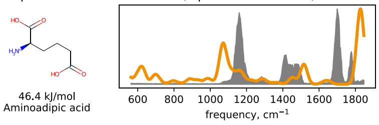

46.4 kJ/mol
Aminoadipic acid

protonated HMDB0000510, spectral sim. = 570, #10

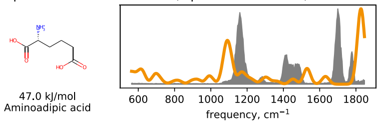

47.0 kJ/mol
Aminoadipic acid

67 sodiated HMDB0000510

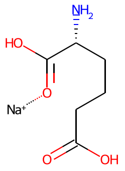

Aminoadipic acid

Spectra of sodiated HMDB0000510, spectral sim. = 892, #1

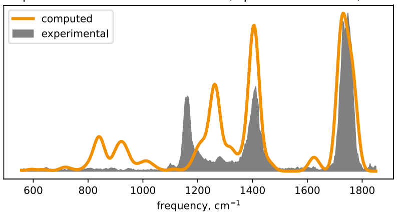


Structural similarity plot of sodiated HMDB0000510

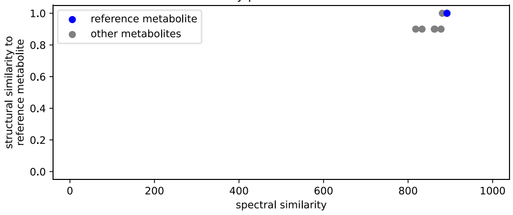


sodiated HMDB0000510, spectral sim. = 892, #1

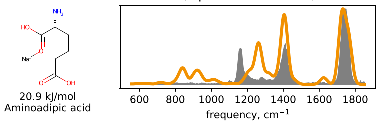

20.9 kJ/mol
Aminoadipic acid

sodiated HMDB0000510, spectral sim. = 881, #2

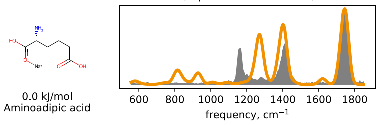

0.0 kJ/mol
Aminoadipic acid

sodiated HMDB0000510, spectral sim. = 878, #3

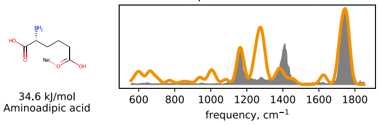

34.6 kJ/mol
Aminoadipic acid

sodiated HMDB0000510, spectral sim. = 863, #4

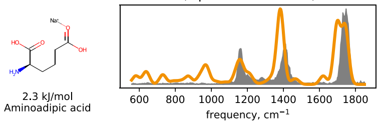

2.3 kJ/mol
Aminoadipic acid

sodiated HMDB0000510, spectral sim. = 862, #5

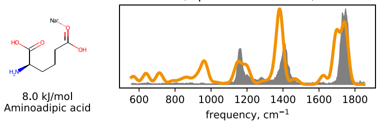

8.0 kJ/mol
Aminoadipic acid

sodiated HMDB0000510, spectral sim. = 833, #6

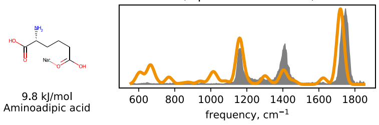

9.8 kJ/mol
Aminoadipic acid

sodiated HMDB0000510, spectral sim. = 818, #7

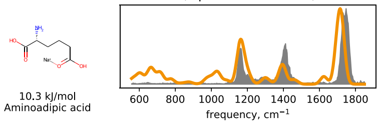

10.3 kJ/mol
Aminoadipic acid

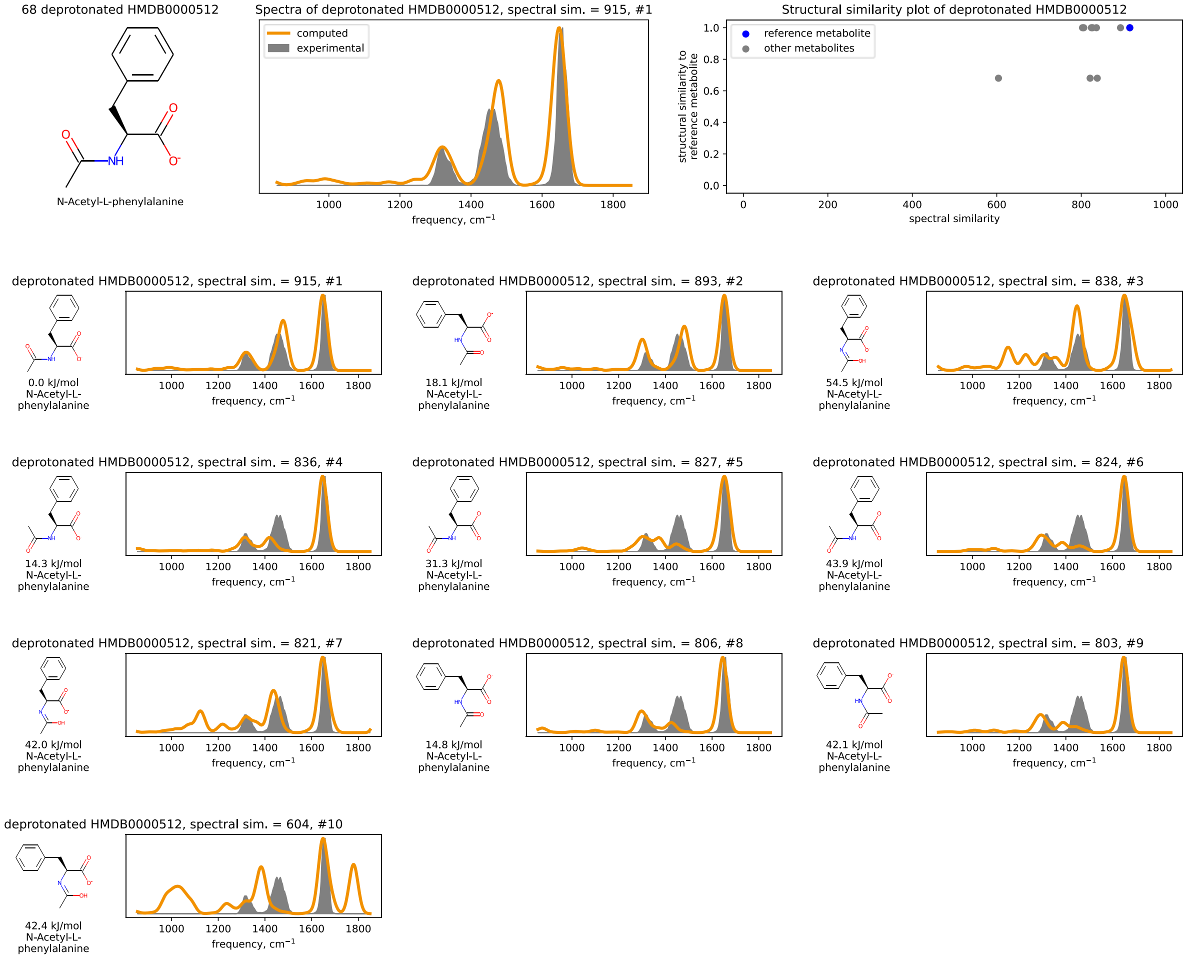
68 deprotonated HMDB0000512
N-Acetyl-L-phenylalanine
Spectra of deprotonated HMDB0000512, spectral sim. = 915, #1
computed
experimental
frequency, cm$^{-1}$
Structural similarity plot of deprotonated HMDB0000512
reference metabolite
other metabolites
structural similarity to reference metabolite
spectral similarity
deprotonated HMDB0000512, spectral sim. = 915, #1
0.0 kJ/mol
N-Acetyl-L-phenylalanine
deprotonated HMDB0000512, spectral sim. = 893, #2
18.1 kJ/mol
N-Acetyl-L-phenylalanine
deprotonated HMDB0000512, spectral sim. = 838, #3
54.5 kJ/mol
N-Acetyl-L-phenylalanine
deprotonated HMDB0000512, spectral sim. = 836, #4
14.3 kJ/mol
N-Acetyl-L-phenylalanine
deprotonated HMDB0000512, spectral sim. = 827, #5
31.3 kJ/mol
N-Acetyl-L-phenylalanine
deprotonated HMDB0000512, spectral sim. = 824, #6
43.9 kJ/mol
N-Acetyl-L-phenylalanine
deprotonated HMDB0000512, spectral sim. = 821, #7
42.0 kJ/mol
N-Acetyl-L-phenylalanine
deprotonated HMDB0000512, spectral sim. = 806, #8
14.8 kJ/mol
N-Acetyl-L-phenylalanine
deprotonated HMDB0000512, spectral sim. = 803, #9
42.1 kJ/mol
N-Acetyl-L-phenylalanine
deprotonated HMDB0000512, spectral sim. = 604, #10
42.4 kJ/mol
N-Acetyl-L-phenylalanine
frequency, cm$^{-1}$

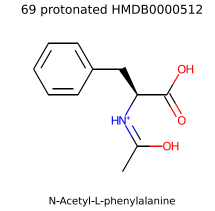
69 protonated HMDB0000512
OH
O
HN+
OH
N-Acetyl-L-phenylalanine

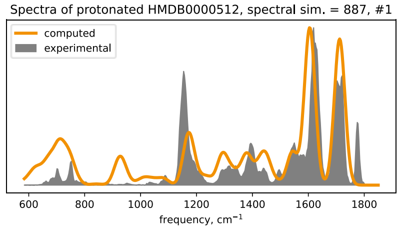
Spectra of protonated HMDB0000512, spectral sim. = 887, #1
computed
experimental
frequency, cm$^{-1}$

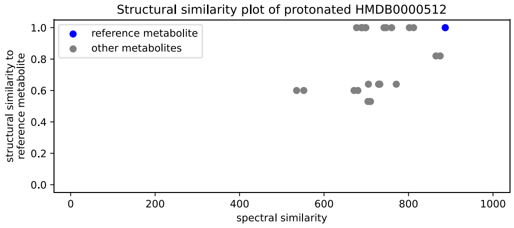
Structural similarity plot of protonated HMDB0000512
reference metabolite
other metabolites
structural similarity to reference metabolite
spectral similarity

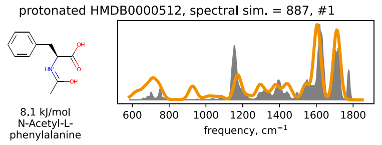
protonated HMDB0000512, spectral sim. = 887, #1
8.1 kJ/mol
N-Acetyl-L-
phenylalanine
frequency, cm$^{-1}$

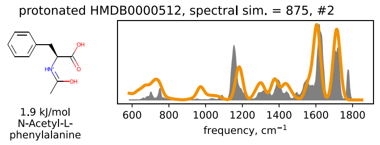
protonated HMDB0000512, spectral sim. = 875, #2
1.9 kJ/mol
N-Acetyl-L-
phenylalanine
frequency, cm$^{-1}$

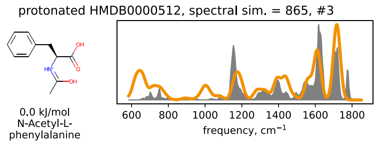
protonated HMDB0000512, spectral sim. = 865, #3
0.0 kJ/mol
N-Acetyl-L-
phenylalanine
frequency, cm$^{-1}$

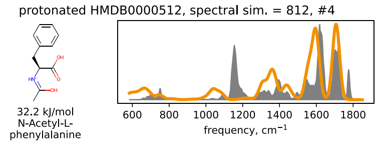
protonated HMDB0000512, spectral sim. = 812, #4
32.2 kJ/mol
N-Acetyl-L-
phenylalanine
frequency, cm$^{-1}$

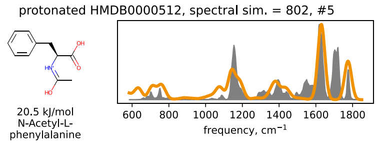
protonated HMDB0000512, spectral sim. = 802, #5
20.5 kJ/mol
N-Acetyl-L-
phenylalanine
frequency, cm$^{-1}$

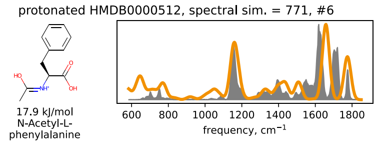
protonated HMDB0000512, spectral sim. = 771, #6
17.9 kJ/mol
N-Acetyl-L-
phenylalanine
frequency, cm$^{-1}$

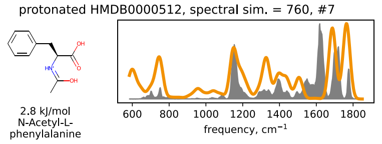
protonated HMDB0000512, spectral sim. = 760, #7
2.8 kJ/mol
N-Acetyl-L-
phenylalanine
frequency, cm$^{-1}$

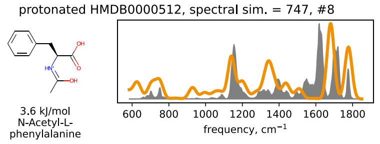
protonated HMDB0000512, spectral sim. = 747, #8
3.6 kJ/mol
N-Acetyl-L-
phenylalanine
frequency, cm$^{-1}$

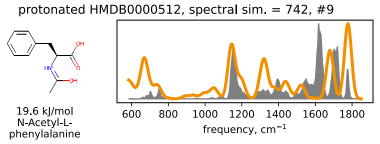
protonated HMDB0000512, spectral sim. = 742, #9
19.6 kJ/mol
N-Acetyl-L-
phenylalanine
frequency, cm$^{-1}$

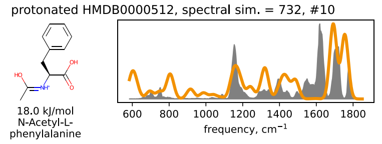
protonated HMDB0000512, spectral sim. = 732, #10
18.0 kJ/mol
N-Acetyl-L-
phenylalanine
frequency, cm$^{-1}$

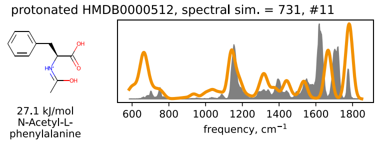
protonated HMDB0000512, spectral sim. = 731, #11
27.1 kJ/mol
N-Acetyl-L-
phenylalanine
frequency, cm$^{-1}$

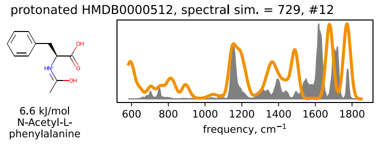
protonated HMDB0000512, spectral sim. = 729, #12
6.6 kJ/mol
N-Acetyl-L-
phenylalanine
frequency, cm$^{-1}$

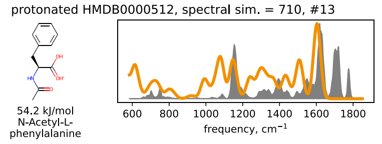
protonated HMDB0000512, spectral sim. = 710, #13
54.2 kJ/mol
N-Acetyl-L-
phenylalanine
frequency, cm$^{-1}$

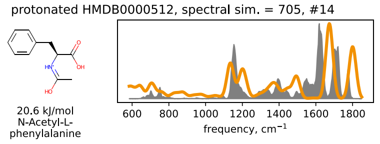
protonated HMDB0000512, spectral sim. = 705, #14
20.6 kJ/mol
N-Acetyl-L-
phenylalanine
frequency, cm$^{-1}$

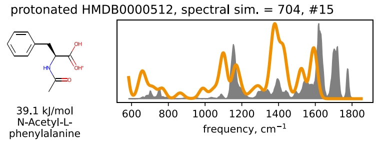
protonated HMDB0000512, spectral sim. = 704, #15
39.1 kJ/mol
N-Acetyl-L-
phenylalanine
frequency, cm$^{-1}$

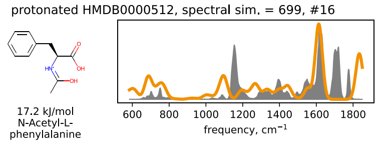
protonated HMDB0000512, spectral sim. = 699, #16
17.2 kJ/mol
N-Acetyl-L-
phenylalanine
frequency, cm$^{-1}$

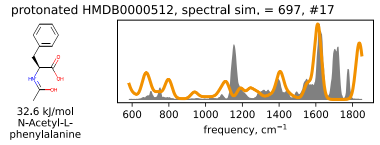
protonated HMDB0000512, spectral sim. = 697, #17
32.6 kJ/mol
N-Acetyl-L-
phenylalanine
frequency, cm$^{-1}$

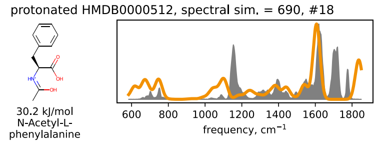
protonated HMDB0000512, spectral sim. = 690, #18
30.2 kJ/mol
N-Acetyl-L-
phenylalanine
frequency, cm$^{-1}$

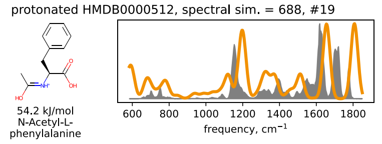
protonated HMDB0000512, spectral sim. = 688, #19
54.2 kJ/mol
N-Acetyl-L-
phenylalanine
frequency, cm$^{-1}$

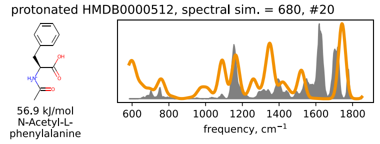
protonated HMDB0000512, spectral sim. = 680, #20
56.9 kJ/mol
N-Acetyl-L-
phenylalanine
frequency, cm$^{-1}$

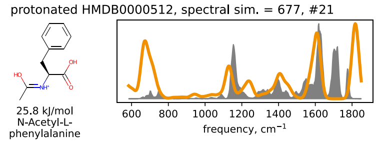
protonated HMDB0000512, spectral sim. = 677, #21
25.8 kJ/mol
N-Acetyl-L-
phenylalanine
frequency, cm$^{-1}$

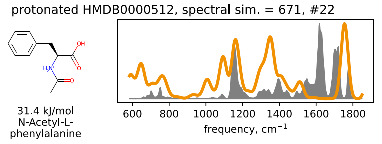
protonated HMDB0000512, spectral sim. = 671, #22
31.4 kJ/mol
N-Acetyl-L-
phenylalanine
frequency, cm$^{-1}$

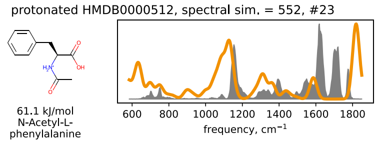
protonated HMDB0000512, spectral sim. = 552, #23
61.1 kJ/mol
N-Acetyl-L-
phenylalanine
frequency, cm$^{-1}$

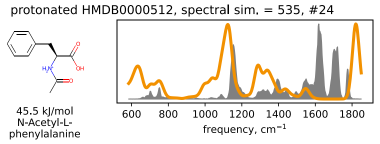
protonated HMDB0000512, spectral sim. = 535, #24
45.5 kJ/mol
N-Acetyl-L-
phenylalanine
frequency, cm$^{-1}$

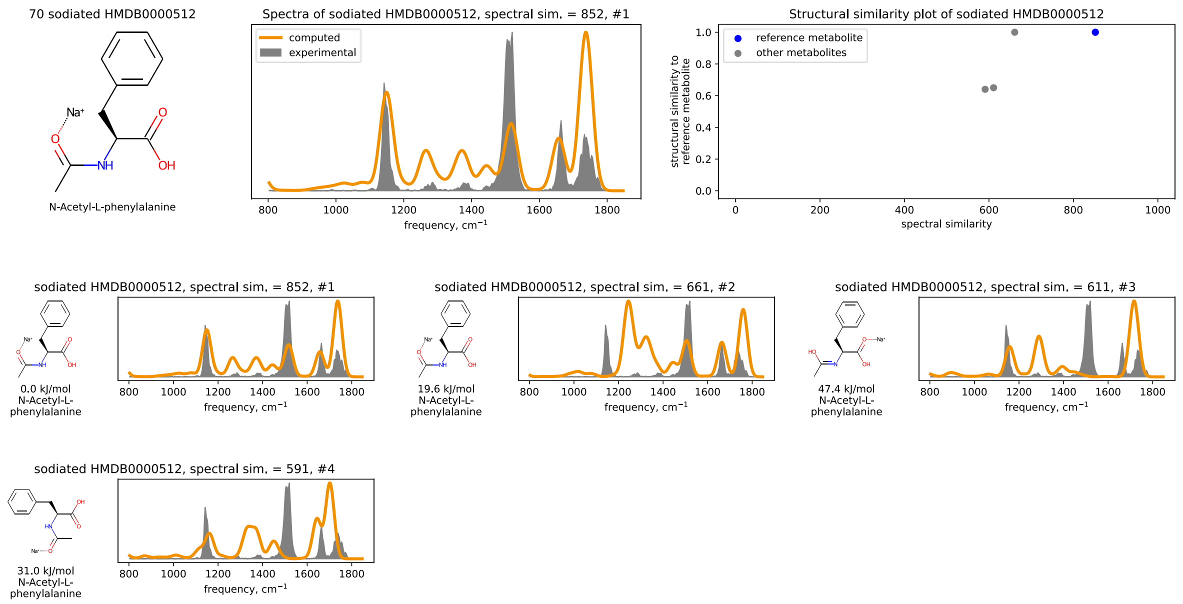
70 sodiated HMDB0000512
Na+
O
NH
O
OH
N-Acetyl-L-phenylalanine
Spectra of sodiated HMDB0000512, spectral sim. = 852, #1
computed
experimental
800 1000 1200 1400 1600 1800
frequency, cm−1
Structural similarity plot of sodiated HMDB0000512
reference metabolite
other metabolites
structural similarity to reference metabolite
0.0 0.2 0.4 0.6 0.8 1.0
0 200 400 600 800 1000
spectral similarity
sodiated HMDB0000512, spectral sim. = 852, #1
0.0 kJ/mol
N-Acetyl-L-phenylalanine
sodiated HMDB0000512, spectral sim. = 661, #2
19.6 kJ/mol
N-Acetyl-L-phenylalanine
sodiated HMDB0000512, spectral sim. = 611, #3
47.4 kJ/mol
N-Acetyl-L-phenylalanine
sodiated HMDB0000512, spectral sim. = 591, #4
31.0 kJ/mol
N-Acetyl-L-phenylalanine
frequency, cm−1

71 protonated HMDB0000517

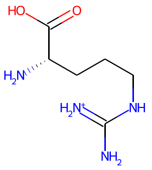


L-Arginine

Spectra of protonated HMDB0000517, spectral sim. = 935, #1

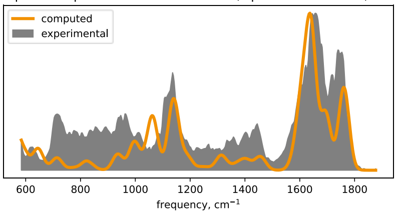


Structural similarity plot of protonated HMDB0000517

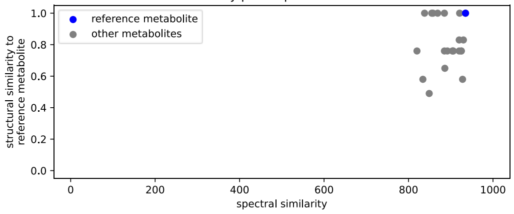


protonated HMDB0000517, spectral sim. = 935, #1

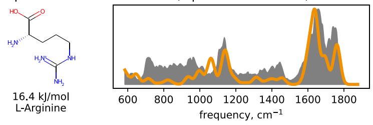


protonated HMDB0000517, spectral sim. = 930, #2

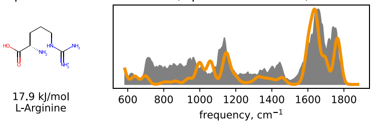


protonated HMDB0000517, spectral sim. = 928, #3

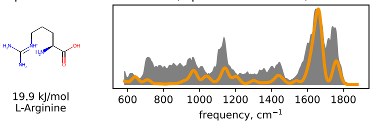


protonated HMDB0000517, spectral sim. = 925, #4

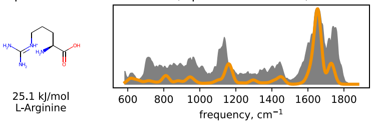


protonated HMDB0000517, spectral sim. = 925, #5

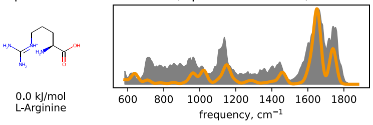


protonated HMDB0000517, spectral sim. = 921, #6

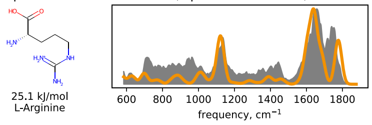


protonated HMDB0000517, spectral sim. = 920, #7

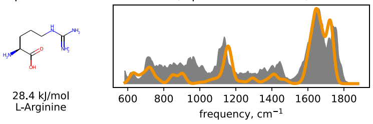


protonated HMDB0000517, spectral sim. = 920, #8

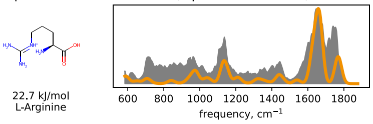


protonated HMDB0000517, spectral sim. = 906, #9

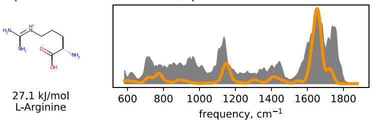


protonated HMDB0000517, spectral sim. = 905, #10

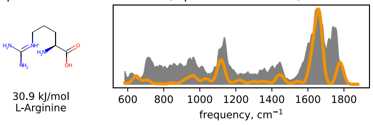


protonated HMDB0000517, spectral sim. = 904, #11

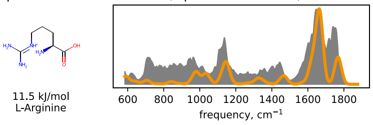


protonated HMDB0000517, spectral sim. = 892, #12

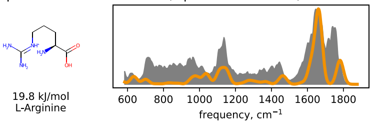


protonated HMDB0000517, spectral sim. = 886, #13

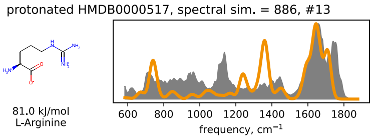


protonated HMDB0000517, spectral sim. = 885, #14

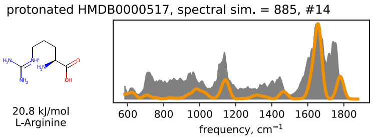


protonated HMDB0000517, spectral sim. = 885, #15

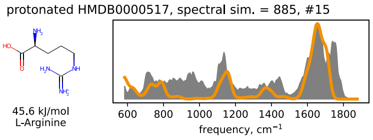


protonated HMDB0000517, spectral sim. = 869, #16

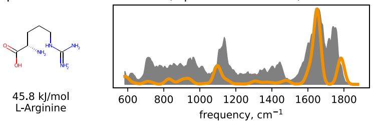


protonated HMDB0000517, spectral sim. = 858, #17

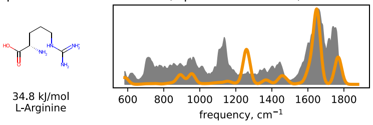


protonated HMDB0000517, spectral sim. = 855, #18

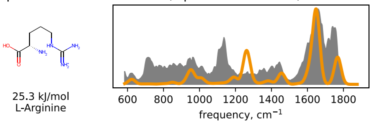


protonated HMDB0000517, spectral sim. = 849, #19

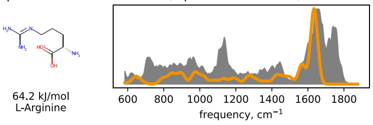


protonated HMDB0000517, spectral sim. = 838, #20

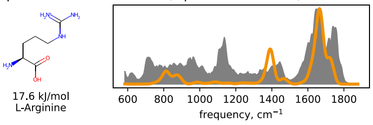


protonated HMDB0000517, spectral sim. = 834, #21

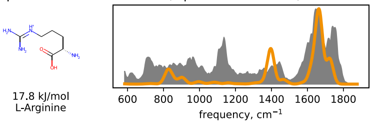


protonated HMDB0000517, spectral sim. = 820, #22

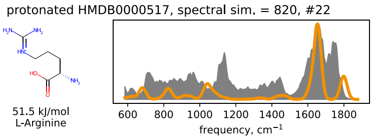

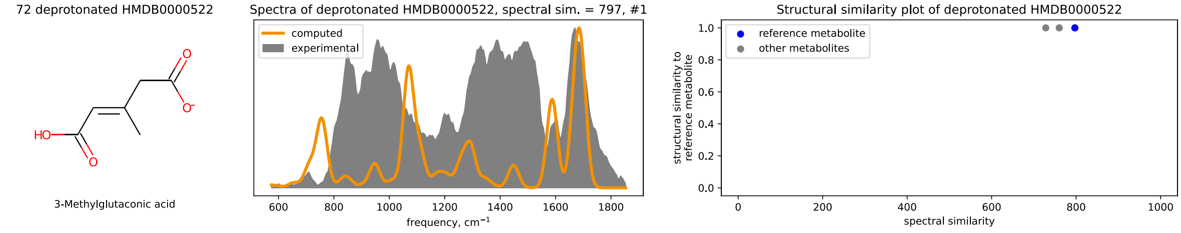
72 deprotonated HMDB0000522
3-Methylglutaconic acid
Spectra of deprotonated HMDB0000522, spectral sim. = 797, #1
computed
experimental
frequency, cm⁻¹
Structural similarity plot of deprotonated HMDB0000522
reference metabolite
other metabolites
structural similarity to reference metabolite
spectral similarity

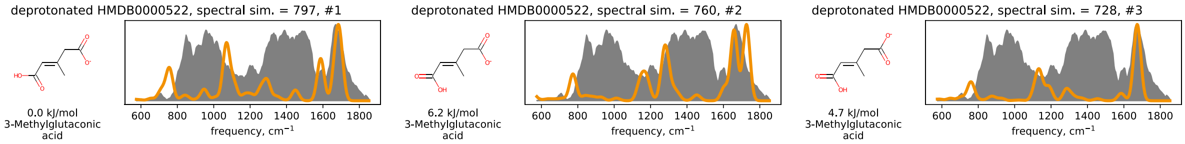
deprotonated HMDB0000522, spectral sim. = 797, #1
0.0 kJ/mol
3-Methylglutaconic acid
deprotonated HMDB0000522, spectral sim. = 760, #2
6.2 kJ/mol
3-Methylglutaconic acid
deprotonated HMDB0000522, spectral sim. = 728, #3
4.7 kJ/mol
3-Methylglutaconic acid
frequency, cm⁻¹

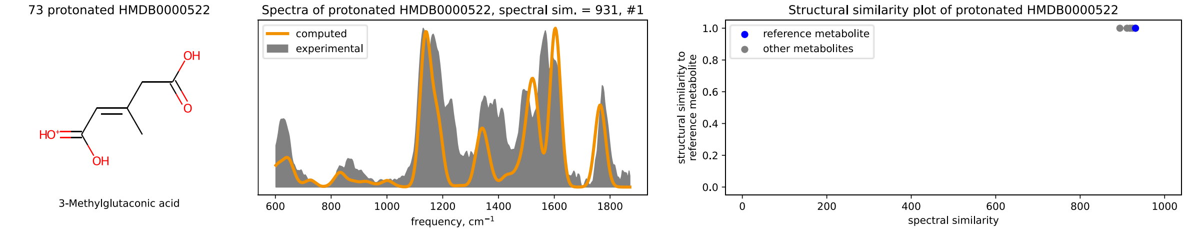
73 protonated HMDB0000522
3-Methylglutaconic acid
Spectra of protonated HMDB0000522, spectral sim. = 931, #1
computed
experimental
frequency, cm⁻¹
Structural similarity plot of protonated HMDB0000522
reference metabolite
other metabolites
structural similarity to reference metabolite
spectral similarity

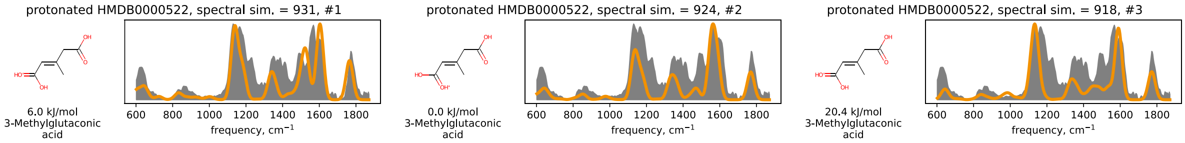
protonated HMDB0000522, spectral sim. = 931, #1
6.0 kJ/mol
3-Methylglutaconic acid
protonated HMDB0000522, spectral sim. = 924, #2
0.0 kJ/mol
3-Methylglutaconic acid
protonated HMDB0000522, spectral sim. = 918, #3
20.4 kJ/mol
3-Methylglutaconic acid
frequency, cm⁻¹

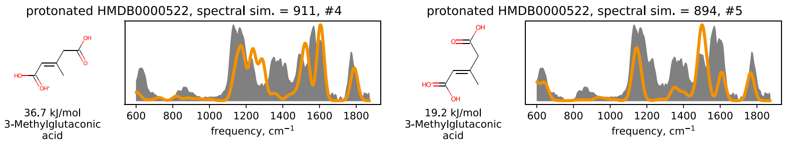
protonated HMDB0000522, spectral sim. = 911, #4
36.7 kJ/mol
3-Methylglutaconic acid
protonated HMDB0000522, spectral sim. = 894, #5
19.2 kJ/mol
3-Methylglutaconic acid
frequency, cm⁻¹

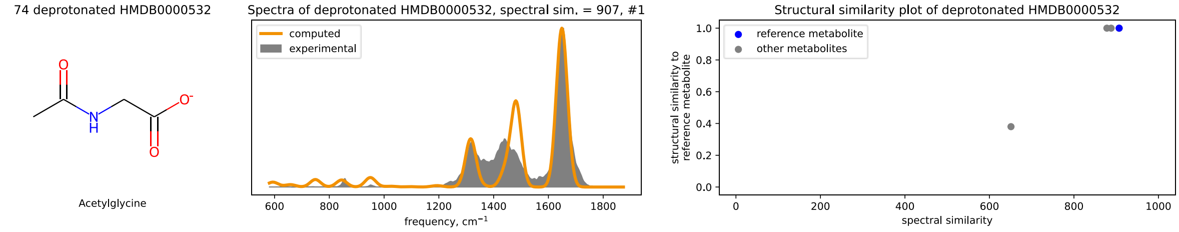
74 deprotonated HMDB0000532
Acetylglycine
Spectra of deprotonated HMDB0000532, spectral sim. = 907, #1
computed
experimental
frequency, cm⁻¹
Structural similarity plot of deprotonated HMDB0000532
reference metabolite
other metabolites
structural similarity to reference metabolite
spectral similarity

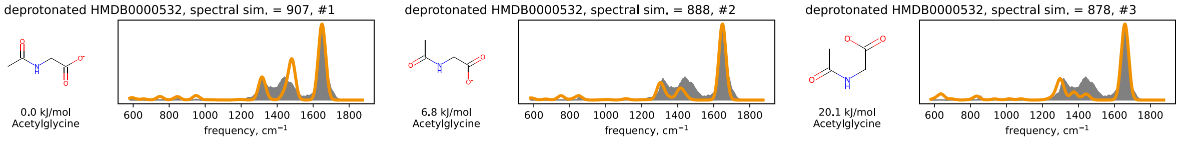
deprotonated HMDB0000532, spectral sim. = 907, #1
0.0 kJ/mol
Acetylglycine
deprotonated HMDB0000532, spectral sim. = 888, #2
6.8 kJ/mol
Acetylglycine
deprotonated HMDB0000532, spectral sim. = 878, #3
20.1 kJ/mol
Acetylglycine
frequency, cm⁻¹

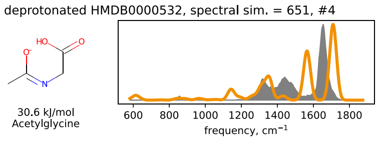
deprotonated HMDB0000532, spectral sim. = 651, #4
30.6 kJ/mol
Acetylglycine
frequency, cm⁻¹

75 protonated HMDB0000532

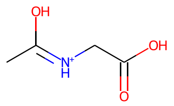


Acetylglycine

Spectra of protonated HMDB0000532, spectral sim. = 925, #1

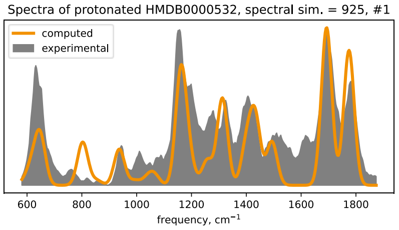


Structural similarity plot of protonated HMDB0000532

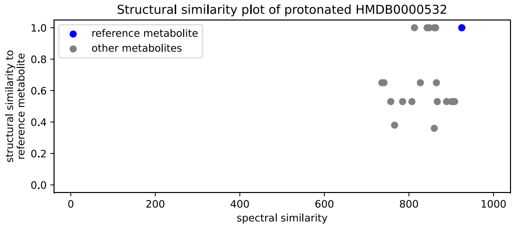


protonated HMDB0000532, spectral sim. = 925, #1

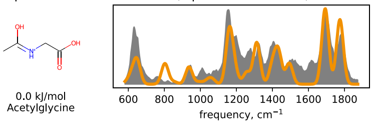


protonated HMDB0000532, spectral sim. = 908, #2

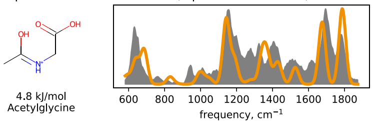


protonated HMDB0000532, spectral sim. = 901, #3

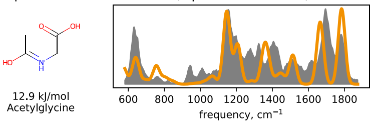


protonated HMDB0000532, spectral sim. = 889, #4

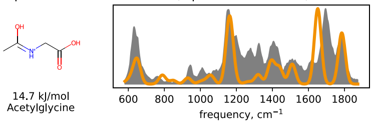


protonated HMDB0000532, spectral sim. = 867, #5

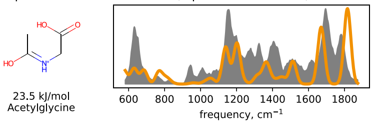


protonated HMDB0000532, spectral sim. = 865, #6

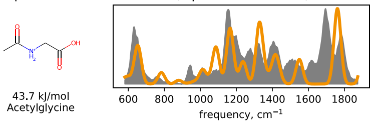


protonated HMDB0000532, spectral sim. = 863, #7

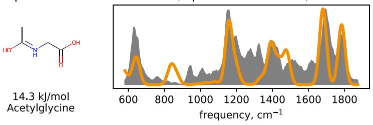


protonated HMDB0000532, spectral sim. = 860, #8

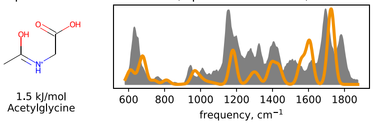


protonated HMDB0000532, spectral sim. = 860, #9

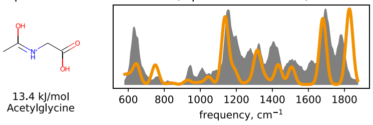


protonated HMDB0000532, spectral sim. = 848, #10

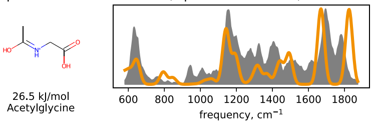


protonated HMDB0000532, spectral sim. = 843, #11

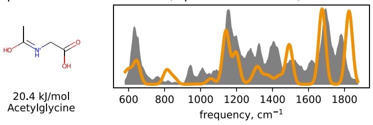


protonated HMDB0000532, spectral sim. = 827, #12

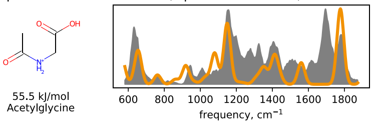


protonated HMDB0000532, spectral sim. = 813, #13

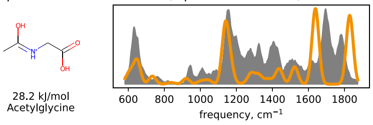


protonated HMDB0000532, spectral sim. = 807, #14

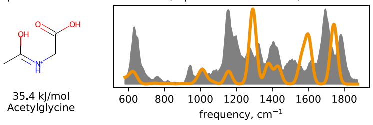


protonated HMDB0000532, spectral sim. = 785, #15

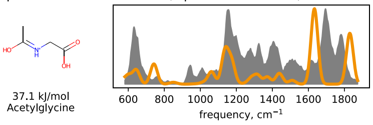


protonated HMDB0000532, spectral sim. = 766, #16

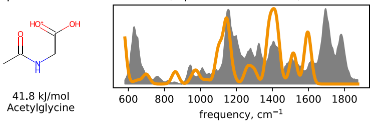


protonated HMDB0000532, spectral sim. = 757, #17

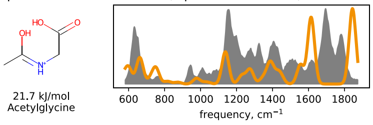


protonated HMDB0000532, spectral sim. = 741, #18

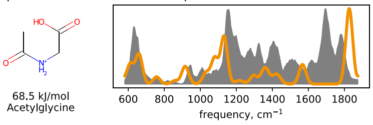


protonated HMDB0000532, spectral sim. = 736, #19

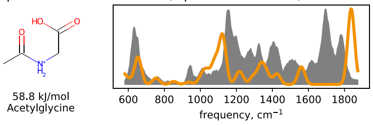

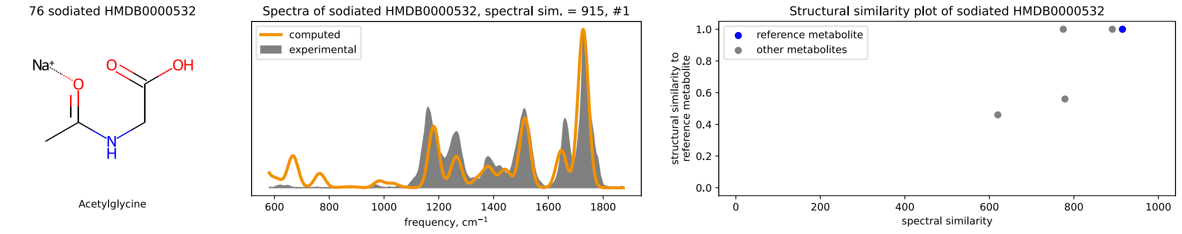
76 sodiated HMDB0000532
Na+
O
OH
N
H
Acetylglycine
Spectra of sodiated HMDB0000532, spectral sim. = 915, #1
computed
experimental
600 800 1000 1200 1400 1600 1800
frequency, cm−1
Structural similarity plot of sodiated HMDB0000532
reference metabolite
other metabolites
structural similarity to reference metabolite
0.0 0.2 0.4 0.6 0.8 1.0
0 200 400 600 800 1000
spectral similarity

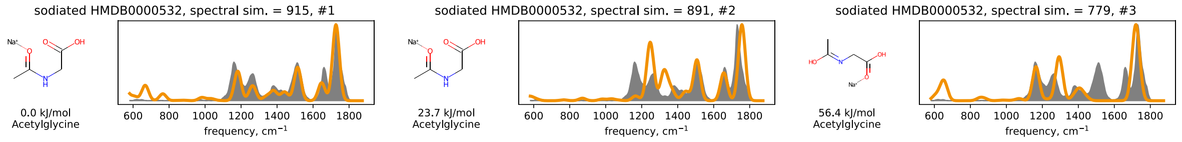
sodiated HMDB0000532, spectral sim. = 915, #1
0.0 kJ/mol
Acetylglycine
sodiated HMDB0000532, spectral sim. = 891, #2
23.7 kJ/mol
Acetylglycine
sodiated HMDB0000532, spectral sim. = 779, #3
56.4 kJ/mol
Acetylglycine
600 800 1000 1200 1400 1600 1800
frequency, cm−1

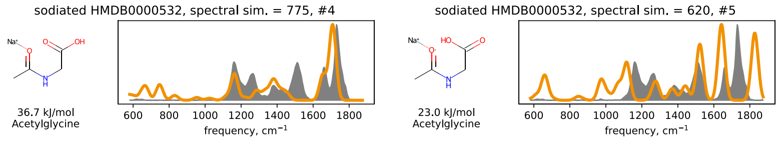
sodiated HMDB0000532, spectral sim. = 775, #4
36.7 kJ/mol
Acetylglycine
sodiated HMDB0000532, spectral sim. = 620, #5
23.0 kJ/mol
Acetylglycine
600 800 1000 1200 1400 1600 1800
frequency, cm−1

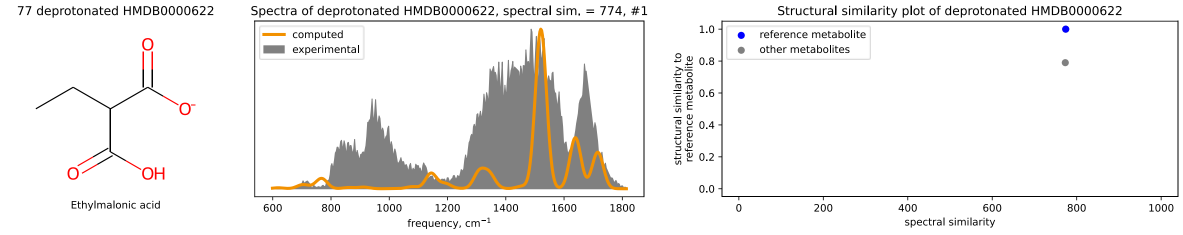
77 deprotonated HMDB0000622
O
O-
O
OH
Ethylmalonic acid
Spectra of deprotonated HMDB0000622, spectral sim. = 774, #1
computed
experimental
600 800 1000 1200 1400 1600 1800
frequency, cm−1
Structural similarity plot of deprotonated HMDB0000622
reference metabolite
other metabolites
structural similarity to reference metabolite
0.0 0.2 0.4 0.6 0.8 1.0
0 200 400 600 800 1000
spectral similarity

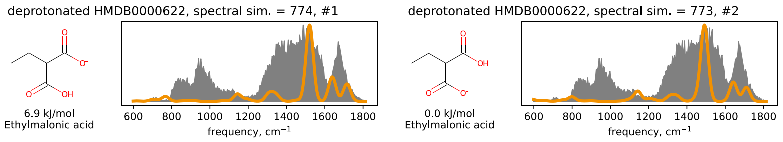
deprotonated HMDB0000622, spectral sim. = 774, #1
6.9 kJ/mol
Ethylmalonic acid
deprotonated HMDB0000622, spectral sim. = 773, #2
0.0 kJ/mol
Ethylmalonic acid
600 800 1000 1200 1400 1600 1800
frequency, cm−1

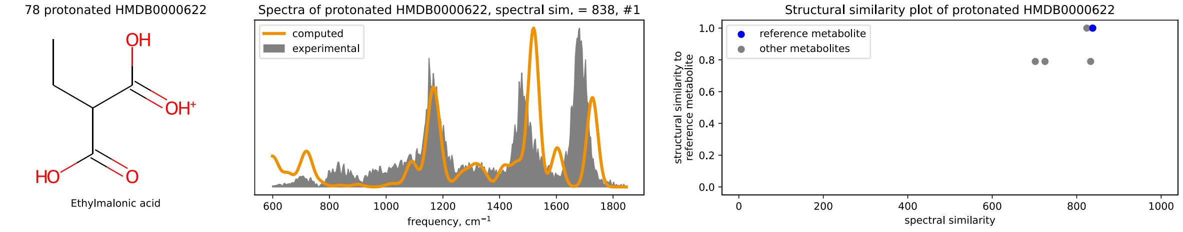
78 protonated HMDB0000622
OH
OH+
HO
O
Ethylmalonic acid
Spectra of protonated HMDB0000622, spectral sim. = 838, #1
computed
experimental
600 800 1000 1200 1400 1600 1800
frequency, cm−1
Structural similarity plot of protonated HMDB0000622
reference metabolite
other metabolites
structural similarity to reference metabolite
0.0 0.2 0.4 0.6 0.8 1.0
0 200 400 600 800 1000
spectral similarity

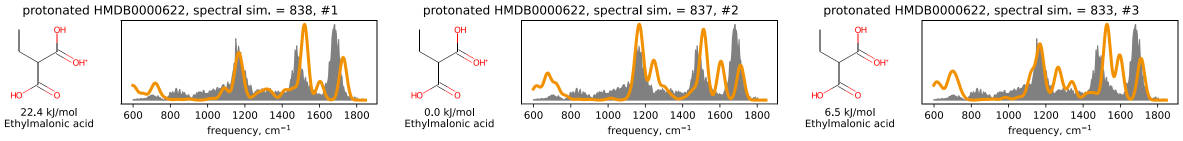
protonated HMDB0000622, spectral sim. = 838, #1
22.4 kJ/mol
Ethylmalonic acid
protonated HMDB0000622, spectral sim. = 837, #2
0.0 kJ/mol
Ethylmalonic acid
protonated HMDB0000622, spectral sim. = 833, #3
6.5 kJ/mol
Ethylmalonic acid
600 800 1000 1200 1400 1600 1800
frequency, cm−1

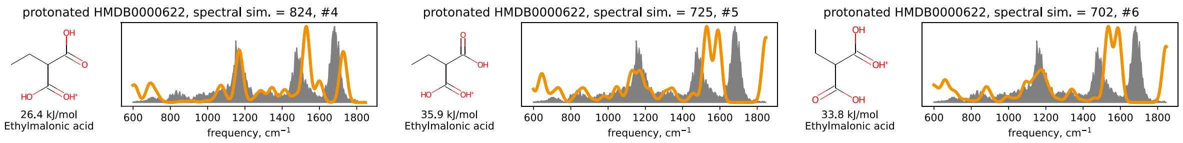
protonated HMDB0000622, spectral sim. = 824, #4
26.4 kJ/mol
Ethylmalonic acid
protonated HMDB0000622, spectral sim. = 725, #5
35.9 kJ/mol
Ethylmalonic acid
protonated HMDB0000622, spectral sim. = 702, #6
33.8 kJ/mol
Ethylmalonic acid
600 800 1000 1200 1400 1600 1800
frequency, cm−1

79 deprotonated HMDB0000630

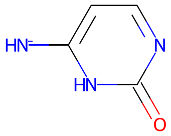


Cytosine

Spectra of deprotonated HMDB0000630, spectral sim. = 913, #1

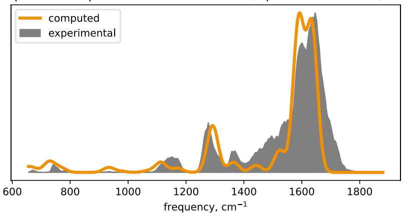


Structural similarity plot of deprotonated HMDB0000630

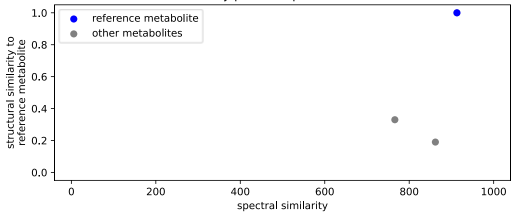


deprotonated HMDB0000630, spectral sim. = 913, #1

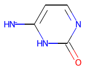

0.0 kJ/mol
Cytosine

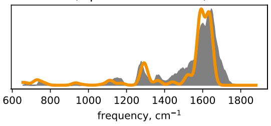


deprotonated HMDB0000630, spectral sim. = 862, #2

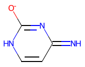

30.3 kJ/mol
Cytosine

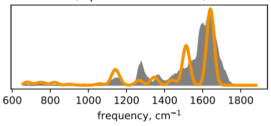


deprotonated HMDB0000630, spectral sim. = 766, #3

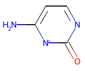

11.3 kJ/mol
Cytosine

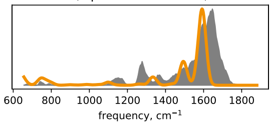


80 protonated HMDB0000630

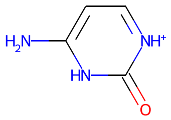


Cytosine

Spectra of protonated HMDB0000630, spectral sim. = 796, #1

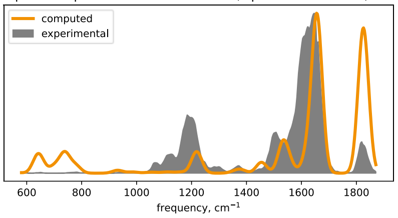


Structural similarity plot of protonated HMDB0000630

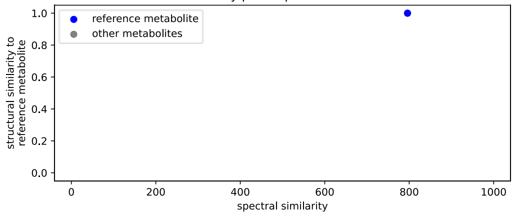


protonated HMDB0000630, spectral sim. = 796, #1

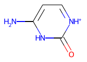

0.0 kJ/mol
Cytosine

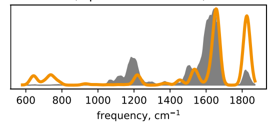


81 sodiated HMDB0000630

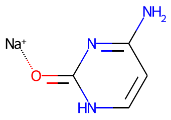


Cytosine

Spectra of sodiated HMDB0000630, spectral sim. = 933, #1

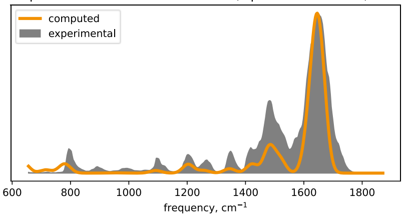


Structural similarity plot of sodiated HMDB0000630

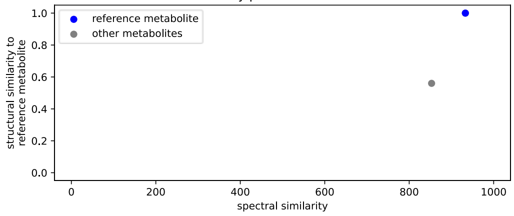


sodiated HMDB0000630, spectral sim. = 933, #1

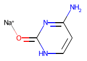

0.0 kJ/mol
Cytosine

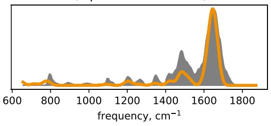


sodiated HMDB0000630, spectral sim. = 853, #2

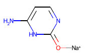

19.2 kJ/mol
Cytosine

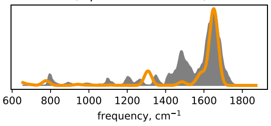

82 deprotonated HMDB0000641

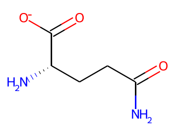

L-Glutamine

Spectra of deprotonated HMDB0000641, spectral sim. = 913, #1

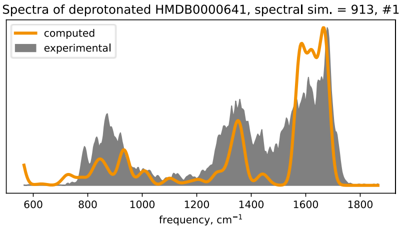


Structural similarity plot of deprotonated HMDB0000641

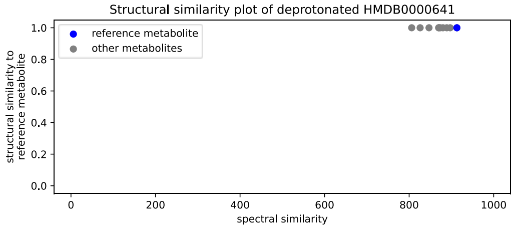


deprotonated HMDB0000641, spectral sim. = 913, #1

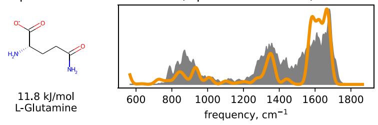


deprotonated HMDB0000641, spectral sim. = 897, #2

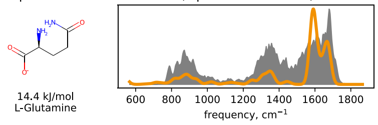


deprotonated HMDB0000641, spectral sim. = 889, #3

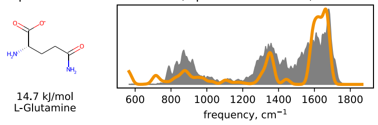


deprotonated HMDB0000641, spectral sim. = 880, #4

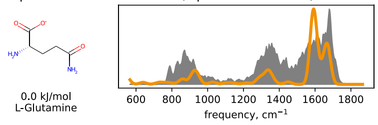


deprotonated HMDB0000641, spectral sim. = 874, #5

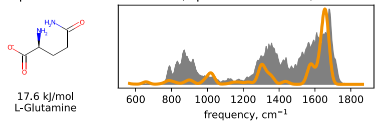


deprotonated HMDB0000641, spectral sim. = 870, #6

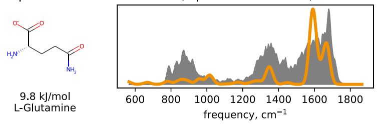


deprotonated HMDB0000641, spectral sim. = 870, #7

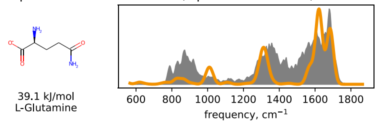


deprotonated HMDB0000641, spectral sim. = 847, #8

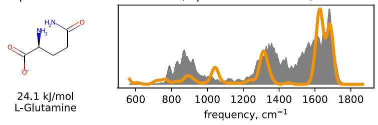


deprotonated HMDB0000641, spectral sim. = 826, #9

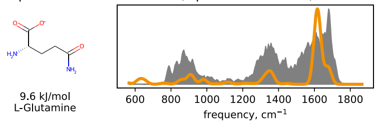


deprotonated HMDB0000641, spectral sim. = 806, #10

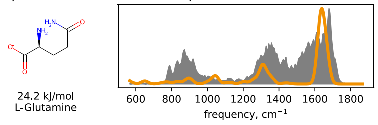


83 protonated HMDB0000641

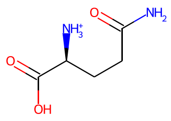

L-Glutamine

Spectra of protonated HMDB0000641, spectral sim. = 911, #1

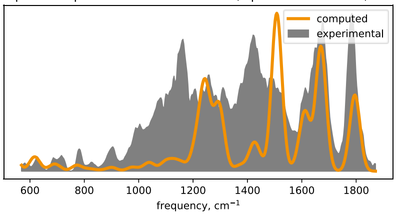


Structural similarity plot of protonated HMDB0000641

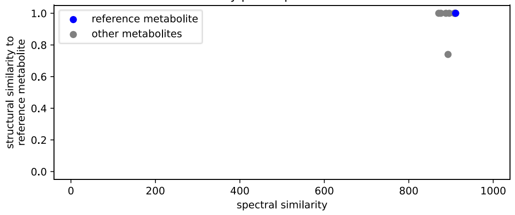


protonated HMDB0000641, spectral sim. = 911, #1

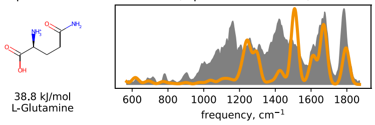


protonated HMDB0000641, spectral sim. = 909, #2

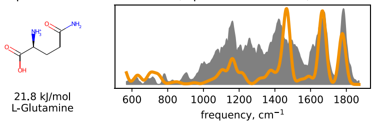


protonated HMDB0000641, spectral sim. = 908, #3

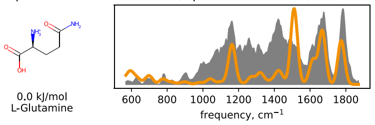


protonated HMDB0000641, spectral sim. = 896, #4

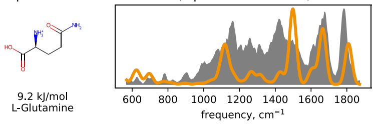


protonated HMDB0000641, spectral sim. = 893, #5

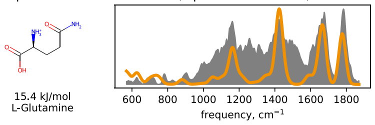


protonated HMDB0000641, spectral sim. = 888, #6

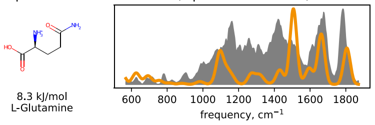


protonated HMDB0000641, spectral sim. = 876, #7

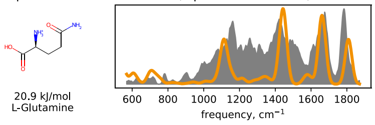


protonated HMDB0000641, spectral sim. = 871, #8

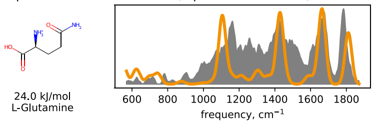

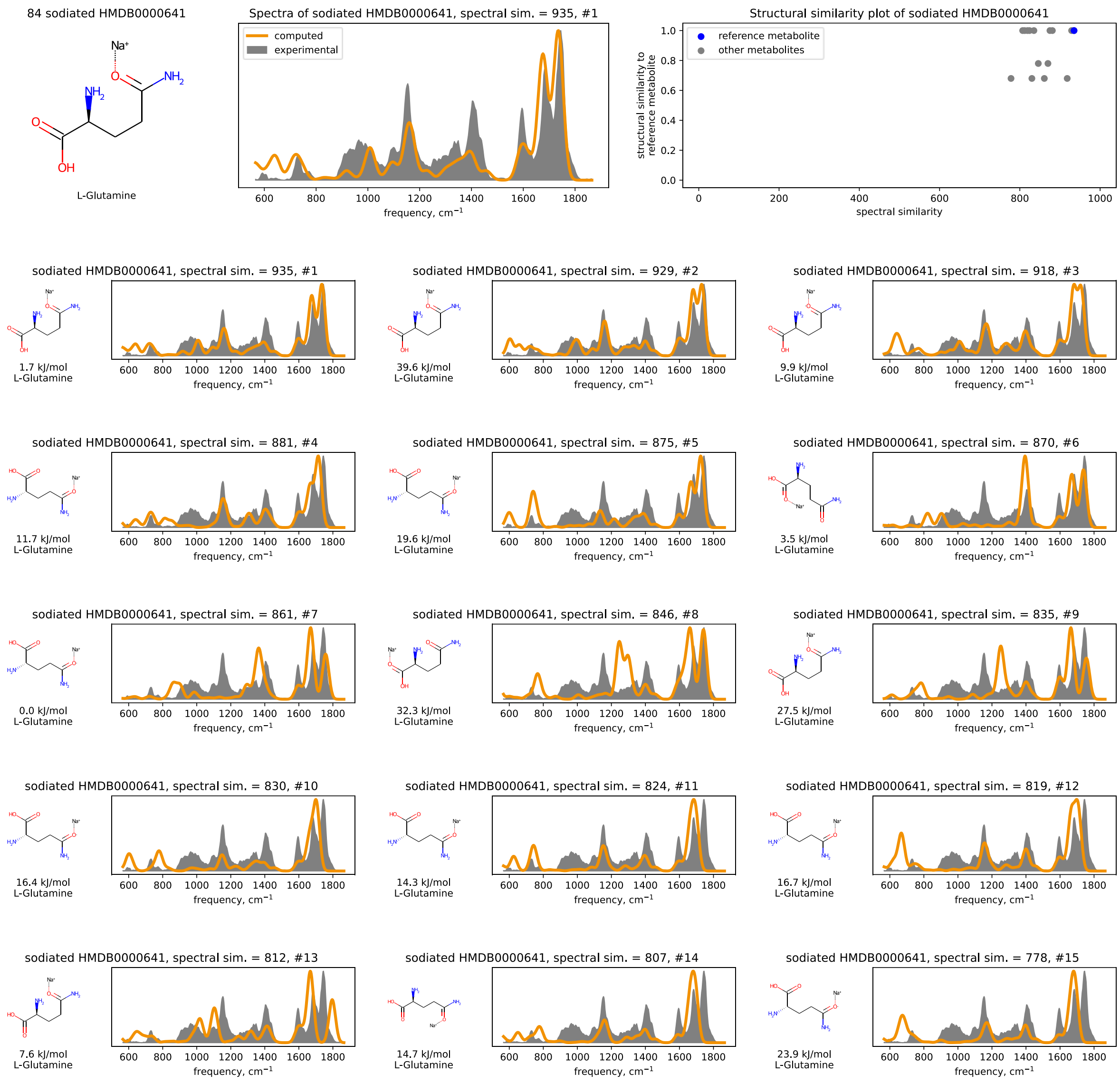
84 sodiated HMDB0000641
Na⁺
O
NH2
NH2
O
OH
L-Glutamine
Spectra of sodiated HMDB0000641, spectral sim. = 935, #1
computed
experimental
frequency, cm⁻¹
Structural similarity plot of sodiated HMDB0000641
reference metabolite
other metabolites
structural similarity to reference metabolite
spectral similarity
sodiated HMDB0000641, spectral sim. = 935, #1
1.7 kJ/mol
L-Glutamine
sodiated HMDB0000641, spectral sim. = 929, #2
39.6 kJ/mol
L-Glutamine
sodiated HMDB0000641, spectral sim. = 918, #3
9.9 kJ/mol
L-Glutamine
sodiated HMDB0000641, spectral sim. = 881, #4
11.7 kJ/mol
L-Glutamine
sodiated HMDB0000641, spectral sim. = 875, #5
19.6 kJ/mol
L-Glutamine
sodiated HMDB0000641, spectral sim. = 870, #6
3.5 kJ/mol
L-Glutamine
sodiated HMDB0000641, spectral sim. = 861, #7
0.0 kJ/mol
L-Glutamine
sodiated HMDB0000641, spectral sim. = 846, #8
32.3 kJ/mol
L-Glutamine
sodiated HMDB0000641, spectral sim. = 835, #9
27.5 kJ/mol
L-Glutamine
sodiated HMDB0000641, spectral sim. = 830, #10
16.4 kJ/mol
L-Glutamine
sodiated HMDB0000641, spectral sim. = 824, #11
14.3 kJ/mol
L-Glutamine
sodiated HMDB0000641, spectral sim. = 819, #12
16.7 kJ/mol
L-Glutamine
sodiated HMDB0000641, spectral sim. = 812, #13
7.6 kJ/mol
L-Glutamine
sodiated HMDB0000641, spectral sim. = 807, #14
14.7 kJ/mol
L-Glutamine
sodiated HMDB0000641, spectral sim. = 778, #15
23.9 kJ/mol
L-Glutamine
frequency, cm⁻¹

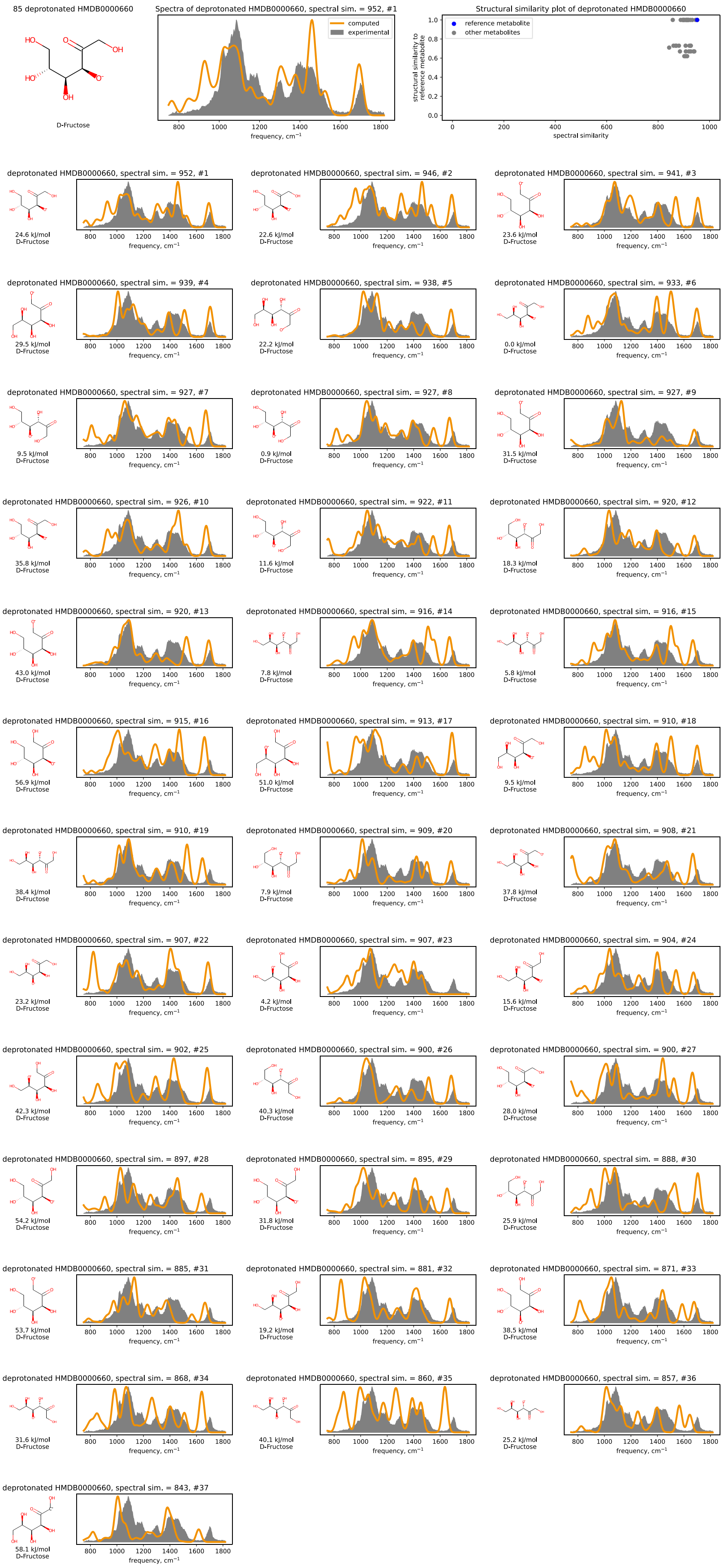

85 deprotonated HMDB0000660
D-Fructose
Spectra of deprotonated HMDB0000660, spectral sim. = 952, #1
computed
experimental
frequency, cm⁻¹
Structural similarity plot of deprotonated HMDB0000660
reference metabolite
other metabolites
structural similarity to reference metabolite
spectral similarity
deprotonated HMDB0000660, spectral sim. = 952, #1
24.6 kJ/mol D-Fructose
deprotonated HMDB0000660, spectral sim. = 946, #2
22.6 kJ/mol D-Fructose
deprotonated HMDB0000660, spectral sim. = 941, #3
23.6 kJ/mol D-Fructose
deprotonated HMDB0000660, spectral sim. = 939, #4
29.5 kJ/mol D-Fructose
deprotonated HMDB0000660, spectral sim. = 938, #5
22.2 kJ/mol D-Fructose
deprotonated HMDB0000660, spectral sim. = 933, #6
0.0 kJ/mol D-Fructose
deprotonated HMDB0000660, spectral sim. = 927, #7
9.5 kJ/mol D-Fructose
deprotonated HMDB0000660, spectral sim. = 927, #8
0.9 kJ/mol D-Fructose
deprotonated HMDB0000660, spectral sim. = 927, #9
31.5 kJ/mol D-Fructose
deprotonated HMDB0000660, spectral sim. = 926, #10
35.8 kJ/mol D-Fructose
deprotonated HMDB0000660, spectral sim. = 922, #11
11.6 kJ/mol D-Fructose
deprotonated HMDB0000660, spectral sim. = 920, #12
18.3 kJ/mol D-Fructose
deprotonated HMDB0000660, spectral sim. = 920, #13
43.0 kJ/mol D-Fructose
deprotonated HMDB0000660, spectral sim. = 916, #14
7.8 kJ/mol D-Fructose
deprotonated HMDB0000660, spectral sim. = 916, #15
5.8 kJ/mol D-Fructose
deprotonated HMDB0000660, spectral sim. = 915, #16
56.9 kJ/mol D-Fructose
deprotonated HMDB0000660, spectral sim. = 913, #17
51.0 kJ/mol D-Fructose
deprotonated HMDB0000660, spectral sim. = 910, #18
9.5 kJ/mol D-Fructose
deprotonated HMDB0000660, spectral sim. = 910, #19
38.4 kJ/mol D-Fructose
deprotonated HMDB0000660, spectral sim. = 909, #20
7.9 kJ/mol D-Fructose
deprotonated HMDB0000660, spectral sim. = 908, #21
37.8 kJ/mol D-Fructose
deprotonated HMDB0000660, spectral sim. = 907, #22
23.2 kJ/mol D-Fructose
deprotonated HMDB0000660, spectral sim. = 907, #23
4.2 kJ/mol D-Fructose
deprotonated HMDB0000660, spectral sim. = 904, #24
15.6 kJ/mol D-Fructose
deprotonated HMDB0000660, spectral sim. = 902, #25
42.3 kJ/mol D-Fructose
deprotonated HMDB0000660, spectral sim. = 900, #26
40.3 kJ/mol D-Fructose
deprotonated HMDB0000660, spectral sim. = 900, #27
28.0 kJ/mol D-Fructose
deprotonated HMDB0000660, spectral sim. = 897, #28
54.2 kJ/mol D-Fructose
deprotonated HMDB0000660, spectral sim. = 895, #29
31.8 kJ/mol D-Fructose
deprotonated HMDB0000660, spectral sim. = 888, #30
25.9 kJ/mol D-Fructose
deprotonated HMDB0000660, spectral sim. = 885, #31
53.7 kJ/mol D-Fructose
deprotonated HMDB0000660, spectral sim. = 881, #32
19.2 kJ/mol D-Fructose
deprotonated HMDB0000660, spectral sim. = 871, #33
38.5 kJ/mol D-Fructose
deprotonated HMDB0000660, spectral sim. = 868, #34
31.6 kJ/mol D-Fructose
deprotonated HMDB0000660, spectral sim. = 860, #35
40.1 kJ/mol D-Fructose
deprotonated HMDB0000660, spectral sim. = 857, #36
25.2 kJ/mol D-Fructose
deprotonated HMDB0000660, spectral sim. = 843, #37
58.1 kJ/mol D-Fructose

86 deprotonated HMDB0000661

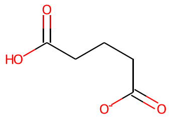


Glutaric acid

Spectra of deprotonated HMDB0000661, spectral sim. = 717, #1

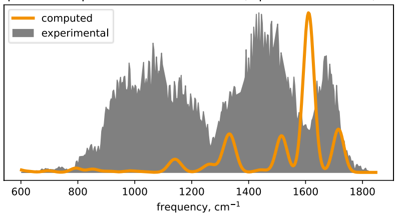


Structural similarity plot of deprotonated HMDB0000661

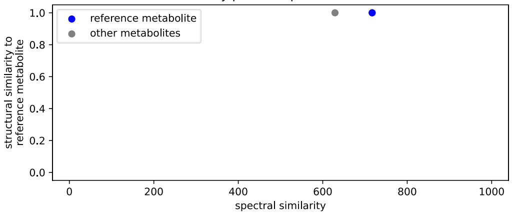


deprotonated HMDB0000661, spectral sim. = 717, #1

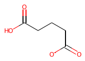

11.1 kJ/mol
Glutaric acid

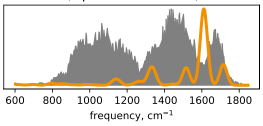


deprotonated HMDB0000661, spectral sim. = 629, #2

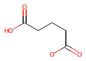

0.0 kJ/mol
Glutaric acid

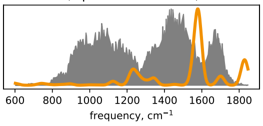


87 protonated HMDB0000661

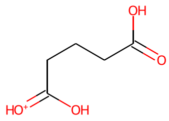


Glutaric acid

Spectra of protonated HMDB0000661, spectral sim. = 798, #1

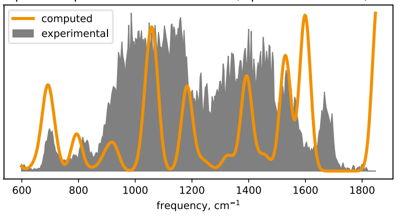


Structural similarity plot of protonated HMDB0000661

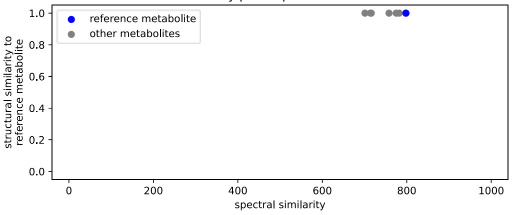


protonated HMDB0000661, spectral sim. = 798, #1

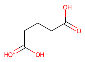

48.6 kJ/mol
Glutaric acid

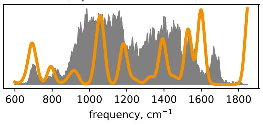


protonated HMDB0000661, spectral sim. = 782, #2

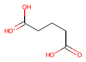

47.2 kJ/mol
Glutaric acid

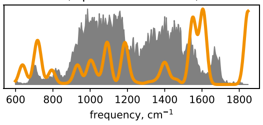


protonated HMDB0000661, spectral sim. = 775, #3

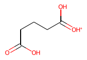

44.0 kJ/mol
Glutaric acid

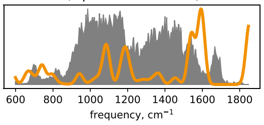


protonated HMDB0000661, spectral sim. = 758, #4

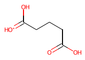

56.9 kJ/mol
Glutaric acid

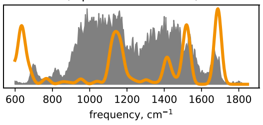


protonated HMDB0000661, spectral sim. = 716, #5

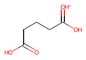

22.8 kJ/mol
Glutaric acid

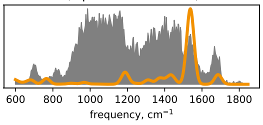


protonated HMDB0000661, spectral sim. = 714, #6

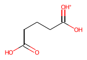

1.9 kJ/mol
Glutaric acid

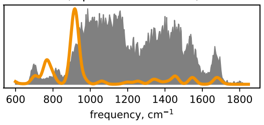


protonated HMDB0000661, spectral sim. = 701, #7

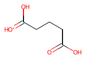

0.0 kJ/mol
Glutaric acid

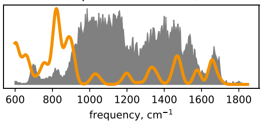

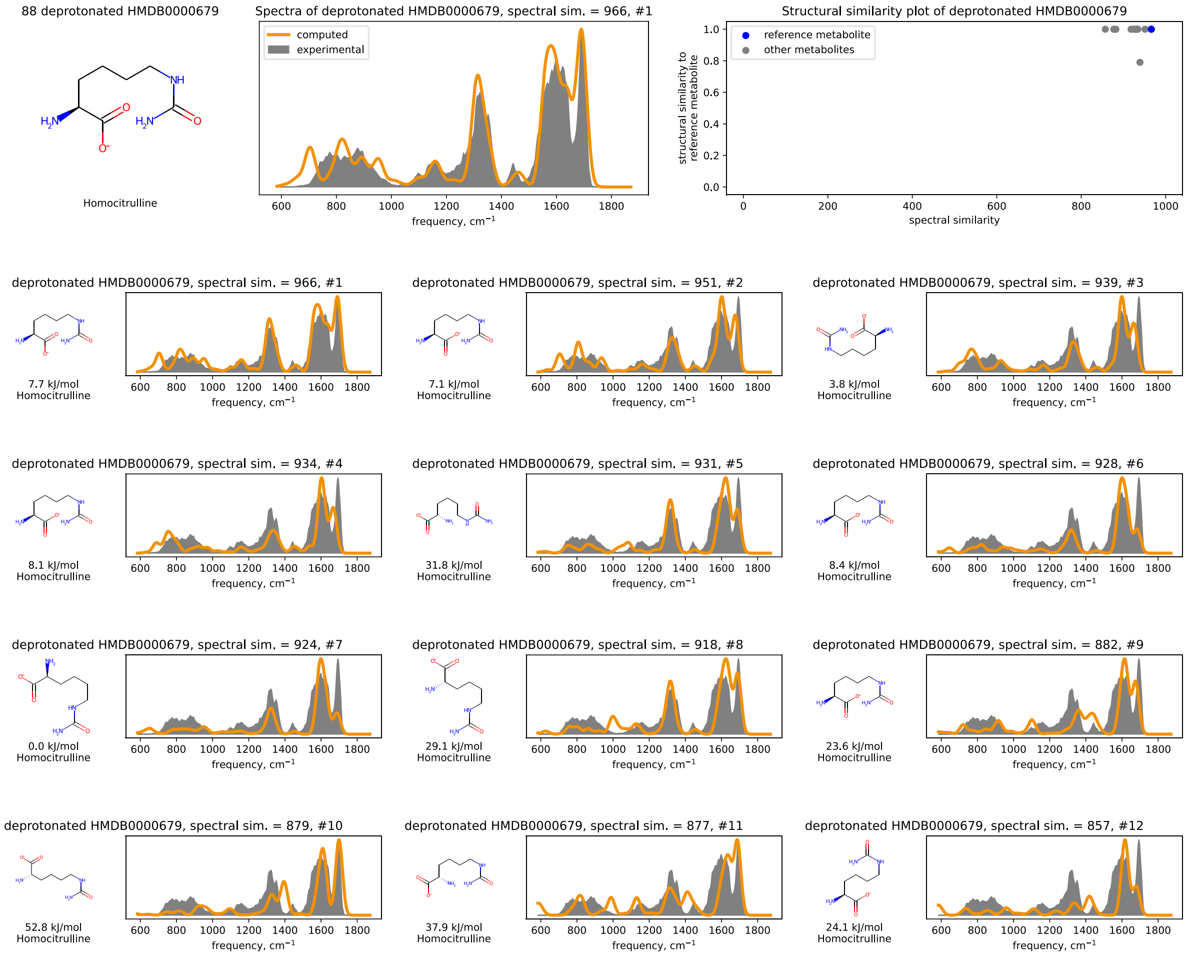

88 deprotonated HMDB0000679
Homocitrulline
Spectra of deprotonated HMDB0000679, spectral sim. = 966, #1
computed
experimental
frequency, cm⁻¹
Structural similarity plot of deprotonated HMDB0000679
reference metabolite
other metabolites
structural similarity to reference metabolite
spectral similarity
deprotonated HMDB0000679, spectral sim. = 966, #1
7.7 kJ/mol
Homocitrulline
deprotonated HMDB0000679, spectral sim. = 951, #2
7.1 kJ/mol
Homocitrulline
deprotonated HMDB0000679, spectral sim. = 939, #3
3.8 kJ/mol
Homocitrulline
deprotonated HMDB0000679, spectral sim. = 934, #4
8.1 kJ/mol
Homocitrulline
deprotonated HMDB0000679, spectral sim. = 931, #5
31.8 kJ/mol
Homocitrulline
deprotonated HMDB0000679, spectral sim. = 928, #6
8.4 kJ/mol
Homocitrulline
deprotonated HMDB0000679, spectral sim. = 924, #7
0.0 kJ/mol
Homocitrulline
deprotonated HMDB0000679, spectral sim. = 918, #8
29.1 kJ/mol
Homocitrulline
deprotonated HMDB0000679, spectral sim. = 882, #9
23.6 kJ/mol
Homocitrulline
deprotonated HMDB0000679, spectral sim. = 879, #10
52.8 kJ/mol
Homocitrulline
deprotonated HMDB0000679, spectral sim. = 877, #11
37.9 kJ/mol
Homocitrulline
deprotonated HMDB0000679, spectral sim. = 857, #12
24.1 kJ/mol
Homocitrulline

89 protonated HMDB0000679

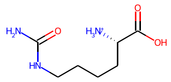


Homocitrulline

Spectra of protonated HMDB0000679, spectral sim. = 956, #1

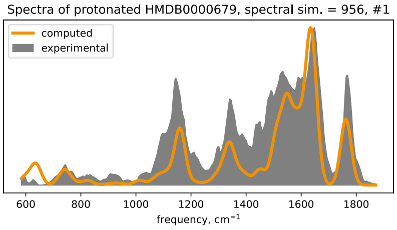


Structural similarity plot of protonated HMDB0000679

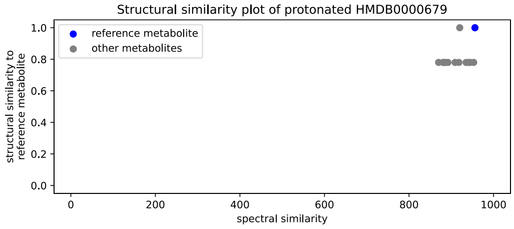


protonated HMDB0000679, spectral sim. = 956, #1

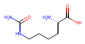

0.0 kJ/mol
Homocitrulline

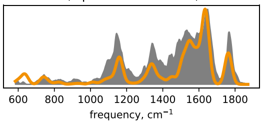


protonated HMDB0000679, spectral sim. = 953, #2

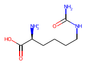

10.0 kJ/mol
Homocitrulline

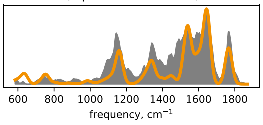


protonated HMDB0000679, spectral sim. = 945, #3

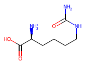

21.4 kJ/mol
Homocitrulline

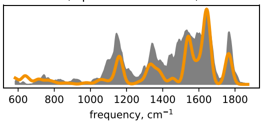


protonated HMDB0000679, spectral sim. = 940, #4

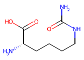

44.6 kJ/mol
Homocitrulline

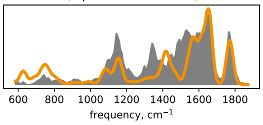


protonated HMDB0000679, spectral sim. = 935, #5

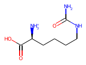

34.6 kJ/mol
Homocitrulline

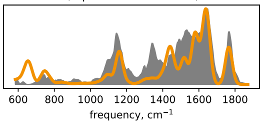


protonated HMDB0000679, spectral sim. = 920, #6

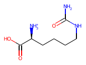

32.9 kJ/mol
Homocitrulline

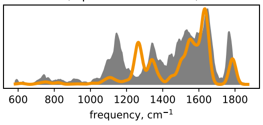


protonated HMDB0000679, spectral sim. = 918, #7

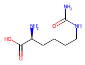

36.2 kJ/mol
Homocitrulline

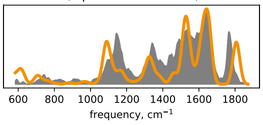


protonated HMDB0000679, spectral sim. = 909, #8

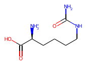

42.4 kJ/mol
Homocitrulline

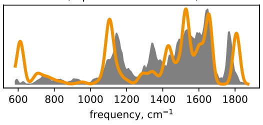


protonated HMDB0000679, spectral sim. = 892, #9

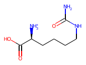

14.3 kJ/mol
Homocitrulline

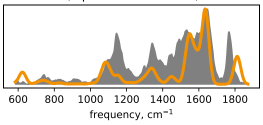


protonated HMDB0000679, spectral sim. = 886, #10

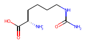

20.7 kJ/mol
Homocitrulline

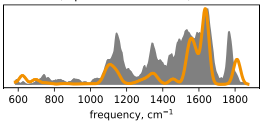


protonated HMDB0000679, spectral sim. = 882, #11

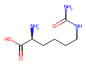

47.7 kJ/mol
Homocitrulline

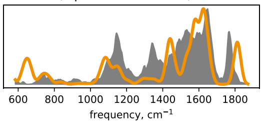


protonated HMDB0000679, spectral sim. = 881, #12

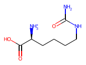

45.2 kJ/mol
Homocitrulline

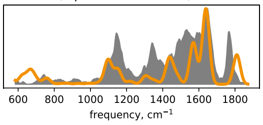


protonated HMDB0000679, spectral sim. = 870, #13

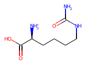

48.4 kJ/mol
Homocitrulline

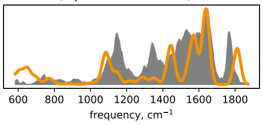

90 sodiated HMDB0000679

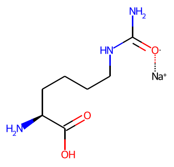


Homocitrulline

Spectra of sodiated HMDB0000679, spectral sim. = 939, #1

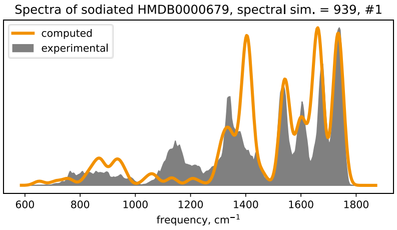


Structural similarity plot of sodiated HMDB0000679

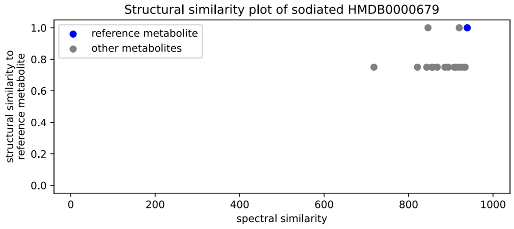


sodiated HMDB0000679, spectral sim. = 939, #1

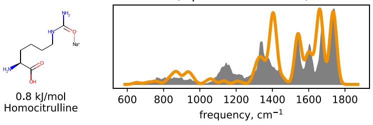


sodiated HMDB0000679, spectral sim. = 934, #2

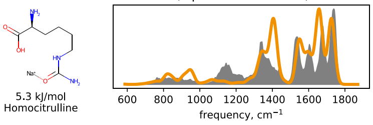


sodiated HMDB0000679, spectral sim. = 927, #3

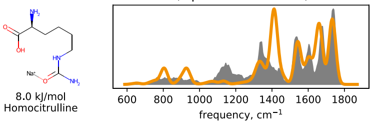


sodiated HMDB0000679, spectral sim. = 920, #4

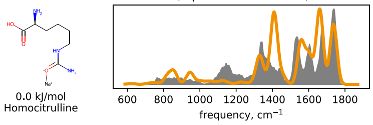


sodiated HMDB0000679, spectral sim. = 920, #5

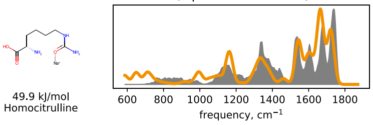


sodiated HMDB0000679, spectral sim. = 912, #6

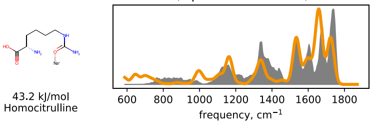


sodiated HMDB0000679, spectral sim. = 908, #7

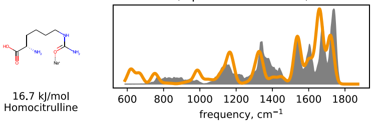


sodiated HMDB0000679, spectral sim. = 894, #8

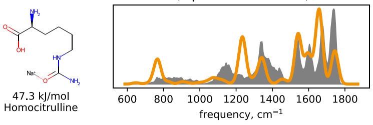


sodiated HMDB0000679, spectral sim. = 886, #9

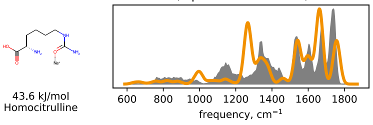


sodiated HMDB0000679, spectral sim. = 868, #10

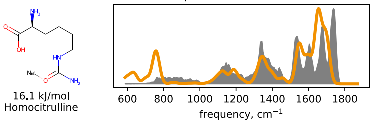


sodiated HMDB0000679, spectral sim. = 858, #11

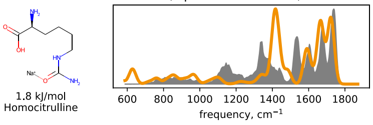


sodiated HMDB0000679, spectral sim. = 855, #12

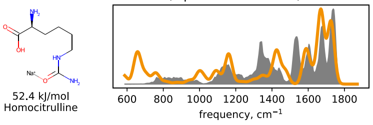


sodiated HMDB0000679, spectral sim. = 846, #13

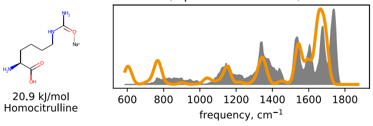


sodiated HMDB0000679, spectral sim. = 843, #14

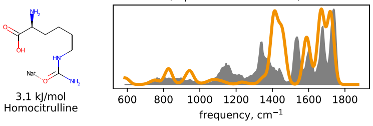


sodiated HMDB0000679, spectral sim. = 821, #15

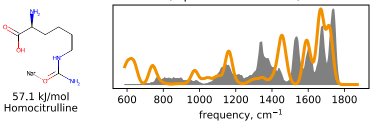


sodiated HMDB0000679, spectral sim. = 718, #16

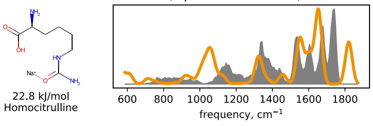

91 protonated HMDB0000687

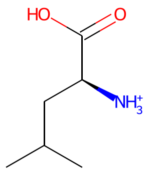


L-Leucine

Spectra of protonated HMDB0000687, spectral sim. = 917, #1

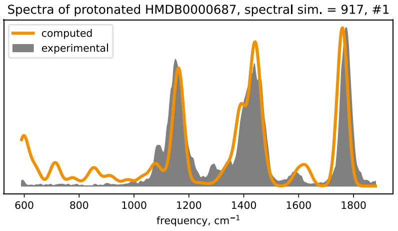


Structural similarity plot of protonated HMDB0000687

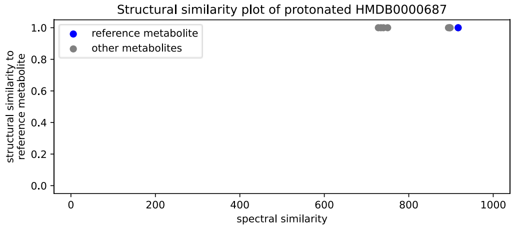


protonated HMDB0000687, spectral sim. = 917, #1

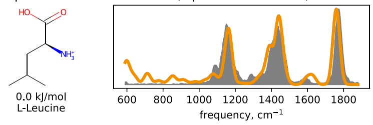


protonated HMDB0000687, spectral sim. = 898, #2

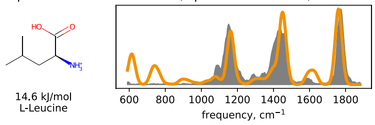


protonated HMDB0000687, spectral sim. = 897, #3

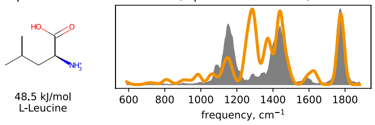


protonated HMDB0000687, spectral sim. = 894, #4

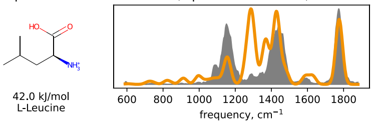


protonated HMDB0000687, spectral sim. = 750, #5

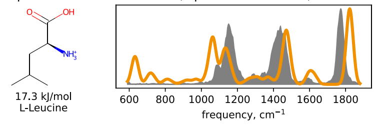


protonated HMDB0000687, spectral sim. = 740, #6

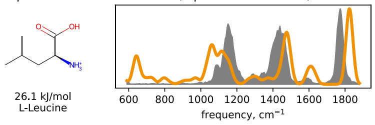


protonated HMDB0000687, spectral sim. = 734, #7

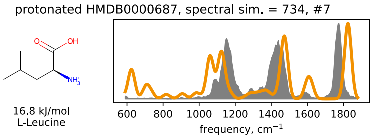


protonated HMDB0000687, spectral sim. = 728, #8

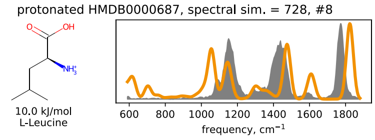

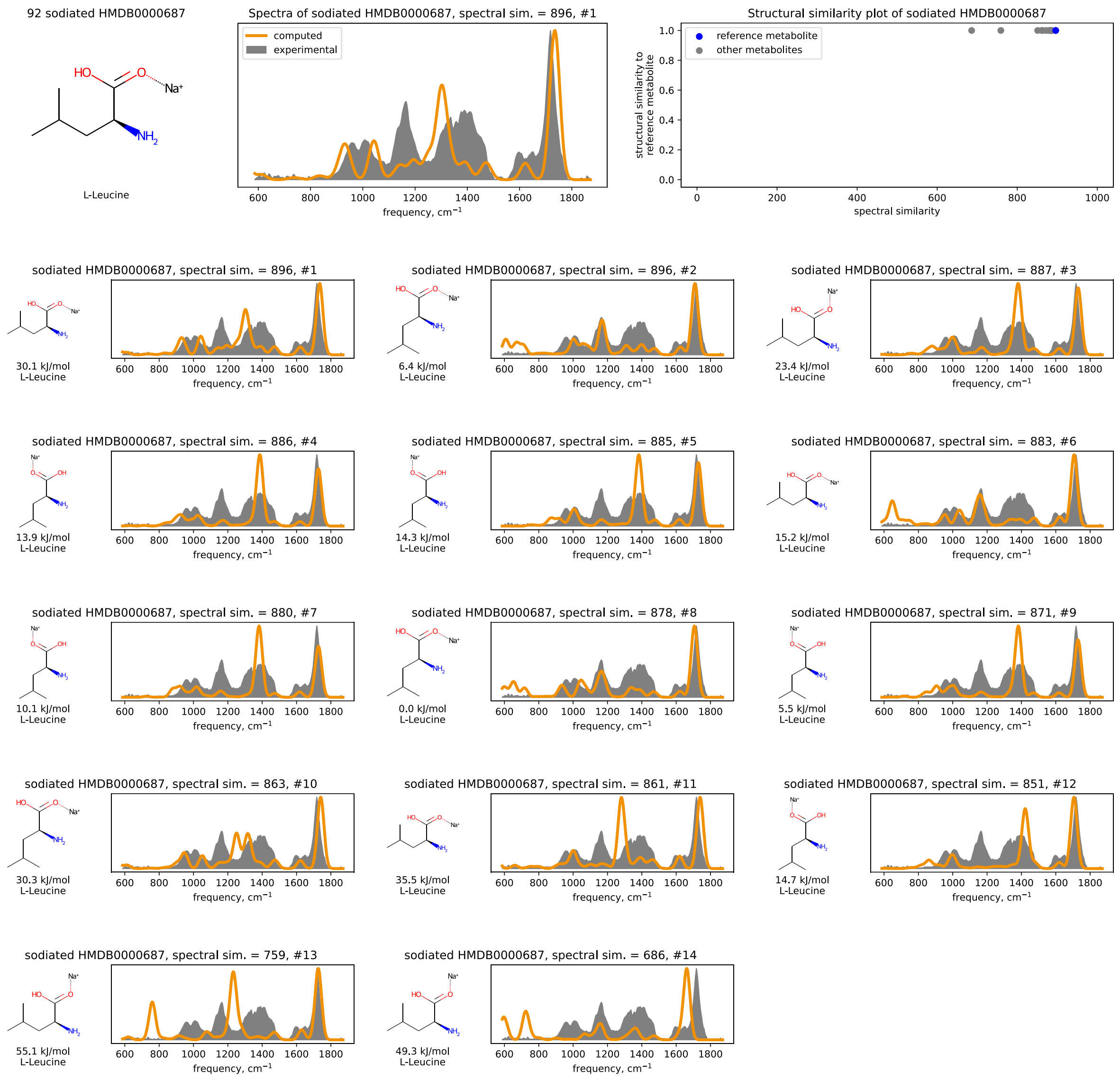

92 sodiated HMDB0000687
L-Leucine
Spectra of sodiated HMDB0000687, spectral sim. = 896, #1
computed
experimental
frequency, cm$^{-1}$
Structural similarity plot of sodiated HMDB0000687
reference metabolite
other metabolites
structural similarity to reference metabolite
spectral similarity
sodiated HMDB0000687, spectral sim. = 896, #1
30.1 kJ/mol L-Leucine
sodiated HMDB0000687, spectral sim. = 896, #2
6.4 kJ/mol L-Leucine
sodiated HMDB0000687, spectral sim. = 887, #3
23.4 kJ/mol L-Leucine
sodiated HMDB0000687, spectral sim. = 886, #4
13.9 kJ/mol L-Leucine
sodiated HMDB0000687, spectral sim. = 885, #5
14.3 kJ/mol L-Leucine
sodiated HMDB0000687, spectral sim. = 883, #6
15.2 kJ/mol L-Leucine
sodiated HMDB0000687, spectral sim. = 880, #7
10.1 kJ/mol L-Leucine
sodiated HMDB0000687, spectral sim. = 878, #8
0.0 kJ/mol L-Leucine
sodiated HMDB0000687, spectral sim. = 871, #9
5.5 kJ/mol L-Leucine
sodiated HMDB0000687, spectral sim. = 863, #10
30.3 kJ/mol L-Leucine
sodiated HMDB0000687, spectral sim. = 861, #11
35.5 kJ/mol L-Leucine
sodiated HMDB0000687, spectral sim. = 851, #12
14.7 kJ/mol L-Leucine
sodiated HMDB0000687, spectral sim. = 759, #13
55.1 kJ/mol L-Leucine
sodiated HMDB0000687, spectral sim. = 686, #14
49.3 kJ/mol L-Leucine


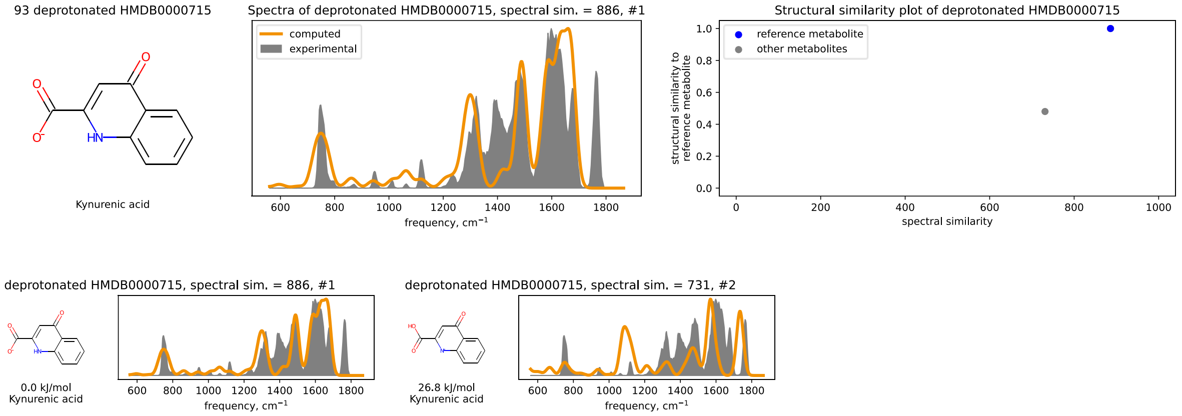

93 deprotonated HMDB0000715
Kynurenic acid
Spectra of deprotonated HMDB0000715, spectral sim. = 886, #1
computed
experimental
frequency, cm$^{-1}$
Structural similarity plot of deprotonated HMDB0000715
reference metabolite
other metabolites
structural similarity to reference metabolite
spectral similarity
deprotonated HMDB0000715, spectral sim. = 886, #1
0.0 kJ/mol Kynurenic acid
deprotonated HMDB0000715, spectral sim. = 731, #2
26.8 kJ/mol Kynurenic acid

94 protonated HMDB0000715

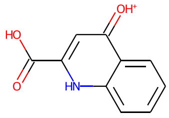


Kynurenic acid

Spectra of protonated HMDB0000715, spectral sim. = 842, #1

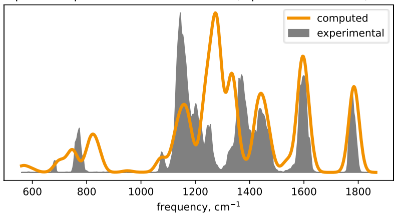


Structural similarity plot of protonated HMDB0000715

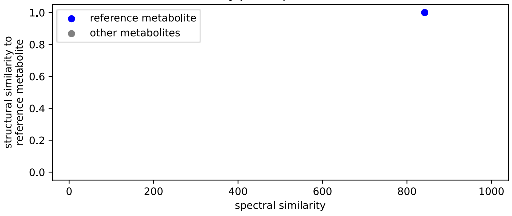


protonated HMDB0000715, spectral sim. = 842, #1

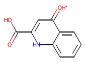

0.0 kJ/mol
Kynurenic acid

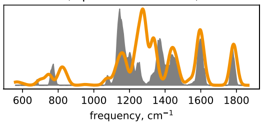


95 sodiated HMDB0000715

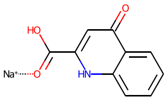


Kynurenic acid

Spectra of sodiated HMDB0000715, spectral sim. = 820, #1

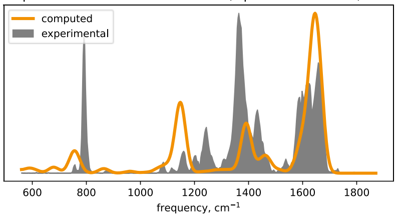


Structural similarity plot of sodiated HMDB0000715

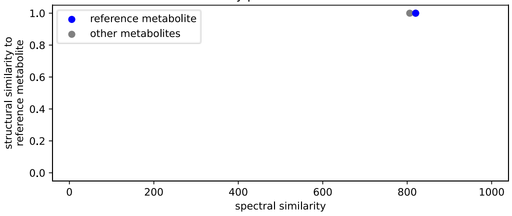


sodiated HMDB0000715, spectral sim. = 820, #1

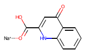

21.6 kJ/mol
Kynurenic acid

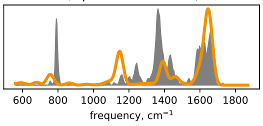


sodiated HMDB0000715, spectral sim. = 806, #2

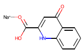

0.0 kJ/mol
Kynurenic acid

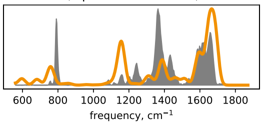

96 deprotonated HMDB0000759

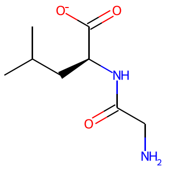

Glycylleucine

Spectra of deprotonated HMDB0000759, spectral sim. = 908, #1

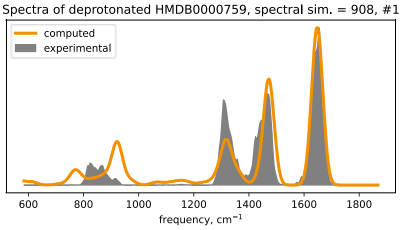

Structural similarity plot of deprotonated HMDB0000759

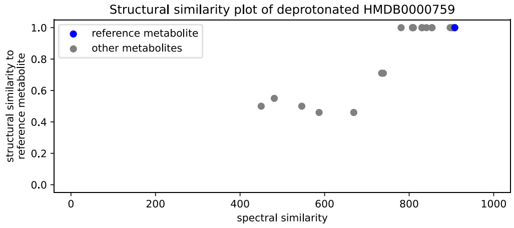

deprotonated HMDB0000759, spectral sim. = 908, #1

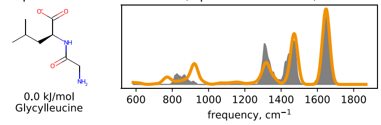

0.0 kJ/mol
Glycylleucine

deprotonated HMDB0000759, spectral sim. = 906, #2

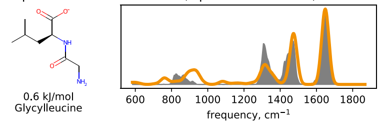

0.6 kJ/mol
Glycylleucine

deprotonated HMDB0000759, spectral sim. = 901, #3

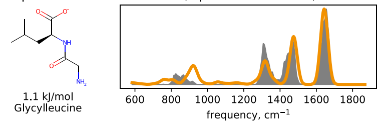

1.1 kJ/mol
Glycylleucine

deprotonated HMDB0000759, spectral sim. = 901, #4

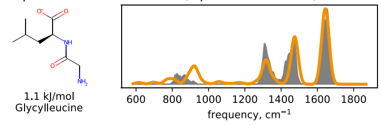

1.1 kJ/mol
Glycylleucine

deprotonated HMDB0000759, spectral sim. = 897, #5

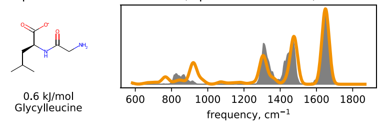

0.6 kJ/mol
Glycylleucine

deprotonated HMDB0000759, spectral sim. = 854, #6

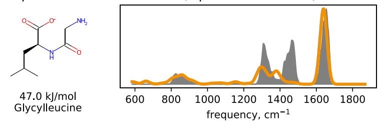

47.0 kJ/mol
Glycylleucine

deprotonated HMDB0000759, spectral sim. = 854, #7

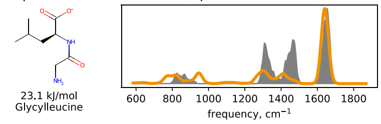

23.1 kJ/mol
Glycylleucine

deprotonated HMDB0000759, spectral sim. = 841, #8

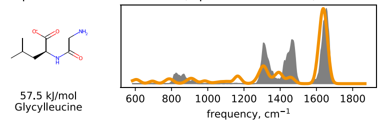

57.5 kJ/mol
Glycylleucine

deprotonated HMDB0000759, spectral sim. = 831, #9

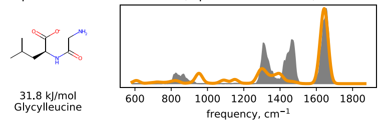

31.8 kJ/mol
Glycylleucine

deprotonated HMDB0000759, spectral sim. = 830, #10

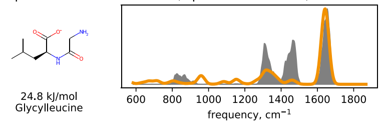

24.8 kJ/mol
Glycylleucine

deprotonated HMDB0000759, spectral sim. = 810, #11

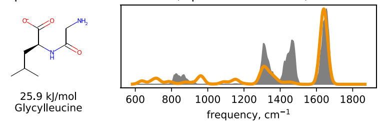

25.9 kJ/mol
Glycylleucine

deprotonated HMDB0000759, spectral sim. = 809, #12

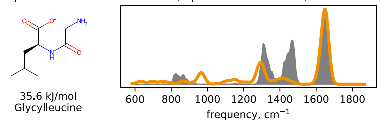

35.6 kJ/mol
Glycylleucine

deprotonated HMDB0000759, spectral sim. = 808, #13

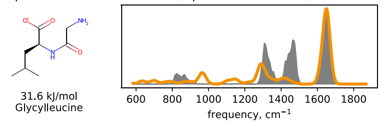

31.6 kJ/mol
Glycylleucine

deprotonated HMDB0000759, spectral sim. = 808, #14

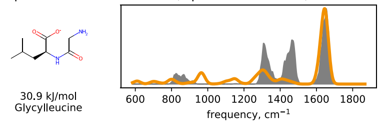

30.9 kJ/mol
Glycylleucine

deprotonated HMDB0000759, spectral sim. = 781, #15

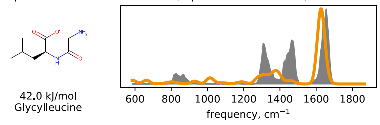

42.0 kJ/mol
Glycylleucine

deprotonated HMDB0000759, spectral sim. = 739, #16

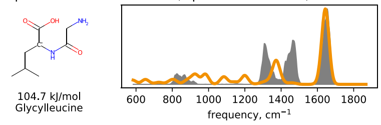

104.7 kJ/mol
Glycylleucine

deprotonated HMDB0000759, spectral sim. = 735, #17

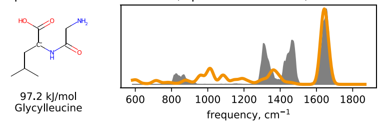

97.2 kJ/mol
Glycylleucine

deprotonated HMDB0000759, spectral sim. = 669, #18

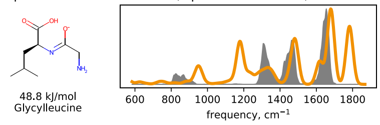

48.8 kJ/mol
Glycylleucine

deprotonated HMDB0000759, spectral sim. = 587, #19

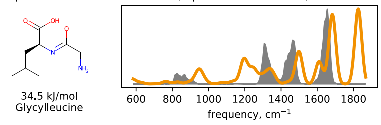

34.5 kJ/mol
Glycylleucine

deprotonated HMDB0000759, spectral sim. = 546, #20

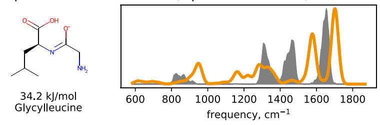

34.2 kJ/mol
Glycylleucine

deprotonated HMDB0000759, spectral sim. = 481, #21

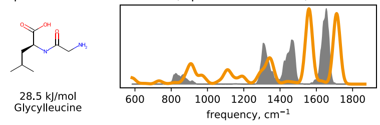

28.5 kJ/mol
Glycylleucine

deprotonated HMDB0000759, spectral sim. = 450, #22

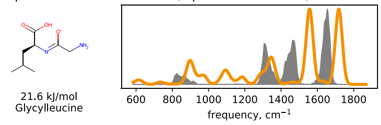

21.6 kJ/mol
Glycylleucine

97 protonated HMDB0000759

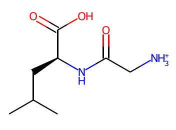


Glycylleucine

Spectra of protonated HMDB0000759, spectral sim. = 920, #1

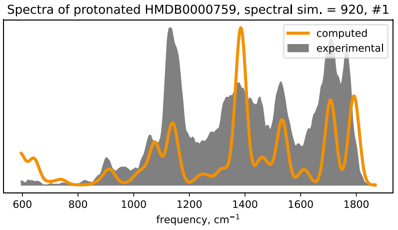


Structural similarity plot of protonated HMDB0000759

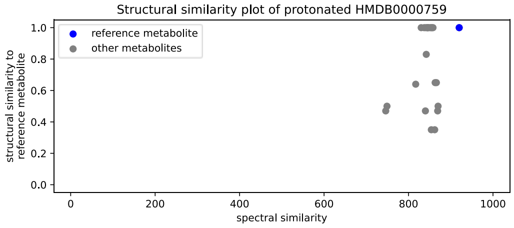


protonated HMDB0000759, spectral sim. = 920, #1

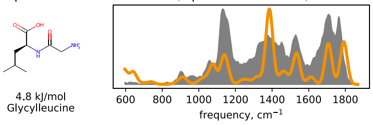


protonated HMDB0000759, spectral sim. = 870, #2

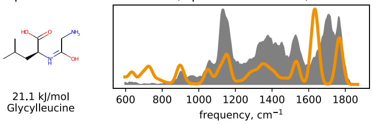


protonated HMDB0000759, spectral sim. = 869, #3

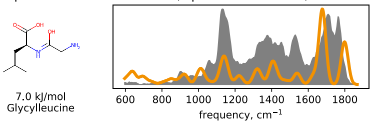


protonated HMDB0000759, spectral sim. = 866, #4

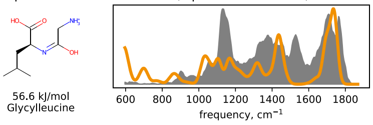


protonated HMDB0000759, spectral sim. = 863, #5

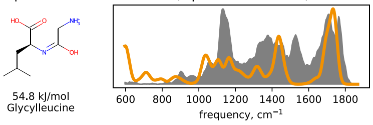


protonated HMDB0000759, spectral sim. = 862, #6

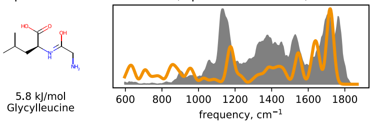


protonated HMDB0000759, spectral sim. = 858, #7

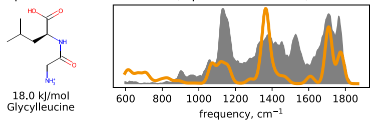


protonated HMDB0000759, spectral sim. = 856, #8

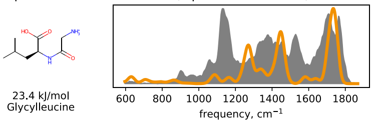


protonated HMDB0000759, spectral sim. = 854, #9

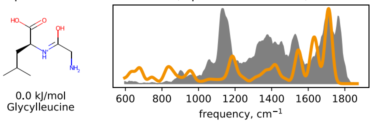


protonated HMDB0000759, spectral sim. = 854, #10

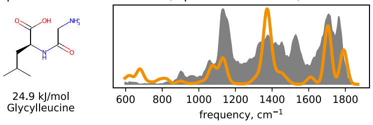


protonated HMDB0000759, spectral sim. = 852, #11

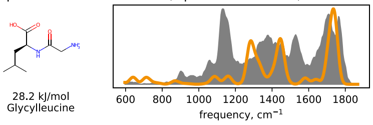


protonated HMDB0000759, spectral sim. = 848, #12

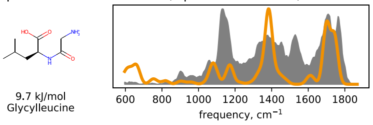


protonated HMDB0000759, spectral sim. = 845, #13

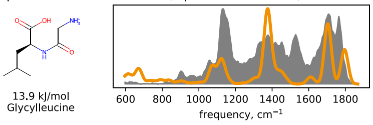


protonated HMDB0000759, spectral sim. = 845, #14

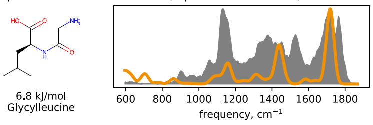


protonated HMDB0000759, spectral sim. = 845, #15

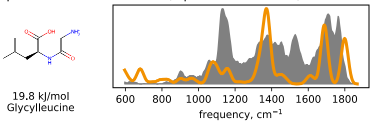


protonated HMDB0000759, spectral sim. = 842, #16

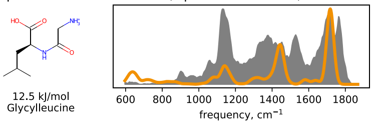


protonated HMDB0000759, spectral sim. = 841, #17

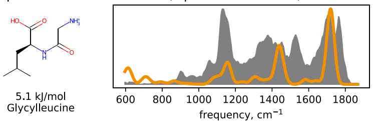


protonated HMDB0000759, spectral sim. = 840, #18

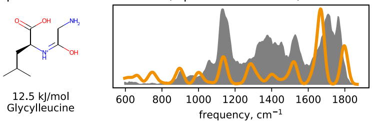


protonated HMDB0000759, spectral sim. = 837, #19

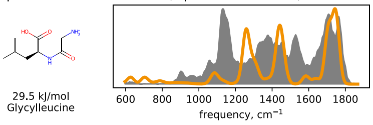


protonated HMDB0000759, spectral sim. = 830, #20

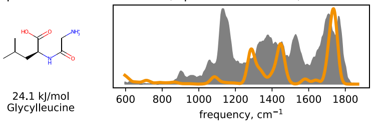


protonated HMDB0000759, spectral sim. = 817, #21

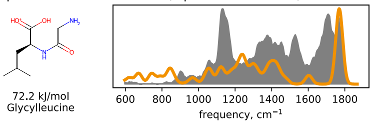


protonated HMDB0000759, spectral sim. = 749, #22

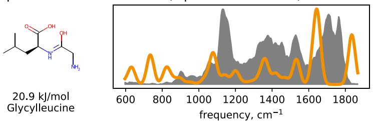


protonated HMDB0000759, spectral sim. = 746, #23

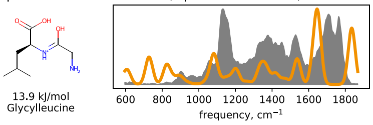

98 deprotonated HMDB0000765

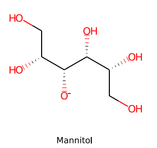


Spectra of deprotonated HMDB0000765, spectral sim. = 960, #1

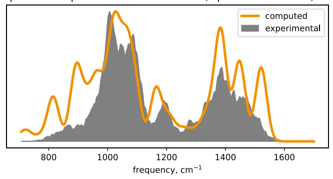


Structural similarity plot of deprotonated HMDB0000765

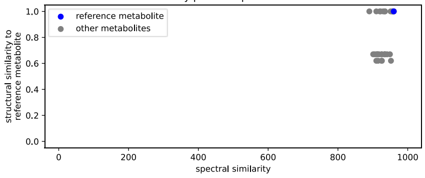


deprotonated HMDB0000765, spectral sim. = 960, #1

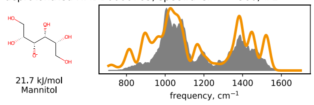


deprotonated HMDB0000765, spectral sim. = 960, #2

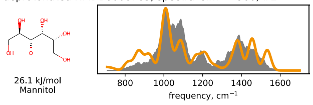


deprotonated HMDB0000765, spectral sim. = 956, #3

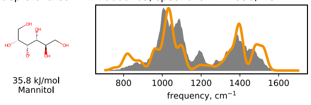


deprotonated HMDB0000765, spectral sim. = 956, #4

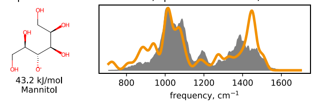


deprotonated HMDB0000765, spectral sim. = 956, #5

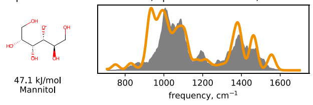


deprotonated HMDB0000765, spectral sim. = 952, #6

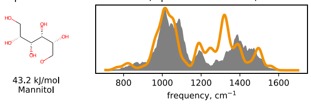


deprotonated HMDB0000765, spectral sim. = 951, #7

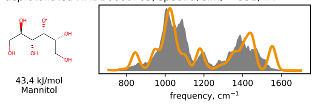


deprotonated HMDB0000765, spectral sim. = 949, #8

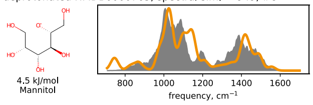


deprotonated HMDB0000765, spectral sim. = 941, #9

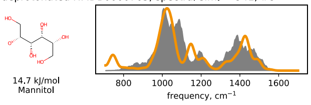


deprotonated HMDB0000765, spectral sim. = 936, #10

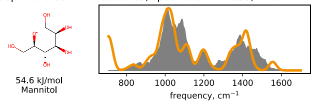


deprotonated HMDB0000765, spectral sim. = 935, #11

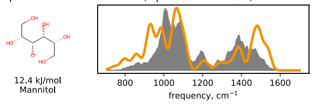


deprotonated HMDB0000765, spectral sim. = 934, #12

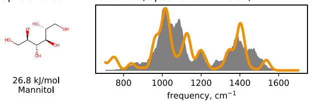


deprotonated HMDB0000765, spectral sim. = 934, #13

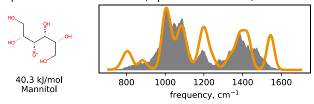


deprotonated HMDB0000765, spectral sim. = 933, #14

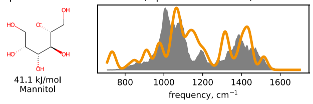


deprotonated HMDB0000765, spectral sim. = 930, #15

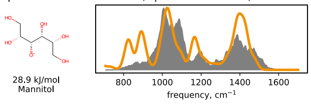


deprotonated HMDB0000765, spectral sim. = 926, #16

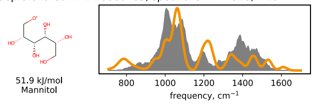


deprotonated HMDB0000765, spectral sim. = 926, #17

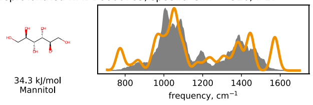


deprotonated HMDB0000765, spectral sim. = 925, #18

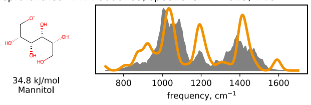


deprotonated HMDB0000765, spectral sim. = 925, #19

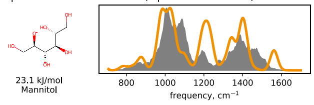


deprotonated HMDB0000765, spectral sim. = 922, #20

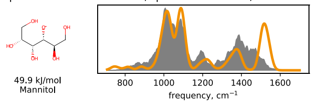


deprotonated HMDB0000765, spectral sim. = 920, #21

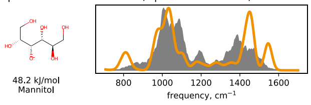


deprotonated HMDB0000765, spectral sim. = 918, #22

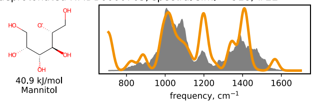


deprotonated HMDB0000765, spectral sim. = 915, #23

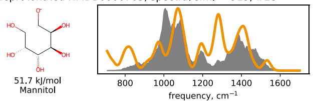


deprotonated HMDB0000765, spectral sim. = 914, #24

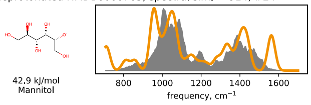


deprotonated HMDB0000765, spectral sim. = 913, #25

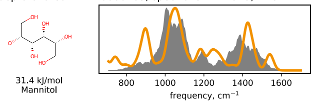


deprotonated HMDB0000765, spectral sim. = 910, #26

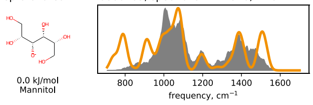


deprotonated HMDB0000765, spectral sim. = 910, #27

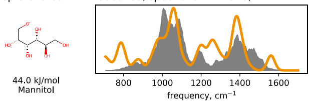


deprotonated HMDB0000765, spectral sim. = 910, #28

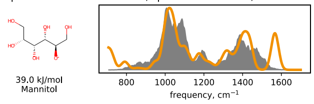


deprotonated HMDB0000765, spectral sim. = 905, #29

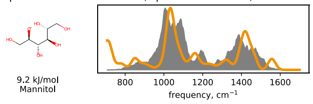


deprotonated HMDB0000765, spectral sim. = 901, #30

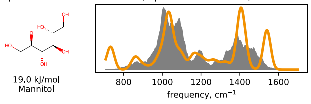


deprotonated HMDB0000765, spectral sim. = 890, #31

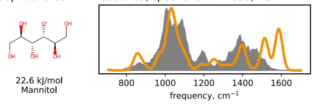

99 deprotonated HMDB0000821

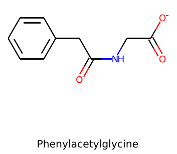


Spectra of deprotonated HMDB0000821, spectral sim. = 914, #1

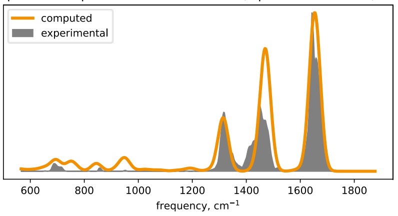


Structural similarity plot of deprotonated HMDB0000821

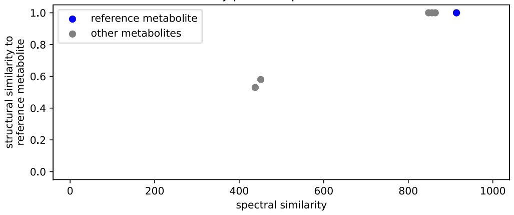


deprotonated HMDB0000821, spectral sim. = 914, #1

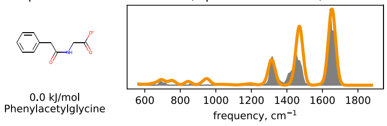


deprotonated HMDB0000821, spectral sim. = 864, #2

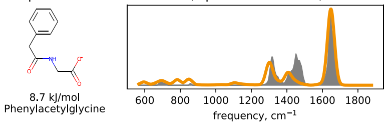


deprotonated HMDB0000821, spectral sim. = 856, #3

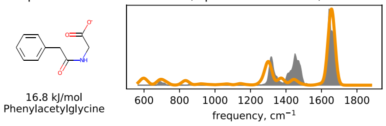


deprotonated HMDB0000821, spectral sim. = 848, #4

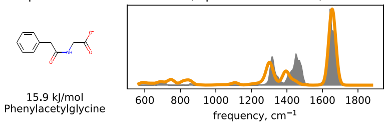


deprotonated HMDB0000821, spectral sim. = 451, #5

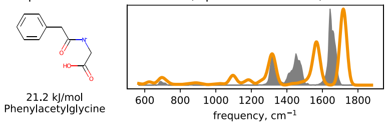


deprotonated HMDB0000821, spectral sim. = 438, #6

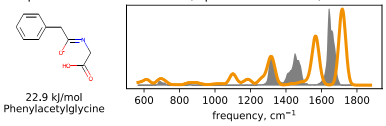


100 protonated HMDB0000821

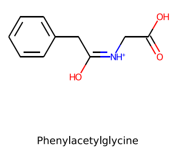


Spectra of protonated HMDB0000821, spectral sim. = 913, #1

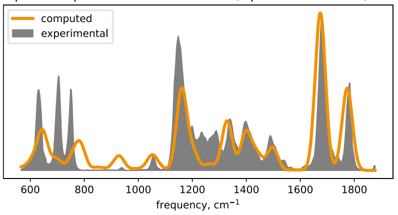


Structural similarity plot of protonated HMDB0000821

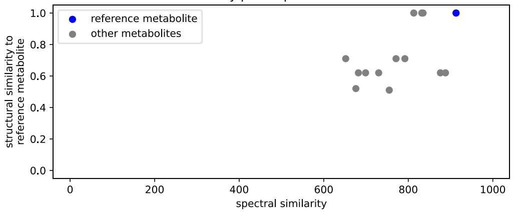


protonated HMDB0000821, spectral sim. = 913, #1

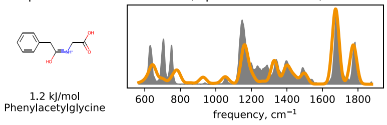


protonated HMDB0000821, spectral sim. = 888, #2

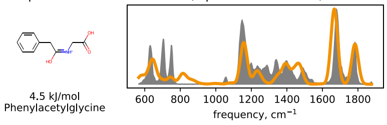


protonated HMDB0000821, spectral sim. = 876, #3

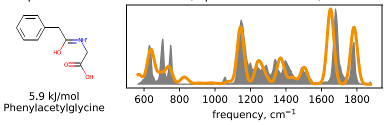


protonated HMDB0000821, spectral sim. = 835, #4

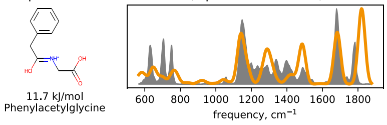


protonated HMDB0000821, spectral sim. = 833, #5

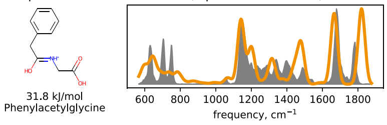


protonated HMDB0000821, spectral sim. = 832, #6

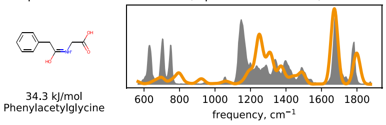


protonated HMDB0000821, spectral sim. = 813, #7

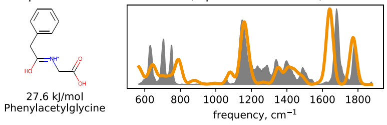


protonated HMDB0000821, spectral sim. = 792, #8

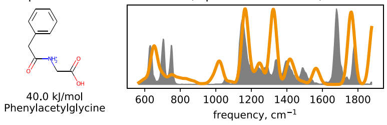


protonated HMDB0000821, spectral sim. = 771, #9

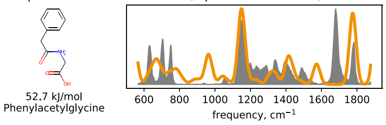


protonated HMDB0000821, spectral sim. = 755, #10

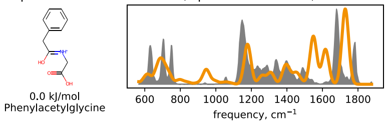


protonated HMDB0000821, spectral sim. = 730, #11

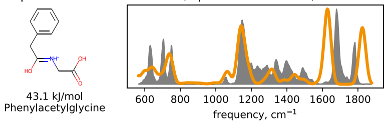


protonated HMDB0000821, spectral sim. = 699, #12

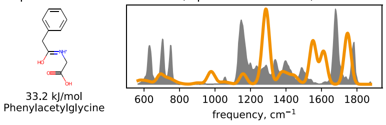


protonated HMDB0000821, spectral sim. = 682, #13

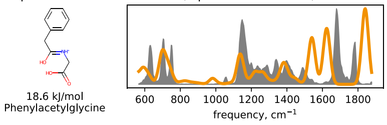


protonated HMDB0000821, spectral sim. = 676, #14

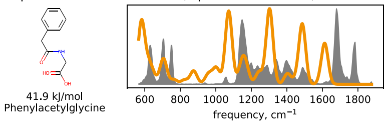


protonated HMDB0000821, spectral sim. = 652, #15

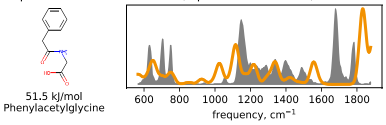

101 sodiated HMDB0000821

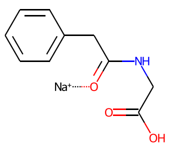


Phenylacetylglycine

Spectra of sodiated HMDB0000821, spectral sim. = 896, #1

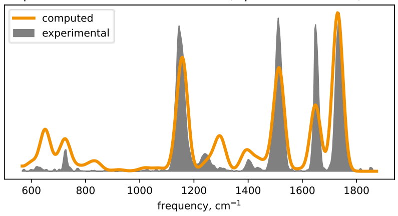


Structural similarity plot of sodiated HMDB0000821

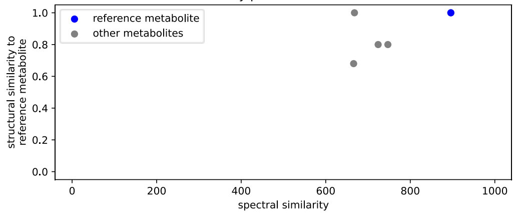


sodiated HMDB0000821, spectral sim. = 896, #1

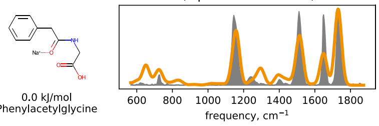

0.0 kJ/mol
Phenylacetylglycine

sodiated HMDB0000821, spectral sim. = 747, #2

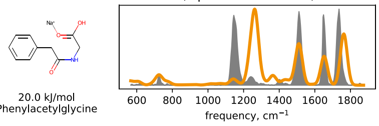

20.0 kJ/mol
Phenylacetylglycine

sodiated HMDB0000821, spectral sim. = 724, #3

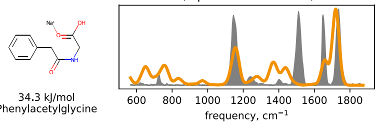

34.3 kJ/mol
Phenylacetylglycine

sodiated HMDB0000821, spectral sim. = 668, #4

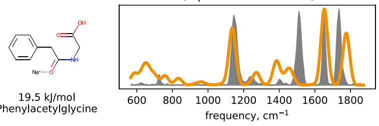

19.5 kJ/mol
Phenylacetylglycine

sodiated HMDB0000821, spectral sim. = 666, #5

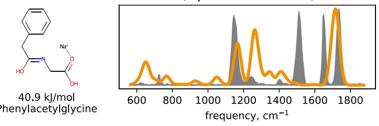

40.9 kJ/mol
Phenylacetylglycine

102 deprotonated HMDB0000822

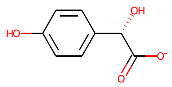


p-Hydroxymandelic acid

Spectra of deprotonated HMDB0000822, spectral sim. = 879, #1

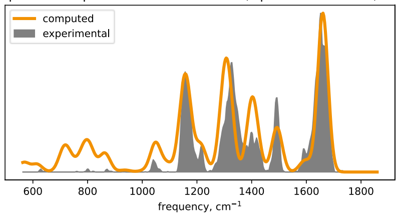


Structural similarity plot of deprotonated HMDB0000822

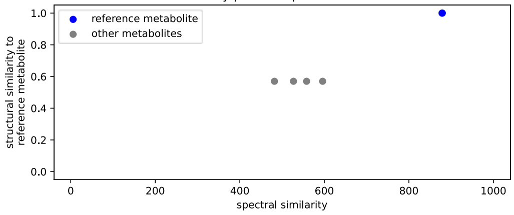


deprotonated HMDB0000822, spectral sim. = 879, #1

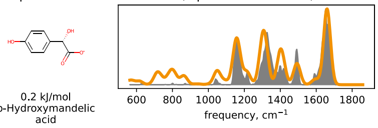

0.2 kJ/mol
p-Hydroxymandelic acid

deprotonated HMDB0000822, spectral sim. = 878, #2

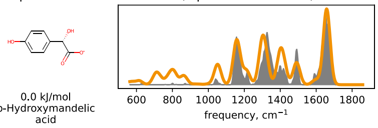

0.0 kJ/mol
p-Hydroxymandelic acid

deprotonated HMDB0000822, spectral sim. = 596, #3

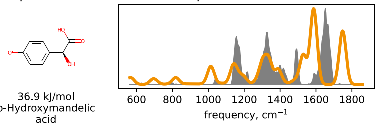

36.9 kJ/mol
p-Hydroxymandelic acid

deprotonated HMDB0000822, spectral sim. = 558, #4

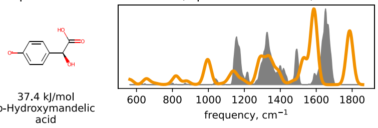

37.4 kJ/mol
p-Hydroxymandelic acid

deprotonated HMDB0000822, spectral sim. = 527, #5

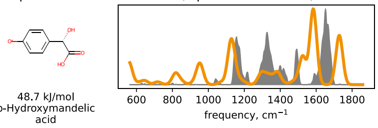

48.7 kJ/mol
p-Hydroxymandelic acid

deprotonated HMDB0000822, spectral sim. = 482, #6

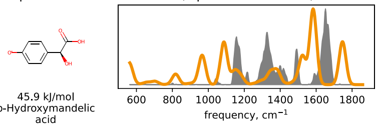

45.9 kJ/mol
p-Hydroxymandelic acid

103 sodiated HMDB0000822

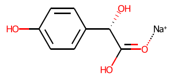


p-Hydroxymandelic acid

Spectra of sodiated HMDB0000822, spectral sim. = 850, #1

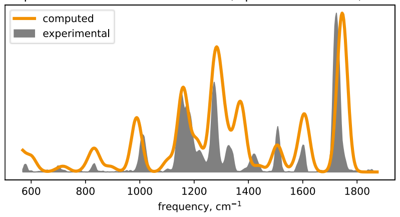


Structural similarity plot of sodiated HMDB0000822

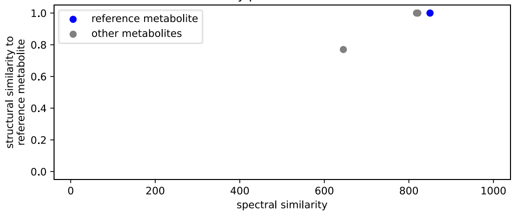


sodiated HMDB0000822, spectral sim. = 850, #1

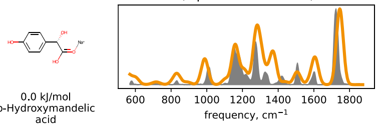

0.0 kJ/mol
p-Hydroxymandelic acid

sodiated HMDB0000822, spectral sim. = 821, #2

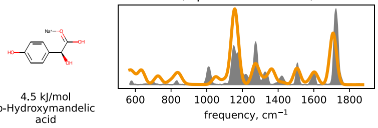

4.5 kJ/mol
p-Hydroxymandelic acid

sodiated HMDB0000822, spectral sim. = 821, #3

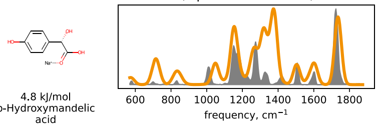

4.8 kJ/mol
p-Hydroxymandelic acid

sodiated HMDB0000822, spectral sim. = 818, #4

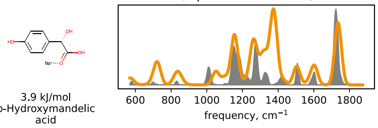

3.9 kJ/mol
p-Hydroxymandelic acid

sodiated HMDB0000822, spectral sim. = 645, #5

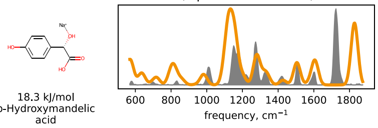

18.3 kJ/mol
p-Hydroxymandelic acid

104 deprotonated HMDB0000828

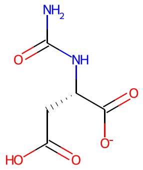


Ureidosuccinic acid

Spectra of deprotonated HMDB0000828, spectral sim. = 896, #1

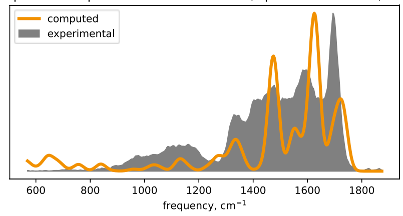


Structural similarity plot of deprotonated HMDB0000828

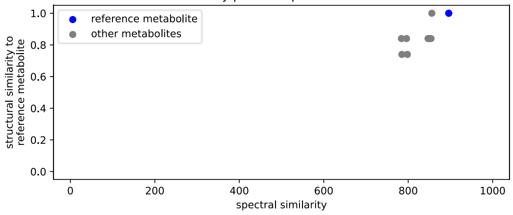


deprotonated HMDB0000828, spectral sim. = 896, #1

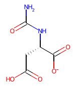

5.4 kJ/mol
Ureidosuccinic acid

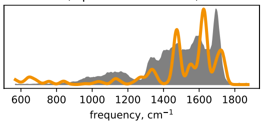


deprotonated HMDB0000828, spectral sim. = 856, #2

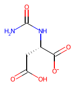

20.3 kJ/mol
Ureidosuccinic acid

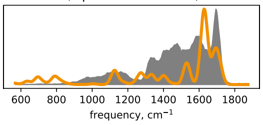


deprotonated HMDB0000828, spectral sim. = 854, #3

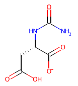

0.0 kJ/mol
Ureidosuccinic acid

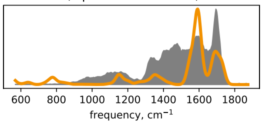


deprotonated HMDB0000828, spectral sim. = 851, #4

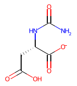

10.7 kJ/mol
Ureidosuccinic acid

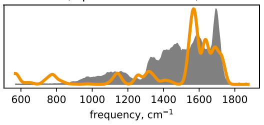


deprotonated HMDB0000828, spectral sim. = 847, #5

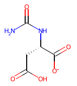

1.3 kJ/mol
Ureidosuccinic acid

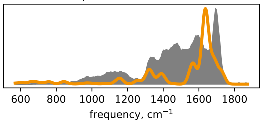


deprotonated HMDB0000828, spectral sim. = 798, #6

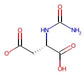

50.9 kJ/mol
Ureidosuccinic acid

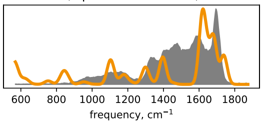


deprotonated HMDB0000828, spectral sim. = 796, #7

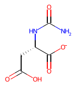

52.9 kJ/mol
Ureidosuccinic acid

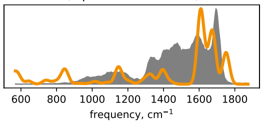


deprotonated HMDB0000828, spectral sim. = 785, #8

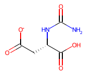

55.0 kJ/mol
Ureidosuccinic acid

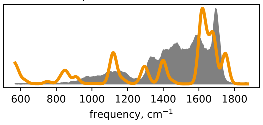


deprotonated HMDB0000828, spectral sim. = 784, #9

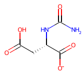

35.6 kJ/mol
Ureidosuccinic acid

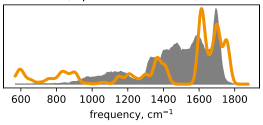

105 protonated HMDB0000828

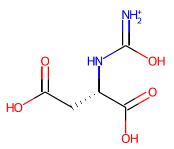


Ureidosuccinic acid

Spectra of protonated HMDB0000828, spectral sim. = 946, #1

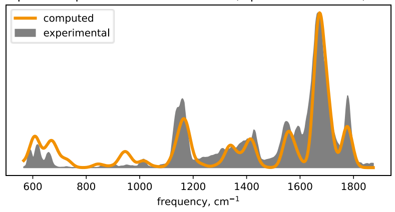


Structural similarity plot of protonated HMDB0000828

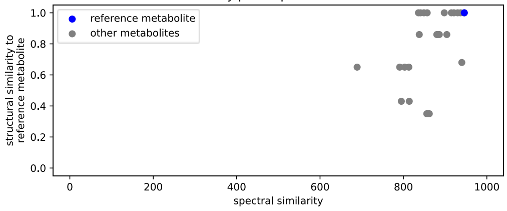


protonated HMDB0000828, spectral sim. = 946, #1

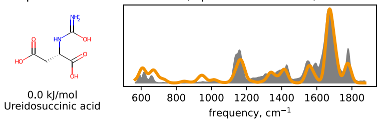


protonated HMDB0000828, spectral sim. = 940, #2

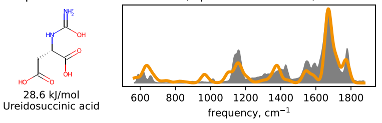


protonated HMDB0000828, spectral sim. = 937, #3

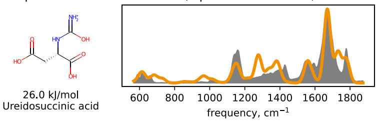


protonated HMDB0000828, spectral sim. = 930, #4

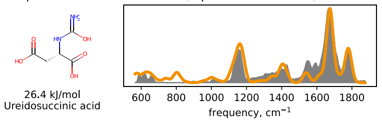


protonated HMDB0000828, spectral sim. = 921, #5

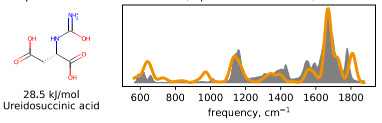


protonated HMDB0000828, spectral sim. = 915, #6

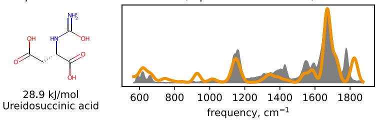


protonated HMDB0000828, spectral sim. = 904, #7

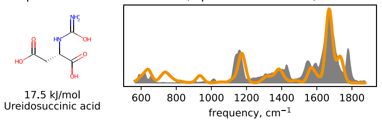


protonated HMDB0000828, spectral sim. = 898, #8

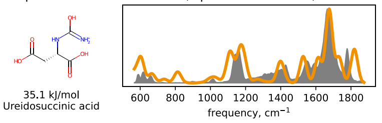


protonated HMDB0000828, spectral sim. = 886, #9

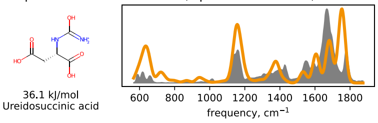


protonated HMDB0000828, spectral sim. = 882, #10

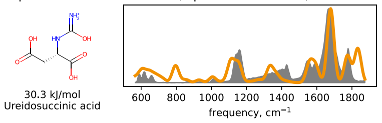


protonated HMDB0000828, spectral sim. = 880, #11

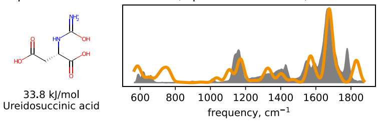


protonated HMDB0000828, spectral sim. = 862, #12

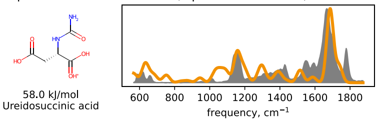


protonated HMDB0000828, spectral sim. = 859, #13

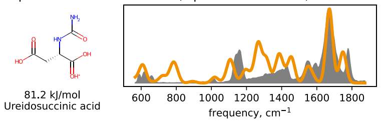


protonated HMDB0000828, spectral sim. = 857, #14

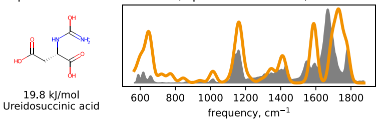


protonated HMDB0000828, spectral sim. = 856, #15

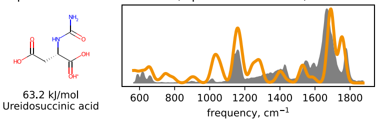


protonated HMDB0000828, spectral sim. = 849, #16

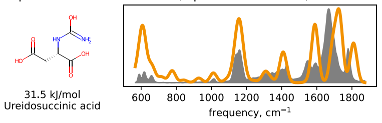


protonated HMDB0000828, spectral sim. = 841, #17

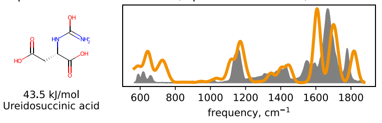


protonated HMDB0000828, spectral sim. = 838, #18

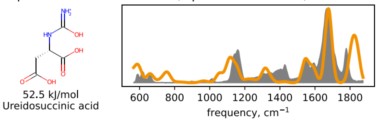


protonated HMDB0000828, spectral sim. = 836, #19

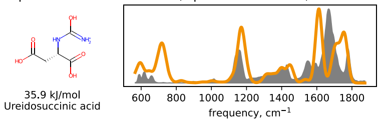


protonated HMDB0000828, spectral sim. = 814, #20

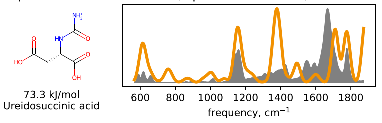


protonated HMDB0000828, spectral sim. = 813, #21

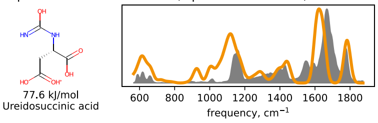


protonated HMDB0000828, spectral sim. = 803, #22

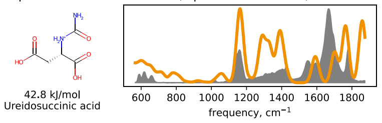


protonated HMDB0000828, spectral sim. = 795, #23

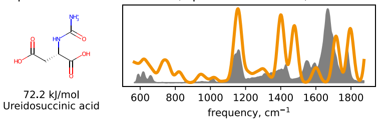


protonated HMDB0000828, spectral sim. = 791, #24

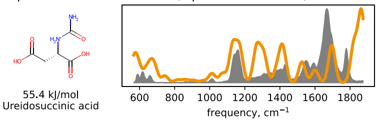


protonated HMDB0000828, spectral sim. = 689, #25

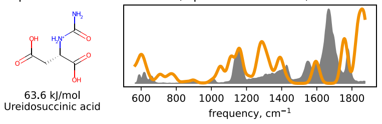

106 sodiated HMDB0000828

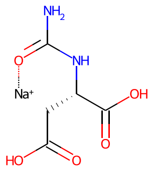


Ureidosuccinic acid

Spectra of sodiated HMDB0000828, spectral sim. = 935, #1

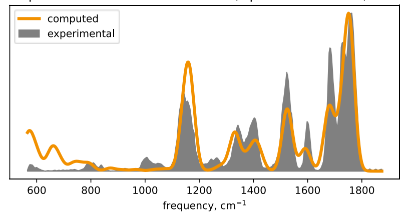


Structural similarity plot of sodiated HMDB0000828

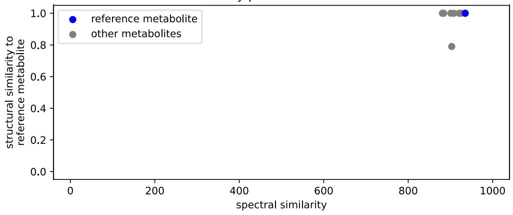


sodiated HMDB0000828, spectral sim. = 935, #1

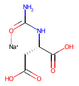

32.7 kJ/mol
Ureidosuccinic acid

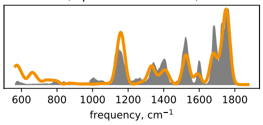

sodiated HMDB0000828, spectral sim. = 925, #2

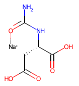

31.5 kJ/mol
Ureidosuccinic acid

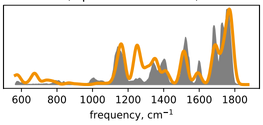

sodiated HMDB0000828, spectral sim. = 921, #3

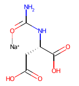

16.0 kJ/mol
Ureidosuccinic acid

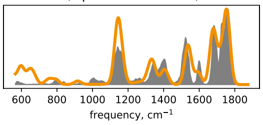

sodiated HMDB0000828, spectral sim. = 908, #4

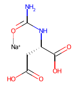

30.4 kJ/mol
Ureidosuccinic acid

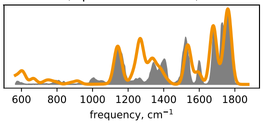

sodiated HMDB0000828, spectral sim. = 903, #5

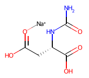

60.3 kJ/mol
Ureidosuccinic acid

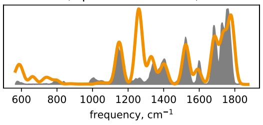

sodiated HMDB0000828, spectral sim. = 901, #6

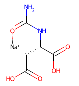

42.5 kJ/mol
Ureidosuccinic acid

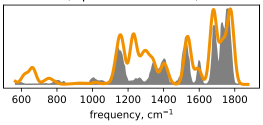

sodiated HMDB0000828, spectral sim. = 884, #7

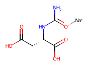

0.0 kJ/mol
Ureidosuccinic acid

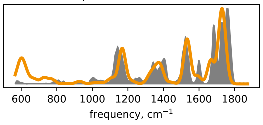

sodiated HMDB0000828, spectral sim. = 881, #8

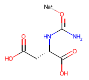

0.2 kJ/mol
Ureidosuccinic acid

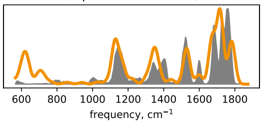

107 deprotonated HMDB0000842

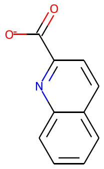


Quinaldic acid

Spectra of deprotonated HMDB0000842, spectral sim. = 889, #1

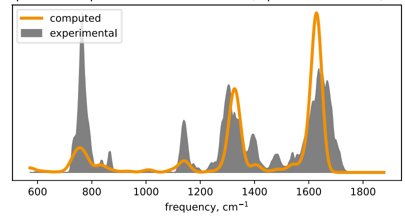


Structural similarity plot of deprotonated HMDB0000842

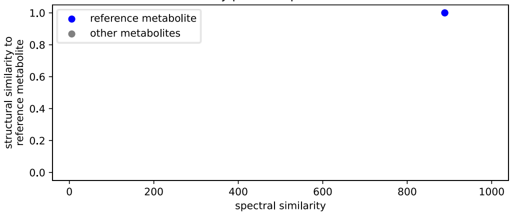


deprotonated HMDB0000842, spectral sim. = 889, #1

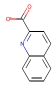

0.0 kJ/mol
Quinaldic acid

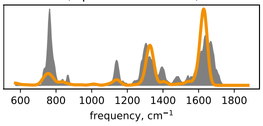

108 protonated HMDB0000842

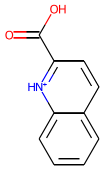


Quinaldic acid

Spectra of protonated HMDB0000842, spectral sim. = 900, #1

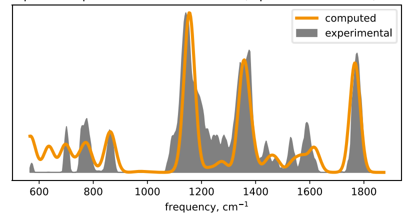


Structural similarity plot of protonated HMDB0000842

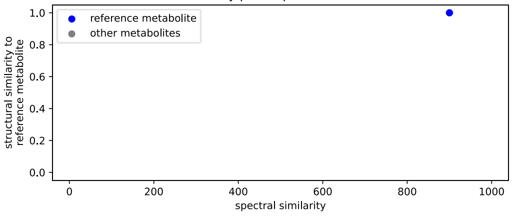


protonated HMDB0000842, spectral sim. = 900, #1

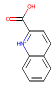

0.0 kJ/mol
Quinaldic acid

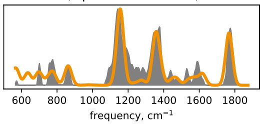

109 sodiated HMDB0000842

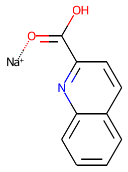


Quinaldic acid

Spectra of sodiated HMDB0000842, spectral sim. = 818, #1

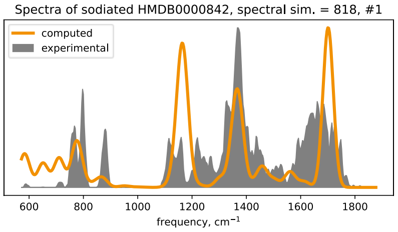


Structural similarity plot of sodiated HMDB0000842

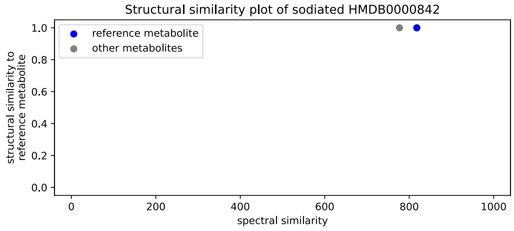


sodiated HMDB0000842, spectral sim. = 818, #1

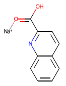

0.0 kJ/mol
Quinaldic acid

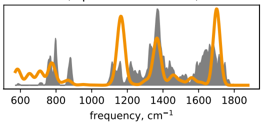


sodiated HMDB0000842, spectral sim. = 777, #2

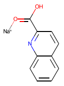

30.0 kJ/mol
Quinaldic acid

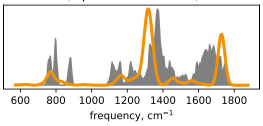


110 deprotonated HMDB0000860

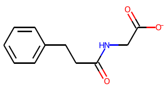


Phenylpropionylglycine

Spectra of deprotonated HMDB0000860, spectral sim. = 910, #1

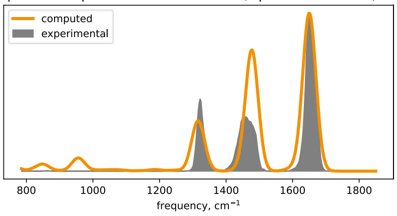


Structural similarity plot of deprotonated HMDB0000860

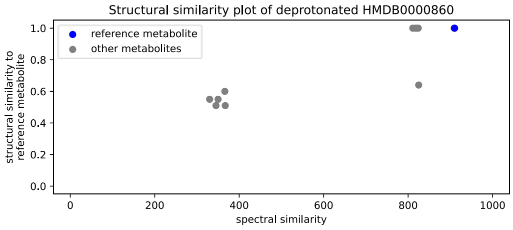


deprotonated HMDB0000860, spectral sim. = 910, #1

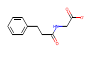

0.0 kJ/mol
Phenylpropionylglycine

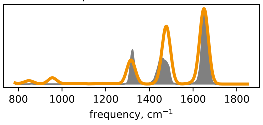


deprotonated HMDB0000860, spectral sim. = 909, #2

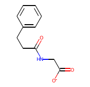

0.0 kJ/mol
Phenylpropionylglycine

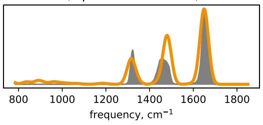


deprotonated HMDB0000860, spectral sim. = 825, #3

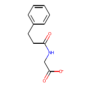

8.7 kJ/mol
Phenylpropionylglycine

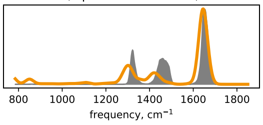


deprotonated HMDB0000860, spectral sim. = 824, #4

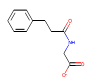

6.5 kJ/mol
Phenylpropionylglycine

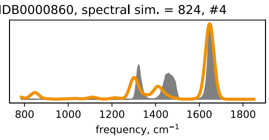


deprotonated HMDB0000860, spectral sim. = 819, #5

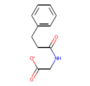

31.5 kJ/mol
Phenylpropionylglycine

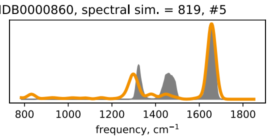


deprotonated HMDB0000860, spectral sim. = 818, #6

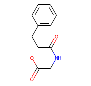

20.1 kJ/mol
Phenylpropionylglycine

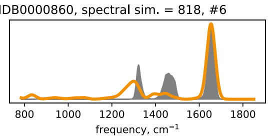


deprotonated HMDB0000860, spectral sim. = 811, #7

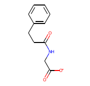

19.2 kJ/mol
Phenylpropionylglycine

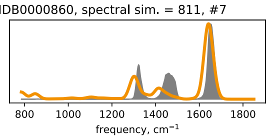


deprotonated HMDB0000860, spectral sim. = 367, #8

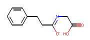

24.3 kJ/mol
Phenylpropionylglycine

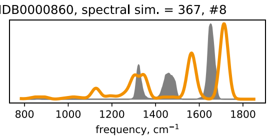


deprotonated HMDB0000860, spectral sim. = 366, #9

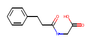

23.9 kJ/mol
Phenylpropionylglycine

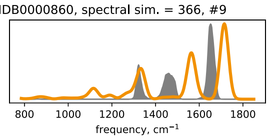


deprotonated HMDB0000860, spectral sim. = 350, #10

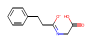

31.3 kJ/mol
Phenylpropionylglycine

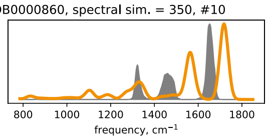


deprotonated HMDB0000860, spectral sim. = 345, #11

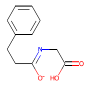

25.1 kJ/mol
Phenylpropionylglycine

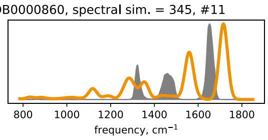


deprotonated HMDB0000860, spectral sim. = 330, #12

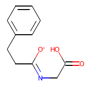

21.7 kJ/mol
Phenylpropionylglycine

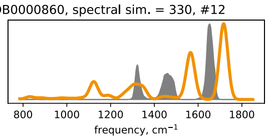

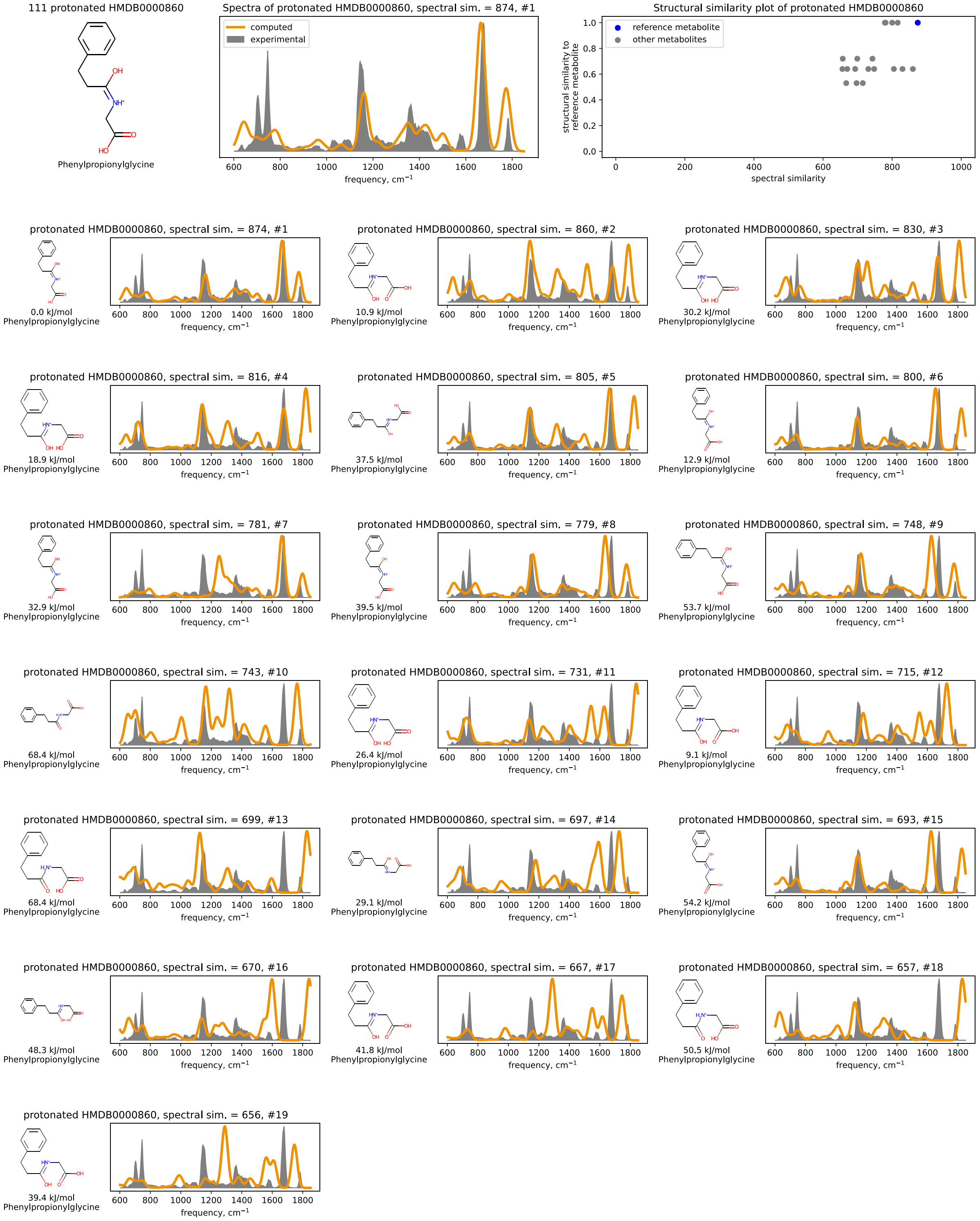

111 protonated HMDB0000860
Phenylpropionylglycine
Spectra of protonated HMDB0000860, spectral sim. = 874, #1
computed
experimental
frequency, cm$^{-1}$
Structural similarity plot of protonated HMDB0000860
reference metabolite
other metabolites
structural similarity to reference metabolite
spectral similarity
protonated HMDB0000860, spectral sim. = 874, #1
0.0 kJ/mol
Phenylpropionylglycine
protonated HMDB0000860, spectral sim. = 860, #2
10.9 kJ/mol
Phenylpropionylglycine
protonated HMDB0000860, spectral sim. = 830, #3
30.2 kJ/mol
Phenylpropionylglycine
protonated HMDB0000860, spectral sim. = 816, #4
18.9 kJ/mol
Phenylpropionylglycine
protonated HMDB0000860, spectral sim. = 805, #5
37.5 kJ/mol
Phenylpropionylglycine
protonated HMDB0000860, spectral sim. = 800, #6
12.9 kJ/mol
Phenylpropionylglycine
protonated HMDB0000860, spectral sim. = 781, #7
32.9 kJ/mol
Phenylpropionylglycine
protonated HMDB0000860, spectral sim. = 779, #8
39.5 kJ/mol
Phenylpropionylglycine
protonated HMDB0000860, spectral sim. = 748, #9
53.7 kJ/mol
Phenylpropionylglycine
protonated HMDB0000860, spectral sim. = 743, #10
68.4 kJ/mol
Phenylpropionylglycine
protonated HMDB0000860, spectral sim. = 731, #11
26.4 kJ/mol
Phenylpropionylglycine
protonated HMDB0000860, spectral sim. = 715, #12
9.1 kJ/mol
Phenylpropionylglycine
protonated HMDB0000860, spectral sim. = 699, #13
68.4 kJ/mol
Phenylpropionylglycine
protonated HMDB0000860, spectral sim. = 697, #14
29.1 kJ/mol
Phenylpropionylglycine
protonated HMDB0000860, spectral sim. = 693, #15
54.2 kJ/mol
Phenylpropionylglycine
protonated HMDB0000860, spectral sim. = 670, #16
48.3 kJ/mol
Phenylpropionylglycine
protonated HMDB0000860, spectral sim. = 667, #17
41.8 kJ/mol
Phenylpropionylglycine
protonated HMDB0000860, spectral sim. = 657, #18
50.5 kJ/mol
Phenylpropionylglycine
protonated HMDB0000860, spectral sim. = 656, #19
39.4 kJ/mol
Phenylpropionylglycine

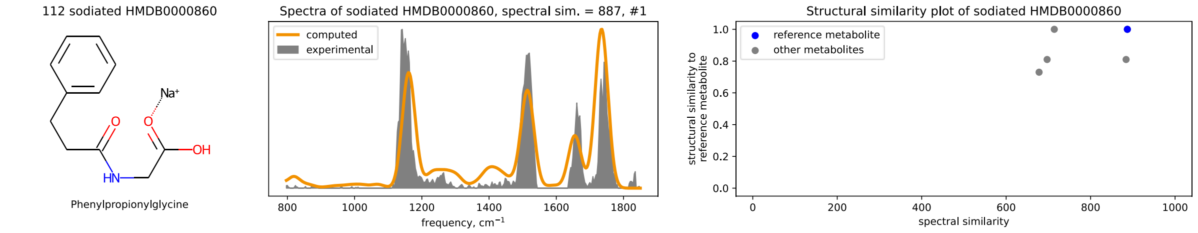

112 sodiated HMDB0000860
Na+
O
O
OH
HN
Phenylpropionylglycine
Spectra of sodiated HMDB0000860, spectral sim. = 887, #1
computed
experimental
frequency, cm−1
Structural similarity plot of sodiated HMDB0000860
reference metabolite
other metabolites
structural similarity to reference metabolite
spectral similarity


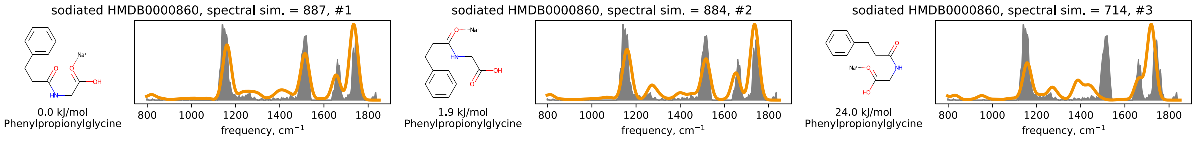

sodiated HMDB0000860, spectral sim. = 887, #1
0.0 kJ/mol
Phenylpropionylglycine
frequency, cm−1
sodiated HMDB0000860, spectral sim. = 884, #2
1.9 kJ/mol
Phenylpropionylglycine
frequency, cm−1
sodiated HMDB0000860, spectral sim. = 714, #3
24.0 kJ/mol
Phenylpropionylglycine
frequency, cm−1


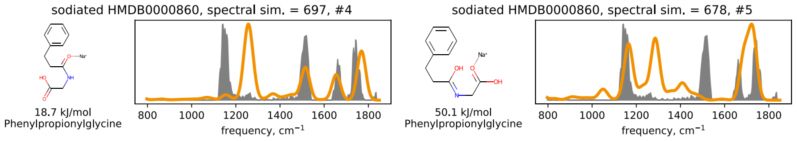

sodiated HMDB0000860, spectral sim. = 697, #4
18.7 kJ/mol
Phenylpropionylglycine
frequency, cm−1
sodiated HMDB0000860, spectral sim. = 678, #5
50.1 kJ/mol
Phenylpropionylglycine
frequency, cm−1


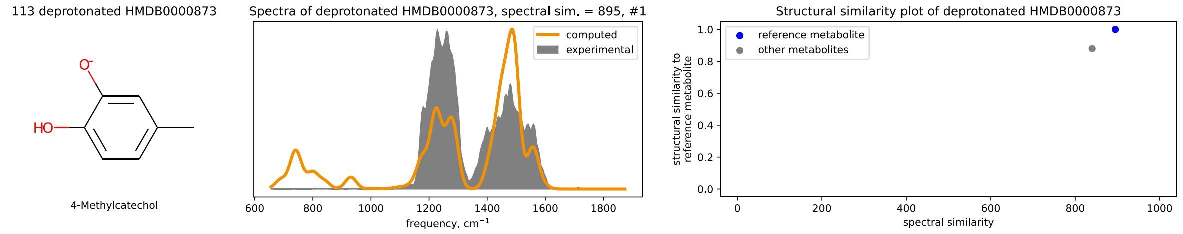

113 deprotonated HMDB0000873
O-
HO
4-Methylcatechol
Spectra of deprotonated HMDB0000873, spectral sim. = 895, #1
computed
experimental
frequency, cm−1
Structural similarity plot of deprotonated HMDB0000873
reference metabolite
other metabolites
structural similarity to reference metabolite
spectral similarity


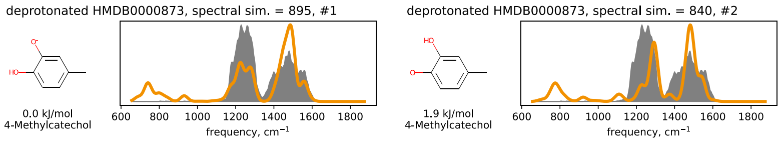

deprotonated HMDB0000873, spectral sim. = 895, #1
0.0 kJ/mol
4-Methylcatechol
frequency, cm−1
deprotonated HMDB0000873, spectral sim. = 840, #2
1.9 kJ/mol
4-Methylcatechol
frequency, cm−1


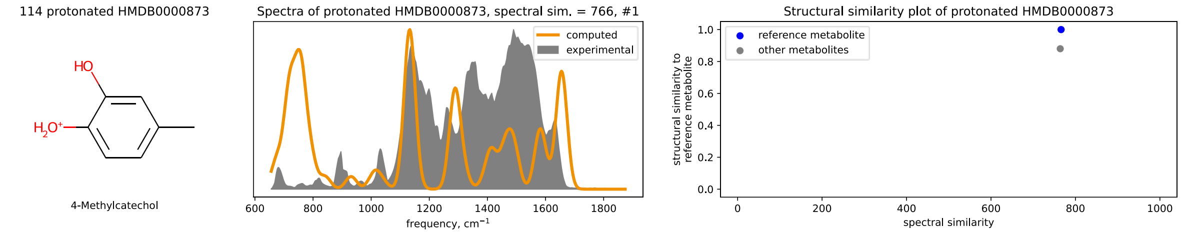

114 protonated HMDB0000873
HO
H2O+
4-Methylcatechol
Spectra of protonated HMDB0000873, spectral sim. = 766, #1
computed
experimental
frequency, cm−1
Structural similarity plot of protonated HMDB0000873
reference metabolite
other metabolites
structural similarity to reference metabolite
spectral similarity


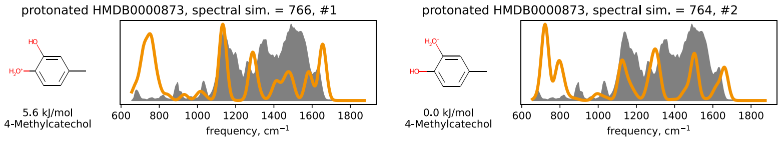

protonated HMDB0000873, spectral sim. = 766, #1
5.6 kJ/mol
4-Methylcatechol
frequency, cm−1
protonated HMDB0000873, spectral sim. = 764, #2
0.0 kJ/mol
4-Methylcatechol
frequency, cm−1

115 sodiated HMDB0000873

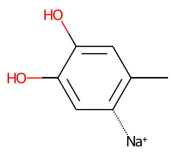


4-Methylcatechol

Spectra of sodiated HMDB0000873, spectral sim. = 898, #1

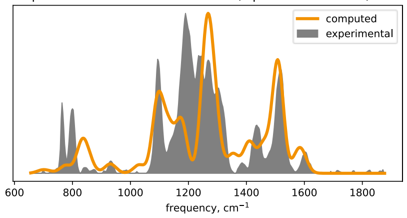


Structural similarity plot of sodiated HMDB0000873

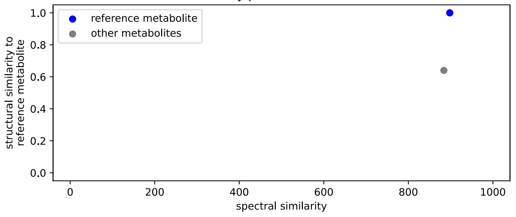


sodiated HMDB0000873, spectral sim. = 898, #1

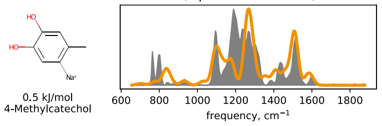


sodiated HMDB0000873, spectral sim. = 884, #2

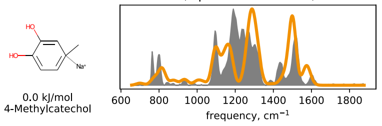


116 deprotonated HMDB0000904

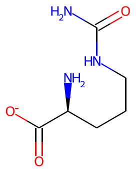


Citrulline

Spectra of deprotonated HMDB0000904, spectral sim. = 914, #1

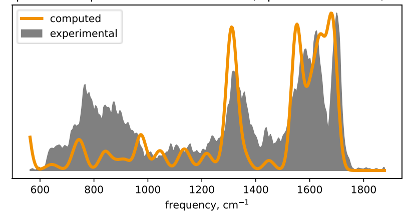


Structural similarity plot of deprotonated HMDB0000904

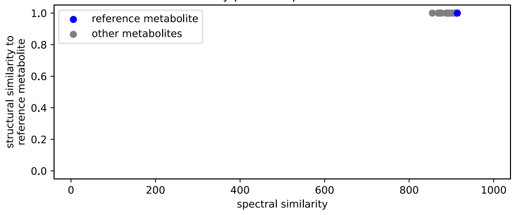


deprotonated HMDB0000904, spectral sim. = 914, #1

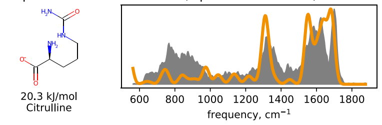


deprotonated HMDB0000904, spectral sim. = 908, #2

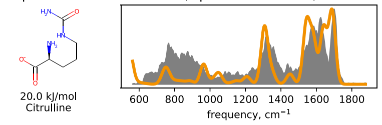


deprotonated HMDB0000904, spectral sim. = 908, #3

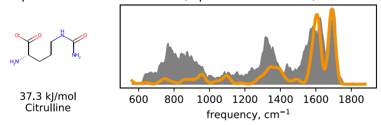


deprotonated HMDB0000904, spectral sim. = 901, #4

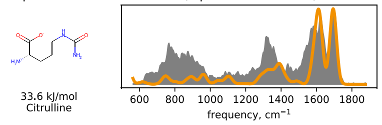


deprotonated HMDB0000904, spectral sim. = 894, #5

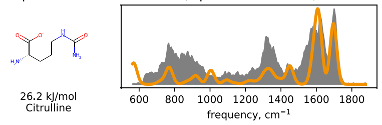


deprotonated HMDB0000904, spectral sim. = 894, #6

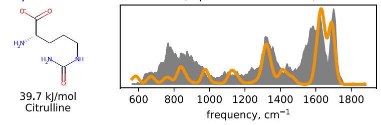


deprotonated HMDB0000904, spectral sim. = 891, #7

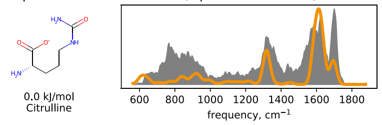


deprotonated HMDB0000904, spectral sim. = 890, #8

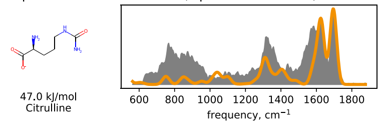


deprotonated HMDB0000904, spectral sim. = 887, #9

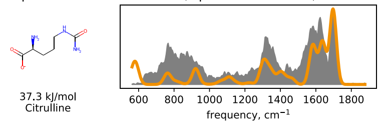


deprotonated HMDB0000904, spectral sim. = 876, #10

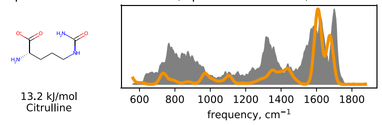


deprotonated HMDB0000904, spectral sim. = 873, #11

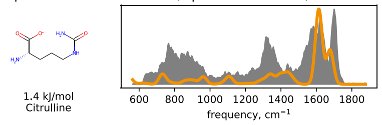


deprotonated HMDB0000904, spectral sim. = 870, #12

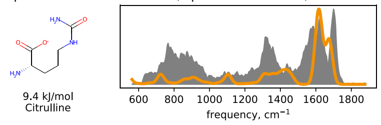


deprotonated HMDB0000904, spectral sim. = 869, #13

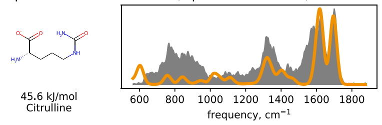


deprotonated HMDB0000904, spectral sim. = 855, #14

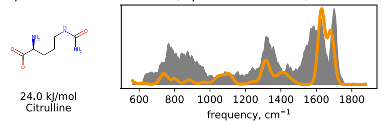

117 protonated HMDB0000904

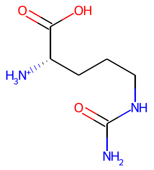


Citrulline

Spectra of protonated HMDB0000904, spectral sim. = 953, #1

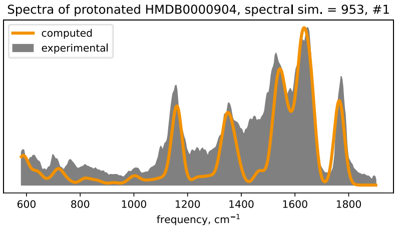


Structural similarity plot of protonated HMDB0000904

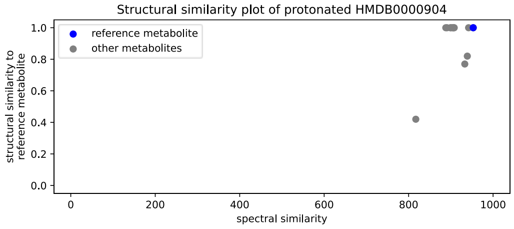


protonated HMDB0000904, spectral sim. = 953, #1

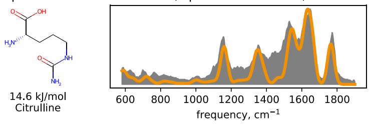


protonated HMDB0000904, spectral sim. = 942, #2

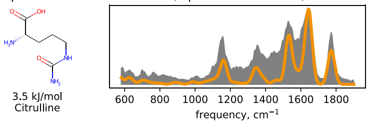


protonated HMDB0000904, spectral sim. = 939, #3

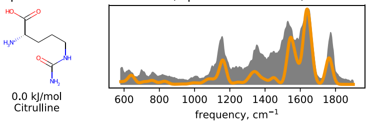


protonated HMDB0000904, spectral sim. = 933, #4

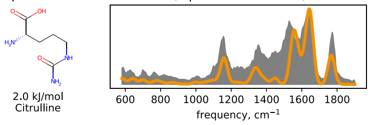


protonated HMDB0000904, spectral sim. = 908, #5

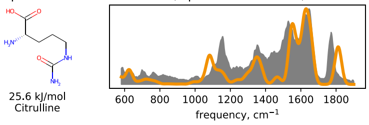


protonated HMDB0000904, spectral sim. = 907, #6

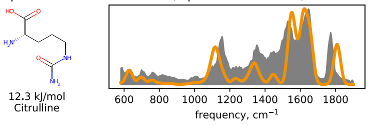


protonated HMDB0000904, spectral sim. = 905, #7

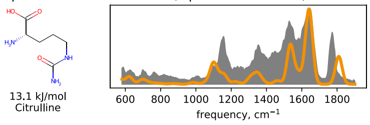


protonated HMDB0000904, spectral sim. = 904, #8

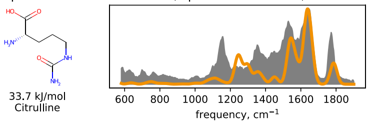


protonated HMDB0000904, spectral sim. = 900, #9

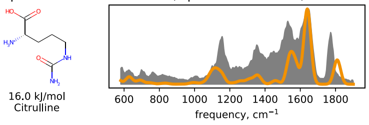


protonated HMDB0000904, spectral sim. = 899, #10

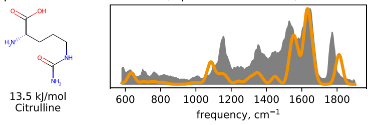


protonated HMDB0000904, spectral sim. = 891, #11

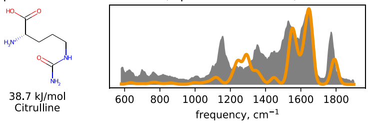


protonated HMDB0000904, spectral sim. = 888, #12

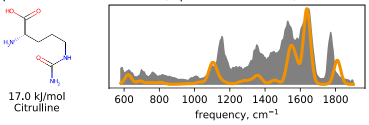


protonated HMDB0000904, spectral sim. = 817, #13

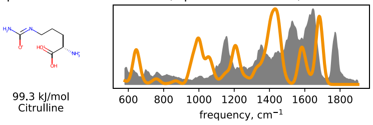

118 sodiated HMDB0000904

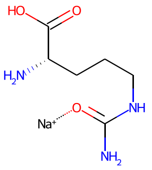


Citrulline

Spectra of sodiated HMDB0000904, spectral sim. = 918, #1

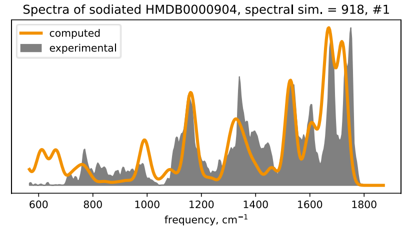


Structural similarity plot of sodiated HMDB0000904

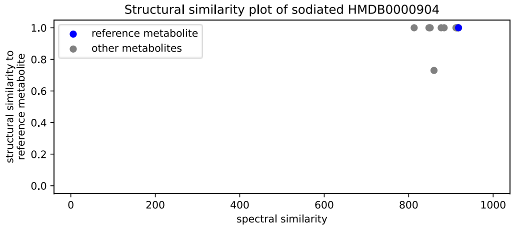


sodiated HMDB0000904, spectral sim. = 918, #1

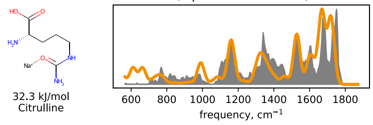


sodiated HMDB0000904, spectral sim. = 916, #2

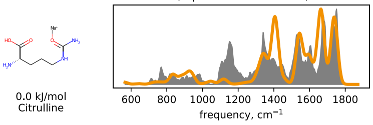


sodiated HMDB0000904, spectral sim. = 914, #3

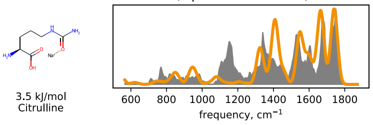


sodiated HMDB0000904, spectral sim. = 912, #4

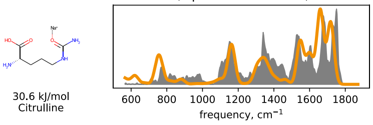


sodiated HMDB0000904, spectral sim. = 884, #5

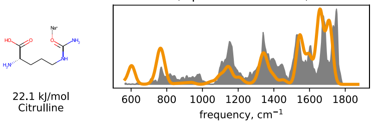


sodiated HMDB0000904, spectral sim. = 877, #6

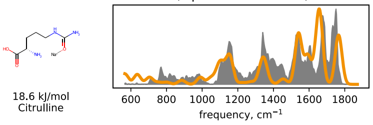


sodiated HMDB0000904, spectral sim. = 860, #7

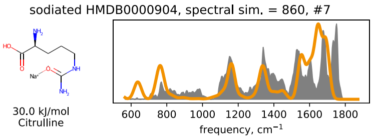


sodiated HMDB0000904, spectral sim. = 851, #8

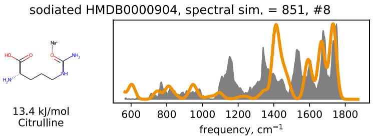


sodiated HMDB0000904, spectral sim. = 848, #9

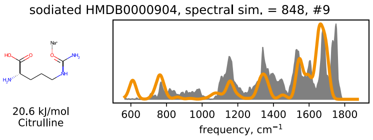


sodiated HMDB0000904, spectral sim. = 813, #10

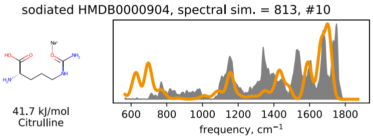

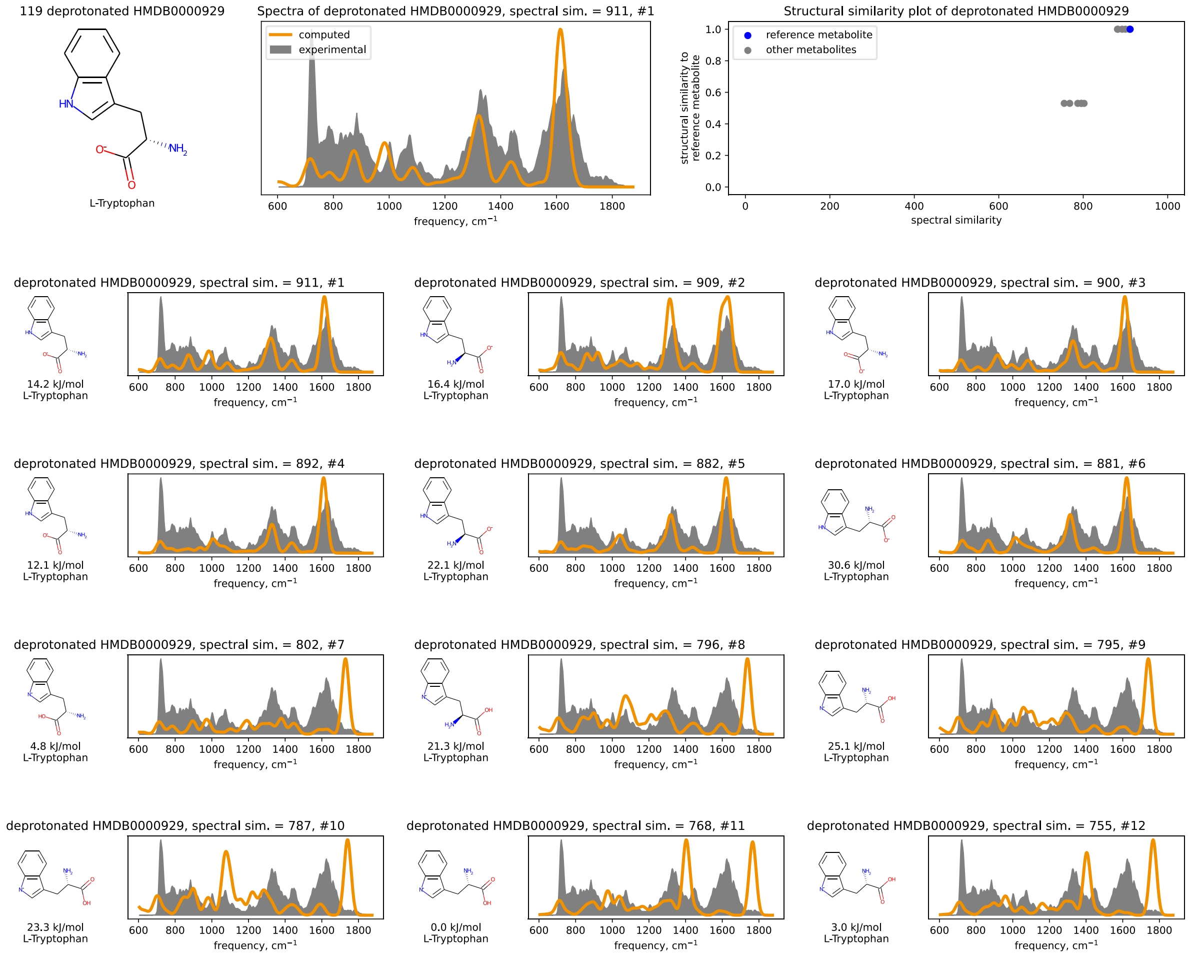
119 deprotonated HMDB0000929
HN
O-
NH2
O
L-Tryptophan
Spectra of deprotonated HMDB0000929, spectral sim. = 911, #1
computed
experimental
frequency, cm−1
Structural similarity plot of deprotonated HMDB0000929
reference metabolite
other metabolites
structural similarity to reference metabolite
spectral similarity
deprotonated HMDB0000929, spectral sim. = 911, #1
14.2 kJ/mol
L-Tryptophan
deprotonated HMDB0000929, spectral sim. = 909, #2
16.4 kJ/mol
L-Tryptophan
deprotonated HMDB0000929, spectral sim. = 900, #3
17.0 kJ/mol
L-Tryptophan
deprotonated HMDB0000929, spectral sim. = 892, #4
12.1 kJ/mol
L-Tryptophan
deprotonated HMDB0000929, spectral sim. = 882, #5
22.1 kJ/mol
L-Tryptophan
deprotonated HMDB0000929, spectral sim. = 881, #6
30.6 kJ/mol
L-Tryptophan
deprotonated HMDB0000929, spectral sim. = 802, #7
4.8 kJ/mol
L-Tryptophan
deprotonated HMDB0000929, spectral sim. = 796, #8
21.3 kJ/mol
L-Tryptophan
deprotonated HMDB0000929, spectral sim. = 795, #9
25.1 kJ/mol
L-Tryptophan
deprotonated HMDB0000929, spectral sim. = 787, #10
23.3 kJ/mol
L-Tryptophan
deprotonated HMDB0000929, spectral sim. = 768, #11
0.0 kJ/mol
L-Tryptophan
deprotonated HMDB0000929, spectral sim. = 755, #12
3.0 kJ/mol
L-Tryptophan
frequency, cm−1

120 protonated HMDB0000929

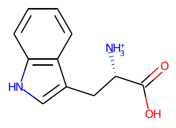


L-Tryptophan

Spectra of protonated HMDB0000929, spectral sim. = 938, #1

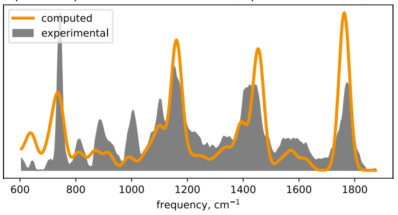


Structural similarity plot of protonated HMDB0000929

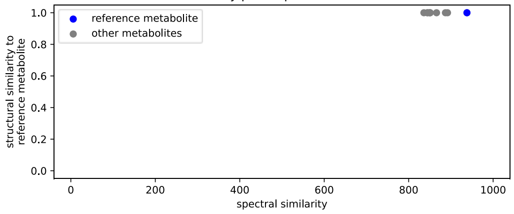


protonated HMDB0000929, spectral sim. = 938, #1

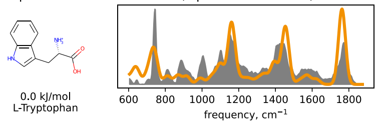

0.0 kJ/mol
L-Tryptophan

protonated HMDB0000929, spectral sim. = 892, #2

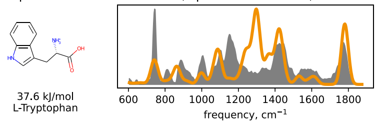

37.6 kJ/mol
L-Tryptophan

protonated HMDB0000929, spectral sim. = 887, #3

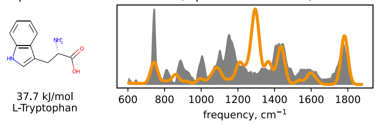

37.7 kJ/mol
L-Tryptophan

protonated HMDB0000929, spectral sim. = 866, #4

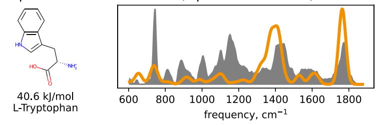

40.6 kJ/mol
L-Tryptophan

protonated HMDB0000929, spectral sim. = 851, #5

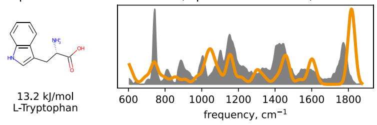

13.2 kJ/mol
L-Tryptophan

protonated HMDB0000929, spectral sim. = 850, #6

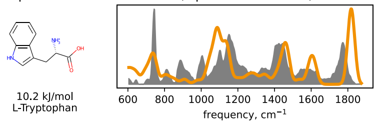

10.2 kJ/mol
L-Tryptophan

protonated HMDB0000929, spectral sim. = 850, #7

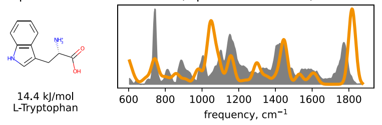

14.4 kJ/mol
L-Tryptophan

protonated HMDB0000929, spectral sim. = 845, #8

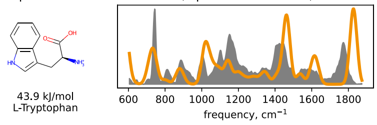

43.9 kJ/mol
L-Tryptophan

protonated HMDB0000929, spectral sim. = 844, #9

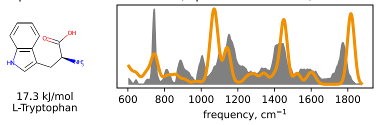

17.3 kJ/mol
L-Tryptophan

protonated HMDB0000929, spectral sim. = 836, #10

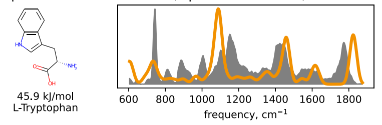

45.9 kJ/mol
L-Tryptophan

121 sodiated HMDB0000929

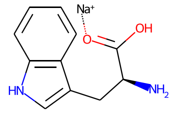


L-Tryptophan

Spectra of sodiated HMDB0000929, spectral sim. = 895, #1

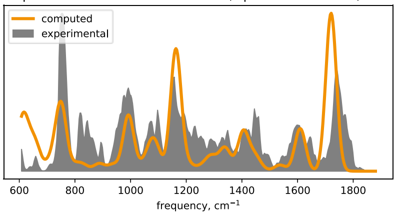


Structural similarity plot of sodiated HMDB0000929

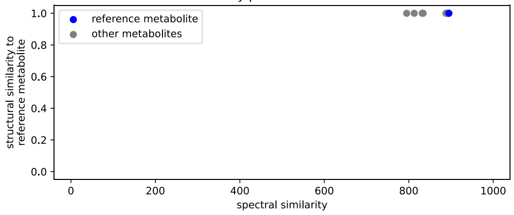


sodiated HMDB0000929, spectral sim. = 895, #1

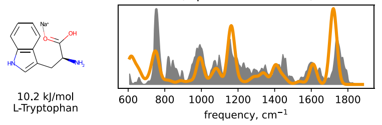

10.2 kJ/mol
L-Tryptophan

sodiated HMDB0000929, spectral sim. = 891, #2

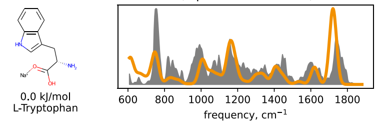

0.0 kJ/mol
L-Tryptophan

sodiated HMDB0000929, spectral sim. = 888, #3

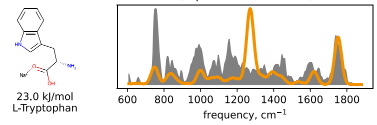

23.0 kJ/mol
L-Tryptophan

sodiated HMDB0000929, spectral sim. = 834, #4

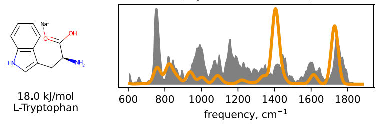

18.0 kJ/mol
L-Tryptophan

sodiated HMDB0000929, spectral sim. = 832, #5

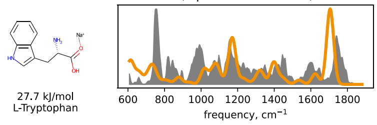

27.7 kJ/mol
L-Tryptophan

sodiated HMDB0000929, spectral sim. = 813, #6

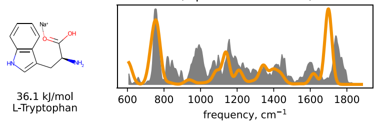

36.1 kJ/mol
L-Tryptophan

sodiated HMDB0000929, spectral sim. = 795, #7

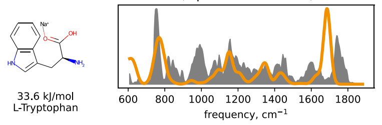

33.6 kJ/mol
L-Tryptophan

122 deprotonated HMDB0000930

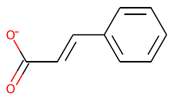

trans-Cinnamic acid

Spectra of deprotonated HMDB0000930, spectral sim. = 880, #1

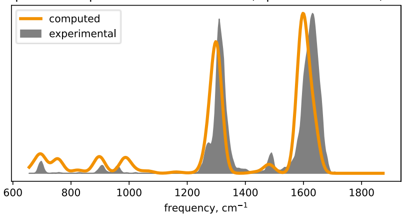


Structural similarity plot of deprotonated HMDB0000930

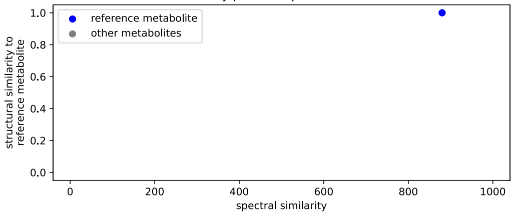


deprotonated HMDB0000930, spectral sim. = 880, #1

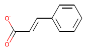

0.0 kJ/mol
trans-Cinnamic acid

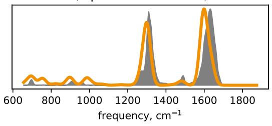


123 sodiated HMDB0000930

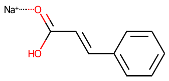

trans-Cinnamic acid

Spectra of sodiated HMDB0000930, spectral sim. = 894, #1

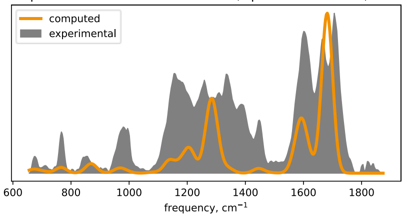


Structural similarity plot of sodiated HMDB0000930

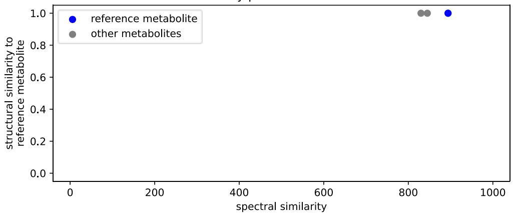


sodiated HMDB0000930, spectral sim. = 894, #1

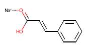

5.1 kJ/mol
trans-Cinnamic acid

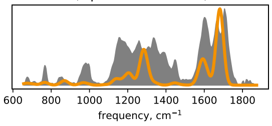


sodiated HMDB0000930, spectral sim. = 845, #2

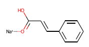

2.1 kJ/mol
trans-Cinnamic acid

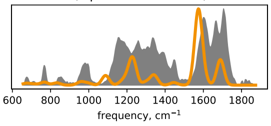


sodiated HMDB0000930, spectral sim. = 830, #3

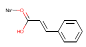

0.0 kJ/mol
trans-Cinnamic acid

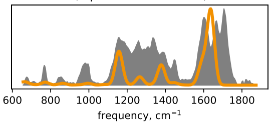

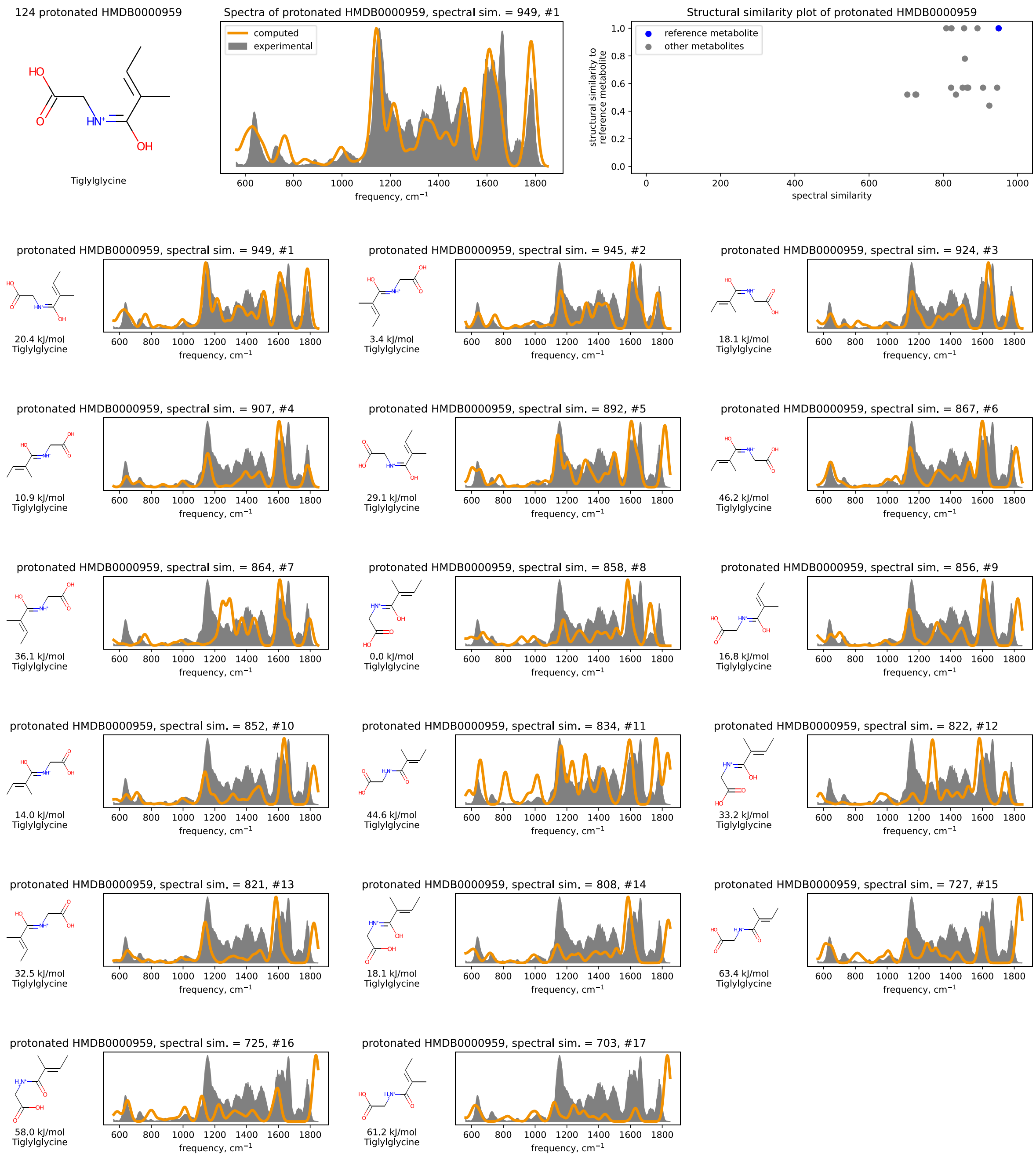

124 protonated HMDB0000959
Tiglylglycine
Spectra of protonated HMDB0000959, spectral sim. = 949, #1
computed
experimental
frequency, cm⁻¹
Structural similarity plot of protonated HMDB0000959
reference metabolite
other metabolites
structural similarity to reference metabolite
spectral similarity
protonated HMDB0000959, spectral sim. = 949, #1
20.4 kJ/mol
Tiglylglycine
protonated HMDB0000959, spectral sim. = 945, #2
3.4 kJ/mol
Tiglylglycine
protonated HMDB0000959, spectral sim. = 924, #3
18.1 kJ/mol
Tiglylglycine
protonated HMDB0000959, spectral sim. = 907, #4
10.9 kJ/mol
Tiglylglycine
protonated HMDB0000959, spectral sim. = 892, #5
29.1 kJ/mol
Tiglylglycine
protonated HMDB0000959, spectral sim. = 867, #6
46.2 kJ/mol
Tiglylglycine
protonated HMDB0000959, spectral sim. = 864, #7
36.1 kJ/mol
Tiglylglycine
protonated HMDB0000959, spectral sim. = 858, #8
0.0 kJ/mol
Tiglylglycine
protonated HMDB0000959, spectral sim. = 856, #9
16.8 kJ/mol
Tiglylglycine
protonated HMDB0000959, spectral sim. = 852, #10
14.0 kJ/mol
Tiglylglycine
protonated HMDB0000959, spectral sim. = 834, #11
44.6 kJ/mol
Tiglylglycine
protonated HMDB0000959, spectral sim. = 822, #12
33.2 kJ/mol
Tiglylglycine
protonated HMDB0000959, spectral sim. = 821, #13
32.5 kJ/mol
Tiglylglycine
protonated HMDB0000959, spectral sim. = 808, #14
18.1 kJ/mol
Tiglylglycine
protonated HMDB0000959, spectral sim. = 727, #15
63.4 kJ/mol
Tiglylglycine
protonated HMDB0000959, spectral sim. = 725, #16
58.0 kJ/mol
Tiglylglycine
protonated HMDB0000959, spectral sim. = 703, #17
61.2 kJ/mol
Tiglylglycine

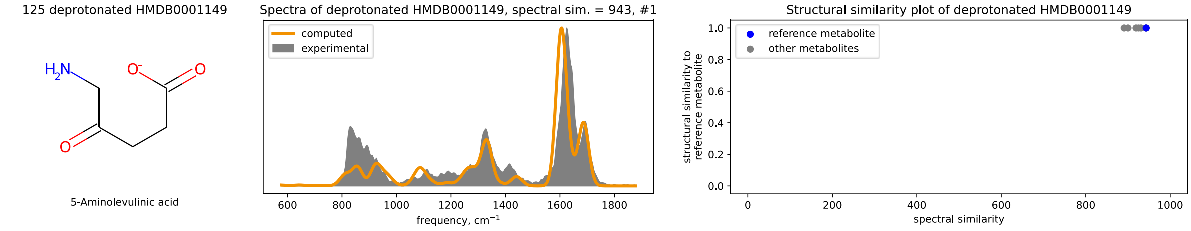
125 deprotonated HMDB0001149
5-Aminolevulinic acid
Spectra of deprotonated HMDB0001149, spectral sim. = 943, #1
computed
experimental
frequency, cm$^{-1}$
Structural similarity plot of deprotonated HMDB0001149
reference metabolite
other metabolites
structural similarity to reference metabolite
spectral similarity

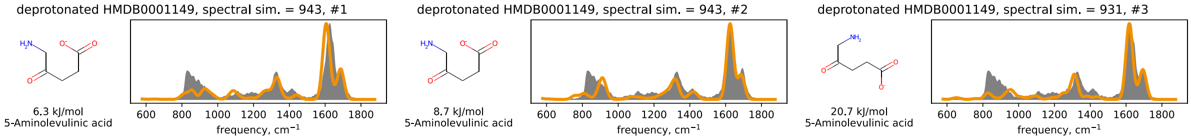
deprotonated HMDB0001149, spectral sim. = 943, #1
6.3 kJ/mol
5-Aminolevulinic acid
deprotonated HMDB0001149, spectral sim. = 943, #2
8.7 kJ/mol
5-Aminolevulinic acid
deprotonated HMDB0001149, spectral sim. = 931, #3
20.7 kJ/mol
5-Aminolevulinic acid
frequency, cm$^{-1}$

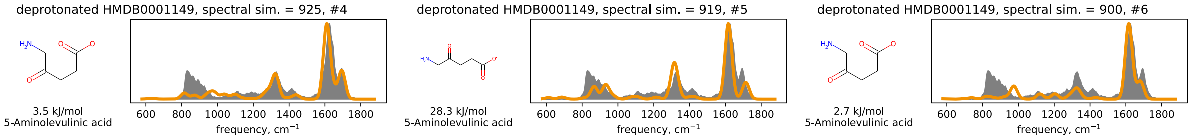
deprotonated HMDB0001149, spectral sim. = 925, #4
3.5 kJ/mol
5-Aminolevulinic acid
deprotonated HMDB0001149, spectral sim. = 919, #5
28.3 kJ/mol
5-Aminolevulinic acid
deprotonated HMDB0001149, spectral sim. = 900, #6
2.7 kJ/mol
5-Aminolevulinic acid
frequency, cm$^{-1}$

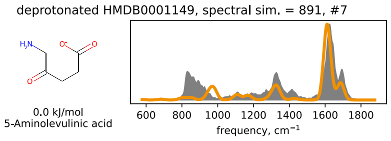
deprotonated HMDB0001149, spectral sim. = 891, #7
0.0 kJ/mol
5-Aminolevulinic acid
frequency, cm$^{-1}$

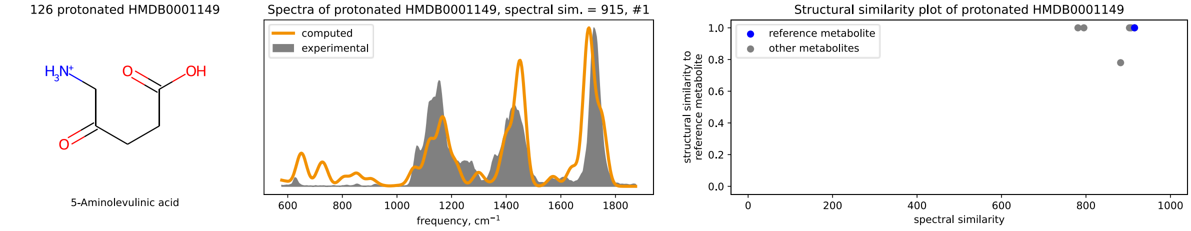
126 protonated HMDB0001149
5-Aminolevulinic acid
Spectra of protonated HMDB0001149, spectral sim. = 915, #1
computed
experimental
frequency, cm$^{-1}$
Structural similarity plot of protonated HMDB0001149
reference metabolite
other metabolites
structural similarity to reference metabolite
spectral similarity

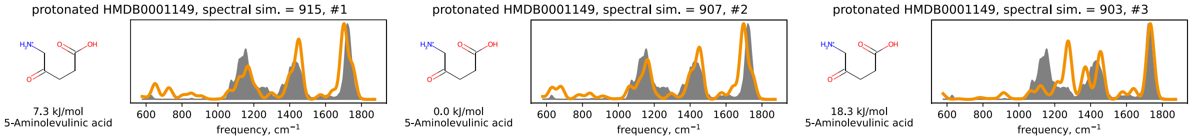
protonated HMDB0001149, spectral sim. = 915, #1
7.3 kJ/mol
5-Aminolevulinic acid
protonated HMDB0001149, spectral sim. = 907, #2
0.0 kJ/mol
5-Aminolevulinic acid
protonated HMDB0001149, spectral sim. = 903, #3
18.3 kJ/mol
5-Aminolevulinic acid
frequency, cm$^{-1}$

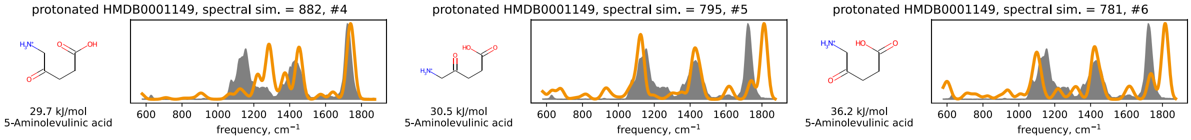
protonated HMDB0001149, spectral sim. = 882, #4
29.7 kJ/mol
5-Aminolevulinic acid
protonated HMDB0001149, spectral sim. = 795, #5
30.5 kJ/mol
5-Aminolevulinic acid
protonated HMDB0001149, spectral sim. = 781, #6
36.2 kJ/mol
5-Aminolevulinic acid
frequency, cm$^{-1}$

127 sodiated HMDB0001149

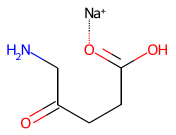


5-Aminolevulinic acid

Spectra of sodiated HMDB0001149, spectral sim. = 902, #1

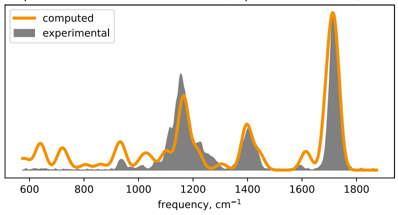


Structural similarity plot of sodiated HMDB0001149

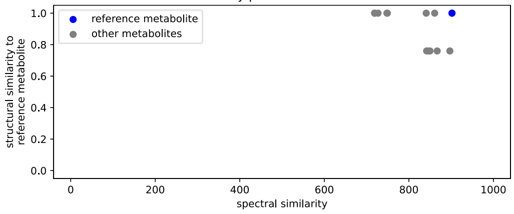


sodiated HMDB0001149, spectral sim. = 902, #1

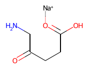

3.9 kJ/mol
5-Aminolevulinic acid

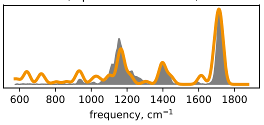

sodiated HMDB0001149, spectral sim. = 897, #2

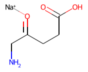

31.6 kJ/mol
5-Aminolevulinic acid

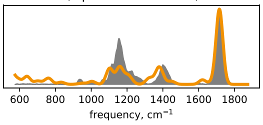

sodiated HMDB0001149, spectral sim. = 867, #3

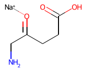

23.1 kJ/mol
5-Aminolevulinic acid

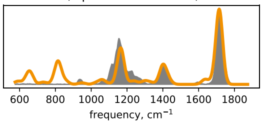

sodiated HMDB0001149, spectral sim. = 861, #4

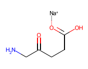

0.0 kJ/mol
5-Aminolevulinic acid

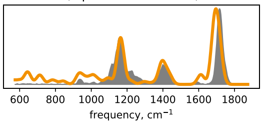

sodiated HMDB0001149, spectral sim. = 850, #5

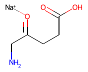

21.2 kJ/mol
5-Aminolevulinic acid

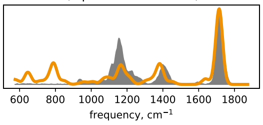

sodiated HMDB0001149, spectral sim. = 846, #6

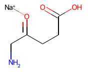

20.8 kJ/mol
5-Aminolevulinic acid

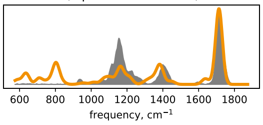

sodiated HMDB0001149, spectral sim. = 842, #7

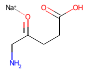

29.4 kJ/mol
5-Aminolevulinic acid

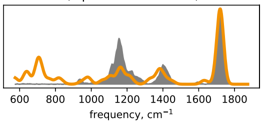

sodiated HMDB0001149, spectral sim. = 841, #8

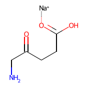

41.5 kJ/mol
5-Aminolevulinic acid

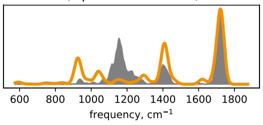

sodiated HMDB0001149, spectral sim. = 749, #9

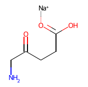

42.2 kJ/mol
5-Aminolevulinic acid

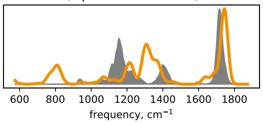

sodiated HMDB0001149, spectral sim. = 748, #10

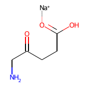

42.5 kJ/mol
5-Aminolevulinic acid

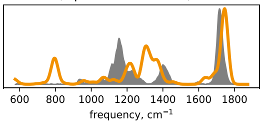

sodiated HMDB0001149, spectral sim. = 727, #11

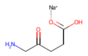

49.8 kJ/mol
5-Aminolevulinic acid

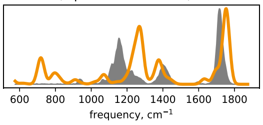

sodiated HMDB0001149, spectral sim. = 719, #12

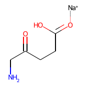

41.5 kJ/mol
5-Aminolevulinic acid

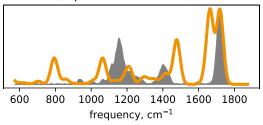

sodiated HMDB0001149, spectral sim. = 719, #13

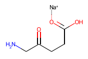

52.3 kJ/mol
5-Aminolevulinic acid

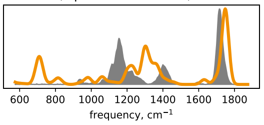

128 deprotonated HMDB0001232

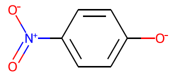

4-Nitrophenol

Spectra of deprotonated HMDB0001232, spectral sim. = 817, #1

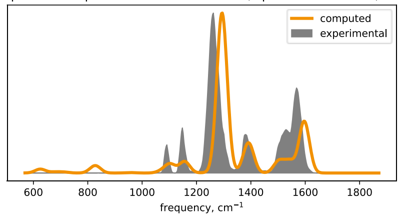


Structural similarity plot of deprotonated HMDB0001232

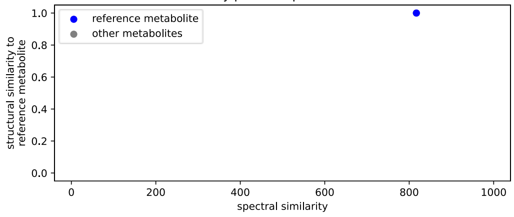


deprotonated HMDB0001232, spectral sim. = 817, #1

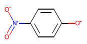

0.0 kJ/mol
4-Nitrophenol

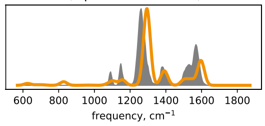

129 protonated HMDB0001232

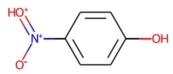

4-Nitrophenol

Spectra of protonated HMDB0001232, spectral sim. = 834, #1

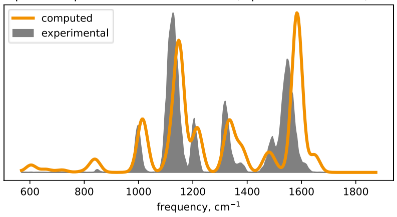


Structural similarity plot of protonated HMDB0001232

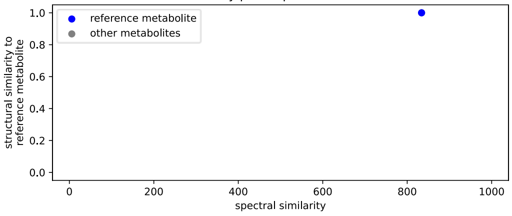


protonated HMDB0001232, spectral sim. = 834, #1

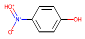

0.0 kJ/mol
4-Nitrophenol

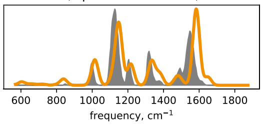


130 deprotonated HMDB0001336

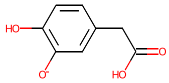

3,4-Dihydroxybenzeneacetic acid

Spectra of deprotonated HMDB0001336, spectral sim. = 948, #1

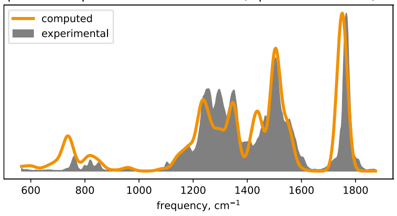


Structural similarity plot of deprotonated HMDB0001336

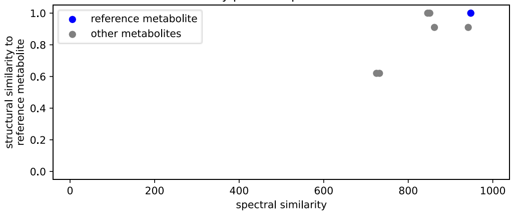


deprotonated HMDB0001336, spectral sim. = 948, #1

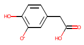

0.0 kJ/mol
3,4-
Dihydroxybenzeneacetic
acid

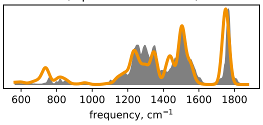

deprotonated HMDB0001336, spectral sim. = 942, #2

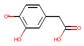

0.1 kJ/mol
3,4-
Dihydroxybenzeneacetic
acid

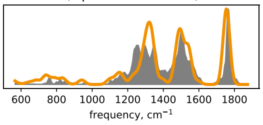

deprotonated HMDB0001336, spectral sim. = 862, #3

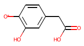

23.6 kJ/mol
3,4-
Dihydroxybenzeneacetic
acid

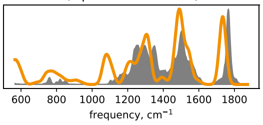

deprotonated HMDB0001336, spectral sim. = 851, #4

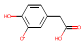

23.3 kJ/mol
3,4-
Dihydroxybenzeneacetic
acid

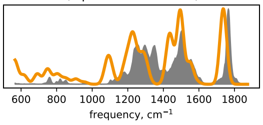

deprotonated HMDB0001336, spectral sim. = 846, #5

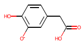

22.2 kJ/mol
3,4-
Dihydroxybenzeneacetic
acid

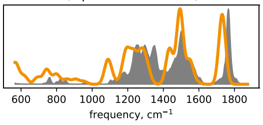

deprotonated HMDB0001336, spectral sim. = 732, #6

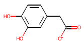

39.1 kJ/mol
3,4-
Dihydroxybenzeneacetic
acid

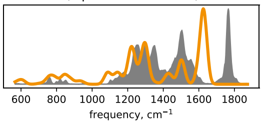

deprotonated HMDB0001336, spectral sim. = 725, #7

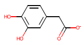

56.9 kJ/mol
3,4-
Dihydroxybenzeneacetic
acid

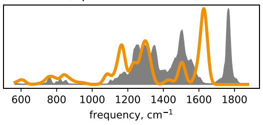

131 sodiated HMDB0001336

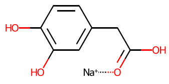

3,4-Dihydroxybenzeneacetic acid

Spectra of sodiated HMDB0001336, spectral sim. = 923, #1

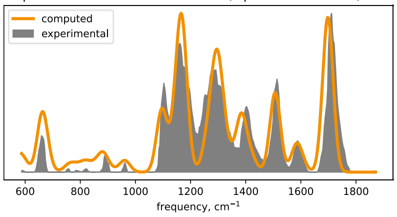


Structural similarity plot of sodiated HMDB0001336

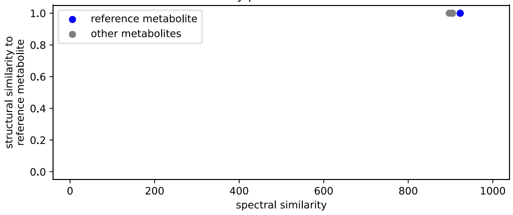


sodiated HMDB0001336, spectral sim. = 923, #1

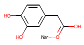

0.0 kJ/mol
3,4-
Dihydroxybenzeneacetic
acid

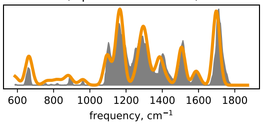

sodiated HMDB0001336, spectral sim. = 905, #2

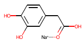

21.8 kJ/mol
3,4-
Dihydroxybenzeneacetic
acid

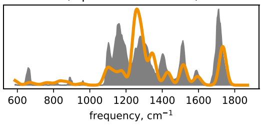

sodiated HMDB0001336, spectral sim. = 903, #3

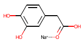

18.9 kJ/mol
3,4-
Dihydroxybenzeneacetic
acid

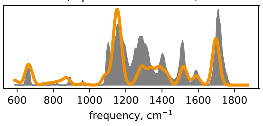

sodiated HMDB0001336, spectral sim. = 897, #4

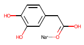

19.8 kJ/mol
3,4-
Dihydroxybenzeneacetic
acid

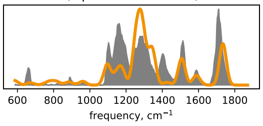

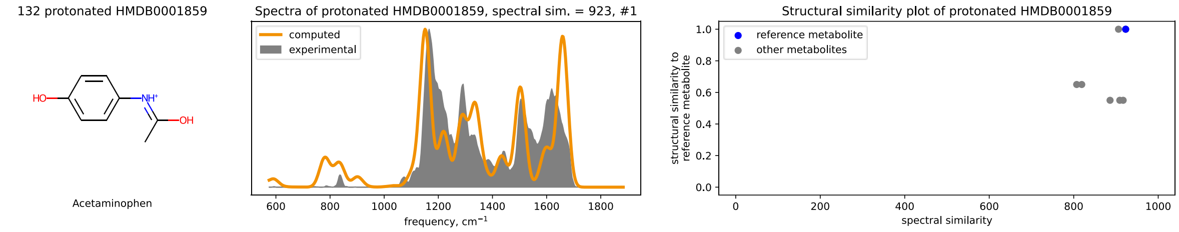
132 protonated HMDB0001859
Acetaminophen
Spectra of protonated HMDB0001859, spectral sim. = 923, #1
computed
experimental
frequency, cm$^{-1}$
Structural similarity plot of protonated HMDB0001859
reference metabolite
other metabolites
structural similarity to reference metabolite
spectral similarity

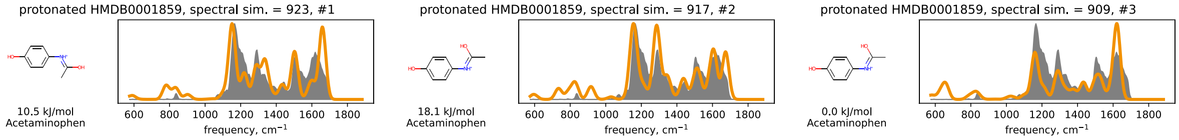
protonated HMDB0001859, spectral sim. = 923, #1
10.5 kJ/mol
Acetaminophen
protonated HMDB0001859, spectral sim. = 917, #2
18.1 kJ/mol
Acetaminophen
protonated HMDB0001859, spectral sim. = 909, #3
0.0 kJ/mol
Acetaminophen
frequency, cm$^{-1}$

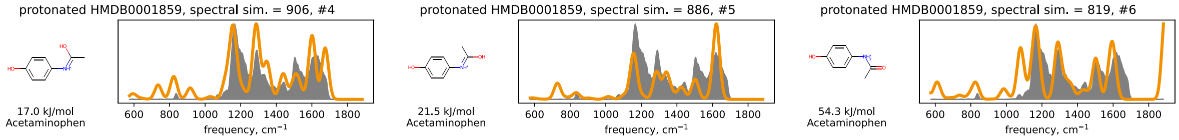
protonated HMDB0001859, spectral sim. = 906, #4
17.0 kJ/mol
Acetaminophen
protonated HMDB0001859, spectral sim. = 886, #5
21.5 kJ/mol
Acetaminophen
protonated HMDB0001859, spectral sim. = 819, #6
54.3 kJ/mol
Acetaminophen
frequency, cm$^{-1}$

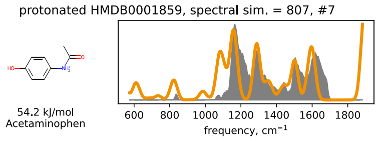
protonated HMDB0001859, spectral sim. = 807, #7
54.2 kJ/mol
Acetaminophen
frequency, cm$^{-1}$

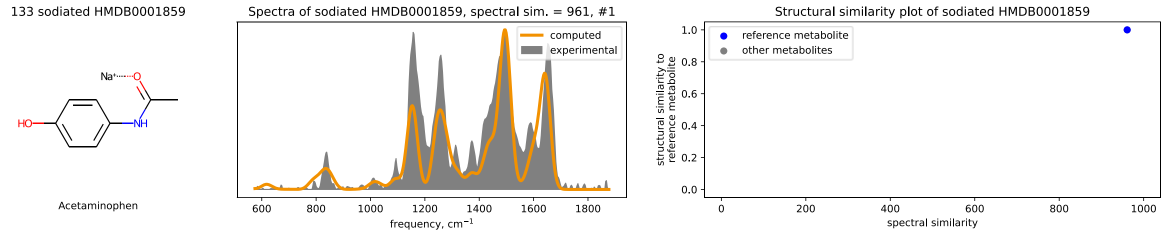
133 sodiated HMDB0001859
Acetaminophen
Spectra of sodiated HMDB0001859, spectral sim. = 961, #1
computed
experimental
frequency, cm$^{-1}$
Structural similarity plot of sodiated HMDB0001859
reference metabolite
other metabolites
structural similarity to reference metabolite
spectral similarity

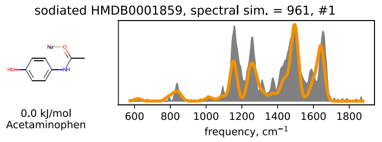
sodiated HMDB0001859, spectral sim. = 961, #1
0.0 kJ/mol
Acetaminophen
frequency, cm$^{-1}$

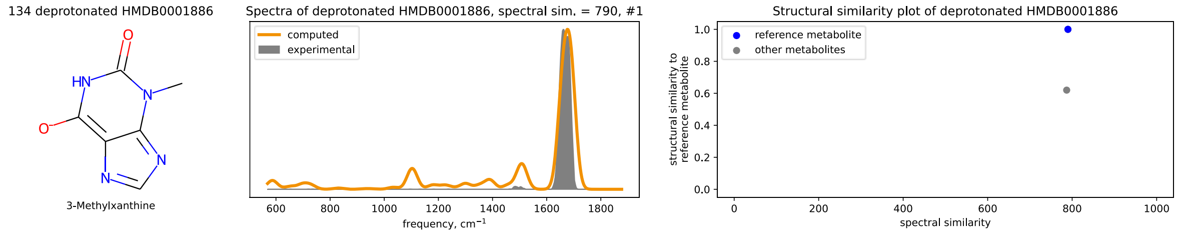
134 deprotonated HMDB0001886
3-Methylxanthine
Spectra of deprotonated HMDB0001886, spectral sim. = 790, #1
computed
experimental
frequency, cm$^{-1}$
Structural similarity plot of deprotonated HMDB0001886
reference metabolite
other metabolites
structural similarity to reference metabolite
spectral similarity

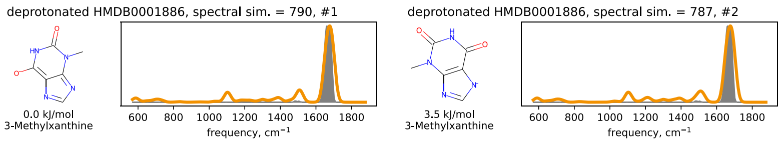
deprotonated HMDB0001886, spectral sim. = 790, #1
0.0 kJ/mol
3-Methylxanthine
deprotonated HMDB0001886, spectral sim. = 787, #2
3.5 kJ/mol
3-Methylxanthine
frequency, cm$^{-1}$

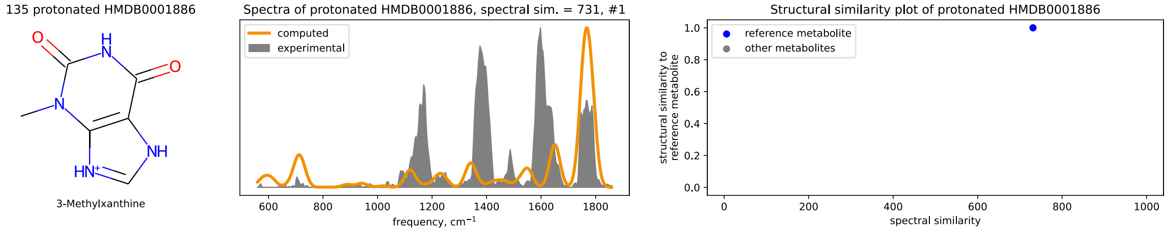

135 protonated HMDB0001886
3-Methylxanthine
Spectra of protonated HMDB0001886, spectral sim. = 731, #1
computed
experimental
frequency, cm⁻¹
Structural similarity plot of protonated HMDB0001886
reference metabolite
other metabolites
structural similarity to reference metabolite
spectral similarity


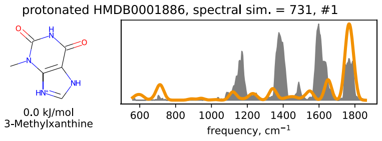

protonated HMDB0001886, spectral sim. = 731, #1
0.0 kJ/mol
3-Methylxanthine
frequency, cm⁻¹


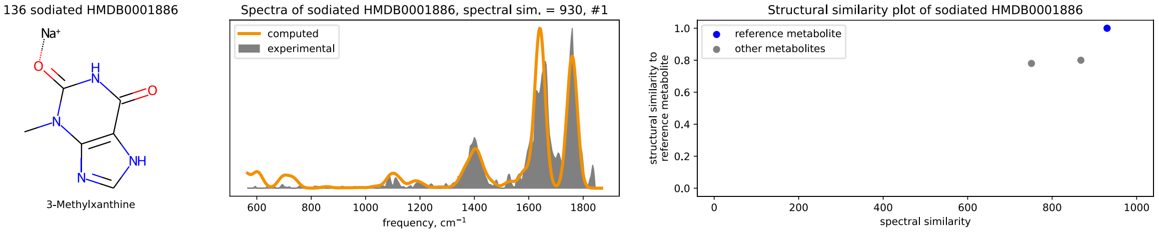

136 sodiated HMDB0001886
3-Methylxanthine
Spectra of sodiated HMDB0001886, spectral sim. = 930, #1
computed
experimental
frequency, cm⁻¹
Structural similarity plot of sodiated HMDB0001886
reference metabolite
other metabolites
structural similarity to reference metabolite
spectral similarity


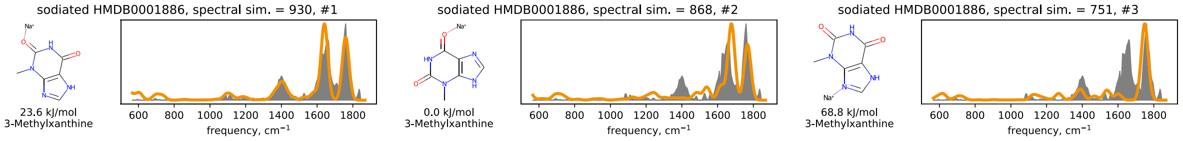

sodiated HMDB0001886, spectral sim. = 930, #1
23.6 kJ/mol
3-Methylxanthine
sodiated HMDB0001886, spectral sim. = 868, #2
0.0 kJ/mol
3-Methylxanthine
sodiated HMDB0001886, spectral sim. = 751, #3
68.8 kJ/mol
3-Methylxanthine
frequency, cm⁻¹


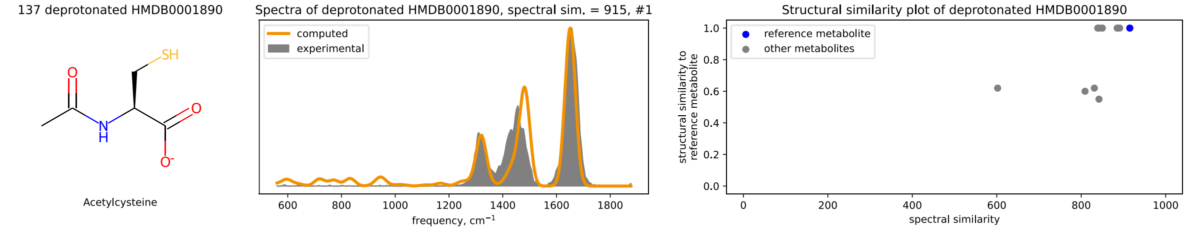

137 deprotonated HMDB0001890
Acetylcysteine
Spectra of deprotonated HMDB0001890, spectral sim. = 915, #1
computed
experimental
frequency, cm⁻¹
Structural similarity plot of deprotonated HMDB0001890
reference metabolite
other metabolites
structural similarity to reference metabolite
spectral similarity


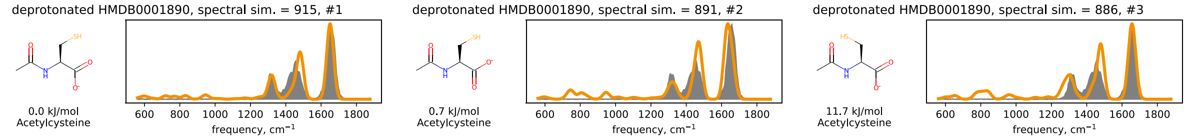

deprotonated HMDB0001890, spectral sim. = 915, #1
0.0 kJ/mol
Acetylcysteine
deprotonated HMDB0001890, spectral sim. = 891, #2
0.7 kJ/mol
Acetylcysteine
deprotonated HMDB0001890, spectral sim. = 886, #3
11.7 kJ/mol
Acetylcysteine
frequency, cm⁻¹


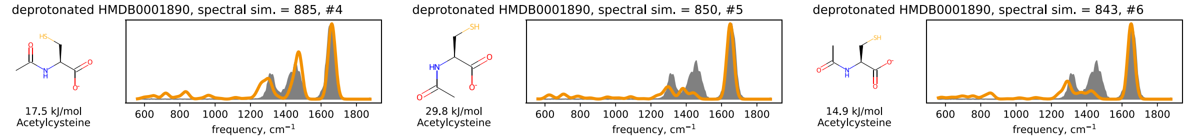

deprotonated HMDB0001890, spectral sim. = 885, #4
17.5 kJ/mol
Acetylcysteine
deprotonated HMDB0001890, spectral sim. = 850, #5
29.8 kJ/mol
Acetylcysteine
deprotonated HMDB0001890, spectral sim. = 843, #6
14.9 kJ/mol
Acetylcysteine
frequency, cm⁻¹


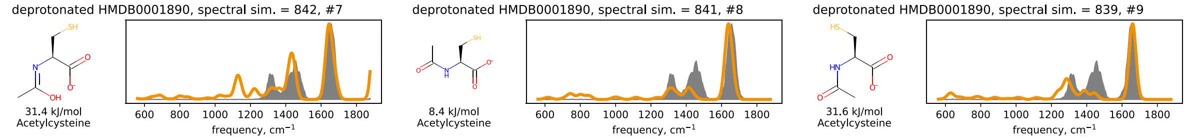

deprotonated HMDB0001890, spectral sim. = 842, #7
31.4 kJ/mol
Acetylcysteine
deprotonated HMDB0001890, spectral sim. = 841, #8
8.4 kJ/mol
Acetylcysteine
deprotonated HMDB0001890, spectral sim. = 839, #9
31.6 kJ/mol
Acetylcysteine
frequency, cm⁻¹


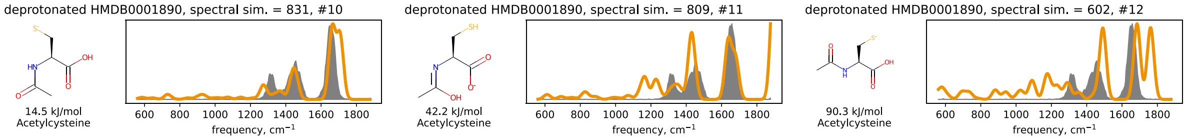

deprotonated HMDB0001890, spectral sim. = 831, #10
14.5 kJ/mol
Acetylcysteine
deprotonated HMDB0001890, spectral sim. = 809, #11
42.2 kJ/mol
Acetylcysteine
deprotonated HMDB0001890, spectral sim. = 602, #12
90.3 kJ/mol
Acetylcysteine
frequency, cm⁻¹

138 protonated HMDB0001890

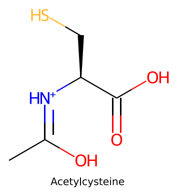


Spectra of protonated HMDB0001890, spectral sim. = 841, #1

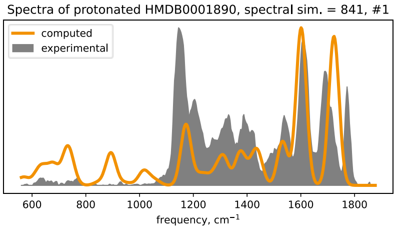


Structural similarity plot of protonated HMDB0001890

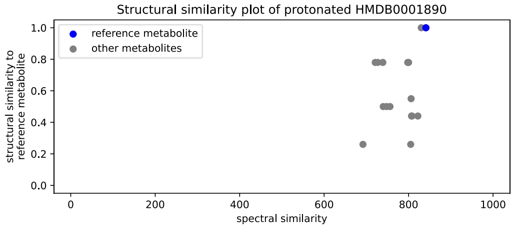


protonated HMDB0001890, spectral sim. = 841, #1

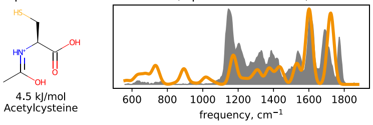


protonated HMDB0001890, spectral sim. = 830, #2

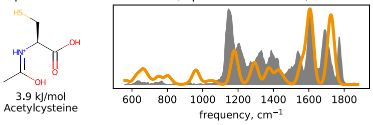


protonated HMDB0001890, spectral sim. = 822, #3

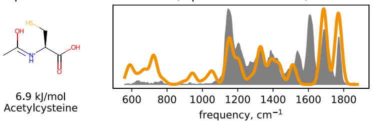


protonated HMDB0001890, spectral sim. = 808, #4

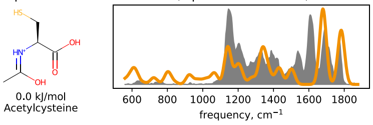


protonated HMDB0001890, spectral sim. = 807, #5

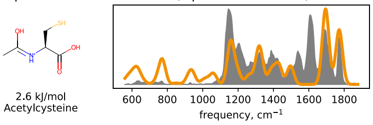


protonated HMDB0001890, spectral sim. = 806, #6

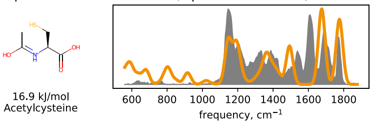


protonated HMDB0001890, spectral sim. = 805, #7

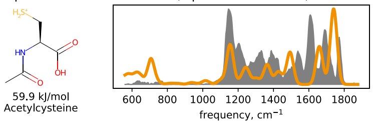


protonated HMDB0001890, spectral sim. = 800, #8

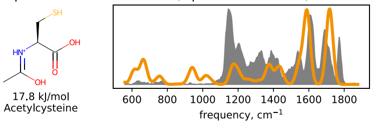


protonated HMDB0001890, spectral sim. = 798, #9

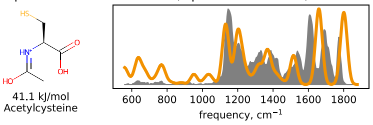


protonated HMDB0001890, spectral sim. = 756, #10

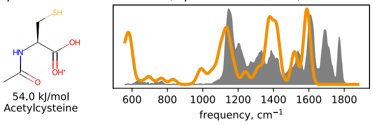


protonated HMDB0001890, spectral sim. = 748, #11

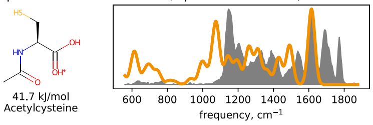


protonated HMDB0001890, spectral sim. = 740, #12

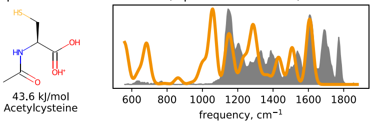


protonated HMDB0001890, spectral sim. = 739, #13

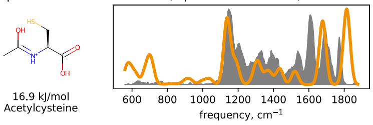


protonated HMDB0001890, spectral sim. = 727, #14

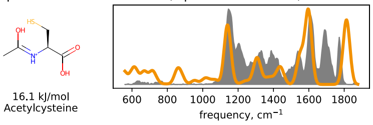


protonated HMDB0001890, spectral sim. = 721, #15

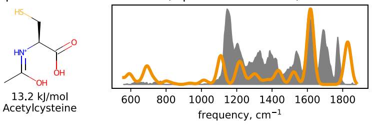


protonated HMDB0001890, spectral sim. = 692, #16

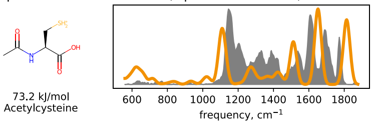

139 sodiated HMDB0001890

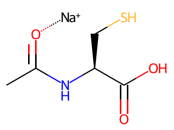


Acetylcysteine

Spectra of sodiated HMDB0001890, spectral sim. = 838, #1

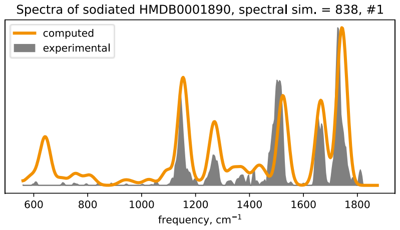


Structural similarity plot of sodiated HMDB0001890

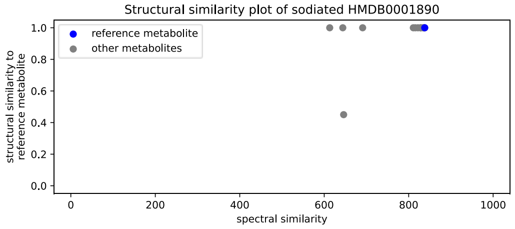


sodiated HMDB0001890, spectral sim. = 838, #1

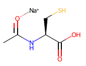

13.0 kJ/mol
Acetylcysteine

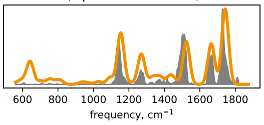


sodiated HMDB0001890, spectral sim. = 836, #2

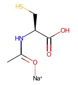

6.8 kJ/mol
Acetylcysteine

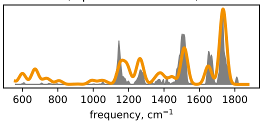


sodiated HMDB0001890, spectral sim. = 834, #3

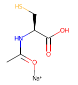

14.8 kJ/mol
Acetylcysteine

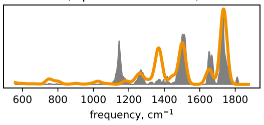


sodiated HMDB0001890, spectral sim. = 829, #4

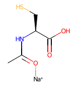

0.0 kJ/mol
Acetylcysteine

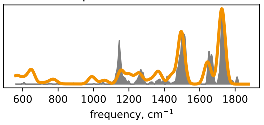


sodiated HMDB0001890, spectral sim. = 828, #5

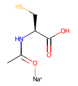

24.9 kJ/mol
Acetylcysteine

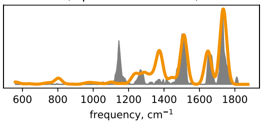


sodiated HMDB0001890, spectral sim. = 822, #6

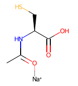

1.4 kJ/mol
Acetylcysteine

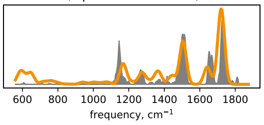


sodiated HMDB0001890, spectral sim. = 816, #7

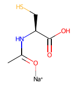

22.5 kJ/mol
Acetylcysteine

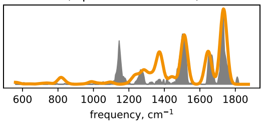


sodiated HMDB0001890, spectral sim. = 811, #8

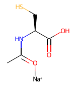

8.1 kJ/mol
Acetylcysteine

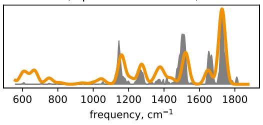


sodiated HMDB0001890, spectral sim. = 691, #9

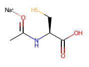

11.7 kJ/mol
Acetylcysteine

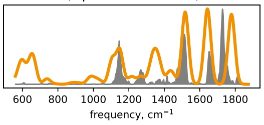


sodiated HMDB0001890, spectral sim. = 646, #10

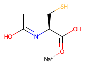

49.5 kJ/mol
Acetylcysteine

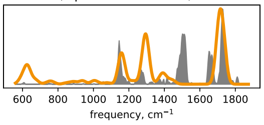


sodiated HMDB0001890, spectral sim. = 644, #11

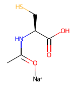

28.9 kJ/mol
Acetylcysteine

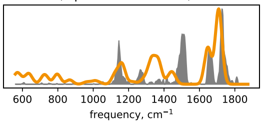


sodiated HMDB0001890, spectral sim. = 613, #12

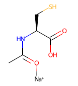

21.1 kJ/mol
Acetylcysteine

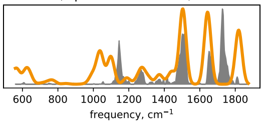

140 deprotonated HMDB0001964

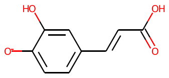


Caffeic acid

Spectra of deprotonated HMDB0001964, spectral sim. = 892, #1

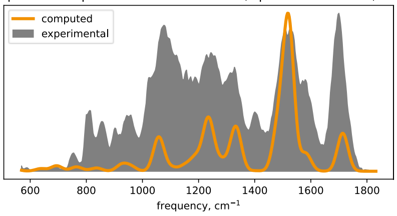


Structural similarity plot of deprotonated HMDB0001964

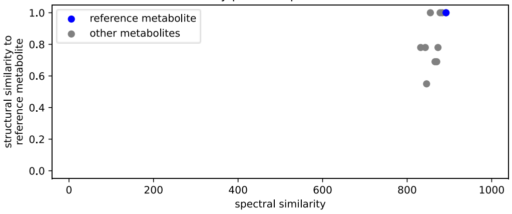


deprotonated HMDB0001964, spectral sim. = 892, #1

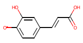

9.1 kJ/mol
Caffeic acid

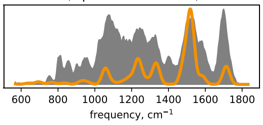


deprotonated HMDB0001964, spectral sim. = 883, #2

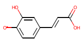

12.0 kJ/mol
Caffeic acid

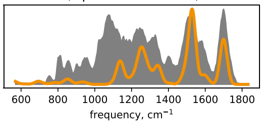


deprotonated HMDB0001964, spectral sim. = 878, #3

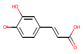

0.0 kJ/mol
Caffeic acid

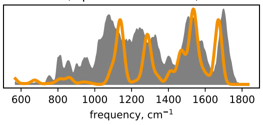


deprotonated HMDB0001964, spectral sim. = 873, #4

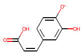

18.9 kJ/mol
Caffeic acid

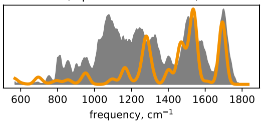


deprotonated HMDB0001964, spectral sim. = 870, #5

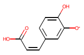

46.1 kJ/mol
Caffeic acid

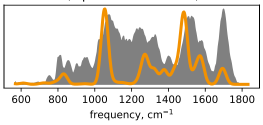


deprotonated HMDB0001964, spectral sim. = 866, #6

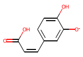

23.8 kJ/mol
Caffeic acid

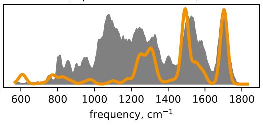


deprotonated HMDB0001964, spectral sim. = 855, #7

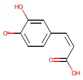

29.0 kJ/mol
Caffeic acid

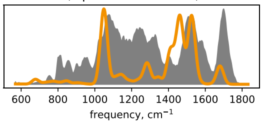


deprotonated HMDB0001964, spectral sim. = 846, #8

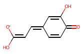

43.6 kJ/mol
Caffeic acid

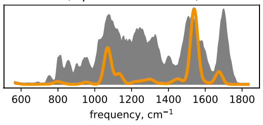


deprotonated HMDB0001964, spectral sim. = 843, #9

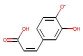

34.0 kJ/mol
Caffeic acid

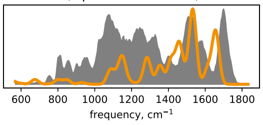


deprotonated HMDB0001964, spectral sim. = 832, #10

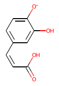

31.4 kJ/mol
Caffeic acid

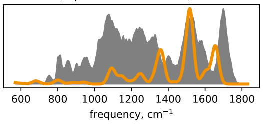


141 protonated HMDB0001964

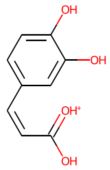


Caffeic acid

Spectra of protonated HMDB0001964, spectral sim. = 911, #1

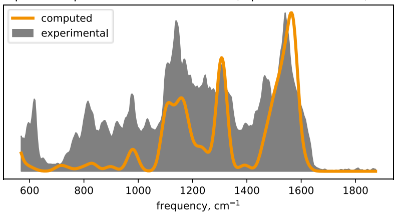


Structural similarity plot of protonated HMDB0001964

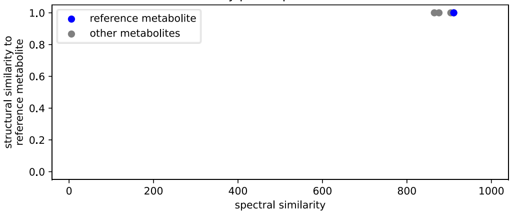


protonated HMDB0001964, spectral sim. = 911, #1

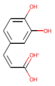

0.0 kJ/mol
Caffeic acid

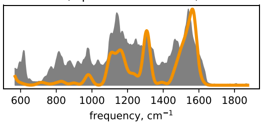


protonated HMDB0001964, spectral sim. = 904, #2

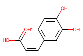

3.2 kJ/mol
Caffeic acid

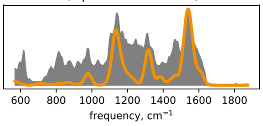


protonated HMDB0001964, spectral sim. = 876, #3

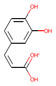

12.9 kJ/mol
Caffeic acid

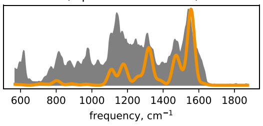


protonated HMDB0001964, spectral sim. = 865, #4

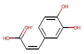

14.3 kJ/mol
Caffeic acid

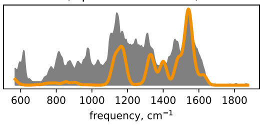

142 sodiated HMDB0001964

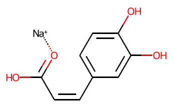


Caffeic acid

Spectra of sodiated HMDB0001964, spectral sim. = 945, #1

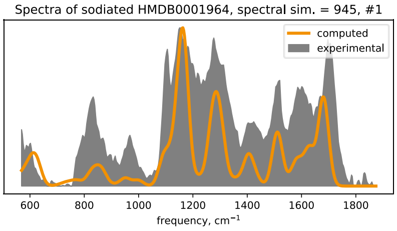


Structural similarity plot of sodiated HMDB0001964

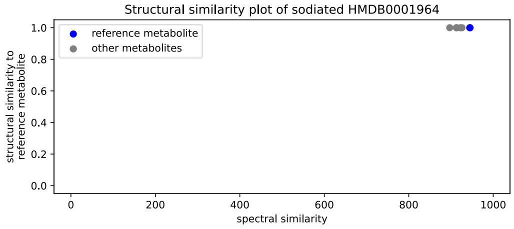


sodiated HMDB0001964, spectral sim. = 945, #1

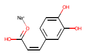

0.5 kJ/mol
Caffeic acid

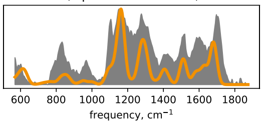


sodiated HMDB0001964, spectral sim. = 944, #2

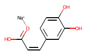

0.0 kJ/mol
Caffeic acid

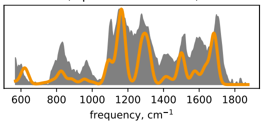


sodiated HMDB0001964, spectral sim. = 926, #3

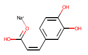

17.5 kJ/mol
Caffeic acid

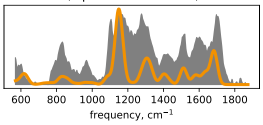


sodiated HMDB0001964, spectral sim. = 922, #4

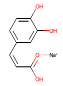

0.7 kJ/mol
Caffeic acid

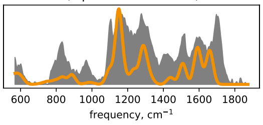


sodiated HMDB0001964, spectral sim. = 913, #5

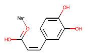

34.4 kJ/mol
Caffeic acid

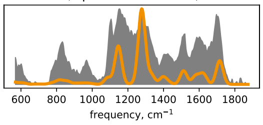


sodiated HMDB0001964, spectral sim. = 913, #6

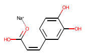

19.6 kJ/mol
Caffeic acid

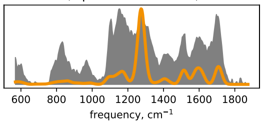


sodiated HMDB0001964, spectral sim. = 897, #7

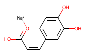

16.9 kJ/mol
Caffeic acid

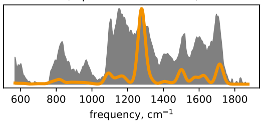


143 protonated HMDB0002266

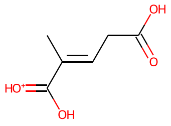


(E)-2-Methylglutaconic acid

Spectra of protonated HMDB0002266, spectral sim. = 949, #1

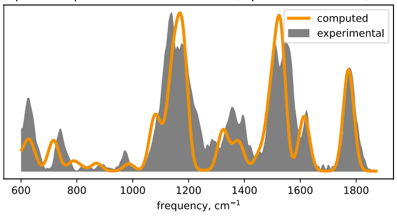


Structural similarity plot of protonated HMDB0002266

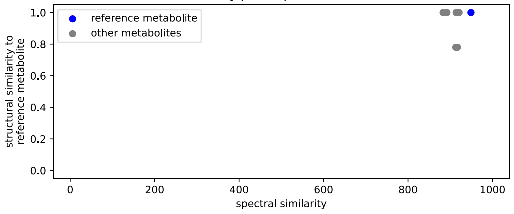


protonated HMDB0002266, spectral sim. = 949, #1

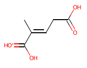

0.0 kJ/mol
(E)-2-
Methylglutaconic acid

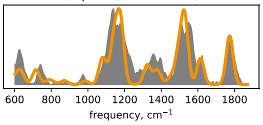


protonated HMDB0002266, spectral sim. = 921, #2

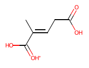

17.4 kJ/mol
(E)-2-
Methylglutaconic acid

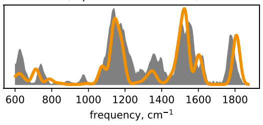


protonated HMDB0002266, spectral sim. = 917, #3

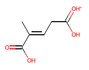

11.1 kJ/mol
(E)-2-
Methylglutaconic acid

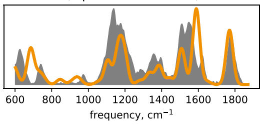


protonated HMDB0002266, spectral sim. = 914, #4

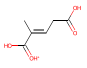

32.7 kJ/mol
(E)-2-
Methylglutaconic acid

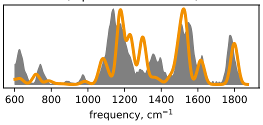


protonated HMDB0002266, spectral sim. = 913, #5

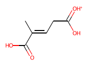

10.9 kJ/mol
(E)-2-
Methylglutaconic acid

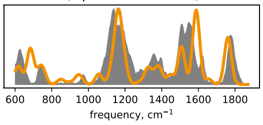


protonated HMDB0002266, spectral sim. = 892, #6

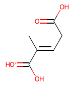

9.1 kJ/mol
(E)-2-
Methylglutaconic acid

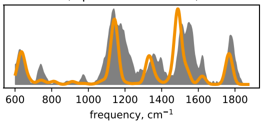


protonated HMDB0002266, spectral sim. = 883, #7

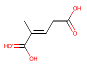

7.6 kJ/mol
(E)-2-
Methylglutaconic acid

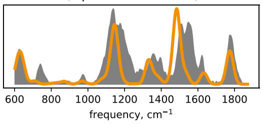

144 deprotonated HMDB0002285

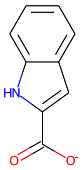


2-Indolecarboxylic acid

Spectra of deprotonated HMDB0002285, spectral sim. = 836, #1

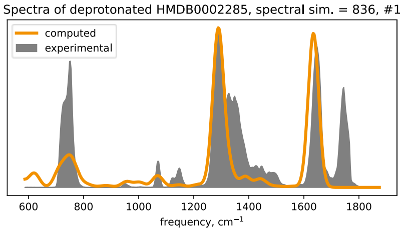


Structural similarity plot of deprotonated HMDB0002285

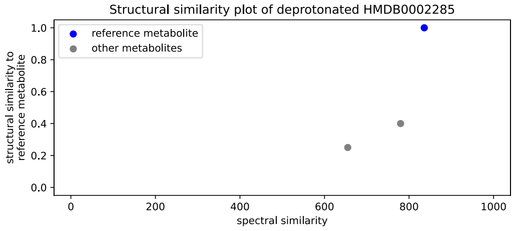


deprotonated HMDB0002285, spectral sim. = 836, #1

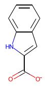

10.4 kJ/mol
2-Indolecarboxylic acid

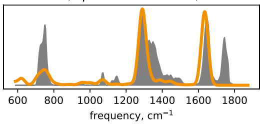


deprotonated HMDB0002285, spectral sim. = 780, #2

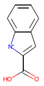

0.0 kJ/mol
2-Indolecarboxylic acid

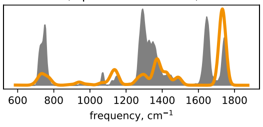


deprotonated HMDB0002285, spectral sim. = 655, #3

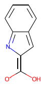

36.3 kJ/mol
2-Indolecarboxylic acid

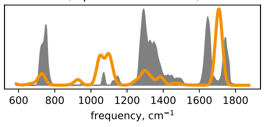


145 protonated HMDB0002285

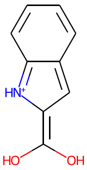


2-Indolecarboxylic acid

Spectra of protonated HMDB0002285, spectral sim. = 903, #1

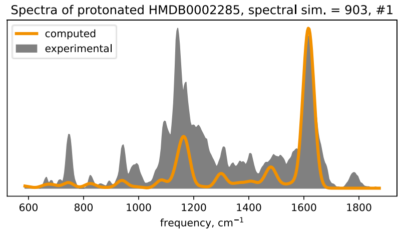


Structural similarity plot of protonated HMDB0002285

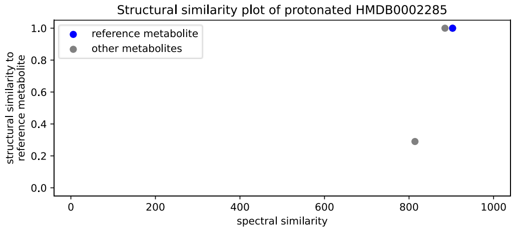


protonated HMDB0002285, spectral sim. = 903, #1

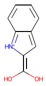

0.0 kJ/mol
2-Indolecarboxylic acid

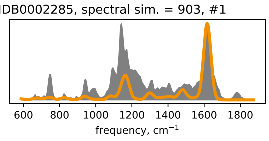


protonated HMDB0002285, spectral sim. = 885, #2

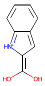

16.4 kJ/mol
2-Indolecarboxylic acid

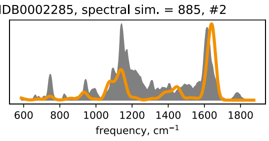


protonated HMDB0002285, spectral sim. = 814, #3

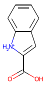

17.0 kJ/mol
2-Indolecarboxylic acid

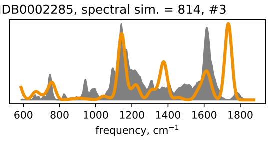


146 deprotonated HMDB0002302

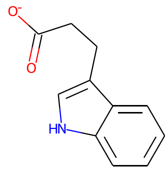


Indole-3-propionic acid

Spectra of deprotonated HMDB0002302, spectral sim. = 850, #1

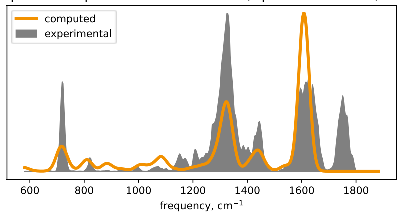


Structural similarity plot of deprotonated HMDB0002302

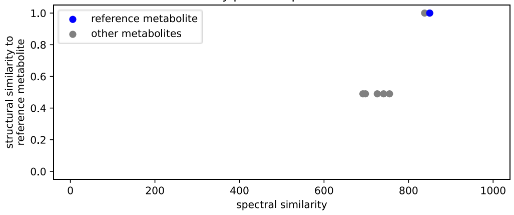


deprotonated HMDB0002302, spectral sim. = 850, #1

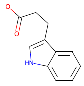

22.7 kJ/mol
Indole-3-propionic acid

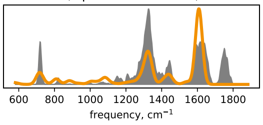


deprotonated HMDB0002302, spectral sim. = 838, #2

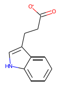

32.6 kJ/mol
Indole-3-propionic acid

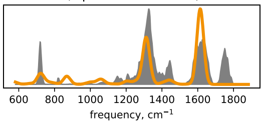


deprotonated HMDB0002302, spectral sim. = 755, #3

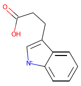

0.0 kJ/mol
Indole-3-propionic acid

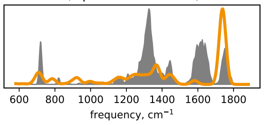


deprotonated HMDB0002302, spectral sim. = 741, #4

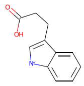

4.0 kJ/mol
Indole-3-propionic acid

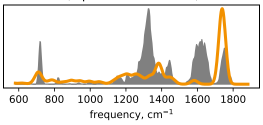


deprotonated HMDB0002302, spectral sim. = 726, #5

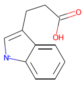

29.1 kJ/mol
Indole-3-propionic acid

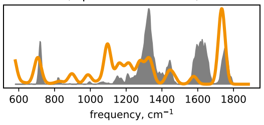


deprotonated HMDB0002302, spectral sim. = 698, #6

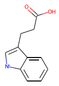

25.1 kJ/mol
Indole-3-propionic acid

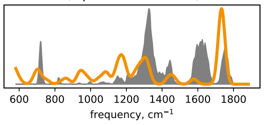


deprotonated HMDB0002302, spectral sim. = 692, #7

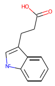

25.5 kJ/mol
Indole-3-propionic acid

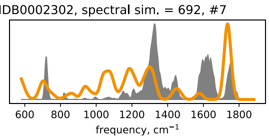

147 protonated HMDB0002302

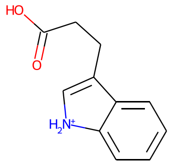


Indole-3-propionic acid

Spectra of protonated HMDB0002302, spectral sim. = 731, #1

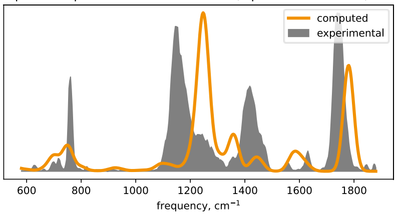


Structural similarity plot of protonated HMDB0002302

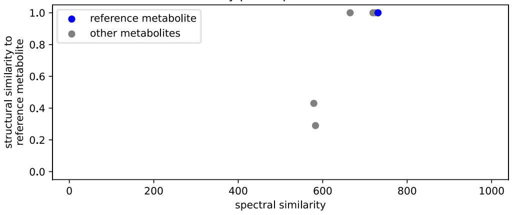


protonated HMDB0002302, spectral sim. = 731, #1

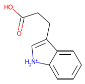

35.7 kJ/mol
Indole-3-propionic acid

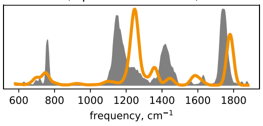


protonated HMDB0002302, spectral sim. = 725, #2

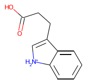

43.6 kJ/mol
Indole-3-propionic acid

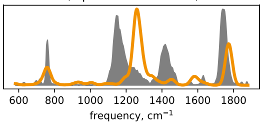


protonated HMDB0002302, spectral sim. = 719, #3

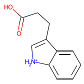

33.7 kJ/mol
Indole-3-propionic acid

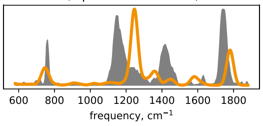


protonated HMDB0002302, spectral sim. = 665, #4

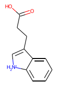

52.7 kJ/mol
Indole-3-propionic acid

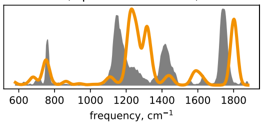


protonated HMDB0002302, spectral sim. = 583, #5

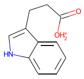

46.0 kJ/mol
Indole-3-propionic acid

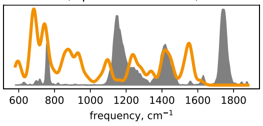


protonated HMDB0002302, spectral sim. = 579, #6

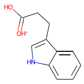

0.0 kJ/mol
Indole-3-propionic acid

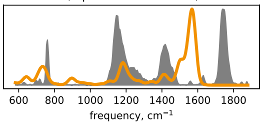


148 sodiated HMDB0002302

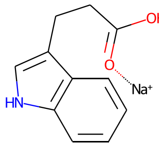


Indole-3-propionic acid

Spectra of sodiated HMDB0002302, spectral sim. = 862, #1

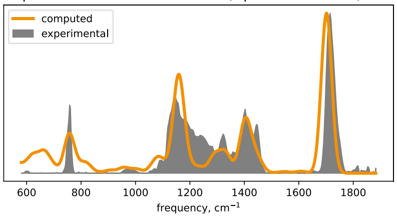


Structural similarity plot of sodiated HMDB0002302

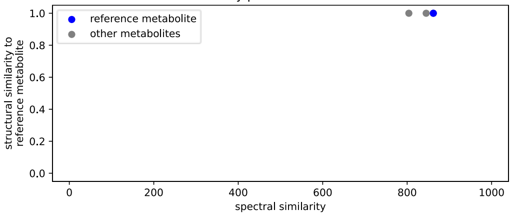


sodiated HMDB0002302, spectral sim. = 862, #1

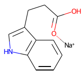

0.0 kJ/mol
Indole-3-propionic acid

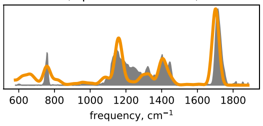


sodiated HMDB0002302, spectral sim. = 845, #2

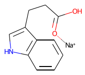

10.4 kJ/mol
Indole-3-propionic acid

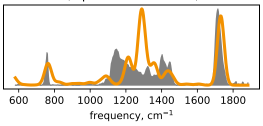


sodiated HMDB0002302, spectral sim. = 804, #3

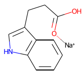

12.6 kJ/mol
Indole-3-propionic acid

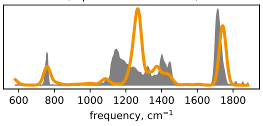


149 deprotonated HMDB0002432

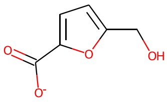


Sumiki's acid

Spectra of deprotonated HMDB0002432, spectral sim. = 924, #1

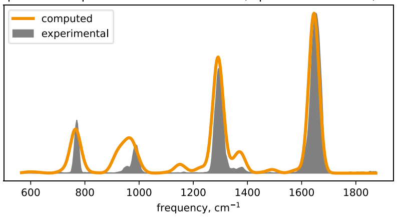


Structural similarity plot of deprotonated HMDB0002432

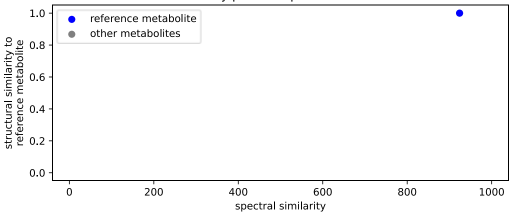


deprotonated HMDB0002432, spectral sim. = 924, #1

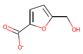

0.0 kJ/mol
Sumiki's acid

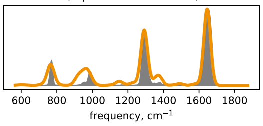

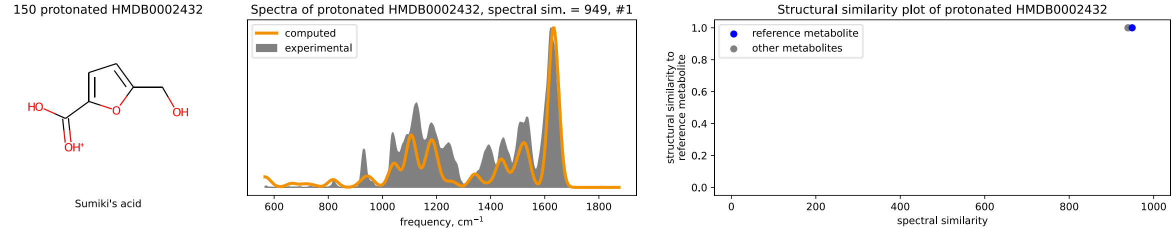
150 protonated HMDB0002432
Sumiki's acid
Spectra of protonated HMDB0002432, spectral sim. = 949, #1
computed
experimental
frequency, cm$^{-1}$
Structural similarity plot of protonated HMDB0002432
reference metabolite
other metabolites
structural similarity to reference metabolite
spectral similarity

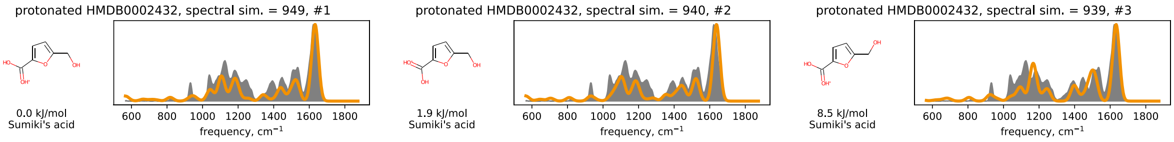
protonated HMDB0002432, spectral sim. = 949, #1
0.0 kJ/mol
Sumiki's acid
protonated HMDB0002432, spectral sim. = 940, #2
1.9 kJ/mol
Sumiki's acid
protonated HMDB0002432, spectral sim. = 939, #3
8.5 kJ/mol
Sumiki's acid
frequency, cm$^{-1}$

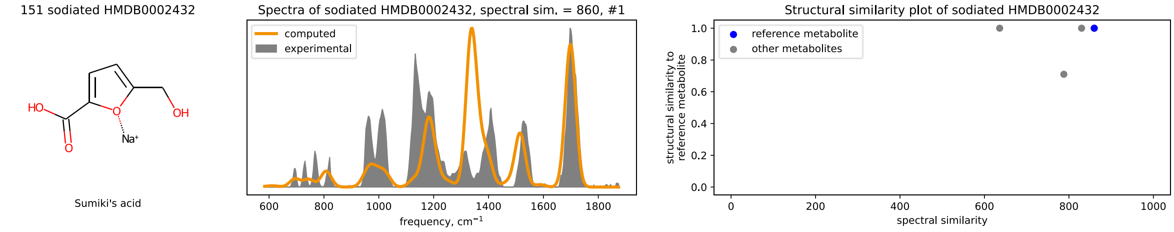
151 sodiated HMDB0002432
Sumiki's acid
Spectra of sodiated HMDB0002432, spectral sim. = 860, #1
computed
experimental
frequency, cm$^{-1}$
Structural similarity plot of sodiated HMDB0002432
reference metabolite
other metabolites
structural similarity to reference metabolite
spectral similarity

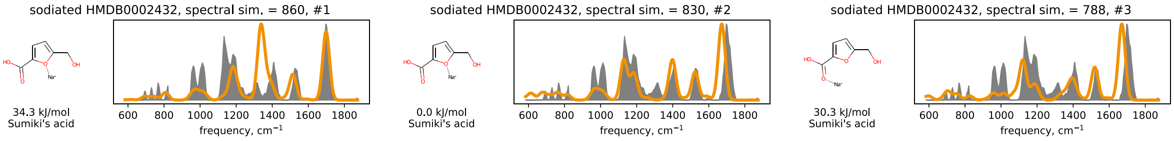
sodiated HMDB0002432, spectral sim. = 860, #1
34.3 kJ/mol
Sumiki's acid
sodiated HMDB0002432, spectral sim. = 830, #2
0.0 kJ/mol
Sumiki's acid
sodiated HMDB0002432, spectral sim. = 788, #3
30.3 kJ/mol
Sumiki's acid
frequency, cm$^{-1}$

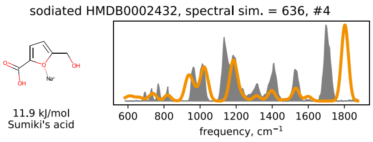
sodiated HMDB0002432, spectral sim. = 636, #4
11.9 kJ/mol
Sumiki's acid
frequency, cm$^{-1}$

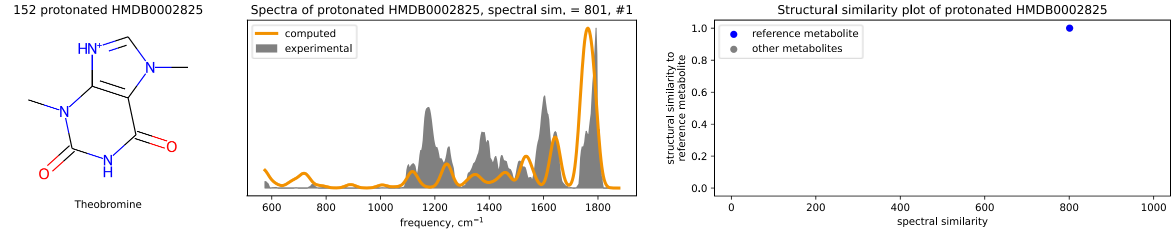
152 protonated HMDB0002825
Theobromine
Spectra of protonated HMDB0002825, spectral sim. = 801, #1
computed
experimental
frequency, cm$^{-1}$
Structural similarity plot of protonated HMDB0002825
reference metabolite
other metabolites
structural similarity to reference metabolite
spectral similarity

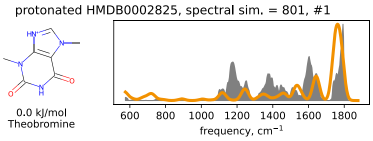
protonated HMDB0002825, spectral sim. = 801, #1
0.0 kJ/mol
Theobromine
frequency, cm$^{-1}$

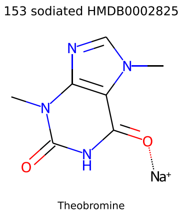
153 sodiated HMDB0002825
Na+
Theobromine

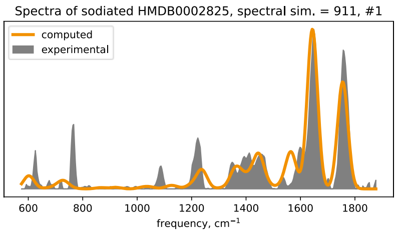
Spectra of sodiated HMDB0002825, spectral sim. = 911, #1
computed
experimental
frequency, cm−1

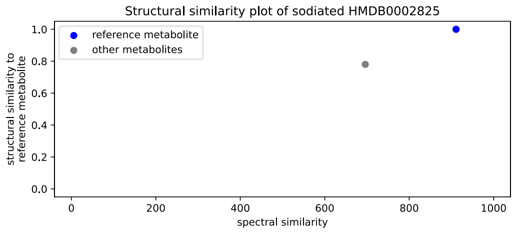
Structural similarity plot of sodiated HMDB0002825
reference metabolite
other metabolites
structural similarity to reference metabolite
spectral similarity

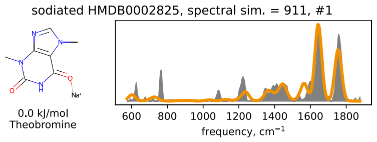
sodiated HMDB0002825, spectral sim. = 911, #1
0.0 kJ/mol
Theobromine
frequency, cm−1

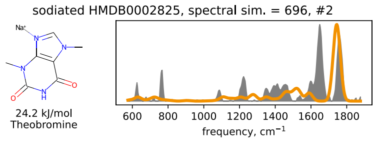
sodiated HMDB0002825, spectral sim. = 696, #2
24.2 kJ/mol
Theobromine
frequency, cm−1

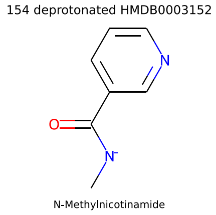
154 deprotonated HMDB0003152
N-Methylnicotinamide

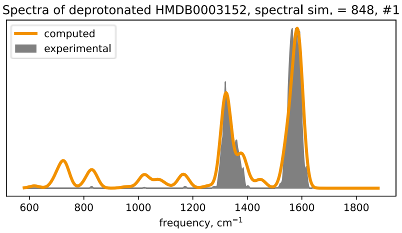
Spectra of deprotonated HMDB0003152, spectral sim. = 848, #1
computed
experimental
frequency, cm−1

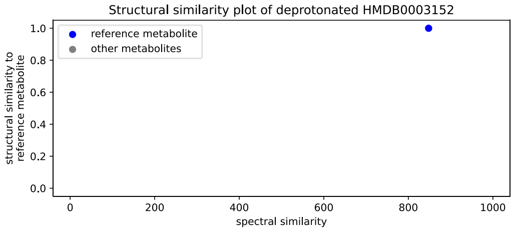
Structural similarity plot of deprotonated HMDB0003152
reference metabolite
other metabolites
structural similarity to reference metabolite
spectral similarity

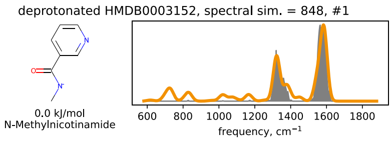
deprotonated HMDB0003152, spectral sim. = 848, #1
0.0 kJ/mol
N-Methylnicotinamide
frequency, cm−1

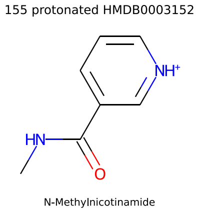
155 protonated HMDB0003152
NH+
HN
N-Methylnicotinamide

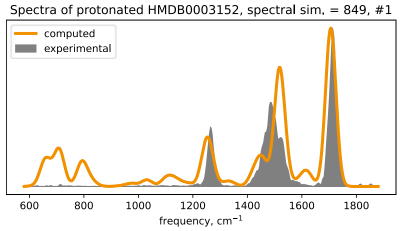
Spectra of protonated HMDB0003152, spectral sim. = 849, #1
computed
experimental
frequency, cm−1

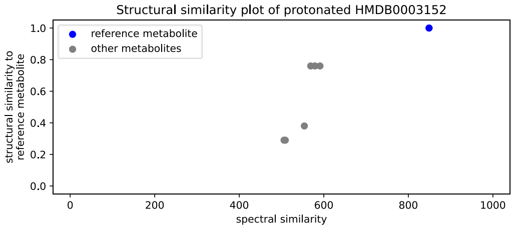
Structural similarity plot of protonated HMDB0003152
reference metabolite
other metabolites
structural similarity to reference metabolite
spectral similarity

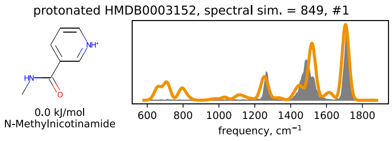
protonated HMDB0003152, spectral sim. = 849, #1
0.0 kJ/mol
N-Methylnicotinamide
frequency, cm−1

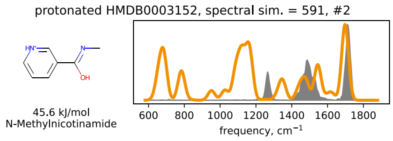
protonated HMDB0003152, spectral sim. = 591, #2
45.6 kJ/mol
N-Methylnicotinamide
frequency, cm−1

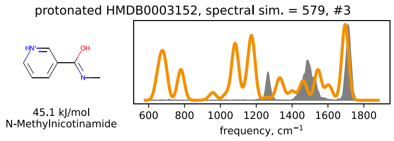
protonated HMDB0003152, spectral sim. = 579, #3
45.1 kJ/mol
N-Methylnicotinamide
frequency, cm−1

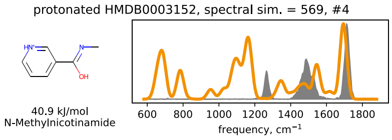
protonated HMDB0003152, spectral sim. = 569, #4
40.9 kJ/mol
N-Methylnicotinamide
frequency, cm−1

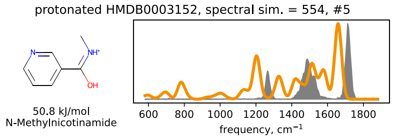
protonated HMDB0003152, spectral sim. = 554, #5
50.8 kJ/mol
N-Methylnicotinamide
frequency, cm−1

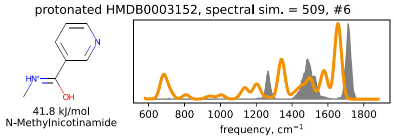
protonated HMDB0003152, spectral sim. = 509, #6
41.8 kJ/mol
N-Methylnicotinamide
frequency, cm−1

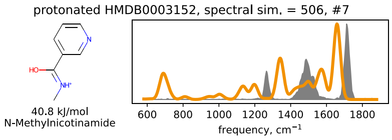
protonated HMDB0003152, spectral sim. = 506, #7
40.8 kJ/mol
N-Methylnicotinamide
frequency, cm−1

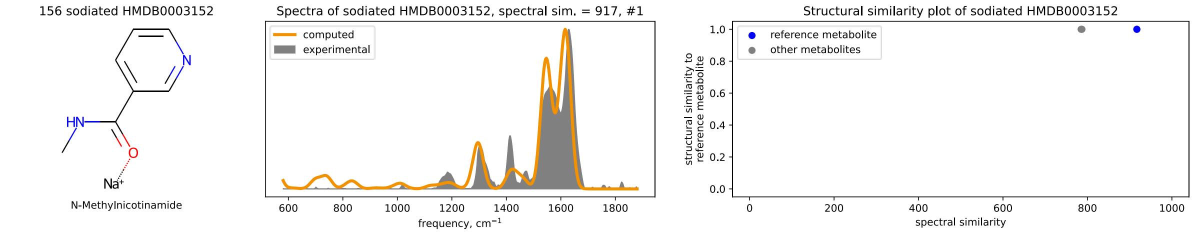
156 sodiated HMDB0003152
N-Methylnicotinamide
Spectra of sodiated HMDB0003152, spectral sim. = 917, #1
computed
experimental
frequency, cm⁻¹
Structural similarity plot of sodiated HMDB0003152
reference metabolite
other metabolites
structural similarity to reference metabolite
spectral similarity

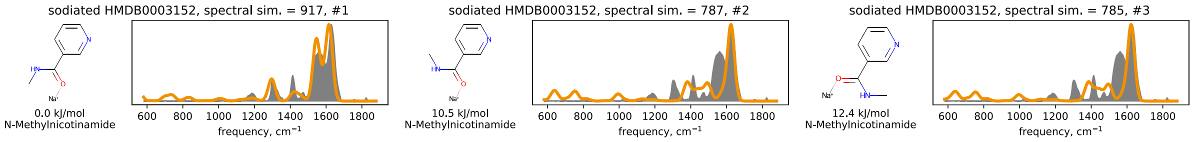
sodiated HMDB0003152, spectral sim. = 917, #1
0.0 kJ/mol
N-Methylnicotinamide
sodiated HMDB0003152, spectral sim. = 787, #2
10.5 kJ/mol
N-Methylnicotinamide
sodiated HMDB0003152, spectral sim. = 785, #3
12.4 kJ/mol
N-Methylnicotinamide
frequency, cm⁻¹

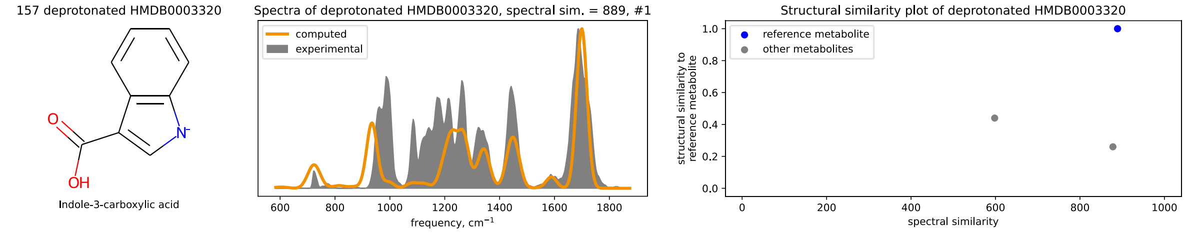
157 deprotonated HMDB0003320
Indole-3-carboxylic acid
Spectra of deprotonated HMDB0003320, spectral sim. = 889, #1
computed
experimental
frequency, cm⁻¹
Structural similarity plot of deprotonated HMDB0003320
reference metabolite
other metabolites
structural similarity to reference metabolite
spectral similarity

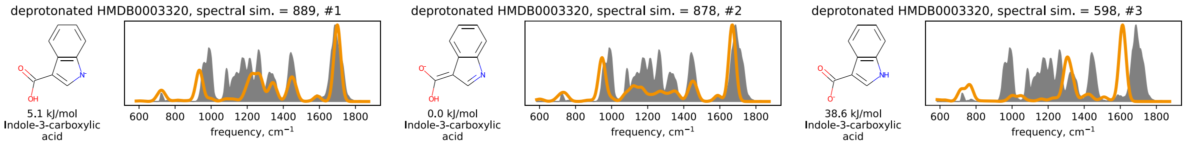
deprotonated HMDB0003320, spectral sim. = 889, #1
5.1 kJ/mol
Indole-3-carboxylic acid
deprotonated HMDB0003320, spectral sim. = 878, #2
0.0 kJ/mol
Indole-3-carboxylic acid
deprotonated HMDB0003320, spectral sim. = 598, #3
38.6 kJ/mol
Indole-3-carboxylic acid
frequency, cm⁻¹

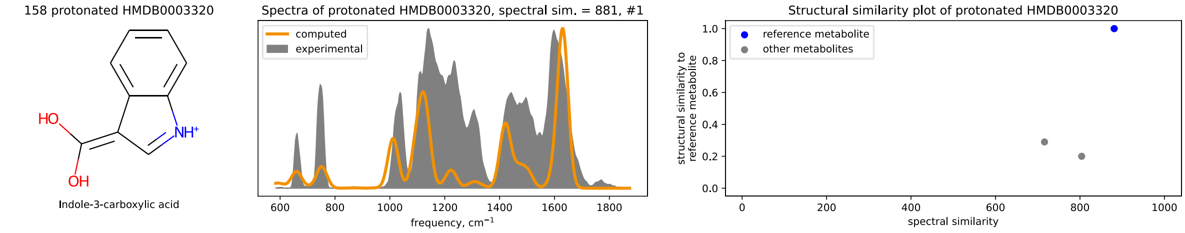
158 protonated HMDB0003320
Indole-3-carboxylic acid
Spectra of protonated HMDB0003320, spectral sim. = 881, #1
computed
experimental
frequency, cm⁻¹
Structural similarity plot of protonated HMDB0003320
reference metabolite
other metabolites
structural similarity to reference metabolite
spectral similarity

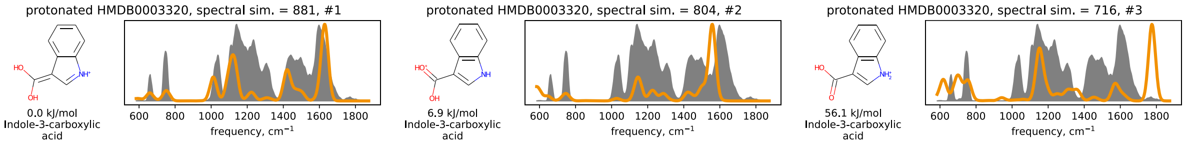
protonated HMDB0003320, spectral sim. = 881, #1
0.0 kJ/mol
Indole-3-carboxylic acid
protonated HMDB0003320, spectral sim. = 804, #2
6.9 kJ/mol
Indole-3-carboxylic acid
protonated HMDB0003320, spectral sim. = 716, #3
56.1 kJ/mol
Indole-3-carboxylic acid
frequency, cm⁻¹

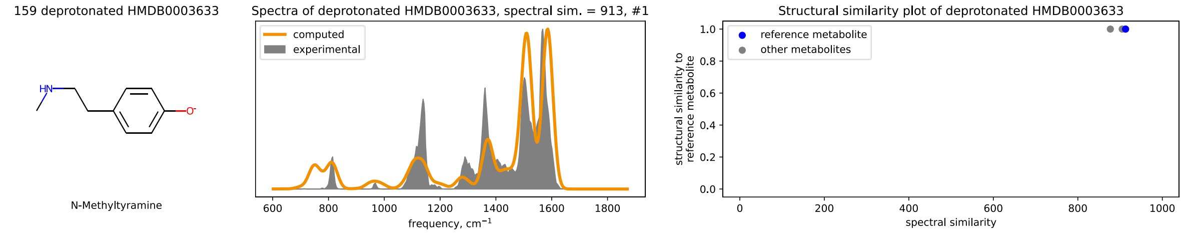
159 deprotonated HMDB0003633
N-Methyltyramine
Spectra of deprotonated HMDB0003633, spectral sim. = 913, #1
computed
experimental
frequency, cm⁻¹
Structural similarity plot of deprotonated HMDB0003633
reference metabolite
other metabolites
structural similarity to reference metabolite
spectral similarity

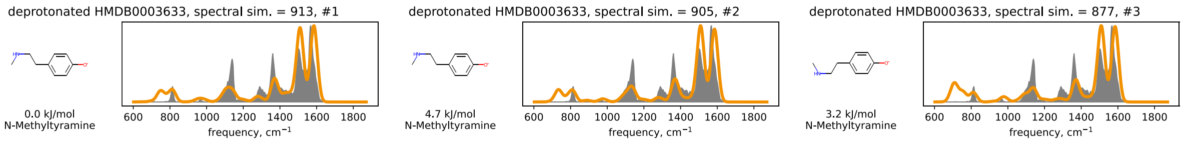
deprotonated HMDB0003633, spectral sim. = 913, #1
0.0 kJ/mol
N-Methyltyramine
deprotonated HMDB0003633, spectral sim. = 905, #2
4.7 kJ/mol
N-Methyltyramine
deprotonated HMDB0003633, spectral sim. = 877, #3
3.2 kJ/mol
N-Methyltyramine
frequency, cm⁻¹

160 protonated HMDB0003633

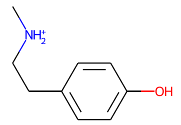


N-Methyltyramine

Spectra of protonated HMDB0003633, spectral sim. = 911, #1

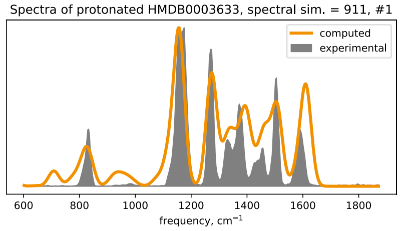


Structural similarity plot of protonated HMDB0003633

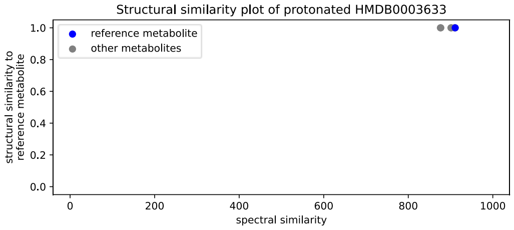


protonated HMDB0003633, spectral sim. = 911, #1

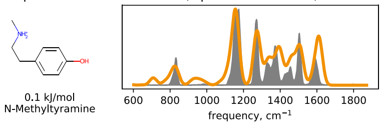


protonated HMDB0003633, spectral sim. = 909, #2

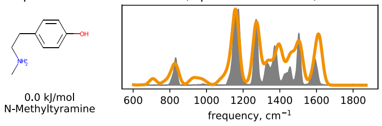


protonated HMDB0003633, spectral sim. = 902, #3

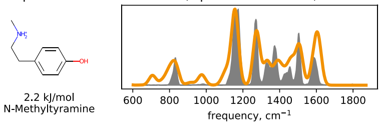


protonated HMDB0003633, spectral sim. = 901, #4

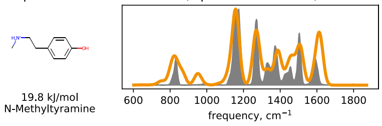


protonated HMDB0003633, spectral sim. = 877, #5

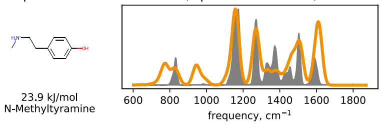


protonated HMDB0003633, spectral sim. = 876, #6

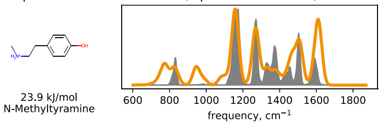


161 deprotonated HMDB0004095

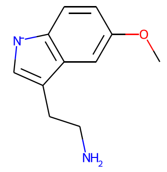


5-Methoxytryptamine

Spectra of deprotonated HMDB0004095, spectral sim. = 954, #1

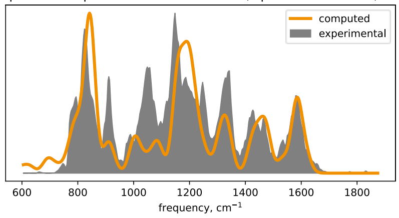


Structural similarity plot of deprotonated HMDB0004095

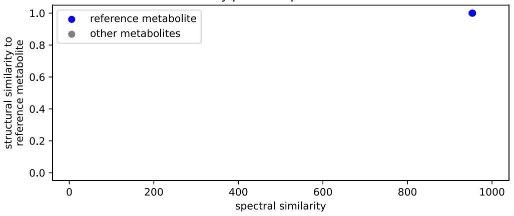


deprotonated HMDB0004095, spectral sim. = 954, #1

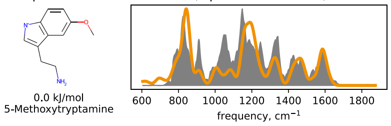


deprotonated HMDB0004095, spectral sim. = 952, #2

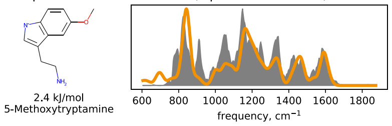


162 protonated HMDB0004095

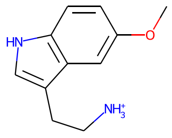


5-Methoxytryptamine

Spectra of protonated HMDB0004095, spectral sim. = 961, #1

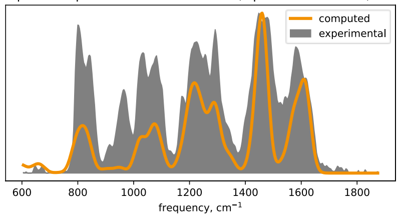


Structural similarity plot of protonated HMDB0004095

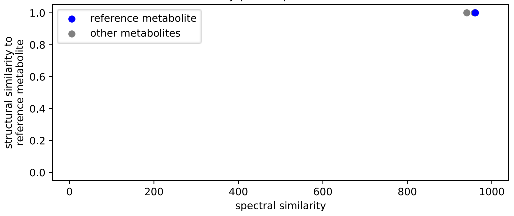


protonated HMDB0004095, spectral sim. = 961, #1

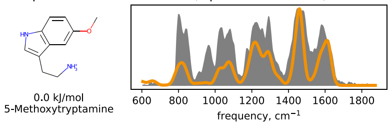


protonated HMDB0004095, spectral sim. = 959, #2

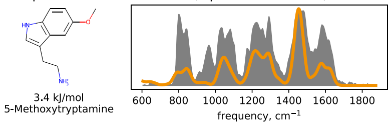


protonated HMDB0004095, spectral sim. = 941, #3

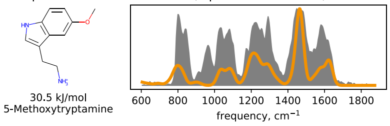

163 sodiated HMDB0004095

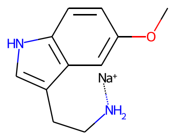

5-Methoxytryptamine

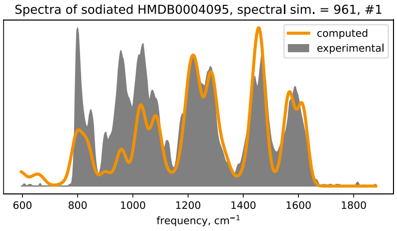


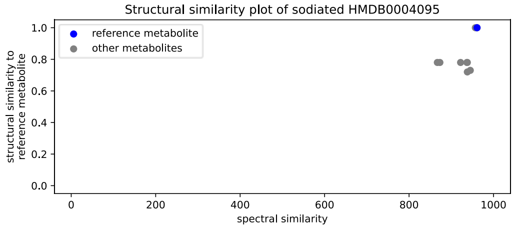


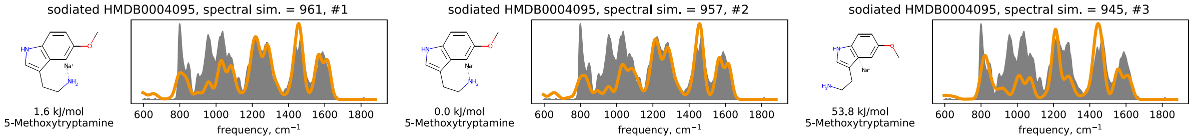


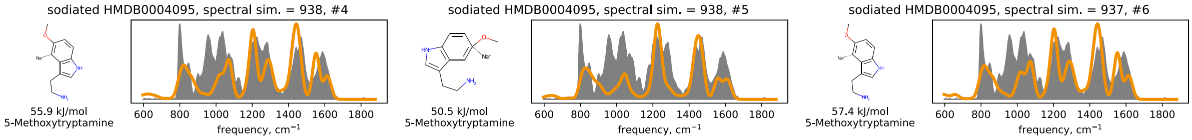


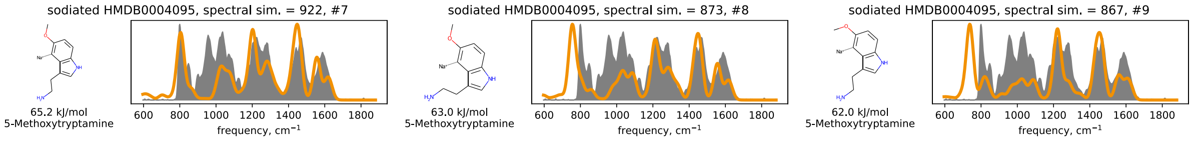


164 deprotonated HMDB0005807

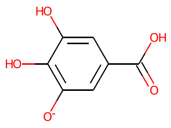

Gallic acid

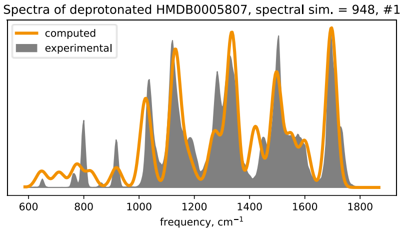


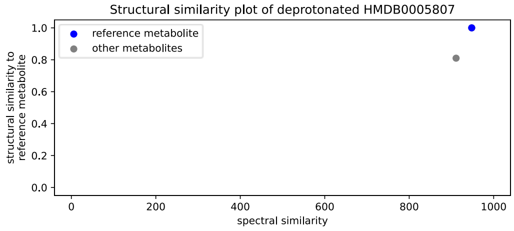


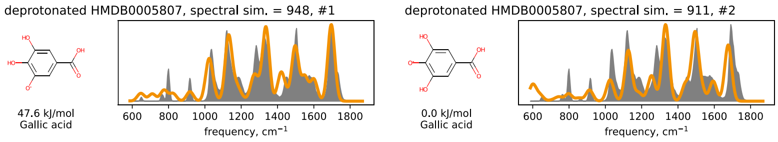


165 protonated HMDB0005807

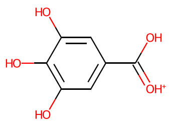

Gallic acid

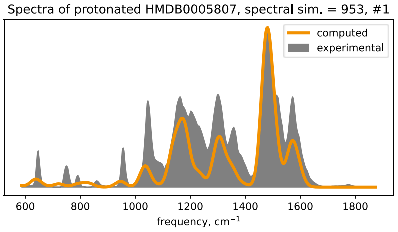


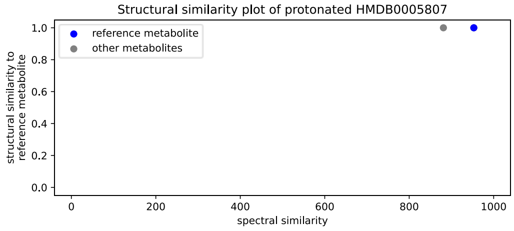


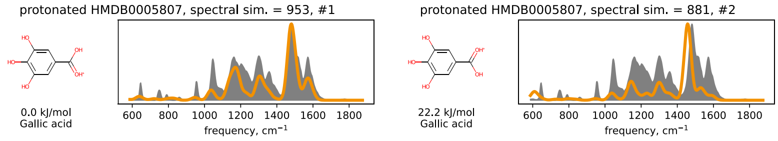

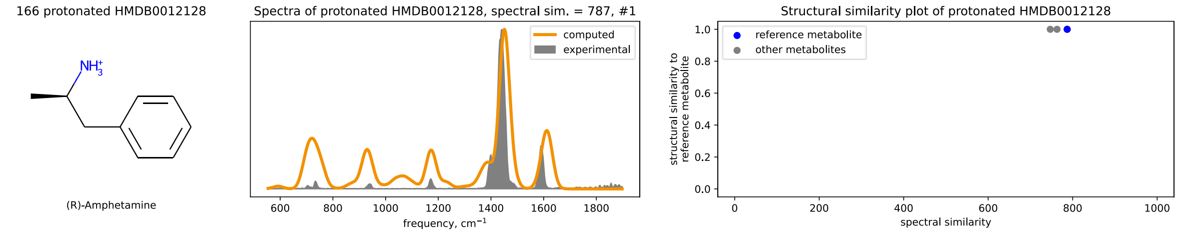
166 protonated HMDB0012128
(R)-Amphetamine
Spectra of protonated HMDB0012128, spectral sim. = 787, #1
computed
experimental
frequency, cm$^{-1}$
Structural similarity plot of protonated HMDB0012128
reference metabolite
other metabolites
structural similarity to reference metabolite
spectral similarity

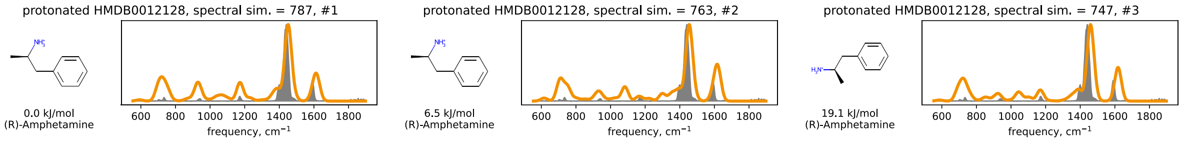
protonated HMDB0012128, spectral sim. = 787, #1
0.0 kJ/mol
(R)-Amphetamine
protonated HMDB0012128, spectral sim. = 763, #2
6.5 kJ/mol
(R)-Amphetamine
protonated HMDB0012128, spectral sim. = 747, #3
19.1 kJ/mol
(R)-Amphetamine
frequency, cm$^{-1}$

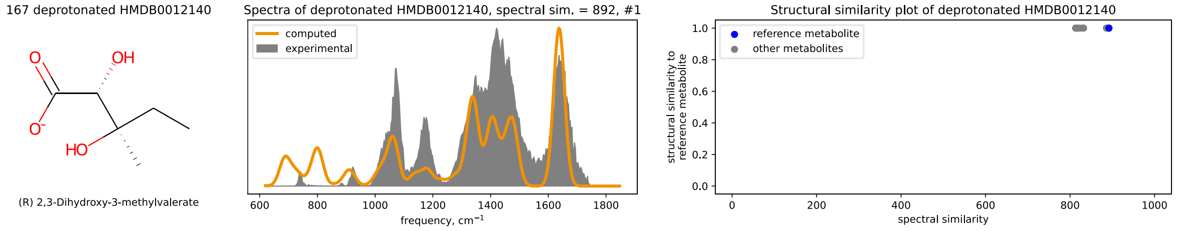
167 deprotonated HMDB0012140
(R) 2,3-Dihydroxy-3-methylvalerate
Spectra of deprotonated HMDB0012140, spectral sim. = 892, #1
computed
experimental
frequency, cm$^{-1}$
Structural similarity plot of deprotonated HMDB0012140
reference metabolite
other metabolites
structural similarity to reference metabolite
spectral similarity

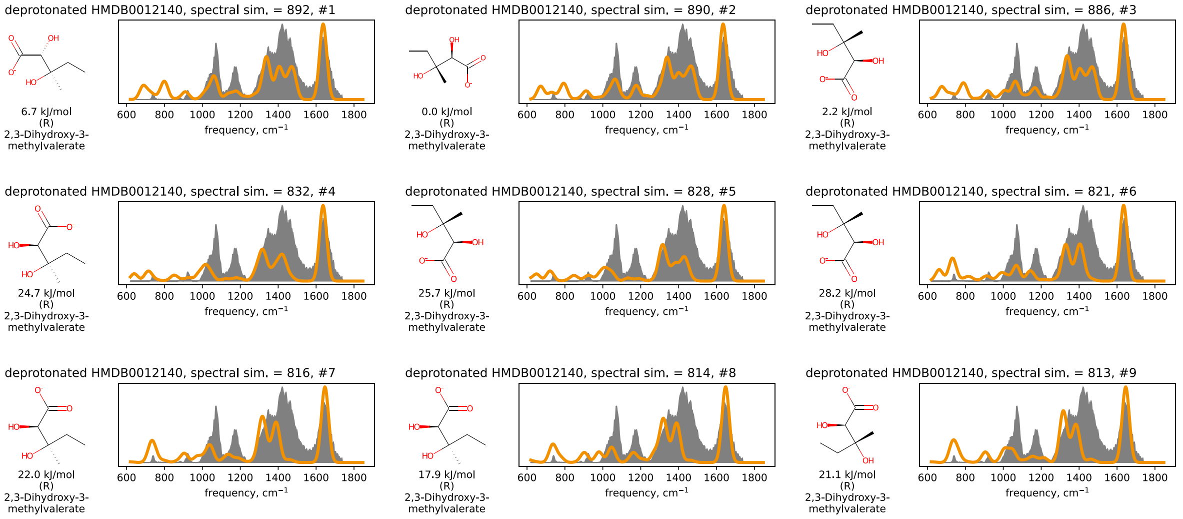
deprotonated HMDB0012140, spectral sim. = 892, #1
6.7 kJ/mol
(R)
2,3-Dihydroxy-3-methylvalerate
deprotonated HMDB0012140, spectral sim. = 890, #2
0.0 kJ/mol
(R)
2,3-Dihydroxy-3-methylvalerate
deprotonated HMDB0012140, spectral sim. = 886, #3
2.2 kJ/mol
(R)
2,3-Dihydroxy-3-methylvalerate
deprotonated HMDB0012140, spectral sim. = 832, #4
24.7 kJ/mol
(R)
2,3-Dihydroxy-3-methylvalerate
deprotonated HMDB0012140, spectral sim. = 828, #5
25.7 kJ/mol
(R)
2,3-Dihydroxy-3-methylvalerate
deprotonated HMDB0012140, spectral sim. = 821, #6
28.2 kJ/mol
(R)
2,3-Dihydroxy-3-methylvalerate
deprotonated HMDB0012140, spectral sim. = 816, #7
22.0 kJ/mol
(R)
2,3-Dihydroxy-3-methylvalerate
deprotonated HMDB0012140, spectral sim. = 814, #8
17.9 kJ/mol
(R)
2,3-Dihydroxy-3-methylvalerate
deprotonated HMDB0012140, spectral sim. = 813, #9
21.1 kJ/mol
(R)
2,3-Dihydroxy-3-methylvalerate
frequency, cm$^{-1}$

168 deprotonated HMDB0013318

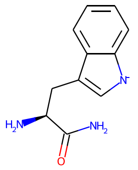


Spectra of deprotonated HMDB0013318, spectral sim. = 921, #1

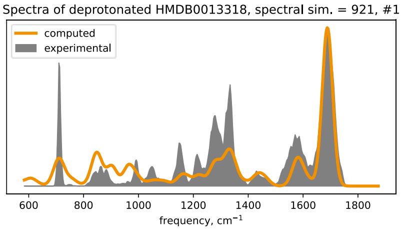


Structural similarity plot of deprotonated HMDB0013318

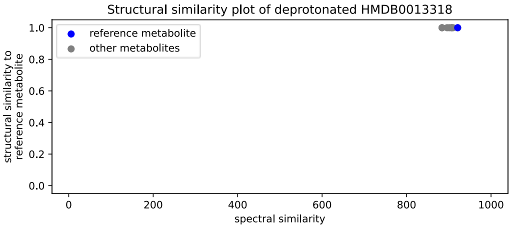


deprotonated HMDB0013318, spectral sim. = 921, #1

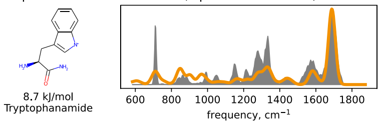


deprotonated HMDB0013318, spectral sim. = 910, #2

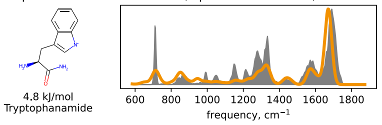


deprotonated HMDB0013318, spectral sim. = 907, #3

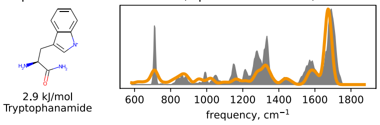


deprotonated HMDB0013318, spectral sim. = 906, #4

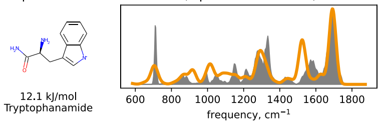


deprotonated HMDB0013318, spectral sim. = 901, #5

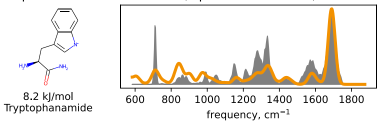


deprotonated HMDB0013318, spectral sim. = 896, #6

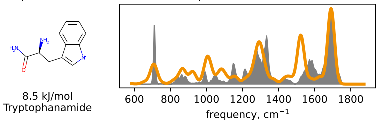


deprotonated HMDB0013318, spectral sim. = 884, #7

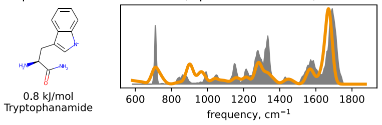


deprotonated HMDB0013318, spectral sim. = 884, #8

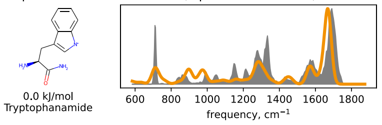


169 protonated HMDB0013318

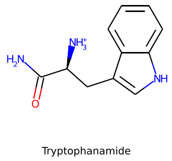


Spectra of protonated HMDB0013318, spectral sim. = 931, #1

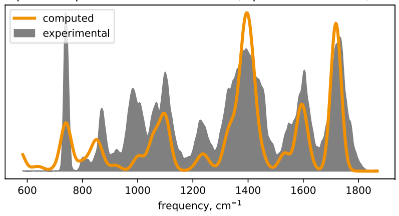


Structural similarity plot of protonated HMDB0013318

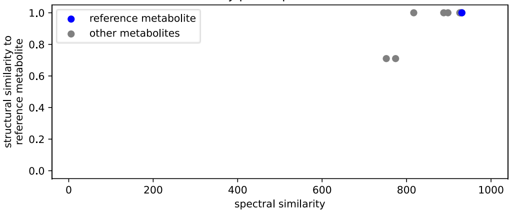


protonated HMDB0013318, spectral sim. = 931, #1

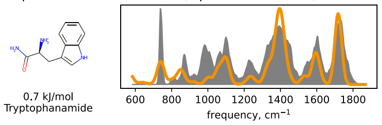


protonated HMDB0013318, spectral sim. = 927, #2

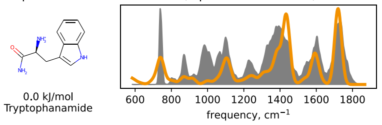


protonated HMDB0013318, spectral sim. = 926, #3

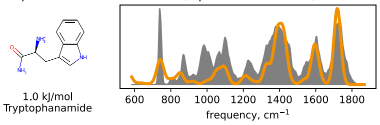


protonated HMDB0013318, spectral sim. = 898, #4

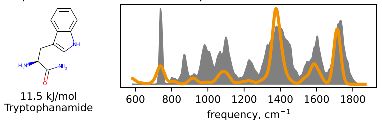


protonated HMDB0013318, spectral sim. = 888, #5

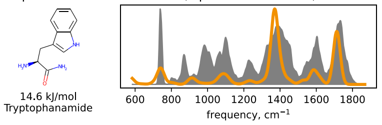


protonated HMDB0013318, spectral sim. = 817, #6

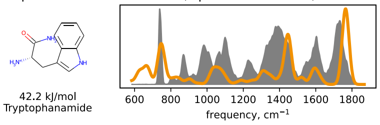


protonated HMDB0013318, spectral sim. = 774, #7

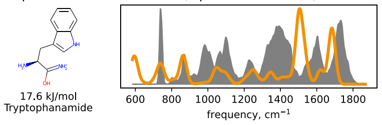


protonated HMDB0013318, spectral sim. = 752, #8

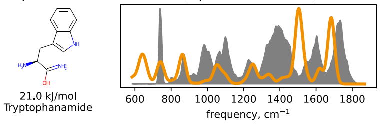

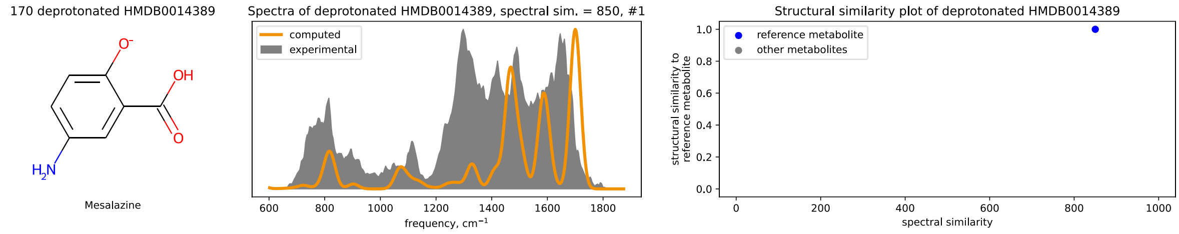
170 deprotonated HMDB0014389
Mesalazine
Spectra of deprotonated HMDB0014389, spectral sim. = 850, #1
computed
experimental
frequency, cm$^{-1}$
Structural similarity plot of deprotonated HMDB0014389
reference metabolite
other metabolites
structural similarity to reference metabolite
spectral similarity

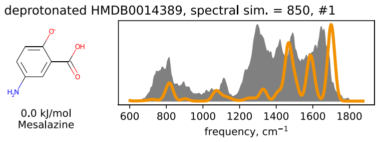
deprotonated HMDB0014389, spectral sim. = 850, #1
0.0 kJ/mol
Mesalazine
frequency, cm$^{-1}$

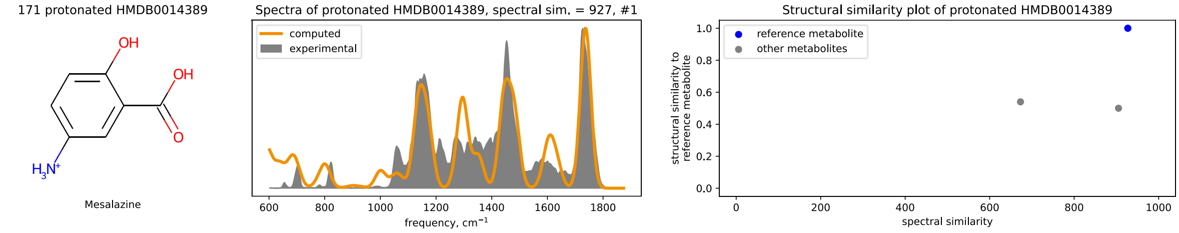
171 protonated HMDB0014389
Mesalazine
Spectra of protonated HMDB0014389, spectral sim. = 927, #1
computed
experimental
frequency, cm$^{-1}$
Structural similarity plot of protonated HMDB0014389
reference metabolite
other metabolites
structural similarity to reference metabolite
spectral similarity

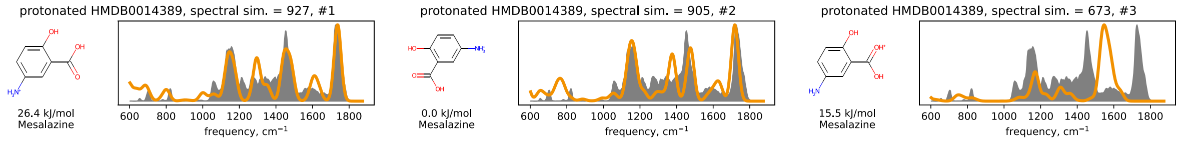
protonated HMDB0014389, spectral sim. = 927, #1
26.4 kJ/mol
Mesalazine
protonated HMDB0014389, spectral sim. = 905, #2
0.0 kJ/mol
Mesalazine
protonated HMDB0014389, spectral sim. = 673, #3
15.5 kJ/mol
Mesalazine
frequency, cm$^{-1}$

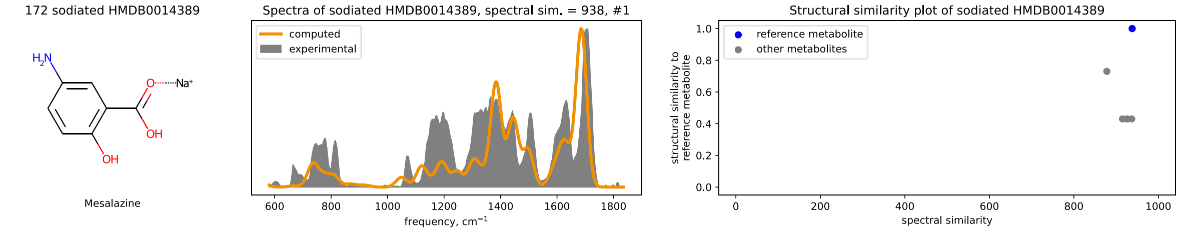
172 sodiated HMDB0014389
Mesalazine
Spectra of sodiated HMDB0014389, spectral sim. = 938, #1
computed
experimental
frequency, cm$^{-1}$
Structural similarity plot of sodiated HMDB0014389
reference metabolite
other metabolites
structural similarity to reference metabolite
spectral similarity

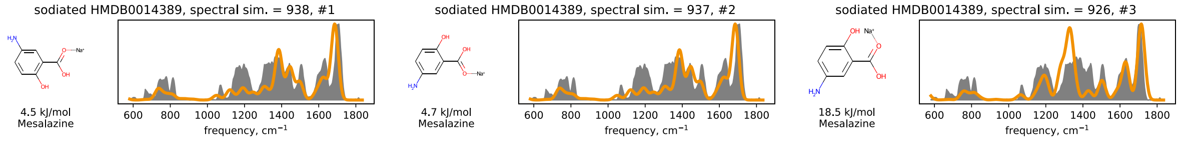
sodiated HMDB0014389, spectral sim. = 938, #1
4.5 kJ/mol
Mesalazine
sodiated HMDB0014389, spectral sim. = 937, #2
4.7 kJ/mol
Mesalazine
sodiated HMDB0014389, spectral sim. = 926, #3
18.5 kJ/mol
Mesalazine
frequency, cm$^{-1}$

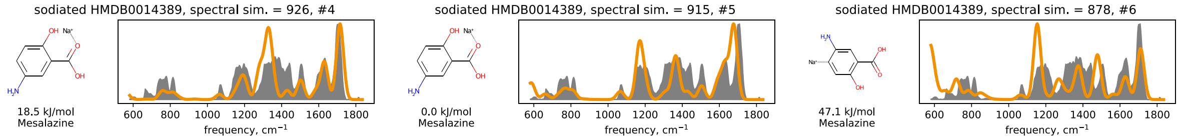
sodiated HMDB0014389, spectral sim. = 926, #4
18.5 kJ/mol
Mesalazine
sodiated HMDB0014389, spectral sim. = 915, #5
0.0 kJ/mol
Mesalazine
sodiated HMDB0014389, spectral sim. = 878, #6
47.1 kJ/mol
Mesalazine
frequency, cm$^{-1}$

173 protonated HMDB0015517

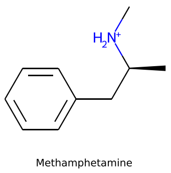


Spectra of protonated HMDB0015517, spectral sim. = 740, #1

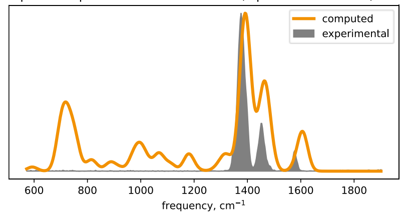


Structural similarity plot of protonated HMDB0015517

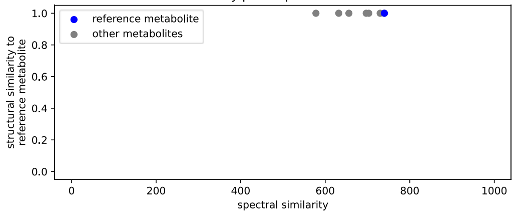


protonated HMDB0015517, spectral sim. = 740, #1

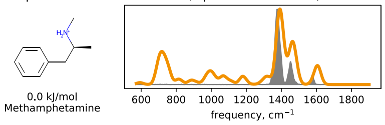


protonated HMDB0015517, spectral sim. = 730, #2

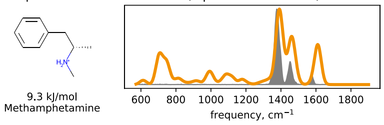


protonated HMDB0015517, spectral sim. = 703, #3

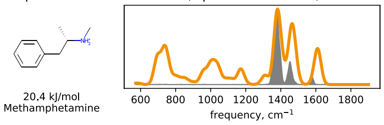


protonated HMDB0015517, spectral sim. = 697, #4

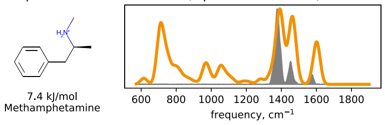


protonated HMDB0015517, spectral sim. = 656, #5

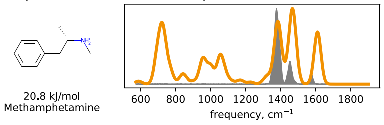


protonated HMDB0015517, spectral sim. = 632, #6

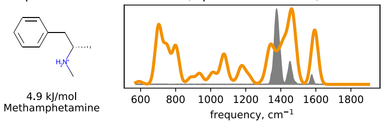


protonated HMDB0015517, spectral sim. = 578, #7

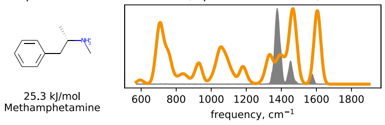

174 protonated HMDB0028850

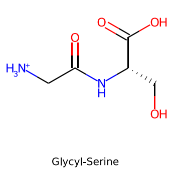


Spectra of protonated HMDB0028850, spectral sim. = 928, #1

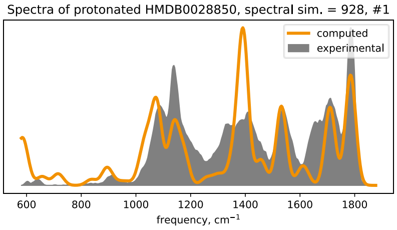


Structural similarity plot of protonated HMDB0028850

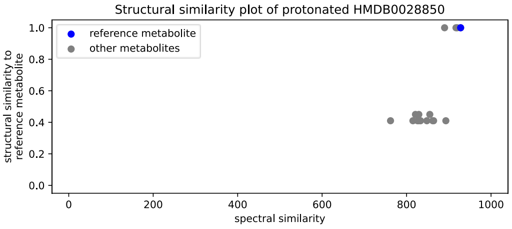


protonated HMDB0028850, spectral sim. = 928, #1

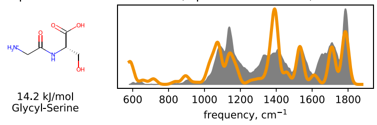


protonated HMDB0028850, spectral sim. = 920, #2

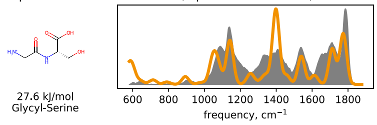


protonated HMDB0028850, spectral sim. = 917, #3

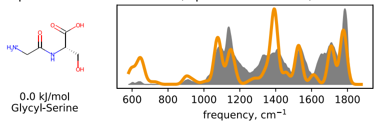


protonated HMDB0028850, spectral sim. = 893, #4

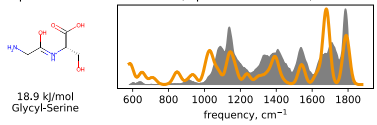


protonated HMDB0028850, spectral sim. = 890, #5

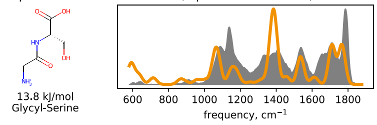


protonated HMDB0028850, spectral sim. = 864, #6

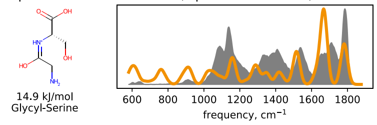


protonated HMDB0028850, spectral sim. = 862, #7

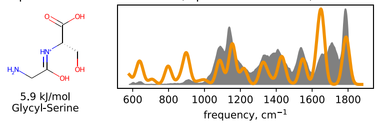


protonated HMDB0028850, spectral sim. = 855, #8

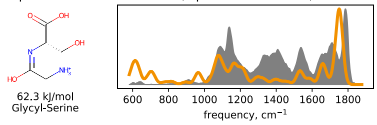


protonated HMDB0028850, spectral sim. = 848, #9

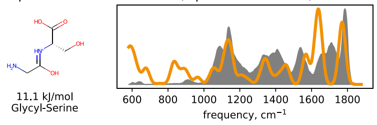


protonated HMDB0028850, spectral sim. = 833, #10

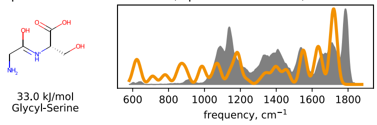


protonated HMDB0028850, spectral sim. = 829, #11

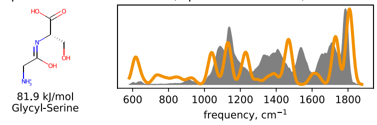


protonated HMDB0028850, spectral sim. = 828, #12

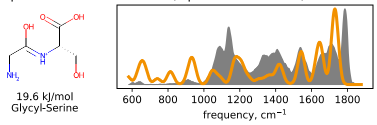


protonated HMDB0028850, spectral sim. = 826, #13

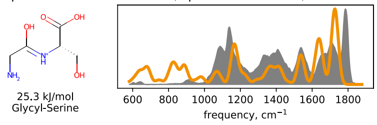


protonated HMDB0028850, spectral sim. = 821, #14

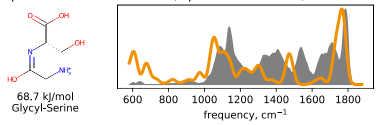


protonated HMDB0028850, spectral sim. = 815, #15

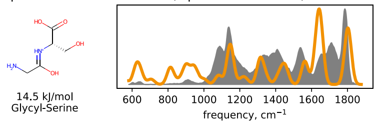


protonated HMDB0028850, spectral sim. = 762, #16

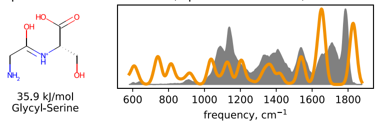


175 protonated HMDB0031861

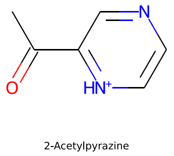


Spectra of protonated HMDB0031861, spectral sim. = 822, #1

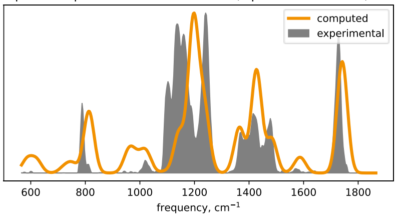


Structural similarity plot of protonated HMDB0031861

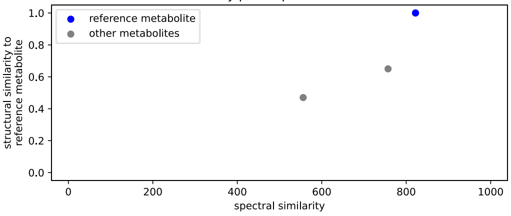


protonated HMDB0031861, spectral sim. = 822, #1

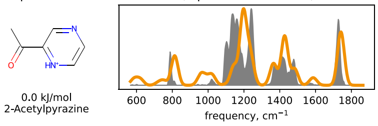


protonated HMDB0031861, spectral sim. = 757, #2

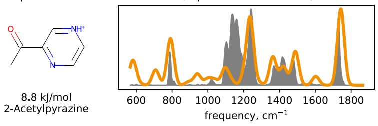


protonated HMDB0031861, spectral sim. = 556, #3

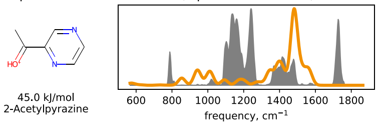

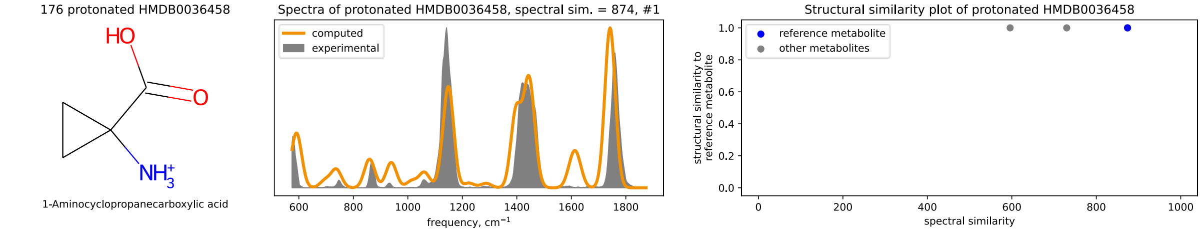
176 protonated HMDB0036458
1-Aminocyclopropanecarboxylic acid
Spectra of protonated HMDB0036458, spectral sim. = 874, #1
computed
experimental
frequency, cm⁻¹
Structural similarity plot of protonated HMDB0036458
reference metabolite
other metabolites
structural similarity to reference metabolite
spectral similarity

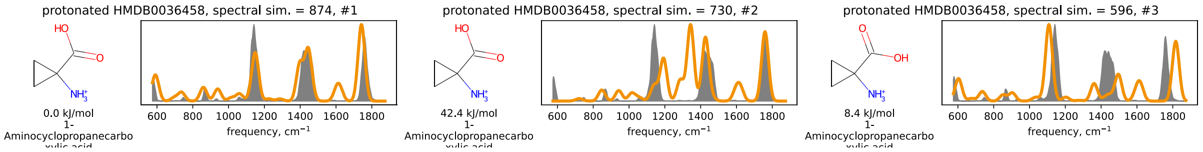
protonated HMDB0036458, spectral sim. = 874, #1
0.0 kJ/mol
1-Aminocyclopropanecarbo
protonated HMDB0036458, spectral sim. = 730, #2
42.4 kJ/mol
1-Aminocyclopropanecarbo
protonated HMDB0036458, spectral sim. = 596, #3
8.4 kJ/mol
1-Aminocyclopropanecarbo
frequency, cm⁻¹

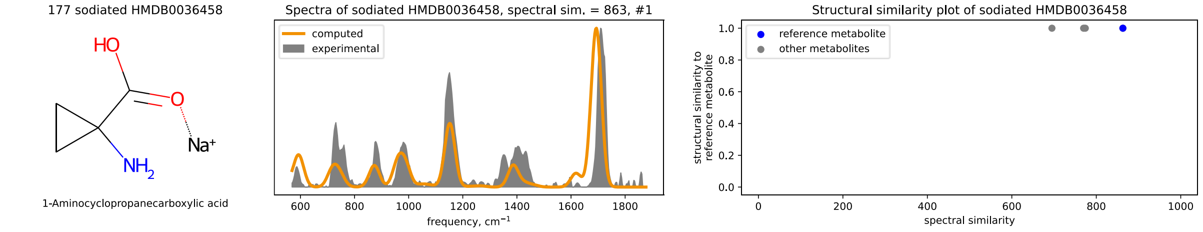
177 sodiated HMDB0036458
1-Aminocyclopropanecarboxylic acid
Spectra of sodiated HMDB0036458, spectral sim. = 863, #1
computed
experimental
frequency, cm⁻¹
Structural similarity plot of sodiated HMDB0036458
reference metabolite
other metabolites
structural similarity to reference metabolite
spectral similarity

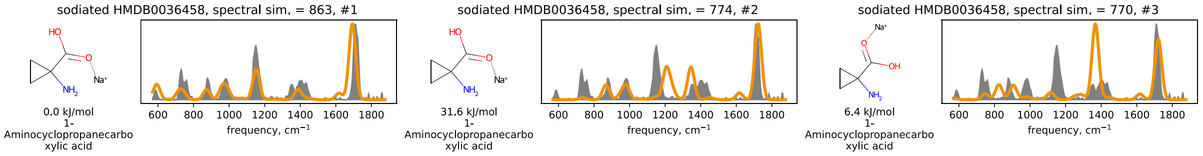
sodiated HMDB0036458, spectral sim. = 863, #1
0.0 kJ/mol
1-Aminocyclopropanecarbo
xylic acid
sodiated HMDB0036458, spectral sim. = 774, #2
31.6 kJ/mol
1-Aminocyclopropanecarbo
xylic acid
sodiated HMDB0036458, spectral sim. = 770, #3
6.4 kJ/mol
1-Aminocyclopropanecarbo
xylic acid
frequency, cm⁻¹

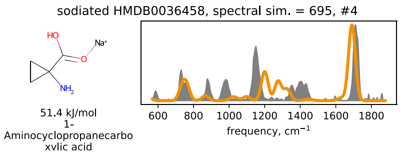
sodiated HMDB0036458, spectral sim. = 695, #4
51.4 kJ/mol
1-Aminocyclopropanecarbo
xylic acid
frequency, cm⁻¹

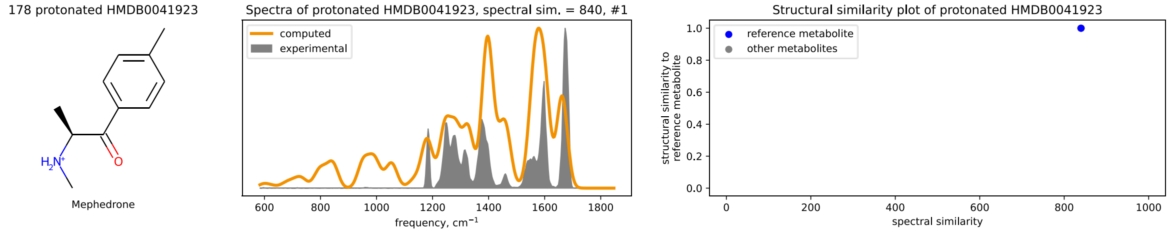
178 protonated HMDB0041923
Mephedrone
Spectra of protonated HMDB0041923, spectral sim. = 840, #1
computed
experimental
frequency, cm⁻¹
Structural similarity plot of protonated HMDB0041923
reference metabolite
other metabolites
structural similarity to reference metabolite
spectral similarity

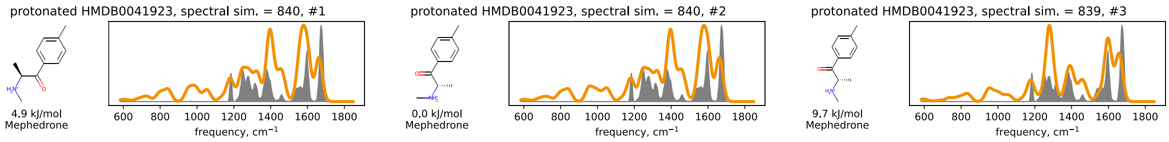
protonated HMDB0041923, spectral sim. = 840, #1
4.9 kJ/mol
Mephedrone
protonated HMDB0041923, spectral sim. = 840, #2
0.0 kJ/mol
Mephedrone
protonated HMDB0041923, spectral sim. = 839, #3
9.7 kJ/mol
Mephedrone
frequency, cm⁻¹

179 protonated HMDB0041931

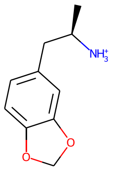


3,4-Methylenedioxyamphetamine

Spectra of protonated HMDB0041931, spectral sim. = 924, #1

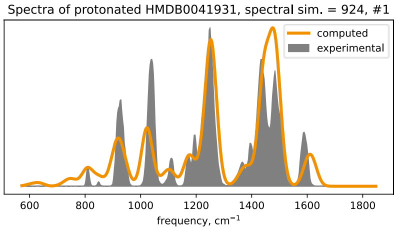


Structural similarity plot of protonated HMDB0041931

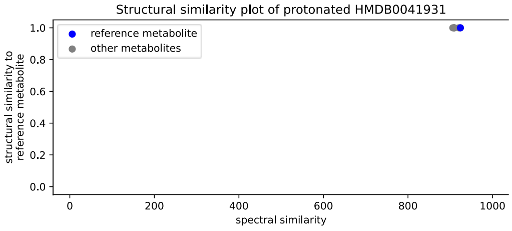


protonated HMDB0041931, spectral sim. = 924, #1

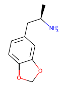

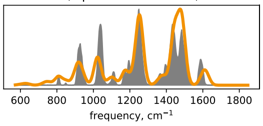


0.0 kJ/mol
3,4-Methylenedioxyamphetamine

protonated HMDB0041931, spectral sim. = 920, #2

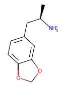

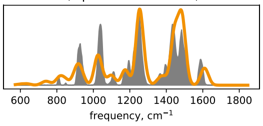


0.8 kJ/mol
3,4-Methylenedioxyamphetamine

protonated HMDB0041931, spectral sim. = 910, #3

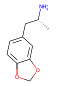

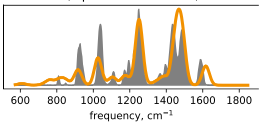


20.7 kJ/mol
3,4-Methylenedioxyamphetamine

protonated HMDB0041931, spectral sim. = 909, #4

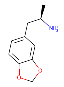

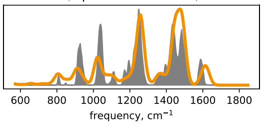


5.8 kJ/mol
3,4-Methylenedioxyamphetamine

protonated HMDB0041931, spectral sim. = 909, #5

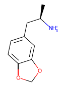

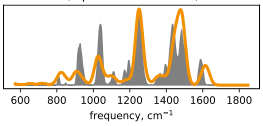


7.3 kJ/mol
3,4-Methylenedioxyamphetamine

protonated HMDB0041931, spectral sim. = 906, #6

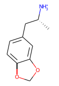

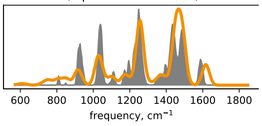


21.2 kJ/mol
3,4-Methylenedioxyamphetamine

180 deprotonated HMDB0059720

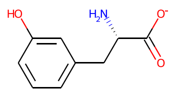


Meta-Tyrosine

Spectra of deprotonated HMDB0059720, spectral sim. = 930, #1

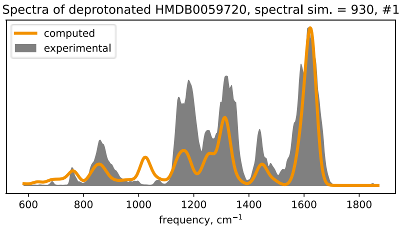


Structural similarity plot of deprotonated HMDB0059720

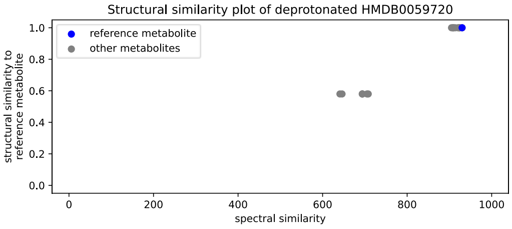


deprotonated HMDB0059720, spectral sim. = 930, #1

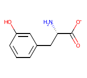

13.7 kJ/mol
Meta-Tyrosine

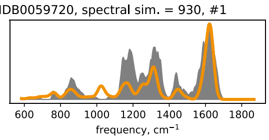

deprotonated HMDB0059720, spectral sim. = 924, #2

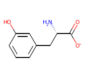

19.6 kJ/mol
Meta-Tyrosine

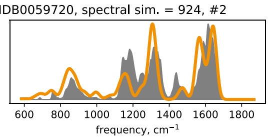

deprotonated HMDB0059720, spectral sim. = 924, #3

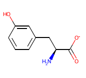

18.0 kJ/mol
Meta-Tyrosine

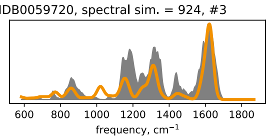

deprotonated HMDB0059720, spectral sim. = 923, #4

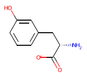

9.7 kJ/mol
Meta-Tyrosine

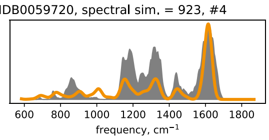

deprotonated HMDB0059720, spectral sim. = 920, #5

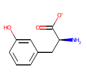

0.0 kJ/mol
Meta-Tyrosine

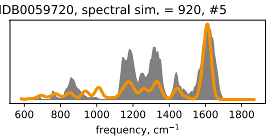

deprotonated HMDB0059720, spectral sim. = 914, #6

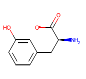

7.5 kJ/mol
Meta-Tyrosine

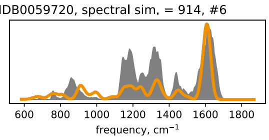

deprotonated HMDB0059720, spectral sim. = 910, #7

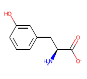

23.1 kJ/mol
Meta-Tyrosine

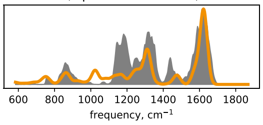

deprotonated HMDB0059720, spectral sim. = 909, #8

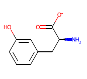

16.7 kJ/mol
Meta-Tyrosine

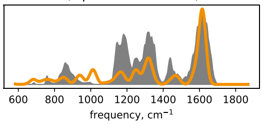

deprotonated HMDB0059720, spectral sim. = 908, #9

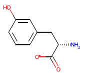

22.3 kJ/mol
Meta-Tyrosine

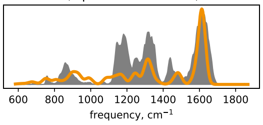

deprotonated HMDB0059720, spectral sim. = 905, #10

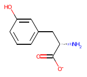

13.3 kJ/mol
Meta-Tyrosine

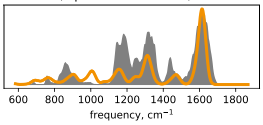

deprotonated HMDB0059720, spectral sim. = 708, #11

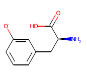

38.2 kJ/mol
Meta-Tyrosine

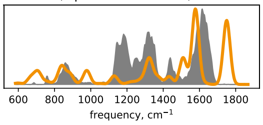

deprotonated HMDB0059720, spectral sim. = 705, #12

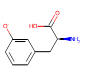

43.9 kJ/mol
Meta-Tyrosine

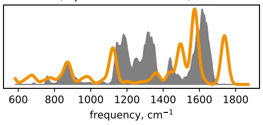

deprotonated HMDB0059720, spectral sim. = 694, #13

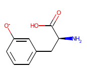

50.0 kJ/mol
Meta-Tyrosine

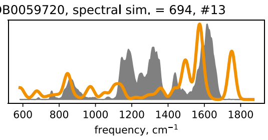

deprotonated HMDB0059720, spectral sim. = 694, #14

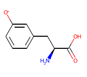

34.2 kJ/mol
Meta-Tyrosine

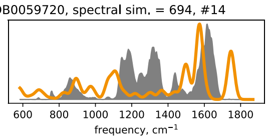

deprotonated HMDB0059720, spectral sim. = 694, #15

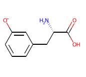

33.6 kJ/mol
Meta-Tyrosine

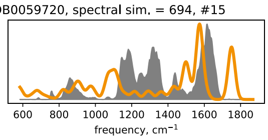

deprotonated HMDB0059720, spectral sim. = 646, #16

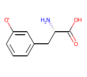

13.2 kJ/mol
Meta-Tyrosine

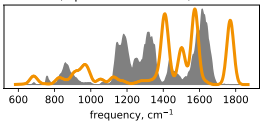

deprotonated HMDB0059720, spectral sim. = 641, #17

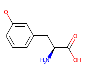

14.3 kJ/mol
Meta-Tyrosine

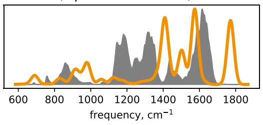

181 protonated HMDB0059720

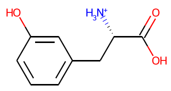


Meta-Tyrosine

Spectra of protonated HMDB0059720, spectral sim. = 918, #1

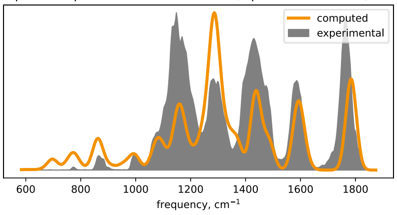


Structural similarity plot of protonated HMDB0059720

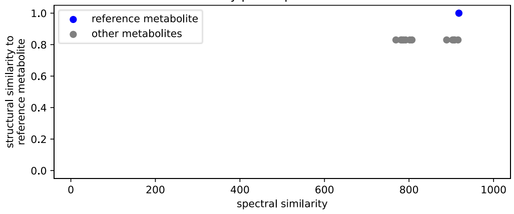


protonated HMDB0059720, spectral sim. = 918, #1

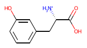

36.5 kJ/mol
Meta-Tyrosine

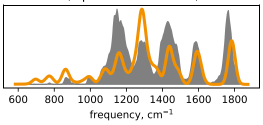


protonated HMDB0059720, spectral sim. = 916, #2

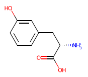

35.7 kJ/mol
Meta-Tyrosine

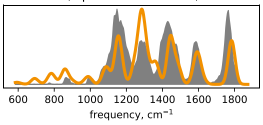


protonated HMDB0059720, spectral sim. = 908, #3

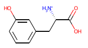

7.1 kJ/mol
Meta-Tyrosine

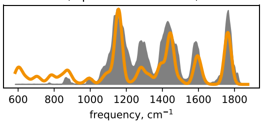


protonated HMDB0059720, spectral sim. = 906, #4

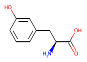

0.1 kJ/mol
Meta-Tyrosine

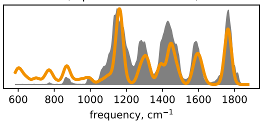


protonated HMDB0059720, spectral sim. = 903, #5

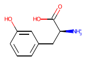

0.0 kJ/mol
Meta-Tyrosine

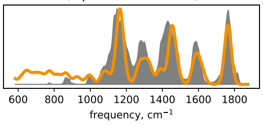


protonated HMDB0059720, spectral sim. = 889, #6

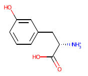

29.5 kJ/mol
Meta-Tyrosine

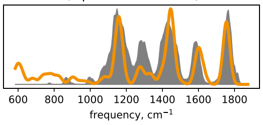


protonated HMDB0059720, spectral sim. = 807, #7

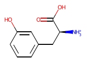

15.8 kJ/mol
Meta-Tyrosine

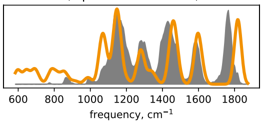


protonated HMDB0059720, spectral sim. = 802, #8

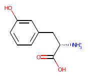

19.6 kJ/mol
Meta-Tyrosine

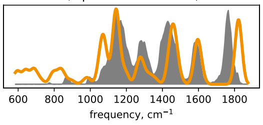


protonated HMDB0059720, spectral sim. = 792, #9

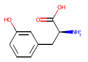

11.7 kJ/mol
Meta-Tyrosine

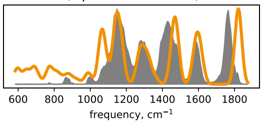


protonated HMDB0059720, spectral sim. = 787, #10

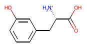

11.4 kJ/mol
Meta-Tyrosine

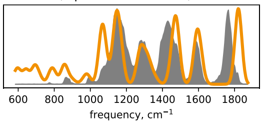


protonated HMDB0059720, spectral sim. = 783, #11

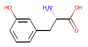

17.9 kJ/mol
Meta-Tyrosine

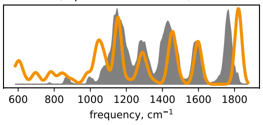


protonated HMDB0059720, spectral sim. = 781, #12

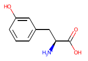

16.9 kJ/mol
Meta-Tyrosine

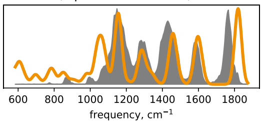


protonated HMDB0059720, spectral sim. = 769, #13

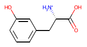

11.5 kJ/mol
Meta-Tyrosine

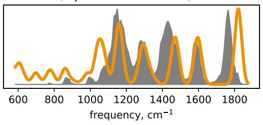

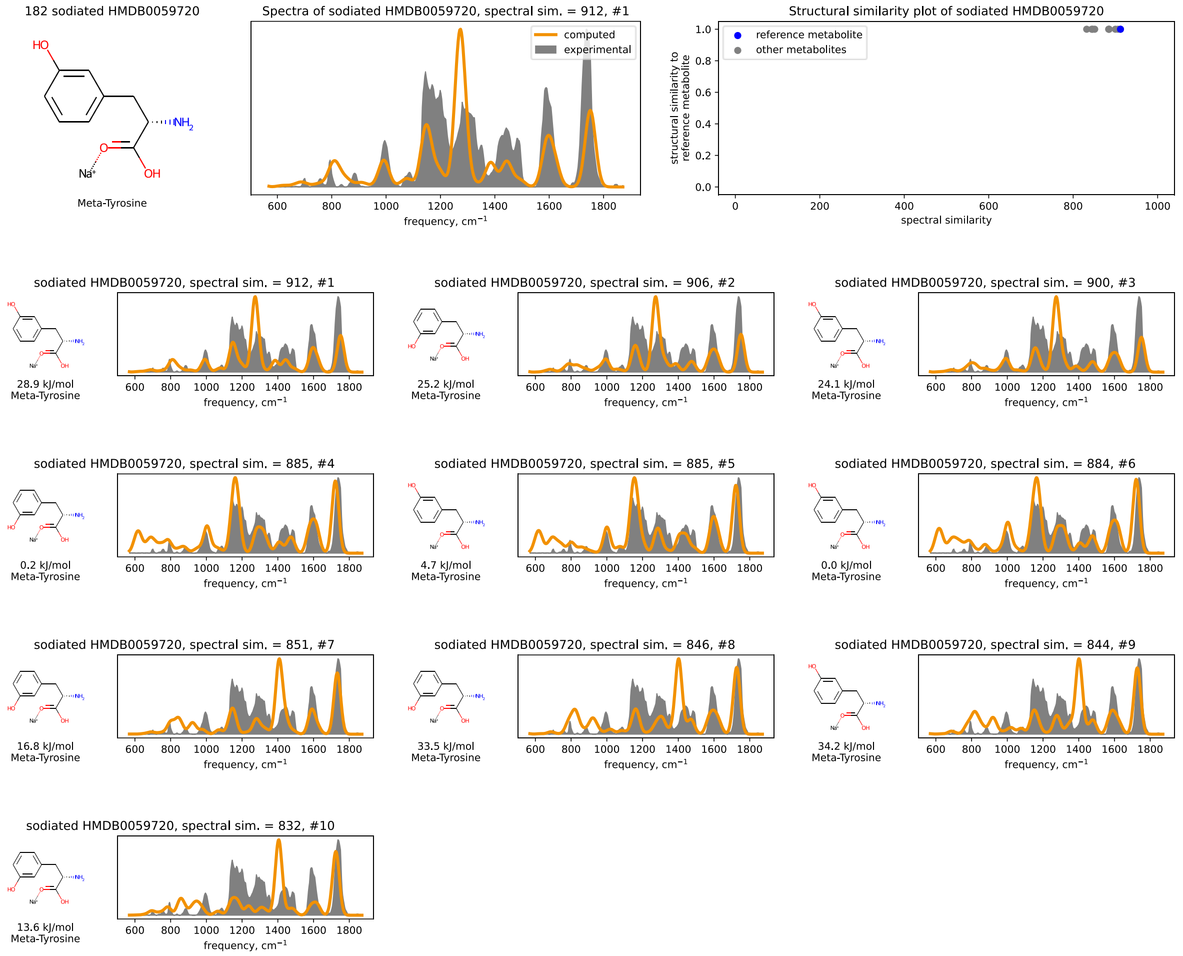

182 sodiated HMDB0059720
Meta-Tyrosine
Spectra of sodiated HMDB0059720, spectral sim. = 912, #1
computed
experimental
frequency, cm$^{-1}$
Structural similarity plot of sodiated HMDB0059720
reference metabolite
other metabolites
structural similarity to reference metabolite
spectral similarity
sodiated HMDB0059720, spectral sim. = 912, #1
28.9 kJ/mol
Meta-Tyrosine
sodiated HMDB0059720, spectral sim. = 906, #2
25.2 kJ/mol
Meta-Tyrosine
sodiated HMDB0059720, spectral sim. = 900, #3
24.1 kJ/mol
Meta-Tyrosine
sodiated HMDB0059720, spectral sim. = 885, #4
0.2 kJ/mol
Meta-Tyrosine
sodiated HMDB0059720, spectral sim. = 885, #5
4.7 kJ/mol
Meta-Tyrosine
sodiated HMDB0059720, spectral sim. = 884, #6
0.0 kJ/mol
Meta-Tyrosine
sodiated HMDB0059720, spectral sim. = 851, #7
16.8 kJ/mol
Meta-Tyrosine
sodiated HMDB0059720, spectral sim. = 846, #8
33.5 kJ/mol
Meta-Tyrosine
sodiated HMDB0059720, spectral sim. = 844, #9
34.2 kJ/mol
Meta-Tyrosine
sodiated HMDB0059720, spectral sim. = 832, #10
13.6 kJ/mol
Meta-Tyrosine


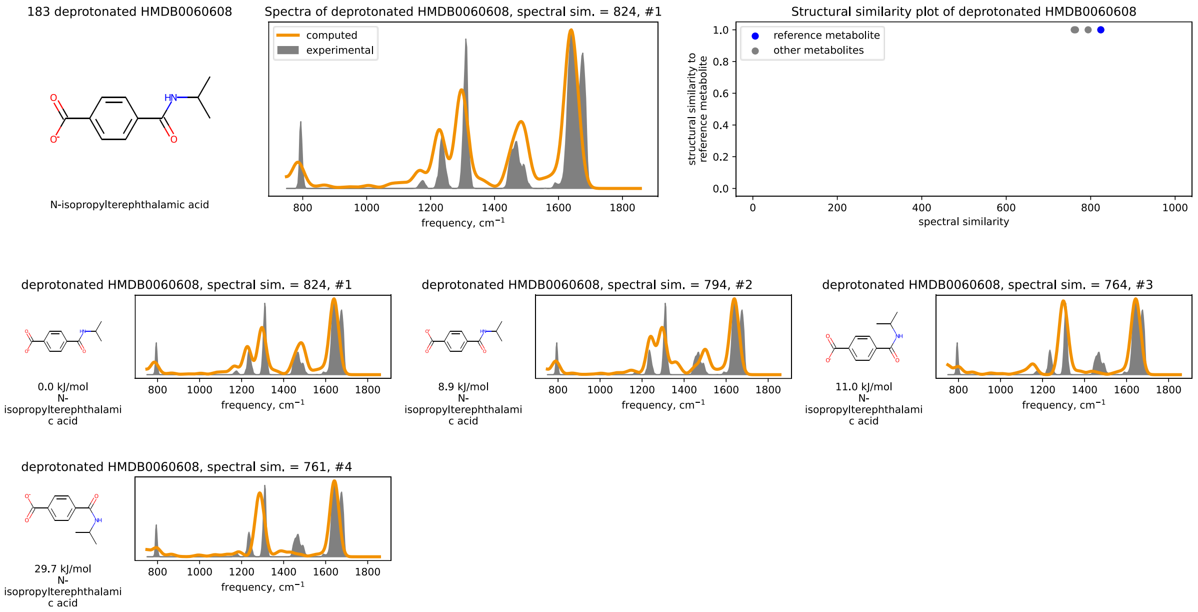

183 deprotonated HMDB0060608
N-isopropylterephthalamic acid
Spectra of deprotonated HMDB0060608, spectral sim. = 824, #1
computed
experimental
frequency, cm$^{-1}$
Structural similarity plot of deprotonated HMDB0060608
reference metabolite
other metabolites
structural similarity to reference metabolite
spectral similarity
deprotonated HMDB0060608, spectral sim. = 824, #1
0.0 kJ/mol
N-isopropylterephthalamic acid
deprotonated HMDB0060608, spectral sim. = 794, #2
8.9 kJ/mol
N-isopropylterephthalamic acid
deprotonated HMDB0060608, spectral sim. = 764, #3
11.0 kJ/mol
N-isopropylterephthalamic acid
deprotonated HMDB0060608, spectral sim. = 761, #4
29.7 kJ/mol
N-isopropylterephthalamic acid

184 protonated HMDB0060608

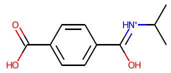


N-isopropylterephthalamic acid

Spectra of protonated HMDB0060608, spectral sim. = 897, #1

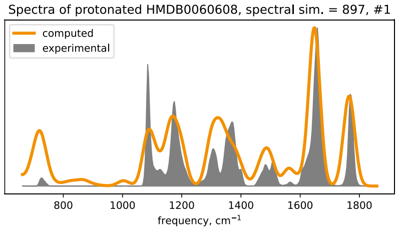


Structural similarity plot of protonated HMDB0060608

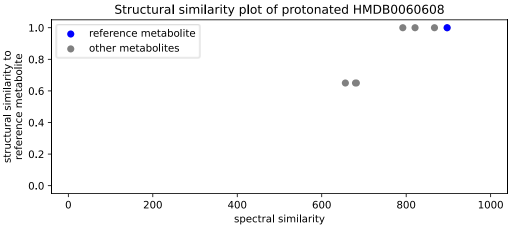


protonated HMDB0060608, spectral sim. = 897, #1

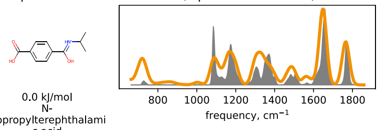


0.0 kJ/mol
N-
isopropylterephthalami
c acid

protonated HMDB0060608, spectral sim. = 867, #2

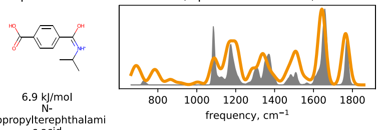


6.9 kJ/mol
N-
isopropylterephthalami
c acid

protonated HMDB0060608, spectral sim. = 821, #3

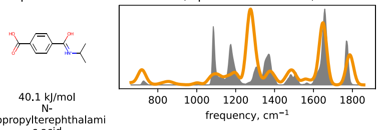


40.1 kJ/mol
N-
isopropylterephthalami
c acid

protonated HMDB0060608, spectral sim. = 792, #4

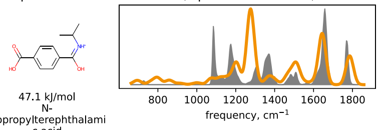


47.1 kJ/mol
N-
isopropylterephthalami
c acid

protonated HMDB0060608, spectral sim. = 682, #5

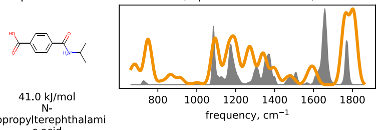


41.0 kJ/mol
N-
isopropylterephthalami
c acid

protonated HMDB0060608, spectral sim. = 680, #6

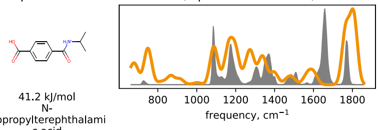


41.2 kJ/mol
N-
isopropylterephthalami
c acid

protonated HMDB0060608, spectral sim. = 656, #7

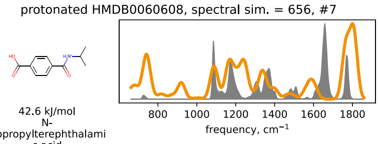


42.6 kJ/mol
N-
isopropylterephthalami
c acid

185 sodiated HMDB0060608

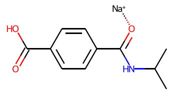


N-isopropylterephthalamic acid

Spectra of sodiated HMDB0060608, spectral sim. = 934, #1

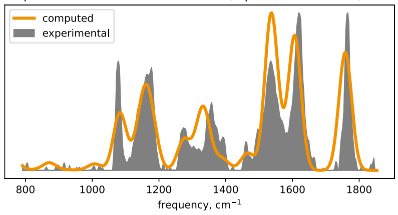


Structural similarity plot of sodiated HMDB0060608

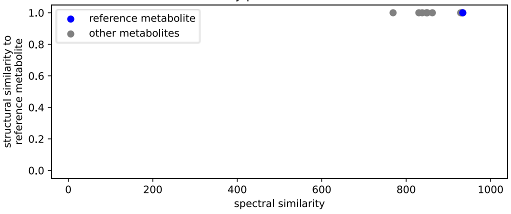


sodiated HMDB0060608, spectral sim. = 934, #1

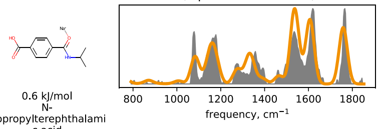


0.6 kJ/mol
N-
isopropylterephthalami
c acid

sodiated HMDB0060608, spectral sim. = 932, #2

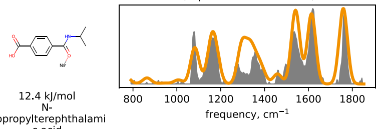


12.4 kJ/mol
N-
isopropylterephthalami
c acid

sodiated HMDB0060608, spectral sim. = 931, #3

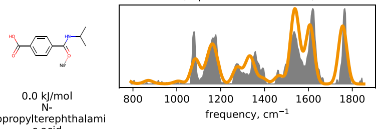


0.0 kJ/mol
N-
isopropylterephthalami
c acid

sodiated HMDB0060608, spectral sim. = 929, #4

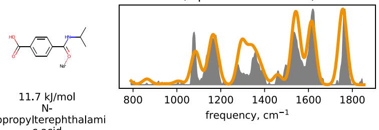


11.7 kJ/mol
N-
isopropylterephthalami
c acid

sodiated HMDB0060608, spectral sim. = 862, #5

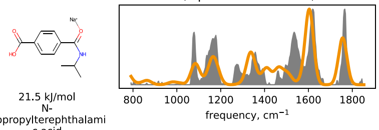


21.5 kJ/mol
N-
isopropylterephthalami
c acid

sodiated HMDB0060608, spectral sim. = 850, #6

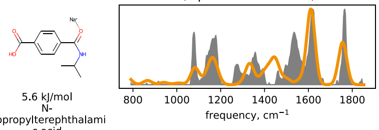


5.6 kJ/mol
N-
isopropylterephthalami
c acid

sodiated HMDB0060608, spectral sim. = 848, #7

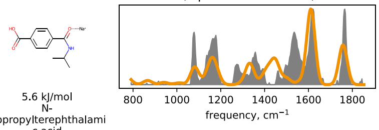


5.6 kJ/mol
N-
isopropylterephthalami
c acid

sodiated HMDB0060608, spectral sim. = 838, #8

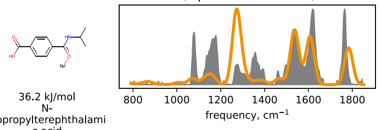


36.2 kJ/mol
N-
isopropylterephthalami
c acid

sodiated HMDB0060608, spectral sim. = 830, #9

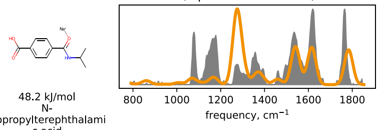


48.2 kJ/mol
N-
isopropylterephthalami
c acid

sodiated HMDB0060608, spectral sim. = 769, #10

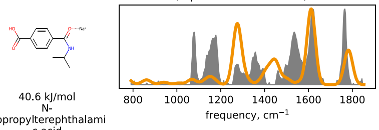


40.6 kJ/mol
N-
isopropylterephthalami
c acid

186 deprotonated HMDB0061705

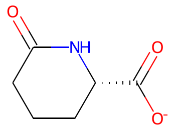


6-Oxopiperidine-2-carboxylic acid

Spectra of deprotonated HMDB0061705, spectral sim. = 902, #1

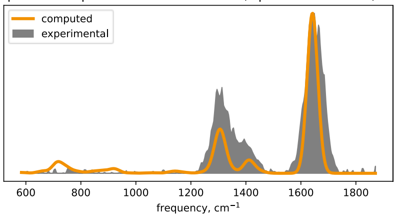


Structural similarity plot of deprotonated HMDB0061705

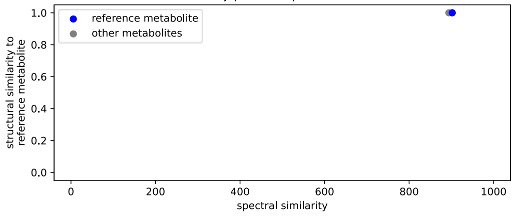


deprotonated HMDB0061705, spectral sim. = 902, #1

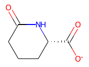

0.0 kJ/mol
6-Oxopiperidine-2-carboxylic acid

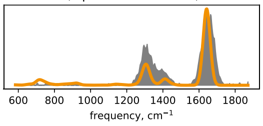


deprotonated HMDB0061705, spectral sim. = 894, #2

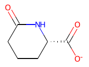

8.6 kJ/mol
6-Oxopiperidine-2-carboxylic acid

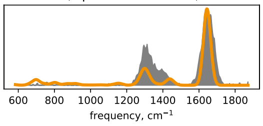


187 protonated HMDB0061705

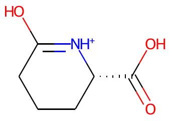


6-Oxopiperidine-2-carboxylic acid

Spectra of protonated HMDB0061705, spectral sim. = 824, #1

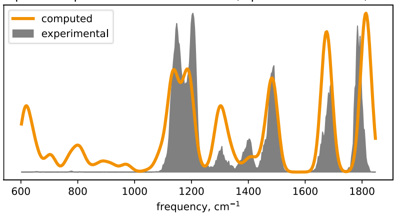


Structural similarity plot of protonated HMDB0061705

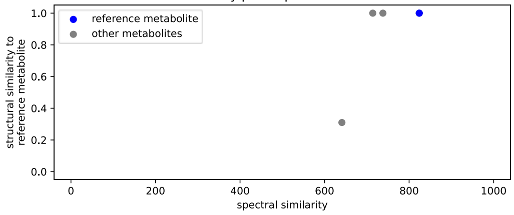


protonated HMDB0061705, spectral sim. = 824, #1

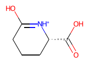

0.2 kJ/mol
6-Oxopiperidine-2-carboxylic acid

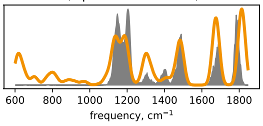


protonated HMDB0061705, spectral sim. = 738, #2

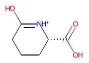

37.2 kJ/mol
6-Oxopiperidine-2-carboxylic acid

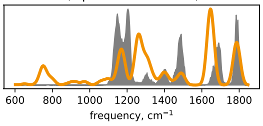


protonated HMDB0061705, spectral sim. = 714, #3

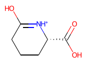

0.0 kJ/mol
6-Oxopiperidine-2-carboxylic acid

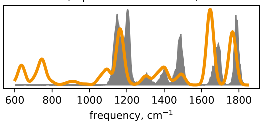


protonated HMDB0061705, spectral sim. = 641, #4

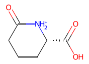

36.6 kJ/mol
6-Oxopiperidine-2-carboxylic acid

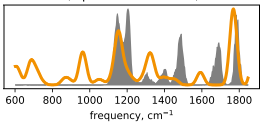

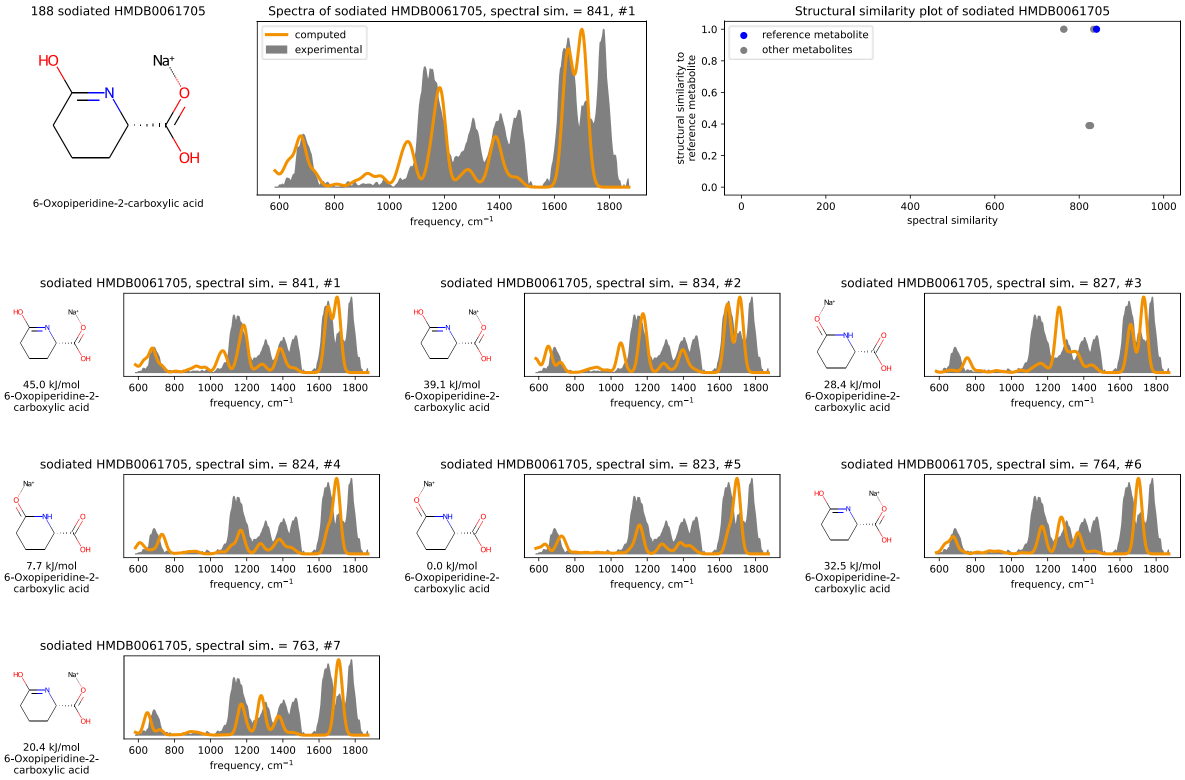

188 sodiated HMDB0061705
6-Oxopiperidine-2-carboxylic acid
Spectra of sodiated HMDB0061705, spectral sim. = 841, #1
computed
experimental
frequency, cm⁻¹
Structural similarity plot of sodiated HMDB0061705
reference metabolite
other metabolites
structural similarity to reference metabolite
spectral similarity
sodiated HMDB0061705, spectral sim. = 841, #1
45.0 kJ/mol
6-Oxopiperidine-2-carboxylic acid
sodiated HMDB0061705, spectral sim. = 834, #2
39.1 kJ/mol
6-Oxopiperidine-2-carboxylic acid
sodiated HMDB0061705, spectral sim. = 827, #3
28.4 kJ/mol
6-Oxopiperidine-2-carboxylic acid
sodiated HMDB0061705, spectral sim. = 824, #4
7.7 kJ/mol
6-Oxopiperidine-2-carboxylic acid
sodiated HMDB0061705, spectral sim. = 823, #5
0.0 kJ/mol
6-Oxopiperidine-2-carboxylic acid
sodiated HMDB0061705, spectral sim. = 764, #6
32.5 kJ/mol
6-Oxopiperidine-2-carboxylic acid
sodiated HMDB0061705, spectral sim. = 763, #7
20.4 kJ/mol
6-Oxopiperidine-2-carboxylic acid


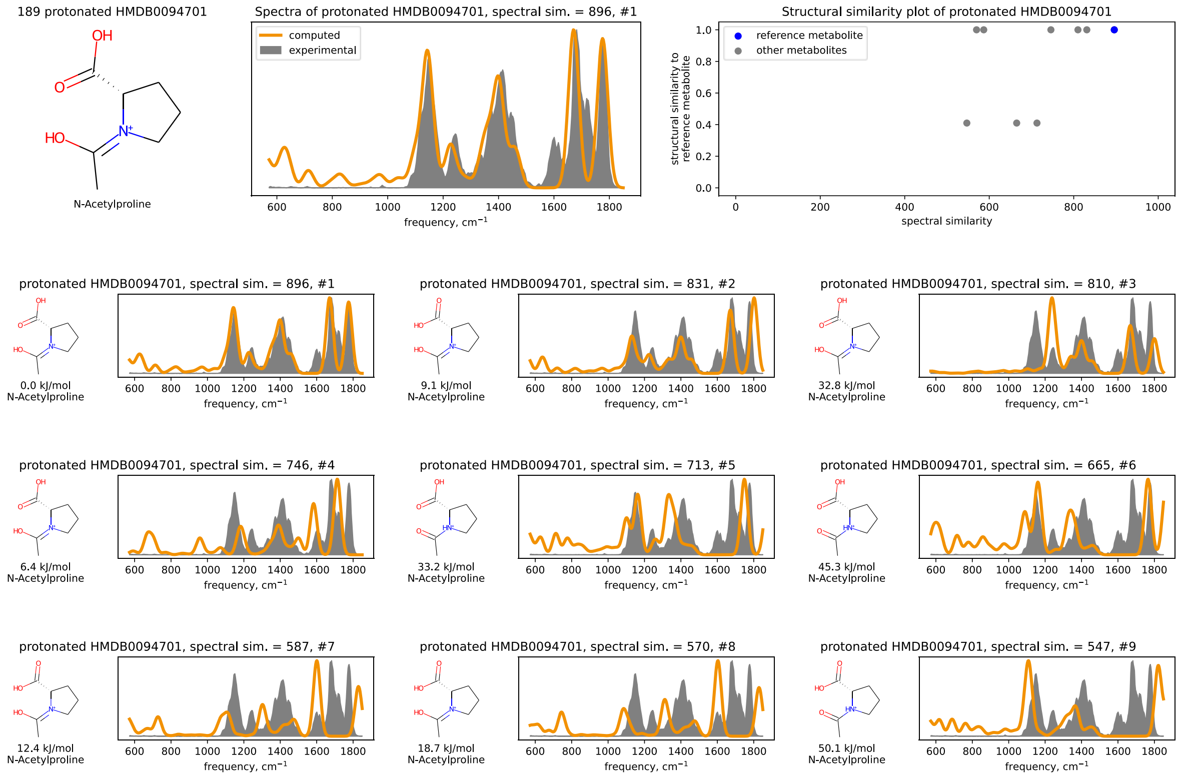

189 protonated HMDB0094701
N-Acetylproline
Spectra of protonated HMDB0094701, spectral sim. = 896, #1
computed
experimental
frequency, cm⁻¹
Structural similarity plot of protonated HMDB0094701
reference metabolite
other metabolites
structural similarity to reference metabolite
spectral similarity
protonated HMDB0094701, spectral sim. = 896, #1
0.0 kJ/mol
N-Acetylproline
protonated HMDB0094701, spectral sim. = 831, #2
9.1 kJ/mol
N-Acetylproline
protonated HMDB0094701, spectral sim. = 810, #3
32.8 kJ/mol
N-Acetylproline
protonated HMDB0094701, spectral sim. = 746, #4
6.4 kJ/mol
N-Acetylproline
protonated HMDB0094701, spectral sim. = 713, #5
33.2 kJ/mol
N-Acetylproline
protonated HMDB0094701, spectral sim. = 665, #6
45.3 kJ/mol
N-Acetylproline
protonated HMDB0094701, spectral sim. = 587, #7
12.4 kJ/mol
N-Acetylproline
protonated HMDB0094701, spectral sim. = 570, #8
18.7 kJ/mol
N-Acetylproline
protonated HMDB0094701, spectral sim. = 547, #9
50.1 kJ/mol
N-Acetylproline
