# Supplementary material for: An In Silico Infrared Spectral Library of Molecular Ions for Metabolite Identification
Source: Anal Chem. 2023 Jun 1;95(23):8998–9005. doi: 10.1021/acs.analchem.3c01078 (PMC10267894; doi:10.1021/acs.analchem.3c01078)

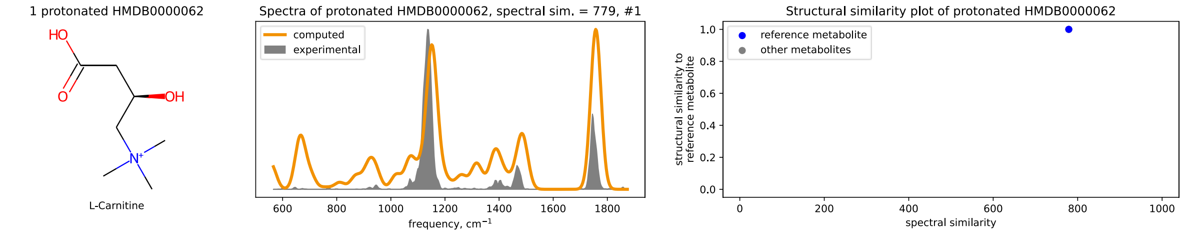
1 protonated HMDB0000062
L-Carnitine
Spectra of protonated HMDB0000062, spectral sim. = 779, #1
computed
experimental
frequency, cm−1
Structural similarity plot of protonated HMDB0000062
reference metabolite
other metabolites
structural similarity to reference metabolite
spectral similarity

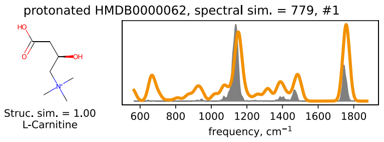
protonated HMDB0000062, spectral sim. = 779, #1
Struc. sim. = 1.00
L-Carnitine
frequency, cm−1

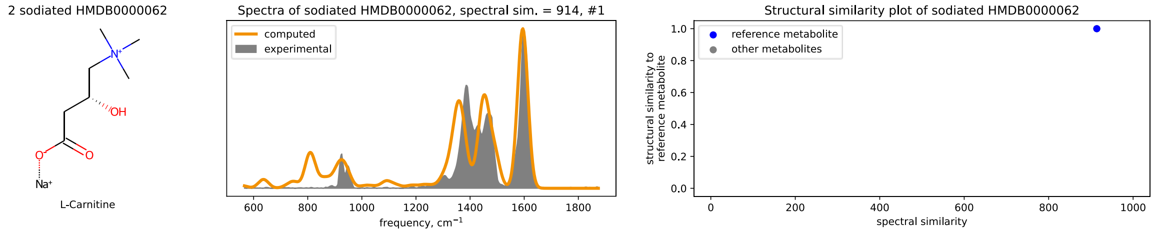
2 sodiated HMDB0000062
L-Carnitine
Spectra of sodiated HMDB0000062, spectral sim. = 914, #1
computed
experimental
frequency, cm−1
Structural similarity plot of sodiated HMDB0000062
reference metabolite
other metabolites
structural similarity to reference metabolite
spectral similarity

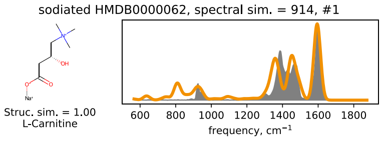
sodiated HMDB0000062, spectral sim. = 914, #1
Struc. sim. = 1.00
L-Carnitine
frequency, cm−1

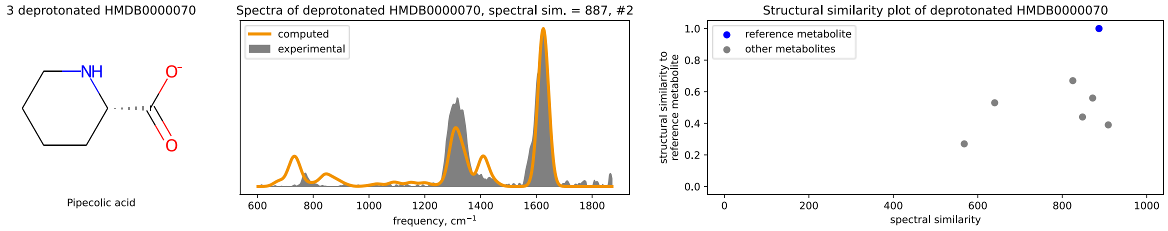
3 deprotonated HMDB0000070
Pipecolic acid
Spectra of deprotonated HMDB0000070, spectral sim. = 887, #2
computed
experimental
frequency, cm−1
Structural similarity plot of deprotonated HMDB0000070
reference metabolite
other metabolites
structural similarity to reference metabolite
spectral similarity

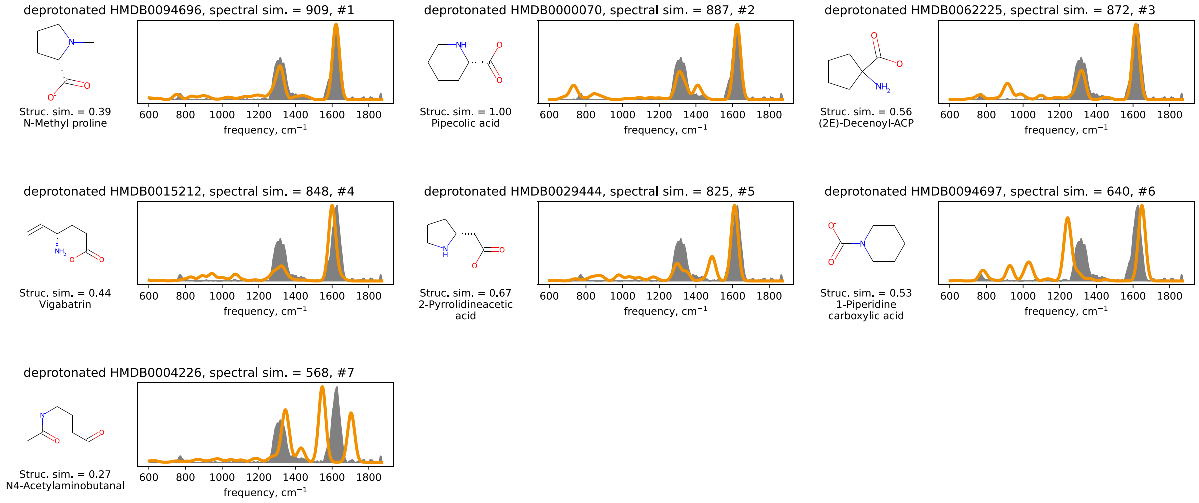
deprotonated HMDB0094696, spectral sim. = 909, #1
Struc. sim. = 0.39
N-Methyl proline
deprotonated HMDB0000070, spectral sim. = 887, #2
Struc. sim. = 1.00
Pipecolic acid
deprotonated HMDB0062225, spectral sim. = 872, #3
Struc. sim. = 0.56
(2E)-Decenoyl-ACP
deprotonated HMDB0015212, spectral sim. = 848, #4
Struc. sim. = 0.44
Vigabatrin
deprotonated HMDB0029444, spectral sim. = 825, #5
Struc. sim. = 0.67
2-Pyrrolidineacetic acid
deprotonated HMDB0094697, spectral sim. = 640, #6
Struc. sim. = 0.53
1-Piperidine carboxylic acid
deprotonated HMDB0004226, spectral sim. = 568, #7
Struc. sim. = 0.27
N4-Acetylaminobutanal
frequency, cm−1

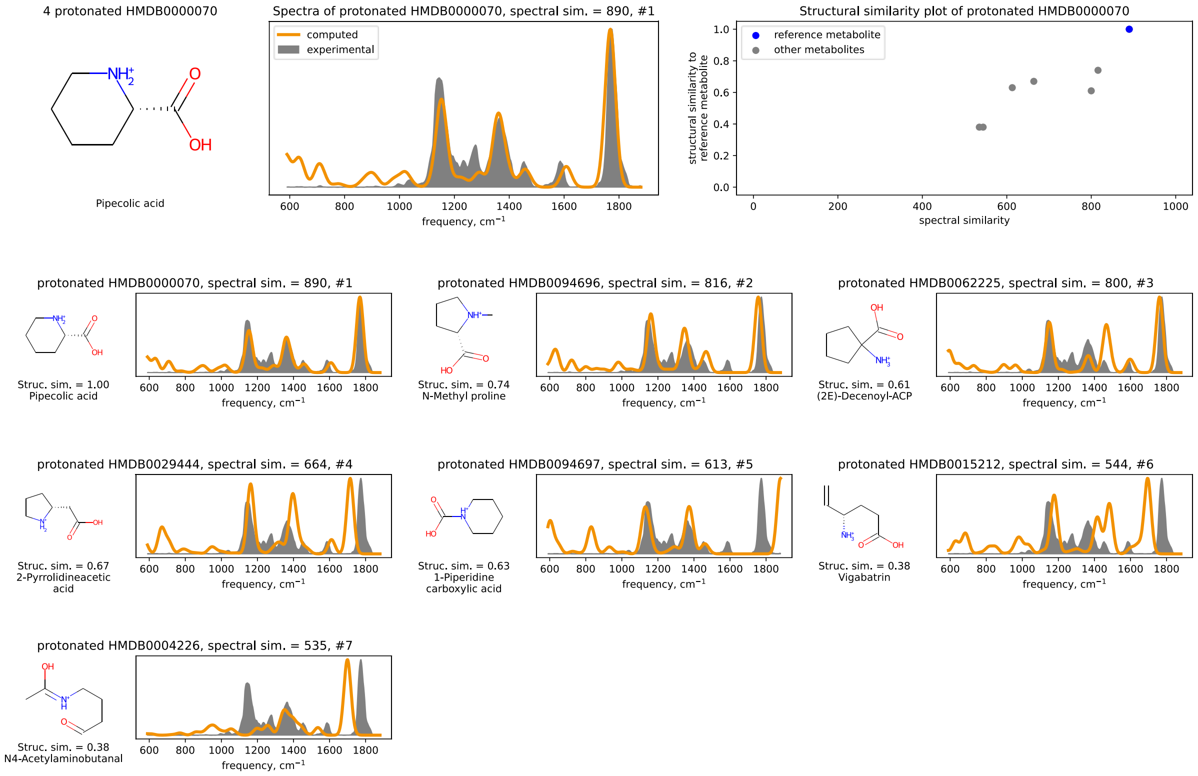

4 protonated HMDB0000070
Pipecolic acid
Spectra of protonated HMDB0000070, spectral sim. = 890, #1
computed
experimental
frequency, cm$^{-1}$
Structural similarity plot of protonated HMDB0000070
reference metabolite
other metabolites
structural similarity to reference metabolite
spectral similarity
protonated HMDB0000070, spectral sim. = 890, #1
Struc. sim. = 1.00
Pipecolic acid
protonated HMDB0094696, spectral sim. = 816, #2
Struc. sim. = 0.74
N-Methyl proline
protonated HMDB0062225, spectral sim. = 800, #3
Struc. sim. = 0.61
(2E)-Decenoyl-ACP
protonated HMDB0029444, spectral sim. = 664, #4
Struc. sim. = 0.67
2-Pyrrolidineacetic acid
protonated HMDB0094697, spectral sim. = 613, #5
Struc. sim. = 0.63
1-Piperidine carboxylic acid
protonated HMDB0015212, spectral sim. = 544, #6
Struc. sim. = 0.38
Vigabatrin
protonated HMDB0004226, spectral sim. = 535, #7
Struc. sim. = 0.38
N4-Acetylaminobutanal


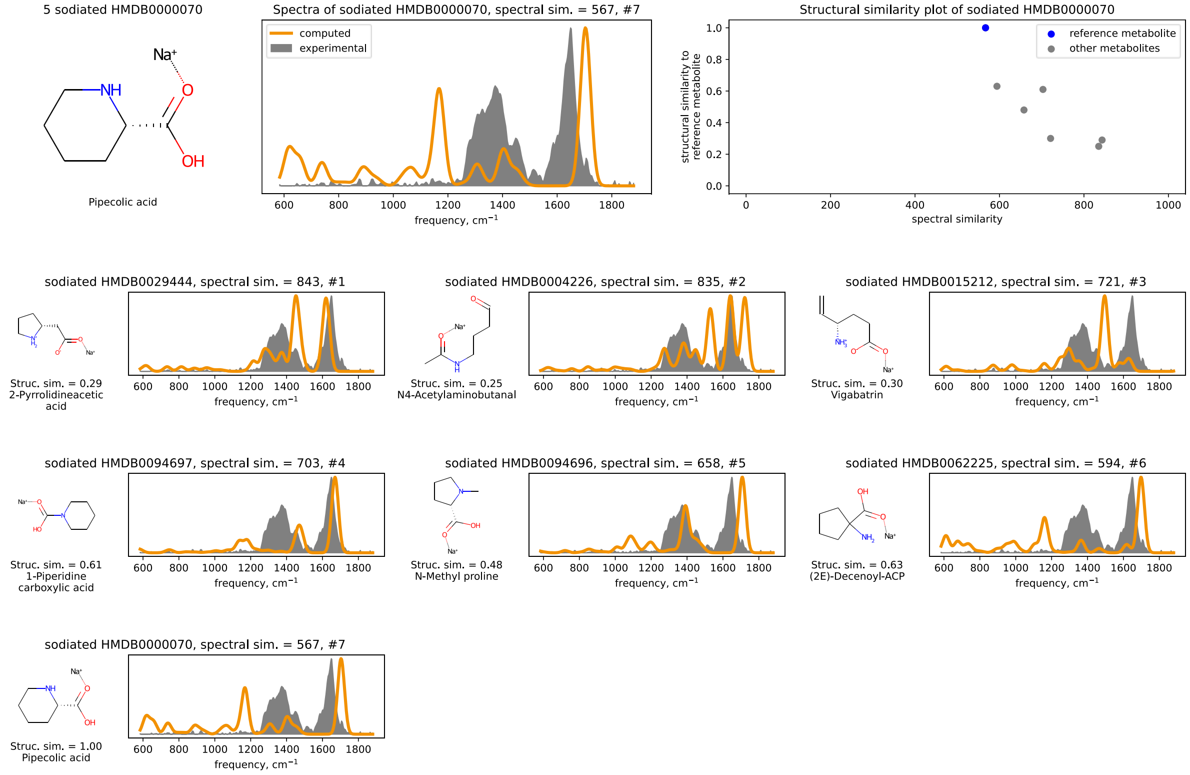

5 sodiated HMDB0000070
Pipecolic acid
Spectra of sodiated HMDB0000070, spectral sim. = 567, #7
computed
experimental
frequency, cm$^{-1}$
Structural similarity plot of sodiated HMDB0000070
reference metabolite
other metabolites
structural similarity to reference metabolite
spectral similarity
sodiated HMDB0029444, spectral sim. = 843, #1
Struc. sim. = 0.29
2-Pyrrolidineacetic acid
sodiated HMDB0004226, spectral sim. = 835, #2
Struc. sim. = 0.25
N4-Acetylaminobutanal
sodiated HMDB0015212, spectral sim. = 721, #3
Struc. sim. = 0.30
Vigabatrin
sodiated HMDB0094697, spectral sim. = 703, #4
Struc. sim. = 0.61
1-Piperidine carboxylic acid
sodiated HMDB0094696, spectral sim. = 658, #5
Struc. sim. = 0.48
N-Methyl proline
sodiated HMDB0062225, spectral sim. = 594, #6
Struc. sim. = 0.63
(2E)-Decenoyl-ACP
sodiated HMDB0000070, spectral sim. = 567, #7
Struc. sim. = 1.00
Pipecolic acid

6 deprotonated HMDB0000073

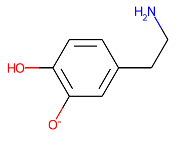

Dopamine

Spectra of deprotonated HMDB0000073, spectral sim. = 682, #1

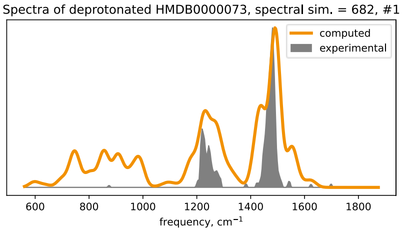


Structural similarity plot of deprotonated HMDB0000073

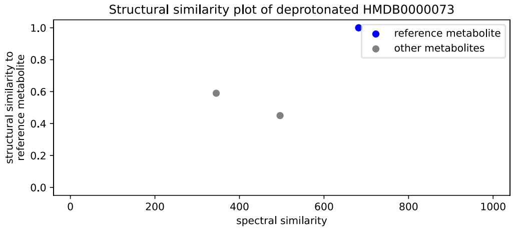


deprotonated HMDB0000073, spectral sim. = 682, #1

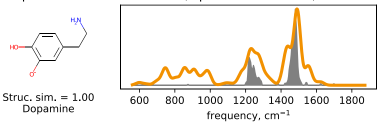


deprotonated HMDB0012309, spectral sim. = 496, #2

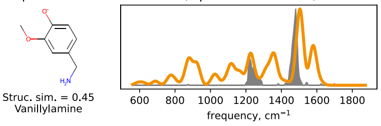


deprotonated HMDB0004825, spectral sim. = 345, #3

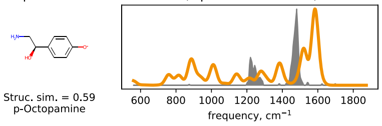


7 protonated HMDB0000073

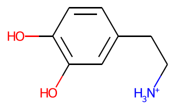

Dopamine

Spectra of protonated HMDB0000073, spectral sim. = 894, #1

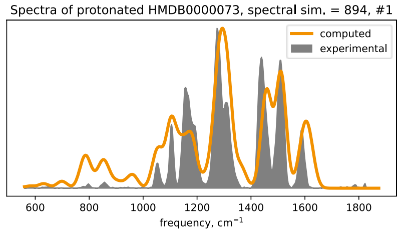


Structural similarity plot of protonated HMDB0000073

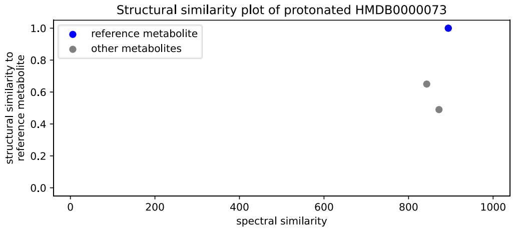


protonated HMDB0000073, spectral sim. = 894, #1

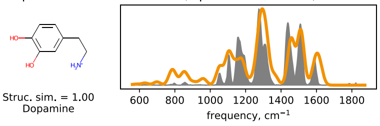


protonated HMDB0012309, spectral sim. = 872, #2

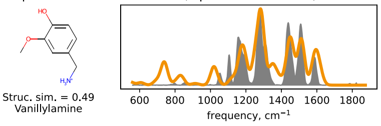


protonated HMDB0004825, spectral sim. = 843, #3

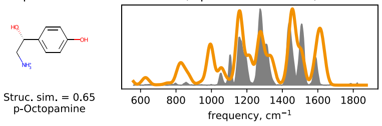


8 deprotonated HMDB0000107

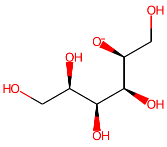

Galactitol

Spectra of deprotonated HMDB0000107, spectral sim. = 913, #1

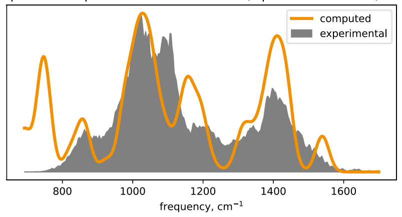


Structural similarity plot of deprotonated HMDB0000107

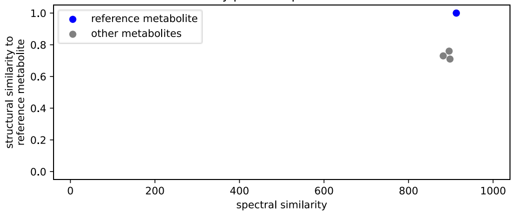


deprotonated HMDB0000107, spectral sim. = 913, #1

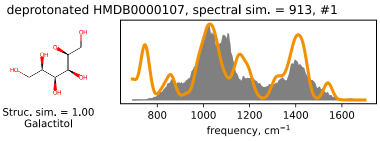


deprotonated HMDB0000765, spectral sim. = 898, #2

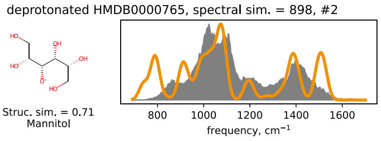


deprotonated HMDB0000247, spectral sim. = 896, #3

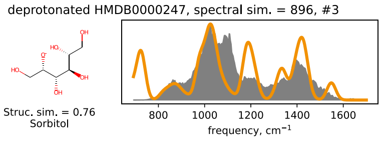


deprotonated HMDB0011632, spectral sim. = 882, #4

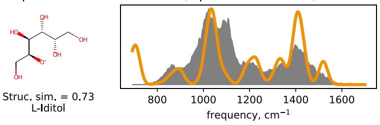

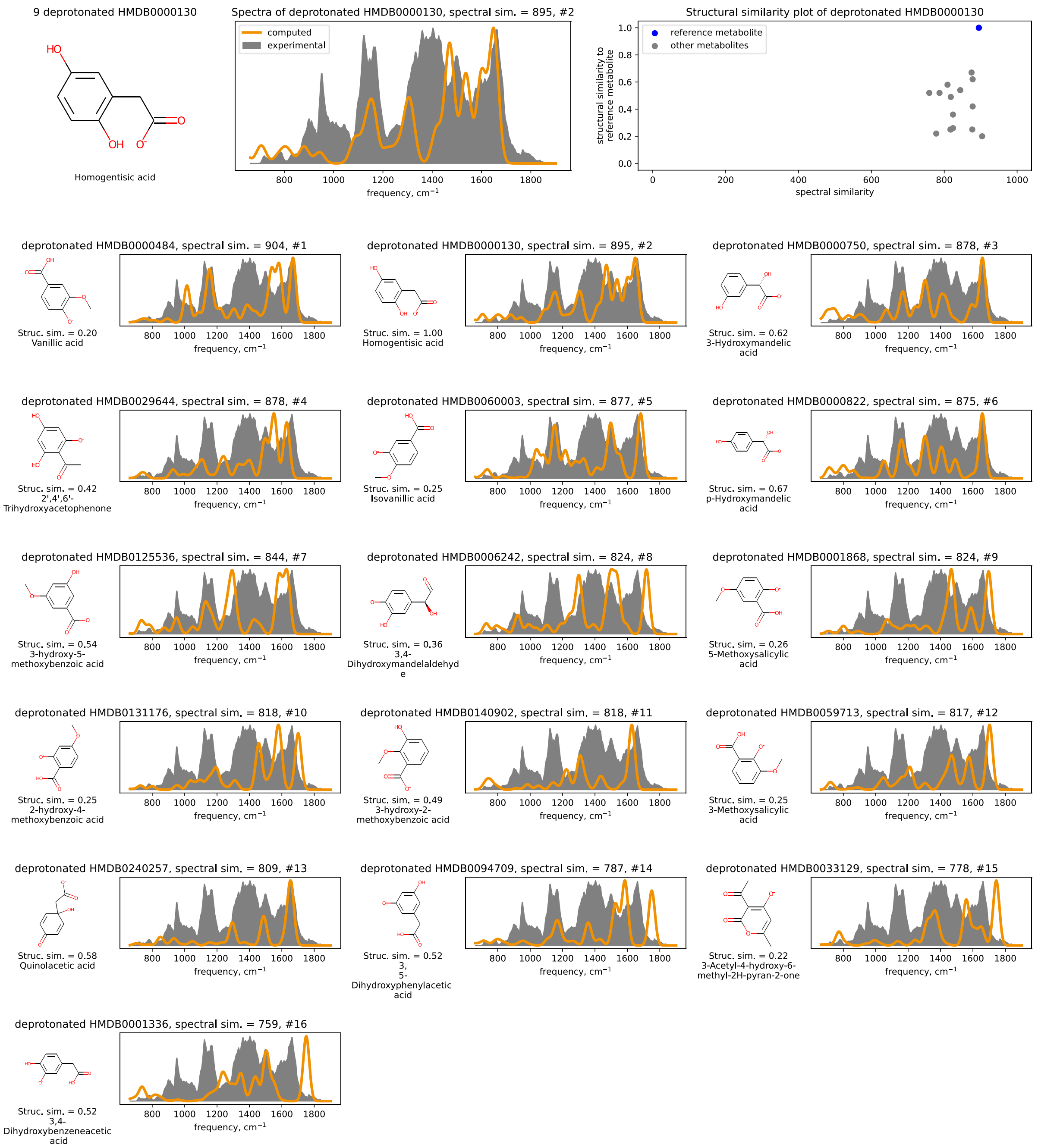

9 deprotonated HMDB0000130
Homogentisic acid
Spectra of deprotonated HMDB0000130, spectral sim. = 895, #2
computed
experimental
frequency, cm⁻¹
Structural similarity plot of deprotonated HMDB0000130
reference metabolite
other metabolites
structural similarity to reference metabolite
spectral similarity
deprotonated HMDB0000484, spectral sim. = 904, #1
Struc. sim. = 0.20
Vanillic acid
deprotonated HMDB0000130, spectral sim. = 895, #2
Struc. sim. = 1.00
Homogentisic acid
deprotonated HMDB0000750, spectral sim. = 878, #3
Struc. sim. = 0.62
3-Hydroxymandelic acid
deprotonated HMDB0029644, spectral sim. = 878, #4
Struc. sim. = 0.42
2',4',6'-Trihydroxyacetophenone
deprotonated HMDB0060003, spectral sim. = 877, #5
Struc. sim. = 0.25
Isovanillic acid
deprotonated HMDB0000822, spectral sim. = 875, #6
Struc. sim. = 0.67
p-Hydroxymandelic acid
deprotonated HMDB0125536, spectral sim. = 844, #7
Struc. sim. = 0.54
3-hydroxy-5-methoxybenzoic acid
deprotonated HMDB0006242, spectral sim. = 824, #8
Struc. sim. = 0.36
3,4-Dihydroxymandelaldehyde
deprotonated HMDB0001868, spectral sim. = 824, #9
Struc. sim. = 0.26
5-Methoxysalicylic acid
deprotonated HMDB0131176, spectral sim. = 818, #10
Struc. sim. = 0.25
2-hydroxy-4-methoxybenzoic acid
deprotonated HMDB0140902, spectral sim. = 818, #11
Struc. sim. = 0.49
3-hydroxy-2-methoxybenzoic acid
deprotonated HMDB0059713, spectral sim. = 817, #12
Struc. sim. = 0.25
3-Methoxysalicylic acid
deprotonated HMDB0240257, spectral sim. = 809, #13
Struc. sim. = 0.58
Quinolacetic acid
deprotonated HMDB0094709, spectral sim. = 787, #14
Struc. sim. = 0.52
3,5-Dihydroxyphenylacetic acid
deprotonated HMDB0033129, spectral sim. = 778, #15
Struc. sim. = 0.22
3-Acetyl-4-hydroxy-6-methyl-2H-pyran-2-one
deprotonated HMDB0001336, spectral sim. = 759, #16
Struc. sim. = 0.52
3,4-Dihydroxybenzeneacetic acid

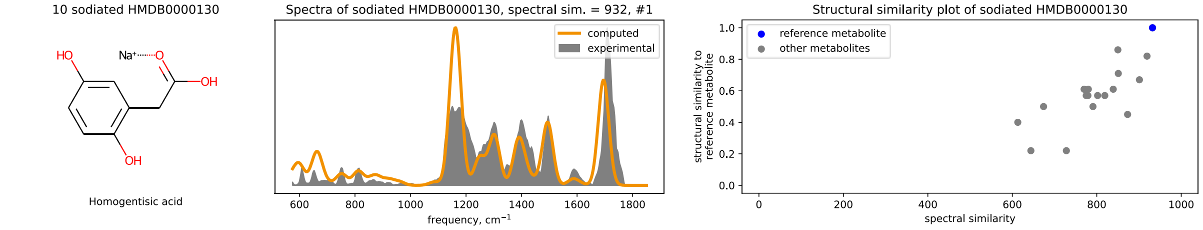
10 sodiated HMDB0000130
Homogentisic acid
Spectra of sodiated HMDB0000130, spectral sim. = 932, #1
computed
experimental
frequency, cm⁻¹
Structural similarity plot of sodiated HMDB0000130
reference metabolite
other metabolites
structural similarity to reference metabolite
spectral similarity

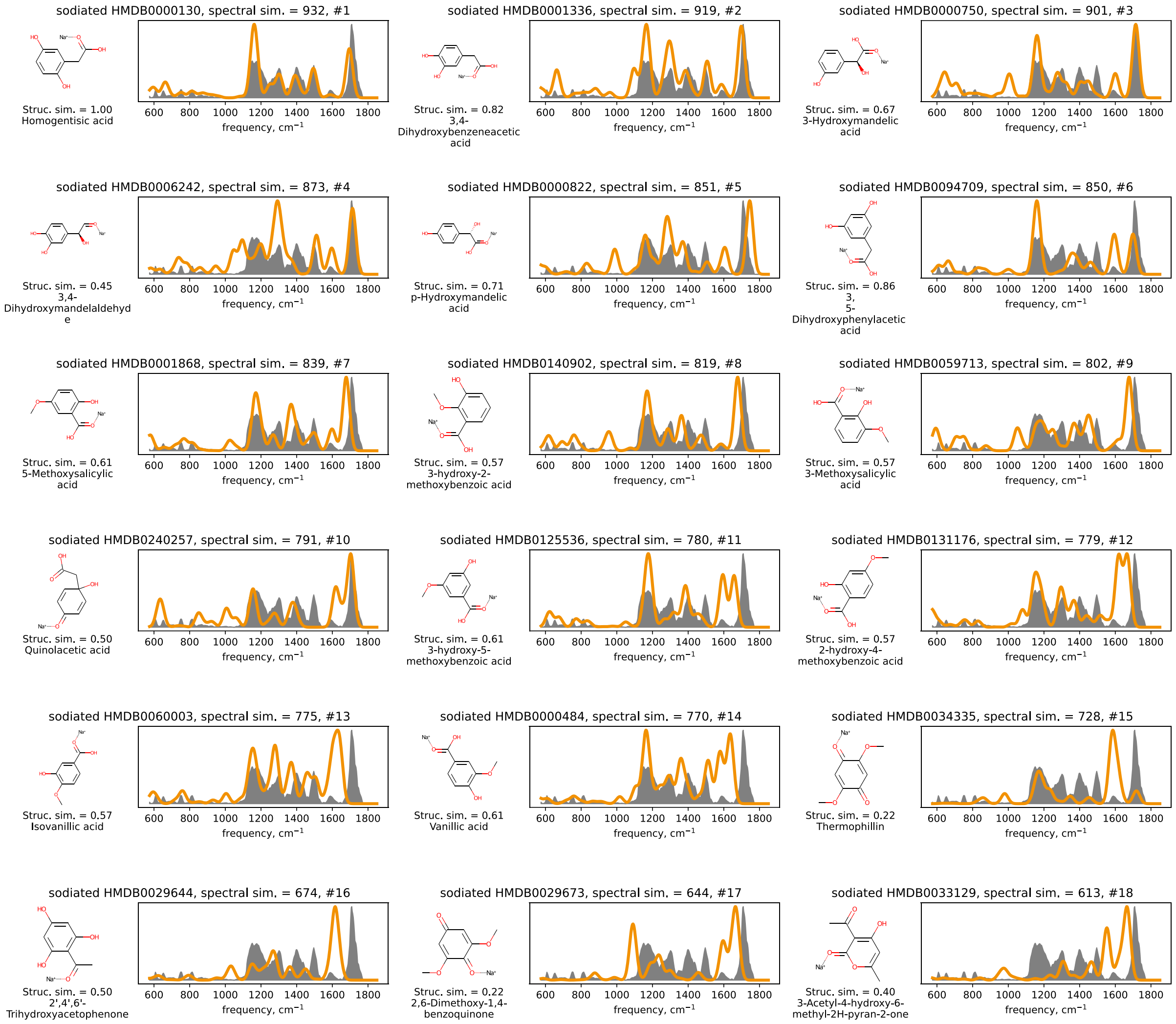
sodiated HMDB0000130, spectral sim. = 932, #1
Struc. sim. = 1.00
Homogentisic acid
sodiated HMDB0001336, spectral sim. = 919, #2
Struc. sim. = 0.82
3,4-Dihydroxybenzeneacetic acid
sodiated HMDB0000750, spectral sim. = 901, #3
Struc. sim. = 0.67
3-Hydroxymandelic acid
sodiated HMDB0006242, spectral sim. = 873, #4
Struc. sim. = 0.45
3,4-Dihydroxymandelaldehyde
sodiated HMDB0000822, spectral sim. = 851, #5
Struc. sim. = 0.71
p-Hydroxymandelic acid
sodiated HMDB0094709, spectral sim. = 850, #6
Struc. sim. = 0.86
3,5-Dihydroxyphenylacetic acid
sodiated HMDB0001868, spectral sim. = 839, #7
Struc. sim. = 0.61
5-Methoxysalicylic acid
sodiated HMDB0140902, spectral sim. = 819, #8
Struc. sim. = 0.57
3-hydroxy-2-methoxybenzoic acid
sodiated HMDB0059713, spectral sim. = 802, #9
Struc. sim. = 0.57
3-Methoxysalicylic acid
sodiated HMDB0240257, spectral sim. = 791, #10
Struc. sim. = 0.50
Quinolacetic acid
sodiated HMDB0125536, spectral sim. = 780, #11
Struc. sim. = 0.61
3-hydroxy-5-methoxybenzoic acid
sodiated HMDB0131176, spectral sim. = 779, #12
Struc. sim. = 0.57
2-hydroxy-4-methoxybenzoic acid
sodiated HMDB0060003, spectral sim. = 775, #13
Struc. sim. = 0.57
Isovanillic acid
sodiated HMDB0000484, spectral sim. = 770, #14
Struc. sim. = 0.61
Vanillic acid
sodiated HMDB0034335, spectral sim. = 728, #15
Struc. sim. = 0.22
Thermophillin
sodiated HMDB0029644, spectral sim. = 674, #16
Struc. sim. = 0.50
2',4',6'-Trihydroxyacetophenone
sodiated HMDB0029673, spectral sim. = 644, #17
Struc. sim. = 0.22
2,6-Dimethoxy-1,4-benzoquinone
sodiated HMDB0033129, spectral sim. = 613, #18
Struc. sim. = 0.40
3-Acetyl-4-hydroxy-6-methyl-2H-pyran-2-one
frequency, cm⁻¹

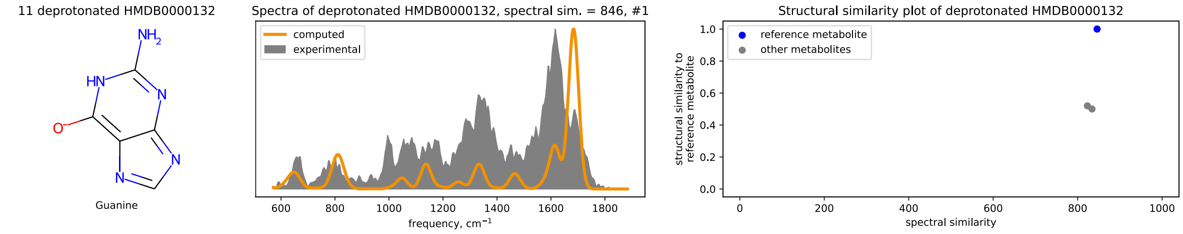
11 deprotonated HMDB0000132
Guanine
Spectra of deprotonated HMDB0000132, spectral sim. = 846, #1
computed
experimental
frequency, cm⁻¹
Structural similarity plot of deprotonated HMDB0000132
reference metabolite
other metabolites
structural similarity to reference metabolite
spectral similarity

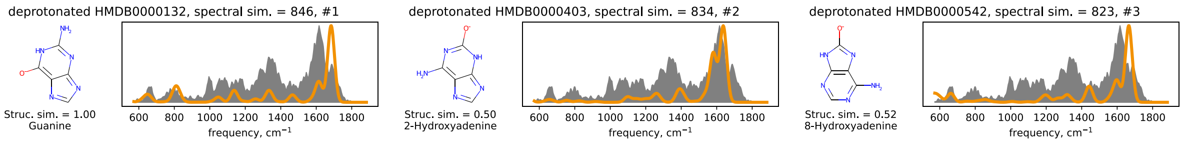
deprotonated HMDB0000132, spectral sim. = 846, #1
Struc. sim. = 1.00
Guanine
deprotonated HMDB0000403, spectral sim. = 834, #2
Struc. sim. = 0.50
2-Hydroxyadenine
deprotonated HMDB0000542, spectral sim. = 823, #3
Struc. sim. = 0.52
8-Hydroxyadenine
frequency, cm⁻¹

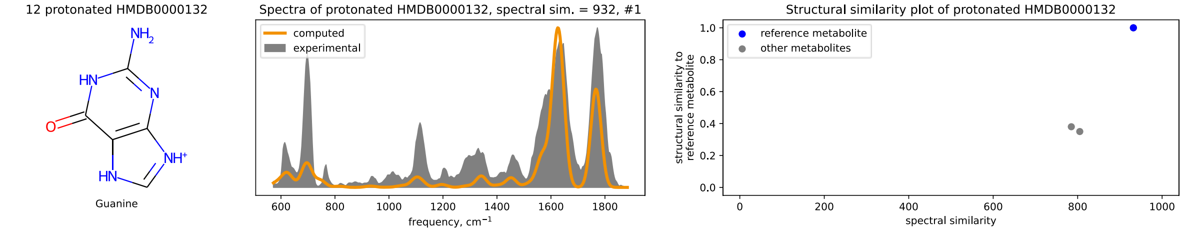
12 protonated HMDB0000132
Guanine
Spectra of protonated HMDB0000132, spectral sim. = 932, #1
computed
experimental
frequency, cm$^{-1}$
Structural similarity plot of protonated HMDB0000132
reference metabolite
other metabolites
structural similarity to reference metabolite
spectral similarity

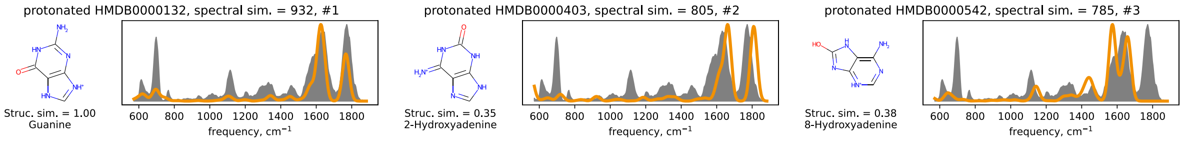
protonated HMDB0000132, spectral sim. = 932, #1
Struc. sim. = 1.00
Guanine
protonated HMDB0000403, spectral sim. = 805, #2
Struc. sim. = 0.35
2-Hydroxyadenine
protonated HMDB0000542, spectral sim. = 785, #3
Struc. sim. = 0.38
8-Hydroxyadenine
frequency, cm$^{-1}$

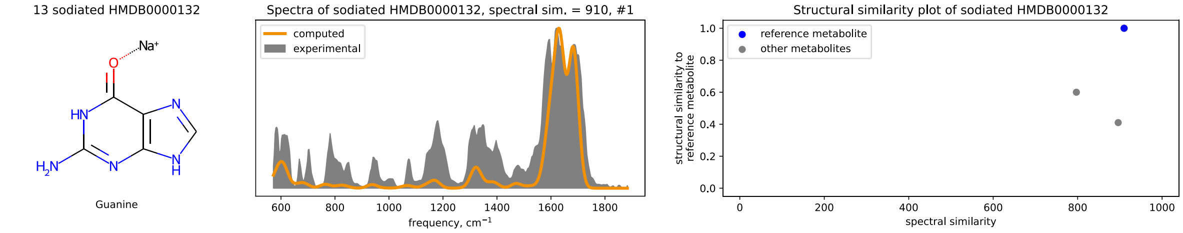
13 sodiated HMDB0000132
Guanine
Spectra of sodiated HMDB0000132, spectral sim. = 910, #1
computed
experimental
frequency, cm$^{-1}$
Structural similarity plot of sodiated HMDB0000132
reference metabolite
other metabolites
structural similarity to reference metabolite
spectral similarity

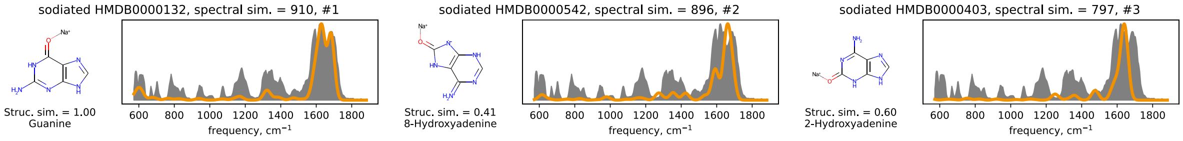
sodiated HMDB0000132, spectral sim. = 910, #1
Struc. sim. = 1.00
Guanine
sodiated HMDB0000542, spectral sim. = 896, #2
Struc. sim. = 0.41
8-Hydroxyadenine
sodiated HMDB0000403, spectral sim. = 797, #3
Struc. sim. = 0.60
2-Hydroxyadenine
frequency, cm$^{-1}$

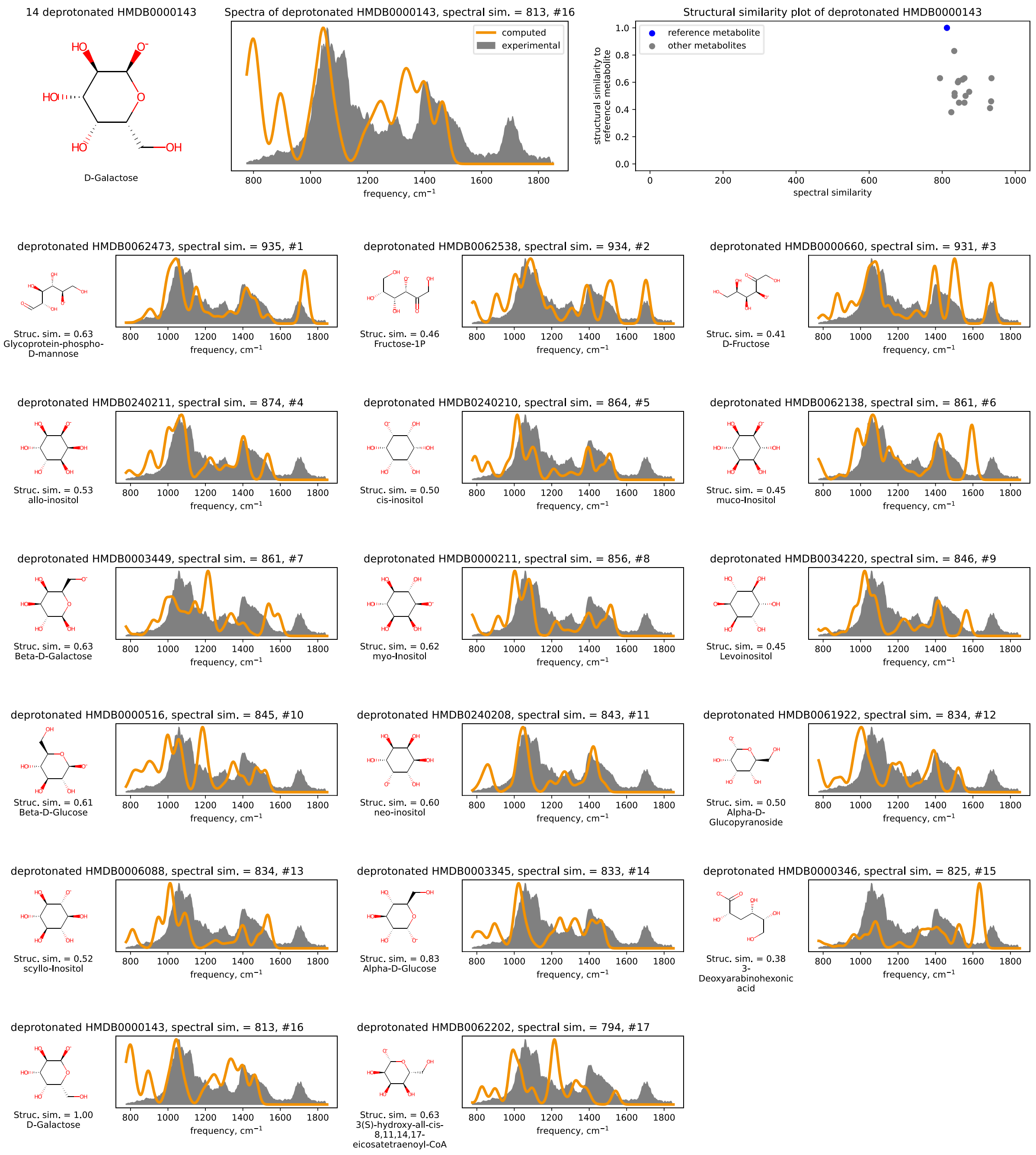

14 deprotonated HMDB0000143
D-Galactose
Spectra of deprotonated HMDB0000143, spectral sim. = 813, #16
computed
experimental
frequency, cm$^{-1}$
Structural similarity plot of deprotonated HMDB0000143
reference metabolite
other metabolites
structural similarity to reference metabolite
spectral similarity
deprotonated HMDB0062473, spectral sim. = 935, #1
Struc. sim. = 0.63
Glycoprotein-phospho-D-mannose
deprotonated HMDB0062538, spectral sim. = 934, #2
Struc. sim. = 0.46
Fructose-1P
deprotonated HMDB0000660, spectral sim. = 931, #3
Struc. sim. = 0.41
D-Fructose
deprotonated HMDB0240211, spectral sim. = 874, #4
Struc. sim. = 0.53
allo-inositol
deprotonated HMDB0240210, spectral sim. = 864, #5
Struc. sim. = 0.50
cis-inositol
deprotonated HMDB0062138, spectral sim. = 861, #6
Struc. sim. = 0.45
muco-Inositol
deprotonated HMDB0003449, spectral sim. = 861, #7
Struc. sim. = 0.63
Beta-D-Galactose
deprotonated HMDB0000211, spectral sim. = 856, #8
Struc. sim. = 0.62
myo-Inositol
deprotonated HMDB0034220, spectral sim. = 846, #9
Struc. sim. = 0.45
Levoinositol
deprotonated HMDB0000516, spectral sim. = 845, #10
Struc. sim. = 0.61
Beta-D-Glucose
deprotonated HMDB0240208, spectral sim. = 843, #11
Struc. sim. = 0.60
neo-inositol
deprotonated HMDB0061922, spectral sim. = 834, #12
Struc. sim. = 0.50
Alpha-D-Glucopyranoside
deprotonated HMDB0006088, spectral sim. = 834, #13
Struc. sim. = 0.52
scyllo-Inositol
deprotonated HMDB0003345, spectral sim. = 833, #14
Struc. sim. = 0.83
Alpha-D-Glucose
deprotonated HMDB0000346, spectral sim. = 825, #15
Struc. sim. = 0.38
3-Deoxyarabinohexonic acid
deprotonated HMDB0000143, spectral sim. = 813, #16
Struc. sim. = 1.00
D-Galactose
deprotonated HMDB0062202, spectral sim. = 794, #17
Struc. sim. = 0.63
3(S)-hydroxy-all-cis-8,11,14,17-eicosatetraenoyl-CoA

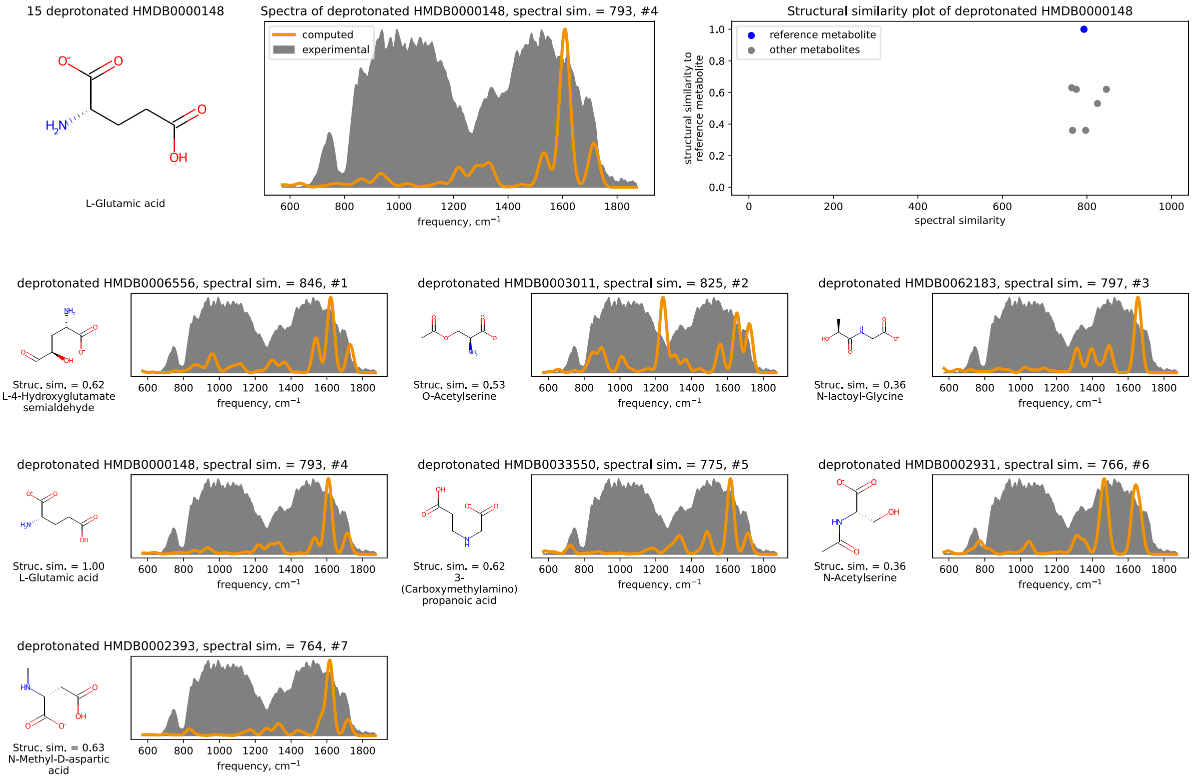
15 deprotonated HMDB0000148
L-Glutamic acid
Spectra of deprotonated HMDB0000148, spectral sim. = 793, #4
computed
experimental
frequency, cm$^{-1}$
Structural similarity plot of deprotonated HMDB0000148
reference metabolite
other metabolites
structural similarity to reference metabolite
spectral similarity
deprotonated HMDB0006556, spectral sim. = 846, #1
Struc. sim. = 0.62
L-4-Hydroxyglutamate semialdehyde
deprotonated HMDB0003011, spectral sim. = 825, #2
Struc. sim. = 0.53
O-Acetylserine
deprotonated HMDB0062183, spectral sim. = 797, #3
Struc. sim. = 0.36
N-lactoyl-Glycine
deprotonated HMDB0000148, spectral sim. = 793, #4
Struc. sim. = 1.00
L-Glutamic acid
deprotonated HMDB0033550, spectral sim. = 775, #5
Struc. sim. = 0.62
3-(Carboxymethylamino) propanoic acid
deprotonated HMDB0002931, spectral sim. = 766, #6
Struc. sim. = 0.36
N-Acetylserine
deprotonated HMDB0002393, spectral sim. = 764, #7
Struc. sim. = 0.63
N-Methyl-D-aspartic acid

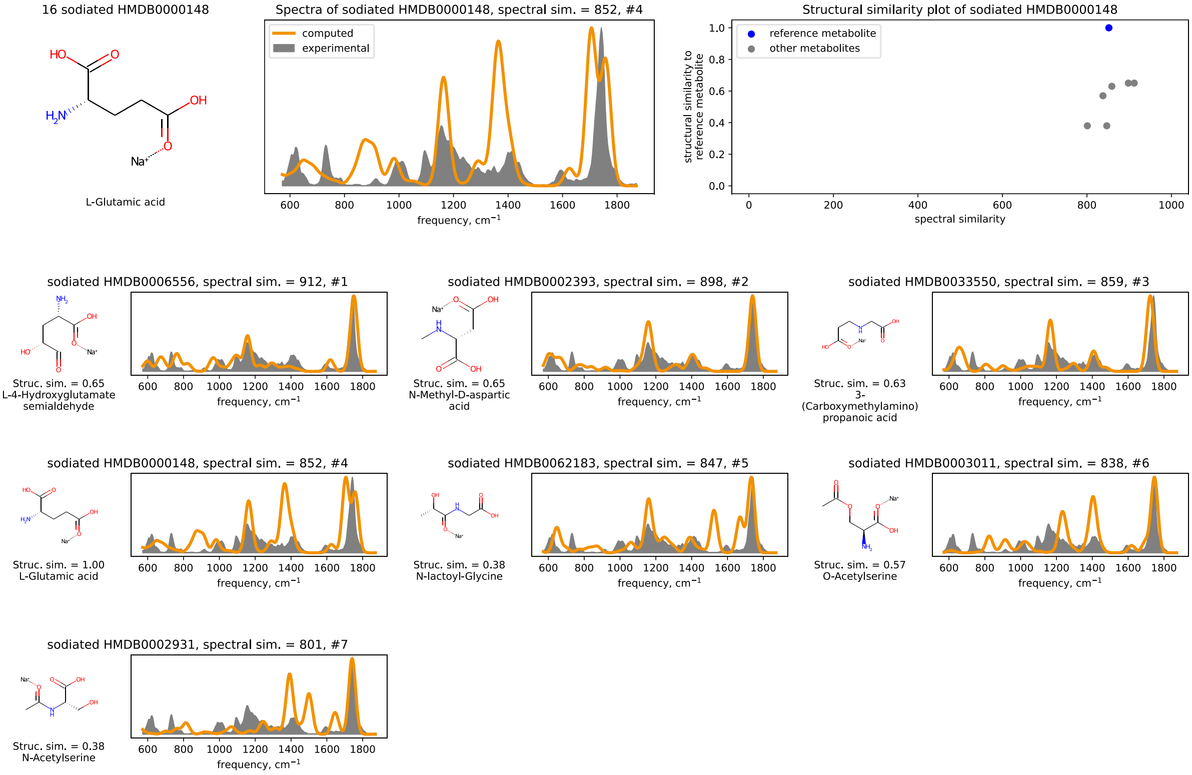
16 sodiated HMDB0000148
L-Glutamic acid
Spectra of sodiated HMDB0000148, spectral sim. = 852, #4
computed
experimental
frequency, cm$^{-1}$
Structural similarity plot of sodiated HMDB0000148
reference metabolite
other metabolites
structural similarity to reference metabolite
spectral similarity
sodiated HMDB0006556, spectral sim. = 912, #1
Struc. sim. = 0.65
L-4-Hydroxyglutamate semialdehyde
sodiated HMDB0002393, spectral sim. = 898, #2
Struc. sim. = 0.65
N-Methyl-D-aspartic acid
sodiated HMDB0033550, spectral sim. = 859, #3
Struc. sim. = 0.63
3-(Carboxymethylamino) propanoic acid
sodiated HMDB0000148, spectral sim. = 852, #4
Struc. sim. = 1.00
L-Glutamic acid
sodiated HMDB0062183, spectral sim. = 847, #5
Struc. sim. = 0.38
N-lactoyl-Glycine
sodiated HMDB0003011, spectral sim. = 838, #6
Struc. sim. = 0.57
O-Acetylserine
sodiated HMDB0002931, spectral sim. = 801, #7
Struc. sim. = 0.38
N-Acetylserine

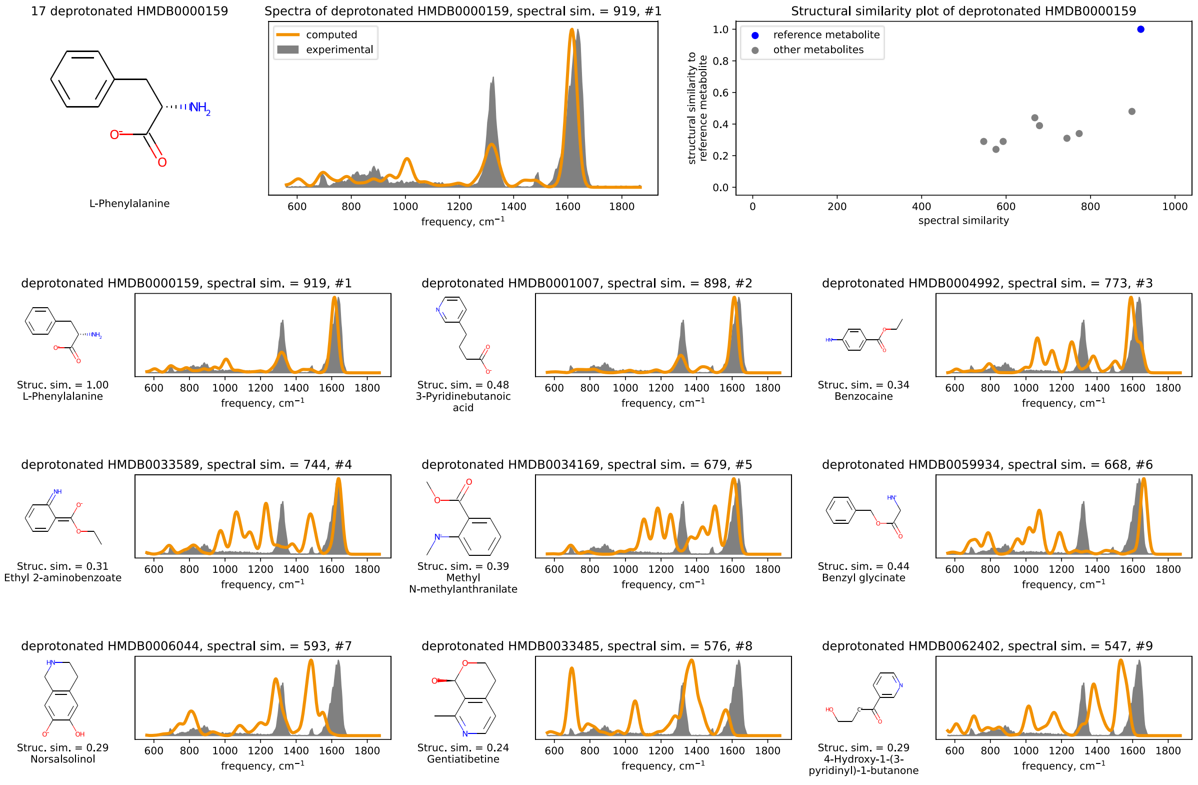
17 deprotonated HMDB0000159
L-Phenylalanine
Spectra of deprotonated HMDB0000159, spectral sim. = 919, #1
computed
experimental
frequency, cm−1
Structural similarity plot of deprotonated HMDB0000159
reference metabolite
other metabolites
structural similarity to reference metabolite
spectral similarity
deprotonated HMDB0000159, spectral sim. = 919, #1
Struc. sim. = 1.00
L-Phenylalanine
deprotonated HMDB0001007, spectral sim. = 898, #2
Struc. sim. = 0.48
3-Pyridinebutanoic acid
deprotonated HMDB0004992, spectral sim. = 773, #3
Struc. sim. = 0.34
Benzocaine
deprotonated HMDB0033589, spectral sim. = 744, #4
Struc. sim. = 0.31
Ethyl 2-aminobenzoate
deprotonated HMDB0034169, spectral sim. = 679, #5
Struc. sim. = 0.39
Methyl N-methylanthranilate
deprotonated HMDB0059934, spectral sim. = 668, #6
Struc. sim. = 0.44
Benzyl glycinate
deprotonated HMDB0006044, spectral sim. = 593, #7
Struc. sim. = 0.29
Norsalsolinol
deprotonated HMDB0033485, spectral sim. = 576, #8
Struc. sim. = 0.24
Gentiatibetine
deprotonated HMDB0062402, spectral sim. = 547, #9
Struc. sim. = 0.29
4-Hydroxy-1-(3-pyridinyl)-1-butanone

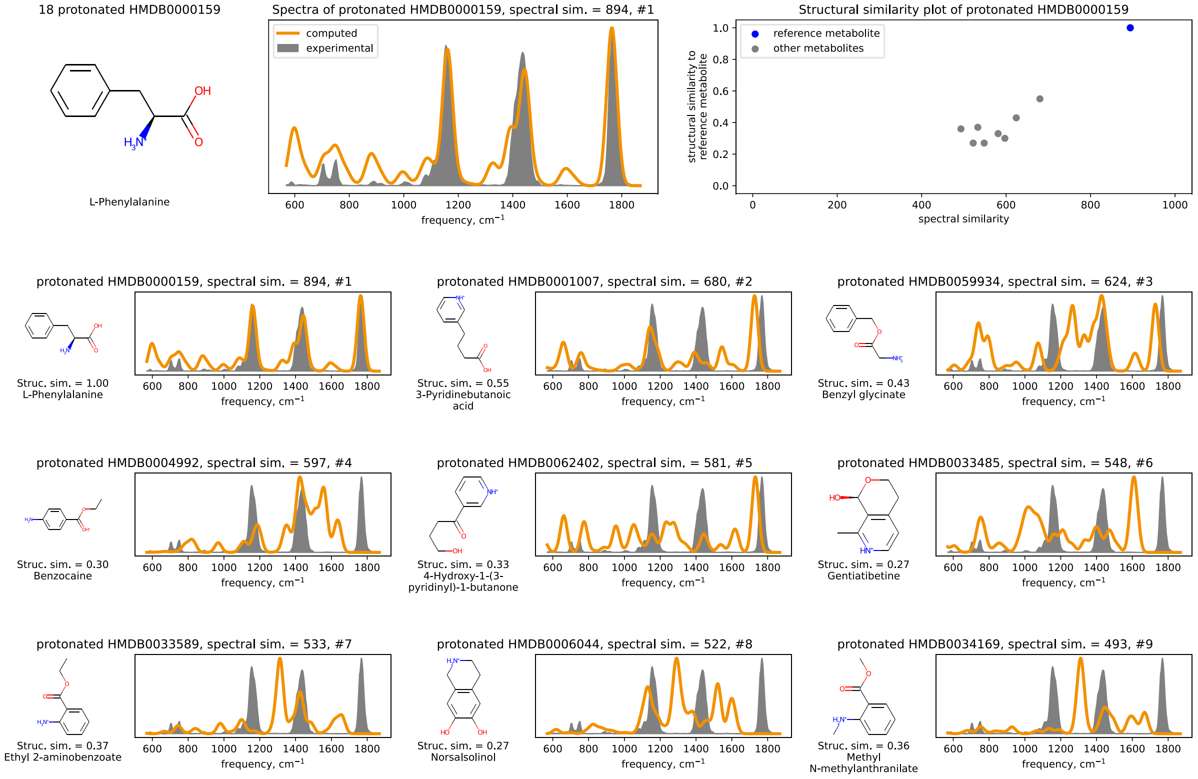
18 protonated HMDB0000159
L-Phenylalanine
Spectra of protonated HMDB0000159, spectral sim. = 894, #1
computed
experimental
frequency, cm−1
Structural similarity plot of protonated HMDB0000159
reference metabolite
other metabolites
structural similarity to reference metabolite
spectral similarity
protonated HMDB0000159, spectral sim. = 894, #1
Struc. sim. = 1.00
L-Phenylalanine
protonated HMDB0001007, spectral sim. = 680, #2
Struc. sim. = 0.55
3-Pyridinebutanoic acid
protonated HMDB0059934, spectral sim. = 624, #3
Struc. sim. = 0.43
Benzyl glycinate
protonated HMDB0004992, spectral sim. = 597, #4
Struc. sim. = 0.30
Benzocaine
protonated HMDB0062402, spectral sim. = 581, #5
Struc. sim. = 0.33
4-Hydroxy-1-(3-pyridinyl)-1-butanone
protonated HMDB0033485, spectral sim. = 548, #6
Struc. sim. = 0.27
Gentiatibetine
protonated HMDB0033589, spectral sim. = 533, #7
Struc. sim. = 0.37
Ethyl 2-aminobenzoate
protonated HMDB0006044, spectral sim. = 522, #8
Struc. sim. = 0.27
Norsalsolinol
protonated HMDB0034169, spectral sim. = 493, #9
Struc. sim. = 0.36
Methyl N-methylanthranilate

19 sodiated HMDB0000159

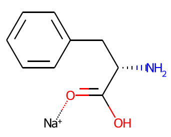


L-Phenylalanine

Spectra of sodiated HMDB0000159, spectral sim. = 825, #1

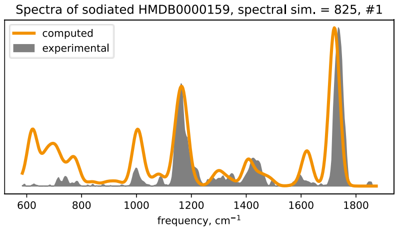


Structural similarity plot of sodiated HMDB0000159

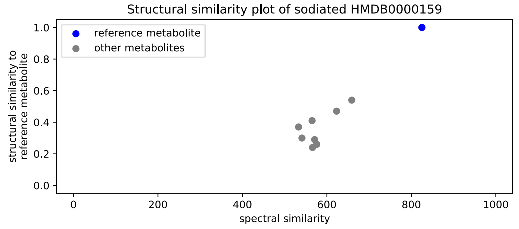


sodiated HMDB0000159, spectral sim. = 825, #1

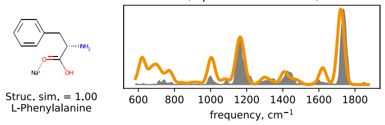


sodiated HMDB0001007, spectral sim. = 659, #2

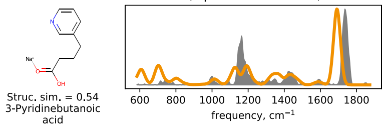


sodiated HMDB0059934, spectral sim. = 623, #3

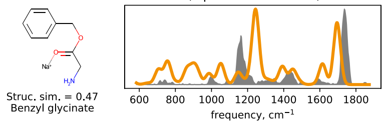


sodiated HMDB0033485, spectral sim. = 576, #4

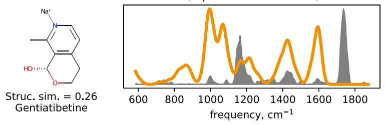


sodiated HMDB0004992, spectral sim. = 571, #5

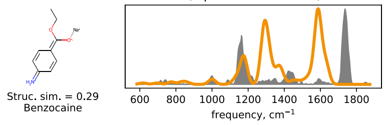


sodiated HMDB0006044, spectral sim. = 566, #6

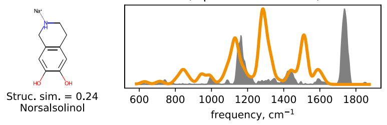


sodiated HMDB0033589, spectral sim. = 565, #7

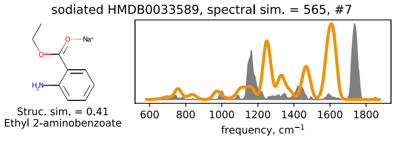


sodiated HMDB0062402, spectral sim. = 541, #8

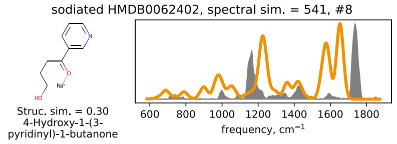


sodiated HMDB0034169, spectral sim. = 533, #9

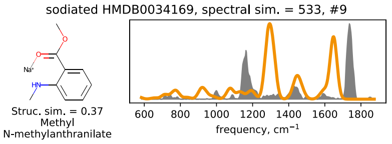


20 protonated HMDB0000172

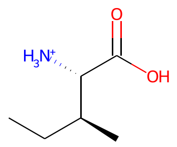


L-Isoleucine

Spectra of protonated HMDB0000172, spectral sim. = 900, #2

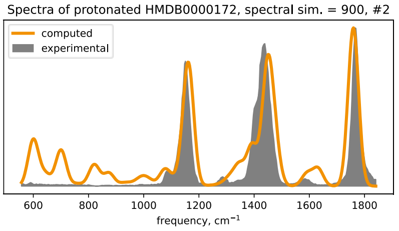


Structural similarity plot of protonated HMDB0000172

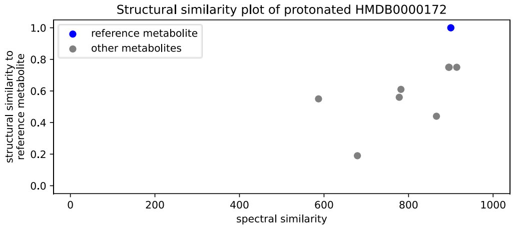


protonated HMDB0000687, spectral sim. = 914, #1

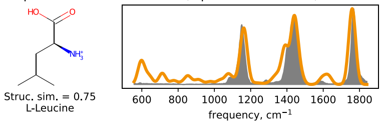


protonated HMDB0000172, spectral sim. = 900, #2

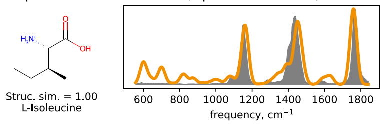


protonated HMDB0000557, spectral sim. = 896, #3

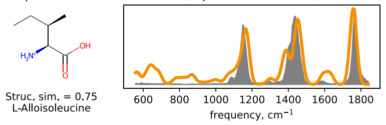


protonated HMDB0001645, spectral sim. = 895, #4

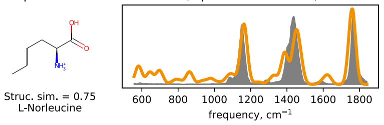


protonated HMDB0061716, spectral sim. = 866, #5

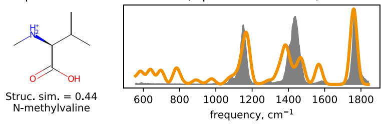


protonated HMDB0029168, spectral sim. = 782, #6

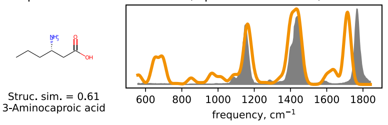


protonated HMDB0003640, spectral sim. = 778, #7

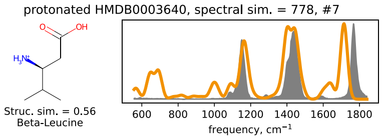


protonated HMDB0060650, spectral sim. = 679, #8

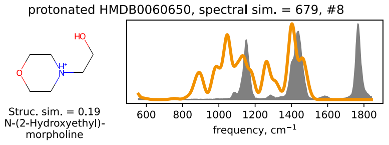


protonated HMDB0001901, spectral sim. = 587, #9

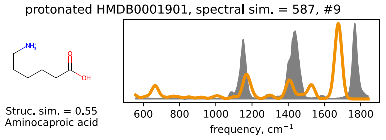

21 deprotonated HMDB0000182

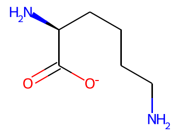


L-Lysine

Spectra of deprotonated HMDB0000182, spectral sim. = 852, #3

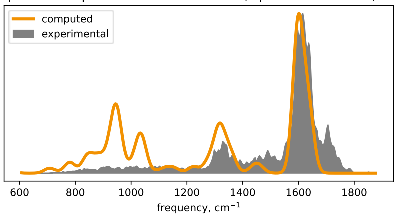


Structural similarity plot of deprotonated HMDB0000182

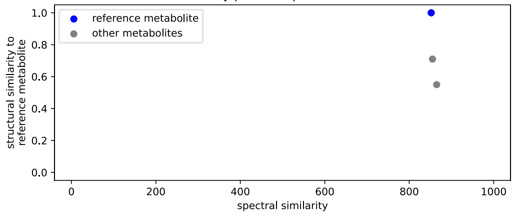


deprotonated HMDB0012115, spectral sim. = 865, #1

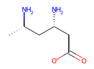

Struc. sim. = 0.55
(3S,5S)-3,5-Diaminohexanoate

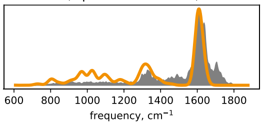


deprotonated HMDB0012114, spectral sim. = 855, #2

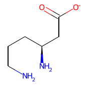

Struc. sim. = 0.71
(3S)-3,6-Diaminohexanoate

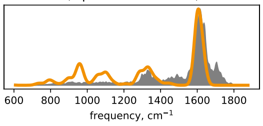


deprotonated HMDB0000182, spectral sim. = 852, #3

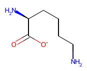

Struc. sim. = 1.00
L-Lysine

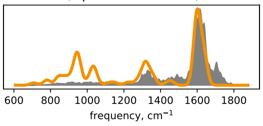


22 protonated HMDB0000182

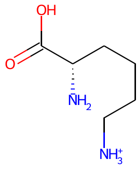


L-Lysine

Spectra of protonated HMDB0000182, spectral sim. = 871, #3

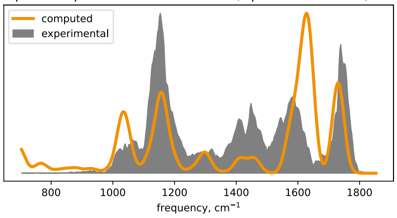


Structural similarity plot of protonated HMDB0000182

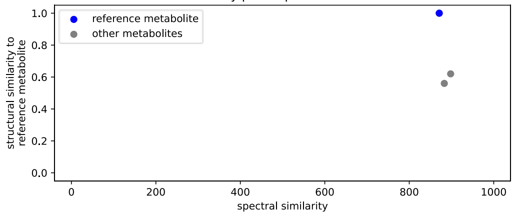


protonated HMDB0012114, spectral sim. = 898, #1

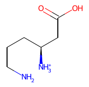

Struc. sim. = 0.62
(3S)-3,6-Diaminohexanoate

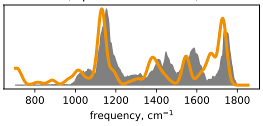


protonated HMDB0012115, spectral sim. = 883, #2

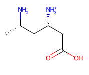

Struc. sim. = 0.56
(3S,5S)-3,5-Diaminohexanoate

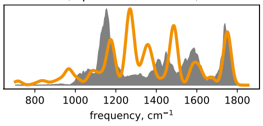


protonated HMDB0000182, spectral sim. = 871, #3

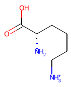

Struc. sim. = 1.00
L-Lysine

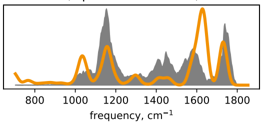


23 sodiated HMDB0000182

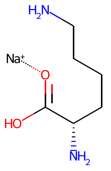


L-Lysine

Spectra of sodiated HMDB0000182, spectral sim. = 869, #1

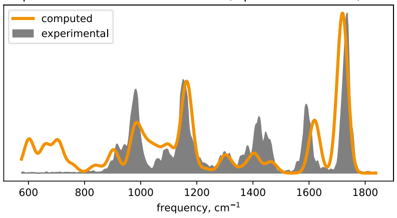


Structural similarity plot of sodiated HMDB0000182

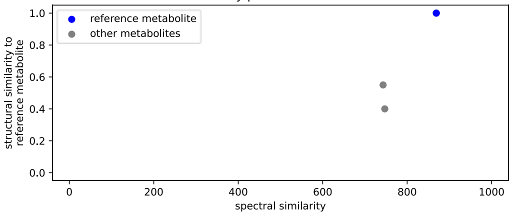


sodiated HMDB0000182, spectral sim. = 869, #1

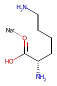

Struc. sim. = 1.00
L-Lysine

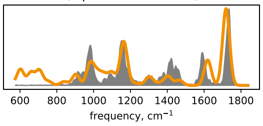


sodiated HMDB0012115, spectral sim. = 747, #2

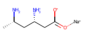

Struc. sim. = 0.40
(3S,5S)-3,5-Diaminohexanoate

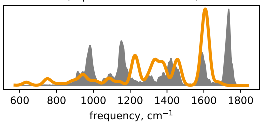


sodiated HMDB0012114, spectral sim. = 743, #3

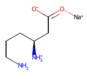

Struc. sim. = 0.55
(3S)-3,6-Diaminohexanoate

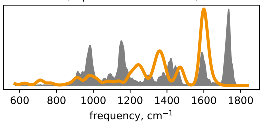


24 deprotonated HMDB0000191

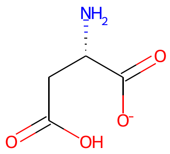


L-Aspartic acid

Spectra of deprotonated HMDB0000191, spectral sim. = 716, #1

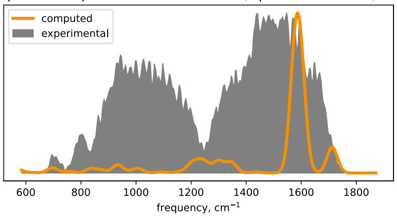


Structural similarity plot of deprotonated HMDB0000191

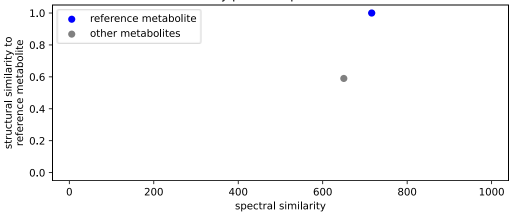


deprotonated HMDB0000191, spectral sim. = 716, #1

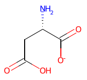

Struc. sim. = 1.00
L-Aspartic acid

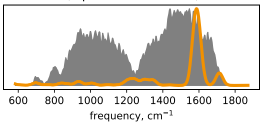


deprotonated HMDB0011753, spectral sim. = 650, #2

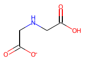

Struc. sim. = 0.59
Iminodiacetic acid

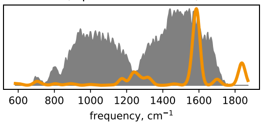

25 protonated HMDB0000191 | Spectra of protonated HMDB0000191, spectral sim. = 940, #1 | Structural similarity plot of protonated HMDB0000191

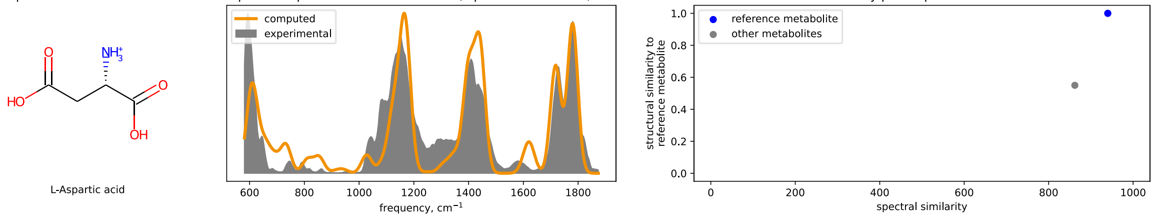


protonated HMDB0000191, spectral sim. = 940, #1 | protonated HMDB0011753, spectral sim. = 862, #2

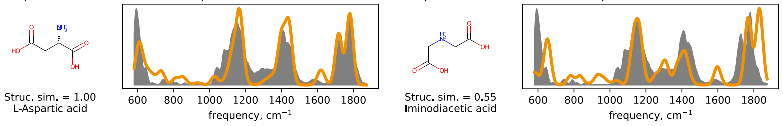


26 sodiated HMDB0000191 | Spectra of sodiated HMDB0000191, spectral sim. = 909, #1 | Structural similarity plot of sodiated HMDB0000191

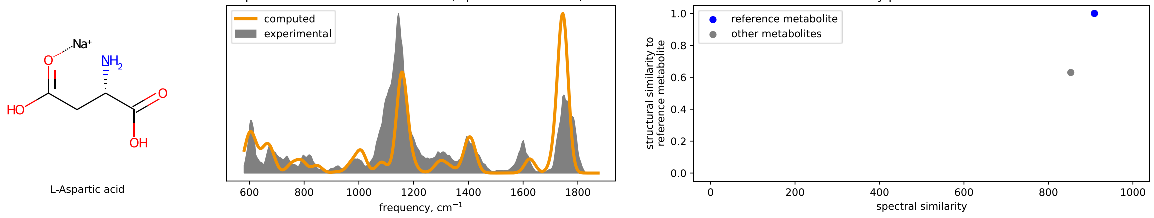


sodiated HMDB0000191, spectral sim. = 909, #1 | sodiated HMDB0011753, spectral sim. = 853, #2

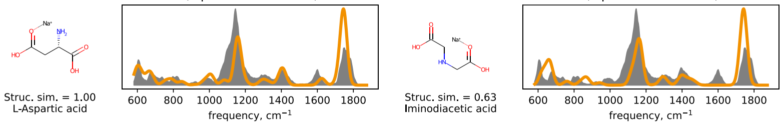


27 protonated HMDB0000201 | Spectra of protonated HMDB0000201, spectral sim. = 848, #1 | Structural similarity plot of protonated HMDB0000201

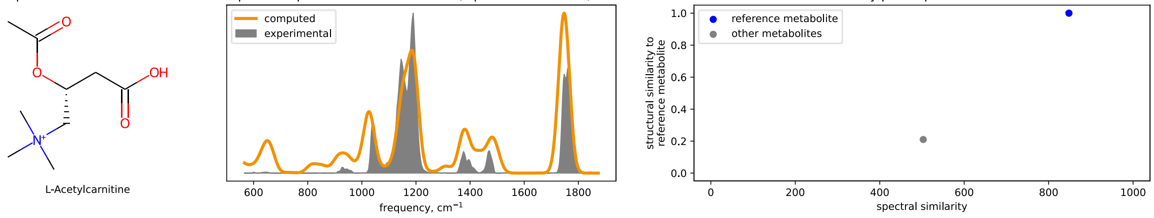


protonated HMDB0000201, spectral sim. = 848, #1 | protonated HMDB0062176, spectral sim. = 503, #2

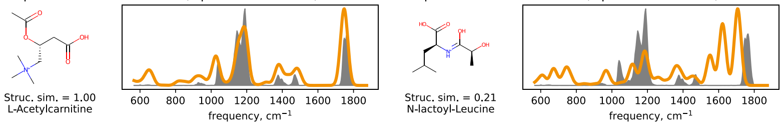


28 sodiated HMDB0000201 | Spectra of sodiated HMDB0000201, spectral sim. = 893, #1 | Structural similarity plot of sodiated HMDB0000201

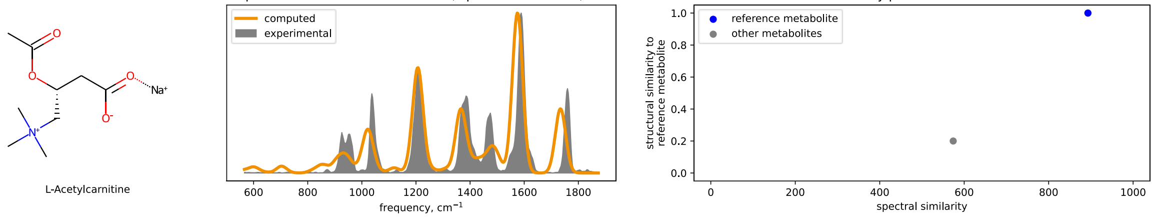


sodiated HMDB0000201, spectral sim. = 893, #1 | sodiated HMDB0062176, spectral sim. = 574, #2

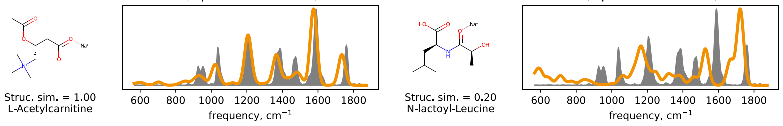

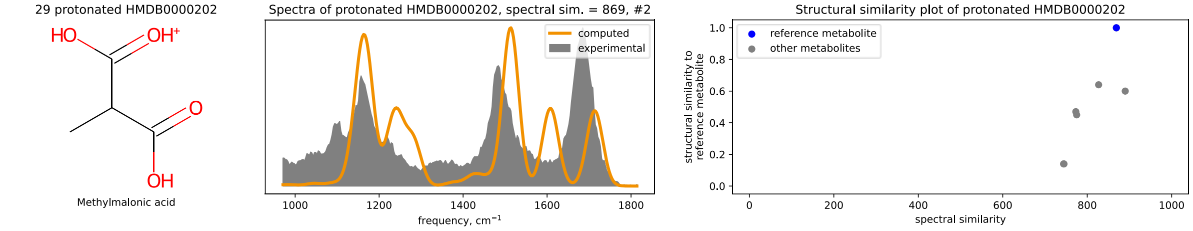
29 protonated HMDB0000202
Methylmalonic acid
Spectra of protonated HMDB0000202, spectral sim. = 869, #2
computed
experimental
frequency, cm⁻¹
Structural similarity plot of protonated HMDB0000202
reference metabolite
other metabolites
structural similarity to reference metabolite
spectral similarity

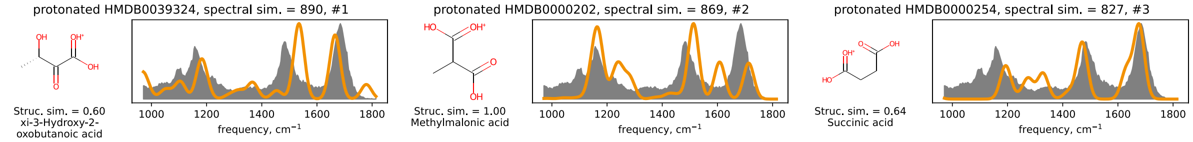
protonated HMDB0039324, spectral sim. = 890, #1
Struc. sim. = 0.60
xi-3-Hydroxy-2-oxobutanoic acid
protonated HMDB0000202, spectral sim. = 869, #2
Struc. sim. = 1.00
Methylmalonic acid
protonated HMDB0000254, spectral sim. = 827, #3
Struc. sim. = 0.64
Succinic acid
frequency, cm⁻¹

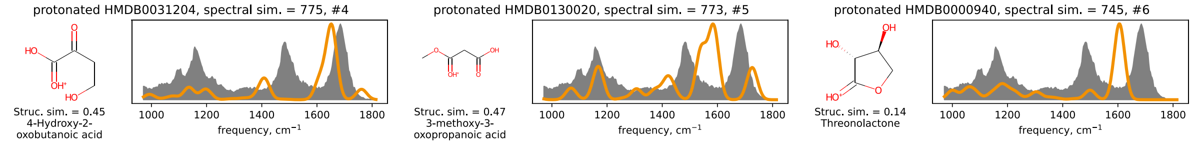
protonated HMDB0031204, spectral sim. = 775, #4
Struc. sim. = 0.45
4-Hydroxy-2-oxobutanoic acid
protonated HMDB0130020, spectral sim. = 773, #5
Struc. sim. = 0.47
3-methoxy-3-oxopropanoic acid
protonated HMDB0000940, spectral sim. = 745, #6
Struc. sim. = 0.14
Threonolactone
frequency, cm⁻¹

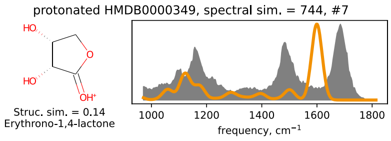
protonated HMDB0000349, spectral sim. = 744, #7
Struc. sim. = 0.14
Erythrono-1,4-lactone
frequency, cm⁻¹

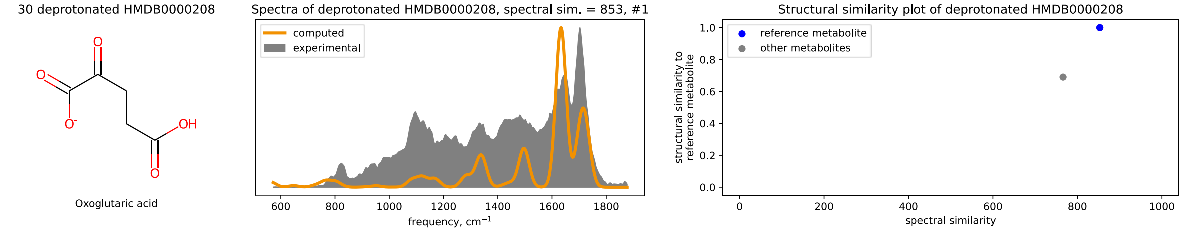
30 deprotonated HMDB0000208
Oxoglutaric acid
Spectra of deprotonated HMDB0000208, spectral sim. = 853, #1
computed
experimental
frequency, cm⁻¹
Structural similarity plot of deprotonated HMDB0000208
reference metabolite
other metabolites
structural similarity to reference metabolite
spectral similarity

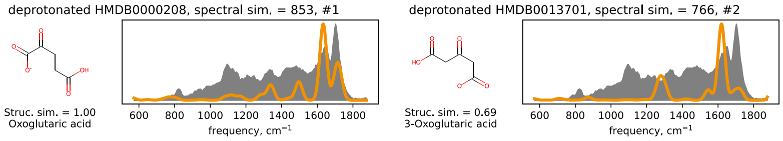
deprotonated HMDB0000208, spectral sim. = 853, #1
Struc. sim. = 1.00
Oxoglutaric acid
deprotonated HMDB0013701, spectral sim. = 766, #2
Struc. sim. = 0.69
3-Oxoglutaric acid
frequency, cm⁻¹

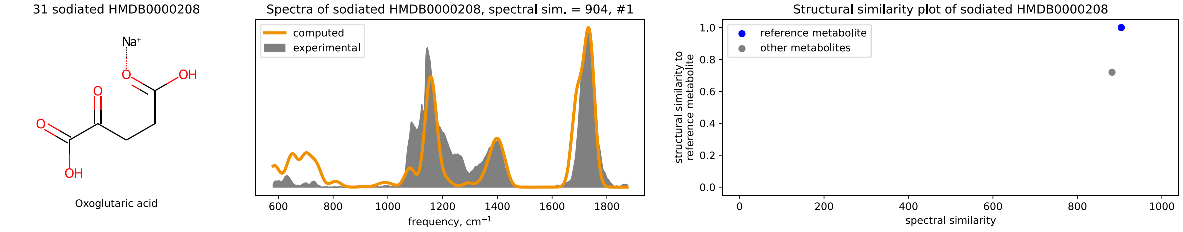
31 sodiated HMDB0000208
Oxoglutaric acid
Spectra of sodiated HMDB0000208, spectral sim. = 904, #1
computed
experimental
frequency, cm⁻¹
Structural similarity plot of sodiated HMDB0000208
reference metabolite
other metabolites
structural similarity to reference metabolite
spectral similarity

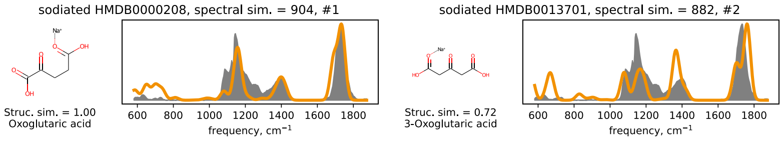
sodiated HMDB0000208, spectral sim. = 904, #1
Struc. sim. = 1.00
Oxoglutaric acid
sodiated HMDB0013701, spectral sim. = 882, #2
Struc. sim. = 0.72
3-Oxoglutaric acid
frequency, cm⁻¹

32 deprotonated HMDB0000211

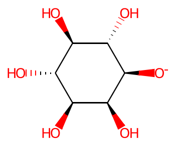

myo-Inositol

Spectra of deprotonated HMDB0000211, spectral sim. = 838, #3

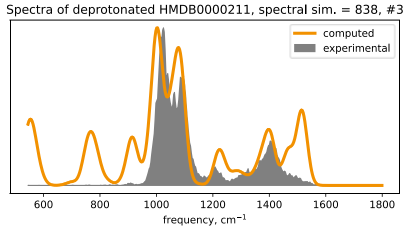


Structural similarity plot of deprotonated HMDB0000211

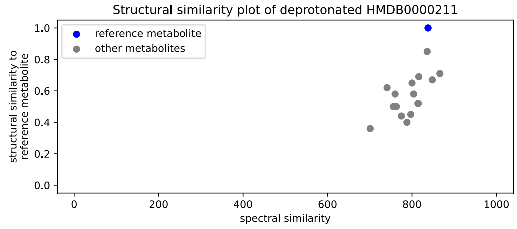


deprotonated HMDB0240210, spectral sim. = 866, #1

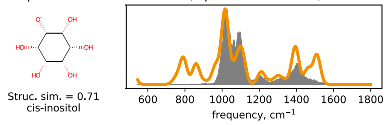

Struc. sim. = 0.71
cis-inositol

deprotonated HMDB0006088, spectral sim. = 848, #2

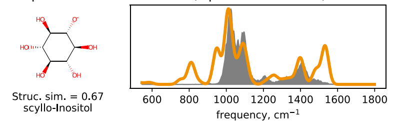

Struc. sim. = 0.67
scyllo-Inositol

deprotonated HMDB0000211, spectral sim. = 838, #3

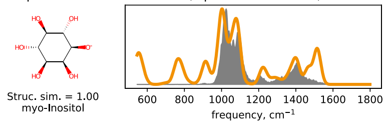

Struc. sim. = 1.00
myo-Inositol

deprotonated HMDB0240211, spectral sim. = 836, #4

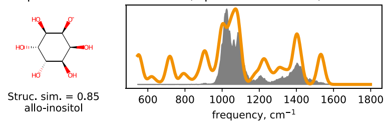

Struc. sim. = 0.85
allo-inositol

deprotonated HMDB0240208, spectral sim. = 816, #5

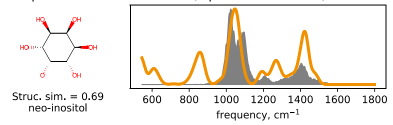

Struc. sim. = 0.69
neo-inositol

deprotonated HMDB0034220, spectral sim. = 815, #6

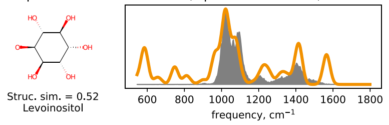

Struc. sim. = 0.52
Levoinositol

deprotonated HMDB0062138, spectral sim. = 814, #7

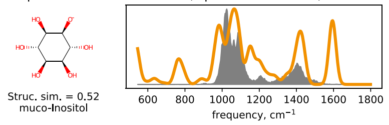

Struc. sim. = 0.52
muco-Inositol

deprotonated HMDB0062473, spectral sim. = 804, #8

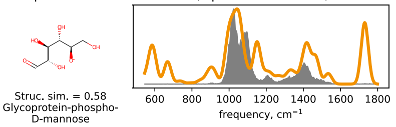

Struc. sim. = 0.58
Glycoprotein-phospho-D-mannose

deprotonated HMDB0003449, spectral sim. = 800, #9

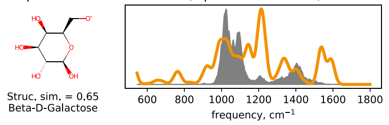

Struc. sim. = 0.65
Beta-D-Galactose

deprotonated HMDB0062538, spectral sim. = 797, #10

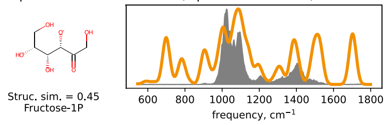

Struc. sim. = 0.45
Fructose-1P

deprotonated HMDB0000660, spectral sim. = 788, #11

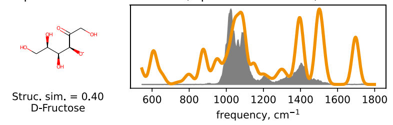

Struc. sim. = 0.40
D-Fructose

deprotonated HMDB0061922, spectral sim. = 775, #12

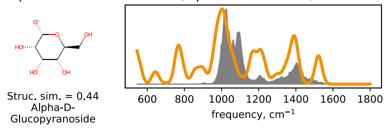

Struc. sim. = 0.44
Alpha-D-Glucopyranoside

deprotonated HMDB0000516, spectral sim. = 763, #13

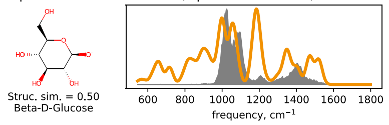

Struc. sim. = 0.50
Beta-D-Glucose

deprotonated HMDB0062202, spectral sim. = 760, #14

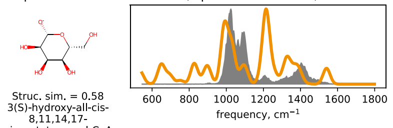

Struc. sim. = 0.58
3(S)-hydroxy-all-cis-8,11,14,17-eicosatetraenoyl-CoA

deprotonated HMDB0003345, spectral sim. = 756, #15

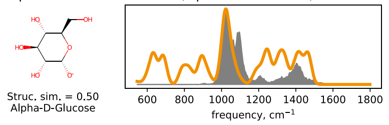

Struc. sim. = 0.50
Alpha-D-Glucose

deprotonated HMDB0000143, spectral sim. = 741, #16

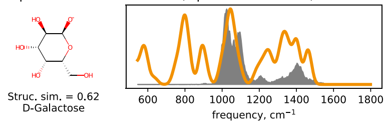

Struc. sim. = 0.62
D-Galactose

deprotonated HMDB0000346, spectral sim. = 701, #17

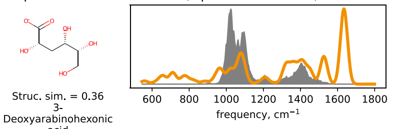

Struc. sim. = 0.36
3-Deoxyarabinohexonic acid

33 deprotonated HMDB0000214

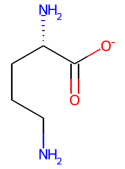

Ornithine

Spectra of deprotonated HMDB0000214, spectral sim. = 871, #1

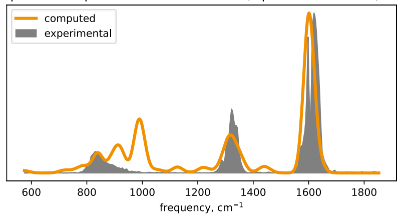


Structural similarity plot of deprotonated HMDB0000214

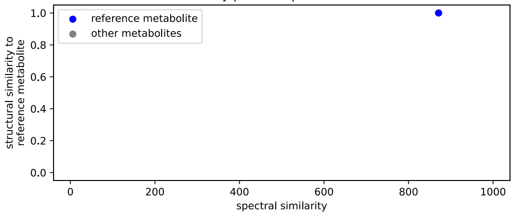


deprotonated HMDB0000214, spectral sim. = 871, #1

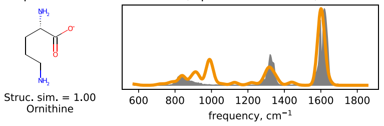

Struc. sim. = 1.00
Ornithine

34 protonated HMDB0000214

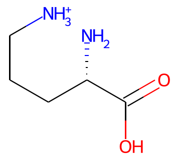


Ornithine

Spectra of protonated HMDB0000214, spectral sim. = 938, #1

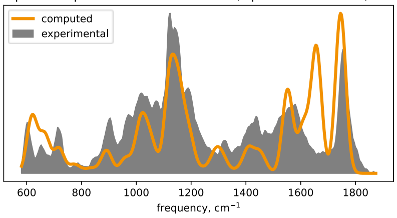


Structural similarity plot of protonated HMDB0000214

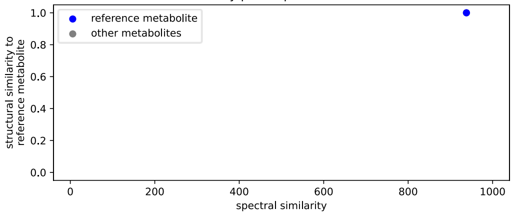


protonated HMDB0000214, spectral sim. = 938, #1

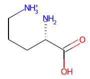

Struc. sim. = 1.00
Ornithine

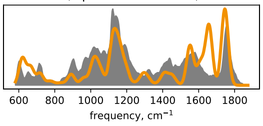


35 sodiated HMDB0000214

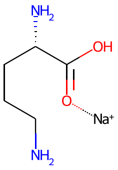


Ornithine

Spectra of sodiated HMDB0000214, spectral sim. = 770, #1

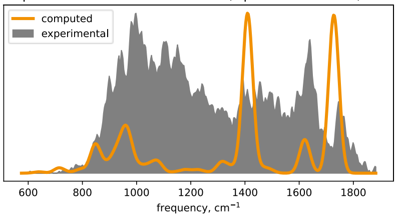


Structural similarity plot of sodiated HMDB0000214

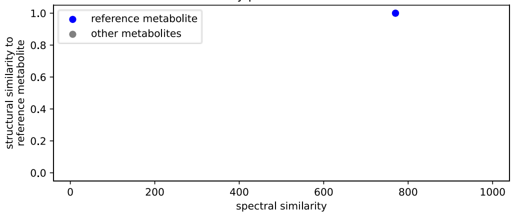


sodiated HMDB0000214, spectral sim. = 770, #1

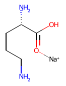

Struc. sim. = 1.00
Ornithine

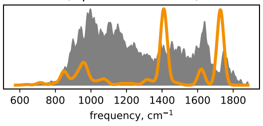


36 deprotonated HMDB0000247

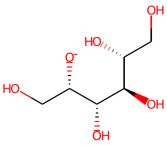


Sorbitol

Spectra of deprotonated HMDB0000247, spectral sim. = 914, #2

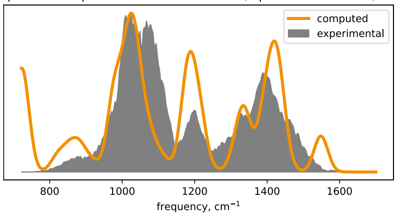


Structural similarity plot of deprotonated HMDB0000247

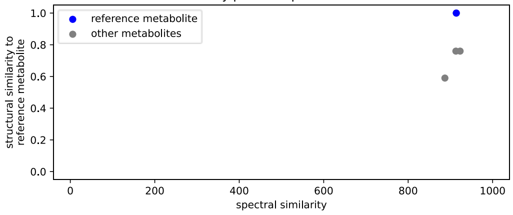


deprotonated HMDB0011632, spectral sim. = 923, #1

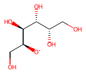

Struc. sim. = 0.76
L-Iditol

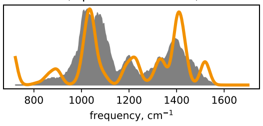


deprotonated HMDB0000247, spectral sim. = 914, #2

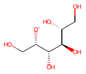

Struc. sim. = 1.00
Sorbitol

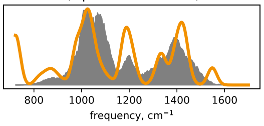


deprotonated HMDB0000107, spectral sim. = 913, #3

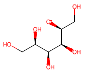

Struc. sim. = 0.76
Galactitol

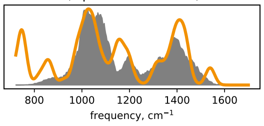


deprotonated HMDB0000765, spectral sim. = 887, #4

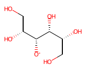

Struc. sim. = 0.59
Mannitol

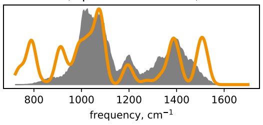

37 deprotonated HMDB0000251 — Spectra of deprotonated HMDB0000251, spectral sim. = 459, #1 — Structural similarity plot of deprotonated HMDB0000251

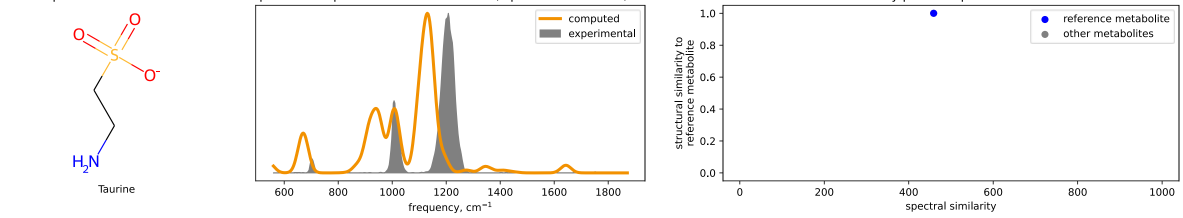


deprotonated HMDB0000251, spectral sim. = 459, #1

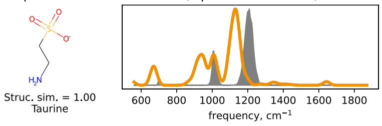


38 sodiated HMDB0000251 — Spectra of sodiated HMDB0000251, spectral sim. = 744, #1 — Structural similarity plot of sodiated HMDB0000251

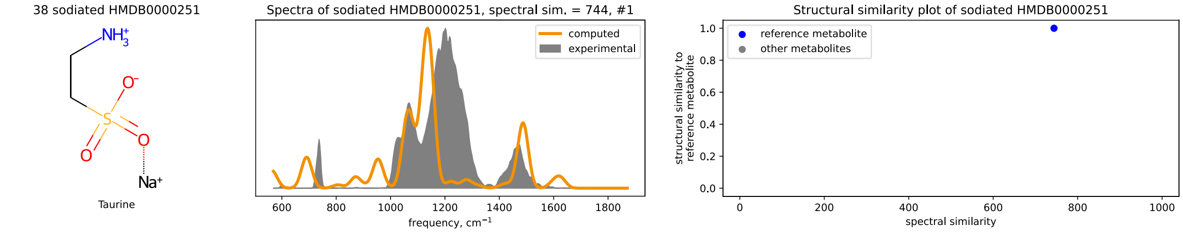


sodiated HMDB0000251, spectral sim. = 744, #1

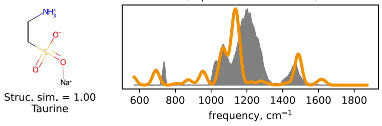


39 deprotonated HMDB0000254 — Spectra of deprotonated HMDB0000254, spectral sim. = 754, #3 — Structural similarity plot of deprotonated HMDB0000254

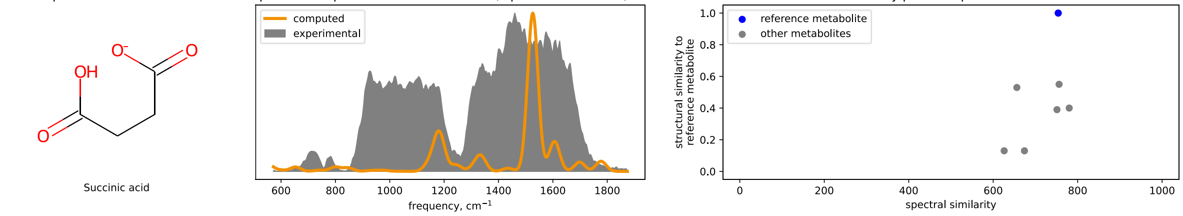


deprotonated HMDB0039324, spectral sim. = 780, #1

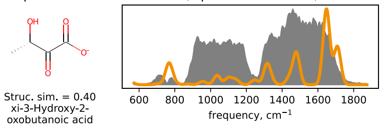


deprotonated HMDB0000202, spectral sim. = 756, #2

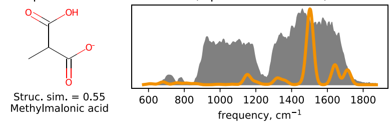


deprotonated HMDB0000254, spectral sim. = 754, #3

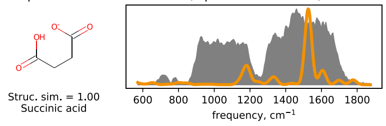


deprotonated HMDB0031204, spectral sim. = 751, #4

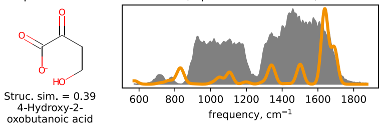


deprotonated HMDB0000349, spectral sim. = 674, #5

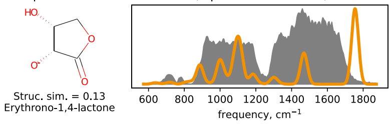


deprotonated HMDB0130020, spectral sim. = 656, #6

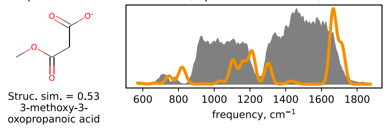


deprotonated HMDB0000940, spectral sim. = 626, #7

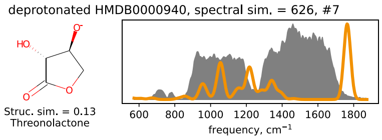

40 protonated HMDB0000254

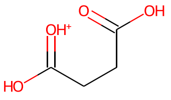

Succinic acid

Spectra of protonated HMDB0000254, spectral sim. = 718, #6

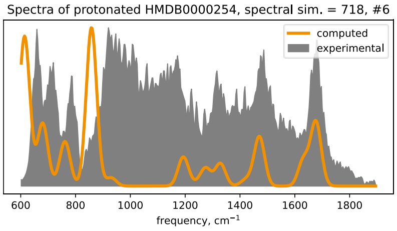


Structural similarity plot of protonated HMDB0000254

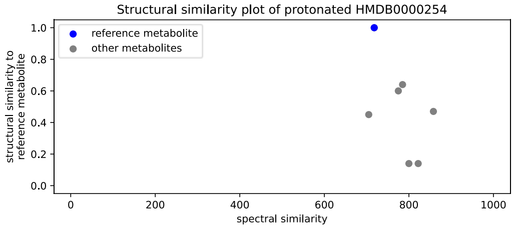


protonated HMDB0039324, spectral sim. = 858, #1

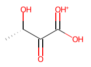

Struc. sim. = 0.47
xi-3-Hydroxy-2-oxobutanoic acid

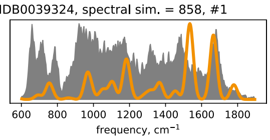

protonated HMDB0000940, spectral sim. = 822, #2

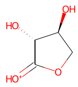

Struc. sim. = 0.14
Threonolactone

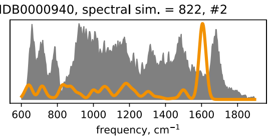

protonated HMDB0000349, spectral sim. = 800, #3

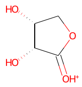

Struc. sim. = 0.14
Erythrono-1,4-lactone

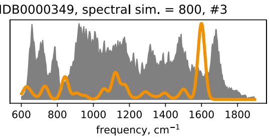

protonated HMDB0000202, spectral sim. = 785, #4

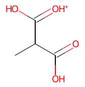

Struc. sim. = 0.64
Methylmalonic acid

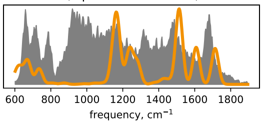

protonated HMDB0130020, spectral sim. = 775, #5

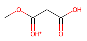

Struc. sim. = 0.60
3-methoxy-3-oxopropanoic acid

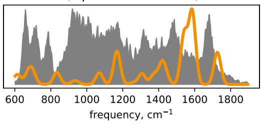

protonated HMDB0000254, spectral sim. = 718, #6

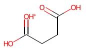

Struc. sim. = 1.00
Succinic acid

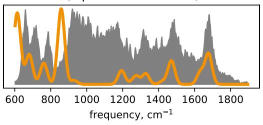

protonated HMDB0031204, spectral sim. = 705, #7

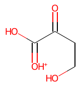

Struc. sim. = 0.45
4-Hydroxy-2-oxobutanoic acid

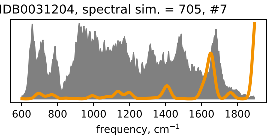

41 deprotonated HMDB0000263

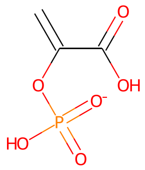

Phosphoenolpyruvic acid

Spectra of deprotonated HMDB0000263, spectral sim. = 817, #1

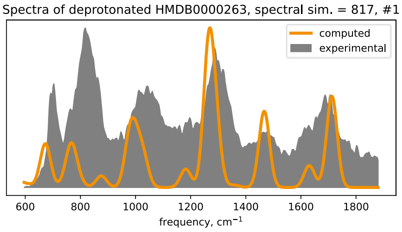


Structural similarity plot of deprotonated HMDB0000263

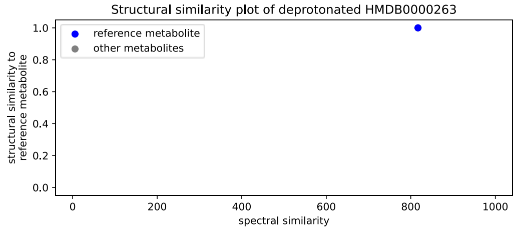


deprotonated HMDB0000263, spectral sim. = 817, #1

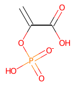

Struc. sim. = 1.00
Phosphoenolpyruvic acid

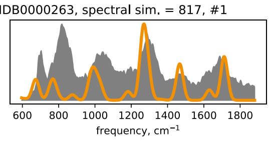

42 deprotonated HMDB0000272

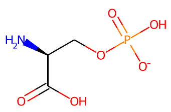

Phosphoserine

Spectra of deprotonated HMDB0000272, spectral sim. = 875, #1

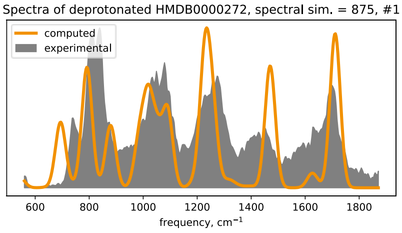


Structural similarity plot of deprotonated HMDB0000272

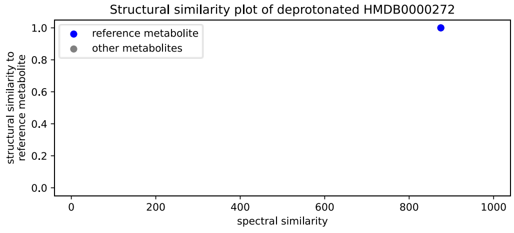


deprotonated HMDB0000272, spectral sim. = 875, #1

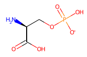

Struc. sim. = 1.00
Phosphoserine

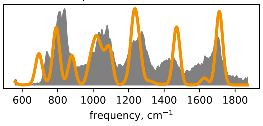

43 protonated HMDB0000272

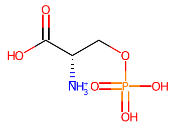

Phosphoserine

Spectra of protonated HMDB0000272, spectral sim. = 869, #1

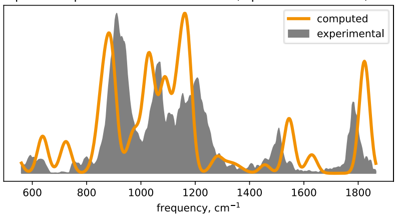


Structural similarity plot of protonated HMDB0000272

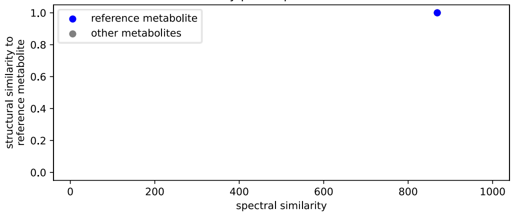


protonated HMDB0000272, spectral sim. = 869, #1

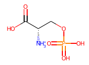

Struc. sim. = 1.00
Phosphoserine

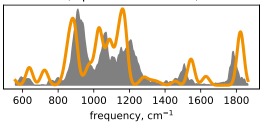


44 sodiated HMDB0000272

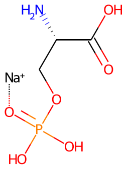

Phosphoserine

Spectra of sodiated HMDB0000272, spectral sim. = 878, #1

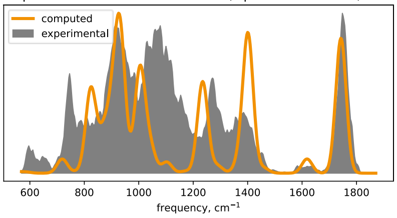


Structural similarity plot of sodiated HMDB0000272

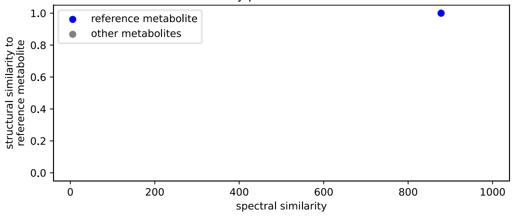


sodiated HMDB0000272, spectral sim. = 878, #1

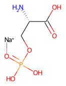

Struc. sim. = 1.00
Phosphoserine

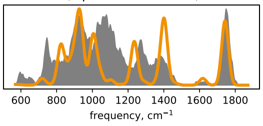


45 deprotonated HMDB0000292

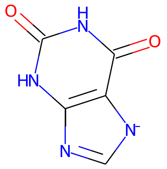

Xanthine

Spectra of deprotonated HMDB0000292, spectral sim. = 782, #2

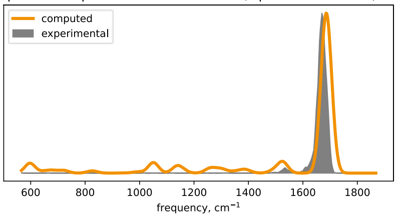


Structural similarity plot of deprotonated HMDB0000292

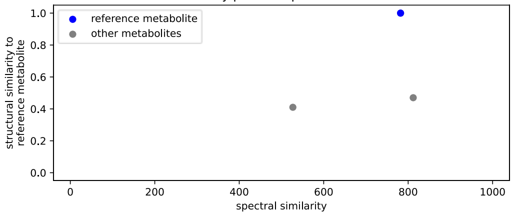


deprotonated HMDB0000786, spectral sim. = 812, #1

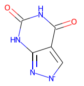

Struc. sim. = 0.47
Oxypurinol

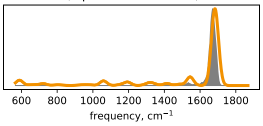


deprotonated HMDB0000292, spectral sim. = 782, #2

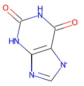

Struc. sim. = 1.00
Xanthine

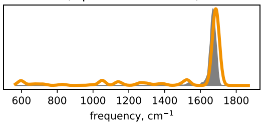


deprotonated HMDB0001182, spectral sim. = 527, #3

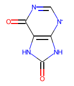

Struc. sim. = 0.41
6,8-Dihydroxypurine

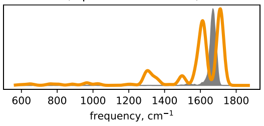


46 protonated HMDB0000292

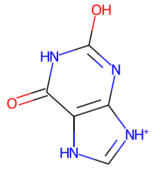

Xanthine

Spectra of protonated HMDB0000292, spectral sim. = 894, #1

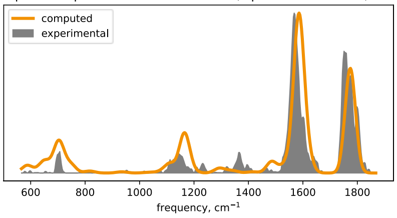


Structural similarity plot of protonated HMDB0000292

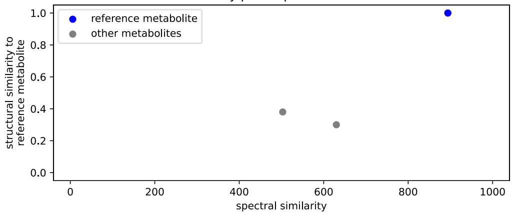


protonated HMDB0000292, spectral sim. = 894, #1

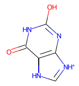

Struc. sim. = 1.00
Xanthine

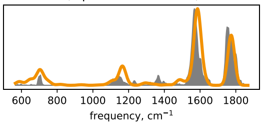


protonated HMDB0000786, spectral sim. = 630, #2

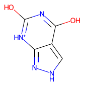

Struc. sim. = 0.30
Oxypurinol

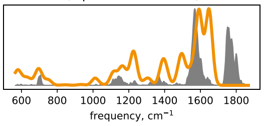


protonated HMDB0001182, spectral sim. = 503, #3

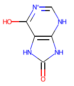

Struc. sim. = 0.38
6,8-Dihydroxypurine

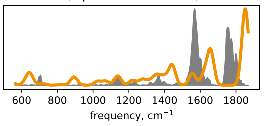

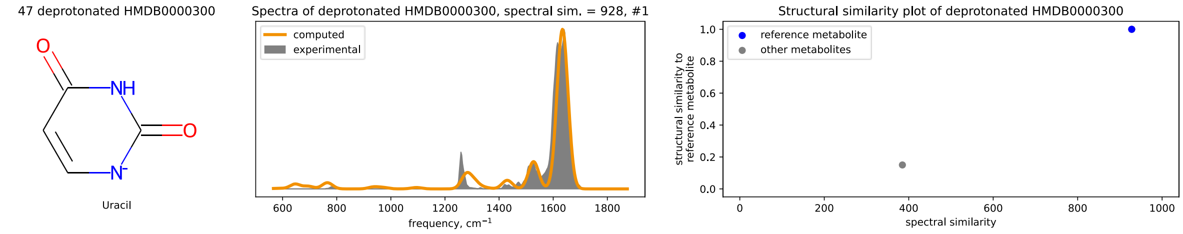
47 deprotonated HMDB0000300
Uracil
Spectra of deprotonated HMDB0000300, spectral sim. = 928, #1
computed
experimental
frequency, cm$^{-1}$
Structural similarity plot of deprotonated HMDB0000300
reference metabolite
other metabolites
structural similarity to reference metabolite
spectral similarity

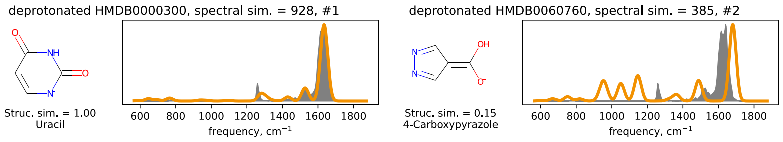
deprotonated HMDB0000300, spectral sim. = 928, #1
Struc. sim. = 1.00
Uracil
deprotonated HMDB0060760, spectral sim. = 385, #2
Struc. sim. = 0.15
4-Carboxypyrazole
frequency, cm$^{-1}$

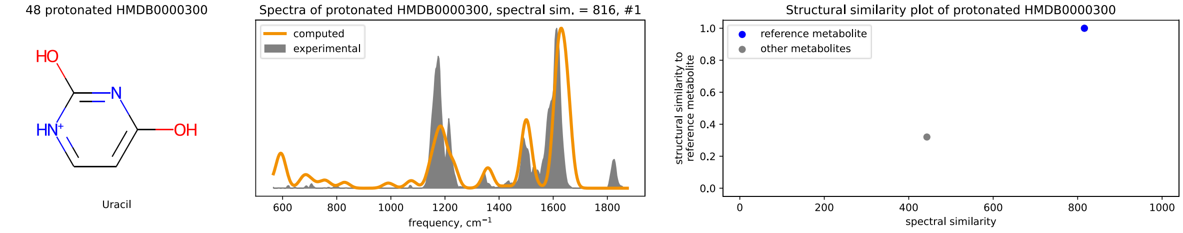
48 protonated HMDB0000300
Uracil
Spectra of protonated HMDB0000300, spectral sim. = 816, #1
computed
experimental
frequency, cm$^{-1}$
Structural similarity plot of protonated HMDB0000300
reference metabolite
other metabolites
structural similarity to reference metabolite
spectral similarity

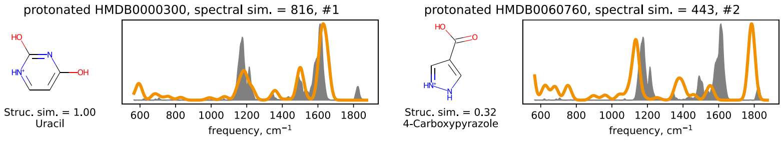
protonated HMDB0000300, spectral sim. = 816, #1
Struc. sim. = 1.00
Uracil
protonated HMDB0060760, spectral sim. = 443, #2
Struc. sim. = 0.32
4-Carboxypyrazole
frequency, cm$^{-1}$

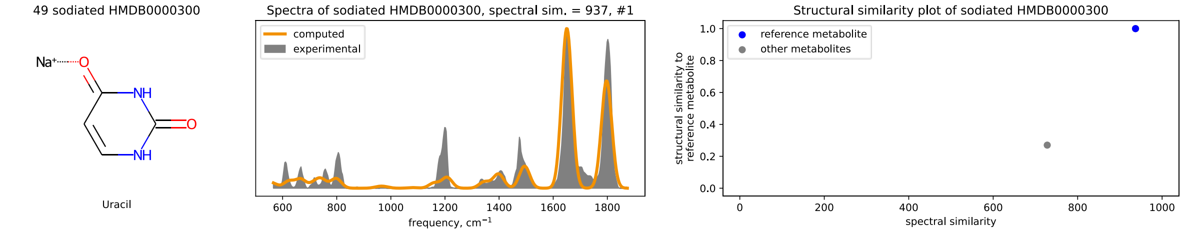
49 sodiated HMDB0000300
Uracil
Spectra of sodiated HMDB0000300, spectral sim. = 937, #1
computed
experimental
frequency, cm$^{-1}$
Structural similarity plot of sodiated HMDB0000300
reference metabolite
other metabolites
structural similarity to reference metabolite
spectral similarity

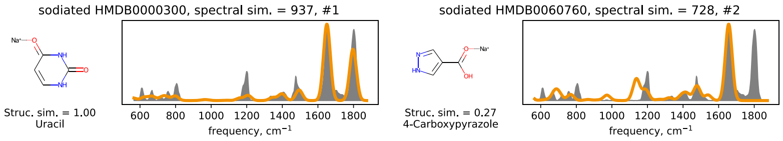
sodiated HMDB0000300, spectral sim. = 937, #1
Struc. sim. = 1.00
Uracil
sodiated HMDB0060760, spectral sim. = 728, #2
Struc. sim. = 0.27
4-Carboxypyrazole
frequency, cm$^{-1}$

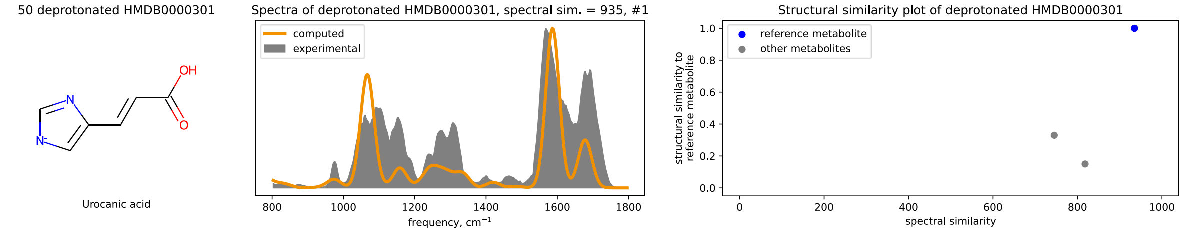
50 deprotonated HMDB0000301
Urocanic acid
Spectra of deprotonated HMDB0000301, spectral sim. = 935, #1
computed
experimental
frequency, cm$^{-1}$
Structural similarity plot of deprotonated HMDB0000301
reference metabolite
other metabolites
structural similarity to reference metabolite
spectral similarity

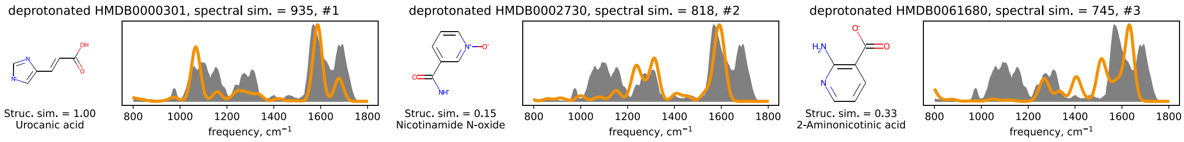
deprotonated HMDB0000301, spectral sim. = 935, #1
Struc. sim. = 1.00
Urocanic acid
deprotonated HMDB0002730, spectral sim. = 818, #2
Struc. sim. = 0.15
Nicotinamide N-oxide
deprotonated HMDB0061680, spectral sim. = 745, #3
Struc. sim. = 0.33
2-Aminonicotinic acid
frequency, cm$^{-1}$

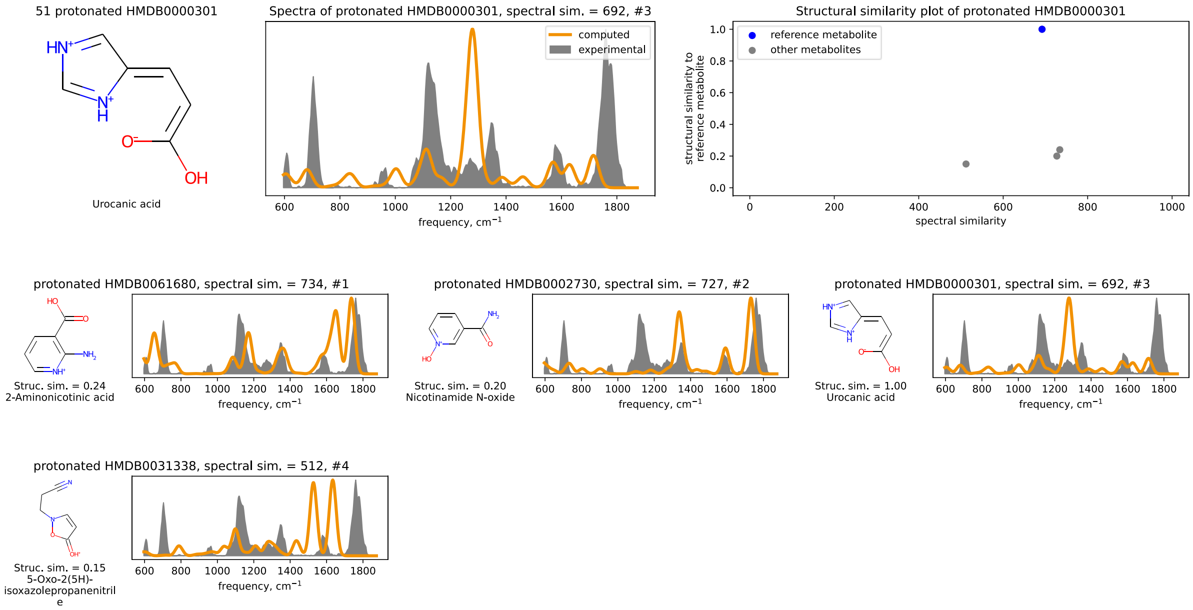
51 protonated HMDB0000301
Urocanic acid
Spectra of protonated HMDB0000301, spectral sim. = 692, #3
computed
experimental
frequency, cm⁻¹
Structural similarity plot of protonated HMDB0000301
reference metabolite
other metabolites
structural similarity to reference metabolite
spectral similarity
protonated HMDB0061680, spectral sim. = 734, #1
Struc. sim. = 0.24
2-Aminonicotinic acid
protonated HMDB0002730, spectral sim. = 727, #2
Struc. sim. = 0.20
Nicotinamide N-oxide
protonated HMDB0000301, spectral sim. = 692, #3
Struc. sim. = 1.00
Urocanic acid
protonated HMDB0031338, spectral sim. = 512, #4
Struc. sim. = 0.15
5-Oxo-2(5H)-
isoxazolepropanenitril
e

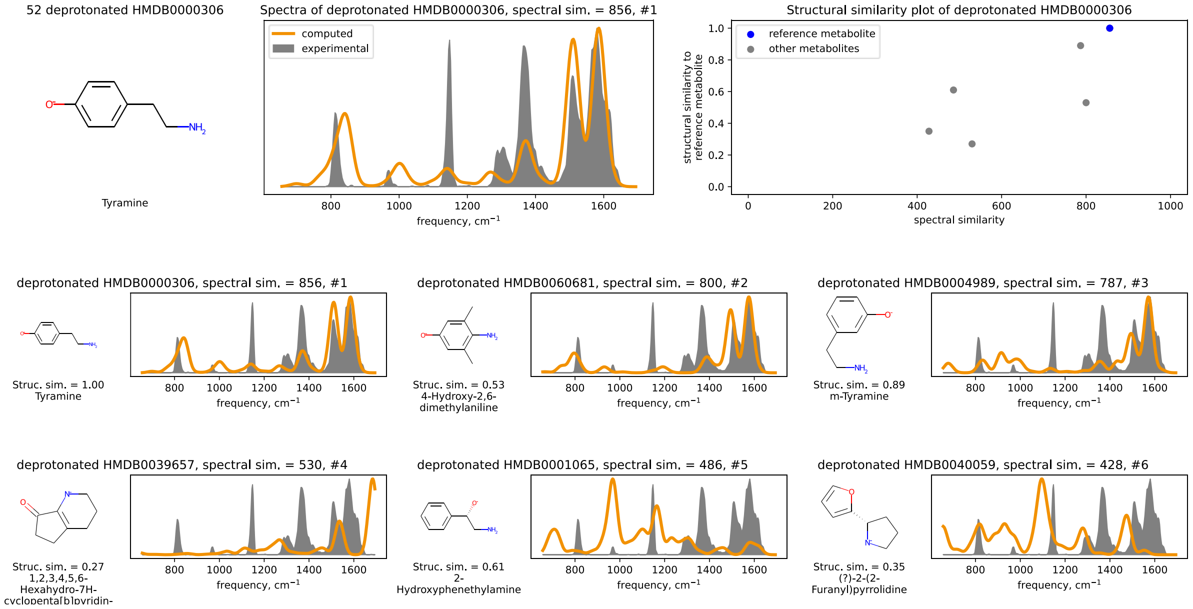
52 deprotonated HMDB0000306
Tyramine
Spectra of deprotonated HMDB0000306, spectral sim. = 856, #1
computed
experimental
frequency, cm⁻¹
Structural similarity plot of deprotonated HMDB0000306
reference metabolite
other metabolites
structural similarity to reference metabolite
spectral similarity
deprotonated HMDB0000306, spectral sim. = 856, #1
Struc. sim. = 1.00
Tyramine
deprotonated HMDB0060681, spectral sim. = 800, #2
Struc. sim. = 0.53
4-Hydroxy-2,6-
dimethylaniline
deprotonated HMDB0004989, spectral sim. = 787, #3
Struc. sim. = 0.89
m-Tyramine
deprotonated HMDB0039657, spectral sim. = 530, #4
Struc. sim. = 0.27
1,2,3,4,5,6-
Hexahydro-7H-
cyclopenta[b]pyridin-
deprotonated HMDB0001065, spectral sim. = 486, #5
Struc. sim. = 0.61
2-
Hydroxyphenethylamine
deprotonated HMDB0040059, spectral sim. = 428, #6
Struc. sim. = 0.35
(?)-2-(2-
Furanyl)pyrrolidine

53 protonated HMDB0000306

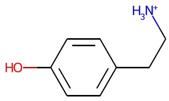

Tyramine

Spectra of protonated HMDB0000306, spectral sim. = 876, #1

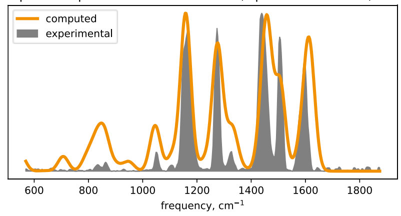


Structural similarity plot of protonated HMDB0000306

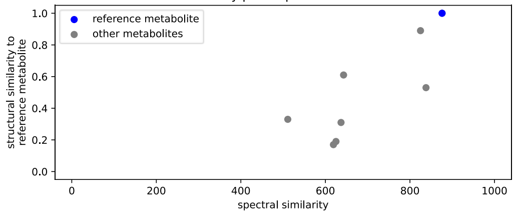


protonated HMDB0000306, spectral sim. = 876, #1

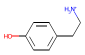

Struc. sim. = 1.00
Tyramine

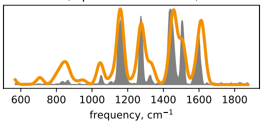


protonated HMDB0060681, spectral sim. = 838, #2

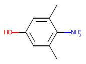

Struc. sim. = 0.53
4-Hydroxy-2,6-dimethylaniline

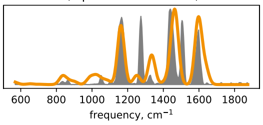


protonated HMDB0004989, spectral sim. = 825, #3

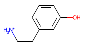

Struc. sim. = 0.89
m-Tyramine

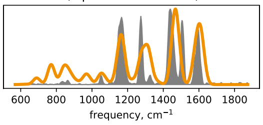


protonated HMDB0001065, spectral sim. = 643, #4

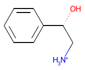

Struc. sim. = 0.61
2-Hydroxyphenethylamine

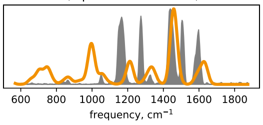


protonated HMDB0040059, spectral sim. = 637, #5

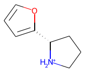

Struc. sim. = 0.31
(?)-2-(2-Furanyl)pyrrolidine

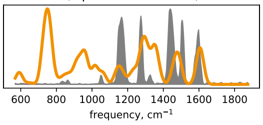


protonated HMDB0001466, spectral sim. = 625, #6

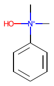

Struc. sim. = 0.19
Dimethylaniline-N-oxide

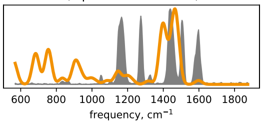


protonated HMDB0029759, spectral sim. = 619, #7

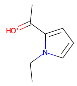

Struc. sim. = 0.17
2-Acetyl-1-ethylpyrrole

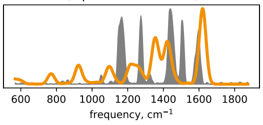


protonated HMDB0039657, spectral sim. = 511, #8

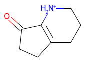

Struc. sim. = 0.33
1,2,3,4,5,6-Hexahydro-7H-cyclopenta[b]pyridin-

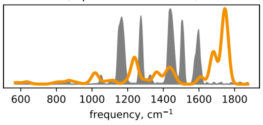


54 sodiated HMDB0000306

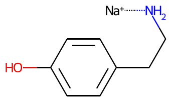

Tyramine

Spectra of sodiated HMDB0000306, spectral sim. = 934, #1

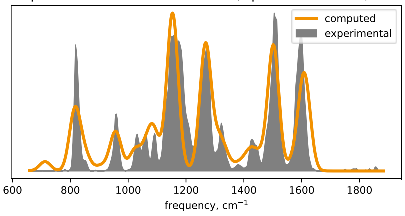


Structural similarity plot of sodiated HMDB0000306

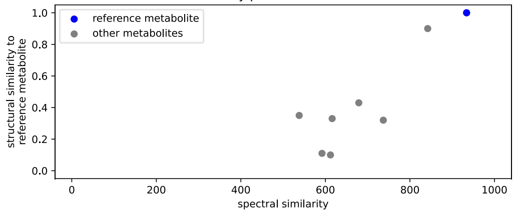


sodiated HMDB0000306, spectral sim. = 934, #1

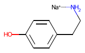

Struc. sim. = 1.00
Tyramine

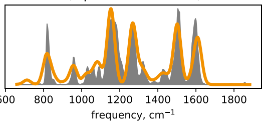


sodiated HMDB0004989, spectral sim. = 842, #2

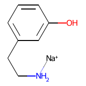

Struc. sim. = 0.90
m-Tyramine

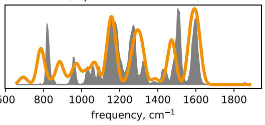


sodiated HMDB0060681, spectral sim. = 737, #3

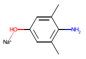

Struc. sim. = 0.32
4-Hydroxy-2,6-dimethylaniline

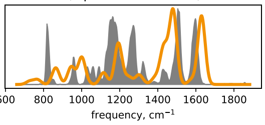


sodiated HMDB0001065, spectral sim. = 679, #4

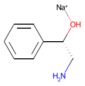

Struc. sim. = 0.43
2-Hydroxyphenethylamine

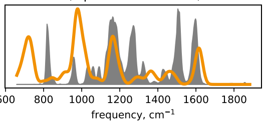


sodiated HMDB0040059, spectral sim. = 616, #5

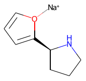

Struc. sim. = 0.33
(?)-2-(2-Furanyl)pyrrolidine

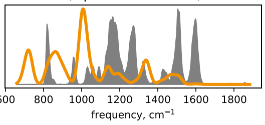


sodiated HMDB0029759, spectral sim. = 612, #6

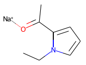

Struc. sim. = 0.10
2-Acetyl-1-ethylpyrrole

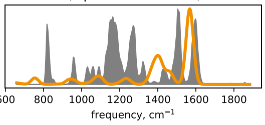


sodiated HMDB0001466, spectral sim. = 592, #7

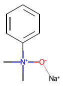

Struc. sim. = 0.11
Dimethylaniline-N-oxide

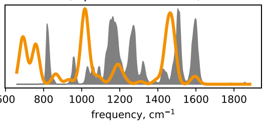


sodiated HMDB0039657, spectral sim. = 538, #8

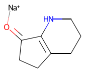

Struc. sim. = 0.35
1,2,3,4,5,6-Hexahydro-7H-cyclopenta[b]pyridin-

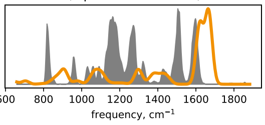

55 protonated HMDB0000323

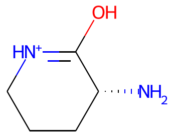


3-Amino-2-piperidone

Spectra of protonated HMDB0000323, spectral sim. = 688, #1

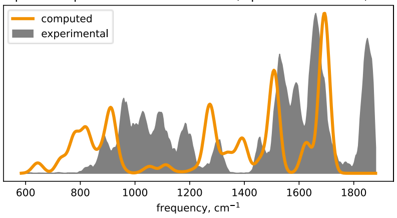


Structural similarity plot of protonated HMDB0000323

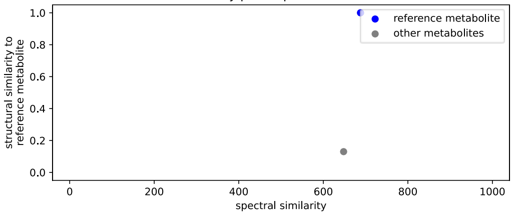


protonated HMDB0000323, spectral sim. = 688, #1

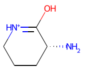

Struc. sim. = 1.00
3-Amino-2-piperidone

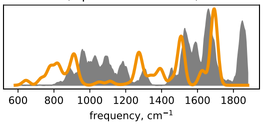


protonated HMDB0061162, spectral sim. = 648, #2

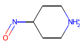

Struc. sim. = 0.13
N-
Mononitrosopiperazine

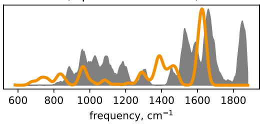

56 deprotonated HMDB0000434

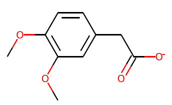

Homoveratric acid

Spectra of deprotonated HMDB0000434, spectral sim. = 970, #1

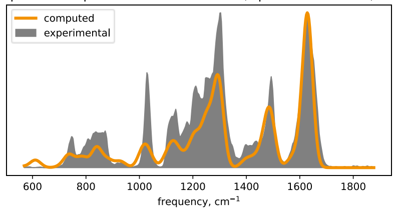


Structural similarity plot of deprotonated HMDB0000434

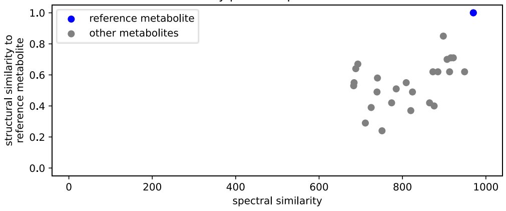


deprotonated HMDB0000434, spectral sim. = 970, #1

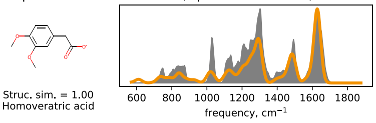

Struc. sim. = 1.00
Homoveratric acid

deprotonated HMDB0130895, spectral sim. = 949, #2

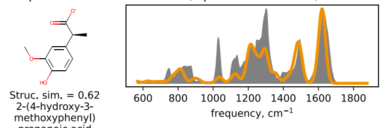

Struc. sim. = 0.62
2-(4-hydroxy-3-methoxyphenyl) propanoic acid

deprotonated HMDB0133490, spectral sim. = 922, #3

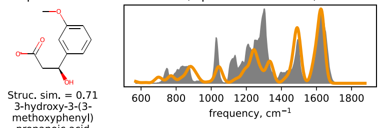

Struc. sim. = 0.71
3-hydroxy-3-(3-methoxyphenyl) propanoic acid

deprotonated HMDB0140293, spectral sim. = 916, #4

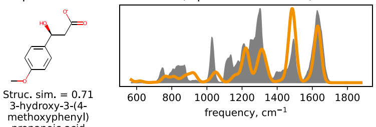

Struc. sim. = 0.71
3-hydroxy-3-(4-methoxyphenyl) propanoic acid

deprotonated HMDB0040256, spectral sim. = 913, #5

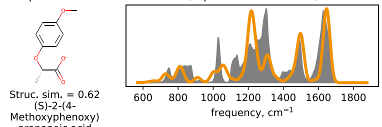

Struc. sim. = 0.62
(S)-2-(4-Methoxyphenoxy) propanoic acid

deprotonated HMDB0062121, spectral sim. = 907, #6

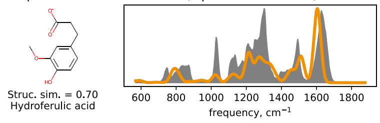

Struc. sim. = 0.70
Hydroferulic acid

deprotonated HMDB0131427, spectral sim. = 898, #7

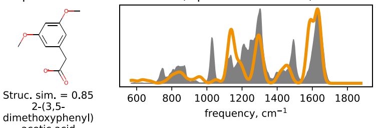

Struc. sim. = 0.85
2-(3,5-dimethoxyphenyl) acetic acid

deprotonated HMDB0039427, spectral sim. = 885, #8

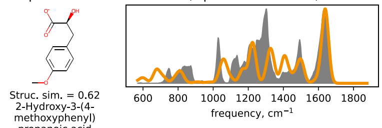

Struc. sim. = 0.62
2-Hydroxy-3-(4-methoxyphenyl) propanoic acid

deprotonated HMDB0035721, spectral sim. = 876, #9

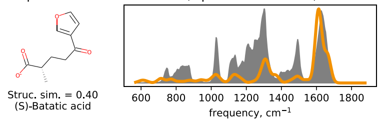

Struc. sim. = 0.40
(S)-Batatic acid

deprotonated HMDB0133530, spectral sim. = 873, #10

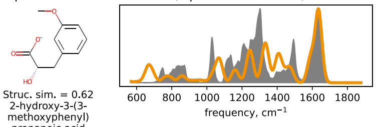

Struc. sim. = 0.62
2-hydroxy-3-(3-methoxyphenyl) propanoic acid

deprotonated HMDB0060733, spectral sim. = 865, #11

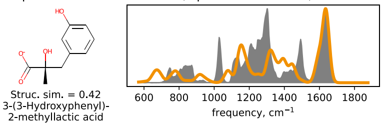

Struc. sim. = 0.42
3-(3-Hydroxyphenyl)-2-methyllactic acid

deprotonated HMDB0135753, spectral sim. = 824, #12

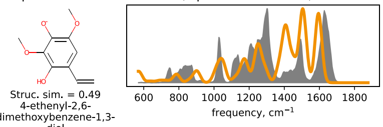

Struc. sim. = 0.49
4-ethenyl-2,6-dimethoxybenzene-1,3-diol

deprotonated HMDB0060398, spectral sim. = 820, #13

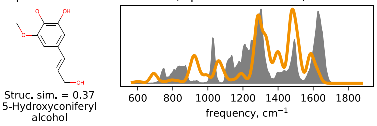

Struc. sim. = 0.37
5-Hydroxyconiferyl alcohol

deprotonated HMDB0133782, spectral sim. = 809, #14

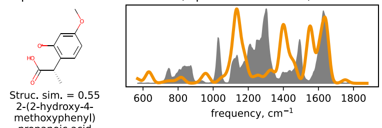

Struc. sim. = 0.55
2-(2-hydroxy-4-methoxyphenyl) propanoic acid

deprotonated HMDB0030571, spectral sim. = 785, #15

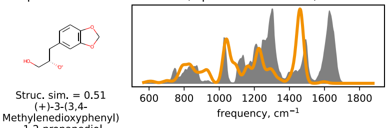

Struc. sim. = 0.51
(+)-3-(3,4-Methylenedioxyphenyl) -1,2-propanediol

deprotonated HMDB0029645, spectral sim. = 774, #16

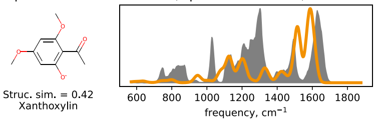

Struc. sim. = 0.42
Xanthoxylin

deprotonated HMDB0037177, spectral sim. = 751, #17

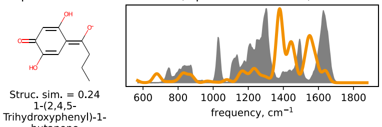

Struc. sim. = 0.24
1-(2,4,5-Trihydroxyphenyl)-1-butanone

deprotonated HMDB0131175, spectral sim. = 740, #18

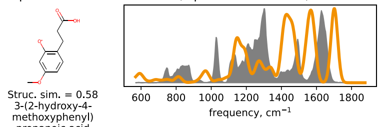

Struc. sim. = 0.58
3-(2-hydroxy-4-methoxyphenyl) propanoic acid

deprotonated HMDB0134100, spectral sim. = 739, #19

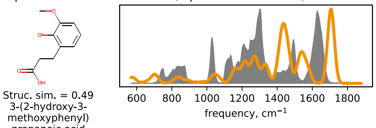

Struc. sim. = 0.49
3-(2-hydroxy-3-methoxyphenyl) propanoic acid

deprotonated HMDB0060735, spectral sim. = 725, #20

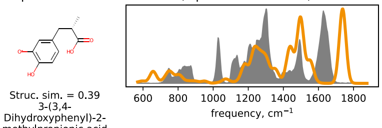

Struc. sim. = 0.39
3-(3,4-Dihydroxyphenyl)-2-methylpropionic acid

deprotonated HMDB0040637, spectral sim. = 711, #21

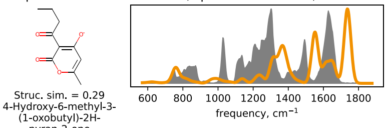

Struc. sim. = 0.29
4-Hydroxy-6-methyl-3-(1-oxobutyl)-2H-pyran-2-one

deprotonated HMDB0131138, spectral sim. = 693, #22

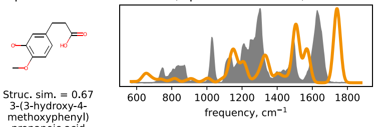

Struc. sim. = 0.67
3-(3-hydroxy-4-methoxyphenyl) propanoic acid

deprotonated HMDB0129339, spectral sim. = 688, #23

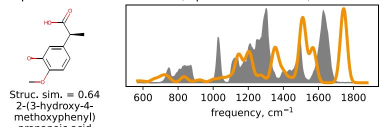

Struc. sim. = 0.64
2-(3-hydroxy-4-methoxyphenyl) propanoic acid

deprotonated HMDB0140897, spectral sim. = 684, #24

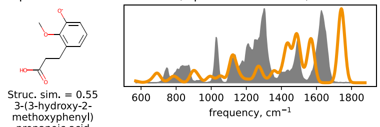

Struc. sim. = 0.55
3-(3-hydroxy-2-methoxyphenyl) propanoic acid

deprotonated HMDB0125530, spectral sim. = 683, #25

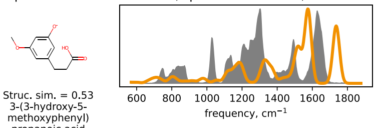

Struc. sim. = 0.53
3-(3-hydroxy-5-methoxyphenyl) propanoic acid

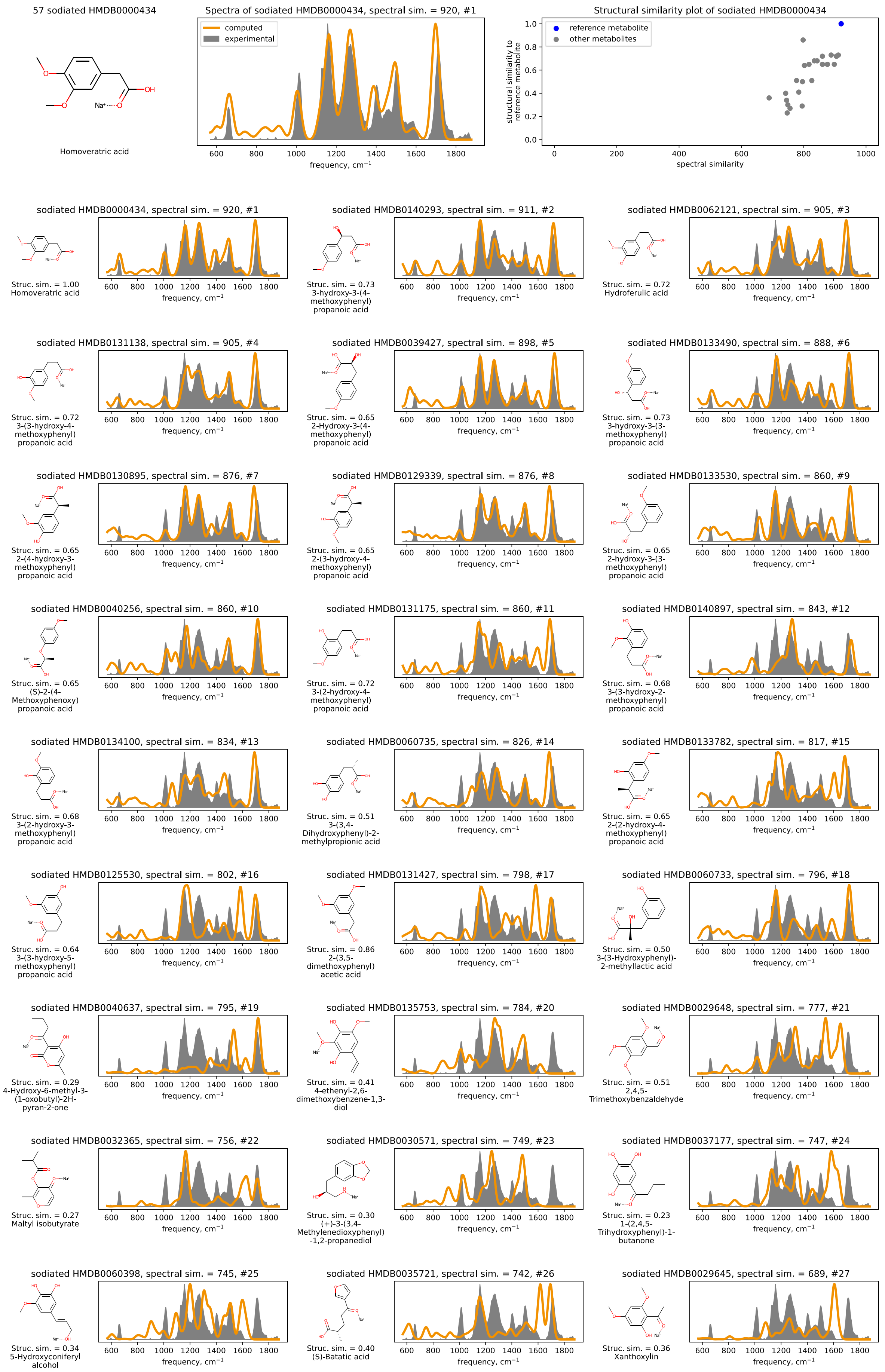
57 sodiated HMDB0000434
Homoveratric acid
Spectra of sodiated HMDB0000434, spectral sim. = 920, #1
computed
experimental
frequency, cm$^{-1}$
Structural similarity plot of sodiated HMDB0000434
reference metabolite
other metabolites
structural similarity to reference metabolite
spectral similarity
sodiated HMDB0000434, spectral sim. = 920, #1
Struc. sim. = 1.00
Homoveratric acid
sodiated HMDB0140293, spectral sim. = 911, #2
Struc. sim. = 0.73
3-hydroxy-3-(4-methoxyphenyl) propanoic acid
sodiated HMDB0062121, spectral sim. = 905, #3
Struc. sim. = 0.72
Hydroferulic acid
sodiated HMDB0131138, spectral sim. = 905, #4
Struc. sim. = 0.72
3-(3-hydroxy-4-methoxyphenyl) propanoic acid
sodiated HMDB0039427, spectral sim. = 898, #5
Struc. sim. = 0.65
2-Hydroxy-3-(4-methoxyphenyl) propanoic acid
sodiated HMDB0133490, spectral sim. = 888, #6
Struc. sim. = 0.73
3-hydroxy-3-(3-methoxyphenyl) propanoic acid
sodiated HMDB0130895, spectral sim. = 876, #7
Struc. sim. = 0.65
2-(4-hydroxy-3-methoxyphenyl) propanoic acid
sodiated HMDB0129339, spectral sim. = 876, #8
Struc. sim. = 0.65
2-(3-hydroxy-4-methoxyphenyl) propanoic acid
sodiated HMDB0133530, spectral sim. = 860, #9
Struc. sim. = 0.65
2-hydroxy-3-(3-methoxyphenyl) propanoic acid
sodiated HMDB0040256, spectral sim. = 860, #10
Struc. sim. = 0.65
(S)-2-(4-Methoxyphenoxy) propanoic acid
sodiated HMDB0131175, spectral sim. = 860, #11
Struc. sim. = 0.72
3-(2-hydroxy-4-methoxyphenyl) propanoic acid
sodiated HMDB0140897, spectral sim. = 843, #12
Struc. sim. = 0.68
3-(3-hydroxy-2-methoxyphenyl) propanoic acid
sodiated HMDB0134100, spectral sim. = 834, #13
Struc. sim. = 0.68
3-(2-hydroxy-3-methoxyphenyl) propanoic acid
sodiated HMDB0060735, spectral sim. = 826, #14
Struc. sim. = 0.51
3-(3,4-Dihydroxyphenyl)-2-methylpropionic acid
sodiated HMDB0133782, spectral sim. = 817, #15
Struc. sim. = 0.65
2-(2-hydroxy-4-methoxyphenyl) propanoic acid
sodiated HMDB0125530, spectral sim. = 802, #16
Struc. sim. = 0.64
3-(3-hydroxy-5-methoxyphenyl) propanoic acid
sodiated HMDB0131427, spectral sim. = 798, #17
Struc. sim. = 0.86
2-(3,5-dimethoxyphenyl) acetic acid
sodiated HMDB0060733, spectral sim. = 796, #18
Struc. sim. = 0.50
3-(3-Hydroxyphenyl)-2-methyllactic acid
sodiated HMDB0040637, spectral sim. = 795, #19
Struc. sim. = 0.29
4-Hydroxy-6-methyl-3-(1-oxobutyl)-2H-pyran-2-one
sodiated HMDB0135753, spectral sim. = 784, #20
Struc. sim. = 0.41
4-ethenyl-2,6-dimethoxybenzene-1,3-diol
sodiated HMDB0029648, spectral sim. = 777, #21
Struc. sim. = 0.51
2,4,5-Trimethoxybenzaldehyde
sodiated HMDB0032365, spectral sim. = 756, #22
Struc. sim. = 0.27
Maltyl isobutyrate
sodiated HMDB0030571, spectral sim. = 749, #23
Struc. sim. = 0.30
(+)-3-(3,4-Methylenedioxyphenyl) -1,2-propanediol
sodiated HMDB0037177, spectral sim. = 747, #24
Struc. sim. = 0.23
1-(2,4,5-Trihydroxyphenyl)-1-butanone
sodiated HMDB0060398, spectral sim. = 745, #25
Struc. sim. = 0.34
5-Hydroxyconiferyl alcohol
sodiated HMDB0035721, spectral sim. = 742, #26
Struc. sim. = 0.40
(S)-Batatic acid
sodiated HMDB0029645, spectral sim. = 689, #27
Struc. sim. = 0.36
Xanthoxylin

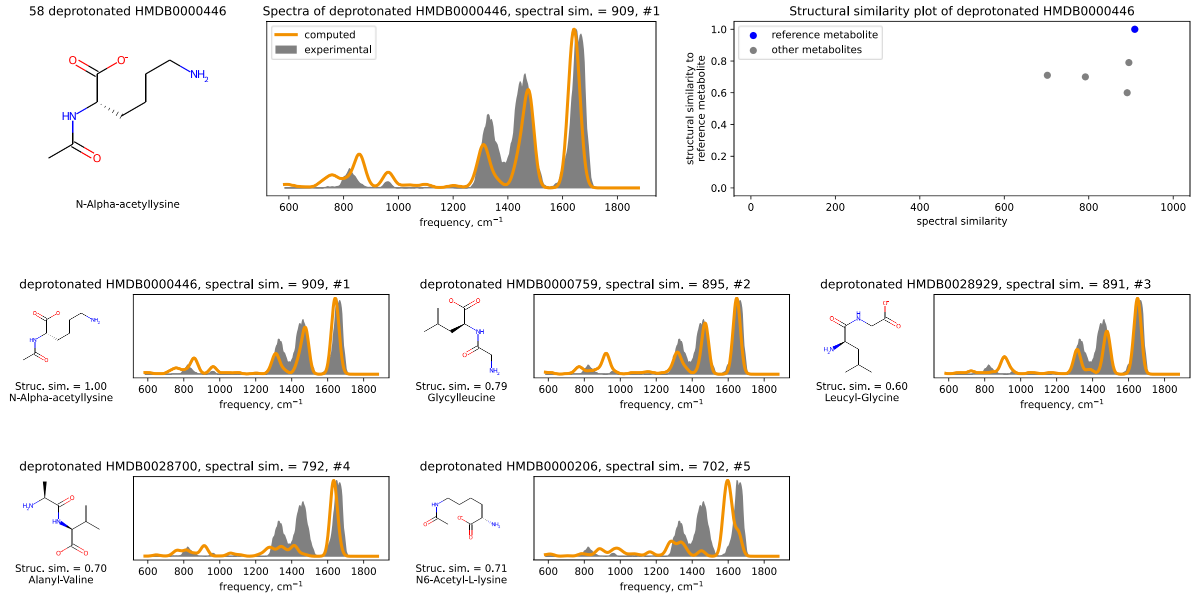
58 deprotonated HMDB0000446
N-Alpha-acetyllysine
Spectra of deprotonated HMDB0000446, spectral sim. = 909, #1
computed
experimental
frequency, cm−1
Structural similarity plot of deprotonated HMDB0000446
reference metabolite
other metabolites
structural similarity to reference metabolite
spectral similarity
deprotonated HMDB0000446, spectral sim. = 909, #1
Struc. sim. = 1.00
N-Alpha-acetyllysine
deprotonated HMDB0000759, spectral sim. = 895, #2
Struc. sim. = 0.79
Glycylleucine
deprotonated HMDB0028929, spectral sim. = 891, #3
Struc. sim. = 0.60
Leucyl-Glycine
deprotonated HMDB0028700, spectral sim. = 792, #4
Struc. sim. = 0.70
Alanyl-Valine
deprotonated HMDB0000206, spectral sim. = 702, #5
Struc. sim. = 0.71
N6-Acetyl-L-lysine

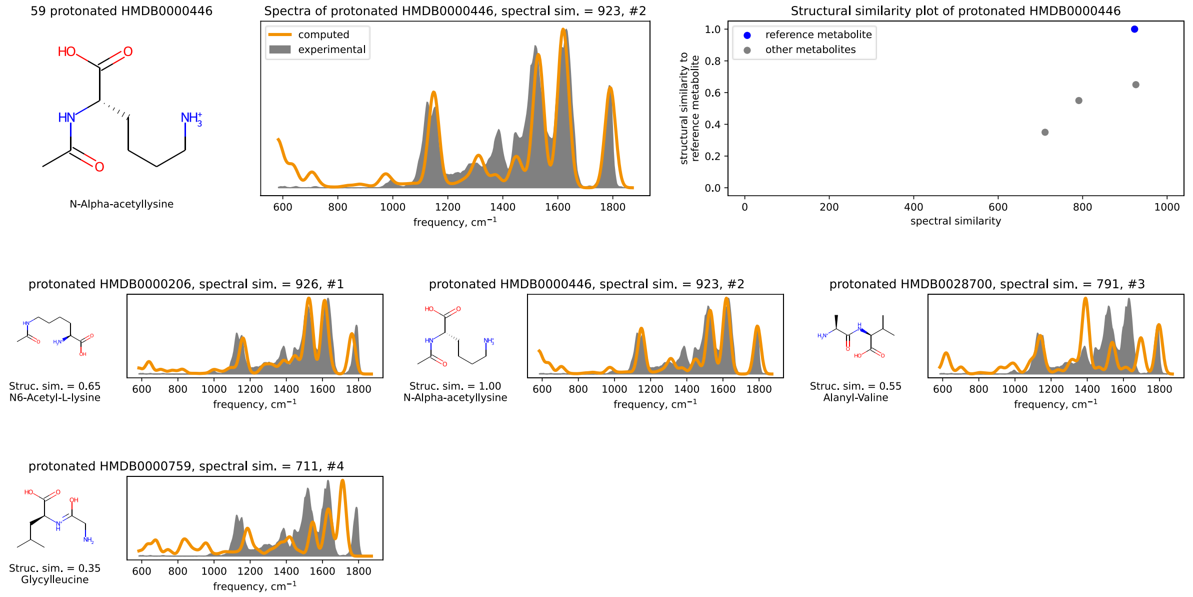
59 protonated HMDB0000446
N-Alpha-acetyllysine
Spectra of protonated HMDB0000446, spectral sim. = 923, #2
computed
experimental
frequency, cm−1
Structural similarity plot of protonated HMDB0000446
reference metabolite
other metabolites
structural similarity to reference metabolite
spectral similarity
protonated HMDB0000206, spectral sim. = 926, #1
Struc. sim. = 0.65
N6-Acetyl-L-lysine
protonated HMDB0000446, spectral sim. = 923, #2
Struc. sim. = 1.00
N-Alpha-acetyllysine
protonated HMDB0028700, spectral sim. = 791, #3
Struc. sim. = 0.55
Alanyl-Valine
protonated HMDB0000759, spectral sim. = 711, #4
Struc. sim. = 0.35
Glycylleucine

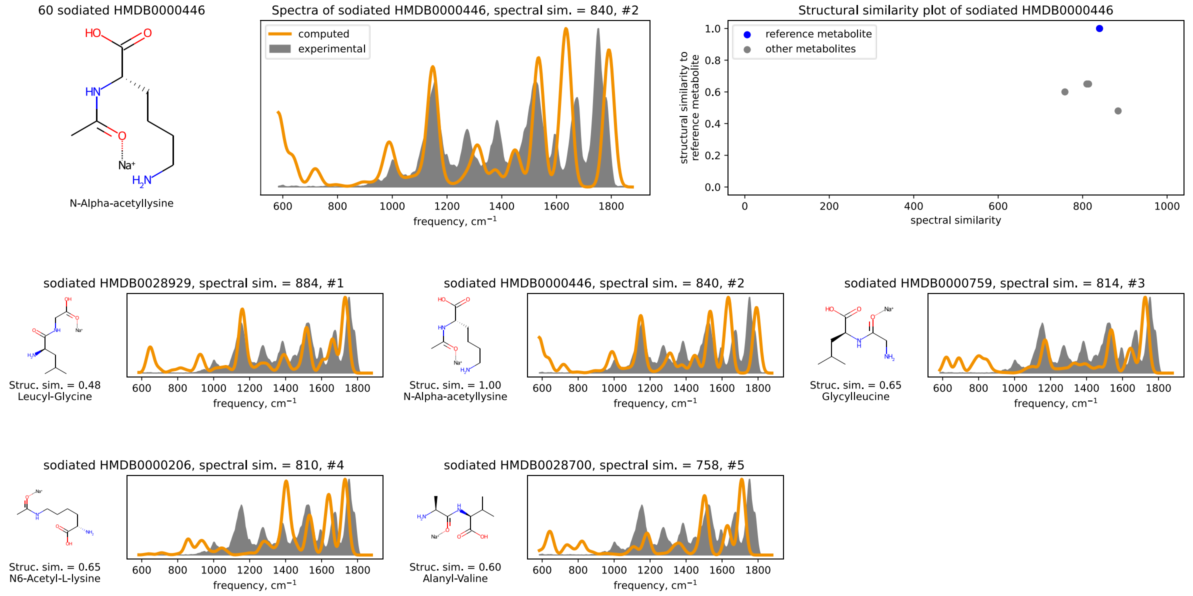
60 sodiated HMDB0000446
N-Alpha-acetyllysine
Spectra of sodiated HMDB0000446, spectral sim. = 840, #2
computed
experimental
frequency, cm−1
Structural similarity plot of sodiated HMDB0000446
reference metabolite
other metabolites
structural similarity to reference metabolite
spectral similarity
sodiated HMDB0028929, spectral sim. = 884, #1
Struc. sim. = 0.48
Leucyl-Glycine
sodiated HMDB0000446, spectral sim. = 840, #2
Struc. sim. = 1.00
N-Alpha-acetyllysine
sodiated HMDB0000759, spectral sim. = 814, #3
Struc. sim. = 0.65
Glycylleucine
sodiated HMDB0000206, spectral sim. = 810, #4
Struc. sim. = 0.65
N6-Acetyl-L-lysine
sodiated HMDB0028700, spectral sim. = 758, #5
Struc. sim. = 0.60
Alanyl-Valine

61 deprotonated HMDB0000448

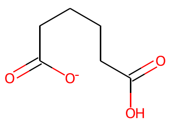


Adipic acid

Spectra of deprotonated HMDB0000448, spectral sim. = 847, #2

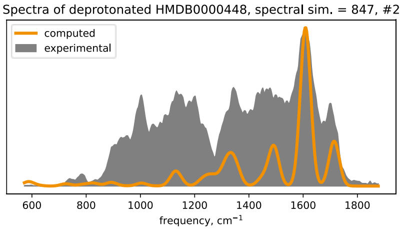


Structural similarity plot of deprotonated HMDB0000448

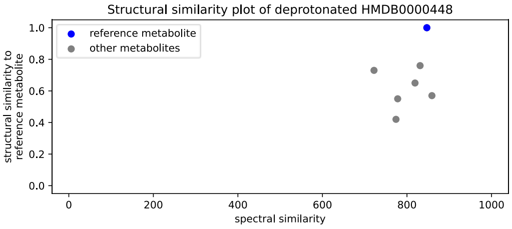


deprotonated HMDB0061930, spectral sim. = 859, #1

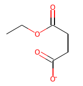

Struc. sim. = 0.57
4-Ethoxy-4-oxobutanoic acid

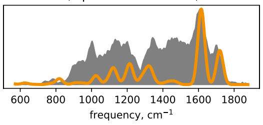


deprotonated HMDB0000448, spectral sim. = 847, #2

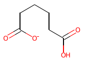

Struc. sim. = 1.00
Adipic acid

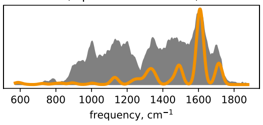


deprotonated HMDB0000422, spectral sim. = 831, #3

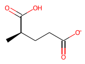

Struc. sim. = 0.76
2-Methylglutaric acid

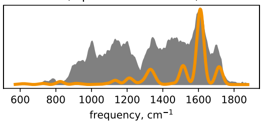


deprotonated HMDB0000858, spectral sim. = 819, #4

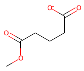

Struc. sim. = 0.65
Monomethyl glutaric acid

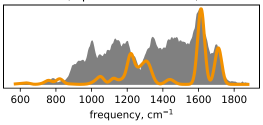


deprotonated HMDB0002074, spectral sim. = 778, #5

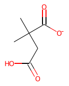

Struc. sim. = 0.55
2,2-Dimethylsuccinic acid

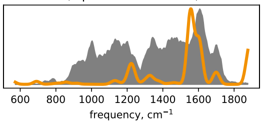


deprotonated HMDB0006900, spectral sim. = 774, #6

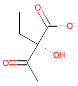

Struc. sim. = 0.42
(S)-2-Aceto-2-hydroxybutanoic acid

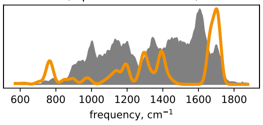


deprotonated HMDB0000752, spectral sim. = 722, #7

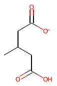

Struc. sim. = 0.73
Methylglutaric acid

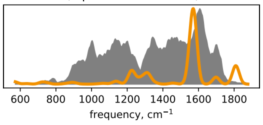


62 sodiated HMDB0000448

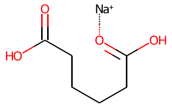


Adipic acid

Spectra of sodiated HMDB0000448, spectral sim. = 875, #2

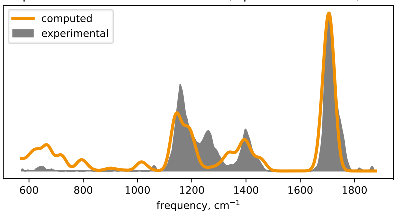


Structural similarity plot of sodiated HMDB0000448

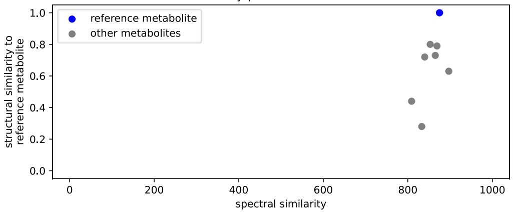


sodiated HMDB0061930, spectral sim. = 897, #1

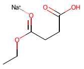

Struc. sim. = 0.63
4-Ethoxy-4-oxobutanoic acid

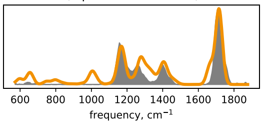


sodiated HMDB0000448, spectral sim. = 875, #2

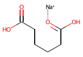

Struc. sim. = 1.00
Adipic acid

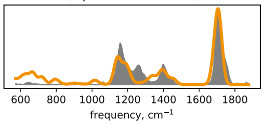


sodiated HMDB0000752, spectral sim. = 869, #3

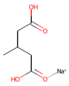

Struc. sim. = 0.79
Methylglutaric acid

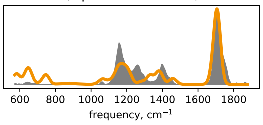


sodiated HMDB0002074, spectral sim. = 865, #4

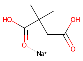

Struc. sim. = 0.73
2,2-Dimethylsuccinic acid

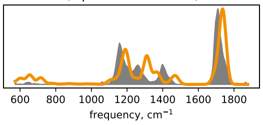


sodiated HMDB0000422, spectral sim. = 853, #5

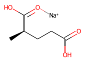

Struc. sim. = 0.80
2-Methylglutaric acid

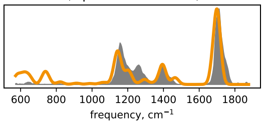


sodiated HMDB0000858, spectral sim. = 840, #6

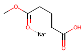

Struc. sim. = 0.72
Monomethyl glutaric acid

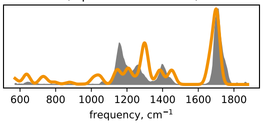


sodiated HMDB0033837, spectral sim. = 833, #7

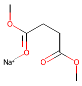

Struc. sim. = 0.28
Dimethyl succinate

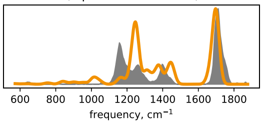


sodiated HMDB0006900, spectral sim. = 809, #8

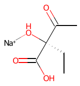

Struc. sim. = 0.44
(S)-2-Aceto-2-hydroxybutanoic acid

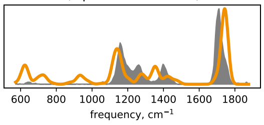

63 deprotonated HMDB0000500

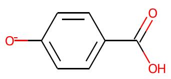

4-Hydroxybenzoic acid

Spectra of deprotonated HMDB0000500, spectral sim. = 947, #1

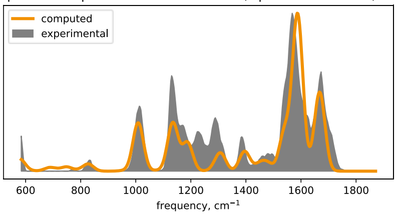


Structural similarity plot of deprotonated HMDB0000500

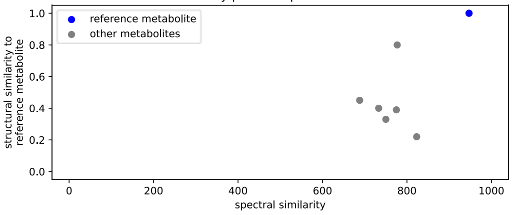


deprotonated HMDB0000500, spectral sim. = 947, #1

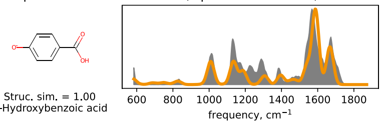

Struc. sim. = 1.00
4-Hydroxybenzoic acid

deprotonated HMDB0062484, spectral sim. = 823, #2

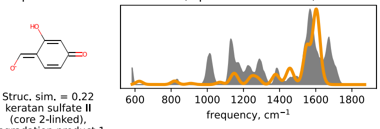

Struc. sim. = 0.22
keratan sulfate II (core 2-linked), degradation product 1

deprotonated HMDB0001895, spectral sim. = 777, #3

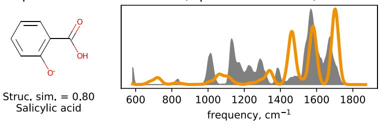

Struc. sim. = 0.80
Salicylic acid

deprotonated HMDB0002466, spectral sim. = 775, #4

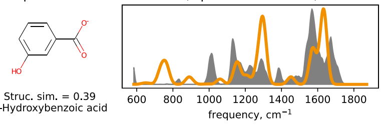

Struc. sim. = 0.39
3-Hydroxybenzoic acid

deprotonated HMDB0059965, spectral sim. = 750, #5

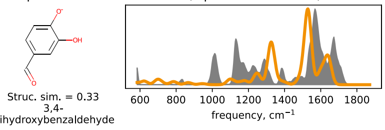

Struc. sim. = 0.33
3,4-Dihydroxybenzaldehyde

deprotonated HMDB0004062, spectral sim. = 733, #6

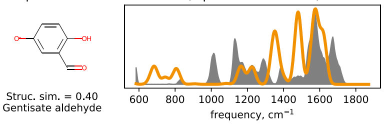

Struc. sim. = 0.40
Gentisate aldehyde

deprotonated HMDB0033812, spectral sim. = 688, #7

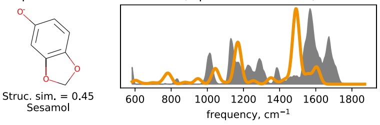

Struc. sim. = 0.45
Sesamol

64 protonated HMDB0000500

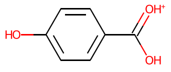

4-Hydroxybenzoic acid

Spectra of protonated HMDB0000500, spectral sim. = 957, #1

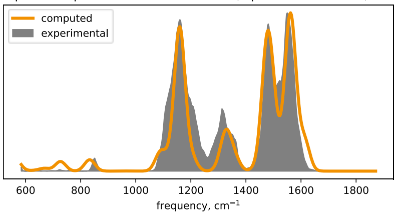


Structural similarity plot of protonated HMDB0000500

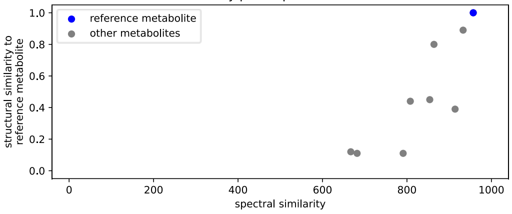


protonated HMDB0000500, spectral sim. = 957, #1

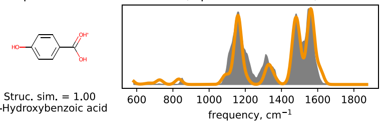

Struc. sim. = 1.00
4-Hydroxybenzoic acid

protonated HMDB0002466, spectral sim. = 933, #2

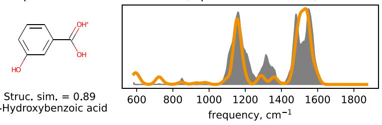

Struc. sim. = 0.89
3-Hydroxybenzoic acid

protonated HMDB0059965, spectral sim. = 914, #3

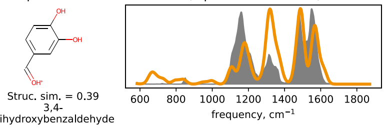

Struc. sim. = 0.39
3,4-Dihydroxybenzaldehyde

protonated HMDB0001895, spectral sim. = 864, #4

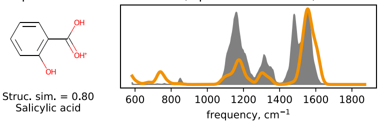

Struc. sim. = 0.80
Salicylic acid

protonated HMDB0004062, spectral sim. = 854, #5

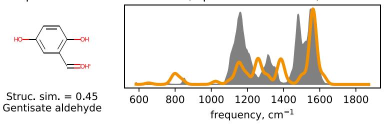

Struc. sim. = 0.45
Gentisate aldehyde

protonated HMDB0062484, spectral sim. = 808, #6

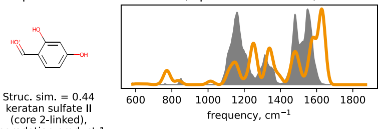

Struc. sim. = 0.44
keratan sulfate II (core 2-linked), degradation product 1

protonated HMDB0032576, spectral sim. = 791, #7

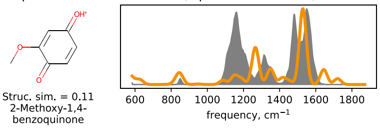

Struc. sim. = 0.11
2-Methoxy-1,4-benzoquinone

protonated HMDB0032920, spectral sim. = 682, #8

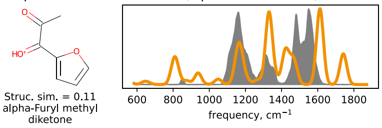

Struc. sim. = 0.11
alpha-Furyl methyl diketone

protonated HMDB0033812, spectral sim. = 667, #9

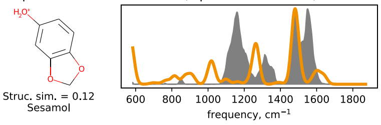

Struc. sim. = 0.12
Sesamol

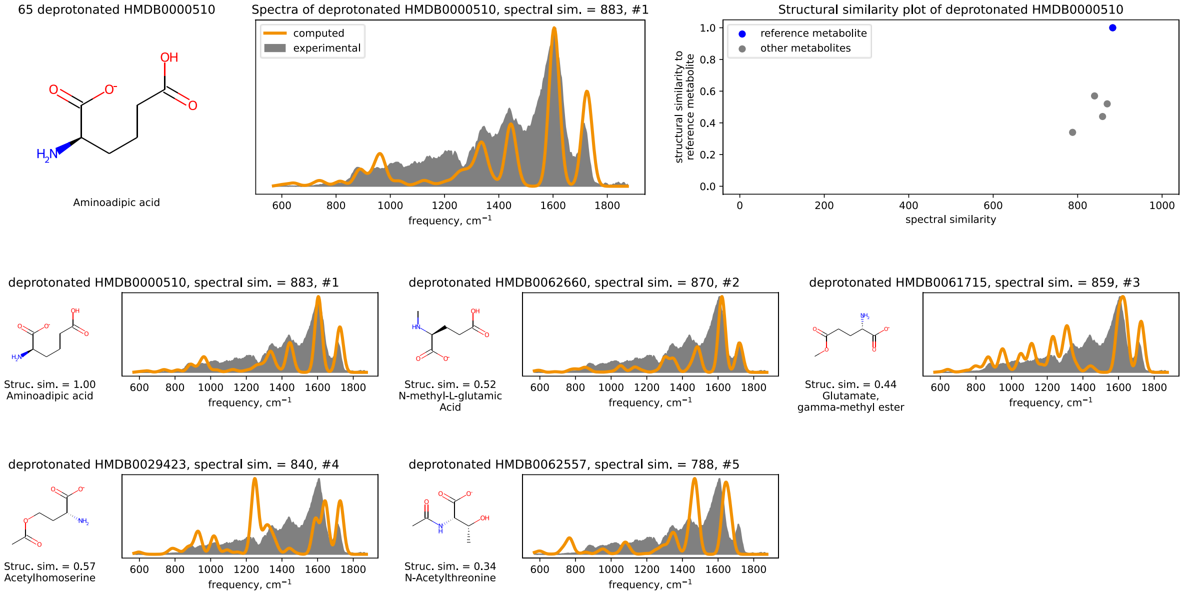
65 deprotonated HMDB0000510
Aminoadipic acid
Spectra of deprotonated HMDB0000510, spectral sim. = 883, #1
computed
experimental
frequency, cm⁻¹
Structural similarity plot of deprotonated HMDB0000510
reference metabolite
other metabolites
structural similarity to reference metabolite
spectral similarity
deprotonated HMDB0000510, spectral sim. = 883, #1
Struc. sim. = 1.00
Aminoadipic acid
deprotonated HMDB0062660, spectral sim. = 870, #2
Struc. sim. = 0.52
N-methyl-L-glutamic Acid
deprotonated HMDB0061715, spectral sim. = 859, #3
Struc. sim. = 0.44
Glutamate, gamma-methyl ester
deprotonated HMDB0029423, spectral sim. = 840, #4
Struc. sim. = 0.57
Acetylhomoserine
deprotonated HMDB0062557, spectral sim. = 788, #5
Struc. sim. = 0.34
N-Acetylthreonine

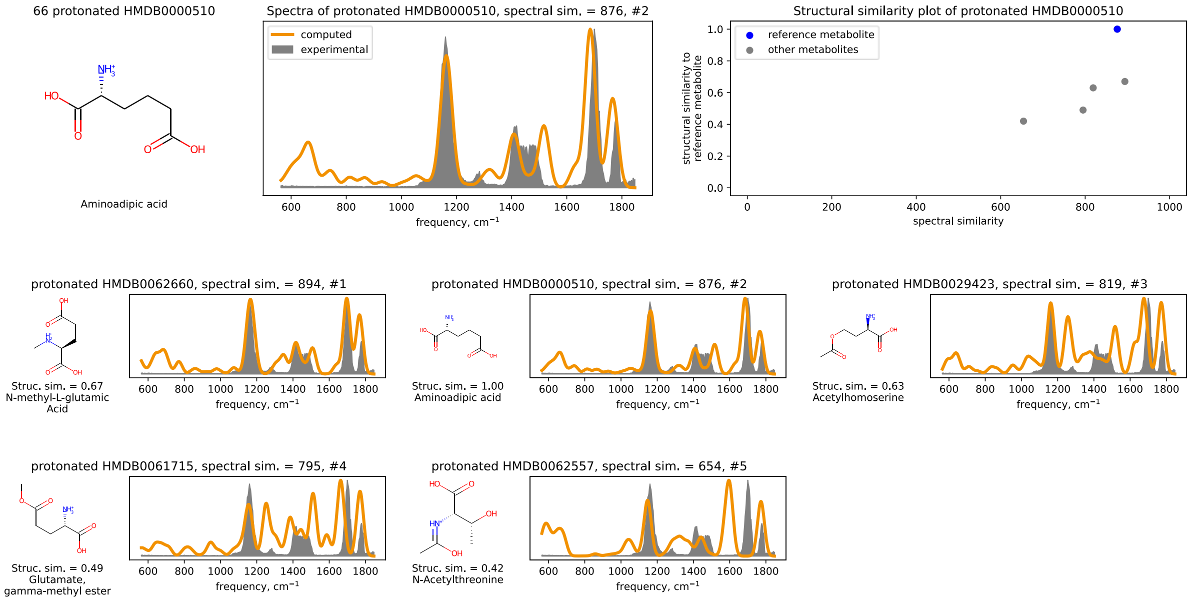
66 protonated HMDB0000510
Aminoadipic acid
Spectra of protonated HMDB0000510, spectral sim. = 876, #2
computed
experimental
frequency, cm⁻¹
Structural similarity plot of protonated HMDB0000510
reference metabolite
other metabolites
structural similarity to reference metabolite
spectral similarity
protonated HMDB0062660, spectral sim. = 894, #1
Struc. sim. = 0.67
N-methyl-L-glutamic Acid
protonated HMDB0000510, spectral sim. = 876, #2
Struc. sim. = 1.00
Aminoadipic acid
protonated HMDB0029423, spectral sim. = 819, #3
Struc. sim. = 0.63
Acetylhomoserine
protonated HMDB0061715, spectral sim. = 795, #4
Struc. sim. = 0.49
Glutamate, gamma-methyl ester
protonated HMDB0062557, spectral sim. = 654, #5
Struc. sim. = 0.42
N-Acetylthreonine

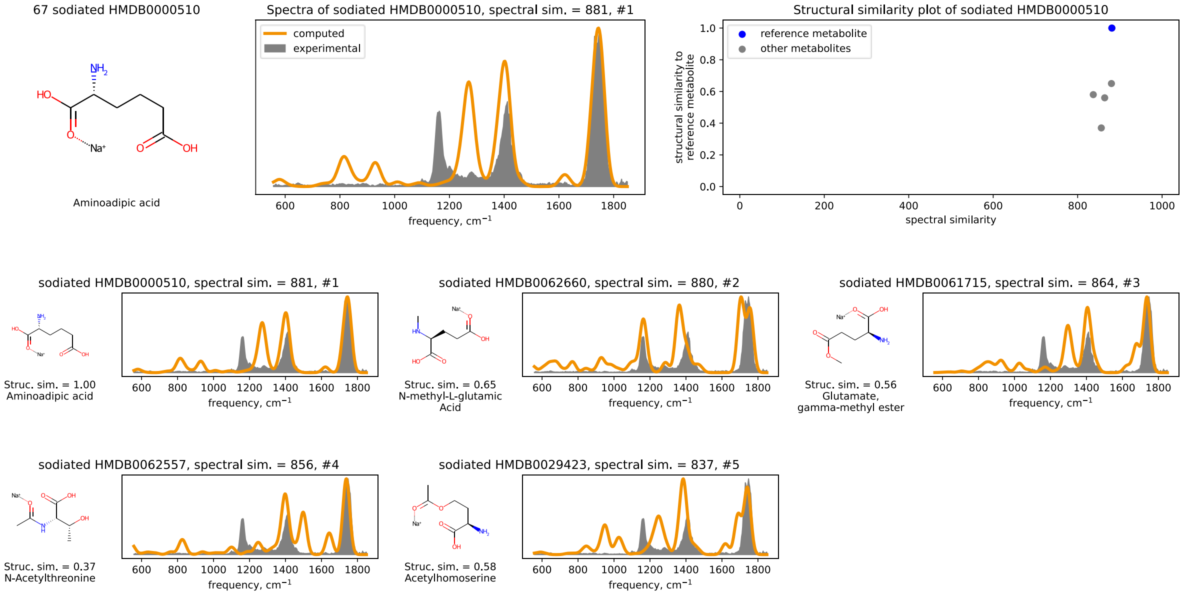
67 sodiated HMDB0000510
Aminoadipic acid
Spectra of sodiated HMDB0000510, spectral sim. = 881, #1
computed
experimental
frequency, cm⁻¹
Structural similarity plot of sodiated HMDB0000510
reference metabolite
other metabolites
structural similarity to reference metabolite
spectral similarity
sodiated HMDB0000510, spectral sim. = 881, #1
Struc. sim. = 1.00
Aminoadipic acid
sodiated HMDB0062660, spectral sim. = 880, #2
Struc. sim. = 0.65
N-methyl-L-glutamic Acid
sodiated HMDB0061715, spectral sim. = 864, #3
Struc. sim. = 0.56
Glutamate, gamma-methyl ester
sodiated HMDB0062557, spectral sim. = 856, #4
Struc. sim. = 0.37
N-Acetylthreonine
sodiated HMDB0029423, spectral sim. = 837, #5
Struc. sim. = 0.58
Acetylhomoserine

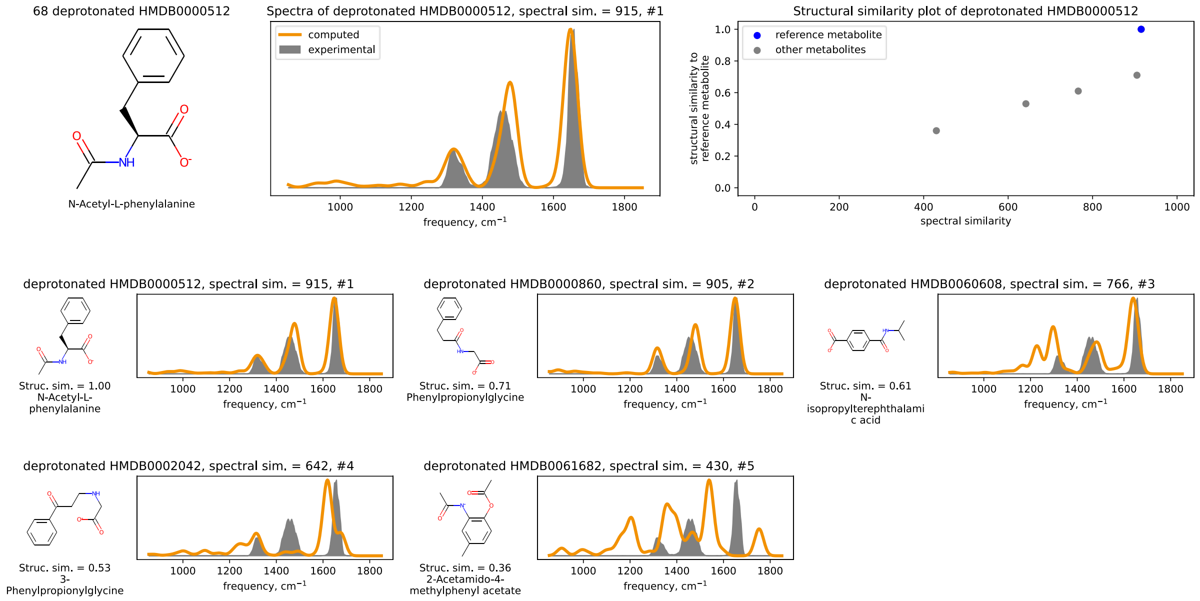

68 deprotonated HMDB0000512
N-Acetyl-L-phenylalanine
Spectra of deprotonated HMDB0000512, spectral sim. = 915, #1
computed
experimental
frequency, cm$^{-1}$
Structural similarity plot of deprotonated HMDB0000512
reference metabolite
other metabolites
structural similarity to reference metabolite
spectral similarity
deprotonated HMDB0000512, spectral sim. = 915, #1
Struc. sim. = 1.00
N-Acetyl-L-phenylalanine
deprotonated HMDB0000860, spectral sim. = 905, #2
Struc. sim. = 0.71
Phenylpropionylglycine
deprotonated HMDB0060608, spectral sim. = 766, #3
Struc. sim. = 0.61
N-isopropylterephthalamic acid
deprotonated HMDB0002042, spectral sim. = 642, #4
Struc. sim. = 0.53
3-Phenylpropionylglycine
deprotonated HMDB0061682, spectral sim. = 430, #5
Struc. sim. = 0.36
2-Acetamido-4-methylphenyl acetate


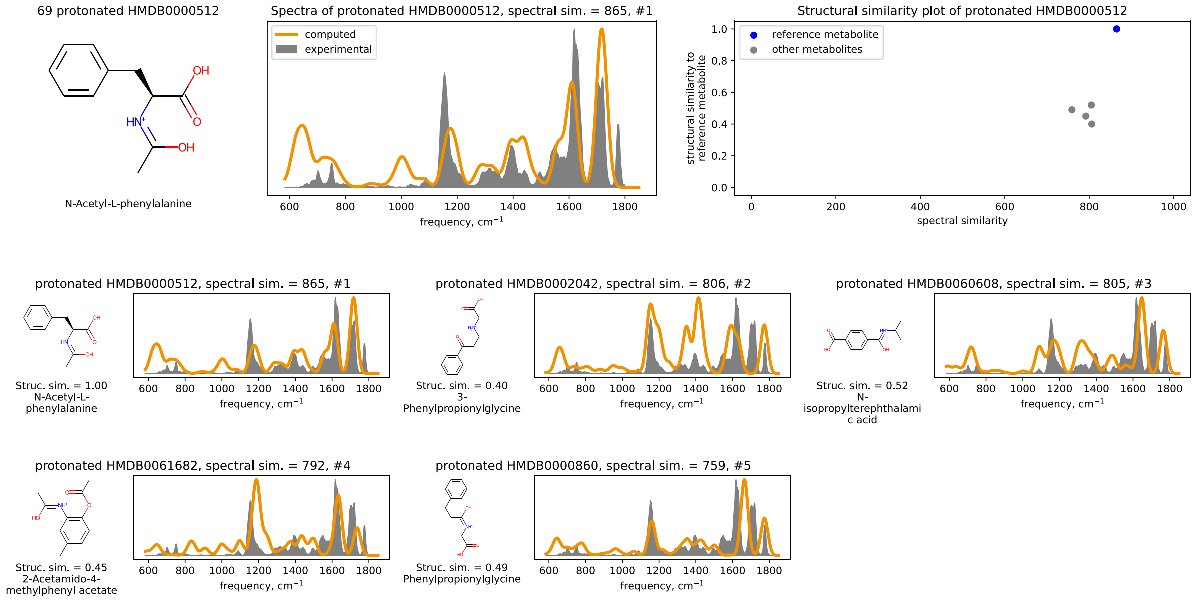

69 protonated HMDB0000512
N-Acetyl-L-phenylalanine
Spectra of protonated HMDB0000512, spectral sim. = 865, #1
computed
experimental
frequency, cm$^{-1}$
Structural similarity plot of protonated HMDB0000512
reference metabolite
other metabolites
structural similarity to reference metabolite
spectral similarity
protonated HMDB0000512, spectral sim. = 865, #1
Struc. sim. = 1.00
N-Acetyl-L-phenylalanine
protonated HMDB0002042, spectral sim. = 806, #2
Struc. sim. = 0.40
3-Phenylpropionylglycine
protonated HMDB0060608, spectral sim. = 805, #3
Struc. sim. = 0.52
N-isopropylterephthalamic acid
protonated HMDB0061682, spectral sim. = 792, #4
Struc. sim. = 0.45
2-Acetamido-4-methylphenyl acetate
protonated HMDB0000860, spectral sim. = 759, #5
Struc. sim. = 0.49
Phenylpropionylglycine


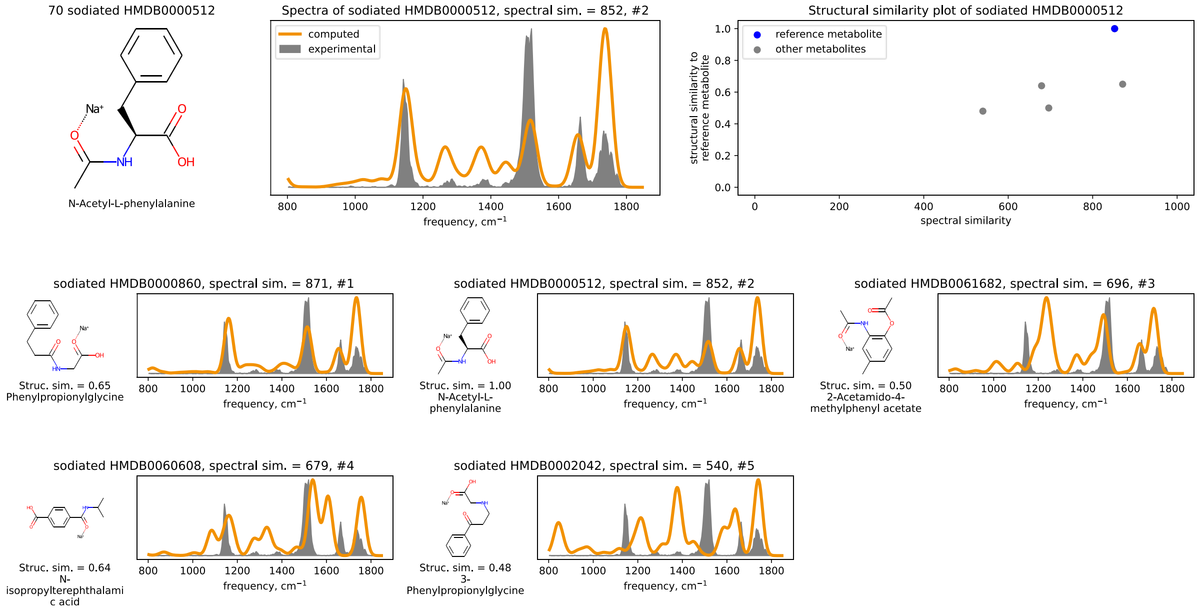

70 sodiated HMDB0000512
N-Acetyl-L-phenylalanine
Spectra of sodiated HMDB0000512, spectral sim. = 852, #2
computed
experimental
frequency, cm$^{-1}$
Structural similarity plot of sodiated HMDB0000512
reference metabolite
other metabolites
structural similarity to reference metabolite
spectral similarity
sodiated HMDB0000860, spectral sim. = 871, #1
Struc. sim. = 0.65
Phenylpropionylglycine
sodiated HMDB0000512, spectral sim. = 852, #2
Struc. sim. = 1.00
N-Acetyl-L-phenylalanine
sodiated HMDB0061682, spectral sim. = 696, #3
Struc. sim. = 0.50
2-Acetamido-4-methylphenyl acetate
sodiated HMDB0060608, spectral sim. = 679, #4
Struc. sim. = 0.64
N-isopropylterephthalamic acid
sodiated HMDB0002042, spectral sim. = 540, #5
Struc. sim. = 0.48
3-Phenylpropionylglycine

71 protonated HMDB0000517

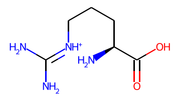


L-Arginine

Spectra of protonated HMDB0000517, spectral sim. = 925, #1

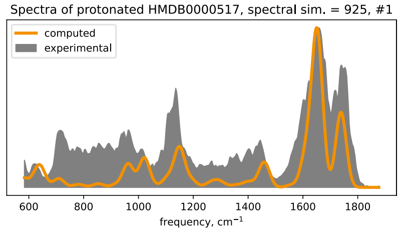


Structural similarity plot of protonated HMDB0000517

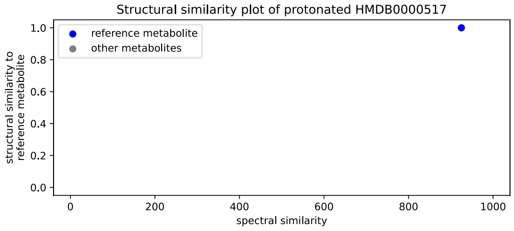


protonated HMDB0000517, spectral sim. = 925, #1

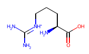

Struc. sim. = 1.00
L-Arginine

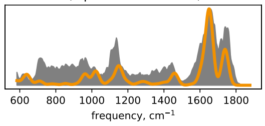


72 deprotonated HMDB0000522

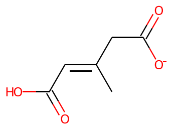


3-Methylglutaconic acid

Spectra of deprotonated HMDB0000522, spectral sim. = 797, #1

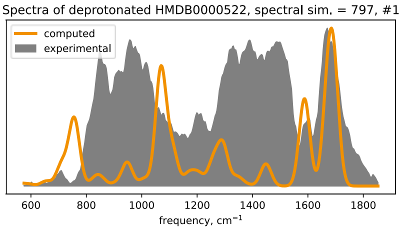


Structural similarity plot of deprotonated HMDB0000522

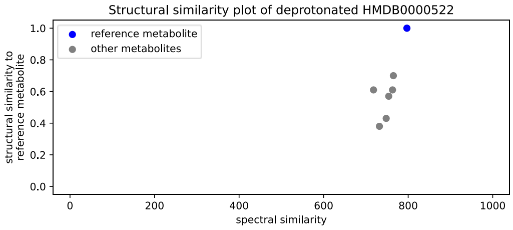


deprotonated HMDB0000522, spectral sim. = 797, #1

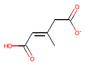

Struc. sim. = 1.00
3-Methylglutaconic acid

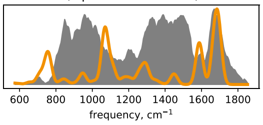


deprotonated HMDB0013311, spectral sim. = 765, #2

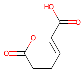

Struc. sim. = 0.70
trans-2-Hexenedioic acid

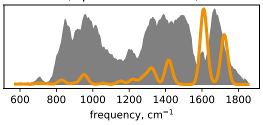


deprotonated HMDB0036232, spectral sim. = 763, #3

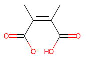

Struc. sim. = 0.61
Maleic acid homopolymer

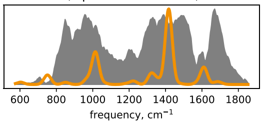


deprotonated HMDB0000393, spectral sim. = 754, #4

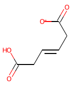

Struc. sim. = 0.57
3-Hexenedioic acid

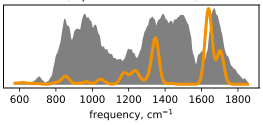


deprotonated HMDB0029171, spectral sim. = 748, #5

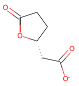

Struc. sim. = 0.43
3-Hydroxyadipic acid 3,6-lactone

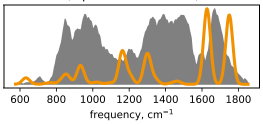


deprotonated HMDB0040207, spectral sim. = 732, #6

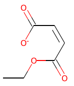

Struc. sim. = 0.38
Ethyl hydrogen fumarate

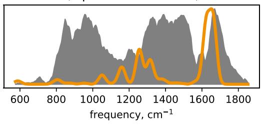


deprotonated HMDB0002266, spectral sim. = 718, #7

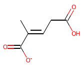

Struc. sim. = 0.61
(E)-2-Methylglutaconic acid

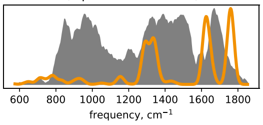

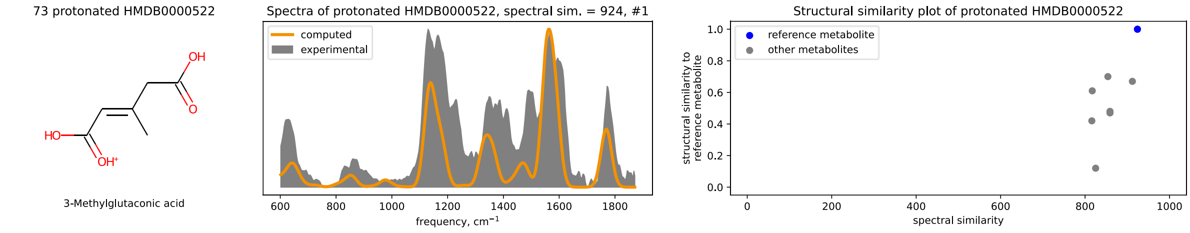
73 protonated HMDB0000522
3-Methylglutaconic acid
Spectra of protonated HMDB0000522, spectral sim. = 924, #1
computed
experimental
frequency, cm$^{-1}$
Structural similarity plot of protonated HMDB0000522
reference metabolite
other metabolites
structural similarity to reference metabolite
spectral similarity

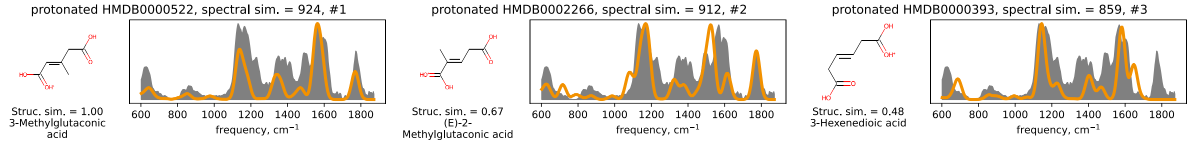
protonated HMDB0000522, spectral sim. = 924, #1
Struc. sim. = 1.00
3-Methylglutaconic acid
protonated HMDB0002266, spectral sim. = 912, #2
Struc. sim. = 0.67
(E)-2-Methylglutaconic acid
protonated HMDB0000393, spectral sim. = 859, #3
Struc. sim. = 0.48
3-Hexenedioic acid
frequency, cm$^{-1}$

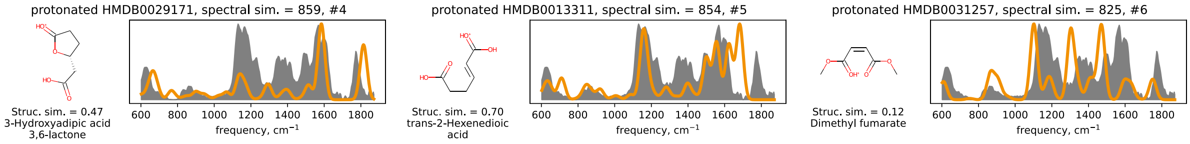
protonated HMDB0029171, spectral sim. = 859, #4
Struc. sim. = 0.47
3-Hydroxyadipic acid 3,6-lactone
protonated HMDB0013311, spectral sim. = 854, #5
Struc. sim. = 0.70
trans-2-Hexenedioic acid
protonated HMDB0031257, spectral sim. = 825, #6
Struc. sim. = 0.12
Dimethyl fumarate
frequency, cm$^{-1}$

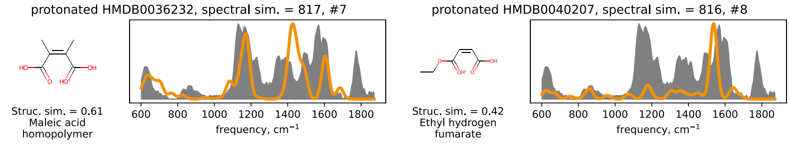
protonated HMDB0036232, spectral sim. = 817, #7
Struc. sim. = 0.61
Maleic acid homopolymer
protonated HMDB0040207, spectral sim. = 816, #8
Struc. sim. = 0.42
Ethyl hydrogen fumarate
frequency, cm$^{-1}$

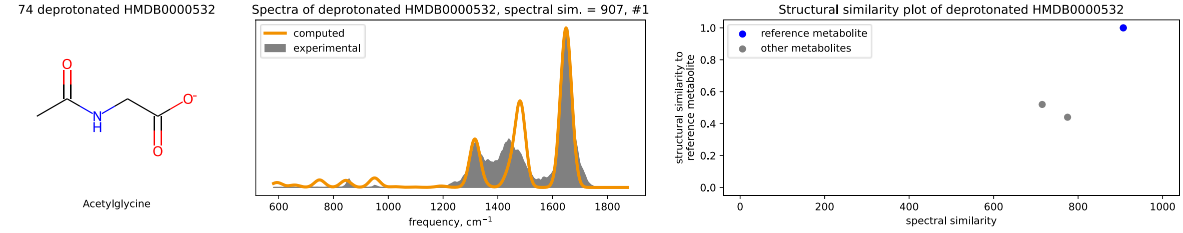
74 deprotonated HMDB0000532
Acetylglycine
Spectra of deprotonated HMDB0000532, spectral sim. = 907, #1
computed
experimental
frequency, cm$^{-1}$
Structural similarity plot of deprotonated HMDB0000532
reference metabolite
other metabolites
structural similarity to reference metabolite
spectral similarity

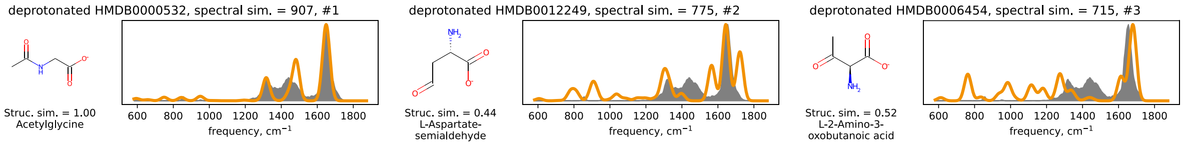
deprotonated HMDB0000532, spectral sim. = 907, #1
Struc. sim. = 1.00
Acetylglycine
deprotonated HMDB0012249, spectral sim. = 775, #2
Struc. sim. = 0.44
L-Aspartate-semialdehyde
deprotonated HMDB0006454, spectral sim. = 715, #3
Struc. sim. = 0.52
L-2-Amino-3-oxobutanoic acid
frequency, cm$^{-1}$

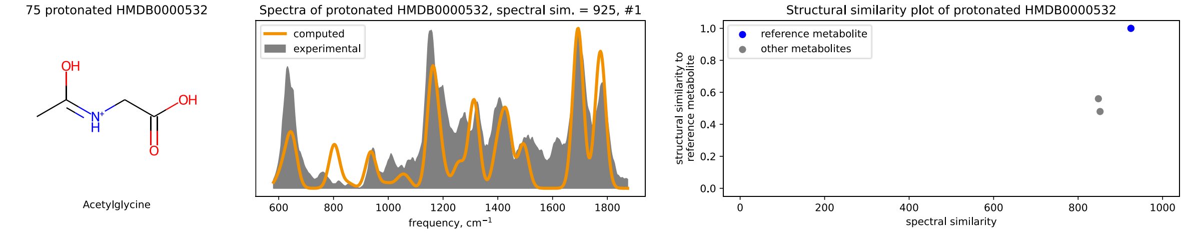
75 protonated HMDB0000532
Acetylglycine
Spectra of protonated HMDB0000532, spectral sim. = 925, #1
computed
experimental
frequency, cm$^{-1}$
Structural similarity plot of protonated HMDB0000532
reference metabolite
other metabolites
structural similarity to reference metabolite
spectral similarity

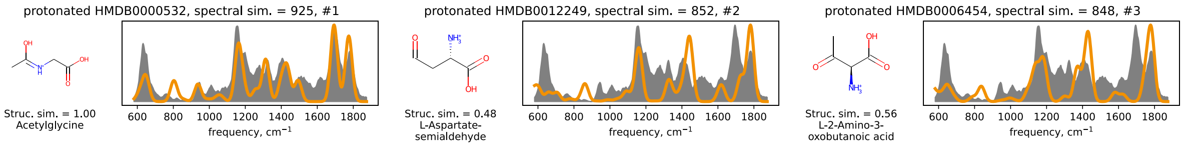
protonated HMDB0000532, spectral sim. = 925, #1
Struc. sim. = 1.00
Acetylglycine
protonated HMDB0012249, spectral sim. = 852, #2
Struc. sim. = 0.48
L-Aspartate-semialdehyde
protonated HMDB0006454, spectral sim. = 848, #3
Struc. sim. = 0.56
L-2-Amino-3-oxobutanoic acid
frequency, cm$^{-1}$

76 sodiated HMDB0000532

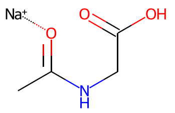


Acetylglycine

Spectra of sodiated HMDB0000532, spectral sim. = 915, #1

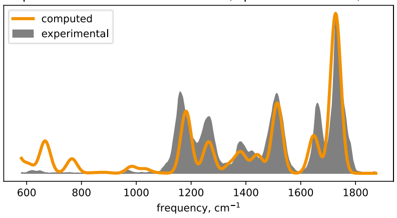


Structural similarity plot of sodiated HMDB0000532

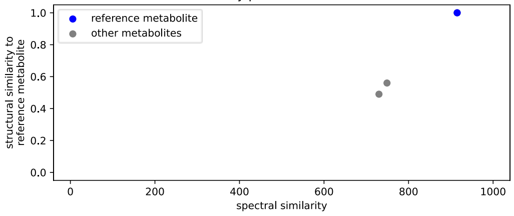


sodiated HMDB0000532, spectral sim. = 915, #1

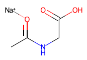

Struc. sim. = 1.00
Acetylglycine

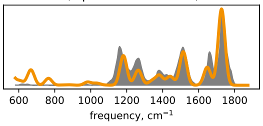


sodiated HMDB0006454, spectral sim. = 749, #2

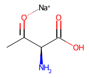

Struc. sim. = 0.56
L-2-Amino-3-oxobutanoic acid

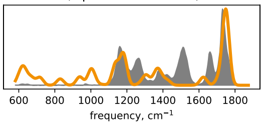


sodiated HMDB0012249, spectral sim. = 730, #3

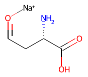

Struc. sim. = 0.49
L-Aspartate-semialdehyde

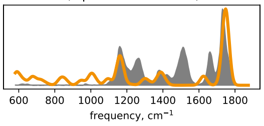


77 deprotonated HMDB0000622

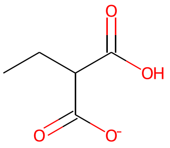


Ethylmalonic acid

Spectra of deprotonated HMDB0000622, spectral sim. = 773, #1

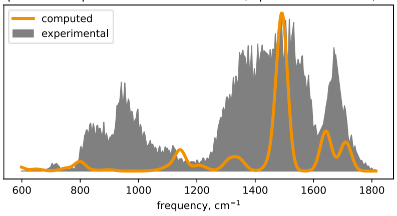


Structural similarity plot of deprotonated HMDB0000622

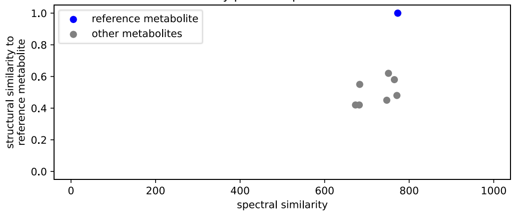


deprotonated HMDB0000622, spectral sim. = 773, #1

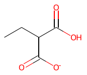

Struc. sim. = 1.00
Ethylmalonic acid

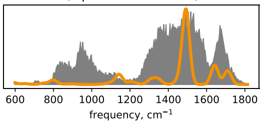


deprotonated HMDB0040531, spectral sim. = 771, #2

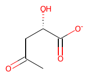

Struc. sim. = 0.48
2-Hydroxy-4-oxopentanoic acid

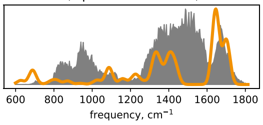


deprotonated HMDB0002001, spectral sim. = 765, #3

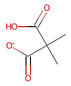

Struc. sim. = 0.58
Dimethylmalonic acid

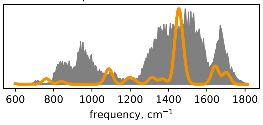


deprotonated HMDB0001844, spectral sim. = 751, #4

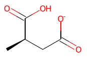

Struc. sim. = 0.62
Methylsuccinic acid

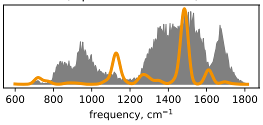


deprotonated HMDB0006833, spectral sim. = 747, #5

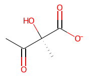

Struc. sim. = 0.45
2-Acetolactate

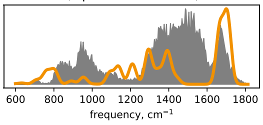


deprotonated HMDB0000661, spectral sim. = 683, #6

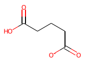

Struc. sim. = 0.55
Glutaric acid

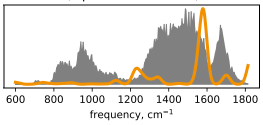


deprotonated HMDB0061879, spectral sim. = 682, #7

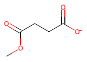

Struc. sim. = 0.42
(S)-Methylbutanethioic acid

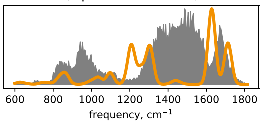


deprotonated HMDB0000576, spectral sim. = 673, #8

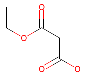

Struc. sim. = 0.42
Monoethyl malonic acid

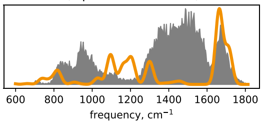

78 protonated HMDB0000622

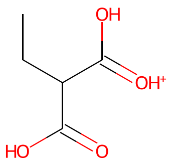


Ethylmalonic acid

Spectra of protonated HMDB0000622, spectral sim. = 837, #2

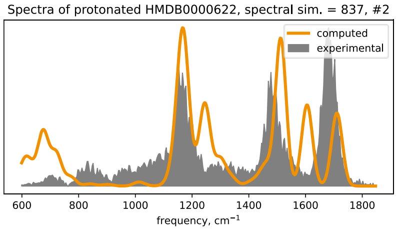


Structural similarity plot of protonated HMDB0000622

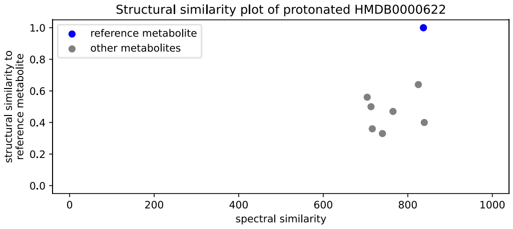


protonated HMDB0040531, spectral sim. = 839, #1

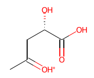

Struc. sim. = 0.40
2-Hydroxy-4-oxopentanoic acid

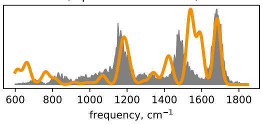


protonated HMDB0000622, spectral sim. = 837, #2

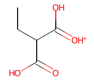

Struc. sim. = 1.00
Ethylmalonic acid

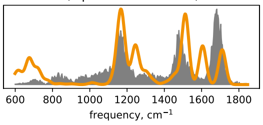


protonated HMDB0002001, spectral sim. = 825, #3

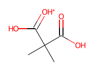

Struc. sim. = 0.64
Dimethylmalonic acid

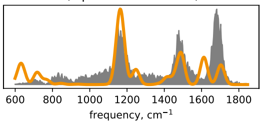


protonated HMDB0000576, spectral sim. = 765, #4

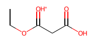

Struc. sim. = 0.47
Monoethyl malonic acid

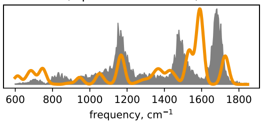


protonated HMDB0061879, spectral sim. = 740, #5

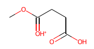

Struc. sim. = 0.33
(S)-Methylbutanethioic acid

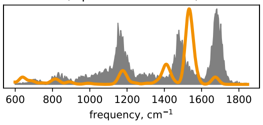


protonated HMDB0006833, spectral sim. = 716, #6

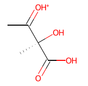

Struc. sim. = 0.36
2-Acetolactate

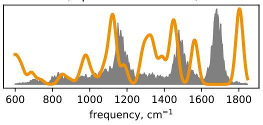


protonated HMDB0000661, spectral sim. = 713, #7

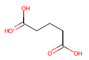

Struc. sim. = 0.50
Glutaric acid

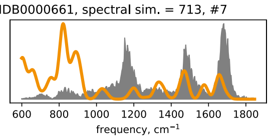


protonated HMDB0001844, spectral sim. = 704, #8

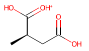

Struc. sim. = 0.56
Methylsuccinic acid

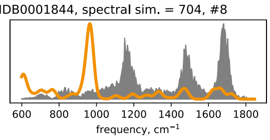


79 deprotonated HMDB0000630

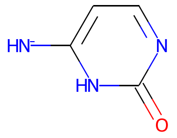


Cytosine

Spectra of deprotonated HMDB0000630, spectral sim. = 913, #1

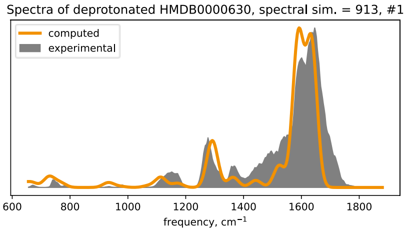


Structural similarity plot of deprotonated HMDB0000630

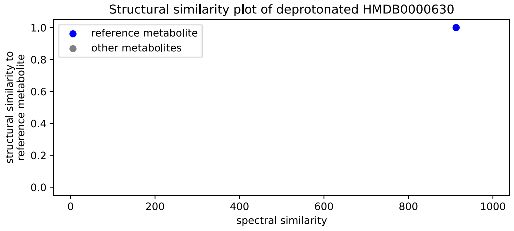


deprotonated HMDB0000630, spectral sim. = 913, #1

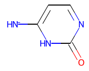

Struc. sim. = 1.00
Cytosine

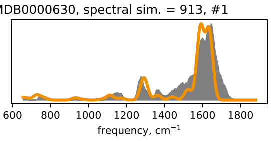


80 protonated HMDB0000630

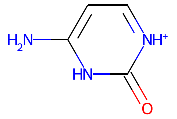


Cytosine

Spectra of protonated HMDB0000630, spectral sim. = 796, #1

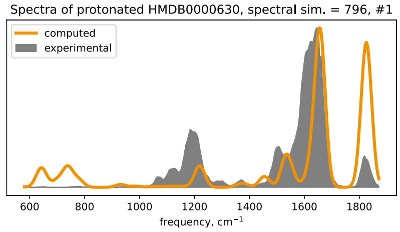


Structural similarity plot of protonated HMDB0000630

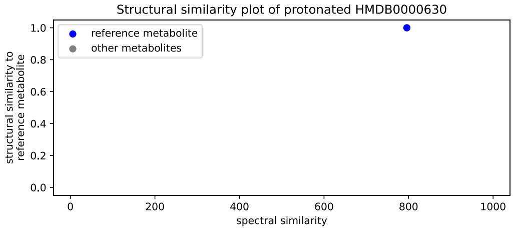


protonated HMDB0000630, spectral sim. = 796, #1

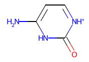

Struc. sim. = 1.00
Cytosine

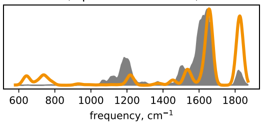

81 sodiated HMDB0000630

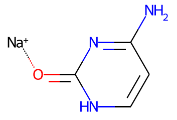

Cytosine

Spectra of sodiated HMDB0000630, spectral sim. = 933, #1

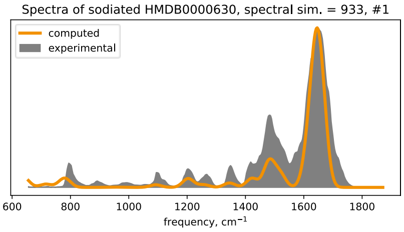


Structural similarity plot of sodiated HMDB0000630

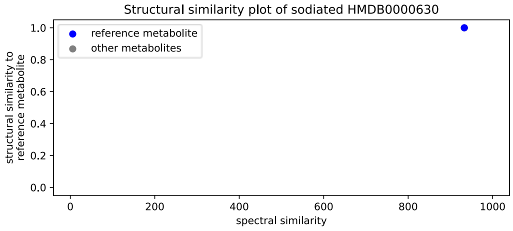


sodiated HMDB0000630, spectral sim. = 933, #1

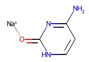

Struc. sim. = 1.00
Cytosine

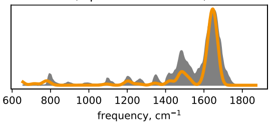


82 deprotonated HMDB0000641

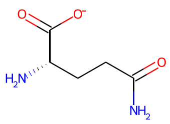

L-Glutamine

Spectra of deprotonated HMDB0000641, spectral sim. = 880, #1

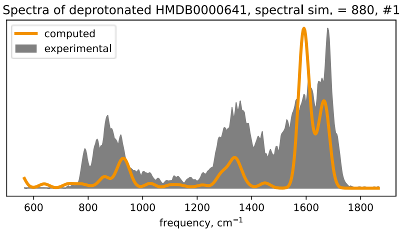


Structural similarity plot of deprotonated HMDB0000641

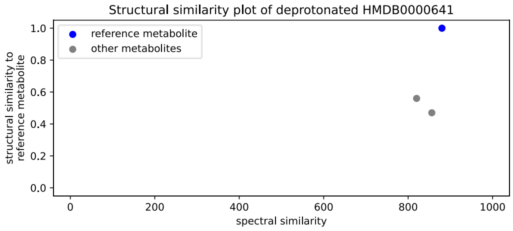


deprotonated HMDB0000641, spectral sim. = 880, #1

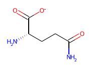

Struc. sim. = 1.00
L-Glutamine

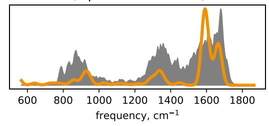


deprotonated HMDB0002031, spectral sim. = 856, #2

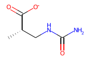

Struc. sim. = 0.47
Ureidoisobutyric acid

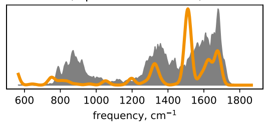


deprotonated HMDB0006899, spectral sim. = 820, #3

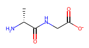

Struc. sim. = 0.56
Alanylglycine

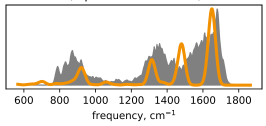


83 protonated HMDB0000641

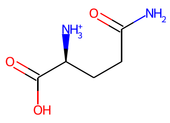

L-Glutamine

Spectra of protonated HMDB0000641, spectral sim. = 908, #1

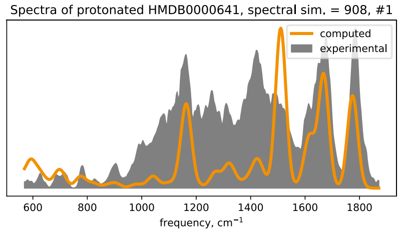


Structural similarity plot of protonated HMDB0000641

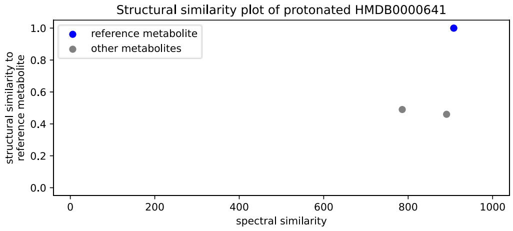


protonated HMDB0000641, spectral sim. = 908, #1

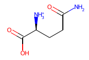

Struc. sim. = 1.00
L-Glutamine

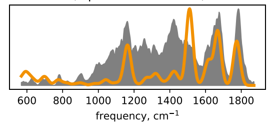


protonated HMDB0006899, spectral sim. = 891, #2

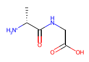

Struc. sim. = 0.46
Alanylglycine

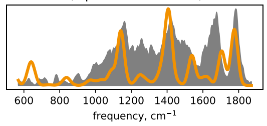


protonated HMDB0002031, spectral sim. = 786, #3

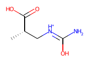

Struc. sim. = 0.49
Ureidoisobutyric acid

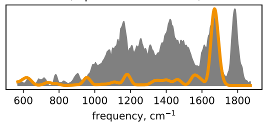


84 sodiated HMDB0000641

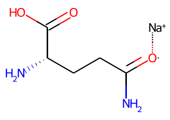

L-Glutamine

Spectra of sodiated HMDB0000641, spectral sim. = 861, #1

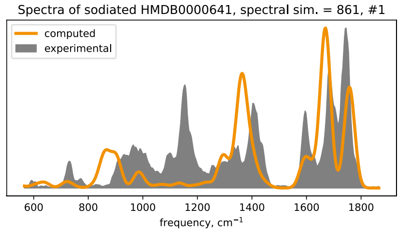


Structural similarity plot of sodiated HMDB0000641

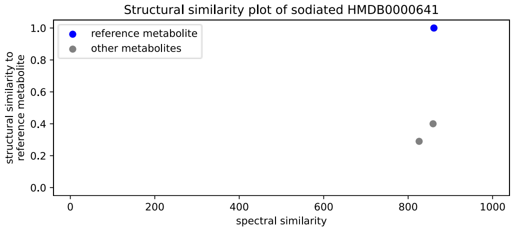


sodiated HMDB0000641, spectral sim. = 861, #1

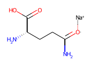

Struc. sim. = 1.00
L-Glutamine

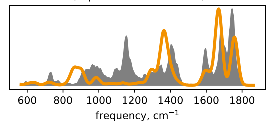


sodiated HMDB0006899, spectral sim. = 859, #2

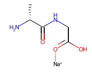

Struc. sim. = 0.40
Alanylglycine

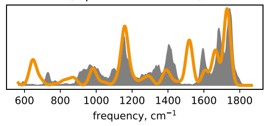


sodiated HMDB0002031, spectral sim. = 826, #3

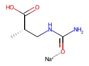

Struc. sim. = 0.29
Ureidoisobutyric acid

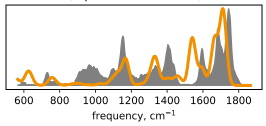

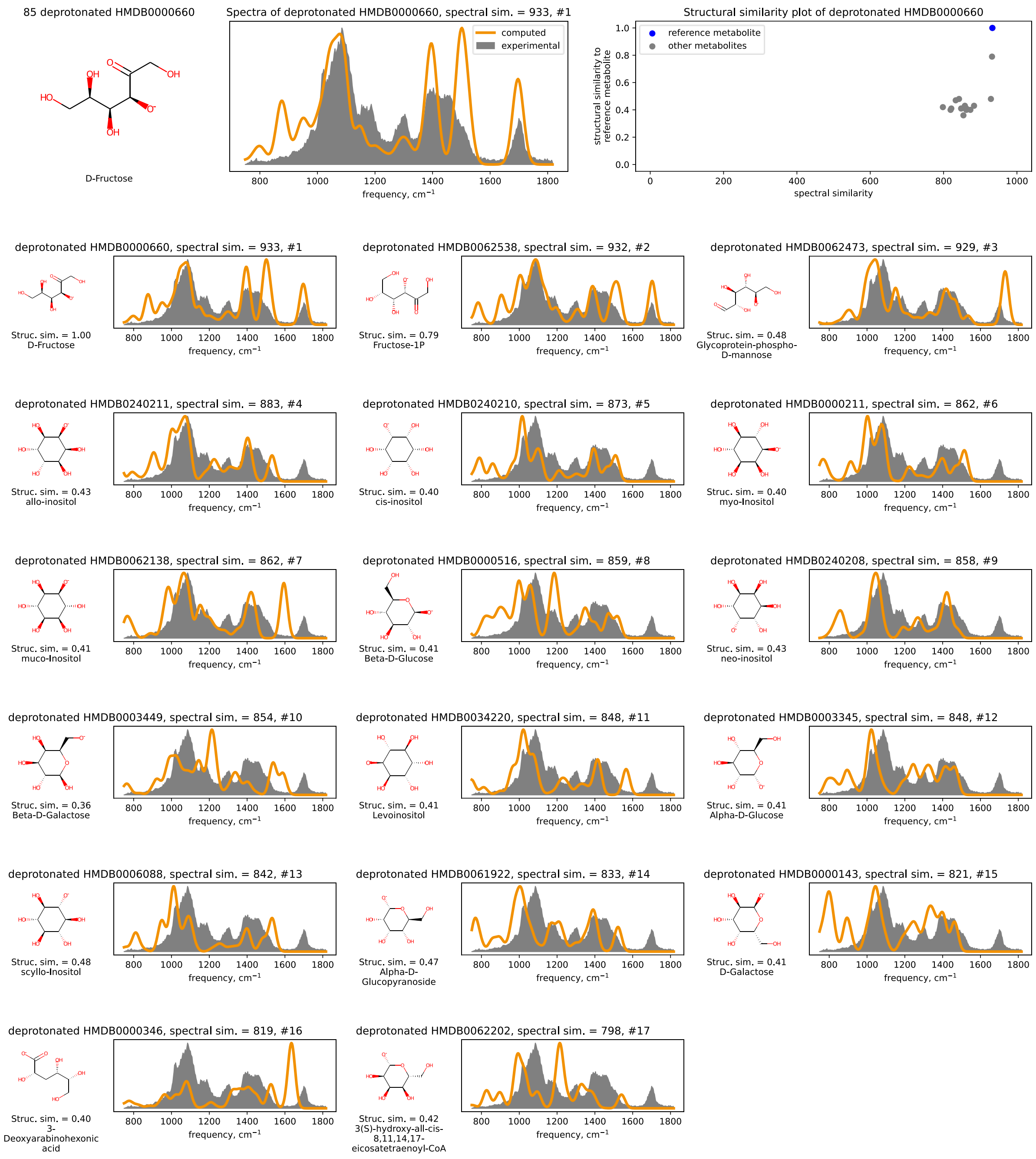
85 deprotonated HMDB0000660
D-Fructose
Spectra of deprotonated HMDB0000660, spectral sim. = 933, #1
computed
experimental
frequency, cm⁻¹
Structural similarity plot of deprotonated HMDB0000660
reference metabolite
other metabolites
structural similarity to reference metabolite
spectral similarity
deprotonated HMDB0000660, spectral sim. = 933, #1
Struc. sim. = 1.00
D-Fructose
deprotonated HMDB0062538, spectral sim. = 932, #2
Struc. sim. = 0.79
Fructose-1P
deprotonated HMDB0062473, spectral sim. = 929, #3
Struc. sim. = 0.48
Glycoprotein-phospho-D-mannose
deprotonated HMDB0240211, spectral sim. = 883, #4
Struc. sim. = 0.43
allo-inositol
deprotonated HMDB0240210, spectral sim. = 873, #5
Struc. sim. = 0.40
cis-inositol
deprotonated HMDB0000211, spectral sim. = 862, #6
Struc. sim. = 0.40
myo-Inositol
deprotonated HMDB0062138, spectral sim. = 862, #7
Struc. sim. = 0.41
muco-Inositol
deprotonated HMDB0000516, spectral sim. = 859, #8
Struc. sim. = 0.41
Beta-D-Glucose
deprotonated HMDB0240208, spectral sim. = 858, #9
Struc. sim. = 0.43
neo-inositol
deprotonated HMDB0003449, spectral sim. = 854, #10
Struc. sim. = 0.36
Beta-D-Galactose
deprotonated HMDB0034220, spectral sim. = 848, #11
Struc. sim. = 0.41
Levoinositol
deprotonated HMDB0003345, spectral sim. = 848, #12
Struc. sim. = 0.41
Alpha-D-Glucose
deprotonated HMDB0006088, spectral sim. = 842, #13
Struc. sim. = 0.48
scyllo-Inositol
deprotonated HMDB0061922, spectral sim. = 833, #14
Struc. sim. = 0.47
Alpha-D-Glucopyranoside
deprotonated HMDB0000143, spectral sim. = 821, #15
Struc. sim. = 0.41
D-Galactose
deprotonated HMDB0000346, spectral sim. = 819, #16
Struc. sim. = 0.40
3-Deoxyarabinohexonic acid
deprotonated HMDB0062202, spectral sim. = 798, #17
Struc. sim. = 0.42
3(S)-hydroxy-all-cis-8,11,14,17-eicosatetraenoyl-CoA
frequency, cm⁻¹

86 deprotonated HMDB0000661

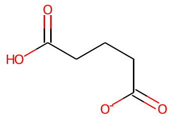

Glutaric acid

Spectra of deprotonated HMDB0000661, spectral sim. = 629, #8

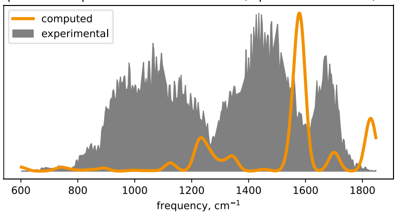


Structural similarity plot of deprotonated HMDB0000661

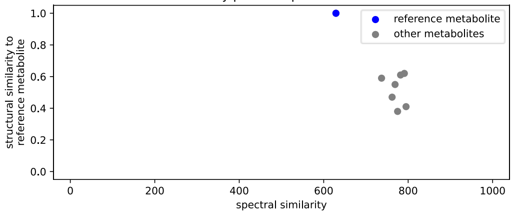


deprotonated HMDB0040531, spectral sim. = 795, #1

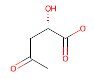

Struc. sim. = 0.41
2-Hydroxy-4-oxopentanoic acid

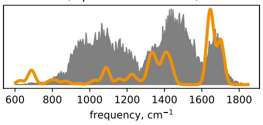

deprotonated HMDB0002001, spectral sim. = 791, #2

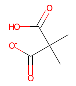

Struc. sim. = 0.62
Dimethylmalonic acid

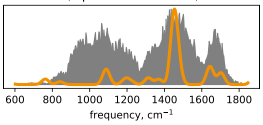

deprotonated HMDB0001844, spectral sim. = 782, #3

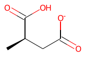

Struc. sim. = 0.61
Methylsuccinic acid

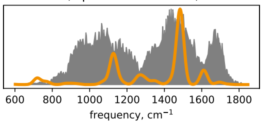

deprotonated HMDB0006833, spectral sim. = 775, #4

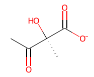

Struc. sim. = 0.38
2-Acetolactate

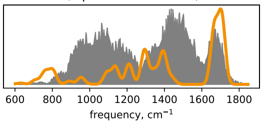

deprotonated HMDB0000622, spectral sim. = 769, #5

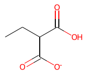

Struc. sim. = 0.55
Ethylmalonic acid

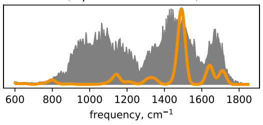

deprotonated HMDB0000576, spectral sim. = 762, #6

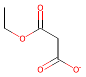

Struc. sim. = 0.47
Monoethyl malonic acid

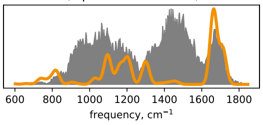

deprotonated HMDB0061879, spectral sim. = 737, #7

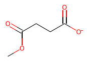

Struc. sim. = 0.59
(S)-Methylbutanethioic acid

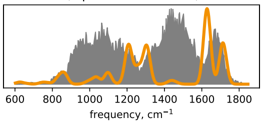

deprotonated HMDB0000661, spectral sim. = 629, #8

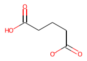

Struc. sim. = 1.00
Glutaric acid

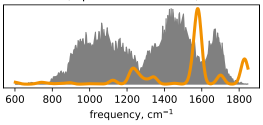

87 protonated HMDB0000661

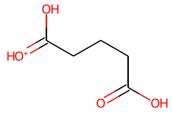

Glutaric acid

Spectra of protonated HMDB0000661, spectral sim. = 701, #7

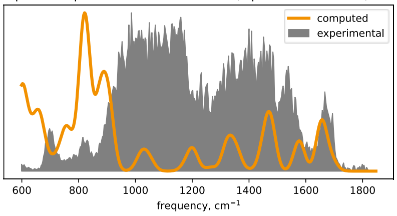


Structural similarity plot of protonated HMDB0000661

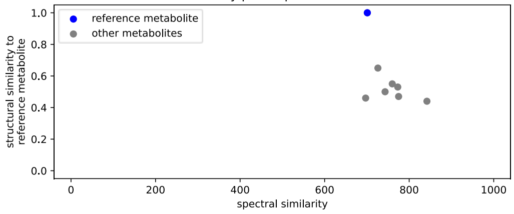


protonated HMDB0006833, spectral sim. = 842, #1

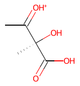

Struc. sim. = 0.44
2-Acetolactate

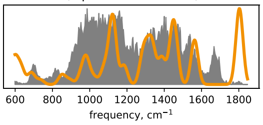

protonated HMDB0040531, spectral sim. = 775, #2

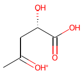

Struc. sim. = 0.47
2-Hydroxy-4-oxopentanoic acid

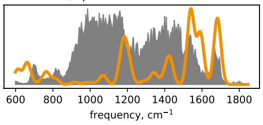

protonated HMDB0000576, spectral sim. = 773, #3

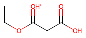

Struc. sim. = 0.53
Monoethyl malonic acid

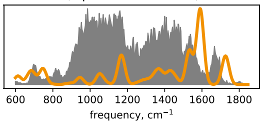

protonated HMDB0001844, spectral sim. = 760, #4

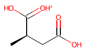

Struc. sim. = 0.55
Methylsuccinic acid

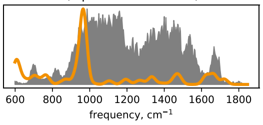

protonated HMDB0000622, spectral sim. = 743, #5

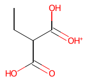

Struc. sim. = 0.50
Ethylmalonic acid

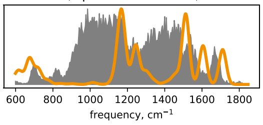

protonated HMDB0061879, spectral sim. = 726, #6

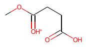

Struc. sim. = 0.65
(S)-Methylbutanethioic acid

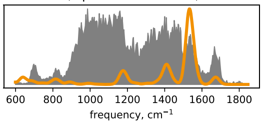

protonated HMDB0000661, spectral sim. = 701, #7

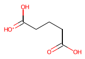

Struc. sim. = 1.00
Glutaric acid

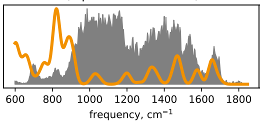

protonated HMDB0002001, spectral sim. = 697, #8

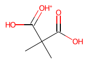

Struc. sim. = 0.46
Dimethylmalonic acid

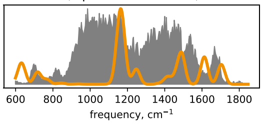

88 deprotonated HMDB0000679

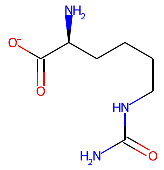

Homocitrulline

Spectra of deprotonated HMDB0000679, spectral sim. = 924, #1

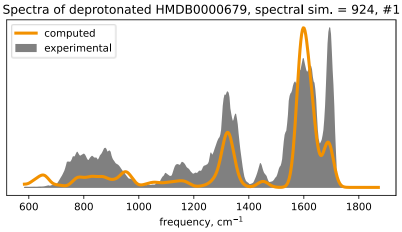


Structural similarity plot of deprotonated HMDB0000679

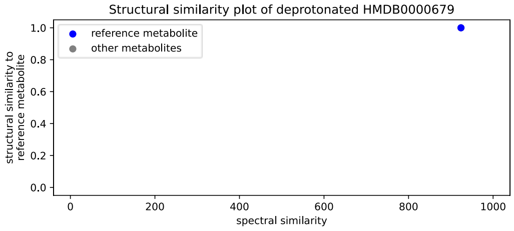


deprotonated HMDB0000679, spectral sim. = 924, #1

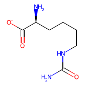

Struc. sim. = 1.00
Homocitrulline

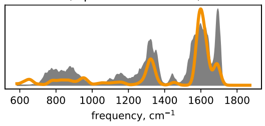


89 protonated HMDB0000679

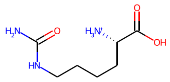

Homocitrulline

Spectra of protonated HMDB0000679, spectral sim. = 956, #1

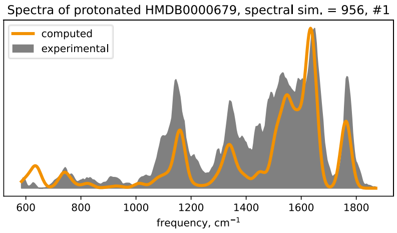


Structural similarity plot of protonated HMDB0000679

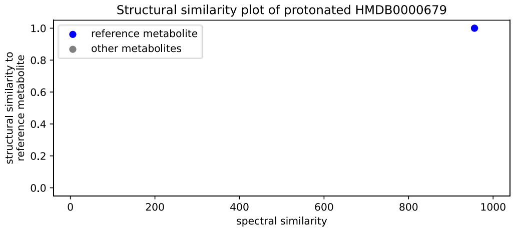


protonated HMDB0000679, spectral sim. = 956, #1

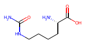

Struc. sim. = 1.00
Homocitrulline

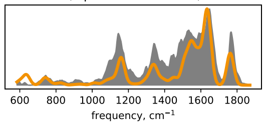


90 sodiated HMDB0000679

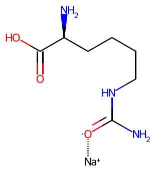

Homocitrulline

Spectra of sodiated HMDB0000679, spectral sim. = 920, #1

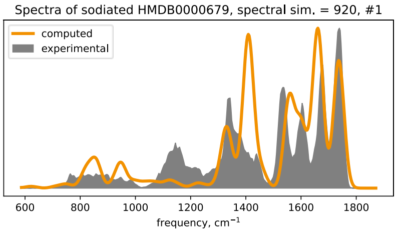


Structural similarity plot of sodiated HMDB0000679

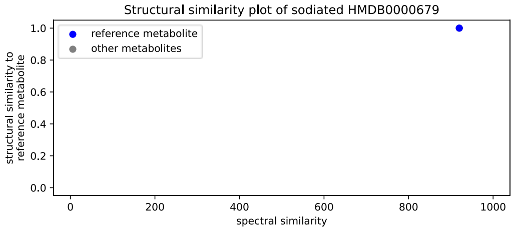


sodiated HMDB0000679, spectral sim. = 920, #1

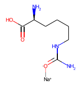

Struc. sim. = 1.00
Homocitrulline

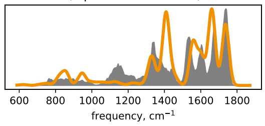

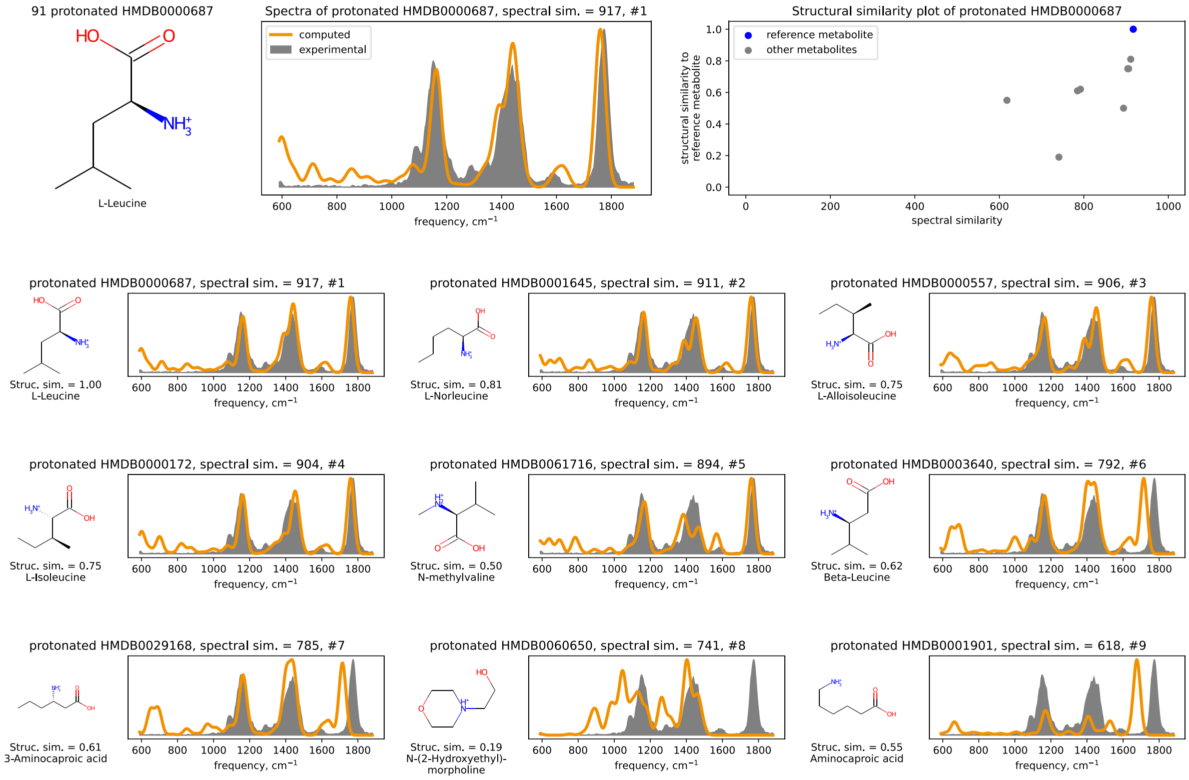
91 protonated HMDB0000687
L-Leucine
Spectra of protonated HMDB0000687, spectral sim. = 917, #1
computed
experimental
frequency, cm⁻¹
Structural similarity plot of protonated HMDB0000687
reference metabolite
other metabolites
structural similarity to reference metabolite
spectral similarity
protonated HMDB0000687, spectral sim. = 917, #1
Struc. sim. = 1.00
L-Leucine
protonated HMDB0001645, spectral sim. = 911, #2
Struc. sim. = 0.81
L-Norleucine
protonated HMDB0000557, spectral sim. = 906, #3
Struc. sim. = 0.75
L-Alloisoleucine
protonated HMDB0000172, spectral sim. = 904, #4
Struc. sim. = 0.75
L-Isoleucine
protonated HMDB0061716, spectral sim. = 894, #5
Struc. sim. = 0.50
N-methylvaline
protonated HMDB0003640, spectral sim. = 792, #6
Struc. sim. = 0.62
Beta-Leucine
protonated HMDB0029168, spectral sim. = 785, #7
Struc. sim. = 0.61
3-Aminocaproic acid
protonated HMDB0060650, spectral sim. = 741, #8
Struc. sim. = 0.19
N-(2-Hydroxyethyl)-morpholine
protonated HMDB0001901, spectral sim. = 618, #9
Struc. sim. = 0.55
Aminocaproic acid

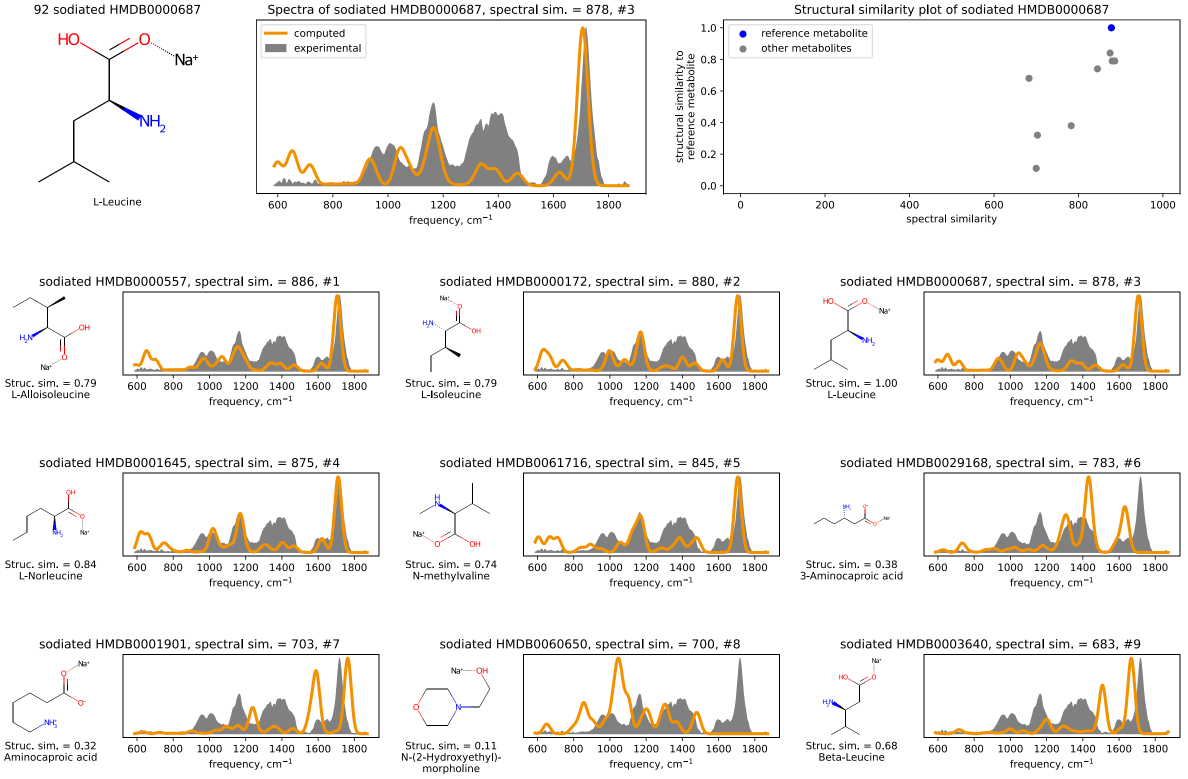
92 sodiated HMDB0000687
L-Leucine
Spectra of sodiated HMDB0000687, spectral sim. = 878, #3
computed
experimental
frequency, cm⁻¹
Structural similarity plot of sodiated HMDB0000687
reference metabolite
other metabolites
structural similarity to reference metabolite
spectral similarity
sodiated HMDB0000557, spectral sim. = 886, #1
Struc. sim. = 0.79
L-Alloisoleucine
sodiated HMDB0000172, spectral sim. = 880, #2
Struc. sim. = 0.79
L-Isoleucine
sodiated HMDB0000687, spectral sim. = 878, #3
Struc. sim. = 1.00
L-Leucine
sodiated HMDB0001645, spectral sim. = 875, #4
Struc. sim. = 0.84
L-Norleucine
sodiated HMDB0061716, spectral sim. = 845, #5
Struc. sim. = 0.74
N-methylvaline
sodiated HMDB0029168, spectral sim. = 783, #6
Struc. sim. = 0.38
3-Aminocaproic acid
sodiated HMDB0001901, spectral sim. = 703, #7
Struc. sim. = 0.32
Aminocaproic acid
sodiated HMDB0060650, spectral sim. = 700, #8
Struc. sim. = 0.11
N-(2-Hydroxyethyl)-morpholine
sodiated HMDB0003640, spectral sim. = 683, #9
Struc. sim. = 0.68
Beta-Leucine

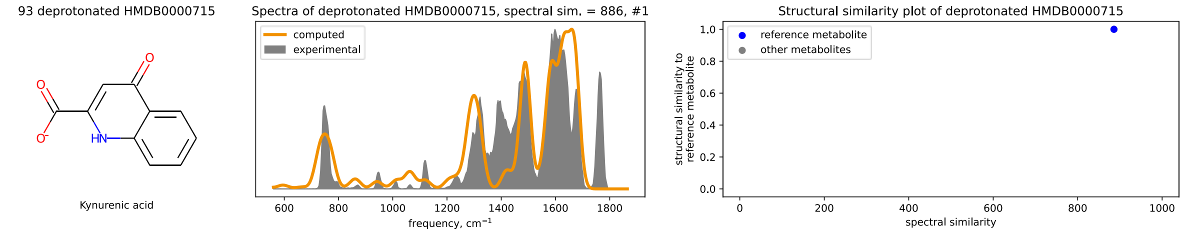
93 deprotonated HMDB0000715
Kynurenic acid
Spectra of deprotonated HMDB0000715, spectral sim. = 886, #1
computed
experimental
frequency, cm⁻¹
Structural similarity plot of deprotonated HMDB0000715
reference metabolite
other metabolites
structural similarity to reference metabolite
spectral similarity

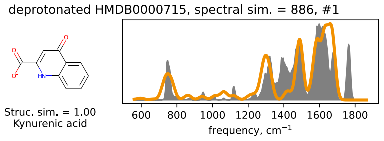
deprotonated HMDB0000715, spectral sim. = 886, #1
Struc. sim. = 1.00
Kynurenic acid
frequency, cm⁻¹

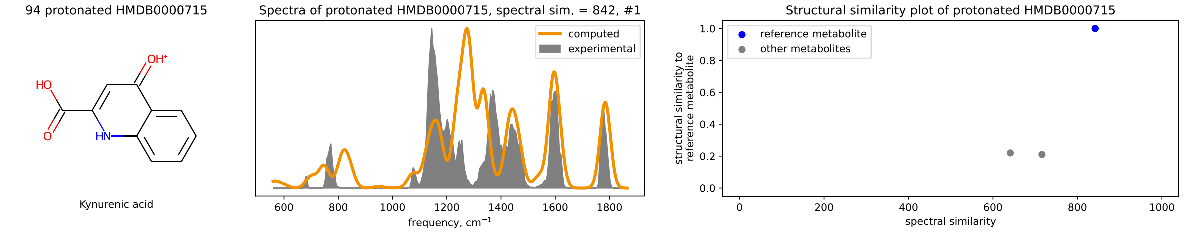
94 protonated HMDB0000715
Kynurenic acid
Spectra of protonated HMDB0000715, spectral sim. = 842, #1
computed
experimental
frequency, cm⁻¹
Structural similarity plot of protonated HMDB0000715
reference metabolite
other metabolites
structural similarity to reference metabolite
spectral similarity

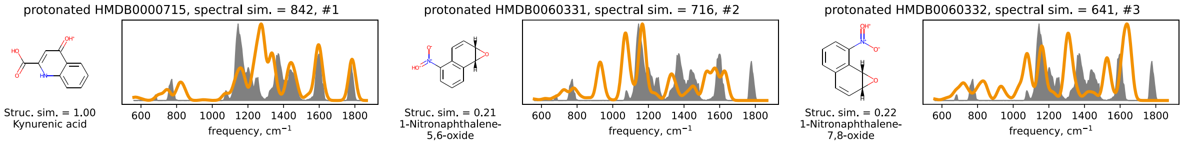
protonated HMDB0000715, spectral sim. = 842, #1
Struc. sim. = 1.00
Kynurenic acid
protonated HMDB0060331, spectral sim. = 716, #2
Struc. sim. = 0.21
1-Nitronaphthalene-5,6-oxide
protonated HMDB0060332, spectral sim. = 641, #3
Struc. sim. = 0.22
1-Nitronaphthalene-7,8-oxide
frequency, cm⁻¹

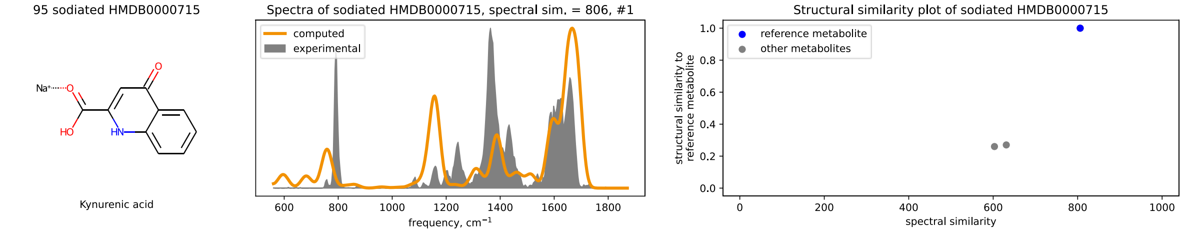
95 sodiated HMDB0000715
Kynurenic acid
Spectra of sodiated HMDB0000715, spectral sim. = 806, #1
computed
experimental
frequency, cm⁻¹
Structural similarity plot of sodiated HMDB0000715
reference metabolite
other metabolites
structural similarity to reference metabolite
spectral similarity

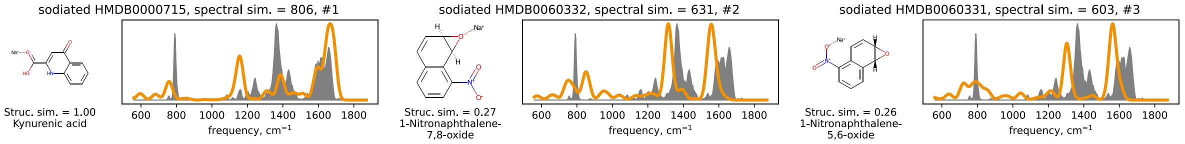
sodiated HMDB0000715, spectral sim. = 806, #1
Struc. sim. = 1.00
Kynurenic acid
sodiated HMDB0060332, spectral sim. = 631, #2
Struc. sim. = 0.27
1-Nitronaphthalene-7,8-oxide
sodiated HMDB0060331, spectral sim. = 603, #3
Struc. sim. = 0.26
1-Nitronaphthalene-5,6-oxide
frequency, cm⁻¹

96 deprotonated HMDB0000759

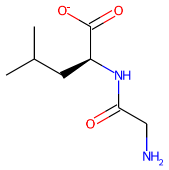


Glycylleucine

Spectra of deprotonated HMDB0000759, spectral sim. = 908, #2

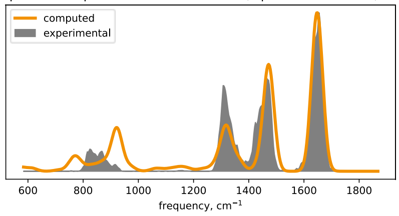


Structural similarity plot of deprotonated HMDB0000759

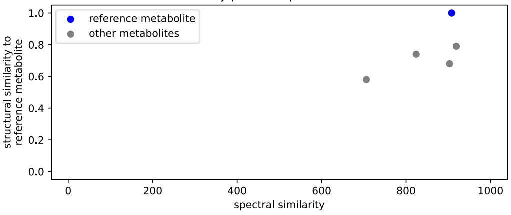


deprotonated HMDB0000446, spectral sim. = 919, #1

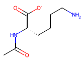

Struc. sim. = 0.79
N-Alpha-acetyllysine

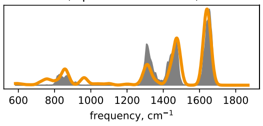


deprotonated HMDB0000759, spectral sim. = 908, #2

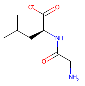

Struc. sim. = 1.00
Glycylleucine

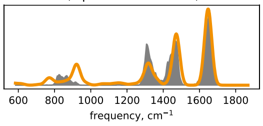


deprotonated HMDB0028929, spectral sim. = 903, #3

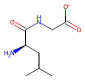

Struc. sim. = 0.68
Leucyl-Glycine

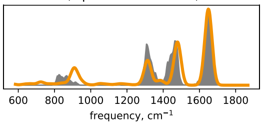


deprotonated HMDB0028700, spectral sim. = 824, #4

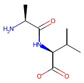

Struc. sim. = 0.74
Alanyl-Valine

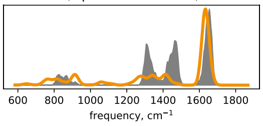


deprotonated HMDB0000206, spectral sim. = 706, #5

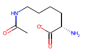

Struc. sim. = 0.58
N6-Acetyl-L-lysine

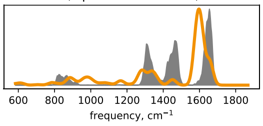


97 protonated HMDB0000759

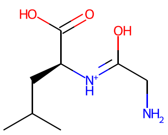


Glycylleucine

Spectra of protonated HMDB0000759, spectral sim. = 854, #3

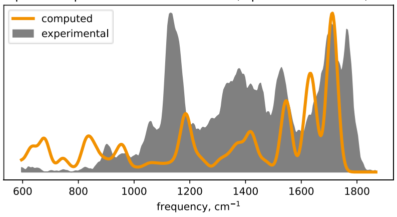


Structural similarity plot of protonated HMDB0000759

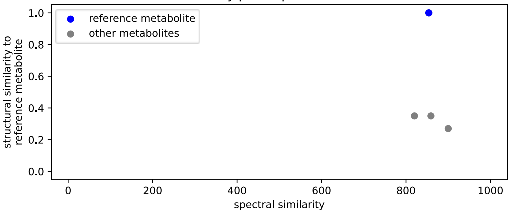


protonated HMDB0028700, spectral sim. = 900, #1

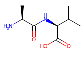

Struc. sim. = 0.27
Alanyl-Valine

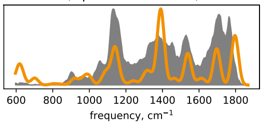


protonated HMDB0000206, spectral sim. = 859, #2

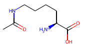

Struc. sim. = 0.35
N6-Acetyl-L-lysine

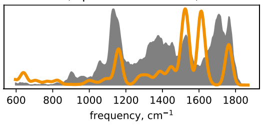


protonated HMDB0000759, spectral sim. = 854, #3

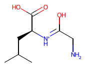

Struc. sim. = 1.00
Glycylleucine

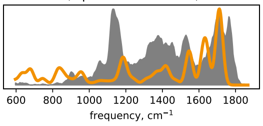


protonated HMDB0000446, spectral sim. = 820, #4

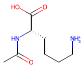

Struc. sim. = 0.35
N-Alpha-acetyllysine

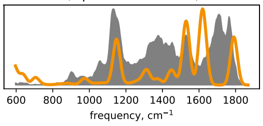


98 deprotonated HMDB0000765

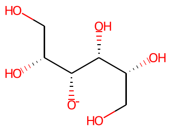


Mannitol

Spectra of deprotonated HMDB0000765, spectral sim. = 910, #2

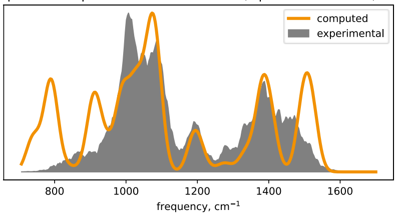


Structural similarity plot of deprotonated HMDB0000765

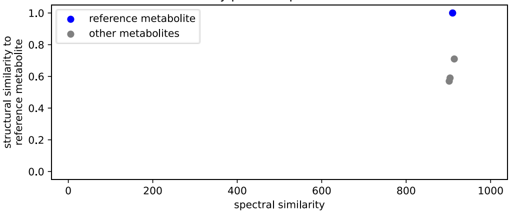


deprotonated HMDB0000107, spectral sim. = 914, #1

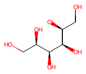

Struc. sim. = 0.71
Galactitol

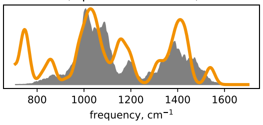


deprotonated HMDB0000765, spectral sim. = 910, #2

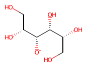

Struc. sim. = 1.00
Mannitol

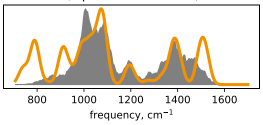


deprotonated HMDB0000247, spectral sim. = 904, #3

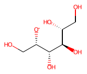

Struc. sim. = 0.59
Sorbitol

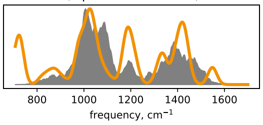


deprotonated HMDB0011632, spectral sim. = 902, #4

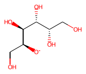

Struc. sim. = 0.57
L-Iditol

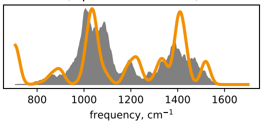

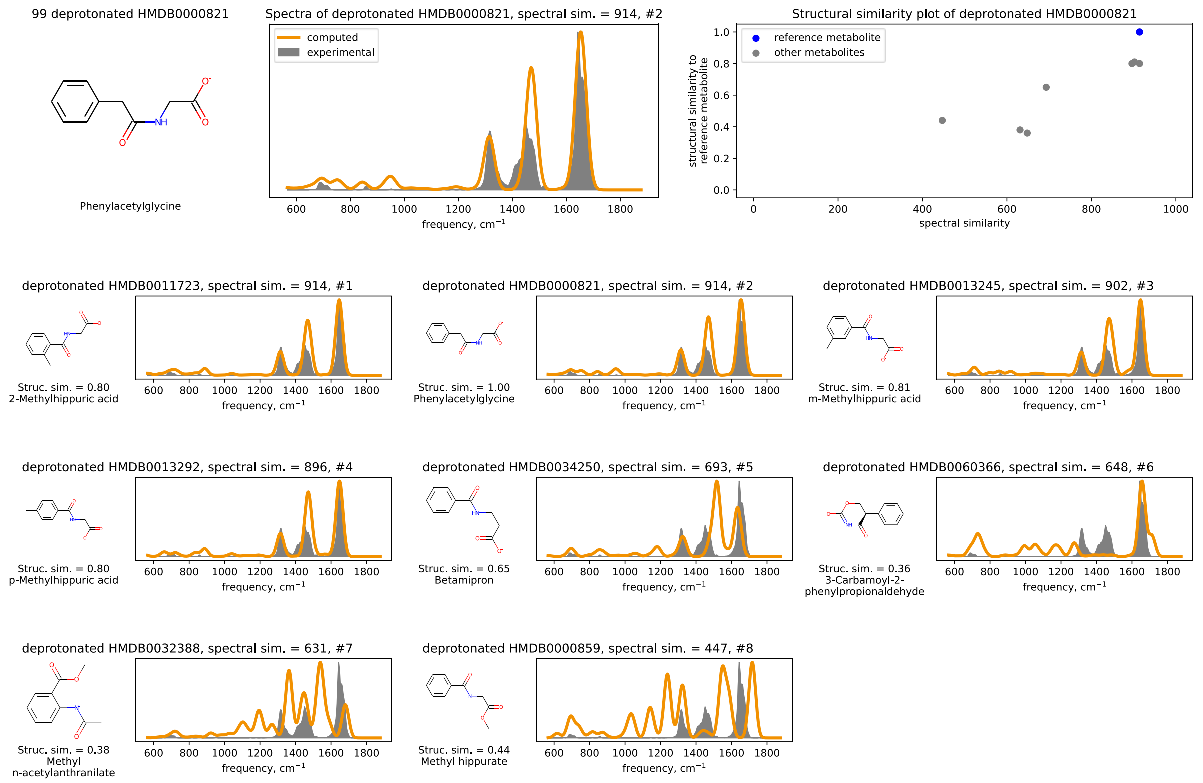

99 deprotonated HMDB0000821
Phenylacetylglycine
Spectra of deprotonated HMDB0000821, spectral sim. = 914, #2
computed
experimental
frequency, cm$^{-1}$
Structural similarity plot of deprotonated HMDB0000821
reference metabolite
other metabolites
structural similarity to reference metabolite
spectral similarity
deprotonated HMDB0011723, spectral sim. = 914, #1
Struc. sim. = 0.80
2-Methylhippuric acid
deprotonated HMDB0000821, spectral sim. = 914, #2
Struc. sim. = 1.00
Phenylacetylglycine
deprotonated HMDB0013245, spectral sim. = 902, #3
Struc. sim. = 0.81
m-Methylhippuric acid
deprotonated HMDB0013292, spectral sim. = 896, #4
Struc. sim. = 0.80
p-Methylhippuric acid
deprotonated HMDB0034250, spectral sim. = 693, #5
Struc. sim. = 0.65
Betamipron
deprotonated HMDB0060366, spectral sim. = 648, #6
Struc. sim. = 0.36
3-Carbamoyl-2-phenylpropionaldehyde
deprotonated HMDB0032388, spectral sim. = 631, #7
Struc. sim. = 0.38
Methyl n-acetylanthranilate
deprotonated HMDB0000859, spectral sim. = 447, #8
Struc. sim. = 0.44
Methyl hippurate


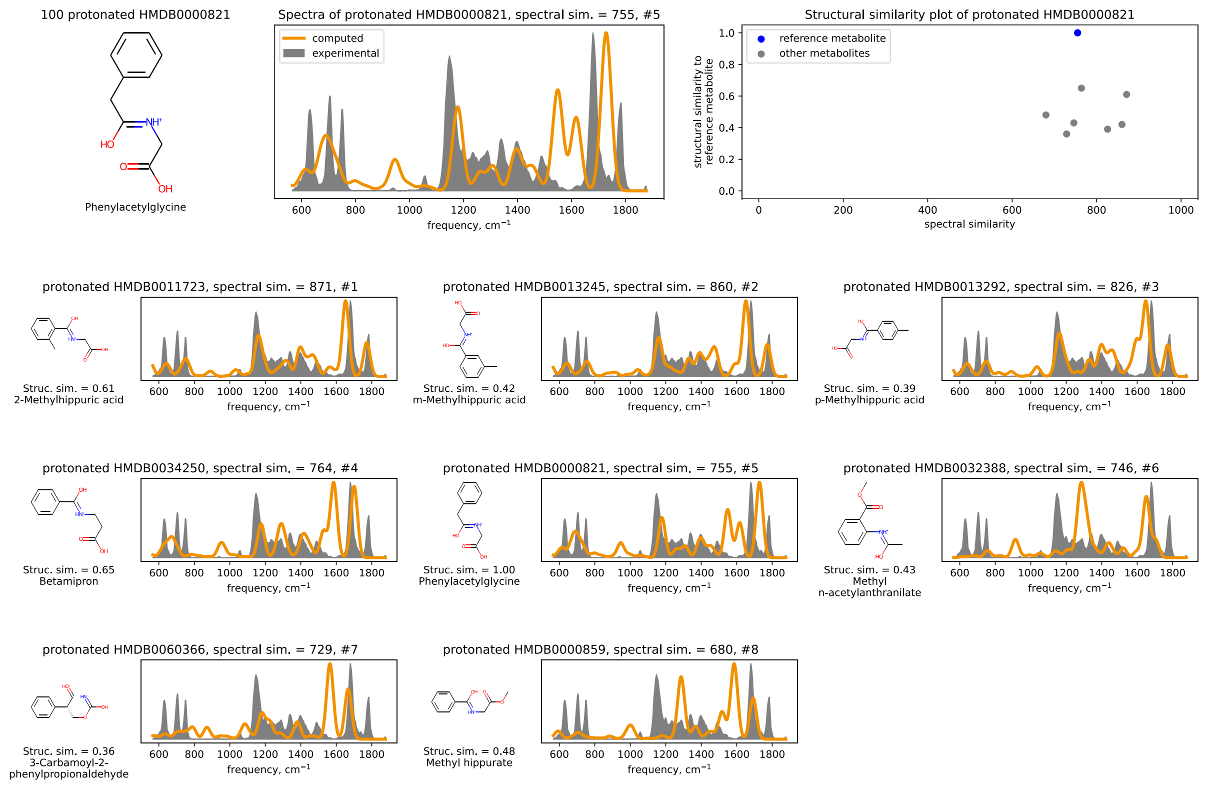

100 protonated HMDB0000821
Phenylacetylglycine
Spectra of protonated HMDB0000821, spectral sim. = 755, #5
computed
experimental
frequency, cm$^{-1}$
Structural similarity plot of protonated HMDB0000821
reference metabolite
other metabolites
structural similarity to reference metabolite
spectral similarity
protonated HMDB0011723, spectral sim. = 871, #1
Struc. sim. = 0.61
2-Methylhippuric acid
protonated HMDB0013245, spectral sim. = 860, #2
Struc. sim. = 0.42
m-Methylhippuric acid
protonated HMDB0013292, spectral sim. = 826, #3
Struc. sim. = 0.39
p-Methylhippuric acid
protonated HMDB0034250, spectral sim. = 764, #4
Struc. sim. = 0.65
Betamipron
protonated HMDB0000821, spectral sim. = 755, #5
Struc. sim. = 1.00
Phenylacetylglycine
protonated HMDB0032388, spectral sim. = 746, #6
Struc. sim. = 0.43
Methyl n-acetylanthranilate
protonated HMDB0060366, spectral sim. = 729, #7
Struc. sim. = 0.36
3-Carbamoyl-2-phenylpropionaldehyde
protonated HMDB0000859, spectral sim. = 680, #8
Struc. sim. = 0.48
Methyl hippurate

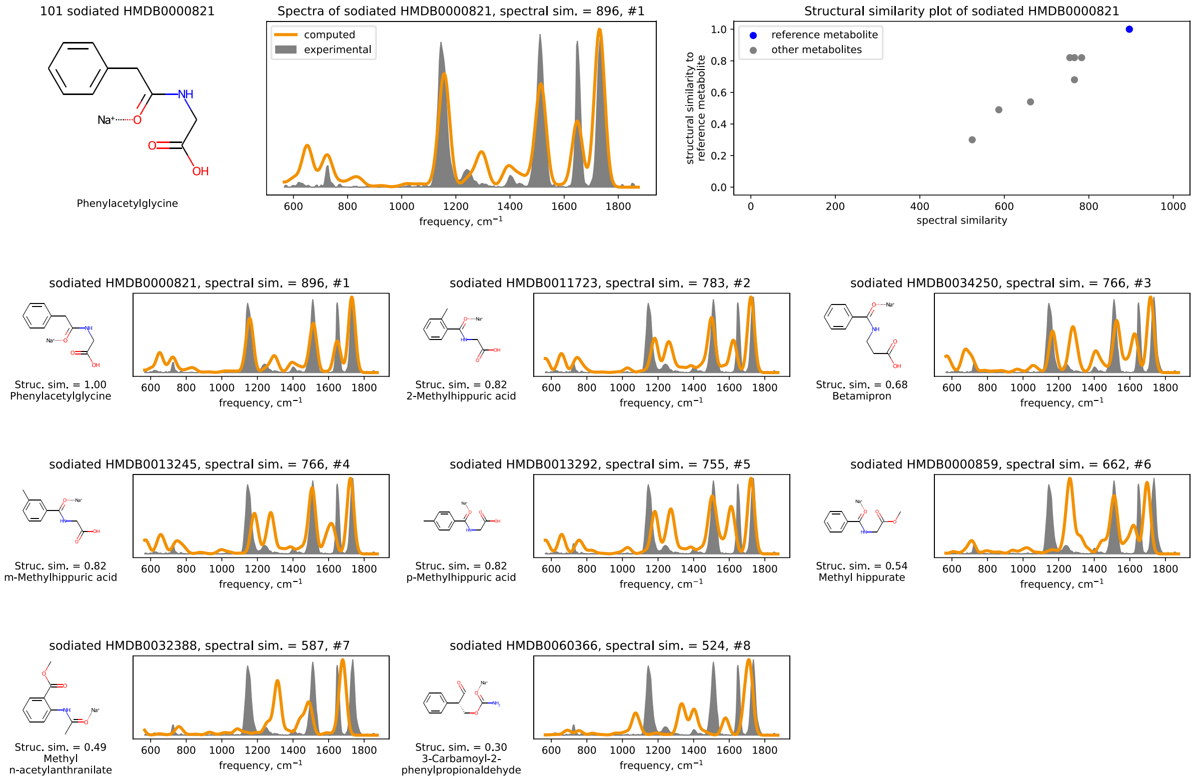

101 sodiated HMDB0000821
Phenylacetylglycine
Spectra of sodiated HMDB0000821, spectral sim. = 896, #1
computed
experimental
frequency, cm$^{-1}$
Structural similarity plot of sodiated HMDB0000821
reference metabolite
other metabolites
structural similarity to reference metabolite
spectral similarity
sodiated HMDB0000821, spectral sim. = 896, #1
Struc. sim. = 1.00
Phenylacetylglycine
sodiated HMDB0011723, spectral sim. = 783, #2
Struc. sim. = 0.82
2-Methylhippuric acid
sodiated HMDB0034250, spectral sim. = 766, #3
Struc. sim. = 0.68
Betamipron
sodiated HMDB0013245, spectral sim. = 766, #4
Struc. sim. = 0.82
m-Methylhippuric acid
sodiated HMDB0013292, spectral sim. = 755, #5
Struc. sim. = 0.82
p-Methylhippuric acid
sodiated HMDB0000859, spectral sim. = 662, #6
Struc. sim. = 0.54
Methyl hippurate
sodiated HMDB0032388, spectral sim. = 587, #7
Struc. sim. = 0.49
Methyl
n-acetylanthranilate
sodiated HMDB0060366, spectral sim. = 524, #8
Struc. sim. = 0.30
3-Carbamoyl-2-
phenylpropionaldehyde

102 deprotonated HMDB0000822

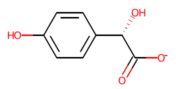


p-Hydroxymandelic acid

Spectra of deprotonated HMDB0000822, spectral sim. = 878, #1

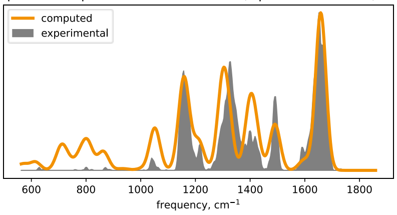


Structural similarity plot of deprotonated HMDB0000822

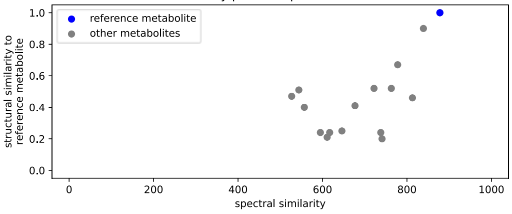


deprotonated HMDB0000822, spectral sim. = 878, #1

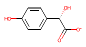

Struc. sim. = 1.00
p-Hydroxymandelic acid

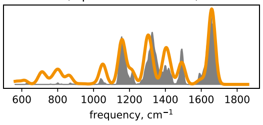


deprotonated HMDB0000750, spectral sim. = 839, #2

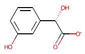

Struc. sim. = 0.90
3-Hydroxymandelic acid

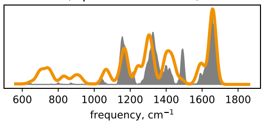


deprotonated HMDB0240257, spectral sim. = 813, #3

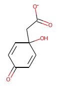

Struc. sim. = 0.46
Quinolacetic acid

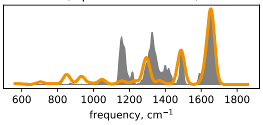


deprotonated HMDB0000130, spectral sim. = 778, #4

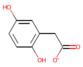

Struc. sim. = 0.67
Homogentisic acid

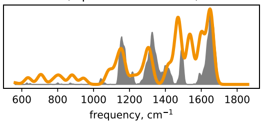


deprotonated HMDB0140902, spectral sim. = 763, #5

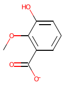

Struc. sim. = 0.52
3-hydroxy-2-methoxybenzoic acid

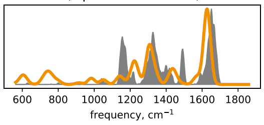


deprotonated HMDB0000484, spectral sim. = 741, #6

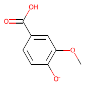

Struc. sim. = 0.20
Vanillic acid

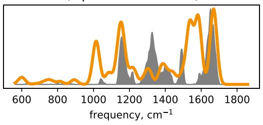


deprotonated HMDB0060003, spectral sim. = 738, #7

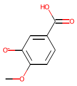

Struc. sim. = 0.24
Isovanillic acid

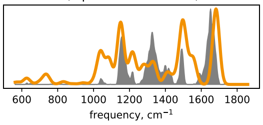


deprotonated HMDB0125536, spectral sim. = 722, #8

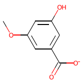

Struc. sim. = 0.52
3-hydroxy-5-methoxybenzoic acid

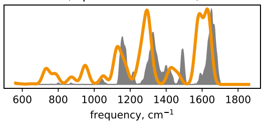


deprotonated HMDB0029644, spectral sim. = 677, #9

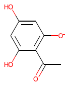

Struc. sim. = 0.41
2',4',6'-Trihydroxyacetophenone

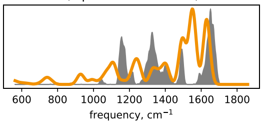


deprotonated HMDB0001868, spectral sim. = 646, #10

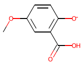

Struc. sim. = 0.25
5-Methoxysalicylic acid

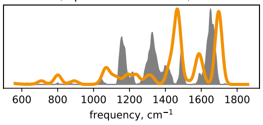


deprotonated HMDB0059713, spectral sim. = 617, #11

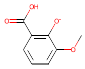

Struc. sim. = 0.24
3-Methoxysalicylic acid

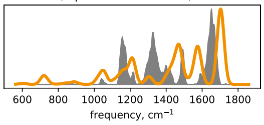


deprotonated HMDB0033129, spectral sim. = 611, #12

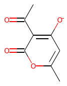

Struc. sim. = 0.21
3-Acetyl-4-hydroxy-6-methyl-2H-pyran-2-one

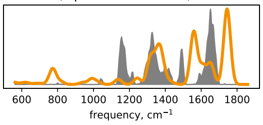


deprotonated HMDB0131176, spectral sim. = 595, #13

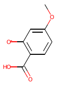

Struc. sim. = 0.24
2-hydroxy-4-methoxybenzoic acid

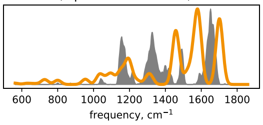


deprotonated HMDB0006242, spectral sim. = 557, #14

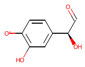

Struc. sim. = 0.40
3,4-Dihydroxymandelaldehyde

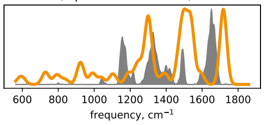


deprotonated HMDB0001336, spectral sim. = 544, #15

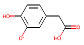

Struc. sim. = 0.51
3,4-Dihydroxybenzeneacetic acid

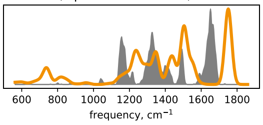


deprotonated HMDB0094709, spectral sim. = 527, #16

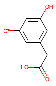

Struc. sim. = 0.47
3,5-Dihydroxyphenylacetic acid

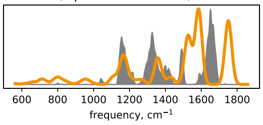

103 sodiated HMDB0000822

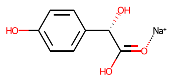


p-Hydroxymandelic acid

Spectra of sodiated HMDB0000822, spectral sim. = 850, #2

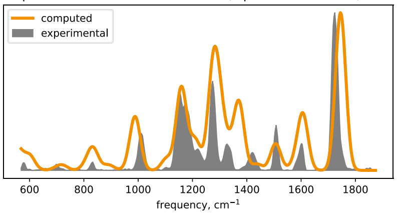


Structural similarity plot of sodiated HMDB0000822

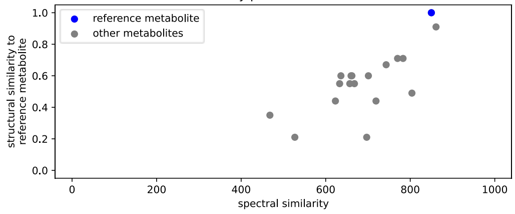


sodiated HMDB0000750, spectral sim. = 861, #1

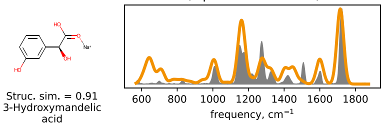

Struc. sim. = 0.91
3-Hydroxymandelic acid

sodiated HMDB0000822, spectral sim. = 850, #2

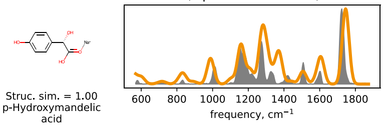

Struc. sim. = 1.00
p-Hydroxymandelic acid

sodiated HMDB0006242, spectral sim. = 804, #3

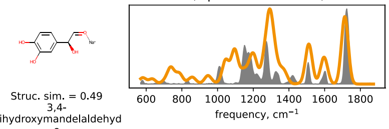

Struc. sim. = 0.49
3,4-Dihydroxymandelaldehyde

sodiated HMDB0001336, spectral sim. = 783, #4

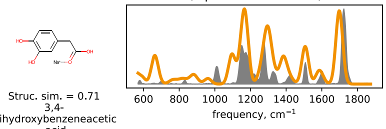

Struc. sim. = 0.71
3,4-Dihydroxybenzeneacetic acid

sodiated HMDB0000130, spectral sim. = 770, #5

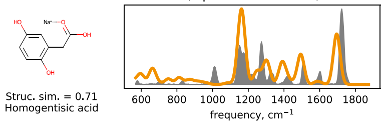

Struc. sim. = 0.71
Homogentisic acid

sodiated HMDB0094709, spectral sim. = 743, #6

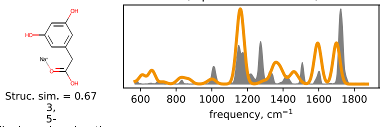

Struc. sim. = 0.67
3,5-Dihydroxyphenylacetic acid

sodiated HMDB0240257, spectral sim. = 719, #7

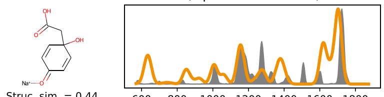

Struc. sim. = 0.44
Quinolacetic acid

sodiated HMDB0001868, spectral sim. = 701, #8

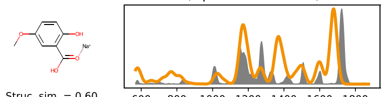

Struc. sim. = 0.60
5-Methoxysalicylic acid

sodiated HMDB0034335, spectral sim. = 697, #9

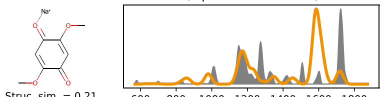

Struc. sim. = 0.21
Thermophillin

sodiated HMDB0060003, spectral sim. = 668, #10

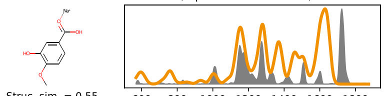

Struc. sim. = 0.55
Isovanillic acid

sodiated HMDB0000484, spectral sim. = 662, #11

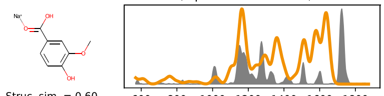

Struc. sim. = 0.60
Vanillic acid

sodiated HMDB0140902, spectral sim. = 660, #12

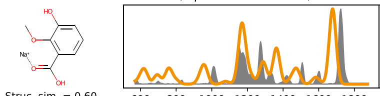

Struc. sim. = 0.60
3-hydroxy-2-methoxybenzoic acid

sodiated HMDB0131176, spectral sim. = 657, #13

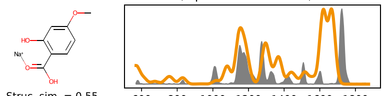

Struc. sim. = 0.55
2-hydroxy-4-methoxybenzoic acid

sodiated HMDB0125536, spectral sim. = 636, #14

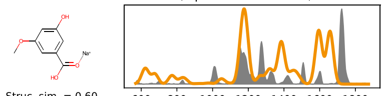

Struc. sim. = 0.60
3-hydroxy-5-methoxybenzoic acid

sodiated HMDB0059713, spectral sim. = 633, #15

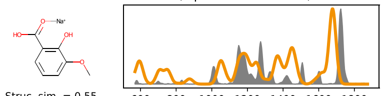

Struc. sim. = 0.55
3-Methoxysalicylic acid

sodiated HMDB0029644, spectral sim. = 623, #16

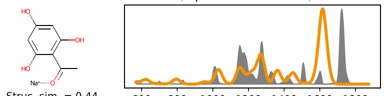

Struc. sim. = 0.44
2',4',6'-Trihydroxyacetophenone

sodiated HMDB0029673, spectral sim. = 527, #17

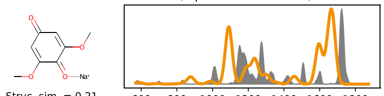

Struc. sim. = 0.21
2,6-Dimethoxy-1,4-benzoquinone

sodiated HMDB0033129, spectral sim. = 468, #18

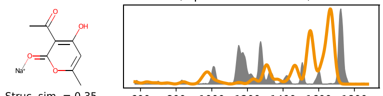

Struc. sim. = 0.35
3-Acetyl-4-hydroxy-6-methyl-2H-pyran-2-one

104 deprotonated HMDB0000828

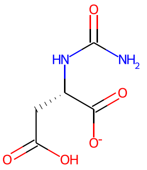


Ureidosuccinic acid

Spectra of deprotonated HMDB0000828, spectral sim. = 854, #1

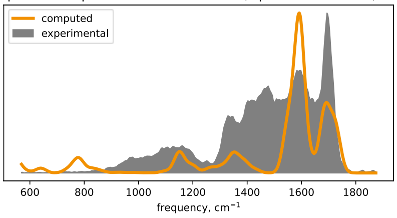


Structural similarity plot of deprotonated HMDB0000828

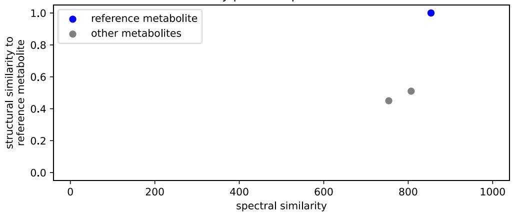


deprotonated HMDB0000828, spectral sim. = 854, #1

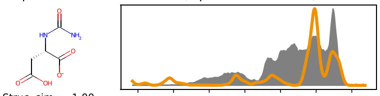

Struc. sim. = 1.00
Ureidosuccinic acid

deprotonated HMDB0029402, spectral sim. = 807, #2

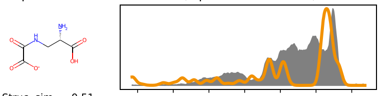

Struc. sim. = 0.51
L-2-Amino-3-(oxalylamino)propanoic acid

deprotonated HMDB0038668, spectral sim. = 754, #3

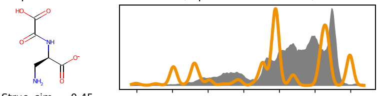

Struc. sim. = 0.45
L-3-Amino-2-(oxalylamino)propanoic acid

105 protonated HMDB0000828 | Spectra of protonated HMDB0000828, spectral sim. = 946, #1 | Structural similarity plot of protonated HMDB0000828

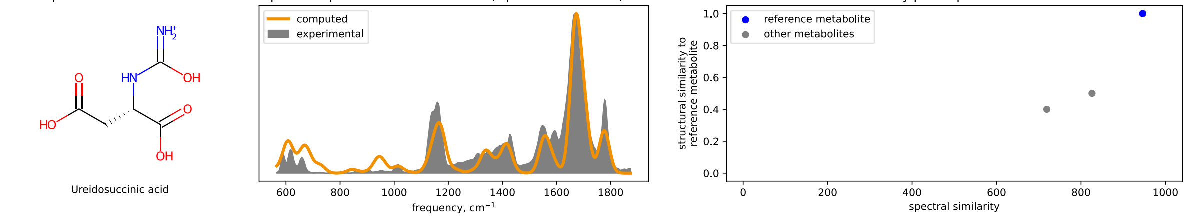


protonated HMDB0000828, spectral sim. = 946, #1 | protonated HMDB0029402, spectral sim. = 826, #2 | protonated HMDB0038668, spectral sim. = 719, #3

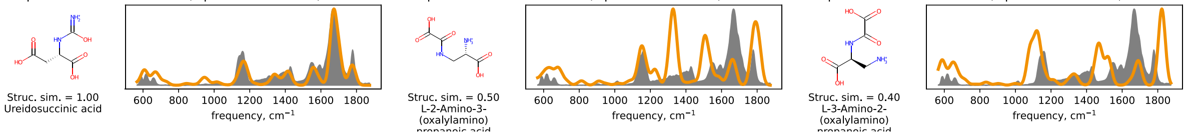


106 sodiated HMDB0000828 | Spectra of sodiated HMDB0000828, spectral sim. = 884, #2 | Structural similarity plot of sodiated HMDB0000828

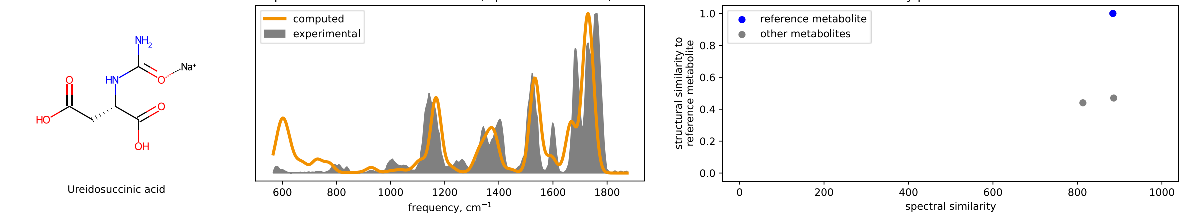


sodiated HMDB0029402, spectral sim. = 886, #1 | sodiated HMDB0000828, spectral sim. = 884, #2 | sodiated HMDB0038668, spectral sim. = 813, #3

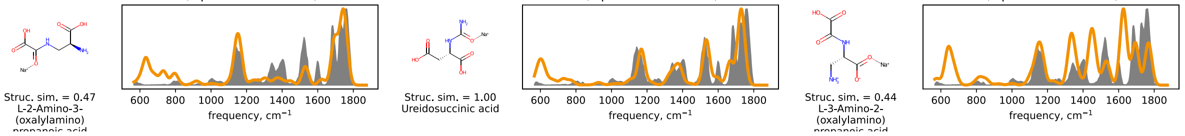


107 deprotonated HMDB0000842 | Spectra of deprotonated HMDB0000842, spectral sim. = 889, #1 | Structural similarity plot of deprotonated HMDB0000842

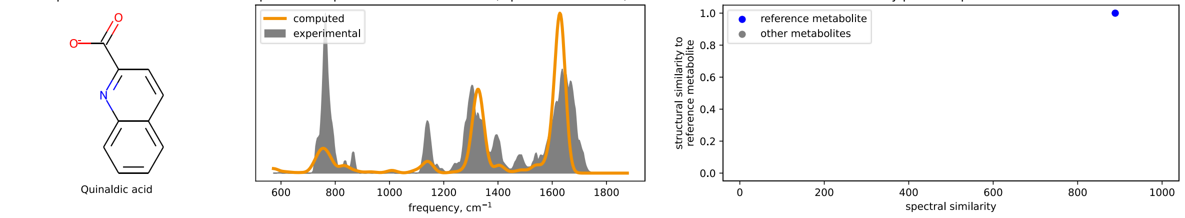


deprotonated HMDB0000842, spectral sim. = 889, #1

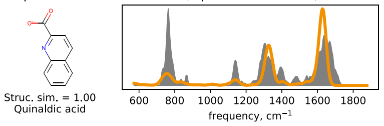


108 protonated HMDB0000842 | Spectra of protonated HMDB0000842, spectral sim. = 900, #1 | Structural similarity plot of protonated HMDB0000842

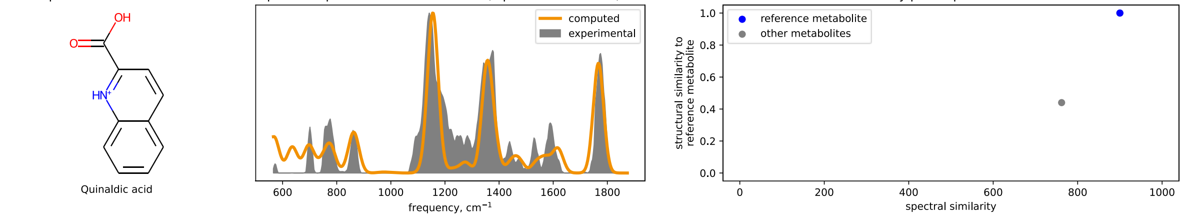


protonated HMDB0000842, spectral sim. = 900, #1 | protonated HMDB0062188, spectral sim. = 762, #2

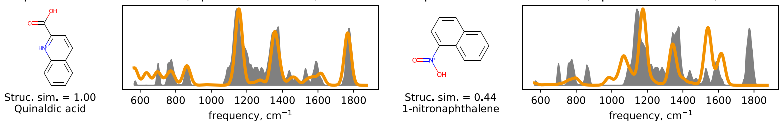

109 sodiated HMDB0000842

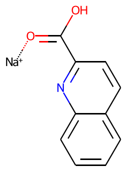


Quinaldic acid

Spectra of sodiated HMDB0000842, spectral sim. = 818, #1

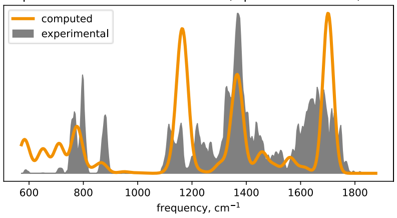


Structural similarity plot of sodiated HMDB0000842

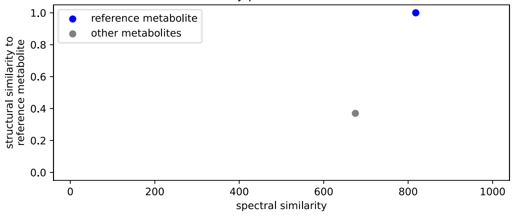


sodiated HMDB0000842, spectral sim. = 818, #1

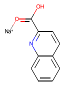

Struc. sim. = 1.00
Quinaldic acid

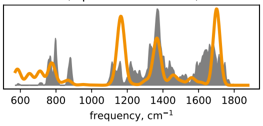


sodiated HMDB0062188, spectral sim. = 675, #2

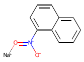

Struc. sim. = 0.37
1-nitronaphthalene

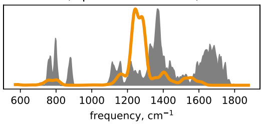


110 deprotonated HMDB0000860

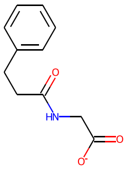


Phenylpropionylglycine

Spectra of deprotonated HMDB0000860, spectral sim. = 909, #1

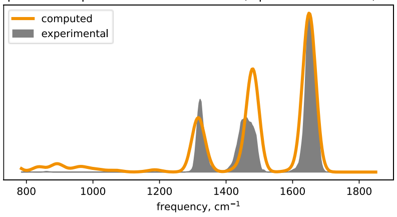


Structural similarity plot of deprotonated HMDB0000860

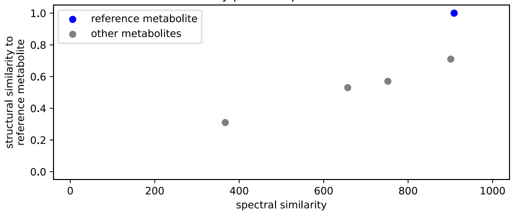


deprotonated HMDB0000860, spectral sim. = 909, #1

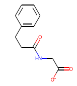

Struc. sim. = 1.00
Phenylpropionylglycine

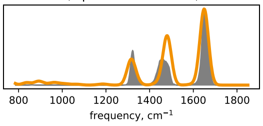


deprotonated HMDB0000512, spectral sim. = 901, #2

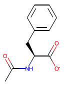

Struc. sim. = 0.71
N-Acetyl-L-phenylalanine

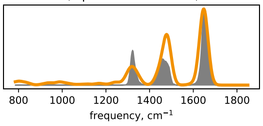


deprotonated HMDB0060608, spectral sim. = 752, #3

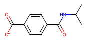

Struc. sim. = 0.57
N-isopropylterephthalamic acid

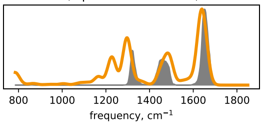


deprotonated HMDB0002042, spectral sim. = 657, #4

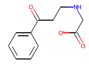

Struc. sim. = 0.53
3-Phenylpropionylglycine

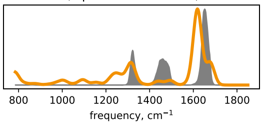


deprotonated HMDB0061682, spectral sim. = 367, #5

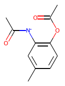

Struc. sim. = 0.31
2-Acetamido-4-methylphenyl acetate

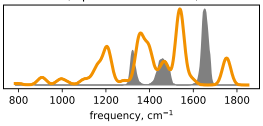


111 protonated HMDB0000860

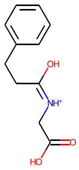


Phenylpropionylglycine

Spectra of protonated HMDB0000860, spectral sim. = 874, #1

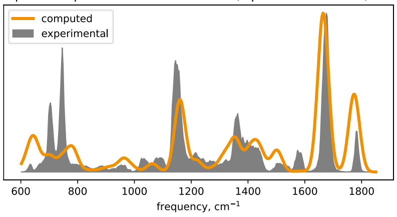


Structural similarity plot of protonated HMDB0000860

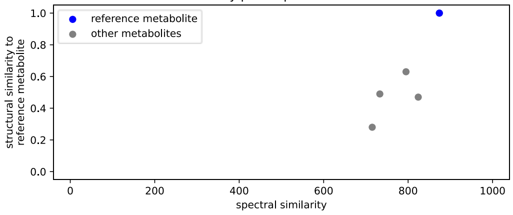


protonated HMDB0000860, spectral sim. = 874, #1

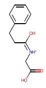

Struc. sim. = 1.00
Phenylpropionylglycine

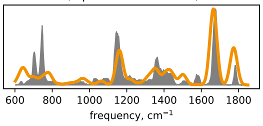


protonated HMDB0060608, spectral sim. = 824, #2

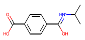

Struc. sim. = 0.47
N-isopropylterephthalamic acid

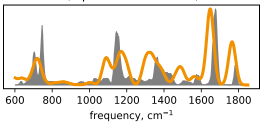


protonated HMDB0002042, spectral sim. = 795, #3

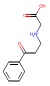

Struc. sim. = 0.63
3-Phenylpropionylglycine

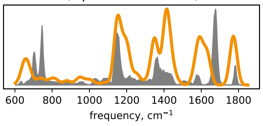


protonated HMDB0000512, spectral sim. = 733, #4

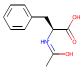

Struc. sim. = 0.49
N-Acetyl-L-phenylalanine

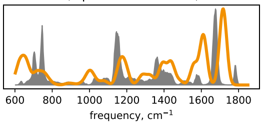


protonated HMDB0061682, spectral sim. = 715, #5

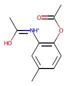

Struc. sim. = 0.28
2-Acetamido-4-methylphenyl acetate

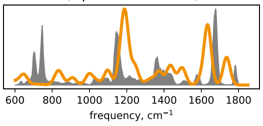

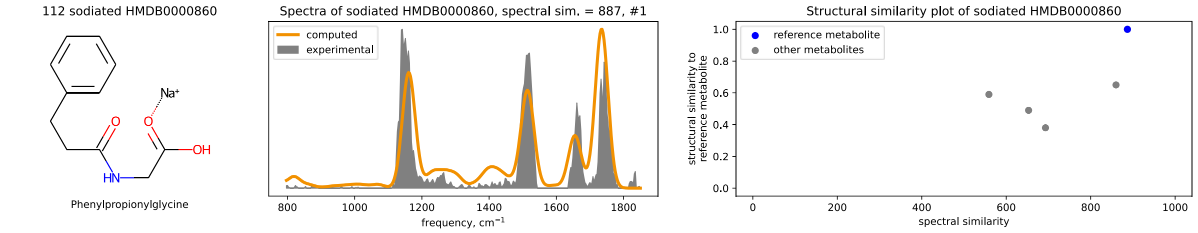

112 sodiated HMDB0000860
Na⁺
O
O
OH
HN
Phenylpropionylglycine
Spectra of sodiated HMDB0000860, spectral sim. = 887, #1
computed
experimental
frequency, cm⁻¹
Structural similarity plot of sodiated HMDB0000860
reference metabolite
other metabolites
structural similarity to reference metabolite
spectral similarity


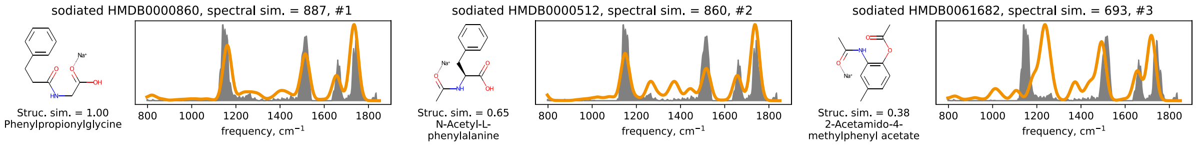

sodiated HMDB0000860, spectral sim. = 887, #1
Struc. sim. = 1.00
Phenylpropionylglycine
frequency, cm⁻¹
sodiated HMDB0000512, spectral sim. = 860, #2
Struc. sim. = 0.65
N-Acetyl-L-phenylalanine
frequency, cm⁻¹
sodiated HMDB0061682, spectral sim. = 693, #3
Struc. sim. = 0.38
2-Acetamido-4-methylphenyl acetate
frequency, cm⁻¹


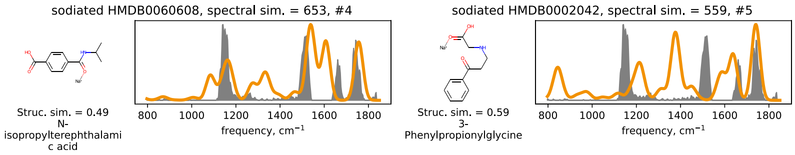

sodiated HMDB0060608, spectral sim. = 653, #4
Struc. sim. = 0.49
N-isopropylterephthalamic acid
frequency, cm⁻¹
sodiated HMDB0002042, spectral sim. = 559, #5
Struc. sim. = 0.59
3-Phenylpropionylglycine
frequency, cm⁻¹


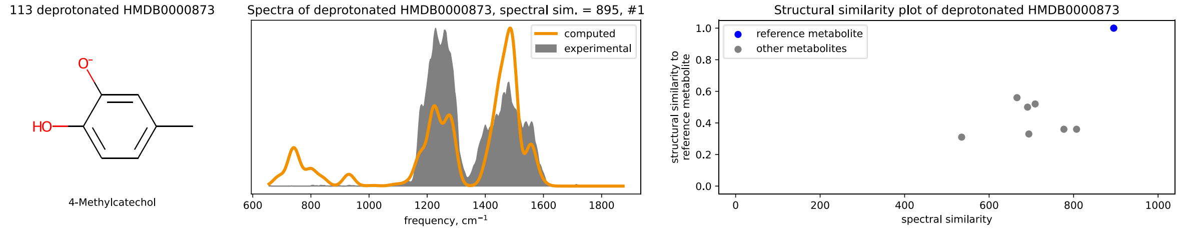

113 deprotonated HMDB0000873
O⁻
HO
4-Methylcatechol
Spectra of deprotonated HMDB0000873, spectral sim. = 895, #1
computed
experimental
frequency, cm⁻¹
Structural similarity plot of deprotonated HMDB0000873
reference metabolite
other metabolites
structural similarity to reference metabolite
spectral similarity


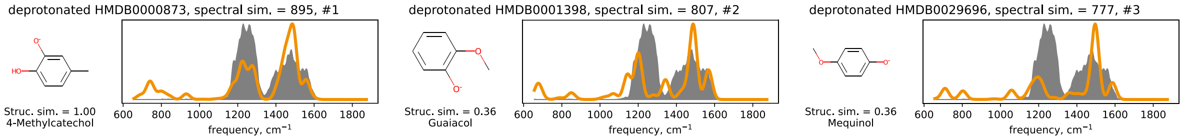

deprotonated HMDB0000873, spectral sim. = 895, #1
Struc. sim. = 1.00
4-Methylcatechol
frequency, cm⁻¹
deprotonated HMDB0001398, spectral sim. = 807, #2
Struc. sim. = 0.36
Guaiacol
frequency, cm⁻¹
deprotonated HMDB0029696, spectral sim. = 777, #3
Struc. sim. = 0.36
Mequinol
frequency, cm⁻¹


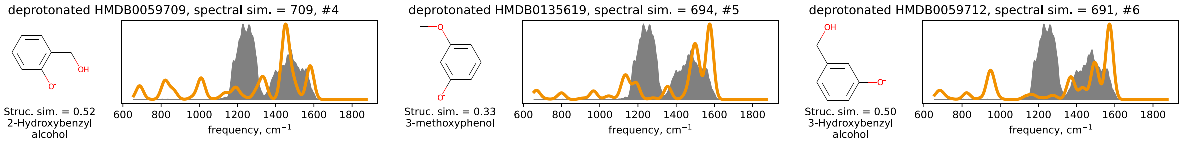

deprotonated HMDB0059709, spectral sim. = 709, #4
Struc. sim. = 0.52
2-Hydroxybenzyl alcohol
frequency, cm⁻¹
deprotonated HMDB0135619, spectral sim. = 694, #5
Struc. sim. = 0.33
3-methoxyphenol
frequency, cm⁻¹
deprotonated HMDB0059712, spectral sim. = 691, #6
Struc. sim. = 0.50
3-Hydroxybenzyl alcohol
frequency, cm⁻¹


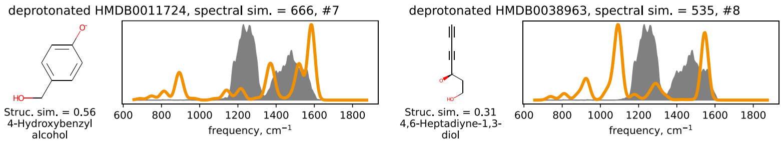

deprotonated HMDB0011724, spectral sim. = 666, #7
Struc. sim. = 0.56
4-Hydroxybenzyl alcohol
frequency, cm⁻¹
deprotonated HMDB0038963, spectral sim. = 535, #8
Struc. sim. = 0.31
4,6-Heptadiyne-1,3-diol
frequency, cm⁻¹

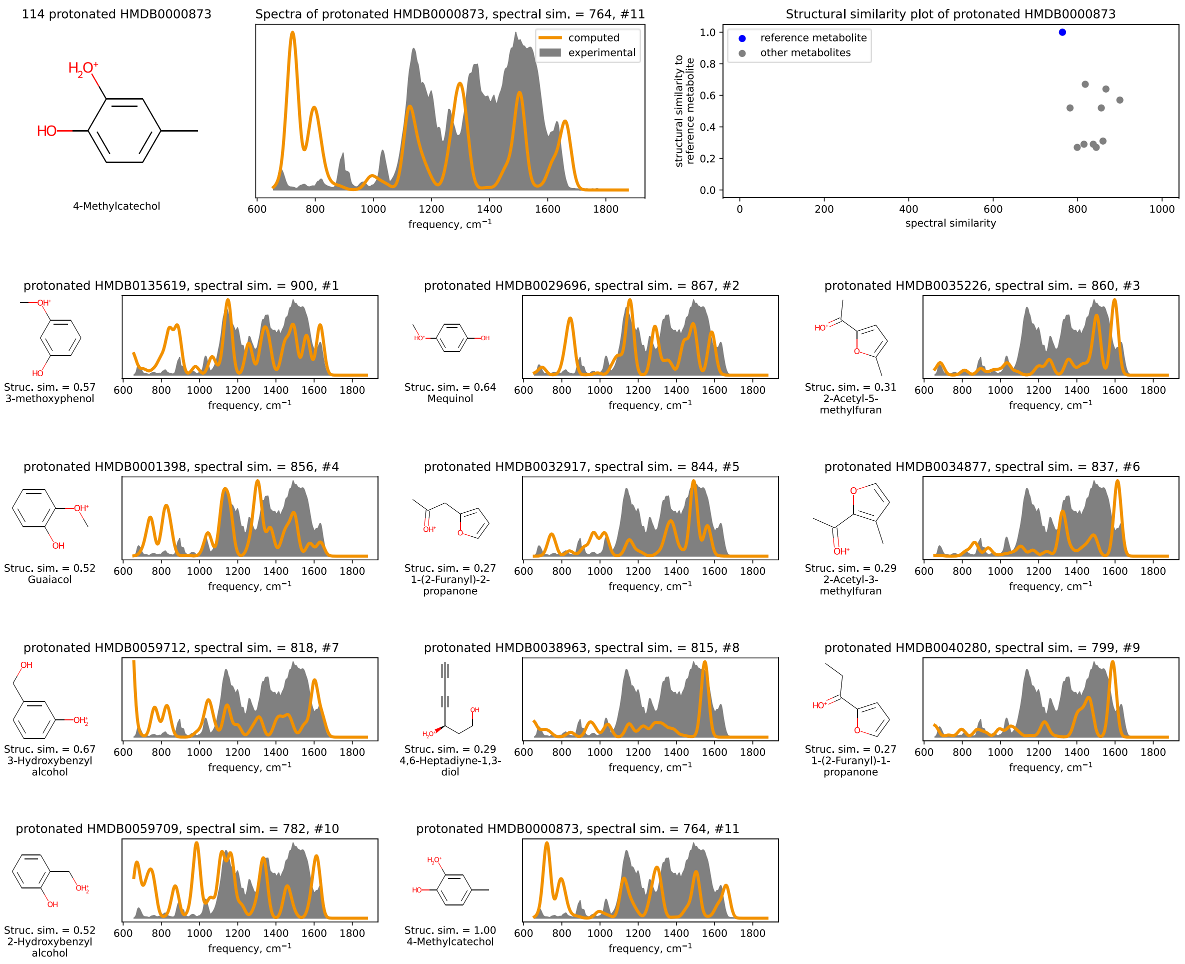

114 protonated HMDB0000873
4-Methylcatechol
Spectra of protonated HMDB0000873, spectral sim. = 764, #11
computed
experimental
frequency, cm⁻¹
Structural similarity plot of protonated HMDB0000873
reference metabolite
other metabolites
structural similarity to reference metabolite
spectral similarity
protonated HMDB0135619, spectral sim. = 900, #1
Struc. sim. = 0.57
3-methoxyphenol
protonated HMDB0029696, spectral sim. = 867, #2
Struc. sim. = 0.64
Mequinol
protonated HMDB0035226, spectral sim. = 860, #3
Struc. sim. = 0.31
2-Acetyl-5-methylfuran
protonated HMDB0001398, spectral sim. = 856, #4
Struc. sim. = 0.52
Guaiacol
protonated HMDB0032917, spectral sim. = 844, #5
Struc. sim. = 0.27
1-(2-Furanyl)-2-propanone
protonated HMDB0034877, spectral sim. = 837, #6
Struc. sim. = 0.29
2-Acetyl-3-methylfuran
protonated HMDB0059712, spectral sim. = 818, #7
Struc. sim. = 0.67
3-Hydroxybenzyl alcohol
protonated HMDB0038963, spectral sim. = 815, #8
Struc. sim. = 0.29
4,6-Heptadiyne-1,3-diol
protonated HMDB0040280, spectral sim. = 799, #9
Struc. sim. = 0.27
1-(2-Furanyl)-1-propanone
protonated HMDB0059709, spectral sim. = 782, #10
Struc. sim. = 0.52
2-Hydroxybenzyl alcohol
protonated HMDB0000873, spectral sim. = 764, #11
Struc. sim. = 1.00
4-Methylcatechol

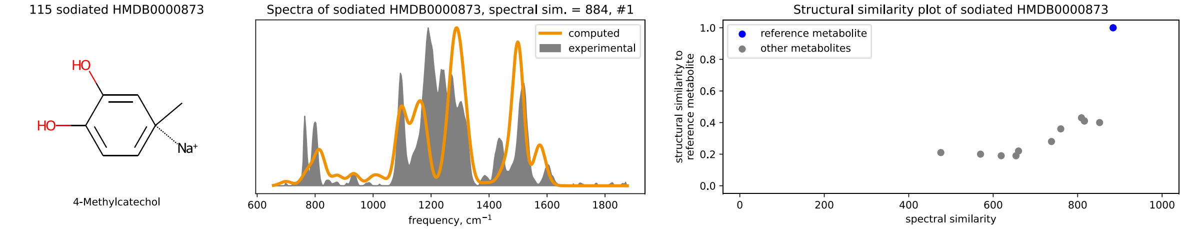
115 sodiated HMDB0000873
4-Methylcatechol
Spectra of sodiated HMDB0000873, spectral sim. = 884, #1
computed
experimental
frequency, cm⁻¹
Structural similarity plot of sodiated HMDB0000873
reference metabolite
other metabolites
structural similarity to reference metabolite
spectral similarity

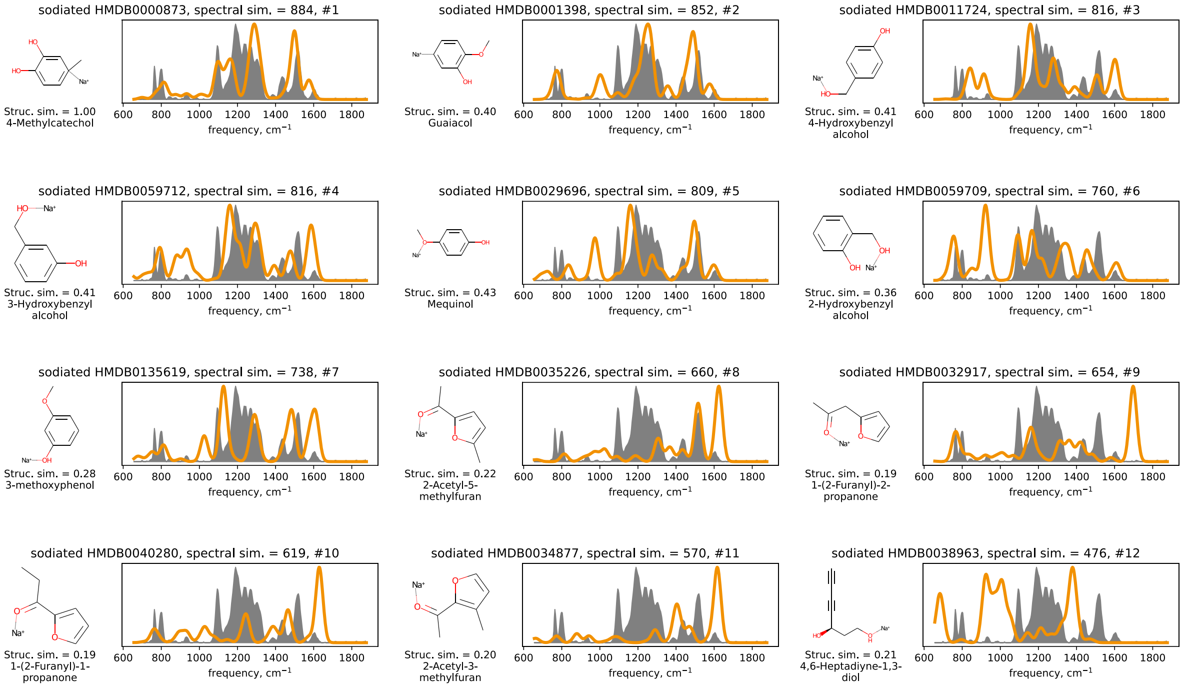
sodiated HMDB0000873, spectral sim. = 884, #1
Struc. sim. = 1.00
4-Methylcatechol
sodiated HMDB0001398, spectral sim. = 852, #2
Struc. sim. = 0.40
Guaiacol
sodiated HMDB0011724, spectral sim. = 816, #3
Struc. sim. = 0.41
4-Hydroxybenzyl alcohol
sodiated HMDB0059712, spectral sim. = 816, #4
Struc. sim. = 0.41
3-Hydroxybenzyl alcohol
sodiated HMDB0029696, spectral sim. = 809, #5
Struc. sim. = 0.43
Mequinol
sodiated HMDB0059709, spectral sim. = 760, #6
Struc. sim. = 0.36
2-Hydroxybenzyl alcohol
sodiated HMDB0135619, spectral sim. = 738, #7
Struc. sim. = 0.28
3-methoxyphenol
sodiated HMDB0035226, spectral sim. = 660, #8
Struc. sim. = 0.22
2-Acetyl-5-methylfuran
sodiated HMDB0032917, spectral sim. = 654, #9
Struc. sim. = 0.19
1-(2-Furanyl)-2-propanone
sodiated HMDB0040280, spectral sim. = 619, #10
Struc. sim. = 0.19
1-(2-Furanyl)-1-propanone
sodiated HMDB0034877, spectral sim. = 570, #11
Struc. sim. = 0.20
2-Acetyl-3-methylfuran
sodiated HMDB0038963, spectral sim. = 476, #12
Struc. sim. = 0.21
4,6-Heptadiyne-1,3-diol
frequency, cm⁻¹

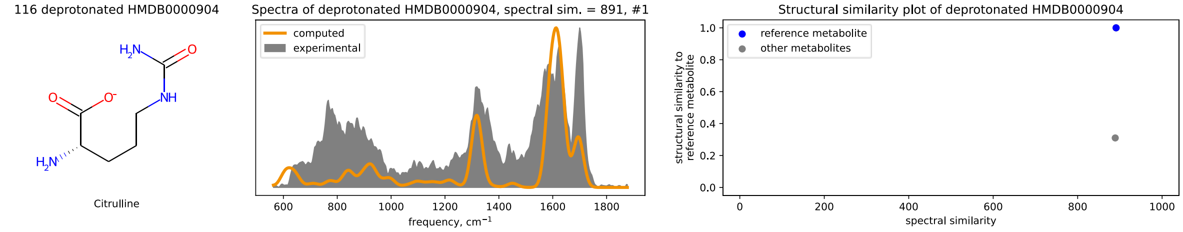
116 deprotonated HMDB0000904
Citrulline
Spectra of deprotonated HMDB0000904, spectral sim. = 891, #1
computed
experimental
frequency, cm⁻¹
Structural similarity plot of deprotonated HMDB0000904
reference metabolite
other metabolites
structural similarity to reference metabolite
spectral similarity

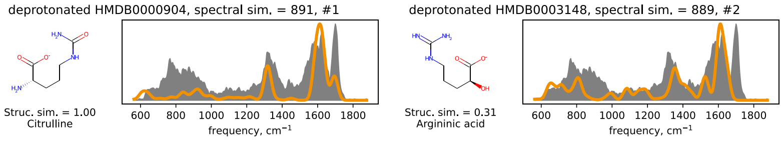
deprotonated HMDB0000904, spectral sim. = 891, #1
Struc. sim. = 1.00
Citrulline
deprotonated HMDB0003148, spectral sim. = 889, #2
Struc. sim. = 0.31
Argininic acid
frequency, cm⁻¹

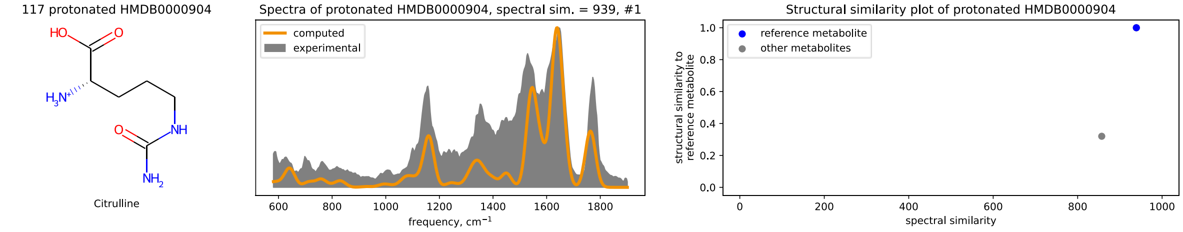
117 protonated HMDB0000904
Citrulline
Spectra of protonated HMDB0000904, spectral sim. = 939, #1
computed
experimental
frequency, cm⁻¹
Structural similarity plot of protonated HMDB0000904
reference metabolite
other metabolites
structural similarity to reference metabolite
spectral similarity

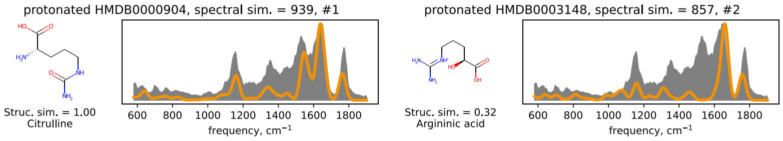
protonated HMDB0000904, spectral sim. = 939, #1
Struc. sim. = 1.00
Citrulline
protonated HMDB0003148, spectral sim. = 857, #2
Struc. sim. = 0.32
Argininic acid
frequency, cm⁻¹

118 sodiated HMDB0000904

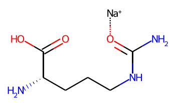


Citrulline

Spectra of sodiated HMDB0000904, spectral sim. = 916, #1

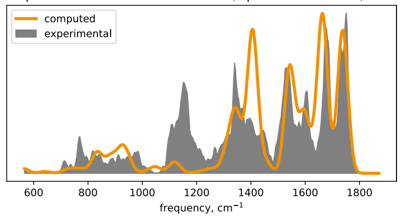


Structural similarity plot of sodiated HMDB0000904

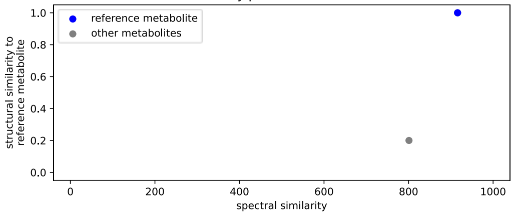


sodiated HMDB0000904, spectral sim. = 916, #1

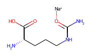

Struc. sim. = 1.00
Citrulline

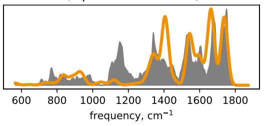


sodiated HMDB0003148, spectral sim. = 801, #2

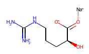

Struc. sim. = 0.20
Argininic acid

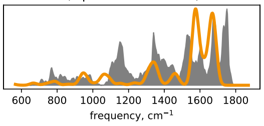


119 deprotonated HMDB0000929

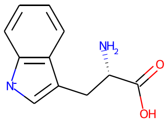


L-Tryptophan

Spectra of deprotonated HMDB0000929, spectral sim. = 768, #2

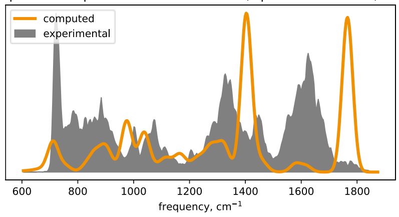


Structural similarity plot of deprotonated HMDB0000929

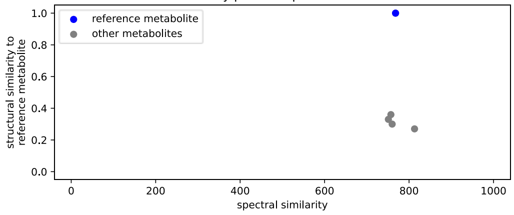


deprotonated HMDB0060878, spectral sim. = 813, #1

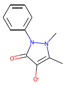

Struc. sim. = 0.27
4-Hydroxyantipyrine

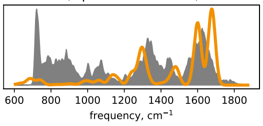


deprotonated HMDB0000929, spectral sim. = 768, #2

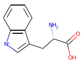

Struc. sim. = 1.00
L-Tryptophan

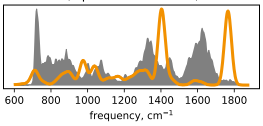


deprotonated HMDB0060967, spectral sim. = 760, #3

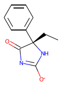

Struc. sim. = 0.30
S-nirvanol

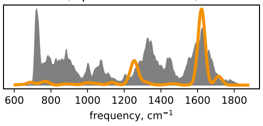


deprotonated HMDB0060533, spectral sim. = 757, #4

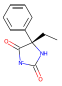

Struc. sim. = 0.36
Nirvanol

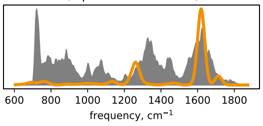


deprotonated HMDB0014892, spectral sim. = 751, #5

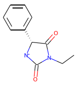

Struc. sim. = 0.33
Ethotoin

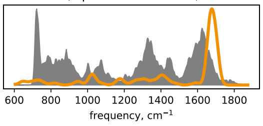


120 protonated HMDB0000929

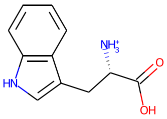


L-Tryptophan

Spectra of protonated HMDB0000929, spectral sim. = 938, #1

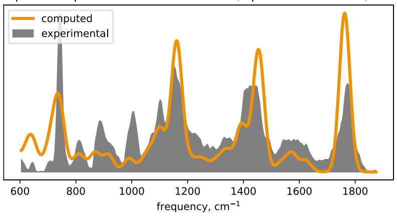


Structural similarity plot of protonated HMDB0000929

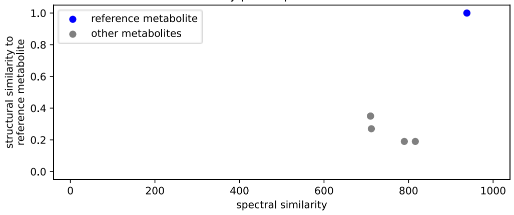


protonated HMDB0000929, spectral sim. = 938, #1

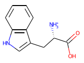

Struc. sim. = 1.00
L-Tryptophan

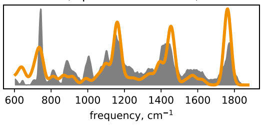


protonated HMDB0060878, spectral sim. = 816, #2

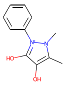

Struc. sim. = 0.19
4-Hydroxyantipyrine

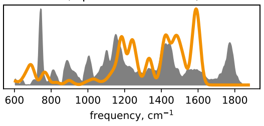


protonated HMDB0013840, spectral sim. = 790, #3

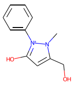

Struc. sim. = 0.19
3-
Hydroxymethylantipyrin
e

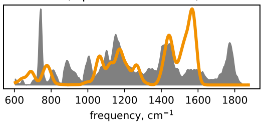


protonated HMDB0014892, spectral sim. = 712, #4

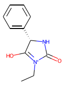

Struc. sim. = 0.27
Ethotoin

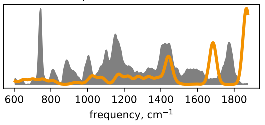


protonated HMDB0060533, spectral sim. = 710, #5

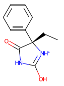

Struc. sim. = 0.35
Nirvanol

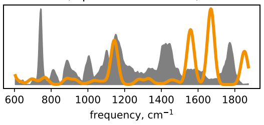


protonated HMDB0060967, spectral sim. = 710, #6

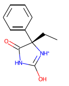

Struc. sim. = 0.35
S-nirvanol

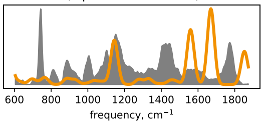

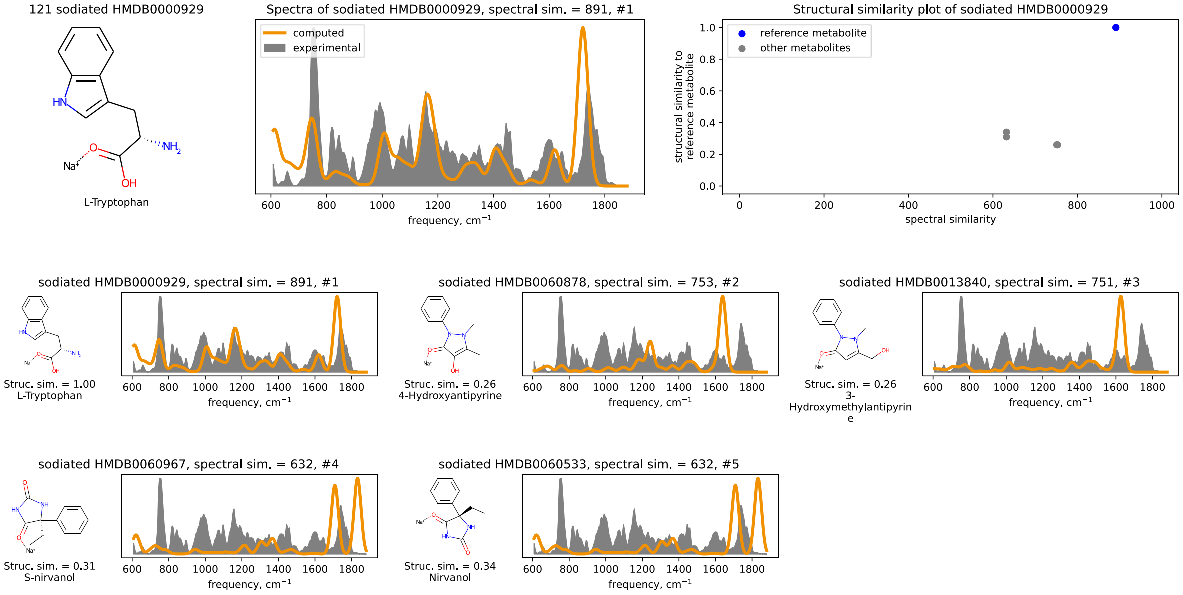
121 sodiated HMDB0000929
L-Tryptophan
Spectra of sodiated HMDB0000929, spectral sim. = 891, #1
computed
experimental
frequency, cm−1
Structural similarity plot of sodiated HMDB0000929
reference metabolite
other metabolites
structural similarity to reference metabolite
spectral similarity
sodiated HMDB0000929, spectral sim. = 891, #1
Struc. sim. = 1.00
L-Tryptophan
sodiated HMDB0060878, spectral sim. = 753, #2
Struc. sim. = 0.26
4-Hydroxyantipyrine
sodiated HMDB0013840, spectral sim. = 751, #3
Struc. sim. = 0.26
3-Hydroxymethylantipyrine
sodiated HMDB0060967, spectral sim. = 632, #4
Struc. sim. = 0.31
S-nirvanol
sodiated HMDB0060533, spectral sim. = 632, #5
Struc. sim. = 0.34
Nirvanol

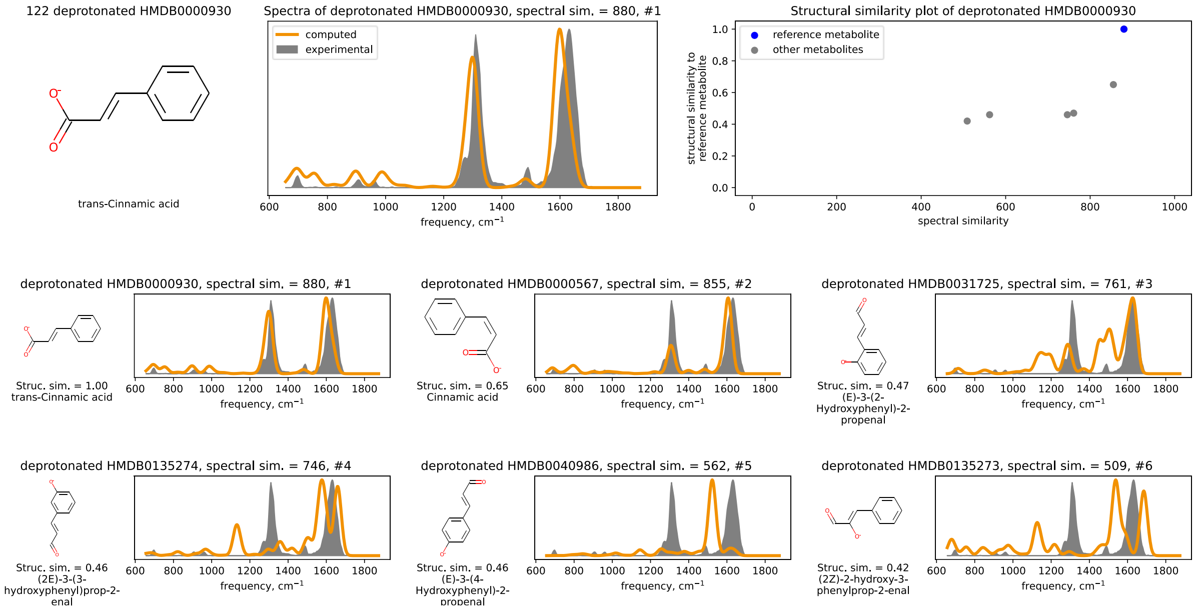
122 deprotonated HMDB0000930
trans-Cinnamic acid
Spectra of deprotonated HMDB0000930, spectral sim. = 880, #1
computed
experimental
frequency, cm−1
Structural similarity plot of deprotonated HMDB0000930
reference metabolite
other metabolites
structural similarity to reference metabolite
spectral similarity
deprotonated HMDB0000930, spectral sim. = 880, #1
Struc. sim. = 1.00
trans-Cinnamic acid
deprotonated HMDB0000567, spectral sim. = 855, #2
Struc. sim. = 0.65
Cinnamic acid
deprotonated HMDB0031725, spectral sim. = 761, #3
Struc. sim. = 0.47
(E)-3-(2-Hydroxyphenyl)-2-propenal
deprotonated HMDB0135274, spectral sim. = 746, #4
Struc. sim. = 0.46
(2E)-3-(3-hydroxyphenyl)prop-2-enal
deprotonated HMDB0040986, spectral sim. = 562, #5
Struc. sim. = 0.46
(E)-3-(4-Hydroxyphenyl)-2-propenal
deprotonated HMDB0135273, spectral sim. = 509, #6
Struc. sim. = 0.42
(2Z)-2-hydroxy-3-phenylprop-2-enal

123 sodiated HMDB0000930

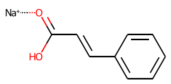


trans-Cinnamic acid

Spectra of sodiated HMDB0000930, spectral sim. = 830, #4

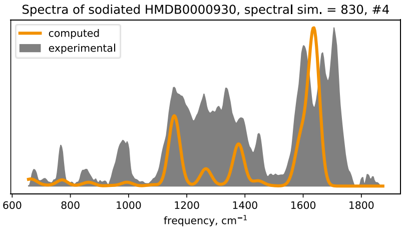


Structural similarity plot of sodiated HMDB0000930

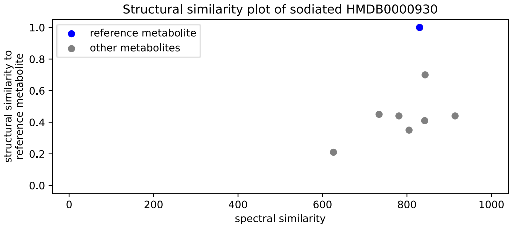


sodiated HMDB0035243, spectral sim. = 914, #1

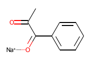

Struc. sim. = 0.44
1-Phenyl-1,2-propanedione

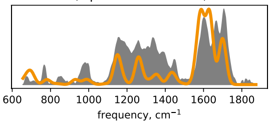


sodiated HMDB0000567, spectral sim. = 843, #2

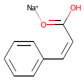

Struc. sim. = 0.70
Cinnamic acid

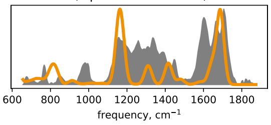


sodiated HMDB0135273, spectral sim. = 842, #3

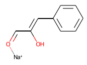

Struc. sim. = 0.41
(2Z)-2-hydroxy-3-phenylprop-2-enal

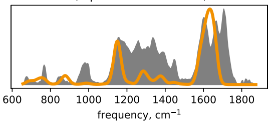


sodiated HMDB0000930, spectral sim. = 830, #4

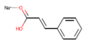

Struc. sim. = 1.00
trans-Cinnamic acid

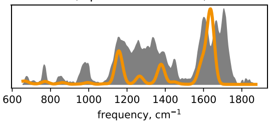


sodiated HMDB0036626, spectral sim. = 805, #5

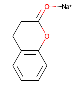

Struc. sim. = 0.35
3,4-Dihydro-2H-1-benzopyran-2-one

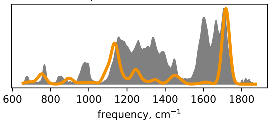


sodiated HMDB0040986, spectral sim. = 781, #6

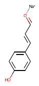

Struc. sim. = 0.44
(E)-3-(4-Hydroxyphenyl)-2-propenal

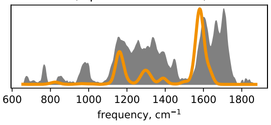


sodiated HMDB0135274, spectral sim. = 734, #7

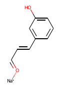

Struc. sim. = 0.45
(2E)-3-(3-hydroxyphenyl)prop-2-enal

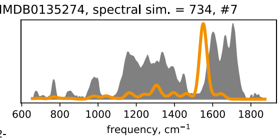


sodiated HMDB0032947, spectral sim. = 626, #8

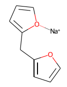

Struc. sim. = 0.21
Di-2-furanylmethane

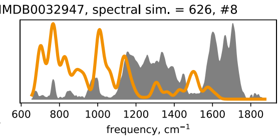


124 protonated HMDB0000959

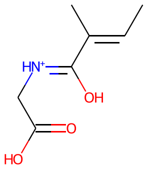


Tiglylglycine

Spectra of protonated HMDB0000959, spectral sim. = 858, #3

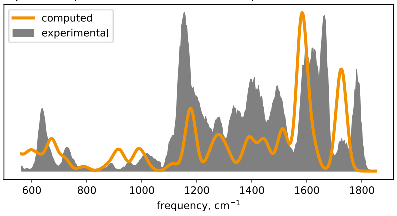


Structural similarity plot of protonated HMDB0000959

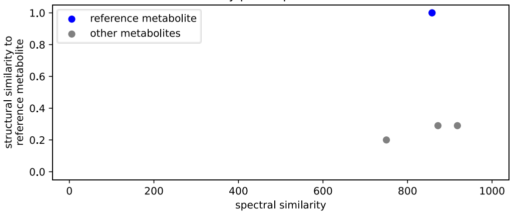


protonated HMDB0000459, spectral sim. = 918, #1

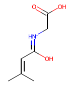

Struc. sim. = 0.29
3-Methylcrotonylglycine

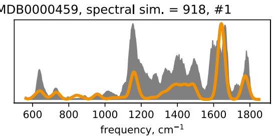


protonated HMDB0094701, spectral sim. = 872, #2

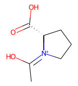

Struc. sim. = 0.29
N-Acetylproline

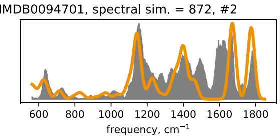


protonated HMDB0000959, spectral sim. = 858, #3

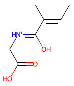

Struc. sim. = 1.00
Tiglylglycine

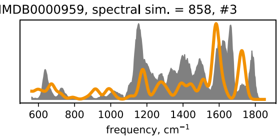


protonated HMDB0014755, spectral sim. = 750, #4

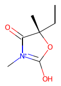

Struc. sim. = 0.20
Paramethadione

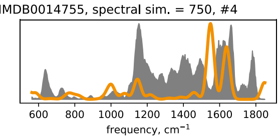

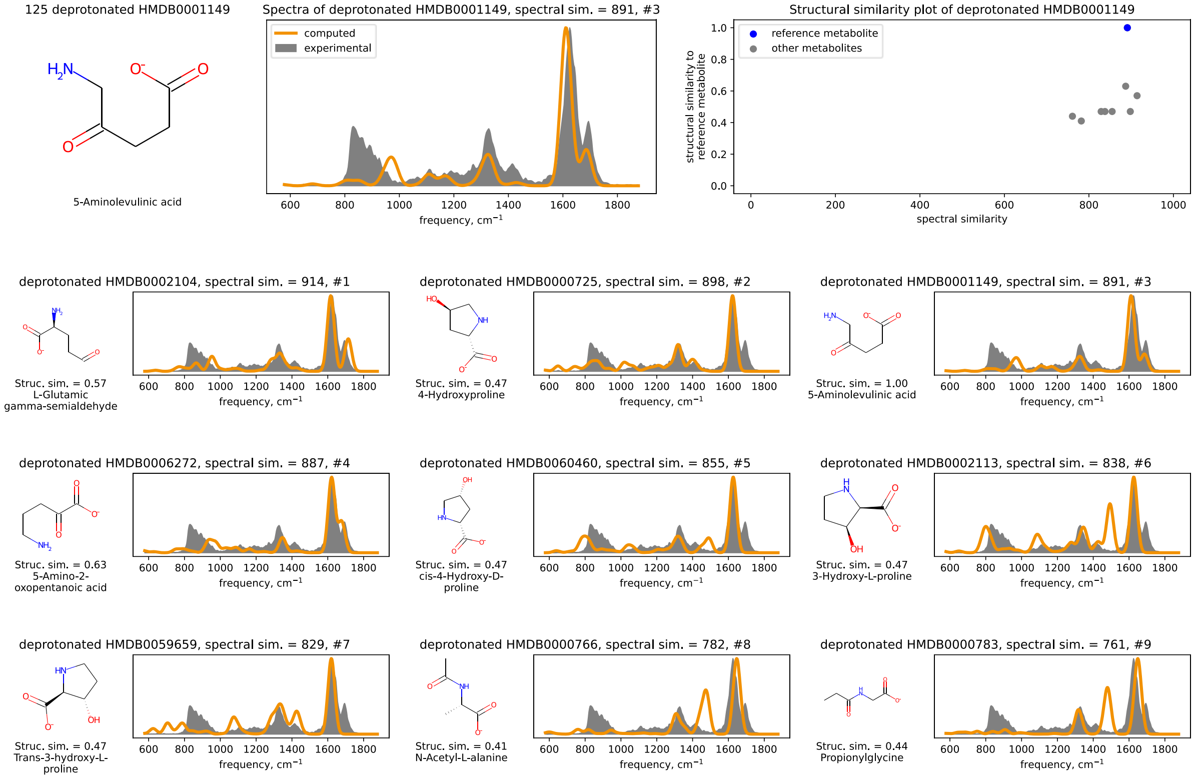
125 deprotonated HMDB0001149
5-Aminolevulinic acid
Spectra of deprotonated HMDB0001149, spectral sim. = 891, #3
computed
experimental
frequency, cm⁻¹
Structural similarity plot of deprotonated HMDB0001149
reference metabolite
other metabolites
structural similarity to reference metabolite
spectral similarity
deprotonated HMDB0002104, spectral sim. = 914, #1
Struc. sim. = 0.57
L-Glutamic gamma-semialdehyde
deprotonated HMDB0000725, spectral sim. = 898, #2
Struc. sim. = 0.47
4-Hydroxyproline
deprotonated HMDB0001149, spectral sim. = 891, #3
Struc. sim. = 1.00
5-Aminolevulinic acid
deprotonated HMDB0006272, spectral sim. = 887, #4
Struc. sim. = 0.63
5-Amino-2-oxopentanoic acid
deprotonated HMDB0060460, spectral sim. = 855, #5
Struc. sim. = 0.47
cis-4-Hydroxy-D-proline
deprotonated HMDB0002113, spectral sim. = 838, #6
Struc. sim. = 0.47
3-Hydroxy-L-proline
deprotonated HMDB0059659, spectral sim. = 829, #7
Struc. sim. = 0.47
Trans-3-hydroxy-L-proline
deprotonated HMDB0000766, spectral sim. = 782, #8
Struc. sim. = 0.41
N-Acetyl-L-alanine
deprotonated HMDB0000783, spectral sim. = 761, #9
Struc. sim. = 0.44
Propionylglycine

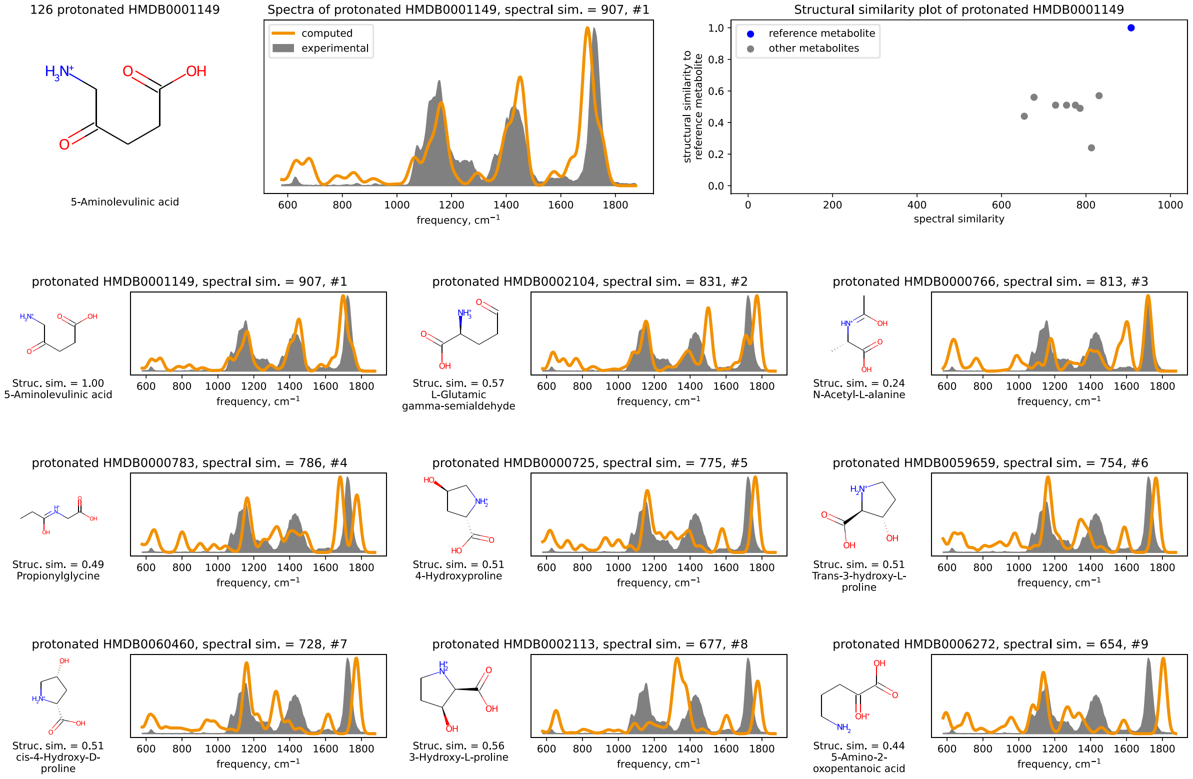
126 protonated HMDB0001149
5-Aminolevulinic acid
Spectra of protonated HMDB0001149, spectral sim. = 907, #1
computed
experimental
frequency, cm⁻¹
Structural similarity plot of protonated HMDB0001149
reference metabolite
other metabolites
structural similarity to reference metabolite
spectral similarity
protonated HMDB0001149, spectral sim. = 907, #1
Struc. sim. = 1.00
5-Aminolevulinic acid
protonated HMDB0002104, spectral sim. = 831, #2
Struc. sim. = 0.57
L-Glutamic gamma-semialdehyde
protonated HMDB0000766, spectral sim. = 813, #3
Struc. sim. = 0.24
N-Acetyl-L-alanine
protonated HMDB0000783, spectral sim. = 786, #4
Struc. sim. = 0.49
Propionylglycine
protonated HMDB0000725, spectral sim. = 775, #5
Struc. sim. = 0.51
4-Hydroxyproline
protonated HMDB0059659, spectral sim. = 754, #6
Struc. sim. = 0.51
Trans-3-hydroxy-L-proline
protonated HMDB0060460, spectral sim. = 728, #7
Struc. sim. = 0.51
cis-4-Hydroxy-D-proline
protonated HMDB0002113, spectral sim. = 677, #8
Struc. sim. = 0.56
3-Hydroxy-L-proline
protonated HMDB0006272, spectral sim. = 654, #9
Struc. sim. = 0.44
5-Amino-2-oxopentanoic acid

127 sodiated HMDB0001149

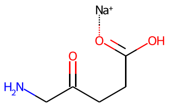

5-Aminolevulinic acid

Spectra of sodiated HMDB0001149, spectral sim. = 861, #2

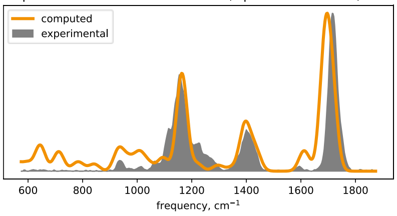


Structural similarity plot of sodiated HMDB0001149

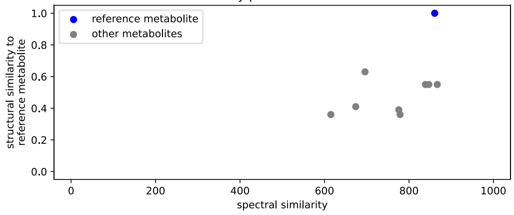


sodiated HMDB0060460, spectral sim. = 867, #1

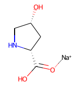

Struc. sim. = 0.55
cis-4-Hydroxy-D-proline

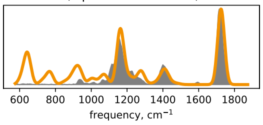


sodiated HMDB0001149, spectral sim. = 861, #2

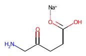

Struc. sim. = 1.00
5-Aminolevulinic acid

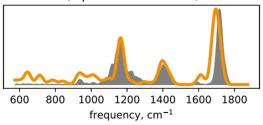


sodiated HMDB0002113, spectral sim. = 847, #3

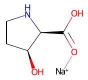

Struc. sim. = 0.55
3-Hydroxy-L-proline

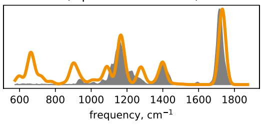


sodiated HMDB0059659, spectral sim. = 839, #4

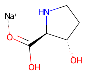

Struc. sim. = 0.55
Trans-3-hydroxy-L-proline

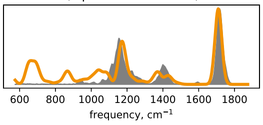


sodiated HMDB0000766, spectral sim. = 779, #5

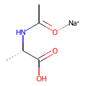

Struc. sim. = 0.36
N-Acetyl-L-alanine

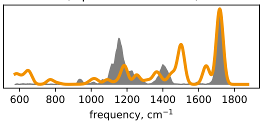


sodiated HMDB0000783, spectral sim. = 776, #6

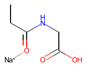

Struc. sim. = 0.39
Propionylglycine

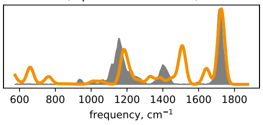


sodiated HMDB0002104, spectral sim. = 696, #7

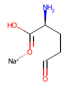

Struc. sim. = 0.63
L-Glutamic gamma-semialdehyde

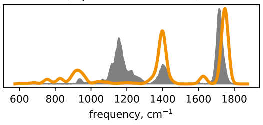


sodiated HMDB0000725, spectral sim. = 674, #8

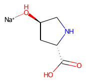

Struc. sim. = 0.41
4-Hydroxyproline

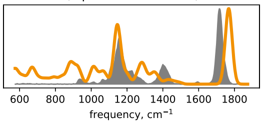


sodiated HMDB0006272, spectral sim. = 615, #9

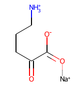

Struc. sim. = 0.36
5-Amino-2-oxopentanoic acid

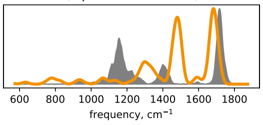


128 deprotonated HMDB0001232

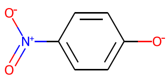

4-Nitrophenol

Spectra of deprotonated HMDB0001232, spectral sim. = 817, #1

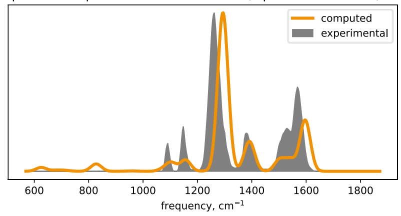


Structural similarity plot of deprotonated HMDB0001232

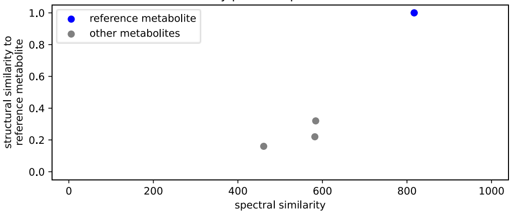


deprotonated HMDB0001232, spectral sim. = 817, #1

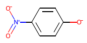

Struc. sim. = 1.00
4-Nitrophenol

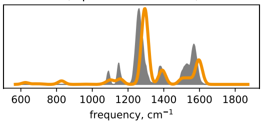


deprotonated HMDB0013188, spectral sim. = 584, #2

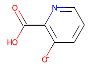

Struc. sim. = 0.32
3-Hydroxypicolinic acid

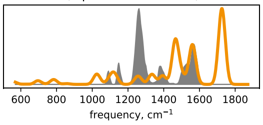


deprotonated HMDB0059710, spectral sim. = 582, #3

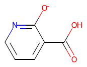

Struc. sim. = 0.22
2-Hydroxynicotinic acid

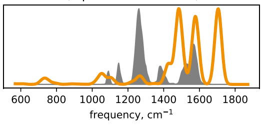


deprotonated HMDB0002658, spectral sim. = 461, #4

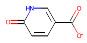

Struc. sim. = 0.16
6-Hydroxynicotinic acid

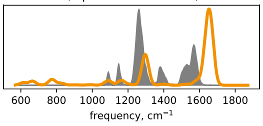

129 protonated HMDB0001232

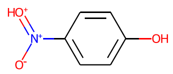


4-Nitrophenol

Spectra of protonated HMDB0001232, spectral sim. = 834, #1

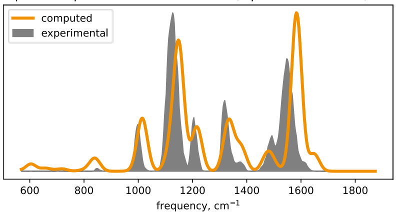


Structural similarity plot of protonated HMDB0001232

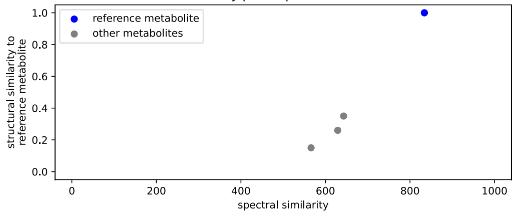


protonated HMDB0001232, spectral sim. = 834, #1

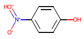

Struc. sim. = 1.00
4-Nitrophenol

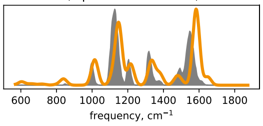


protonated HMDB0013188, spectral sim. = 643, #2

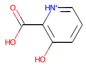


Struc. sim. = 0.35
3-Hydroxypicolinic acid

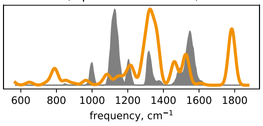


protonated HMDB0002658, spectral sim. = 629, #3

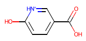

Struc. sim. = 0.26
6-Hydroxynicotinic acid

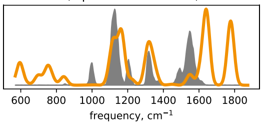


protonated HMDB0059710, spectral sim. = 566, #4

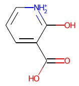


Struc. sim. = 0.15
2-Hydroxynicotinic acid

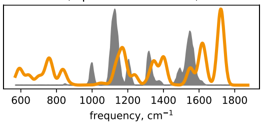

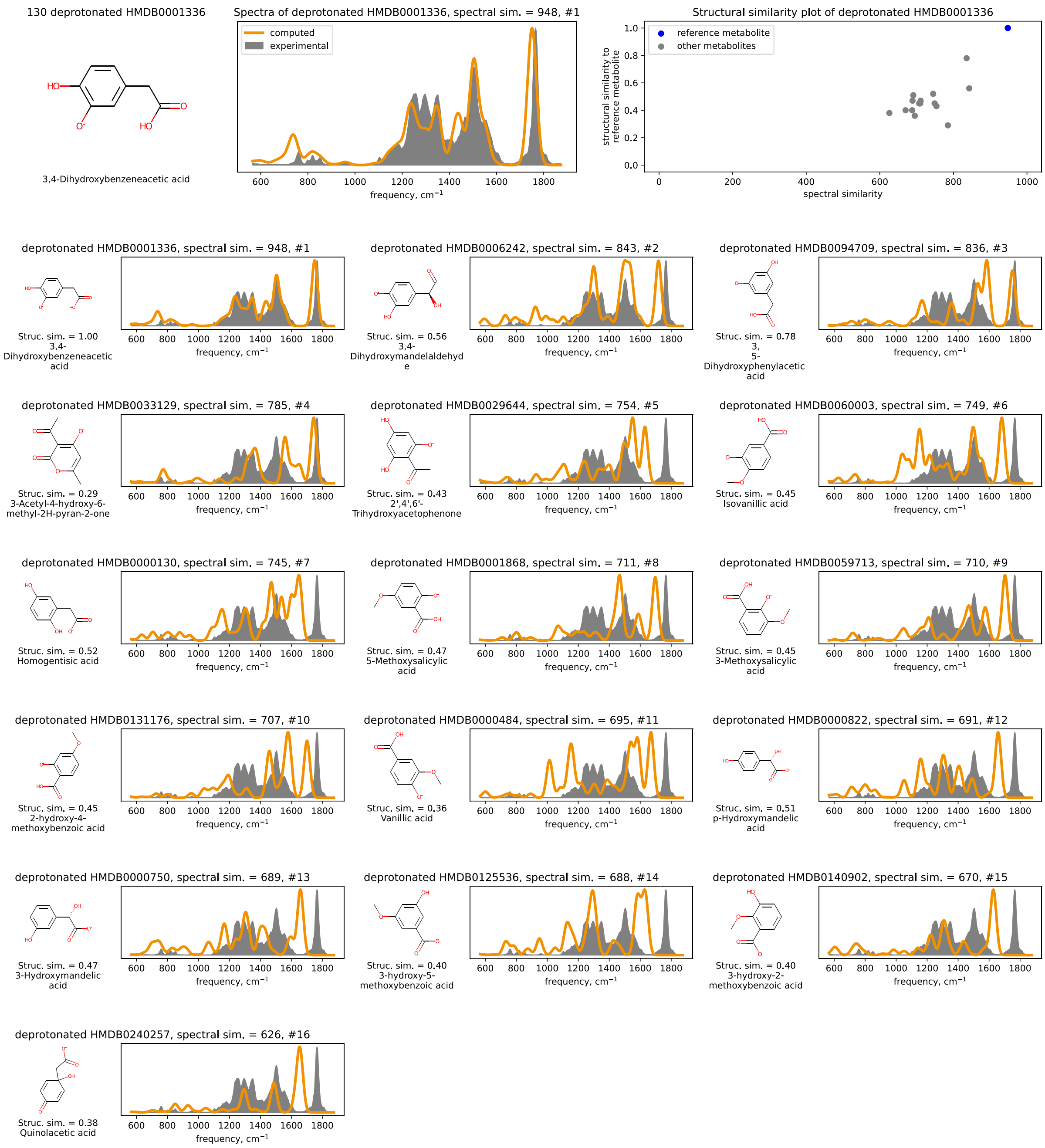

130 deprotonated HMDB0001336
3,4-Dihydroxybenzeneacetic acid
Spectra of deprotonated HMDB0001336, spectral sim. = 948, #1
computed
experimental
frequency, cm⁻¹
Structural similarity plot of deprotonated HMDB0001336
reference metabolite
other metabolites
structural similarity to reference metabolite
spectral similarity
deprotonated HMDB0001336, spectral sim. = 948, #1
Struc. sim. = 1.00
3,4-Dihydroxybenzeneacetic acid
deprotonated HMDB0006242, spectral sim. = 843, #2
Struc. sim. = 0.56
3,4-Dihydroxymandelaldehyde
deprotonated HMDB0094709, spectral sim. = 836, #3
Struc. sim. = 0.78
3,5-Dihydroxyphenylacetic acid
deprotonated HMDB0033129, spectral sim. = 785, #4
Struc. sim. = 0.29
3-Acetyl-4-hydroxy-6-methyl-2H-pyran-2-one
deprotonated HMDB0029644, spectral sim. = 754, #5
Struc. sim. = 0.43
2',4',6'-Trihydroxyacetophenone
deprotonated HMDB0060003, spectral sim. = 749, #6
Struc. sim. = 0.45
Isovanillic acid
deprotonated HMDB0000130, spectral sim. = 745, #7
Struc. sim. = 0.52
Homogentisic acid
deprotonated HMDB0001868, spectral sim. = 711, #8
Struc. sim. = 0.47
5-Methoxysalicylic acid
deprotonated HMDB0059713, spectral sim. = 710, #9
Struc. sim. = 0.45
3-Methoxysalicylic acid
deprotonated HMDB0131176, spectral sim. = 707, #10
Struc. sim. = 0.45
2-hydroxy-4-methoxybenzoic acid
deprotonated HMDB0000484, spectral sim. = 695, #11
Struc. sim. = 0.36
Vanillic acid
deprotonated HMDB0000822, spectral sim. = 691, #12
Struc. sim. = 0.51
p-Hydroxymandelic acid
deprotonated HMDB0000750, spectral sim. = 689, #13
Struc. sim. = 0.47
3-Hydroxymandelic acid
deprotonated HMDB0125536, spectral sim. = 688, #14
Struc. sim. = 0.40
3-hydroxy-5-methoxybenzoic acid
deprotonated HMDB0140902, spectral sim. = 670, #15
Struc. sim. = 0.40
3-hydroxy-2-methoxybenzoic acid
deprotonated HMDB0240257, spectral sim. = 626, #16
Struc. sim. = 0.38
Quinolacetic acid

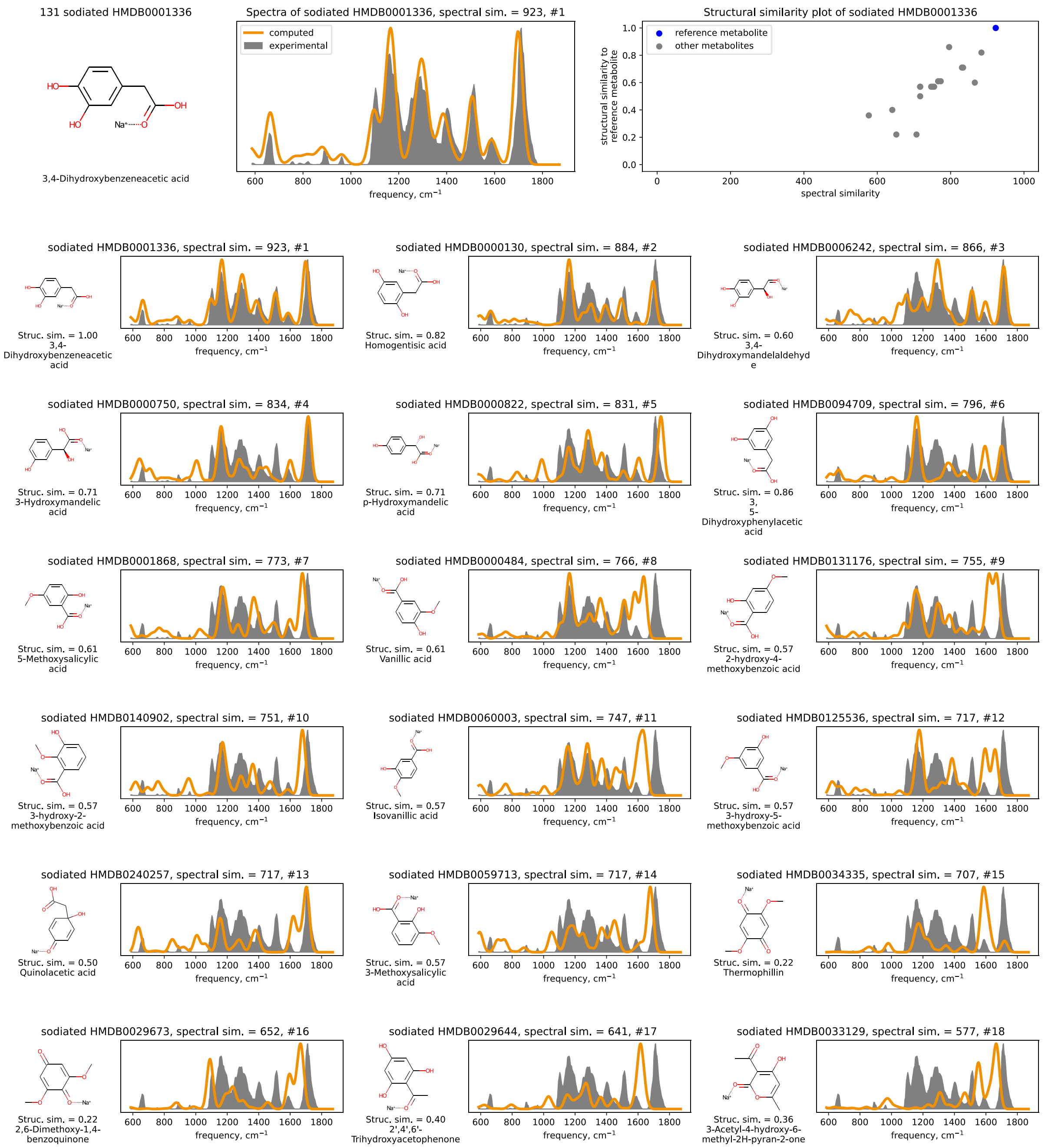

131 sodiated HMDB0001336
3,4-Dihydroxybenzeneacetic acid
Spectra of sodiated HMDB0001336, spectral sim. = 923, #1
computed
experimental
frequency, cm$^{-1}$
Structural similarity plot of sodiated HMDB0001336
reference metabolite
other metabolites
structural similarity to reference metabolite
spectral similarity
sodiated HMDB0001336, spectral sim. = 923, #1
Struc. sim. = 1.00
3,4-Dihydroxybenzeneacetic acid
sodiated HMDB0000130, spectral sim. = 884, #2
Struc. sim. = 0.82
Homogentisic acid
sodiated HMDB0006242, spectral sim. = 866, #3
Struc. sim. = 0.60
3,4-Dihydroxymandelaldehyde
sodiated HMDB0000750, spectral sim. = 834, #4
Struc. sim. = 0.71
3-Hydroxymandelic acid
sodiated HMDB0000822, spectral sim. = 831, #5
Struc. sim. = 0.71
p-Hydroxymandelic acid
sodiated HMDB0094709, spectral sim. = 796, #6
Struc. sim. = 0.86
3,5-Dihydroxyphenylacetic acid
sodiated HMDB0001868, spectral sim. = 773, #7
Struc. sim. = 0.61
5-Methoxysalicylic acid
sodiated HMDB0000484, spectral sim. = 766, #8
Struc. sim. = 0.61
Vanillic acid
sodiated HMDB0131176, spectral sim. = 755, #9
Struc. sim. = 0.57
2-hydroxy-4-methoxybenzoic acid
sodiated HMDB0140902, spectral sim. = 751, #10
Struc. sim. = 0.57
3-hydroxy-2-methoxybenzoic acid
sodiated HMDB0060003, spectral sim. = 747, #11
Struc. sim. = 0.57
Isovanillic acid
sodiated HMDB0125536, spectral sim. = 717, #12
Struc. sim. = 0.57
3-hydroxy-5-methoxybenzoic acid
sodiated HMDB0240257, spectral sim. = 717, #13
Struc. sim. = 0.50
Quinolacetic acid
sodiated HMDB0059713, spectral sim. = 717, #14
Struc. sim. = 0.57
3-Methoxysalicylic acid
sodiated HMDB0034335, spectral sim. = 707, #15
Struc. sim. = 0.22
Thermophillin
sodiated HMDB0029673, spectral sim. = 652, #16
Struc. sim. = 0.22
2,6-Dimethoxy-1,4-benzoquinone
sodiated HMDB0029644, spectral sim. = 641, #17
Struc. sim. = 0.40
2',4',6'-Trihydroxyacetophenone
sodiated HMDB0033129, spectral sim. = 577, #18
Struc. sim. = 0.36
3-Acetyl-4-hydroxy-6-methyl-2H-pyran-2-one

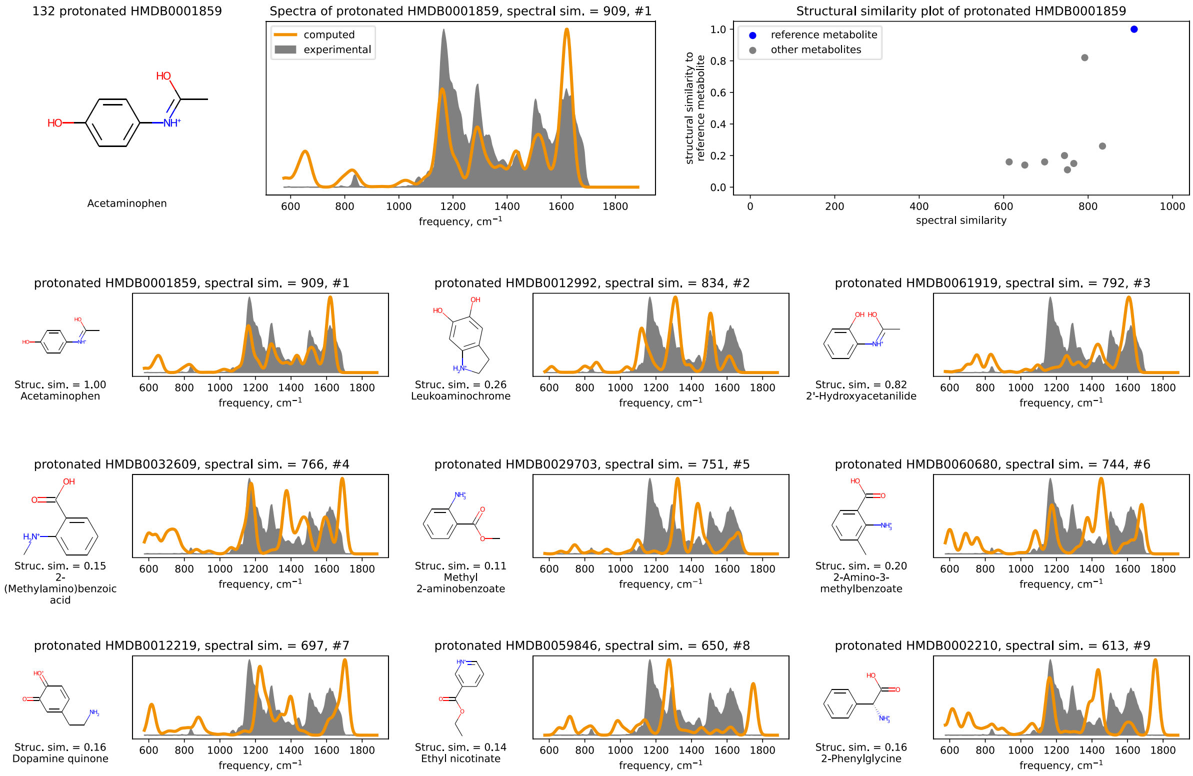
132 protonated HMDB0001859
Acetaminophen
Spectra of protonated HMDB0001859, spectral sim. = 909, #1
computed
experimental
frequency, cm$^{-1}$
Structural similarity plot of protonated HMDB0001859
reference metabolite
other metabolites
structural similarity to reference metabolite
spectral similarity
protonated HMDB0001859, spectral sim. = 909, #1
Struc. sim. = 1.00
Acetaminophen
protonated HMDB0012992, spectral sim. = 834, #2
Struc. sim. = 0.26
Leukoaminochrome
protonated HMDB0061919, spectral sim. = 792, #3
Struc. sim. = 0.82
2'-Hydroxyacetanilide
protonated HMDB0032609, spectral sim. = 766, #4
Struc. sim. = 0.15
2-(Methylamino)benzoic acid
protonated HMDB0029703, spectral sim. = 751, #5
Struc. sim. = 0.11
Methyl 2-aminobenzoate
protonated HMDB0060680, spectral sim. = 744, #6
Struc. sim. = 0.20
2-Amino-3-methylbenzoate
protonated HMDB0012219, spectral sim. = 697, #7
Struc. sim. = 0.16
Dopamine quinone
protonated HMDB0059846, spectral sim. = 650, #8
Struc. sim. = 0.14
Ethyl nicotinate
protonated HMDB0002210, spectral sim. = 613, #9
Struc. sim. = 0.16
2-Phenylglycine

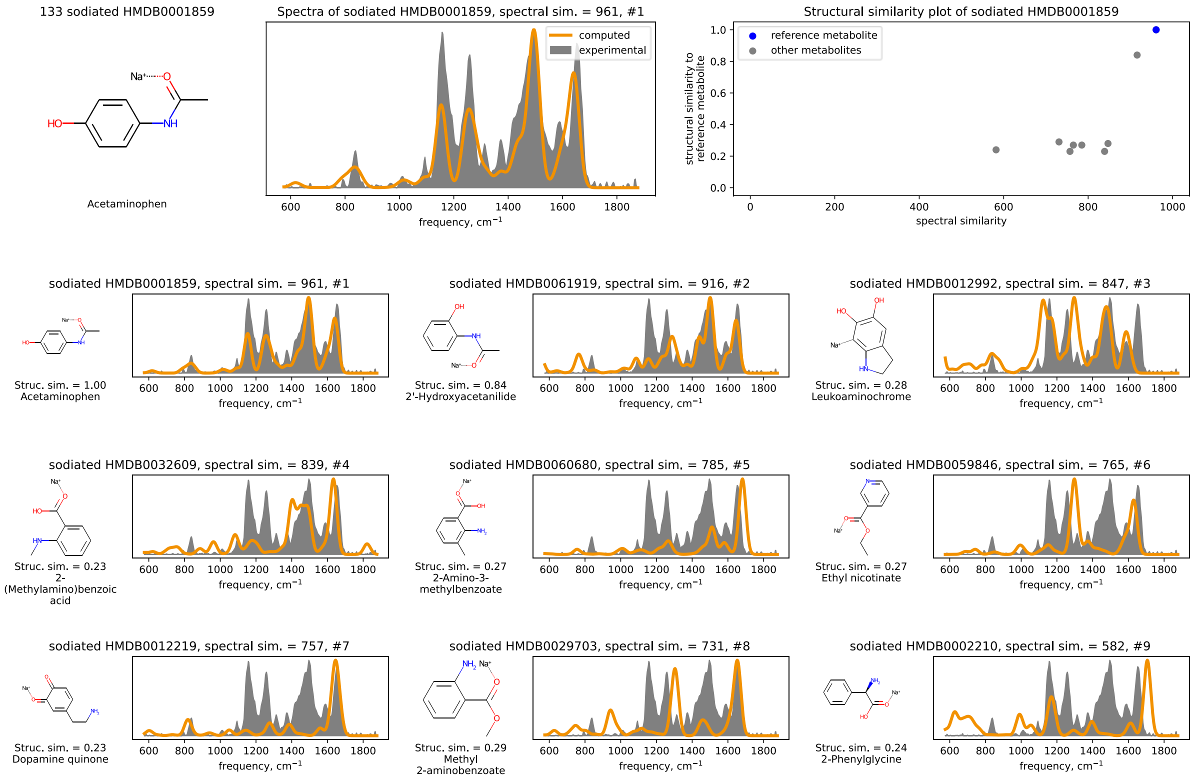
133 sodiated HMDB0001859
Acetaminophen
Spectra of sodiated HMDB0001859, spectral sim. = 961, #1
computed
experimental
frequency, cm$^{-1}$
Structural similarity plot of sodiated HMDB0001859
reference metabolite
other metabolites
structural similarity to reference metabolite
spectral similarity
sodiated HMDB0001859, spectral sim. = 961, #1
Struc. sim. = 1.00
Acetaminophen
sodiated HMDB0061919, spectral sim. = 916, #2
Struc. sim. = 0.84
2'-Hydroxyacetanilide
sodiated HMDB0012992, spectral sim. = 847, #3
Struc. sim. = 0.28
Leukoaminochrome
sodiated HMDB0032609, spectral sim. = 839, #4
Struc. sim. = 0.23
2-(Methylamino)benzoic acid
sodiated HMDB0060680, spectral sim. = 785, #5
Struc. sim. = 0.27
2-Amino-3-methylbenzoate
sodiated HMDB0059846, spectral sim. = 765, #6
Struc. sim. = 0.27
Ethyl nicotinate
sodiated HMDB0012219, spectral sim. = 757, #7
Struc. sim. = 0.23
Dopamine quinone
sodiated HMDB0029703, spectral sim. = 731, #8
Struc. sim. = 0.29
Methyl 2-aminobenzoate
sodiated HMDB0002210, spectral sim. = 582, #9
Struc. sim. = 0.24
2-Phenylglycine

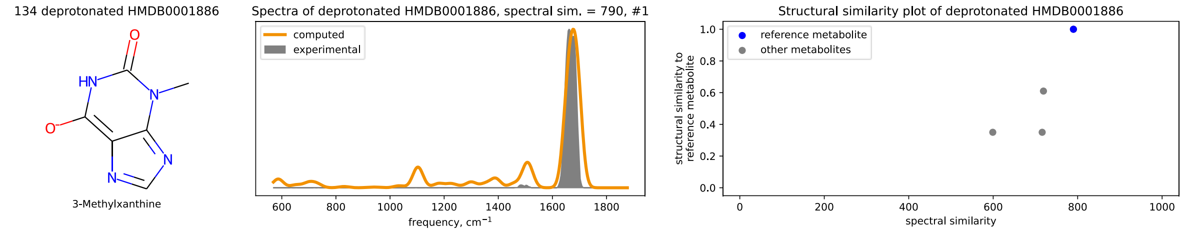
134 deprotonated HMDB0001886
3-Methylxanthine
Spectra of deprotonated HMDB0001886, spectral sim. = 790, #1
computed
experimental
frequency, cm$^{-1}$
Structural similarity plot of deprotonated HMDB0001886
reference metabolite
other metabolites
structural similarity to reference metabolite
spectral similarity

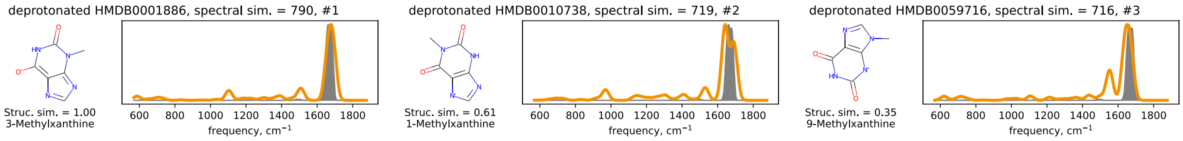
deprotonated HMDB0001886, spectral sim. = 790, #1
Struc. sim. = 1.00
3-Methylxanthine
deprotonated HMDB0010738, spectral sim. = 719, #2
Struc. sim. = 0.61
1-Methylxanthine
deprotonated HMDB0059716, spectral sim. = 716, #3
Struc. sim. = 0.35
9-Methylxanthine
frequency, cm$^{-1}$

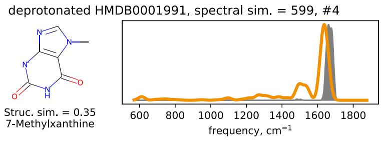
deprotonated HMDB0001991, spectral sim. = 599, #4
Struc. sim. = 0.35
7-Methylxanthine
frequency, cm$^{-1}$

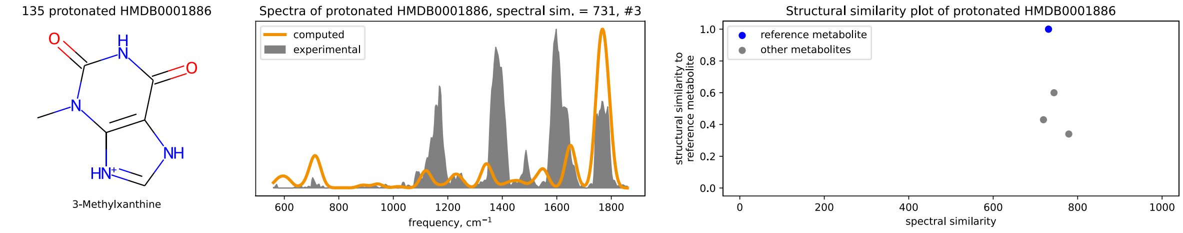
135 protonated HMDB0001886
3-Methylxanthine
Spectra of protonated HMDB0001886, spectral sim. = 731, #3
computed
experimental
frequency, cm$^{-1}$
Structural similarity plot of protonated HMDB0001886
reference metabolite
other metabolites
structural similarity to reference metabolite
spectral similarity

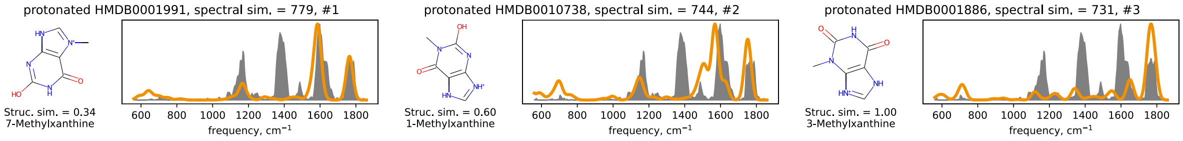
protonated HMDB0001991, spectral sim. = 779, #1
Struc. sim. = 0.34
7-Methylxanthine
protonated HMDB0010738, spectral sim. = 744, #2
Struc. sim. = 0.60
1-Methylxanthine
protonated HMDB0001886, spectral sim. = 731, #3
Struc. sim. = 1.00
3-Methylxanthine
frequency, cm$^{-1}$

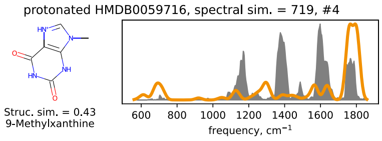
protonated HMDB0059716, spectral sim. = 719, #4
Struc. sim. = 0.43
9-Methylxanthine
frequency, cm$^{-1}$

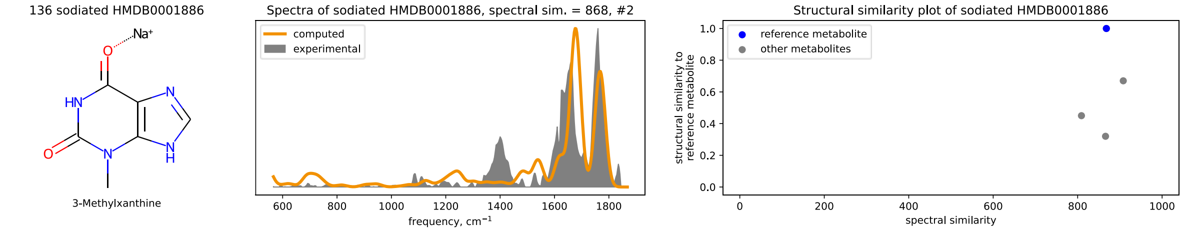
136 sodiated HMDB0001886
3-Methylxanthine
Spectra of sodiated HMDB0001886, spectral sim. = 868, #2
computed
experimental
frequency, cm$^{-1}$
Structural similarity plot of sodiated HMDB0001886
reference metabolite
other metabolites
structural similarity to reference metabolite
spectral similarity

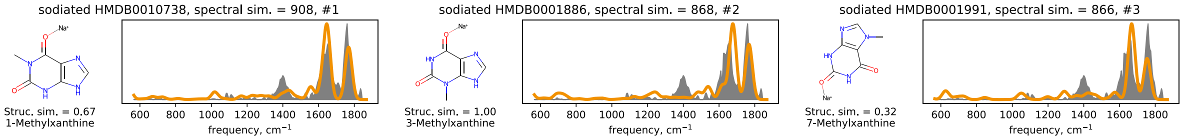
sodiated HMDB0010738, spectral sim. = 908, #1
Struc. sim. = 0.67
1-Methylxanthine
sodiated HMDB0001886, spectral sim. = 868, #2
Struc. sim. = 1.00
3-Methylxanthine
sodiated HMDB0001991, spectral sim. = 866, #3
Struc. sim. = 0.32
7-Methylxanthine
frequency, cm$^{-1}$

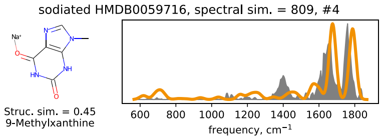
sodiated HMDB0059716, spectral sim. = 809, #4
Struc. sim. = 0.45
9-Methylxanthine
frequency, cm$^{-1}$

137 deprotonated HMDB0001890

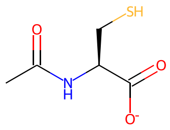


Acetylcysteine

Spectra of deprotonated HMDB0001890, spectral sim. = 915, #1

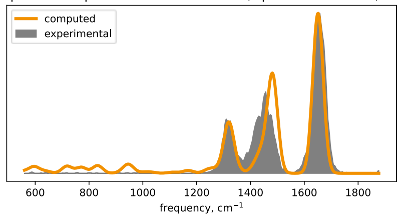


Structural similarity plot of deprotonated HMDB0001890

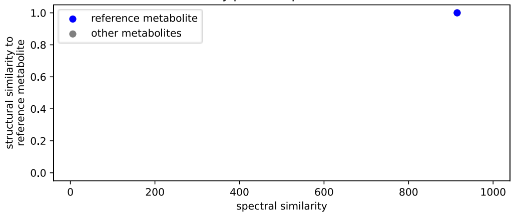


deprotonated HMDB0001890, spectral sim. = 915, #1

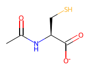

Struc. sim. = 1.00
Acetylcysteine

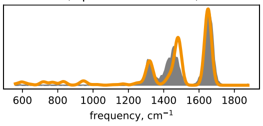


138 protonated HMDB0001890

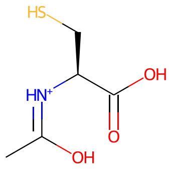


Acetylcysteine

Spectra of protonated HMDB0001890, spectral sim. = 808, #1

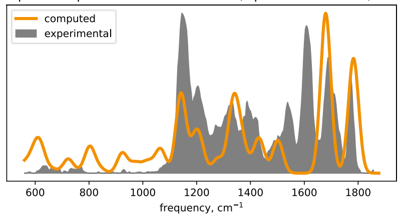


Structural similarity plot of protonated HMDB0001890

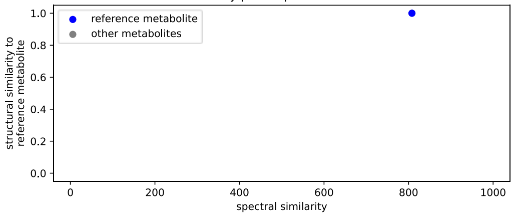


protonated HMDB0001890, spectral sim. = 808, #1

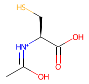

Struc. sim. = 1.00
Acetylcysteine

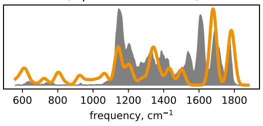


139 sodiated HMDB0001890

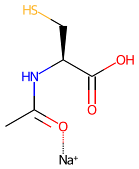


Acetylcysteine

Spectra of sodiated HMDB0001890, spectral sim. = 829, #1

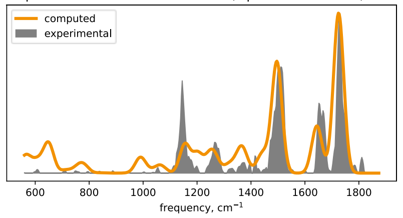


Structural similarity plot of sodiated HMDB0001890

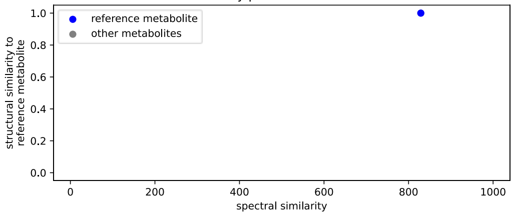


sodiated HMDB0001890, spectral sim. = 829, #1

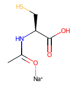

Struc. sim. = 1.00
Acetylcysteine

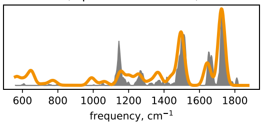

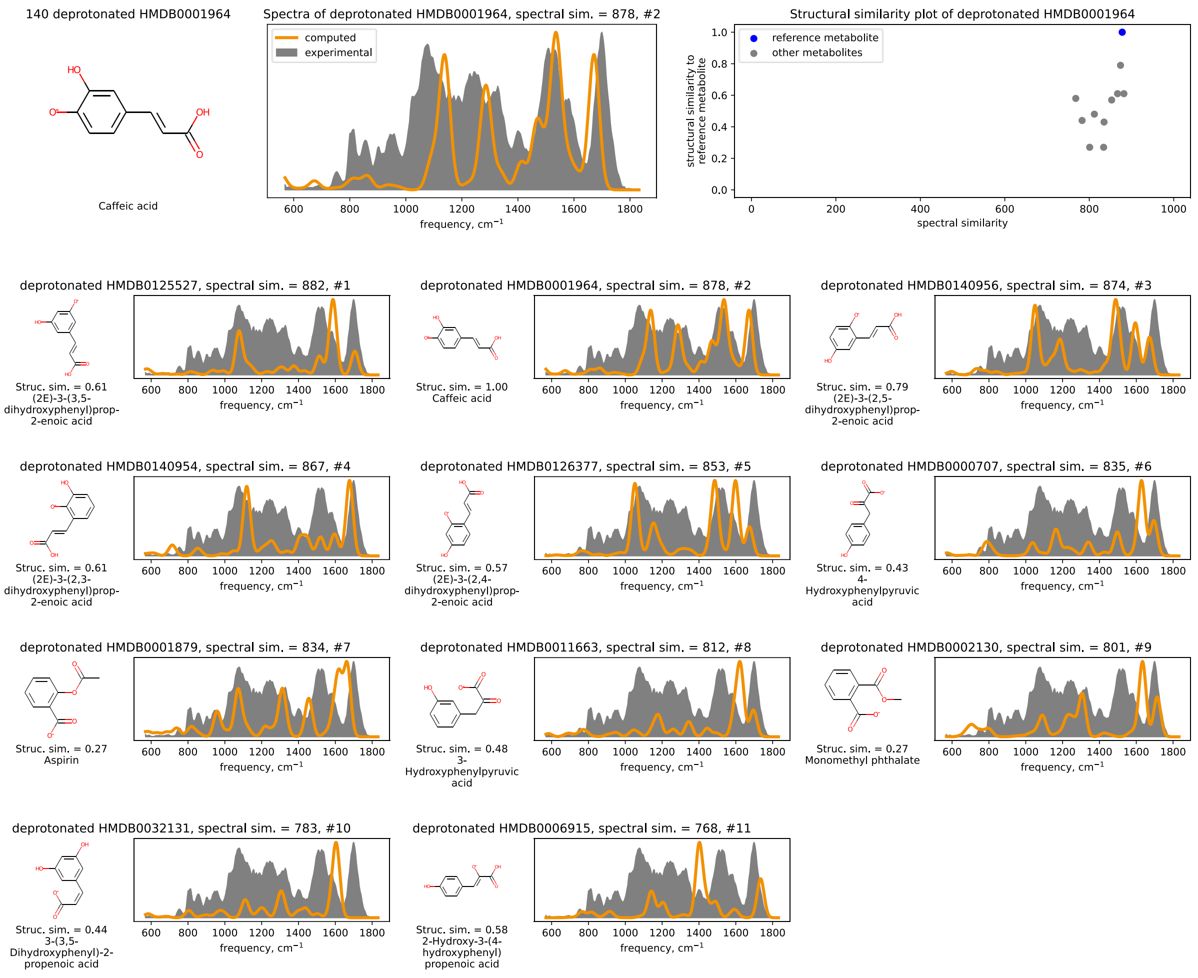
140 deprotonated HMDB0001964
Caffeic acid
Spectra of deprotonated HMDB0001964, spectral sim. = 878, #2
computed
experimental
frequency, cm⁻¹
Structural similarity plot of deprotonated HMDB0001964
reference metabolite
other metabolites
structural similarity to reference metabolite
spectral similarity
deprotonated HMDB0125527, spectral sim. = 882, #1
Struc. sim. = 0.61
(2E)-3-(3,5-dihydroxyphenyl)prop-2-enoic acid
deprotonated HMDB0001964, spectral sim. = 878, #2
Struc. sim. = 1.00
Caffeic acid
deprotonated HMDB0140956, spectral sim. = 874, #3
Struc. sim. = 0.79
(2E)-3-(2,5-dihydroxyphenyl)prop-2-enoic acid
deprotonated HMDB0140954, spectral sim. = 867, #4
Struc. sim. = 0.61
(2E)-3-(2,3-dihydroxyphenyl)prop-2-enoic acid
deprotonated HMDB0126377, spectral sim. = 853, #5
Struc. sim. = 0.57
(2E)-3-(2,4-dihydroxyphenyl)prop-2-enoic acid
deprotonated HMDB0000707, spectral sim. = 835, #6
Struc. sim. = 0.43
4-Hydroxyphenylpyruvic acid
deprotonated HMDB0001879, spectral sim. = 834, #7
Struc. sim. = 0.27
Aspirin
deprotonated HMDB0011663, spectral sim. = 812, #8
Struc. sim. = 0.48
3-Hydroxyphenylpyruvic acid
deprotonated HMDB0002130, spectral sim. = 801, #9
Struc. sim. = 0.27
Monomethyl phthalate
deprotonated HMDB0032131, spectral sim. = 783, #10
Struc. sim. = 0.44
3-(3,5-Dihydroxyphenyl)-2-propenoic acid
deprotonated HMDB0006915, spectral sim. = 768, #11
Struc. sim. = 0.58
2-Hydroxy-3-(4-hydroxyphenyl) propenoic acid

141 protonated HMDB0001964

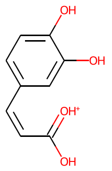


Caffeic acid

Spectra of protonated HMDB0001964, spectral sim. = 911, #1

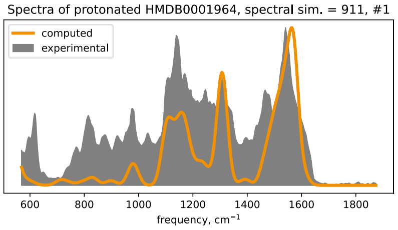


Structural similarity plot of protonated HMDB0001964

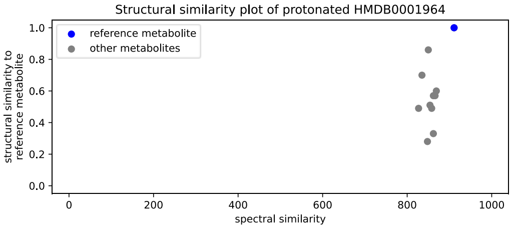


protonated HMDB0001964, spectral sim. = 911, #1

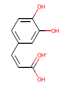

Struc. sim. = 1.00
Caffeic acid

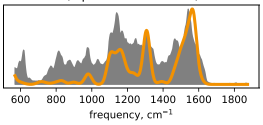


protonated HMDB0140954, spectral sim. = 869, #2

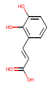

Struc. sim. = 0.60
(2E)-3-(2,3-dihydroxyphenyl)prop-2-enoic acid

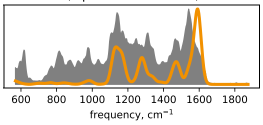


protonated HMDB0140956, spectral sim. = 866, #3

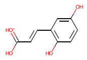

Struc. sim. = 0.57
(2E)-3-(2,5-dihydroxyphenyl)prop-2-enoic acid

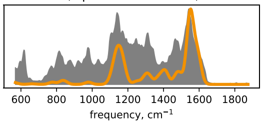


protonated HMDB0001879, spectral sim. = 862, #4

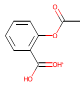

Struc. sim. = 0.33
Aspirin

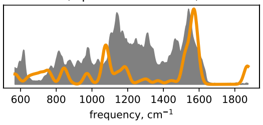


protonated HMDB0125527, spectral sim. = 862, #5

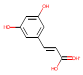

Struc. sim. = 0.57
(2E)-3-(3,5-dihydroxyphenyl)prop-2-enoic acid

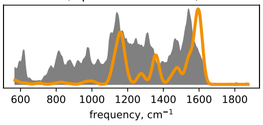


protonated HMDB0000707, spectral sim. = 858, #6

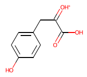

Struc. sim. = 0.49
4-Hydroxyphenylpyruvic acid

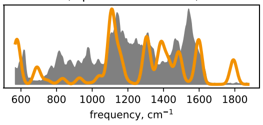


protonated HMDB0126377, spectral sim. = 854, #7

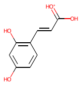

Struc. sim. = 0.51
(2E)-3-(2,4-dihydroxyphenyl)prop-2-enoic acid

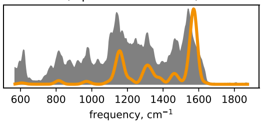


protonated HMDB0032131, spectral sim. = 850, #8

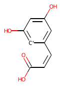

Struc. sim. = 0.86
3-(3,5-Dihydroxyphenyl)-2-propenoic acid

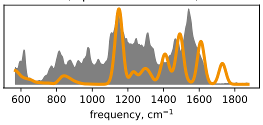


protonated HMDB0002130, spectral sim. = 848, #9

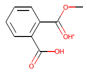

Struc. sim. = 0.28
Monomethyl phthalate

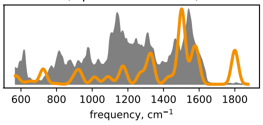


protonated HMDB0006915, spectral sim. = 835, #10

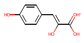

Struc. sim. = 0.70
2-Hydroxy-3-(4-hydroxyphenyl) propenoic acid

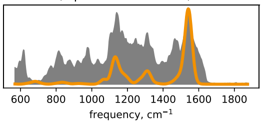


protonated HMDB0011663, spectral sim. = 827, #11

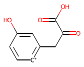

Struc. sim. = 0.49
3-Hydroxyphenylpyruvic acid

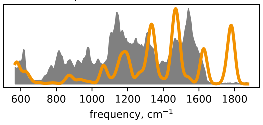

142 sodiated HMDB0001964

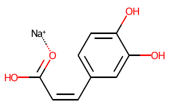


Caffeic acid

Spectra of sodiated HMDB0001964, spectral sim. = 944, #1

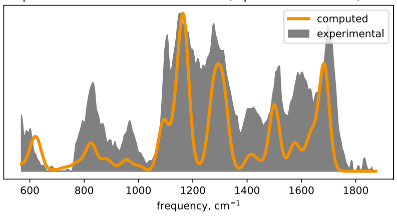


Structural similarity plot of sodiated HMDB0001964

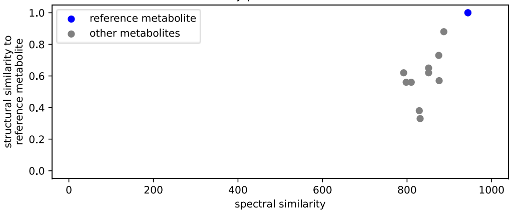


sodiated HMDB0001964, spectral sim. = 944, #1

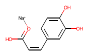

Struc. sim. = 1.00
Caffeic acid

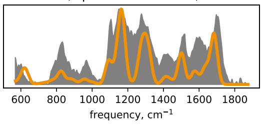


sodiated HMDB0032131, spectral sim. = 887, #2

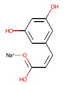

Struc. sim. = 0.88
3-(3,5-Dihydroxyphenyl)-2-propenoic acid

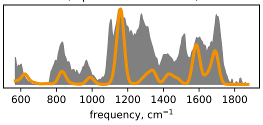


sodiated HMDB0126377, spectral sim. = 876, #3

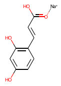

Struc. sim. = 0.57
(2E)-3-(2,4-dihydroxyphenyl)prop-2-enoic acid

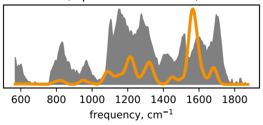


sodiated HMDB0006915, spectral sim. = 875, #4

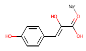

Struc. sim. = 0.73
2-Hydroxy-3-(4-hydroxyphenyl) propenoic acid

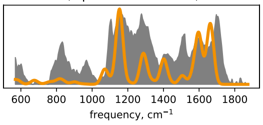


sodiated HMDB0140954, spectral sim. = 851, #5

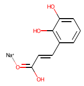

Struc. sim. = 0.65
(2E)-3-(2,3-dihydroxyphenyl)prop-2-enoic acid

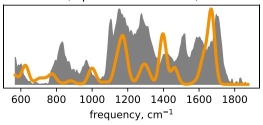


sodiated HMDB0140956, spectral sim. = 851, #6

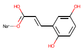

Struc. sim. = 0.62
(2E)-3-(2,5-dihydroxyphenyl)prop-2-enoic acid

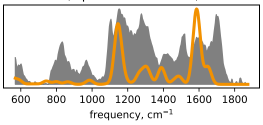


sodiated HMDB0001879, spectral sim. = 831, #7

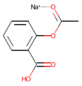

Struc. sim. = 0.33
Aspirin

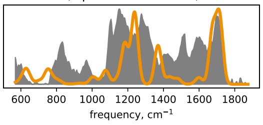


sodiated HMDB0002130, spectral sim. = 829, #8

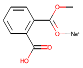

Struc. sim. = 0.38
Monomethyl phthalate

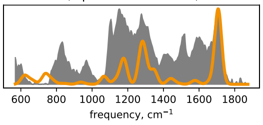


sodiated HMDB0000707, spectral sim. = 810, #9

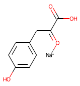

Struc. sim. = 0.56
4-Hydroxyphenylpyruvic acid

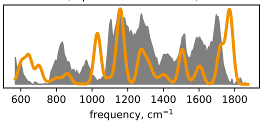


sodiated HMDB0011663, spectral sim. = 798, #10

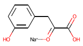

Struc. sim. = 0.56
3-Hydroxyphenylpyruvic acid

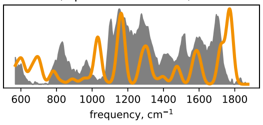


sodiated HMDB0125527, spectral sim. = 792, #11

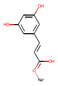

Struc. sim. = 0.62
(2E)-3-(3,5-dihydroxyphenyl)prop-2-enoic acid

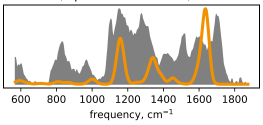


143 protonated HMDB0002266

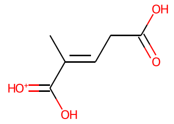


(E)-2-Methylglutaconic acid

Spectra of protonated HMDB0002266, spectral sim. = 949, #1

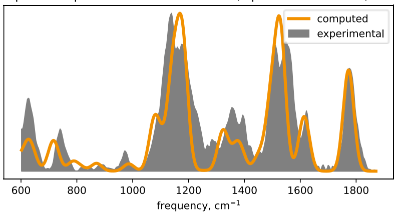


Structural similarity plot of protonated HMDB0002266

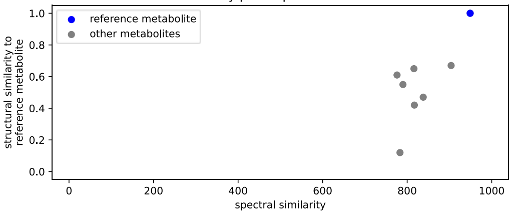


protonated HMDB0002266, spectral sim. = 949, #1

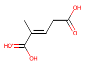

Struc. sim. = 1.00
(E)-2-Methylglutaconic acid

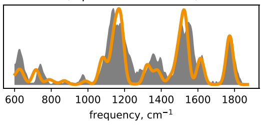


protonated HMDB0000522, spectral sim. = 904, #2

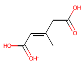

Struc. sim. = 0.67
3-Methylglutaconic acid

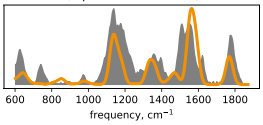


protonated HMDB0029171, spectral sim. = 838, #3

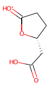

Struc. sim. = 0.47
3-Hydroxyadipic acid 3,6-lactone

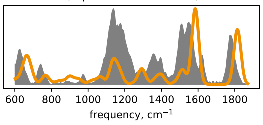


protonated HMDB0040207, spectral sim. = 817, #4

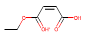

Struc. sim. = 0.42
Ethyl hydrogen fumarate

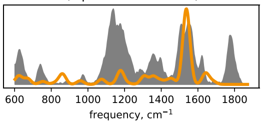


protonated HMDB0013311, spectral sim. = 816, #5

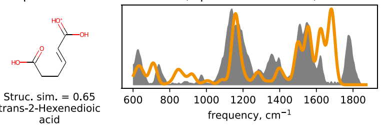


Struc. sim. = 0.65
trans-2-Hexenedioic acid

protonated HMDB0000393, spectral sim. = 790, #6

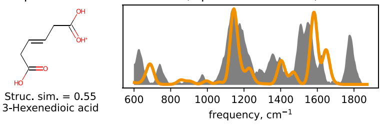


Struc. sim. = 0.55
3-Hexenedioic acid

protonated HMDB0031257, spectral sim. = 783, #7

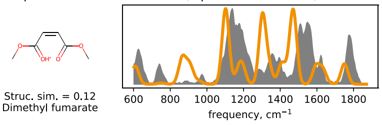


Struc. sim. = 0.12
Dimethyl fumarate

protonated HMDB0036232, spectral sim. = 776, #8

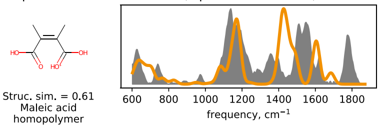


Struc. sim. = 0.61
Maleic acid homopolymer

144 deprotonated HMDB0002285

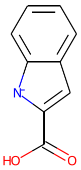


2-Indolecarboxylic acid

Spectra of deprotonated HMDB0002285, spectral sim. = 780, #1

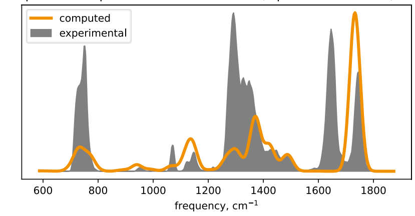


Structural similarity plot of deprotonated HMDB0002285

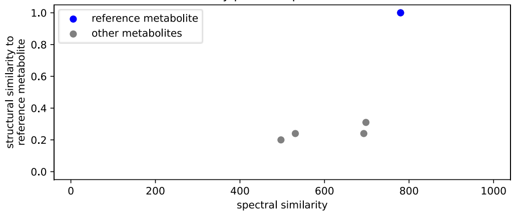


deprotonated HMDB0002285, spectral sim. = 780, #1

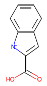

Struc. sim. = 1.00
2-Indolecarboxylic acid

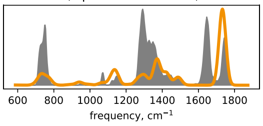


deprotonated HMDB0003320, spectral sim. = 698, #2

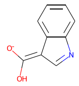

Struc. sim. = 0.31
Indole-3-carboxylic acid

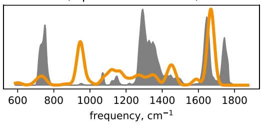


deprotonated HMDB0031172, spectral sim. = 693, #3

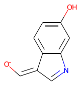

Struc. sim. = 0.24
3-Formyl-6-hydroxyindole

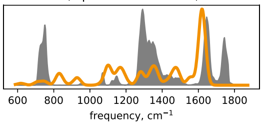


deprotonated HMDB0060289, spectral sim. = 531, #4

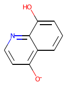

Struc. sim. = 0.24
Quinoline-4,8-diol

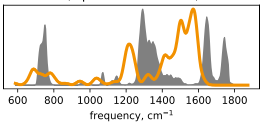


deprotonated HMDB0004077, spectral sim. = 497, #5

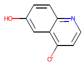

Struc. sim. = 0.20
4,6-Dihydroxyquinoline

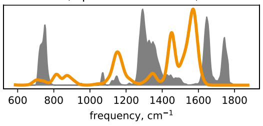


145 protonated HMDB0002285

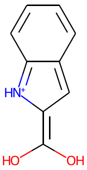


2-Indolecarboxylic acid

Spectra of protonated HMDB0002285, spectral sim. = 903, #1

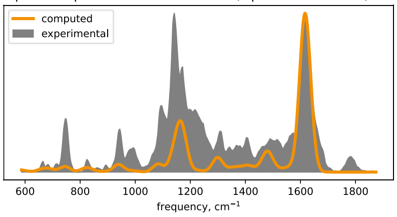


Structural similarity plot of protonated HMDB0002285

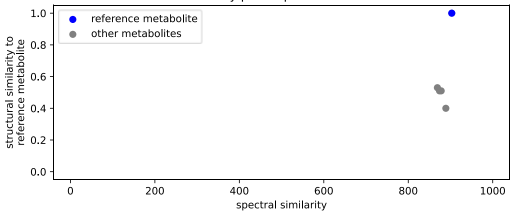


protonated HMDB0002285, spectral sim. = 903, #1

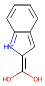

Struc. sim. = 1.00
2-Indolecarboxylic acid

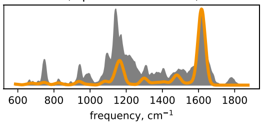


protonated HMDB0031172, spectral sim. = 889, #2

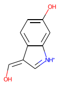

Struc. sim. = 0.40
3-Formyl-6-hydroxyindole

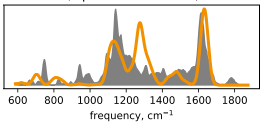


protonated HMDB0060289, spectral sim. = 878, #3

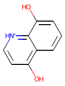

Struc. sim. = 0.51
Quinoline-4,8-diol

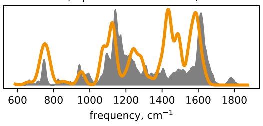


protonated HMDB0004077, spectral sim. = 874, #4

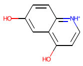

Struc. sim. = 0.51
4,6-Dihydroxyquinoline

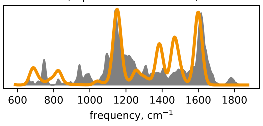


protonated HMDB0003320, spectral sim. = 869, #5

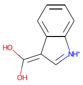

Struc. sim. = 0.53
Indole-3-carboxylic acid

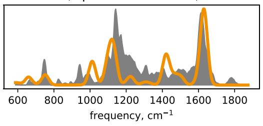


146 deprotonated HMDB0002302

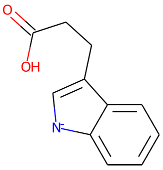


Indole-3-propionic acid

Spectra of deprotonated HMDB0002302, spectral sim. = 755, #1

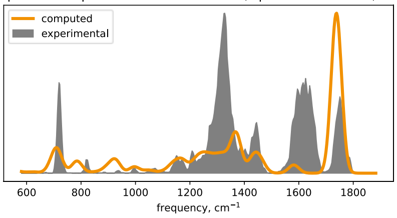


Structural similarity plot of deprotonated HMDB0002302

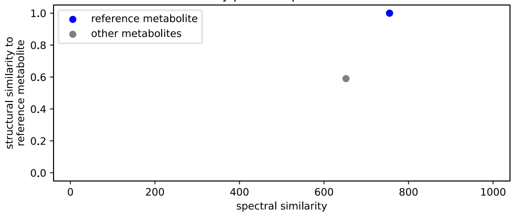


deprotonated HMDB0002302, spectral sim. = 755, #1

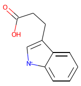

Struc. sim. = 1.00
Indole-3-propionic acid

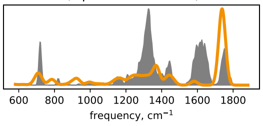


deprotonated HMDB0029738, spectral sim. = 652, #2

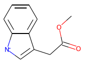

Struc. sim. = 0.59
Indole-3-methyl acetate

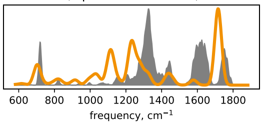

147 protonated HMDB0002302

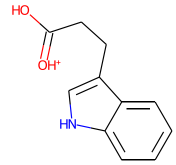


Indole-3-propionic acid

Spectra of protonated HMDB0002302, spectral sim. = 579, #2

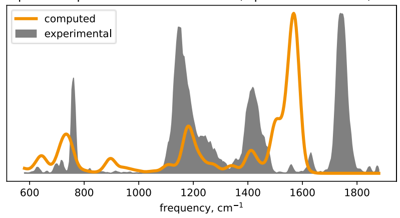


Structural similarity plot of protonated HMDB0002302

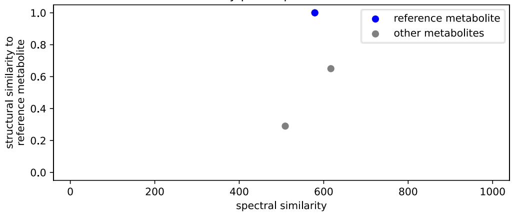


protonated HMDB0029738, spectral sim. = 617, #1

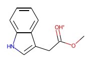

Struc. sim. = 0.65
Indole-3-methyl acetate

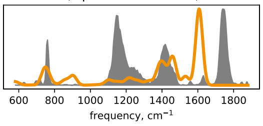


protonated HMDB0002302, spectral sim. = 579, #2

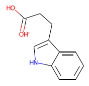

Struc. sim. = 1.00
Indole-3-propionic acid

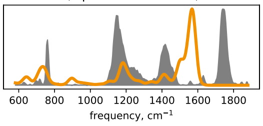


protonated HMDB0014970, spectral sim. = 509, #3

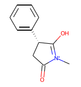

Struc. sim. = 0.29
Phensuximide

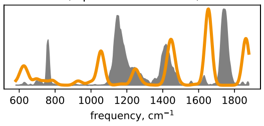


148 sodiated HMDB0002302

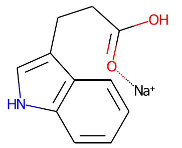


Indole-3-propionic acid

Spectra of sodiated HMDB0002302, spectral sim. = 862, #1

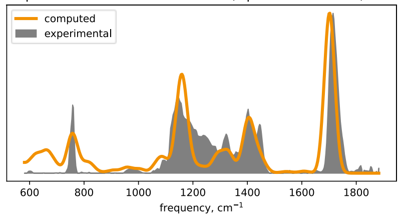


Structural similarity plot of sodiated HMDB0002302

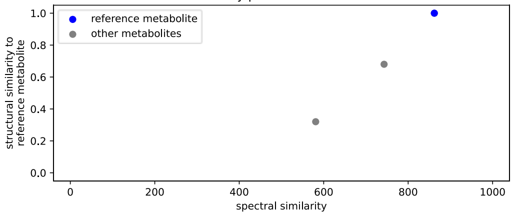


sodiated HMDB0002302, spectral sim. = 862, #1

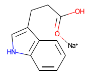

Struc. sim. = 1.00
Indole-3-propionic acid

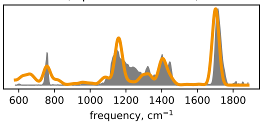


sodiated HMDB0029738, spectral sim. = 743, #2

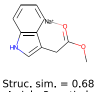

Struc. sim. = 0.68
Indole-3-methyl acetate

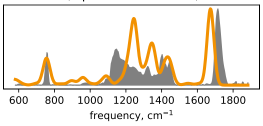


sodiated HMDB0014970, spectral sim. = 581, #3

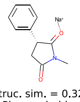

Struc. sim. = 0.32
Phensuximide

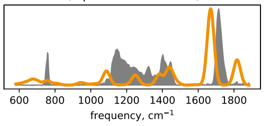


149 deprotonated HMDB0002432

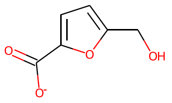


Sumiki's acid

Spectra of deprotonated HMDB0002432, spectral sim. = 924, #1

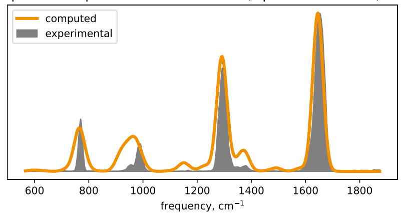


Structural similarity plot of deprotonated HMDB0002432

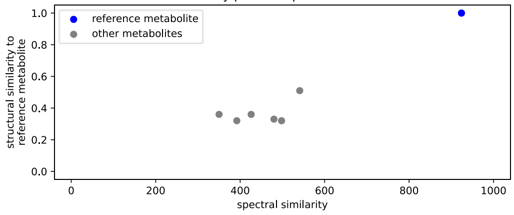


deprotonated HMDB0002432, spectral sim. = 924, #1

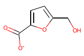

Struc. sim. = 1.00
Sumiki's acid

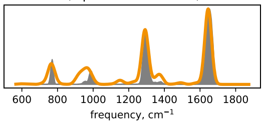


deprotonated HMDB0032923, spectral sim. = 541, #2

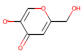

Struc. sim. = 0.51
Kojic acid

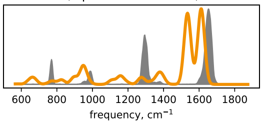


deprotonated HMDB0002349, spectral sim. = 498, #3

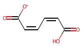

Struc. sim. = 0.32
trans-trans-Muconic acid

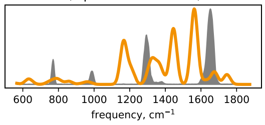


deprotonated HMDB0059762, spectral sim. = 480, #4

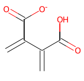

Struc. sim. = 0.33
2,3-Methylenesuccinic acid

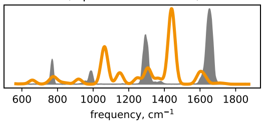


deprotonated HMDB0134002, spectral sim. = 426, #5

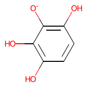

Struc. sim. = 0.36
benzene-1,2,3,4-tetrol

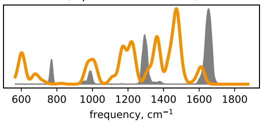


deprotonated HMDB0032988, spectral sim. = 392, #6

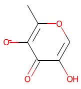

Struc. sim. = 0.32
5-Hydroxymaltol

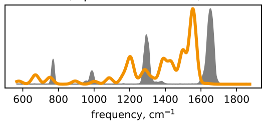


deprotonated HMDB0125218, spectral sim. = 350, #7

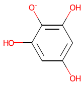

Struc. sim. = 0.36
benzene-1,2,3,5-tetrol

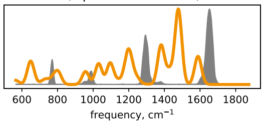

150 protonated HMDB0002432

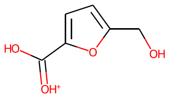

Sumiki's acid

Spectra of protonated HMDB0002432, spectral sim. = 949, #1

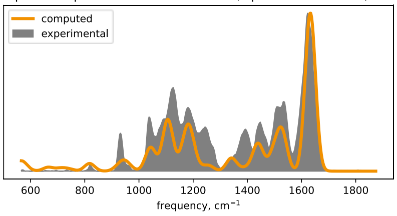


Structural similarity plot of protonated HMDB0002432

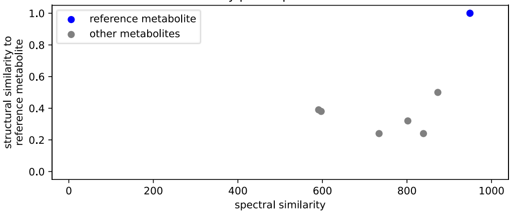


protonated HMDB0002432, spectral sim. = 949, #1

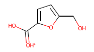

Struc. sim. = 1.00
Sumiki's acid

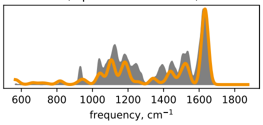

protonated HMDB0032923, spectral sim. = 873, #2

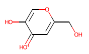

Struc. sim. = 0.50
Kojic acid

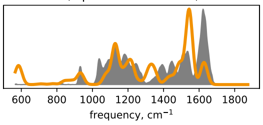

protonated HMDB0125218, spectral sim. = 839, #3

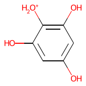

Struc. sim. = 0.24
benzene-1,2,3,5-tetrol

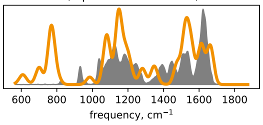

protonated HMDB0032988, spectral sim. = 802, #4

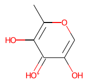

Struc. sim. = 0.32
5-Hydroxymaltol

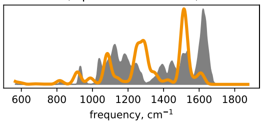

protonated HMDB0134002, spectral sim. = 734, #5

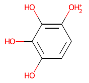

Struc. sim. = 0.24
benzene-1,2,3,4-tetrol

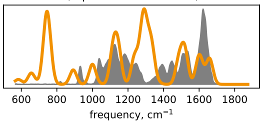

protonated HMDB0002349, spectral sim. = 597, #6

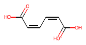

Struc. sim. = 0.38
trans-trans-Muconic acid

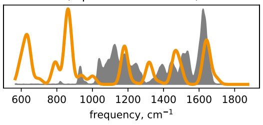

protonated HMDB0059762, spectral sim. = 591, #7

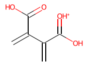

Struc. sim. = 0.39
2,3-Methylenesuccinic acid

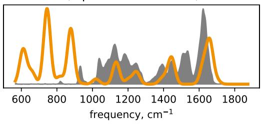

151 sodiated HMDB0002432

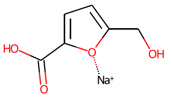

Sumiki's acid

Spectra of sodiated HMDB0002432, spectral sim. = 830, #1

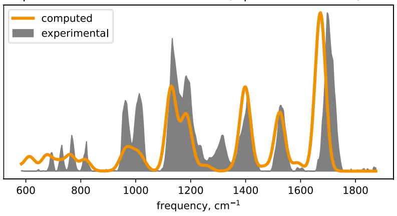


Structural similarity plot of sodiated HMDB0002432

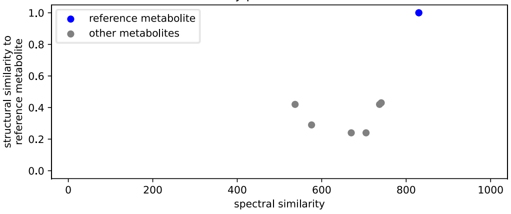


sodiated HMDB0002432, spectral sim. = 830, #1

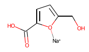

Struc. sim. = 1.00
Sumiki's acid

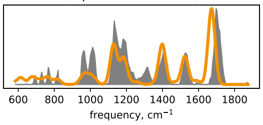

sodiated HMDB0059762, spectral sim. = 741, #2

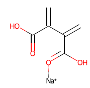

Struc. sim. = 0.43
2,3-Methylenesuccinic acid

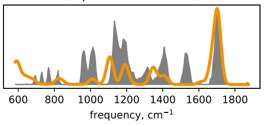

sodiated HMDB0002349, spectral sim. = 737, #3

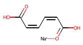

Struc. sim. = 0.42
trans-trans-Muconic acid

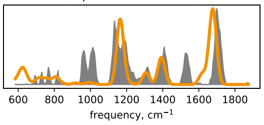

sodiated HMDB0125218, spectral sim. = 705, #4

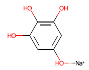

Struc. sim. = 0.24
benzene-1,2,3,5-tetrol

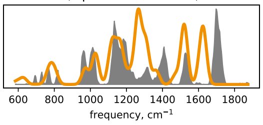

sodiated HMDB0134002, spectral sim. = 670, #5

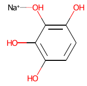

Struc. sim. = 0.24
benzene-1,2,3,4-tetrol

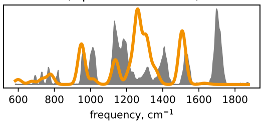

sodiated HMDB0032988, spectral sim. = 576, #6

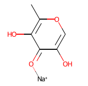

Struc. sim. = 0.29
5-Hydroxymaltol

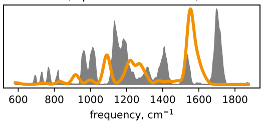

sodiated HMDB0032923, spectral sim. = 537, #7

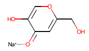

Struc. sim. = 0.42
Kojic acid

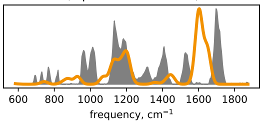

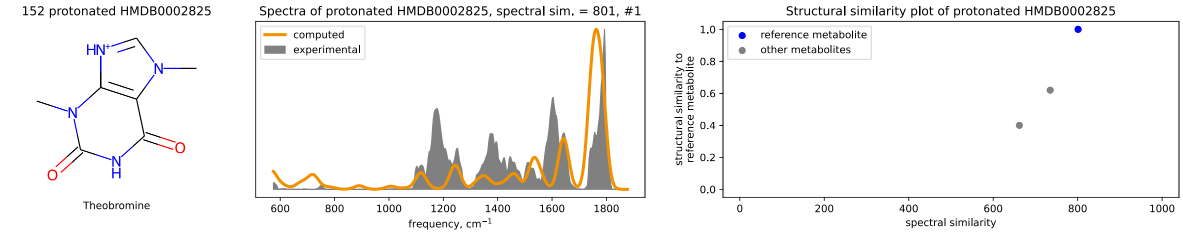
152 protonated HMDB0002825
Theobromine
Spectra of protonated HMDB0002825, spectral sim. = 801, #1
computed
experimental
frequency, cm⁻¹
Structural similarity plot of protonated HMDB0002825
reference metabolite
other metabolites
structural similarity to reference metabolite
spectral similarity

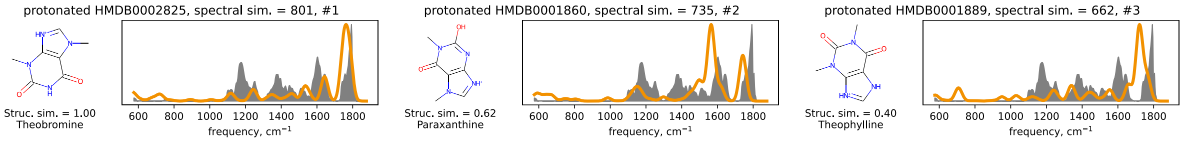
protonated HMDB0002825, spectral sim. = 801, #1
Struc. sim. = 1.00
Theobromine
protonated HMDB0001860, spectral sim. = 735, #2
Struc. sim. = 0.62
Paraxanthine
protonated HMDB0001889, spectral sim. = 662, #3
Struc. sim. = 0.40
Theophylline
frequency, cm⁻¹

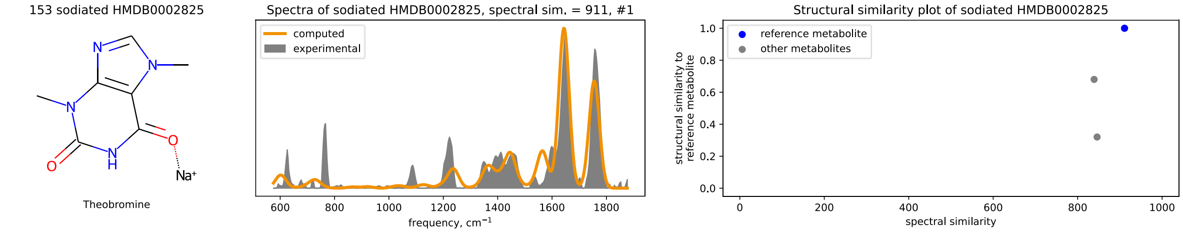
153 sodiated HMDB0002825
Theobromine
Spectra of sodiated HMDB0002825, spectral sim. = 911, #1
computed
experimental
frequency, cm⁻¹
Structural similarity plot of sodiated HMDB0002825
reference metabolite
other metabolites
structural similarity to reference metabolite
spectral similarity

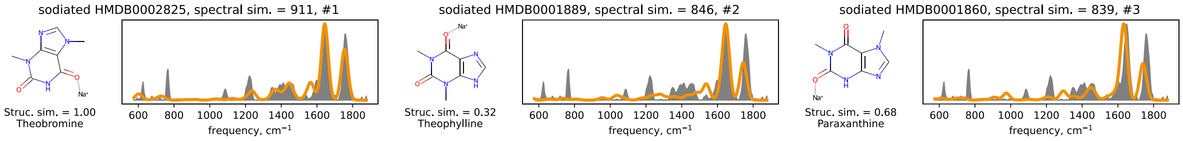
sodiated HMDB0002825, spectral sim. = 911, #1
Struc. sim. = 1.00
Theobromine
sodiated HMDB0001889, spectral sim. = 846, #2
Struc. sim. = 0.32
Theophylline
sodiated HMDB0001860, spectral sim. = 839, #3
Struc. sim. = 0.68
Paraxanthine
frequency, cm⁻¹

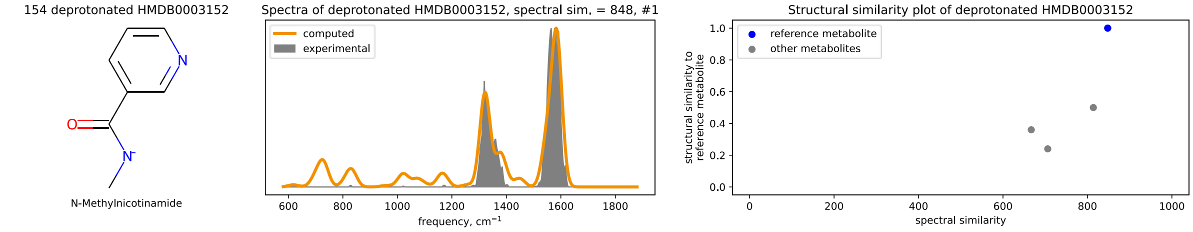
154 deprotonated HMDB0003152
N-Methylnicotinamide
Spectra of deprotonated HMDB0003152, spectral sim. = 848, #1
computed
experimental
frequency, cm⁻¹
Structural similarity plot of deprotonated HMDB0003152
reference metabolite
other metabolites
structural similarity to reference metabolite
spectral similarity

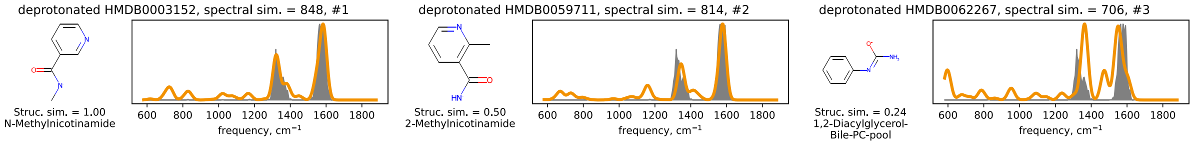
deprotonated HMDB0003152, spectral sim. = 848, #1
Struc. sim. = 1.00
N-Methylnicotinamide
deprotonated HMDB0059711, spectral sim. = 814, #2
Struc. sim. = 0.50
2-Methylnicotinamide
deprotonated HMDB0062267, spectral sim. = 706, #3
Struc. sim. = 0.24
1,2-Diacylglycerol-Bile-PC-pool
frequency, cm⁻¹

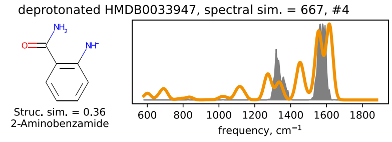
deprotonated HMDB0033947, spectral sim. = 667, #4
Struc. sim. = 0.36
2-Aminobenzamide
frequency, cm⁻¹

155 protonated HMDB0003152

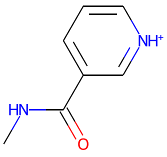


N-Methylnicotinamide

Spectra of protonated HMDB0003152, spectral sim. = 849, #1

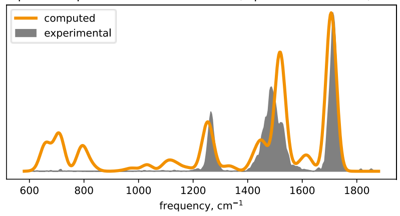


Structural similarity plot of protonated HMDB0003152

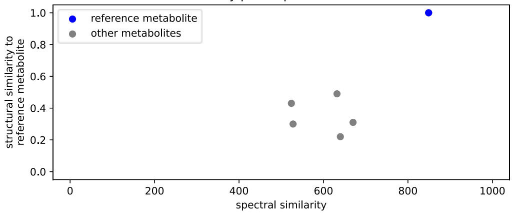


protonated HMDB0003152, spectral sim. = 849, #1

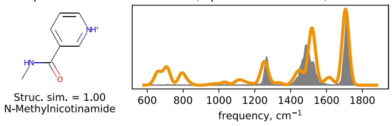

Struc. sim. = 1.00
N-Methylnicotinamide

protonated HMDB0030001, spectral sim. = 670, #2

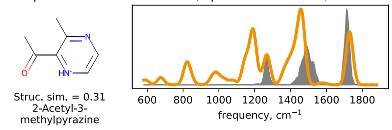

Struc. sim. = 0.31
2-Acetyl-3-methylpyrazine

protonated HMDB0062267, spectral sim. = 640, #3

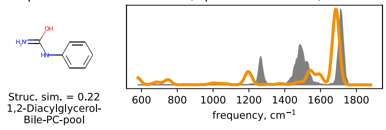

Struc. sim. = 0.22
1,2-Diacylglycerol-Bile-PC-pool

protonated HMDB0059711, spectral sim. = 632, #4

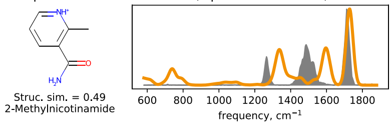

Struc. sim. = 0.49
2-Methylnicotinamide

protonated HMDB0037819, spectral sim. = 528, #5

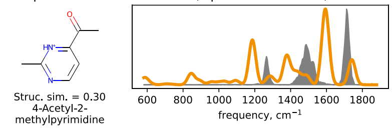

Struc. sim. = 0.30
4-Acetyl-2-methylpyrimidine

protonated HMDB0033947, spectral sim. = 524, #6

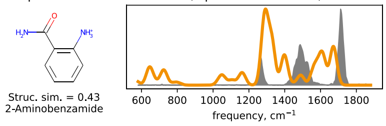

Struc. sim. = 0.43
2-Aminobenzamide

156 sodiated HMDB0003152

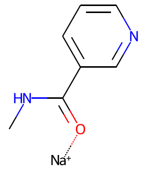


N-Methylnicotinamide

Spectra of sodiated HMDB0003152, spectral sim. = 917, #1

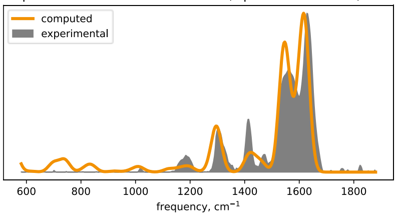


Structural similarity plot of sodiated HMDB0003152

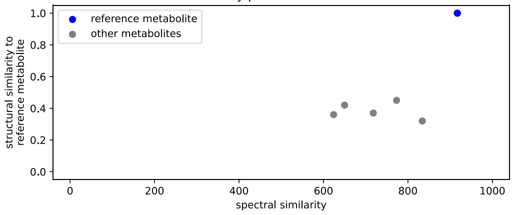


sodiated HMDB0003152, spectral sim. = 917, #1

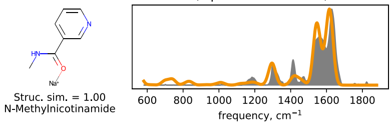

Struc. sim. = 1.00
N-Methylnicotinamide

sodiated HMDB0059711, spectral sim. = 834, #2

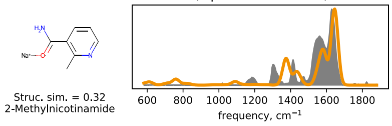

Struc. sim. = 0.32
2-Methylnicotinamide

sodiated HMDB0033947, spectral sim. = 773, #3

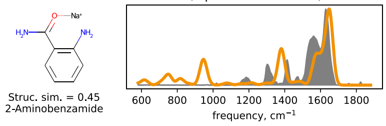

Struc. sim. = 0.45
2-Aminobenzamide

sodiated HMDB0062267, spectral sim. = 718, #4

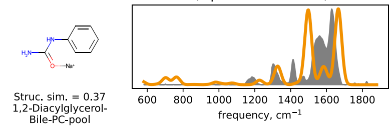

Struc. sim. = 0.37
1,2-Diacylglycerol-Bile-PC-pool

sodiated HMDB0037819, spectral sim. = 650, #5

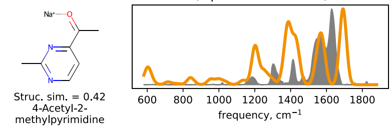

Struc. sim. = 0.42
4-Acetyl-2-methylpyrimidine

sodiated HMDB0030001, spectral sim. = 624, #6

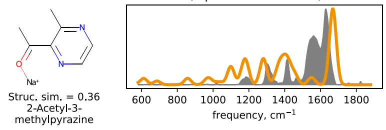

Struc. sim. = 0.36
2-Acetyl-3-methylpyrazine

157 deprotonated HMDB0003320

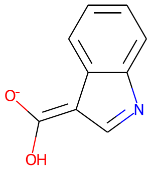


Indole-3-carboxylic acid

Spectra of deprotonated HMDB0003320, spectral sim. = 878, #1

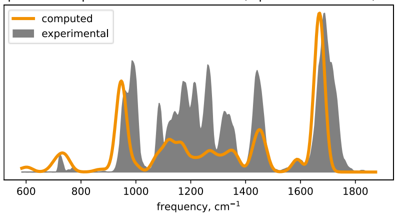


Structural similarity plot of deprotonated HMDB0003320

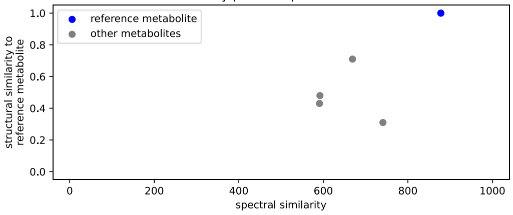


deprotonated HMDB0003320, spectral sim. = 878, #1

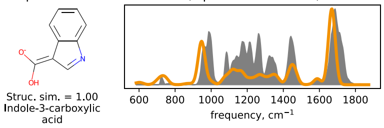

Struc. sim. = 1.00
Indole-3-carboxylic acid

deprotonated HMDB0002285, spectral sim. = 741, #2

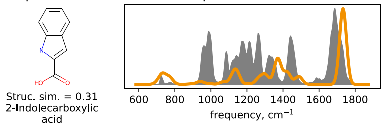

Struc. sim. = 0.31
2-Indolecarboxylic acid

deprotonated HMDB0031172, spectral sim. = 669, #3

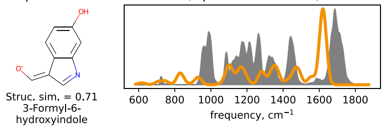

Struc. sim. = 0.71
3-Formyl-6-hydroxyindole

deprotonated HMDB0060289, spectral sim. = 592, #4

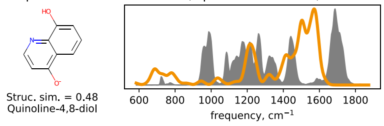

Struc. sim. = 0.48
Quinoline-4,8-diol

deprotonated HMDB0004077, spectral sim. = 591, #5

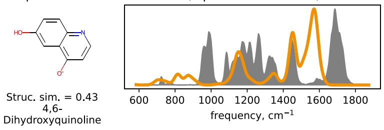

Struc. sim. = 0.43
4,6-Dihydroxyquinoline

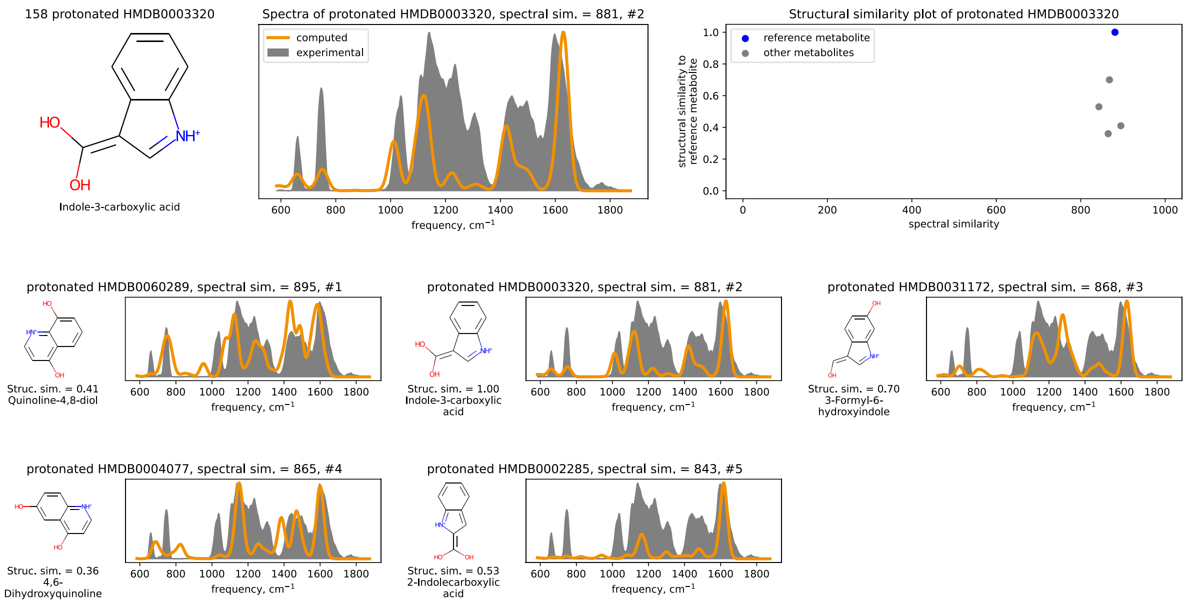
158 protonated HMDB0003320
Indole-3-carboxylic acid
Spectra of protonated HMDB0003320, spectral sim. = 881, #2
computed
experimental
frequency, cm⁻¹
Structural similarity plot of protonated HMDB0003320
reference metabolite
other metabolites
structural similarity to reference metabolite
spectral similarity
protonated HMDB0060289, spectral sim. = 895, #1
Struc. sim. = 0.41
Quinoline-4,8-diol
protonated HMDB0003320, spectral sim. = 881, #2
Struc. sim. = 1.00
Indole-3-carboxylic acid
protonated HMDB0031172, spectral sim. = 868, #3
Struc. sim. = 0.70
3-Formyl-6-hydroxyindole
protonated HMDB0004077, spectral sim. = 865, #4
Struc. sim. = 0.36
4,6-Dihydroxyquinoline
protonated HMDB0002285, spectral sim. = 843, #5
Struc. sim. = 0.53
2-Indolecarboxylic acid

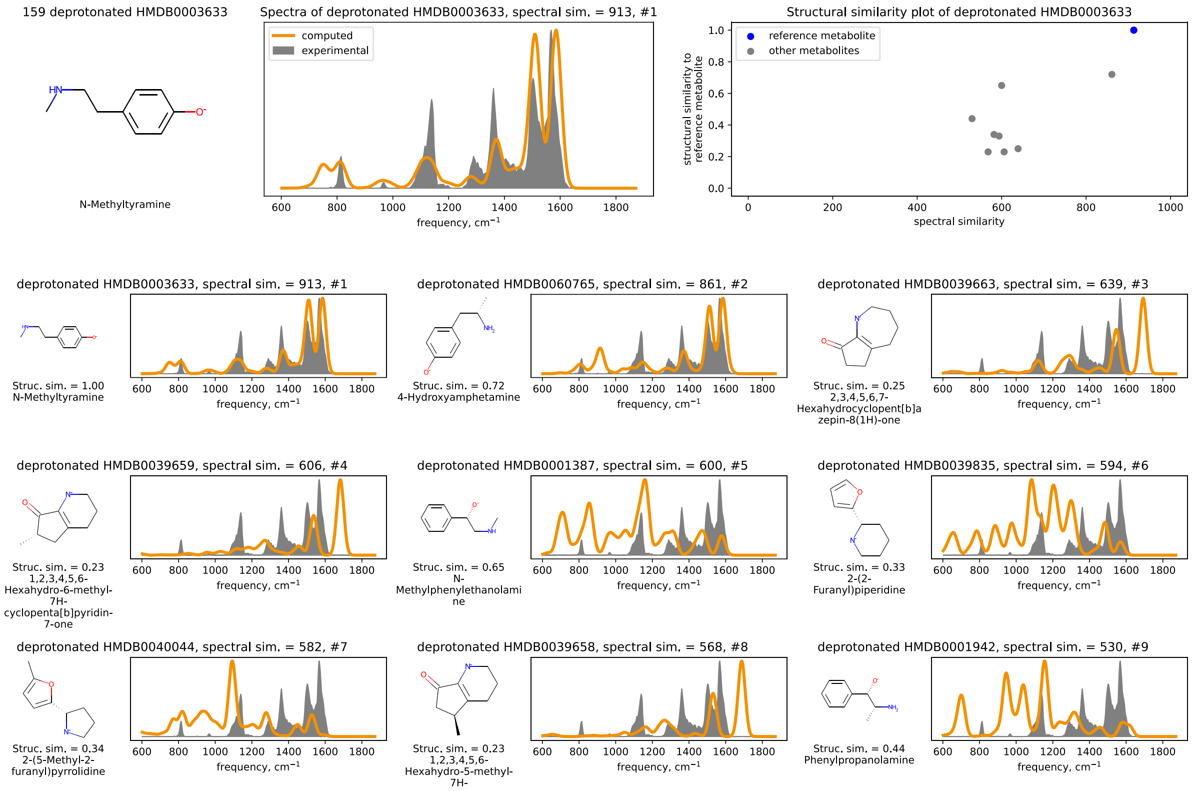
159 deprotonated HMDB0003633
N-Methyltyramine
Spectra of deprotonated HMDB0003633, spectral sim. = 913, #1
computed
experimental
frequency, cm⁻¹
Structural similarity plot of deprotonated HMDB0003633
reference metabolite
other metabolites
structural similarity to reference metabolite
spectral similarity
deprotonated HMDB0003633, spectral sim. = 913, #1
Struc. sim. = 1.00
N-Methyltyramine
deprotonated HMDB0060765, spectral sim. = 861, #2
Struc. sim. = 0.72
4-Hydroxyamphetamine
deprotonated HMDB0039663, spectral sim. = 639, #3
Struc. sim. = 0.25
2,3,4,5,6,7-Hexahydrocyclopent[b]azepin-8(1H)-one
deprotonated HMDB0039659, spectral sim. = 606, #4
Struc. sim. = 0.23
1,2,3,4,5,6-Hexahydro-6-methyl-7H-cyclopenta[b]pyridin-7-one
deprotonated HMDB0001387, spectral sim. = 600, #5
Struc. sim. = 0.65
N-Methylphenylethanolamine
deprotonated HMDB0039835, spectral sim. = 594, #6
Struc. sim. = 0.33
2-(2-Furanyl)piperidine
deprotonated HMDB0040044, spectral sim. = 582, #7
Struc. sim. = 0.34
2-(5-Methyl-2-furanyl)pyrrolidine
deprotonated HMDB0039658, spectral sim. = 568, #8
Struc. sim. = 0.23
1,2,3,4,5,6-Hexahydro-5-methyl-7H-
deprotonated HMDB0001942, spectral sim. = 530, #9
Struc. sim. = 0.44
Phenylpropanolamine

160 protonated HMDB0003633

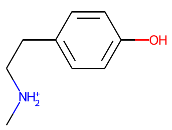

N-Methyltyramine

Spectra of protonated HMDB0003633, spectral sim. = 909, #1

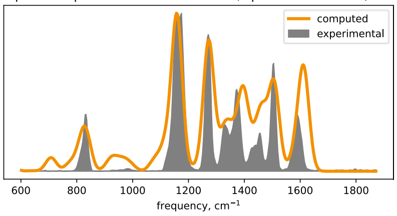


Structural similarity plot of protonated HMDB0003633

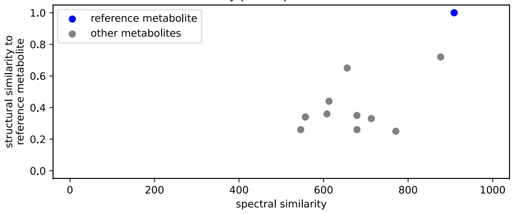


protonated HMDB0003633, spectral sim. = 909, #1

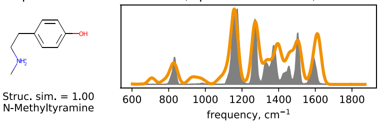

Struc. sim. = 1.00
N-Methyltyramine

protonated HMDB0060765, spectral sim. = 877, #2

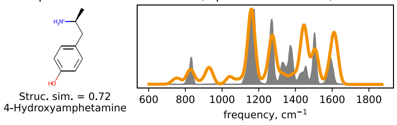

Struc. sim. = 0.72
4-Hydroxyamphetamine

protonated HMDB0040039, spectral sim. = 771, #3

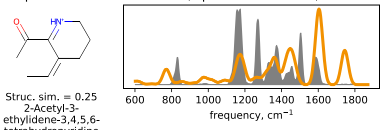

Struc. sim. = 0.25
2-Acetyl-3-ethylidene-3,4,5,6-tetrahydropyridine

protonated HMDB0039835, spectral sim. = 713, #4

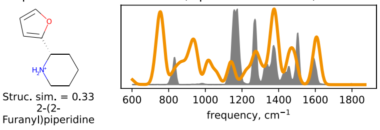

Struc. sim. = 0.33
2-(2-Furanyl)piperidine

protonated HMDB0040044, spectral sim. = 679, #5

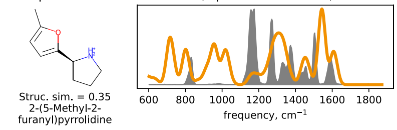

Struc. sim. = 0.35
2-(5-Methyl-2-furanyl)pyrrolidine

protonated HMDB0040037, spectral sim. = 679, #6

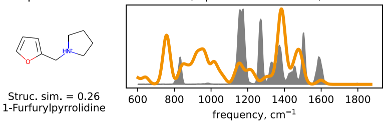

Struc. sim. = 0.26
1-Furfurylpyrrolidine

protonated HMDB0001387, spectral sim. = 656, #7

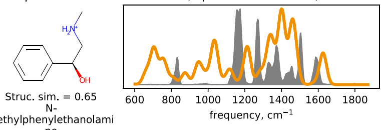

Struc. sim. = 0.65
N-Methylphenylethanolamine

protonated HMDB0001942, spectral sim. = 613, #8

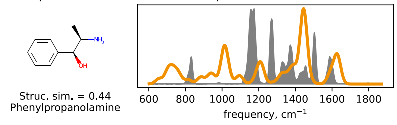

Struc. sim. = 0.44
Phenylpropanolamine

protonated HMDB0039663, spectral sim. = 608, #9

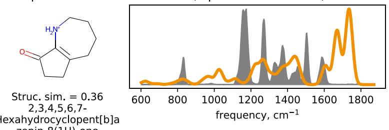

Struc. sim. = 0.36
2,3,4,5,6,7-Hexahydrocyclopent[b]azepin-8(1H)-one

protonated HMDB0039658, spectral sim. = 557, #10

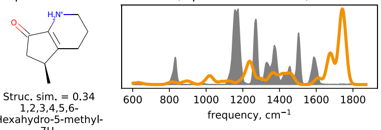

Struc. sim. = 0.34
1,2,3,4,5,6-Hexahydro-5-methyl-7H-
[illegible]

protonated HMDB0039659, spectral sim. = 557, #11

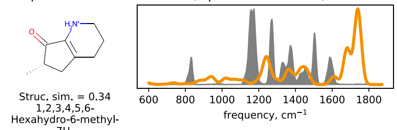

Struc. sim. = 0.34
1,2,3,4,5,6-Hexahydro-6-methyl-7H-
[illegible]

protonated HMDB0039674, spectral sim. = 546, #12

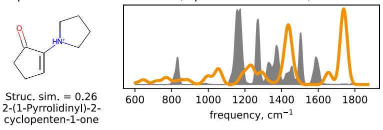

Struc. sim. = 0.26
2-(1-Pyrrolidinyl)-2-cyclopenten-1-one

161 deprotonated HMDB0004095

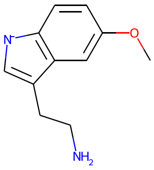

5-Methoxytryptamine

Spectra of deprotonated HMDB0004095, spectral sim. = 954, #1

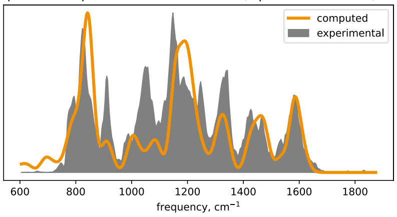


Structural similarity plot of deprotonated HMDB0004095

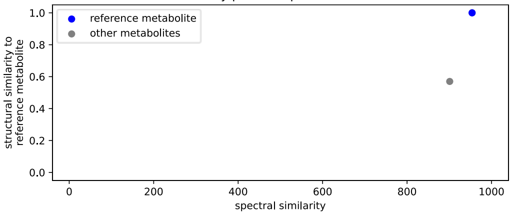


deprotonated HMDB0004095, spectral sim. = 954, #1

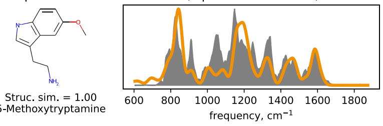

Struc. sim. = 1.00
5-Methoxytryptamine

deprotonated HMDB0004369, spectral sim. = 901, #2

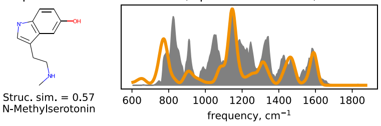

Struc. sim. = 0.57
N-Methylserotonin

162 protonated HMDB0004095

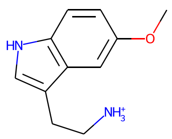

5-Methoxytryptamine

Spectra of protonated HMDB0004095, spectral sim. = 961, #1

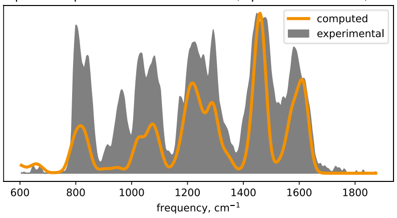


Structural similarity plot of protonated HMDB0004095

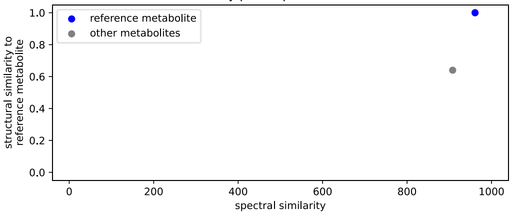


protonated HMDB0004095, spectral sim. = 961, #1

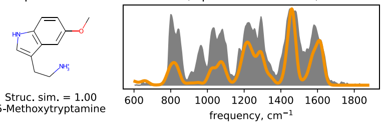

Struc. sim. = 1.00
5-Methoxytryptamine

protonated HMDB0004369, spectral sim. = 908, #2

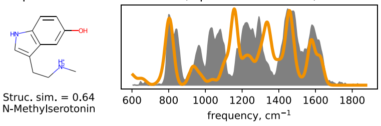

Struc. sim. = 0.64
N-Methylserotonin

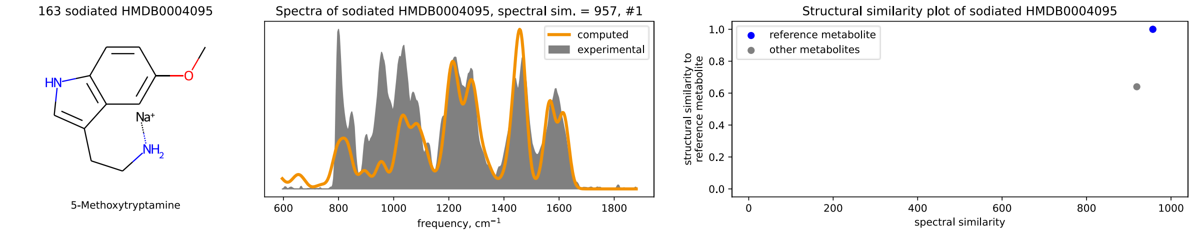
163 sodiated HMDB0004095
5-Methoxytryptamine
Spectra of sodiated HMDB0004095, spectral sim. = 957, #1
computed
experimental
frequency, cm⁻¹
Structural similarity plot of sodiated HMDB0004095
reference metabolite
other metabolites
structural similarity to reference metabolite
spectral similarity

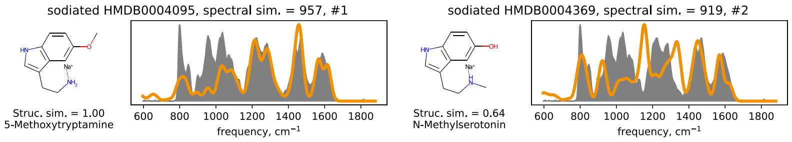
sodiated HMDB0004095, spectral sim. = 957, #1
Struc. sim. = 1.00
5-Methoxytryptamine
frequency, cm⁻¹
sodiated HMDB0004369, spectral sim. = 919, #2
Struc. sim. = 0.64
N-Methylserotonin
frequency, cm⁻¹

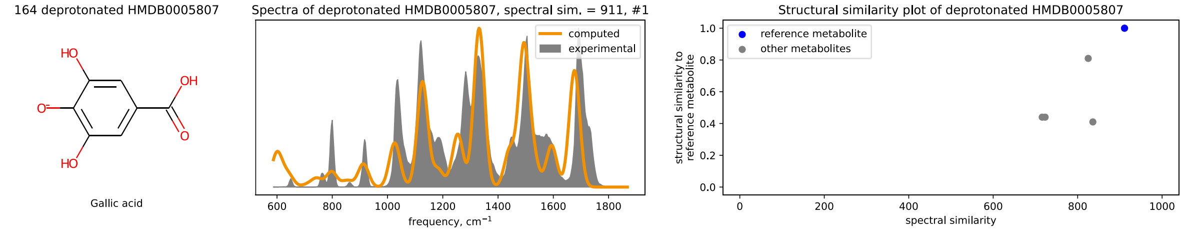
164 deprotonated HMDB0005807
Gallic acid
Spectra of deprotonated HMDB0005807, spectral sim. = 911, #1
computed
experimental
frequency, cm⁻¹
Structural similarity plot of deprotonated HMDB0005807
reference metabolite
other metabolites
structural similarity to reference metabolite
spectral similarity

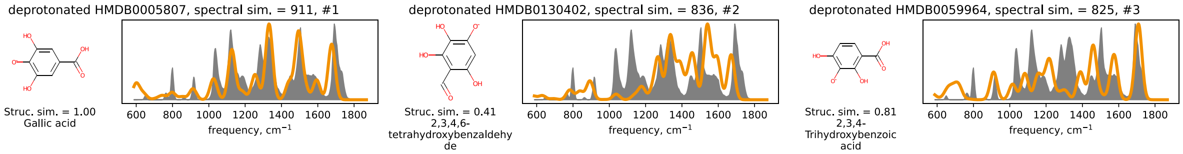
deprotonated HMDB0005807, spectral sim. = 911, #1
Struc. sim. = 1.00
Gallic acid
frequency, cm⁻¹
deprotonated HMDB0130402, spectral sim. = 836, #2
Struc. sim. = 0.41
2,3,4,6-tetrahydroxybenzaldehyde
frequency, cm⁻¹
deprotonated HMDB0059964, spectral sim. = 825, #3
Struc. sim. = 0.81
2,3,4-Trihydroxybenzoic acid
frequency, cm⁻¹

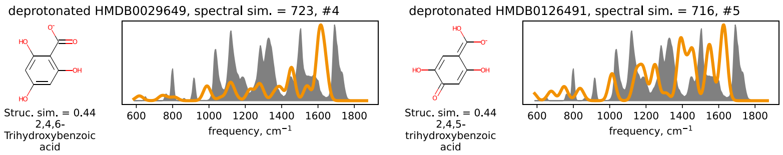
deprotonated HMDB0029649, spectral sim. = 723, #4
Struc. sim. = 0.44
2,4,6-Trihydroxybenzoic acid
frequency, cm⁻¹
deprotonated HMDB0126491, spectral sim. = 716, #5
Struc. sim. = 0.44
2,4,5-trihydroxybenzoic acid
frequency, cm⁻¹

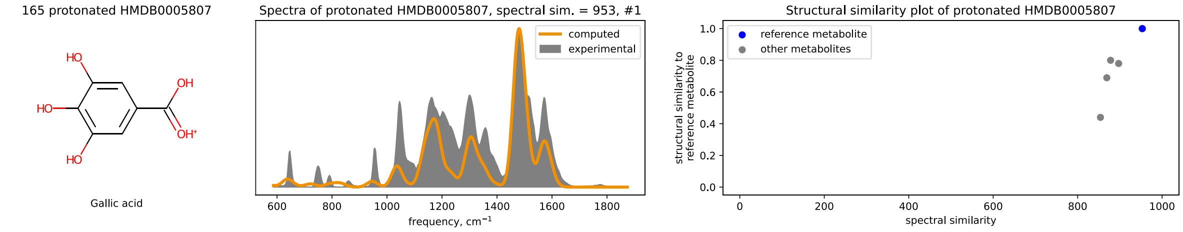
165 protonated HMDB0005807
Gallic acid
Spectra of protonated HMDB0005807, spectral sim. = 953, #1
computed
experimental
frequency, cm⁻¹
Structural similarity plot of protonated HMDB0005807
reference metabolite
other metabolites
structural similarity to reference metabolite
spectral similarity

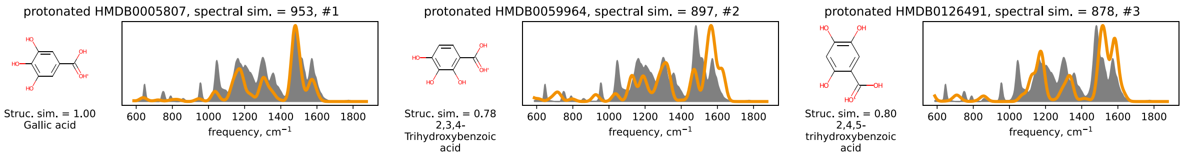
protonated HMDB0005807, spectral sim. = 953, #1
Struc. sim. = 1.00
Gallic acid
frequency, cm⁻¹
protonated HMDB0059964, spectral sim. = 897, #2
Struc. sim. = 0.78
2,3,4-Trihydroxybenzoic acid
frequency, cm⁻¹
protonated HMDB0126491, spectral sim. = 878, #3
Struc. sim. = 0.80
2,4,5-trihydroxybenzoic acid
frequency, cm⁻¹

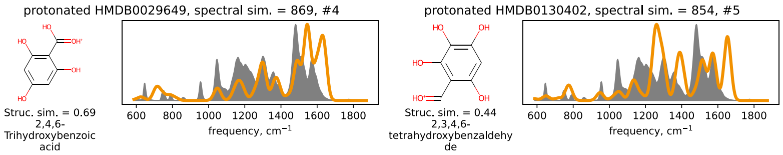
protonated HMDB0029649, spectral sim. = 869, #4
Struc. sim. = 0.69
2,4,6-Trihydroxybenzoic acid
frequency, cm⁻¹
protonated HMDB0130402, spectral sim. = 854, #5
Struc. sim. = 0.44
2,3,4,6-tetrahydroxybenzaldehyde
frequency, cm⁻¹

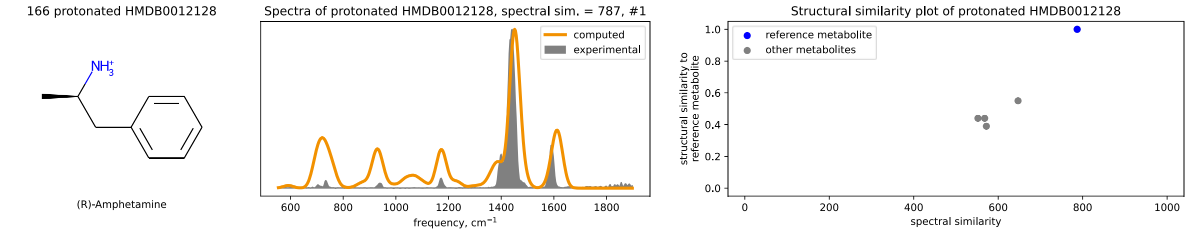
166 protonated HMDB0012128
(R)-Amphetamine
Spectra of protonated HMDB0012128, spectral sim. = 787, #1
computed
experimental
frequency, cm⁻¹
Structural similarity plot of protonated HMDB0012128
reference metabolite
other metabolites
structural similarity to reference metabolite
spectral similarity

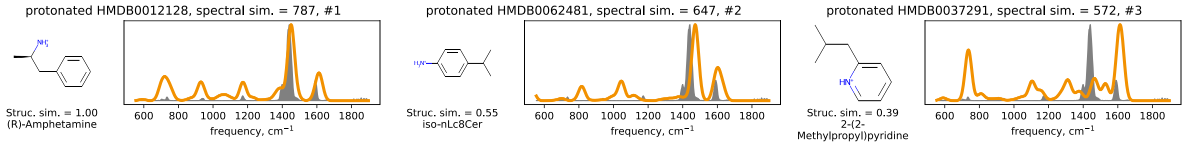
protonated HMDB0012128, spectral sim. = 787, #1
Struc. sim. = 1.00
(R)-Amphetamine
protonated HMDB0062481, spectral sim. = 647, #2
Struc. sim. = 0.55
iso-nLc8Cer
protonated HMDB0037291, spectral sim. = 572, #3
Struc. sim. = 0.39
2-(2-Methylpropyl)pyridine
frequency, cm⁻¹

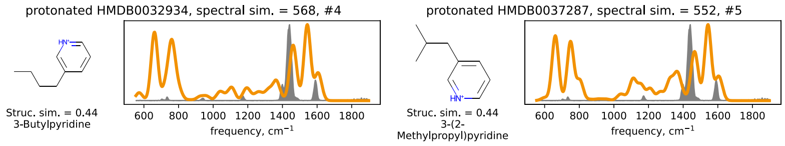
protonated HMDB0032934, spectral sim. = 568, #4
Struc. sim. = 0.44
3-Butylpyridine
protonated HMDB0037287, spectral sim. = 552, #5
Struc. sim. = 0.44
3-(2-Methylpropyl)pyridine
frequency, cm⁻¹

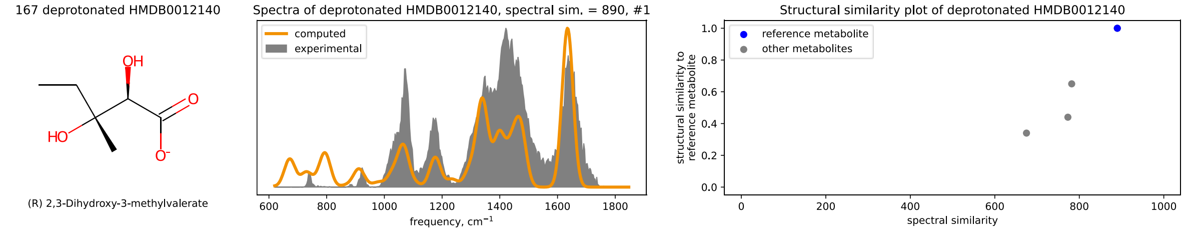
167 deprotonated HMDB0012140
(R) 2,3-Dihydroxy-3-methylvalerate
Spectra of deprotonated HMDB0012140, spectral sim. = 890, #1
computed
experimental
frequency, cm⁻¹
Structural similarity plot of deprotonated HMDB0012140
reference metabolite
other metabolites
structural similarity to reference metabolite
spectral similarity

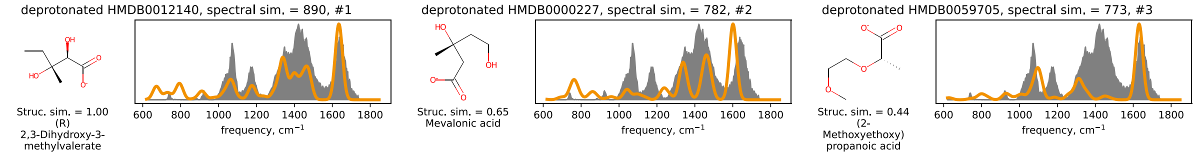
deprotonated HMDB0012140, spectral sim. = 890, #1
Struc. sim. = 1.00
(R) 2,3-Dihydroxy-3-methylvalerate
deprotonated HMDB0000227, spectral sim. = 782, #2
Struc. sim. = 0.65
Mevalonic acid
deprotonated HMDB0059705, spectral sim. = 773, #3
Struc. sim. = 0.44
(2-Methoxyethoxy) propanoic acid
frequency, cm⁻¹

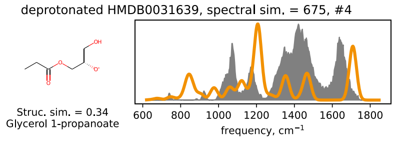
deprotonated HMDB0031639, spectral sim. = 675, #4
Struc. sim. = 0.34
Glycerol 1-propanoate
frequency, cm⁻¹

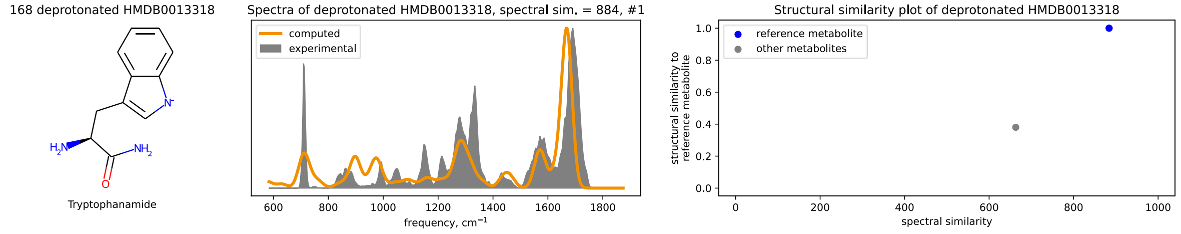
168 deprotonated HMDB0013318
Tryptophanamide
Spectra of deprotonated HMDB0013318, spectral sim. = 884, #1
computed
experimental
frequency, cm⁻¹
Structural similarity plot of deprotonated HMDB0013318
reference metabolite
other metabolites
structural similarity to reference metabolite
spectral similarity

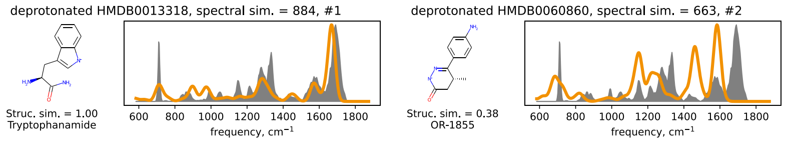
deprotonated HMDB0013318, spectral sim. = 884, #1
Struc. sim. = 1.00
Tryptophanamide
deprotonated HMDB0060860, spectral sim. = 663, #2
Struc. sim. = 0.38
OR-1855
frequency, cm⁻¹

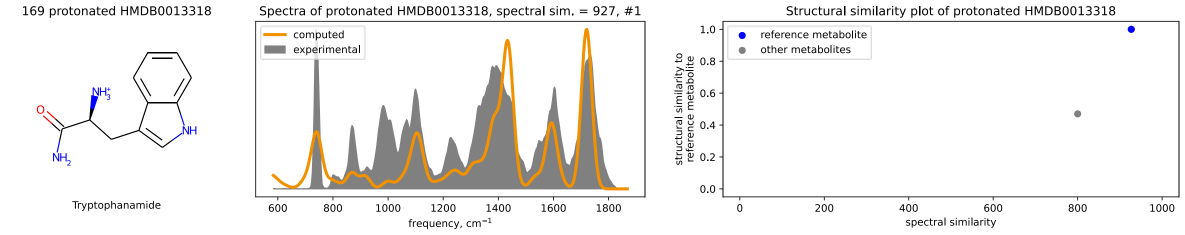
169 protonated HMDB0013318
Tryptophanamide
Spectra of protonated HMDB0013318, spectral sim. = 927, #1
computed
experimental
frequency, cm$^{-1}$
Structural similarity plot of protonated HMDB0013318
reference metabolite
other metabolites
structural similarity to reference metabolite
spectral similarity

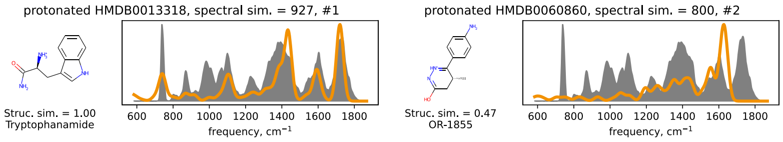
protonated HMDB0013318, spectral sim. = 927, #1
Struc. sim. = 1.00
Tryptophanamide
protonated HMDB0060860, spectral sim. = 800, #2
Struc. sim. = 0.47
OR-1855
frequency, cm$^{-1}$

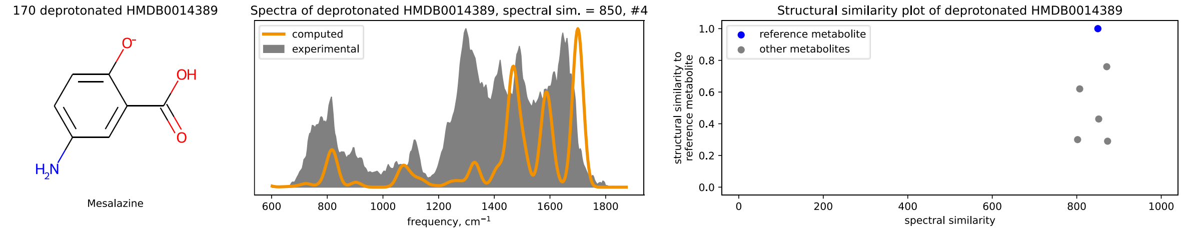
170 deprotonated HMDB0014389
Mesalazine
Spectra of deprotonated HMDB0014389, spectral sim. = 850, #4
computed
experimental
frequency, cm$^{-1}$
Structural similarity plot of deprotonated HMDB0014389
reference metabolite
other metabolites
structural similarity to reference metabolite
spectral similarity

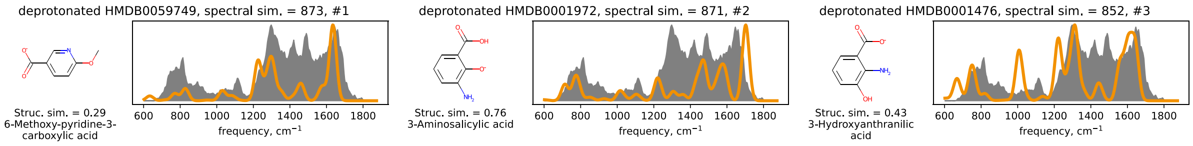
deprotonated HMDB0059749, spectral sim. = 873, #1
Struc. sim. = 0.29
6-Methoxy-pyridine-3-carboxylic acid
deprotonated HMDB0001972, spectral sim. = 871, #2
Struc. sim. = 0.76
3-Aminosalicylic acid
deprotonated HMDB0001476, spectral sim. = 852, #3
Struc. sim. = 0.43
3-Hydroxyanthranilic acid
frequency, cm$^{-1}$

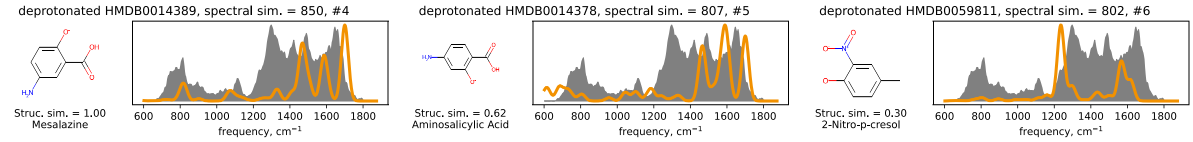
deprotonated HMDB0014389, spectral sim. = 850, #4
Struc. sim. = 1.00
Mesalazine
deprotonated HMDB0014378, spectral sim. = 807, #5
Struc. sim. = 0.62
Aminosalicylic Acid
deprotonated HMDB0059811, spectral sim. = 802, #6
Struc. sim. = 0.30
2-Nitro-p-cresol
frequency, cm$^{-1}$

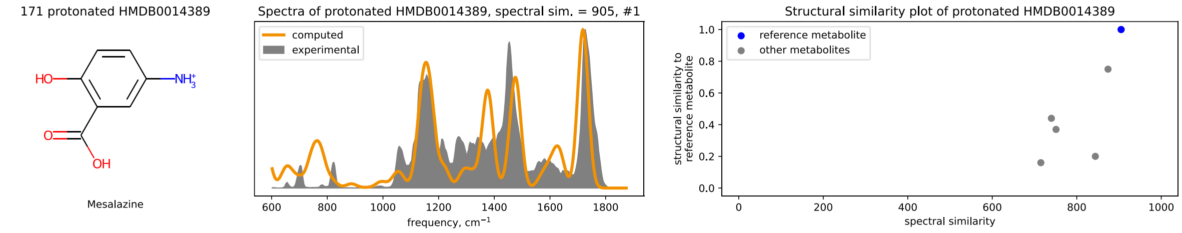
171 protonated HMDB0014389
Mesalazine
Spectra of protonated HMDB0014389, spectral sim. = 905, #1
computed
experimental
frequency, cm$^{-1}$
Structural similarity plot of protonated HMDB0014389
reference metabolite
other metabolites
structural similarity to reference metabolite
spectral similarity

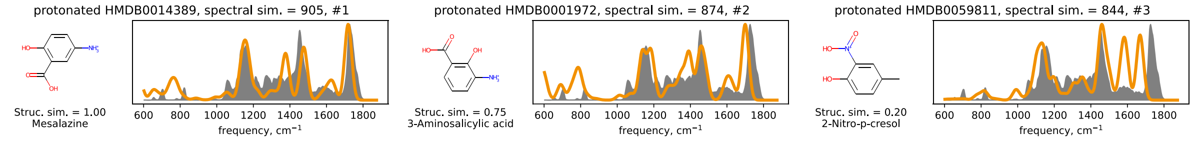
protonated HMDB0014389, spectral sim. = 905, #1
Struc. sim. = 1.00
Mesalazine
protonated HMDB0001972, spectral sim. = 874, #2
Struc. sim. = 0.75
3-Aminosalicylic acid
protonated HMDB0059811, spectral sim. = 844, #3
Struc. sim. = 0.20
2-Nitro-p-cresol
frequency, cm$^{-1}$

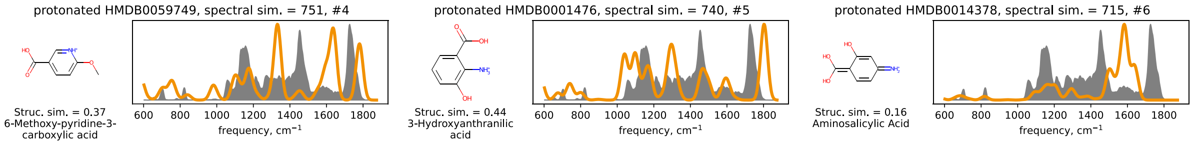
protonated HMDB0059749, spectral sim. = 751, #4
Struc. sim. = 0.37
6-Methoxy-pyridine-3-carboxylic acid
protonated HMDB0001476, spectral sim. = 740, #5
Struc. sim. = 0.44
3-Hydroxyanthranilic acid
protonated HMDB0014378, spectral sim. = 715, #6
Struc. sim. = 0.16
Aminosalicylic Acid
frequency, cm$^{-1}$

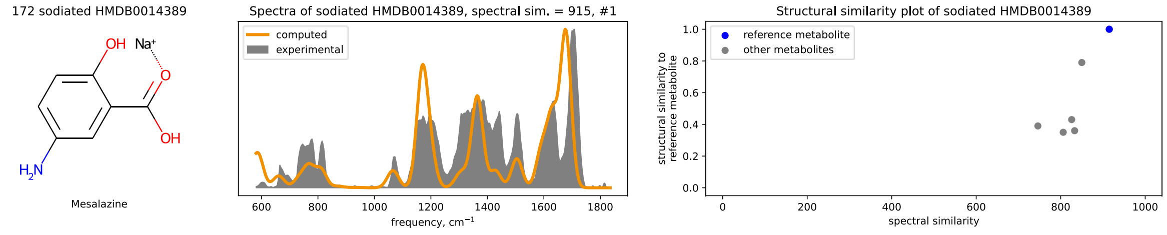
172 sodiated HMDB0014389
Mesalazine
Spectra of sodiated HMDB0014389, spectral sim. = 915, #1
computed
experimental
frequency, cm⁻¹
Structural similarity plot of sodiated HMDB0014389
reference metabolite
other metabolites
structural similarity to reference metabolite
spectral similarity

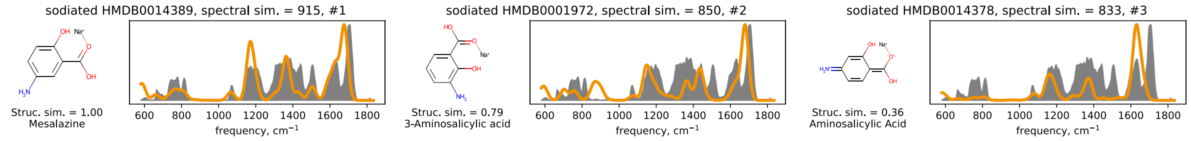
sodiated HMDB0014389, spectral sim. = 915, #1
Struc. sim. = 1.00
Mesalazine
sodiated HMDB0001972, spectral sim. = 850, #2
Struc. sim. = 0.79
3-Aminosalicylic acid
sodiated HMDB0014378, spectral sim. = 833, #3
Struc. sim. = 0.36
Aminosalicylic Acid
frequency, cm⁻¹

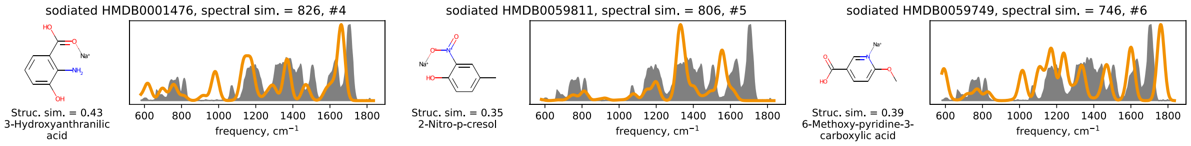
sodiated HMDB0001476, spectral sim. = 826, #4
Struc. sim. = 0.43
3-Hydroxyanthranilic acid
sodiated HMDB0059811, spectral sim. = 806, #5
Struc. sim. = 0.35
2-Nitro-p-cresol
sodiated HMDB0059749, spectral sim. = 746, #6
Struc. sim. = 0.39
6-Methoxy-pyridine-3-carboxylic acid
frequency, cm⁻¹

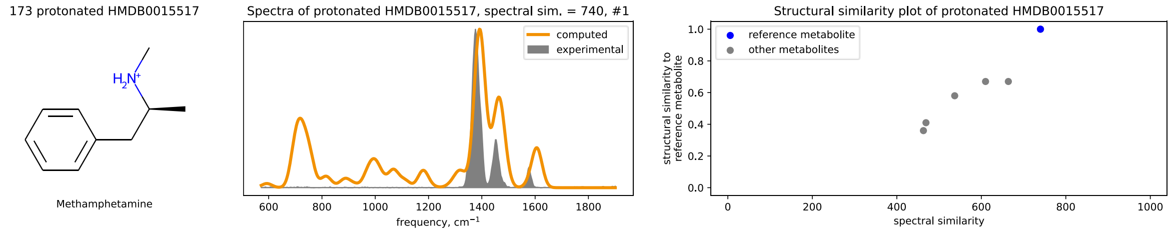
173 protonated HMDB0015517
Methamphetamine
Spectra of protonated HMDB0015517, spectral sim. = 740, #1
computed
experimental
frequency, cm⁻¹
Structural similarity plot of protonated HMDB0015517
reference metabolite
other metabolites
structural similarity to reference metabolite
spectral similarity

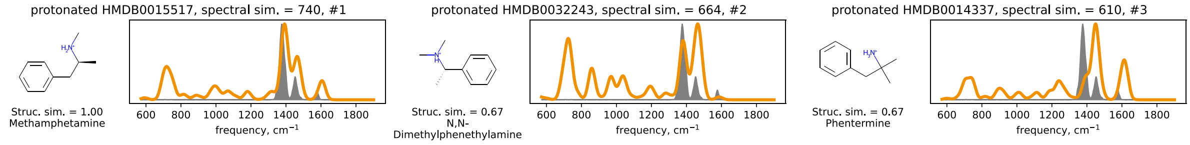
protonated HMDB0015517, spectral sim. = 740, #1
Struc. sim. = 1.00
Methamphetamine
protonated HMDB0032243, spectral sim. = 664, #2
Struc. sim. = 0.67
N,N-Dimethylphenethylamine
protonated HMDB0014337, spectral sim. = 610, #3
Struc. sim. = 0.67
Phentermine
frequency, cm⁻¹

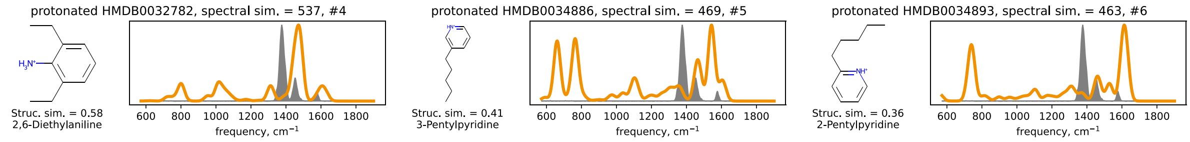
protonated HMDB0032782, spectral sim. = 537, #4
Struc. sim. = 0.58
2,6-Diethylaniline
protonated HMDB0034886, spectral sim. = 469, #5
Struc. sim. = 0.41
3-Pentylpyridine
protonated HMDB0034893, spectral sim. = 463, #6
Struc. sim. = 0.36
2-Pentylpyridine
frequency, cm⁻¹

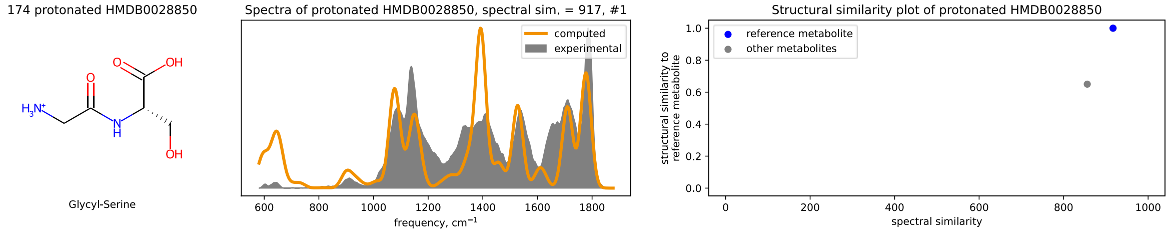
174 protonated HMDB0028850
Glycyl-Serine
Spectra of protonated HMDB0028850, spectral sim. = 917, #1
computed
experimental
frequency, cm⁻¹
Structural similarity plot of protonated HMDB0028850
reference metabolite
other metabolites
structural similarity to reference metabolite
spectral similarity

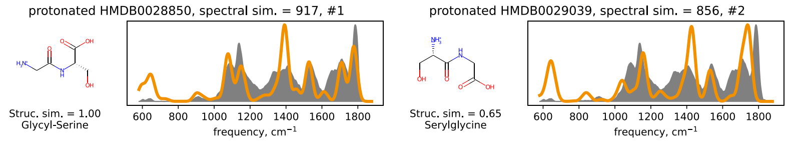
protonated HMDB0028850, spectral sim. = 917, #1
Struc. sim. = 1.00
Glycyl-Serine
protonated HMDB0029039, spectral sim. = 856, #2
Struc. sim. = 0.65
Serylglycine
frequency, cm⁻¹

175 protonated HMDB0031861

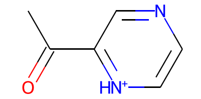


2-Acetylpyrazine

Spectra of protonated HMDB0031861, spectral sim. = 822, #1

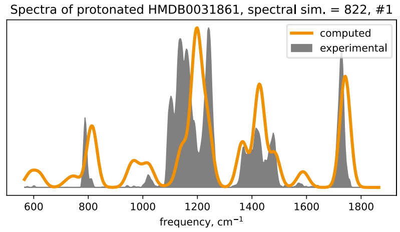


Structural similarity plot of protonated HMDB0031861

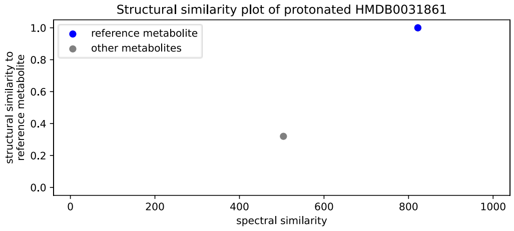


protonated HMDB0031861, spectral sim. = 822, #1

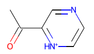

Struc. sim. = 1.00
2-Acetylpyrazine

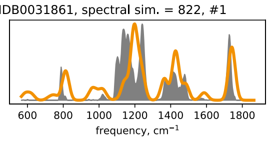


protonated HMDB0001406, spectral sim. = 504, #2

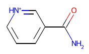

Struc. sim. = 0.32
Niacinamide

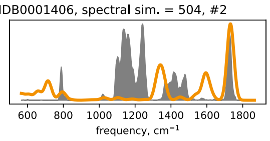


176 protonated HMDB0036458

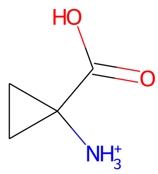


1-Aminocyclopropanecarboxylic acid

Spectra of protonated HMDB0036458, spectral sim. = 874, #1

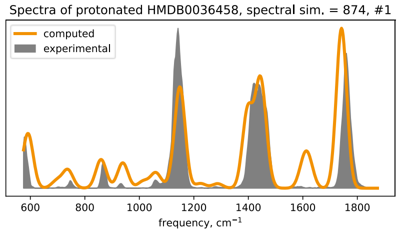


Structural similarity plot of protonated HMDB0036458

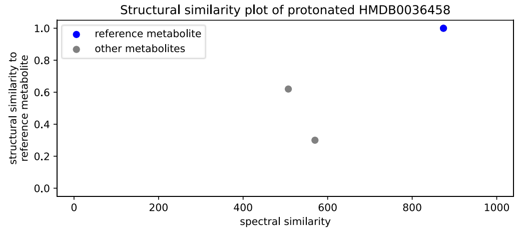


protonated HMDB0036458, spectral sim. = 874, #1

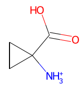

Struc. sim. = 1.00
1-
Aminocyclopropanecarbo
xylic acid

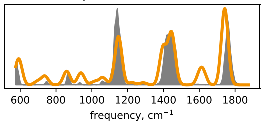


protonated HMDB0029387, spectral sim. = 570, #2

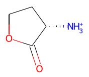

Struc. sim. = 0.30
L-3-Aminodihydro-
2(3H)-furanone

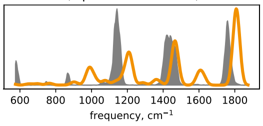


protonated HMDB0029615, spectral sim. = 507, #3

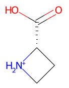

Struc. sim. = 0.62
(S)-2-
Azetidinecarboxylic
acid

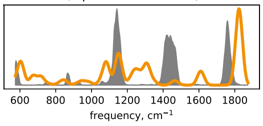


177 sodiated HMDB0036458

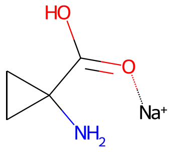


1-Aminocyclopropanecarboxylic acid

Spectra of sodiated HMDB0036458, spectral sim. = 863, #1

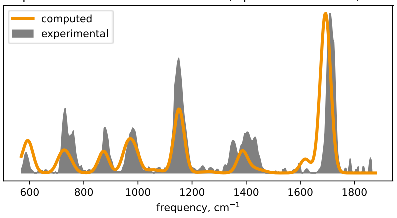


Structural similarity plot of sodiated HMDB0036458

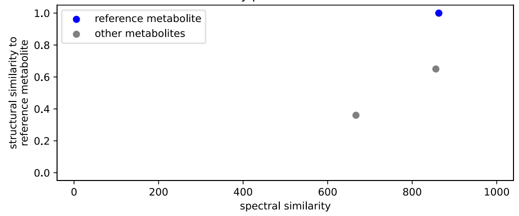


sodiated HMDB0036458, spectral sim. = 863, #1

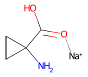

Struc. sim. = 1.00
1-
Aminocyclopropanecarbo
xylic acid

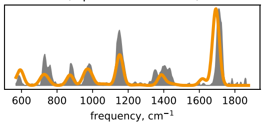


sodiated HMDB0029615, spectral sim. = 856, #2

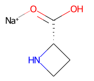

Struc. sim. = 0.65
(S)-2-
Azetidinecarboxylic
acid

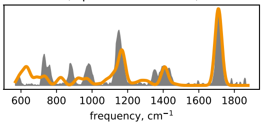


sodiated HMDB0029387, spectral sim. = 667, #3

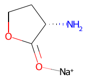

Struc. sim. = 0.36
L-3-Aminodihydro-
2(3H)-furanone

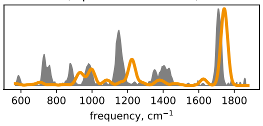

178 protonated HMDB0041923

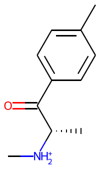

Mephedrone

Spectra of protonated HMDB0041923, spectral sim. = 840, #1

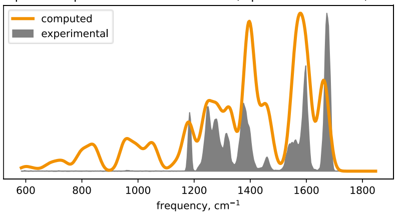


Structural similarity plot of protonated HMDB0041923

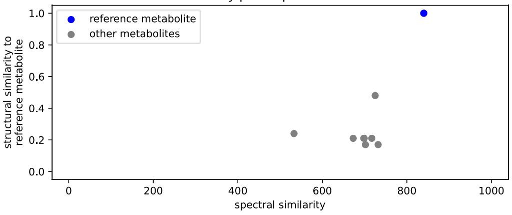


protonated HMDB0041923, spectral sim. = 840, #1

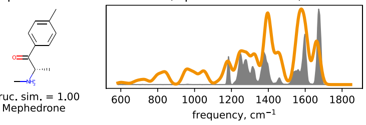

Struc. sim. = 1.00
Mephedrone

protonated HMDB0040027, spectral sim. = 732, #2

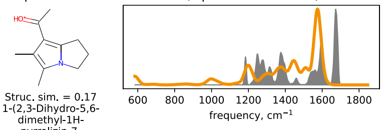

Struc. sim. = 0.17
1-(2,3-Dihydro-5,6-dimethyl-1H-pyrrolizin-7-yl)ethanone

protonated HMDB0039664, spectral sim. = 725, #3

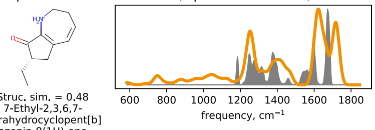

Struc. sim. = 0.48
7-Ethyl-2,3,6,7-tetrahydrocyclopent[b]azepin-8(1H)-one

protonated HMDB0040017, spectral sim. = 717, #4

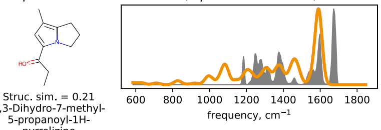

Struc. sim. = 0.21
2,3-Dihydro-7-methyl-5-propanoyl-1H-pyrrolizine

protonated HMDB0040020, spectral sim. = 702, #5

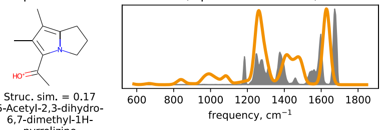

Struc. sim. = 0.17
5-Acetyl-2,3-dihydro-6,7-dimethyl-1H-pyrrolizine

protonated HMDB0040019, spectral sim. = 699, #6

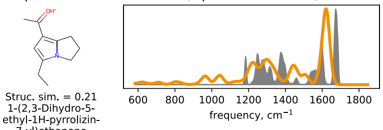

Struc. sim. = 0.21
1-(2,3-Dihydro-5-ethyl-1H-pyrrolizin-7-yl)ethanone

protonated HMDB0040018, spectral sim. = 698, #7

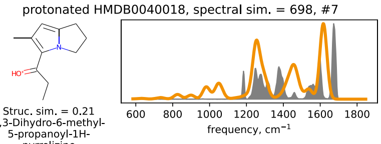

Struc. sim. = 0.21
2,3-Dihydro-6-methyl-5-propanoyl-1H-pyrrolizine

protonated HMDB0040014, spectral sim. = 673, #8

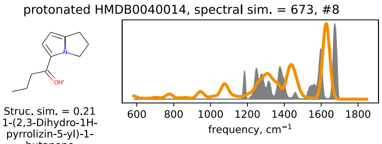

Struc. sim. = 0.21
1-(2,3-Dihydro-1H-pyrrolizin-5-yl)-1-butanone

protonated HMDB0033484, spectral sim. = 533, #9

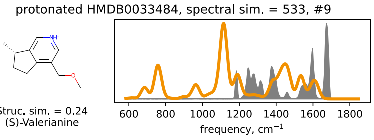

Struc. sim. = 0.24
(S)-Valerianine

179 protonated HMDB0041931

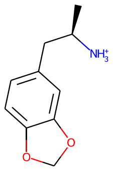

3,4-Methylenedioxyamphetamine

Spectra of protonated HMDB0041931, spectral sim. = 924, #1

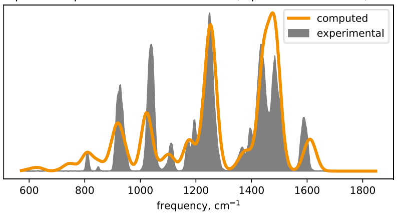


Structural similarity plot of protonated HMDB0041931

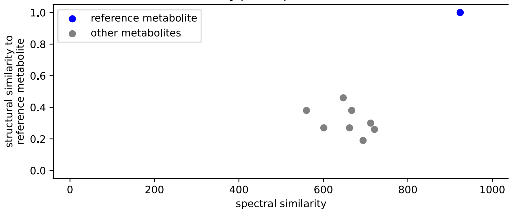


protonated HMDB0041931, spectral sim. = 924, #1

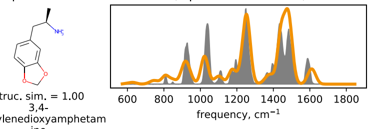

Struc. sim. = 1.00
3,4-Methylenedioxyamphetamine

protonated HMDB0001189, spectral sim. = 721, #2

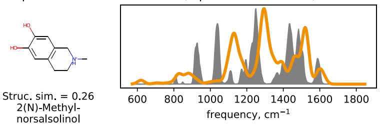

Struc. sim. = 0.26
2(N)-Methyl-norsalsolinol

protonated HMDB0005199, spectral sim. = 712, #3

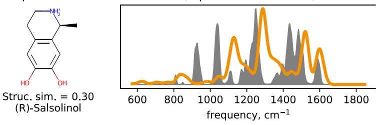

Struc. sim. = 0.30
(R)-Salsolinol

protonated HMDB0040021, spectral sim. = 694, #4

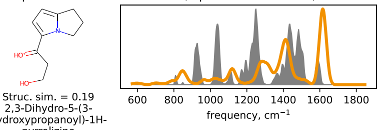

Struc. sim. = 0.19
2,3-Dihydro-5-(3-hydroxypropanoyl)-1H-pyrrolizine

protonated HMDB0033763, spectral sim. = 667, #5

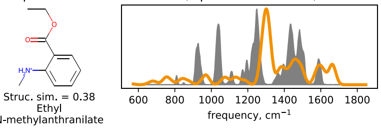

Struc. sim. = 0.38
Ethyl N-methylanthranilate

protonated HMDB0030372, spectral sim. = 662, #6

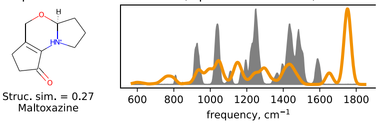

Struc. sim. = 0.27
Maltoxazine

protonated HMDB0029223, spectral sim. = 647, #7

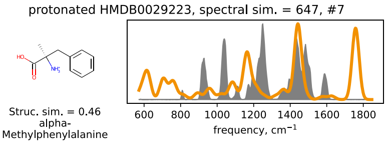

Struc. sim. = 0.46
alpha-Methylphenylalanine

protonated HMDB0039655, spectral sim. = 601, #8

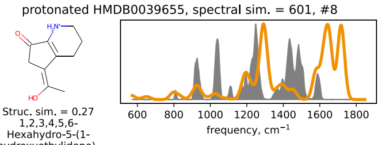

Struc. sim. = 0.27
1,2,3,4,5,6-Hexahydro-5-(1-hydroxyethylidene)-

protonated HMDB0029224, spectral sim. = 560, #9

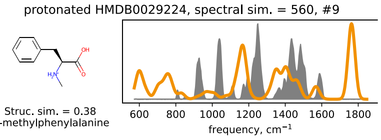

Struc. sim. = 0.38
N-methylphenylalanine

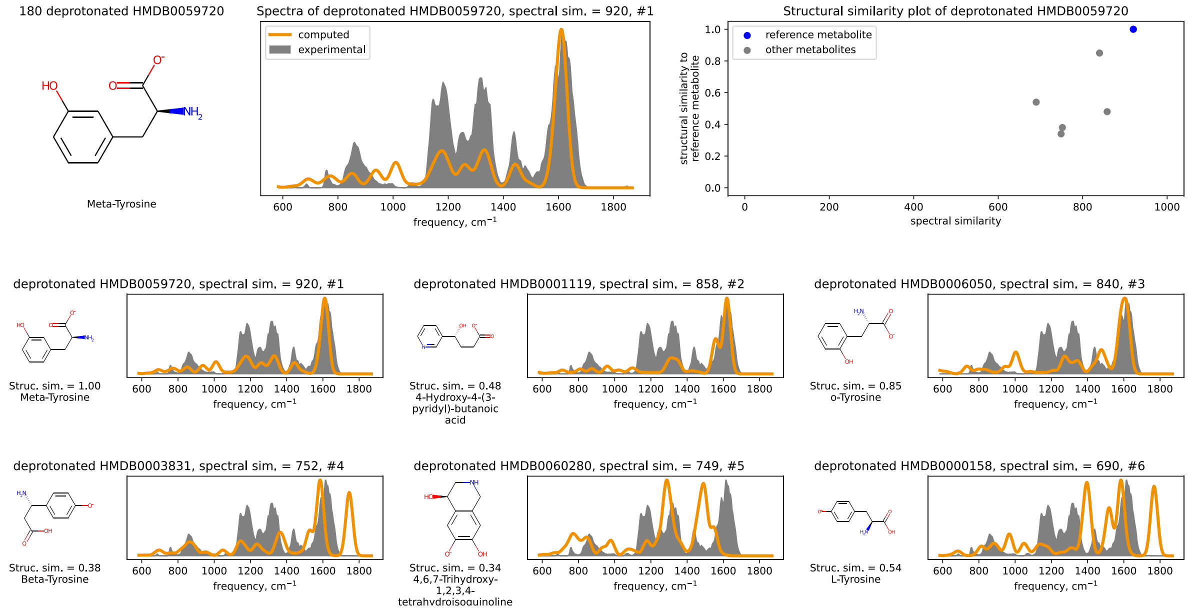

180 deprotonated HMDB0059720
Meta-Tyrosine
Spectra of deprotonated HMDB0059720, spectral sim. = 920, #1
computed
experimental
frequency, cm$^{-1}$
Structural similarity plot of deprotonated HMDB0059720
reference metabolite
other metabolites
structural similarity to reference metabolite
spectral similarity
deprotonated HMDB0059720, spectral sim. = 920, #1
Struc. sim. = 1.00
Meta-Tyrosine
deprotonated HMDB0001119, spectral sim. = 858, #2
Struc. sim. = 0.48
4-Hydroxy-4-(3-pyridyl)-butanoic acid
deprotonated HMDB0006050, spectral sim. = 840, #3
Struc. sim. = 0.85
o-Tyrosine
deprotonated HMDB0003831, spectral sim. = 752, #4
Struc. sim. = 0.38
Beta-Tyrosine
deprotonated HMDB0060280, spectral sim. = 749, #5
Struc. sim. = 0.34
4,6,7-Trihydroxy-1,2,3,4-tetrahydroisoquinoline
deprotonated HMDB0000158, spectral sim. = 690, #6
Struc. sim. = 0.54
L-Tyrosine


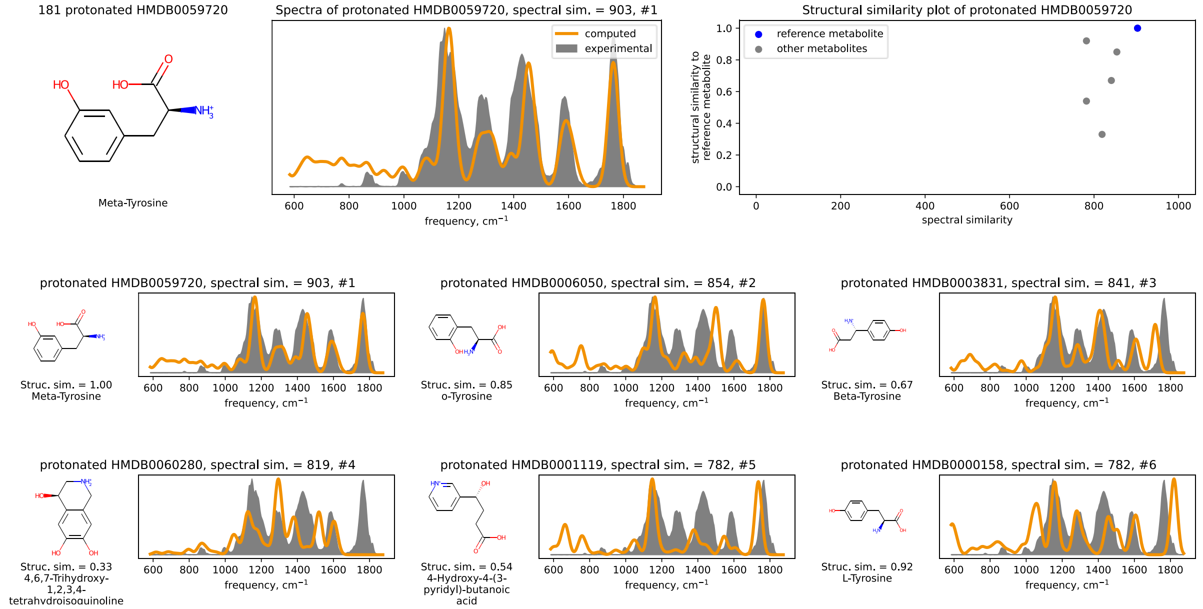

181 protonated HMDB0059720
Meta-Tyrosine
Spectra of protonated HMDB0059720, spectral sim. = 903, #1
computed
experimental
frequency, cm$^{-1}$
Structural similarity plot of protonated HMDB0059720
reference metabolite
other metabolites
structural similarity to reference metabolite
spectral similarity
protonated HMDB0059720, spectral sim. = 903, #1
Struc. sim. = 1.00
Meta-Tyrosine
protonated HMDB0006050, spectral sim. = 854, #2
Struc. sim. = 0.85
o-Tyrosine
protonated HMDB0003831, spectral sim. = 841, #3
Struc. sim. = 0.67
Beta-Tyrosine
protonated HMDB0060280, spectral sim. = 819, #4
Struc. sim. = 0.33
4,6,7-Trihydroxy-1,2,3,4-tetrahydroisoquinoline
protonated HMDB0001119, spectral sim. = 782, #5
Struc. sim. = 0.54
4-Hydroxy-4-(3-pyridyl)-butanoic acid
protonated HMDB0000158, spectral sim. = 782, #6
Struc. sim. = 0.92
L-Tyrosine


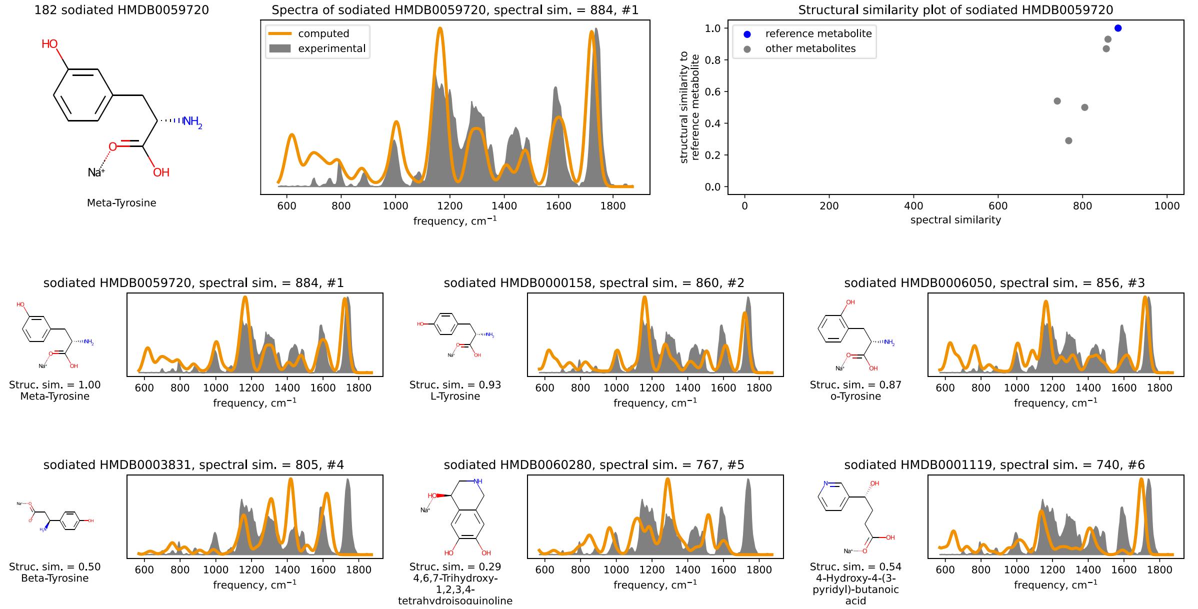

182 sodiated HMDB0059720
Meta-Tyrosine
Spectra of sodiated HMDB0059720, spectral sim. = 884, #1
computed
experimental
frequency, cm$^{-1}$
Structural similarity plot of sodiated HMDB0059720
reference metabolite
other metabolites
structural similarity to reference metabolite
spectral similarity
sodiated HMDB0059720, spectral sim. = 884, #1
Struc. sim. = 1.00
Meta-Tyrosine
sodiated HMDB0000158, spectral sim. = 860, #2
Struc. sim. = 0.93
L-Tyrosine
sodiated HMDB0006050, spectral sim. = 856, #3
Struc. sim. = 0.87
o-Tyrosine
sodiated HMDB0003831, spectral sim. = 805, #4
Struc. sim. = 0.50
Beta-Tyrosine
sodiated HMDB0060280, spectral sim. = 767, #5
Struc. sim. = 0.29
4,6,7-Trihydroxy-1,2,3,4-tetrahydroisoquinoline
sodiated HMDB0001119, spectral sim. = 740, #6
Struc. sim. = 0.54
4-Hydroxy-4-(3-pyridyl)-butanoic acid

183 deprotonated HMDB0060608

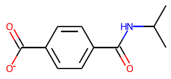

N-isopropylterephthalamic acid

Spectra of deprotonated HMDB0060608, spectral sim. = 824, #3

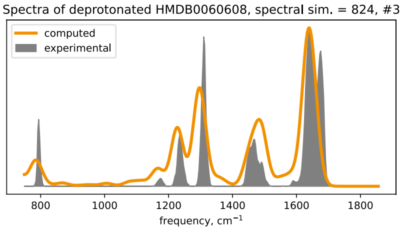


Structural similarity plot of deprotonated HMDB0060608

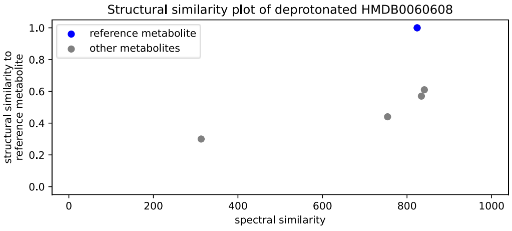


deprotonated HMDB0000512, spectral sim. = 841, #1

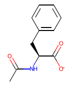

Struc. sim. = 0.61
N-Acetyl-L-phenylalanine

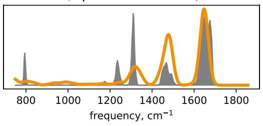


deprotonated HMDB0000860, spectral sim. = 834, #2

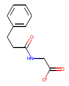

Struc. sim. = 0.57
Phenylpropionylglycine

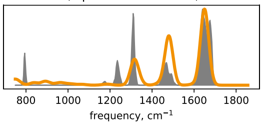


deprotonated HMDB0060608, spectral sim. = 824, #3

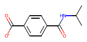

Struc. sim. = 1.00
N-isopropylterephthalamic acid

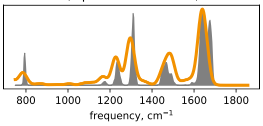


deprotonated HMDB0002042, spectral sim. = 754, #4

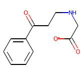

Struc. sim. = 0.44
3-Phenylpropionylglycine

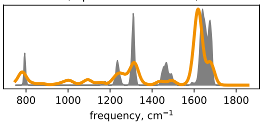


deprotonated HMDB0061682, spectral sim. = 313, #5

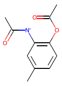

Struc. sim. = 0.30
2-Acetamido-4-methylphenyl acetate

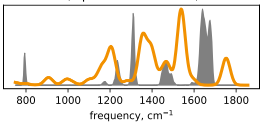


184 protonated HMDB0060608

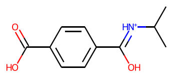

N-isopropylterephthalamic acid

Spectra of protonated HMDB0060608, spectral sim. = 897, #1

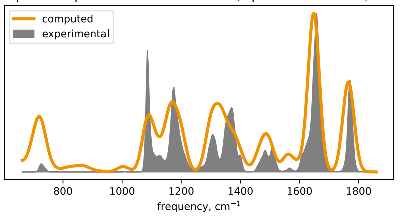


Structural similarity plot of protonated HMDB0060608

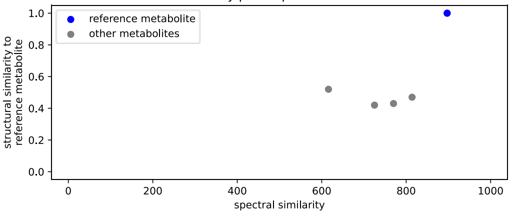


protonated HMDB0060608, spectral sim. = 897, #1

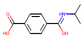

Struc. sim. = 1.00
N-isopropylterephthalamic acid

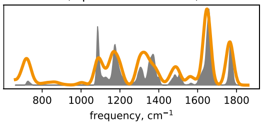


protonated HMDB0000860, spectral sim. = 814, #2

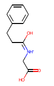

Struc. sim. = 0.47
Phenylpropionylglycine

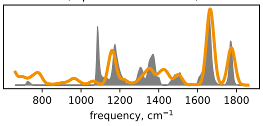


protonated HMDB0061682, spectral sim. = 770, #3

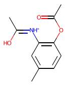

Struc. sim. = 0.43
2-Acetamido-4-methylphenyl acetate

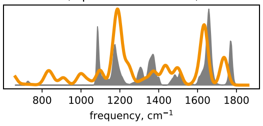


protonated HMDB0002042, spectral sim. = 725, #4

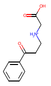

Struc. sim. = 0.42
3-Phenylpropionylglycine

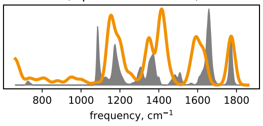


protonated HMDB0000512, spectral sim. = 616, #5

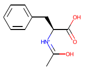

Struc. sim. = 0.52
N-Acetyl-L-phenylalanine

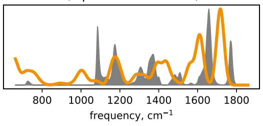


185 sodiated HMDB0060608

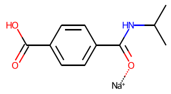

N-isopropylterephthalamic acid

Spectra of sodiated HMDB0060608, spectral sim. = 931, #1

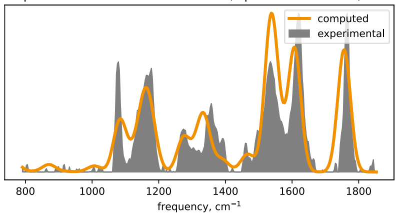


Structural similarity plot of sodiated HMDB0060608

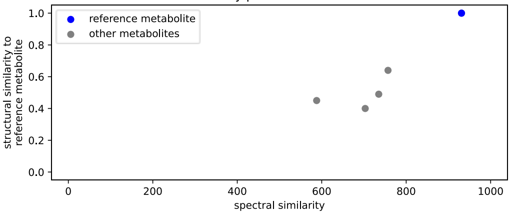


sodiated HMDB0060608, spectral sim. = 931, #1

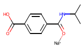

Struc. sim. = 1.00
N-isopropylterephthalamic acid

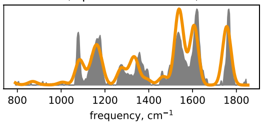


sodiated HMDB0000512, spectral sim. = 757, #2

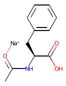

Struc. sim. = 0.64
N-Acetyl-L-phenylalanine

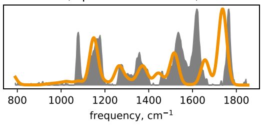


sodiated HMDB0000860, spectral sim. = 735, #3

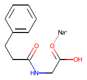

Struc. sim. = 0.49
Phenylpropionylglycine

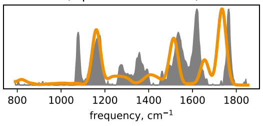


sodiated HMDB0002042, spectral sim. = 703, #4

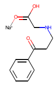

Struc. sim. = 0.40
3-Phenylpropionylglycine

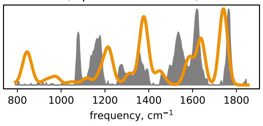


sodiated HMDB0061682, spectral sim. = 588, #5

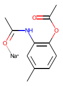

Struc. sim. = 0.45
2-Acetamido-4-methylphenyl acetate

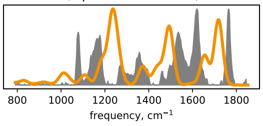

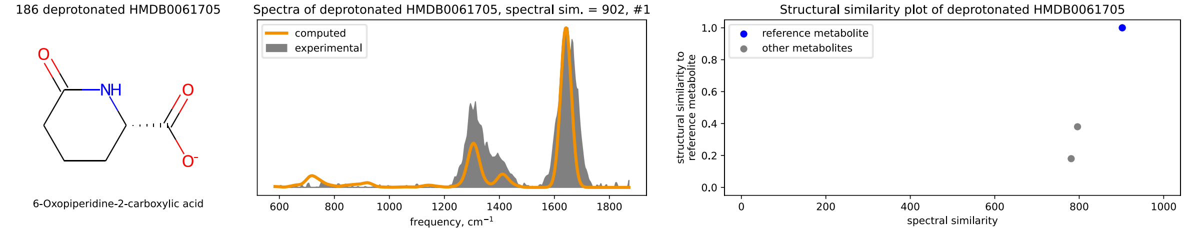
186 deprotonated HMDB0061705
6-Oxopiperidine-2-carboxylic acid
Spectra of deprotonated HMDB0061705, spectral sim. = 902, #1
computed
experimental
frequency, cm⁻¹
Structural similarity plot of deprotonated HMDB0061705
reference metabolite
other metabolites
structural similarity to reference metabolite
spectral similarity

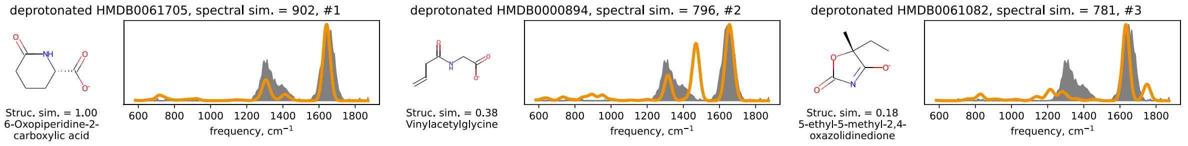
deprotonated HMDB0061705, spectral sim. = 902, #1
Struc. sim. = 1.00
6-Oxopiperidine-2-carboxylic acid
deprotonated HMDB0000894, spectral sim. = 796, #2
Struc. sim. = 0.38
Vinylacetylglycine
deprotonated HMDB0061082, spectral sim. = 781, #3
Struc. sim. = 0.18
5-ethyl-5-methyl-2,4-oxazolidinedione
frequency, cm⁻¹

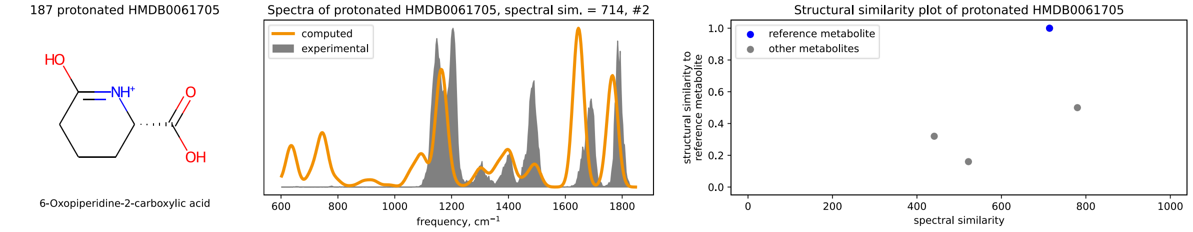
187 protonated HMDB0061705
6-Oxopiperidine-2-carboxylic acid
Spectra of protonated HMDB0061705, spectral sim. = 714, #2
computed
experimental
frequency, cm⁻¹
Structural similarity plot of protonated HMDB0061705
reference metabolite
other metabolites
structural similarity to reference metabolite
spectral similarity

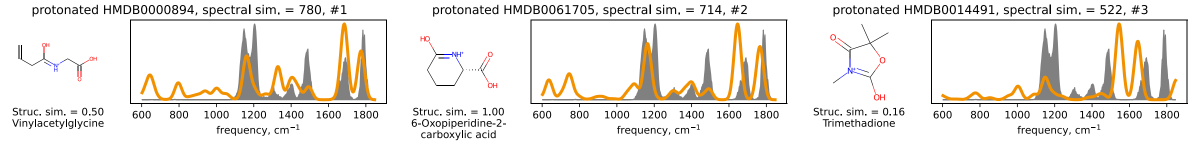
protonated HMDB0000894, spectral sim. = 780, #1
Struc. sim. = 0.50
Vinylacetylglycine
protonated HMDB0061705, spectral sim. = 714, #2
Struc. sim. = 1.00
6-Oxopiperidine-2-carboxylic acid
protonated HMDB0014491, spectral sim. = 522, #3
Struc. sim. = 0.16
Trimethadione
frequency, cm⁻¹

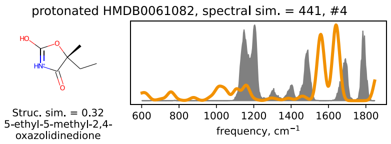
protonated HMDB0061082, spectral sim. = 441, #4
Struc. sim. = 0.32
5-ethyl-5-methyl-2,4-oxazolidinedione
frequency, cm⁻¹

188 sodiated HMDB0061705

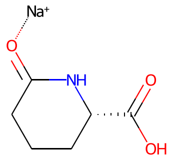


6-Oxopiperidine-2-carboxylic acid

Spectra of sodiated HMDB0061705, spectral sim. = 823, #1

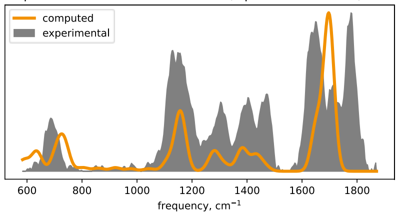


Structural similarity plot of sodiated HMDB0061705

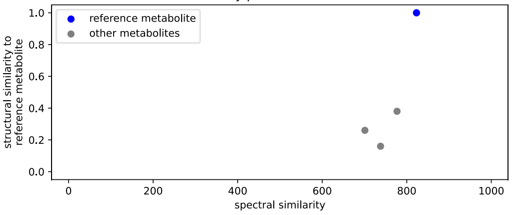


sodiated HMDB0061705, spectral sim. = 823, #1

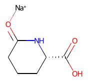

Struc. sim. = 1.00
6-Oxopiperidine-2-carboxylic acid

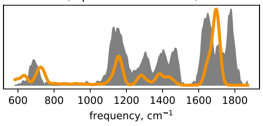


sodiated HMDB0000894, spectral sim. = 777, #2

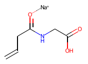

Struc. sim. = 0.38
Vinylacetylglycine

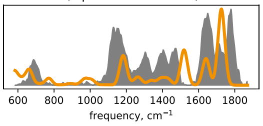


sodiated HMDB0014491, spectral sim. = 738, #3

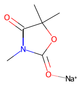

Struc. sim. = 0.16
Trimethadione

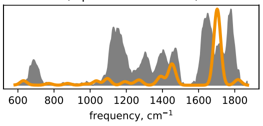


sodiated HMDB0061082, spectral sim. = 701, #4

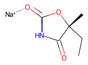

Struc. sim. = 0.26
5-ethyl-5-methyl-2,4-oxazolidinedione

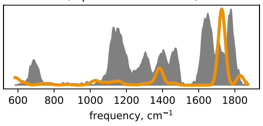


189 protonated HMDB0094701

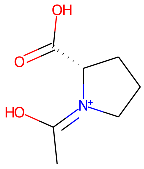


N-Acetylproline

Spectra of protonated HMDB0094701, spectral sim. = 896, #1

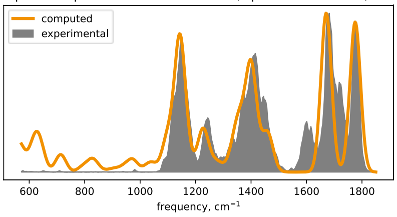


Structural similarity plot of protonated HMDB0094701

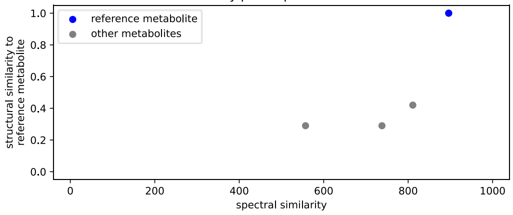


protonated HMDB0094701, spectral sim. = 896, #1

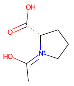

Struc. sim. = 1.00
N-Acetylproline

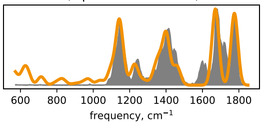


protonated HMDB0000459, spectral sim. = 811, #2

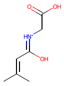

Struc. sim. = 0.42
3-Methylcrotonylglycine

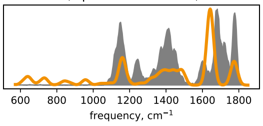


protonated HMDB0000959, spectral sim. = 738, #3

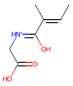

Struc. sim. = 0.29
Tiglylglycine

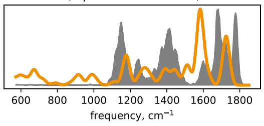


protonated HMDB0014755, spectral sim. = 557, #4

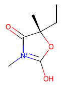

Struc. sim. = 0.29
Paramethadione

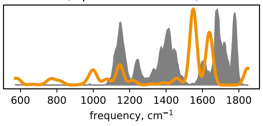

Supplement: Supplementary file 3 — ac3c01078_si_003.pdf [file ac3c01078_si_003.pdf]
