# Supplementary material for: An In Silico Infrared Spectral Library of Molecular Ions for Metabolite Identification
Source: Anal Chem. 2023 Jun 1;95(23):8998–9005. doi: 10.1021/acs.analchem.3c01078 (PMC10267894; doi:10.1021/acs.analchem.3c01078)
Supplement: Supplementary file 4 — ac3c01078_si_004.pdf [file ac3c01078_si_004.pdf]

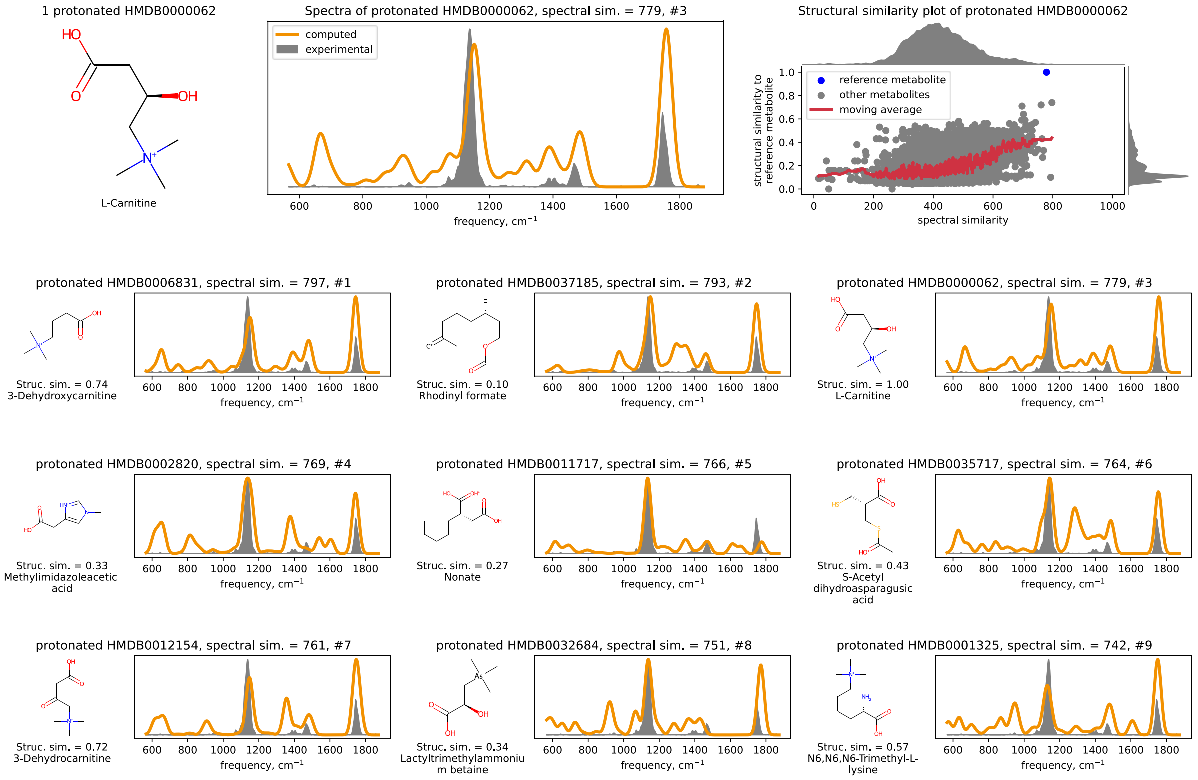
1 protonated HMDB0000062
L-Carnitine
Spectra of protonated HMDB0000062, spectral sim. = 779, #3
computed
experimental
frequency, cm^{-1}
Structural similarity plot of protonated HMDB0000062
reference metabolite
other metabolites
moving average
structural similarity to reference metabolite
spectral similarity
protonated HMDB0006831, spectral sim. = 797, #1
Struc. sim. = 0.74
3-Dehydroxycarnitine
protonated HMDB0037185, spectral sim. = 793, #2
Struc. sim. = 0.10
Rhodinyl formate
protonated HMDB0000062, spectral sim. = 779, #3
Struc. sim. = 1.00
L-Carnitine
protonated HMDB0002820, spectral sim. = 769, #4
Struc. sim. = 0.33
Methylimidazoleacetic acid
protonated HMDB0011717, spectral sim. = 766, #5
Struc. sim. = 0.27
Nonate
protonated HMDB0035717, spectral sim. = 764, #6
Struc. sim. = 0.43
S-Acetyl dihydroasparagusic acid
protonated HMDB0012154, spectral sim. = 761, #7
Struc. sim. = 0.72
3-Dehydrocarnitine
protonated HMDB0032684, spectral sim. = 751, #8
Struc. sim. = 0.34
Lactyltrimethylammonium betaine
protonated HMDB0001325, spectral sim. = 742, #9
Struc. sim. = 0.57
N6,N6,N6-Trimethyl-L-lysine

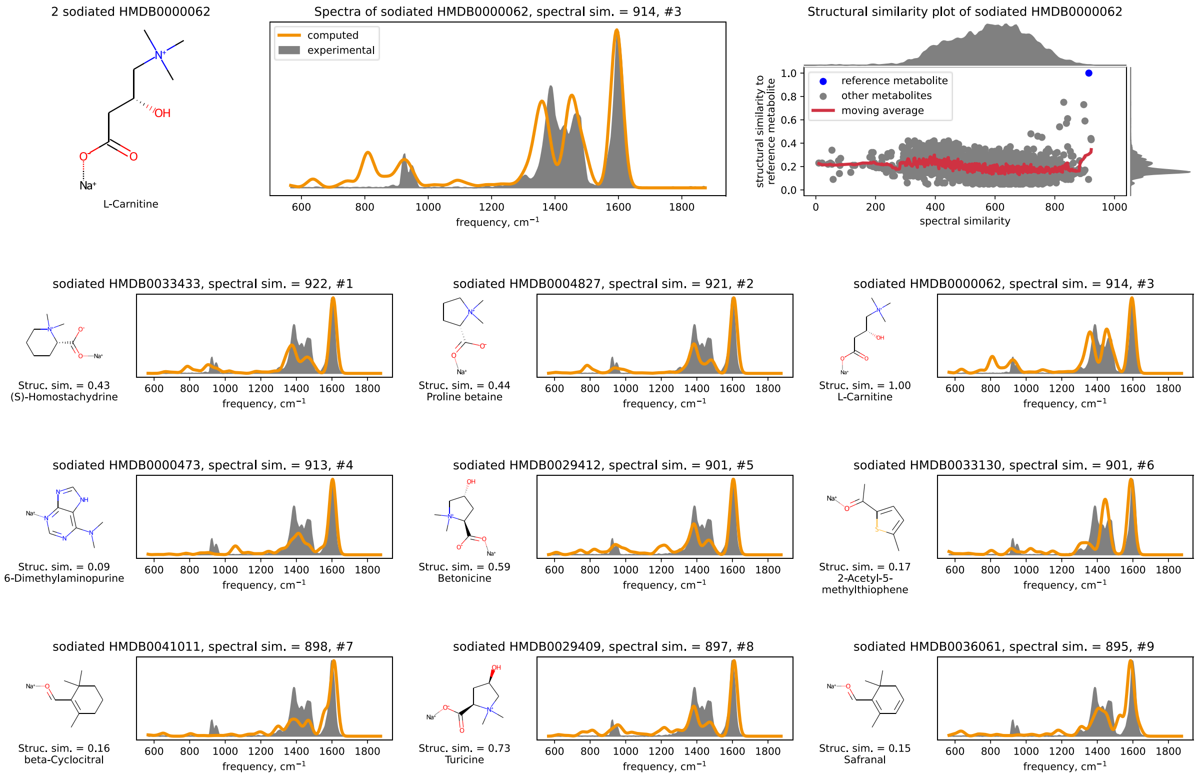
2 sodiated HMDB0000062
L-Carnitine
Spectra of sodiated HMDB0000062, spectral sim. = 914, #3
computed
experimental
frequency, cm^{-1}
Structural similarity plot of sodiated HMDB0000062
reference metabolite
other metabolites
moving average
structural similarity to reference metabolite
spectral similarity
sodiated HMDB0033433, spectral sim. = 922, #1
Struc. sim. = 0.43
(S)-Homostachydrine
sodiated HMDB0004827, spectral sim. = 921, #2
Struc. sim. = 0.44
Proline betaine
sodiated HMDB0000062, spectral sim. = 914, #3
Struc. sim. = 1.00
L-Carnitine
sodiated HMDB0000473, spectral sim. = 913, #4
Struc. sim. = 0.09
6-Dimethylaminopurine
sodiated HMDB0029412, spectral sim. = 901, #5
Struc. sim. = 0.59
Betonicine
sodiated HMDB0033130, spectral sim. = 901, #6
Struc. sim. = 0.17
2-Acetyl-5-methylthiophene
sodiated HMDB0041011, spectral sim. = 898, #7
Struc. sim. = 0.16
beta-Cyclocitral
sodiated HMDB0029409, spectral sim. = 897, #8
Struc. sim. = 0.73
Turicine
sodiated HMDB0036061, spectral sim. = 895, #9
Struc. sim. = 0.15
Safranal

3 deprotonated HMDB0000070

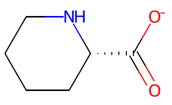

Pipecolic acid

Spectra of deprotonated HMDB0000070, spectral sim. = 887, #85

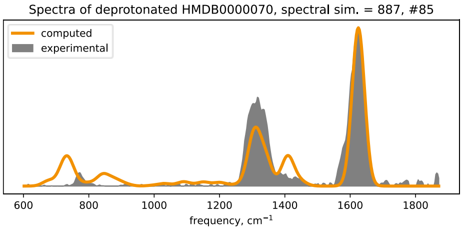

Structural similarity plot of deprotonated HMDB0000070

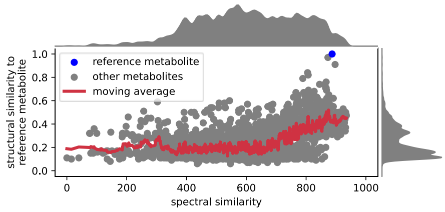

deprotonated HMDB0030331, spectral sim. = 934, #1

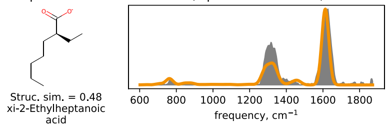

deprotonated HMDB0000039, spectral sim. = 934, #2

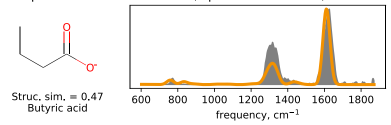

deprotonated HMDB0002176, spectral sim. = 934, #3

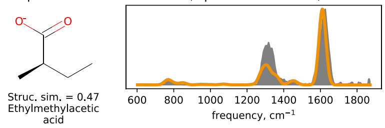

deprotonated HMDB0031221, spectral sim. = 933, #4

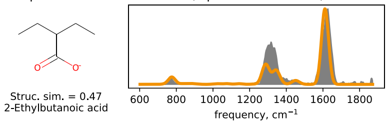

deprotonated HMDB0031594, spectral sim. = 932, #5

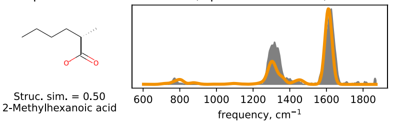

deprotonated HMDB0031587, spectral sim. = 932, #6

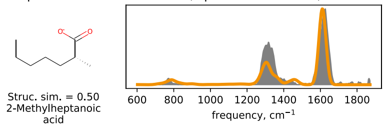

deprotonated HMDB0037175, spectral sim. = 932, #7

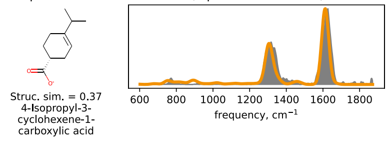

deprotonated HMDB0032708, spectral sim. = 931, #8

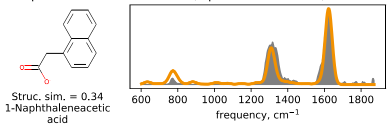

deprotonated HMDB0001873, spectral sim. = 930, #9

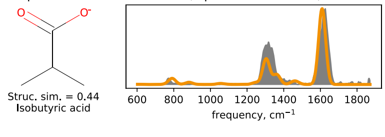

4 protonated HMDB0000070

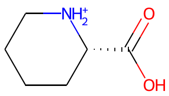

Pipecolic acid

Spectra of protonated HMDB0000070, spectral sim. = 890, #4

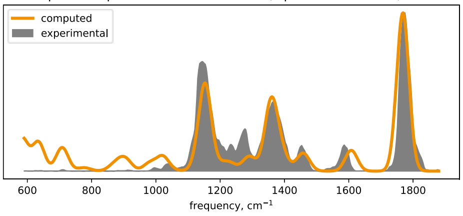

Structural similarity plot of protonated HMDB0000070

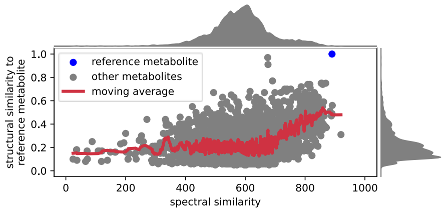

protonated HMDB0031179, spectral sim. = 920, #1

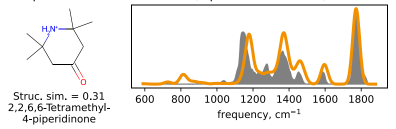

protonated HMDB0030393, spectral sim. = 891, #2

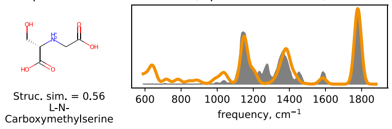

protonated HMDB0029873, spectral sim. = 890, #3

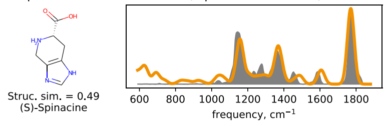

protonated HMDB0000070, spectral sim. = 890, #4

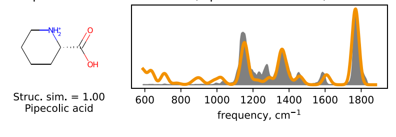

protonated HMDB0012151, spectral sim. = 888, #5

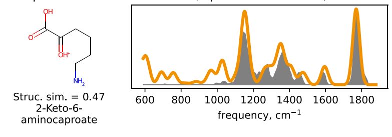

protonated HMDB0060385, spectral sim. = 883, #6

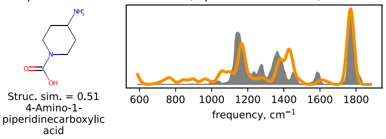

protonated HMDB0061156, spectral sim. = 871, #7

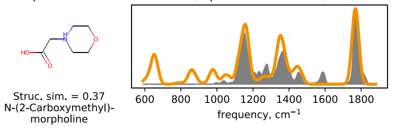

protonated HMDB0000725, spectral sim. = 869, #8

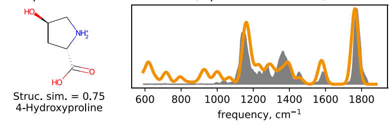

protonated HMDB0006955, spectral sim. = 867, #9

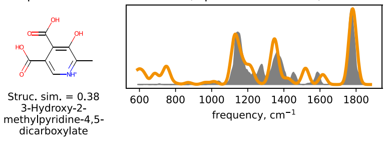

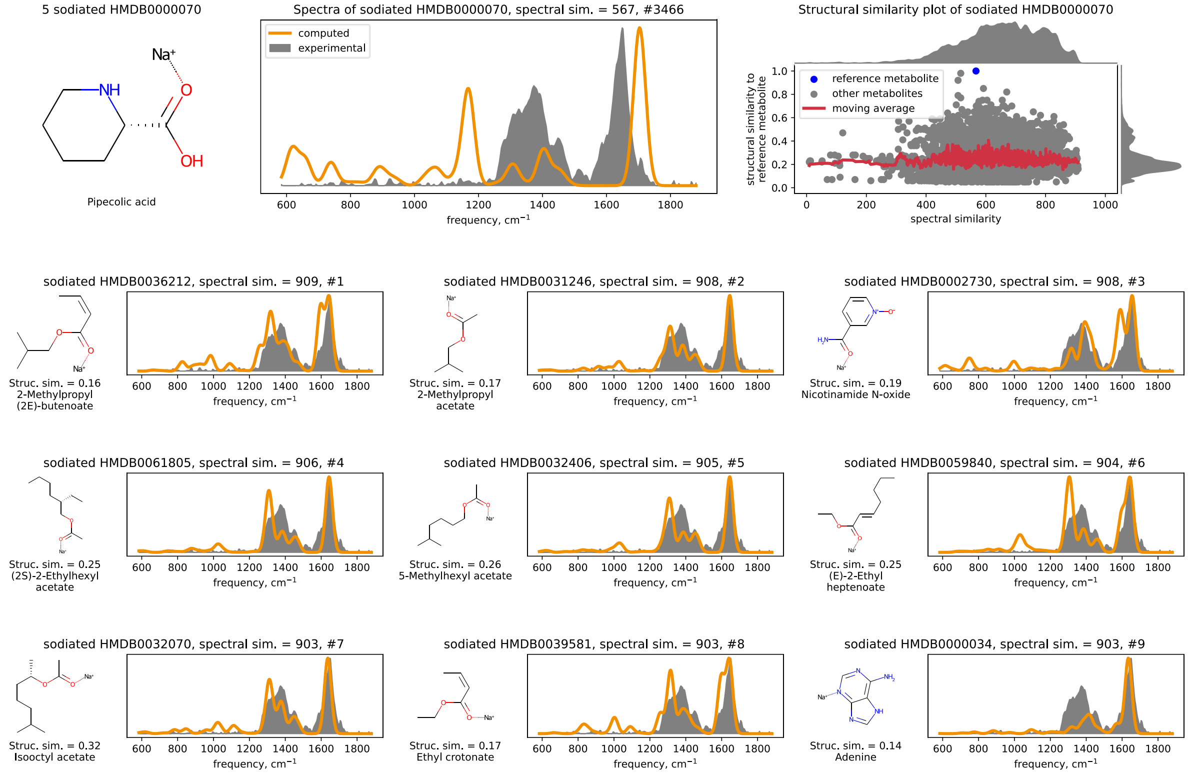

5 sodiated HMDB0000070
Pipecolic acid
Spectra of sodiated HMDB0000070, spectral sim. = 567, #3466
computed
experimental
frequency, cm⁻¹
Structural similarity plot of sodiated HMDB0000070
reference metabolite
other metabolites
moving average
structural similarity to reference metabolite
spectral similarity
sodiated HMDB0036212, spectral sim. = 909, #1
Struc. sim. = 0.16
2-Methylpropyl (2E)-butenoate
sodiated HMDB0031246, spectral sim. = 908, #2
Struc. sim. = 0.17
2-Methylpropyl acetate
sodiated HMDB0002730, spectral sim. = 908, #3
Struc. sim. = 0.19
Nicotinamide N-oxide
sodiated HMDB0061805, spectral sim. = 906, #4
Struc. sim. = 0.25
(2S)-2-Ethylhexyl acetate
sodiated HMDB0032406, spectral sim. = 905, #5
Struc. sim. = 0.26
5-Methylhexyl acetate
sodiated HMDB0059840, spectral sim. = 904, #6
Struc. sim. = 0.25
(E)-2-Ethyl heptenoate
sodiated HMDB0032070, spectral sim. = 903, #7
Struc. sim. = 0.32
Isooctyl acetate
sodiated HMDB0039581, spectral sim. = 903, #8
Struc. sim. = 0.17
Ethyl crotonate
sodiated HMDB0000034, spectral sim. = 903, #9
Struc. sim. = 0.14
Adenine

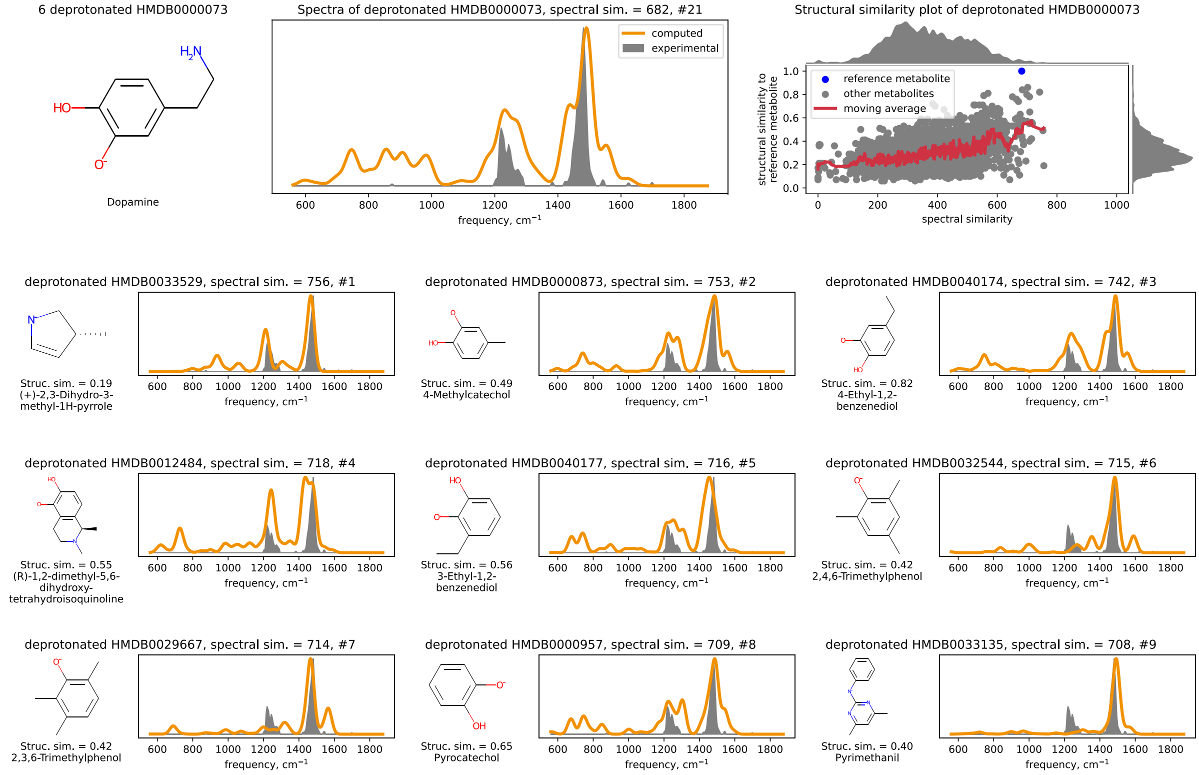

6 deprotonated HMDB0000073
Dopamine
Spectra of deprotonated HMDB0000073, spectral sim. = 682, #21
computed
experimental
frequency, cm⁻¹
Structural similarity plot of deprotonated HMDB0000073
reference metabolite
other metabolites
moving average
structural similarity to reference metabolite
spectral similarity
deprotonated HMDB0033529, spectral sim. = 756, #1
Struc. sim. = 0.19
(+)-2,3-Dihydro-3-methyl-1H-pyrrole
deprotonated HMDB0000873, spectral sim. = 753, #2
Struc. sim. = 0.49
4-Methylcatechol
deprotonated HMDB0040174, spectral sim. = 742, #3
Struc. sim. = 0.82
4-Ethyl-1,2-benzenediol
deprotonated HMDB0012484, spectral sim. = 718, #4
Struc. sim. = 0.55
(R)-1,2-dimethyl-5,6-dihydroxy-tetrahydroisoquinoline
deprotonated HMDB0040177, spectral sim. = 716, #5
Struc. sim. = 0.56
3-Ethyl-1,2-benzenediol
deprotonated HMDB0032544, spectral sim. = 715, #6
Struc. sim. = 0.42
2,4,6-Trimethylphenol
deprotonated HMDB0029667, spectral sim. = 714, #7
Struc. sim. = 0.42
2,3,6-Trimethylphenol
deprotonated HMDB0000957, spectral sim. = 709, #8
Struc. sim. = 0.65
Pyrocatechol
deprotonated HMDB0033135, spectral sim. = 708, #9
Struc. sim. = 0.40
Pyrimethanil

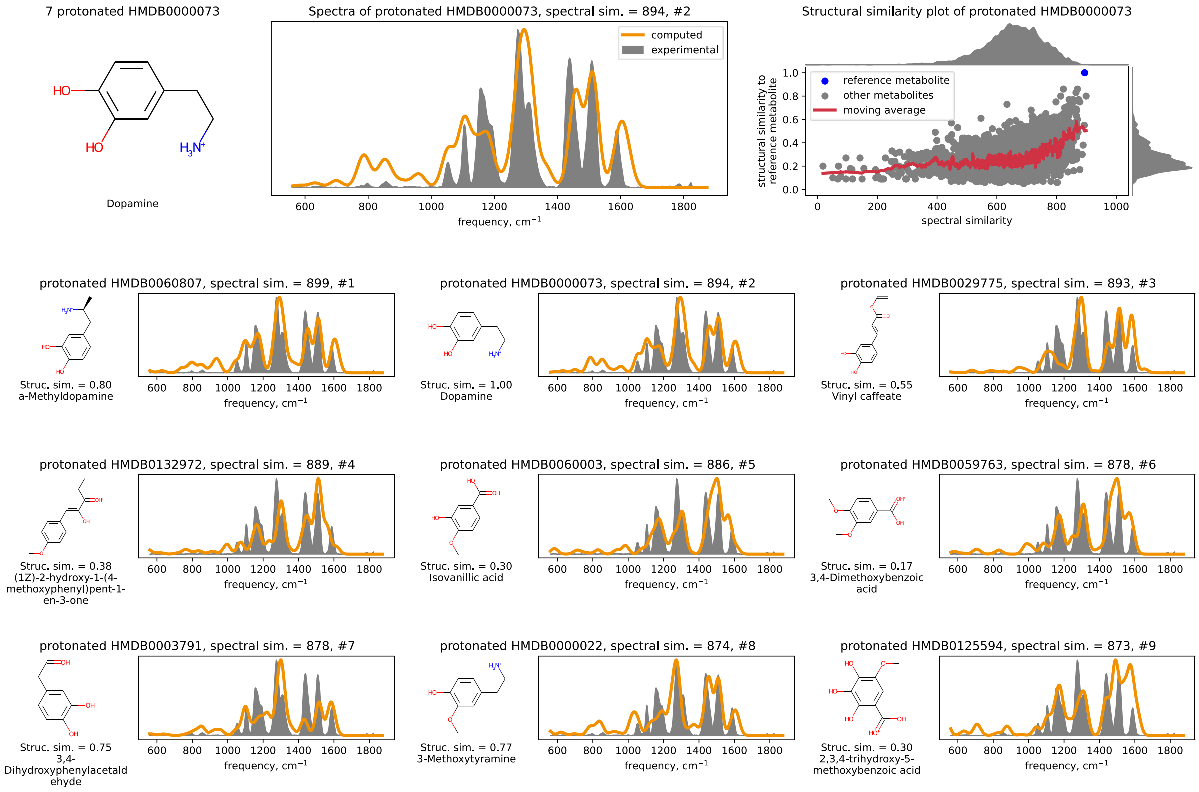
7 protonated HMDB0000073
Dopamine
Spectra of protonated HMDB0000073, spectral sim. = 894, #2
computed
experimental
frequency, cm⁻¹
Structural similarity plot of protonated HMDB0000073
reference metabolite
other metabolites
moving average
structural similarity to reference metabolite
spectral similarity
protonated HMDB0060807, spectral sim. = 899, #1
Struc. sim. = 0.80
a-Methyldopamine
protonated HMDB0000073, spectral sim. = 894, #2
Struc. sim. = 1.00
Dopamine
protonated HMDB0029775, spectral sim. = 893, #3
Struc. sim. = 0.55
Vinyl caffeate
protonated HMDB0132972, spectral sim. = 889, #4
Struc. sim. = 0.38
(1Z)-2-hydroxy-1-(4-methoxyphenyl)pent-1-en-3-one
protonated HMDB0060003, spectral sim. = 886, #5
Struc. sim. = 0.30
Isovanillic acid
protonated HMDB0059763, spectral sim. = 878, #6
Struc. sim. = 0.17
3,4-Dimethoxybenzoic acid
protonated HMDB0003791, spectral sim. = 878, #7
Struc. sim. = 0.75
3,4-Dihydroxyphenylacetaldehyde
protonated HMDB0000022, spectral sim. = 874, #8
Struc. sim. = 0.77
3-Methoxytyramine
protonated HMDB0125594, spectral sim. = 873, #9
Struc. sim. = 0.30
2,3,4-trihydroxy-5-methoxybenzoic acid
frequency, cm⁻¹

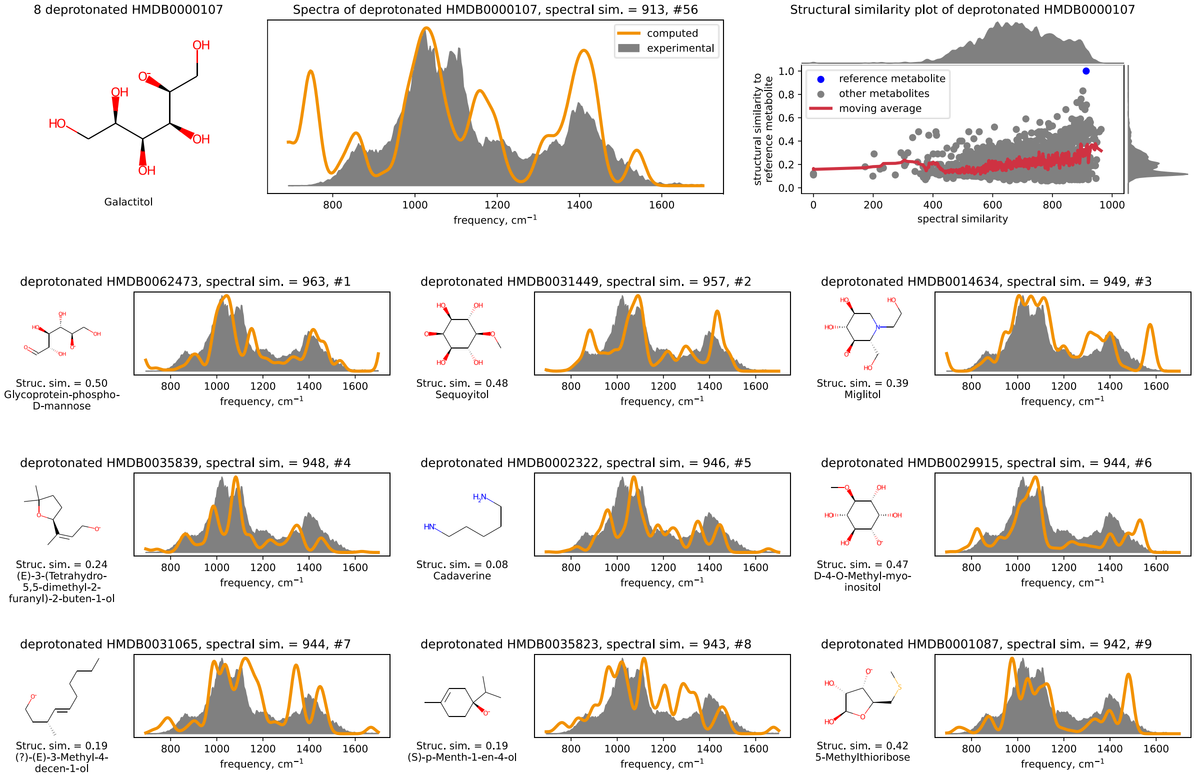
8 deprotonated HMDB0000107
Galactitol
Spectra of deprotonated HMDB0000107, spectral sim. = 913, #56
computed
experimental
frequency, cm⁻¹
Structural similarity plot of deprotonated HMDB0000107
reference metabolite
other metabolites
moving average
structural similarity to reference metabolite
spectral similarity
deprotonated HMDB0062473, spectral sim. = 963, #1
Struc. sim. = 0.50
Glycoprotein-phospho-D-mannose
deprotonated HMDB0031449, spectral sim. = 957, #2
Struc. sim. = 0.48
Sequoyitol
deprotonated HMDB0014634, spectral sim. = 949, #3
Struc. sim. = 0.39
Miglitol
deprotonated HMDB0035839, spectral sim. = 948, #4
Struc. sim. = 0.24
(E)-3-(Tetrahydro-5,5-dimethyl-2-furanyl)-2-buten-1-ol
deprotonated HMDB0002322, spectral sim. = 946, #5
Struc. sim. = 0.08
Cadaverine
deprotonated HMDB0029915, spectral sim. = 944, #6
Struc. sim. = 0.47
D-4-O-Methyl-myo-inositol
deprotonated HMDB0031065, spectral sim. = 944, #7
Struc. sim. = 0.19
(?)-(E)-3-Methyl-4-decen-1-ol
deprotonated HMDB0035823, spectral sim. = 943, #8
Struc. sim. = 0.19
(S)-p-Menth-1-en-4-ol
deprotonated HMDB0001087, spectral sim. = 942, #9
Struc. sim. = 0.42
5-Methylthioribose
frequency, cm⁻¹

9 deprotonated HMDB0000130

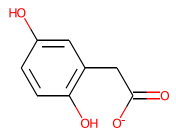

Homogentisic acid

Spectra of deprotonated HMDB0000130, spectral sim. = 895, #38

Structural similarity plot of deprotonated HMDB0000130

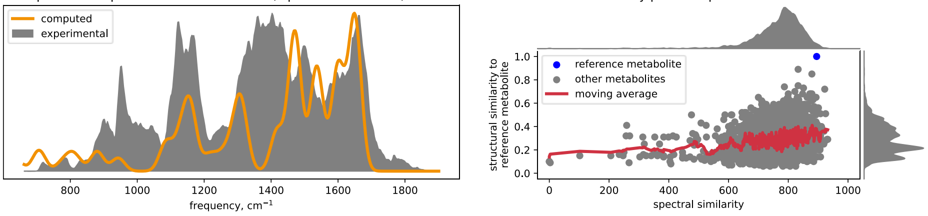

deprotonated HMDB0030580, spectral sim. = 932, #1

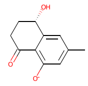

Struc. sim. = 0.29
(R)-Shinanolone

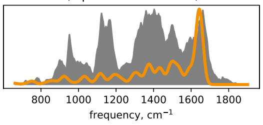

deprotonated HMDB0038712, spectral sim. = 930, #2

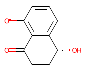

Struc. sim. = 0.29
(S)-Isosclerone

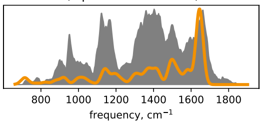

deprotonated HMDB0133782, spectral sim. = 925, #3

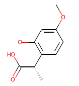

Struc. sim. = 0.28
2-(2-hydroxy-4-methoxyphenyl) propanoic acid

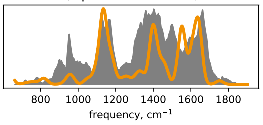

deprotonated HMDB0137136, spectral sim. = 925, #4

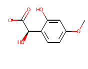

Struc. sim. = 0.51
2-hydroxy-2-(2-hydroxy-4-methoxyphenyl)acetic acid

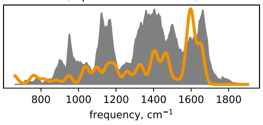

deprotonated HMDB0126480, spectral sim. = 922, #5

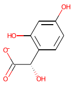

Struc. sim. = 0.72
2-(2,4-dihydroxyphenyl)-2-hydroxyacetic acid

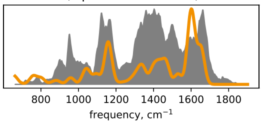

deprotonated HMDB0133526, spectral sim. = 920, #6

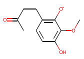

Struc. sim. = 0.36
4-(2,4-dihydroxy-3-methoxyphenyl)butan-2-one

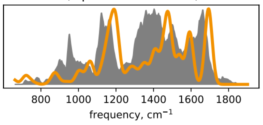

deprotonated HMDB0002085, spectral sim. = 919, #7

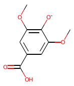

Struc. sim. = 0.25
Syringic acid

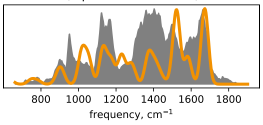

deprotonated HMDB0133538, spectral sim. = 914, #8

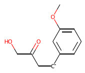

Struc. sim. = 0.28
1-hydroxy-4-(3-methoxyphenyl)butan-2-one

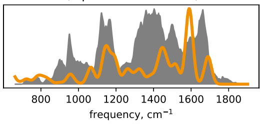

deprotonated HMDB0125598, spectral sim. = 912, #9

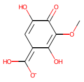

Struc. sim. = 0.39
2,4,5-trihydroxy-3-methoxybenzoic acid

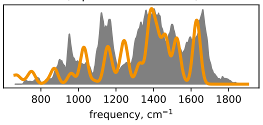

10 sodiated HMDB0000130

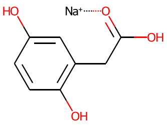

Homogentisic acid

Spectra of sodiated HMDB0000130, spectral sim. = 932, #9

Structural similarity plot of sodiated HMDB0000130

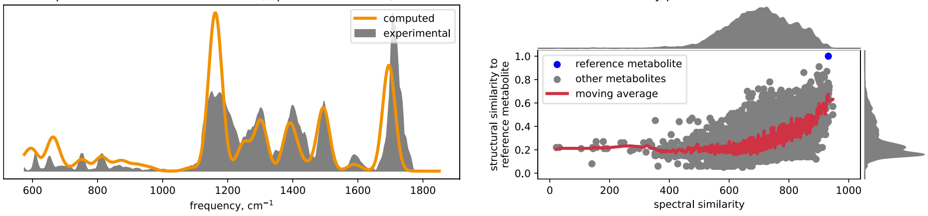

sodiated HMDB0141258, spectral sim. = 947, #1

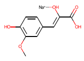

Struc. sim. = 0.50
(2Z)-2-hydroxy-3-(4-hydroxy-3-methoxyphenyl)prop-2-enoic acid

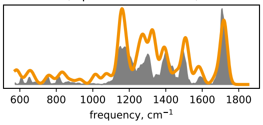

sodiated HMDB0134033, spectral sim. = 947, #2

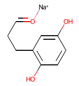

Struc. sim. = 0.57
3-(2,5-dihydroxyphenyl) propanal

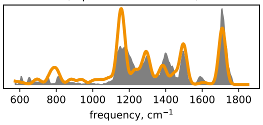

sodiated HMDB0000423, spectral sim. = 935, #3

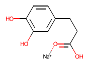

Struc. sim. = 0.74
3,4-Dihydroxyhydrocinnamic acid

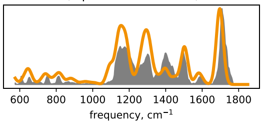

sodiated HMDB0000333, spectral sim. = 934, #4

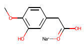

Struc. sim. = 0.71
Isohomovanillic acid

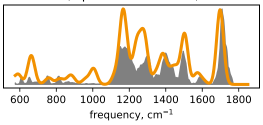

sodiated HMDB0062121, spectral sim. = 933, #5

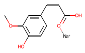

Struc. sim. = 0.68
Hydroferulic acid

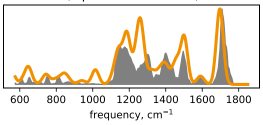

sodiated HMDB0131138, spectral sim. = 932, #6

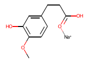

Struc. sim. = 0.64
3-(3-hydroxy-4-methoxyphenyl) propanoic acid

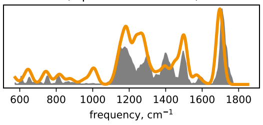

sodiated HMDB0124923, spectral sim. = 932, #7

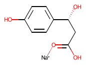

Struc. sim. = 0.77
3-hydroxy-3-(4-hydroxyphenyl) propanoic acid

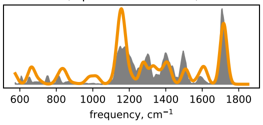

sodiated HMDB0141118, spectral sim. = 932, #8

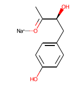

Struc. sim. = 0.44
3-hydroxy-4-(4-hydroxyphenyl)butan-2-one

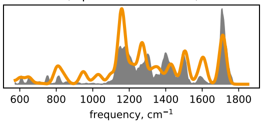

sodiated HMDB0000130, spectral sim. = 932, #9

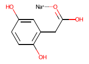

Struc. sim. = 1.00
Homogentisic acid

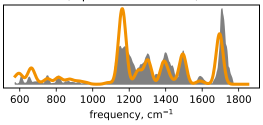

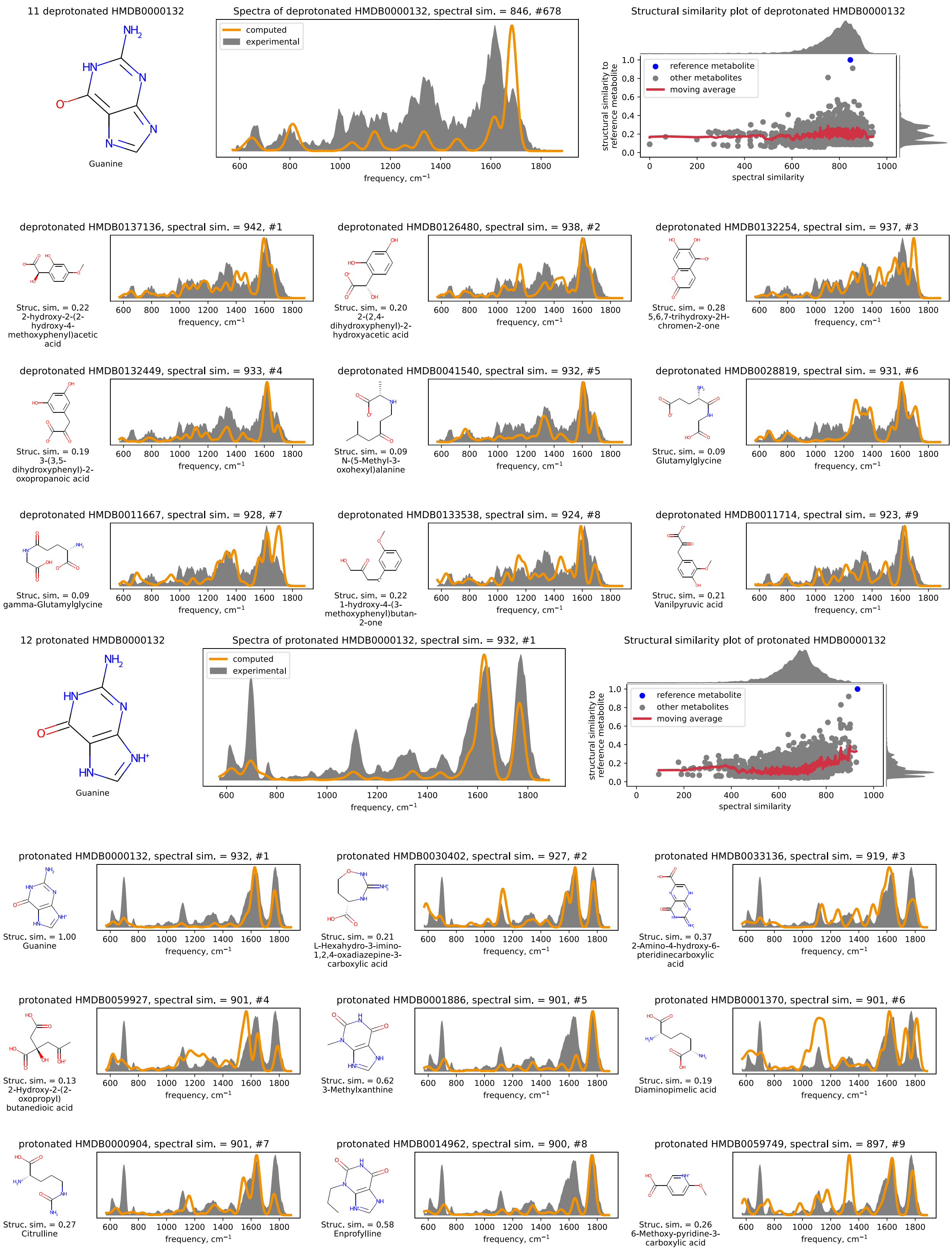

11 deprotonated HMDB0000132
Guanine
Spectra of deprotonated HMDB0000132, spectral sim. = 846, #678
computed
experimental
frequency, cm⁻¹
Structural similarity plot of deprotonated HMDB0000132
reference metabolite
other metabolites
moving average
structural similarity to reference metabolite
spectral similarity
deprotonated HMDB0137136, spectral sim. = 942, #1
Struc. sim. = 0.22
2-hydroxy-2-(2-hydroxy-4-methoxyphenyl)acetic acid
deprotonated HMDB0126480, spectral sim. = 938, #2
Struc. sim. = 0.20
2-(2,4-dihydroxyphenyl)-2-hydroxyacetic acid
deprotonated HMDB0132254, spectral sim. = 937, #3
Struc. sim. = 0.28
5,6,7-trihydroxy-2H-chromen-2-one
deprotonated HMDB0132449, spectral sim. = 933, #4
Struc. sim. = 0.19
3-(3,5-dihydroxyphenyl)-2-oxopropanoic acid
deprotonated HMDB0041540, spectral sim. = 932, #5
Struc. sim. = 0.09
N-(5-Methyl-3-oxohexyl)alanine
deprotonated HMDB0028819, spectral sim. = 931, #6
Struc. sim. = 0.09
Glutamylglycine
deprotonated HMDB0011667, spectral sim. = 928, #7
Struc. sim. = 0.09
gamma-Glutamylglycine
deprotonated HMDB0133538, spectral sim. = 924, #8
Struc. sim. = 0.22
1-hydroxy-4-(3-methoxyphenyl)butan-2-one
deprotonated HMDB0011714, spectral sim. = 923, #9
Struc. sim. = 0.21
Vanilpyruvic acid
12 protonated HMDB0000132
Guanine
Spectra of protonated HMDB0000132, spectral sim. = 932, #1
Structural similarity plot of protonated HMDB0000132
protonated HMDB0000132, spectral sim. = 932, #1
Struc. sim. = 1.00
Guanine
protonated HMDB0030402, spectral sim. = 927, #2
Struc. sim. = 0.21
L-Hexahydro-3-imino-1,2,4-oxadiazepine-3-carboxylic acid
protonated HMDB0033136, spectral sim. = 919, #3
Struc. sim. = 0.37
2-Amino-4-hydroxy-6-pteridinecarboxylic acid
protonated HMDB0059927, spectral sim. = 901, #4
Struc. sim. = 0.13
2-Hydroxy-2-(2-oxopropyl) butanedioic acid
protonated HMDB0001886, spectral sim. = 901, #5
Struc. sim. = 0.62
3-Methylxanthine
protonated HMDB0001370, spectral sim. = 901, #6
Struc. sim. = 0.19
Diaminopimelic acid
protonated HMDB0000904, spectral sim. = 901, #7
Struc. sim. = 0.27
Citrulline
protonated HMDB0014962, spectral sim. = 900, #8
Struc. sim. = 0.58
Enprofylline
protonated HMDB0059749, spectral sim. = 897, #9
Struc. sim. = 0.26
6-Methoxy-pyridine-3-carboxylic acid

13 sodiated HMDB0000132

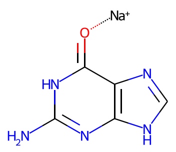

Guanine

Spectra of sodiated HMDB0000132, spectral sim. = 910, #21

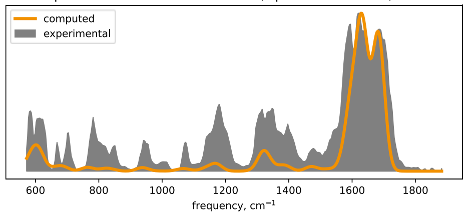

Structural similarity plot of sodiated HMDB0000132

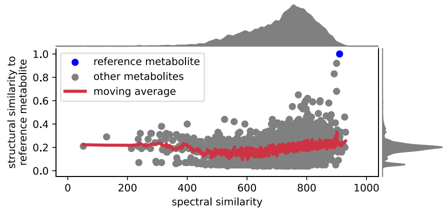

sodiated HMDB0028797, spectral sim. = 930, #1

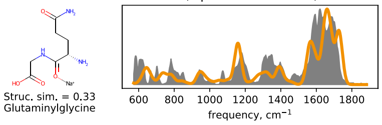

Struc. sim. = 0.33
Glutaminylglycine

sodiated HMDB0035721, spectral sim. = 928, #2

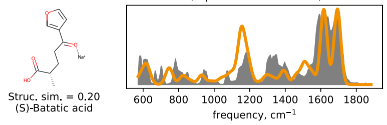

Struc. sim. = 0.20
(S)-Batatic acid

sodiated HMDB0033136, spectral sim. = 927, #3

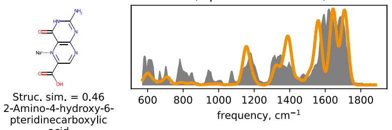

Struc. sim. = 0.46
2-Amino-4-hydroxy-6-pteridinecarboxylic acid

sodiated HMDB0029939, spectral sim. = 924, #4

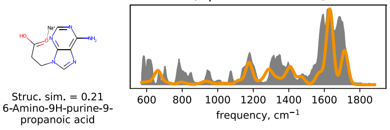

Struc. sim. = 0.21
6-Amino-9H-purine-9-propanoic acid

sodiated HMDB0032603, spectral sim. = 922, #5

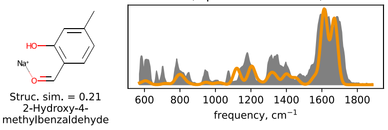

Struc. sim. = 0.21
2-Hydroxy-4-methylbenzaldehyde

sodiated HMDB0039147, spectral sim. = 922, #6

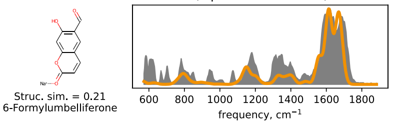

Struc. sim. = 0.21
6-Formylumbelliferone

sodiated HMDB0014389, spectral sim. = 918, #7

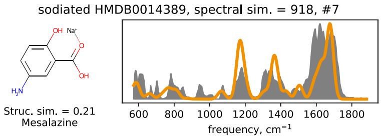

Struc. sim. = 0.21
Mesalazine

sodiated HMDB0137120, spectral sim. = 918, #8

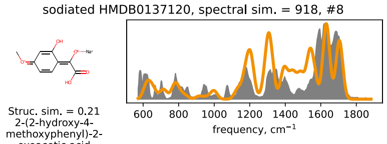

Struc. sim. = 0.21
2-(2-hydroxy-4-methoxyphenyl)-2-oxoacetic acid

sodiated HMDB0035243, spectral sim. = 918, #9

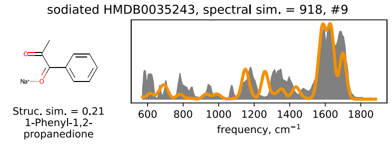

Struc. sim. = 0.21
1-Phenyl-1,2-propanedione

14 deprotonated HMDB0000143

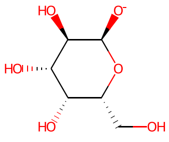

D-Galactose

Spectra of deprotonated HMDB0000143, spectral sim. = 813, #604

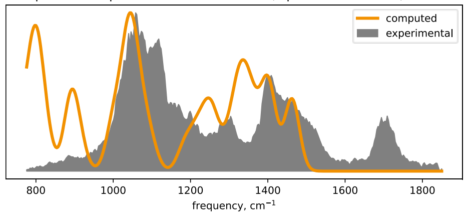

Structural similarity plot of deprotonated HMDB0000143

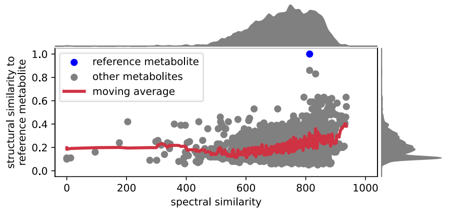

deprotonated HMDB0000621, spectral sim. = 936, #1

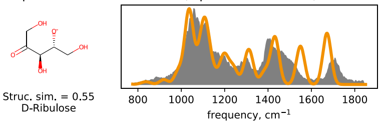

Struc. sim. = 0.55
D-Ribulose

deprotonated HMDB0062473, spectral sim. = 935, #2

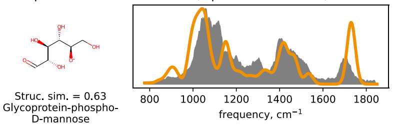

Struc. sim. = 0.63
Glycoprotein-phospho-D-mannose

deprotonated HMDB0062538, spectral sim. = 934, #3

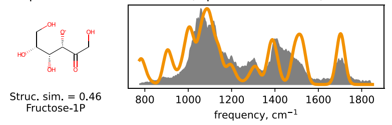

Struc. sim. = 0.46
Fructose-1P

deprotonated HMDB0000660, spectral sim. = 931, #4

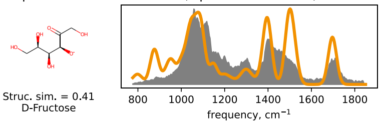

Struc. sim. = 0.41
D-Fructose

deprotonated HMDB0005876, spectral sim. = 931, #5

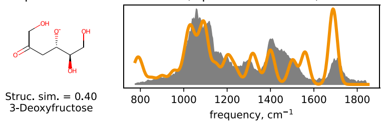

Struc. sim. = 0.40
3-Deoxyfructose

deprotonated HMDB0140947, spectral sim. = 929, #6

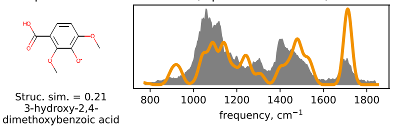

Struc. sim. = 0.21
3-hydroxy-2,4-dimethoxybenzoic acid

deprotonated HMDB0060953, spectral sim. = 924, #7

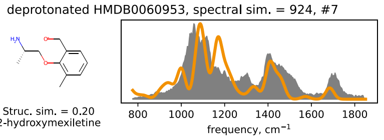

Struc. sim. = 0.20
2-hydroxymexiletine

deprotonated HMDB0040892, spectral sim. = 922, #8

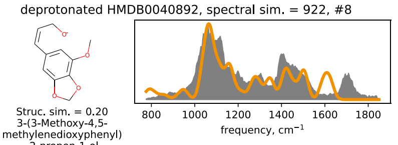

Struc. sim. = 0.20
3-(3-Methoxy-4,5-methylenedioxyphenyl)-2-propen-1-ol

deprotonated HMDB0060267, spectral sim. = 920, #9

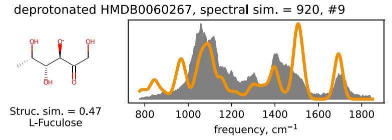

Struc. sim. = 0.47
L-Fuculose

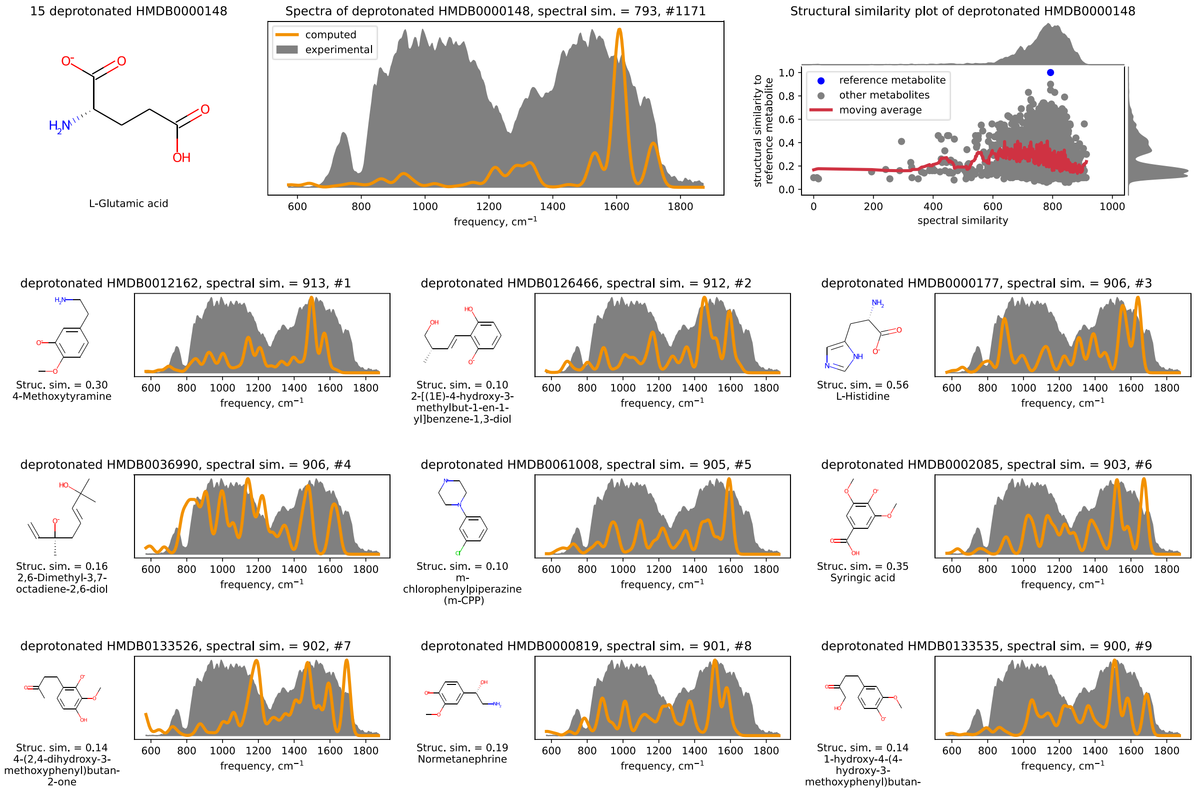

15 deprotonated HMDB0000148
L-Glutamic acid
Spectra of deprotonated HMDB0000148, spectral sim. = 793, #1171
computed
experimental
frequency, cm^{-1}
Structural similarity plot of deprotonated HMDB0000148
reference metabolite
other metabolites
moving average
structural similarity to reference metabolite
spectral similarity
deprotonated HMDB0012162, spectral sim. = 913, #1
Struc. sim. = 0.30
4-Methoxytyramine
deprotonated HMDB0126466, spectral sim. = 912, #2
Struc. sim. = 0.10
2-[(1E)-4-hydroxy-3-methylbut-1-en-1-yl]benzene-1,3-diol
deprotonated HMDB0000177, spectral sim. = 906, #3
Struc. sim. = 0.56
L-Histidine
deprotonated HMDB0036990, spectral sim. = 906, #4
Struc. sim. = 0.16
2,6-Dimethyl-3,7-octadiene-2,6-diol
deprotonated HMDB0061008, spectral sim. = 905, #5
Struc. sim. = 0.10
m-chlorophenylpiperazine (m-CPP)
deprotonated HMDB0002085, spectral sim. = 903, #6
Struc. sim. = 0.35
Syringic acid
deprotonated HMDB0133526, spectral sim. = 902, #7
Struc. sim. = 0.14
4-(2,4-dihydroxy-3-methoxyphenyl)butan-2-one
deprotonated HMDB0000819, spectral sim. = 901, #8
Struc. sim. = 0.19
Normetanephrine
deprotonated HMDB0133535, spectral sim. = 900, #9
Struc. sim. = 0.14
1-hydroxy-4-(4-hydroxy-3-methoxyphenyl)butan-

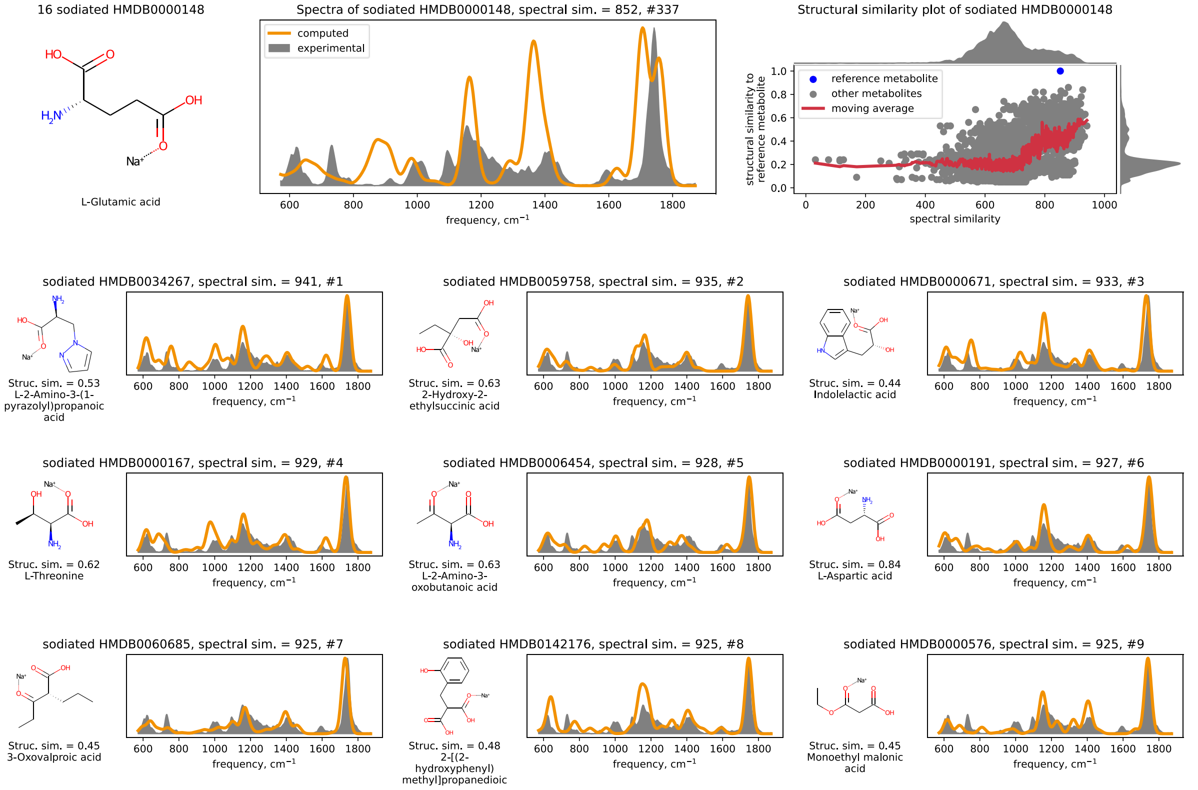

16 sodiated HMDB0000148
L-Glutamic acid
Spectra of sodiated HMDB0000148, spectral sim. = 852, #337
computed
experimental
frequency, cm^{-1}
Structural similarity plot of sodiated HMDB0000148
reference metabolite
other metabolites
moving average
structural similarity to reference metabolite
spectral similarity
sodiated HMDB0034267, spectral sim. = 941, #1
Struc. sim. = 0.53
L-2-Amino-3-(1-pyrazolyl)propanoic acid
sodiated HMDB0059758, spectral sim. = 935, #2
Struc. sim. = 0.63
2-Hydroxy-2-ethylsuccinic acid
sodiated HMDB0000671, spectral sim. = 933, #3
Struc. sim. = 0.44
Indolelactic acid
sodiated HMDB0000167, spectral sim. = 929, #4
Struc. sim. = 0.62
L-Threonine
sodiated HMDB0006454, spectral sim. = 928, #5
Struc. sim. = 0.63
L-2-Amino-3-oxobutanoic acid
sodiated HMDB0000191, spectral sim. = 927, #6
Struc. sim. = 0.84
L-Aspartic acid
sodiated HMDB0060685, spectral sim. = 925, #7
Struc. sim. = 0.45
3-Oxovalproic acid
sodiated HMDB0142176, spectral sim. = 925, #8
Struc. sim. = 0.48
2-[(2-hydroxyphenyl)methyl]propanedioic
sodiated HMDB0000576, spectral sim. = 925, #9
Struc. sim. = 0.45
Monoethyl malonic acid

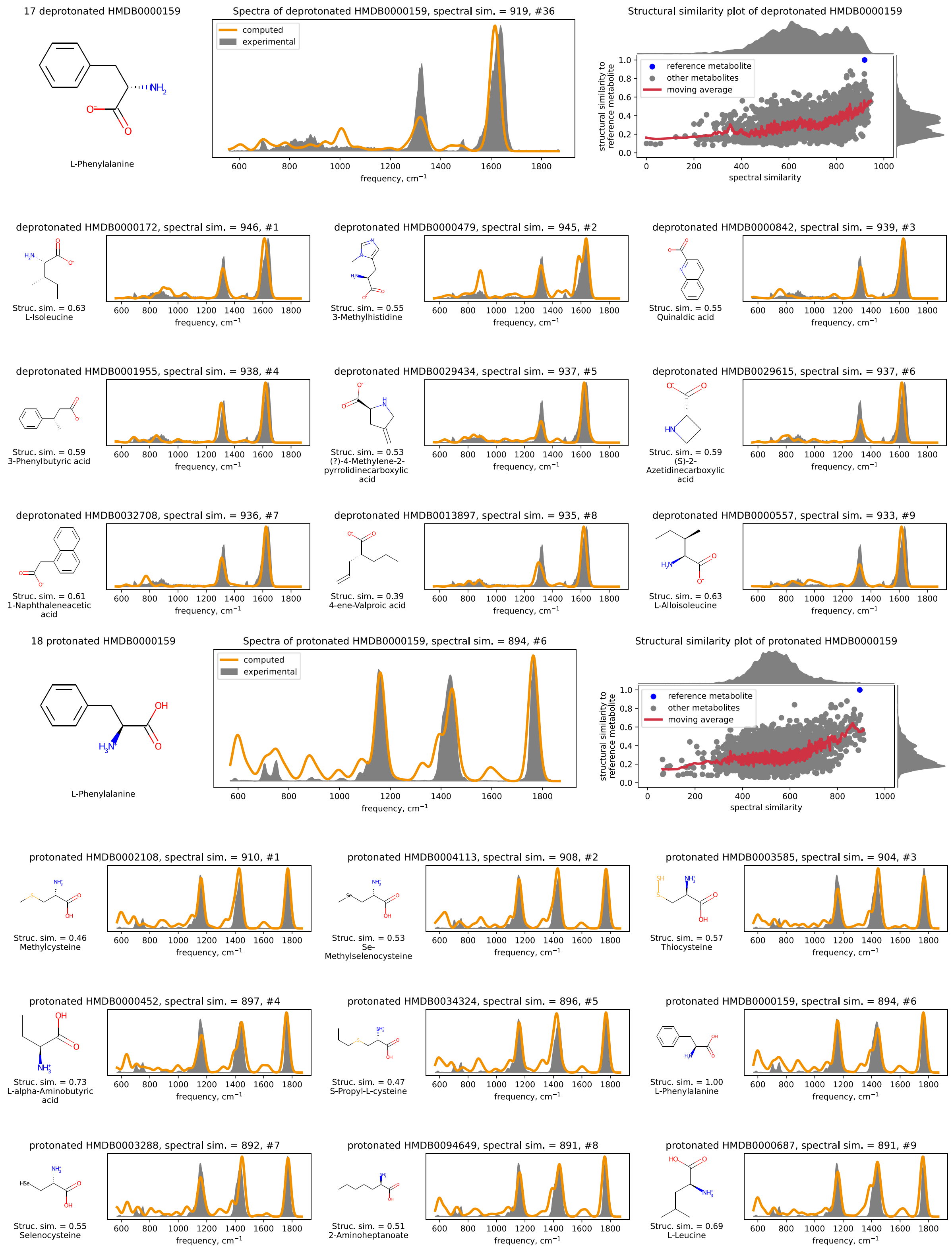

17 deprotonated HMDB0000159
L-Phenylalanine
Spectra of deprotonated HMDB0000159, spectral sim. = 919, #36
computed
experimental
frequency, cm⁻¹
Structural similarity plot of deprotonated HMDB0000159
reference metabolite
other metabolites
moving average
structural similarity to reference metabolite
spectral similarity
deprotonated HMDB0000172, spectral sim. = 946, #1
Struc. sim. = 0.63
L-Isoleucine
deprotonated HMDB0000479, spectral sim. = 945, #2
Struc. sim. = 0.55
3-Methylhistidine
deprotonated HMDB0000842, spectral sim. = 939, #3
Struc. sim. = 0.55
Quinaldic acid
deprotonated HMDB0001955, spectral sim. = 938, #4
Struc. sim. = 0.59
3-Phenylbutyric acid
deprotonated HMDB0029434, spectral sim. = 937, #5
Struc. sim. = 0.53
(?)-4-Methylene-2-pyrrolidinecarboxylic acid
deprotonated HMDB0029615, spectral sim. = 937, #6
Struc. sim. = 0.59
(S)-2-Azetidinecarboxylic acid
deprotonated HMDB0032708, spectral sim. = 936, #7
Struc. sim. = 0.61
1-Naphthaleneacetic acid
deprotonated HMDB0013897, spectral sim. = 935, #8
Struc. sim. = 0.39
4-ene-Valproic acid
deprotonated HMDB0000557, spectral sim. = 933, #9
Struc. sim. = 0.63
L-Alloisoleucine
18 protonated HMDB0000159
L-Phenylalanine
Spectra of protonated HMDB0000159, spectral sim. = 894, #6
computed
experimental
frequency, cm⁻¹
Structural similarity plot of protonated HMDB0000159
reference metabolite
other metabolites
moving average
structural similarity to reference metabolite
spectral similarity
protonated HMDB0002108, spectral sim. = 910, #1
Struc. sim. = 0.46
Methylcysteine
protonated HMDB0004113, spectral sim. = 908, #2
Struc. sim. = 0.53
Se-Methylselenocysteine
protonated HMDB0003585, spectral sim. = 904, #3
Struc. sim. = 0.57
Thiocysteine
protonated HMDB0000452, spectral sim. = 897, #4
Struc. sim. = 0.73
L-alpha-Aminobutyric acid
protonated HMDB0034324, spectral sim. = 896, #5
Struc. sim. = 0.47
S-Propyl-L-cysteine
protonated HMDB0000159, spectral sim. = 894, #6
Struc. sim. = 1.00
L-Phenylalanine
protonated HMDB0003288, spectral sim. = 892, #7
Struc. sim. = 0.55
Selenocysteine
protonated HMDB0094649, spectral sim. = 891, #8
Struc. sim. = 0.51
2-Aminoheptanoate
protonated HMDB0000687, spectral sim. = 891, #9
Struc. sim. = 0.69
L-Leucine

19 sodiated HMDB0000159

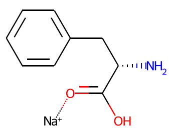

L-Phenylalanine

Spectra of sodiated HMDB0000159, spectral sim. = 825, #126

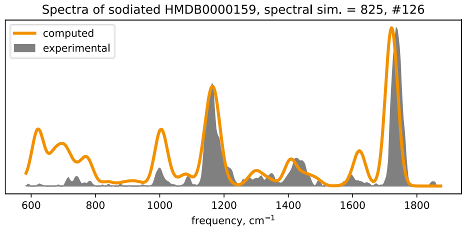

Structural similarity plot of sodiated HMDB0000159

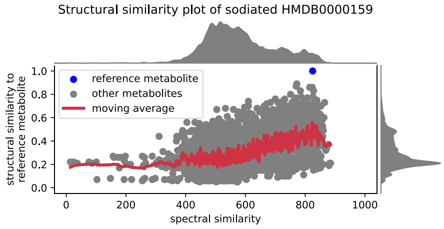

sodiated HMDB0031683, spectral sim. = 885, #1

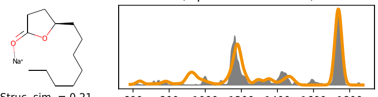

Struc. sim. = 0.21
xi-Dihydro-5-octyl-2(3H)-furanone

sodiated HMDB0035143, spectral sim. = 882, #2

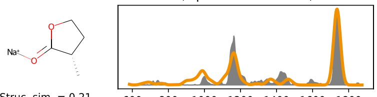

Struc. sim. = 0.21
Dihydro-3-methyl-2(3H)-furanone

sodiated HMDB0032331, spectral sim. = 880, #3

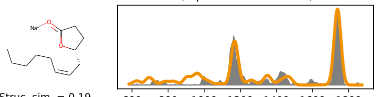

Struc. sim. = 0.19
(Z)-4-Hydroxy-6-dodecenoic acid lactone

sodiated HMDB0003771, spectral sim. = 878, #4

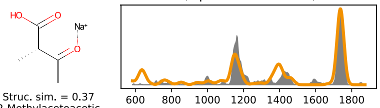

Struc. sim. = 0.37
2-Methylacetoacetic acid

sodiated HMDB0000576, spectral sim. = 871, #5

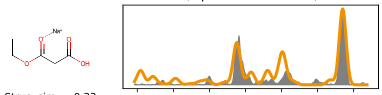

Struc. sim. = 0.33
Monoethyl malonic acid

sodiated HMDB0059809, spectral sim. = 869, #6

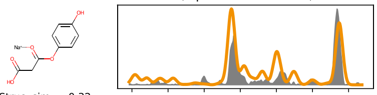

Struc. sim. = 0.32
4-Hydroxy-benzenepropanedioate

sodiated HMDB0060245, spectral sim. = 865, #7

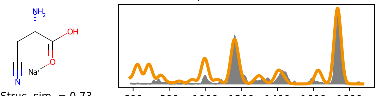

Struc. sim. = 0.73
L-3-Cyanoalanine

sodiated HMDB0000191, spectral sim. = 863, #8

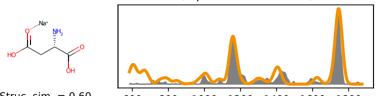

Struc. sim. = 0.60
L-Aspartic acid

sodiated HMDB0060461, spectral sim. = 863, #9

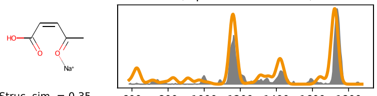

Struc. sim. = 0.35
cis-Acetylacrylate

20 protonated HMDB0000172

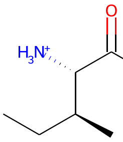

L-Isoleucine

Spectra of protonated HMDB0000172, spectral sim. = 900, #16

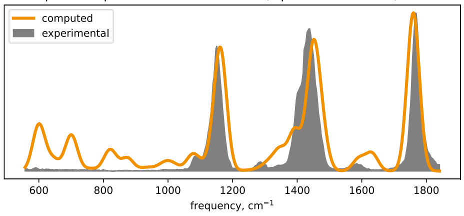

Structural similarity plot of protonated HMDB0000172

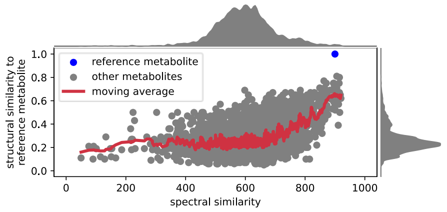

protonated HMDB0003585, spectral sim. = 920, #1

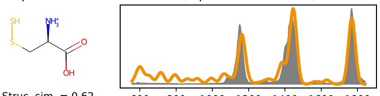

Struc. sim. = 0.62
Thiocysteine

protonated HMDB0004113, spectral sim. = 918, #2

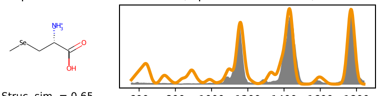

Struc. sim. = 0.65
Se-Methylselenocysteine

protonated HMDB0002108, spectral sim. = 916, #3

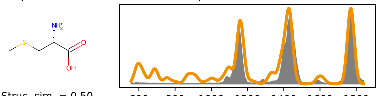

Struc. sim. = 0.50
Methylcysteine

protonated HMDB0003288, spectral sim. = 915, #4

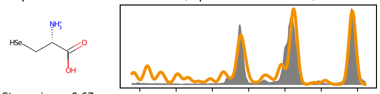

Struc. sim. = 0.67
Selenocysteine

protonated HMDB0000452, spectral sim. = 915, #5

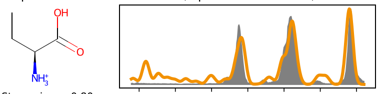

Struc. sim. = 0.80
L-alpha-Aminobutyric acid

protonated HMDB0000687, spectral sim. = 914, #6

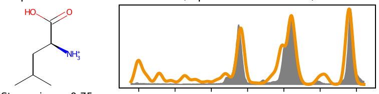

Struc. sim. = 0.75
L-Leucine

protonated HMDB0034324, spectral sim. = 912, #7

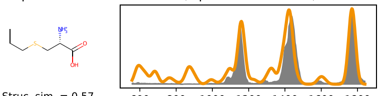

Struc. sim. = 0.57
S-Propyl-L-cysteine

protonated HMDB0094649, spectral sim. = 911, #8

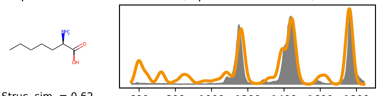

Struc. sim. = 0.62
2-Aminoheptanoate

protonated HMDB0000574, spectral sim. = 911, #9

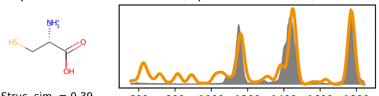

Struc. sim. = 0.39
L-Cysteine

21 deprotonated HMDB0000182

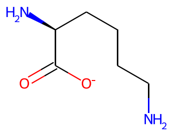

Spectra of deprotonated HMDB0000182, spectral sim. = 852, #404

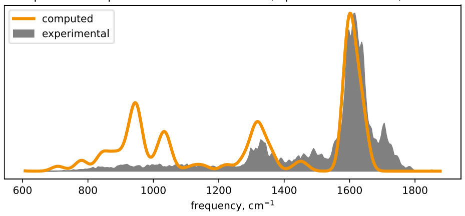

Structural similarity plot of deprotonated HMDB0000182

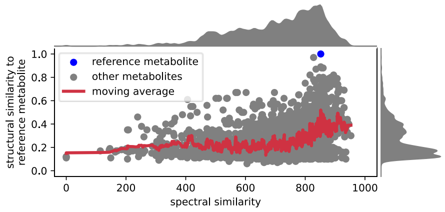

deprotonated HMDB0033091, spectral sim. = 953, #1

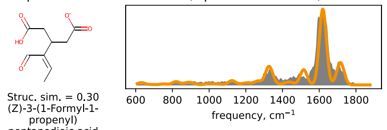

deprotonated HMDB0002393, spectral sim. = 946, #2

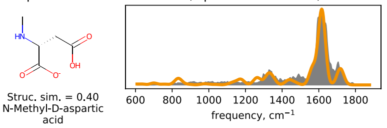

deprotonated HMDB0000448, spectral sim. = 941, #3

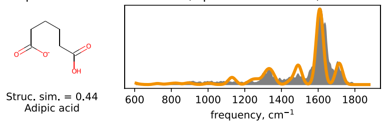

deprotonated HMDB0060348, spectral sim. = 941, #4

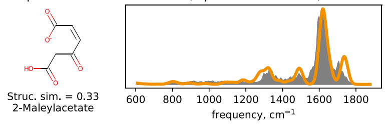

deprotonated HMDB0012150, spectral sim. = 941, #5

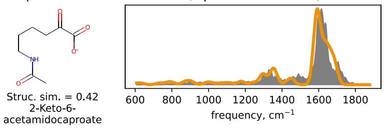

deprotonated HMDB0000398, spectral sim. = 940, #6

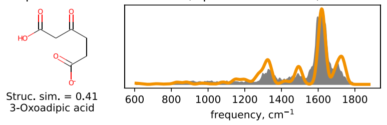

deprotonated HMDB0000422, spectral sim. = 939, #7

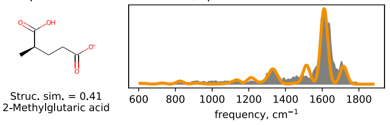

deprotonated HMDB0061676, spectral sim. = 939, #8

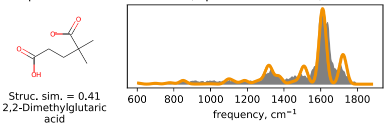

deprotonated HMDB0029167, spectral sim. = 939, #9

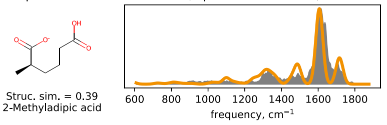

22 protonated HMDB0000182

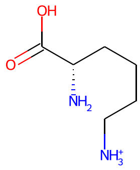

Spectra of protonated HMDB0000182, spectral sim. = 871, #107

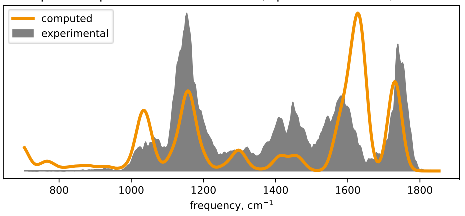

Structural similarity plot of protonated HMDB0000182

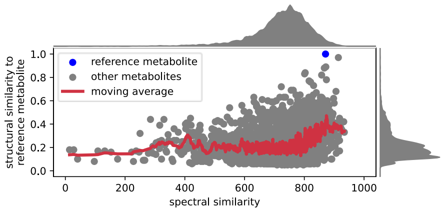

protonated HMDB0060346, spectral sim. = 934, #1

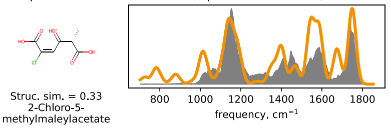

protonated HMDB0011667, spectral sim. = 932, #2

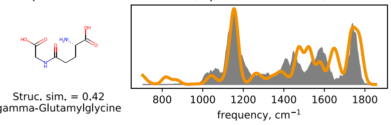

protonated HMDB0060369, spectral sim. = 926, #3

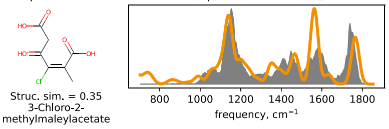

protonated HMDB0060347, spectral sim. = 923, #4

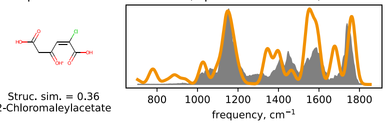

protonated HMDB0032797, spectral sim. = 922, #5

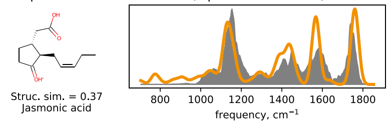

protonated HMDB0040216, spectral sim. = 918, #6

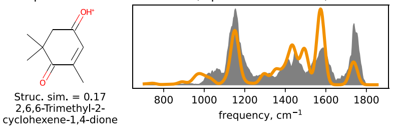

protonated HMDB0060722, spectral sim. = 918, #7

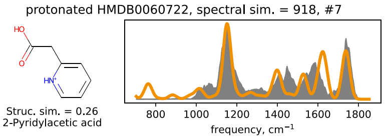

protonated HMDB0029419, spectral sim. = 918, #8

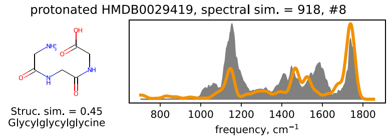

protonated HMDB0001268, spectral sim. = 917, #9

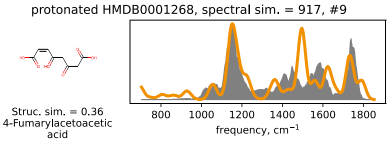

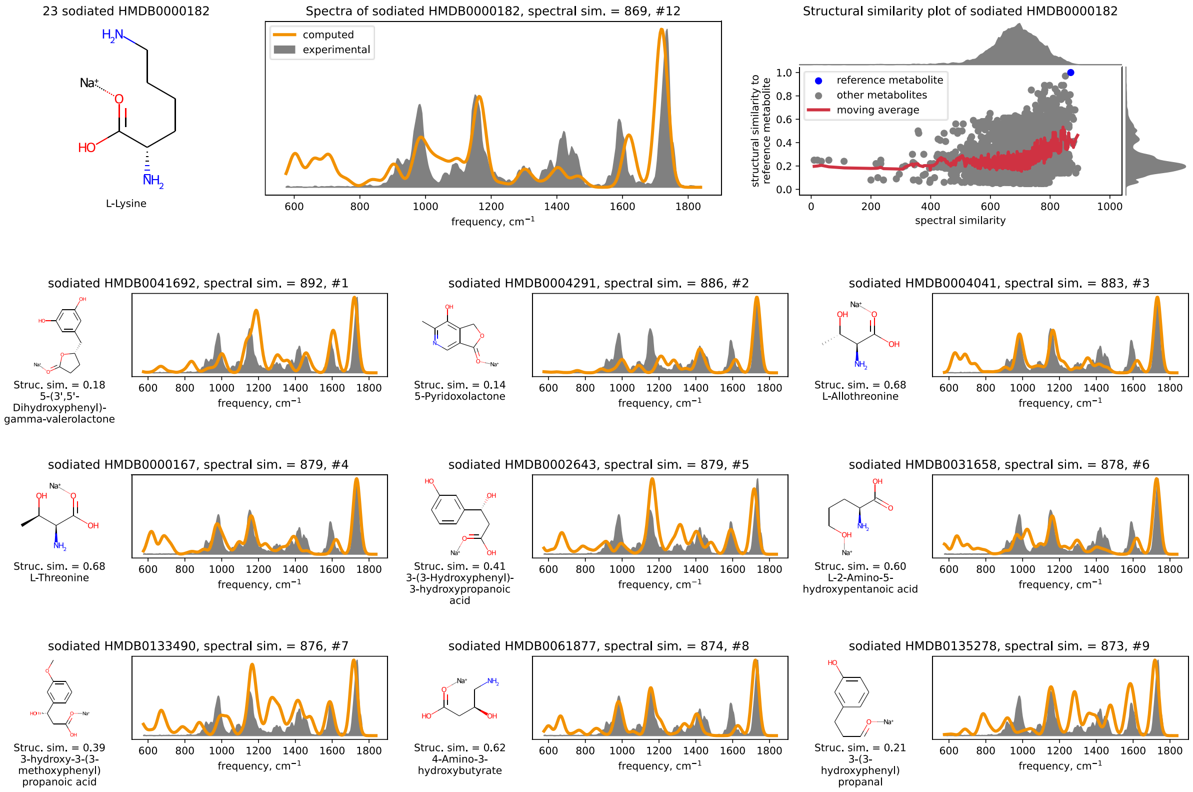
23 sodiated HMDB0000182
L-Lysine
Spectra of sodiated HMDB0000182, spectral sim. = 869, #12
computed
experimental
frequency, cm^{-1}
Structural similarity plot of sodiated HMDB0000182
reference metabolite
other metabolites
moving average
structural similarity to reference metabolite
spectral similarity
sodiated HMDB0041692, spectral sim. = 892, #1
Struc. sim. = 0.18
5-(3',5'-Dihydroxyphenyl)-gamma-valerolactone
sodiated HMDB0004291, spectral sim. = 886, #2
Struc. sim. = 0.14
5-Pyridoxolactone
sodiated HMDB0004041, spectral sim. = 883, #3
Struc. sim. = 0.68
L-Allothreonine
sodiated HMDB0000167, spectral sim. = 879, #4
Struc. sim. = 0.68
L-Threonine
sodiated HMDB0002643, spectral sim. = 879, #5
Struc. sim. = 0.41
3-(3-Hydroxyphenyl)-3-hydroxypropanoic acid
sodiated HMDB0031658, spectral sim. = 878, #6
Struc. sim. = 0.60
L-2-Amino-5-hydroxypentanoic acid
sodiated HMDB0133490, spectral sim. = 876, #7
Struc. sim. = 0.39
3-hydroxy-3-(3-methoxyphenyl) propanoic acid
sodiated HMDB0061877, spectral sim. = 874, #8
Struc. sim. = 0.62
4-Amino-3-hydroxybutyrate
sodiated HMDB0135278, spectral sim. = 873, #9
Struc. sim. = 0.21
3-(3-hydroxyphenyl) propanal

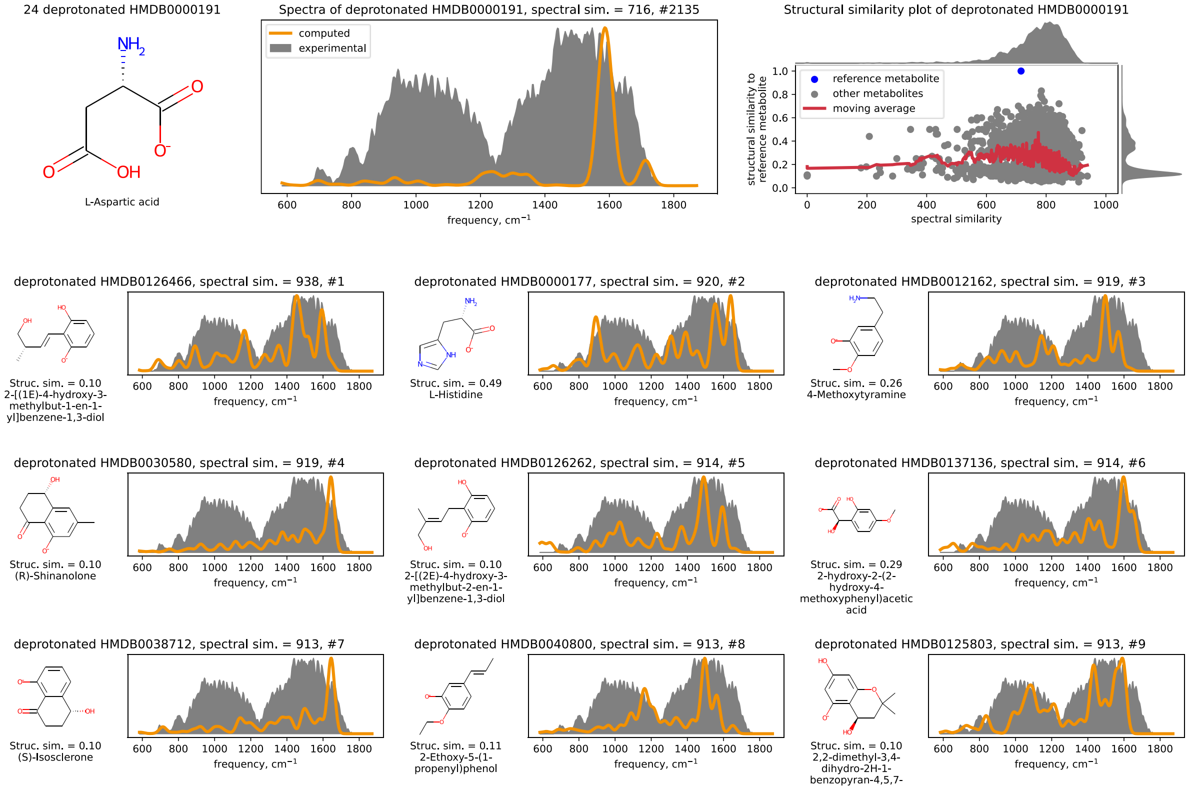
24 deprotonated HMDB0000191
L-Aspartic acid
Spectra of deprotonated HMDB0000191, spectral sim. = 716, #2135
computed
experimental
frequency, cm^{-1}
Structural similarity plot of deprotonated HMDB0000191
reference metabolite
other metabolites
moving average
structural similarity to reference metabolite
spectral similarity
deprotonated HMDB0126466, spectral sim. = 938, #1
Struc. sim. = 0.10
2-[(1E)-4-hydroxy-3-methylbut-1-en-1-yl]benzene-1,3-diol
deprotonated HMDB0000177, spectral sim. = 920, #2
Struc. sim. = 0.49
L-Histidine
deprotonated HMDB0012162, spectral sim. = 919, #3
Struc. sim. = 0.26
4-Methoxytyramine
deprotonated HMDB0030580, spectral sim. = 919, #4
Struc. sim. = 0.10
(R)-Shinanolone
deprotonated HMDB0126262, spectral sim. = 914, #5
Struc. sim. = 0.10
2-[(2E)-4-hydroxy-3-methylbut-2-en-1-yl]benzene-1,3-diol
deprotonated HMDB0137136, spectral sim. = 914, #6
Struc. sim. = 0.29
2-hydroxy-2-(2-hydroxy-4-methoxyphenyl)acetic acid
deprotonated HMDB0038712, spectral sim. = 913, #7
Struc. sim. = 0.10
(S)-Isosclerone
deprotonated HMDB0040800, spectral sim. = 913, #8
Struc. sim. = 0.11
2-Ethoxy-5-(1-propenyl)phenol
deprotonated HMDB0125803, spectral sim. = 913, #9
Struc. sim. = 0.10
2,2-dimethyl-3,4-dihydro-2H-1-benzopyran-4,5,7-

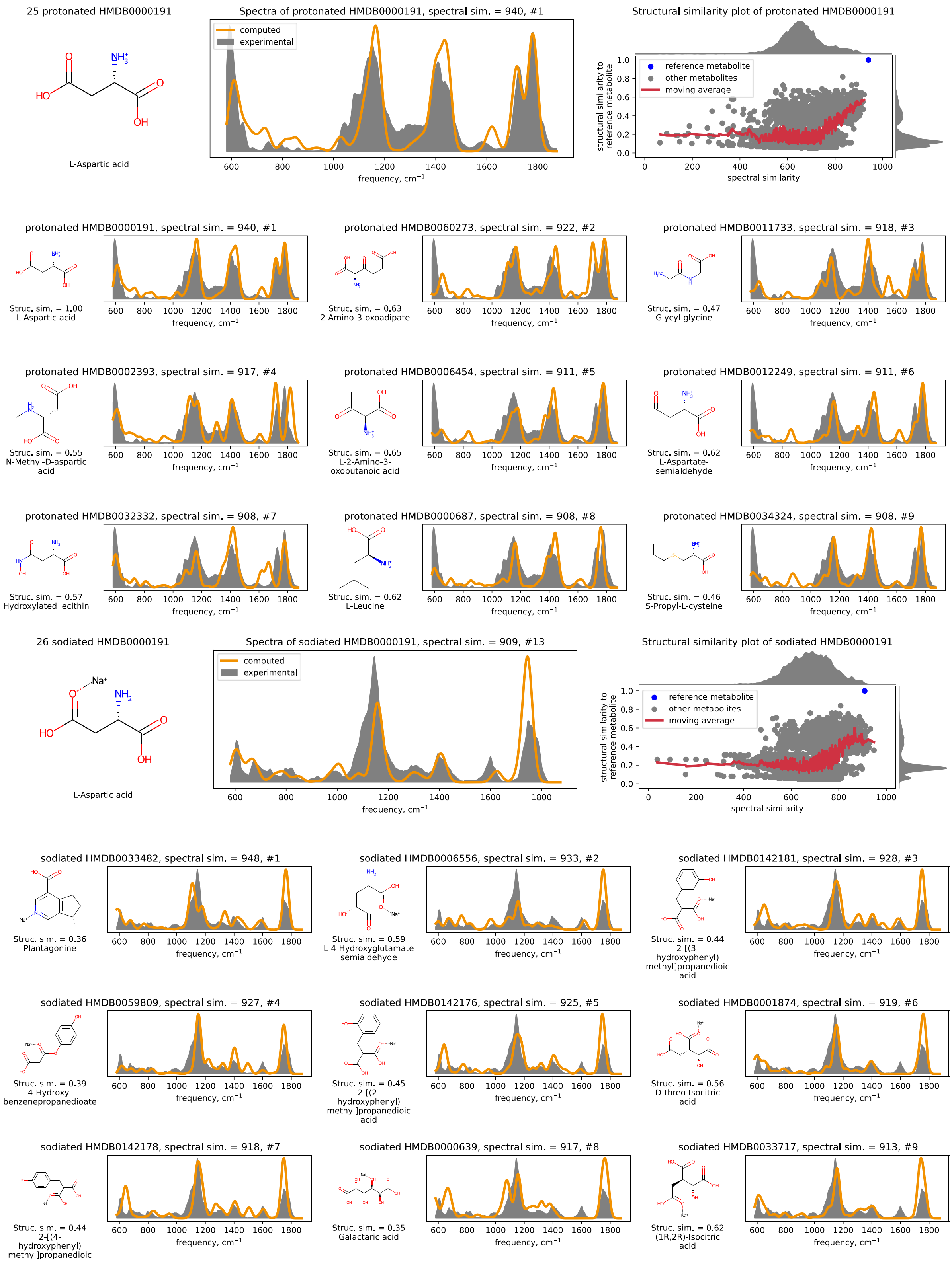
25 protonated HMDB0000191
L-Aspartic acid
Spectra of protonated HMDB0000191, spectral sim. = 940, #1
computed
experimental
frequency, cm^{-1}
Structural similarity plot of protonated HMDB0000191
reference metabolite
other metabolites
moving average
structural similarity to reference metabolite
spectral similarity
protonated HMDB0000191, spectral sim. = 940, #1
Struc. sim. = 1.00
L-Aspartic acid
protonated HMDB0060273, spectral sim. = 922, #2
Struc. sim. = 0.63
2-Amino-3-oxoadipate
protonated HMDB0011733, spectral sim. = 918, #3
Struc. sim. = 0.47
Glycyl-glycine
protonated HMDB0002393, spectral sim. = 917, #4
Struc. sim. = 0.55
N-Methyl-D-aspartic acid
protonated HMDB0006454, spectral sim. = 911, #5
Struc. sim. = 0.65
L-2-Amino-3-oxobutanoic acid
protonated HMDB0012249, spectral sim. = 911, #6
Struc. sim. = 0.62
L-Aspartate-semialdehyde
protonated HMDB0032332, spectral sim. = 908, #7
Struc. sim. = 0.57
Hydroxylated lecithin
protonated HMDB0000687, spectral sim. = 908, #8
Struc. sim. = 0.62
L-Leucine
protonated HMDB0034324, spectral sim. = 908, #9
Struc. sim. = 0.46
S-Propyl-L-cysteine
26 sodiated HMDB0000191
L-Aspartic acid
Spectra of sodiated HMDB0000191, spectral sim. = 909, #13
Structural similarity plot of sodiated HMDB0000191
sodiated HMDB0033482, spectral sim. = 948, #1
Struc. sim. = 0.36
Plantagonine
sodiated HMDB0006556, spectral sim. = 933, #2
Struc. sim. = 0.59
L-4-Hydroxyglutamate semialdehyde
sodiated HMDB0142181, spectral sim. = 928, #3
Struc. sim. = 0.44
2-[(3-hydroxyphenyl) methyl]propanedioic acid
sodiated HMDB0059809, spectral sim. = 927, #4
Struc. sim. = 0.39
4-Hydroxy-benzenepropanedioate
sodiated HMDB0142176, spectral sim. = 925, #5
Struc. sim. = 0.45
2-[(2-hydroxyphenyl) methyl]propanedioic acid
sodiated HMDB0001874, spectral sim. = 919, #6
Struc. sim. = 0.56
D-threo-Isocitric acid
sodiated HMDB0142178, spectral sim. = 918, #7
Struc. sim. = 0.44
2-[(4-hydroxyphenyl) methyl]propanedioic
sodiated HMDB0000639, spectral sim. = 917, #8
Struc. sim. = 0.35
Galactaric acid
sodiated HMDB0033717, spectral sim. = 913, #9
Struc. sim. = 0.62
(1R,2R)-Isocitric acid

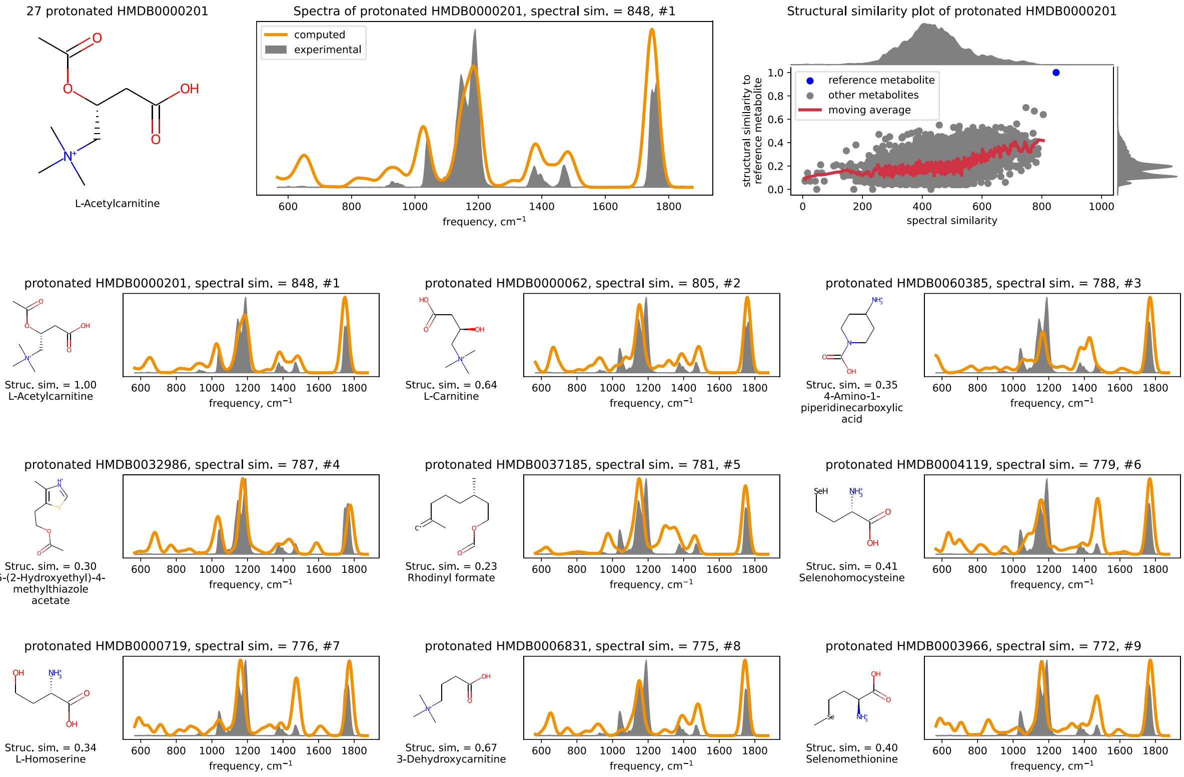
27 protonated HMDB0000201
L-Acetylcarnitine
Spectra of protonated HMDB0000201, spectral sim. = 848, #1
computed
experimental
frequency, cm⁻¹
Structural similarity plot of protonated HMDB0000201
reference metabolite
other metabolites
moving average
structural similarity to reference metabolite
spectral similarity
protonated HMDB0000201, spectral sim. = 848, #1
Struc. sim. = 1.00
L-Acetylcarnitine
protonated HMDB0000062, spectral sim. = 805, #2
Struc. sim. = 0.64
L-Carnitine
protonated HMDB0060385, spectral sim. = 788, #3
Struc. sim. = 0.35
4-Amino-1-piperidinecarboxylic acid
protonated HMDB0032986, spectral sim. = 787, #4
Struc. sim. = 0.30
5-(2-Hydroxyethyl)-4-methylthiazole acetate
protonated HMDB0037185, spectral sim. = 781, #5
Struc. sim. = 0.23
Rhodinyl formate
protonated HMDB0004119, spectral sim. = 779, #6
Struc. sim. = 0.41
Selenohomocysteine
protonated HMDB0000719, spectral sim. = 776, #7
Struc. sim. = 0.34
L-Homoserine
protonated HMDB0006831, spectral sim. = 775, #8
Struc. sim. = 0.67
3-Dehydroxycarnitine
protonated HMDB0003966, spectral sim. = 772, #9
Struc. sim. = 0.40
Selenomethionine

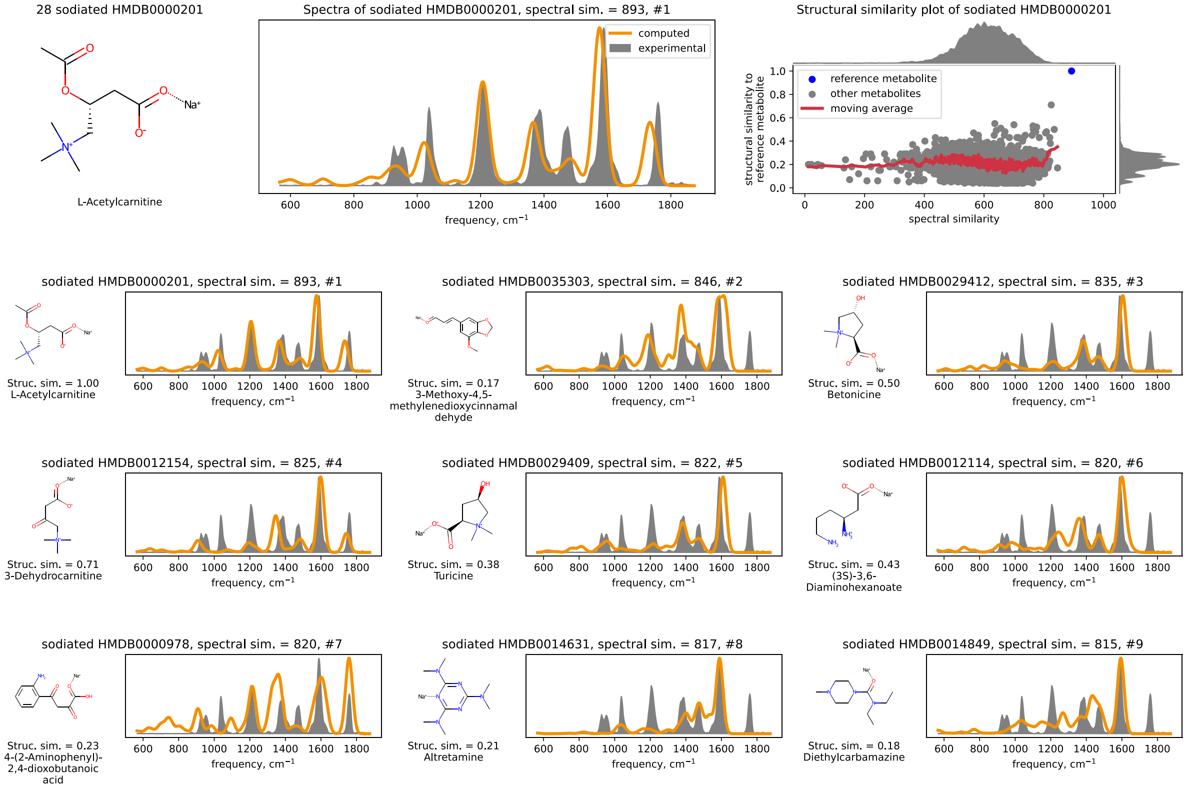
28 sodiated HMDB0000201
L-Acetylcarnitine
Spectra of sodiated HMDB0000201, spectral sim. = 893, #1
computed
experimental
frequency, cm⁻¹
Structural similarity plot of sodiated HMDB0000201
reference metabolite
other metabolites
moving average
structural similarity to reference metabolite
spectral similarity
sodiated HMDB0000201, spectral sim. = 893, #1
Struc. sim. = 1.00
L-Acetylcarnitine
sodiated HMDB0035303, spectral sim. = 846, #2
Struc. sim. = 0.17
3-Methoxy-4,5-methylenedioxycinnamaldehyde
sodiated HMDB0029412, spectral sim. = 835, #3
Struc. sim. = 0.50
Betonicine
sodiated HMDB0012154, spectral sim. = 825, #4
Struc. sim. = 0.71
3-Dehydrocarnitine
sodiated HMDB0029409, spectral sim. = 822, #5
Struc. sim. = 0.38
Turicine
sodiated HMDB0012114, spectral sim. = 820, #6
Struc. sim. = 0.43
(3S)-3,6-Diaminohexanoate
sodiated HMDB0000978, spectral sim. = 820, #7
Struc. sim. = 0.23
4-(2-Aminophenyl)-2,4-dioxobutanoic acid
sodiated HMDB0014631, spectral sim. = 817, #8
Struc. sim. = 0.21
Altretamine
sodiated HMDB0014849, spectral sim. = 815, #9
Struc. sim. = 0.18
Diethylcarbamazine

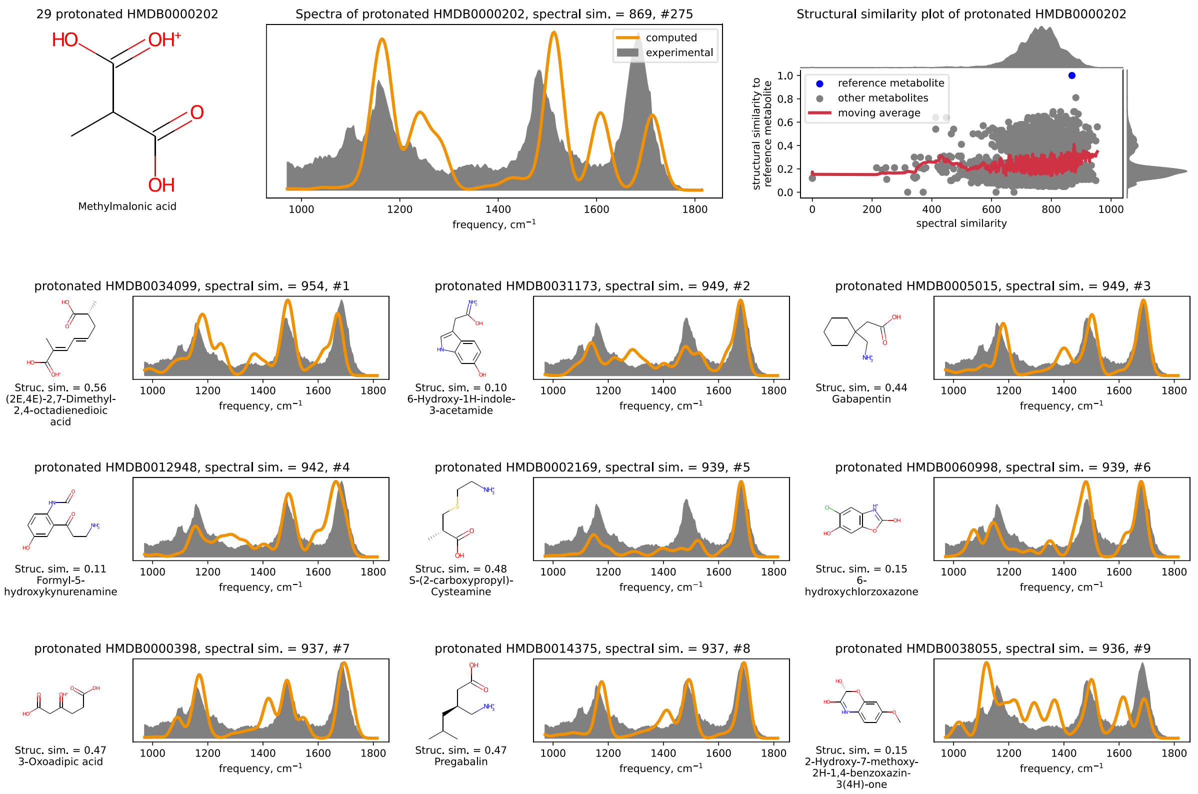
29 protonated HMDB0000202
Methylmalonic acid
Spectra of protonated HMDB0000202, spectral sim. = 869, #275
computed
experimental
frequency, cm^{-1}
Structural similarity plot of protonated HMDB0000202
reference metabolite
other metabolites
moving average
structural similarity to reference metabolite
spectral similarity
protonated HMDB0034099, spectral sim. = 954, #1
Struc. sim. = 0.56
(2E,4E)-2,7-Dimethyl-2,4-octadienedioic acid
protonated HMDB0031173, spectral sim. = 949, #2
Struc. sim. = 0.10
6-Hydroxy-1H-indole-3-acetamide
protonated HMDB0005015, spectral sim. = 949, #3
Struc. sim. = 0.44
Gabapentin
protonated HMDB0012948, spectral sim. = 942, #4
Struc. sim. = 0.11
Formyl-5-hydroxykynurenamine
protonated HMDB0002169, spectral sim. = 939, #5
Struc. sim. = 0.48
S-(2-carboxypropyl)-Cysteamine
protonated HMDB0060998, spectral sim. = 939, #6
Struc. sim. = 0.15
6-hydroxychlorzoxazone
protonated HMDB0000398, spectral sim. = 937, #7
Struc. sim. = 0.47
3-Oxoadipic acid
protonated HMDB0014375, spectral sim. = 937, #8
Struc. sim. = 0.47
Pregabalin
protonated HMDB0038055, spectral sim. = 936, #9
Struc. sim. = 0.15
2-Hydroxy-7-methoxy-2H-1,4-benzoxazin-3(4H)-one
frequency, cm^{-1}

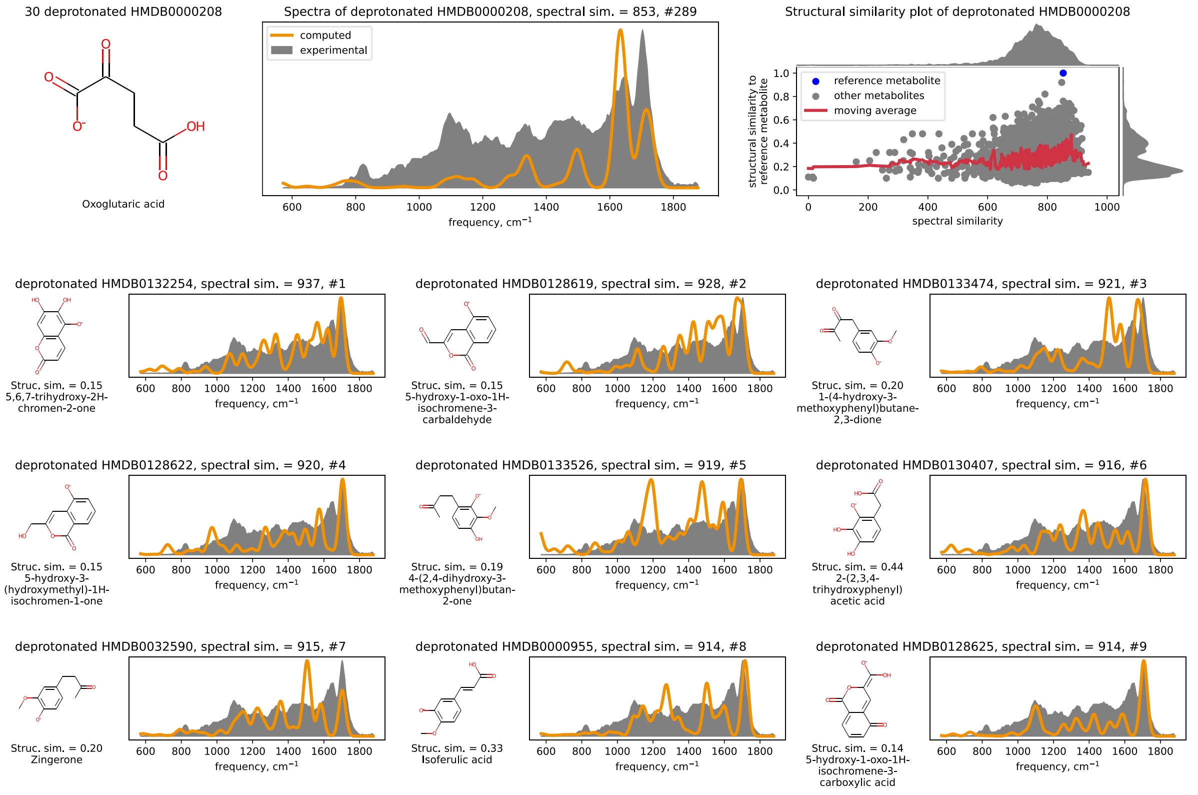
30 deprotonated HMDB0000208
Oxoglutaric acid
Spectra of deprotonated HMDB0000208, spectral sim. = 853, #289
computed
experimental
frequency, cm^{-1}
Structural similarity plot of deprotonated HMDB0000208
reference metabolite
other metabolites
moving average
structural similarity to reference metabolite
spectral similarity
deprotonated HMDB0132254, spectral sim. = 937, #1
Struc. sim. = 0.15
5,6,7-trihydroxy-2H-chromen-2-one
deprotonated HMDB0128619, spectral sim. = 928, #2
Struc. sim. = 0.15
5-hydroxy-1-oxo-1H-isochromene-3-carbaldehyde
deprotonated HMDB0133474, spectral sim. = 921, #3
Struc. sim. = 0.20
1-(4-hydroxy-3-methoxyphenyl)butane-2,3-dione
deprotonated HMDB0128622, spectral sim. = 920, #4
Struc. sim. = 0.15
5-hydroxy-3-(hydroxymethyl)-1H-isochromen-1-one
deprotonated HMDB0133526, spectral sim. = 919, #5
Struc. sim. = 0.19
4-(2,4-dihydroxy-3-methoxyphenyl)butan-2-one
deprotonated HMDB0130407, spectral sim. = 916, #6
Struc. sim. = 0.44
2-(2,3,4-trihydroxyphenyl)acetic acid
deprotonated HMDB0032590, spectral sim. = 915, #7
Struc. sim. = 0.20
Zingerone
deprotonated HMDB0000955, spectral sim. = 914, #8
Struc. sim. = 0.33
Isoferulic acid
deprotonated HMDB0128625, spectral sim. = 914, #9
Struc. sim. = 0.14
5-hydroxy-1-oxo-1H-isochromene-3-carboxylic acid
frequency, cm^{-1}

31 sodiated HMDB0000208

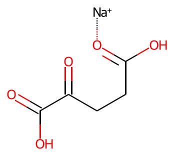

Oxoglutaric acid

Spectra of sodiated HMDB0000208, spectral sim. = 904, #12

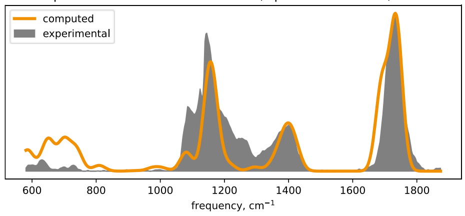

Structural similarity plot of sodiated HMDB0000208

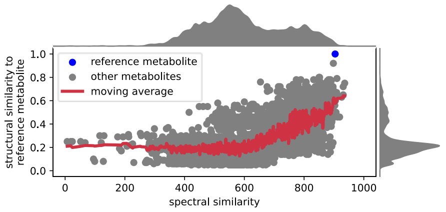

sodiated HMDB0240258, spectral sim. = 936, #1

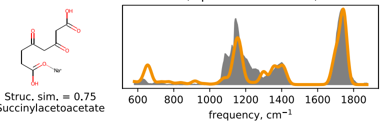

Struc. sim. = 0.75
Succinylacetoacetate

sodiated HMDB0059932, spectral sim. = 931, #2

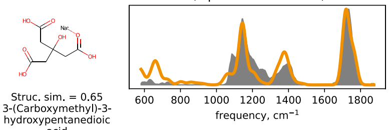

Struc. sim. = 0.65
3-(Carboxymethyl)-3-hydroxypentanedioic acid

sodiated HMDB0000635, spectral sim. = 931, #3

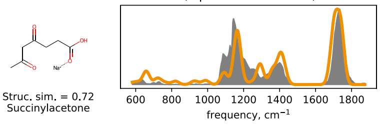

Struc. sim. = 0.72
Succinylacetone

sodiated HMDB0000094, spectral sim. = 926, #4

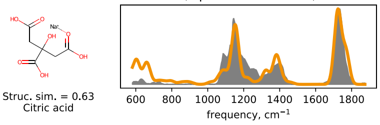

Struc. sim. = 0.63
Citric acid

sodiated HMDB0040531, spectral sim. = 919, #5

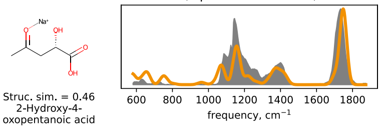

Struc. sim. = 0.46
2-Hydroxy-4-oxopentanoic acid

sodiated HMDB0000576, spectral sim. = 912, #6

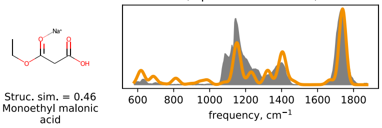

Struc. sim. = 0.46
Monoethyl malonic acid

sodiated HMDB0000398, spectral sim. = 910, #7

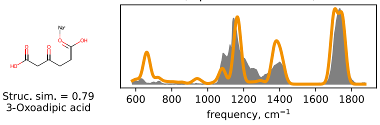

Struc. sim. = 0.79
3-Oxoadipic acid

sodiated HMDB0031193, spectral sim. = 909, #8

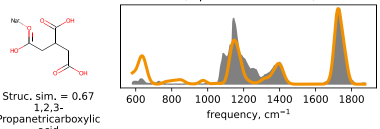

Struc. sim. = 0.67
1,2,3-Propanetricarboxylic acid

sodiated HMDB0060320, spectral sim. = 909, #9

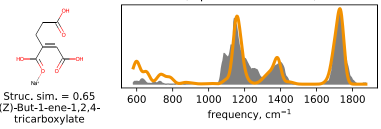

Struc. sim. = 0.65
(Z)-But-1-ene-1,2,4-tricarboxylate

32 deprotonated HMDB0000211

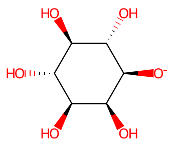

myo-Inositol

Spectra of deprotonated HMDB0000211, spectral sim. = 838, #38

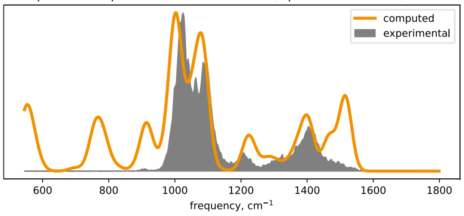

Structural similarity plot of deprotonated HMDB0000211

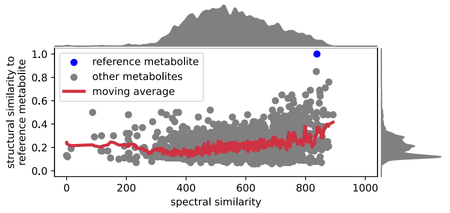

deprotonated HMDB0033942, spectral sim. = 892, #1

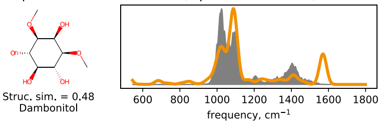

Struc. sim. = 0.48
Dambonitol

deprotonated HMDB0029915, spectral sim. = 878, #2

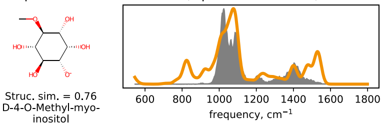

Struc. sim. = 0.76
D-4-O-Methyl-myo-inositol

deprotonated HMDB0031102, spectral sim. = 877, #3

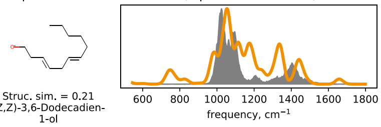

Struc. sim. = 0.21
(Z,Z)-3,6-Dodecadien-1-ol

deprotonated HMDB0034221, spectral sim. = 875, #4

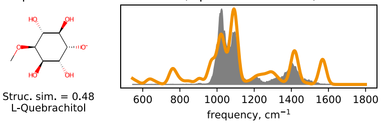

Struc. sim. = 0.48
L-Quebrachitol

deprotonated HMDB0011624, spectral sim. = 874, #5

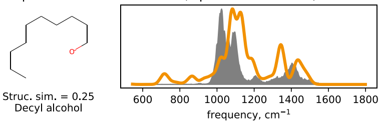

Struc. sim. = 0.25
Decyl alcohol

deprotonated HMDB0031065, spectral sim. = 871, #6

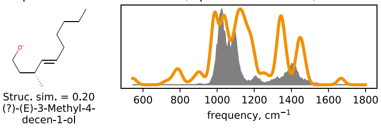

Struc. sim. = 0.20
(?)-(E)-3-Methyl-4-decen-1-ol

deprotonated HMDB0059889, spectral sim. = 866, #7

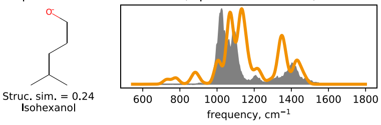

Struc. sim. = 0.24
Isohexanol

deprotonated HMDB0240210, spectral sim. = 866, #8

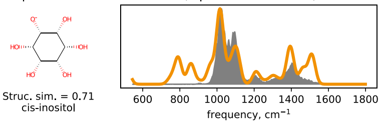

Struc. sim. = 0.71
cis-inositol

deprotonated HMDB0013113, spectral sim. = 861, #9

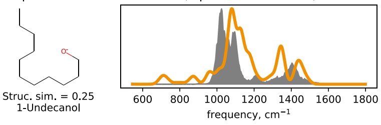

Struc. sim. = 0.25
1-Undecanol

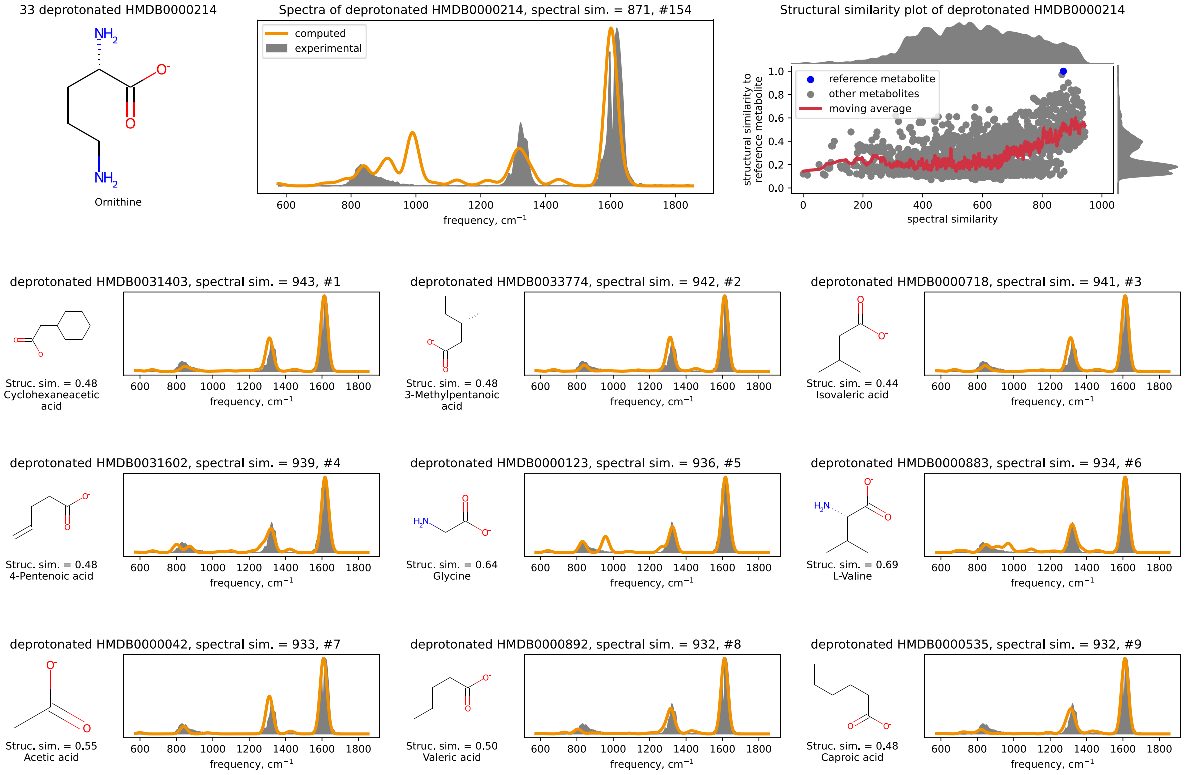
33 deprotonated HMDB0000214
Ornithine
Spectra of deprotonated HMDB0000214, spectral sim. = 871, #154
computed
experimental
frequency, cm⁻¹
Structural similarity plot of deprotonated HMDB0000214
reference metabolite
other metabolites
moving average
structural similarity to reference metabolite
spectral similarity
deprotonated HMDB0031403, spectral sim. = 943, #1
Struc. sim. = 0.48
Cyclohexaneacetic acid
deprotonated HMDB0033774, spectral sim. = 942, #2
Struc. sim. = 0.48
3-Methylpentanoic acid
deprotonated HMDB0000718, spectral sim. = 941, #3
Struc. sim. = 0.44
Isovaleric acid
deprotonated HMDB0031602, spectral sim. = 939, #4
Struc. sim. = 0.48
4-Pentenoic acid
deprotonated HMDB0000123, spectral sim. = 936, #5
Struc. sim. = 0.64
Glycine
deprotonated HMDB0000883, spectral sim. = 934, #6
Struc. sim. = 0.69
L-Valine
deprotonated HMDB0000042, spectral sim. = 933, #7
Struc. sim. = 0.55
Acetic acid
deprotonated HMDB0000892, spectral sim. = 932, #8
Struc. sim. = 0.50
Valeric acid
deprotonated HMDB0000535, spectral sim. = 932, #9
Struc. sim. = 0.48
Caproic acid

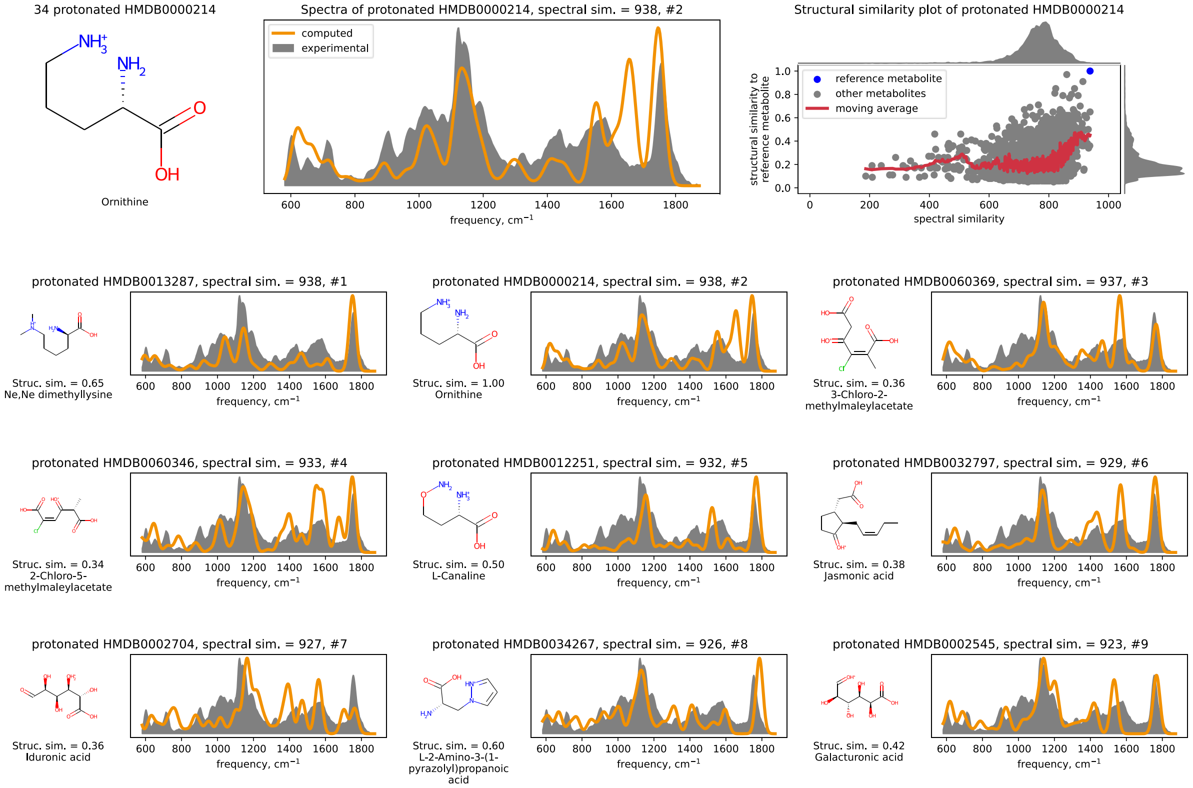
34 protonated HMDB0000214
Ornithine
Spectra of protonated HMDB0000214, spectral sim. = 938, #2
computed
experimental
frequency, cm⁻¹
Structural similarity plot of protonated HMDB0000214
reference metabolite
other metabolites
moving average
structural similarity to reference metabolite
spectral similarity
protonated HMDB0013287, spectral sim. = 938, #1
Struc. sim. = 0.65
Ne,Ne dimethyllysine
protonated HMDB0000214, spectral sim. = 938, #2
Struc. sim. = 1.00
Ornithine
protonated HMDB0060369, spectral sim. = 937, #3
Struc. sim. = 0.36
3-Chloro-2-methylmaleylacetate
protonated HMDB0060346, spectral sim. = 933, #4
Struc. sim. = 0.34
2-Chloro-5-methylmaleylacetate
protonated HMDB0012251, spectral sim. = 932, #5
Struc. sim. = 0.50
L-Canaline
protonated HMDB0032797, spectral sim. = 929, #6
Struc. sim. = 0.38
Jasmonic acid
protonated HMDB0002704, spectral sim. = 927, #7
Struc. sim. = 0.36
Iduronic acid
protonated HMDB0034267, spectral sim. = 926, #8
Struc. sim. = 0.60
L-2-Amino-3-(1-pyrazolyl)propanoic acid
protonated HMDB0002545, spectral sim. = 923, #9
Struc. sim. = 0.42
Galacturonic acid

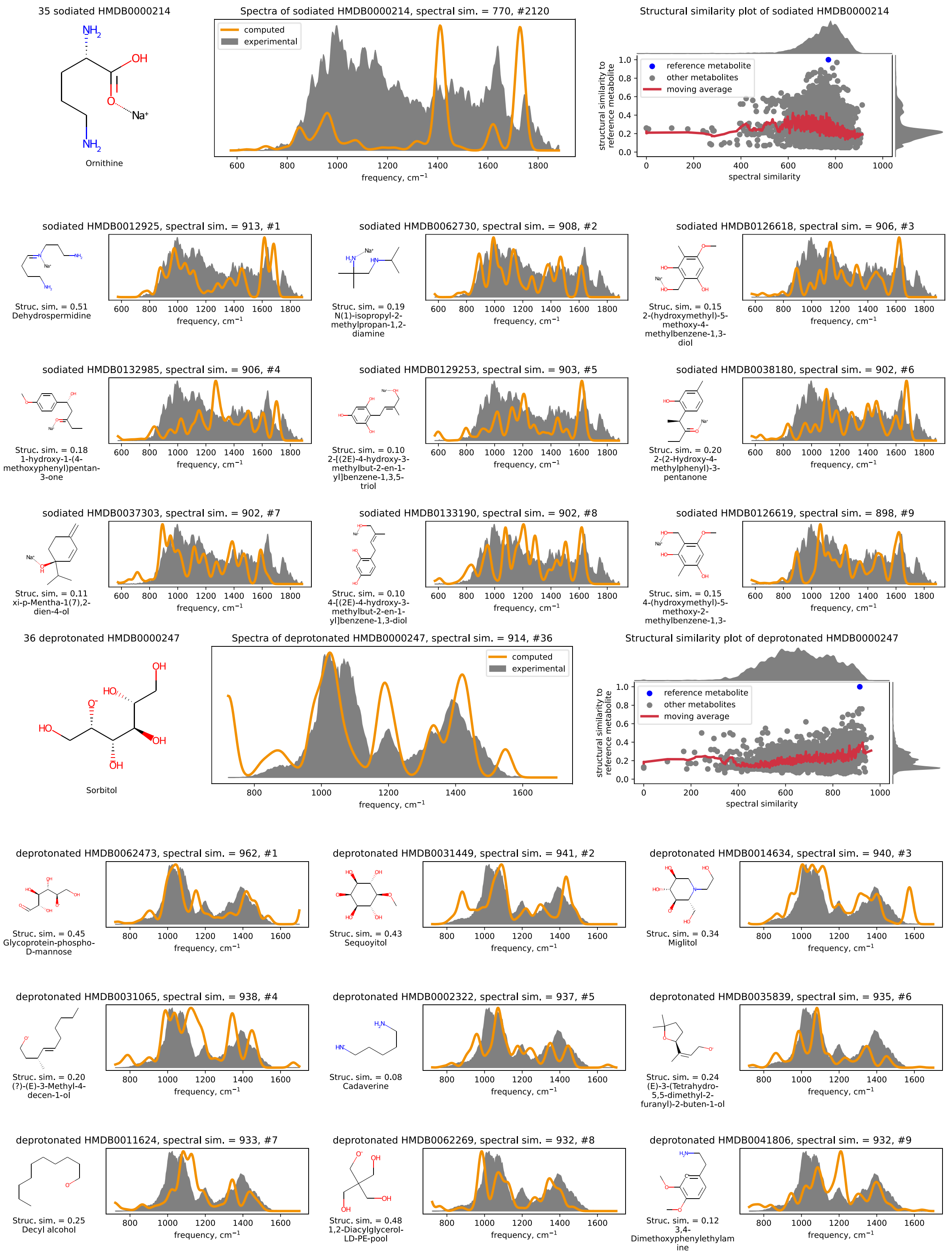

35 sodiated HMDB0000214
Ornithine
Spectra of sodiated HMDB0000214, spectral sim. = 770, #2120
computed
experimental
frequency, cm⁻¹
Structural similarity plot of sodiated HMDB0000214
reference metabolite
other metabolites
moving average
structural similarity to reference metabolite
spectral similarity
sodiated HMDB0012925, spectral sim. = 913, #1
Struc. sim. = 0.51
Dehydrospermidine
sodiated HMDB0062730, spectral sim. = 908, #2
Struc. sim. = 0.19
N(1)-isopropyl-2-methylpropan-1,2-diamine
sodiated HMDB0126618, spectral sim. = 906, #3
Struc. sim. = 0.15
2-(hydroxymethyl)-5-methoxy-4-methylbenzene-1,3-diol
sodiated HMDB0132985, spectral sim. = 906, #4
Struc. sim. = 0.18
1-hydroxy-1-(4-methoxyphenyl)pentan-3-one
sodiated HMDB0129253, spectral sim. = 903, #5
Struc. sim. = 0.10
2-[(2E)-4-hydroxy-3-methylbut-2-en-1-yl]benzene-1,3,5-triol
sodiated HMDB0038180, spectral sim. = 902, #6
Struc. sim. = 0.20
2-(2-Hydroxy-4-methylphenyl)-3-pentanone
sodiated HMDB0037303, spectral sim. = 902, #7
Struc. sim. = 0.11
xi-p-Mentha-1(7),2-dien-4-ol
sodiated HMDB0133190, spectral sim. = 902, #8
Struc. sim. = 0.10
4-[(2E)-4-hydroxy-3-methylbut-2-en-1-yl]benzene-1,3-diol
sodiated HMDB0126619, spectral sim. = 898, #9
Struc. sim. = 0.15
4-(hydroxymethyl)-5-methoxy-2-methylbenzene-1,3-
36 deprotonated HMDB0000247
Sorbitol
Spectra of deprotonated HMDB0000247, spectral sim. = 914, #36
Structural similarity plot of deprotonated HMDB0000247
deprotonated HMDB0062473, spectral sim. = 962, #1
Struc. sim. = 0.45
Glycoprotein-phospho-D-mannose
deprotonated HMDB0031449, spectral sim. = 941, #2
Struc. sim. = 0.43
Sequoyitol
deprotonated HMDB0014634, spectral sim. = 940, #3
Struc. sim. = 0.34
Miglitol
deprotonated HMDB0031065, spectral sim. = 938, #4
Struc. sim. = 0.20
(?)-(E)-3-Methyl-4-decen-1-ol
deprotonated HMDB0002322, spectral sim. = 937, #5
Struc. sim. = 0.08
Cadaverine
deprotonated HMDB0035839, spectral sim. = 935, #6
Struc. sim. = 0.24
(E)-3-(Tetrahydro-5,5-dimethyl-2-furanyl)-2-buten-1-ol
deprotonated HMDB0011624, spectral sim. = 933, #7
Struc. sim. = 0.25
Decyl alcohol
deprotonated HMDB0062269, spectral sim. = 932, #8
Struc. sim. = 0.48
1,2-Diacylglycerol-LD-PE-pool
deprotonated HMDB0041806, spectral sim. = 932, #9
Struc. sim. = 0.12
3,4-Dimethoxyphenylethylamine

37 deprotonated HMDB0000251

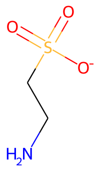

Taurine

Spectra of deprotonated HMDB0000251, spectral sim. = 459, #626

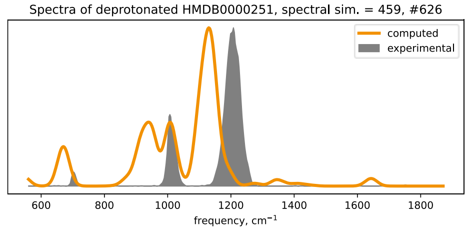

Structural similarity plot of deprotonated HMDB0000251

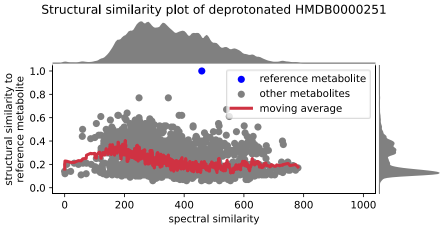

deprotonated HMDB0059803, spectral sim. = 781, #1

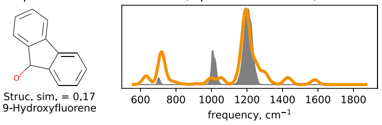

Struc. sim. = 0.17
9-Hydroxyfluorene

deprotonated HMDB0034666, spectral sim. = 773, #2

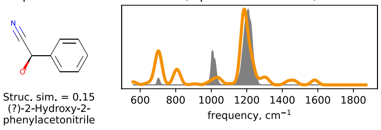

Struc. sim. = 0.15
(?)-2-Hydroxy-2-phenylacetonitrile

deprotonated HMDB0060427, spectral sim. = 765, #3

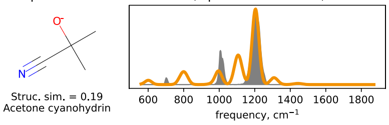

Struc. sim. = 0.19
Acetone cyanohydrin

deprotonated HMDB0059601, spectral sim. = 754, #4

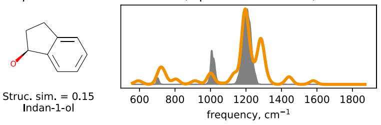

Struc. sim. = 0.15
Indan-1-ol

deprotonated HMDB0032450, spectral sim. = 749, #5

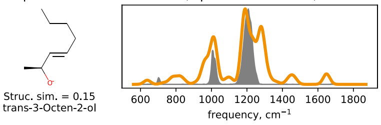

Struc. sim. = 0.15
trans-3-Octen-2-ol

deprotonated HMDB0002078, spectral sim. = 747, #6

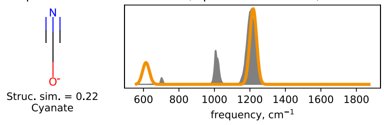

Struc. sim. = 0.22
Cyanate

deprotonated HMDB0001443, spectral sim. = 742, #7

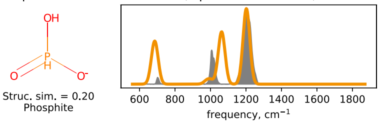

Struc. sim. = 0.20
Phosphite

deprotonated HMDB0059596, spectral sim. = 736, #8

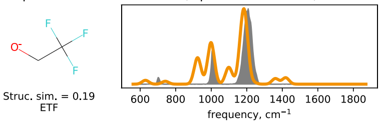

Struc. sim. = 0.19
ETF

deprotonated HMDB0011747, spectral sim. = 732, #9

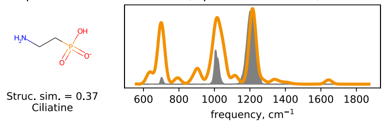

Struc. sim. = 0.37
Ciliatine

38 sodiated HMDB0000251

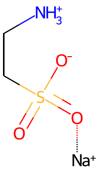

Taurine

Spectra of sodiated HMDB0000251, spectral sim. = 744, #577

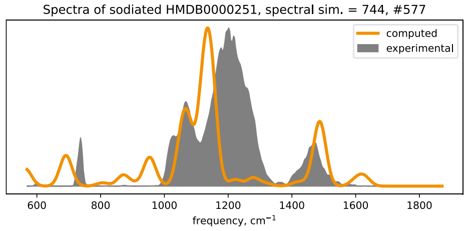

Structural similarity plot of sodiated HMDB0000251

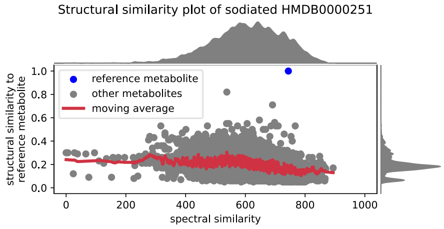

sodiated HMDB0004998, spectral sim. = 894, #1

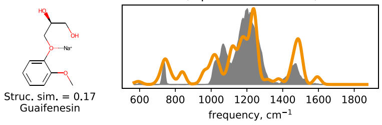

Struc. sim. = 0.17
Guaifenesin

sodiated HMDB0125521, spectral sim. = 869, #2

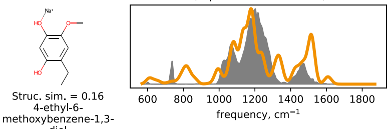

Struc. sim. = 0.16
4-ethyl-6-methoxybenzene-1,3-diol

sodiated HMDB0040175, spectral sim. = 868, #3

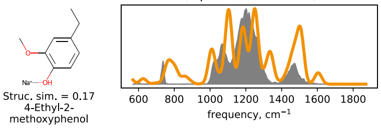

Struc. sim. = 0.17
4-Ethyl-2-methoxyphenol

sodiated HMDB0000957, spectral sim. = 867, #4

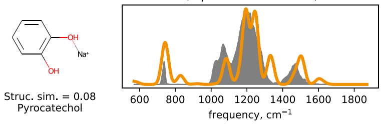

Struc. sim. = 0.08
Pyrocatechol

sodiated HMDB0062396, spectral sim. = 866, #5

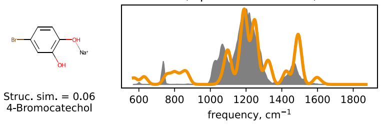

Struc. sim. = 0.06
4-Bromocatechol

sodiated HMDB0032135, spectral sim. = 865, #6

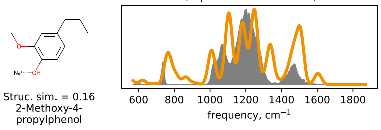

Struc. sim. = 0.16
2-Methoxy-4-propylphenol

sodiated HMDB0014023, spectral sim. = 865, #7

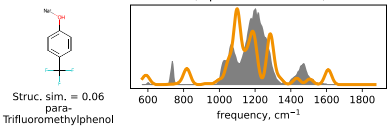

Struc. sim. = 0.06
para-Trifluoromethylphenol

sodiated HMDB0132907, spectral sim. = 864, #8

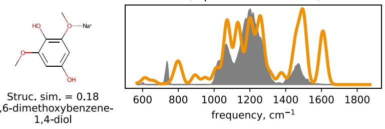

Struc. sim. = 0.18
2,6-dimethoxybenzene-1,4-diol

sodiated HMDB0032136, spectral sim. = 863, #9

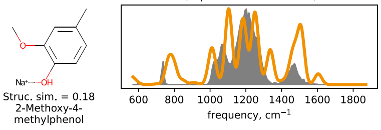

Struc. sim. = 0.18
2-Methoxy-4-methylphenol

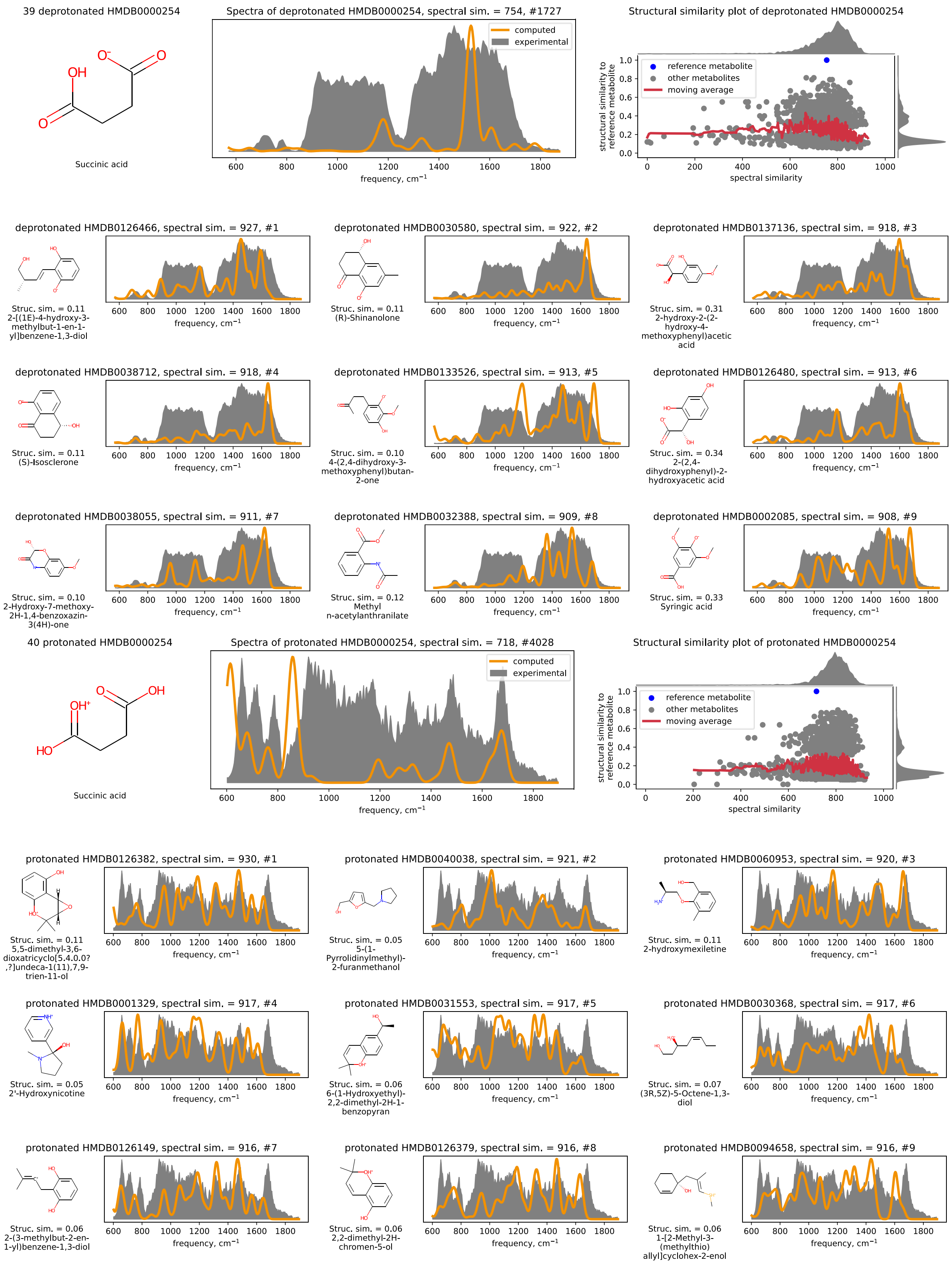
39 deprotonated HMDB0000254
Succinic acid
Spectra of deprotonated HMDB0000254, spectral sim. = 754, #1727
computed
experimental
frequency, cm⁻¹
Structural similarity plot of deprotonated HMDB0000254
reference metabolite
other metabolites
moving average
structural similarity to reference metabolite
spectral similarity
deprotonated HMDB0126466, spectral sim. = 927, #1
Struc. sim. = 0.11
2-[(1E)-4-hydroxy-3-methylbut-1-en-1-yl]benzene-1,3-diol
deprotonated HMDB0030580, spectral sim. = 922, #2
Struc. sim. = 0.11
(R)-Shinanolone
deprotonated HMDB0137136, spectral sim. = 918, #3
Struc. sim. = 0.31
2-hydroxy-2-(2-hydroxy-4-methoxyphenyl)acetic acid
deprotonated HMDB0038712, spectral sim. = 918, #4
Struc. sim. = 0.11
(S)-Isosclerone
deprotonated HMDB0133526, spectral sim. = 913, #5
Struc. sim. = 0.10
4-(2,4-dihydroxy-3-methoxyphenyl)butan-2-one
deprotonated HMDB0126480, spectral sim. = 913, #6
Struc. sim. = 0.34
2-(2,4-dihydroxyphenyl)-2-hydroxyacetic acid
deprotonated HMDB0038055, spectral sim. = 911, #7
Struc. sim. = 0.10
2-Hydroxy-7-methoxy-2H-1,4-benzoxazin-3(4H)-one
deprotonated HMDB0032388, spectral sim. = 909, #8
Struc. sim. = 0.12
Methyl n-acetylanthranilate
deprotonated HMDB0002085, spectral sim. = 908, #9
Struc. sim. = 0.33
Syringic acid
40 protonated HMDB0000254
Succinic acid
Spectra of protonated HMDB0000254, spectral sim. = 718, #4028
computed
experimental
Structural similarity plot of protonated HMDB0000254
reference metabolite
other metabolites
moving average
protonated HMDB0126382, spectral sim. = 930, #1
Struc. sim. = 0.11
5,5-dimethyl-3,6-dioxatricyclo[5.4.0.0?,?]undeca-1(11),7,9-trien-11-ol
protonated HMDB0040038, spectral sim. = 921, #2
Struc. sim. = 0.05
5-(1-Pyrrolidinylmethyl)-2-furanmethanol
protonated HMDB0060953, spectral sim. = 920, #3
Struc. sim. = 0.11
2-hydroxymexiletine
protonated HMDB0001329, spectral sim. = 917, #4
Struc. sim. = 0.05
2'-Hydroxynicotine
protonated HMDB0031553, spectral sim. = 917, #5
Struc. sim. = 0.06
6-(1-Hydroxyethyl)-2,2-dimethyl-2H-1-benzopyran
protonated HMDB0030368, spectral sim. = 917, #6
Struc. sim. = 0.07
(3R,5Z)-5-Octene-1,3-diol
protonated HMDB0126149, spectral sim. = 916, #7
Struc. sim. = 0.06
2-(3-methylbut-2-en-1-yl)benzene-1,3-diol
protonated HMDB0126379, spectral sim. = 916, #8
Struc. sim. = 0.06
2,2-dimethyl-2H-chromen-5-ol
protonated HMDB0094658, spectral sim. = 916, #9
Struc. sim. = 0.06
1-[2-Methyl-3-(methylthio)allyl]cyclohex-2-enol

41 deprotonated HMDB0000263

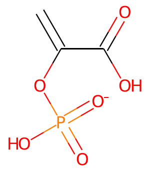

Phosphoenolpyruvic acid

Spectra of deprotonated HMDB0000263, spectral sim. = 817, #320

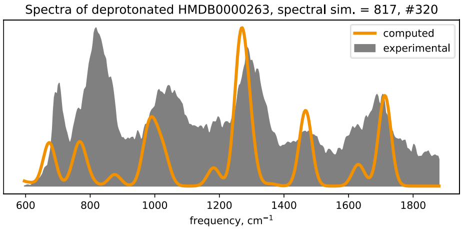

Structural similarity plot of deprotonated HMDB0000263

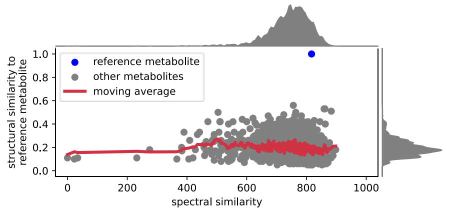

deprotonated HMDB0133682, spectral sim. = 898, #1

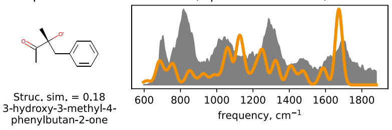

deprotonated HMDB0032465, spectral sim. = 889, #2

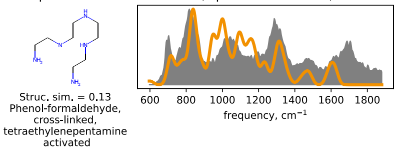

deprotonated HMDB0036990, spectral sim. = 883, #3

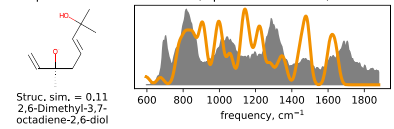

deprotonated HMDB0011185, spectral sim. = 883, #4

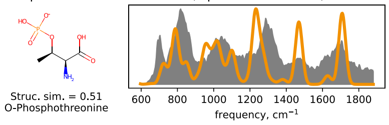

deprotonated HMDB0010207, spectral sim. = 881, #5

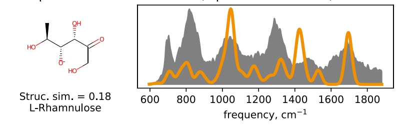

deprotonated HMDB0002016, spectral sim. = 877, #6

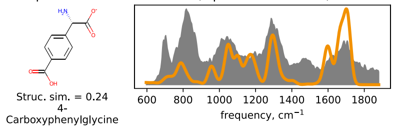

deprotonated HMDB0001385, spectral sim. = 875, #7

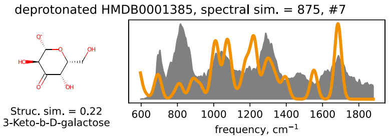

deprotonated HMDB0031625, spectral sim. = 874, #8

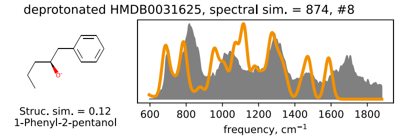

deprotonated HMDB0133484, spectral sim. = 874, #9

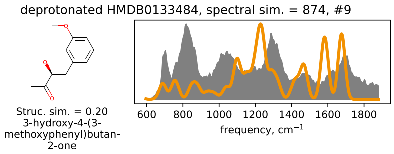

42 deprotonated HMDB0000272

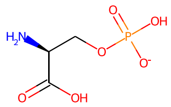

Phosphoserine

Spectra of deprotonated HMDB0000272, spectral sim. = 875, #16

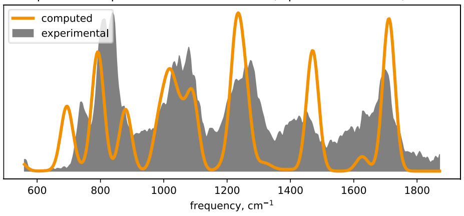

Structural similarity plot of deprotonated HMDB0000272

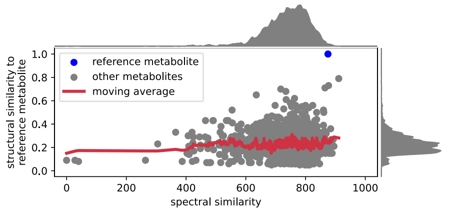

deprotonated HMDB0011185, spectral sim. = 911, #1

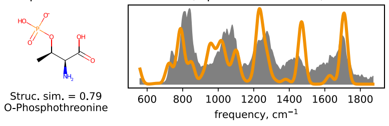

deprotonated HMDB0133682, spectral sim. = 895, #2

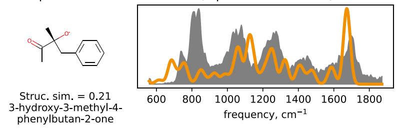

deprotonated HMDB0010207, spectral sim. = 893, #3

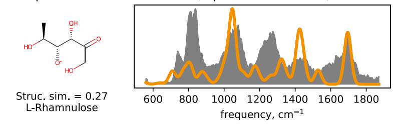

deprotonated HMDB0036990, spectral sim. = 889, #4

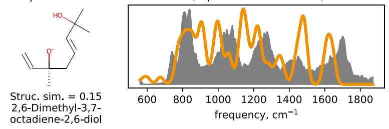

deprotonated HMDB0005876, spectral sim. = 889, #5

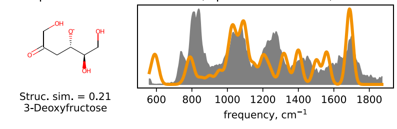

deprotonated HMDB0133484, spectral sim. = 888, #6

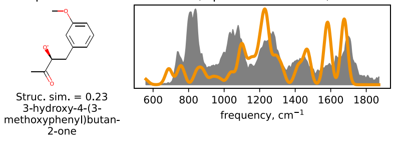

deprotonated HMDB0035122, spectral sim. = 885, #7

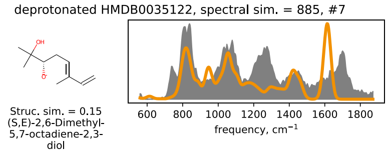

deprotonated HMDB0032465, spectral sim. = 884, #8

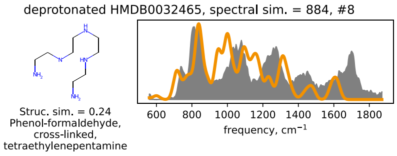

deprotonated HMDB0002016, spectral sim. = 884, #9

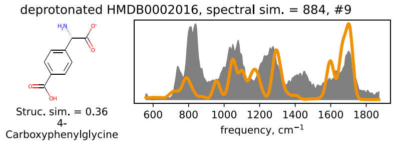

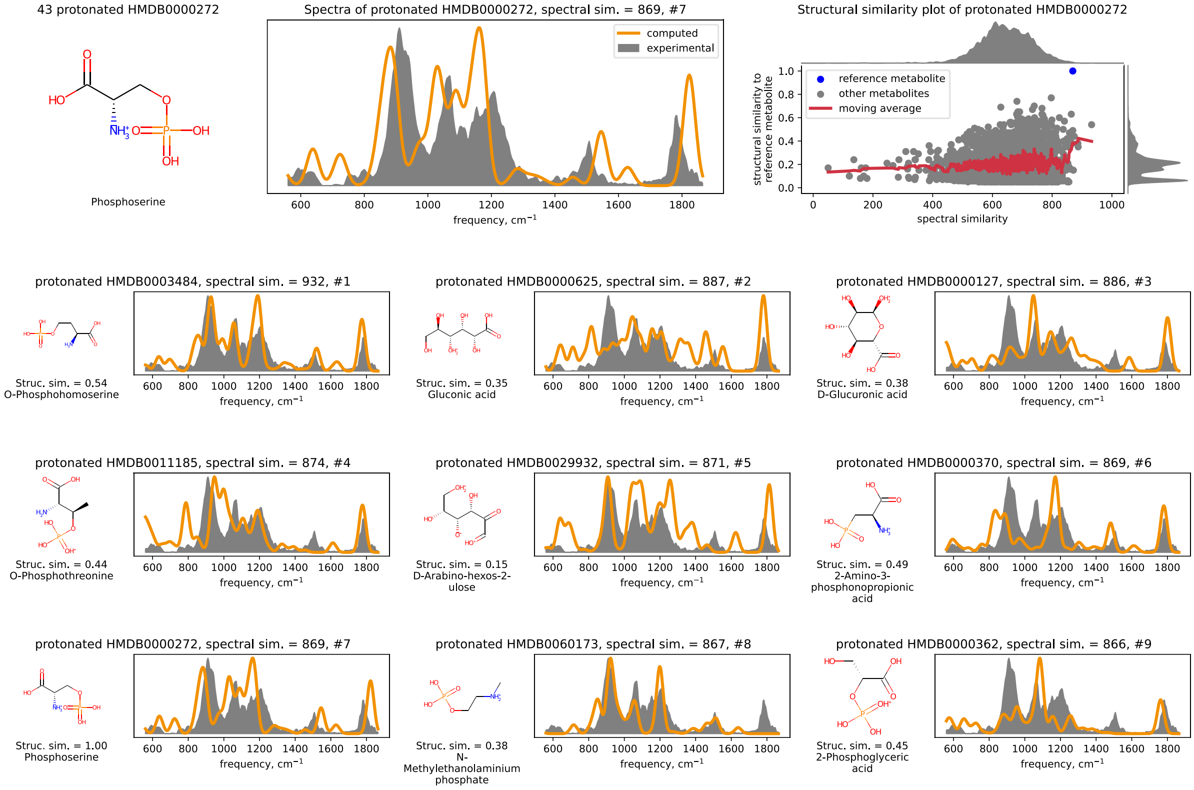
43 protonated HMDB0000272
Phosphoserine
Spectra of protonated HMDB0000272, spectral sim. = 869, #7
computed
experimental
frequency, cm^{-1}
Structural similarity plot of protonated HMDB0000272
reference metabolite
other metabolites
moving average
structural similarity to reference metabolite
spectral similarity
protonated HMDB0003484, spectral sim. = 932, #1
Struc. sim. = 0.54
O-Phosphohomoserine
protonated HMDB0000625, spectral sim. = 887, #2
Struc. sim. = 0.35
Gluconic acid
protonated HMDB0000127, spectral sim. = 886, #3
Struc. sim. = 0.38
D-Glucuronic acid
protonated HMDB0011185, spectral sim. = 874, #4
Struc. sim. = 0.44
O-Phosphothreonine
protonated HMDB0029932, spectral sim. = 871, #5
Struc. sim. = 0.15
D-Arabino-hexos-2-ulose
protonated HMDB0000370, spectral sim. = 869, #6
Struc. sim. = 0.49
2-Amino-3-phosphonopropionic acid
protonated HMDB0000272, spectral sim. = 869, #7
Struc. sim. = 1.00
Phosphoserine
protonated HMDB0060173, spectral sim. = 867, #8
Struc. sim. = 0.38
N-Methylethanolaminium phosphate
protonated HMDB0000362, spectral sim. = 866, #9
Struc. sim. = 0.45
2-Phosphoglyceric acid

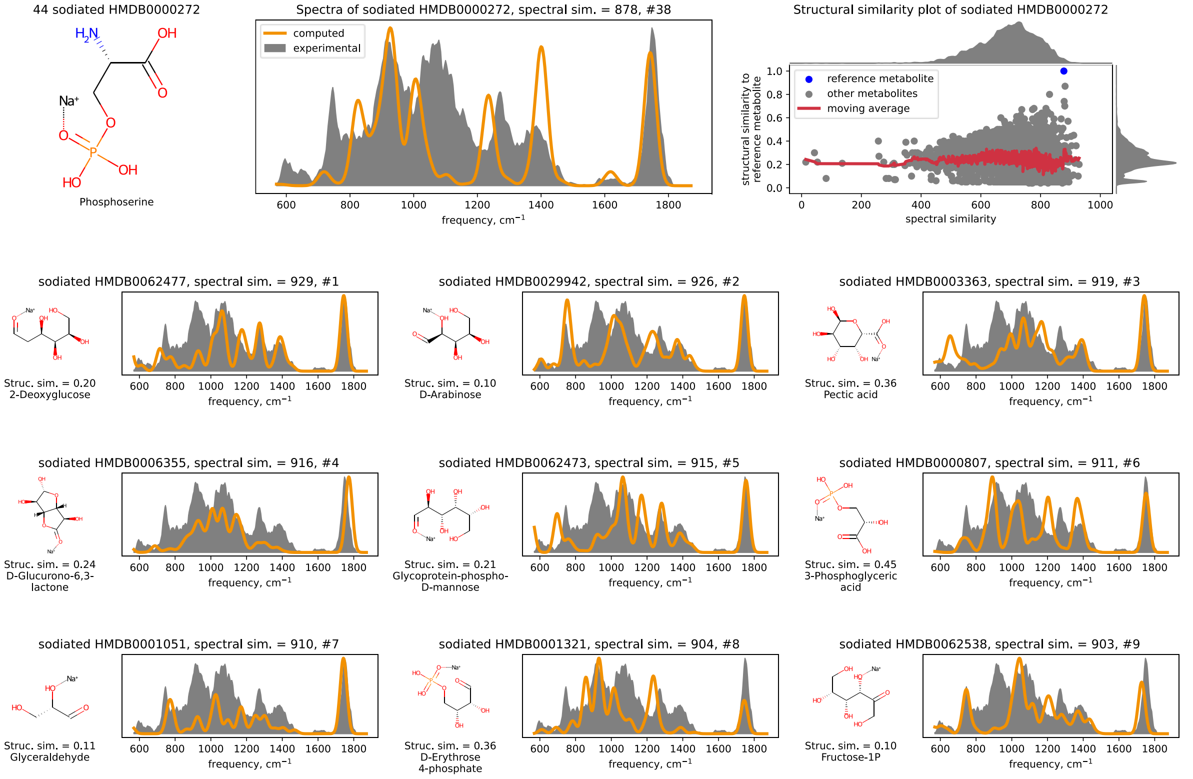
44 sodiated HMDB0000272
Phosphoserine
Spectra of sodiated HMDB0000272, spectral sim. = 878, #38
computed
experimental
frequency, cm^{-1}
Structural similarity plot of sodiated HMDB0000272
reference metabolite
other metabolites
moving average
structural similarity to reference metabolite
spectral similarity
sodiated HMDB0062477, spectral sim. = 929, #1
Struc. sim. = 0.20
2-Deoxyglucose
sodiated HMDB0029942, spectral sim. = 926, #2
Struc. sim. = 0.10
D-Arabinose
sodiated HMDB0003363, spectral sim. = 919, #3
Struc. sim. = 0.36
Pectic acid
sodiated HMDB0006355, spectral sim. = 916, #4
Struc. sim. = 0.24
D-Glucurono-6,3-lactone
sodiated HMDB0062473, spectral sim. = 915, #5
Struc. sim. = 0.21
Glycoprotein-phospho-D-mannose
sodiated HMDB0000807, spectral sim. = 911, #6
Struc. sim. = 0.45
3-Phosphoglyceric acid
sodiated HMDB0001051, spectral sim. = 910, #7
Struc. sim. = 0.11
Glyceraldehyde
sodiated HMDB0001321, spectral sim. = 904, #8
Struc. sim. = 0.36
D-Erythrose 4-phosphate
sodiated HMDB0062538, spectral sim. = 903, #9
Struc. sim. = 0.10
Fructose-1P

45 deprotonated HMDB0000292

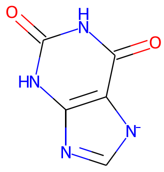

Xanthine

Spectra of deprotonated HMDB0000292, spectral sim. = 782, #18

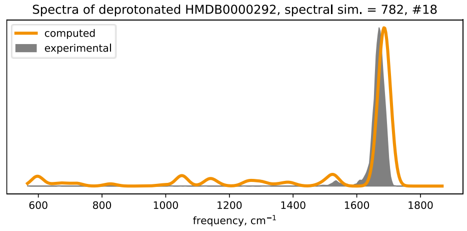

Structural similarity plot of deprotonated HMDB0000292

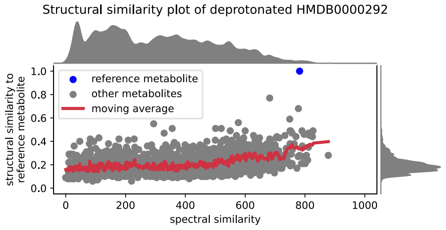

deprotonated HMDB0040261, spectral sim. = 878, #1

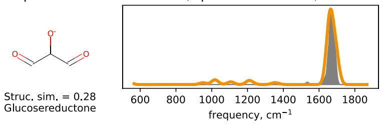

deprotonated HMDB0059704, spectral sim. = 827, #2

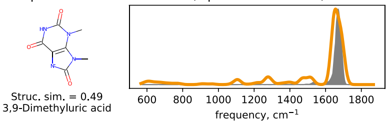

deprotonated HMDB0001970, spectral sim. = 825, #3

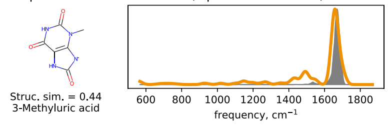

deprotonated HMDB0001886, spectral sim. = 816, #4

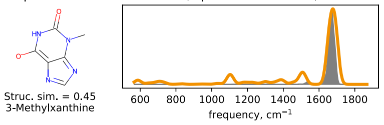

deprotonated HMDB0003099, spectral sim. = 812, #5

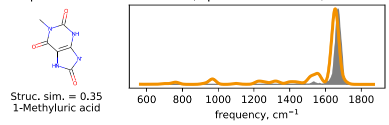

deprotonated HMDB0000786, spectral sim. = 812, #6

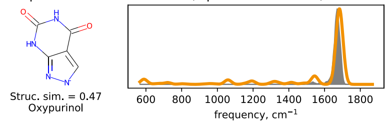

deprotonated HMDB0000157, spectral sim. = 809, #7

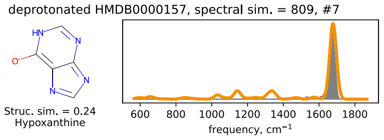

deprotonated HMDB0013141, spectral sim. = 807, #8

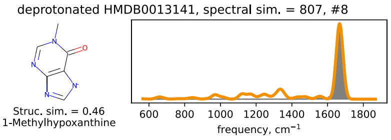

deprotonated HMDB0031223, spectral sim. = 805, #9

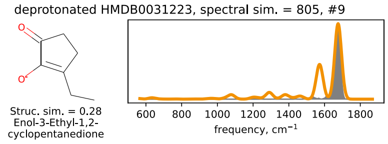

46 protonated HMDB0000292

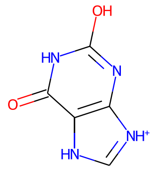

Xanthine

Spectra of protonated HMDB0000292, spectral sim. = 894, #2

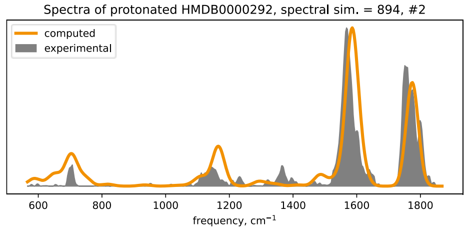

Structural similarity plot of protonated HMDB0000292

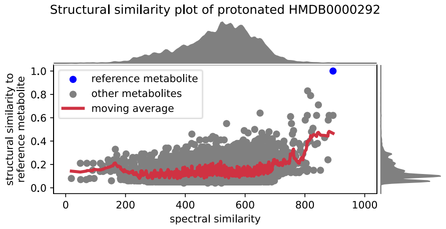

protonated HMDB0001991, spectral sim. = 894, #1

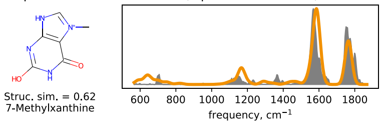

protonated HMDB0000292, spectral sim. = 894, #2

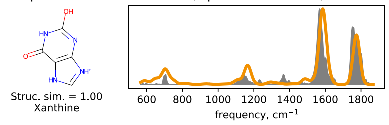

protonated HMDB0011107, spectral sim. = 881, #3

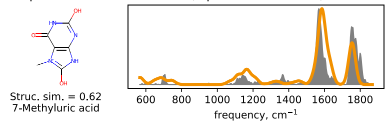

protonated HMDB0000157, spectral sim. = 878, #4

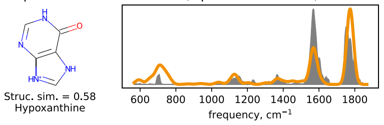

protonated HMDB0002144, spectral sim. = 877, #5

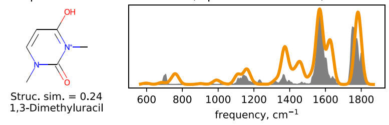

protonated HMDB0003162, spectral sim. = 866, #6

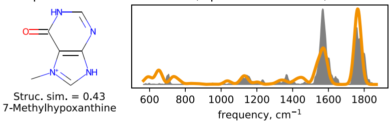

protonated HMDB0000539, spectral sim. = 852, #7

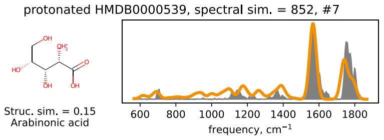

protonated HMDB0010738, spectral sim. = 845, #8

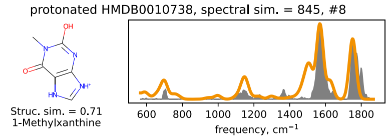

protonated HMDB0004308, spectral sim. = 842, #9

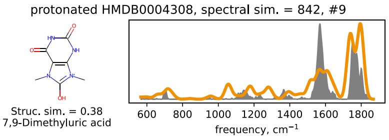

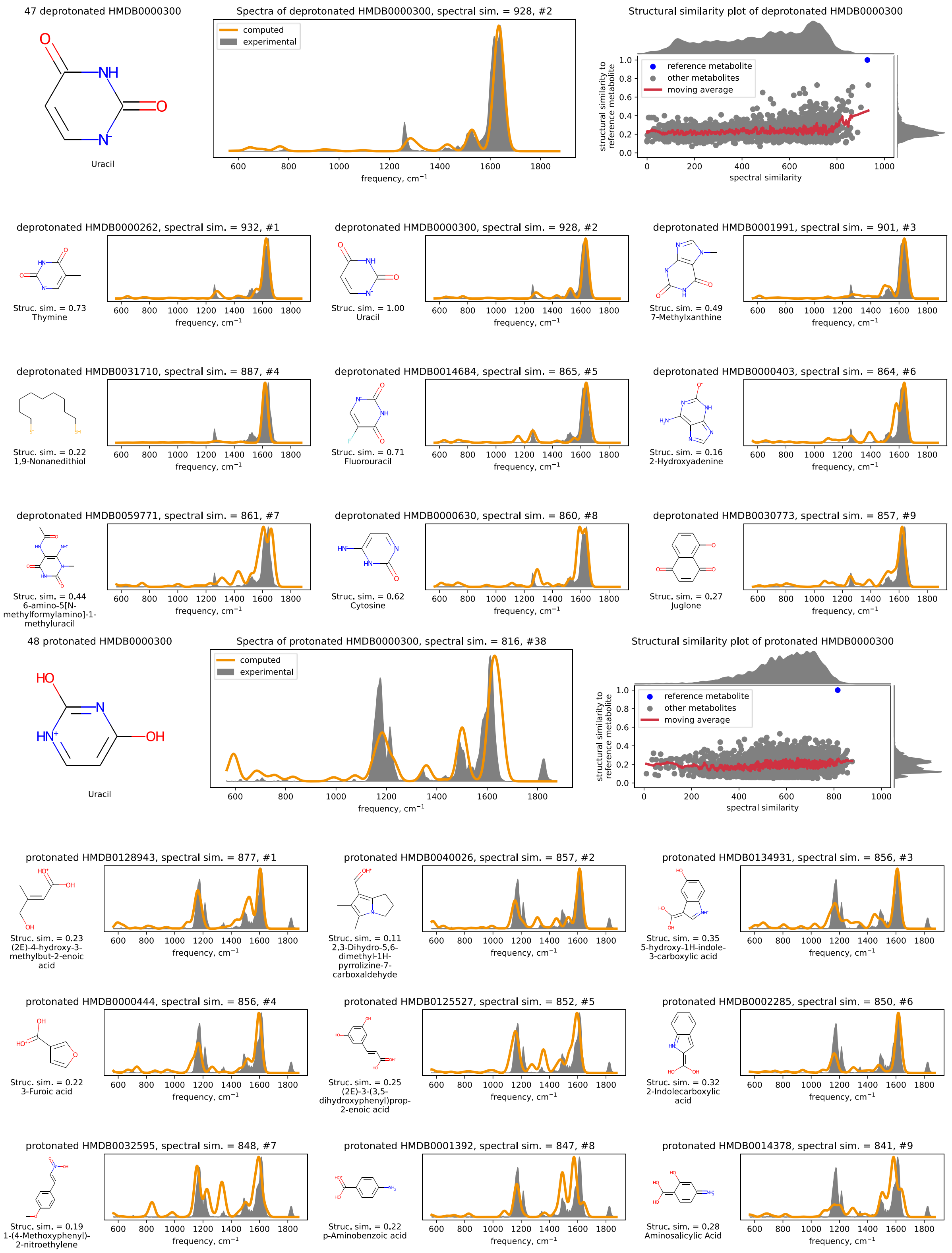
47 deprotonated HMDB0000300
Uracil
Spectra of deprotonated HMDB0000300, spectral sim. = 928, #2
computed
experimental
frequency, cm^{-1}
Structural similarity plot of deprotonated HMDB0000300
reference metabolite
other metabolites
moving average
structural similarity to reference metabolite
spectral similarity
deprotonated HMDB0000262, spectral sim. = 932, #1
Struc. sim. = 0.73
Thymine
deprotonated HMDB0000300, spectral sim. = 928, #2
Struc. sim. = 1.00
Uracil
deprotonated HMDB0001991, spectral sim. = 901, #3
Struc. sim. = 0.49
7-Methylxanthine
deprotonated HMDB0031710, spectral sim. = 887, #4
Struc. sim. = 0.22
1,9-Nonanedithiol
deprotonated HMDB0014684, spectral sim. = 865, #5
Struc. sim. = 0.71
Fluorouracil
deprotonated HMDB0000403, spectral sim. = 864, #6
Struc. sim. = 0.16
2-Hydroxyadenine
deprotonated HMDB0059771, spectral sim. = 861, #7
Struc. sim. = 0.44
6-amino-5[N-methylformylamino]-1-methyluracil
deprotonated HMDB0000630, spectral sim. = 860, #8
Struc. sim. = 0.62
Cytosine
deprotonated HMDB0030773, spectral sim. = 857, #9
Struc. sim. = 0.27
Juglone
48 protonated HMDB0000300
Uracil
Spectra of protonated HMDB0000300, spectral sim. = 816, #38
computed
experimental
frequency, cm^{-1}
Structural similarity plot of protonated HMDB0000300
reference metabolite
other metabolites
moving average
structural similarity to reference metabolite
spectral similarity
protonated HMDB0128943, spectral sim. = 877, #1
Struc. sim. = 0.23
(2E)-4-hydroxy-3-methylbut-2-enoic acid
protonated HMDB0040026, spectral sim. = 857, #2
Struc. sim. = 0.11
2,3-Dihydro-5,6-dimethyl-1H-pyrrolizine-7-carboxaldehyde
protonated HMDB0134931, spectral sim. = 856, #3
Struc. sim. = 0.35
5-hydroxy-1H-indole-3-carboxylic acid
protonated HMDB0000444, spectral sim. = 856, #4
Struc. sim. = 0.22
3-Furoic acid
protonated HMDB0125527, spectral sim. = 852, #5
Struc. sim. = 0.25
(2E)-3-(3,5-dihydroxyphenyl)prop-2-enoic acid
protonated HMDB0002285, spectral sim. = 850, #6
Struc. sim. = 0.32
2-Indolecarboxylic acid
protonated HMDB0032595, spectral sim. = 848, #7
Struc. sim. = 0.19
1-(4-Methoxyphenyl)-2-nitroethylene
protonated HMDB0001392, spectral sim. = 847, #8
Struc. sim. = 0.22
p-Aminobenzoic acid
protonated HMDB0014378, spectral sim. = 841, #9
Struc. sim. = 0.28
Aminosalicylic Acid

49 sodiated HMDB0000300

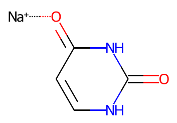

Uracil

Spectra of sodiated HMDB0000300, spectral sim. = 937, #1

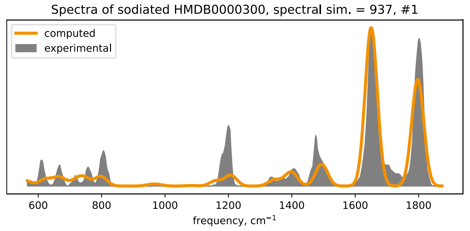

Structural similarity plot of sodiated HMDB0000300

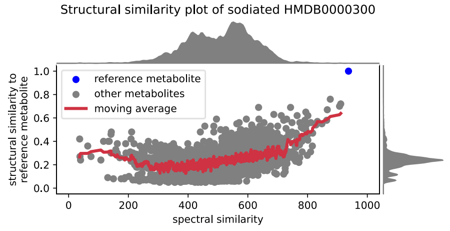

sodiated HMDB0000300, spectral sim. = 937, #1

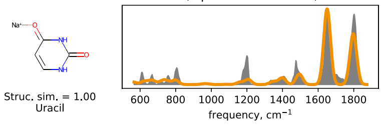

sodiated HMDB0014684, spectral sim. = 911, #2

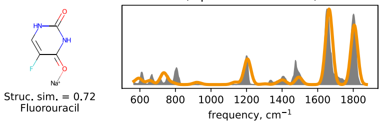

sodiated HMDB0000469, spectral sim. = 907, #3

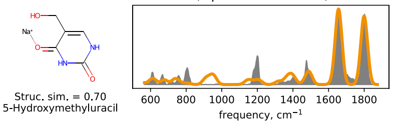

sodiated HMDB0000262, spectral sim. = 876, #4

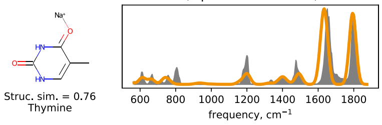

sodiated HMDB0000226, spectral sim. = 866, #5

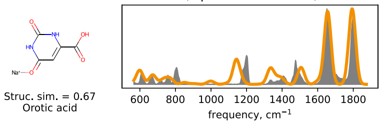

sodiated HMDB0000544, spectral sim. = 864, #6

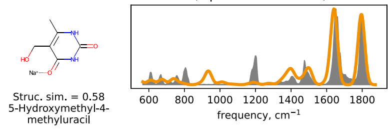

sodiated HMDB0000292, spectral sim. = 848, #7

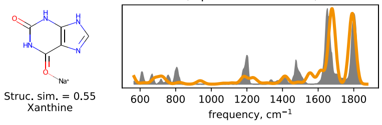

sodiated HMDB0000076, spectral sim. = 833, #8

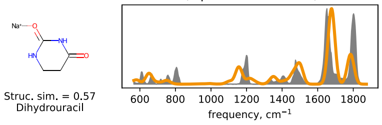

sodiated HMDB0015441, spectral sim. = 832, #9

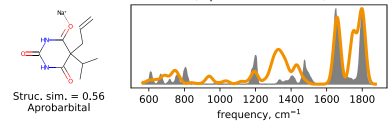

50 deprotonated HMDB0000301

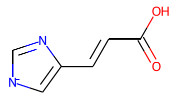

Urocanic acid

Spectra of deprotonated HMDB0000301, spectral sim. = 935, #3

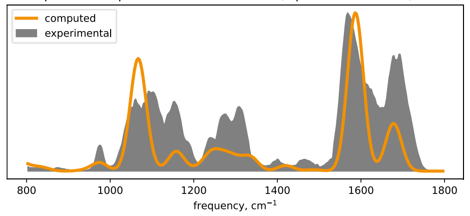

Structural similarity plot of deprotonated HMDB0000301

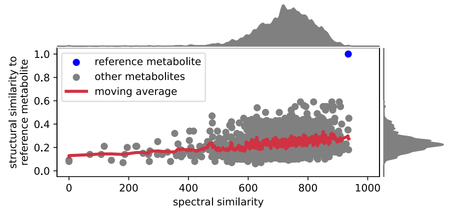

deprotonated HMDB0000522, spectral sim. = 937, #1

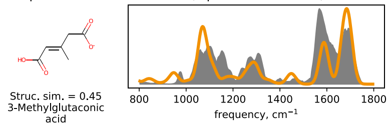

deprotonated HMDB0032951, spectral sim. = 936, #2

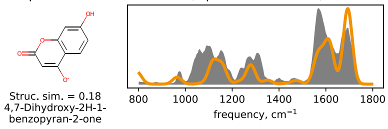

deprotonated HMDB0000301, spectral sim. = 935, #3

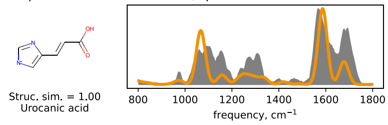

deprotonated HMDB0002016, spectral sim. = 934, #4

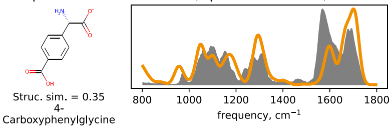

deprotonated HMDB0136661, spectral sim. = 928, #5

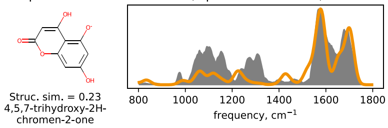

deprotonated HMDB0132254, spectral sim. = 918, #6

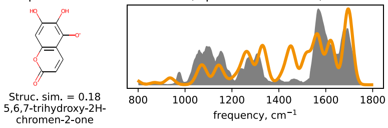

deprotonated HMDB0061933, spectral sim. = 916, #7

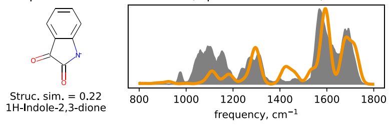

deprotonated HMDB0136660, spectral sim. = 913, #8

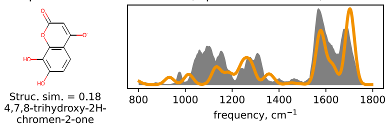

deprotonated HMDB0060664, spectral sim. = 913, #9

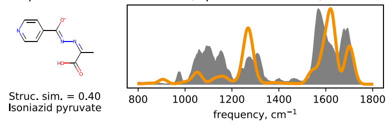

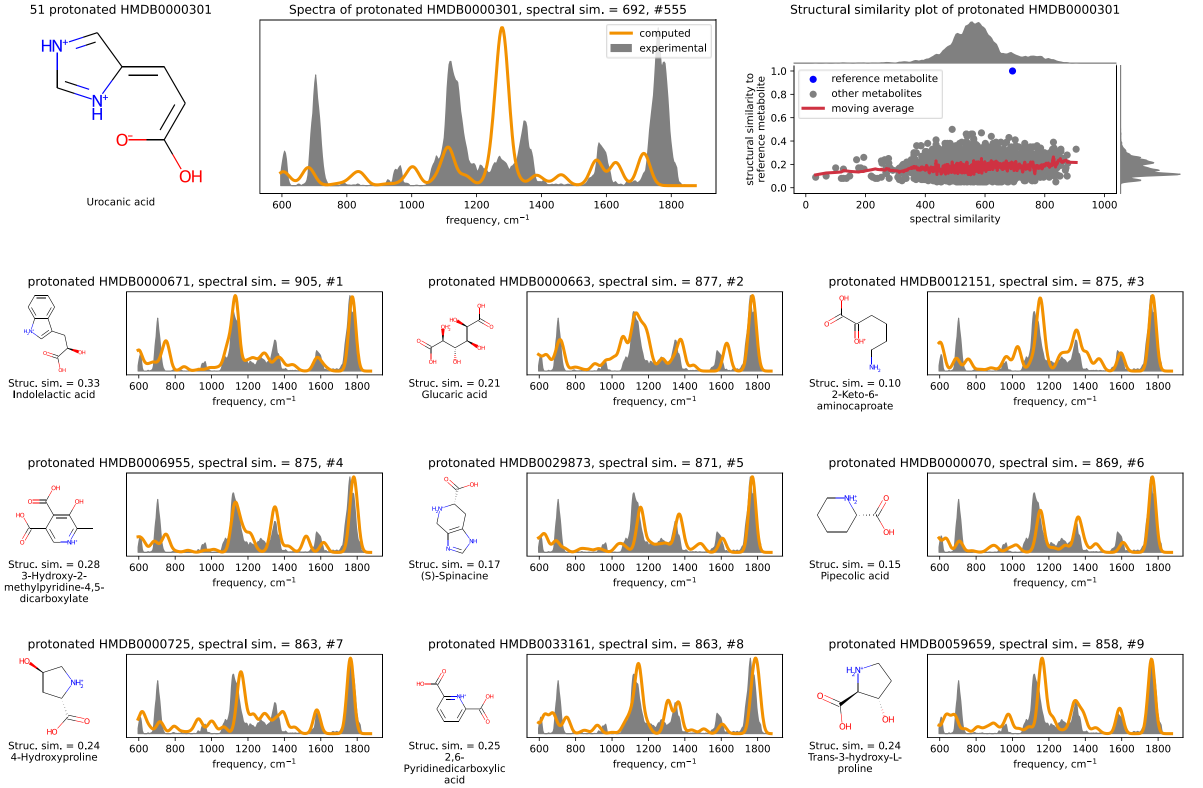
51 protonated HMDB0000301
Urocanic acid
Spectra of protonated HMDB0000301, spectral sim. = 692, #555
computed
experimental
frequency, cm^{-1}
Structural similarity plot of protonated HMDB0000301
reference metabolite
other metabolites
moving average
structural similarity to reference metabolite
spectral similarity
protonated HMDB0000671, spectral sim. = 905, #1
Struc. sim. = 0.33
Indolelactic acid
protonated HMDB0000663, spectral sim. = 877, #2
Struc. sim. = 0.21
Glucaric acid
protonated HMDB0012151, spectral sim. = 875, #3
Struc. sim. = 0.10
2-Keto-6-aminocaproate
protonated HMDB0006955, spectral sim. = 875, #4
Struc. sim. = 0.28
3-Hydroxy-2-methylpyridine-4,5-dicarboxylate
protonated HMDB0029873, spectral sim. = 871, #5
Struc. sim. = 0.17
(S)-Spinacine
protonated HMDB0000070, spectral sim. = 869, #6
Struc. sim. = 0.15
Pipecolic acid
protonated HMDB0000725, spectral sim. = 863, #7
Struc. sim. = 0.24
4-Hydroxyproline
protonated HMDB0033161, spectral sim. = 863, #8
Struc. sim. = 0.25
2,6-Pyridinedicarboxylic acid
protonated HMDB0059659, spectral sim. = 858, #9
Struc. sim. = 0.24
Trans-3-hydroxy-L-proline

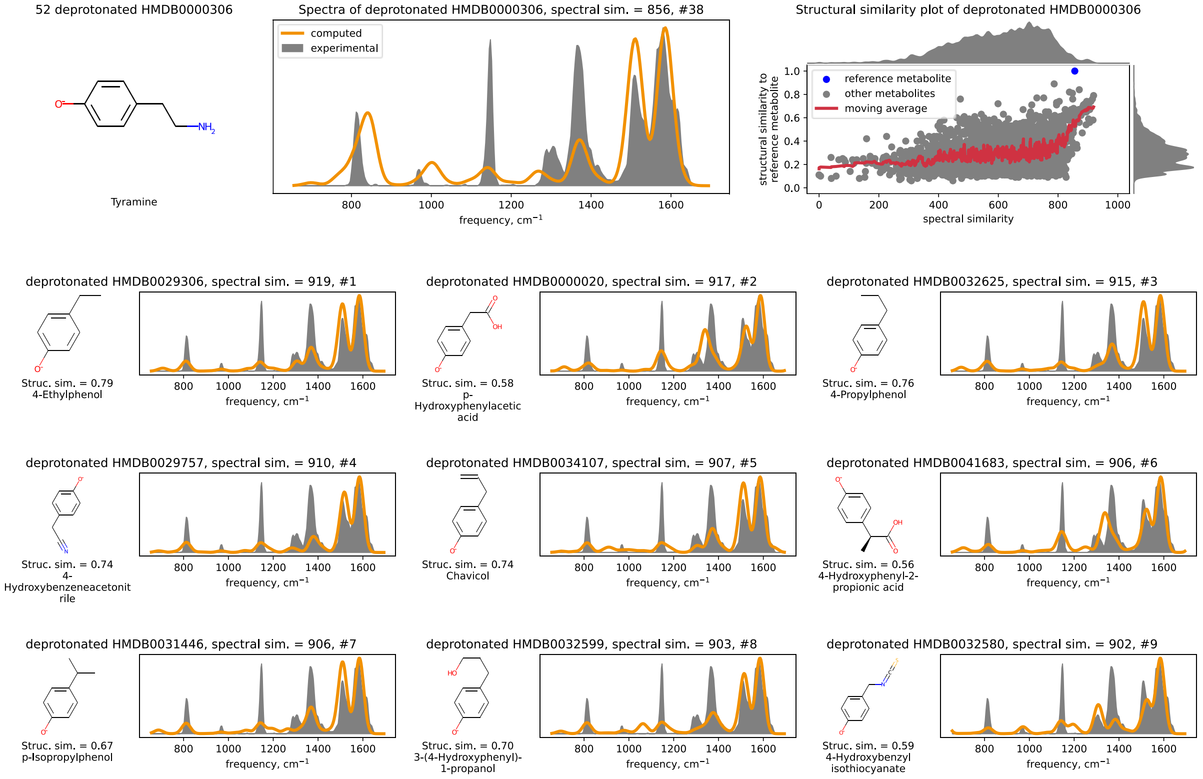
52 deprotonated HMDB0000306
Tyramine
Spectra of deprotonated HMDB0000306, spectral sim. = 856, #38
computed
experimental
frequency, cm^{-1}
Structural similarity plot of deprotonated HMDB0000306
reference metabolite
other metabolites
moving average
structural similarity to reference metabolite
spectral similarity
deprotonated HMDB0029306, spectral sim. = 919, #1
Struc. sim. = 0.79
4-Ethylphenol
deprotonated HMDB0000020, spectral sim. = 917, #2
Struc. sim. = 0.58
p-Hydroxyphenylacetic acid
deprotonated HMDB0032625, spectral sim. = 915, #3
Struc. sim. = 0.76
4-Propylphenol
deprotonated HMDB0029757, spectral sim. = 910, #4
Struc. sim. = 0.74
4-Hydroxybenzeneacetonitrile
deprotonated HMDB0034107, spectral sim. = 907, #5
Struc. sim. = 0.74
Chavicol
deprotonated HMDB0041683, spectral sim. = 906, #6
Struc. sim. = 0.56
4-Hydroxyphenyl-2-propionic acid
deprotonated HMDB0031446, spectral sim. = 906, #7
Struc. sim. = 0.67
p-Isopropylphenol
deprotonated HMDB0032599, spectral sim. = 903, #8
Struc. sim. = 0.70
3-(4-Hydroxyphenyl)-1-propanol
deprotonated HMDB0032580, spectral sim. = 902, #9
Struc. sim. = 0.59
4-Hydroxybenzyl isothiocyanate

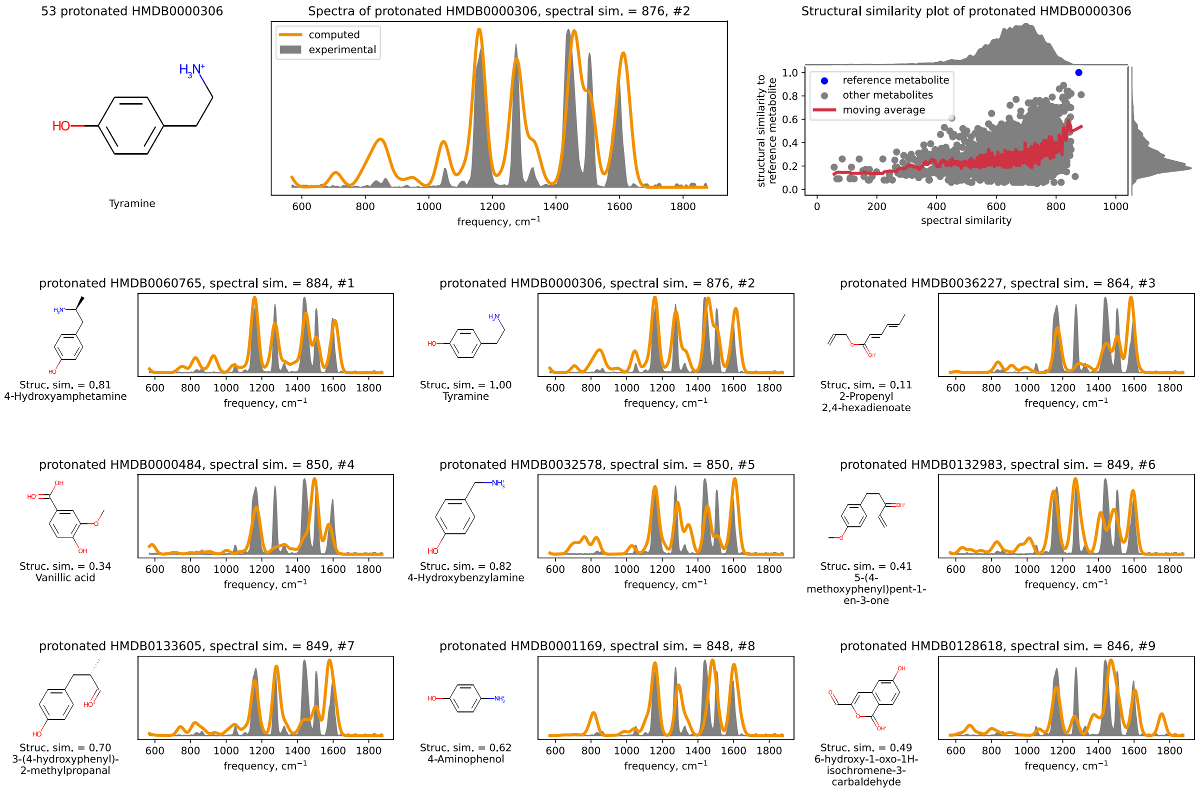
53 protonated HMDB0000306
Tyramine
Spectra of protonated HMDB0000306, spectral sim. = 876, #2
computed
experimental
frequency, cm⁻¹
Structural similarity plot of protonated HMDB0000306
reference metabolite
other metabolites
moving average
structural similarity to reference metabolite
spectral similarity
protonated HMDB0060765, spectral sim. = 884, #1
Struc. sim. = 0.81
4-Hydroxyamphetamine
protonated HMDB0000306, spectral sim. = 876, #2
Struc. sim. = 1.00
Tyramine
protonated HMDB0036227, spectral sim. = 864, #3
Struc. sim. = 0.11
2-Propenyl 2,4-hexadienoate
protonated HMDB0000484, spectral sim. = 850, #4
Struc. sim. = 0.34
Vanillic acid
protonated HMDB0032578, spectral sim. = 850, #5
Struc. sim. = 0.82
4-Hydroxybenzylamine
protonated HMDB0132983, spectral sim. = 849, #6
Struc. sim. = 0.41
5-(4-methoxyphenyl)pent-1-en-3-one
protonated HMDB0133605, spectral sim. = 849, #7
Struc. sim. = 0.70
3-(4-hydroxyphenyl)-2-methylpropanal
protonated HMDB0001169, spectral sim. = 848, #8
Struc. sim. = 0.62
4-Aminophenol
protonated HMDB0128618, spectral sim. = 846, #9
Struc. sim. = 0.49
6-hydroxy-1-oxo-1H-isochromene-3-carbaldehyde
frequency, cm⁻¹

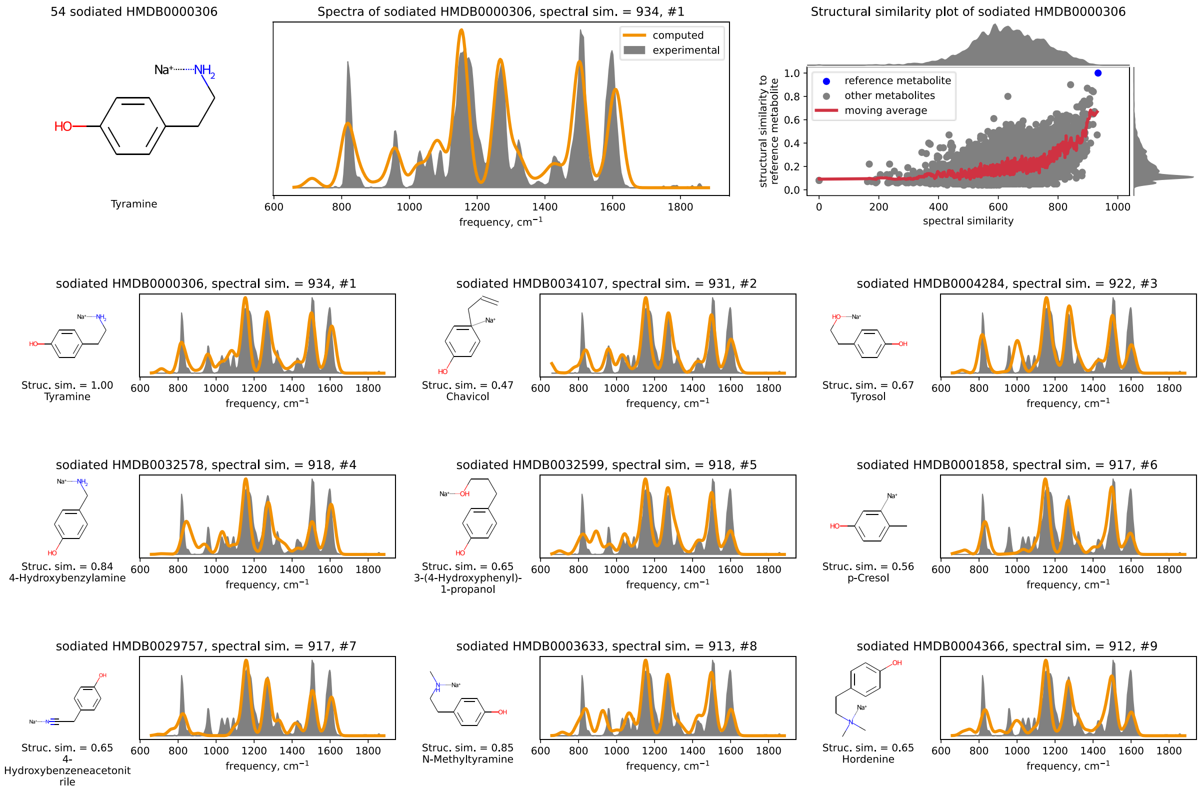
54 sodiated HMDB0000306
Tyramine
Spectra of sodiated HMDB0000306, spectral sim. = 934, #1
computed
experimental
frequency, cm⁻¹
Structural similarity plot of sodiated HMDB0000306
reference metabolite
other metabolites
moving average
structural similarity to reference metabolite
spectral similarity
sodiated HMDB0000306, spectral sim. = 934, #1
Struc. sim. = 1.00
Tyramine
sodiated HMDB0034107, spectral sim. = 931, #2
Struc. sim. = 0.47
Chavicol
sodiated HMDB0004284, spectral sim. = 922, #3
Struc. sim. = 0.67
Tyrosol
sodiated HMDB0032578, spectral sim. = 918, #4
Struc. sim. = 0.84
4-Hydroxybenzylamine
sodiated HMDB0032599, spectral sim. = 918, #5
Struc. sim. = 0.65
3-(4-Hydroxyphenyl)-1-propanol
sodiated HMDB0001858, spectral sim. = 917, #6
Struc. sim. = 0.56
p-Cresol
sodiated HMDB0029757, spectral sim. = 917, #7
Struc. sim. = 0.65
4-Hydroxybenzeneacetonitrile
sodiated HMDB0003633, spectral sim. = 913, #8
Struc. sim. = 0.85
N-Methyltyramine
sodiated HMDB0004366, spectral sim. = 912, #9
Struc. sim. = 0.65
Hordenine
frequency, cm⁻¹

55 protonated HMDB0000323

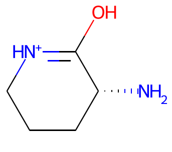

3-Amino-2-piperidone

Spectra of protonated HMDB0000323, spectral sim. = 688, #967

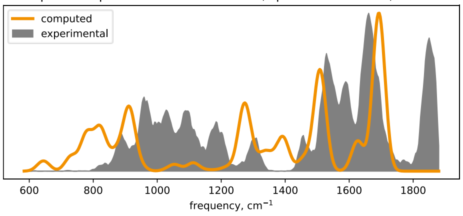

Structural similarity plot of protonated HMDB0000323

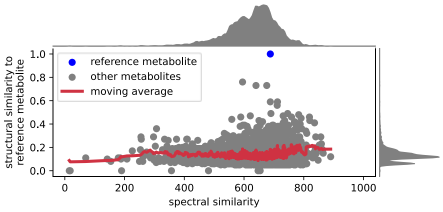

protonated HMDB0000562, spectral sim. = 890, #1

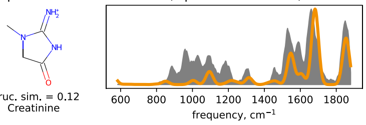

Struc. sim. = 0.12
Creatinine

protonated HMDB0003099, spectral sim. = 865, #2

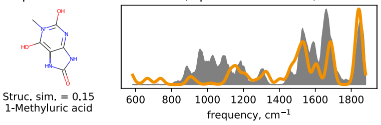

Struc. sim. = 0.15
1-Methyluric acid

protonated HMDB0014491, spectral sim. = 863, #3

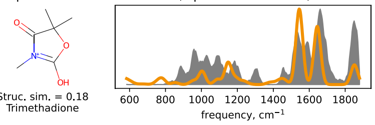

Struc. sim. = 0.18
Trimethadione

protonated HMDB0061082, spectral sim. = 859, #4

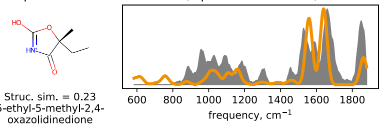

Struc. sim. = 0.23
5-ethyl-5-methyl-2,4-oxazolidinedione

protonated HMDB0060533, spectral sim. = 857, #5

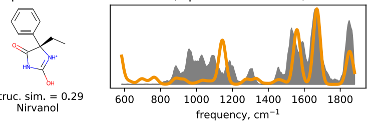

Struc. sim. = 0.29
Nirvanol

protonated HMDB0060967, spectral sim. = 857, #6

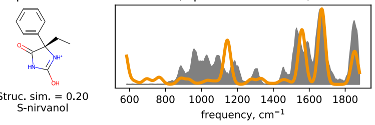

Struc. sim. = 0.20
S-nirvanol

protonated HMDB0014755, spectral sim. = 852, #7

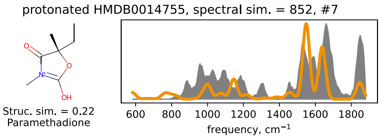

Struc. sim. = 0.22
Paramethadione

protonated HMDB0034912, spectral sim. = 848, #8

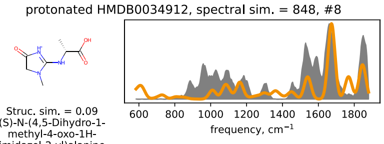

Struc. sim. = 0.09
(S)-N-(4,5-Dihydro-1-methyl-4-oxo-1H-imidazol-2-yl)alanine

protonated HMDB0003646, spectral sim. = 844, #9

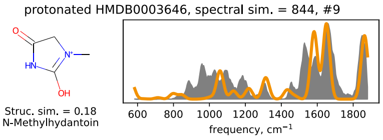

Struc. sim. = 0.18
N-Methylhydantoin

56 deprotonated HMDB0000434

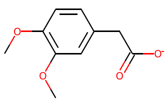

Homoveratric acid

Spectra of deprotonated HMDB0000434, spectral sim. = 970, #1

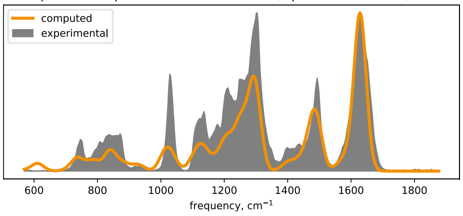

Structural similarity plot of deprotonated HMDB0000434

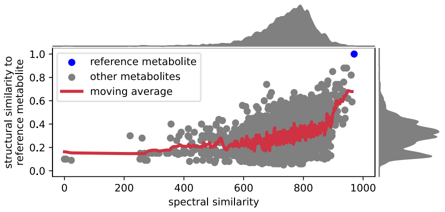

deprotonated HMDB0000434, spectral sim. = 970, #1

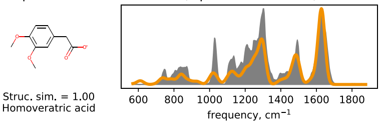

Struc. sim. = 1.00
Homoveratric acid

deprotonated HMDB0130385, spectral sim. = 963, #2

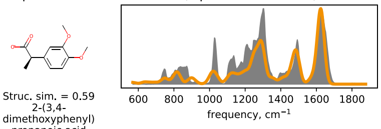

Struc. sim. = 0.59
2-(3,4-dimethoxyphenyl)propanoic acid

deprotonated HMDB0000118, spectral sim. = 960, #3

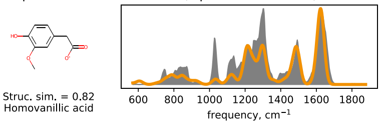

Struc. sim. = 0.82
Homovanillic acid

deprotonated HMDB0130895, spectral sim. = 949, #4

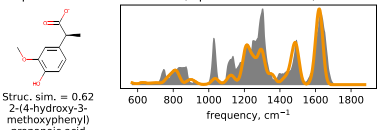

Struc. sim. = 0.62
2-(4-hydroxy-3-methoxyphenyl)propanoic acid

deprotonated HMDB0002072, spectral sim. = 945, #5

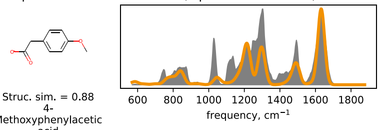

Struc. sim. = 0.88
4-Methoxyphenylacetic acid

deprotonated HMDB0132975, spectral sim. = 940, #6

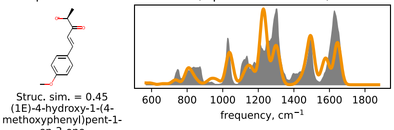

Struc. sim. = 0.45
(1E)-4-hydroxy-1-(4-methoxyphenyl)pent-1-en-3-one

deprotonated HMDB0129958, spectral sim. = 940, #7

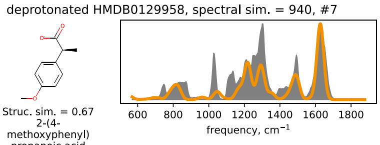

Struc. sim. = 0.67
2-(4-methoxyphenyl)propanoic acid

deprotonated HMDB0060374, spectral sim. = 939, #8

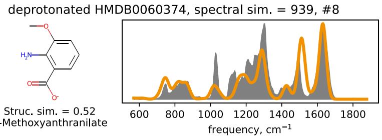

Struc. sim. = 0.52
3-Methoxyanthranilate

deprotonated HMDB0059969, spectral sim. = 936, #9

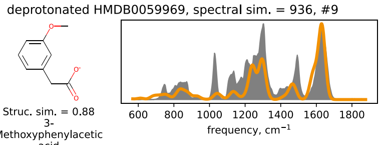

Struc. sim. = 0.88
3-Methoxyphenylacetic acid

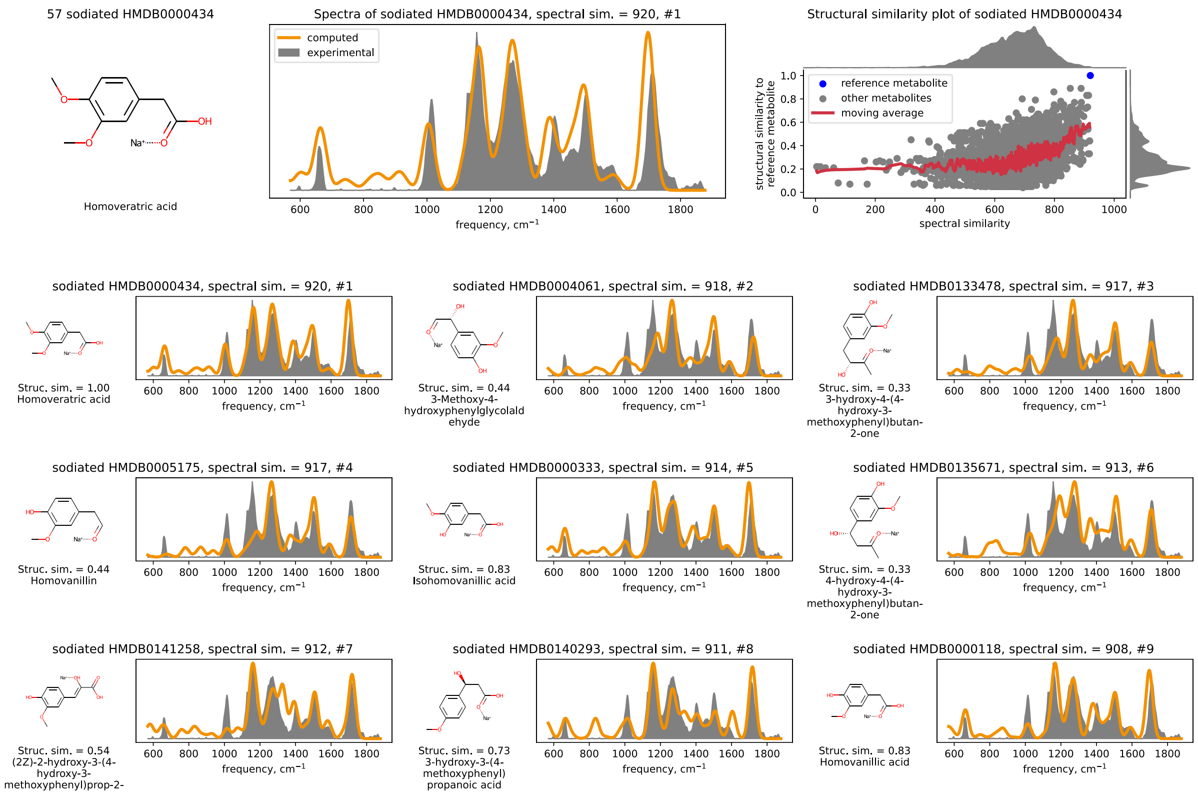

57 sodiated HMDB0000434
Homoveratric acid
Spectra of sodiated HMDB0000434, spectral sim. = 920, #1
computed
experimental
frequency, cm^{-1}
Structural similarity plot of sodiated HMDB0000434
reference metabolite
other metabolites
moving average
structural similarity to reference metabolite
spectral similarity
sodiated HMDB0000434, spectral sim. = 920, #1
Struc. sim. = 1.00
Homoveratric acid
sodiated HMDB0004061, spectral sim. = 918, #2
Struc. sim. = 0.44
3-Methoxy-4-hydroxyphenylglycolaldehyde
sodiated HMDB0133478, spectral sim. = 917, #3
Struc. sim. = 0.33
3-hydroxy-4-(4-hydroxy-3-methoxyphenyl)butan-2-one
sodiated HMDB0005175, spectral sim. = 917, #4
Struc. sim. = 0.44
Homovanillin
sodiated HMDB0000333, spectral sim. = 914, #5
Struc. sim. = 0.83
Isohomovanillic acid
sodiated HMDB0135671, spectral sim. = 913, #6
Struc. sim. = 0.33
4-hydroxy-4-(4-hydroxy-3-methoxyphenyl)butan-2-one
sodiated HMDB0141258, spectral sim. = 912, #7
Struc. sim. = 0.54
(2Z)-2-hydroxy-3-(4-hydroxy-3-methoxyphenyl)prop-2-
sodiated HMDB0140293, spectral sim. = 911, #8
Struc. sim. = 0.73
3-hydroxy-3-(4-methoxyphenyl)propanoic acid
sodiated HMDB0000118, spectral sim. = 908, #9
Struc. sim. = 0.83
Homovanillic acid

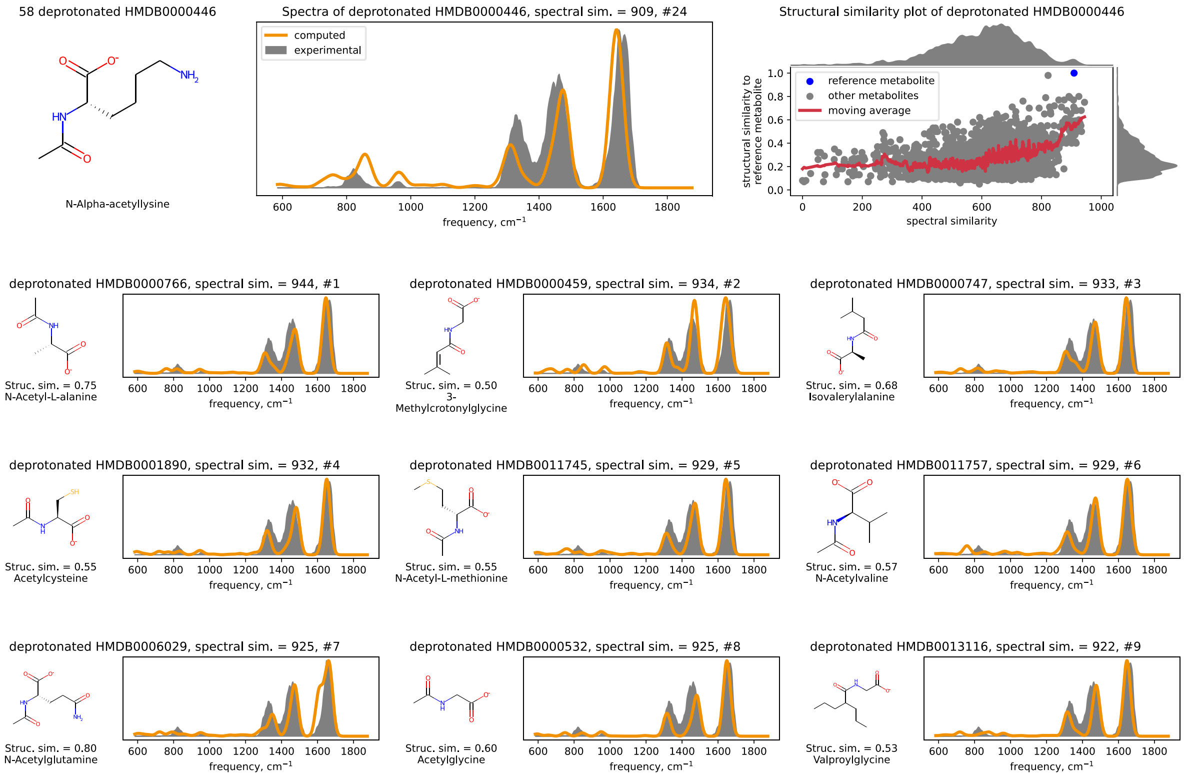

58 deprotonated HMDB0000446
N-Alpha-acetyllysine
Spectra of deprotonated HMDB0000446, spectral sim. = 909, #24
computed
experimental
frequency, cm^{-1}
Structural similarity plot of deprotonated HMDB0000446
reference metabolite
other metabolites
moving average
structural similarity to reference metabolite
spectral similarity
deprotonated HMDB0000766, spectral sim. = 944, #1
Struc. sim. = 0.75
N-Acetyl-L-alanine
deprotonated HMDB0000459, spectral sim. = 934, #2
Struc. sim. = 0.50
3-Methylcrotonylglycine
deprotonated HMDB0000747, spectral sim. = 933, #3
Struc. sim. = 0.68
Isovalerylalanine
deprotonated HMDB0001890, spectral sim. = 932, #4
Struc. sim. = 0.55
Acetylcysteine
deprotonated HMDB0011745, spectral sim. = 929, #5
Struc. sim. = 0.55
N-Acetyl-L-methionine
deprotonated HMDB0011757, spectral sim. = 929, #6
Struc. sim. = 0.57
N-Acetylvaline
deprotonated HMDB0006029, spectral sim. = 925, #7
Struc. sim. = 0.80
N-Acetylglutamine
deprotonated HMDB0000532, spectral sim. = 925, #8
Struc. sim. = 0.60
Acetylglycine
deprotonated HMDB0013116, spectral sim. = 922, #9
Struc. sim. = 0.53
Valproylglycine

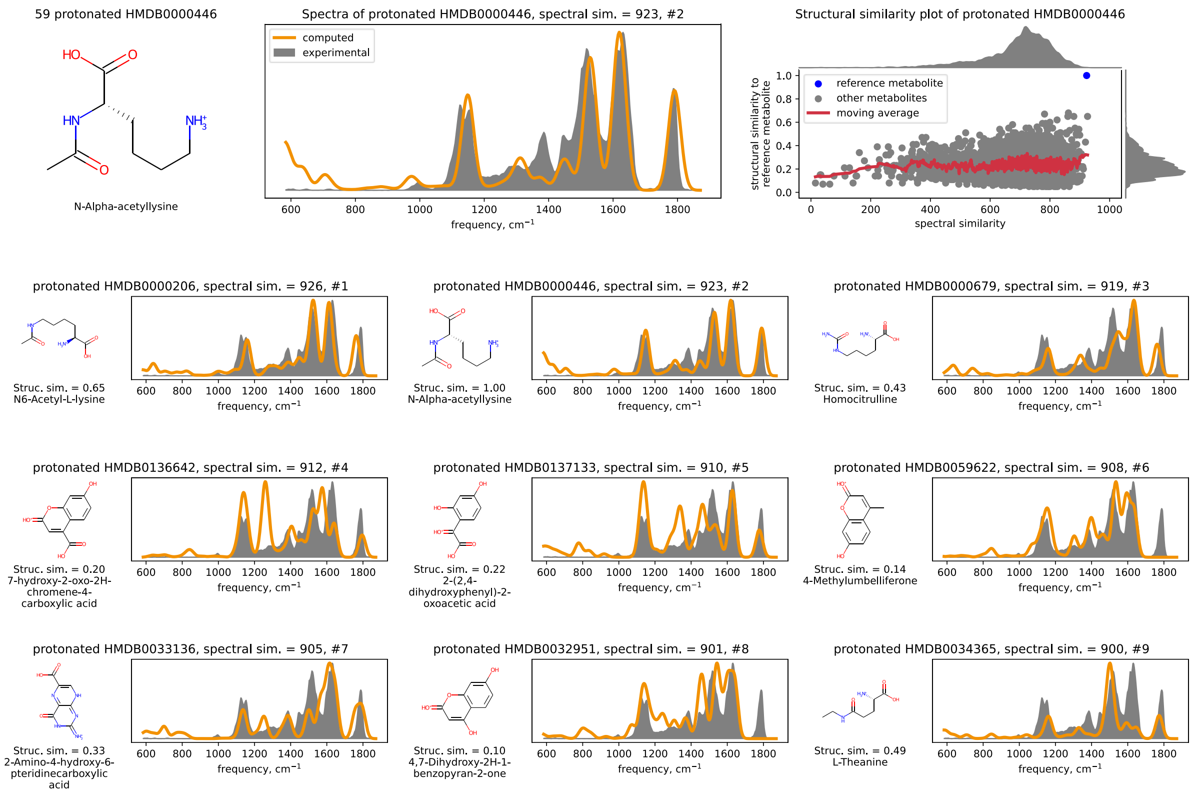
59 protonated HMDB0000446
N-Alpha-acetyllysine
Spectra of protonated HMDB0000446, spectral sim. = 923, #2
computed
experimental
frequency, cm^{-1}
Structural similarity plot of protonated HMDB0000446
reference metabolite
other metabolites
moving average
structural similarity to reference metabolite
spectral similarity
protonated HMDB0000206, spectral sim. = 926, #1
Struc. sim. = 0.65
N6-Acetyl-L-lysine
protonated HMDB0000446, spectral sim. = 923, #2
Struc. sim. = 1.00
N-Alpha-acetyllysine
protonated HMDB0000679, spectral sim. = 919, #3
Struc. sim. = 0.43
Homocitrulline
protonated HMDB0136642, spectral sim. = 912, #4
Struc. sim. = 0.20
7-hydroxy-2-oxo-2H-chromene-4-carboxylic acid
protonated HMDB0137133, spectral sim. = 910, #5
Struc. sim. = 0.22
2-(2,4-dihydroxyphenyl)-2-oxoacetic acid
protonated HMDB0059622, spectral sim. = 908, #6
Struc. sim. = 0.14
4-Methylumbelliferone
protonated HMDB0033136, spectral sim. = 905, #7
Struc. sim. = 0.33
2-Amino-4-hydroxy-6-pteridinecarboxylic acid
protonated HMDB0032951, spectral sim. = 901, #8
Struc. sim. = 0.10
4,7-Dihydroxy-2H-1-benzopyran-2-one
protonated HMDB0034365, spectral sim. = 900, #9
Struc. sim. = 0.49
L-Theanine

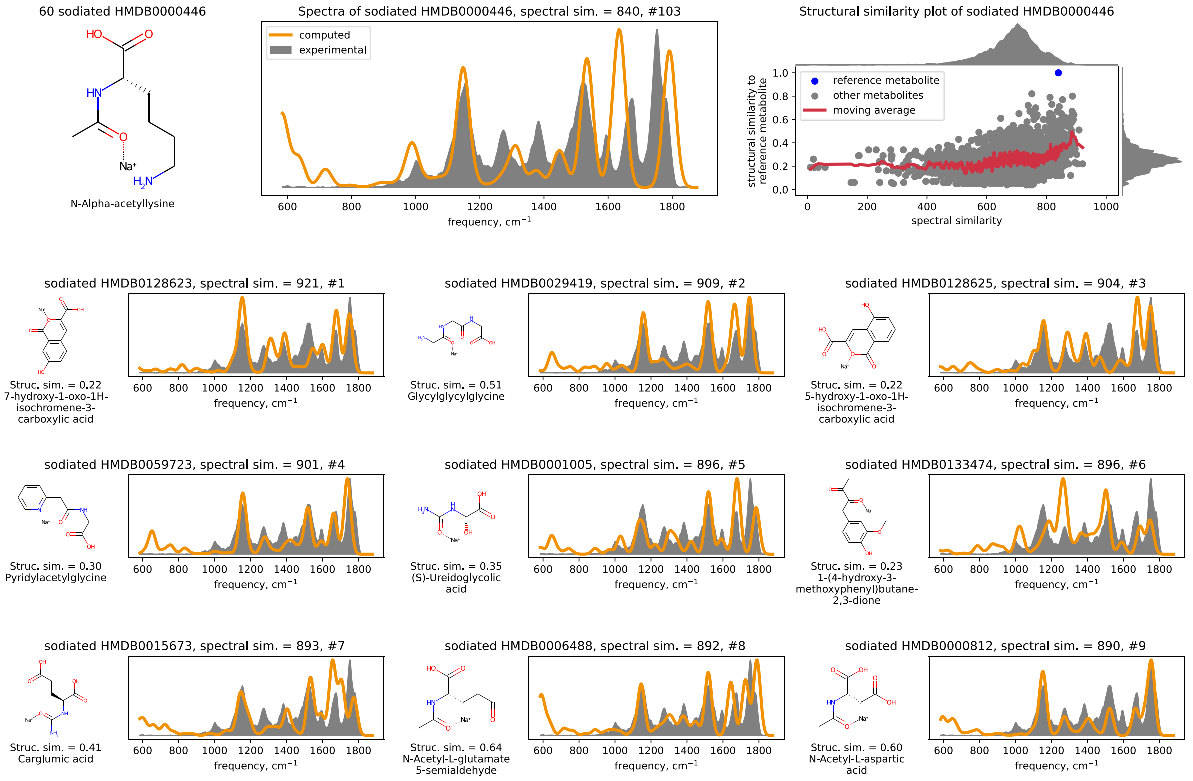
60 sodiated HMDB0000446
N-Alpha-acetyllysine
Spectra of sodiated HMDB0000446, spectral sim. = 840, #103
computed
experimental
frequency, cm^{-1}
Structural similarity plot of sodiated HMDB0000446
reference metabolite
other metabolites
moving average
structural similarity to reference metabolite
spectral similarity
sodiated HMDB0128623, spectral sim. = 921, #1
Struc. sim. = 0.22
7-hydroxy-1-oxo-1H-isochromene-3-carboxylic acid
sodiated HMDB0029419, spectral sim. = 909, #2
Struc. sim. = 0.51
Glycylglycylglycine
sodiated HMDB0128625, spectral sim. = 904, #3
Struc. sim. = 0.22
5-hydroxy-1-oxo-1H-isochromene-3-carboxylic acid
sodiated HMDB0059723, spectral sim. = 901, #4
Struc. sim. = 0.30
Pyridylacetylglycine
sodiated HMDB0001005, spectral sim. = 896, #5
Struc. sim. = 0.35
(S)-Ureidoglycolic acid
sodiated HMDB0133474, spectral sim. = 896, #6
Struc. sim. = 0.23
1-(4-hydroxy-3-methoxyphenyl)butane-2,3-dione
sodiated HMDB0015673, spectral sim. = 893, #7
Struc. sim. = 0.41
Carglumic acid
sodiated HMDB0006488, spectral sim. = 892, #8
Struc. sim. = 0.64
N-Acetyl-L-glutamate 5-semialdehyde
sodiated HMDB0000812, spectral sim. = 890, #9
Struc. sim. = 0.60
N-Acetyl-L-aspartic acid

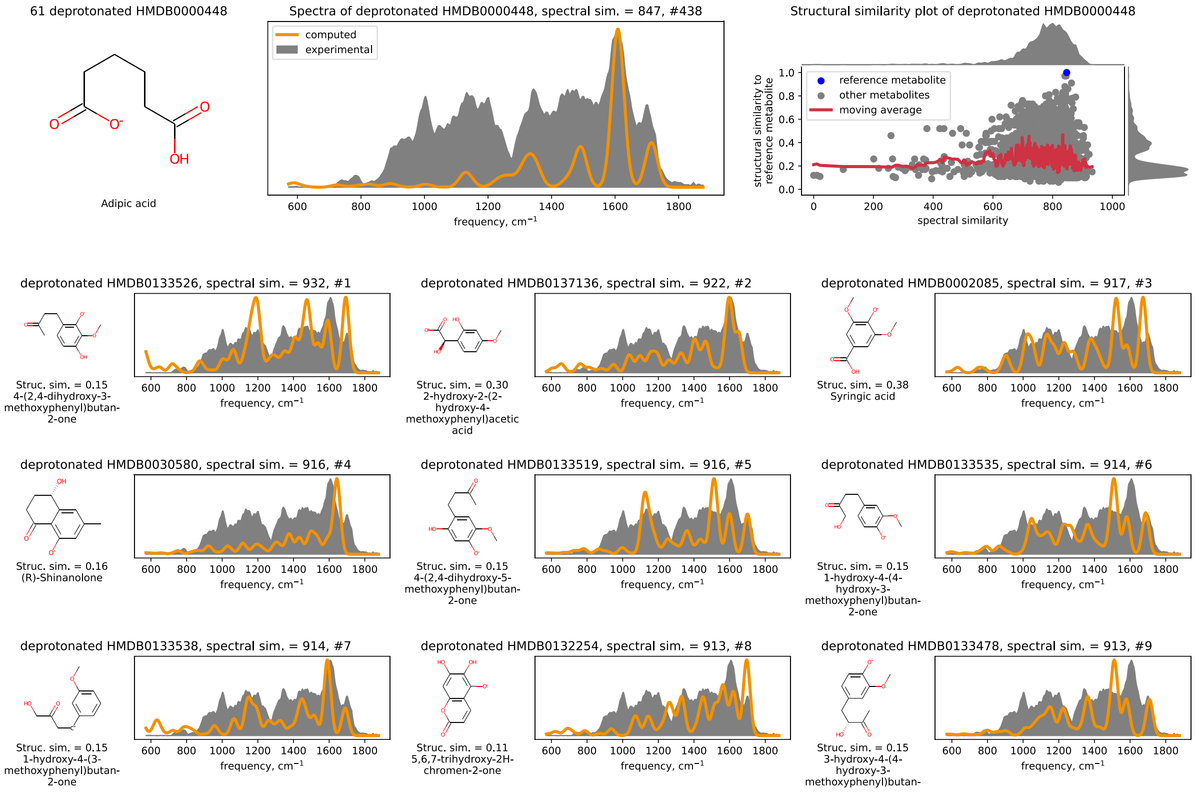
61 deprotonated HMDB0000448
Adipic acid
Spectra of deprotonated HMDB0000448, spectral sim. = 847, #438
computed
experimental
frequency, cm⁻¹
Structural similarity plot of deprotonated HMDB0000448
reference metabolite
other metabolites
moving average
structural similarity to reference metabolite
spectral similarity
deprotonated HMDB0133526, spectral sim. = 932, #1
Struc. sim. = 0.15
4-(2,4-dihydroxy-3-methoxyphenyl)butan-2-one
deprotonated HMDB0137136, spectral sim. = 922, #2
Struc. sim. = 0.30
2-hydroxy-2-(2-hydroxy-4-methoxyphenyl)acetic acid
deprotonated HMDB0002085, spectral sim. = 917, #3
Struc. sim. = 0.38
Syringic acid
deprotonated HMDB0030580, spectral sim. = 916, #4
Struc. sim. = 0.16
(R)-Shinanolone
deprotonated HMDB0133519, spectral sim. = 916, #5
Struc. sim. = 0.15
4-(2,4-dihydroxy-5-methoxyphenyl)butan-2-one
deprotonated HMDB0133535, spectral sim. = 914, #6
Struc. sim. = 0.15
1-hydroxy-4-(4-hydroxy-3-methoxyphenyl)butan-2-one
deprotonated HMDB0133538, spectral sim. = 914, #7
Struc. sim. = 0.15
1-hydroxy-4-(3-methoxyphenyl)butan-2-one
deprotonated HMDB0132254, spectral sim. = 913, #8
Struc. sim. = 0.11
5,6,7-trihydroxy-2H-chromen-2-one
deprotonated HMDB0133478, spectral sim. = 913, #9
Struc. sim. = 0.15
3-hydroxy-4-(4-hydroxy-3-methoxyphenyl)butan-

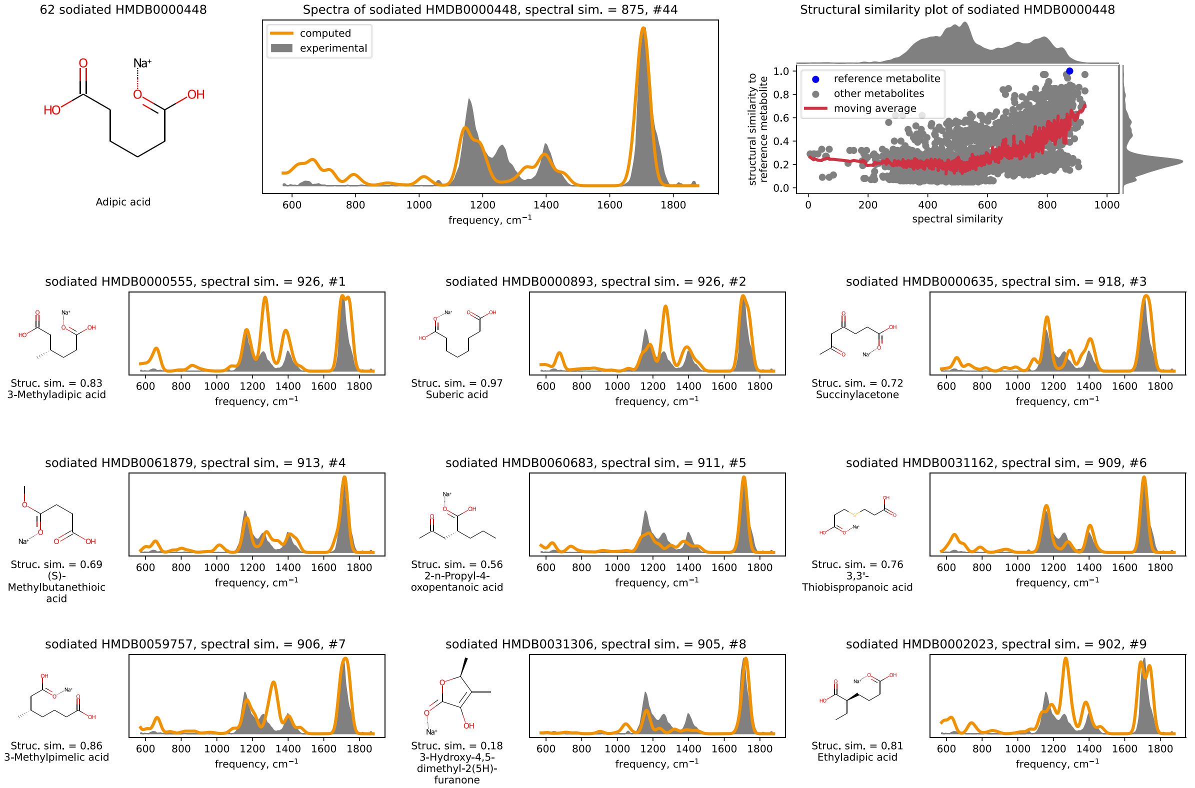
62 sodiated HMDB0000448
Adipic acid
Spectra of sodiated HMDB0000448, spectral sim. = 875, #44
computed
experimental
frequency, cm⁻¹
Structural similarity plot of sodiated HMDB0000448
reference metabolite
other metabolites
moving average
structural similarity to reference metabolite
spectral similarity
sodiated HMDB0000555, spectral sim. = 926, #1
Struc. sim. = 0.83
3-Methyladipic acid
sodiated HMDB0000893, spectral sim. = 926, #2
Struc. sim. = 0.97
Suberic acid
sodiated HMDB0000635, spectral sim. = 918, #3
Struc. sim. = 0.72
Succinylacetone
sodiated HMDB0061879, spectral sim. = 913, #4
Struc. sim. = 0.69
(S)-Methylbutanethioic acid
sodiated HMDB0060683, spectral sim. = 911, #5
Struc. sim. = 0.56
2-n-Propyl-4-oxopentanoic acid
sodiated HMDB0031162, spectral sim. = 909, #6
Struc. sim. = 0.76
3,3'-Thiobispropanoic acid
sodiated HMDB0059757, spectral sim. = 906, #7
Struc. sim. = 0.86
3-Methylpimelic acid
sodiated HMDB0031306, spectral sim. = 905, #8
Struc. sim. = 0.18
3-Hydroxy-4,5-dimethyl-2(5H)-furanone
sodiated HMDB0002023, spectral sim. = 902, #9
Struc. sim. = 0.81
Ethyladipic acid

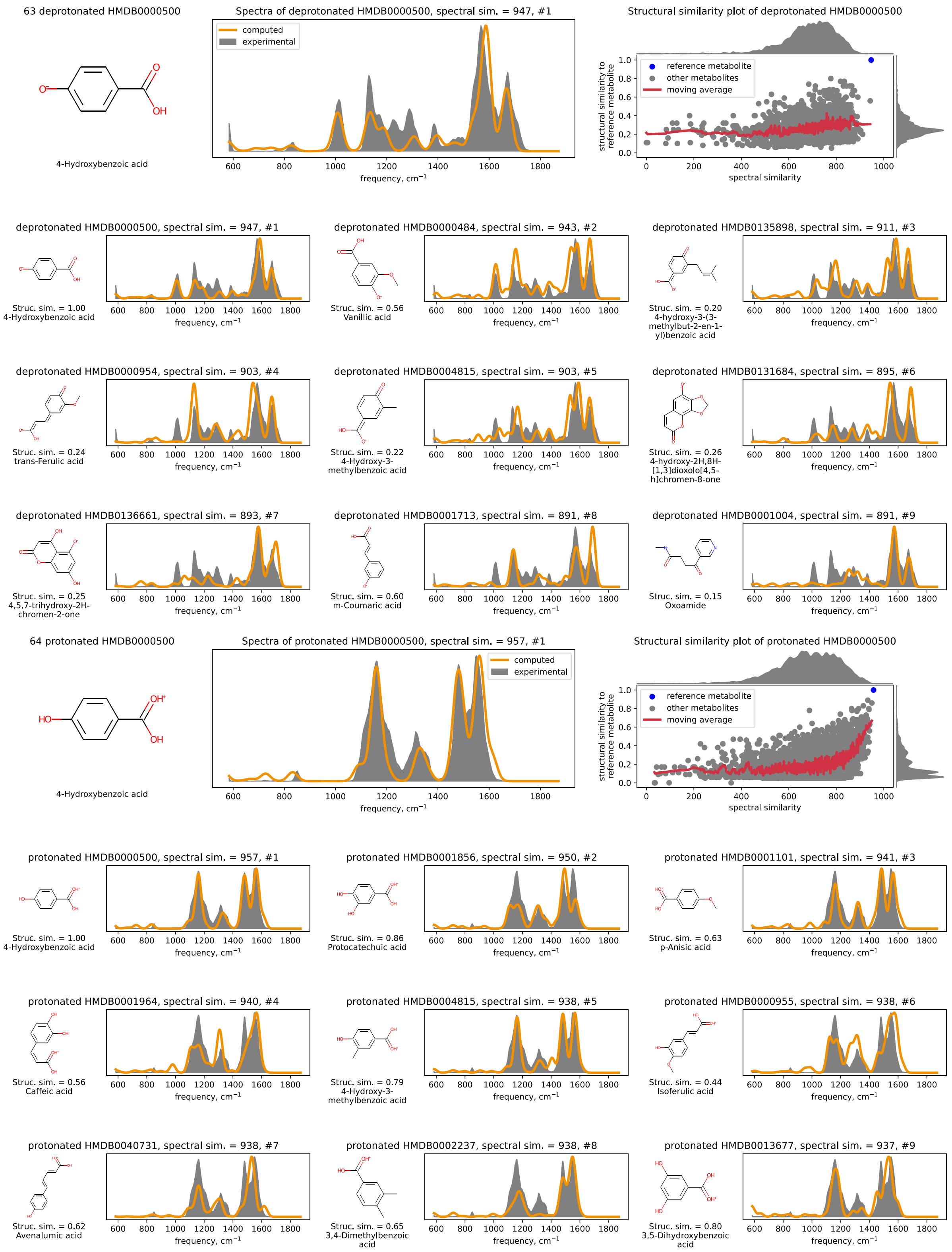
63 deprotonated HMDB0000500
4-Hydroxybenzoic acid
Spectra of deprotonated HMDB0000500, spectral sim. = 947, #1
computed
experimental
frequency, cm⁻¹
Structural similarity plot of deprotonated HMDB0000500
reference metabolite
other metabolites
moving average
structural similarity to reference metabolite
spectral similarity
deprotonated HMDB0000500, spectral sim. = 947, #1
Struc. sim. = 1.00
4-Hydroxybenzoic acid
deprotonated HMDB0000484, spectral sim. = 943, #2
Struc. sim. = 0.56
Vanillic acid
deprotonated HMDB0135898, spectral sim. = 911, #3
Struc. sim. = 0.20
4-hydroxy-3-(3-methylbut-2-en-1-yl)benzoic acid
deprotonated HMDB0000954, spectral sim. = 903, #4
Struc. sim. = 0.24
trans-Ferulic acid
deprotonated HMDB0004815, spectral sim. = 903, #5
Struc. sim. = 0.22
4-Hydroxy-3-methylbenzoic acid
deprotonated HMDB0131684, spectral sim. = 895, #6
Struc. sim. = 0.26
4-hydroxy-2H,8H-[1,3]dioxolo[4,5-h]chromen-8-one
deprotonated HMDB0136661, spectral sim. = 893, #7
Struc. sim. = 0.25
4,5,7-trihydroxy-2H-chromen-2-one
deprotonated HMDB0001713, spectral sim. = 891, #8
Struc. sim. = 0.60
m-Coumaric acid
deprotonated HMDB0001004, spectral sim. = 891, #9
Struc. sim. = 0.15
Oxoamide
64 protonated HMDB0000500
4-Hydroxybenzoic acid
Spectra of protonated HMDB0000500, spectral sim. = 957, #1
Structural similarity plot of protonated HMDB0000500
protonated HMDB0000500, spectral sim. = 957, #1
Struc. sim. = 1.00
4-Hydroxybenzoic acid
protonated HMDB0001856, spectral sim. = 950, #2
Struc. sim. = 0.86
Protocatechuic acid
protonated HMDB0001101, spectral sim. = 941, #3
Struc. sim. = 0.63
p-Anisic acid
protonated HMDB0001964, spectral sim. = 940, #4
Struc. sim. = 0.56
Caffeic acid
protonated HMDB0004815, spectral sim. = 938, #5
Struc. sim. = 0.79
4-Hydroxy-3-methylbenzoic acid
protonated HMDB0000955, spectral sim. = 938, #6
Struc. sim. = 0.44
Isoferulic acid
protonated HMDB0040731, spectral sim. = 938, #7
Struc. sim. = 0.62
Avenalumic acid
protonated HMDB0002237, spectral sim. = 938, #8
Struc. sim. = 0.65
3,4-Dimethylbenzoic acid
protonated HMDB0013677, spectral sim. = 937, #9
Struc. sim. = 0.80
3,5-Dihydroxybenzoic acid

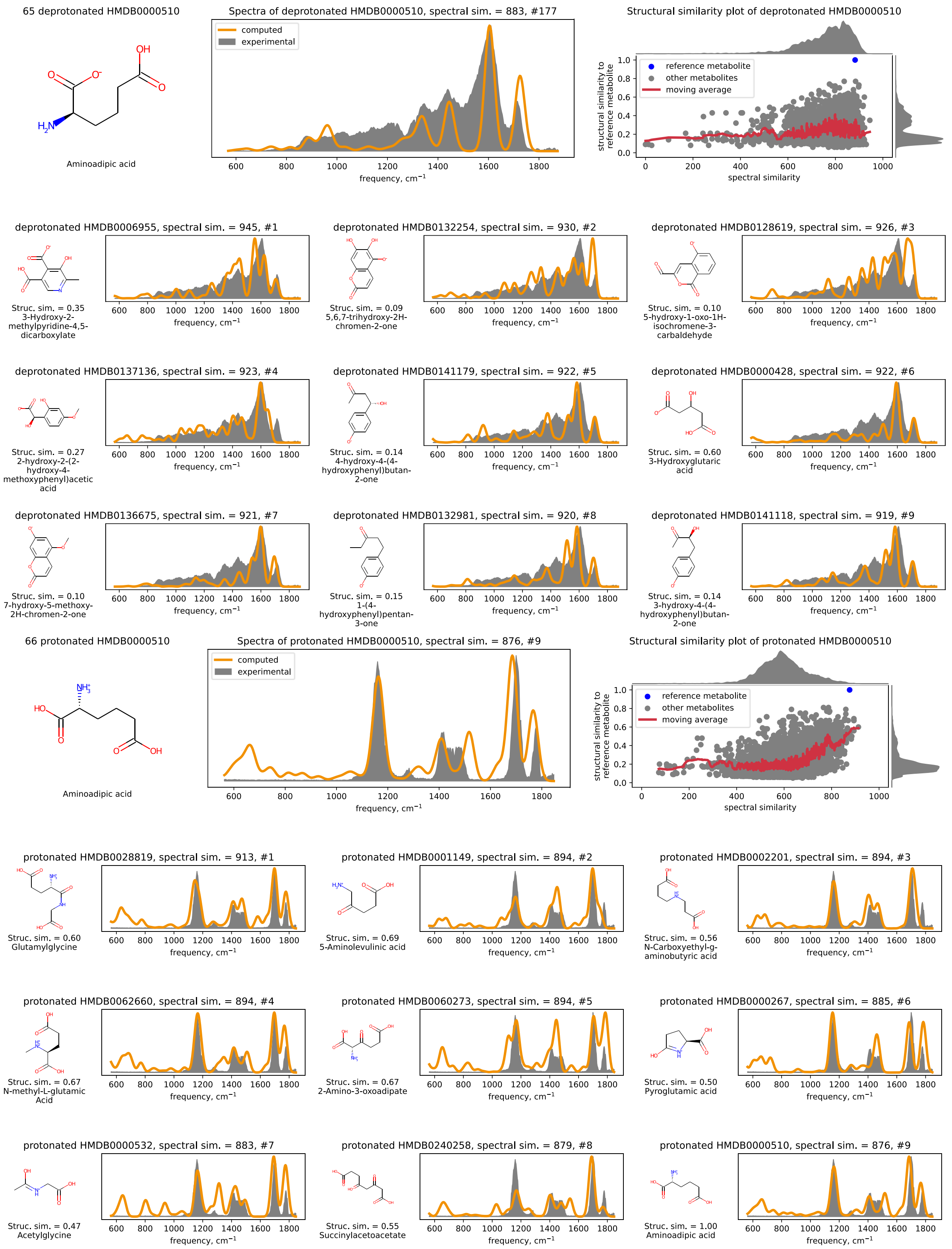

65 deprotonated HMDB0000510
Aminoadipic acid
Spectra of deprotonated HMDB0000510, spectral sim. = 883, #177
computed
experimental
frequency, cm⁻¹
Structural similarity plot of deprotonated HMDB0000510
reference metabolite
other metabolites
moving average
structural similarity to reference metabolite
spectral similarity
deprotonated HMDB0006955, spectral sim. = 945, #1
Struc. sim. = 0.35
3-Hydroxy-2-methylpyridine-4,5-dicarboxylate
deprotonated HMDB0132254, spectral sim. = 930, #2
Struc. sim. = 0.09
5,6,7-trihydroxy-2H-chromen-2-one
deprotonated HMDB0128619, spectral sim. = 926, #3
Struc. sim. = 0.10
5-hydroxy-1-oxo-1H-isochromene-3-carbaldehyde
deprotonated HMDB0137136, spectral sim. = 923, #4
Struc. sim. = 0.27
2-hydroxy-2-(2-hydroxy-4-methoxyphenyl)acetic acid
deprotonated HMDB0141179, spectral sim. = 922, #5
Struc. sim. = 0.14
4-hydroxy-4-(4-hydroxyphenyl)butan-2-one
deprotonated HMDB0000428, spectral sim. = 922, #6
Struc. sim. = 0.60
3-Hydroxyglutaric acid
deprotonated HMDB0136675, spectral sim. = 921, #7
Struc. sim. = 0.10
7-hydroxy-5-methoxy-2H-chromen-2-one
deprotonated HMDB0132981, spectral sim. = 920, #8
Struc. sim. = 0.15
1-(4-hydroxyphenyl)pentan-3-one
deprotonated HMDB0141118, spectral sim. = 919, #9
Struc. sim. = 0.14
3-hydroxy-4-(4-hydroxyphenyl)butan-2-one
66 protonated HMDB0000510
Aminoadipic acid
Spectra of protonated HMDB0000510, spectral sim. = 876, #9
computed
experimental
frequency, cm⁻¹
Structural similarity plot of protonated HMDB0000510
reference metabolite
other metabolites
moving average
structural similarity to reference metabolite
spectral similarity
protonated HMDB0028819, spectral sim. = 913, #1
Struc. sim. = 0.60
Glutamylglycine
protonated HMDB0001149, spectral sim. = 894, #2
Struc. sim. = 0.69
5-Aminolevulinic acid
protonated HMDB0002201, spectral sim. = 894, #3
Struc. sim. = 0.56
N-Carboxyethyl-g-aminobutyric acid
protonated HMDB0062660, spectral sim. = 894, #4
Struc. sim. = 0.67
N-methyl-L-glutamic Acid
protonated HMDB0060273, spectral sim. = 894, #5
Struc. sim. = 0.67
2-Amino-3-oxoadipate
protonated HMDB0000267, spectral sim. = 885, #6
Struc. sim. = 0.50
Pyroglutamic acid
protonated HMDB0000532, spectral sim. = 883, #7
Struc. sim. = 0.47
Acetylglycine
protonated HMDB0240258, spectral sim. = 879, #8
Struc. sim. = 0.55
Succinylacetoacetate
protonated HMDB0000510, spectral sim. = 876, #9
Struc. sim. = 1.00
Aminoadipic acid

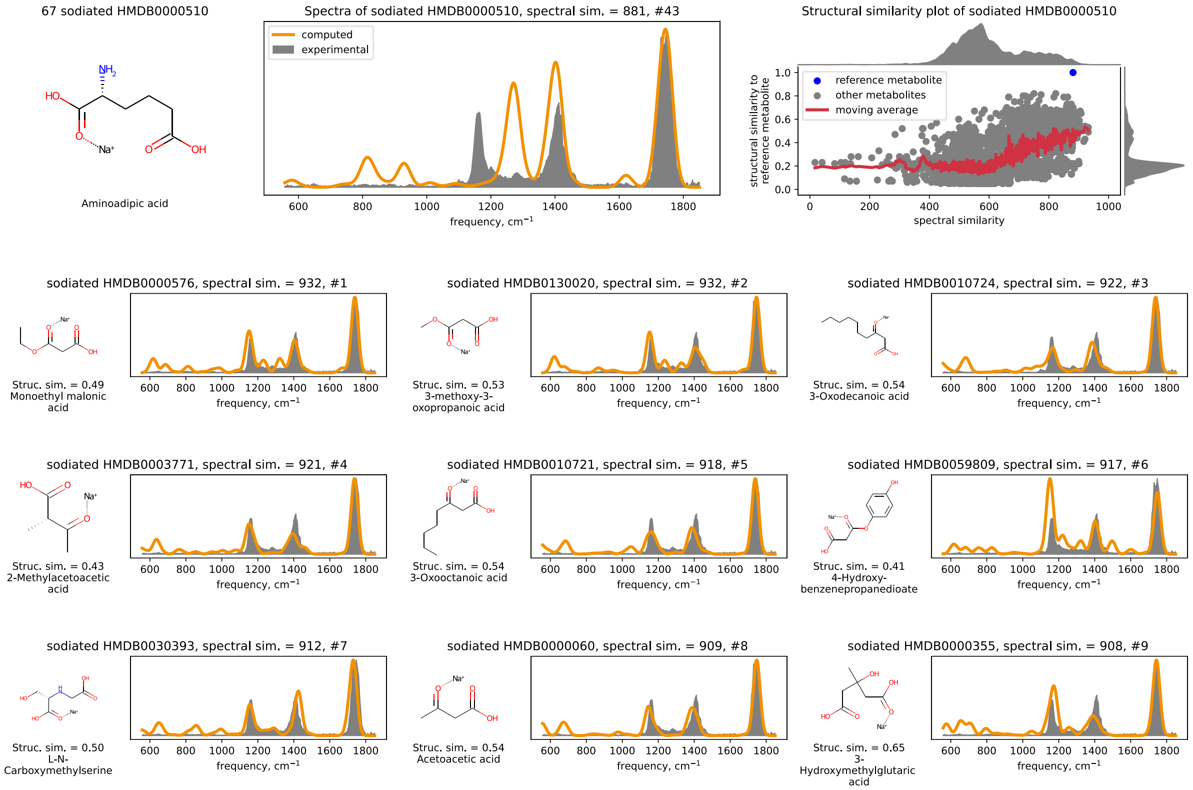
67 sodiated HMDB0000510
Aminoadipic acid
Spectra of sodiated HMDB0000510, spectral sim. = 881, #43
computed
experimental
frequency, cm⁻¹
Structural similarity plot of sodiated HMDB0000510
structural similarity to reference metabolite
reference metabolite
other metabolites
moving average
spectral similarity
sodiated HMDB0000576, spectral sim. = 932, #1
Struc. sim. = 0.49
Monoethyl malonic acid
sodiated HMDB0130020, spectral sim. = 932, #2
Struc. sim. = 0.53
3-methoxy-3-oxopropanoic acid
sodiated HMDB0010724, spectral sim. = 922, #3
Struc. sim. = 0.54
3-Oxodecanoic acid
sodiated HMDB0003771, spectral sim. = 921, #4
Struc. sim. = 0.43
2-Methylacetoacetic acid
sodiated HMDB0010721, spectral sim. = 918, #5
Struc. sim. = 0.54
3-Oxooctanoic acid
sodiated HMDB0059809, spectral sim. = 917, #6
Struc. sim. = 0.41
4-Hydroxy-benzenepropanedioate
sodiated HMDB0030393, spectral sim. = 912, #7
Struc. sim. = 0.50
L-N-Carboxymethylserine
sodiated HMDB0000060, spectral sim. = 909, #8
Struc. sim. = 0.54
Acetoacetic acid
sodiated HMDB0000355, spectral sim. = 908, #9
Struc. sim. = 0.65
3-Hydroxymethylglutaric acid

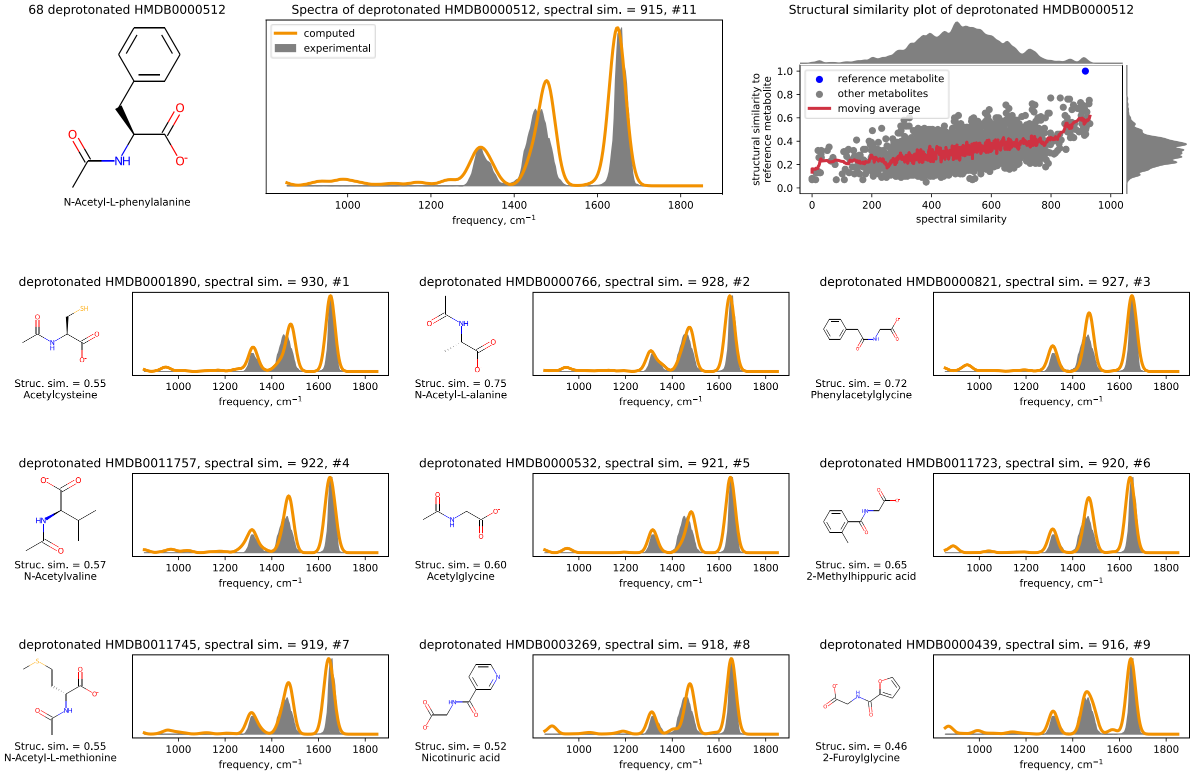
68 deprotonated HMDB0000512
N-Acetyl-L-phenylalanine
Spectra of deprotonated HMDB0000512, spectral sim. = 915, #11
computed
experimental
frequency, cm⁻¹
Structural similarity plot of deprotonated HMDB0000512
structural similarity to reference metabolite
reference metabolite
other metabolites
moving average
spectral similarity
deprotonated HMDB0001890, spectral sim. = 930, #1
Struc. sim. = 0.55
Acetylcysteine
deprotonated HMDB0000766, spectral sim. = 928, #2
Struc. sim. = 0.75
N-Acetyl-L-alanine
deprotonated HMDB0000821, spectral sim. = 927, #3
Struc. sim. = 0.72
Phenylacetylglycine
deprotonated HMDB0011757, spectral sim. = 922, #4
Struc. sim. = 0.57
N-Acetylvaline
deprotonated HMDB0000532, spectral sim. = 921, #5
Struc. sim. = 0.60
Acetylglycine
deprotonated HMDB0011723, spectral sim. = 920, #6
Struc. sim. = 0.65
2-Methylhippuric acid
deprotonated HMDB0011745, spectral sim. = 919, #7
Struc. sim. = 0.55
N-Acetyl-L-methionine
deprotonated HMDB0003269, spectral sim. = 918, #8
Struc. sim. = 0.52
Nicotinuric acid
deprotonated HMDB0000439, spectral sim. = 916, #9
Struc. sim. = 0.46
2-Furoylglycine

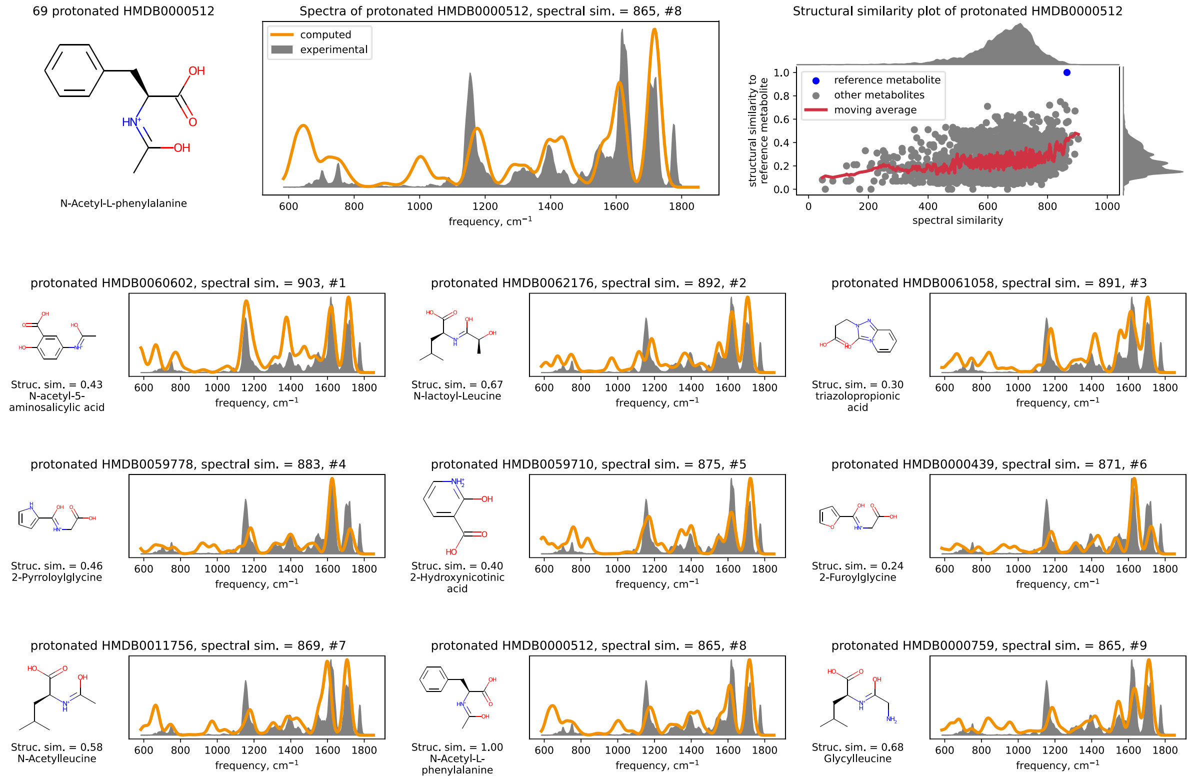
69 protonated HMDB0000512
N-Acetyl-L-phenylalanine
Spectra of protonated HMDB0000512, spectral sim. = 865, #8
computed
experimental
frequency, cm⁻¹
Structural similarity plot of protonated HMDB0000512
reference metabolite
other metabolites
moving average
structural similarity to reference metabolite
spectral similarity
protonated HMDB0060602, spectral sim. = 903, #1
Struc. sim. = 0.43
N-acetyl-5-aminosalicylic acid
protonated HMDB0062176, spectral sim. = 892, #2
Struc. sim. = 0.67
N-lactoyl-Leucine
protonated HMDB0061058, spectral sim. = 891, #3
Struc. sim. = 0.30
triazolopropionic acid
protonated HMDB0059778, spectral sim. = 883, #4
Struc. sim. = 0.46
2-Pyrroloylglycine
protonated HMDB0059710, spectral sim. = 875, #5
Struc. sim. = 0.40
2-Hydroxynicotinic acid
protonated HMDB0000439, spectral sim. = 871, #6
Struc. sim. = 0.24
2-Furoylglycine
protonated HMDB0011756, spectral sim. = 869, #7
Struc. sim. = 0.58
N-Acetylleucine
protonated HMDB0000512, spectral sim. = 865, #8
Struc. sim. = 1.00
N-Acetyl-L-phenylalanine
protonated HMDB0000759, spectral sim. = 865, #9
Struc. sim. = 0.68
Glycylleucine

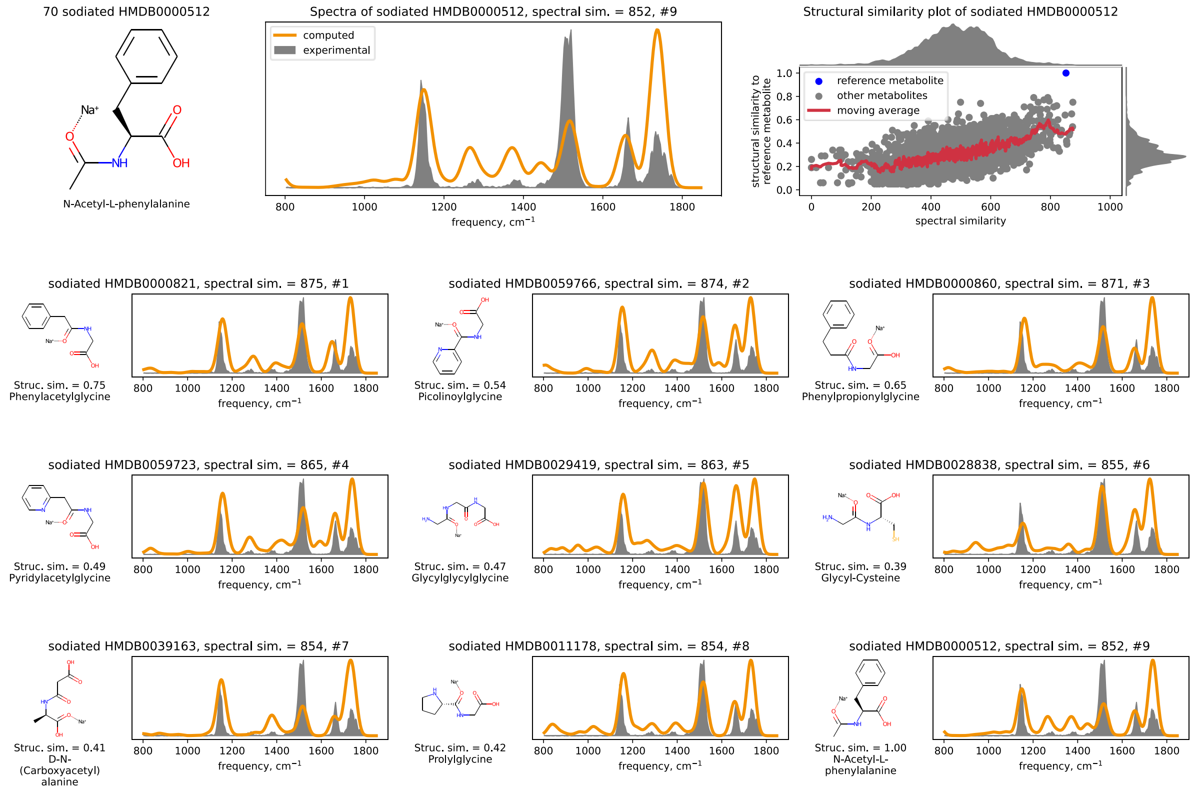
70 sodiated HMDB0000512
N-Acetyl-L-phenylalanine
Spectra of sodiated HMDB0000512, spectral sim. = 852, #9
computed
experimental
frequency, cm⁻¹
Structural similarity plot of sodiated HMDB0000512
reference metabolite
other metabolites
moving average
structural similarity to reference metabolite
spectral similarity
sodiated HMDB0000821, spectral sim. = 875, #1
Struc. sim. = 0.75
Phenylacetylglycine
sodiated HMDB0059766, spectral sim. = 874, #2
Struc. sim. = 0.54
Picolinoylglycine
sodiated HMDB0000860, spectral sim. = 871, #3
Struc. sim. = 0.65
Phenylpropionylglycine
sodiated HMDB0059723, spectral sim. = 865, #4
Struc. sim. = 0.49
Pyridylacetylglycine
sodiated HMDB0029419, spectral sim. = 863, #5
Struc. sim. = 0.47
Glycylglycylglycine
sodiated HMDB0028838, spectral sim. = 855, #6
Struc. sim. = 0.39
Glycyl-Cysteine
sodiated HMDB0039163, spectral sim. = 854, #7
Struc. sim. = 0.41
D-N-(Carboxyacetyl)alanine
sodiated HMDB0011178, spectral sim. = 854, #8
Struc. sim. = 0.42
Prolylglycine
sodiated HMDB0000512, spectral sim. = 852, #9
Struc. sim. = 1.00
N-Acetyl-L-phenylalanine

71 protonated HMDB0000517

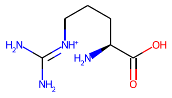

L-Arginine

Spectra of protonated HMDB0000517, spectral sim. = 925, #9

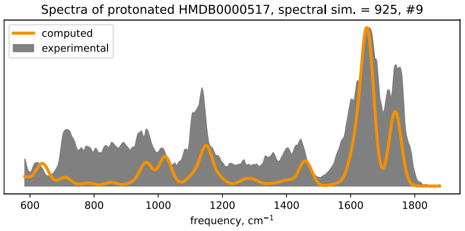

Structural similarity plot of protonated HMDB0000517

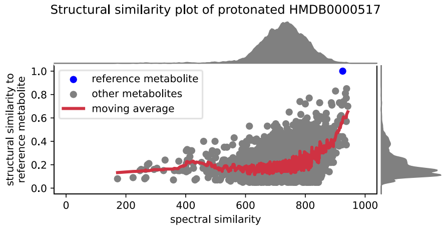

protonated HMDB0003334, spectral sim. = 942, #1

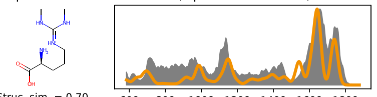

Struc. sim. = 0.70
Symmetric dimethylarginine

protonated HMDB0000214, spectral sim. = 938, #2

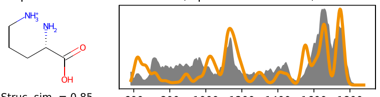

Struc. sim. = 0.85
Ornithine

protonated HMDB0028839, spectral sim. = 937, #3

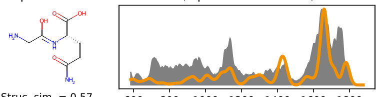

Struc. sim. = 0.57
Glycyl-Glutamine

protonated HMDB0003705, spectral sim. = 937, #4

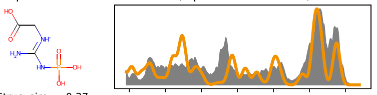

Struc. sim. = 0.37
Phosphoguanidinoacetat
e

protonated HMDB0002038, spectral sim. = 937, #5

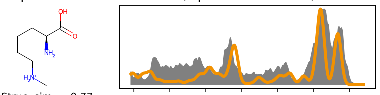

Struc. sim. = 0.77
N(6)-Methyllysine

protonated HMDB0029416, spectral sim. = 934, #6

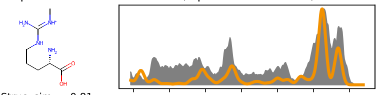

Struc. sim. = 0.81
L-Targinine

protonated HMDB0028854, spectral sim. = 932, #7

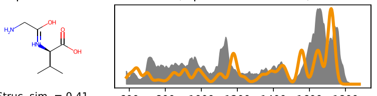

Struc. sim. = 0.41
Glycyl-Valine

protonated HMDB0000670, spectral sim. = 928, #8

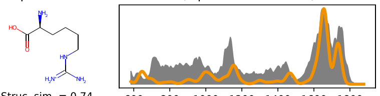

Struc. sim. = 0.74
Homo-L-arginine

protonated HMDB0000517, spectral sim. = 925, #9

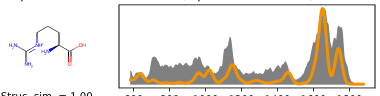

Struc. sim. = 1.00
L-Arginine

72 deprotonated HMDB0000522

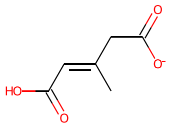

3-Methylglutaconic acid

Spectra of deprotonated HMDB0000522, spectral sim. = 797, #734

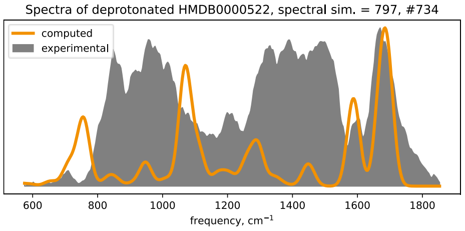

Structural similarity plot of deprotonated HMDB0000522

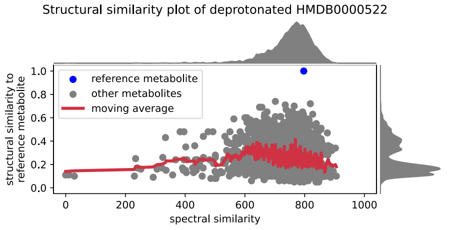

deprotonated HMDB0128622, spectral sim. = 906, #1

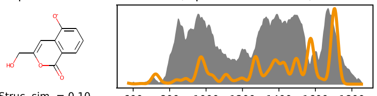

Struc. sim. = 0.10
5-hydroxy-3-(hydroxymethyl)-1H-isochromen-1-one

deprotonated HMDB0125594, spectral sim. = 903, #2

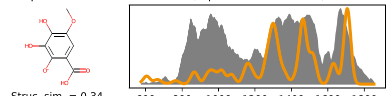

Struc. sim. = 0.34
2,3,4-trihydroxy-5-methoxybenzoic acid

deprotonated HMDB0134035, spectral sim. = 896, #3

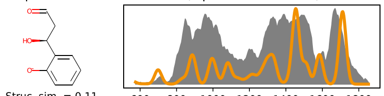

Struc. sim. = 0.11
3-hydroxy-3-(2-hydroxyphenyl)
propanal

deprotonated HMDB0135671, spectral sim. = 896, #4

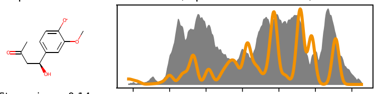

Struc. sim. = 0.14
4-hydroxy-4-(4-hydroxy-3-methoxyphenyl)butan-2-one

deprotonated HMDB0062477, spectral sim. = 895, #5

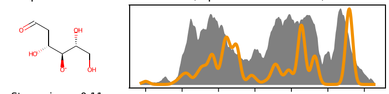

Struc. sim. = 0.11
2-Deoxyglucose

deprotonated HMDB0133511, spectral sim. = 892, #6

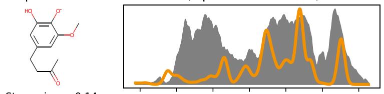

Struc. sim. = 0.14
4-(3,4-dihydroxy-5-methoxyphenyl)butan-2-one

deprotonated HMDB0038491, spectral sim. = 891, #7

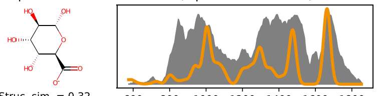

Struc. sim. = 0.32
L-Altruronic acid

deprotonated HMDB0034884, spectral sim. = 888, #8

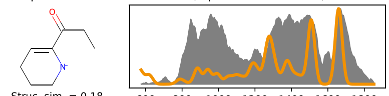

Struc. sim. = 0.18
1,2,3,4-Tetrahydro-6-propanoylpyridine

deprotonated HMDB0060256, spectral sim. = 887, #9

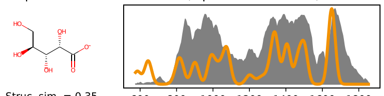

Struc. sim. = 0.35
L-Xylonate

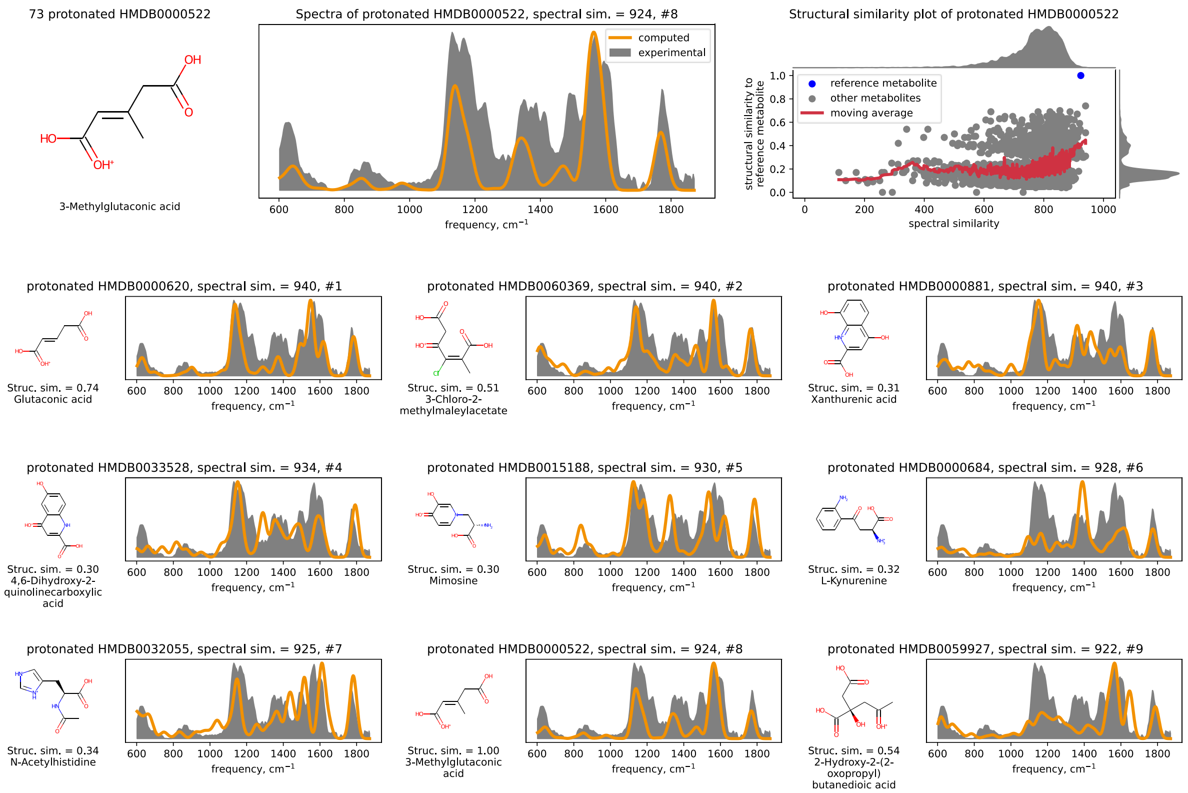
73 protonated HMDB0000522
3-Methylglutaconic acid
Spectra of protonated HMDB0000522, spectral sim. = 924, #8
computed
experimental
frequency, cm^{-1}
Structural similarity plot of protonated HMDB0000522
reference metabolite
other metabolites
moving average
structural similarity to reference metabolite
spectral similarity
protonated HMDB0000620, spectral sim. = 940, #1
Struc. sim. = 0.74
Glutaconic acid
protonated HMDB0060369, spectral sim. = 940, #2
Struc. sim. = 0.51
3-Chloro-2-methylmaleylacetate
protonated HMDB0000881, spectral sim. = 940, #3
Struc. sim. = 0.31
Xanthurenic acid
protonated HMDB0033528, spectral sim. = 934, #4
Struc. sim. = 0.30
4,6-Dihydroxy-2-quinolinecarboxylic acid
protonated HMDB0015188, spectral sim. = 930, #5
Struc. sim. = 0.30
Mimosine
protonated HMDB0000684, spectral sim. = 928, #6
Struc. sim. = 0.32
L-Kynurenine
protonated HMDB0032055, spectral sim. = 925, #7
Struc. sim. = 0.34
N-Acetylhistidine
protonated HMDB0000522, spectral sim. = 924, #8
Struc. sim. = 1.00
3-Methylglutaconic acid
protonated HMDB0059927, spectral sim. = 922, #9
Struc. sim. = 0.54
2-Hydroxy-2-(2-oxopropyl) butanedioic acid

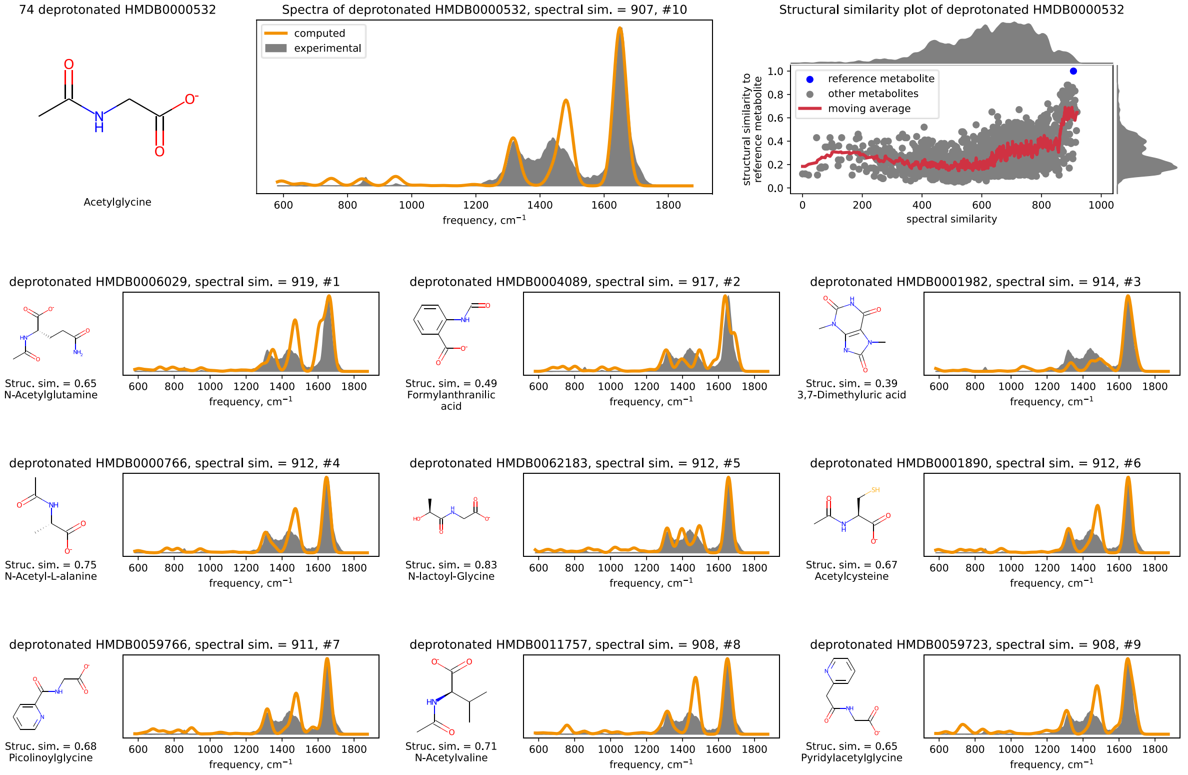
74 deprotonated HMDB0000532
Acetylglycine
Spectra of deprotonated HMDB0000532, spectral sim. = 907, #10
computed
experimental
frequency, cm^{-1}
Structural similarity plot of deprotonated HMDB0000532
reference metabolite
other metabolites
moving average
structural similarity to reference metabolite
spectral similarity
deprotonated HMDB0006029, spectral sim. = 919, #1
Struc. sim. = 0.65
N-Acetylglutamine
deprotonated HMDB0004089, spectral sim. = 917, #2
Struc. sim. = 0.49
Formylanthranilic acid
deprotonated HMDB0001982, spectral sim. = 914, #3
Struc. sim. = 0.39
3,7-Dimethyluric acid
deprotonated HMDB0000766, spectral sim. = 912, #4
Struc. sim. = 0.75
N-Acetyl-L-alanine
deprotonated HMDB0062183, spectral sim. = 912, #5
Struc. sim. = 0.83
N-lactoyl-Glycine
deprotonated HMDB0001890, spectral sim. = 912, #6
Struc. sim. = 0.67
Acetylcysteine
deprotonated HMDB0059766, spectral sim. = 911, #7
Struc. sim. = 0.68
Picolinoylglycine
deprotonated HMDB0011757, spectral sim. = 908, #8
Struc. sim. = 0.71
N-Acetylvaline
deprotonated HMDB0059723, spectral sim. = 908, #9
Struc. sim. = 0.65
Pyridylacetylglycine

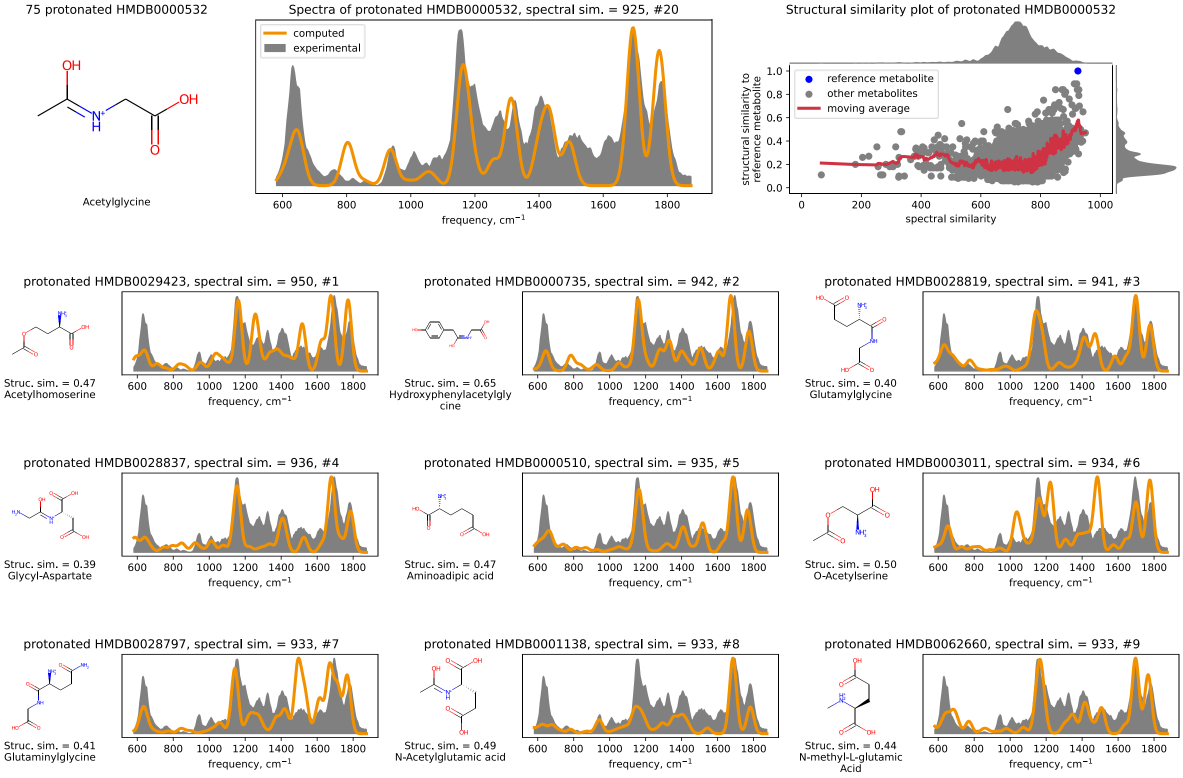
75 protonated HMDB0000532
Acetylglycine
Spectra of protonated HMDB0000532, spectral sim. = 925, #20
computed
experimental
frequency, cm⁻¹
Structural similarity plot of protonated HMDB0000532
reference metabolite
other metabolites
moving average
structural similarity to reference metabolite
spectral similarity
protonated HMDB0029423, spectral sim. = 950, #1
Struc. sim. = 0.47
Acetylhomoserine
protonated HMDB0000735, spectral sim. = 942, #2
Struc. sim. = 0.65
Hydroxyphenylacetylglycine
protonated HMDB0028819, spectral sim. = 941, #3
Struc. sim. = 0.40
Glutamylglycine
protonated HMDB0028837, spectral sim. = 936, #4
Struc. sim. = 0.39
Glycyl-Aspartate
protonated HMDB0000510, spectral sim. = 935, #5
Struc. sim. = 0.47
Aminoadipic acid
protonated HMDB0003011, spectral sim. = 934, #6
Struc. sim. = 0.50
O-Acetylserine
protonated HMDB0028797, spectral sim. = 933, #7
Struc. sim. = 0.41
Glutaminylglycine
protonated HMDB0001138, spectral sim. = 933, #8
Struc. sim. = 0.49
N-Acetylglutamic acid
protonated HMDB0062660, spectral sim. = 933, #9
Struc. sim. = 0.44
N-methyl-L-glutamic Acid
frequency, cm⁻¹

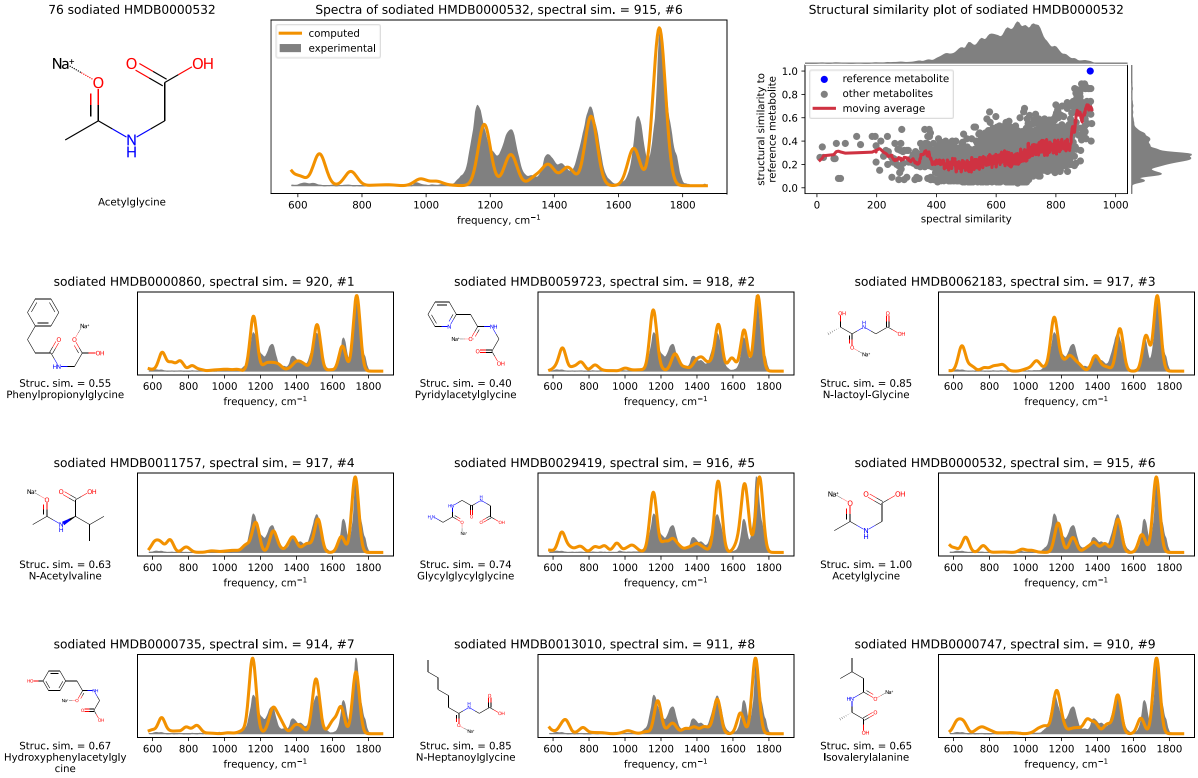
76 sodiated HMDB0000532
Acetylglycine
Spectra of sodiated HMDB0000532, spectral sim. = 915, #6
computed
experimental
frequency, cm⁻¹
Structural similarity plot of sodiated HMDB0000532
reference metabolite
other metabolites
moving average
structural similarity to reference metabolite
spectral similarity
sodiated HMDB0000860, spectral sim. = 920, #1
Struc. sim. = 0.55
Phenylpropionylglycine
sodiated HMDB0059723, spectral sim. = 918, #2
Struc. sim. = 0.40
Pyridylacetylglycine
sodiated HMDB0062183, spectral sim. = 917, #3
Struc. sim. = 0.85
N-lactoyl-Glycine
sodiated HMDB0011757, spectral sim. = 917, #4
Struc. sim. = 0.63
N-Acetylvaline
sodiated HMDB0029419, spectral sim. = 916, #5
Struc. sim. = 0.74
Glycylglycylglycine
sodiated HMDB0000532, spectral sim. = 915, #6
Struc. sim. = 1.00
Acetylglycine
sodiated HMDB0000735, spectral sim. = 914, #7
Struc. sim. = 0.67
Hydroxyphenylacetylglycine
sodiated HMDB0013010, spectral sim. = 911, #8
Struc. sim. = 0.85
N-Heptanoylglycine
sodiated HMDB0000747, spectral sim. = 910, #9
Struc. sim. = 0.65
Isovalerylalanine
frequency, cm⁻¹

77 deprotonated HMDB0000622

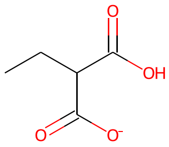

Ethylmalonic acid

Spectra of deprotonated HMDB0000622, spectral sim. = 773, #1059

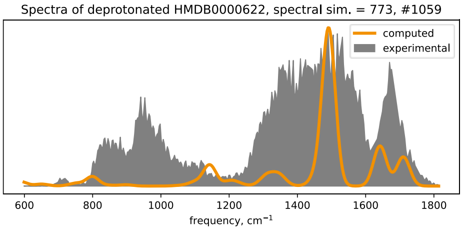

Structural similarity plot of deprotonated HMDB0000622

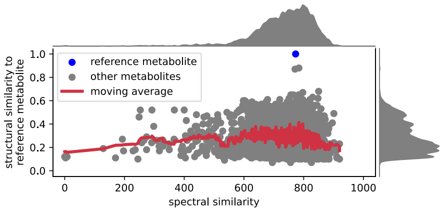

deprotonated HMDB0038180, spectral sim. = 920, #1

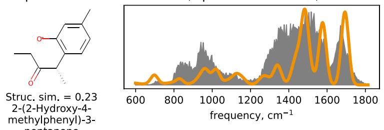

Struc. sim. = 0.23
2-(2-Hydroxy-4-methylphenyl)-3-pentanone

deprotonated HMDB0128622, spectral sim. = 920, #2

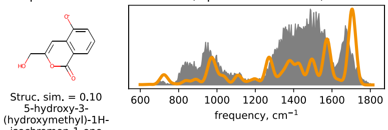

Struc. sim. = 0.10
5-hydroxy-3-(hydroxymethyl)-1H-isochromen-1-one

deprotonated HMDB0130402, spectral sim. = 911, #3

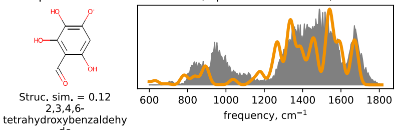

Struc. sim. = 0.12
2,3,4,6-tetrahydroxybenzaldehyde

deprotonated HMDB0128619, spectral sim. = 911, #4

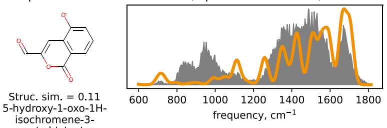

Struc. sim. = 0.11
5-hydroxy-1-oxo-1H-isochromene-3-carbaldehyde

deprotonated HMDB0038179, spectral sim. = 910, #5

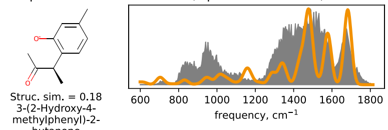

Struc. sim. = 0.18
3-(2-Hydroxy-4-methylphenyl)-2-butanone

deprotonated HMDB0038760, spectral sim. = 909, #6

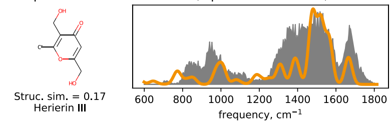

Struc. sim. = 0.17
Herierin III

deprotonated HMDB0032388, spectral sim. = 905, #7

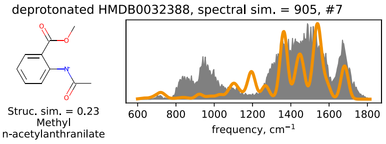

Struc. sim. = 0.23
Methyl n-acetylanthranilate

deprotonated HMDB0001276, spectral sim. = 901, #8

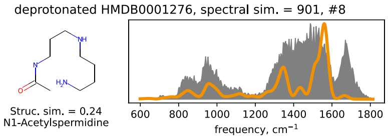

Struc. sim. = 0.24
N1-Acetylspermidine

deprotonated HMDB0006955, spectral sim. = 901, #9

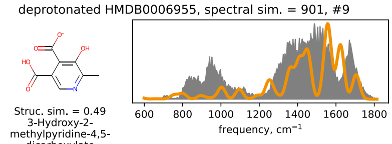

Struc. sim. = 0.49
3-Hydroxy-2-methylpyridine-4,5-dicarboxylate

78 protonated HMDB0000622

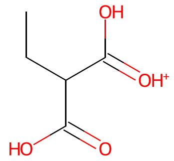

Ethylmalonic acid

Spectra of protonated HMDB0000622, spectral sim. = 837, #367

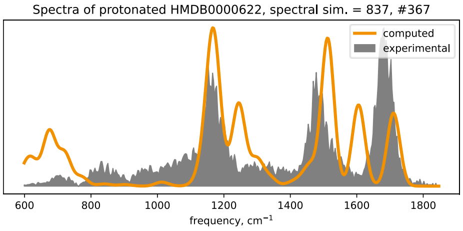

Structural similarity plot of protonated HMDB0000622

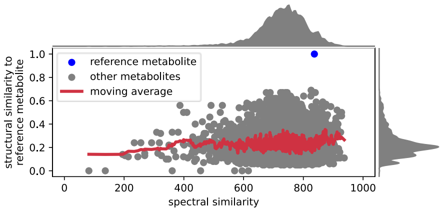

protonated HMDB0012948, spectral sim. = 937, #1

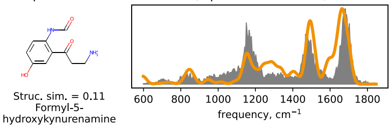

Struc. sim. = 0.11
Formyl-5-hydroxykynurenamine

protonated HMDB0014375, spectral sim. = 928, #2

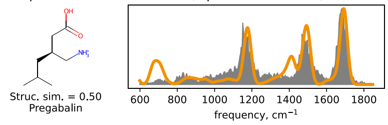

Struc. sim. = 0.50
Pregabalin

protonated HMDB0034099, spectral sim. = 928, #3

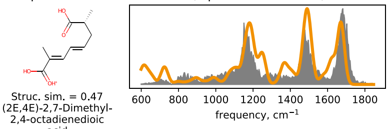

Struc. sim. = 0.47
(2E,4E)-2,7-Dimethyl-2,4-octadienedioic acid

protonated HMDB0005015, spectral sim. = 925, #4

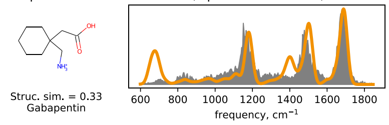

Struc. sim. = 0.33
Gabapentin

protonated HMDB0060998, spectral sim. = 923, #5

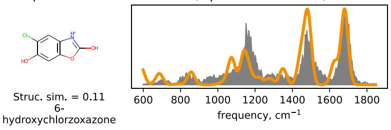

Struc. sim. = 0.11
6-hydroxychlorzoxazone

protonated HMDB0036582, spectral sim. = 923, #6

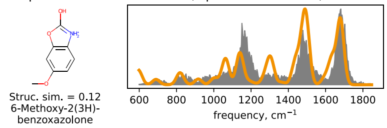

Struc. sim. = 0.12
6-Methoxy-2(3H)-benzoxazolone

protonated HMDB0031173, spectral sim. = 922, #7

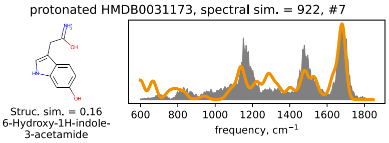

Struc. sim. = 0.16
6-Hydroxy-1H-indole-3-acetamide

protonated HMDB0002169, spectral sim. = 920, #8

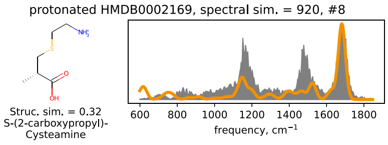

Struc. sim. = 0.32
S-(2-carboxypropyl)-Cysteamine

protonated HMDB0015212, spectral sim. = 920, #9

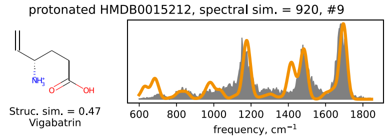

Struc. sim. = 0.47
Vigabatrin

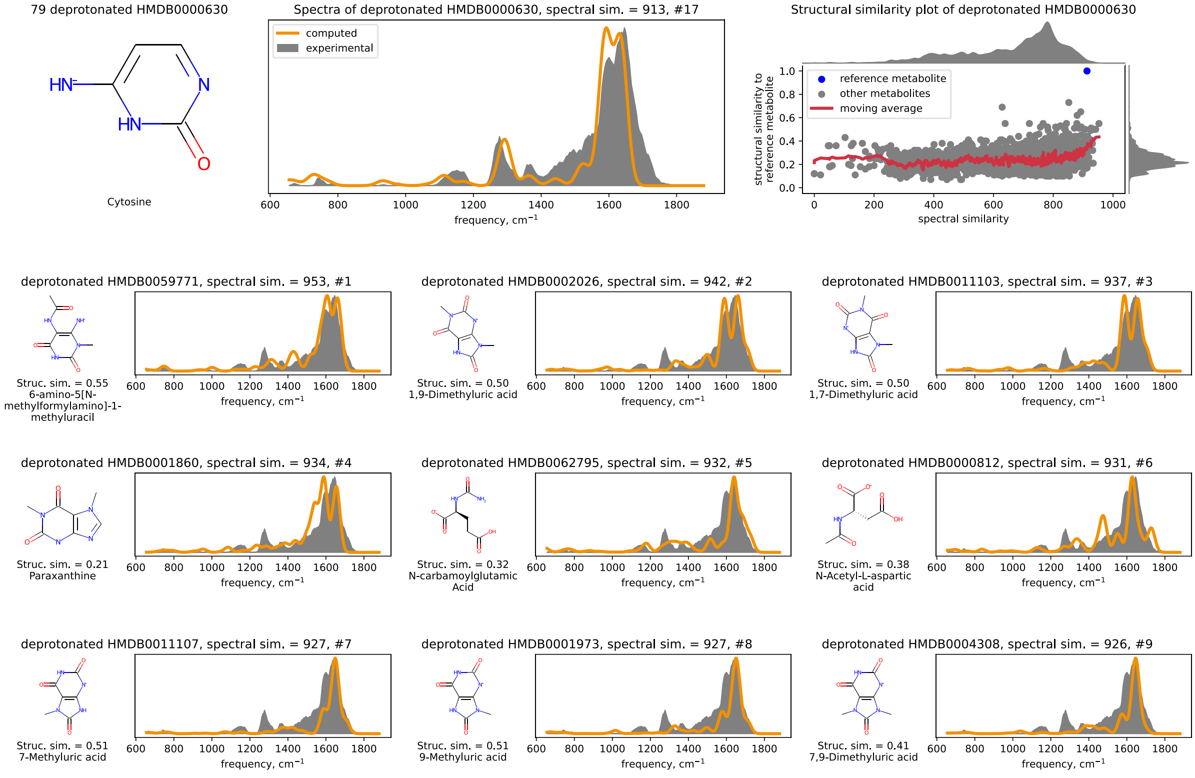
79 deprotonated HMDB0000630
Cytosine
Spectra of deprotonated HMDB0000630, spectral sim. = 913, #17
computed
experimental
frequency, cm^{-1}
Structural similarity plot of deprotonated HMDB0000630
reference metabolite
other metabolites
moving average
structural similarity to reference metabolite
spectral similarity
deprotonated HMDB0059771, spectral sim. = 953, #1
Struc. sim. = 0.55
6-amino-5[N-methylformylamino]-1-methyluracil
deprotonated HMDB0002026, spectral sim. = 942, #2
Struc. sim. = 0.50
1,9-Dimethyluric acid
deprotonated HMDB0011103, spectral sim. = 937, #3
Struc. sim. = 0.50
1,7-Dimethyluric acid
deprotonated HMDB0001860, spectral sim. = 934, #4
Struc. sim. = 0.21
Paraxanthine
deprotonated HMDB0062795, spectral sim. = 932, #5
Struc. sim. = 0.32
N-carbamoylglutamic Acid
deprotonated HMDB0000812, spectral sim. = 931, #6
Struc. sim. = 0.38
N-Acetyl-L-aspartic acid
deprotonated HMDB0011107, spectral sim. = 927, #7
Struc. sim. = 0.51
7-Methyluric acid
deprotonated HMDB0001973, spectral sim. = 927, #8
Struc. sim. = 0.51
9-Methyluric acid
deprotonated HMDB0004308, spectral sim. = 926, #9
Struc. sim. = 0.41
7,9-Dimethyluric acid

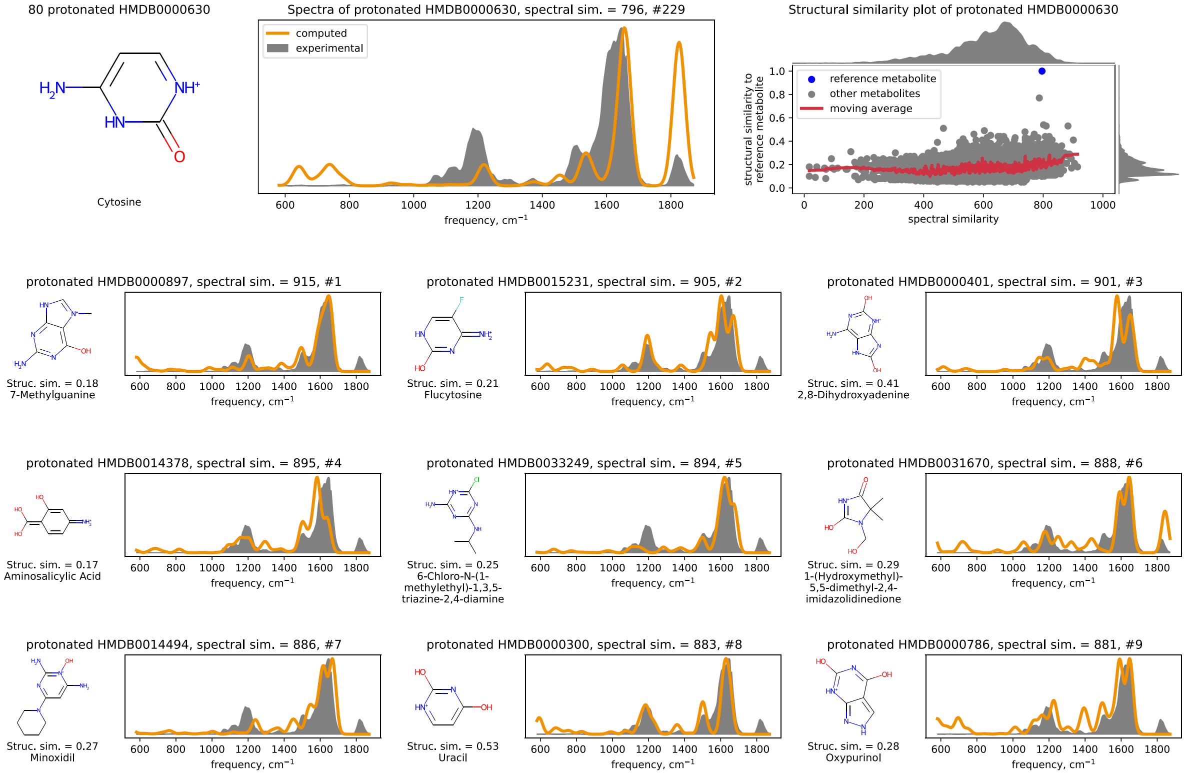
80 protonated HMDB0000630
Cytosine
Spectra of protonated HMDB0000630, spectral sim. = 796, #229
computed
experimental
frequency, cm^{-1}
Structural similarity plot of protonated HMDB0000630
reference metabolite
other metabolites
moving average
structural similarity to reference metabolite
spectral similarity
protonated HMDB0000897, spectral sim. = 915, #1
Struc. sim. = 0.18
7-Methylguanine
protonated HMDB0015231, spectral sim. = 905, #2
Struc. sim. = 0.21
Flucytosine
protonated HMDB0000401, spectral sim. = 901, #3
Struc. sim. = 0.41
2,8-Dihydroxyadenine
protonated HMDB0014378, spectral sim. = 895, #4
Struc. sim. = 0.17
Aminosalicylic Acid
protonated HMDB0033249, spectral sim. = 894, #5
Struc. sim. = 0.25
6-Chloro-N-(1-methylethyl)-1,3,5-triazine-2,4-diamine
protonated HMDB0031670, spectral sim. = 888, #6
Struc. sim. = 0.29
1-(Hydroxymethyl)-5,5-dimethyl-2,4-imidazolidinedione
protonated HMDB0014494, spectral sim. = 886, #7
Struc. sim. = 0.27
Minoxidil
protonated HMDB0000300, spectral sim. = 883, #8
Struc. sim. = 0.53
Uracil
protonated HMDB0000786, spectral sim. = 881, #9
Struc. sim. = 0.28
Oxypurinol

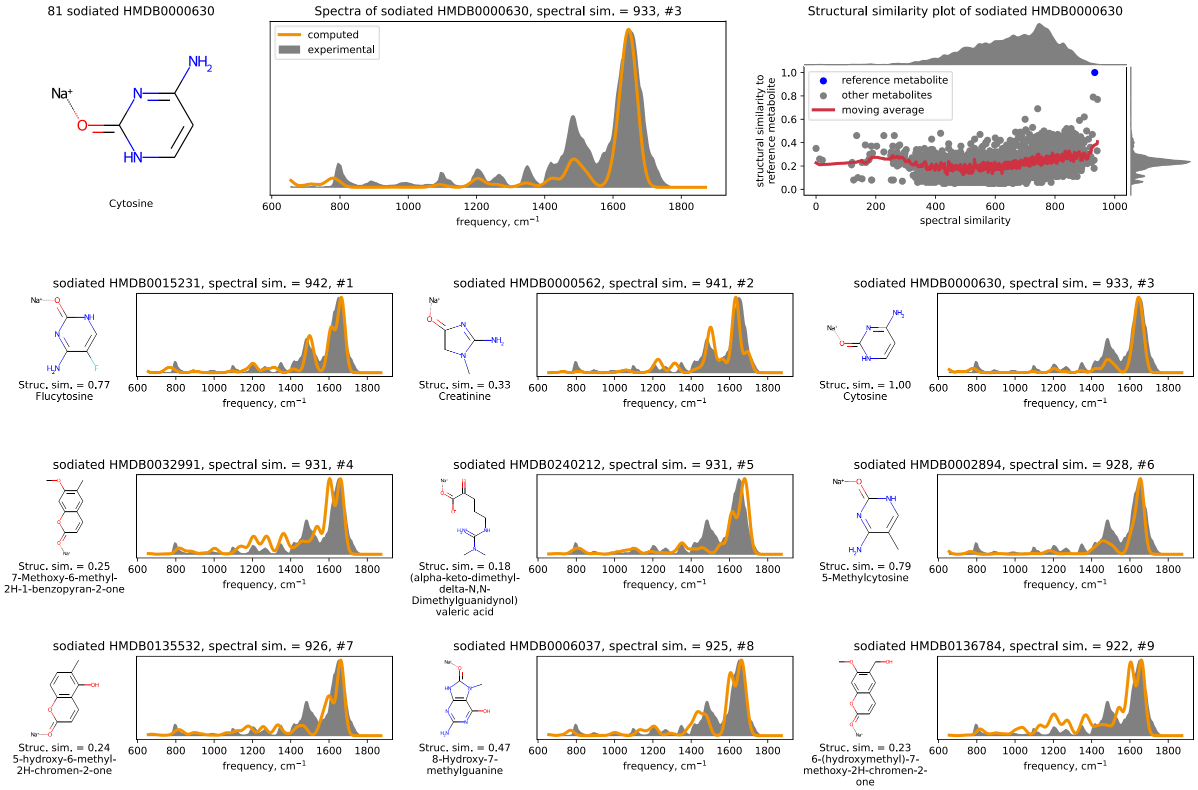
81 sodiated HMDB0000630
Cytosine
Spectra of sodiated HMDB0000630, spectral sim. = 933, #3
computed
experimental
frequency, cm⁻¹
Structural similarity plot of sodiated HMDB0000630
reference metabolite
other metabolites
moving average
structural similarity to reference metabolite
spectral similarity
sodiated HMDB0015231, spectral sim. = 942, #1
Struc. sim. = 0.77
Flucytosine
sodiated HMDB0000562, spectral sim. = 941, #2
Struc. sim. = 0.33
Creatinine
sodiated HMDB0000630, spectral sim. = 933, #3
Struc. sim. = 1.00
Cytosine
sodiated HMDB0032991, spectral sim. = 931, #4
Struc. sim. = 0.25
7-Methoxy-6-methyl-2H-1-benzopyran-2-one
sodiated HMDB0240212, spectral sim. = 931, #5
Struc. sim. = 0.18
(alpha-keto-dimethyl-delta-N,N-Dimethylguanidynol) valeric acid
sodiated HMDB0002894, spectral sim. = 928, #6
Struc. sim. = 0.79
5-Methylcytosine
sodiated HMDB0135532, spectral sim. = 926, #7
Struc. sim. = 0.24
5-hydroxy-6-methyl-2H-chromen-2-one
sodiated HMDB0006037, spectral sim. = 925, #8
Struc. sim. = 0.47
8-Hydroxy-7-methylguanine
sodiated HMDB0136784, spectral sim. = 922, #9
Struc. sim. = 0.23
6-(hydroxymethyl)-7-methoxy-2H-chromen-2-one

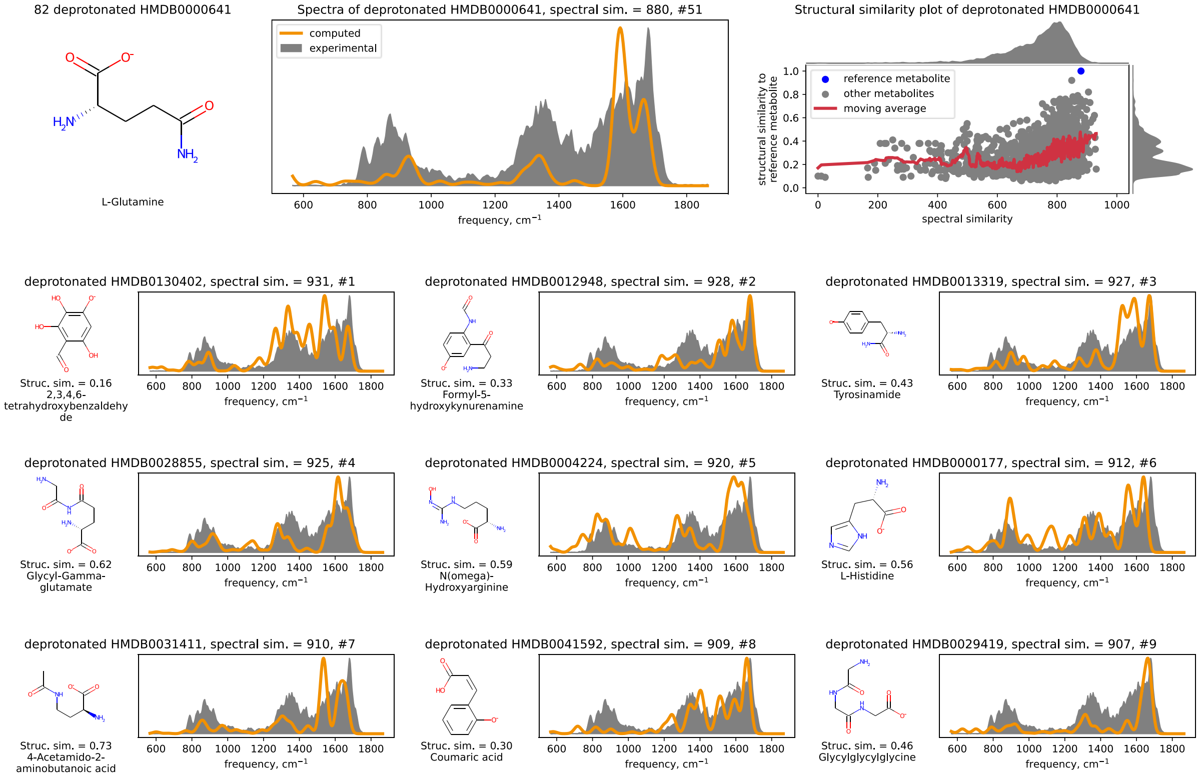
82 deprotonated HMDB0000641
L-Glutamine
Spectra of deprotonated HMDB0000641, spectral sim. = 880, #51
computed
experimental
frequency, cm⁻¹
Structural similarity plot of deprotonated HMDB0000641
reference metabolite
other metabolites
moving average
structural similarity to reference metabolite
spectral similarity
deprotonated HMDB0130402, spectral sim. = 931, #1
Struc. sim. = 0.16
2,3,4,6-tetrahydroxybenzaldehyde
deprotonated HMDB0012948, spectral sim. = 928, #2
Struc. sim. = 0.33
Formyl-5-hydroxykynurenamine
deprotonated HMDB0013319, spectral sim. = 927, #3
Struc. sim. = 0.43
Tyrosinamide
deprotonated HMDB0028855, spectral sim. = 925, #4
Struc. sim. = 0.62
Glycyl-Gamma-glutamate
deprotonated HMDB0004224, spectral sim. = 920, #5
Struc. sim. = 0.59
N(omega)-Hydroxyarginine
deprotonated HMDB0000177, spectral sim. = 912, #6
Struc. sim. = 0.56
L-Histidine
deprotonated HMDB0031411, spectral sim. = 910, #7
Struc. sim. = 0.73
4-Acetamido-2-aminobutanoic acid
deprotonated HMDB0041592, spectral sim. = 909, #8
Struc. sim. = 0.30
Coumaric acid
deprotonated HMDB0029419, spectral sim. = 907, #9
Struc. sim. = 0.46
Glycylglycylglycine

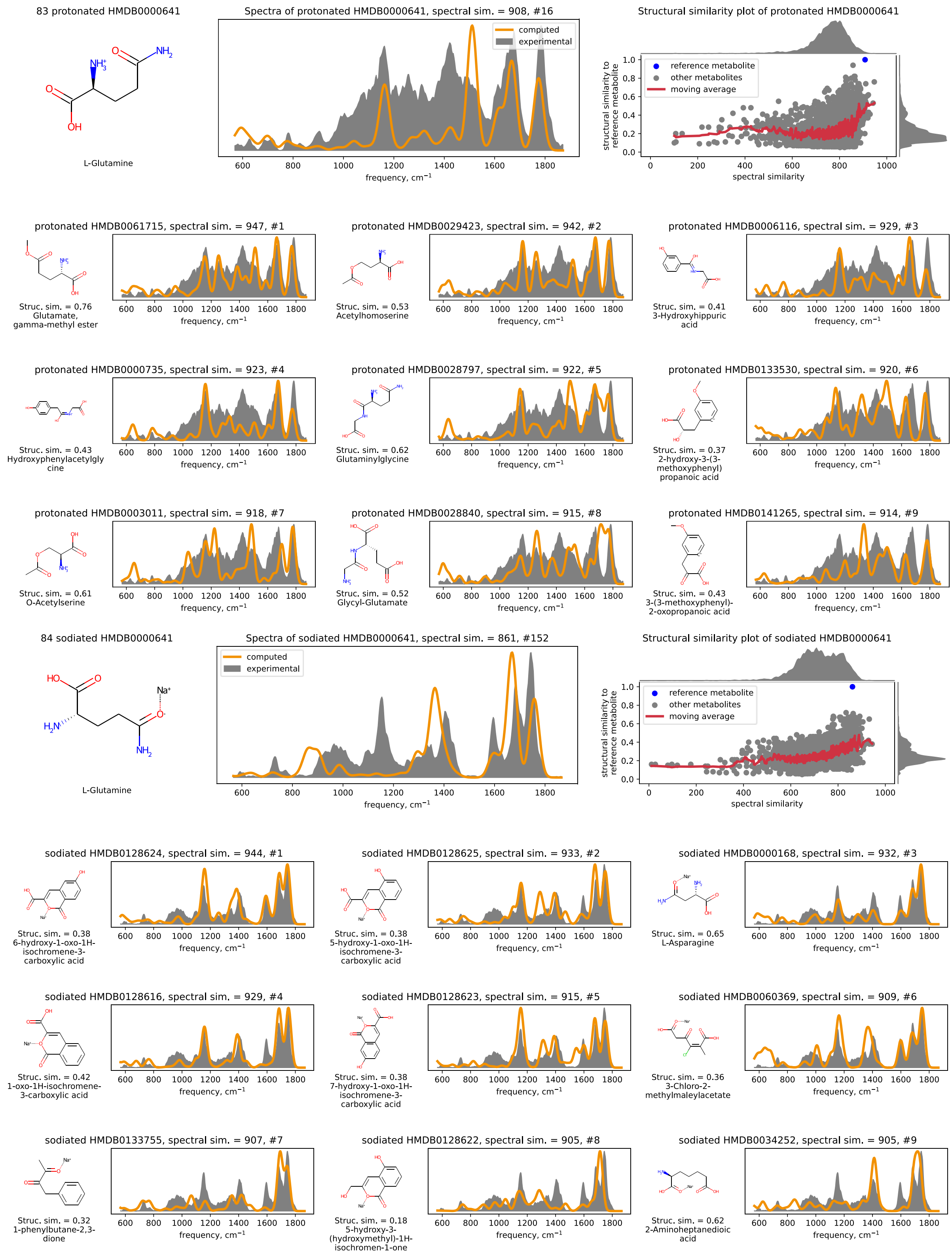
83 protonated HMDB0000641
L-Glutamine
Spectra of protonated HMDB0000641, spectral sim. = 908, #16
computed
experimental
frequency, cm⁻¹
Structural similarity plot of protonated HMDB0000641
reference metabolite
other metabolites
moving average
structural similarity to reference metabolite
spectral similarity
protonated HMDB0061715, spectral sim. = 947, #1
Struc. sim. = 0.76
Glutamate, gamma-methyl ester
protonated HMDB0029423, spectral sim. = 942, #2
Struc. sim. = 0.53
Acetylhomoserine
protonated HMDB0006116, spectral sim. = 929, #3
Struc. sim. = 0.41
3-Hydroxyhippuric acid
protonated HMDB0000735, spectral sim. = 923, #4
Struc. sim. = 0.43
Hydroxyphenylacetylglycine
protonated HMDB0028797, spectral sim. = 922, #5
Struc. sim. = 0.62
Glutaminylglycine
protonated HMDB0133530, spectral sim. = 920, #6
Struc. sim. = 0.37
2-hydroxy-3-(3-methoxyphenyl) propanoic acid
protonated HMDB0003011, spectral sim. = 918, #7
Struc. sim. = 0.61
O-Acetylserine
protonated HMDB0028840, spectral sim. = 915, #8
Struc. sim. = 0.52
Glycyl-Glutamate
protonated HMDB0141265, spectral sim. = 914, #9
Struc. sim. = 0.43
3-(3-methoxyphenyl)-2-oxopropanoic acid
84 sodiated HMDB0000641
L-Glutamine
Spectra of sodiated HMDB0000641, spectral sim. = 861, #152
Structural similarity plot of sodiated HMDB0000641
sodiated HMDB0128624, spectral sim. = 944, #1
Struc. sim. = 0.38
6-hydroxy-1-oxo-1H-isochromene-3-carboxylic acid
sodiated HMDB0128625, spectral sim. = 933, #2
Struc. sim. = 0.38
5-hydroxy-1-oxo-1H-isochromene-3-carboxylic acid
sodiated HMDB0000168, spectral sim. = 932, #3
Struc. sim. = 0.65
L-Asparagine
sodiated HMDB0128616, spectral sim. = 929, #4
Struc. sim. = 0.42
1-oxo-1H-isochromene-3-carboxylic acid
sodiated HMDB0128623, spectral sim. = 915, #5
Struc. sim. = 0.38
7-hydroxy-1-oxo-1H-isochromene-3-carboxylic acid
sodiated HMDB0060369, spectral sim. = 909, #6
Struc. sim. = 0.36
3-Chloro-2-methylmaleylacetate
sodiated HMDB0133755, spectral sim. = 907, #7
Struc. sim. = 0.32
1-phenylbutane-2,3-dione
sodiated HMDB0128622, spectral sim. = 905, #8
Struc. sim. = 0.18
5-hydroxy-3-(hydroxymethyl)-1H-isochromen-1-one
sodiated HMDB0034252, spectral sim. = 905, #9
Struc. sim. = 0.62
2-Aminoheptanedioic acid

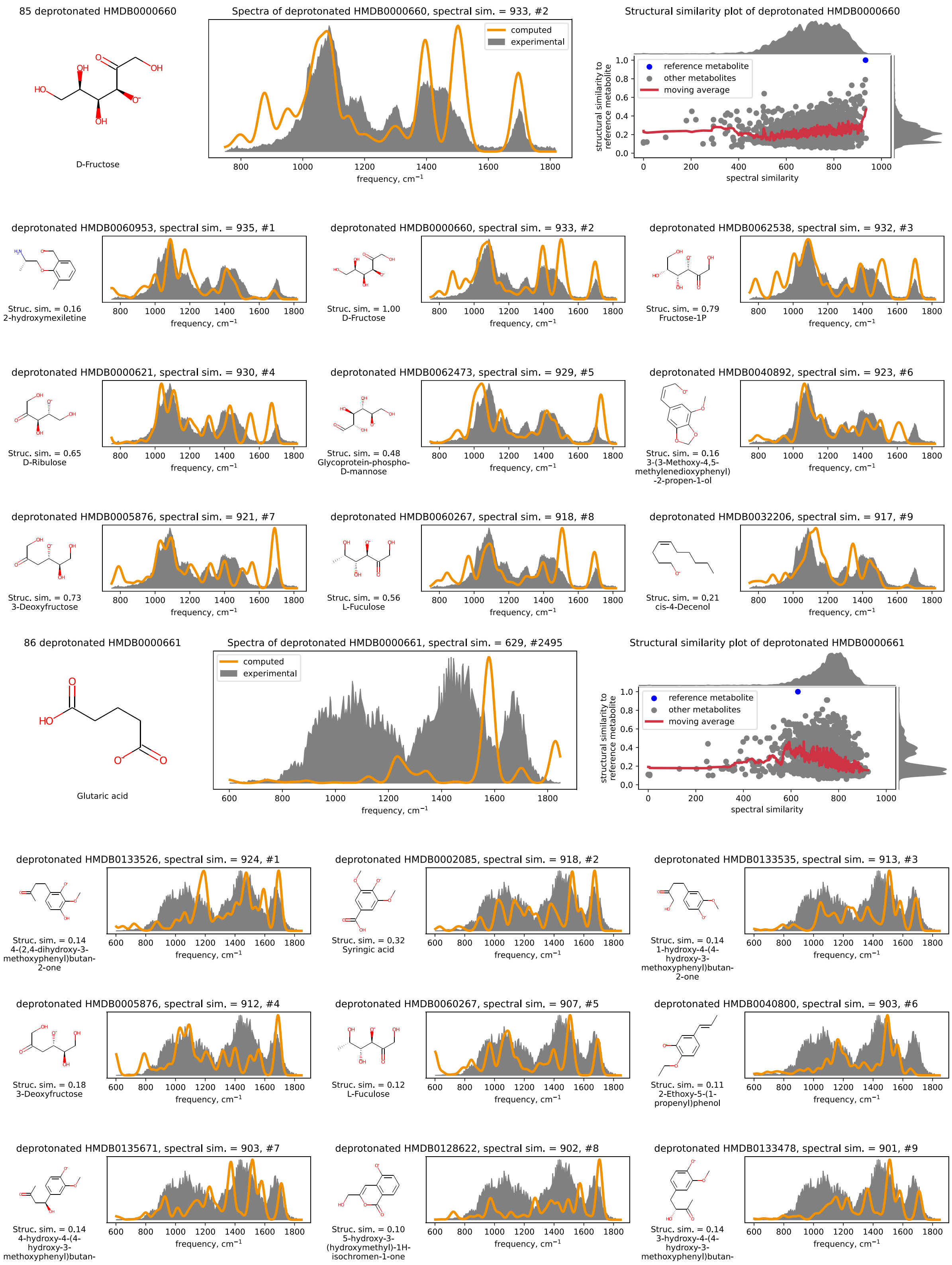
85 deprotonated HMDB0000660
D-Fructose
Spectra of deprotonated HMDB0000660, spectral sim. = 933, #2
computed
experimental
frequency, cm^{-1}
Structural similarity plot of deprotonated HMDB0000660
structural similarity to reference metabolite
reference metabolite
other metabolites
moving average
spectral similarity
deprotonated HMDB0060953, spectral sim. = 935, #1
Struc. sim. = 0.16
2-hydroxymexiletine
deprotonated HMDB0000660, spectral sim. = 933, #2
Struc. sim. = 1.00
D-Fructose
deprotonated HMDB0062538, spectral sim. = 932, #3
Struc. sim. = 0.79
Fructose-1P
deprotonated HMDB0000621, spectral sim. = 930, #4
Struc. sim. = 0.65
D-Ribulose
deprotonated HMDB0062473, spectral sim. = 929, #5
Struc. sim. = 0.48
Glycoprotein-phospho-D-mannose
deprotonated HMDB0040892, spectral sim. = 923, #6
Struc. sim. = 0.16
3-(3-Methoxy-4,5-methylenedioxyphenyl)-2-propen-1-ol
deprotonated HMDB0005876, spectral sim. = 921, #7
Struc. sim. = 0.73
3-Deoxyfructose
deprotonated HMDB0060267, spectral sim. = 918, #8
Struc. sim. = 0.56
L-Fuculose
deprotonated HMDB0032206, spectral sim. = 917, #9
Struc. sim. = 0.21
cis-4-Decenol
86 deprotonated HMDB0000661
Glutaric acid
Spectra of deprotonated HMDB0000661, spectral sim. = 629, #2495
computed
experimental
frequency, cm^{-1}
Structural similarity plot of deprotonated HMDB0000661
structural similarity to reference metabolite
reference metabolite
other metabolites
moving average
spectral similarity
deprotonated HMDB0133526, spectral sim. = 924, #1
Struc. sim. = 0.14
4-(2,4-dihydroxy-3-methoxyphenyl)butan-2-one
deprotonated HMDB0002085, spectral sim. = 918, #2
Struc. sim. = 0.32
Syringic acid
deprotonated HMDB0133535, spectral sim. = 913, #3
Struc. sim. = 0.14
1-hydroxy-4-(4-hydroxy-3-methoxyphenyl)butan-2-one
deprotonated HMDB0005876, spectral sim. = 912, #4
Struc. sim. = 0.18
3-Deoxyfructose
deprotonated HMDB0060267, spectral sim. = 907, #5
Struc. sim. = 0.12
L-Fuculose
deprotonated HMDB0040800, spectral sim. = 903, #6
Struc. sim. = 0.11
2-Ethoxy-5-(1-propenyl)phenol
deprotonated HMDB0135671, spectral sim. = 903, #7
Struc. sim. = 0.14
4-hydroxy-4-(4-hydroxy-3-methoxyphenyl)butan-
deprotonated HMDB0128622, spectral sim. = 902, #8
Struc. sim. = 0.10
5-hydroxy-3-(hydroxymethyl)-1H-isochromen-1-one
deprotonated HMDB0133478, spectral sim. = 901, #9
Struc. sim. = 0.14
3-hydroxy-4-(4-hydroxy-3-methoxyphenyl)butan-
frequency, cm^{-1}

87 protonated HMDB0000661

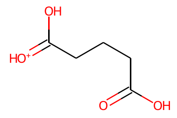

Glutaric acid

Spectra of protonated HMDB0000661, spectral sim. = 701, #4031

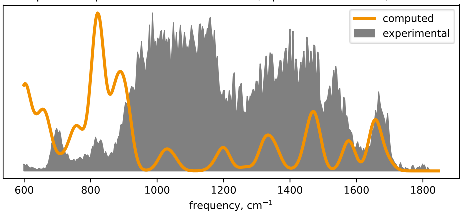

Structural similarity plot of protonated HMDB0000661

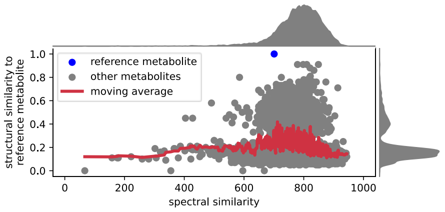

protonated HMDB0031846, spectral sim. = 944, #1

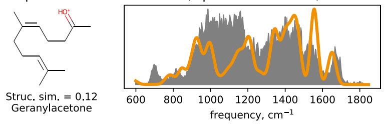

Struc. sim. = 0.12
Geranylacetone

protonated HMDB0013070, spectral sim. = 942, #2

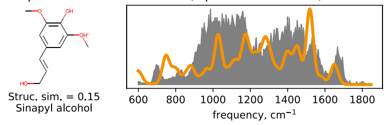

Struc. sim. = 0.15
Sinapyl alcohol

protonated HMDB0126382, spectral sim. = 939, #3

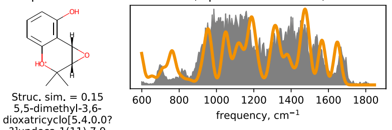

Struc. sim. = 0.15
5,5-dimethyl-3,6-dioxatricyclo[5.4.0.0?,?]undeca-1(11),7,9-trien-11-ol

protonated HMDB0060953, spectral sim. = 938, #4

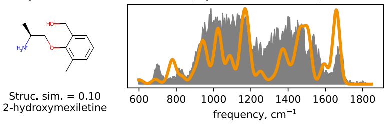

Struc. sim. = 0.10
2-hydroxymexiletine

protonated HMDB0040038, spectral sim. = 935, #5

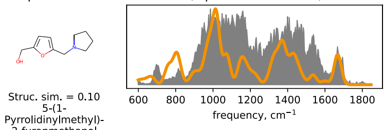

Struc. sim. = 0.10
5-(1-Pyrrolidinylmethyl)-2-furanmethanol

protonated HMDB0041194, spectral sim. = 935, #6

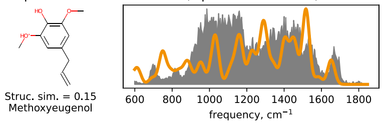

Struc. sim. = 0.15
Methoxyeugenol

protonated HMDB0060267, spectral sim. = 932, #7

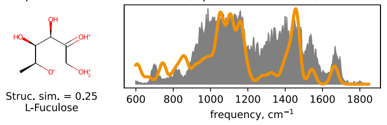

Struc. sim. = 0.25
L-Fuculose

protonated HMDB0033577, spectral sim. = 930, #8

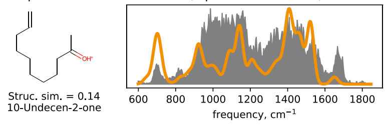

Struc. sim. = 0.14
10-Undecen-2-one

protonated HMDB0036055, spectral sim. = 930, #9

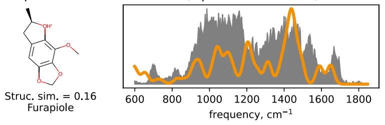

Struc. sim. = 0.16
Furapiole

88 deprotonated HMDB0000679

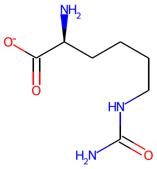

Homocitrulline

Spectra of deprotonated HMDB0000679, spectral sim. = 924, #4

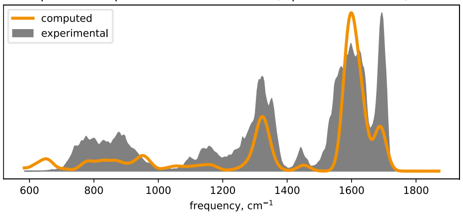

Structural similarity plot of deprotonated HMDB0000679

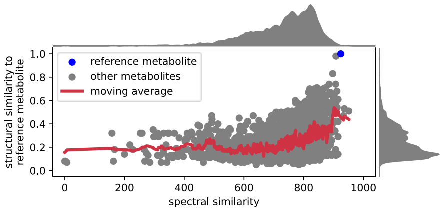

deprotonated HMDB0028855, spectral sim. = 951, #1

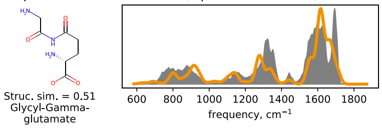

Struc. sim. = 0.51
Glycyl-Gamma-glutamate

deprotonated HMDB0004224, spectral sim. = 936, #2

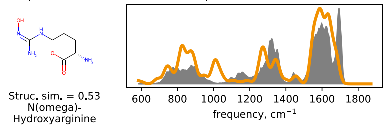

Struc. sim. = 0.53
N(omega)-Hydroxyarginine

deprotonated HMDB0041540, spectral sim. = 933, #3

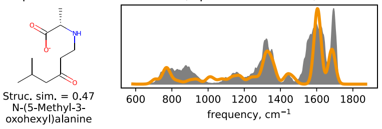

Struc. sim. = 0.47
N-(5-Methyl-3-oxohexyl)alanine

deprotonated HMDB0000679, spectral sim. = 924, #4

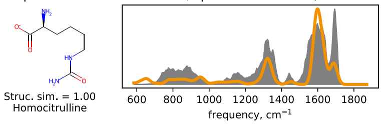

Struc. sim. = 1.00
Homocitrulline

deprotonated HMDB0012948, spectral sim. = 915, #5

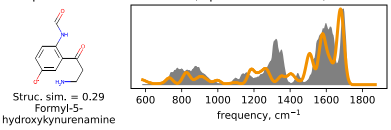

Struc. sim. = 0.29
Formyl-5-hydroxykynurenamine

deprotonated HMDB0040883, spectral sim. = 915, #6

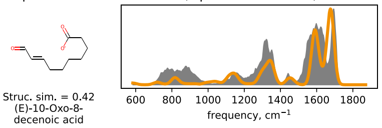

Struc. sim. = 0.42
(E)-10-Oxo-8-decenoic acid

deprotonated HMDB0137904, spectral sim. = 915, #7

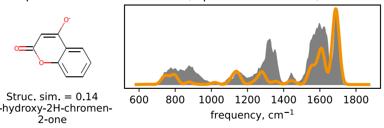

Struc. sim. = 0.14
4-hydroxy-2H-chromen-2-one

deprotonated HMDB0012150, spectral sim. = 915, #8

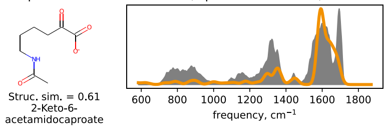

Struc. sim. = 0.61
2-Keto-6-acetamidocaproate

deprotonated HMDB0000684, spectral sim. = 912, #9

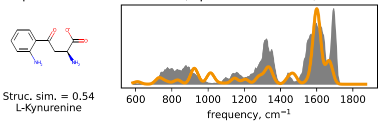

Struc. sim. = 0.54
L-Kynurenine

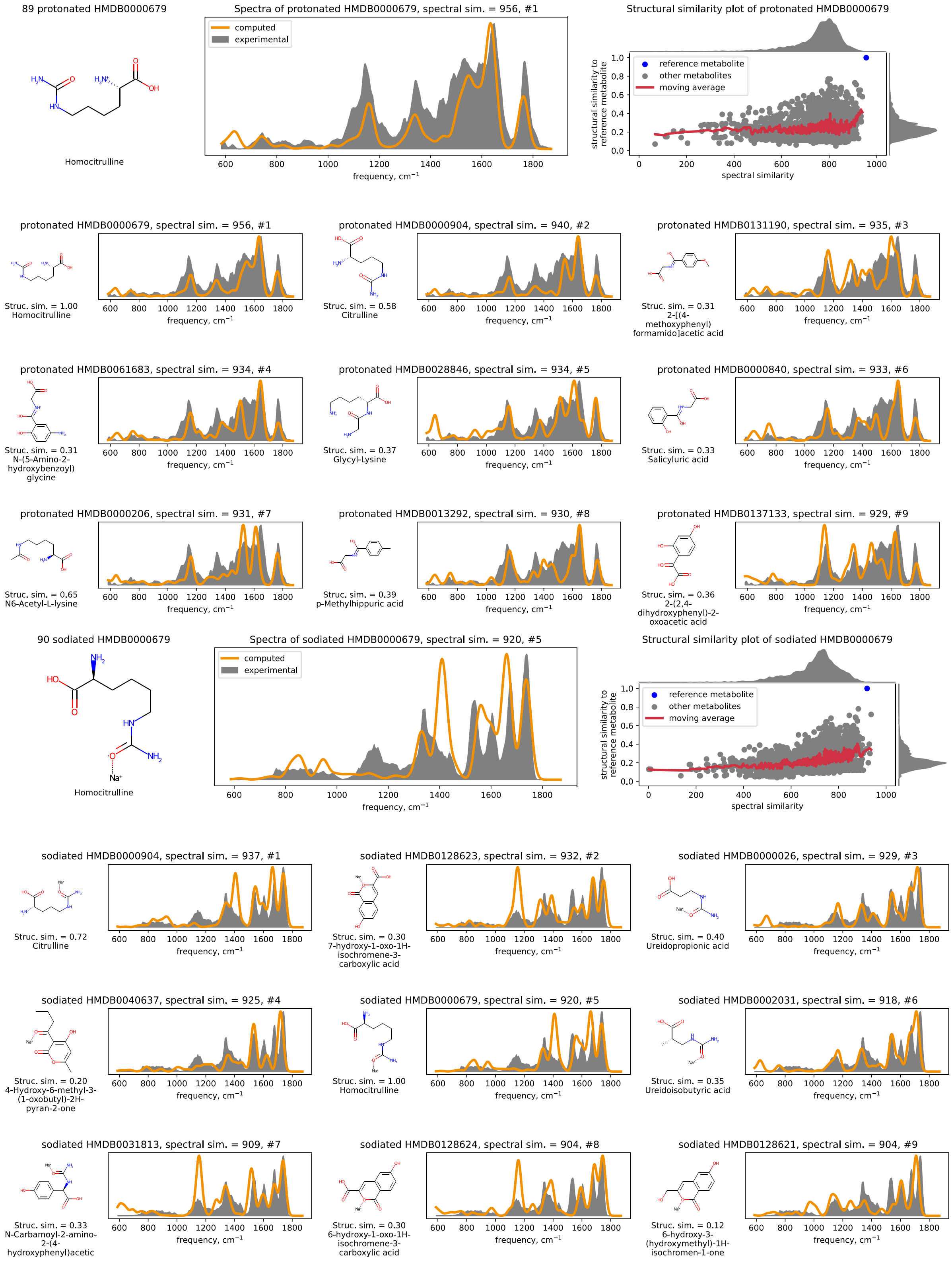
89 protonated HMDB0000679
Homocitrulline
Spectra of protonated HMDB0000679, spectral sim. = 956, #1
computed
experimental
frequency, cm^{-1}
Structural similarity plot of protonated HMDB0000679
reference metabolite
other metabolites
moving average
structural similarity to reference metabolite
spectral similarity
protonated HMDB0000679, spectral sim. = 956, #1
Struc. sim. = 1.00
Homocitrulline
protonated HMDB0000904, spectral sim. = 940, #2
Struc. sim. = 0.58
Citrulline
protonated HMDB0131190, spectral sim. = 935, #3
Struc. sim. = 0.31
2-[(4-methoxyphenyl)formamido]acetic acid
protonated HMDB0061683, spectral sim. = 934, #4
Struc. sim. = 0.31
N-(5-Amino-2-hydroxybenzoyl)glycine
protonated HMDB0028846, spectral sim. = 934, #5
Struc. sim. = 0.37
Glycyl-Lysine
protonated HMDB0000840, spectral sim. = 933, #6
Struc. sim. = 0.33
Salicyluric acid
protonated HMDB0000206, spectral sim. = 931, #7
Struc. sim. = 0.65
N6-Acetyl-L-lysine
protonated HMDB0013292, spectral sim. = 930, #8
Struc. sim. = 0.39
p-Methylhippuric acid
protonated HMDB0137133, spectral sim. = 929, #9
Struc. sim. = 0.36
2-(2,4-dihydroxyphenyl)-2-oxoacetic acid
90 sodiated HMDB0000679
Homocitrulline
Spectra of sodiated HMDB0000679, spectral sim. = 920, #5
computed
experimental
frequency, cm^{-1}
Structural similarity plot of sodiated HMDB0000679
reference metabolite
other metabolites
moving average
structural similarity to reference metabolite
spectral similarity
sodiated HMDB0000904, spectral sim. = 937, #1
Struc. sim. = 0.72
Citrulline
sodiated HMDB0128623, spectral sim. = 932, #2
Struc. sim. = 0.30
7-hydroxy-1-oxo-1H-isochromene-3-carboxylic acid
sodiated HMDB0000026, spectral sim. = 929, #3
Struc. sim. = 0.40
Ureidopropionic acid
sodiated HMDB0040637, spectral sim. = 925, #4
Struc. sim. = 0.20
4-Hydroxy-6-methyl-3-(1-oxobutyl)-2H-pyran-2-one
sodiated HMDB0000679, spectral sim. = 920, #5
Struc. sim. = 1.00
Homocitrulline
sodiated HMDB0002031, spectral sim. = 918, #6
Struc. sim. = 0.35
Ureidoisobutyric acid
sodiated HMDB0031813, spectral sim. = 909, #7
Struc. sim. = 0.33
N-Carbamoyl-2-amino-2-(4-hydroxyphenyl)acetic
sodiated HMDB0128624, spectral sim. = 904, #8
Struc. sim. = 0.30
6-hydroxy-1-oxo-1H-isochromene-3-carboxylic acid
sodiated HMDB0128621, spectral sim. = 904, #9
Struc. sim. = 0.12
6-hydroxy-3-(hydroxymethyl)-1H-isochromen-1-one

91 protonated HMDB0000687

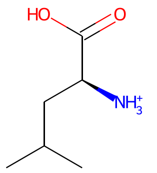

L-Leucine

Spectra of protonated HMDB0000687, spectral sim. = 917, #7

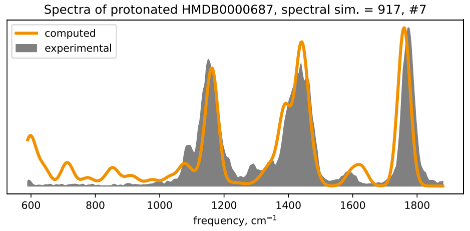

Structural similarity plot of protonated HMDB0000687

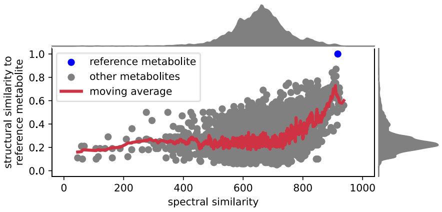

protonated HMDB0060385, spectral sim. = 937, #1

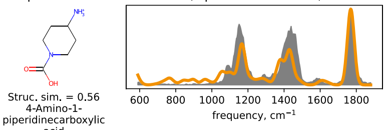

Struc. sim. = 0.56
4-Amino-1-piperidinecarboxylic acid

protonated HMDB0040329, spectral sim. = 930, #2

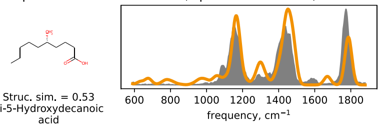

Struc. sim. = 0.53
xi-5-Hydroxydecanoic acid

protonated HMDB0003288, spectral sim. = 923, #3

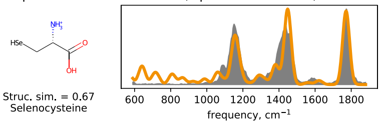

Struc. sim. = 0.67
Selenocysteine

protonated HMDB0006454, spectral sim. = 922, #4

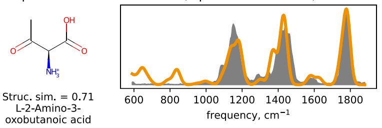

Struc. sim. = 0.71
L-2-Amino-3-oxobutanoic acid

protonated HMDB0003585, spectral sim. = 921, #5

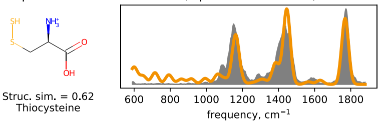

Struc. sim. = 0.62
Thiocysteine

protonated HMDB0034324, spectral sim. = 919, #6

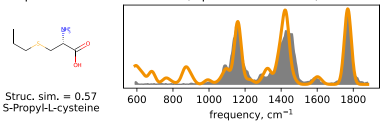

Struc. sim. = 0.57
S-Propyl-L-cysteine

protonated HMDB0000687, spectral sim. = 917, #7

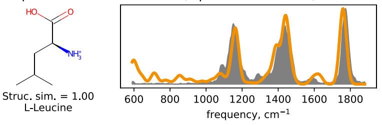

Struc. sim. = 1.00
L-Leucine

protonated HMDB0013716, spectral sim. = 916, #8

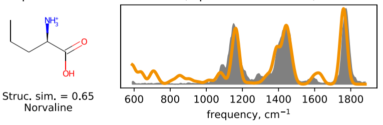

Struc. sim. = 0.65
Norvaline

protonated HMDB0002108, spectral sim. = 915, #9

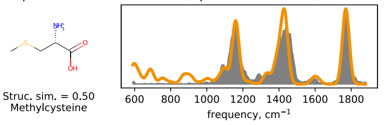

Struc. sim. = 0.50
Methylcysteine

92 sodiated HMDB0000687

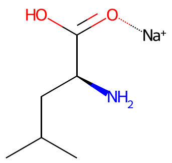

L-Leucine

Spectra of sodiated HMDB0000687, spectral sim. = 878, #242

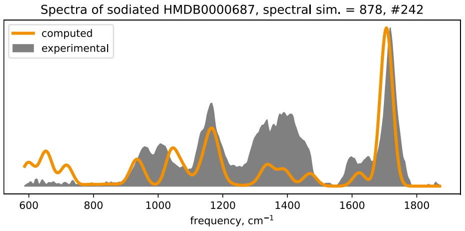

Structural similarity plot of sodiated HMDB0000687

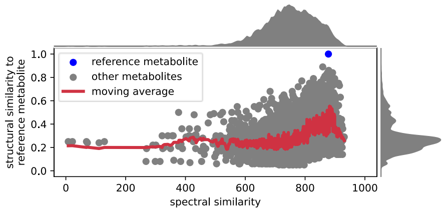

sodiated HMDB0029573, spectral sim. = 931, #1

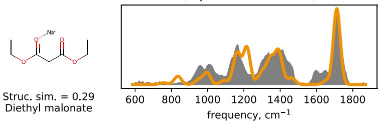

Struc. sim. = 0.29
Diethyl malonate

sodiated HMDB0034880, spectral sim. = 929, #2

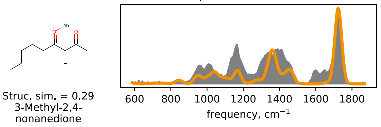

Struc. sim. = 0.29
3-Methyl-2,4-nonanedione

sodiated HMDB0040433, spectral sim. = 927, #3

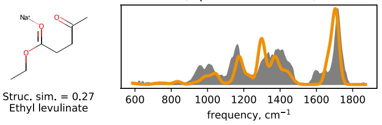

Struc. sim. = 0.27
Ethyl levulinate

sodiated HMDB0036396, spectral sim. = 926, #4

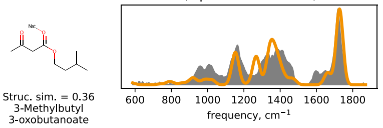

Struc. sim. = 0.36
3-Methylbutyl 3-oxobutanoate

sodiated HMDB0040447, spectral sim. = 926, #5

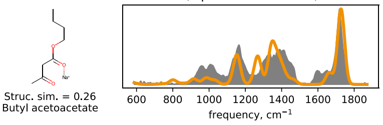

Struc. sim. = 0.26
Butyl acetoacetate

sodiated HMDB0128622, spectral sim. = 925, #6

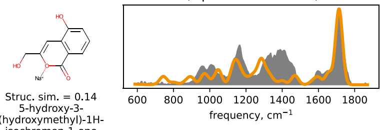

Struc. sim. = 0.14
5-hydroxy-3-(hydroxymethyl)-1H-isochromen-1-one

sodiated HMDB0036233, spectral sim. = 924, #7

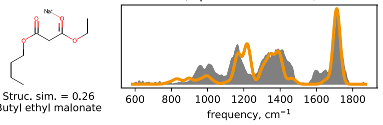

Struc. sim. = 0.26
Butyl ethyl malonate

sodiated HMDB0134032, spectral sim. = 924, #8

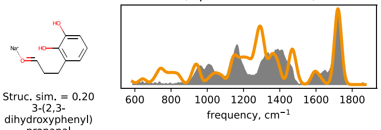

Struc. sim. = 0.20
3-(2,3-dihydroxyphenyl)propanal

sodiated HMDB0133490, spectral sim. = 923, #9

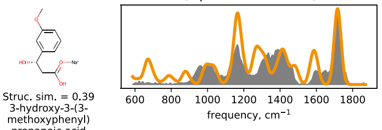

Struc. sim. = 0.39
3-hydroxy-3-(3-methoxyphenyl)propanoic acid

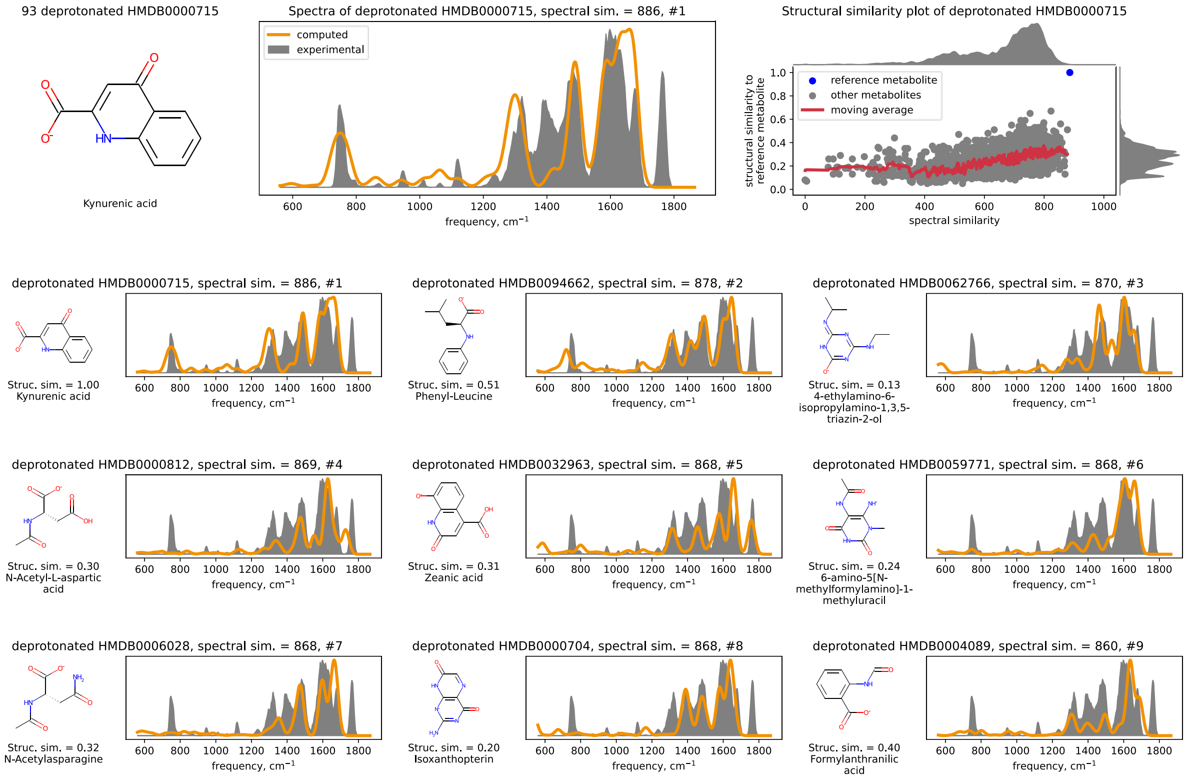
93 deprotonated HMDB0000715
Kynurenic acid
Spectra of deprotonated HMDB0000715, spectral sim. = 886, #1
computed
experimental
frequency, cm^{-1}
Structural similarity plot of deprotonated HMDB0000715
structural similarity to reference metabolite
reference metabolite
other metabolites
moving average
spectral similarity
deprotonated HMDB0000715, spectral sim. = 886, #1
Struc. sim. = 1.00
Kynurenic acid
deprotonated HMDB0094662, spectral sim. = 878, #2
Struc. sim. = 0.51
Phenyl-Leucine
deprotonated HMDB0062766, spectral sim. = 870, #3
Struc. sim. = 0.13
4-ethylamino-6-isopropylamino-1,3,5-triazin-2-ol
deprotonated HMDB0000812, spectral sim. = 869, #4
Struc. sim. = 0.30
N-Acetyl-L-aspartic acid
deprotonated HMDB0032963, spectral sim. = 868, #5
Struc. sim. = 0.31
Zeanic acid
deprotonated HMDB0059771, spectral sim. = 868, #6
Struc. sim. = 0.24
6-amino-5[N-methylformylamino]-1-methyluracil
deprotonated HMDB0006028, spectral sim. = 868, #7
Struc. sim. = 0.32
N-Acetylasparagine
deprotonated HMDB0000704, spectral sim. = 868, #8
Struc. sim. = 0.20
Isoxanthopterin
deprotonated HMDB0004089, spectral sim. = 860, #9
Struc. sim. = 0.40
Formylanthranilic acid

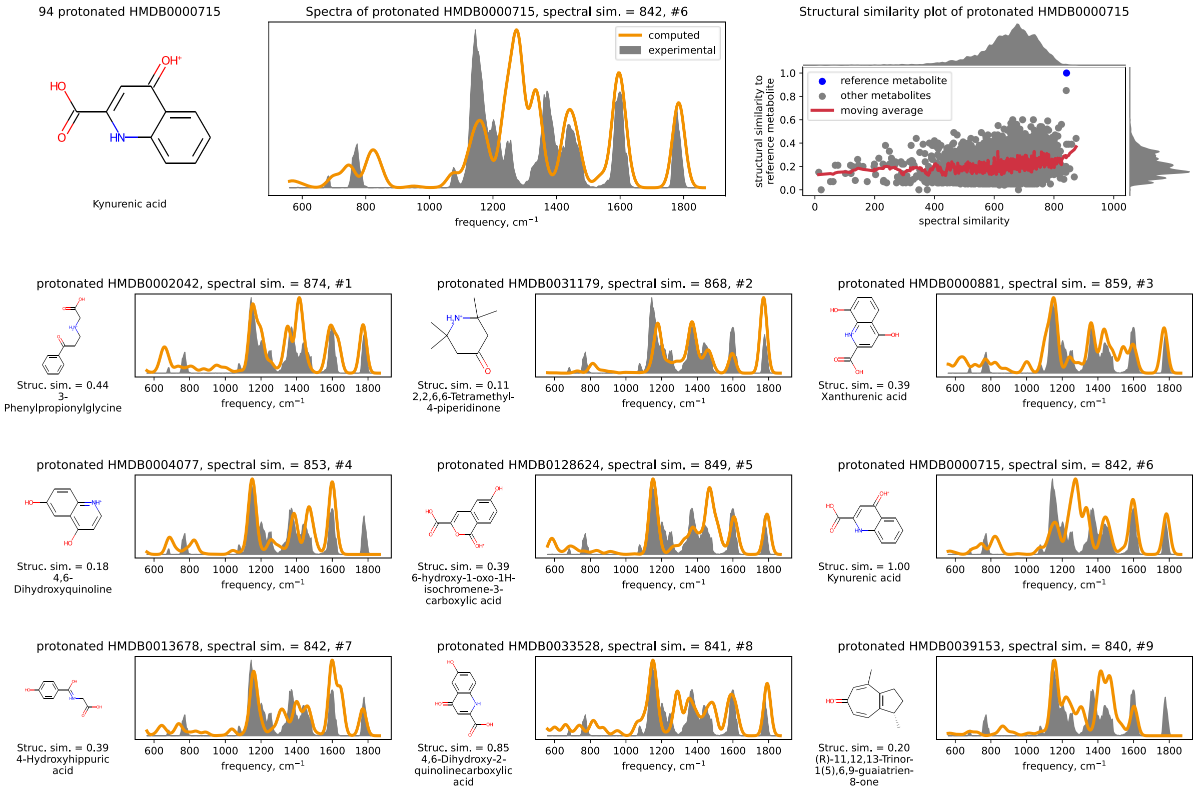
94 protonated HMDB0000715
Kynurenic acid
Spectra of protonated HMDB0000715, spectral sim. = 842, #6
computed
experimental
frequency, cm^{-1}
Structural similarity plot of protonated HMDB0000715
structural similarity to reference metabolite
reference metabolite
other metabolites
moving average
spectral similarity
protonated HMDB0002042, spectral sim. = 874, #1
Struc. sim. = 0.44
3-Phenylpropionylglycine
protonated HMDB0031179, spectral sim. = 868, #2
Struc. sim. = 0.11
2,2,6,6-Tetramethyl-4-piperidinone
protonated HMDB0000881, spectral sim. = 859, #3
Struc. sim. = 0.39
Xanthurenic acid
protonated HMDB0004077, spectral sim. = 853, #4
Struc. sim. = 0.18
4,6-Dihydroxyquinoline
protonated HMDB0128624, spectral sim. = 849, #5
Struc. sim. = 0.39
6-hydroxy-1-oxo-1H-isochromene-3-carboxylic acid
protonated HMDB0000715, spectral sim. = 842, #6
Struc. sim. = 1.00
Kynurenic acid
protonated HMDB0013678, spectral sim. = 842, #7
Struc. sim. = 0.39
4-Hydroxyhippuric acid
protonated HMDB0033528, spectral sim. = 841, #8
Struc. sim. = 0.85
4,6-Dihydroxy-2-quinolinecarboxylic acid
protonated HMDB0039153, spectral sim. = 840, #9
Struc. sim. = 0.20
(R)-11,12,13-Trinor-1(5),6,9-guaiatrien-8-one

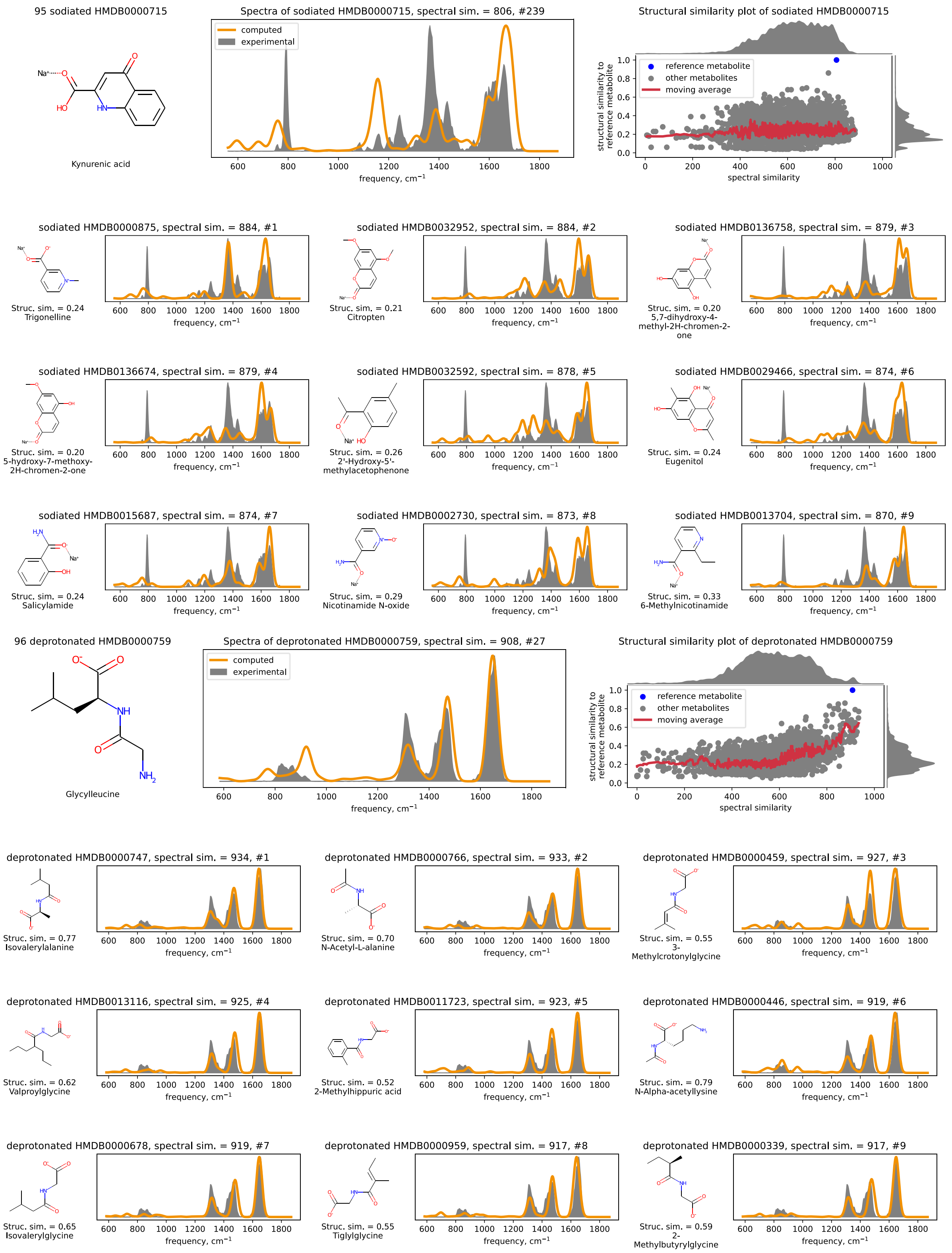
95 sodiated HMDB0000715
Kynurenic acid
Spectra of sodiated HMDB0000715, spectral sim. = 806, #239
computed
experimental
frequency, cm⁻¹
Structural similarity plot of sodiated HMDB0000715
reference metabolite
other metabolites
moving average
structural similarity to reference metabolite
spectral similarity
sodiated HMDB0000875, spectral sim. = 884, #1
Struc. sim. = 0.24
Trigonelline
sodiated HMDB0032952, spectral sim. = 884, #2
Struc. sim. = 0.21
Citropten
sodiated HMDB0136758, spectral sim. = 879, #3
Struc. sim. = 0.20
5,7-dihydroxy-4-methyl-2H-chromen-2-one
sodiated HMDB0136674, spectral sim. = 879, #4
Struc. sim. = 0.20
5-hydroxy-7-methoxy-2H-chromen-2-one
sodiated HMDB0032592, spectral sim. = 878, #5
Struc. sim. = 0.26
2'-Hydroxy-5'-methylacetophenone
sodiated HMDB0029466, spectral sim. = 874, #6
Struc. sim. = 0.24
Eugenitol
sodiated HMDB0015687, spectral sim. = 874, #7
Struc. sim. = 0.24
Salicylamide
sodiated HMDB0002730, spectral sim. = 873, #8
Struc. sim. = 0.29
Nicotinamide N-oxide
sodiated HMDB0013704, spectral sim. = 870, #9
Struc. sim. = 0.33
6-Methylnicotinamide
96 deprotonated HMDB0000759
Glycylleucine
Spectra of deprotonated HMDB0000759, spectral sim. = 908, #27
computed
experimental
frequency, cm⁻¹
Structural similarity plot of deprotonated HMDB0000759
reference metabolite
other metabolites
moving average
structural similarity to reference metabolite
spectral similarity
deprotonated HMDB0000747, spectral sim. = 934, #1
Struc. sim. = 0.77
Isovalerylalanine
deprotonated HMDB0000766, spectral sim. = 933, #2
Struc. sim. = 0.70
N-Acetyl-L-alanine
deprotonated HMDB0000459, spectral sim. = 927, #3
Struc. sim. = 0.55
3-Methylcrotonylglycine
deprotonated HMDB0013116, spectral sim. = 925, #4
Struc. sim. = 0.62
Valproylglycine
deprotonated HMDB0011723, spectral sim. = 923, #5
Struc. sim. = 0.52
2-Methylhippuric acid
deprotonated HMDB0000446, spectral sim. = 919, #6
Struc. sim. = 0.79
N-Alpha-acetyllysine
deprotonated HMDB0000678, spectral sim. = 919, #7
Struc. sim. = 0.65
Isovalerylglycine
deprotonated HMDB0000959, spectral sim. = 917, #8
Struc. sim. = 0.55
Tiglylglycine
deprotonated HMDB0000339, spectral sim. = 917, #9
Struc. sim. = 0.59
2-Methylbutyrylglycine

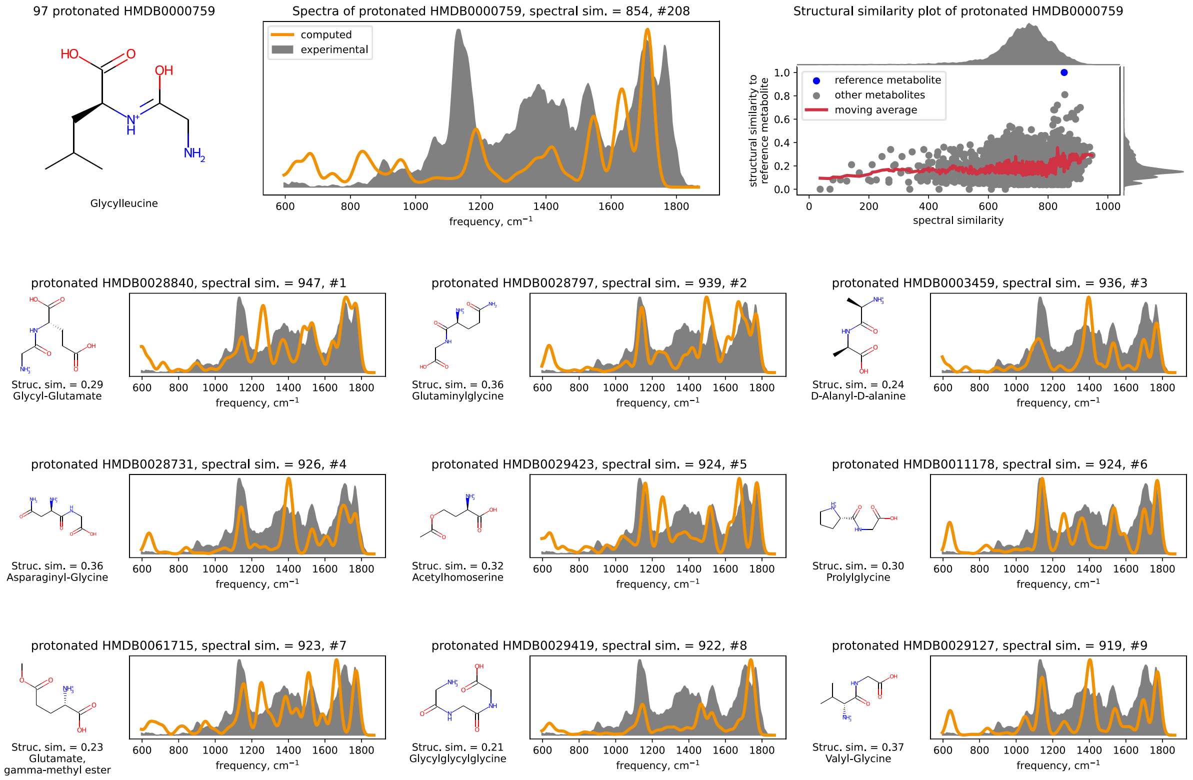
97 protonated HMDB0000759
Glycylleucine
Spectra of protonated HMDB0000759, spectral sim. = 854, #208
computed
experimental
frequency, cm^{-1}
Structural similarity plot of protonated HMDB0000759
reference metabolite
other metabolites
moving average
structural similarity to reference metabolite
spectral similarity
protonated HMDB0028840, spectral sim. = 947, #1
Struc. sim. = 0.29
Glycyl-Glutamate
protonated HMDB0028797, spectral sim. = 939, #2
Struc. sim. = 0.36
Glutaminylglycine
protonated HMDB0003459, spectral sim. = 936, #3
Struc. sim. = 0.24
D-Alanyl-D-alanine
protonated HMDB0028731, spectral sim. = 926, #4
Struc. sim. = 0.36
Asparaginyl-Glycine
protonated HMDB0029423, spectral sim. = 924, #5
Struc. sim. = 0.32
Acetylhomoserine
protonated HMDB0011178, spectral sim. = 924, #6
Struc. sim. = 0.30
Prolylglycine
protonated HMDB0061715, spectral sim. = 923, #7
Struc. sim. = 0.23
Glutamate, gamma-methyl ester
protonated HMDB0029419, spectral sim. = 922, #8
Struc. sim. = 0.21
Glycylglycylglycine
protonated HMDB0029127, spectral sim. = 919, #9
Struc. sim. = 0.37
Valyl-Glycine

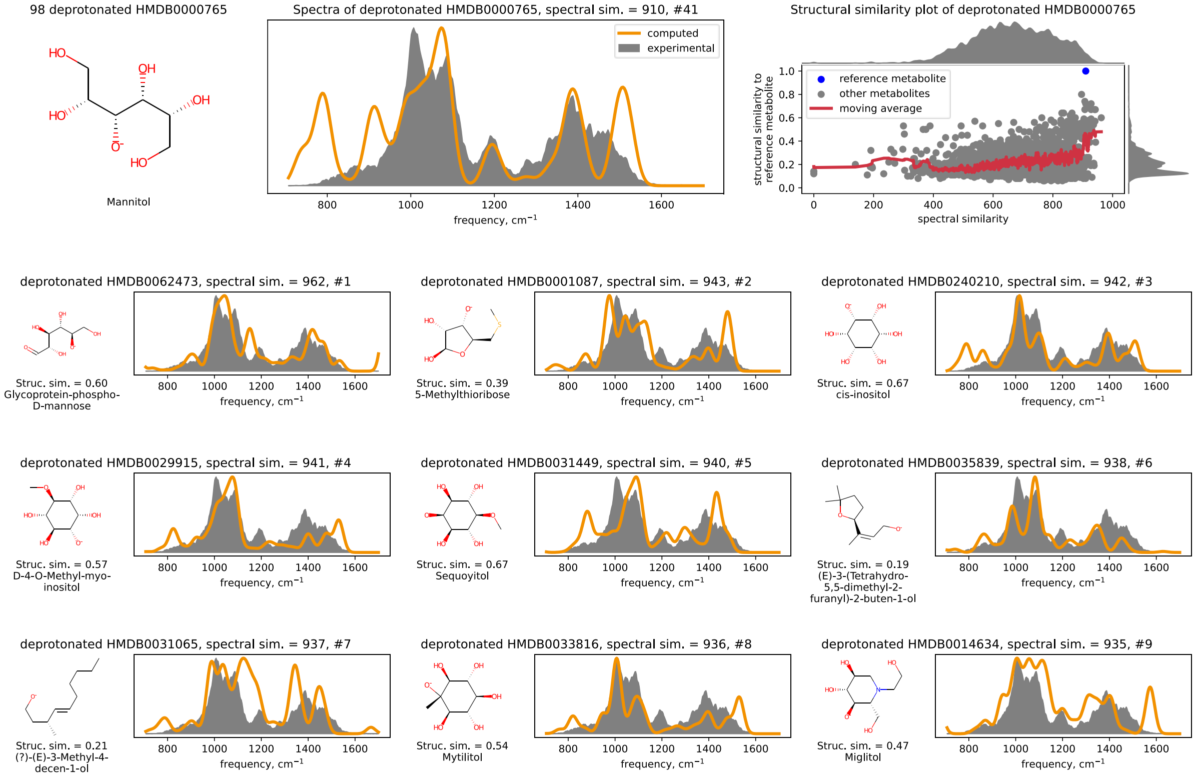
98 deprotonated HMDB0000765
Mannitol
Spectra of deprotonated HMDB0000765, spectral sim. = 910, #41
computed
experimental
frequency, cm^{-1}
Structural similarity plot of deprotonated HMDB0000765
reference metabolite
other metabolites
moving average
structural similarity to reference metabolite
spectral similarity
deprotonated HMDB0062473, spectral sim. = 962, #1
Struc. sim. = 0.60
Glycoprotein-phospho-D-mannose
deprotonated HMDB0001087, spectral sim. = 943, #2
Struc. sim. = 0.39
5-Methylthioribose
deprotonated HMDB0240210, spectral sim. = 942, #3
Struc. sim. = 0.67
cis-inositol
deprotonated HMDB0029915, spectral sim. = 941, #4
Struc. sim. = 0.57
D-4-O-Methyl-myo-inositol
deprotonated HMDB0031449, spectral sim. = 940, #5
Struc. sim. = 0.67
Sequoyitol
deprotonated HMDB0035839, spectral sim. = 938, #6
Struc. sim. = 0.19
(E)-3-(Tetrahydro-5,5-dimethyl-2-furanyl)-2-buten-1-ol
deprotonated HMDB0031065, spectral sim. = 937, #7
Struc. sim. = 0.21
(?)-(E)-3-Methyl-4-decen-1-ol
deprotonated HMDB0033816, spectral sim. = 936, #8
Struc. sim. = 0.54
Mytilitol
deprotonated HMDB0014634, spectral sim. = 935, #9
Struc. sim. = 0.47
Miglitol

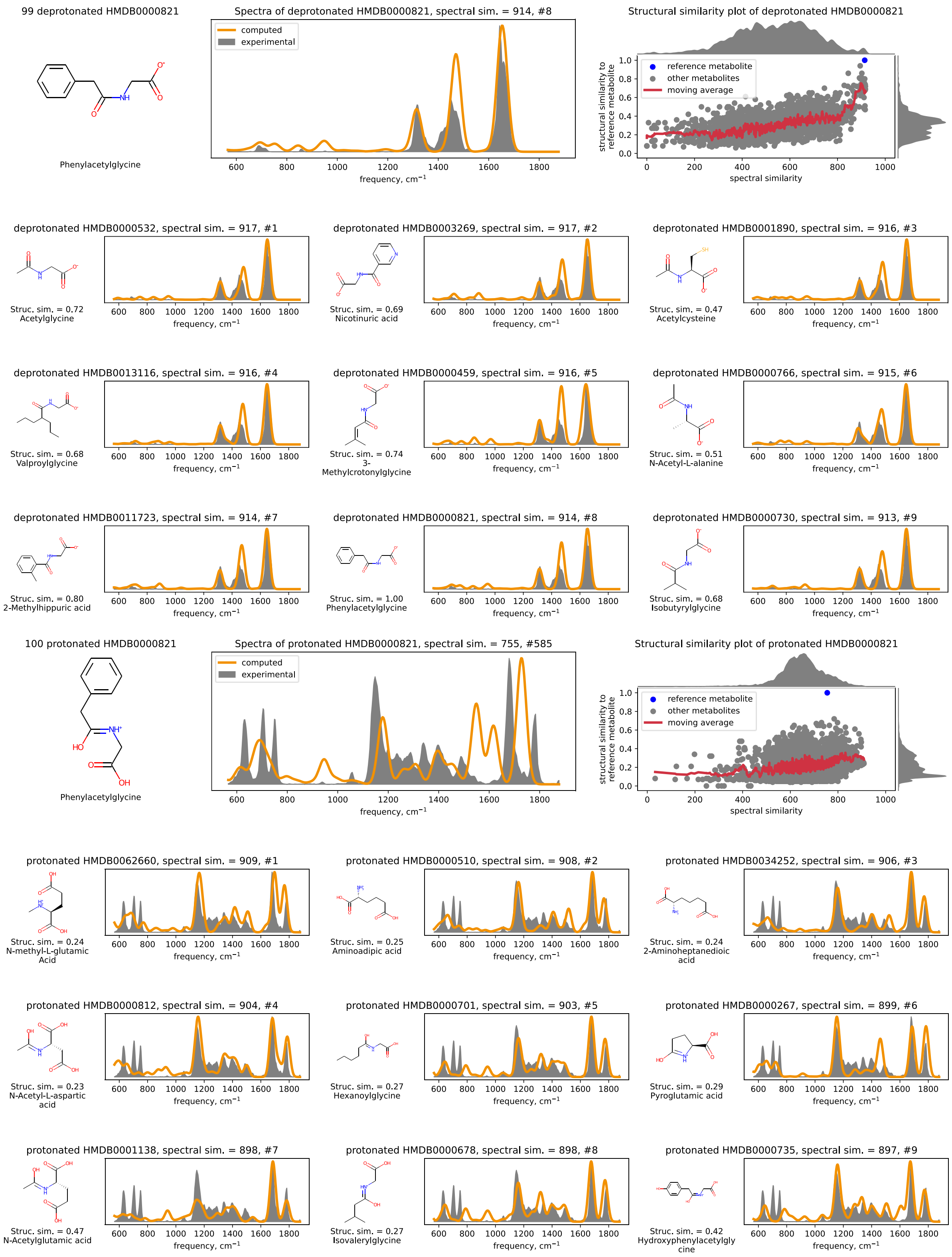

99 deprotonated HMDB0000821
Phenylacetylglycine
Spectra of deprotonated HMDB0000821, spectral sim. = 914, #8
computed
experimental
frequency, cm⁻¹
Structural similarity plot of deprotonated HMDB0000821
reference metabolite
other metabolites
moving average
structural similarity to reference metabolite
spectral similarity
deprotonated HMDB0000532, spectral sim. = 917, #1
Struc. sim. = 0.72
Acetylglycine
deprotonated HMDB0003269, spectral sim. = 917, #2
Struc. sim. = 0.69
Nicotinuric acid
deprotonated HMDB0001890, spectral sim. = 916, #3
Struc. sim. = 0.47
Acetylcysteine
deprotonated HMDB0013116, spectral sim. = 916, #4
Struc. sim. = 0.68
Valproylglycine
deprotonated HMDB0000459, spectral sim. = 916, #5
Struc. sim. = 0.74
3-Methylcrotonylglycine
deprotonated HMDB0000766, spectral sim. = 915, #6
Struc. sim. = 0.51
N-Acetyl-L-alanine
deprotonated HMDB0011723, spectral sim. = 914, #7
Struc. sim. = 0.80
2-Methylhippuric acid
deprotonated HMDB0000821, spectral sim. = 914, #8
Struc. sim. = 1.00
Phenylacetylglycine
deprotonated HMDB0000730, spectral sim. = 913, #9
Struc. sim. = 0.68
Isobutyrylglycine
100 protonated HMDB0000821
Phenylacetylglycine
Spectra of protonated HMDB0000821, spectral sim. = 755, #585
computed
experimental
frequency, cm⁻¹
Structural similarity plot of protonated HMDB0000821
reference metabolite
other metabolites
moving average
structural similarity to reference metabolite
spectral similarity
protonated HMDB0062660, spectral sim. = 909, #1
Struc. sim. = 0.24
N-methyl-L-glutamic Acid
protonated HMDB0000510, spectral sim. = 908, #2
Struc. sim. = 0.25
Aminoadipic acid
protonated HMDB0034252, spectral sim. = 906, #3
Struc. sim. = 0.24
2-Aminoheptanedioic acid
protonated HMDB0000812, spectral sim. = 904, #4
Struc. sim. = 0.23
N-Acetyl-L-aspartic acid
protonated HMDB0000701, spectral sim. = 903, #5
Struc. sim. = 0.27
Hexanoylglycine
protonated HMDB0000267, spectral sim. = 899, #6
Struc. sim. = 0.29
Pyroglutamic acid
protonated HMDB0001138, spectral sim. = 898, #7
Struc. sim. = 0.47
N-Acetylglutamic acid
protonated HMDB0000678, spectral sim. = 898, #8
Struc. sim. = 0.27
Isovalerylglycine
protonated HMDB0000735, spectral sim. = 897, #9
Struc. sim. = 0.42
Hydroxyphenylacetylglycine

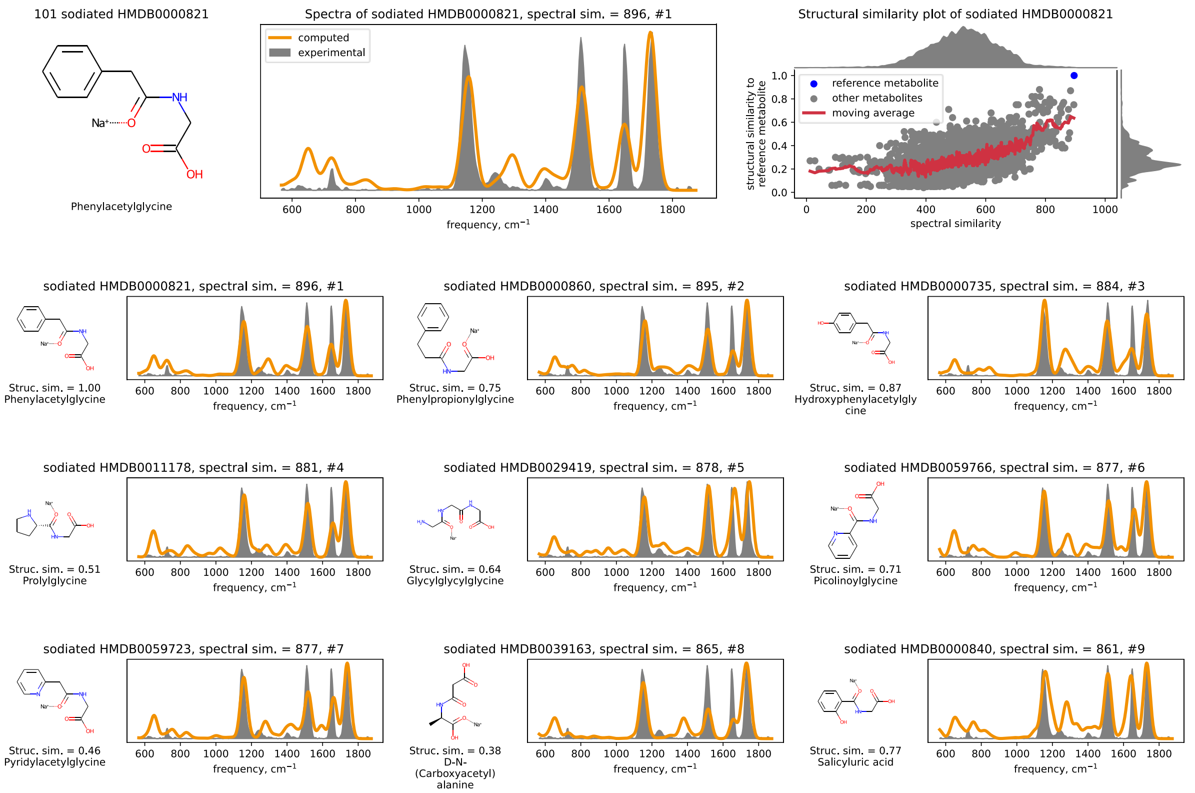

101 sodiated HMDB0000821
Phenylacetylglycine
Spectra of sodiated HMDB0000821, spectral sim. = 896, #1
computed
experimental
frequency, cm⁻¹
Structural similarity plot of sodiated HMDB0000821
reference metabolite
other metabolites
moving average
structural similarity to reference metabolite
spectral similarity
sodiated HMDB0000821, spectral sim. = 896, #1
Struc. sim. = 1.00
Phenylacetylglycine
sodiated HMDB0000860, spectral sim. = 895, #2
Struc. sim. = 0.75
Phenylpropionylglycine
sodiated HMDB0000735, spectral sim. = 884, #3
Struc. sim. = 0.87
Hydroxyphenylacetylglycine
sodiated HMDB0011178, spectral sim. = 881, #4
Struc. sim. = 0.51
Prolylglycine
sodiated HMDB0029419, spectral sim. = 878, #5
Struc. sim. = 0.64
Glycylglycylglycine
sodiated HMDB0059766, spectral sim. = 877, #6
Struc. sim. = 0.71
Picolinoylglycine
sodiated HMDB0059723, spectral sim. = 877, #7
Struc. sim. = 0.46
Pyridylacetylglycine
sodiated HMDB0039163, spectral sim. = 865, #8
Struc. sim. = 0.38
D-N-(Carboxyacetyl)alanine
sodiated HMDB0000840, spectral sim. = 861, #9
Struc. sim. = 0.77
Salicyluric acid

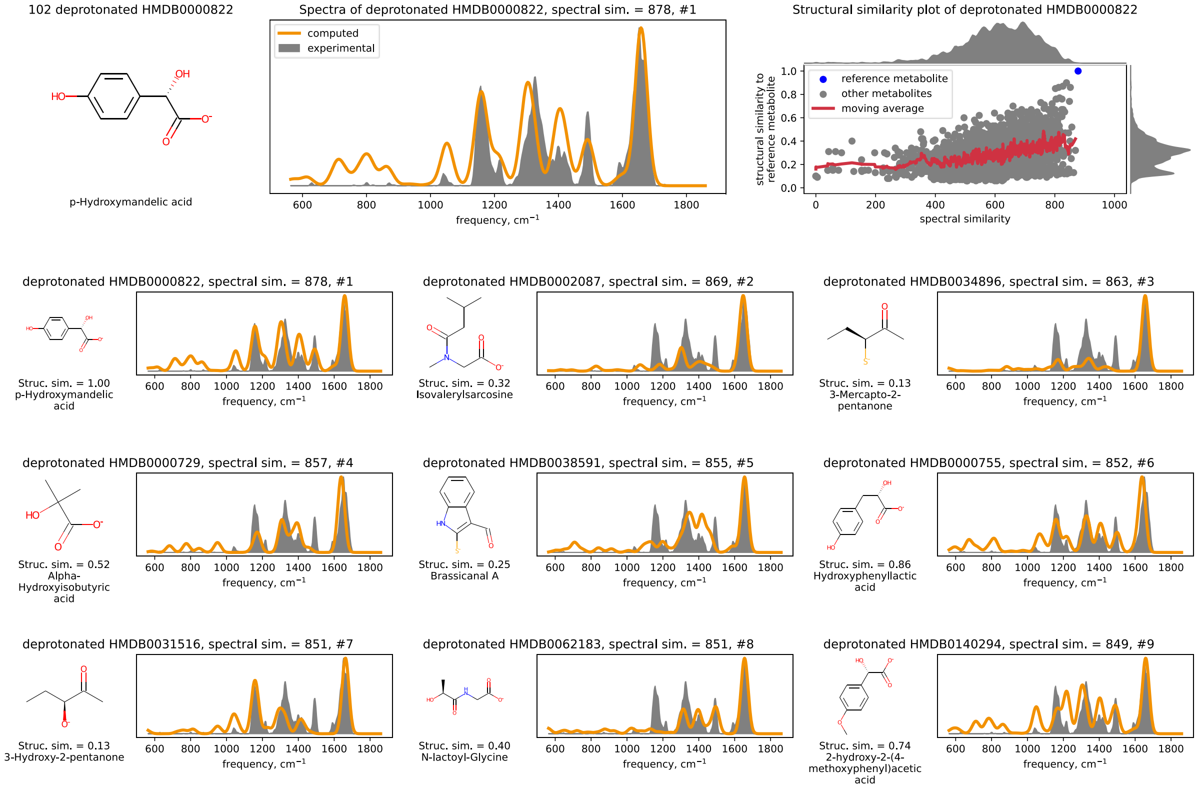

102 deprotonated HMDB0000822
p-Hydroxymandelic acid
Spectra of deprotonated HMDB0000822, spectral sim. = 878, #1
computed
experimental
frequency, cm⁻¹
Structural similarity plot of deprotonated HMDB0000822
reference metabolite
other metabolites
moving average
structural similarity to reference metabolite
spectral similarity
deprotonated HMDB0000822, spectral sim. = 878, #1
Struc. sim. = 1.00
p-Hydroxymandelic acid
deprotonated HMDB0002087, spectral sim. = 869, #2
Struc. sim. = 0.32
Isovalerylsarcosine
deprotonated HMDB0034896, spectral sim. = 863, #3
Struc. sim. = 0.13
3-Mercapto-2-pentanone
deprotonated HMDB0000729, spectral sim. = 857, #4
Struc. sim. = 0.52
Alpha-Hydroxyisobutyric acid
deprotonated HMDB0038591, spectral sim. = 855, #5
Struc. sim. = 0.25
Brassicanal A
deprotonated HMDB0000755, spectral sim. = 852, #6
Struc. sim. = 0.86
Hydroxyphenyllactic acid
deprotonated HMDB0031516, spectral sim. = 851, #7
Struc. sim. = 0.13
3-Hydroxy-2-pentanone
deprotonated HMDB0062183, spectral sim. = 851, #8
Struc. sim. = 0.40
N-lactoyl-Glycine
deprotonated HMDB0140294, spectral sim. = 849, #9
Struc. sim. = 0.74
2-hydroxy-2-(4-methoxyphenyl)acetic acid

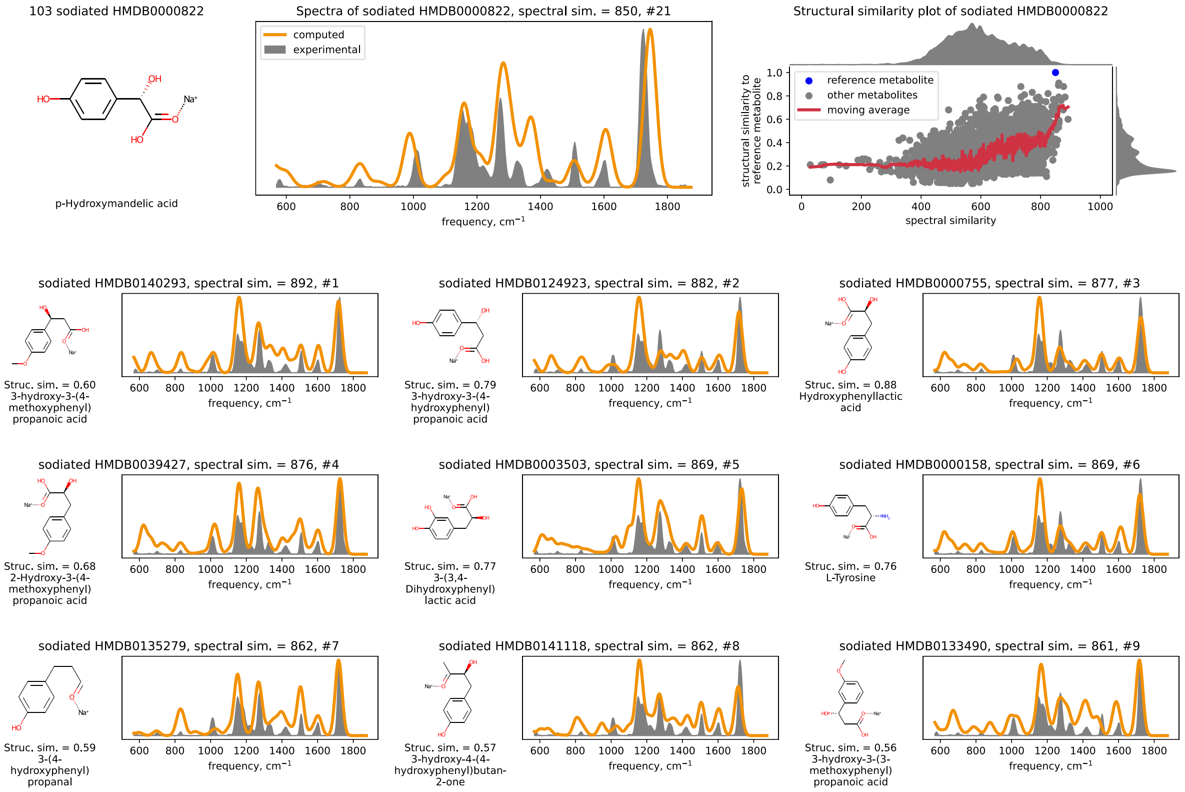

103 sodiated HMDB0000822
p-Hydroxymandelic acid
Spectra of sodiated HMDB0000822, spectral sim. = 850, #21
computed
experimental
frequency, cm^{-1}
Structural similarity plot of sodiated HMDB0000822
reference metabolite
other metabolites
moving average
structural similarity to reference metabolite
spectral similarity
sodiated HMDB0140293, spectral sim. = 892, #1
Struc. sim. = 0.60
3-hydroxy-3-(4-methoxyphenyl) propanoic acid
sodiated HMDB0124923, spectral sim. = 882, #2
Struc. sim. = 0.79
3-hydroxy-3-(4-hydroxyphenyl) propanoic acid
sodiated HMDB0000755, spectral sim. = 877, #3
Struc. sim. = 0.88
Hydroxyphenyllactic acid
sodiated HMDB0039427, spectral sim. = 876, #4
Struc. sim. = 0.68
2-Hydroxy-3-(4-methoxyphenyl) propanoic acid
sodiated HMDB0003503, spectral sim. = 869, #5
Struc. sim. = 0.77
3-(3,4-Dihydroxyphenyl) lactic acid
sodiated HMDB0000158, spectral sim. = 869, #6
Struc. sim. = 0.76
L-Tyrosine
sodiated HMDB0135279, spectral sim. = 862, #7
Struc. sim. = 0.59
3-(4-hydroxyphenyl) propanal
sodiated HMDB0141118, spectral sim. = 862, #8
Struc. sim. = 0.57
3-hydroxy-4-(4-hydroxyphenyl)butan-2-one
sodiated HMDB0133490, spectral sim. = 861, #9
Struc. sim. = 0.56
3-hydroxy-3-(3-methoxyphenyl) propanoic acid

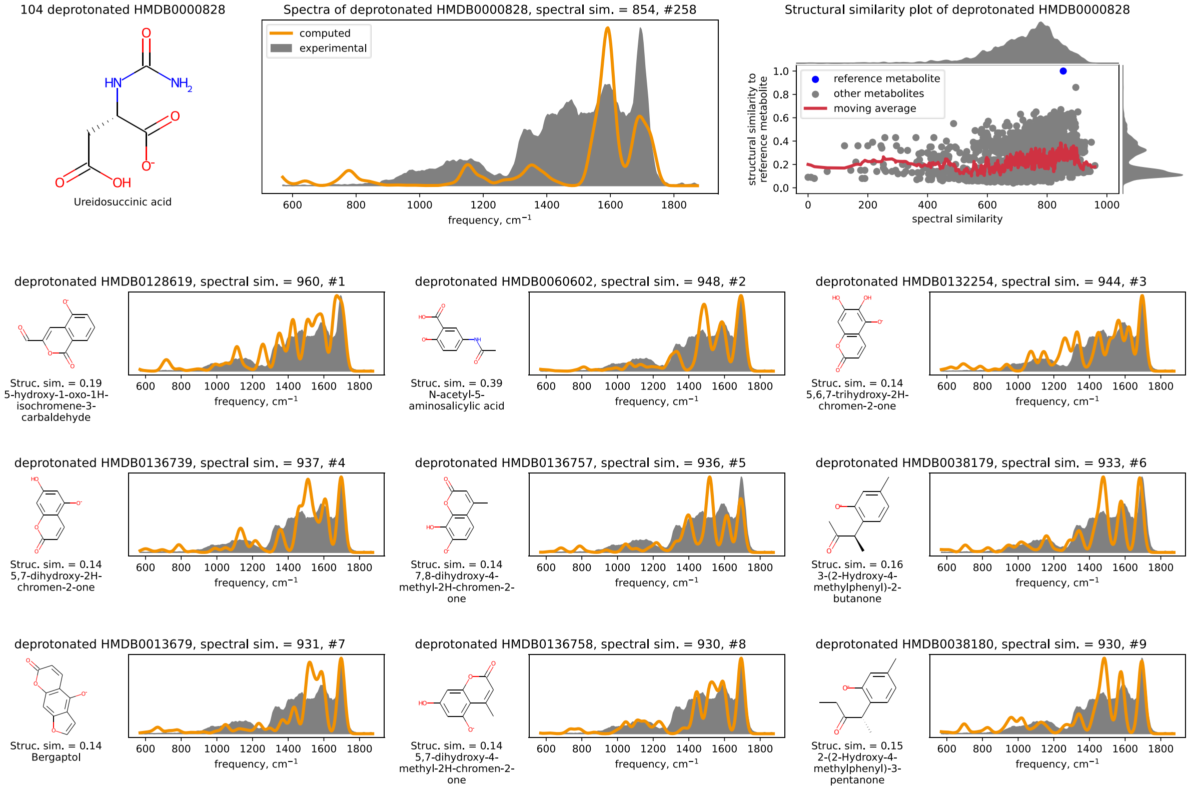

104 deprotonated HMDB0000828
Ureidosuccinic acid
Spectra of deprotonated HMDB0000828, spectral sim. = 854, #258
computed
experimental
frequency, cm^{-1}
Structural similarity plot of deprotonated HMDB0000828
reference metabolite
other metabolites
moving average
structural similarity to reference metabolite
spectral similarity
deprotonated HMDB0128619, spectral sim. = 960, #1
Struc. sim. = 0.19
5-hydroxy-1-oxo-1H-isochromene-3-carbaldehyde
deprotonated HMDB0060602, spectral sim. = 948, #2
Struc. sim. = 0.39
N-acetyl-5-aminosalicylic acid
deprotonated HMDB0132254, spectral sim. = 944, #3
Struc. sim. = 0.14
5,6,7-trihydroxy-2H-chromen-2-one
deprotonated HMDB0136739, spectral sim. = 937, #4
Struc. sim. = 0.14
5,7-dihydroxy-2H-chromen-2-one
deprotonated HMDB0136757, spectral sim. = 936, #5
Struc. sim. = 0.14
7,8-dihydroxy-4-methyl-2H-chromen-2-one
deprotonated HMDB0038179, spectral sim. = 933, #6
Struc. sim. = 0.16
3-(2-Hydroxy-4-methylphenyl)-2-butanone
deprotonated HMDB0013679, spectral sim. = 931, #7
Struc. sim. = 0.14
Bergaptol
deprotonated HMDB0136758, spectral sim. = 930, #8
Struc. sim. = 0.14
5,7-dihydroxy-4-methyl-2H-chromen-2-one
deprotonated HMDB0038180, spectral sim. = 930, #9
Struc. sim. = 0.15
2-(2-Hydroxy-4-methylphenyl)-3-pentanone

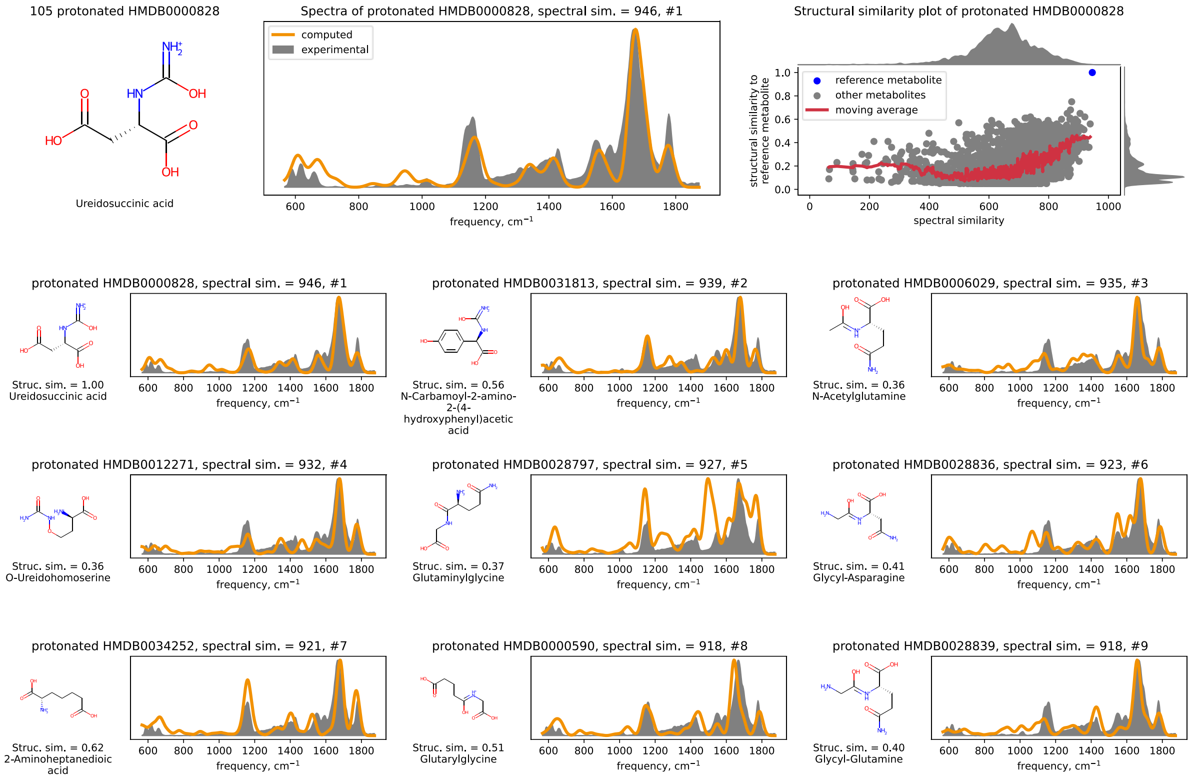
105 protonated HMDB0000828
Ureidosuccinic acid
Spectra of protonated HMDB0000828, spectral sim. = 946, #1
computed
experimental
frequency, cm⁻¹
Structural similarity plot of protonated HMDB0000828
reference metabolite
other metabolites
moving average
structural similarity to reference metabolite
spectral similarity
protonated HMDB0000828, spectral sim. = 946, #1
Struc. sim. = 1.00
Ureidosuccinic acid
protonated HMDB0031813, spectral sim. = 939, #2
Struc. sim. = 0.56
N-Carbamoyl-2-amino-2-(4-hydroxyphenyl)acetic acid
protonated HMDB0006029, spectral sim. = 935, #3
Struc. sim. = 0.36
N-Acetylglutamine
protonated HMDB0012271, spectral sim. = 932, #4
Struc. sim. = 0.36
O-Ureidohomoserine
protonated HMDB0028797, spectral sim. = 927, #5
Struc. sim. = 0.37
Glutaminylglycine
protonated HMDB0028836, spectral sim. = 923, #6
Struc. sim. = 0.41
Glycyl-Asparagine
protonated HMDB0034252, spectral sim. = 921, #7
Struc. sim. = 0.62
2-Aminoheptanedioic acid
protonated HMDB0000590, spectral sim. = 918, #8
Struc. sim. = 0.51
Glutarylglycine
protonated HMDB0028839, spectral sim. = 918, #9
Struc. sim. = 0.40
Glycyl-Glutamine
frequency, cm⁻¹

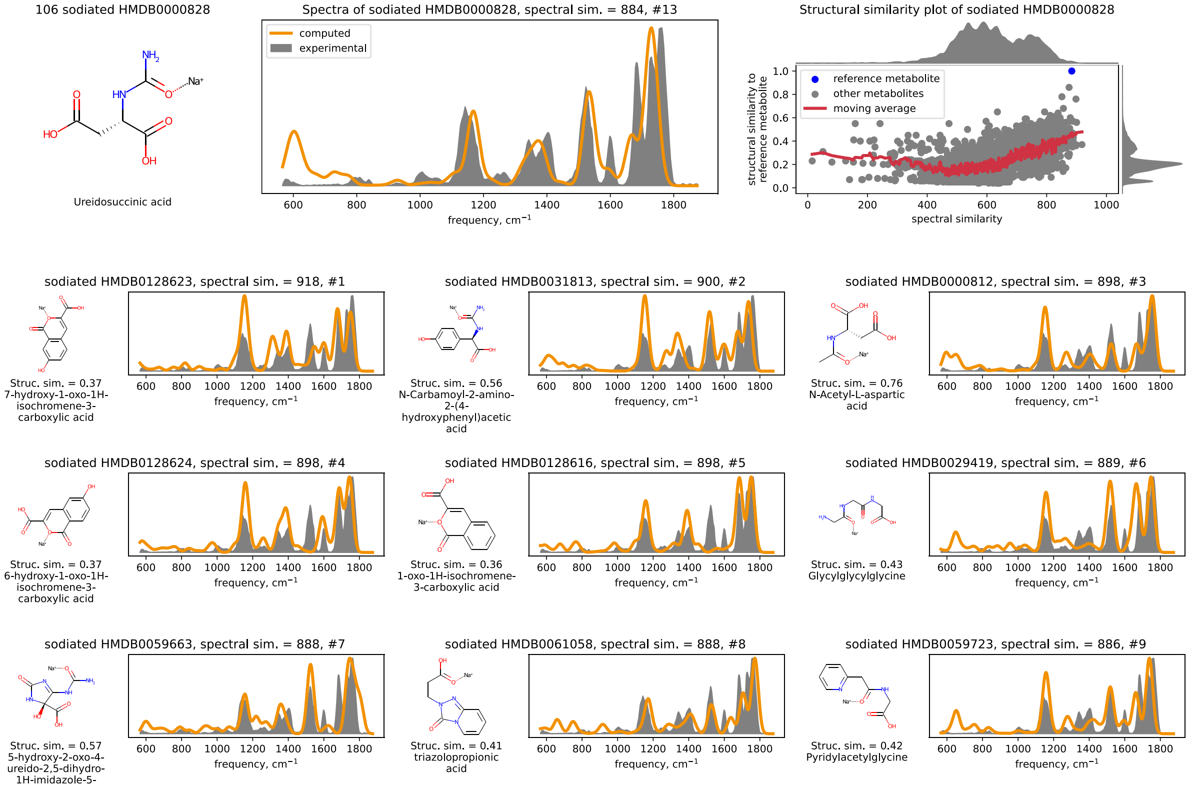
106 sodiated HMDB0000828
Ureidosuccinic acid
Spectra of sodiated HMDB0000828, spectral sim. = 884, #13
computed
experimental
frequency, cm⁻¹
Structural similarity plot of sodiated HMDB0000828
reference metabolite
other metabolites
moving average
structural similarity to reference metabolite
spectral similarity
sodiated HMDB0128623, spectral sim. = 918, #1
Struc. sim. = 0.37
7-hydroxy-1-oxo-1H-isochromene-3-carboxylic acid
sodiated HMDB0031813, spectral sim. = 900, #2
Struc. sim. = 0.56
N-Carbamoyl-2-amino-2-(4-hydroxyphenyl)acetic acid
sodiated HMDB0000812, spectral sim. = 898, #3
Struc. sim. = 0.76
N-Acetyl-L-aspartic acid
sodiated HMDB0128624, spectral sim. = 898, #4
Struc. sim. = 0.37
6-hydroxy-1-oxo-1H-isochromene-3-carboxylic acid
sodiated HMDB0128616, spectral sim. = 898, #5
Struc. sim. = 0.36
1-oxo-1H-isochromene-3-carboxylic acid
sodiated HMDB0029419, spectral sim. = 889, #6
Struc. sim. = 0.43
Glycylglycylglycine
sodiated HMDB0059663, spectral sim. = 888, #7
Struc. sim. = 0.57
5-hydroxy-2-oxo-4-ureido-2,5-dihydro-1H-imidazole-5-
sodiated HMDB0061058, spectral sim. = 888, #8
Struc. sim. = 0.41
triazolopropionic acid
sodiated HMDB0059723, spectral sim. = 886, #9
Struc. sim. = 0.42
Pyridylacetylglycine
frequency, cm⁻¹

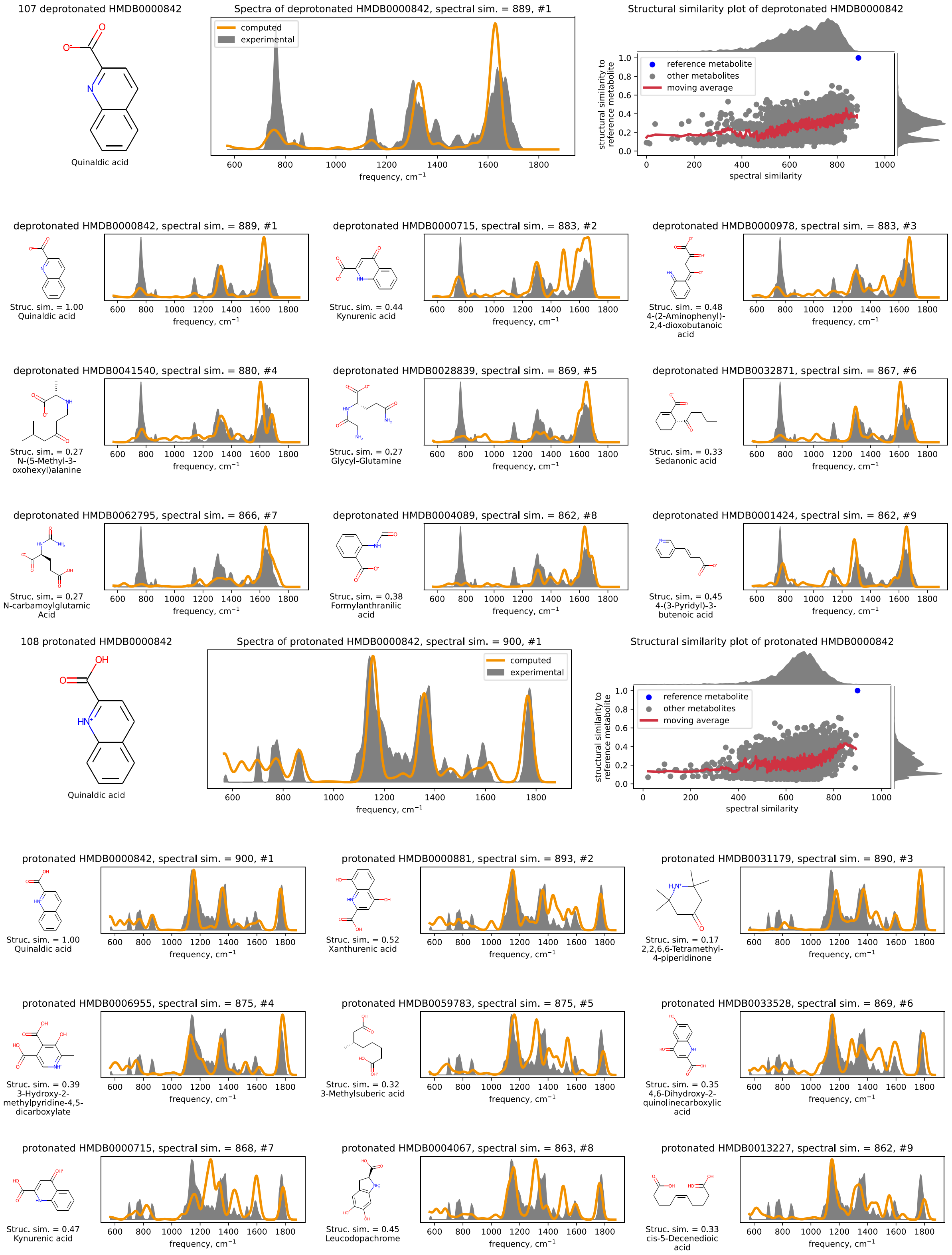
107 deprotonated HMDB0000842
Quinaldic acid
Spectra of deprotonated HMDB0000842, spectral sim. = 889, #1
computed
experimental
frequency, cm⁻¹
Structural similarity plot of deprotonated HMDB0000842
reference metabolite
other metabolites
moving average
structural similarity to reference metabolite
spectral similarity
deprotonated HMDB0000842, spectral sim. = 889, #1
Struc. sim. = 1.00
Quinaldic acid
deprotonated HMDB0000715, spectral sim. = 883, #2
Struc. sim. = 0.44
Kynurenic acid
deprotonated HMDB0000978, spectral sim. = 883, #3
Struc. sim. = 0.48
4-(2-Aminophenyl)-2,4-dioxobutanoic acid
deprotonated HMDB0041540, spectral sim. = 880, #4
Struc. sim. = 0.27
N-(5-Methyl-3-oxohexyl)alanine
deprotonated HMDB0028839, spectral sim. = 869, #5
Struc. sim. = 0.27
Glycyl-Glutamine
deprotonated HMDB0032871, spectral sim. = 867, #6
Struc. sim. = 0.33
Sedanonic acid
deprotonated HMDB0062795, spectral sim. = 866, #7
Struc. sim. = 0.27
N-carbamoylglutamic Acid
deprotonated HMDB0004089, spectral sim. = 862, #8
Struc. sim. = 0.38
Formylanthranilic acid
deprotonated HMDB0001424, spectral sim. = 862, #9
Struc. sim. = 0.45
4-(3-Pyridyl)-3-butenoic acid
108 protonated HMDB0000842
Quinaldic acid
Spectra of protonated HMDB0000842, spectral sim. = 900, #1
computed
experimental
frequency, cm⁻¹
Structural similarity plot of protonated HMDB0000842
reference metabolite
other metabolites
moving average
structural similarity to reference metabolite
spectral similarity
protonated HMDB0000842, spectral sim. = 900, #1
Struc. sim. = 1.00
Quinaldic acid
protonated HMDB0000881, spectral sim. = 893, #2
Struc. sim. = 0.52
Xanthurenic acid
protonated HMDB0031179, spectral sim. = 890, #3
Struc. sim. = 0.17
2,2,6,6-Tetramethyl-4-piperidinone
protonated HMDB0006955, spectral sim. = 875, #4
Struc. sim. = 0.39
3-Hydroxy-2-methylpyridine-4,5-dicarboxylate
protonated HMDB0059783, spectral sim. = 875, #5
Struc. sim. = 0.32
3-Methylsuberic acid
protonated HMDB0033528, spectral sim. = 869, #6
Struc. sim. = 0.35
4,6-Dihydroxy-2-quinolinecarboxylic acid
protonated HMDB0000715, spectral sim. = 868, #7
Struc. sim. = 0.47
Kynurenic acid
protonated HMDB0004067, spectral sim. = 863, #8
Struc. sim. = 0.45
Leucodopachrome
protonated HMDB0013227, spectral sim. = 862, #9
Struc. sim. = 0.33
cis-5-Decenedioic acid
frequency, cm⁻¹

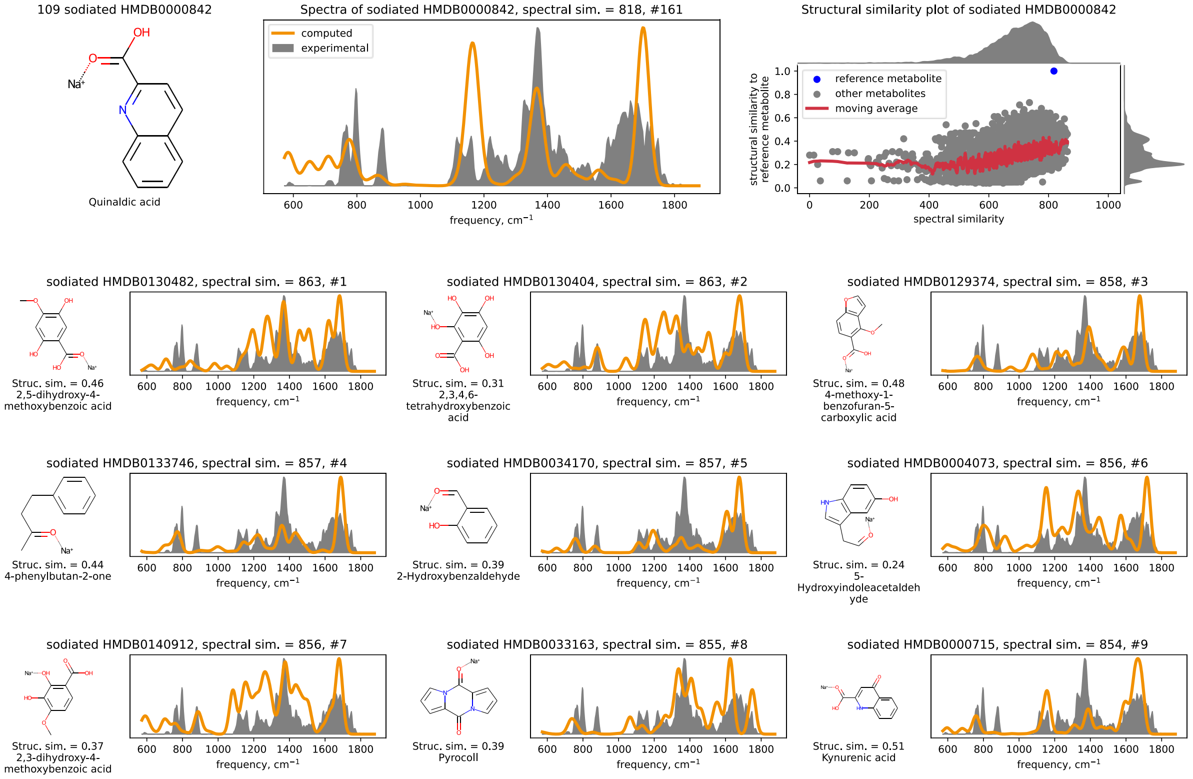
109 sodiated HMDB0000842
Quinaldic acid
Spectra of sodiated HMDB0000842, spectral sim. = 818, #161
computed
experimental
frequency, cm⁻¹
Structural similarity plot of sodiated HMDB0000842
reference metabolite
other metabolites
moving average
structural similarity to reference metabolite
spectral similarity
sodiated HMDB0130482, spectral sim. = 863, #1
Struc. sim. = 0.46
2,5-dihydroxy-4-methoxybenzoic acid
sodiated HMDB0130404, spectral sim. = 863, #2
Struc. sim. = 0.31
2,3,4,6-tetrahydroxybenzoic acid
sodiated HMDB0129374, spectral sim. = 858, #3
Struc. sim. = 0.48
4-methoxy-1-benzofuran-5-carboxylic acid
sodiated HMDB0133746, spectral sim. = 857, #4
Struc. sim. = 0.44
4-phenylbutan-2-one
sodiated HMDB0034170, spectral sim. = 857, #5
Struc. sim. = 0.39
2-Hydroxybenzaldehyde
sodiated HMDB0004073, spectral sim. = 856, #6
Struc. sim. = 0.24
5-Hydroxyindoleacetaldehyde
sodiated HMDB0140912, spectral sim. = 856, #7
Struc. sim. = 0.37
2,3-dihydroxy-4-methoxybenzoic acid
sodiated HMDB0033163, spectral sim. = 855, #8
Struc. sim. = 0.39
Pyrocoll
sodiated HMDB0000715, spectral sim. = 854, #9
Struc. sim. = 0.51
Kynurenic acid

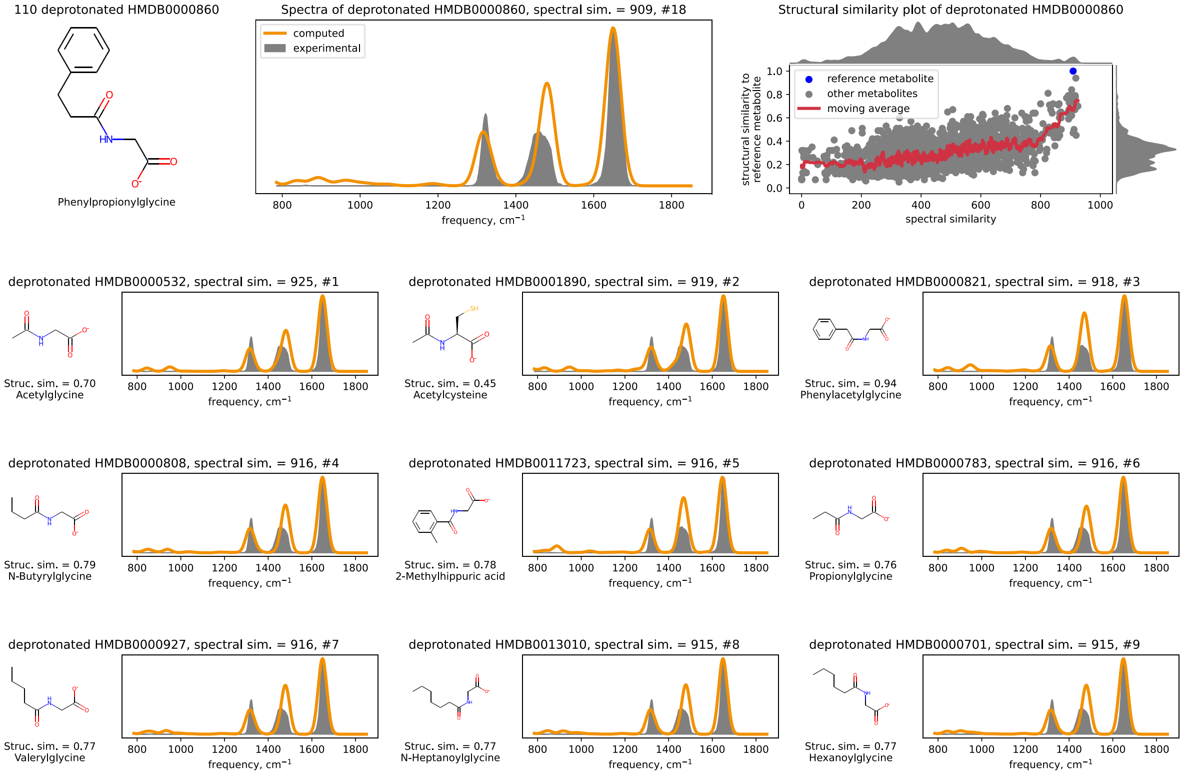
110 deprotonated HMDB0000860
Phenylpropionylglycine
Spectra of deprotonated HMDB0000860, spectral sim. = 909, #18
computed
experimental
frequency, cm⁻¹
Structural similarity plot of deprotonated HMDB0000860
reference metabolite
other metabolites
moving average
structural similarity to reference metabolite
spectral similarity
deprotonated HMDB0000532, spectral sim. = 925, #1
Struc. sim. = 0.70
Acetylglycine
deprotonated HMDB0001890, spectral sim. = 919, #2
Struc. sim. = 0.45
Acetylcysteine
deprotonated HMDB0000821, spectral sim. = 918, #3
Struc. sim. = 0.94
Phenylacetylglycine
deprotonated HMDB0000808, spectral sim. = 916, #4
Struc. sim. = 0.79
N-Butyrylglycine
deprotonated HMDB0011723, spectral sim. = 916, #5
Struc. sim. = 0.78
2-Methylhippuric acid
deprotonated HMDB0000783, spectral sim. = 916, #6
Struc. sim. = 0.76
Propionylglycine
deprotonated HMDB0000927, spectral sim. = 916, #7
Struc. sim. = 0.77
Valerylglycine
deprotonated HMDB0013010, spectral sim. = 915, #8
Struc. sim. = 0.77
N-Heptanoylglycine
deprotonated HMDB0000701, spectral sim. = 915, #9
Struc. sim. = 0.77
Hexanoylglycine

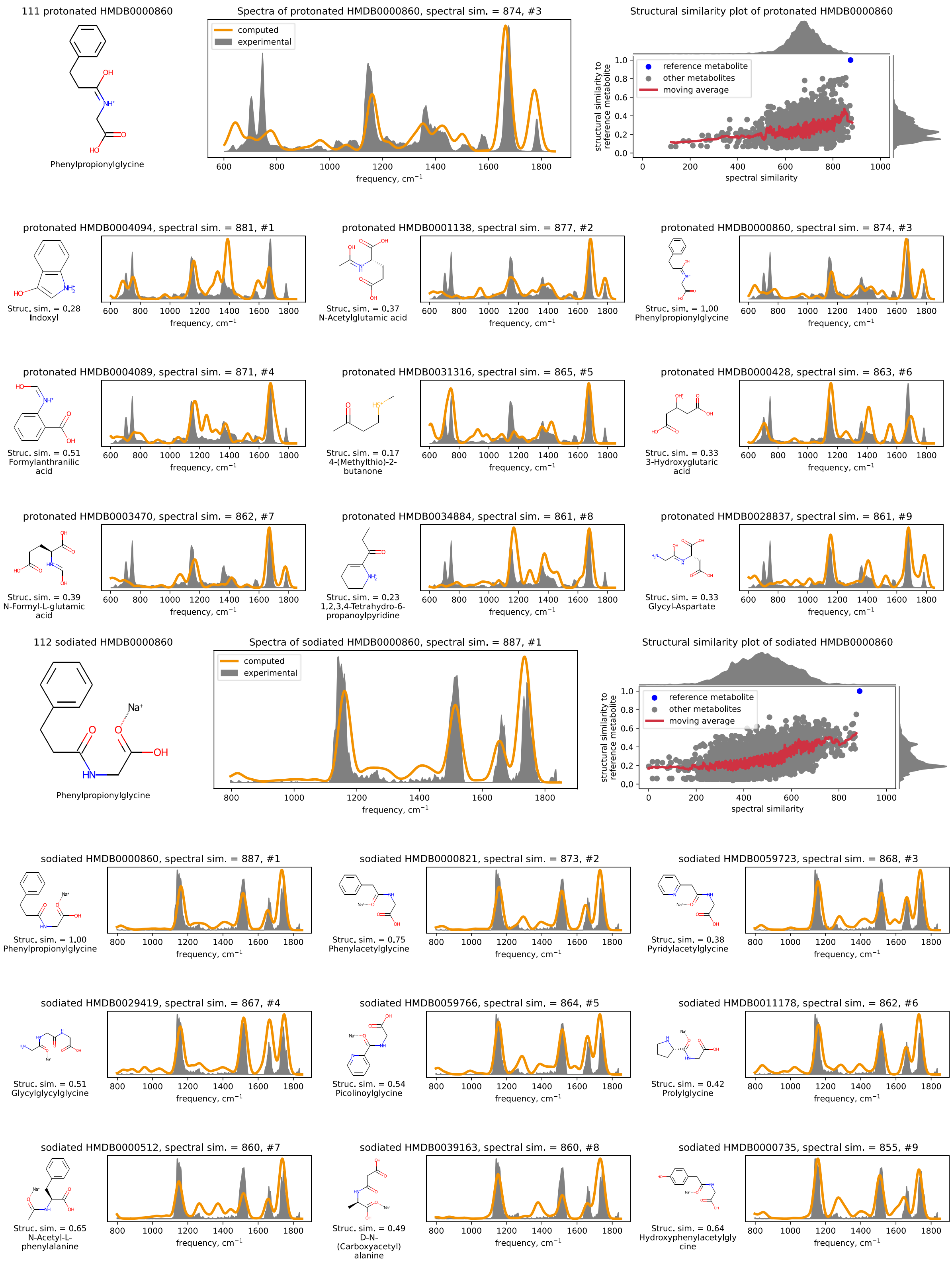

111 protonated HMDB0000860
Phenylpropionylglycine
Spectra of protonated HMDB0000860, spectral sim. = 874, #3
computed
experimental
frequency, cm^{-1}
Structural similarity plot of protonated HMDB0000860
reference metabolite
other metabolites
moving average
structural similarity to reference metabolite
spectral similarity
protonated HMDB0004094, spectral sim. = 881, #1
Struc. sim. = 0.28
Indoxyl
protonated HMDB0001138, spectral sim. = 877, #2
Struc. sim. = 0.37
N-Acetylglutamic acid
protonated HMDB0000860, spectral sim. = 874, #3
Struc. sim. = 1.00
Phenylpropionylglycine
protonated HMDB0004089, spectral sim. = 871, #4
Struc. sim. = 0.51
Formylanthranilic acid
protonated HMDB0031316, spectral sim. = 865, #5
Struc. sim. = 0.17
4-(Methylthio)-2-butanone
protonated HMDB0000428, spectral sim. = 863, #6
Struc. sim. = 0.33
3-Hydroxyglutaric acid
protonated HMDB0003470, spectral sim. = 862, #7
Struc. sim. = 0.39
N-Formyl-L-glutamic acid
protonated HMDB0034884, spectral sim. = 861, #8
Struc. sim. = 0.23
1,2,3,4-Tetrahydro-6-propanoylpyridine
protonated HMDB0028837, spectral sim. = 861, #9
Struc. sim. = 0.33
Glycyl-Aspartate
112 sodiated HMDB0000860
Phenylpropionylglycine
Spectra of sodiated HMDB0000860, spectral sim. = 887, #1
computed
experimental
frequency, cm^{-1}
Structural similarity plot of sodiated HMDB0000860
reference metabolite
other metabolites
moving average
structural similarity to reference metabolite
spectral similarity
sodiated HMDB0000860, spectral sim. = 887, #1
Struc. sim. = 1.00
Phenylpropionylglycine
sodiated HMDB0000821, spectral sim. = 873, #2
Struc. sim. = 0.75
Phenylacetylglycine
sodiated HMDB0059723, spectral sim. = 868, #3
Struc. sim. = 0.38
Pyridylacetylglycine
sodiated HMDB0029419, spectral sim. = 867, #4
Struc. sim. = 0.51
Glycylglycylglycine
sodiated HMDB0059766, spectral sim. = 864, #5
Struc. sim. = 0.54
Picolinoylglycine
sodiated HMDB0011178, spectral sim. = 862, #6
Struc. sim. = 0.42
Prolylglycine
sodiated HMDB0000512, spectral sim. = 860, #7
Struc. sim. = 0.65
N-Acetyl-L-phenylalanine
sodiated HMDB0039163, spectral sim. = 860, #8
Struc. sim. = 0.49
D-N-(Carboxyacetyl)alanine
sodiated HMDB0000735, spectral sim. = 855, #9
Struc. sim. = 0.64
Hydroxyphenylacetylglycine

113 deprotonated HMDB0000873

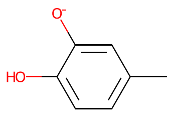

4-Methylcatechol

Spectra of deprotonated HMDB0000873, spectral sim. = 895, #2

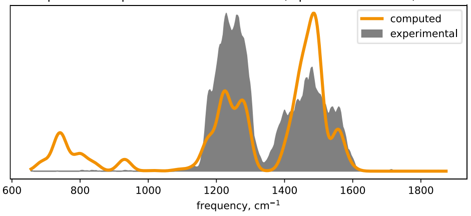

Structural similarity plot of deprotonated HMDB0000873

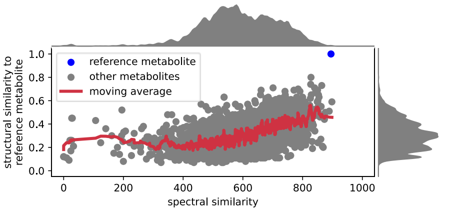

deprotonated HMDB0040174, spectral sim. = 898, #1

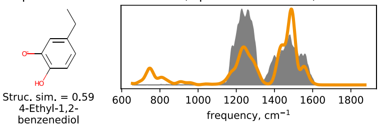

Struc. sim. = 0.59
4-Ethyl-1,2-benzenediol

deprotonated HMDB0000873, spectral sim. = 895, #2

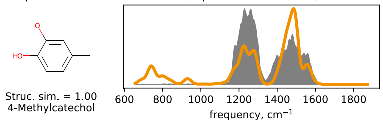

Struc. sim. = 1.00
4-Methylcatechol

deprotonated HMDB0005784, spectral sim. = 889, #3

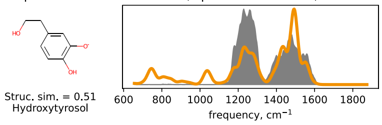

Struc. sim. = 0.51
Hydroxytyrosol

deprotonated HMDB0135239, spectral sim. = 876, #4

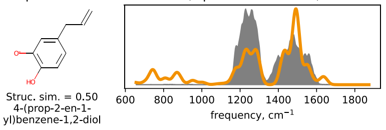

Struc. sim. = 0.50
4-(prop-2-en-1-yl)benzene-1,2-diol

deprotonated HMDB0003626, spectral sim. = 873, #5

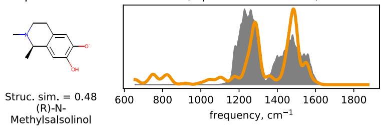

Struc. sim. = 0.48
(R)-N-Methylsalsolinol

deprotonated HMDB0002916, spectral sim. = 873, #6

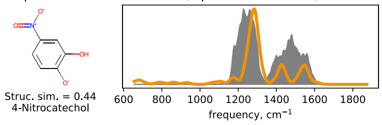

Struc. sim. = 0.44
4-Nitrocatechol

deprotonated HMDB0015052, spectral sim. = 869, #7

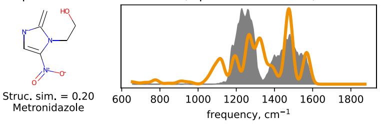

Struc. sim. = 0.20
Metronidazole

deprotonated HMDB0001216, spectral sim. = 866, #8

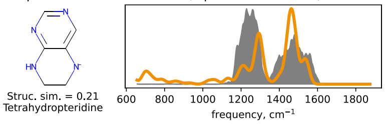

Struc. sim. = 0.21
Tetrahydropteridine

deprotonated HMDB0060497, spectral sim. = 864, #9

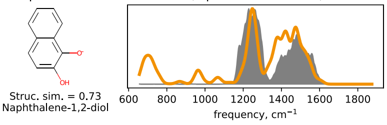

Struc. sim. = 0.73
Naphthalene-1,2-diol

114 protonated HMDB0000873

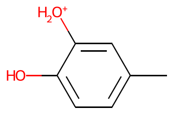

4-Methylcatechol

Spectra of protonated HMDB0000873, spectral sim. = 764, #3094

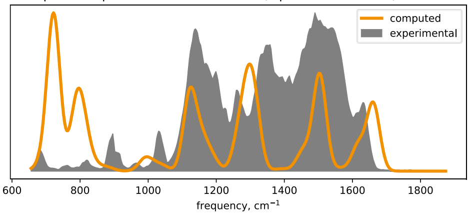

Structural similarity plot of protonated HMDB0000873

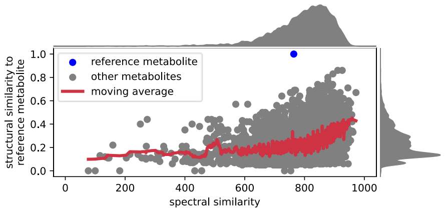

protonated HMDB0029661, spectral sim. = 972, #1

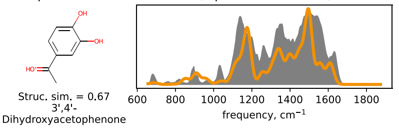

Struc. sim. = 0.67
3',4'-Dihydroxyacetophenone

protonated HMDB0136784, spectral sim. = 962, #2

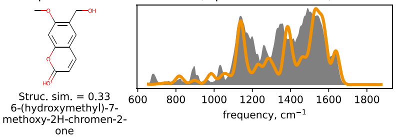

Struc. sim. = 0.33
6-(hydroxymethyl)-7-methoxy-2H-chromen-2-one

protonated HMDB0125523, spectral sim. = 960, #3

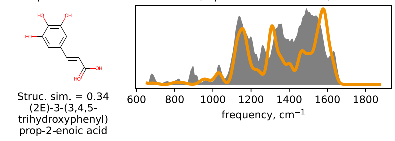

Struc. sim. = 0.34
(2E)-3-(3,4,5-trihydroxyphenyl)prop-2-enoic acid

protonated HMDB0012490, spectral sim. = 959, #4

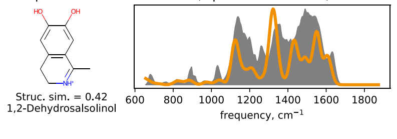

Struc. sim. = 0.42
1,2-Dehydrosalsolinol

protonated HMDB0136755, spectral sim. = 958, #5

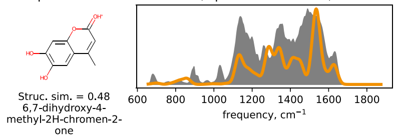

Struc. sim. = 0.48
6,7-dihydroxy-4-methyl-2H-chromen-2-one

protonated HMDB0132981, spectral sim. = 954, #6

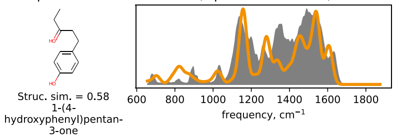

Struc. sim. = 0.58
1-(4-hydroxyphenyl)pentan-3-one

protonated HMDB0030818, spectral sim. = 954, #7

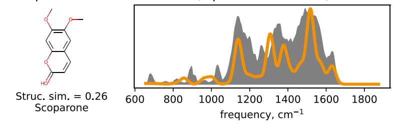

Struc. sim. = 0.26
Scoparone

protonated HMDB0061686, spectral sim. = 950, #8

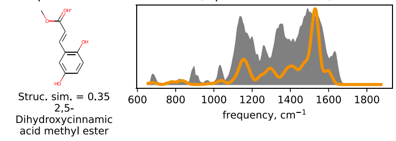

Struc. sim. = 0.35
2,5-Dihydroxycinnamic acid methyl ester

protonated HMDB0037177, spectral sim. = 949, #9

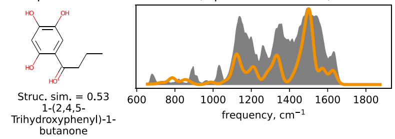

Struc. sim. = 0.53
1-(2,4,5-Trihydroxyphenyl)-1-butanone

115 sodiated HMDB0000873

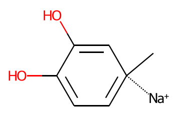

4-Methylcatechol

Spectra of sodiated HMDB0000873, spectral sim. = 884, #13

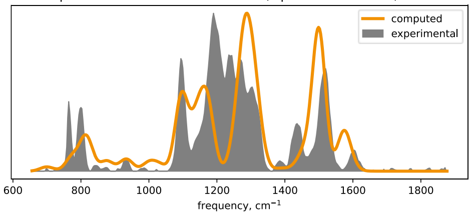

Structural similarity plot of sodiated HMDB0000873

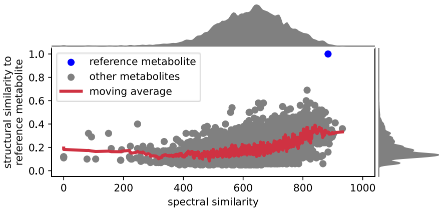

sodiated HMDB0062396, spectral sim. = 932, #1

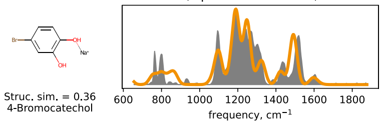

sodiated HMDB0040174, spectral sim. = 908, #2

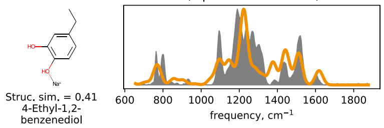

sodiated HMDB0032136, spectral sim. = 903, #3

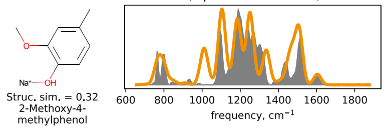

sodiated HMDB0040175, spectral sim. = 899, #4

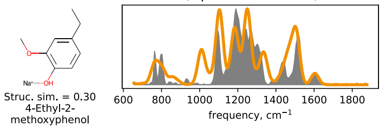

sodiated HMDB0032135, spectral sim. = 898, #5

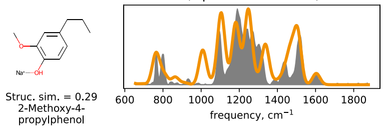

sodiated HMDB0005802, spectral sim. = 896, #6

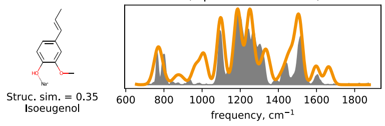

sodiated HMDB0013744, spectral sim. = 894, #7

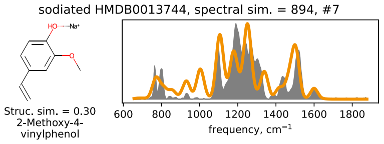

sodiated HMDB0001490, spectral sim. = 893, #8

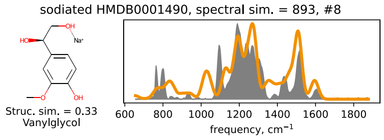

sodiated HMDB0035056, spectral sim. = 892, #9

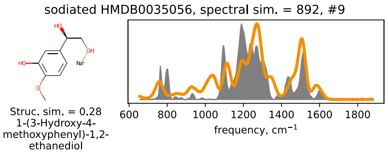

116 deprotonated HMDB0000904

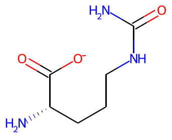

Citrulline

Spectra of deprotonated HMDB0000904, spectral sim. = 891, #22

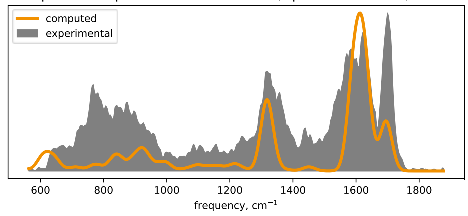

Structural similarity plot of deprotonated HMDB0000904

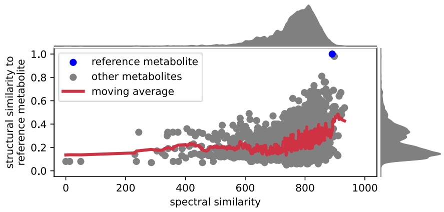

deprotonated HMDB0004224, spectral sim. = 932, #1

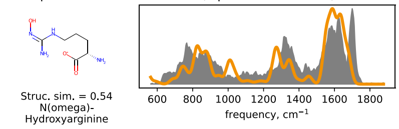

deprotonated HMDB0028855, spectral sim. = 924, #2

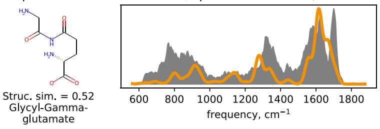

deprotonated HMDB0041540, spectral sim. = 922, #3

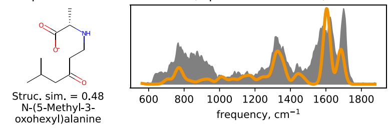

deprotonated HMDB0011667, spectral sim. = 920, #4

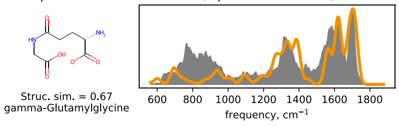

deprotonated HMDB0011165, spectral sim. = 914, #5

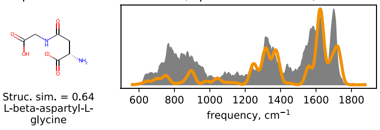

deprotonated HMDB0012948, spectral sim. = 911, #6

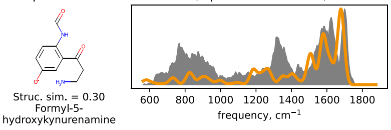

deprotonated HMDB0132254, spectral sim. = 910, #7

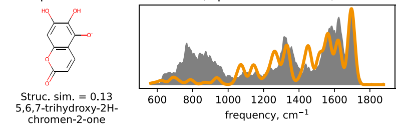

deprotonated HMDB0014991, spectral sim. = 907, #8

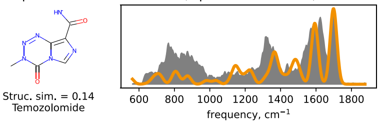

deprotonated HMDB0000177, spectral sim. = 903, #9

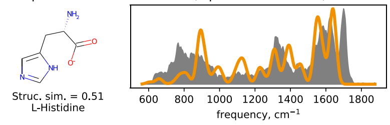

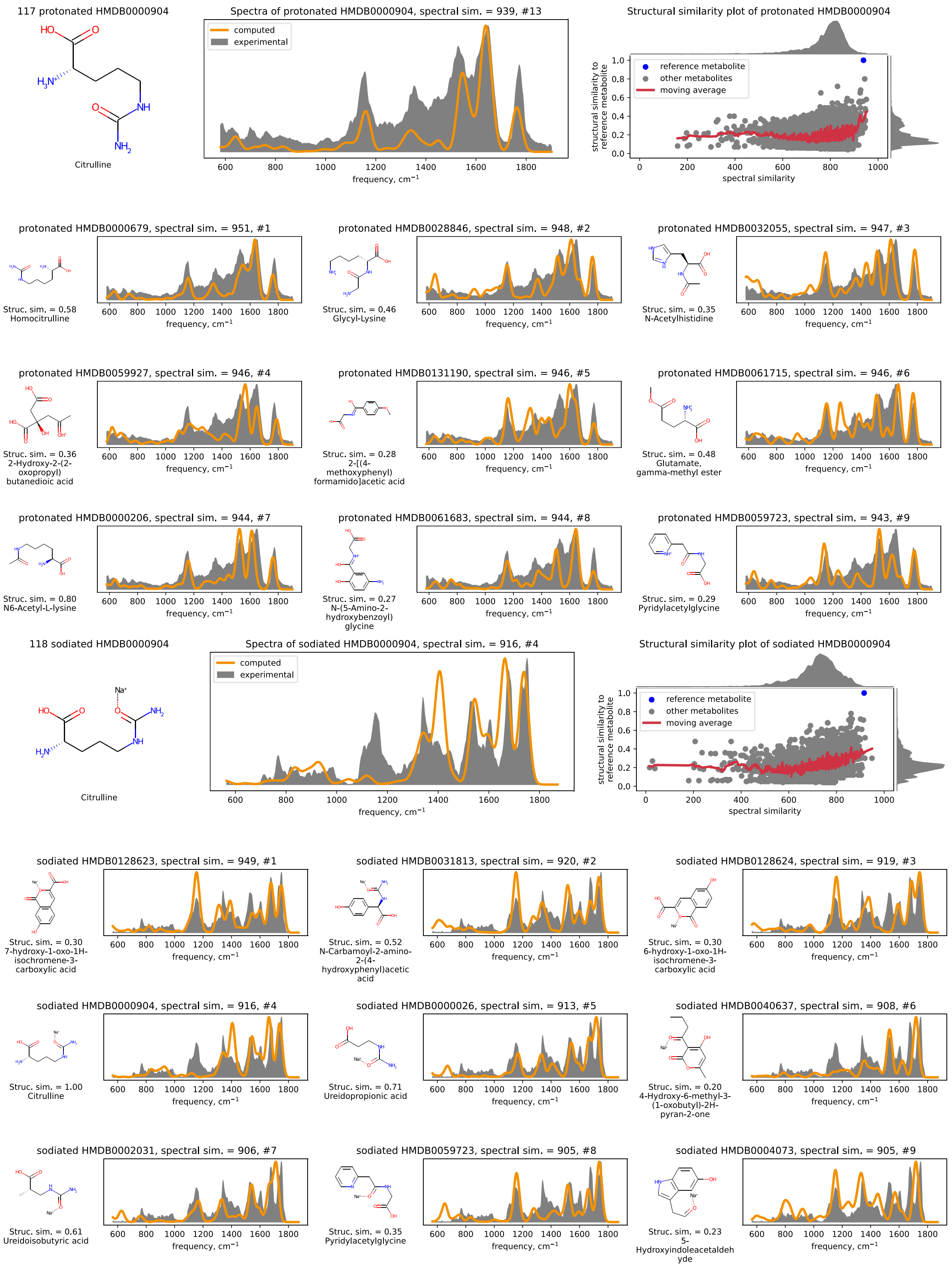
117 protonated HMDB0000904
Citrulline
Spectra of protonated HMDB0000904, spectral sim. = 939, #13
computed
experimental
frequency, cm⁻¹
Structural similarity plot of protonated HMDB0000904
reference metabolite
other metabolites
moving average
structural similarity to reference metabolite
spectral similarity
protonated HMDB0000679, spectral sim. = 951, #1
Struc. sim. = 0.58
Homocitrulline
protonated HMDB0028846, spectral sim. = 948, #2
Struc. sim. = 0.46
Glycyl-Lysine
protonated HMDB0032055, spectral sim. = 947, #3
Struc. sim. = 0.35
N-Acetylhistidine
protonated HMDB0059927, spectral sim. = 946, #4
Struc. sim. = 0.36
2-Hydroxy-2-(2-oxopropyl) butanedioic acid
protonated HMDB0131190, spectral sim. = 946, #5
Struc. sim. = 0.28
2-[(4-methoxyphenyl) formamido]acetic acid
protonated HMDB0061715, spectral sim. = 946, #6
Struc. sim. = 0.48
Glutamate, gamma-methyl ester
protonated HMDB0000206, spectral sim. = 944, #7
Struc. sim. = 0.80
N6-Acetyl-L-lysine
protonated HMDB0061683, spectral sim. = 944, #8
Struc. sim. = 0.27
N-(5-Amino-2-hydroxybenzoyl) glycine
protonated HMDB0059723, spectral sim. = 943, #9
Struc. sim. = 0.29
Pyridylacetylglycine
118 sodiated HMDB0000904
Citrulline
Spectra of sodiated HMDB0000904, spectral sim. = 916, #4
Structural similarity plot of sodiated HMDB0000904
sodiated HMDB0128623, spectral sim. = 949, #1
Struc. sim. = 0.30
7-hydroxy-1-oxo-1H-isochromene-3-carboxylic acid
sodiated HMDB0031813, spectral sim. = 920, #2
Struc. sim. = 0.52
N-Carbamoyl-2-amino-2-(4-hydroxyphenyl)acetic acid
sodiated HMDB0128624, spectral sim. = 919, #3
Struc. sim. = 0.30
6-hydroxy-1-oxo-1H-isochromene-3-carboxylic acid
sodiated HMDB0000904, spectral sim. = 916, #4
Struc. sim. = 1.00
Citrulline
sodiated HMDB0000026, spectral sim. = 913, #5
Struc. sim. = 0.71
Ureidopropionic acid
sodiated HMDB0040637, spectral sim. = 908, #6
Struc. sim. = 0.20
4-Hydroxy-6-methyl-3-(1-oxobutyl)-2H-pyran-2-one
sodiated HMDB0002031, spectral sim. = 906, #7
Struc. sim. = 0.61
Ureidoisobutyric acid
sodiated HMDB0059723, spectral sim. = 905, #8
Struc. sim. = 0.35
Pyridylacetylglycine
sodiated HMDB0004073, spectral sim. = 905, #9
Struc. sim. = 0.23
5-Hydroxyindoleacetaldehyde

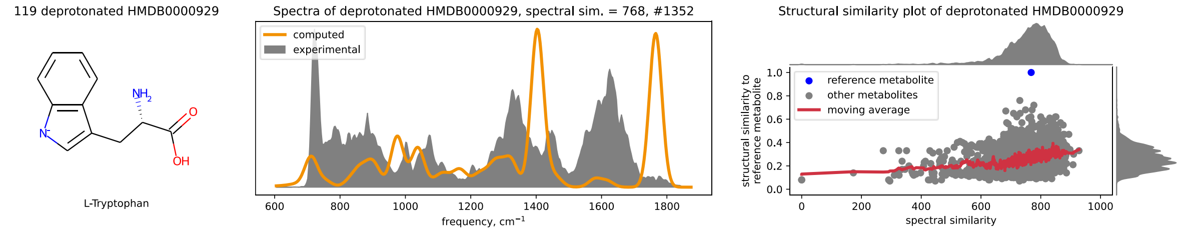
119 deprotonated HMDB0000929
L-Tryptophan
Spectra of deprotonated HMDB0000929, spectral sim. = 768, #1352
computed
experimental
frequency, cm^{-1}
Structural similarity plot of deprotonated HMDB0000929
reference metabolite
other metabolites
moving average
structural similarity to reference metabolite
spectral similarity

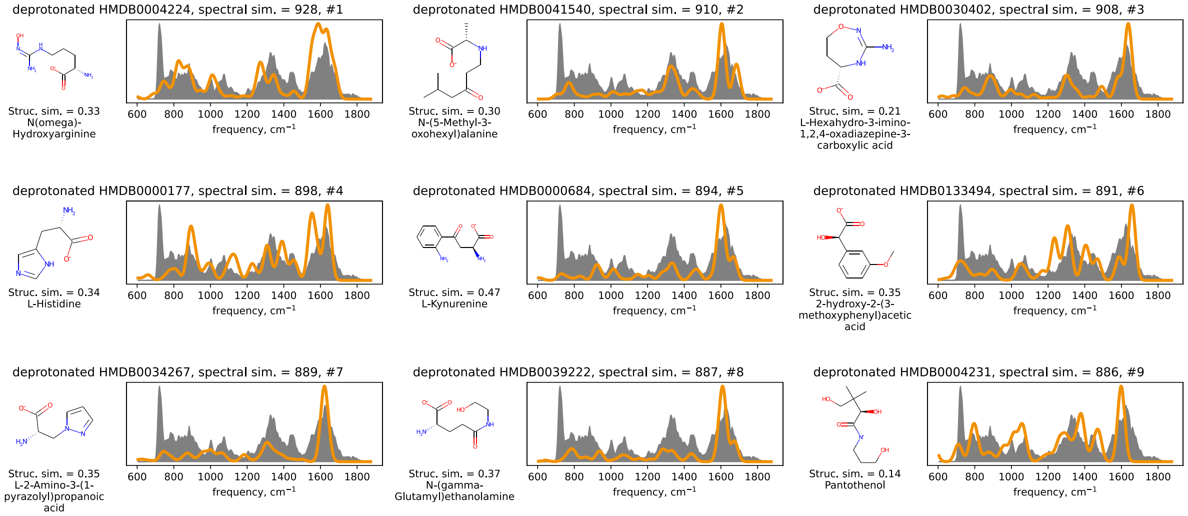
deprotonated HMDB0004224, spectral sim. = 928, #1
Struc. sim. = 0.33
N(omega)-Hydroxyarginine
deprotonated HMDB0041540, spectral sim. = 910, #2
Struc. sim. = 0.30
N-(5-Methyl-3-oxohexyl)alanine
deprotonated HMDB0030402, spectral sim. = 908, #3
Struc. sim. = 0.21
L-Hexahydro-3-imino-1,2,4-oxadiazepine-3-carboxylic acid
deprotonated HMDB0000177, spectral sim. = 898, #4
Struc. sim. = 0.34
L-Histidine
deprotonated HMDB0000684, spectral sim. = 894, #5
Struc. sim. = 0.47
L-Kynurenine
deprotonated HMDB0133494, spectral sim. = 891, #6
Struc. sim. = 0.35
2-hydroxy-2-(3-methoxyphenyl)acetic acid
deprotonated HMDB0034267, spectral sim. = 889, #7
Struc. sim. = 0.35
L-2-Amino-3-(1-pyrazolyl)propanoic acid
deprotonated HMDB0039222, spectral sim. = 887, #8
Struc. sim. = 0.37
N-(gamma-Glutamyl)ethanolamine
deprotonated HMDB0004231, spectral sim. = 886, #9
Struc. sim. = 0.14
Pantothenol
frequency, cm^{-1}

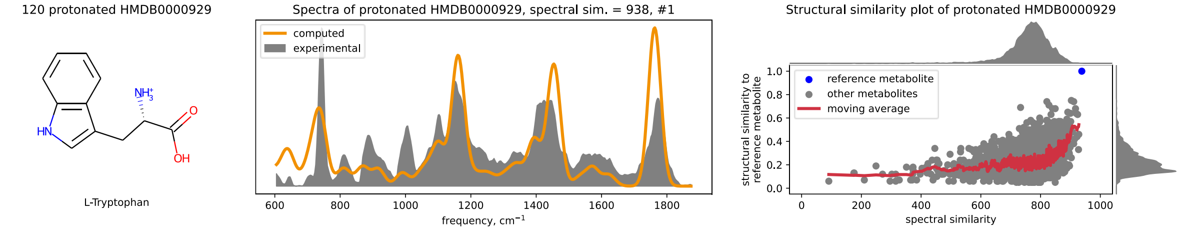
120 protonated HMDB0000929
L-Tryptophan
Spectra of protonated HMDB0000929, spectral sim. = 938, #1
computed
experimental
frequency, cm^{-1}
Structural similarity plot of protonated HMDB0000929
reference metabolite
other metabolites
moving average
structural similarity to reference metabolite
spectral similarity

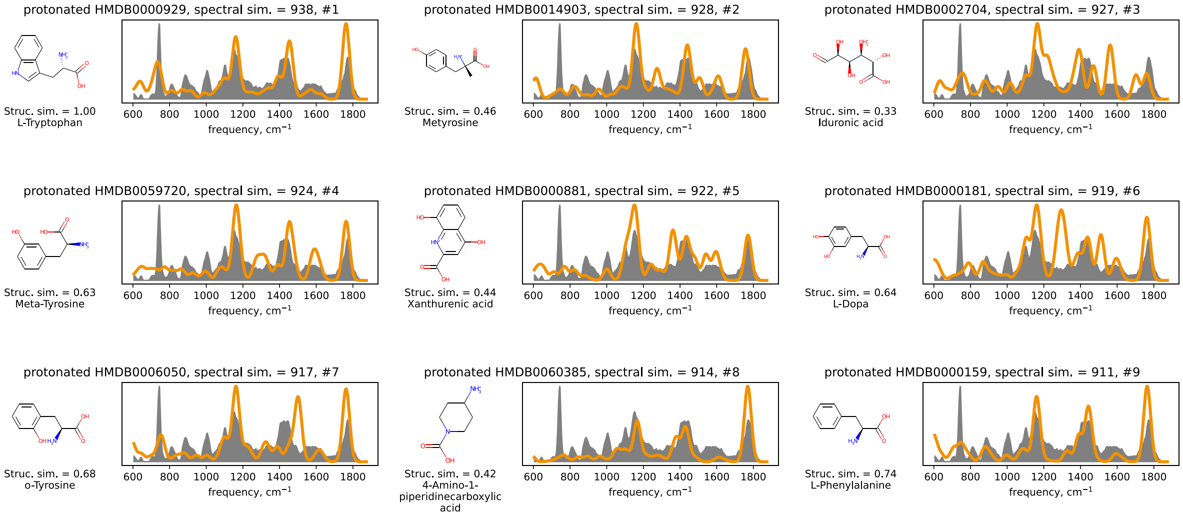
protonated HMDB0000929, spectral sim. = 938, #1
Struc. sim. = 1.00
L-Tryptophan
protonated HMDB0014903, spectral sim. = 928, #2
Struc. sim. = 0.46
Metyrosine
protonated HMDB0002704, spectral sim. = 927, #3
Struc. sim. = 0.33
Iduronic acid
protonated HMDB0059720, spectral sim. = 924, #4
Struc. sim. = 0.63
Meta-Tyrosine
protonated HMDB0000881, spectral sim. = 922, #5
Struc. sim. = 0.44
Xanthurenic acid
protonated HMDB0000181, spectral sim. = 919, #6
Struc. sim. = 0.64
L-Dopa
protonated HMDB0006050, spectral sim. = 917, #7
Struc. sim. = 0.68
o-Tyrosine
protonated HMDB0060385, spectral sim. = 914, #8
Struc. sim. = 0.42
4-Amino-1-piperidinecarboxylic acid
protonated HMDB0000159, spectral sim. = 911, #9
Struc. sim. = 0.74
L-Phenylalanine
frequency, cm^{-1}

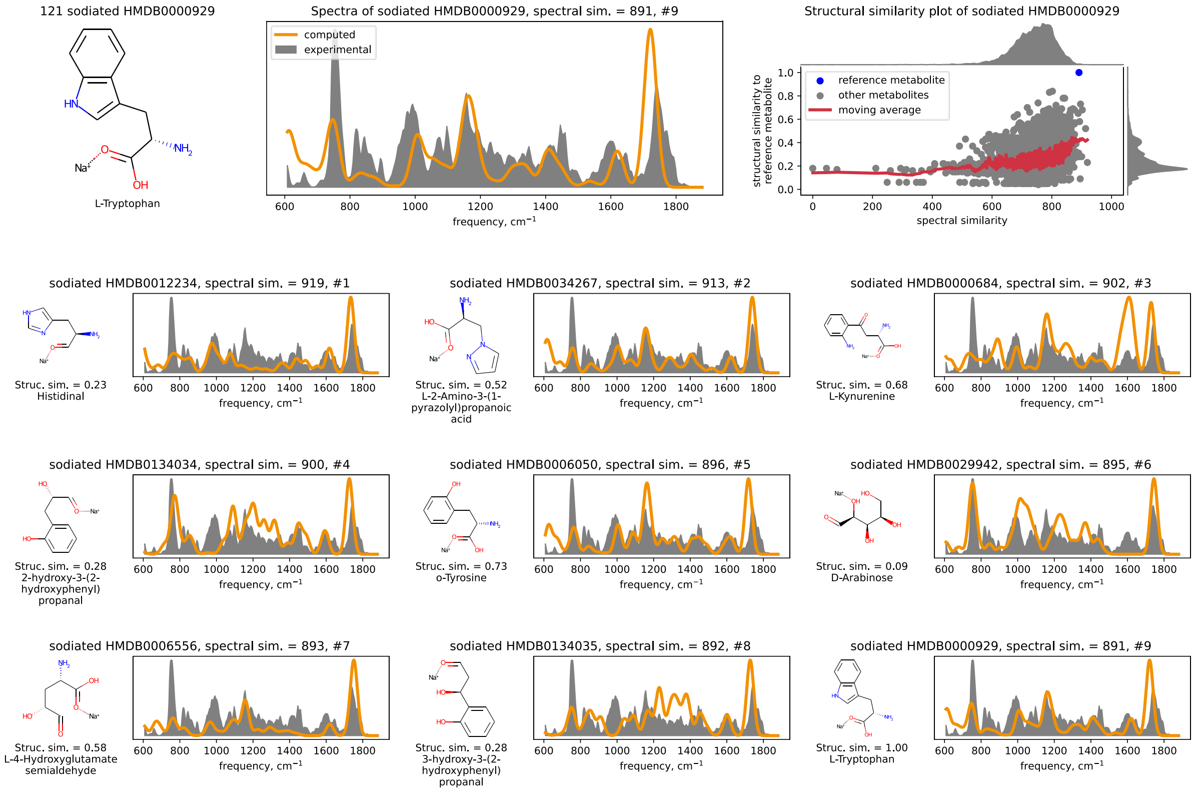
121 sodiated HMDB0000929
L-Tryptophan
Spectra of sodiated HMDB0000929, spectral sim. = 891, #9
computed
experimental
frequency, cm^{-1}
Structural similarity plot of sodiated HMDB0000929
reference metabolite
other metabolites
moving average
structural similarity to reference metabolite
spectral similarity
sodiated HMDB0012234, spectral sim. = 919, #1
Struc. sim. = 0.23
Histidinal
sodiated HMDB0034267, spectral sim. = 913, #2
Struc. sim. = 0.52
L-2-Amino-3-(1-pyrazolyl)propanoic acid
sodiated HMDB0000684, spectral sim. = 902, #3
Struc. sim. = 0.68
L-Kynurenine
sodiated HMDB0134034, spectral sim. = 900, #4
Struc. sim. = 0.28
2-hydroxy-3-(2-hydroxyphenyl)propanal
sodiated HMDB0006050, spectral sim. = 896, #5
Struc. sim. = 0.73
o-Tyrosine
sodiated HMDB0029942, spectral sim. = 895, #6
Struc. sim. = 0.09
D-Arabinose
sodiated HMDB0006556, spectral sim. = 893, #7
Struc. sim. = 0.58
L-4-Hydroxyglutamate semialdehyde
sodiated HMDB0134035, spectral sim. = 892, #8
Struc. sim. = 0.28
3-hydroxy-3-(2-hydroxyphenyl)propanal
sodiated HMDB0000929, spectral sim. = 891, #9
Struc. sim. = 1.00
L-Tryptophan

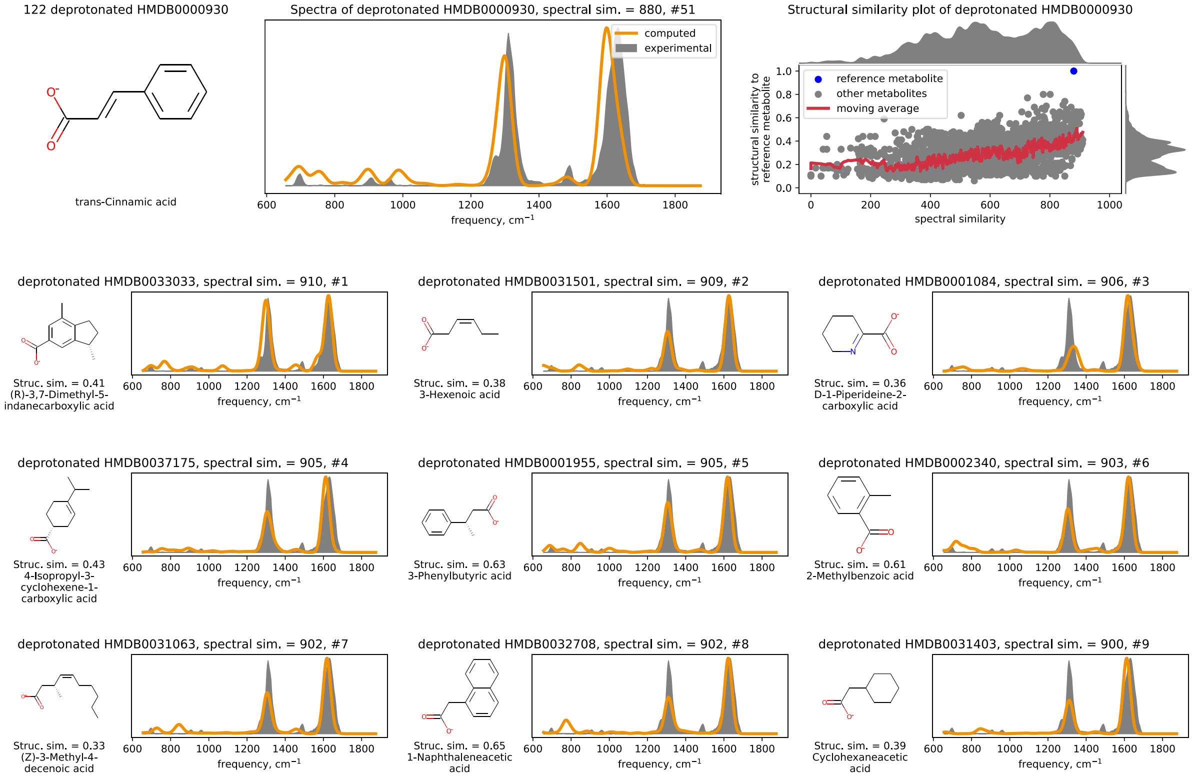
122 deprotonated HMDB0000930
trans-Cinnamic acid
Spectra of deprotonated HMDB0000930, spectral sim. = 880, #51
computed
experimental
frequency, cm^{-1}
Structural similarity plot of deprotonated HMDB0000930
reference metabolite
other metabolites
moving average
structural similarity to reference metabolite
spectral similarity
deprotonated HMDB0033033, spectral sim. = 910, #1
Struc. sim. = 0.41
(R)-3,7-Dimethyl-5-indanecarboxylic acid
deprotonated HMDB0031501, spectral sim. = 909, #2
Struc. sim. = 0.38
3-Hexenoic acid
deprotonated HMDB0001084, spectral sim. = 906, #3
Struc. sim. = 0.36
D-1-Piperideine-2-carboxylic acid
deprotonated HMDB0037175, spectral sim. = 905, #4
Struc. sim. = 0.43
4-Isopropyl-3-cyclohexene-1-carboxylic acid
deprotonated HMDB0001955, spectral sim. = 905, #5
Struc. sim. = 0.63
3-Phenylbutyric acid
deprotonated HMDB0002340, spectral sim. = 903, #6
Struc. sim. = 0.61
2-Methylbenzoic acid
deprotonated HMDB0031063, spectral sim. = 902, #7
Struc. sim. = 0.33
(Z)-3-Methyl-4-decenoic acid
deprotonated HMDB0032708, spectral sim. = 902, #8
Struc. sim. = 0.65
1-Naphthaleneacetic acid
deprotonated HMDB0031403, spectral sim. = 900, #9
Struc. sim. = 0.39
Cyclohexaneacetic acid

123 sodiated HMDB0000930

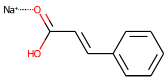

trans-Cinnamic acid

Spectra of sodiated HMDB0000930, spectral sim. = 830, #1001

Structural similarity plot of sodiated HMDB0000930

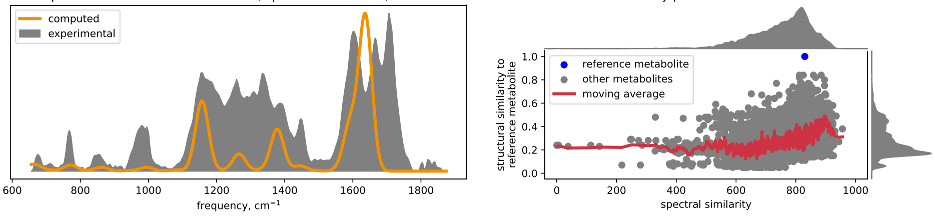

sodiated HMDB0040268, spectral sim. = 956, #1

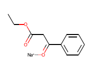

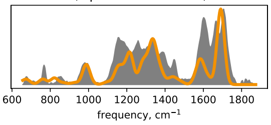

Struc. sim. = 0.38
Ethyl 3-oxo-3-phenylpropanoate

sodiated HMDB0128621, spectral sim. = 944, #2

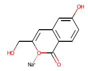

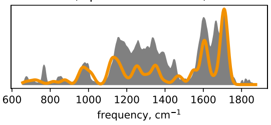

Struc. sim. = 0.27
6-hydroxy-3-(hydroxymethyl)-1H-isochromen-1-one

sodiated HMDB0125529, spectral sim. = 935, #3

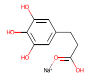

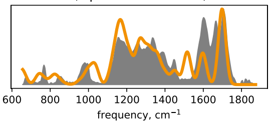

Struc. sim. = 0.49
3-(3,4,5-trihydroxyphenyl) propanoic acid

sodiated HMDB0006954, spectral sim. = 931, #4

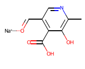

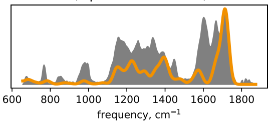

Struc. sim. = 0.36
2-Methyl-3-hydroxy-5-formylpyridine-4-carboxylate

sodiated HMDB0133507, spectral sim. = 929, #5

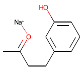

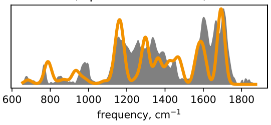

Struc. sim. = 0.33
4-(3-hydroxyphenyl)butan-2-one

sodiated HMDB0012948, spectral sim. = 927, #6

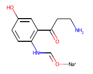

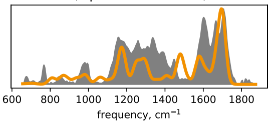

Struc. sim. = 0.24
Formyl-5-hydroxykynurenamine

sodiated HMDB0040039, spectral sim. = 926, #7

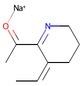

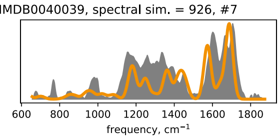

Struc. sim. = 0.15
2-Acetyl-3-ethylidene-3,4,5,6-tetrahydropyridine

sodiated HMDB0037071, spectral sim. = 926, #8

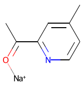

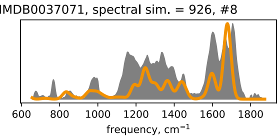

Struc. sim. = 0.27
2-Acetyl-4-methylpyridine

sodiated HMDB0062406, spectral sim. = 925, #9

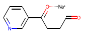

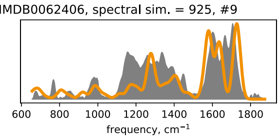

Struc. sim. = 0.29
4-Oxo-1-(3-pyridyl)-1-butanone

124 protonated HMDB0000959

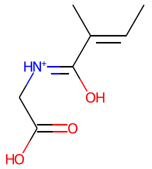

Tiglylglycine

Spectra of protonated HMDB0000959, spectral sim. = 858, #323

Structural similarity plot of protonated HMDB0000959

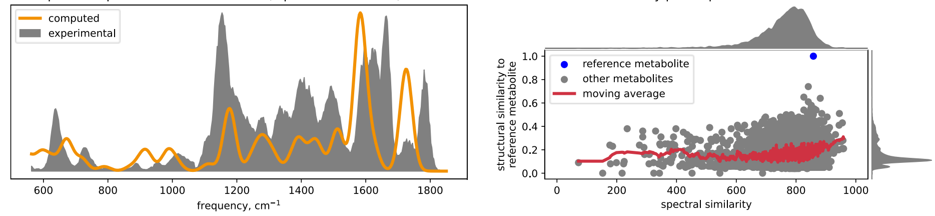

protonated HMDB0006116, spectral sim. = 959, #1

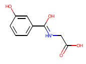

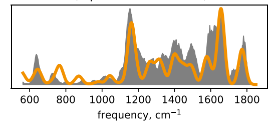

Struc. sim. = 0.21
3-Hydroxyhippuric acid

protonated HMDB0013678, spectral sim. = 958, #2

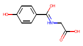

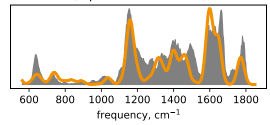

Struc. sim. = 0.21
4-Hydroxyhippuric acid

protonated HMDB0013292, spectral sim. = 957, #3

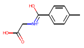

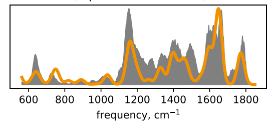

Struc. sim. = 0.28
p-Methylhippuric acid

protonated HMDB0131190, spectral sim. = 955, #4

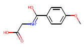

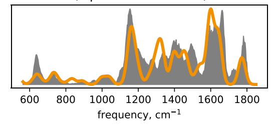

Struc. sim. = 0.21
2-[(4-methoxyphenyl) formamido]acetic acid

protonated HMDB0000840, spectral sim. = 955, #5

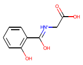

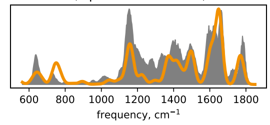

Struc. sim. = 0.43
Salicyluric acid

protonated HMDB0013245, spectral sim. = 951, #6

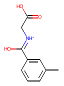

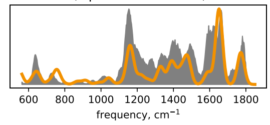

Struc. sim. = 0.27
m-Methylhippuric acid

protonated HMDB0011723, spectral sim. = 945, #7

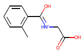

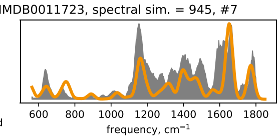

Struc. sim. = 0.51
2-Methylhippuric acid

protonated HMDB0011621, spectral sim. = 945, #8

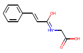

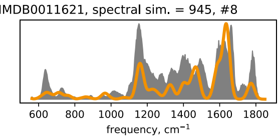

Struc. sim. = 0.27
Cinnamoylglycine

protonated HMDB0061683, spectral sim. = 944, #9

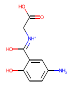

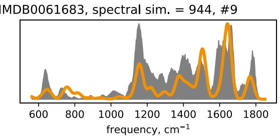

Struc. sim. = 0.41
N-(5-Amino-2-hydroxybenzoyl) glycine

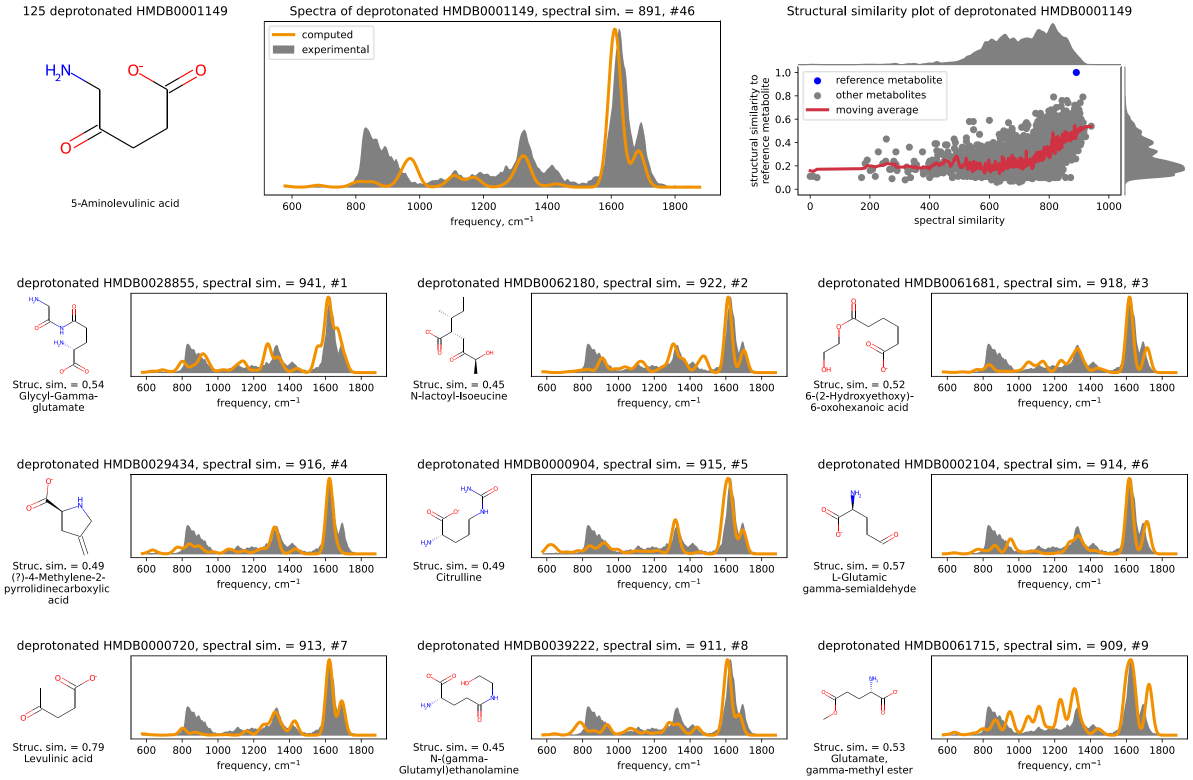
125 deprotonated HMDB0001149
5-Aminolevulinic acid
Spectra of deprotonated HMDB0001149, spectral sim. = 891, #46
computed
experimental
frequency, cm⁻¹
Structural similarity plot of deprotonated HMDB0001149
reference metabolite
other metabolites
moving average
structural similarity to reference metabolite
spectral similarity
deprotonated HMDB0028855, spectral sim. = 941, #1
Struc. sim. = 0.54
Glycyl-Gamma-glutamate
deprotonated HMDB0062180, spectral sim. = 922, #2
Struc. sim. = 0.45
N-lactoyl-Isoeucine
deprotonated HMDB0061681, spectral sim. = 918, #3
Struc. sim. = 0.52
6-(2-Hydroxyethoxy)-6-oxohexanoic acid
deprotonated HMDB0029434, spectral sim. = 916, #4
Struc. sim. = 0.49
(?)-4-Methylene-2-pyrrolidinecarboxylic acid
deprotonated HMDB0000904, spectral sim. = 915, #5
Struc. sim. = 0.49
Citrulline
deprotonated HMDB0002104, spectral sim. = 914, #6
Struc. sim. = 0.57
L-Glutamic gamma-semialdehyde
deprotonated HMDB0000720, spectral sim. = 913, #7
Struc. sim. = 0.79
Levulinic acid
deprotonated HMDB0039222, spectral sim. = 911, #8
Struc. sim. = 0.45
N-(gamma-Glutamyl)ethanolamine
deprotonated HMDB0061715, spectral sim. = 909, #9
Struc. sim. = 0.53
Glutamate, gamma-methyl ester

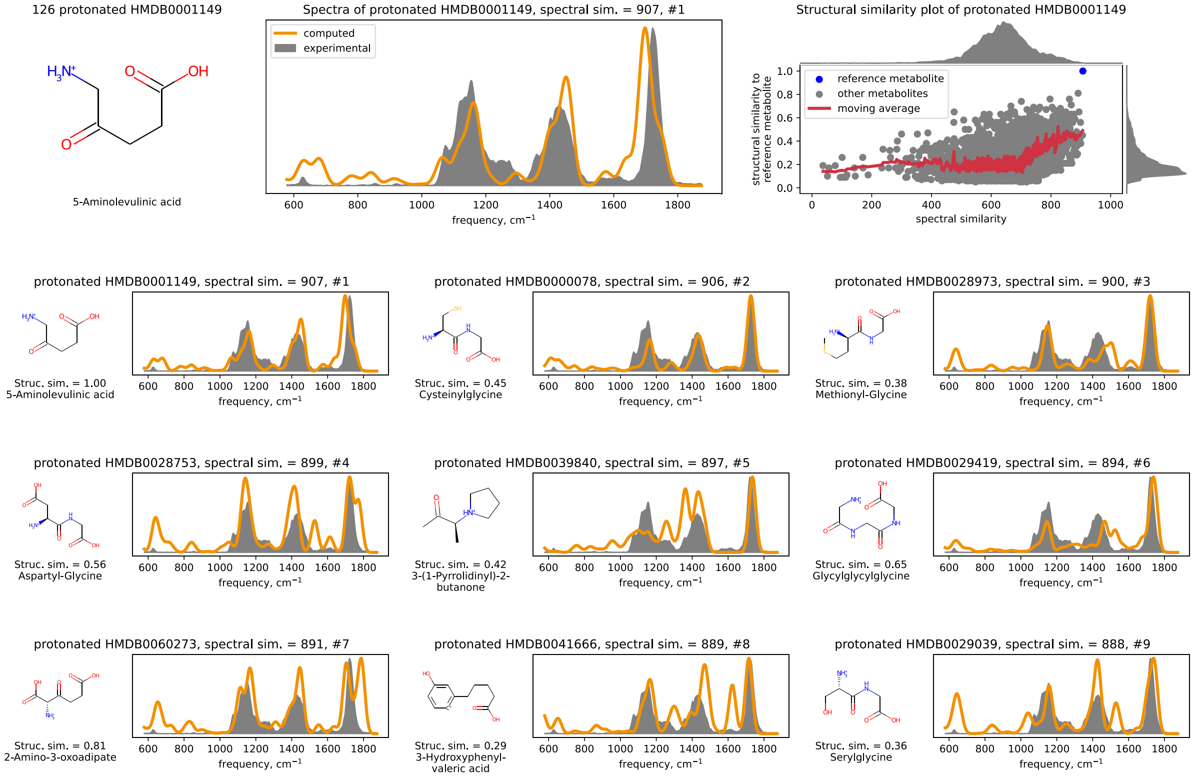
126 protonated HMDB0001149
5-Aminolevulinic acid
Spectra of protonated HMDB0001149, spectral sim. = 907, #1
computed
experimental
frequency, cm⁻¹
Structural similarity plot of protonated HMDB0001149
reference metabolite
other metabolites
moving average
structural similarity to reference metabolite
spectral similarity
protonated HMDB0001149, spectral sim. = 907, #1
Struc. sim. = 1.00
5-Aminolevulinic acid
protonated HMDB0000078, spectral sim. = 906, #2
Struc. sim. = 0.45
Cysteinylglycine
protonated HMDB0028973, spectral sim. = 900, #3
Struc. sim. = 0.38
Methionyl-Glycine
protonated HMDB0028753, spectral sim. = 899, #4
Struc. sim. = 0.56
Aspartyl-Glycine
protonated HMDB0039840, spectral sim. = 897, #5
Struc. sim. = 0.42
3-(1-Pyrrolidinyl)-2-butanone
protonated HMDB0029419, spectral sim. = 894, #6
Struc. sim. = 0.65
Glycylglycylglycine
protonated HMDB0060273, spectral sim. = 891, #7
Struc. sim. = 0.81
2-Amino-3-oxoadipate
protonated HMDB0041666, spectral sim. = 889, #8
Struc. sim. = 0.29
3-Hydroxyphenyl-valeric acid
protonated HMDB0029039, spectral sim. = 888, #9
Struc. sim. = 0.36
Serylglycine

127 sodiated HMDB0001149

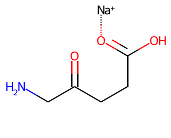

5-Aminolevulinic acid

Spectra of sodiated HMDB0001149, spectral sim. = 861, #120

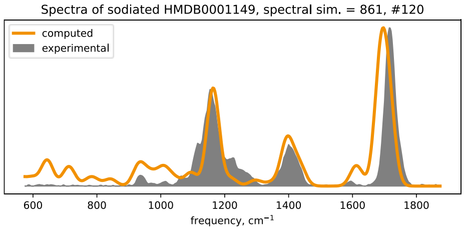

Structural similarity plot of sodiated HMDB0001149

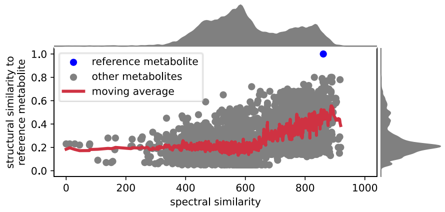

sodiated HMDB0033551, spectral sim. = 919, #1

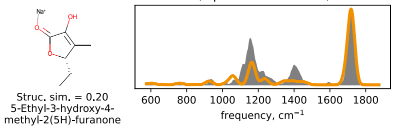

Struc. sim. = 0.20
5-Ethyl-3-hydroxy-4-methyl-2(5H)-furanone

sodiated HMDB0000635, spectral sim. = 917, #2

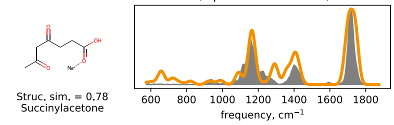

Struc. sim. = 0.78
Succinylacetone

sodiated HMDB0002523, spectral sim. = 916, #3

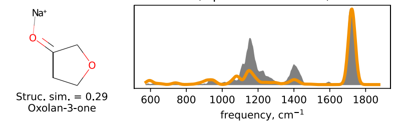

Struc. sim. = 0.29
Oxolan-3-one

sodiated HMDB0031306, spectral sim. = 915, #4

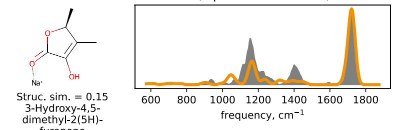

Struc. sim. = 0.15
3-Hydroxy-4,5-dimethyl-2(5H)-furanone

sodiated HMDB0036626, spectral sim. = 911, #5

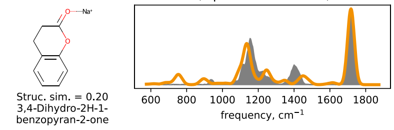

Struc. sim. = 0.20
3,4-Dihydro-2H-1-benzopyran-2-one

sodiated HMDB0031178, spectral sim. = 911, #6

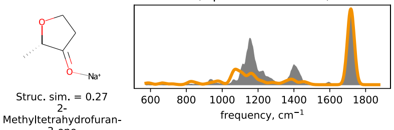

Struc. sim. = 0.27
2-Methyltetrahydrofuran-3-one

sodiated HMDB0060683, spectral sim. = 905, #7

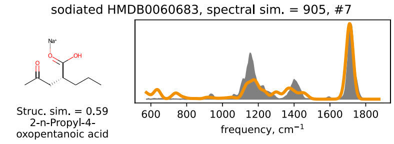

Struc. sim. = 0.59
2-n-Propyl-4-oxopentanoic acid

sodiated HMDB0000345, spectral sim. = 903, #8

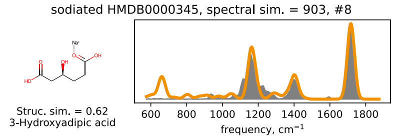

Struc. sim. = 0.62
3-Hydroxyadipic acid

sodiated HMDB0000428, spectral sim. = 903, #9

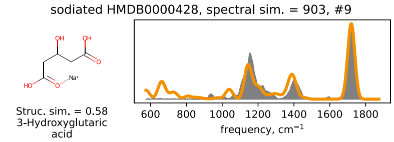

Struc. sim. = 0.58
3-Hydroxyglutaric acid

128 deprotonated HMDB0001232

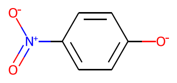

4-Nitrophenol

Spectra of deprotonated HMDB0001232, spectral sim. = 817, #10

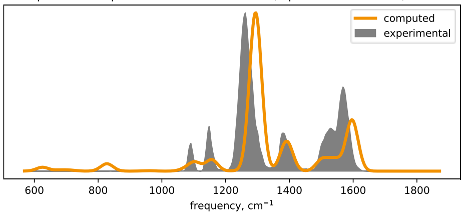

Structural similarity plot of deprotonated HMDB0001232

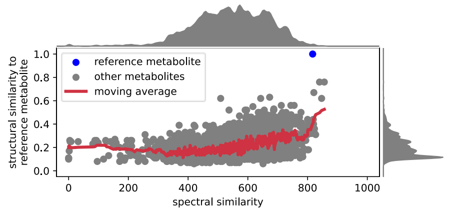

deprotonated HMDB0002916, spectral sim. = 856, #1

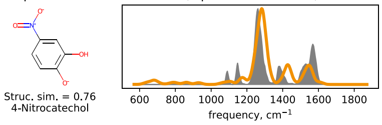

Struc. sim. = 0.76
4-Nitrocatechol

deprotonated HMDB0015491, spectral sim. = 847, #2

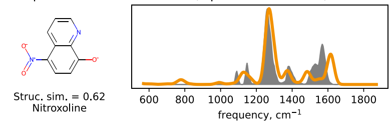

Struc. sim. = 0.62
Nitroxoline

deprotonated HMDB0006200, spectral sim. = 840, #3

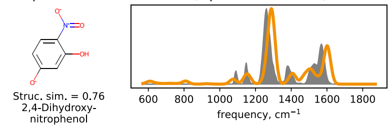

Struc. sim. = 0.76
2,4-Dihydroxy-nitrophenol

deprotonated HMDB0012915, spectral sim. = 827, #4

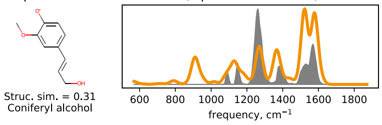

Struc. sim. = 0.31
Coniferyl alcohol

deprotonated HMDB0059824, spectral sim. = 823, #5

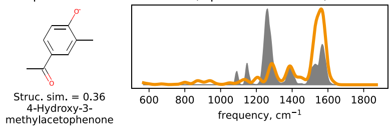

Struc. sim. = 0.36
4-Hydroxy-3-methylacetophenone

deprotonated HMDB0005802, spectral sim. = 822, #6

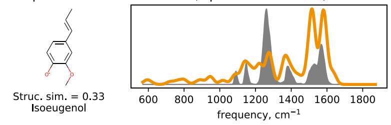

Struc. sim. = 0.33
Isoeugenol

deprotonated HMDB0059811, spectral sim. = 821, #7

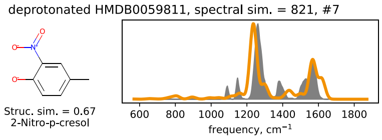

Struc. sim. = 0.67
2-Nitro-p-cresol

deprotonated HMDB0013744, spectral sim. = 820, #8

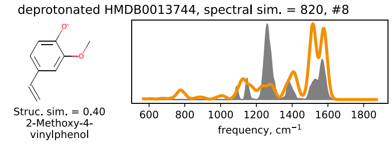

Struc. sim. = 0.40
2-Methoxy-4-vinylphenol

deprotonated HMDB0133749, spectral sim. = 819, #9

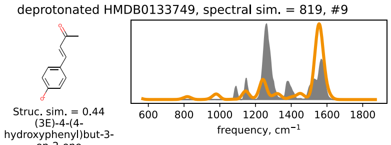

Struc. sim. = 0.44
(3E)-4-(4-hydroxyphenyl)but-3-en-2-one

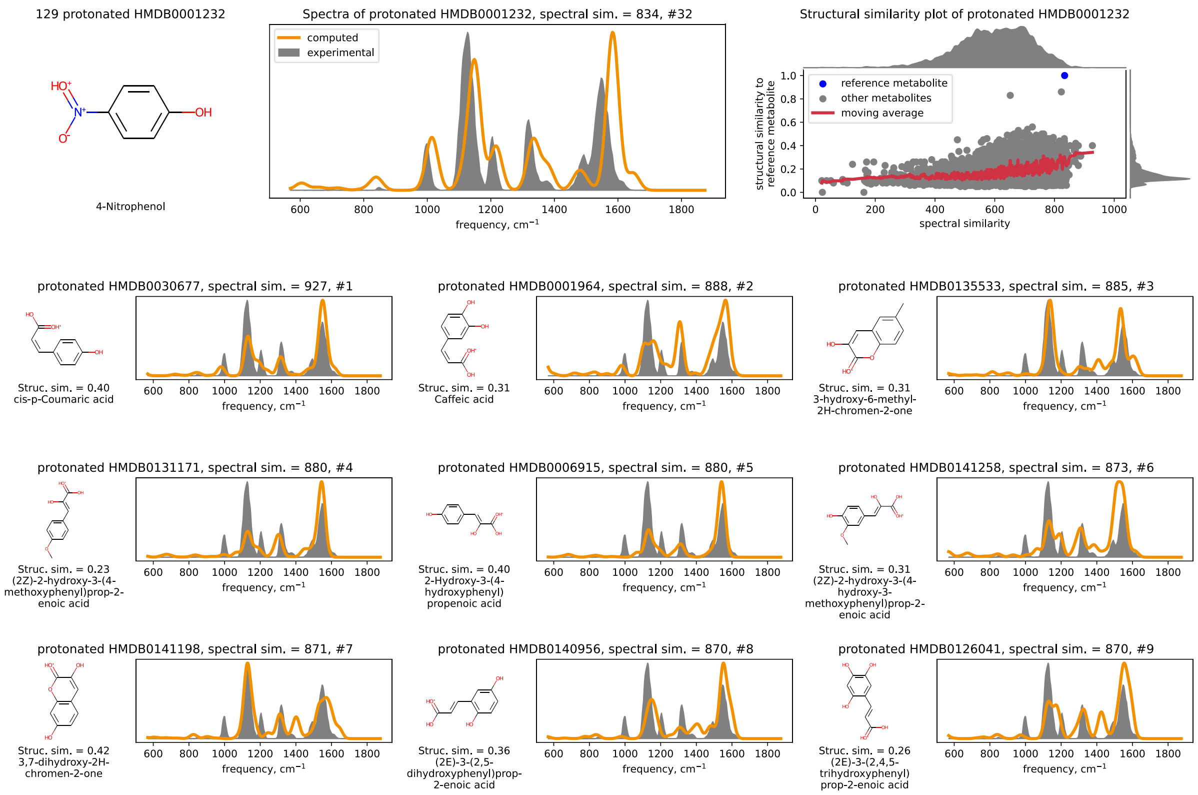

129 protonated HMDB0001232
4-Nitrophenol
Spectra of protonated HMDB0001232, spectral sim. = 834, #32
computed
experimental
frequency, cm⁻¹
Structural similarity plot of protonated HMDB0001232
reference metabolite
other metabolites
moving average
structural similarity to reference metabolite
spectral similarity
protonated HMDB0030677, spectral sim. = 927, #1
Struc. sim. = 0.40
cis-p-Coumaric acid
protonated HMDB0001964, spectral sim. = 888, #2
Struc. sim. = 0.31
Caffeic acid
protonated HMDB0135533, spectral sim. = 885, #3
Struc. sim. = 0.31
3-hydroxy-6-methyl-2H-chromen-2-one
protonated HMDB0131171, spectral sim. = 880, #4
Struc. sim. = 0.23
(2Z)-2-hydroxy-3-(4-methoxyphenyl)prop-2-enoic acid
protonated HMDB0006915, spectral sim. = 880, #5
Struc. sim. = 0.40
2-Hydroxy-3-(4-hydroxyphenyl) propenoic acid
protonated HMDB0141258, spectral sim. = 873, #6
Struc. sim. = 0.31
(2Z)-2-hydroxy-3-(4-hydroxy-3-methoxyphenyl)prop-2-enoic acid
protonated HMDB0141198, spectral sim. = 871, #7
Struc. sim. = 0.42
3,7-dihydroxy-2H-chromen-2-one
protonated HMDB0140956, spectral sim. = 870, #8
Struc. sim. = 0.36
(2E)-3-(2,5-dihydroxyphenyl)prop-2-enoic acid
protonated HMDB0126041, spectral sim. = 870, #9
Struc. sim. = 0.26
(2E)-3-(2,4,5-trihydroxyphenyl) prop-2-enoic acid

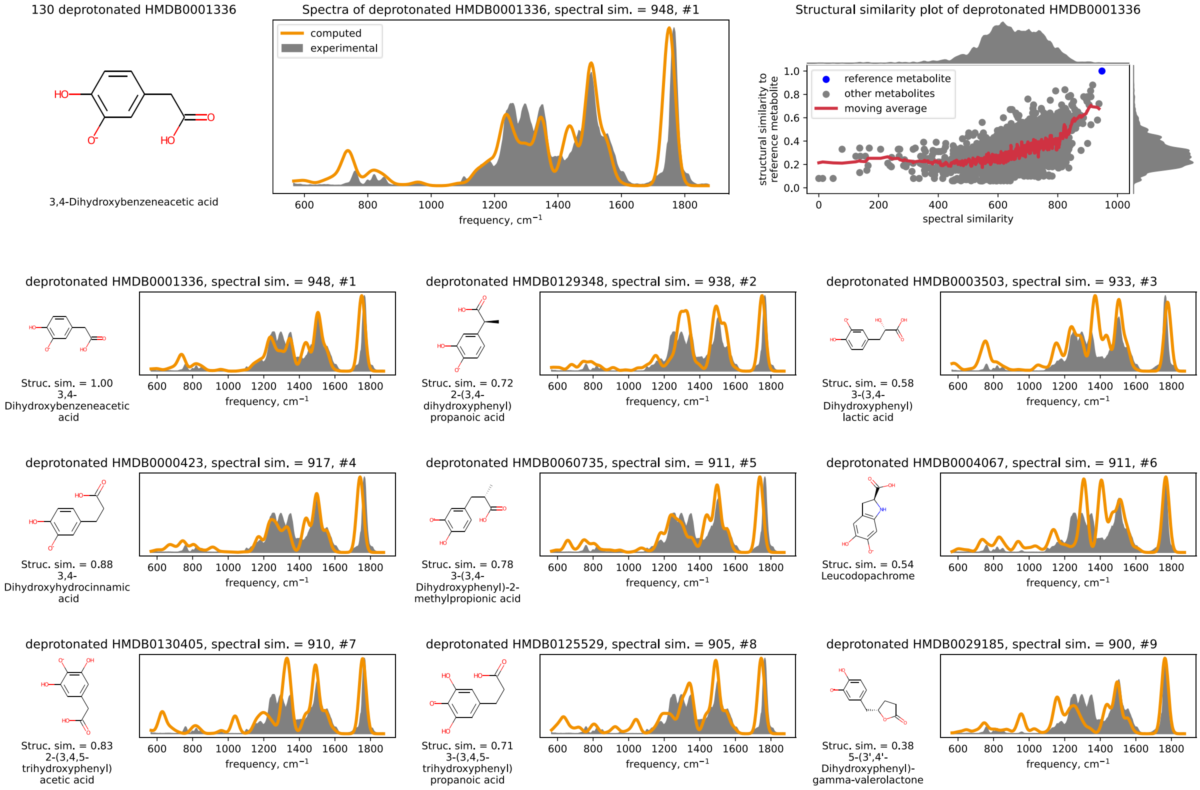

130 deprotonated HMDB0001336
3,4-Dihydroxybenzeneacetic acid
Spectra of deprotonated HMDB0001336, spectral sim. = 948, #1
computed
experimental
frequency, cm⁻¹
Structural similarity plot of deprotonated HMDB0001336
reference metabolite
other metabolites
moving average
structural similarity to reference metabolite
spectral similarity
deprotonated HMDB0001336, spectral sim. = 948, #1
Struc. sim. = 1.00
3,4-Dihydroxybenzeneacetic acid
deprotonated HMDB0129348, spectral sim. = 938, #2
Struc. sim. = 0.72
2-(3,4-dihydroxyphenyl) propanoic acid
deprotonated HMDB0003503, spectral sim. = 933, #3
Struc. sim. = 0.58
3-(3,4-Dihydroxyphenyl) lactic acid
deprotonated HMDB0000423, spectral sim. = 917, #4
Struc. sim. = 0.88
3,4-Dihydroxyhydrocinnamic acid
deprotonated HMDB0060735, spectral sim. = 911, #5
Struc. sim. = 0.78
3-(3,4-Dihydroxyphenyl)-2-methylpropionic acid
deprotonated HMDB0004067, spectral sim. = 911, #6
Struc. sim. = 0.54
Leucodopachrome
deprotonated HMDB0130405, spectral sim. = 910, #7
Struc. sim. = 0.83
2-(3,4,5-trihydroxyphenyl) acetic acid
deprotonated HMDB0125529, spectral sim. = 905, #8
Struc. sim. = 0.71
3-(3,4,5-trihydroxyphenyl) propanoic acid
deprotonated HMDB0029185, spectral sim. = 900, #9
Struc. sim. = 0.38
5-(3',4'-Dihydroxyphenyl)-gamma-valerolactone

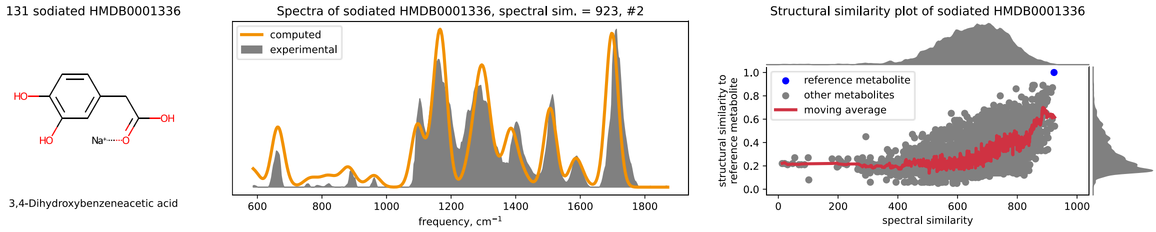
131 sodiated HMDB0001336
3,4-Dihydroxybenzeneacetic acid
Spectra of sodiated HMDB0001336, spectral sim. = 923, #2
computed
experimental
frequency, cm⁻¹
Structural similarity plot of sodiated HMDB0001336
reference metabolite
other metabolites
moving average
structural similarity to reference metabolite
spectral similarity

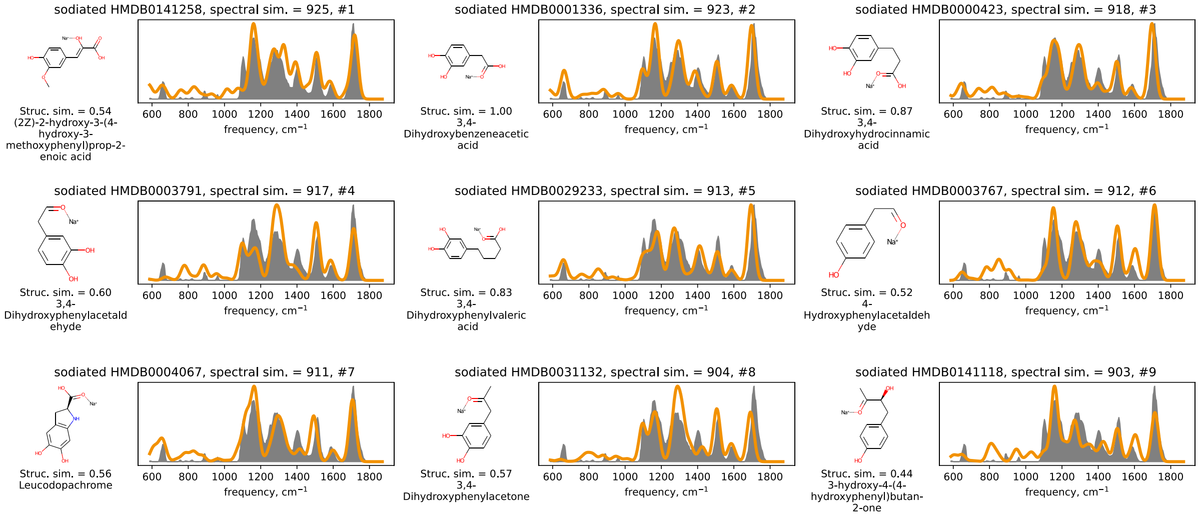
sodiated HMDB0141258, spectral sim. = 925, #1
Struc. sim. = 0.54
(2Z)-2-hydroxy-3-(4-hydroxy-3-methoxyphenyl)prop-2-enoic acid
sodiated HMDB0001336, spectral sim. = 923, #2
Struc. sim. = 1.00
3,4-Dihydroxybenzeneacetic acid
sodiated HMDB0000423, spectral sim. = 918, #3
Struc. sim. = 0.87
3,4-Dihydroxyhydrocinnamic acid
sodiated HMDB0003791, spectral sim. = 917, #4
Struc. sim. = 0.60
3,4-Dihydroxyphenylacetaldehyde
sodiated HMDB0029233, spectral sim. = 913, #5
Struc. sim. = 0.83
3,4-Dihydroxyphenylvaleric acid
sodiated HMDB0003767, spectral sim. = 912, #6
Struc. sim. = 0.52
4-Hydroxyphenylacetaldehyde
sodiated HMDB0004067, spectral sim. = 911, #7
Struc. sim. = 0.56
Leucodopachrome
sodiated HMDB0031132, spectral sim. = 904, #8
Struc. sim. = 0.57
3,4-Dihydroxyphenylacetone
sodiated HMDB0141118, spectral sim. = 903, #9
Struc. sim. = 0.44
3-hydroxy-4-(4-hydroxyphenyl)butan-2-one
frequency, cm⁻¹

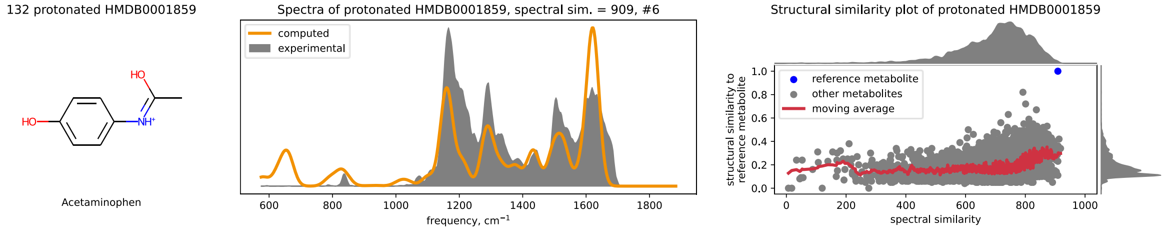
132 protonated HMDB0001859
Acetaminophen
Spectra of protonated HMDB0001859, spectral sim. = 909, #6
computed
experimental
frequency, cm⁻¹
Structural similarity plot of protonated HMDB0001859
reference metabolite
other metabolites
moving average
structural similarity to reference metabolite
spectral similarity

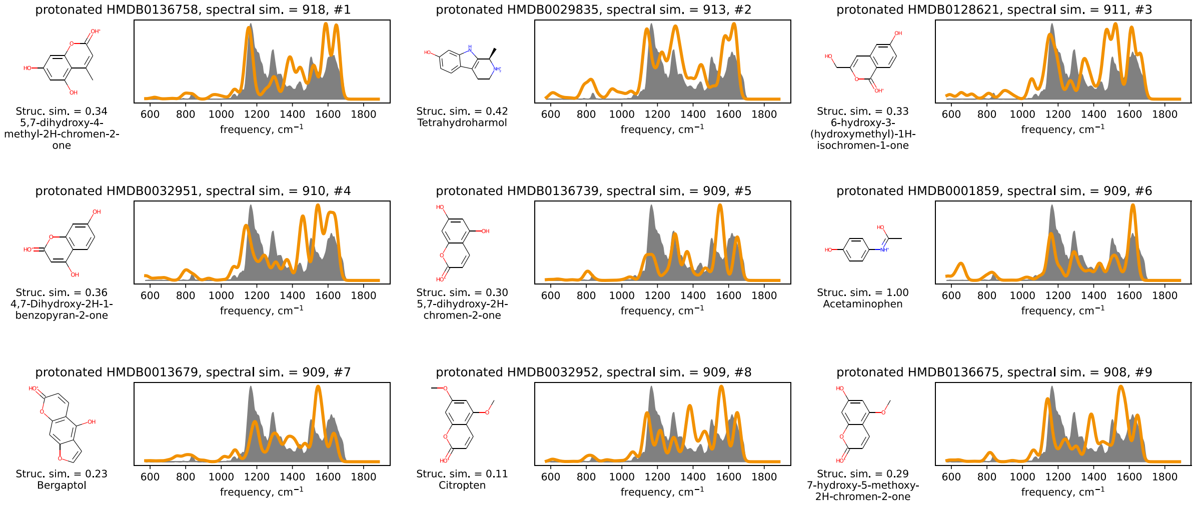
protonated HMDB0136758, spectral sim. = 918, #1
Struc. sim. = 0.34
5,7-dihydroxy-4-methyl-2H-chromen-2-one
protonated HMDB0029835, spectral sim. = 913, #2
Struc. sim. = 0.42
Tetrahydroharmol
protonated HMDB0128621, spectral sim. = 911, #3
Struc. sim. = 0.33
6-hydroxy-3-(hydroxymethyl)-1H-isochromen-1-one
protonated HMDB0032951, spectral sim. = 910, #4
Struc. sim. = 0.36
4,7-Dihydroxy-2H-1-benzopyran-2-one
protonated HMDB0136739, spectral sim. = 909, #5
Struc. sim. = 0.30
5,7-dihydroxy-2H-chromen-2-one
protonated HMDB0001859, spectral sim. = 909, #6
Struc. sim. = 1.00
Acetaminophen
protonated HMDB0013679, spectral sim. = 909, #7
Struc. sim. = 0.23
Bergaptol
protonated HMDB0032952, spectral sim. = 909, #8
Struc. sim. = 0.11
Citropten
protonated HMDB0136675, spectral sim. = 908, #9
Struc. sim. = 0.29
7-hydroxy-5-methoxy-2H-chromen-2-one
frequency, cm⁻¹

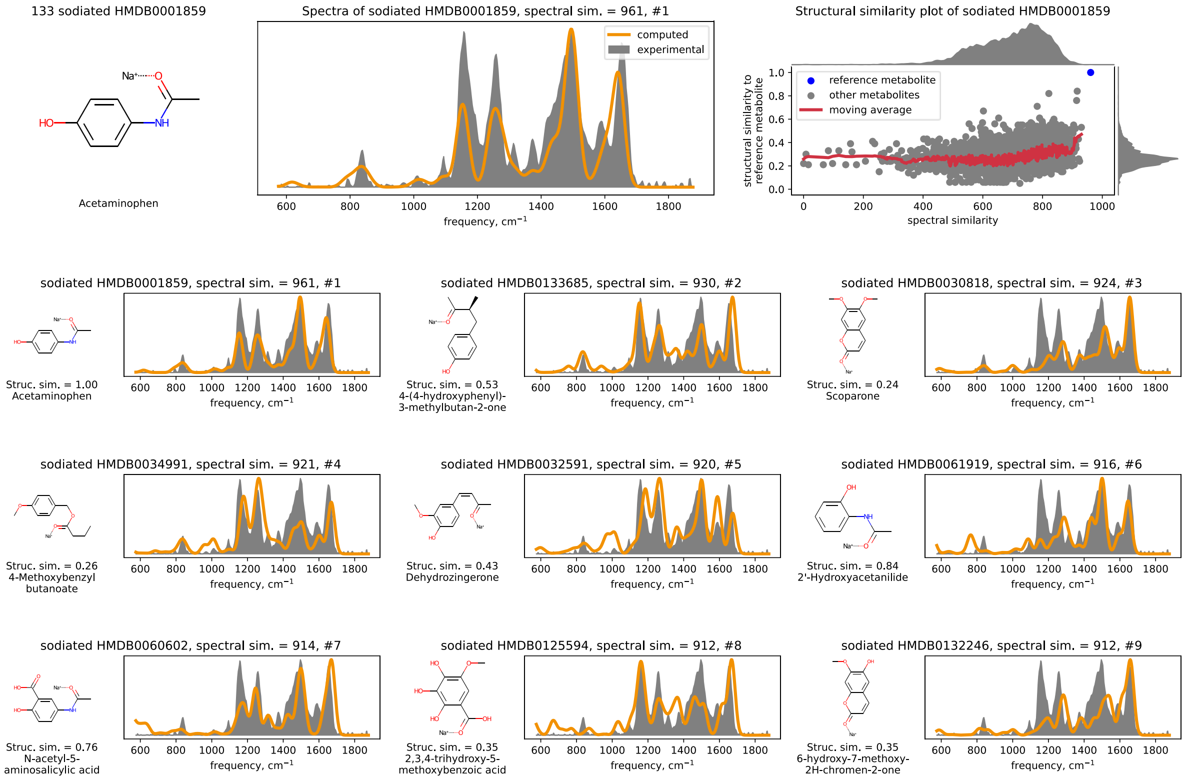
133 sodiated HMDB0001859
Acetaminophen
Spectra of sodiated HMDB0001859, spectral sim. = 961, #1
computed
experimental
frequency, cm⁻¹
Structural similarity plot of sodiated HMDB0001859
structural similarity to reference metabolite
reference metabolite
other metabolites
moving average
spectral similarity
sodiated HMDB0001859, spectral sim. = 961, #1
Struc. sim. = 1.00
Acetaminophen
sodiated HMDB0133685, spectral sim. = 930, #2
Struc. sim. = 0.53
4-(4-hydroxyphenyl)-3-methylbutan-2-one
sodiated HMDB0030818, spectral sim. = 924, #3
Struc. sim. = 0.24
Scoparone
sodiated HMDB0034991, spectral sim. = 921, #4
Struc. sim. = 0.26
4-Methoxybenzyl butanoate
sodiated HMDB0032591, spectral sim. = 920, #5
Struc. sim. = 0.43
Dehydrozingerone
sodiated HMDB0061919, spectral sim. = 916, #6
Struc. sim. = 0.84
2'-Hydroxyacetanilide
sodiated HMDB0060602, spectral sim. = 914, #7
Struc. sim. = 0.76
N-acetyl-5-aminosalicylic acid
sodiated HMDB0125594, spectral sim. = 912, #8
Struc. sim. = 0.35
2,3,4-trihydroxy-5-methoxybenzoic acid
sodiated HMDB0132246, spectral sim. = 912, #9
Struc. sim. = 0.35
6-hydroxy-7-methoxy-2H-chromen-2-one

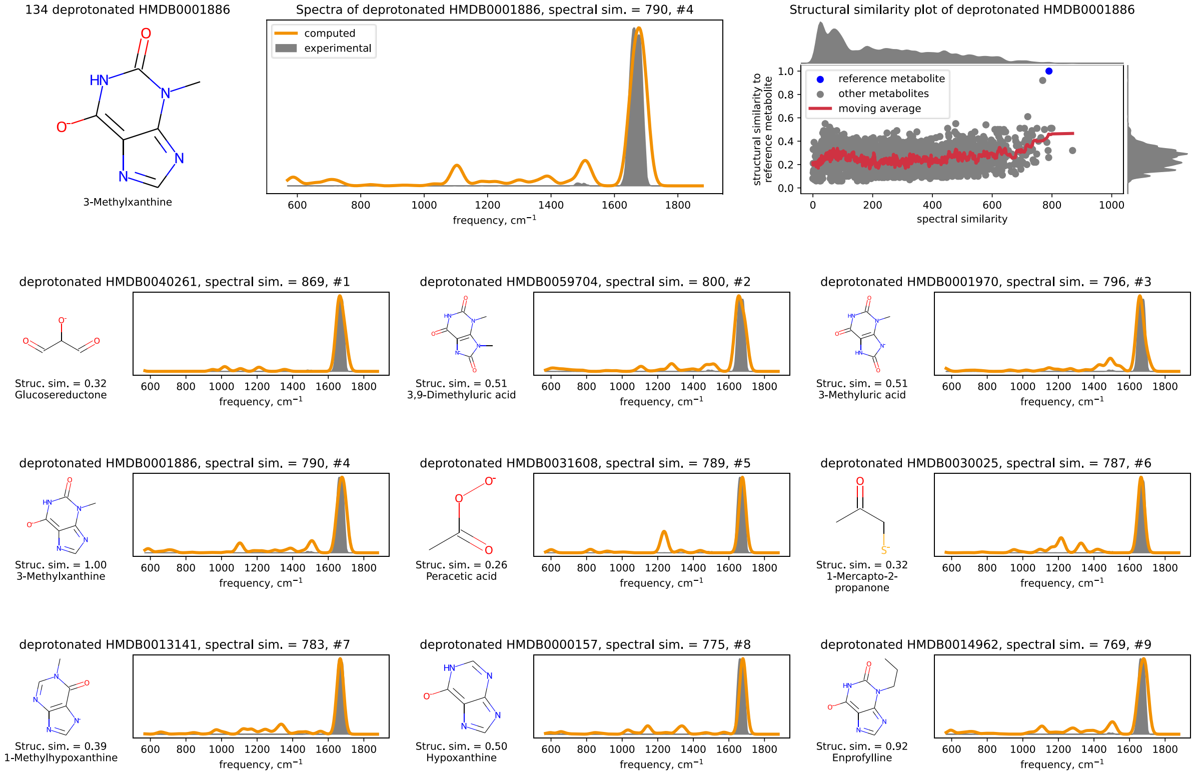
134 deprotonated HMDB0001886
3-Methylxanthine
Spectra of deprotonated HMDB0001886, spectral sim. = 790, #4
computed
experimental
frequency, cm⁻¹
Structural similarity plot of deprotonated HMDB0001886
structural similarity to reference metabolite
reference metabolite
other metabolites
moving average
spectral similarity
deprotonated HMDB0040261, spectral sim. = 869, #1
Struc. sim. = 0.32
Glucosereductone
deprotonated HMDB0059704, spectral sim. = 800, #2
Struc. sim. = 0.51
3,9-Dimethyluric acid
deprotonated HMDB0001970, spectral sim. = 796, #3
Struc. sim. = 0.51
3-Methyluric acid
deprotonated HMDB0001886, spectral sim. = 790, #4
Struc. sim. = 1.00
3-Methylxanthine
deprotonated HMDB0031608, spectral sim. = 789, #5
Struc. sim. = 0.26
Peracetic acid
deprotonated HMDB0030025, spectral sim. = 787, #6
Struc. sim. = 0.32
1-Mercapto-2-propanone
deprotonated HMDB0013141, spectral sim. = 783, #7
Struc. sim. = 0.39
1-Methylhypoxanthine
deprotonated HMDB0000157, spectral sim. = 775, #8
Struc. sim. = 0.50
Hypoxanthine
deprotonated HMDB0014962, spectral sim. = 769, #9
Struc. sim. = 0.92
Enprofylline

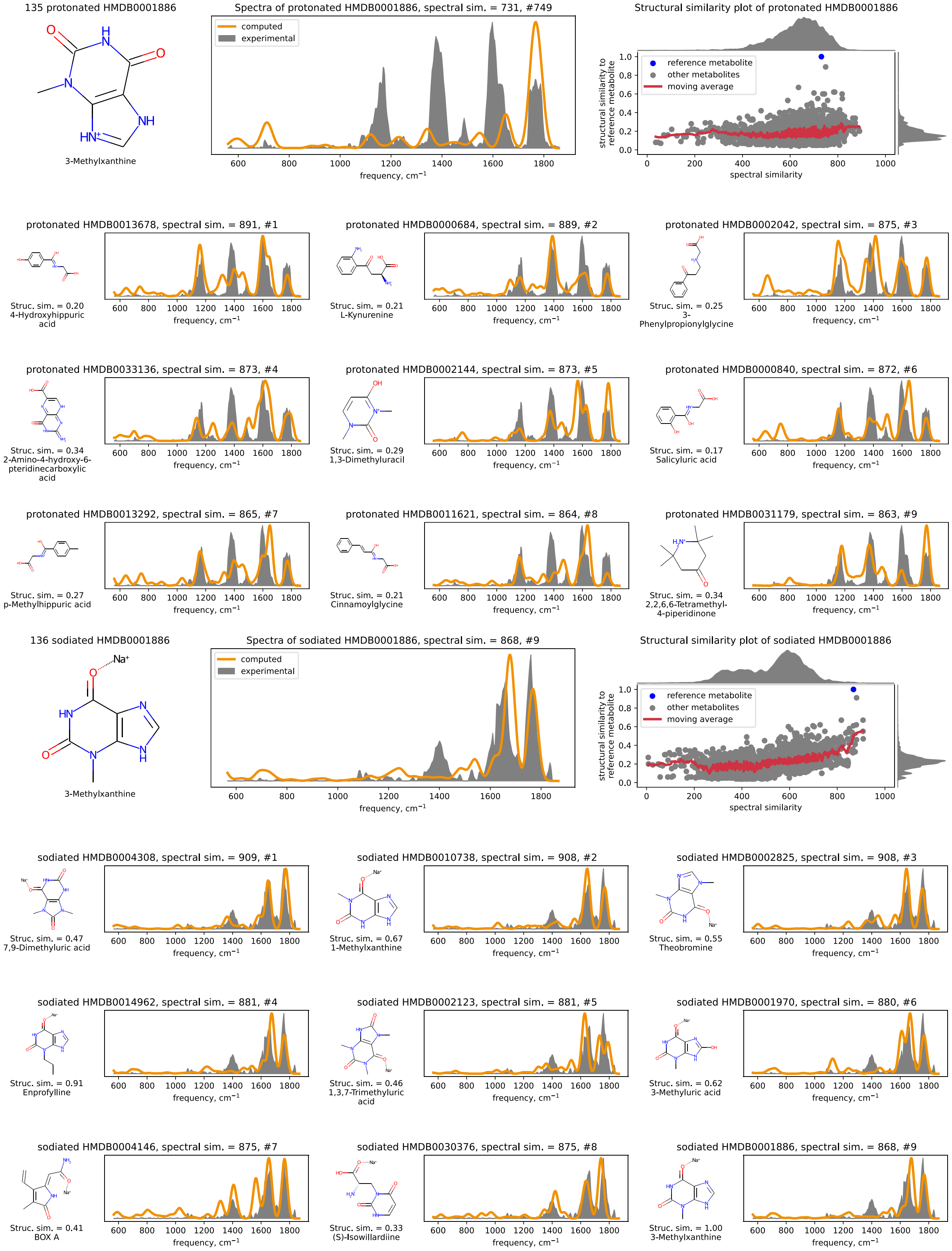

135 protonated HMDB0001886
3-Methylxanthine
Spectra of protonated HMDB0001886, spectral sim. = 731, #749
computed
experimental
frequency, cm^{-1}
Structural similarity plot of protonated HMDB0001886
reference metabolite
other metabolites
moving average
structural similarity to reference metabolite
spectral similarity
protonated HMDB0013678, spectral sim. = 891, #1
Struc. sim. = 0.20
4-Hydroxyhippuric acid
protonated HMDB0000684, spectral sim. = 889, #2
Struc. sim. = 0.21
L-Kynurenine
protonated HMDB0002042, spectral sim. = 875, #3
Struc. sim. = 0.25
3-Phenylpropionylglycine
protonated HMDB0033136, spectral sim. = 873, #4
Struc. sim. = 0.34
2-Amino-4-hydroxy-6-pteridinecarboxylic acid
protonated HMDB0002144, spectral sim. = 873, #5
Struc. sim. = 0.29
1,3-Dimethyluracil
protonated HMDB0000840, spectral sim. = 872, #6
Struc. sim. = 0.17
Salicyluric acid
protonated HMDB0013292, spectral sim. = 865, #7
Struc. sim. = 0.27
p-Methylhippuric acid
protonated HMDB0011621, spectral sim. = 864, #8
Struc. sim. = 0.21
Cinnamoylglycine
protonated HMDB0031179, spectral sim. = 863, #9
Struc. sim. = 0.34
2,2,6,6-Tetramethyl-4-piperidinone
136 sodiated HMDB0001886
3-Methylxanthine
Spectra of sodiated HMDB0001886, spectral sim. = 868, #9
Structural similarity plot of sodiated HMDB0001886
sodiated HMDB0004308, spectral sim. = 909, #1
Struc. sim. = 0.47
7,9-Dimethyluric acid
sodiated HMDB0010738, spectral sim. = 908, #2
Struc. sim. = 0.67
1-Methylxanthine
sodiated HMDB0002825, spectral sim. = 908, #3
Struc. sim. = 0.55
Theobromine
sodiated HMDB0014962, spectral sim. = 881, #4
Struc. sim. = 0.91
Enprofylline
sodiated HMDB0002123, spectral sim. = 881, #5
Struc. sim. = 0.46
1,3,7-Trimethyluric acid
sodiated HMDB0001970, spectral sim. = 880, #6
Struc. sim. = 0.62
3-Methyluric acid
sodiated HMDB0004146, spectral sim. = 875, #7
Struc. sim. = 0.41
BOX A
sodiated HMDB0030376, spectral sim. = 875, #8
Struc. sim. = 0.33
(S)-Isowillardiine
sodiated HMDB0001886, spectral sim. = 868, #9
Struc. sim. = 1.00
3-Methylxanthine

137 deprotonated HMDB0001890

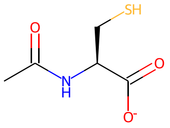

Acetylcysteine

Spectra of deprotonated HMDB0001890, spectral sim. = 915, #4

Structural similarity plot of deprotonated HMDB0001890

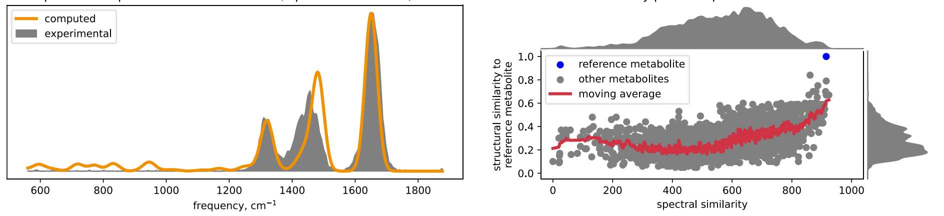

deprotonated HMDB0000766, spectral sim. = 924, #1

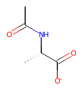

Struc. sim. = 0.67
N-Acetyl-L-alanine

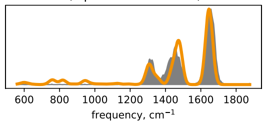

deprotonated HMDB0011757, spectral sim. = 915, #2

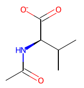

Struc. sim. = 0.79
N-Acetylvaline

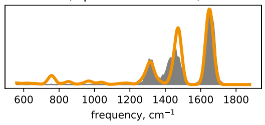

deprotonated HMDB0000532, spectral sim. = 915, #3

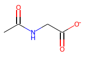

Struc. sim. = 0.67
Acetylglycine

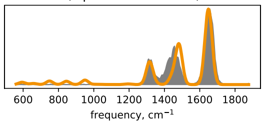

deprotonated HMDB0001890, spectral sim. = 915, #4

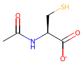

Struc. sim. = 1.00
Acetylcysteine

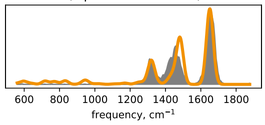

deprotonated HMDB0011745, spectral sim. = 913, #5

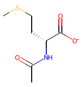

Struc. sim. = 0.70
N-Acetyl-L-methionine

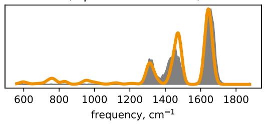

deprotonated HMDB0000459, spectral sim. = 910, #6

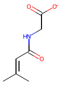

Struc. sim. = 0.55
3-
Methylcrotonylglycine

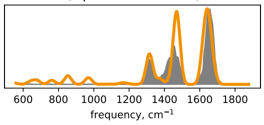

deprotonated HMDB0000747, spectral sim. = 908, #7

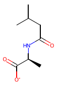

Struc. sim. = 0.55
Isovalerylalanine

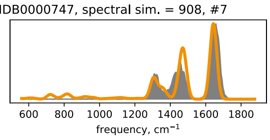

deprotonated HMDB0000894, spectral sim. = 908, #8

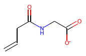

Struc. sim. = 0.50
Vinylacetylglycine

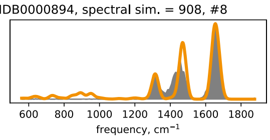

deprotonated HMDB0006029, spectral sim. = 908, #9

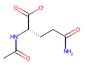

Struc. sim. = 0.59
N-Acetylglutamine

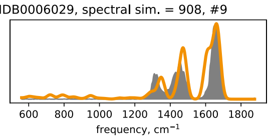

138 protonated HMDB0001890

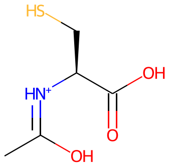

Acetylcysteine

Spectra of protonated HMDB0001890, spectral sim. = 808, #413

Structural similarity plot of protonated HMDB0001890

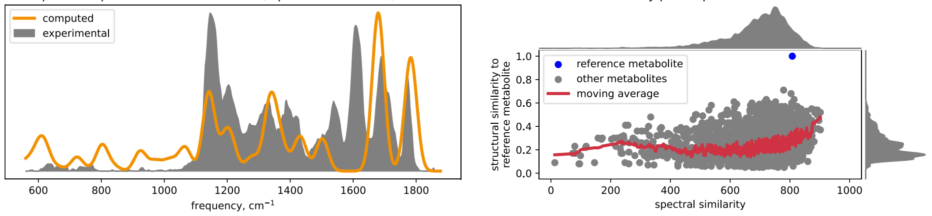

protonated HMDB0000735, spectral sim. = 902, #1

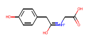

Struc. sim. = 0.52
Hydroxyphenylacetylgly
cine

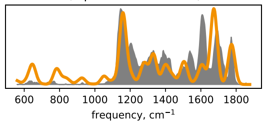

protonated HMDB0028797, spectral sim. = 902, #2

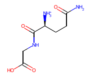

Struc. sim. = 0.37
Glutaminylglycine

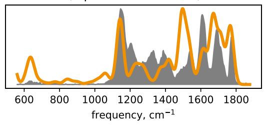

protonated HMDB0131190, spectral sim. = 897, #3

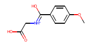

Struc. sim. = 0.45
2-[(4-
methoxyphenyl)
formamido]acetic acid

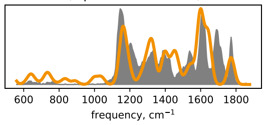

protonated HMDB0028731, spectral sim. = 894, #4

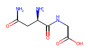

Struc. sim. = 0.38
Asparaginyl-Glycine

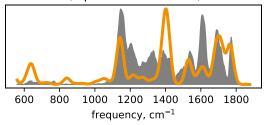

protonated HMDB0013678, spectral sim. = 892, #5

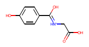

Struc. sim. = 0.54
4-Hydroxyhippuric
acid

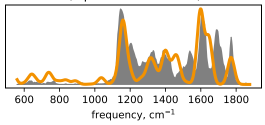

protonated HMDB0060602, spectral sim. = 891, #6

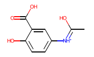

Struc. sim. = 0.50
N-acetyl-5-
aminosalicylic acid

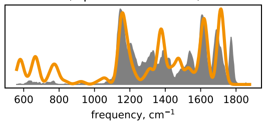

protonated HMDB0031813, spectral sim. = 888, #7

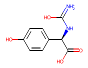

Struc. sim. = 0.52
N-Carbamoyl-2-amino-
2-(4-
hydroxyphenyl)acetic

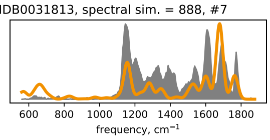

protonated HMDB0029423, spectral sim. = 883, #8

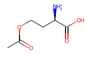

Struc. sim. = 0.52
Acetylhomoserine

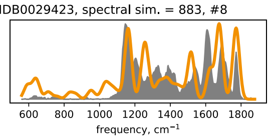

protonated HMDB0006116, spectral sim. = 883, #9

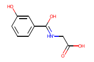

Struc. sim. = 0.54
3-Hydroxyhippuric
acid

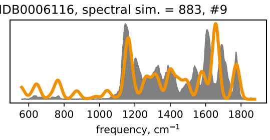

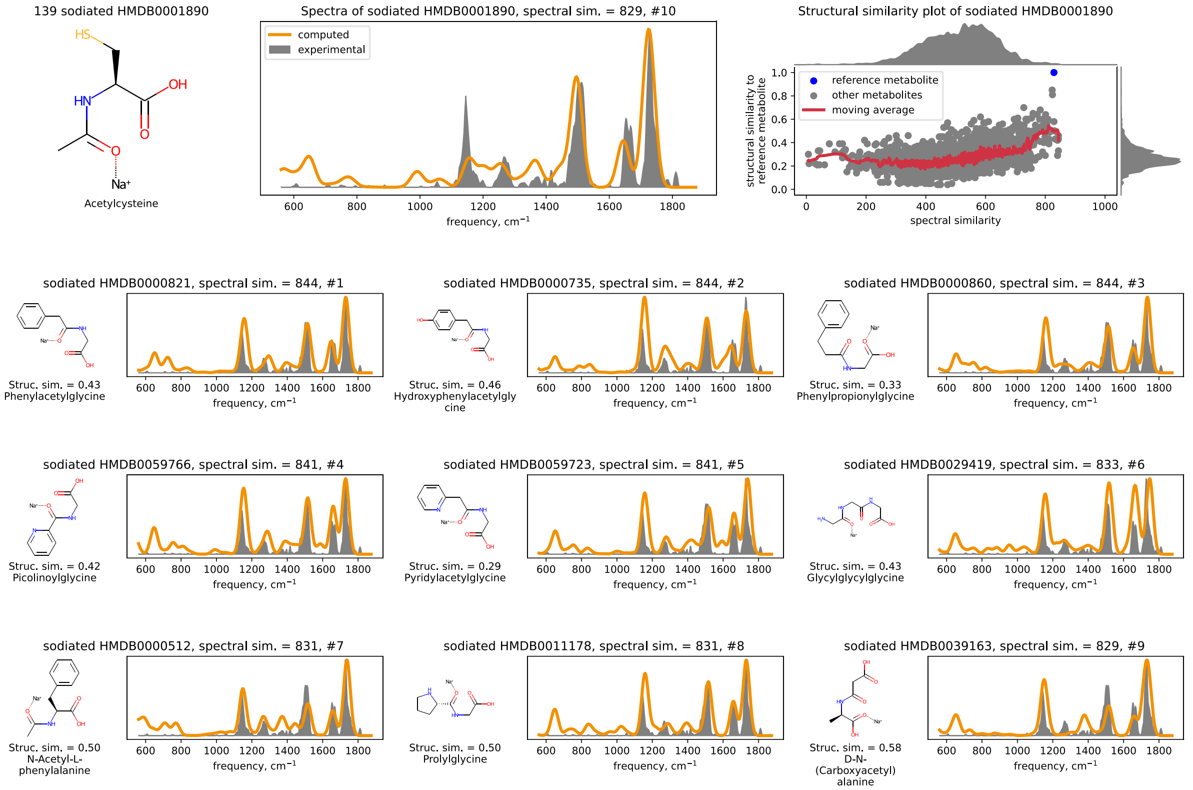
139 sodiated HMDB0001890
HS
HN
OH
O
O
Na+
Acetylcysteine
Spectra of sodiated HMDB0001890, spectral sim. = 829, #10
computed
experimental
frequency, cm⁻¹
Structural similarity plot of sodiated HMDB0001890
structural similarity to reference metabolite
reference metabolite
other metabolites
moving average
spectral similarity
sodiated HMDB0000821, spectral sim. = 844, #1
Struc. sim. = 0.43
Phenylacetylglycine
sodiated HMDB0000735, spectral sim. = 844, #2
Struc. sim. = 0.46
Hydroxyphenylacetylglycine
sodiated HMDB0000860, spectral sim. = 844, #3
Struc. sim. = 0.33
Phenylpropionylglycine
sodiated HMDB0059766, spectral sim. = 841, #4
Struc. sim. = 0.42
Picolinoylglycine
sodiated HMDB0059723, spectral sim. = 841, #5
Struc. sim. = 0.29
Pyridylacetylglycine
sodiated HMDB0029419, spectral sim. = 833, #6
Struc. sim. = 0.43
Glycylglycylglycine
sodiated HMDB0000512, spectral sim. = 831, #7
Struc. sim. = 0.50
N-Acetyl-L-phenylalanine
sodiated HMDB0011178, spectral sim. = 831, #8
Struc. sim. = 0.50
Prolylglycine
sodiated HMDB0039163, spectral sim. = 829, #9
Struc. sim. = 0.58
D-N-(Carboxyacetyl)alanine
frequency, cm⁻¹

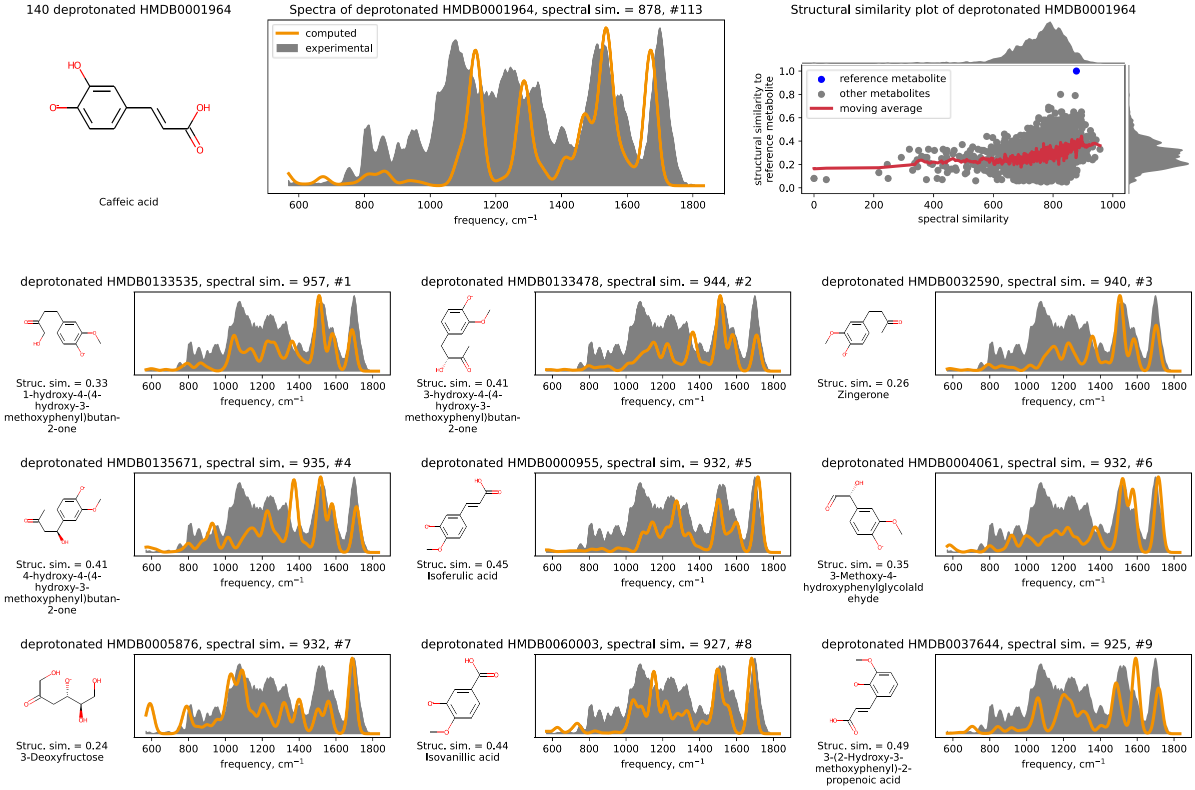
140 deprotonated HMDB0001964
HO
O-
OH
O
Caffeic acid
Spectra of deprotonated HMDB0001964, spectral sim. = 878, #113
computed
experimental
frequency, cm⁻¹
Structural similarity plot of deprotonated HMDB0001964
structural similarity to reference metabolite
reference metabolite
other metabolites
moving average
spectral similarity
deprotonated HMDB0133535, spectral sim. = 957, #1
Struc. sim. = 0.33
1-hydroxy-4-(4-hydroxy-3-methoxyphenyl)butan-2-one
deprotonated HMDB0133478, spectral sim. = 944, #2
Struc. sim. = 0.41
3-hydroxy-4-(4-hydroxy-3-methoxyphenyl)butan-2-one
deprotonated HMDB0032590, spectral sim. = 940, #3
Struc. sim. = 0.26
Zingerone
deprotonated HMDB0135671, spectral sim. = 935, #4
Struc. sim. = 0.41
4-hydroxy-4-(4-hydroxy-3-methoxyphenyl)butan-2-one
deprotonated HMDB0000955, spectral sim. = 932, #5
Struc. sim. = 0.45
Isoferulic acid
deprotonated HMDB0004061, spectral sim. = 932, #6
Struc. sim. = 0.35
3-Methoxy-4-hydroxyphenylglycolaldehyde
deprotonated HMDB0005876, spectral sim. = 932, #7
Struc. sim. = 0.24
3-Deoxyfructose
deprotonated HMDB0060003, spectral sim. = 927, #8
Struc. sim. = 0.44
Isovanillic acid
deprotonated HMDB0037644, spectral sim. = 925, #9
Struc. sim. = 0.49
3-(2-Hydroxy-3-methoxyphenyl)-2-propenoic acid
frequency, cm⁻¹

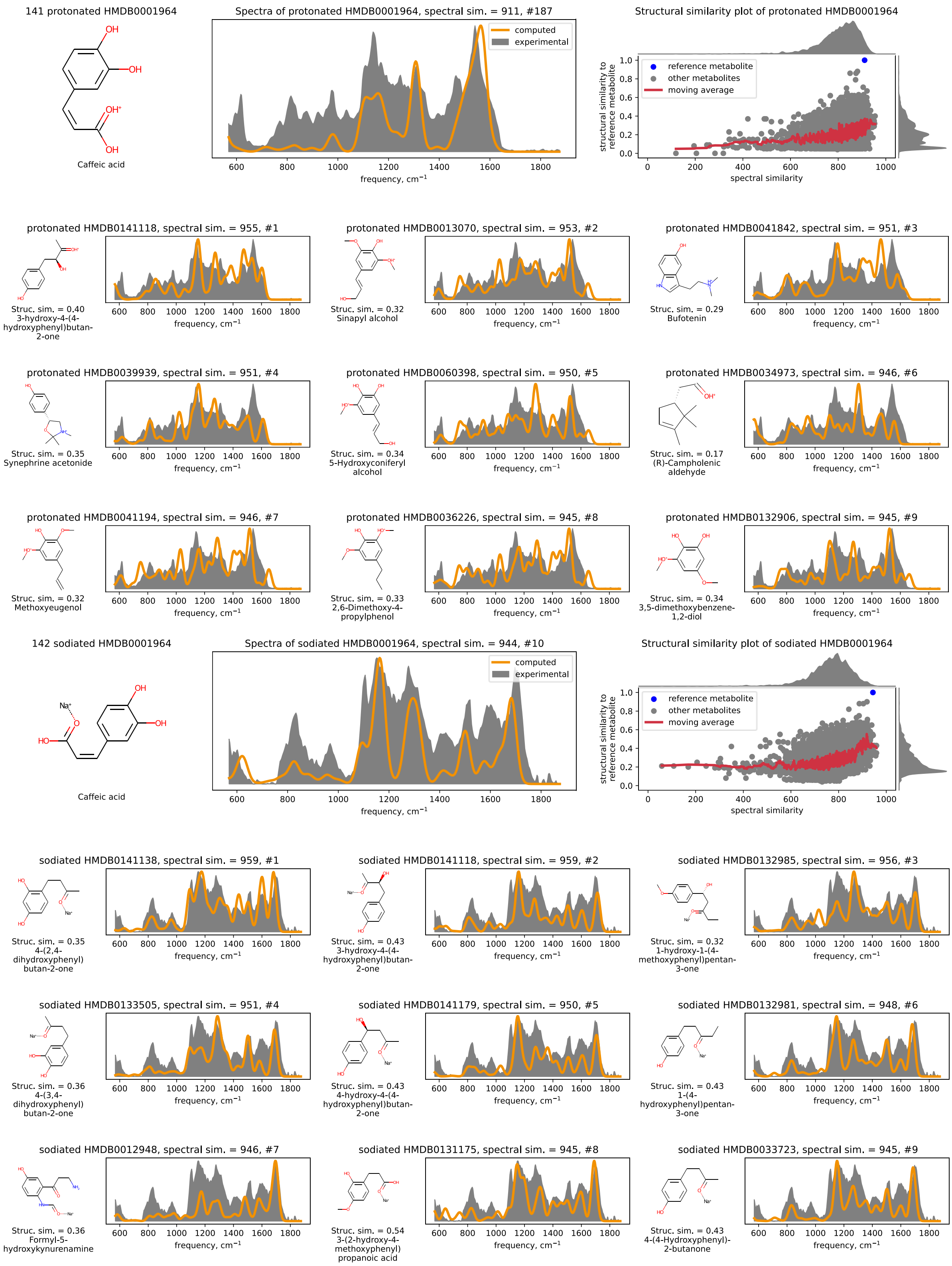
141 protonated HMDB0001964
Caffeic acid
Spectra of protonated HMDB0001964, spectral sim. = 911, #187
computed
experimental
frequency, cm^{-1}
Structural similarity plot of protonated HMDB0001964
reference metabolite
other metabolites
moving average
structural similarity to reference metabolite
spectral similarity
protonated HMDB0141118, spectral sim. = 955, #1
Struc. sim. = 0.40
3-hydroxy-4-(4-hydroxyphenyl)butan-2-one
protonated HMDB0013070, spectral sim. = 953, #2
Struc. sim. = 0.32
Sinapyl alcohol
protonated HMDB0041842, spectral sim. = 951, #3
Struc. sim. = 0.29
Bufotenin
protonated HMDB0039939, spectral sim. = 951, #4
Struc. sim. = 0.35
Synephrine acetonide
protonated HMDB0060398, spectral sim. = 950, #5
Struc. sim. = 0.34
5-Hydroxyconiferyl alcohol
protonated HMDB0034973, spectral sim. = 946, #6
Struc. sim. = 0.17
(R)-Campholenic aldehyde
protonated HMDB0041194, spectral sim. = 946, #7
Struc. sim. = 0.32
Methoxyeugenol
protonated HMDB0036226, spectral sim. = 945, #8
Struc. sim. = 0.33
2,6-Dimethoxy-4-propylphenol
protonated HMDB0132906, spectral sim. = 945, #9
Struc. sim. = 0.34
3,5-dimethoxybenzene-1,2-diol
142 sodiated HMDB0001964
Caffeic acid
Spectra of sodiated HMDB0001964, spectral sim. = 944, #10
computed
experimental
frequency, cm^{-1}
Structural similarity plot of sodiated HMDB0001964
reference metabolite
other metabolites
moving average
structural similarity to reference metabolite
spectral similarity
sodiated HMDB0141138, spectral sim. = 959, #1
Struc. sim. = 0.35
4-(2,4-dihydroxyphenyl)butan-2-one
sodiated HMDB0141118, spectral sim. = 959, #2
Struc. sim. = 0.43
3-hydroxy-4-(4-hydroxyphenyl)butan-2-one
sodiated HMDB0132985, spectral sim. = 956, #3
Struc. sim. = 0.32
1-hydroxy-1-(4-methoxyphenyl)pentan-3-one
sodiated HMDB0133505, spectral sim. = 951, #4
Struc. sim. = 0.36
4-(3,4-dihydroxyphenyl)butan-2-one
sodiated HMDB0141179, spectral sim. = 950, #5
Struc. sim. = 0.43
4-hydroxy-4-(4-hydroxyphenyl)butan-2-one
sodiated HMDB0132981, spectral sim. = 948, #6
Struc. sim. = 0.43
1-(4-hydroxyphenyl)pentan-3-one
sodiated HMDB0012948, spectral sim. = 946, #7
Struc. sim. = 0.36
Formyl-5-hydroxykynurenamine
sodiated HMDB0131175, spectral sim. = 945, #8
Struc. sim. = 0.54
3-(2-hydroxy-4-methoxyphenyl)propanoic acid
sodiated HMDB0033723, spectral sim. = 945, #9
Struc. sim. = 0.43
4-(4-Hydroxyphenyl)-2-butanone

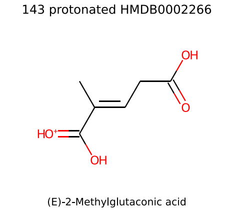
143 protonated HMDB0002266
OH
O
HO+
OH
(E)-2-Methylglutaconic acid

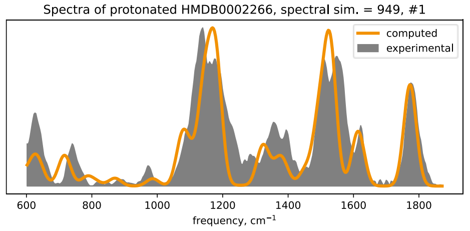
Spectra of protonated HMDB0002266, spectral sim. = 949, #1
computed
experimental
frequency, cm^{-1}

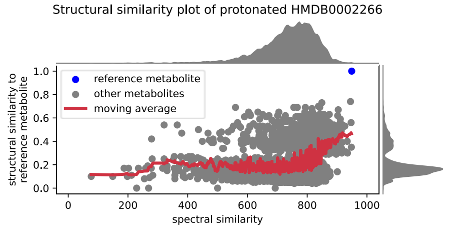
Structural similarity plot of protonated HMDB0002266
reference metabolite
other metabolites
moving average
structural similarity to reference metabolite
spectral similarity

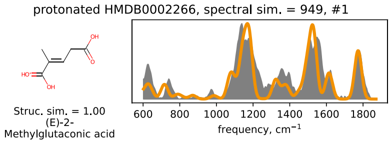
protonated HMDB0002266, spectral sim. = 949, #1
Struc. sim. = 1.00
(E)-2-Methylglutaconic acid
frequency, cm^{-1}

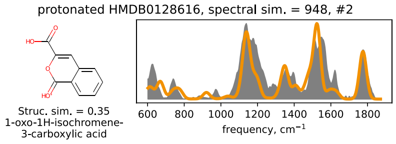
protonated HMDB0128616, spectral sim. = 948, #2
Struc. sim. = 0.35
1-oxo-1H-isochromene-3-carboxylic acid
frequency, cm^{-1}

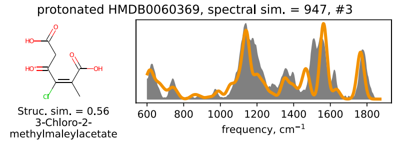
protonated HMDB0060369, spectral sim. = 947, #3
Struc. sim. = 0.56
3-Chloro-2-methylmaleylacetate
frequency, cm^{-1}

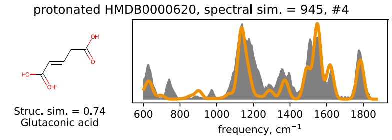
protonated HMDB0000620, spectral sim. = 945, #4
Struc. sim. = 0.74
Glutaconic acid
frequency, cm^{-1}

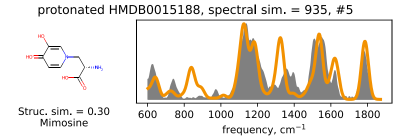
protonated HMDB0015188, spectral sim. = 935, #5
Struc. sim. = 0.30
Mimosine
frequency, cm^{-1}

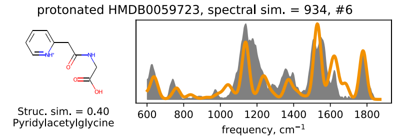
protonated HMDB0059723, spectral sim. = 934, #6
Struc. sim. = 0.40
Pyridylacetylglycine
frequency, cm^{-1}

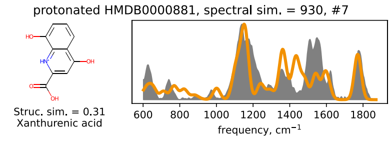
protonated HMDB0000881, spectral sim. = 930, #7
Struc. sim. = 0.31
Xanthurenic acid
frequency, cm^{-1}

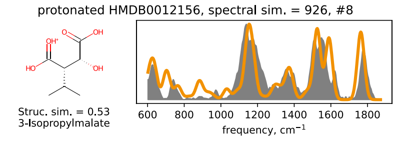
protonated HMDB0012156, spectral sim. = 926, #8
Struc. sim. = 0.53
3-Isopropylmalate
frequency, cm^{-1}

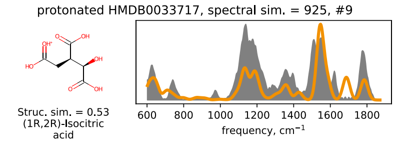
protonated HMDB0033717, spectral sim. = 925, #9
Struc. sim. = 0.53
(1R,2R)-Isocitric acid
frequency, cm^{-1}

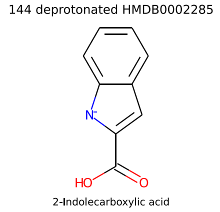
144 deprotonated HMDB0002285
N
HO
O
2-Indolecarboxylic acid

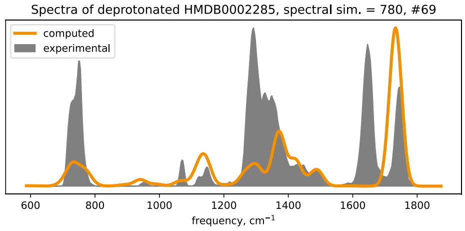
Spectra of deprotonated HMDB0002285, spectral sim. = 780, #69
computed
experimental
frequency, cm^{-1}

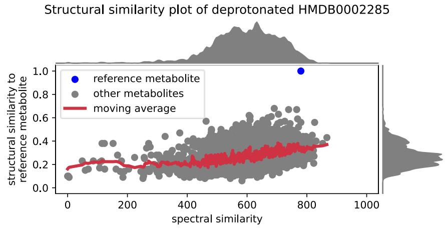
Structural similarity plot of deprotonated HMDB0002285
reference metabolite
other metabolites
moving average
structural similarity to reference metabolite
spectral similarity

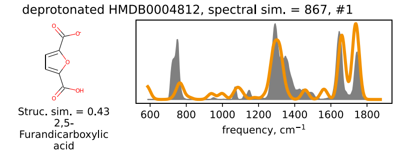
deprotonated HMDB0004812, spectral sim. = 867, #1
Struc. sim. = 0.43
2,5-Furandicarboxylic acid
frequency, cm^{-1}

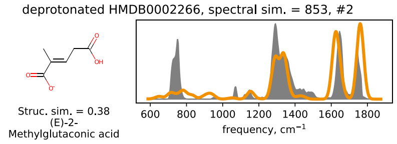
deprotonated HMDB0002266, spectral sim. = 853, #2
Struc. sim. = 0.38
(E)-2-Methylglutaconic acid
frequency, cm^{-1}

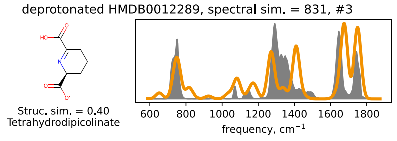
deprotonated HMDB0012289, spectral sim. = 831, #3
Struc. sim. = 0.40
Tetrahydrodipicolinate
frequency, cm^{-1}

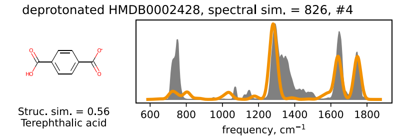
deprotonated HMDB0002428, spectral sim. = 826, #4
Struc. sim. = 0.56
Terephthalic acid
frequency, cm^{-1}

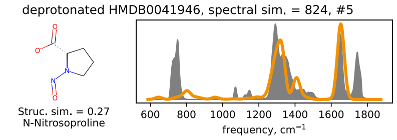
deprotonated HMDB0041946, spectral sim. = 824, #5
Struc. sim. = 0.27
N-Nitrosoproline
frequency, cm^{-1}

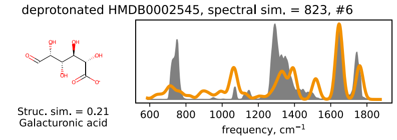
deprotonated HMDB0002545, spectral sim. = 823, #6
Struc. sim. = 0.21
Galacturonic acid
frequency, cm^{-1}

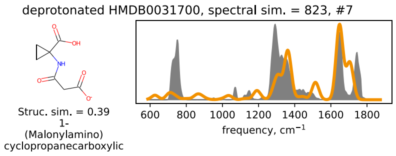
deprotonated HMDB0031700, spectral sim. = 823, #7
Struc. sim. = 0.39
1-(Malonylamino)cyclopropanecarboxylic
frequency, cm^{-1}

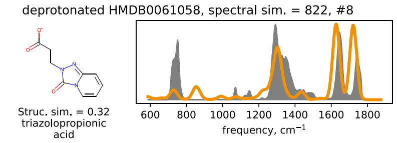
deprotonated HMDB0061058, spectral sim. = 822, #8
Struc. sim. = 0.32
triazolopropionic acid
frequency, cm^{-1}

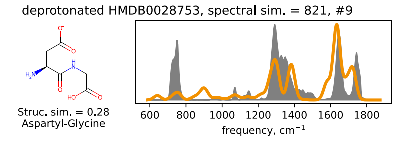
deprotonated HMDB0028753, spectral sim. = 821, #9
Struc. sim. = 0.28
Aspartyl-Glycine
frequency, cm^{-1}

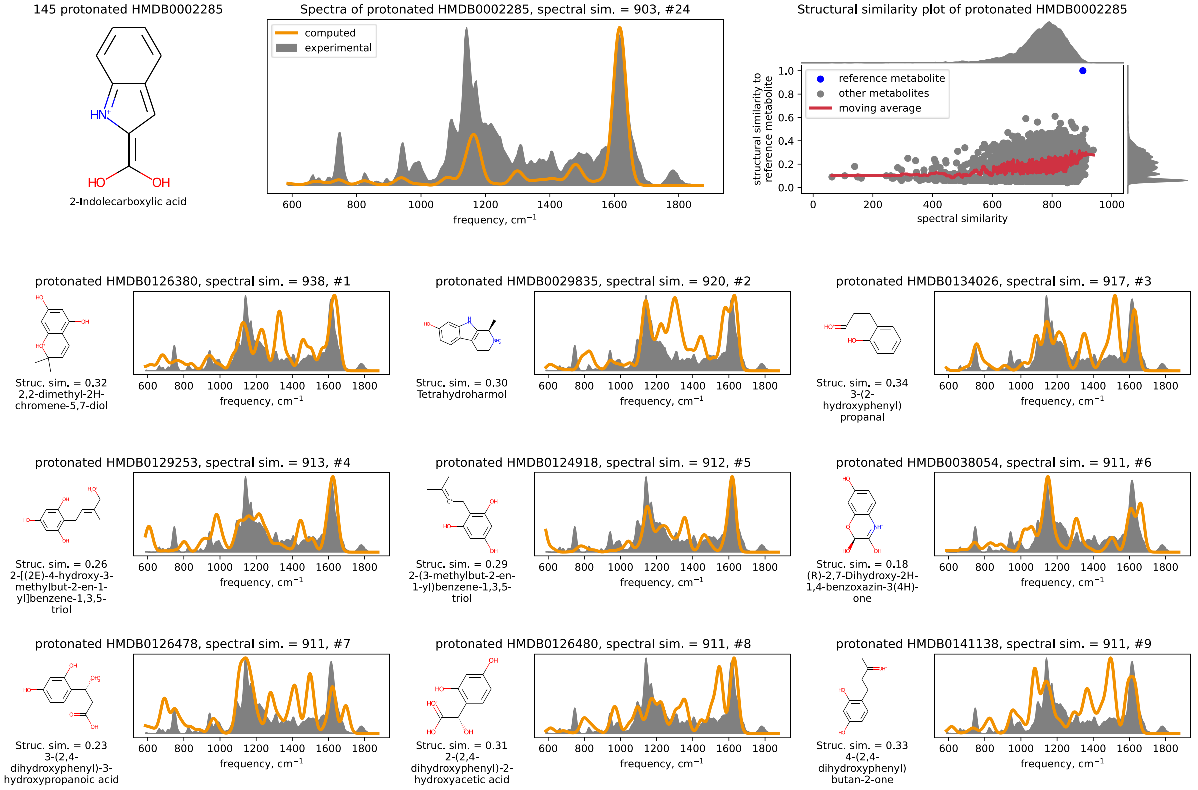
145 protonated HMDB0002285
2-Indolecarboxylic acid
Spectra of protonated HMDB0002285, spectral sim. = 903, #24
computed
experimental
frequency, cm^{-1}
Structural similarity plot of protonated HMDB0002285
reference metabolite
other metabolites
moving average
structural similarity to reference metabolite
spectral similarity
protonated HMDB0126380, spectral sim. = 938, #1
Struc. sim. = 0.32
2,2-dimethyl-2H-chromene-5,7-diol
protonated HMDB0029835, spectral sim. = 920, #2
Struc. sim. = 0.30
Tetrahydroharmol
protonated HMDB0134026, spectral sim. = 917, #3
Struc. sim. = 0.34
3-(2-hydroxyphenyl) propanal
protonated HMDB0129253, spectral sim. = 913, #4
Struc. sim. = 0.26
2-[(2E)-4-hydroxy-3-methylbut-2-en-1-yl]benzene-1,3,5-triol
protonated HMDB0124918, spectral sim. = 912, #5
Struc. sim. = 0.29
2-(3-methylbut-2-en-1-yl)benzene-1,3,5-triol
protonated HMDB0038054, spectral sim. = 911, #6
Struc. sim. = 0.18
(R)-2,7-Dihydroxy-2H-1,4-benzoxazin-3(4H)-one
protonated HMDB0126478, spectral sim. = 911, #7
Struc. sim. = 0.23
3-(2,4-dihydroxyphenyl)-3-hydroxypropanoic acid
protonated HMDB0126480, spectral sim. = 911, #8
Struc. sim. = 0.31
2-(2,4-dihydroxyphenyl)-2-hydroxyacetic acid
protonated HMDB0141138, spectral sim. = 911, #9
Struc. sim. = 0.33
4-(2,4-dihydroxyphenyl) butan-2-one

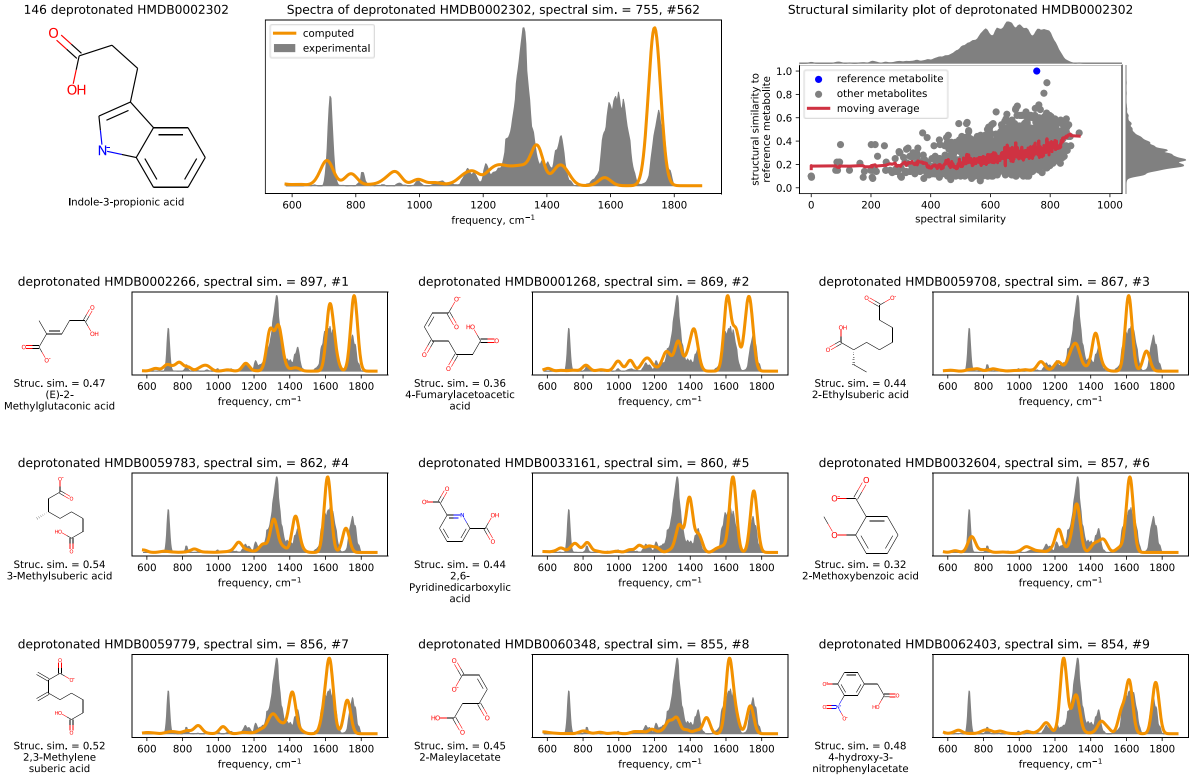
146 deprotonated HMDB0002302
Indole-3-propionic acid
Spectra of deprotonated HMDB0002302, spectral sim. = 755, #562
computed
experimental
frequency, cm^{-1}
Structural similarity plot of deprotonated HMDB0002302
reference metabolite
other metabolites
moving average
structural similarity to reference metabolite
spectral similarity
deprotonated HMDB0002266, spectral sim. = 897, #1
Struc. sim. = 0.47
(E)-2-Methylglutaconic acid
deprotonated HMDB0001268, spectral sim. = 869, #2
Struc. sim. = 0.36
4-Fumarylacetoacetic acid
deprotonated HMDB0059708, spectral sim. = 867, #3
Struc. sim. = 0.44
2-Ethylsuberic acid
deprotonated HMDB0059783, spectral sim. = 862, #4
Struc. sim. = 0.54
3-Methylsuberic acid
deprotonated HMDB0033161, spectral sim. = 860, #5
Struc. sim. = 0.44
2,6-Pyridinedicarboxylic acid
deprotonated HMDB0032604, spectral sim. = 857, #6
Struc. sim. = 0.32
2-Methoxybenzoic acid
deprotonated HMDB0059779, spectral sim. = 856, #7
Struc. sim. = 0.52
2,3-Methylene suberic acid
deprotonated HMDB0060348, spectral sim. = 855, #8
Struc. sim. = 0.45
2-Maleylacetate
deprotonated HMDB0062403, spectral sim. = 854, #9
Struc. sim. = 0.48
4-hydroxy-3-nitrophenylacetate

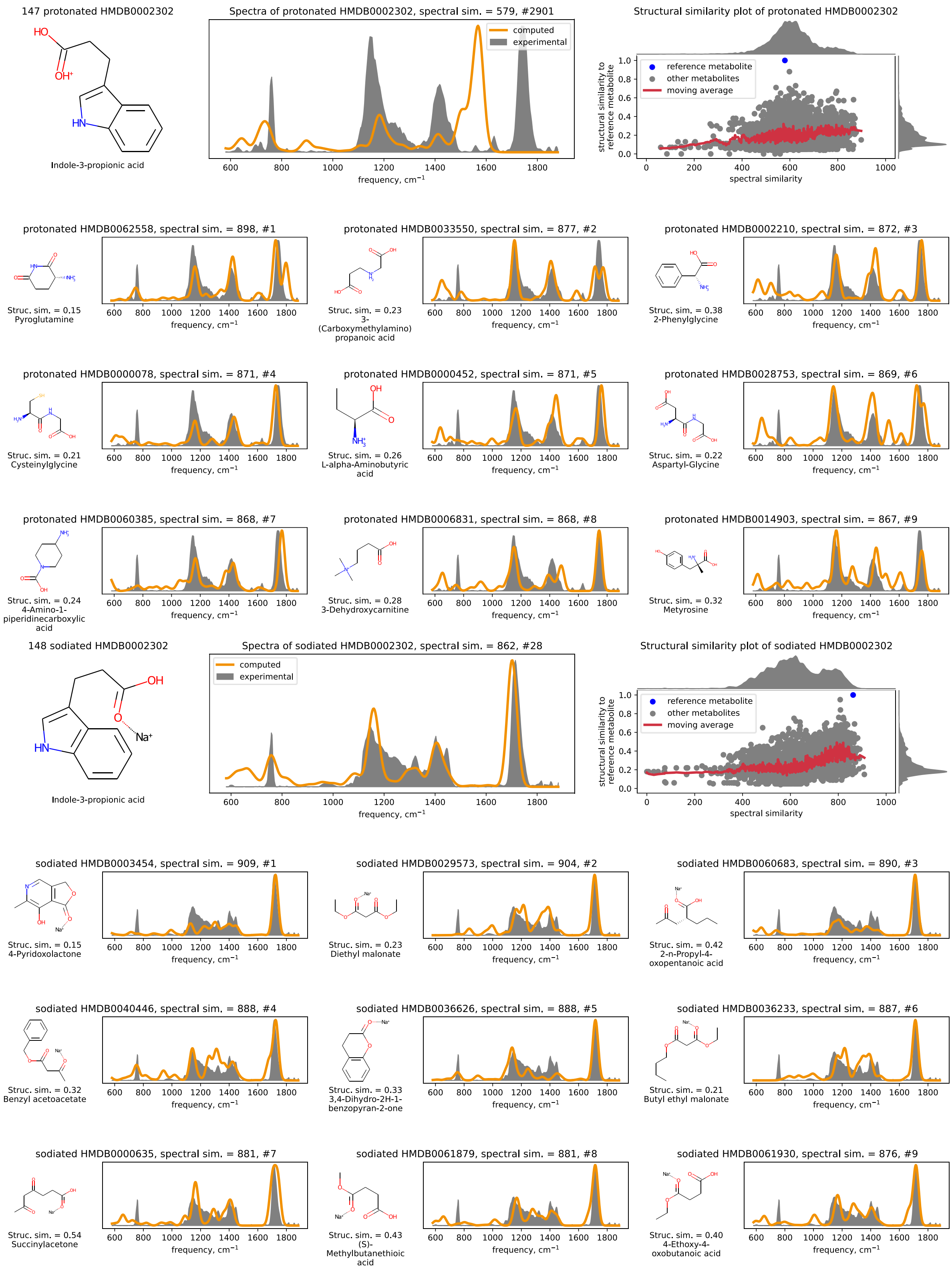
147 protonated HMDB0002302
Indole-3-propionic acid
Spectra of protonated HMDB0002302, spectral sim. = 579, #2901
computed
experimental
frequency, cm⁻¹
Structural similarity plot of protonated HMDB0002302
reference metabolite
other metabolites
moving average
structural similarity to reference metabolite
spectral similarity
protonated HMDB0062558, spectral sim. = 898, #1
Struc. sim. = 0.15
Pyroglutamine
protonated HMDB0033550, spectral sim. = 877, #2
Struc. sim. = 0.23
3-(Carboxymethylamino)propanoic acid
protonated HMDB0002210, spectral sim. = 872, #3
Struc. sim. = 0.38
2-Phenylglycine
protonated HMDB0000078, spectral sim. = 871, #4
Struc. sim. = 0.21
Cysteinylglycine
protonated HMDB0000452, spectral sim. = 871, #5
Struc. sim. = 0.26
L-alpha-Aminobutyric acid
protonated HMDB0028753, spectral sim. = 869, #6
Struc. sim. = 0.22
Aspartyl-Glycine
protonated HMDB0060385, spectral sim. = 868, #7
Struc. sim. = 0.24
4-Amino-1-piperidinecarboxylic acid
protonated HMDB0006831, spectral sim. = 868, #8
Struc. sim. = 0.28
3-Dehydroxycarnitine
protonated HMDB0014903, spectral sim. = 867, #9
Struc. sim. = 0.32
Metyrosine
148 sodiated HMDB0002302
Indole-3-propionic acid
Spectra of sodiated HMDB0002302, spectral sim. = 862, #28
Structural similarity plot of sodiated HMDB0002302
sodiated HMDB0003454, spectral sim. = 909, #1
Struc. sim. = 0.15
4-Pyridoxolactone
sodiated HMDB0029573, spectral sim. = 904, #2
Struc. sim. = 0.23
Diethyl malonate
sodiated HMDB0060683, spectral sim. = 890, #3
Struc. sim. = 0.42
2-n-Propyl-4-oxopentanoic acid
sodiated HMDB0040446, spectral sim. = 888, #4
Struc. sim. = 0.32
Benzyl acetoacetate
sodiated HMDB0036626, spectral sim. = 888, #5
Struc. sim. = 0.33
3,4-Dihydro-2H-1-benzopyran-2-one
sodiated HMDB0036233, spectral sim. = 887, #6
Struc. sim. = 0.21
Butyl ethyl malonate
sodiated HMDB0000635, spectral sim. = 881, #7
Struc. sim. = 0.54
Succinylacetone
sodiated HMDB0061879, spectral sim. = 881, #8
Struc. sim. = 0.43
(S)-Methylbutanethioic acid
sodiated HMDB0061930, spectral sim. = 876, #9
Struc. sim. = 0.40
4-Ethoxy-4-oxobutanoic acid

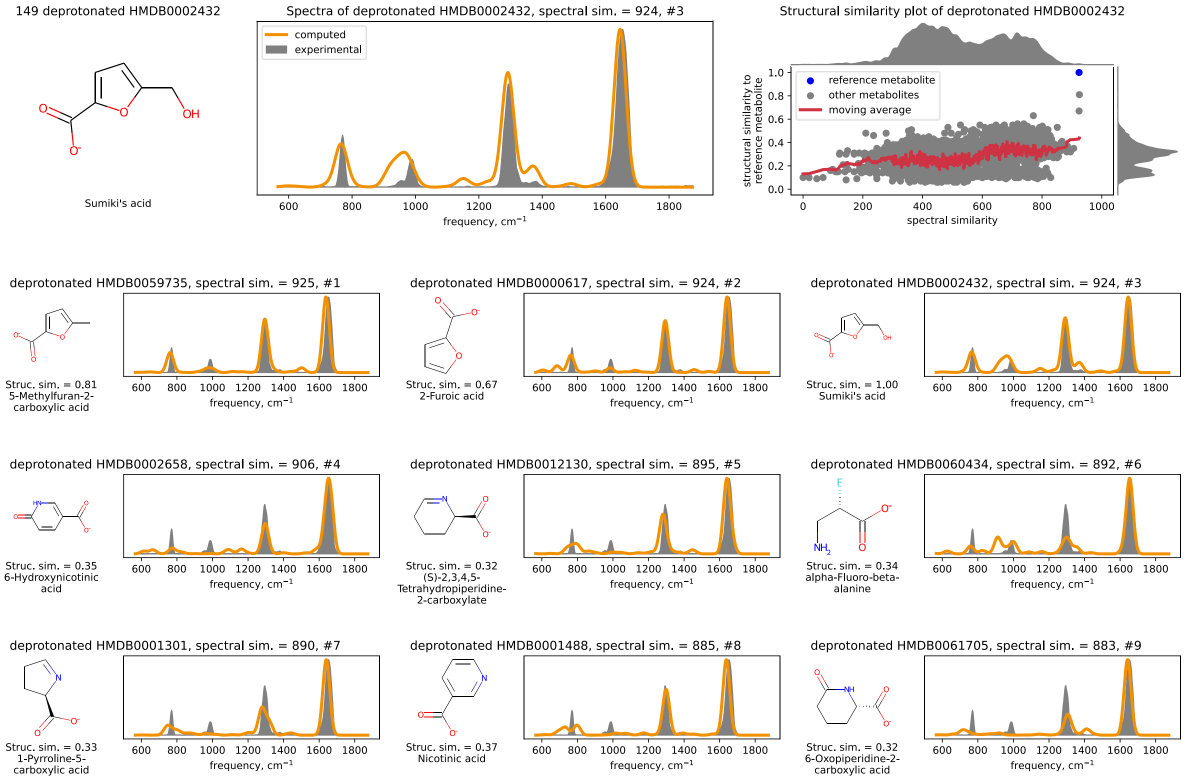
149 deprotonated HMDB0002432
Sumiki's acid
Spectra of deprotonated HMDB0002432, spectral sim. = 924, #3
computed
experimental
frequency, cm^{-1}
Structural similarity plot of deprotonated HMDB0002432
reference metabolite
other metabolites
moving average
structural similarity to reference metabolite
spectral similarity
deprotonated HMDB0059735, spectral sim. = 925, #1
Struc. sim. = 0.81
5-Methylfuran-2-carboxylic acid
deprotonated HMDB0000617, spectral sim. = 924, #2
Struc. sim. = 0.67
2-Furoic acid
deprotonated HMDB0002432, spectral sim. = 924, #3
Struc. sim. = 1.00
Sumiki's acid
deprotonated HMDB0002658, spectral sim. = 906, #4
Struc. sim. = 0.35
6-Hydroxynicotinic acid
deprotonated HMDB0012130, spectral sim. = 895, #5
Struc. sim. = 0.32
(S)-2,3,4,5-Tetrahydropiperidine-2-carboxylate
deprotonated HMDB0060434, spectral sim. = 892, #6
Struc. sim. = 0.34
alpha-Fluoro-beta-alanine
deprotonated HMDB0001301, spectral sim. = 890, #7
Struc. sim. = 0.33
1-Pyrroline-5-carboxylic acid
deprotonated HMDB0001488, spectral sim. = 885, #8
Struc. sim. = 0.37
Nicotinic acid
deprotonated HMDB0061705, spectral sim. = 883, #9
Struc. sim. = 0.32
6-Oxopiperidine-2-carboxylic acid

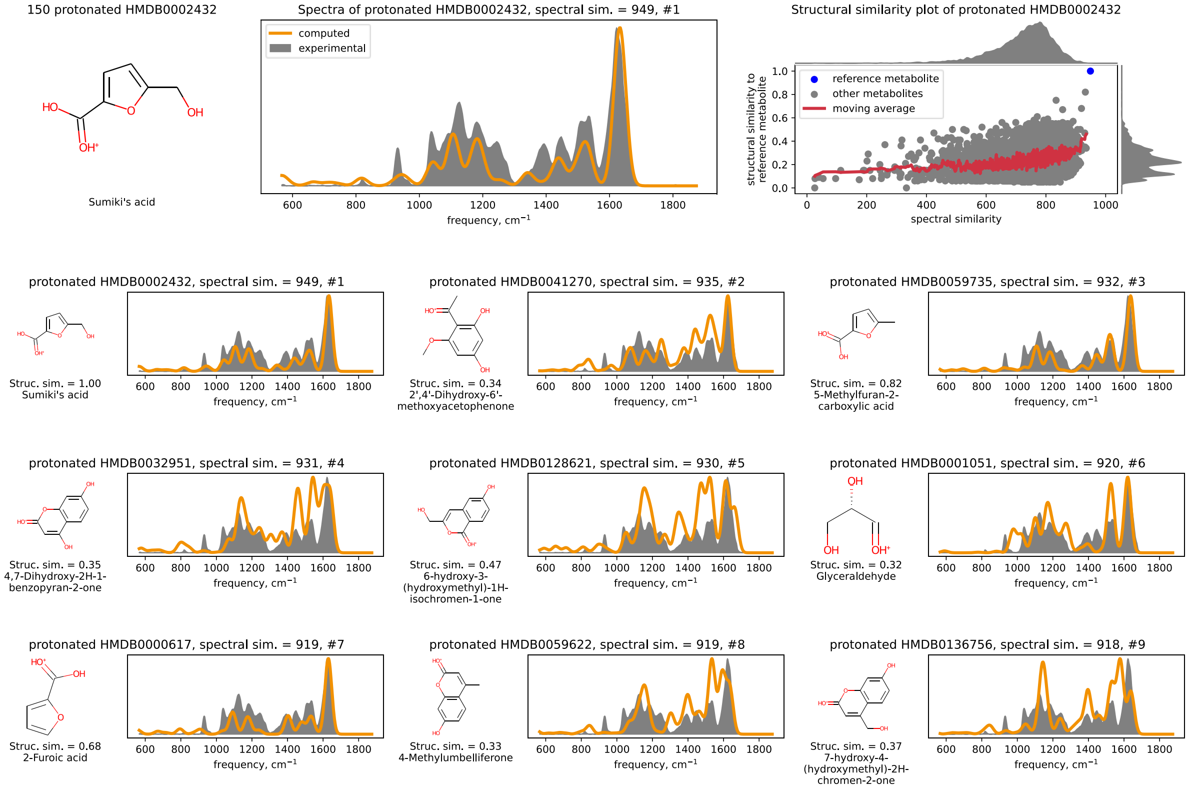
150 protonated HMDB0002432
Sumiki's acid
Spectra of protonated HMDB0002432, spectral sim. = 949, #1
computed
experimental
frequency, cm^{-1}
Structural similarity plot of protonated HMDB0002432
reference metabolite
other metabolites
moving average
structural similarity to reference metabolite
spectral similarity
protonated HMDB0002432, spectral sim. = 949, #1
Struc. sim. = 1.00
Sumiki's acid
protonated HMDB0041270, spectral sim. = 935, #2
Struc. sim. = 0.34
2',4'-Dihydroxy-6'-methoxyacetophenone
protonated HMDB0059735, spectral sim. = 932, #3
Struc. sim. = 0.82
5-Methylfuran-2-carboxylic acid
protonated HMDB0032951, spectral sim. = 931, #4
Struc. sim. = 0.35
4,7-Dihydroxy-2H-1-benzopyran-2-one
protonated HMDB0128621, spectral sim. = 930, #5
Struc. sim. = 0.47
6-hydroxy-3-(hydroxymethyl)-1H-isochromen-1-one
protonated HMDB0001051, spectral sim. = 920, #6
Struc. sim. = 0.32
Glyceraldehyde
protonated HMDB0000617, spectral sim. = 919, #7
Struc. sim. = 0.68
2-Furoic acid
protonated HMDB0059622, spectral sim. = 919, #8
Struc. sim. = 0.33
4-Methylumbelliferone
protonated HMDB0136756, spectral sim. = 918, #9
Struc. sim. = 0.37
7-hydroxy-4-(hydroxymethyl)-2H-chromen-2-one

151 sodiated HMDB0002432

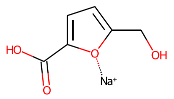

Sumiki's acid

Spectra of sodiated HMDB0002432, spectral sim. = 830, #70

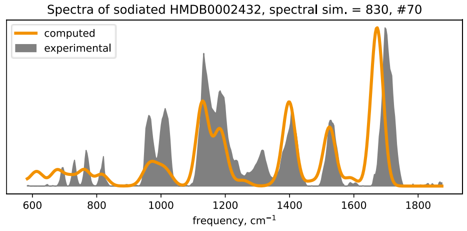

Structural similarity plot of sodiated HMDB0002432

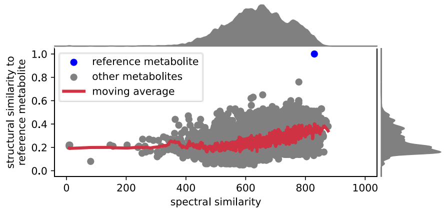

sodiated HMDB0059765, spectral sim. = 876, #1

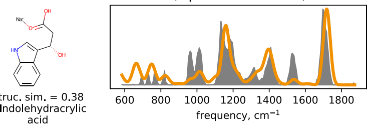

Struc. sim. = 0.38
3-Indolehydracrylic acid

sodiated HMDB0143414, spectral sim. = 870, #2

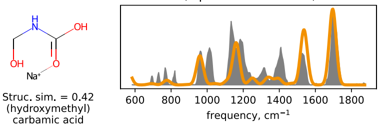

Struc. sim. = 0.42
(hydroxymethyl) carbamic acid

sodiated HMDB0125529, spectral sim. = 867, #3

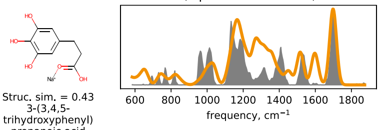

Struc. sim. = 0.43
3-(3,4,5-trihydroxyphenyl) propanoic acid

sodiated HMDB0029573, spectral sim. = 862, #4

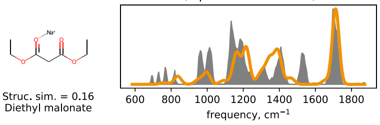

Struc. sim. = 0.16
Diethyl malonate

sodiated HMDB0133754, spectral sim. = 860, #5

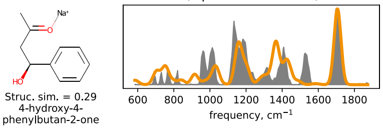

Struc. sim. = 0.29
4-hydroxy-4-phenylbutan-2-one

sodiated HMDB0130546, spectral sim. = 860, #6

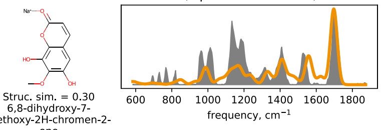

Struc. sim. = 0.30
6,8-dihydroxy-7-methoxy-2H-chromen-2-one

sodiated HMDB0124923, spectral sim. = 860, #7

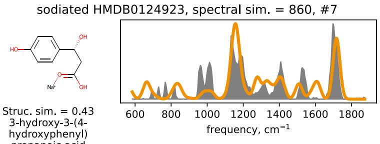

Struc. sim. = 0.43
3-hydroxy-3-(4-hydroxyphenyl) propanoic acid

sodiated HMDB0143050, spectral sim. = 857, #8

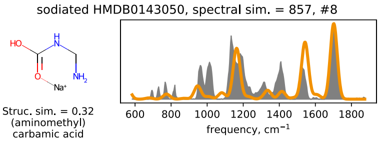

Struc. sim. = 0.32
(aminomethyl) carbamic acid

sodiated HMDB0000700, spectral sim. = 854, #9

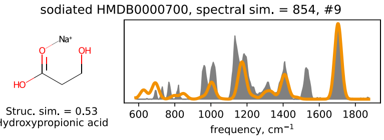

Struc. sim. = 0.53
Hydroxypropionic acid

152 protonated HMDB0002825

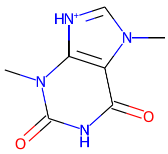

Theobromine

Spectra of protonated HMDB0002825, spectral sim. = 801, #102

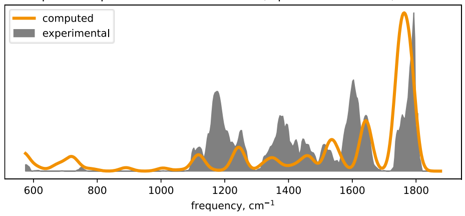

Structural similarity plot of protonated HMDB0002825

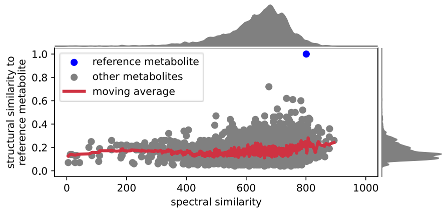

protonated HMDB0031179, spectral sim. = 894, #1

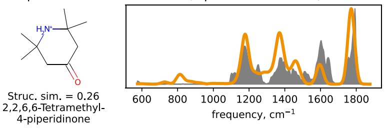

Struc. sim. = 0.26
2,2,6,6-Tetramethyl-4-piperidinone

protonated HMDB0002144, spectral sim. = 879, #2

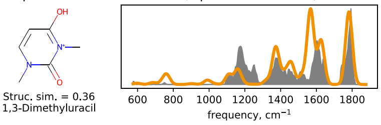

Struc. sim. = 0.36
1,3-Dimethyluracil

protonated HMDB0013678, spectral sim. = 878, #3

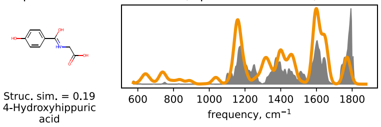

Struc. sim. = 0.19
4-Hydroxyhippuric acid

protonated HMDB0033136, spectral sim. = 878, #4

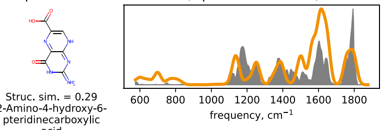

Struc. sim. = 0.29
2-Amino-4-hydroxy-6-pteridinecarboxylic acid

protonated HMDB0002042, spectral sim. = 872, #5

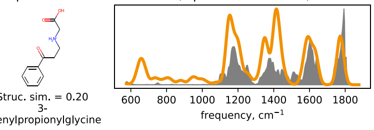

Struc. sim. = 0.20
3-Phenylpropionylglycine

protonated HMDB0000684, spectral sim. = 869, #6

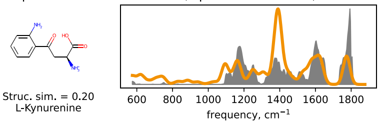

Struc. sim. = 0.20
L-Kynurenine

protonated HMDB0131190, spectral sim. = 867, #7

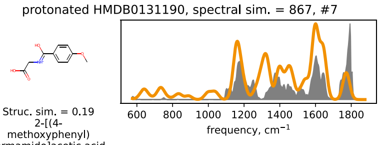

Struc. sim. = 0.19
2-[(4-methoxyphenyl) formamido]acetic acid

protonated HMDB0013292, spectral sim. = 866, #8

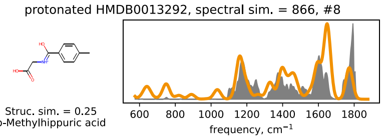

Struc. sim. = 0.25
p-Methylhippuric acid

protonated HMDB0128624, spectral sim. = 863, #9

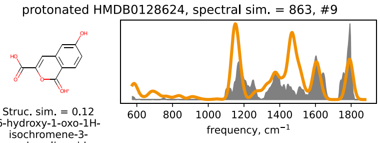

Struc. sim. = 0.12
6-hydroxy-1-oxo-1H-isochromene-3-carboxylic acid

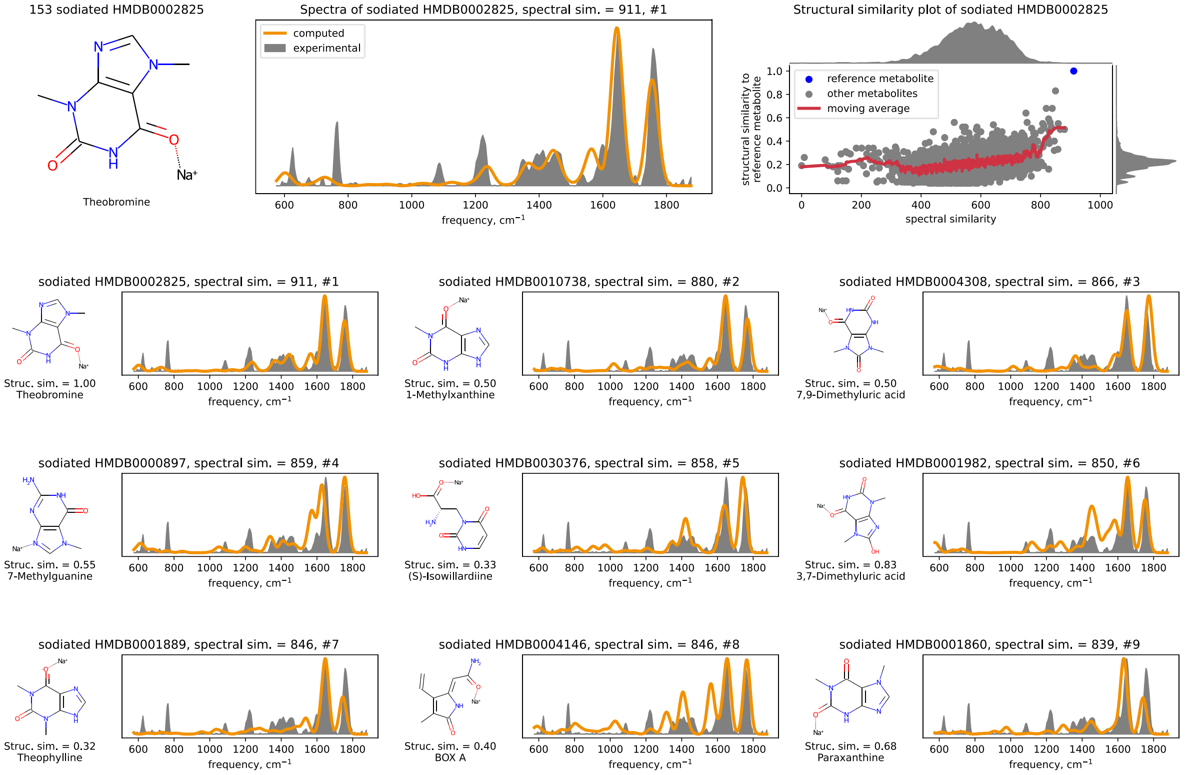
153 sodiated HMDB0002825
Theobromine
Spectra of sodiated HMDB0002825, spectral sim. = 911, #1
computed
experimental
frequency, cm⁻¹
Structural similarity plot of sodiated HMDB0002825
structural similarity to reference metabolite
reference metabolite
other metabolites
moving average
spectral similarity
sodiated HMDB0002825, spectral sim. = 911, #1
Struc. sim. = 1.00
Theobromine
sodiated HMDB0010738, spectral sim. = 880, #2
Struc. sim. = 0.50
1-Methylxanthine
sodiated HMDB0004308, spectral sim. = 866, #3
Struc. sim. = 0.50
7,9-Dimethyluric acid
sodiated HMDB0000897, spectral sim. = 859, #4
Struc. sim. = 0.55
7-Methylguanine
sodiated HMDB0030376, spectral sim. = 858, #5
Struc. sim. = 0.33
(S)-Isowillardiine
sodiated HMDB0001982, spectral sim. = 850, #6
Struc. sim. = 0.83
3,7-Dimethyluric acid
sodiated HMDB0001889, spectral sim. = 846, #7
Struc. sim. = 0.32
Theophylline
sodiated HMDB0004146, spectral sim. = 846, #8
Struc. sim. = 0.40
BOX A
sodiated HMDB0001860, spectral sim. = 839, #9
Struc. sim. = 0.68
Paraxanthine

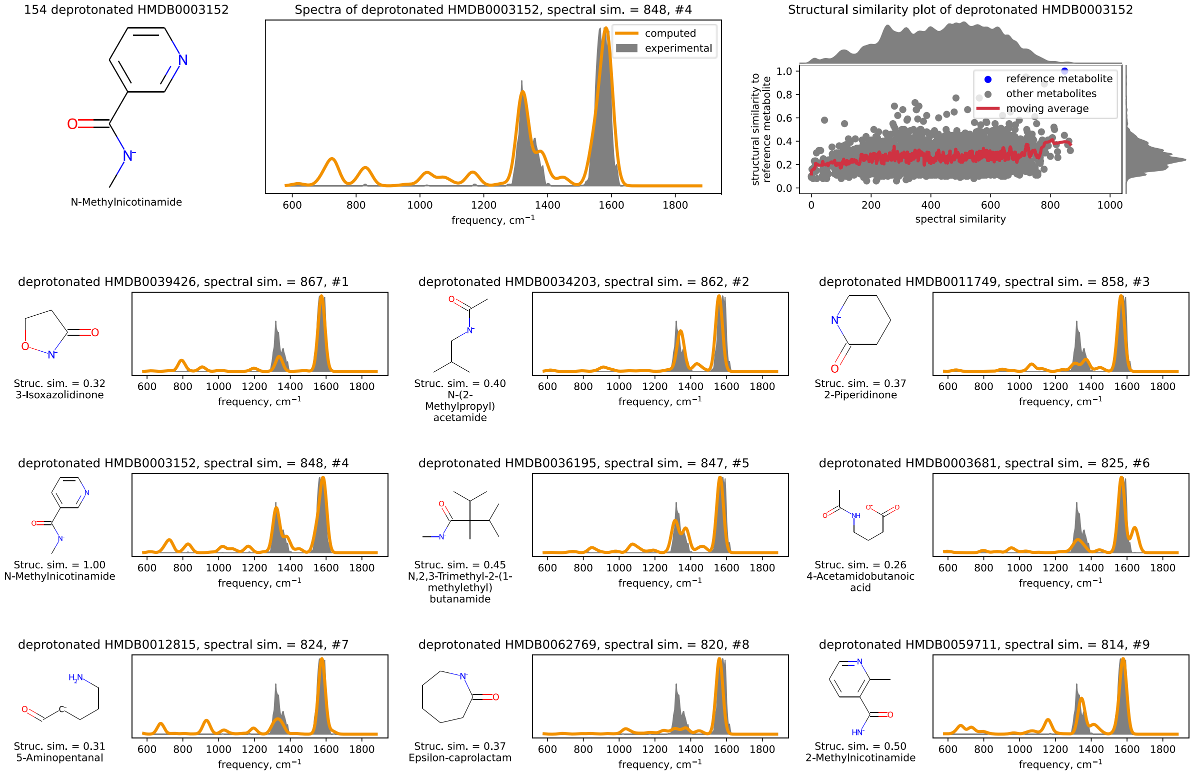
154 deprotonated HMDB0003152
N-Methylnicotinamide
Spectra of deprotonated HMDB0003152, spectral sim. = 848, #4
computed
experimental
frequency, cm⁻¹
Structural similarity plot of deprotonated HMDB0003152
structural similarity to reference metabolite
reference metabolite
other metabolites
moving average
spectral similarity
deprotonated HMDB0039426, spectral sim. = 867, #1
Struc. sim. = 0.32
3-Isoxazolidinone
deprotonated HMDB0034203, spectral sim. = 862, #2
Struc. sim. = 0.40
N-(2-Methylpropyl)acetamide
deprotonated HMDB0011749, spectral sim. = 858, #3
Struc. sim. = 0.37
2-Piperidinone
deprotonated HMDB0003152, spectral sim. = 848, #4
Struc. sim. = 1.00
N-Methylnicotinamide
deprotonated HMDB0036195, spectral sim. = 847, #5
Struc. sim. = 0.45
N,2,3-Trimethyl-2-(1-methylethyl)butanamide
deprotonated HMDB0003681, spectral sim. = 825, #6
Struc. sim. = 0.26
4-Acetamidobutanoic acid
deprotonated HMDB0012815, spectral sim. = 824, #7
Struc. sim. = 0.31
5-Aminopentanal
deprotonated HMDB0062769, spectral sim. = 820, #8
Struc. sim. = 0.37
Epsilon-caprolactam
deprotonated HMDB0059711, spectral sim. = 814, #9
Struc. sim. = 0.50
2-Methylnicotinamide

155 protonated HMDB0003152

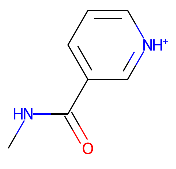

N-Methylnicotinamide

Spectra of protonated HMDB0003152, spectral sim. = 849, #1

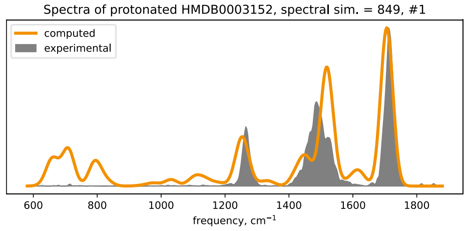

Structural similarity plot of protonated HMDB0003152

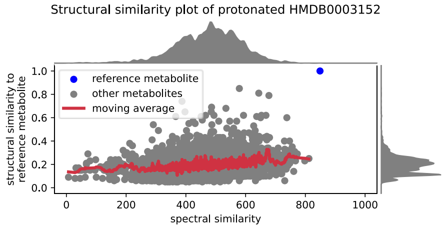

protonated HMDB0003152, spectral sim. = 849, #1

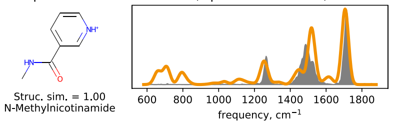

protonated HMDB0001080, spectral sim. = 812, #2

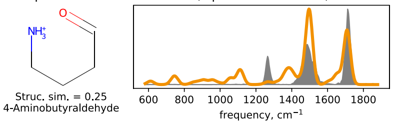

protonated HMDB0001847, spectral sim. = 798, #3

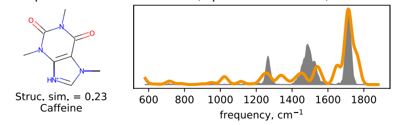

protonated HMDB0030773, spectral sim. = 773, #4

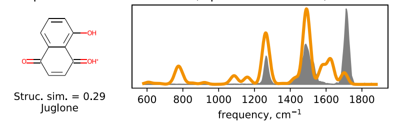

protonated HMDB0042006, spectral sim. = 769, #5

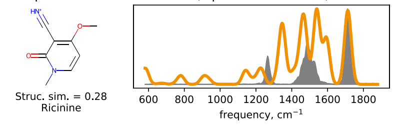

protonated HMDB0040637, spectral sim. = 767, #6

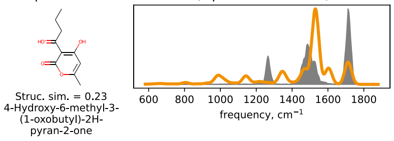

protonated HMDB0028973, spectral sim. = 764, #7

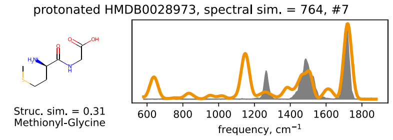

protonated HMDB0035291, spectral sim. = 761, #8

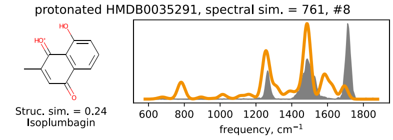

protonated HMDB0031510, spectral sim. = 761, #9

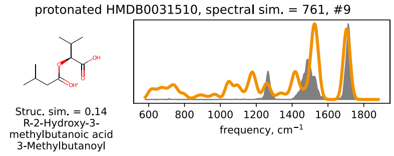

156 sodiated HMDB0003152

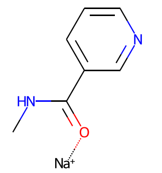

N-Methylnicotinamide

Spectra of sodiated HMDB0003152, spectral sim. = 917, #2

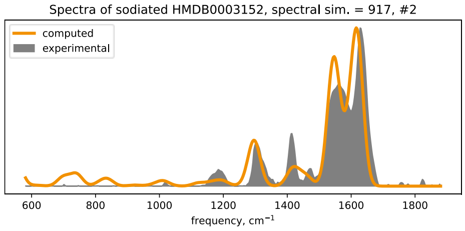

Structural similarity plot of sodiated HMDB0003152

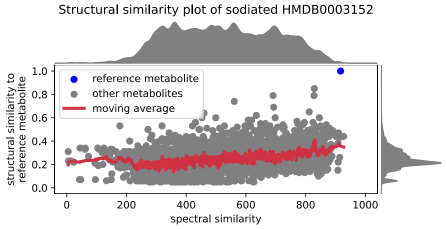

sodiated HMDB0029198, spectral sim. = 927, #1

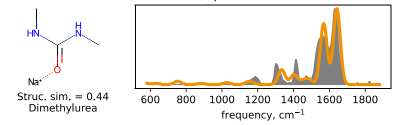

sodiated HMDB0003152, spectral sim. = 917, #2

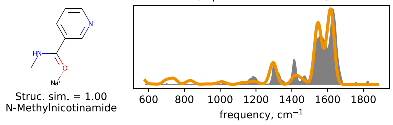

sodiated HMDB0011601, spectral sim. = 914, #3

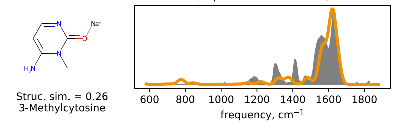

sodiated HMDB0031651, spectral sim. = 913, #4

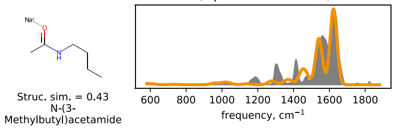

sodiated HMDB0034203, spectral sim. = 912, #5

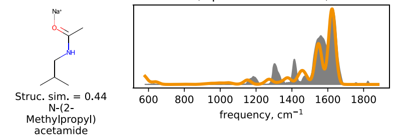

sodiated HMDB0031215, spectral sim. = 910, #6

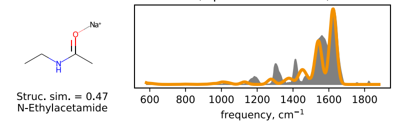

sodiated HMDB0002249, spectral sim. = 909, #7

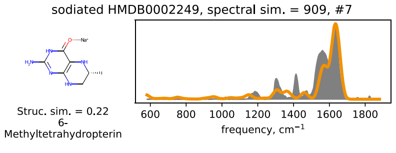

sodiated HMDB0039252, spectral sim. = 908, #8

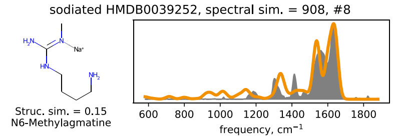

sodiated HMDB0062766, spectral sim. = 908, #9

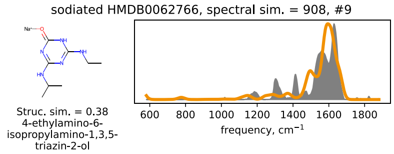

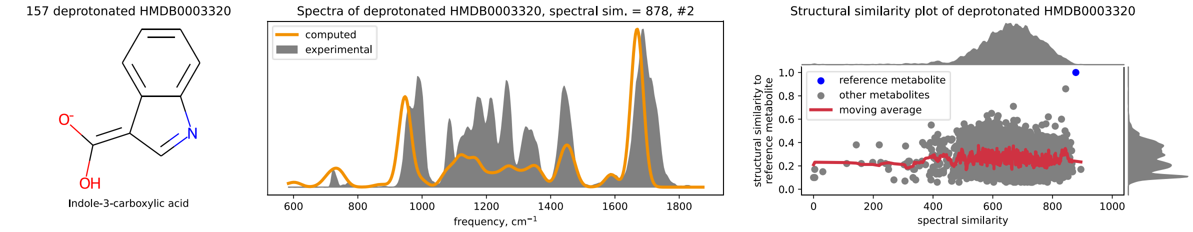
157 deprotonated HMDB0003320
Indole-3-carboxylic acid
Spectra of deprotonated HMDB0003320, spectral sim. = 878, #2
computed
experimental
frequency, cm⁻¹
Structural similarity plot of deprotonated HMDB0003320
reference metabolite
other metabolites
moving average
structural similarity to reference metabolite
spectral similarity

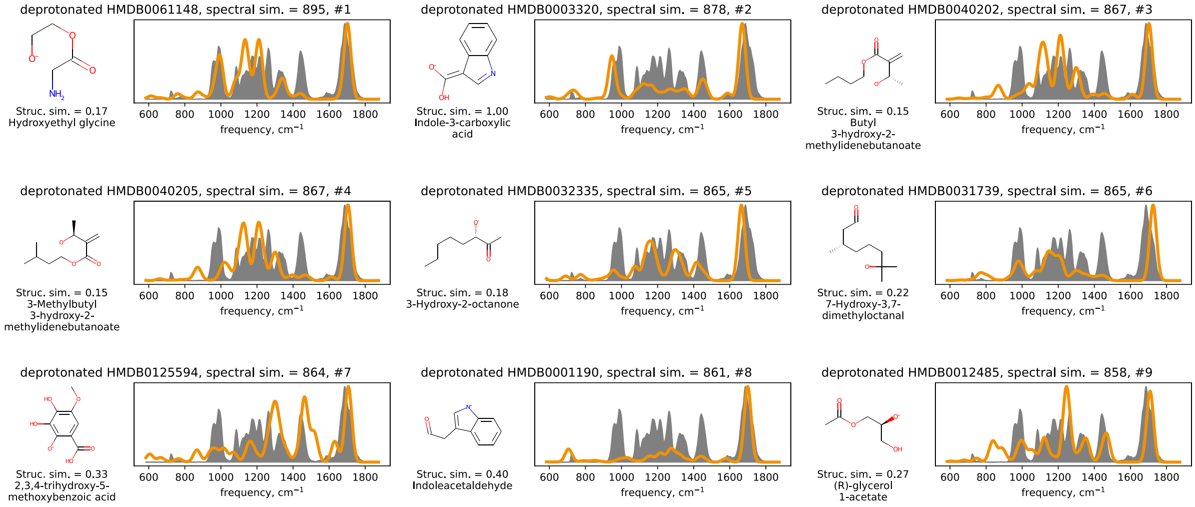
deprotonated HMDB0061148, spectral sim. = 895, #1
Struc. sim. = 0.17
Hydroxyethyl glycine
deprotonated HMDB0003320, spectral sim. = 878, #2
Struc. sim. = 1.00
Indole-3-carboxylic acid
deprotonated HMDB0040202, spectral sim. = 867, #3
Struc. sim. = 0.15
Butyl 3-hydroxy-2-methylidenebutanoate
deprotonated HMDB0040205, spectral sim. = 867, #4
Struc. sim. = 0.15
3-Methylbutyl 3-hydroxy-2-methylidenebutanoate
deprotonated HMDB0032335, spectral sim. = 865, #5
Struc. sim. = 0.18
3-Hydroxy-2-octanone
deprotonated HMDB0031739, spectral sim. = 865, #6
Struc. sim. = 0.22
7-Hydroxy-3,7-dimethyloctanal
deprotonated HMDB0125594, spectral sim. = 864, #7
Struc. sim. = 0.33
2,3,4-trihydroxy-5-methoxybenzoic acid
deprotonated HMDB0001190, spectral sim. = 861, #8
Struc. sim. = 0.40
Indoleacetaldehyde
deprotonated HMDB0012485, spectral sim. = 858, #9
Struc. sim. = 0.27
(R)-glycerol 1-acetate
frequency, cm⁻¹

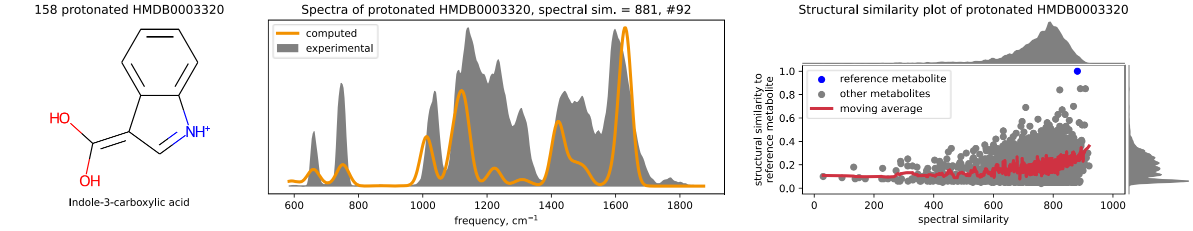
158 protonated HMDB0003320
Indole-3-carboxylic acid
Spectra of protonated HMDB0003320, spectral sim. = 881, #92
computed
experimental
frequency, cm⁻¹
Structural similarity plot of protonated HMDB0003320
reference metabolite
other metabolites
moving average
structural similarity to reference metabolite
spectral similarity

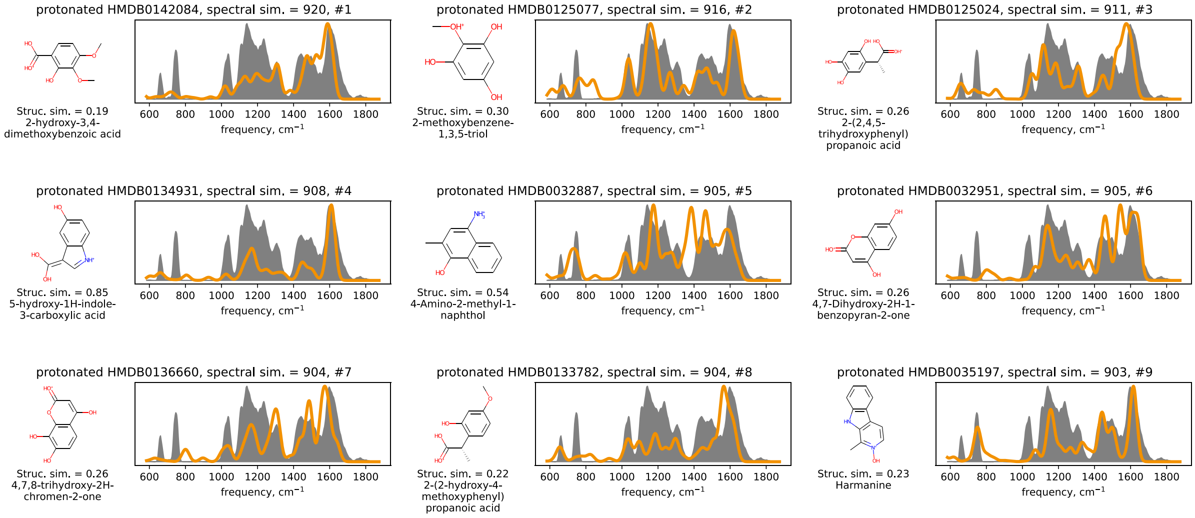
protonated HMDB0142084, spectral sim. = 920, #1
Struc. sim. = 0.19
2-hydroxy-3,4-dimethoxybenzoic acid
protonated HMDB0125077, spectral sim. = 916, #2
Struc. sim. = 0.30
2-methoxybenzene-1,3,5-triol
protonated HMDB0125024, spectral sim. = 911, #3
Struc. sim. = 0.26
2-(2,4,5-trihydroxyphenyl) propanoic acid
protonated HMDB0134931, spectral sim. = 908, #4
Struc. sim. = 0.85
5-hydroxy-1H-indole-3-carboxylic acid
protonated HMDB0032887, spectral sim. = 905, #5
Struc. sim. = 0.54
4-Amino-2-methyl-1-naphthol
protonated HMDB0032951, spectral sim. = 905, #6
Struc. sim. = 0.26
4,7-Dihydroxy-2H-1-benzopyran-2-one
protonated HMDB0136660, spectral sim. = 904, #7
Struc. sim. = 0.26
4,7,8-trihydroxy-2H-chromen-2-one
protonated HMDB0133782, spectral sim. = 904, #8
Struc. sim. = 0.22
2-(2-hydroxy-4-methoxyphenyl) propanoic acid
protonated HMDB0035197, spectral sim. = 903, #9
Struc. sim. = 0.23
Harmanine
frequency, cm⁻¹

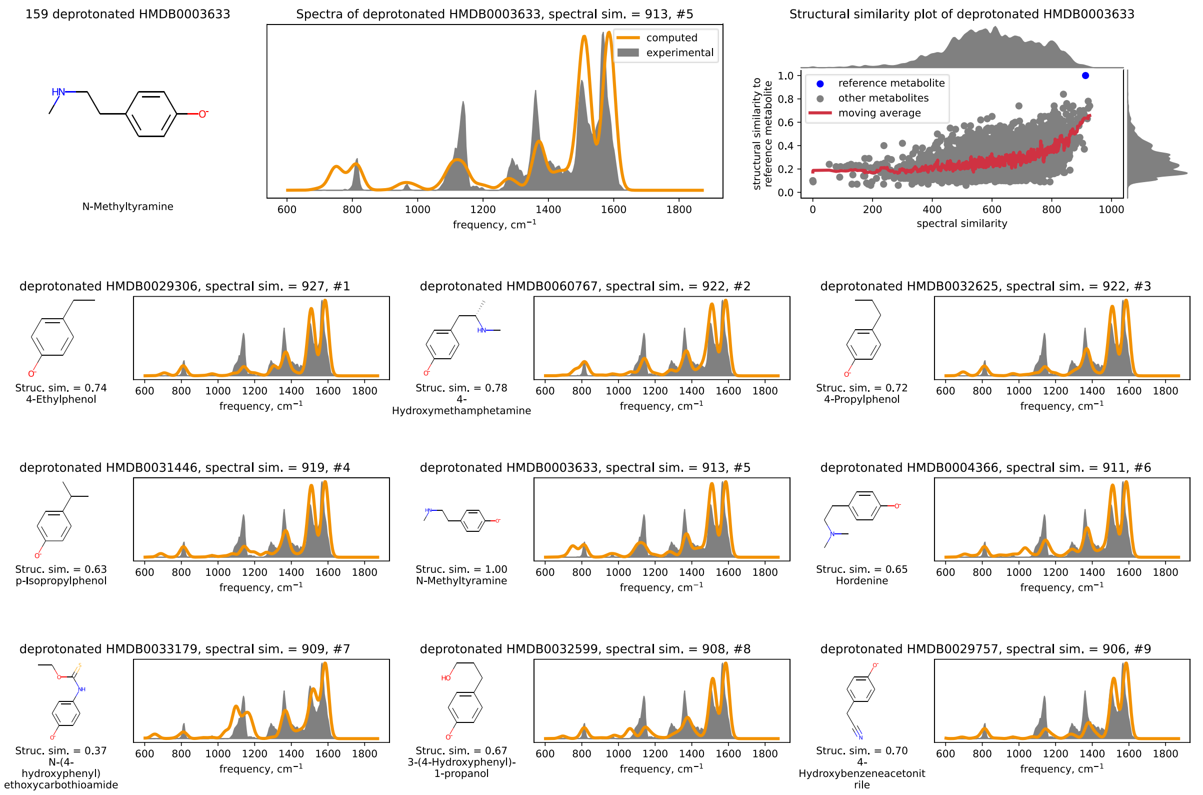
159 deprotonated HMDB0003633
N-Methyltyramine
Spectra of deprotonated HMDB0003633, spectral sim. = 913, #5
computed
experimental
frequency, cm−1
Structural similarity plot of deprotonated HMDB0003633
reference metabolite
other metabolites
moving average
structural similarity to reference metabolite
spectral similarity
deprotonated HMDB0029306, spectral sim. = 927, #1
Struc. sim. = 0.74
4-Ethylphenol
deprotonated HMDB0060767, spectral sim. = 922, #2
Struc. sim. = 0.78
4-Hydroxymethamphetamine
deprotonated HMDB0032625, spectral sim. = 922, #3
Struc. sim. = 0.72
4-Propylphenol
deprotonated HMDB0031446, spectral sim. = 919, #4
Struc. sim. = 0.63
p-Isopropylphenol
deprotonated HMDB0003633, spectral sim. = 913, #5
Struc. sim. = 1.00
N-Methyltyramine
deprotonated HMDB0004366, spectral sim. = 911, #6
Struc. sim. = 0.65
Hordenine
deprotonated HMDB0033179, spectral sim. = 909, #7
Struc. sim. = 0.37
N-(4-hydroxyphenyl) ethoxycarbothioamide
deprotonated HMDB0032599, spectral sim. = 908, #8
Struc. sim. = 0.67
3-(4-Hydroxyphenyl)-1-propanol
deprotonated HMDB0029757, spectral sim. = 906, #9
Struc. sim. = 0.70
4-Hydroxybenzeneacetonitrile

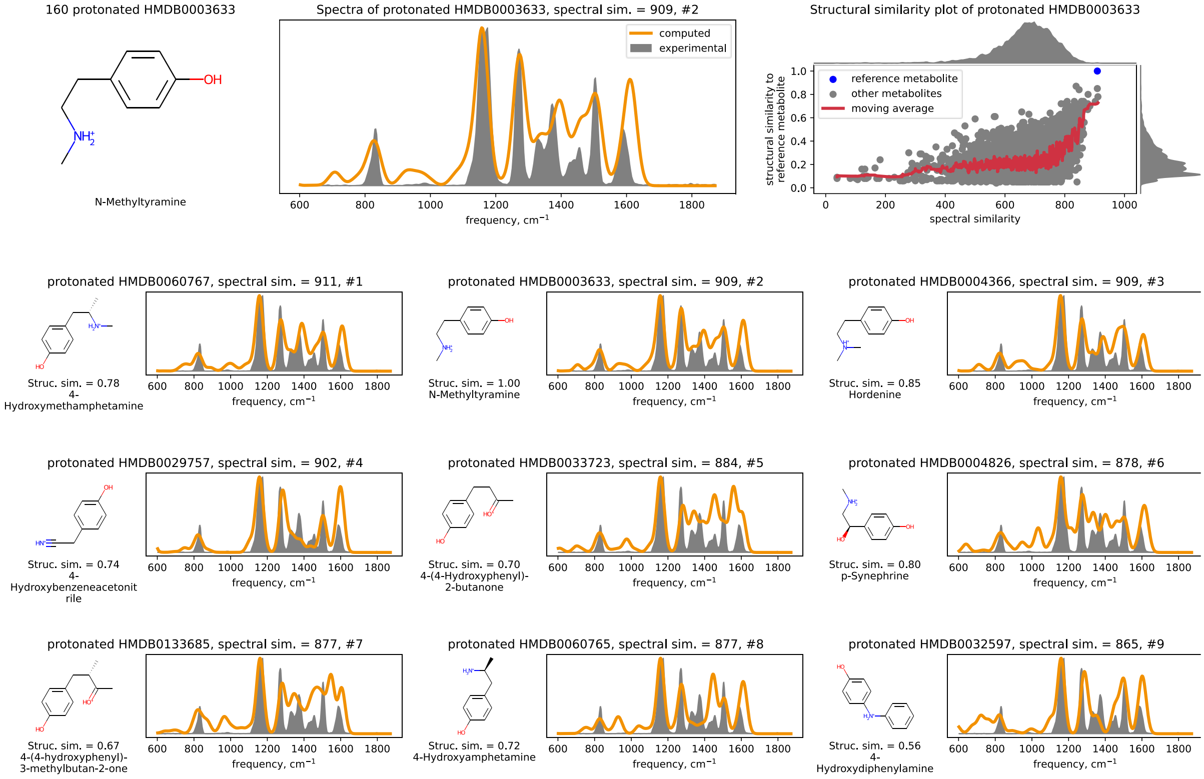
160 protonated HMDB0003633
N-Methyltyramine
Spectra of protonated HMDB0003633, spectral sim. = 909, #2
computed
experimental
frequency, cm−1
Structural similarity plot of protonated HMDB0003633
reference metabolite
other metabolites
moving average
structural similarity to reference metabolite
spectral similarity
protonated HMDB0060767, spectral sim. = 911, #1
Struc. sim. = 0.78
4-Hydroxymethamphetamine
protonated HMDB0003633, spectral sim. = 909, #2
Struc. sim. = 1.00
N-Methyltyramine
protonated HMDB0004366, spectral sim. = 909, #3
Struc. sim. = 0.85
Hordenine
protonated HMDB0029757, spectral sim. = 902, #4
Struc. sim. = 0.74
4-Hydroxybenzeneacetonitrile
protonated HMDB0033723, spectral sim. = 884, #5
Struc. sim. = 0.70
4-(4-Hydroxyphenyl)-2-butanone
protonated HMDB0004826, spectral sim. = 878, #6
Struc. sim. = 0.80
p-Synephrine
protonated HMDB0133685, spectral sim. = 877, #7
Struc. sim. = 0.67
4-(4-hydroxyphenyl)-3-methylbutan-2-one
protonated HMDB0060765, spectral sim. = 877, #8
Struc. sim. = 0.72
4-Hydroxyamphetamine
protonated HMDB0032597, spectral sim. = 865, #9
Struc. sim. = 0.56
4-Hydroxydiphenylamine

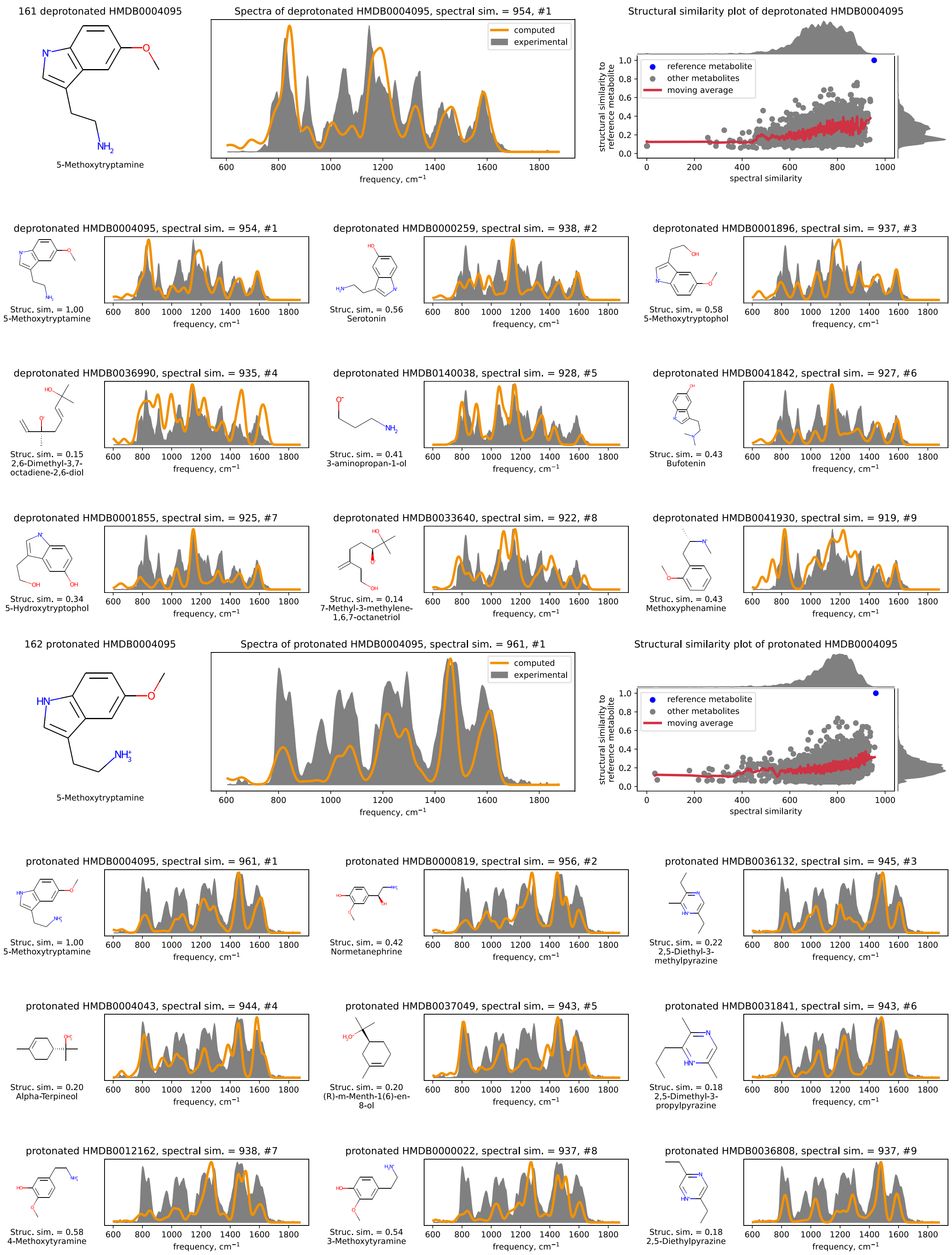
161 deprotonated HMDB0004095
5-Methoxytryptamine
Spectra of deprotonated HMDB0004095, spectral sim. = 954, #1
computed
experimental
frequency, cm⁻¹
Structural similarity plot of deprotonated HMDB0004095
reference metabolite
other metabolites
moving average
structural similarity to reference metabolite
spectral similarity
deprotonated HMDB0004095, spectral sim. = 954, #1
Struc. sim. = 1.00
5-Methoxytryptamine
deprotonated HMDB0000259, spectral sim. = 938, #2
Struc. sim. = 0.56
Serotonin
deprotonated HMDB0001896, spectral sim. = 937, #3
Struc. sim. = 0.58
5-Methoxytryptophol
deprotonated HMDB0036990, spectral sim. = 935, #4
Struc. sim. = 0.15
2,6-Dimethyl-3,7-octadiene-2,6-diol
deprotonated HMDB0140038, spectral sim. = 928, #5
Struc. sim. = 0.41
3-aminopropan-1-ol
deprotonated HMDB0041842, spectral sim. = 927, #6
Struc. sim. = 0.43
Bufotenin
deprotonated HMDB0001855, spectral sim. = 925, #7
Struc. sim. = 0.34
5-Hydroxytryptophol
deprotonated HMDB0033640, spectral sim. = 922, #8
Struc. sim. = 0.14
7-Methyl-3-methylene-1,6,7-octanetriol
deprotonated HMDB0041930, spectral sim. = 919, #9
Struc. sim. = 0.43
Methoxyphenamine
162 protonated HMDB0004095
5-Methoxytryptamine
Spectra of protonated HMDB0004095, spectral sim. = 961, #1
Structural similarity plot of protonated HMDB0004095
protonated HMDB0004095, spectral sim. = 961, #1
Struc. sim. = 1.00
5-Methoxytryptamine
protonated HMDB0000819, spectral sim. = 956, #2
Struc. sim. = 0.42
Normetanephrine
protonated HMDB0036132, spectral sim. = 945, #3
Struc. sim. = 0.22
2,5-Diethyl-3-methylpyrazine
protonated HMDB0004043, spectral sim. = 944, #4
Struc. sim. = 0.20
Alpha-Terpineol
protonated HMDB0037049, spectral sim. = 943, #5
Struc. sim. = 0.20
(R)-m-Menth-1(6)-en-8-ol
protonated HMDB0031841, spectral sim. = 943, #6
Struc. sim. = 0.18
2,5-Dimethyl-3-propylpyrazine
protonated HMDB0012162, spectral sim. = 938, #7
Struc. sim. = 0.58
4-Methoxytyramine
protonated HMDB0000022, spectral sim. = 937, #8
Struc. sim. = 0.54
3-Methoxytyramine
protonated HMDB0036808, spectral sim. = 937, #9
Struc. sim. = 0.18
2,5-Diethylpyrazine

163 sodiated HMDB0004095

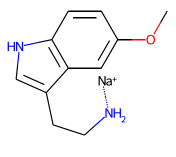

5-Methoxytryptamine

Spectra of sodiated HMDB0004095, spectral sim. = 957, #1

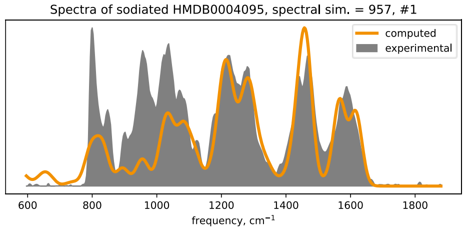

Structural similarity plot of sodiated HMDB0004095

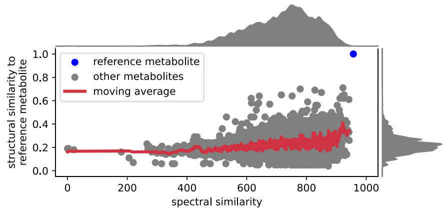

sodiated HMDB0004095, spectral sim. = 957, #1

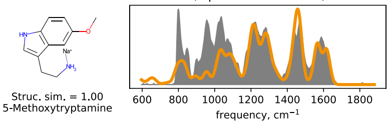

Struc. sim. = 1.00
5-Methoxytryptamine

sodiated HMDB0040049, spectral sim. = 943, #2

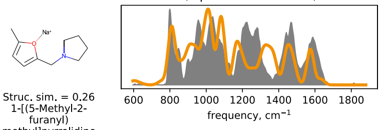

Struc. sim. = 0.26
1-[(5-Methyl-2-furanyl) methyl]pyrrolidine

sodiated HMDB0001896, spectral sim. = 942, #3

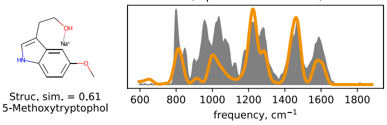

Struc. sim. = 0.61
5-Methoxytryptophol

sodiated HMDB0035275, spectral sim. = 942, #4

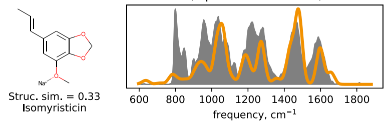

Struc. sim. = 0.33
Isomyristicin

sodiated HMDB0041461, spectral sim. = 938, #5

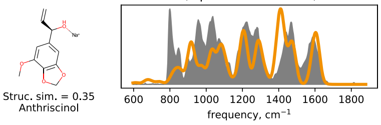

Struc. sim. = 0.35
Anthriscinol

sodiated HMDB0040007, spectral sim. = 937, #6

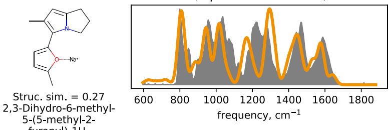

Struc. sim. = 0.27
2,3-Dihydro-6-methyl-5-(5-methyl-2-furanyl)-1H-pyrrolizine

sodiated HMDB0039836, spectral sim. = 936, #7

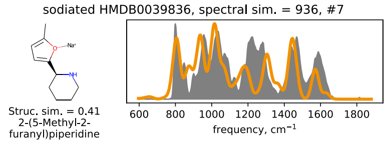

Struc. sim. = 0.41
2-(5-Methyl-2-furanyl)piperidine

sodiated HMDB0029825, spectral sim. = 935, #8

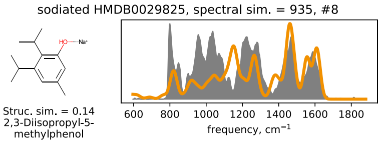

Struc. sim. = 0.14
2,3-Diisopropyl-5-methylphenol

sodiated HMDB0037641, spectral sim. = 934, #9

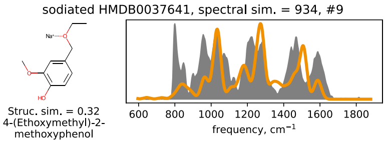

Struc. sim. = 0.32
4-(Ethoxymethyl)-2-methoxyphenol

164 deprotonated HMDB0005807

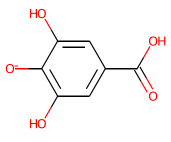

Gallic acid

Spectra of deprotonated HMDB0005807, spectral sim. = 911, #5

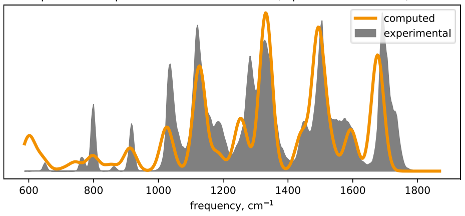

Structural similarity plot of deprotonated HMDB0005807

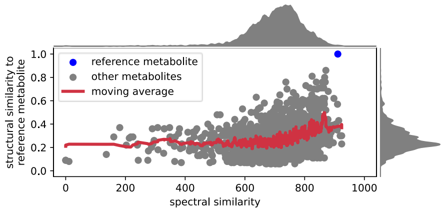

deprotonated HMDB0133535, spectral sim. = 924, #1

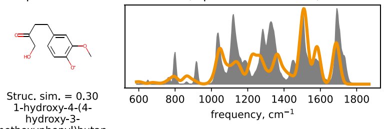

Struc. sim. = 0.30
1-hydroxy-4-(4-hydroxy-3-methoxyphenyl)butan-2-one

deprotonated HMDB0032590, spectral sim. = 924, #2

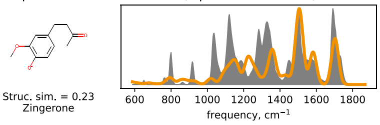

Struc. sim. = 0.23
Zingerone

deprotonated HMDB0133478, spectral sim. = 919, #3

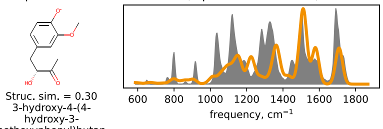

Struc. sim. = 0.30
3-hydroxy-4-(4-hydroxy-3-methoxyphenyl)butan-2-one

deprotonated HMDB0000955, spectral sim. = 914, #4

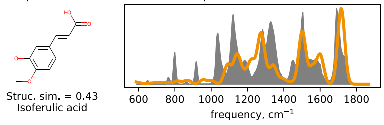

Struc. sim. = 0.43
Isoferulic acid

deprotonated HMDB0005807, spectral sim. = 911, #5

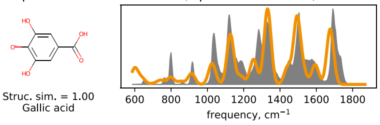

Struc. sim. = 1.00
Gallic acid

deprotonated HMDB0001253, spectral sim. = 910, #6

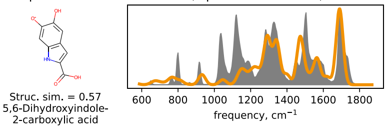

Struc. sim. = 0.57
5,6-Dihydroxyindole-2-carboxylic acid

deprotonated HMDB0133519, spectral sim. = 909, #7

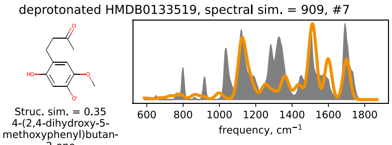

Struc. sim. = 0.35
4-(2,4-dihydroxy-5-methoxyphenyl)butan-2-one

deprotonated HMDB0035484, spectral sim. = 904, #8

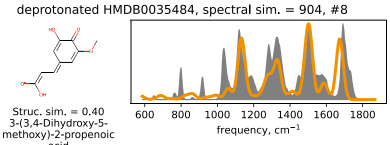

Struc. sim. = 0.40
3-(3,4-Dihydroxy-5-methoxy)-2-propenoic acid

deprotonated HMDB0132254, spectral sim. = 899, #9

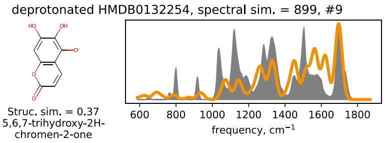

Struc. sim. = 0.37
5,6,7-trihydroxy-2H-chromen-2-one

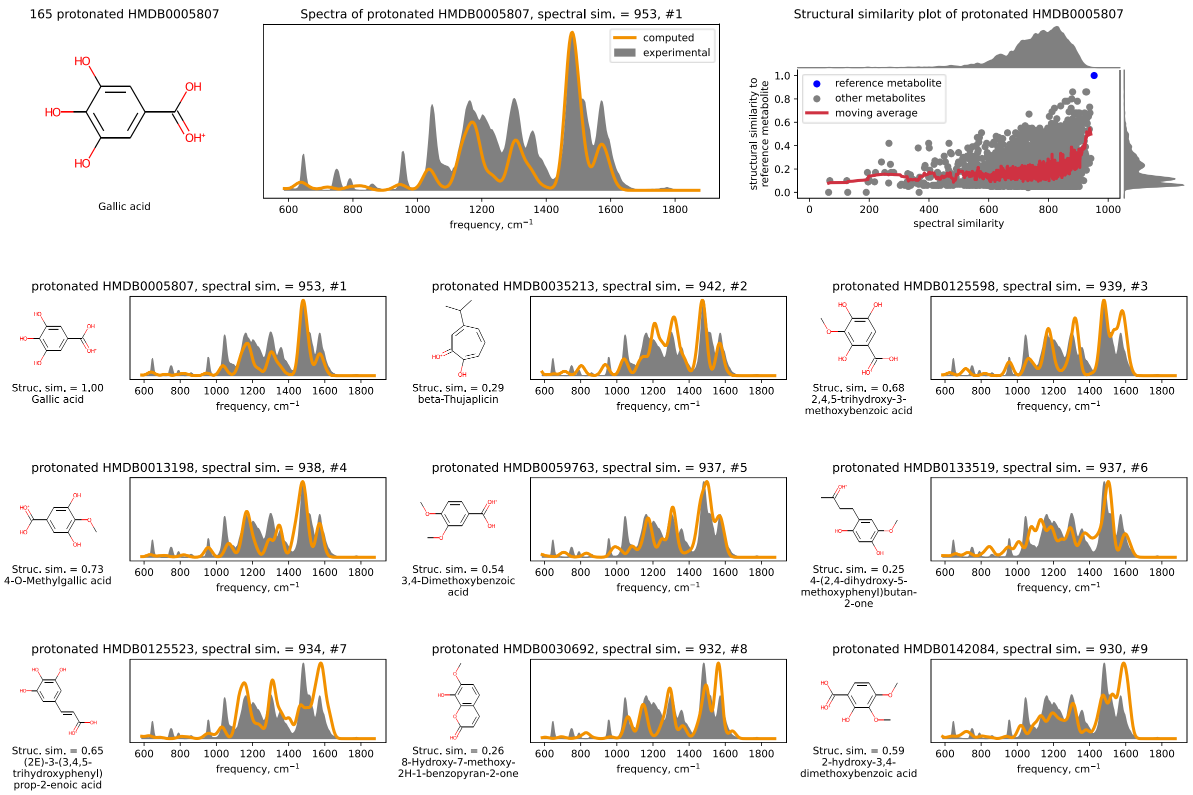

165 protonated HMDB0005807
Gallic acid
Spectra of protonated HMDB0005807, spectral sim. = 953, #1
computed
experimental
frequency, cm^{-1}
Structural similarity plot of protonated HMDB0005807
structural similarity to reference metabolite
reference metabolite
other metabolites
moving average
spectral similarity
protonated HMDB0005807, spectral sim. = 953, #1
Struc. sim. = 1.00
Gallic acid
protonated HMDB0035213, spectral sim. = 942, #2
Struc. sim. = 0.29
beta-Thujaplicin
protonated HMDB0125598, spectral sim. = 939, #3
Struc. sim. = 0.68
2,4,5-trihydroxy-3-methoxybenzoic acid
protonated HMDB0013198, spectral sim. = 938, #4
Struc. sim. = 0.73
4-O-Methylgallic acid
protonated HMDB0059763, spectral sim. = 937, #5
Struc. sim. = 0.54
3,4-Dimethoxybenzoic acid
protonated HMDB0133519, spectral sim. = 937, #6
Struc. sim. = 0.25
4-(2,4-dihydroxy-5-methoxyphenyl)butan-2-one
protonated HMDB0125523, spectral sim. = 934, #7
Struc. sim. = 0.65
(2E)-3-(3,4,5-trihydroxyphenyl)prop-2-enoic acid
protonated HMDB0030692, spectral sim. = 932, #8
Struc. sim. = 0.26
8-Hydroxy-7-methoxy-2H-1-benzopyran-2-one
protonated HMDB0142084, spectral sim. = 930, #9
Struc. sim. = 0.59
2-hydroxy-3,4-dimethoxybenzoic acid
frequency, cm^{-1}

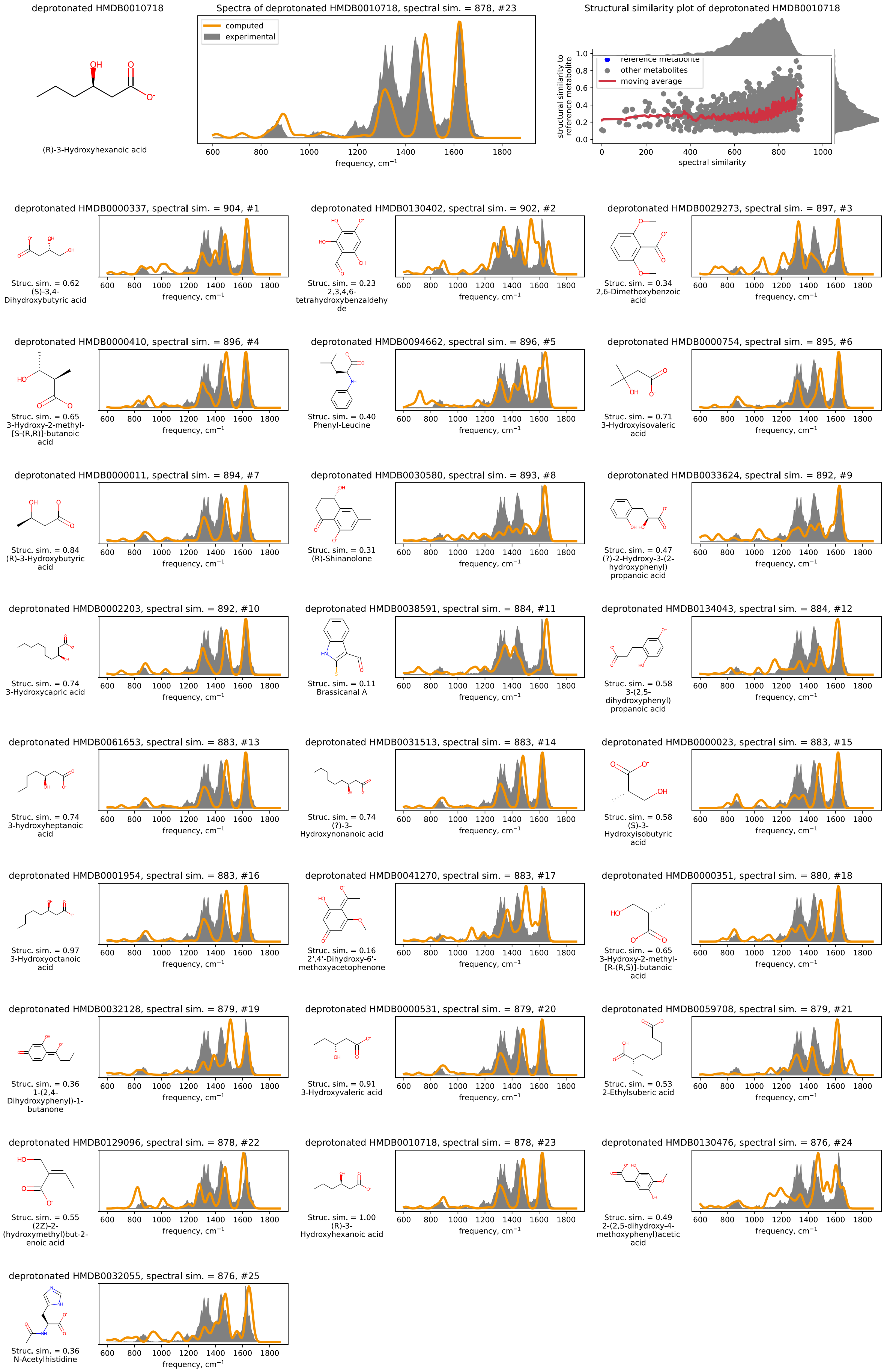
deprotonated HMDB0010718
(R)-3-Hydroxyhexanoic acid
Spectra of deprotonated HMDB0010718, spectral sim. = 878, #23
computed
experimental
frequency, cm⁻¹
Structural similarity plot of deprotonated HMDB0010718
reference metabolite
other metabolites
moving average
structural similarity to reference metabolite
spectral similarity
deprotonated HMDB0000337, spectral sim. = 904, #1
Struc. sim. = 0.62
(S)-3,4-Dihydroxybutyric acid
deprotonated HMDB0130402, spectral sim. = 902, #2
Struc. sim. = 0.23
2,3,4,6-tetrahydroxybenzaldehyde
deprotonated HMDB0029273, spectral sim. = 897, #3
Struc. sim. = 0.34
2,6-Dimethoxybenzoic acid
deprotonated HMDB0000410, spectral sim. = 896, #4
Struc. sim. = 0.65
3-Hydroxy-2-methyl-[S-(R,R)]-butanoic acid
deprotonated HMDB0094662, spectral sim. = 896, #5
Struc. sim. = 0.40
Phenyl-Leucine
deprotonated HMDB0000754, spectral sim. = 895, #6
Struc. sim. = 0.71
3-Hydroxyisovaleric acid
deprotonated HMDB0000011, spectral sim. = 894, #7
Struc. sim. = 0.84
(R)-3-Hydroxybutyric acid
deprotonated HMDB0030580, spectral sim. = 893, #8
Struc. sim. = 0.31
(R)-Shinanolone
deprotonated HMDB0033624, spectral sim. = 892, #9
Struc. sim. = 0.47
(?)-2-Hydroxy-3-(2-hydroxyphenyl)propanoic acid
deprotonated HMDB0002203, spectral sim. = 892, #10
Struc. sim. = 0.74
3-Hydroxycapric acid
deprotonated HMDB0038591, spectral sim. = 884, #11
Struc. sim. = 0.11
Brassicanal A
deprotonated HMDB0134043, spectral sim. = 884, #12
Struc. sim. = 0.58
3-(2,5-dihydroxyphenyl)propanoic acid
deprotonated HMDB0061653, spectral sim. = 883, #13
Struc. sim. = 0.74
3-hydroxyheptanoic acid
deprotonated HMDB0031513, spectral sim. = 883, #14
Struc. sim. = 0.74
(?)-3-Hydroxynonanoic acid
deprotonated HMDB0000023, spectral sim. = 883, #15
Struc. sim. = 0.58
(S)-3-Hydroxyisobutyric acid
deprotonated HMDB0001954, spectral sim. = 883, #16
Struc. sim. = 0.97
3-Hydroxyoctanoic acid
deprotonated HMDB0041270, spectral sim. = 883, #17
Struc. sim. = 0.16
2',4'-Dihydroxy-6'-methoxyacetophenone
deprotonated HMDB0000351, spectral sim. = 880, #18
Struc. sim. = 0.65
3-Hydroxy-2-methyl-[R-(R,S)]-butanoic acid
deprotonated HMDB0032128, spectral sim. = 879, #19
Struc. sim. = 0.36
1-(2,4-Dihydroxyphenyl)-1-butanone
deprotonated HMDB0000531, spectral sim. = 879, #20
Struc. sim. = 0.91
3-Hydroxyvaleric acid
deprotonated HMDB0059708, spectral sim. = 879, #21
Struc. sim. = 0.53
2-Ethylsuberic acid
deprotonated HMDB0129096, spectral sim. = 878, #22
Struc. sim. = 0.55
(2Z)-2-(hydroxymethyl)but-2-enoic acid
deprotonated HMDB0010718, spectral sim. = 878, #23
Struc. sim. = 1.00
(R)-3-Hydroxyhexanoic acid
deprotonated HMDB0130476, spectral sim. = 876, #24
Struc. sim. = 0.49
2-(2,5-dihydroxy-4-methoxyphenyl)acetic acid
deprotonated HMDB0032055, spectral sim. = 876, #25
Struc. sim. = 0.36
N-Acetylhistidine

166 protonated HMDB0012128

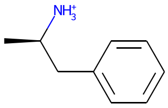

(R)-Amphetamine

Spectra of protonated HMDB0012128, spectral sim. = 787, #11

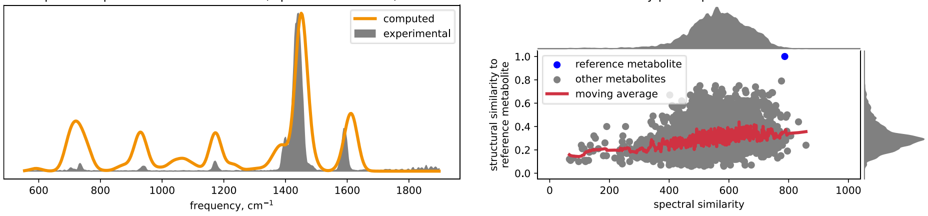

protonated HMDB0001377, spectral sim. = 858, #1

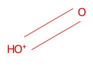

Struc. sim. = 0.24
Oxygen

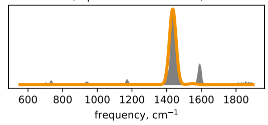

protonated HMDB0014621, spectral sim. = 833, #2

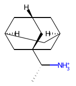

Struc. sim. = 0.44
Rimantadine

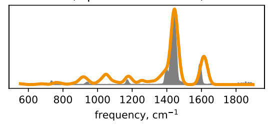

protonated HMDB0040003, spectral sim. = 807, #3

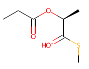

Struc. sim. = 0.32
Methylthio 2-(propanoyloxy) propanoate

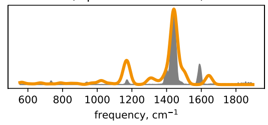

protonated HMDB0032871, spectral sim. = 802, #4

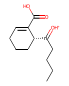

Struc. sim. = 0.32
Sedanonic acid

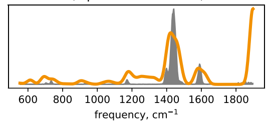

protonated HMDB0031151, spectral sim. = 796, #5

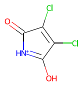

Struc. sim. = 0.19
Dichloromaleimide

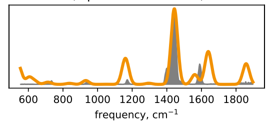

protonated HMDB0033968, spectral sim. = 795, #6

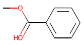

Struc. sim. = 0.50
Methyl benzoate

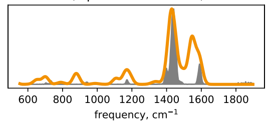

protonated HMDB0062667, spectral sim. = 794, #7

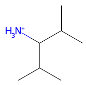

Struc. sim. = 0.36
2,4-dimethylpentan-3-amine

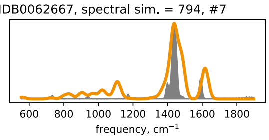

protonated HMDB0001892, spectral sim. = 791, #8

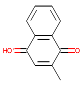

Struc. sim. = 0.48
Menadione

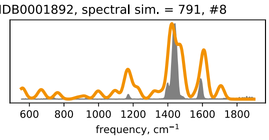

protonated HMDB0061017, spectral sim. = 790, #9

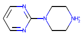

Struc. sim. = 0.28
1-Pyrimidinylpiperazine

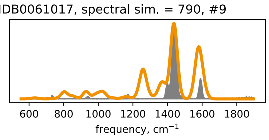

167 deprotonated HMDB0012140

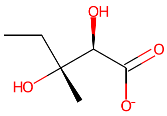

(R) 2,3-Dihydroxy-3-methylvalerate

Spectra of deprotonated HMDB0012140, spectral sim. = 890, #6

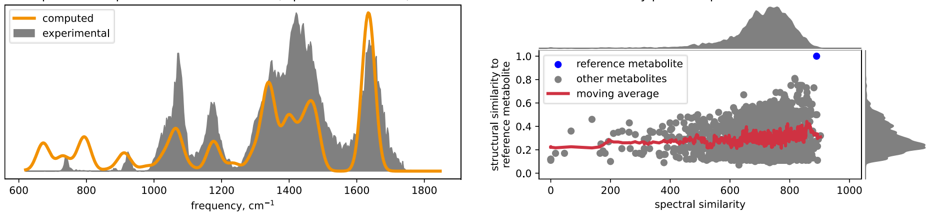

deprotonated HMDB0030580, spectral sim. = 903, #1

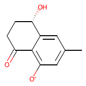

Struc. sim. = 0.32
(R)-Shinanolone

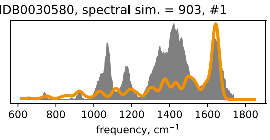

deprotonated HMDB0126620, spectral sim. = 896, #2

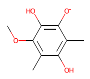

Struc. sim. = 0.29
6-methoxy-3,5-dimethylbenzene-1,2,4-triol

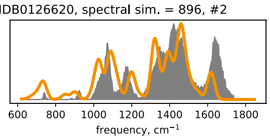

deprotonated HMDB0038591, spectral sim. = 893, #3

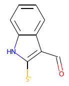

Struc. sim. = 0.11
Brassicanal A

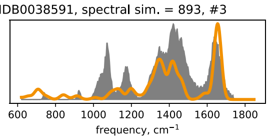

deprotonated HMDB0029650, spectral sim. = 893, #4

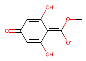

Struc. sim. = 0.23
Methyl 2,4,6-trihydroxybenzoate

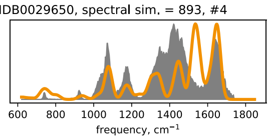

deprotonated HMDB0137136, spectral sim. = 891, #5

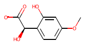

Struc. sim. = 0.50
2-hydroxy-2-(2-hydroxy-4-methoxyphenyl)acetic acid

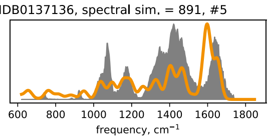

deprotonated HMDB0012140, spectral sim. = 890, #6

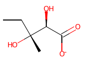

Struc. sim. = 1.00
(R) 2,3-Dihydroxy-3-methylvalerate

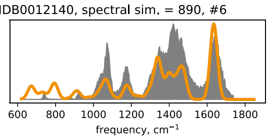

deprotonated HMDB0126480, spectral sim. = 888, #7

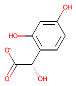

Struc. sim. = 0.50
2-(2,4-dihydroxyphenyl)-2-hydroxyacetic acid

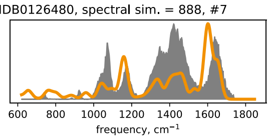

deprotonated HMDB0038712, spectral sim. = 887, #8

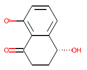

Struc. sim. = 0.26
(S)-Isosclerone

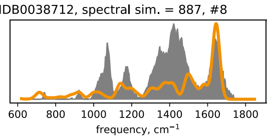

deprotonated HMDB0137818, spectral sim. = 885, #9

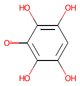

Struc. sim. = 0.27
benzene-1,2,3,4,5-pentol

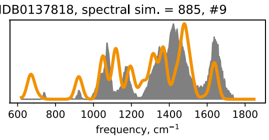

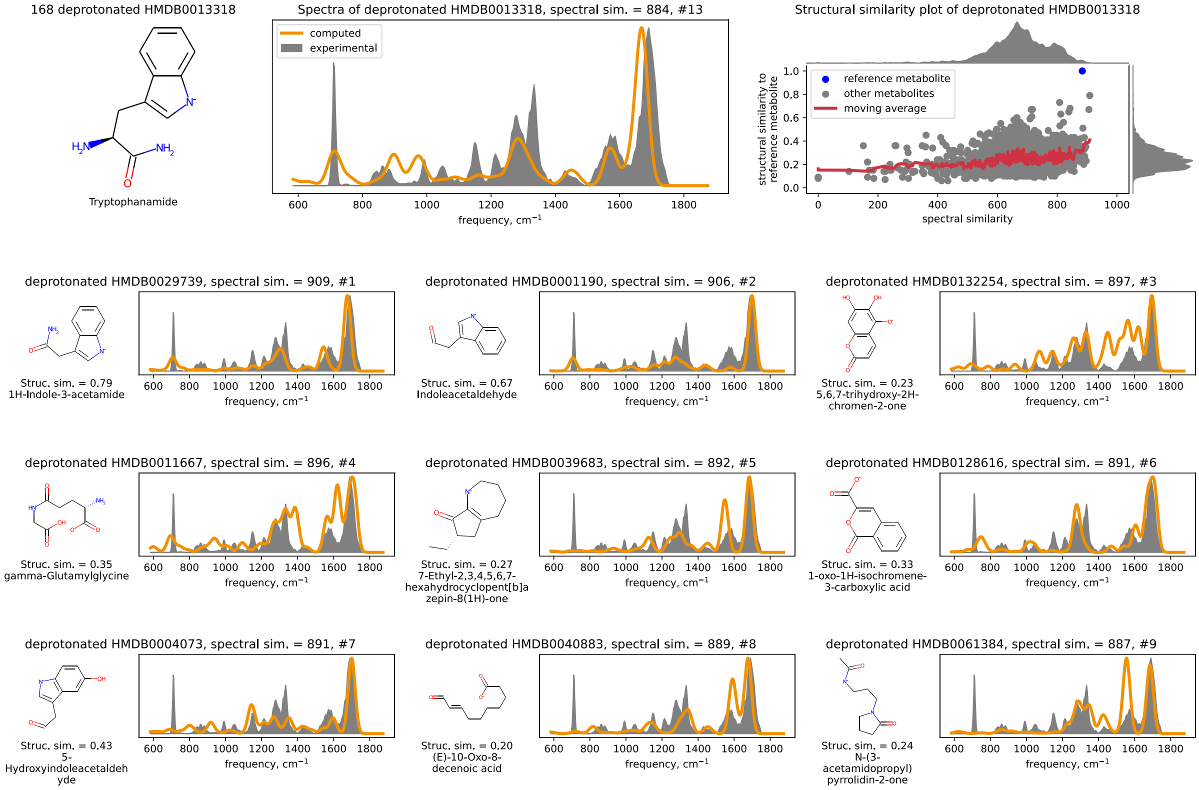
168 deprotonated HMDB0013318
Tryptophanamide
Spectra of deprotonated HMDB0013318, spectral sim. = 884, #13
computed
experimental
frequency, cm⁻¹
Structural similarity plot of deprotonated HMDB0013318
structural similarity to reference metabolite
reference metabolite
other metabolites
moving average
spectral similarity
deprotonated HMDB0029739, spectral sim. = 909, #1
Struc. sim. = 0.79
1H-Indole-3-acetamide
deprotonated HMDB0001190, spectral sim. = 906, #2
Struc. sim. = 0.67
Indoleacetaldehyde
deprotonated HMDB0132254, spectral sim. = 897, #3
Struc. sim. = 0.23
5,6,7-trihydroxy-2H-chromen-2-one
deprotonated HMDB0011667, spectral sim. = 896, #4
Struc. sim. = 0.35
gamma-Glutamylglycine
deprotonated HMDB0039683, spectral sim. = 892, #5
Struc. sim. = 0.27
7-Ethyl-2,3,4,5,6,7-hexahydrocyclopent[b]azepin-8(1H)-one
deprotonated HMDB0128616, spectral sim. = 891, #6
Struc. sim. = 0.33
1-oxo-1H-isochromene-3-carboxylic acid
deprotonated HMDB0004073, spectral sim. = 891, #7
Struc. sim. = 0.43
5-Hydroxyindoleacetaldehyde
deprotonated HMDB0040883, spectral sim. = 889, #8
Struc. sim. = 0.20
(E)-10-Oxo-8-decenoic acid
deprotonated HMDB0061384, spectral sim. = 887, #9
Struc. sim. = 0.24
N-(3-acetamidopropyl)pyrrolidin-2-one
frequency, cm⁻¹

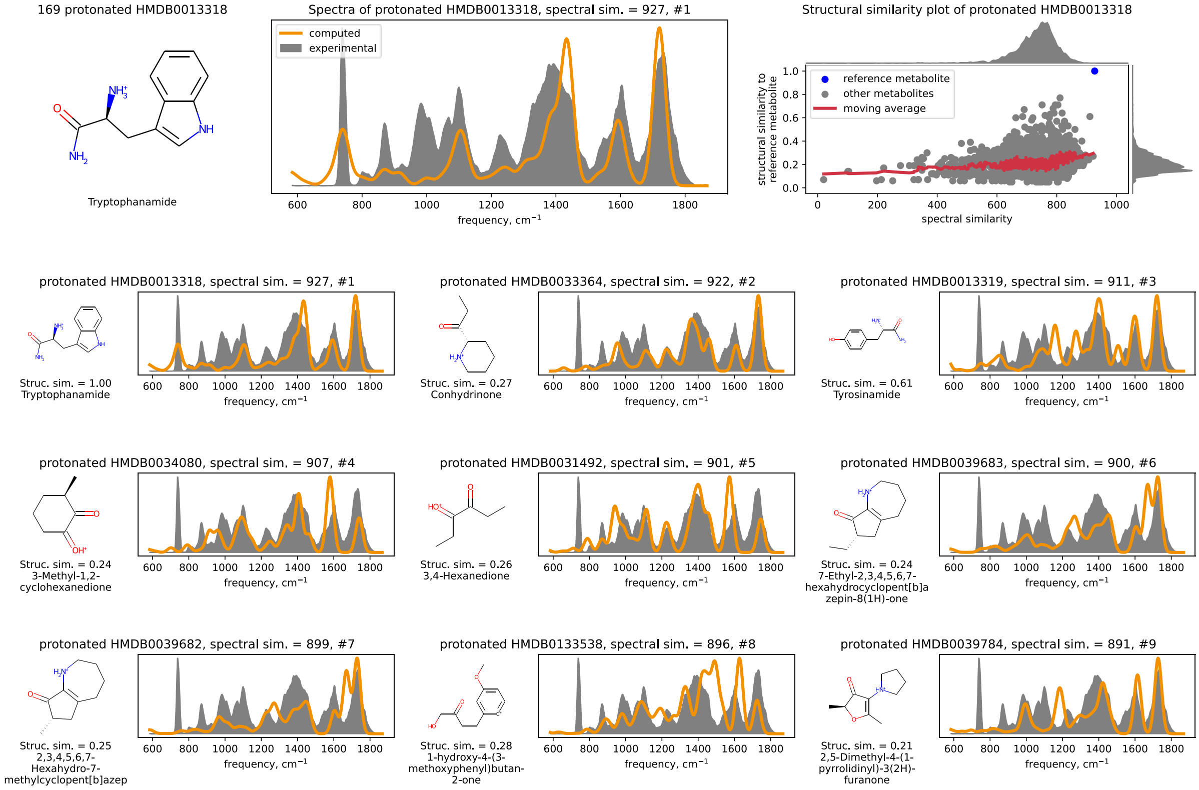
169 protonated HMDB0013318
Tryptophanamide
Spectra of protonated HMDB0013318, spectral sim. = 927, #1
computed
experimental
frequency, cm⁻¹
Structural similarity plot of protonated HMDB0013318
structural similarity to reference metabolite
reference metabolite
other metabolites
moving average
spectral similarity
protonated HMDB0013318, spectral sim. = 927, #1
Struc. sim. = 1.00
Tryptophanamide
protonated HMDB0033364, spectral sim. = 922, #2
Struc. sim. = 0.27
Conhydrinone
protonated HMDB0013319, spectral sim. = 911, #3
Struc. sim. = 0.61
Tyrosinamide
protonated HMDB0034080, spectral sim. = 907, #4
Struc. sim. = 0.24
3-Methyl-1,2-cyclohexanedione
protonated HMDB0031492, spectral sim. = 901, #5
Struc. sim. = 0.26
3,4-Hexanedione
protonated HMDB0039683, spectral sim. = 900, #6
Struc. sim. = 0.24
7-Ethyl-2,3,4,5,6,7-hexahydrocyclopent[b]azepin-8(1H)-one
protonated HMDB0039682, spectral sim. = 899, #7
Struc. sim. = 0.25
2,3,4,5,6,7-Hexahydro-7-methylcyclopent[b]azep
protonated HMDB0133538, spectral sim. = 896, #8
Struc. sim. = 0.28
1-hydroxy-4-(3-methoxyphenyl)butan-2-one
protonated HMDB0039784, spectral sim. = 891, #9
Struc. sim. = 0.21
2,5-Dimethyl-4-(1-pyrrolidinyl)-3(2H)-furanone
frequency, cm⁻¹

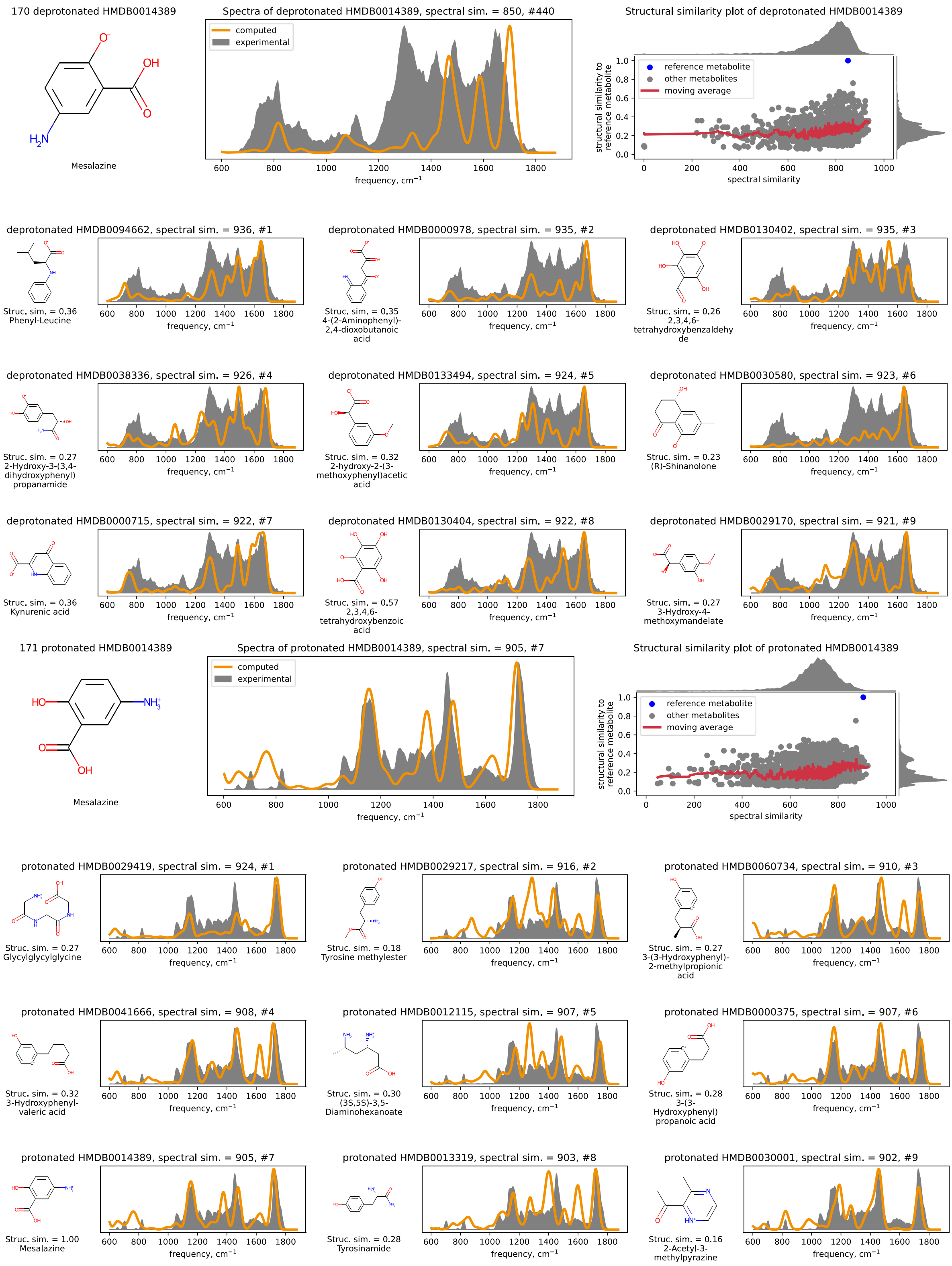
170 deprotonated HMDB0014389
Mesalazine
Spectra of deprotonated HMDB0014389, spectral sim. = 850, #440
computed
experimental
frequency, cm−1
Structural similarity plot of deprotonated HMDB0014389
reference metabolite
other metabolites
moving average
structural similarity to reference metabolite
spectral similarity
deprotonated HMDB0094662, spectral sim. = 936, #1
Struc. sim. = 0.36
Phenyl-Leucine
deprotonated HMDB0000978, spectral sim. = 935, #2
Struc. sim. = 0.35
4-(2-Aminophenyl)-2,4-dioxobutanoic acid
deprotonated HMDB0130402, spectral sim. = 935, #3
Struc. sim. = 0.26
2,3,4,6-tetrahydroxybenzaldehyde
deprotonated HMDB0038336, spectral sim. = 926, #4
Struc. sim. = 0.27
2-Hydroxy-3-(3,4-dihydroxyphenyl) propanamide
deprotonated HMDB0133494, spectral sim. = 924, #5
Struc. sim. = 0.32
2-hydroxy-2-(3-methoxyphenyl)acetic acid
deprotonated HMDB0030580, spectral sim. = 923, #6
Struc. sim. = 0.23
(R)-Shinanolone
deprotonated HMDB0000715, spectral sim. = 922, #7
Struc. sim. = 0.36
Kynurenic acid
deprotonated HMDB0130404, spectral sim. = 922, #8
Struc. sim. = 0.57
2,3,4,6-tetrahydroxybenzoic acid
deprotonated HMDB0029170, spectral sim. = 921, #9
Struc. sim. = 0.27
3-Hydroxy-4-methoxymandelate
171 protonated HMDB0014389
Mesalazine
Spectra of protonated HMDB0014389, spectral sim. = 905, #7
Structural similarity plot of protonated HMDB0014389
protonated HMDB0029419, spectral sim. = 924, #1
Struc. sim. = 0.27
Glycylglycylglycine
protonated HMDB0029217, spectral sim. = 916, #2
Struc. sim. = 0.18
Tyrosine methylester
protonated HMDB0060734, spectral sim. = 910, #3
Struc. sim. = 0.27
3-(3-Hydroxyphenyl)-2-methylpropionic acid
protonated HMDB0041666, spectral sim. = 908, #4
Struc. sim. = 0.32
3-Hydroxyphenyl-valeric acid
protonated HMDB0012115, spectral sim. = 907, #5
Struc. sim. = 0.30
(3S,5S)-3,5-Diaminohexanoate
protonated HMDB0000375, spectral sim. = 907, #6
Struc. sim. = 0.28
3-(3-Hydroxyphenyl) propanoic acid
protonated HMDB0014389, spectral sim. = 905, #7
Struc. sim. = 1.00
Mesalazine
protonated HMDB0013319, spectral sim. = 903, #8
Struc. sim. = 0.28
Tyrosinamide
protonated HMDB0030001, spectral sim. = 902, #9
Struc. sim. = 0.16
2-Acetyl-3-methylpyrazine

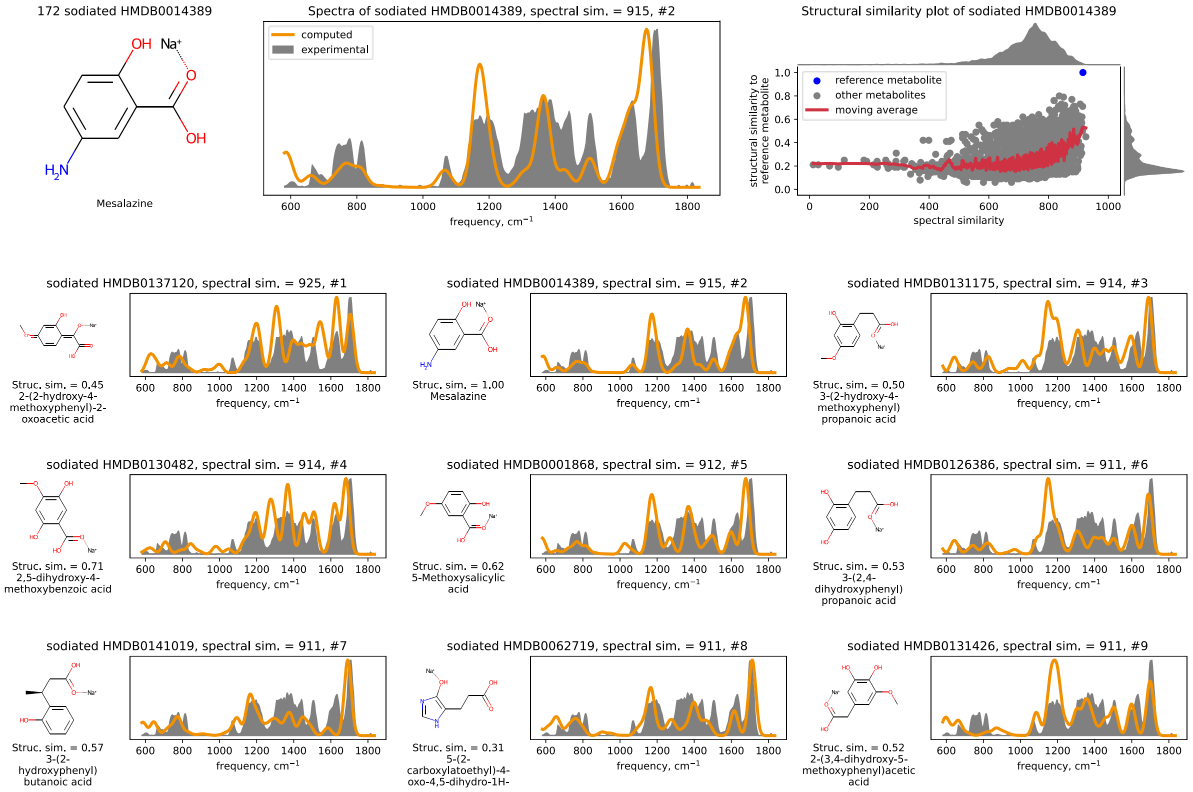
172 sodiated HMDB0014389
Mesalazine
Spectra of sodiated HMDB0014389, spectral sim. = 915, #2
computed
experimental
frequency, cm^{-1}
Structural similarity plot of sodiated HMDB0014389
reference metabolite
other metabolites
moving average
structural similarity to reference metabolite
spectral similarity
sodiated HMDB0137120, spectral sim. = 925, #1
Struc. sim. = 0.45
2-(2-hydroxy-4-methoxyphenyl)-2-oxoacetic acid
sodiated HMDB0014389, spectral sim. = 915, #2
Struc. sim. = 1.00
Mesalazine
sodiated HMDB0131175, spectral sim. = 914, #3
Struc. sim. = 0.50
3-(2-hydroxy-4-methoxyphenyl) propanoic acid
sodiated HMDB0130482, spectral sim. = 914, #4
Struc. sim. = 0.71
2,5-dihydroxy-4-methoxybenzoic acid
sodiated HMDB0001868, spectral sim. = 912, #5
Struc. sim. = 0.62
5-Methoxysalicylic acid
sodiated HMDB0126386, spectral sim. = 911, #6
Struc. sim. = 0.53
3-(2,4-dihydroxyphenyl) propanoic acid
sodiated HMDB0141019, spectral sim. = 911, #7
Struc. sim. = 0.57
3-(2-hydroxyphenyl) butanoic acid
sodiated HMDB0062719, spectral sim. = 911, #8
Struc. sim. = 0.31
5-(2-carboxylatoethyl)-4-oxo-4,5-dihydro-1H-
sodiated HMDB0131426, spectral sim. = 911, #9
Struc. sim. = 0.52
2-(3,4-dihydroxy-5-methoxyphenyl)acetic acid

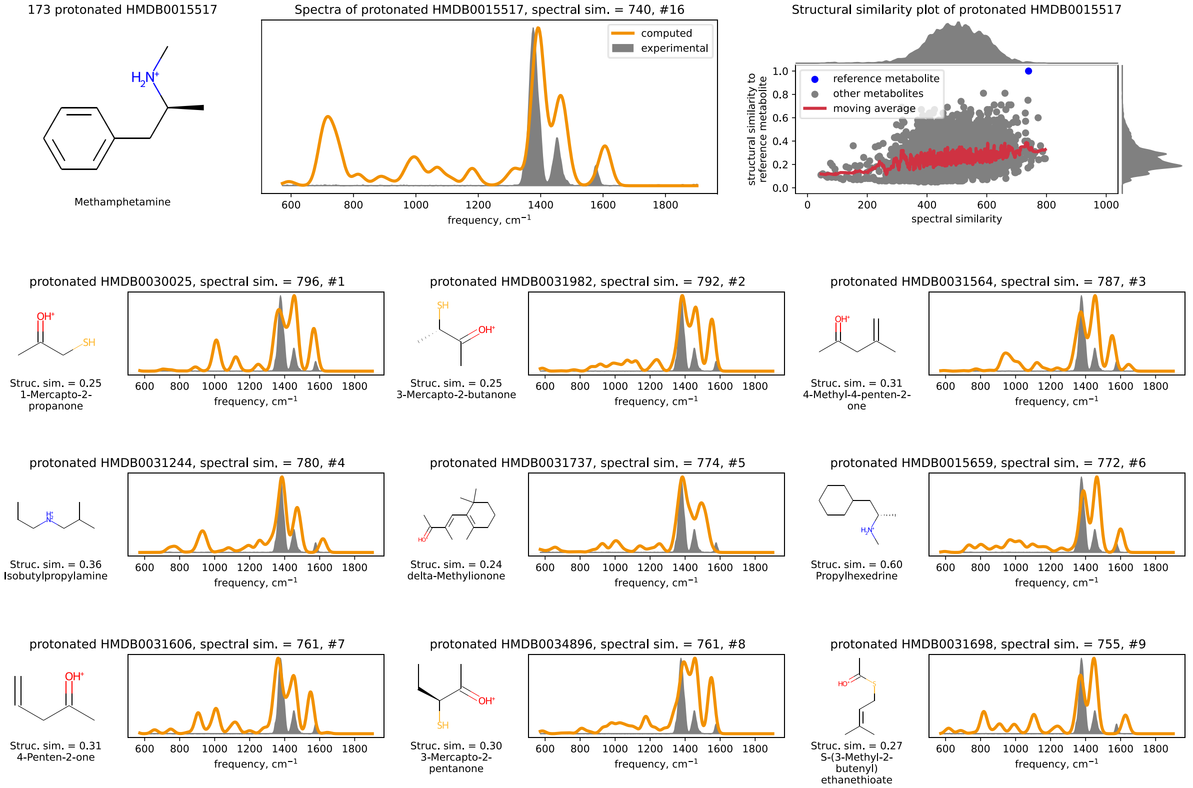
173 protonated HMDB0015517
Methamphetamine
Spectra of protonated HMDB0015517, spectral sim. = 740, #16
computed
experimental
frequency, cm^{-1}
Structural similarity plot of protonated HMDB0015517
reference metabolite
other metabolites
moving average
structural similarity to reference metabolite
spectral similarity
protonated HMDB0030025, spectral sim. = 796, #1
Struc. sim. = 0.25
1-Mercapto-2-propanone
protonated HMDB0031982, spectral sim. = 792, #2
Struc. sim. = 0.25
3-Mercapto-2-butanone
protonated HMDB0031564, spectral sim. = 787, #3
Struc. sim. = 0.31
4-Methyl-4-penten-2-one
protonated HMDB0031244, spectral sim. = 780, #4
Struc. sim. = 0.36
Isobutylpropylamine
protonated HMDB0031737, spectral sim. = 774, #5
Struc. sim. = 0.24
delta-Methylionone
protonated HMDB0015659, spectral sim. = 772, #6
Struc. sim. = 0.60
Propylhexedrine
protonated HMDB0031606, spectral sim. = 761, #7
Struc. sim. = 0.31
4-Penten-2-one
protonated HMDB0034896, spectral sim. = 761, #8
Struc. sim. = 0.30
3-Mercapto-2-pentanone
protonated HMDB0031698, spectral sim. = 755, #9
Struc. sim. = 0.27
S-(3-Methyl-2-butenyl) ethanethioate

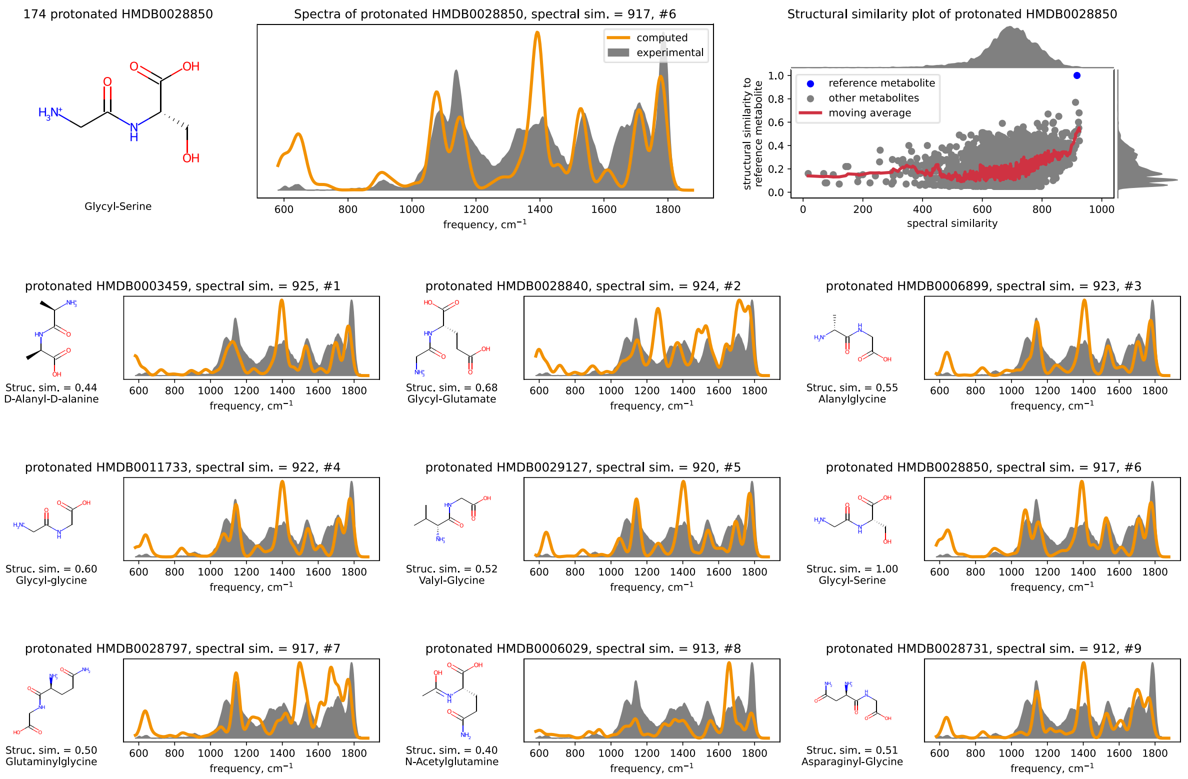
174 protonated HMDB0028850
Glycyl-Serine
Spectra of protonated HMDB0028850, spectral sim. = 917, #6
computed
experimental
frequency, cm⁻¹
Structural similarity plot of protonated HMDB0028850
reference metabolite
other metabolites
moving average
structural similarity to reference metabolite
spectral similarity
protonated HMDB0003459, spectral sim. = 925, #1
Struc. sim. = 0.44
D-Alanyl-D-alanine
protonated HMDB0028840, spectral sim. = 924, #2
Struc. sim. = 0.68
Glycyl-Glutamate
protonated HMDB0006899, spectral sim. = 923, #3
Struc. sim. = 0.55
Alanylglycine
protonated HMDB0011733, spectral sim. = 922, #4
Struc. sim. = 0.60
Glycyl-glycine
protonated HMDB0029127, spectral sim. = 920, #5
Struc. sim. = 0.52
Valyl-Glycine
protonated HMDB0028850, spectral sim. = 917, #6
Struc. sim. = 1.00
Glycyl-Serine
protonated HMDB0028797, spectral sim. = 917, #7
Struc. sim. = 0.50
Glutaminylglycine
protonated HMDB0006029, spectral sim. = 913, #8
Struc. sim. = 0.40
N-Acetylglutamine
protonated HMDB0028731, spectral sim. = 912, #9
Struc. sim. = 0.51
Asparaginyl-Glycine

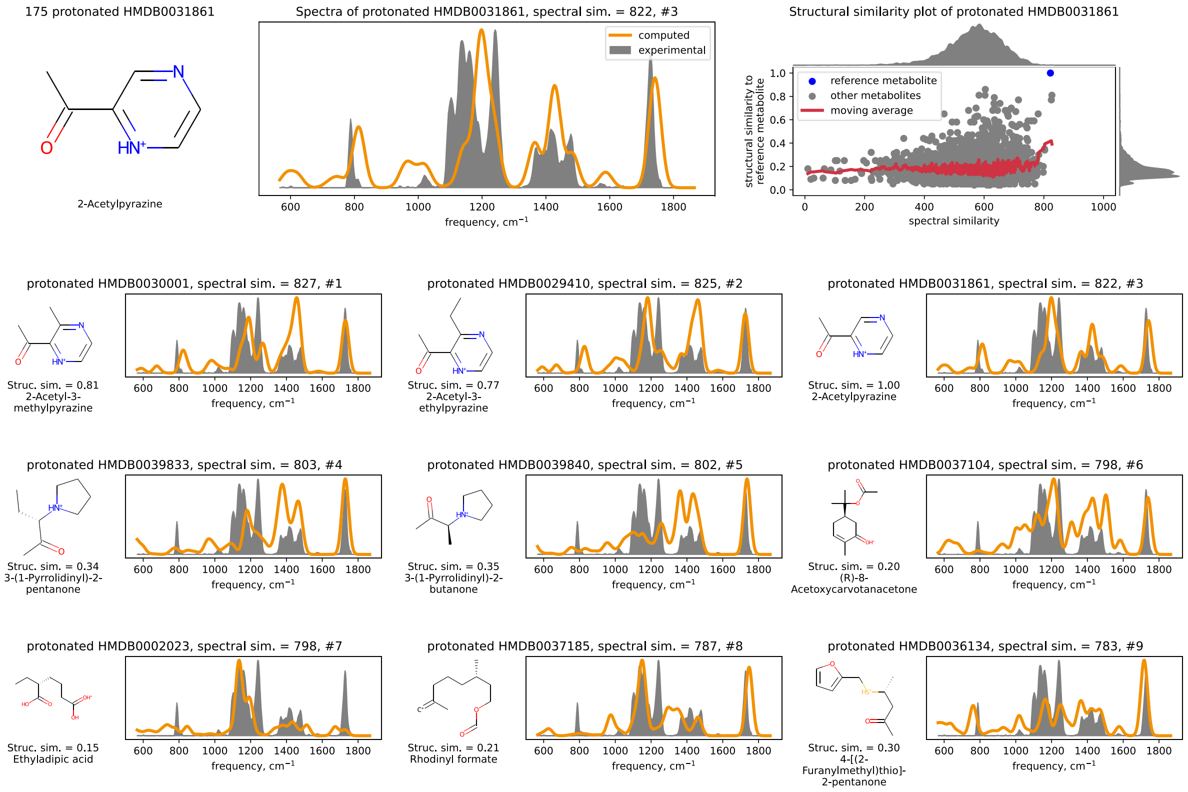
175 protonated HMDB0031861
2-Acetylpyrazine
Spectra of protonated HMDB0031861, spectral sim. = 822, #3
computed
experimental
frequency, cm⁻¹
Structural similarity plot of protonated HMDB0031861
reference metabolite
other metabolites
moving average
structural similarity to reference metabolite
spectral similarity
protonated HMDB0030001, spectral sim. = 827, #1
Struc. sim. = 0.81
2-Acetyl-3-methylpyrazine
protonated HMDB0029410, spectral sim. = 825, #2
Struc. sim. = 0.77
2-Acetyl-3-ethylpyrazine
protonated HMDB0031861, spectral sim. = 822, #3
Struc. sim. = 1.00
2-Acetylpyrazine
protonated HMDB0039833, spectral sim. = 803, #4
Struc. sim. = 0.34
3-(1-Pyrrolidinyl)-2-pentanone
protonated HMDB0039840, spectral sim. = 802, #5
Struc. sim. = 0.35
3-(1-Pyrrolidinyl)-2-butanone
protonated HMDB0037104, spectral sim. = 798, #6
Struc. sim. = 0.20
(R)-8-Acetoxycarvotanacetone
protonated HMDB0002023, spectral sim. = 798, #7
Struc. sim. = 0.15
Ethyladipic acid
protonated HMDB0037185, spectral sim. = 787, #8
Struc. sim. = 0.21
Rhodinyl formate
protonated HMDB0036134, spectral sim. = 783, #9
Struc. sim. = 0.30
4-[(2-Furanylmethyl)thio]-2-pentanone

176 protonated HMDB0036458

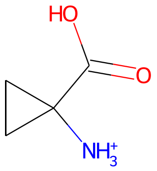

1-Aminocyclopropanecarboxylic acid

Spectra of protonated HMDB0036458, spectral sim. = 874, #2

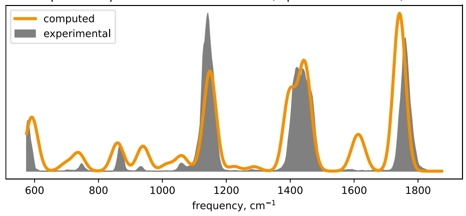

Structural similarity plot of protonated HMDB0036458

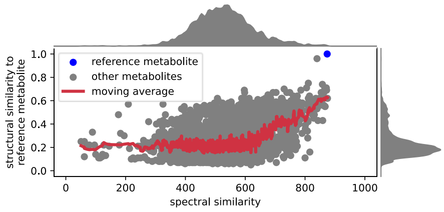

protonated HMDB0000687, spectral sim. = 875, #1

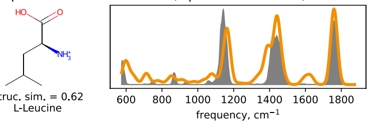

Struc. sim. = 0.62
L-Leucine

protonated HMDB0036458, spectral sim. = 874, #2

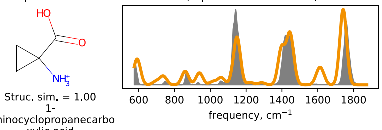

Struc. sim. = 1.00
1-Aminocyclopropanecarboxylic acid

protonated HMDB0094649, spectral sim. = 872, #3

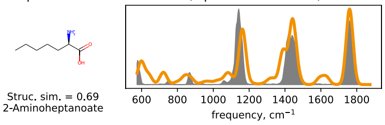

Struc. sim. = 0.69
2-Aminoheptanoate

protonated HMDB0013716, spectral sim. = 871, #4

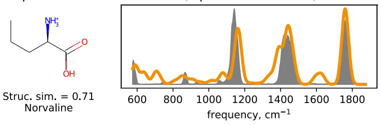

Struc. sim. = 0.71
Norvaline

protonated HMDB0002108, spectral sim. = 867, #5

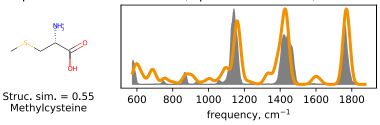

Struc. sim. = 0.55
Methylcysteine

protonated HMDB0003585, spectral sim. = 867, #6

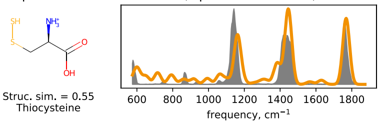

Struc. sim. = 0.55
Thiocysteine

protonated HMDB0004113, spectral sim. = 866, #7

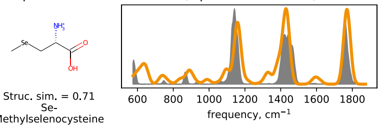

Struc. sim. = 0.71
Se-Methylselenocysteine

protonated HMDB0034324, spectral sim. = 863, #8

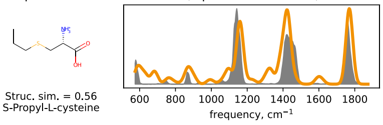

Struc. sim. = 0.56
S-Propyl-L-cysteine

protonated HMDB0000172, spectral sim. = 863, #9

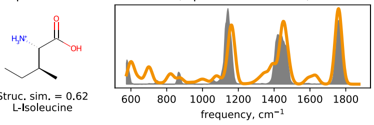

Struc. sim. = 0.62
L-Isoleucine

177 sodiated HMDB0036458

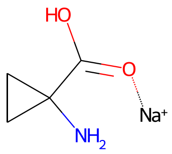

1-Aminocyclopropanecarboxylic acid

Spectra of sodiated HMDB0036458, spectral sim. = 863, #14

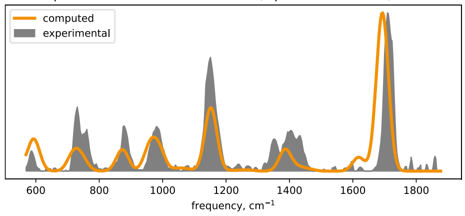

Structural similarity plot of sodiated HMDB0036458

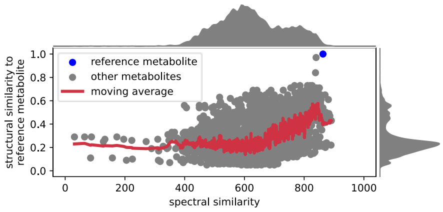

sodiated HMDB0059765, spectral sim. = 891, #1

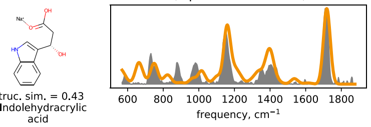

Struc. sim. = 0.43
3-Indolehydracrylic acid

sodiated HMDB0133754, spectral sim. = 890, #2

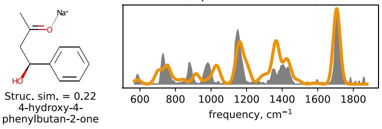

Struc. sim. = 0.22
4-hydroxy-4-phenylbutan-2-one

sodiated HMDB0002229, spectral sim. = 886, #3

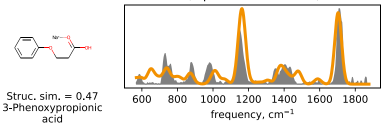

Struc. sim. = 0.47
3-Phenoxypropionic acid

sodiated HMDB0124925, spectral sim. = 885, #4

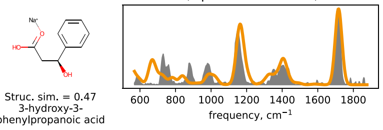

Struc. sim. = 0.47
3-hydroxy-3-phenylpropanoic acid

sodiated HMDB0031517, spectral sim. = 883, #5

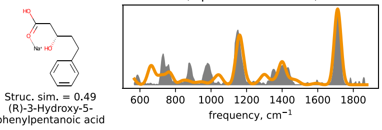

Struc. sim. = 0.49
(R)-3-Hydroxy-5-phenylpentanoic acid

sodiated HMDB0061877, spectral sim. = 881, #6

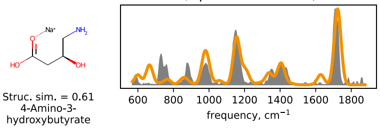

Struc. sim. = 0.61
4-Amino-3-hydroxybutyrate

sodiated HMDB0040230, spectral sim. = 875, #7

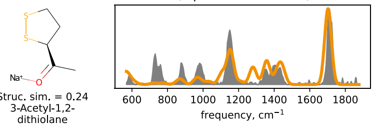

Struc. sim. = 0.24
3-Acetyl-1,2-dithiolane

sodiated HMDB0002302, spectral sim. = 873, #8

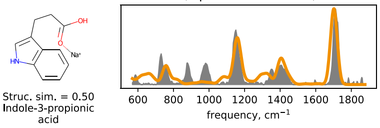

Struc. sim. = 0.50
Indole-3-propionic acid

sodiated HMDB0141022, spectral sim. = 872, #9

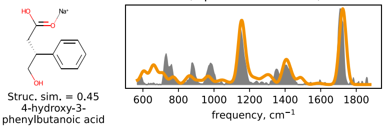

Struc. sim. = 0.45
4-hydroxy-3-phenylbutanoic acid

178 protonated HMDB0041923

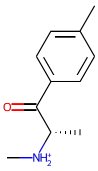

Mephedrone

Spectra of protonated HMDB0041923, spectral sim. = 840, #1

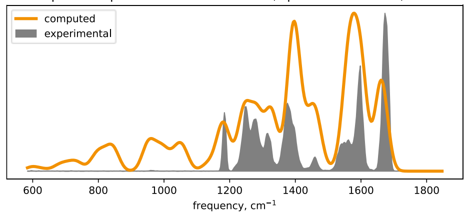

Structural similarity plot of protonated HMDB0041923

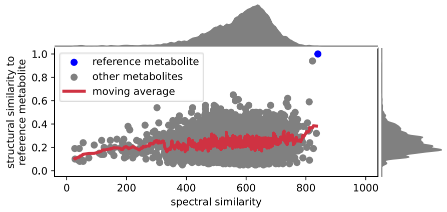

protonated HMDB0041923, spectral sim. = 840, #1

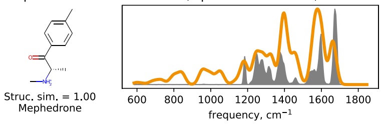

Struc. sim. = 1.00
Mephedrone

protonated HMDB0033568, spectral sim. = 835, #2

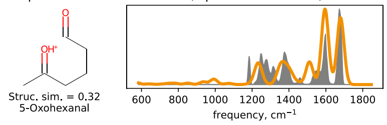

Struc. sim. = 0.32
5-Oxohexanal

protonated HMDB0130402, spectral sim. = 825, #3

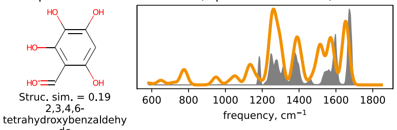

Struc. sim. = 0.19
2,3,4,6-tetrahydroxybenzaldehyde

protonated HMDB0041927, spectral sim. = 822, #4

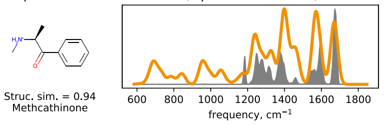

Struc. sim. = 0.94
Methcathinone

protonated HMDB0032565, spectral sim. = 813, #5

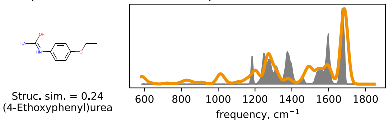

Struc. sim. = 0.24
(4-Ethoxyphenyl)urea

protonated HMDB0061681, spectral sim. = 812, #6

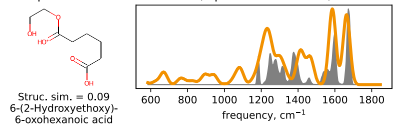

Struc. sim. = 0.09
6-(2-Hydroxyethoxy)-6-oxohexanoic acid

protonated HMDB0037155, spectral sim. = 812, #7

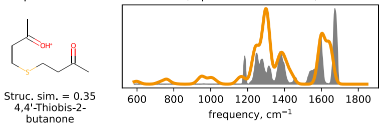

Struc. sim. = 0.35
4,4'-Thiobis-2-butanone

protonated HMDB0015072, spectral sim. = 811, #8

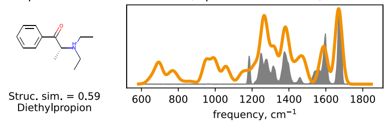

Struc. sim. = 0.59
Diethylpropion

protonated HMDB0000859, spectral sim. = 809, #9

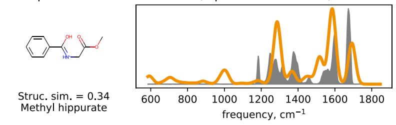

Struc. sim. = 0.34
Methyl hippurate

179 protonated HMDB0041931

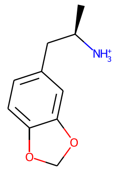

3,4-Methylenedioxyamphetamine

Spectra of protonated HMDB0041931, spectral sim. = 924, #1

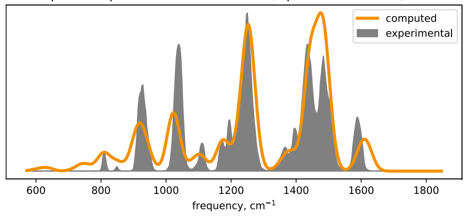

Structural similarity plot of protonated HMDB0041931

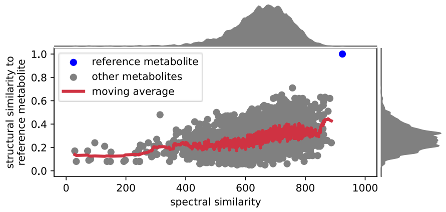

protonated HMDB0041931, spectral sim. = 924, #1

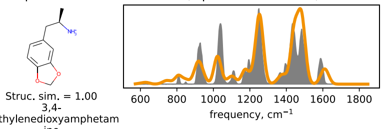

Struc. sim. = 1.00
3,4-Methylenedioxyamphetamine

protonated HMDB0033569, spectral sim. = 887, #2

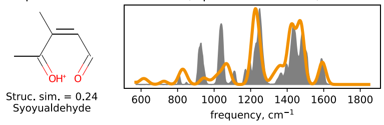

Struc. sim. = 0.24
Syoyualdehyde

protonated HMDB0041481, spectral sim. = 882, #3

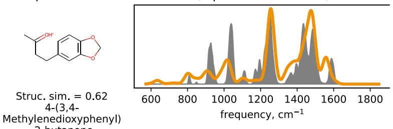

Struc. sim. = 0.62
4-(3,4-Methylenedioxyphenyl)-2-butanone

protonated HMDB0060748, spectral sim. = 876, #4

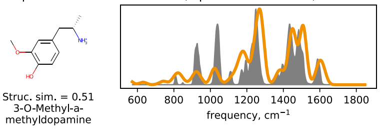

Struc. sim. = 0.51
3-O-Methyl-a-methyldopamine

protonated HMDB0032276, spectral sim. = 873, #5

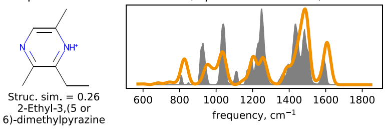

Struc. sim. = 0.26
2-Ethyl-3,(5 or 6)-dimethylpyrazine

protonated HMDB0035894, spectral sim. = 866, #6

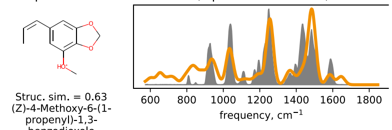

Struc. sim. = 0.63
(Z)-4-Methoxy-6-(1-propenyl)-1,3-benzodioxole

protonated HMDB0031841, spectral sim. = 865, #7

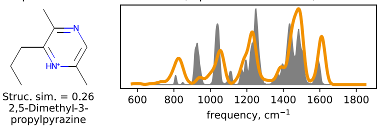

Struc. sim. = 0.26
2,5-Dimethyl-3-propylpyrazine

protonated HMDB0035873, spectral sim. = 861, #8

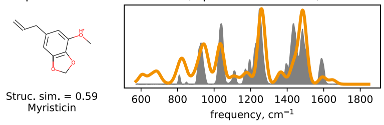

Struc. sim. = 0.59
Myristicin

protonated HMDB0032443, spectral sim. = 861, #9

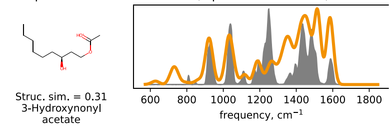

Struc. sim. = 0.31
3-Hydroxynonyl acetate

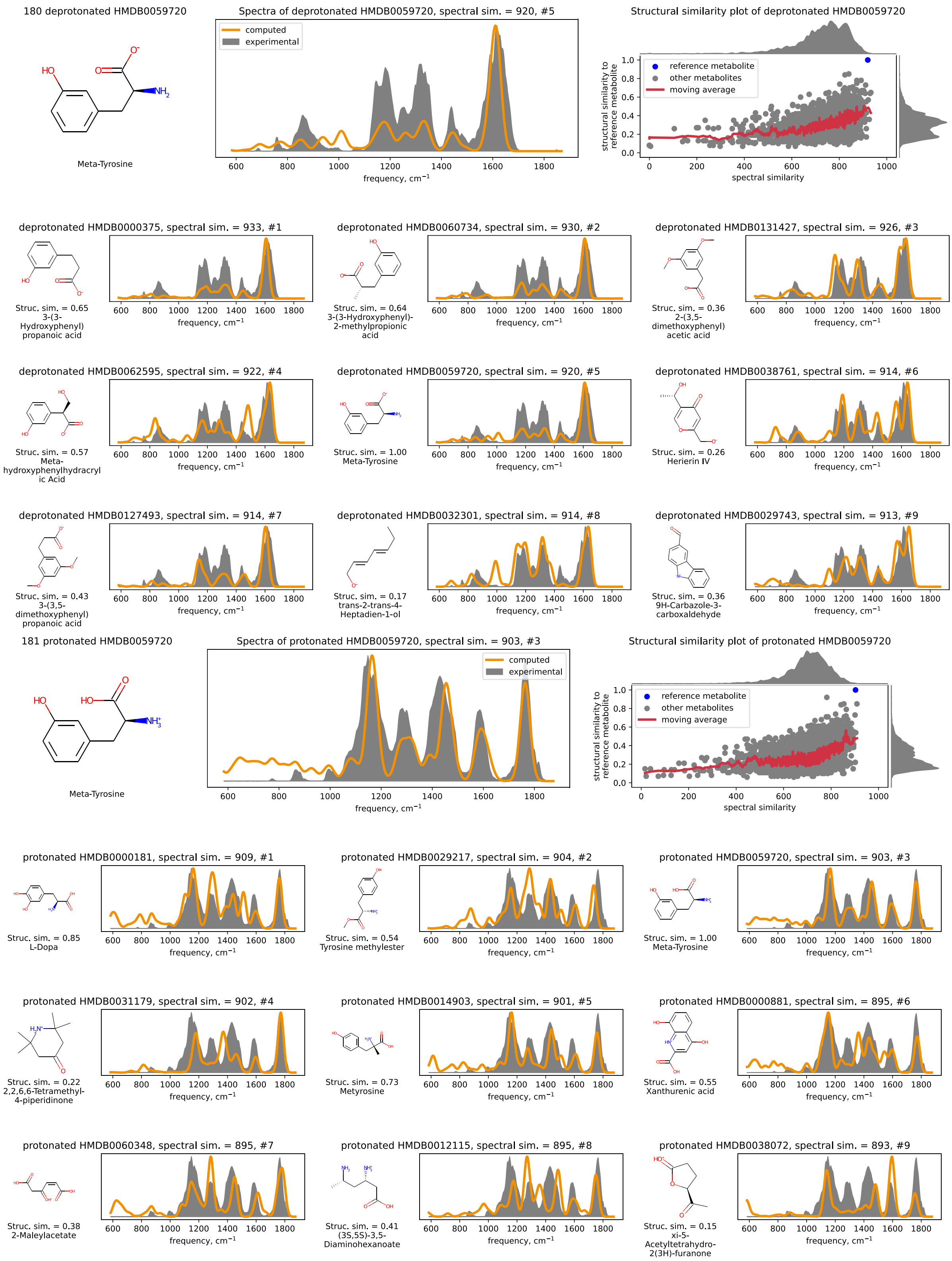
180 deprotonated HMDB0059720
Meta-Tyrosine
Spectra of deprotonated HMDB0059720, spectral sim. = 920, #5
computed
experimental
frequency, cm⁻¹
Structural similarity plot of deprotonated HMDB0059720
reference metabolite
other metabolites
moving average
structural similarity to reference metabolite
spectral similarity
deprotonated HMDB0000375, spectral sim. = 933, #1
Struc. sim. = 0.65
3-(3-Hydroxyphenyl) propanoic acid
deprotonated HMDB0060734, spectral sim. = 930, #2
Struc. sim. = 0.64
3-(3-Hydroxyphenyl)-2-methylpropionic acid
deprotonated HMDB0131427, spectral sim. = 926, #3
Struc. sim. = 0.36
2-(3,5-dimethoxyphenyl) acetic acid
deprotonated HMDB0062595, spectral sim. = 922, #4
Struc. sim. = 0.57
Meta-hydroxyphenylhydracrylic Acid
deprotonated HMDB0059720, spectral sim. = 920, #5
Struc. sim. = 1.00
Meta-Tyrosine
deprotonated HMDB0038761, spectral sim. = 914, #6
Struc. sim. = 0.26
Herierin IV
deprotonated HMDB0127493, spectral sim. = 914, #7
Struc. sim. = 0.43
3-(3,5-dimethoxyphenyl) propanoic acid
deprotonated HMDB0032301, spectral sim. = 914, #8
Struc. sim. = 0.17
trans-2-trans-4-Heptadien-1-ol
deprotonated HMDB0029743, spectral sim. = 913, #9
Struc. sim. = 0.36
9H-Carbazole-3-carboxaldehyde
181 protonated HMDB0059720
Meta-Tyrosine
Spectra of protonated HMDB0059720, spectral sim. = 903, #3
computed
experimental
frequency, cm⁻¹
Structural similarity plot of protonated HMDB0059720
reference metabolite
other metabolites
moving average
structural similarity to reference metabolite
spectral similarity
protonated HMDB0000181, spectral sim. = 909, #1
Struc. sim. = 0.85
L-Dopa
protonated HMDB0029217, spectral sim. = 904, #2
Struc. sim. = 0.54
Tyrosine methylester
protonated HMDB0059720, spectral sim. = 903, #3
Struc. sim. = 1.00
Meta-Tyrosine
protonated HMDB0031179, spectral sim. = 902, #4
Struc. sim. = 0.22
2,2,6,6-Tetramethyl-4-piperidinone
protonated HMDB0014903, spectral sim. = 901, #5
Struc. sim. = 0.73
Metyrosine
protonated HMDB0000881, spectral sim. = 895, #6
Struc. sim. = 0.55
Xanthurenic acid
protonated HMDB0060348, spectral sim. = 895, #7
Struc. sim. = 0.38
2-Maleylacetate
protonated HMDB0012115, spectral sim. = 895, #8
Struc. sim. = 0.41
(3S,5S)-3,5-Diaminohexanoate
protonated HMDB0038072, spectral sim. = 893, #9
Struc. sim. = 0.15
xi-5-Acetyltetrahydro-2(3H)-furanone
frequency, cm⁻¹

182 sodiated HMDB0059720

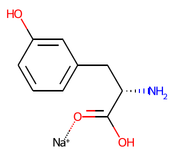

Meta-Tyrosine

Spectra of sodiated HMDB0059720, spectral sim. = 884, #14

Structural similarity plot of sodiated HMDB0059720

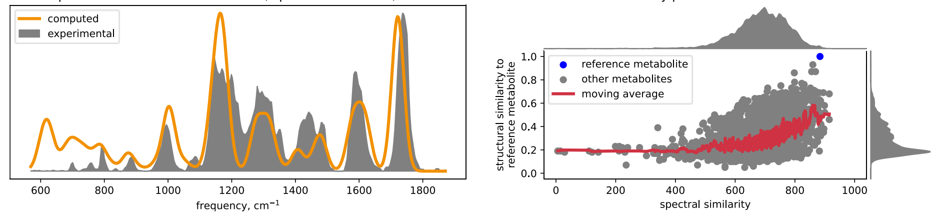

sodiated HMDB0000822, spectral sim. = 915, #1

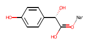

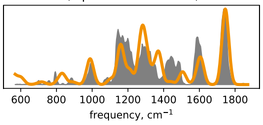

Struc. sim. = 0.68
p-Hydroxymandelic acid

sodiated HMDB0041692, spectral sim. = 915, #2

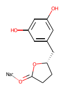

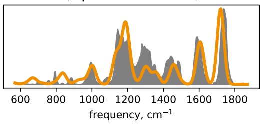

Struc. sim. = 0.46
5-(3',5'-Dihydroxyphenyl)-gamma-valerolactone

sodiated HMDB0133490, spectral sim. = 900, #3

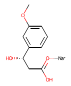

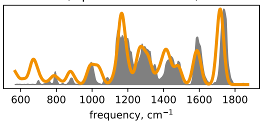

Struc. sim. = 0.52
3-hydroxy-3-(3-methoxyphenyl) propanoic acid

sodiated HMDB0140897, spectral sim. = 899, #4

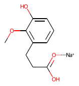

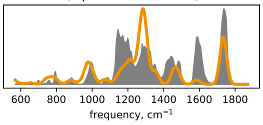

Struc. sim. = 0.58
3-(3-hydroxy-2-methoxyphenyl) propanoic acid

sodiated HMDB0133484, spectral sim. = 897, #5

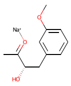

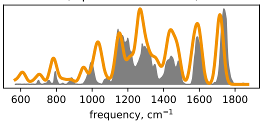

Struc. sim. = 0.38
3-hydroxy-4-(3-methoxyphenyl)butan-2-one

sodiated HMDB0135740, spectral sim. = 896, #6

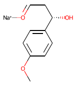

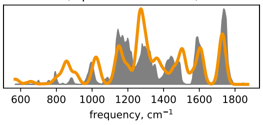

Struc. sim. = 0.35
3-hydroxy-3-(4-methoxyphenyl) propanal

sodiated HMDB0000375, spectral sim. = 895, #7

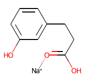

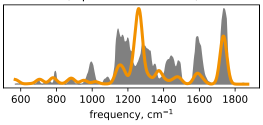

Struc. sim. = 0.69
3-(3-Hydroxyphenyl) propanoic acid

sodiated HMDB0141263, spectral sim. = 888, #8

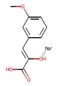

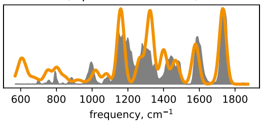

Struc. sim. = 0.40
(2Z)-2-hydroxy-3-(3-methoxyphenyl)prop-2-enoic acid

sodiated HMDB0134032, spectral sim. = 887, #9

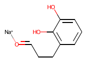

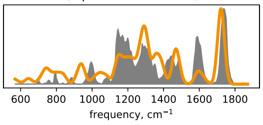

Struc. sim. = 0.42
3-(2,3-dihydroxyphenyl) propanal

183 deprotonated HMDB0060608

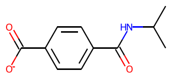

N-isopropylterephthalamic acid

Spectra of deprotonated HMDB0060608, spectral sim. = 824, #38

Structural similarity plot of deprotonated HMDB0060608

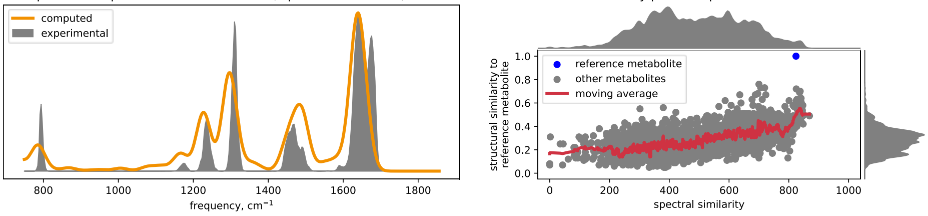

deprotonated HMDB0000832, spectral sim. = 869, #1

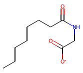

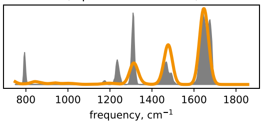

Struc. sim. = 0.49
Capryloylglycine

deprotonated HMDB0000808, spectral sim. = 849, #2

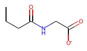

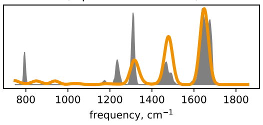

Struc. sim. = 0.50
N-Butyrylglycine

deprotonated HMDB0059723, spectral sim. = 848, #3

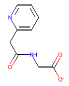

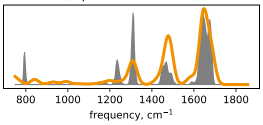

Struc. sim. = 0.42
Pyridylacetylglycine

deprotonated HMDB0001890, spectral sim. = 847, #4

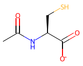

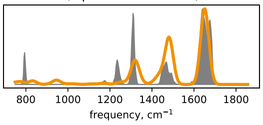

Struc. sim. = 0.49
Acetylcysteine

deprotonated HMDB0000821, spectral sim. = 847, #5

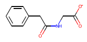

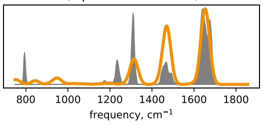

Struc. sim. = 0.58
Phenylacetylglycine

deprotonated HMDB0000532, spectral sim. = 845, #6

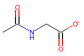

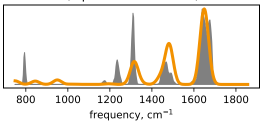

Struc. sim. = 0.54
Acetylglycine

deprotonated HMDB0000735, spectral sim. = 845, #7

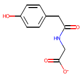

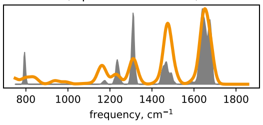

Struc. sim. = 0.53
Hydroxyphenylacetylglycine

deprotonated HMDB0000927, spectral sim. = 845, #8

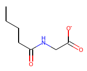

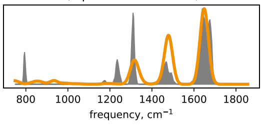

Struc. sim. = 0.49
Valerylglycine

deprotonated HMDB0000783, spectral sim. = 843, #9

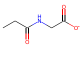

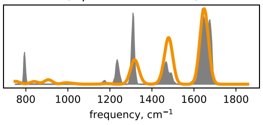

Struc. sim. = 0.51
Propionylglycine

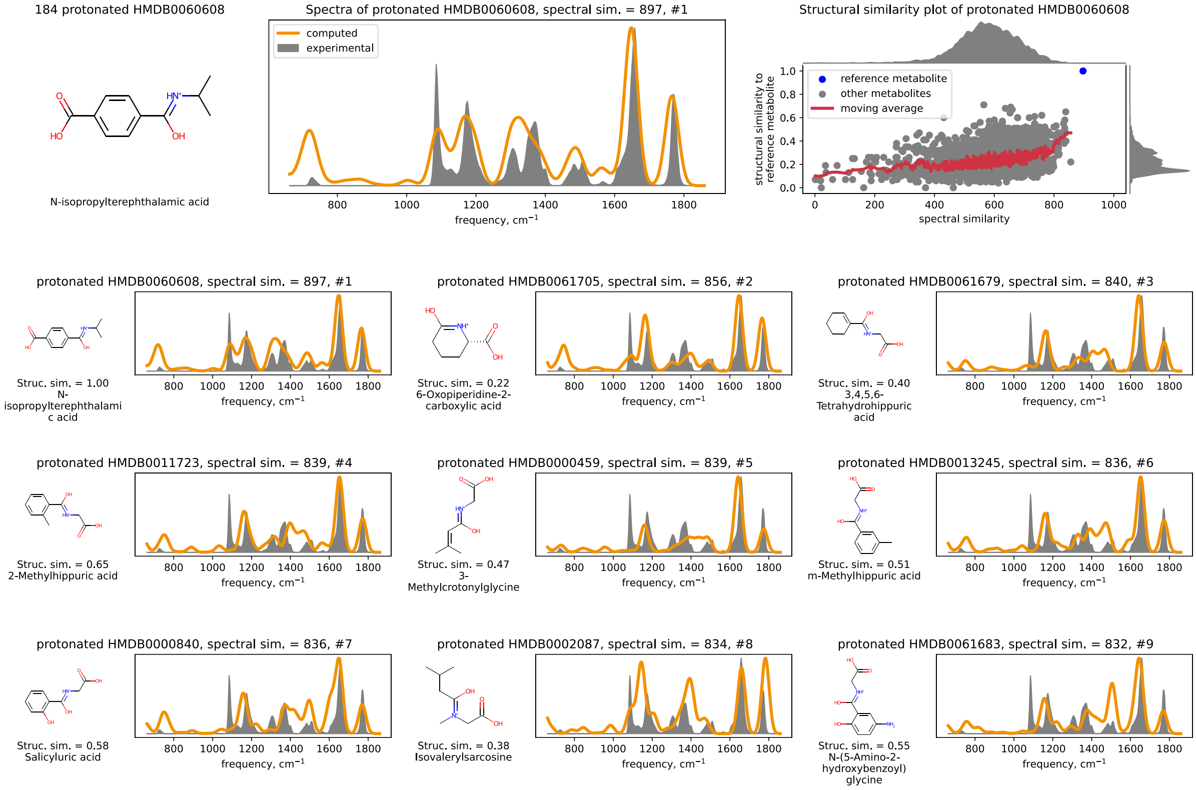

184 protonated HMDB0060608
N-isopropylterephthalamic acid
Spectra of protonated HMDB0060608, spectral sim. = 897, #1
computed
experimental
frequency, cm^{-1}
Structural similarity plot of protonated HMDB0060608
reference metabolite
other metabolites
moving average
structural similarity to reference metabolite
spectral similarity
protonated HMDB0060608, spectral sim. = 897, #1
Struc. sim. = 1.00
N-isopropylterephthalamic acid
protonated HMDB0061705, spectral sim. = 856, #2
Struc. sim. = 0.22
6-Oxopiperidine-2-carboxylic acid
protonated HMDB0061679, spectral sim. = 840, #3
Struc. sim. = 0.40
3,4,5,6-Tetrahydrohippuric acid
protonated HMDB0011723, spectral sim. = 839, #4
Struc. sim. = 0.65
2-Methylhippuric acid
protonated HMDB0000459, spectral sim. = 839, #5
Struc. sim. = 0.47
3-Methylcrotonylglycine
protonated HMDB0013245, spectral sim. = 836, #6
Struc. sim. = 0.51
m-Methylhippuric acid
protonated HMDB0000840, spectral sim. = 836, #7
Struc. sim. = 0.58
Salicyluric acid
protonated HMDB0002087, spectral sim. = 834, #8
Struc. sim. = 0.38
Isovalerylsarcosine
protonated HMDB0061683, spectral sim. = 832, #9
Struc. sim. = 0.55
N-(5-Amino-2-hydroxybenzoyl) glycine

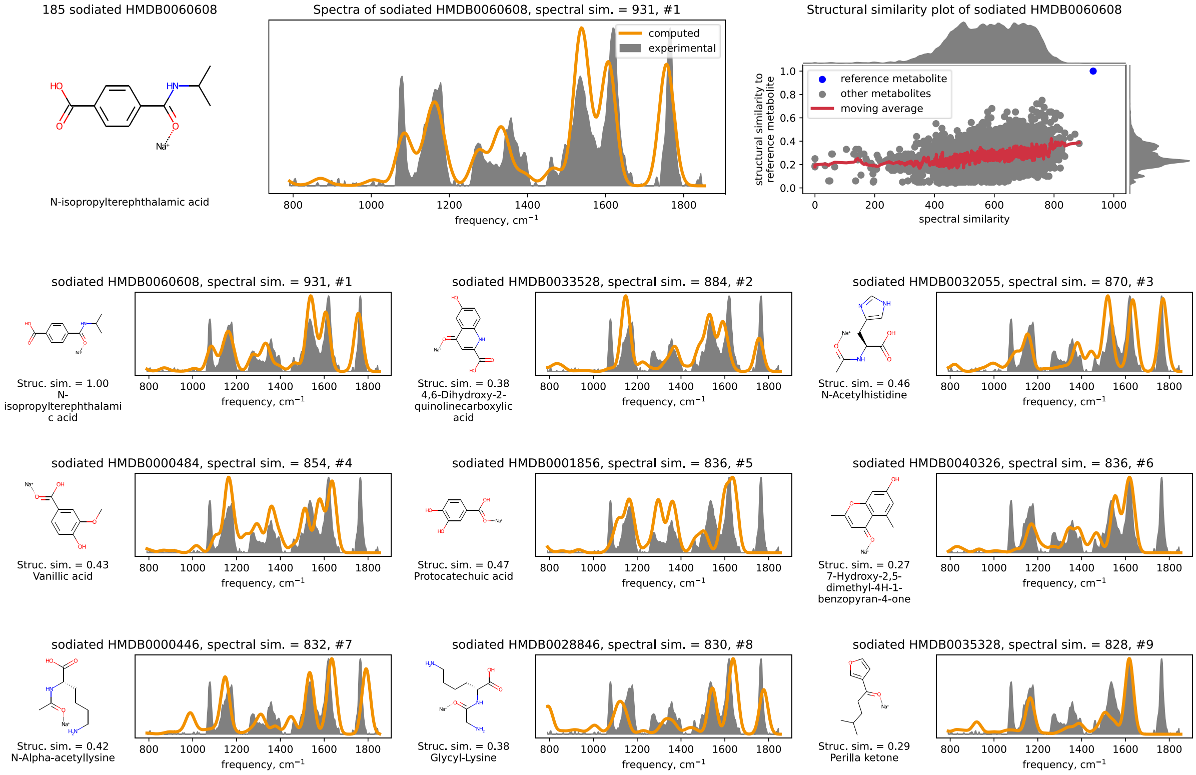

185 sodiated HMDB0060608
N-isopropylterephthalamic acid
Spectra of sodiated HMDB0060608, spectral sim. = 931, #1
computed
experimental
frequency, cm^{-1}
Structural similarity plot of sodiated HMDB0060608
reference metabolite
other metabolites
moving average
structural similarity to reference metabolite
spectral similarity
sodiated HMDB0060608, spectral sim. = 931, #1
Struc. sim. = 1.00
N-isopropylterephthalamic acid
sodiated HMDB0033528, spectral sim. = 884, #2
Struc. sim. = 0.38
4,6-Dihydroxy-2-quinolinecarboxylic acid
sodiated HMDB0032055, spectral sim. = 870, #3
Struc. sim. = 0.46
N-Acetylhistidine
sodiated HMDB0000484, spectral sim. = 854, #4
Struc. sim. = 0.43
Vanillic acid
sodiated HMDB0001856, spectral sim. = 836, #5
Struc. sim. = 0.47
Protocatechuic acid
sodiated HMDB0040326, spectral sim. = 836, #6
Struc. sim. = 0.27
7-Hydroxy-2,5-dimethyl-4H-1-benzopyran-4-one
sodiated HMDB0000446, spectral sim. = 832, #7
Struc. sim. = 0.42
N-Alpha-acetyllysine
sodiated HMDB0028846, spectral sim. = 830, #8
Struc. sim. = 0.38
Glycyl-Lysine
sodiated HMDB0035328, spectral sim. = 828, #9
Struc. sim. = 0.29
Perilla ketone

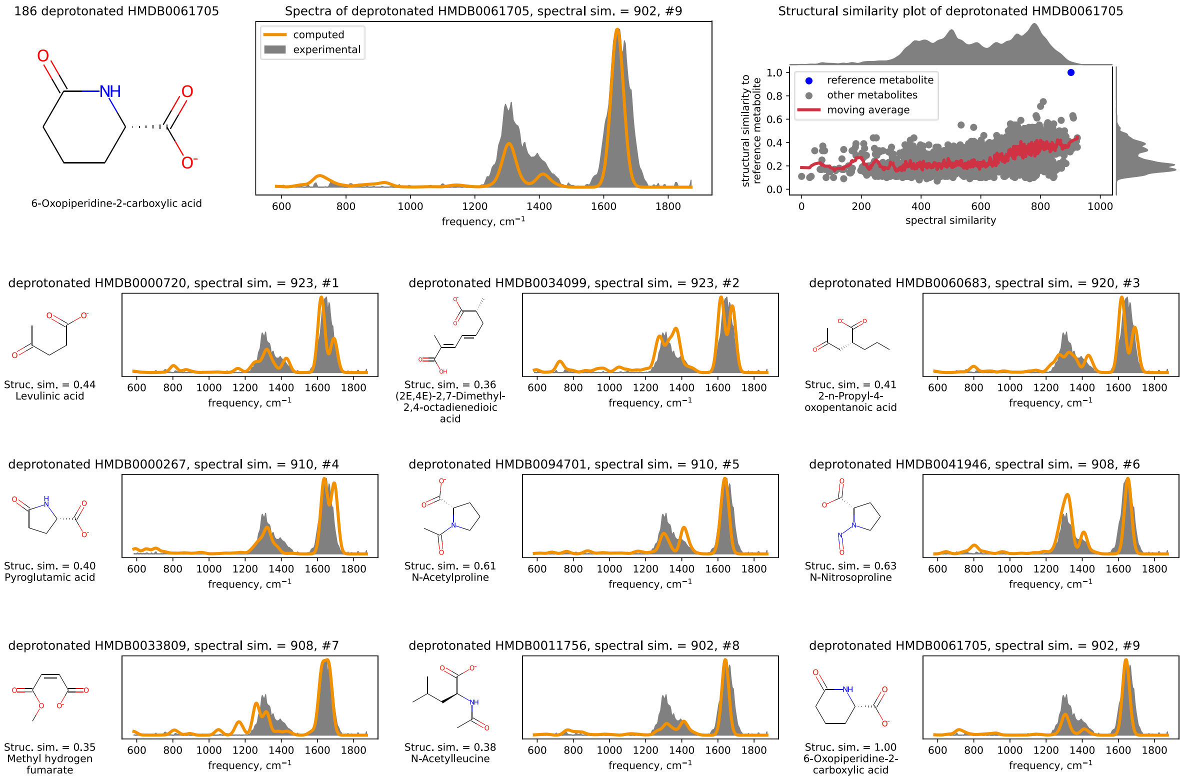
186 deprotonated HMDB0061705
6-Oxopiperidine-2-carboxylic acid
Spectra of deprotonated HMDB0061705, spectral sim. = 902, #9
computed
experimental
frequency, cm⁻¹
Structural similarity plot of deprotonated HMDB0061705
structural similarity to reference metabolite
reference metabolite
other metabolites
moving average
spectral similarity
deprotonated HMDB0000720, spectral sim. = 923, #1
Struc. sim. = 0.44
Levulinic acid
deprotonated HMDB0034099, spectral sim. = 923, #2
Struc. sim. = 0.36
(2E,4E)-2,7-Dimethyl-2,4-octadienedioic acid
deprotonated HMDB0060683, spectral sim. = 920, #3
Struc. sim. = 0.41
2-n-Propyl-4-oxopentanoic acid
deprotonated HMDB0000267, spectral sim. = 910, #4
Struc. sim. = 0.40
Pyroglutamic acid
deprotonated HMDB0094701, spectral sim. = 910, #5
Struc. sim. = 0.61
N-Acetylproline
deprotonated HMDB0041946, spectral sim. = 908, #6
Struc. sim. = 0.63
N-Nitrosoproline
deprotonated HMDB0033809, spectral sim. = 908, #7
Struc. sim. = 0.35
Methyl hydrogen fumarate
deprotonated HMDB0011756, spectral sim. = 902, #8
Struc. sim. = 0.38
N-Acetylleucine
deprotonated HMDB0061705, spectral sim. = 902, #9
Struc. sim. = 1.00
6-Oxopiperidine-2-carboxylic acid

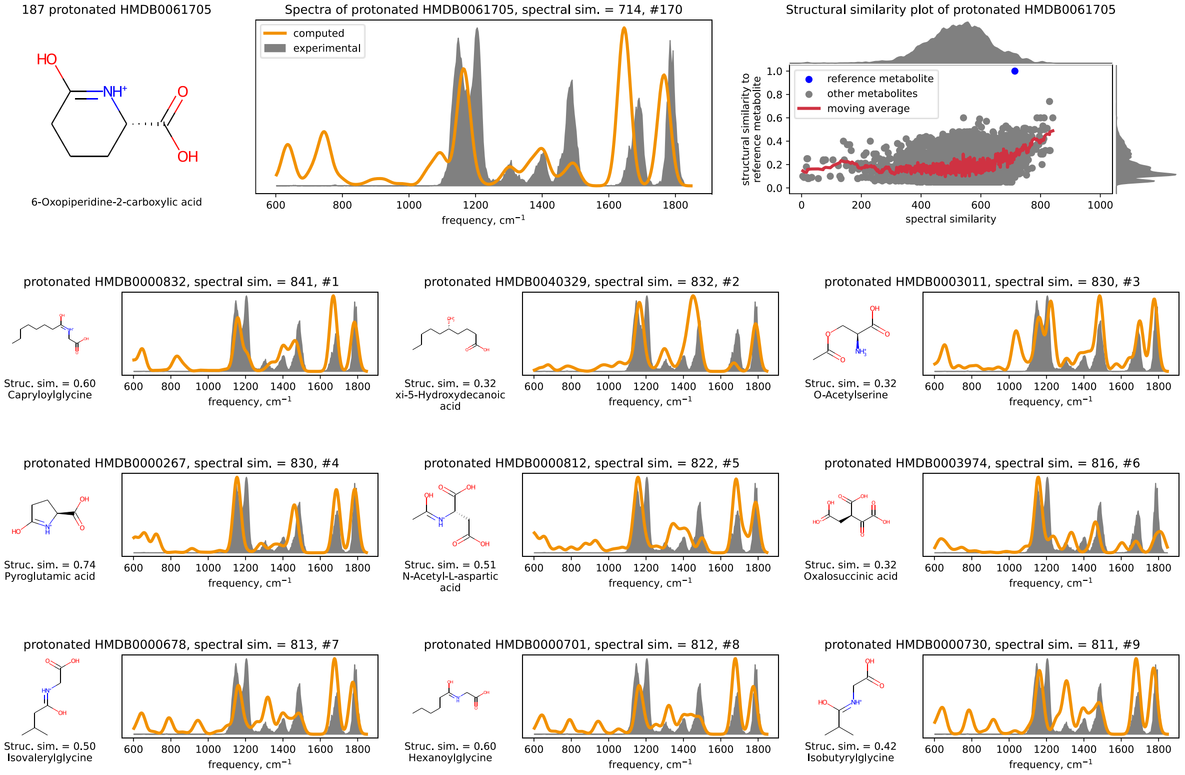
187 protonated HMDB0061705
6-Oxopiperidine-2-carboxylic acid
Spectra of protonated HMDB0061705, spectral sim. = 714, #170
computed
experimental
frequency, cm⁻¹
Structural similarity plot of protonated HMDB0061705
structural similarity to reference metabolite
reference metabolite
other metabolites
moving average
spectral similarity
protonated HMDB0000832, spectral sim. = 841, #1
Struc. sim. = 0.60
Capryloylglycine
protonated HMDB0040329, spectral sim. = 832, #2
Struc. sim. = 0.32
xi-5-Hydroxydecanoic acid
protonated HMDB0003011, spectral sim. = 830, #3
Struc. sim. = 0.32
O-Acetylserine
protonated HMDB0000267, spectral sim. = 830, #4
Struc. sim. = 0.74
Pyroglutamic acid
protonated HMDB0000812, spectral sim. = 822, #5
Struc. sim. = 0.51
N-Acetyl-L-aspartic acid
protonated HMDB0003974, spectral sim. = 816, #6
Struc. sim. = 0.32
Oxalosuccinic acid
protonated HMDB0000678, spectral sim. = 813, #7
Struc. sim. = 0.50
Isovalerylglycine
protonated HMDB0000701, spectral sim. = 812, #8
Struc. sim. = 0.60
Hexanoylglycine
protonated HMDB0000730, spectral sim. = 811, #9
Struc. sim. = 0.42
Isobutyrylglycine

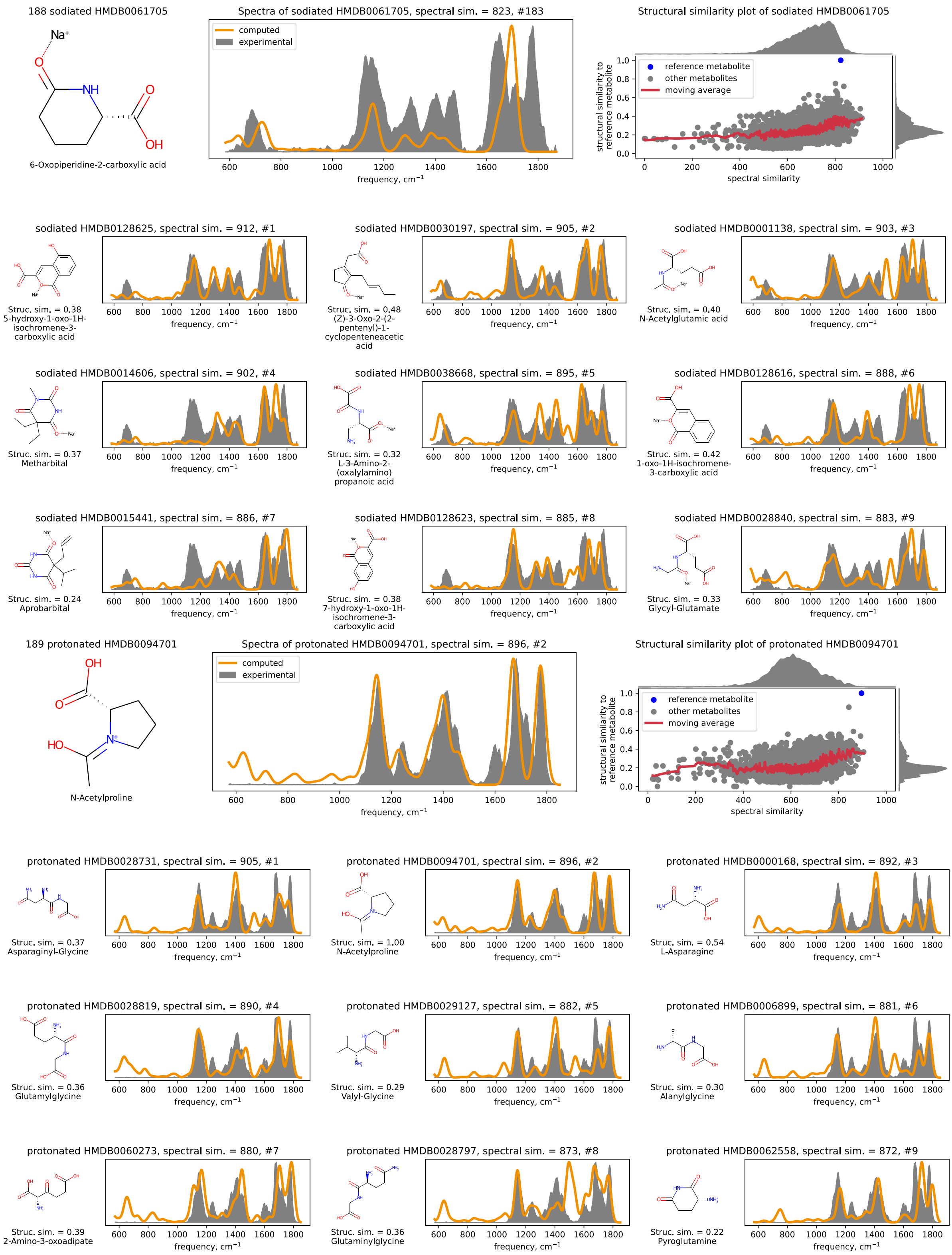

188 sodiated HMDB0061705
Na⁺
NH
O
OH
6-Oxopiperidine-2-carboxylic acid
Spectra of sodiated HMDB0061705, spectral sim. = 823, #183
computed
experimental
frequency, cm⁻¹
Structural similarity plot of sodiated HMDB0061705
reference metabolite
other metabolites
moving average
structural similarity to reference metabolite
spectral similarity
sodiated HMDB0128625, spectral sim. = 912, #1
Struc. sim. = 0.38
5-hydroxy-1-oxo-1H-isochromene-3-carboxylic acid
sodiated HMDB0030197, spectral sim. = 905, #2
Struc. sim. = 0.48
(Z)-3-Oxo-2-(2-pentenyl)-1-cyclopenteneacetic acid
sodiated HMDB0001138, spectral sim. = 903, #3
Struc. sim. = 0.40
N-Acetylglutamic acid
sodiated HMDB0014606, spectral sim. = 902, #4
Struc. sim. = 0.37
Metharbital
sodiated HMDB0038668, spectral sim. = 895, #5
Struc. sim. = 0.32
L-3-Amino-2-(oxalylamino)propanoic acid
sodiated HMDB0128616, spectral sim. = 888, #6
Struc. sim. = 0.42
1-oxo-1H-isochromene-3-carboxylic acid
sodiated HMDB0015441, spectral sim. = 886, #7
Struc. sim. = 0.24
Aprobarbital
sodiated HMDB0128623, spectral sim. = 885, #8
Struc. sim. = 0.38
7-hydroxy-1-oxo-1H-isochromene-3-carboxylic acid
sodiated HMDB0028840, spectral sim. = 883, #9
Struc. sim. = 0.33
Glycyl-Glutamate
189 protonated HMDB0094701
OH
O
HO
N⁺
N-Acetylproline
Spectra of protonated HMDB0094701, spectral sim. = 896, #2
computed
experimental
frequency, cm⁻¹
Structural similarity plot of protonated HMDB0094701
reference metabolite
other metabolites
moving average
structural similarity to reference metabolite
spectral similarity
protonated HMDB0028731, spectral sim. = 905, #1
Struc. sim. = 0.37
Asparaginyl-Glycine
protonated HMDB0094701, spectral sim. = 896, #2
Struc. sim. = 1.00
N-Acetylproline
protonated HMDB0000168, spectral sim. = 892, #3
Struc. sim. = 0.54
L-Asparagine
protonated HMDB0028819, spectral sim. = 890, #4
Struc. sim. = 0.36
Glutamylglycine
protonated HMDB0029127, spectral sim. = 882, #5
Struc. sim. = 0.29
Valyl-Glycine
protonated HMDB0006899, spectral sim. = 881, #6
Struc. sim. = 0.30
Alanylglycine
protonated HMDB0060273, spectral sim. = 880, #7
Struc. sim. = 0.39
2-Amino-3-oxoadipate
protonated HMDB0028797, spectral sim. = 873, #8
Struc. sim. = 0.36
Glutaminylglycine
protonated HMDB0062558, spectral sim. = 872, #9
Struc. sim. = 0.22
Pyroglutamine
