# Supplementary material for: An In Silico Infrared Spectral Library of Molecular Ions for Metabolite Identification
Source: Anal Chem. 2023 Jun 1;95(23):8998–9005. doi: 10.1021/acs.analchem.3c01078 (PMC10267894; doi:10.1021/acs.analchem.3c01078)
Supplement: Supplementary file 5 — ac3c01078_si_005.pdf [file ac3c01078_si_005.pdf]

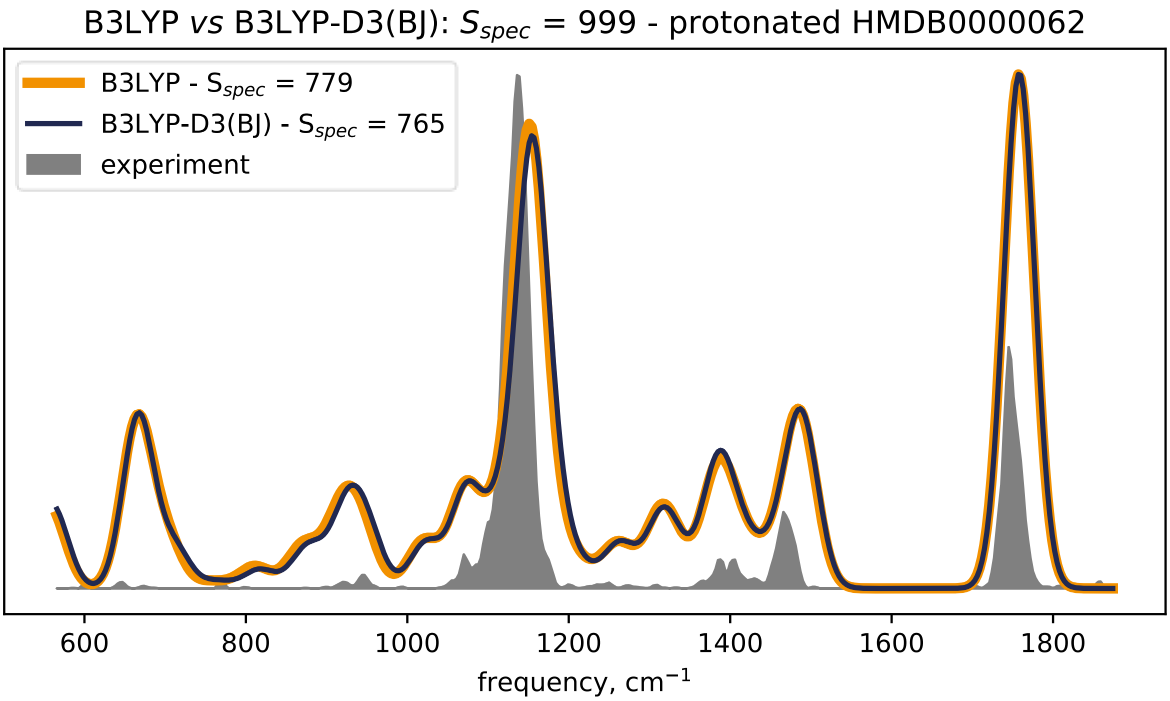
B3LYP vs B3LYP-D3(BJ): S_{spec} = 999 - protonated HMDB0000062
B3LYP - S_{spec} = 779
B3LYP-D3(BJ) - S_{spec} = 765
experiment
600
800
1000
1200
1400
1600
1800
frequency, cm^{-1}

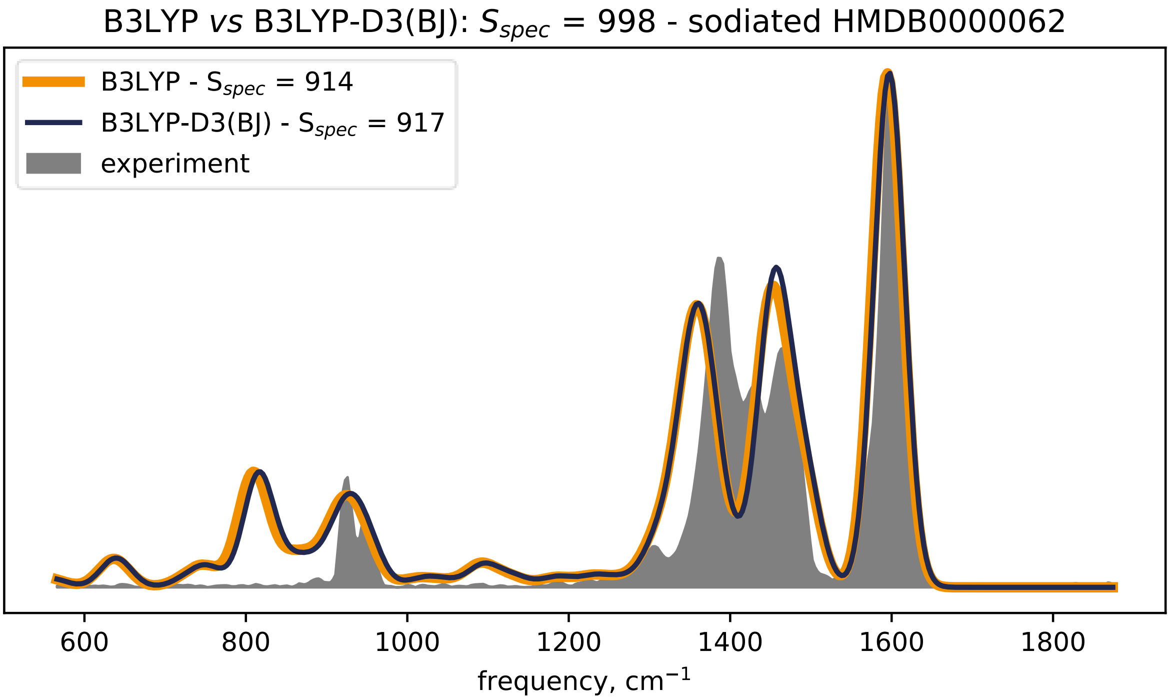
B3LYP vs B3LYP-D3(BJ): S_{spec} = 998 - sodiated HMDB0000062
B3LYP - S_{spec} = 914
B3LYP-D3(BJ) - S_{spec} = 917
experiment
600
800
1000
1200
1400
1600
1800
frequency, cm^{-1}

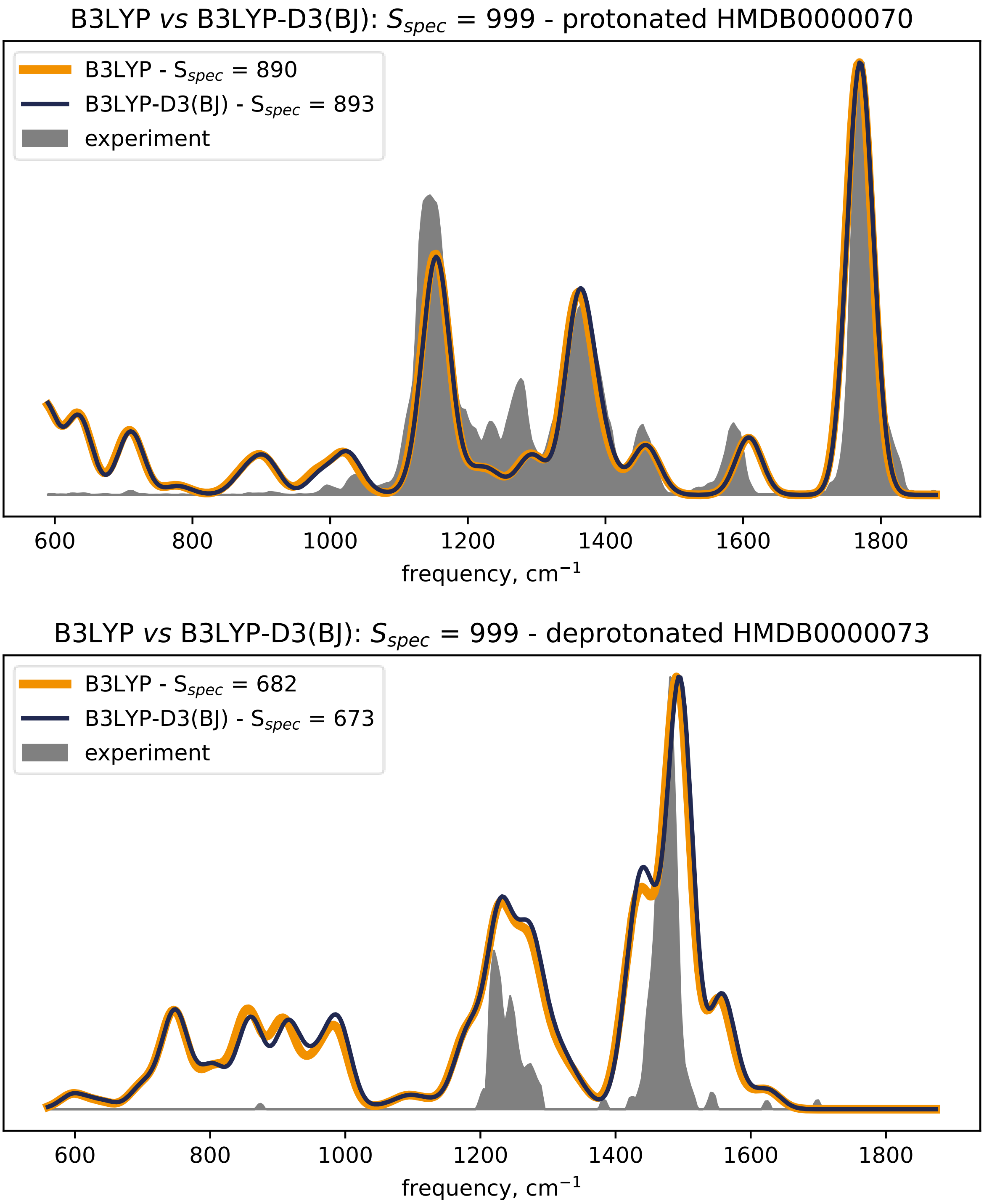
B3LYP vs B3LYP-D3(BJ): S_{spec} = 999 - protonated HMDB0000070
B3LYP - S_{spec} = 890
B3LYP-D3(BJ) - S_{spec} = 893
experiment
600
800
1000
1200
1400
1600
1800
frequency, cm^{-1}
B3LYP vs B3LYP-D3(BJ): S_{spec} = 999 - deprotonated HMDB0000073
B3LYP - S_{spec} = 682
B3LYP-D3(BJ) - S_{spec} = 673
experiment
600
800
1000
1200
1400
1600
1800
frequency, cm^{-1}

B3LYP *vs* B3LYP-D3(BJ): S_{spec} = 999 - protonated HMDB0000073

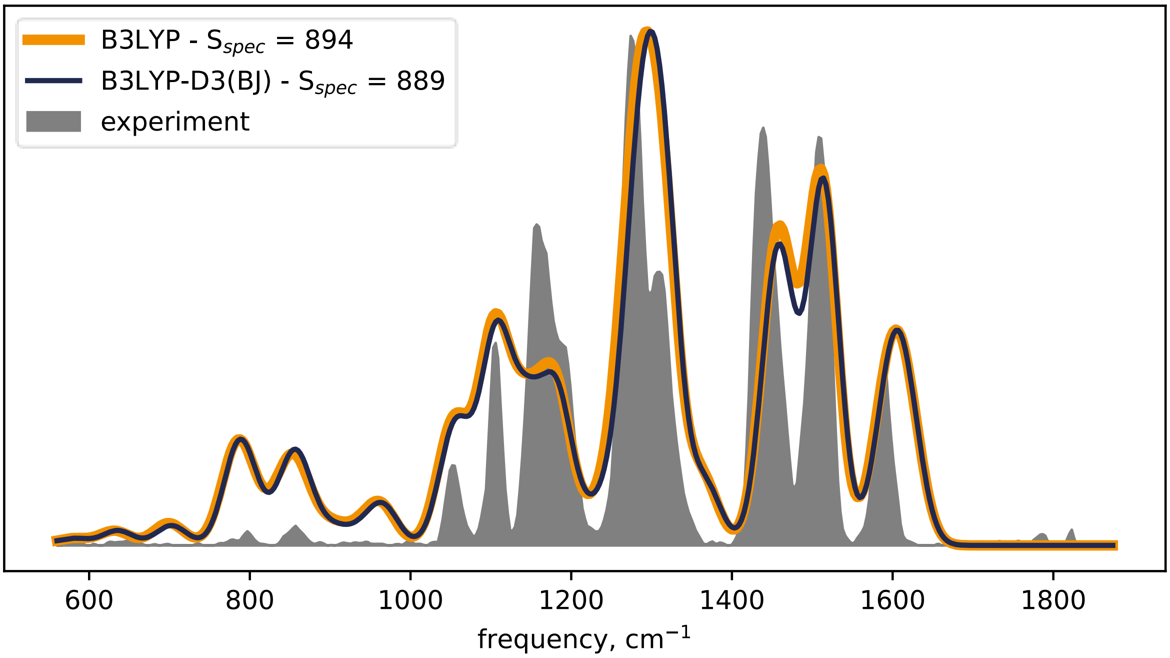

B3LYP *vs* B3LYP-D3(BJ): S_{spec} = 997 - deprotonated HMDB0000107

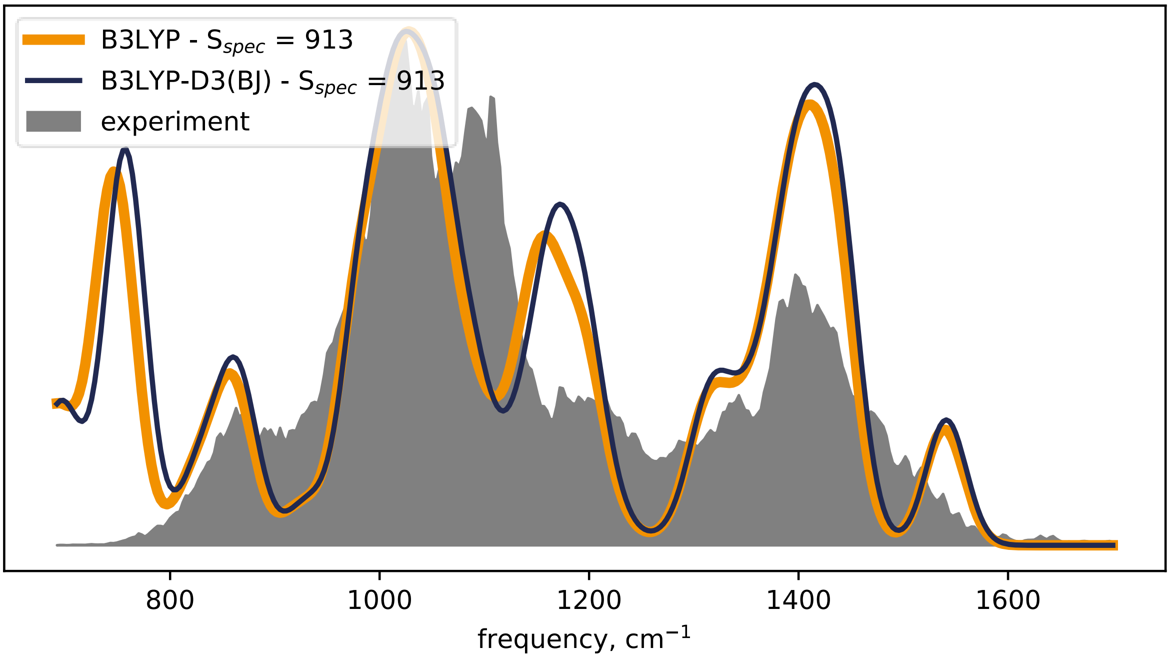

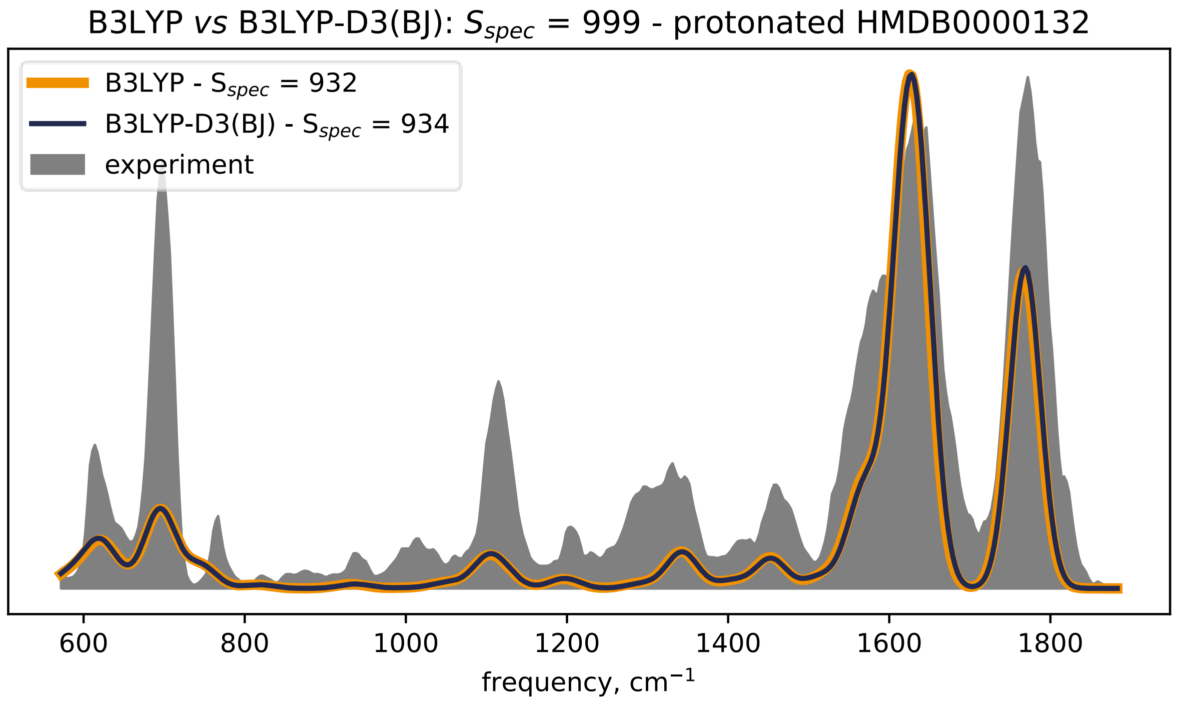
B3LYP vs B3LYP-D3(BJ): S_{spec} = 999 - protonated HMDB0000132
B3LYP - S_{spec} = 932
B3LYP-D3(BJ) - S_{spec} = 934
experiment
600
800
1000
1200
1400
1600
1800
frequency, cm^{-1}

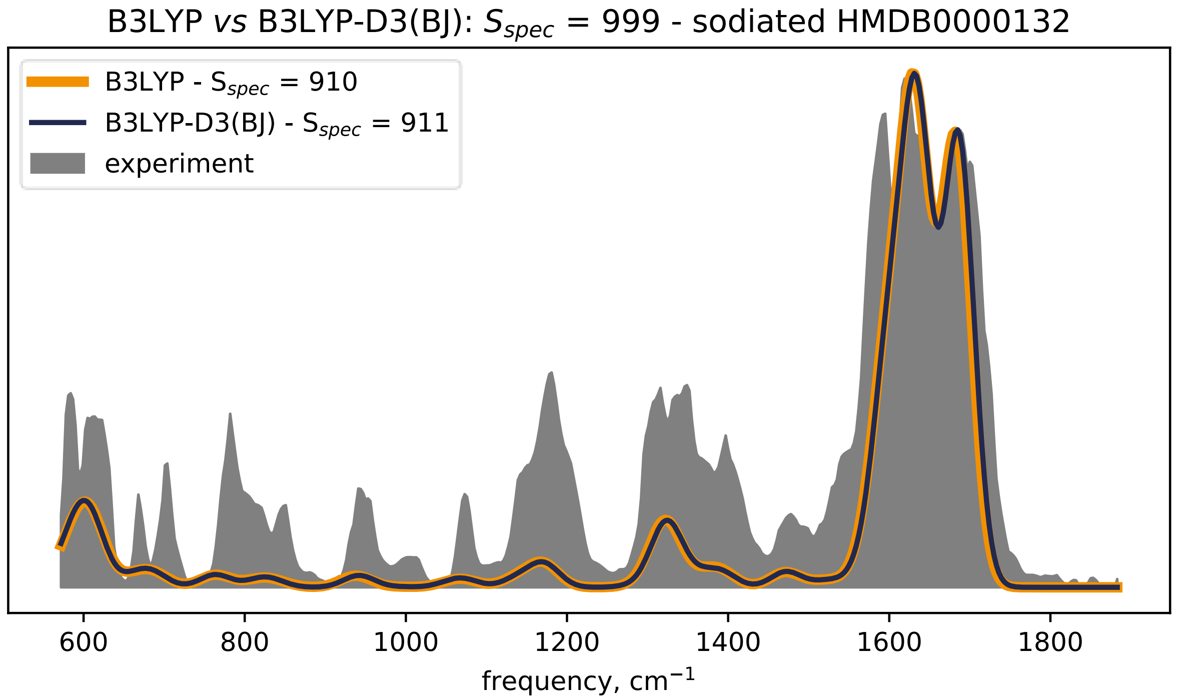
B3LYP vs B3LYP-D3(BJ): S_{spec} = 999 - sodiated HMDB0000132
B3LYP - S_{spec} = 910
B3LYP-D3(BJ) - S_{spec} = 911
experiment
600
800
1000
1200
1400
1600
1800
frequency, cm^{-1}

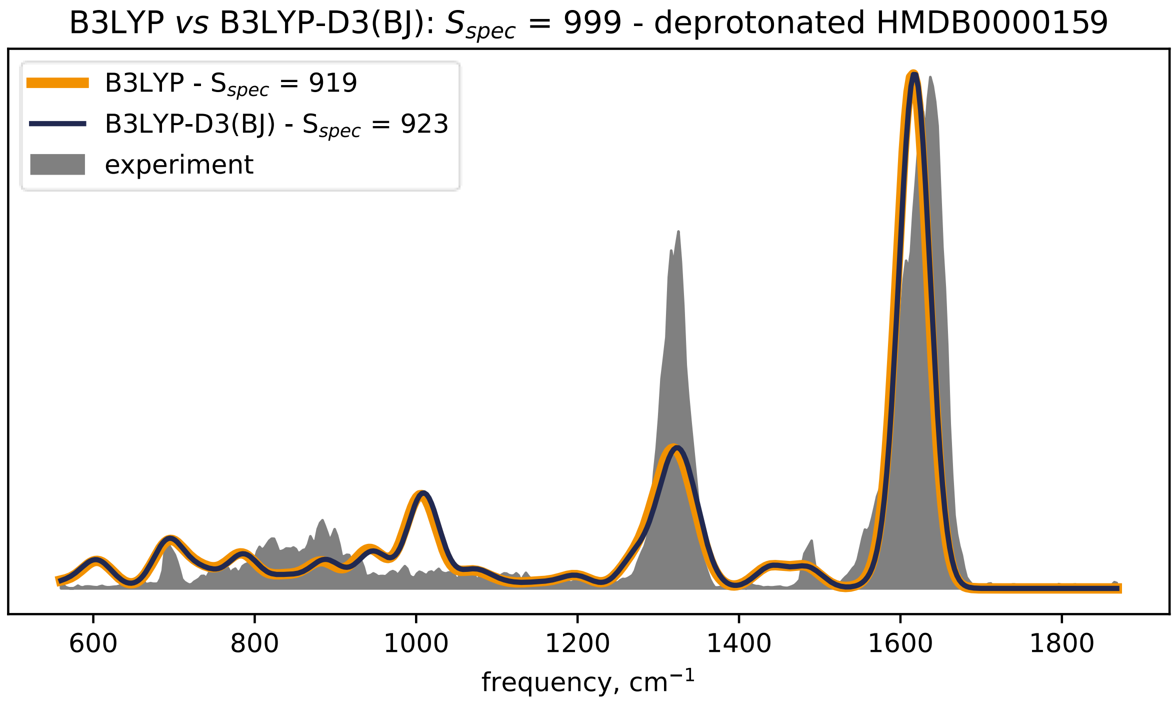
B3LYP vs B3LYP-D3(BJ): S_{spec} = 999 - deprotonated HMDB0000159
B3LYP - S_{spec} = 919
B3LYP-D3(BJ) - S_{spec} = 923
experiment
600
800
1000
1200
1400
1600
1800
frequency, cm^{-1}

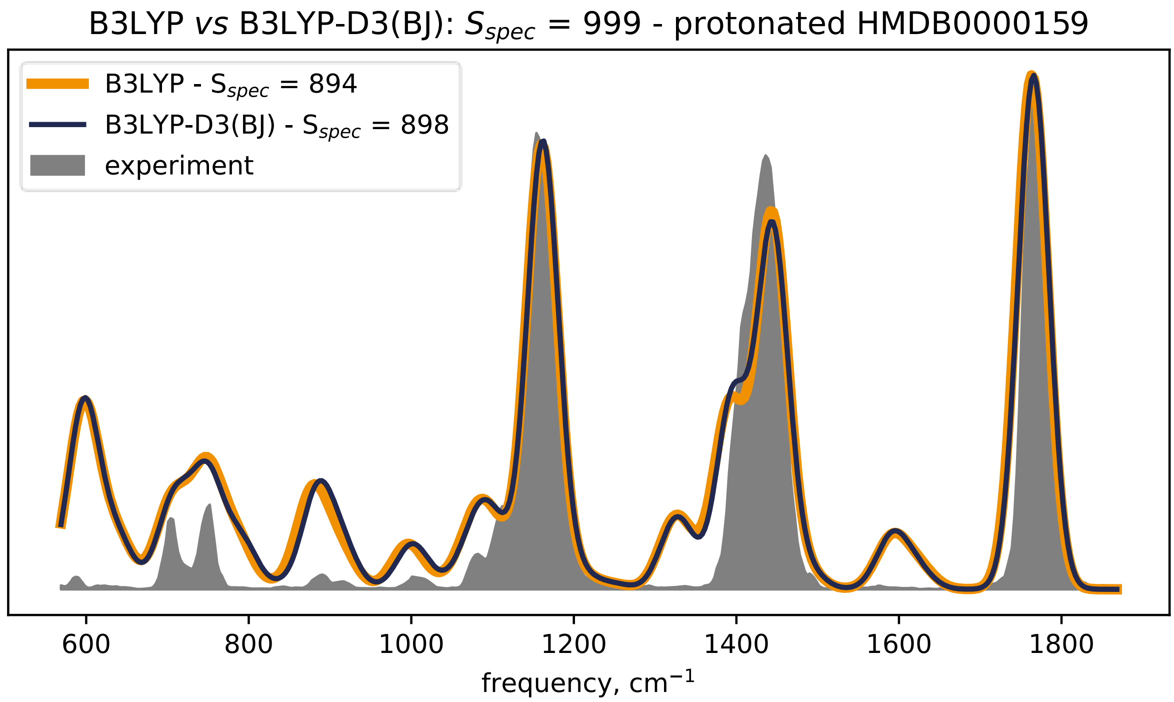
B3LYP vs B3LYP-D3(BJ): S_{spec} = 999 - protonated HMDB0000159
B3LYP - S_{spec} = 894
B3LYP-D3(BJ) - S_{spec} = 898
experiment
600
800
1000
1200
1400
1600
1800
frequency, cm^{-1}

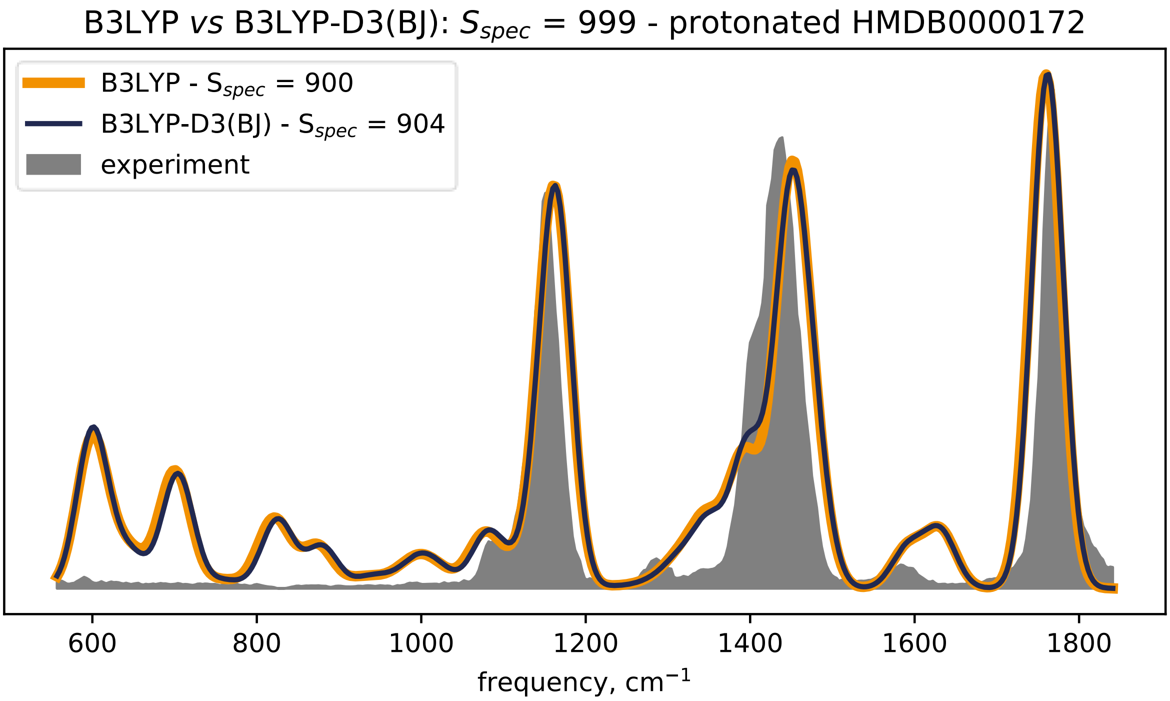
B3LYP vs B3LYP-D3(BJ): S_{spec} = 999 - protonated HMDB0000172
B3LYP - S_{spec} = 900
B3LYP-D3(BJ) - S_{spec} = 904
experiment
600
800
1000
1200
1400
1600
1800
frequency, cm^{-1}

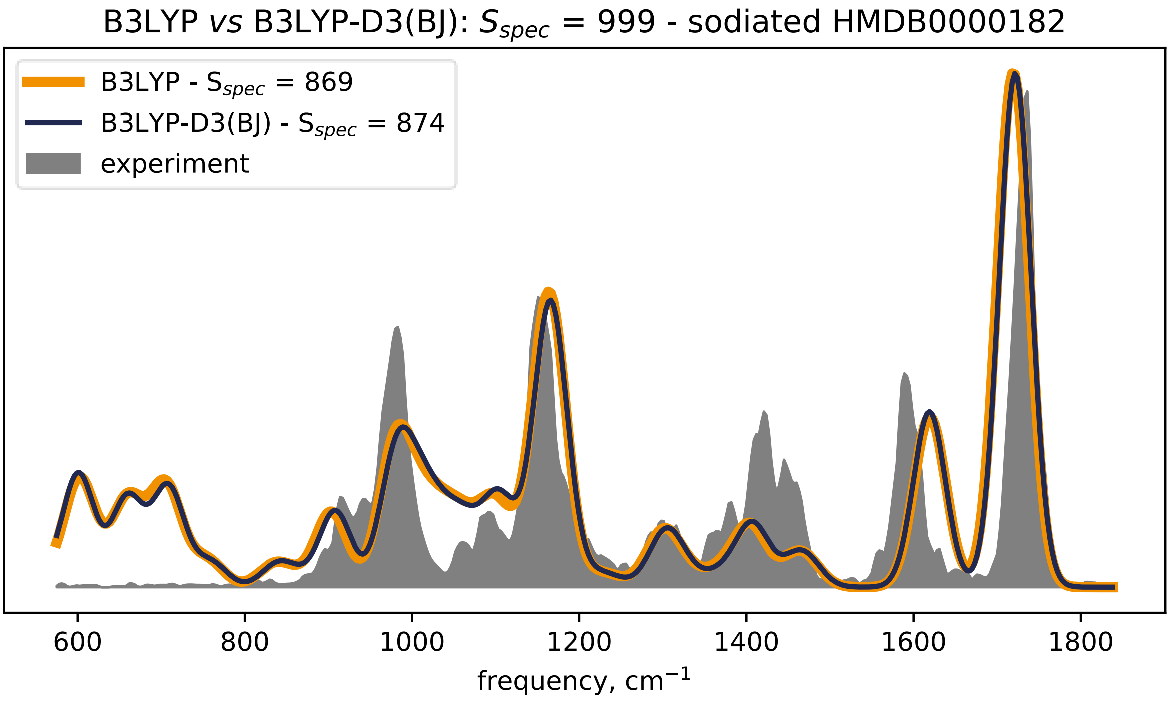
B3LYP vs B3LYP-D3(BJ): S_{spec} = 999 - sodiated HMDB0000182
B3LYP - S_{spec} = 869
B3LYP-D3(BJ) - S_{spec} = 874
experiment
600
800
1000
1200
1400
1600
1800
frequency, cm^{-1}

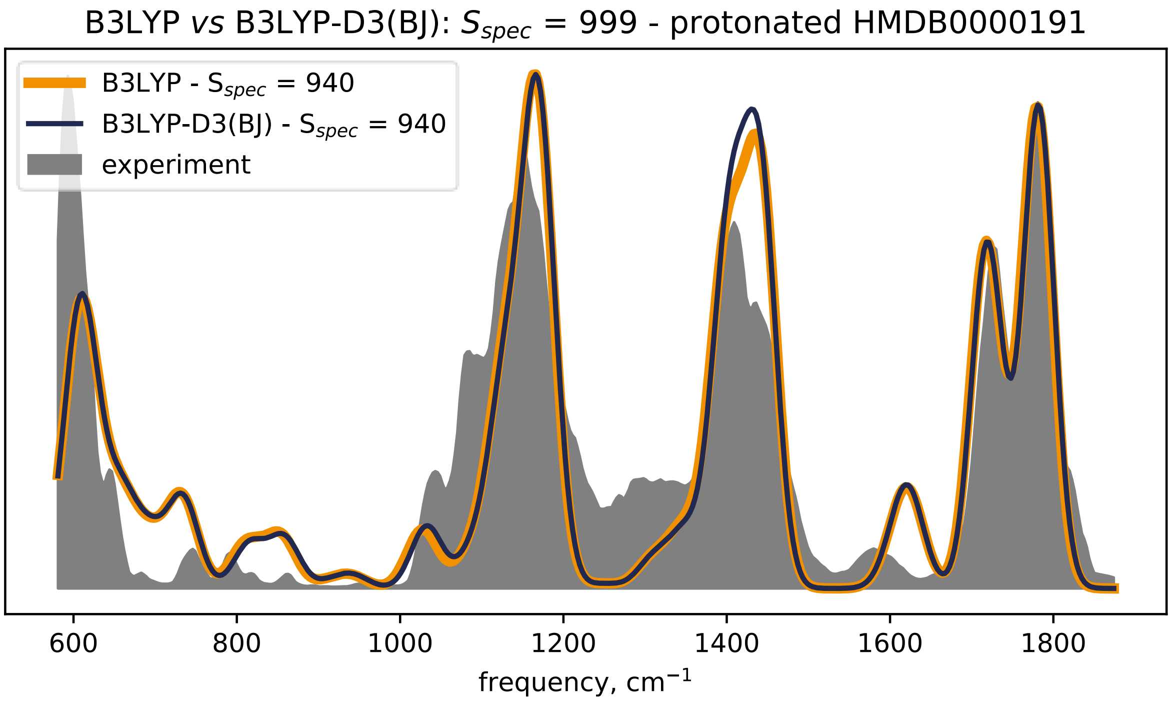

B3LYP vs B3LYP-D3(BJ): S_{spec} = 999 - protonated HMDB0000191
B3LYP - S_{spec} = 940
B3LYP-D3(BJ) - S_{spec} = 940
experiment
600
800
1000
1200
1400
1600
1800
frequency, cm^{-1}

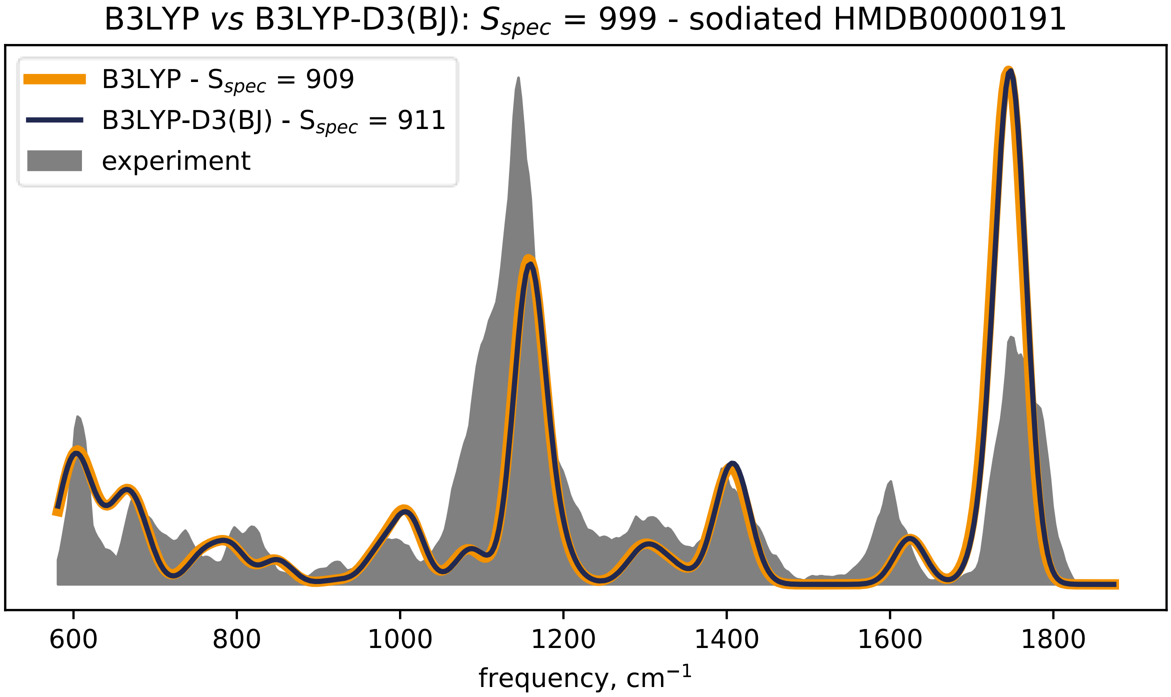

B3LYP vs B3LYP-D3(BJ): S_{spec} = 999 - sodiated HMDB0000191
B3LYP - S_{spec} = 909
B3LYP-D3(BJ) - S_{spec} = 911
experiment
600
800
1000
1200
1400
1600
1800
frequency, cm^{-1}

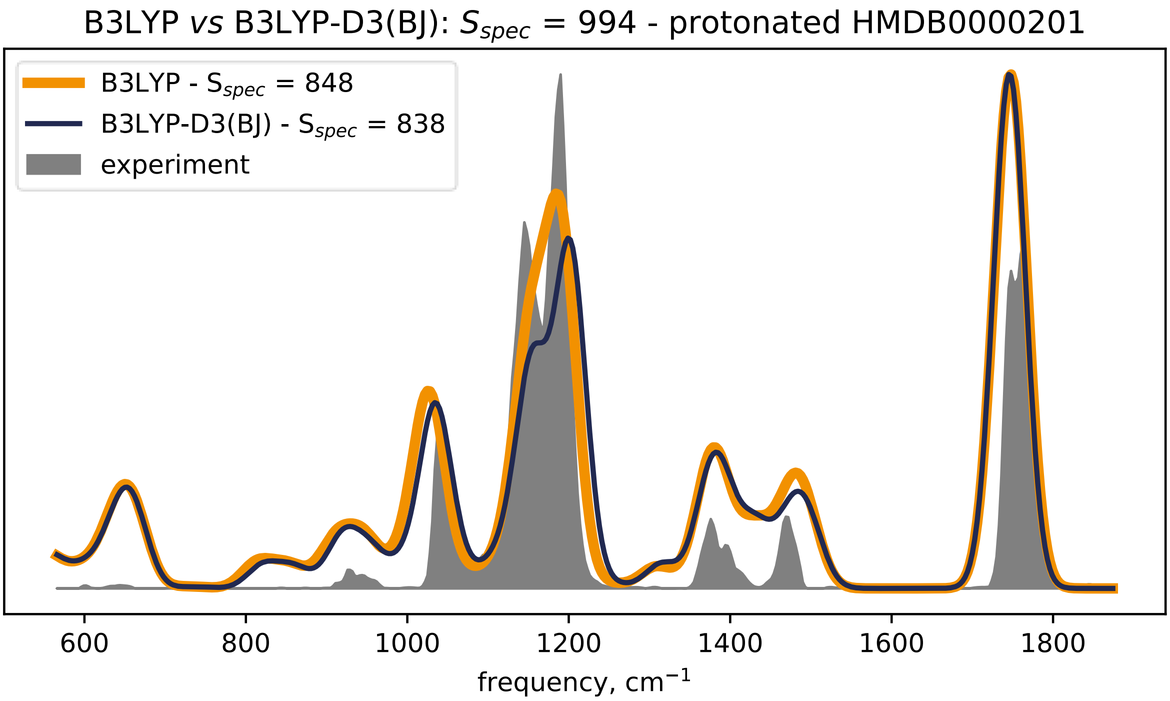

B3LYP vs B3LYP-D3(BJ): S_{spec} = 994 - protonated HMDB0000201
B3LYP - S_{spec} = 848
B3LYP-D3(BJ) - S_{spec} = 838
experiment
600
800
1000
1200
1400
1600
1800
frequency, cm^{-1}

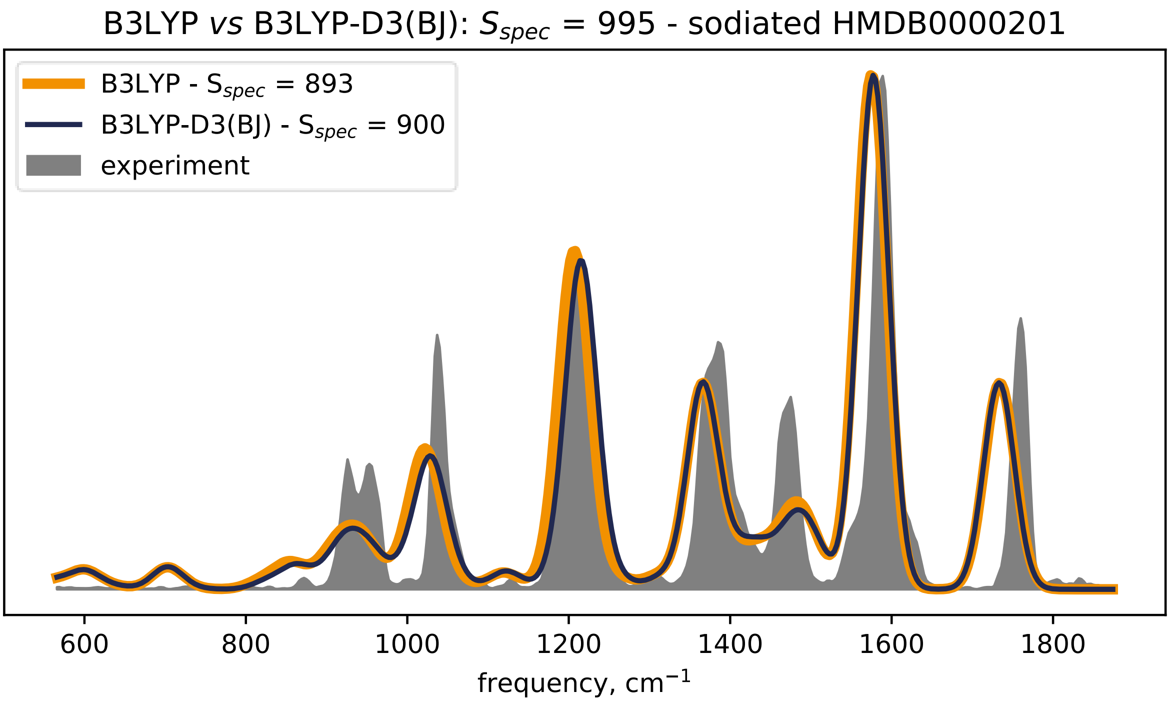

B3LYP vs B3LYP-D3(BJ): S_{spec} = 995 - sodiated HMDB0000201
B3LYP - S_{spec} = 893
B3LYP-D3(BJ) - S_{spec} = 900
experiment
600
800
1000
1200
1400
1600
1800
frequency, cm^{-1}

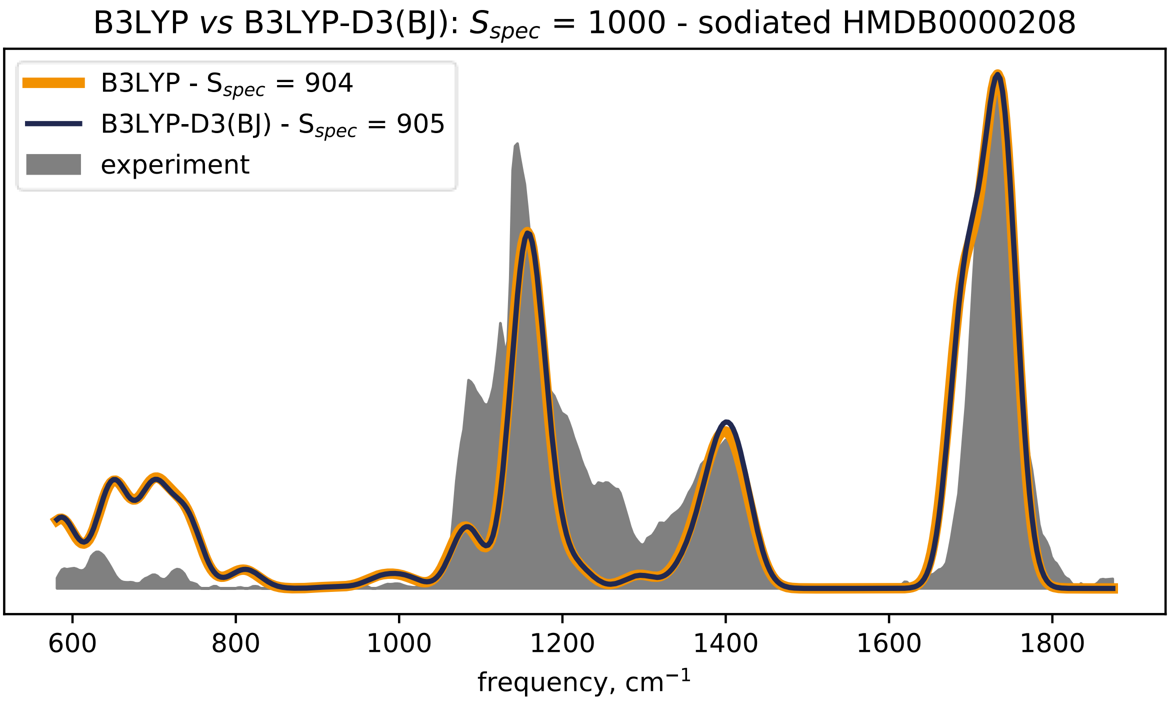
B3LYP vs B3LYP-D3(BJ): S_{spec} = 1000 - sodiated HMDB0000208
B3LYP - S_{spec} = 904
B3LYP-D3(BJ) - S_{spec} = 905
experiment
600
800
1000
1200
1400
1600
1800
frequency, cm^{-1}

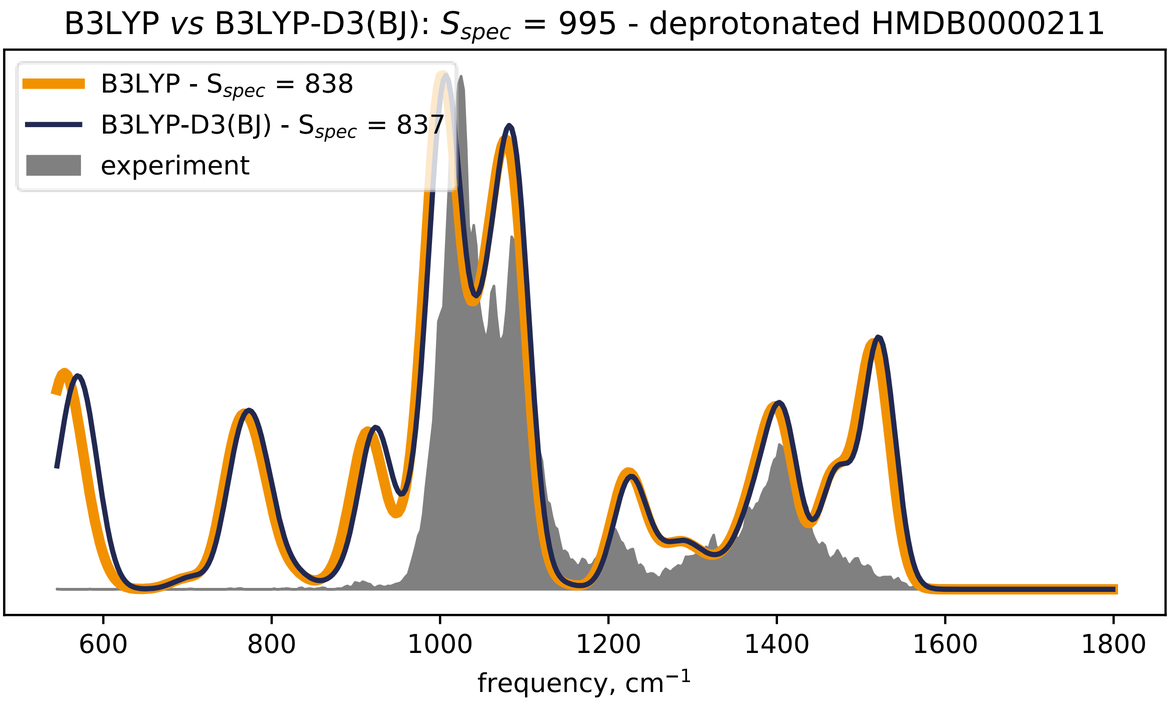
B3LYP vs B3LYP-D3(BJ): S_{spec} = 995 - deprotonated HMDB0000211
B3LYP - S_{spec} = 838
B3LYP-D3(BJ) - S_{spec} = 837
experiment
600
800
1000
1200
1400
1600
1800
frequency, cm^{-1}

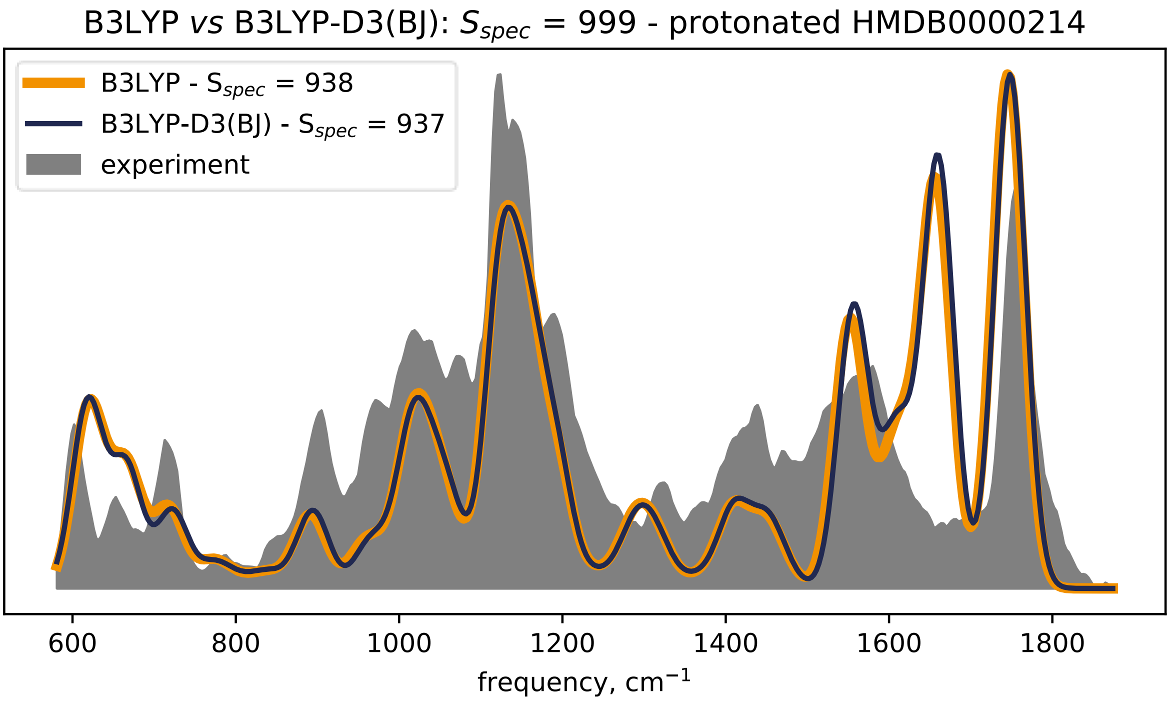

B3LYP vs B3LYP-D3(BJ): S_{spec} = 999 - protonated HMDB0000214
B3LYP - S_{spec} = 938
B3LYP-D3(BJ) - S_{spec} = 937
experiment
600
800
1000
1200
1400
1600
1800
frequency, cm^{-1}

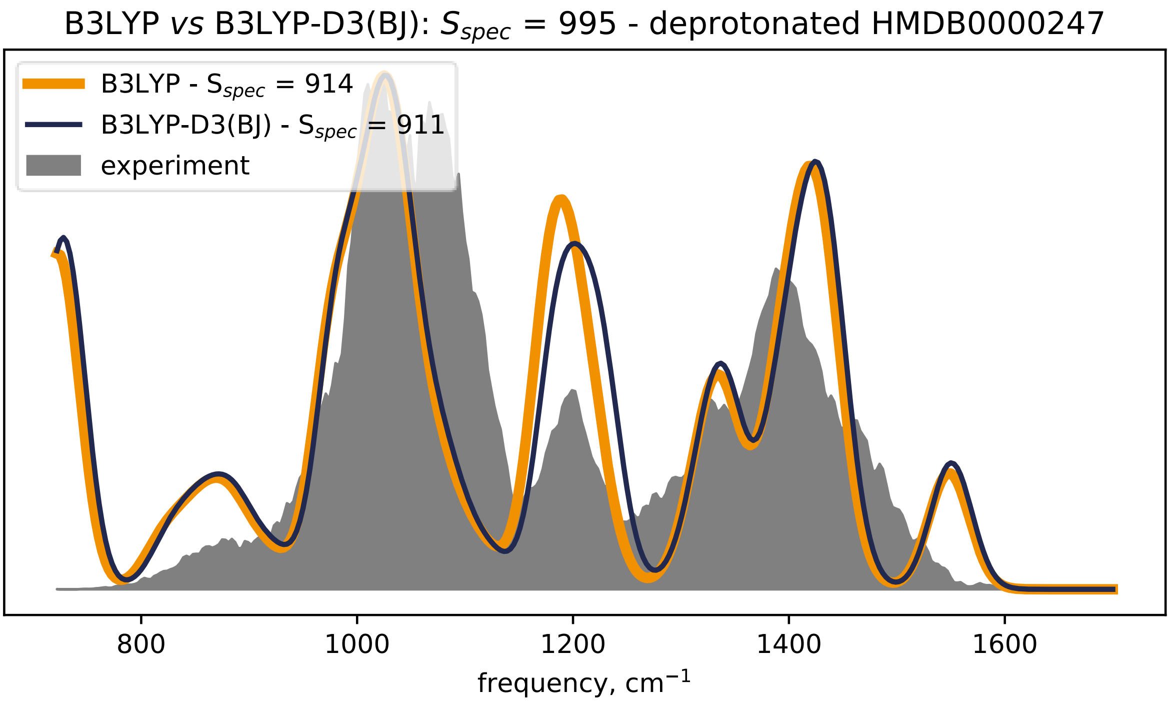

B3LYP vs B3LYP-D3(BJ): S_{spec} = 995 - deprotonated HMDB0000247
B3LYP - S_{spec} = 914
B3LYP-D3(BJ) - S_{spec} = 911
experiment
800
1000
1200
1400
1600
frequency, cm^{-1}

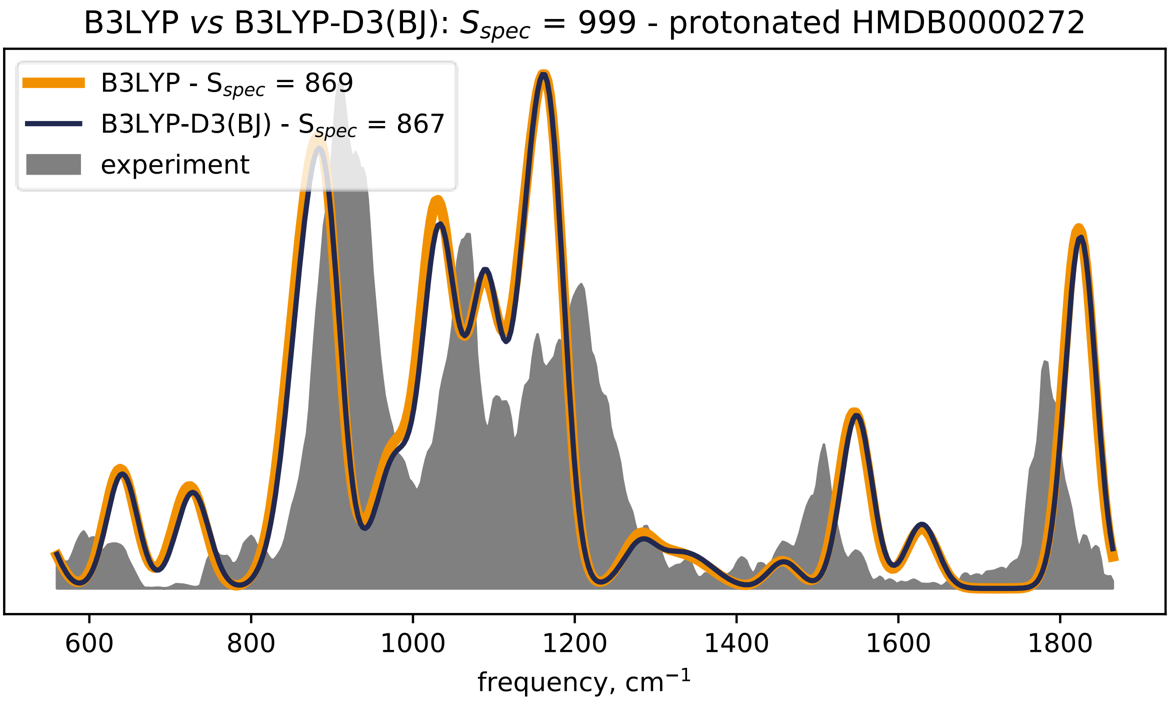
B3LYP vs B3LYP-D3(BJ): S_{spec} = 999 - protonated HMDB0000272
B3LYP - S_{spec} = 869
B3LYP-D3(BJ) - S_{spec} = 867
experiment
600
800
1000
1200
1400
1600
1800
frequency, cm^{-1}

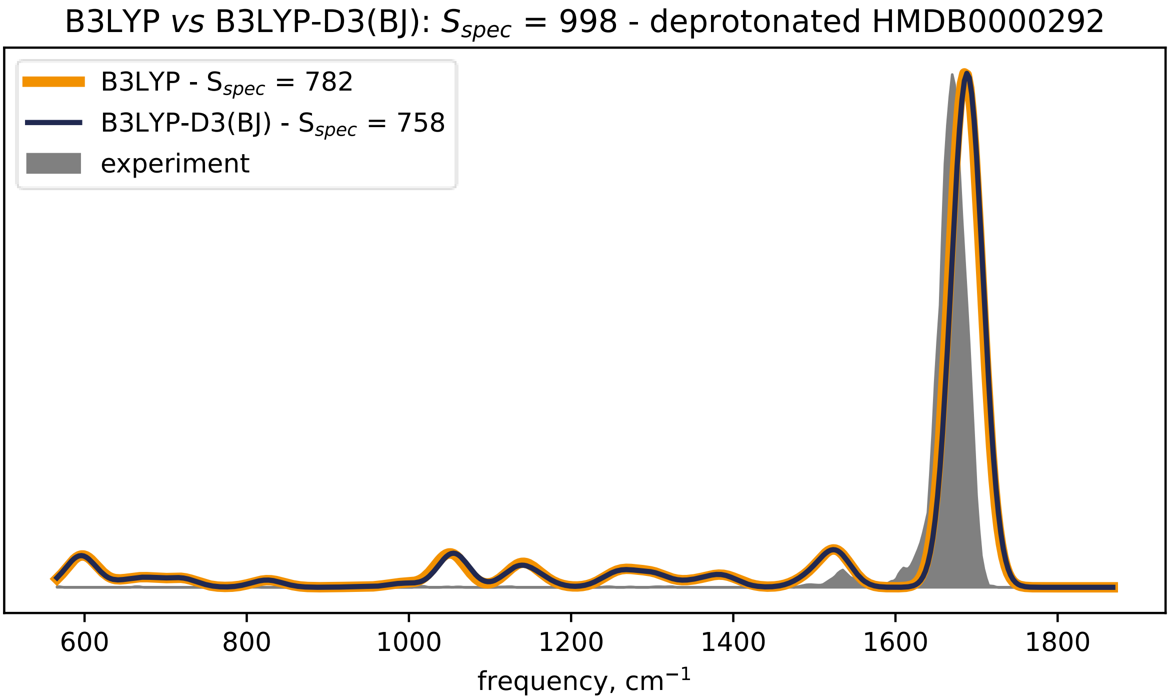
B3LYP vs B3LYP-D3(BJ): S_{spec} = 998 - deprotonated HMDB0000292
B3LYP - S_{spec} = 782
B3LYP-D3(BJ) - S_{spec} = 758
experiment
600
800
1000
1200
1400
1600
1800
frequency, cm^{-1}

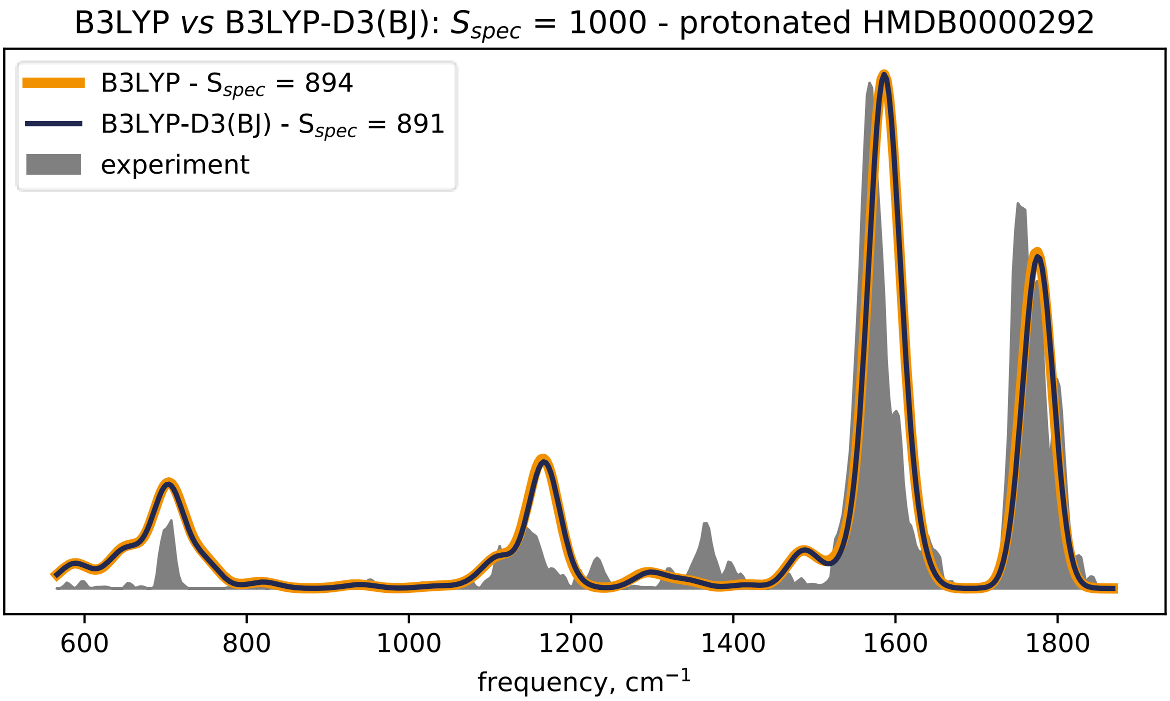
B3LYP vs B3LYP-D3(BJ): S_{spec} = 1000 - protonated HMDB0000292
B3LYP - S_{spec} = 894
B3LYP-D3(BJ) - S_{spec} = 891
experiment
600
800
1000
1200
1400
1600
1800
frequency, cm^{-1}

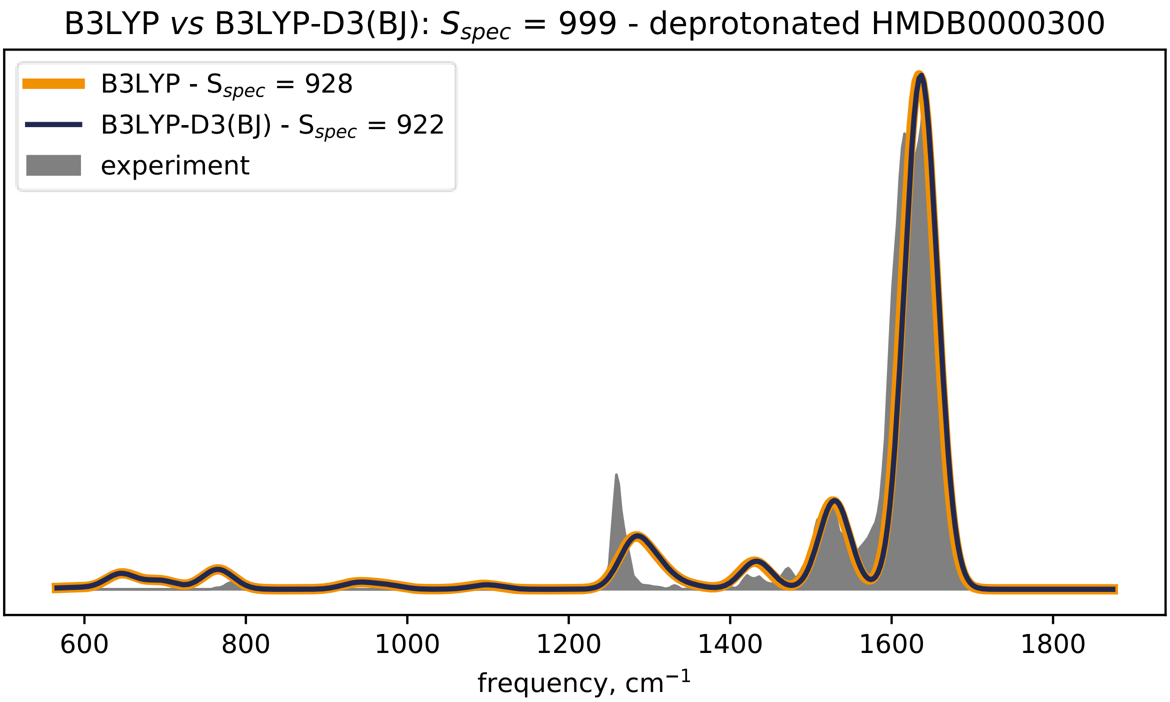
B3LYP vs B3LYP-D3(BJ): S_{spec} = 999 - deprotonated HMDB0000300
B3LYP - S_{spec} = 928
B3LYP-D3(BJ) - S_{spec} = 922
experiment
600
800
1000
1200
1400
1600
1800
frequency, cm^{-1}

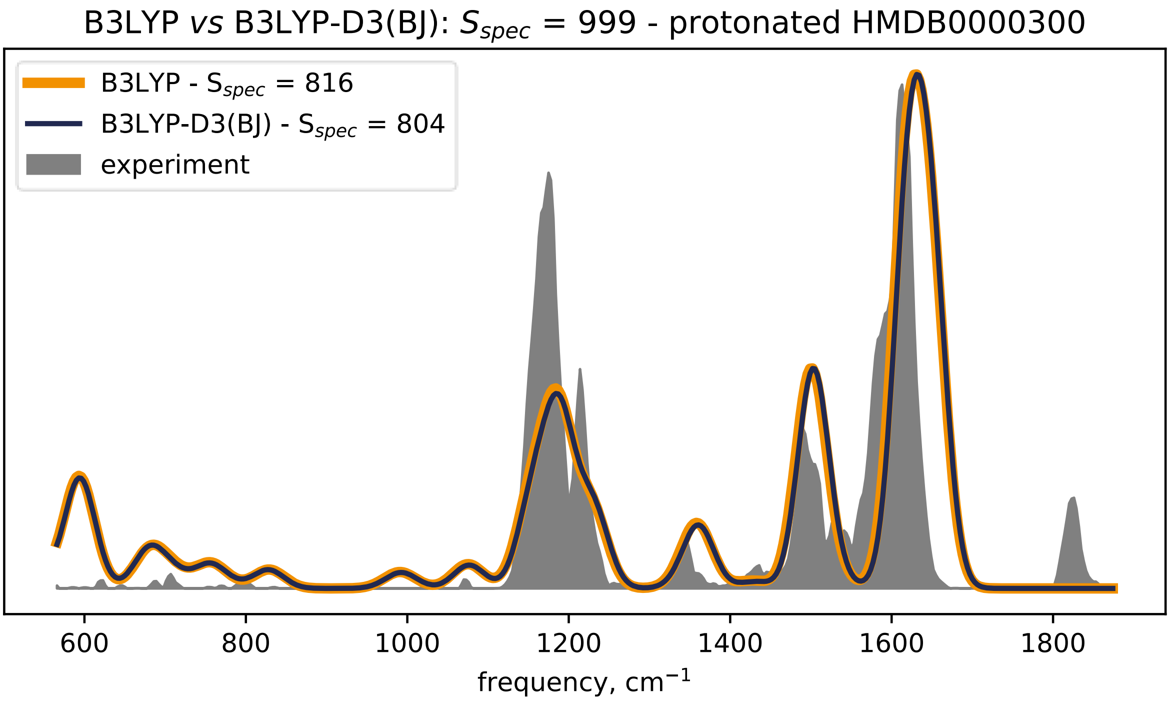
B3LYP vs B3LYP-D3(BJ): S_{spec} = 999 - protonated HMDB0000300
B3LYP - S_{spec} = 816
B3LYP-D3(BJ) - S_{spec} = 804
experiment
600
800
1000
1200
1400
1600
1800
frequency, cm^{-1}

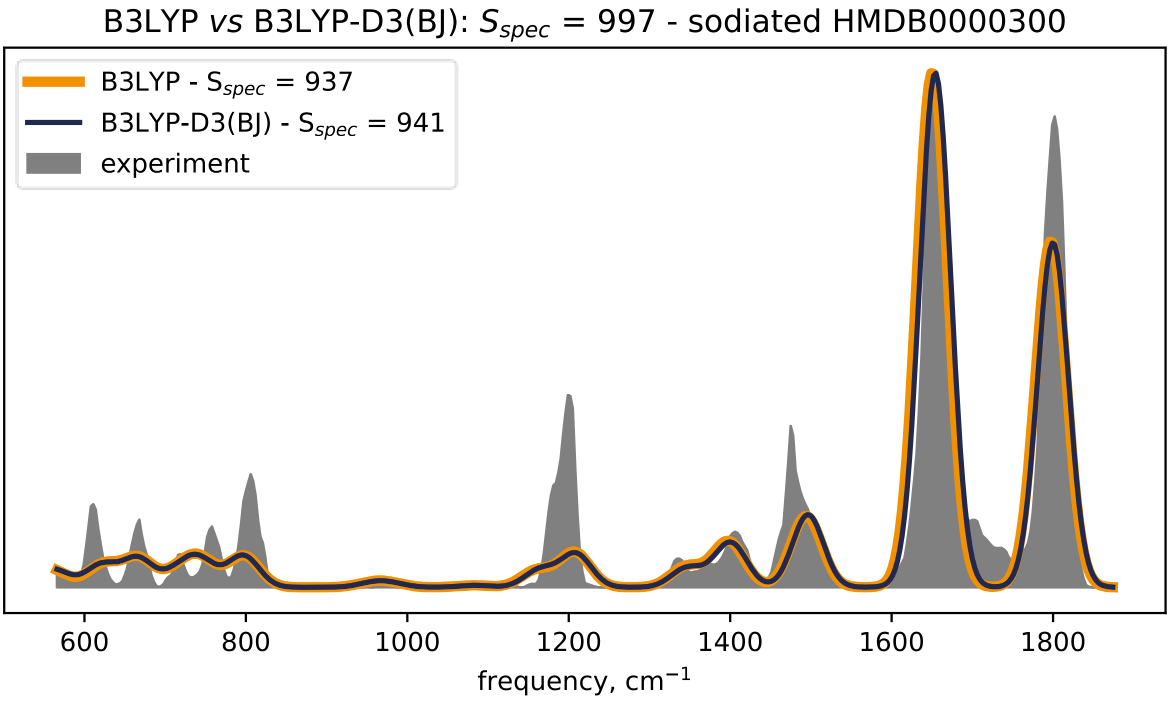
B3LYP vs B3LYP-D3(BJ): S_{spec} = 997 - sodiated HMDB0000300
B3LYP - S_{spec} = 937
B3LYP-D3(BJ) - S_{spec} = 941
experiment
600
800
1000
1200
1400
1600
1800
frequency, cm^{-1}

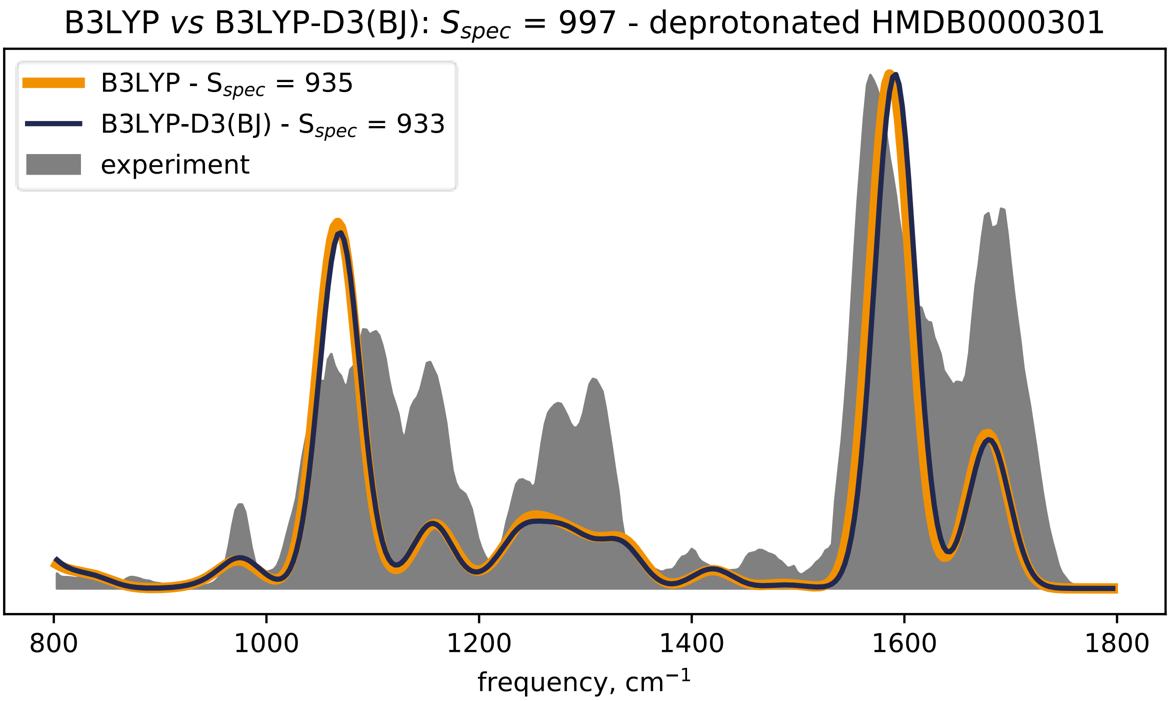

B3LYP vs B3LYP-D3(BJ): S_{spec} = 997 - deprotonated HMDB0000301
B3LYP - S_{spec} = 935
B3LYP-D3(BJ) - S_{spec} = 933
experiment
800
1000
1200
1400
1600
1800
frequency, cm^{-1}

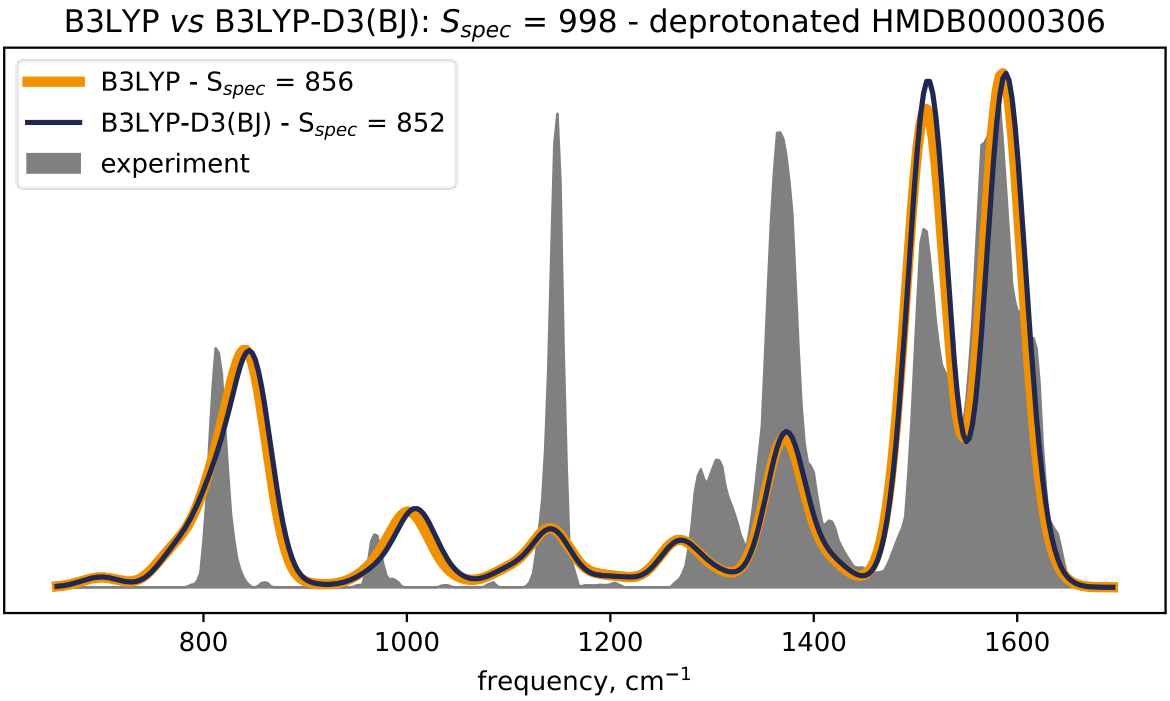

B3LYP vs B3LYP-D3(BJ): S_{spec} = 998 - deprotonated HMDB0000306
B3LYP - S_{spec} = 856
B3LYP-D3(BJ) - S_{spec} = 852
experiment
800
1000
1200
1400
1600
frequency, cm^{-1}

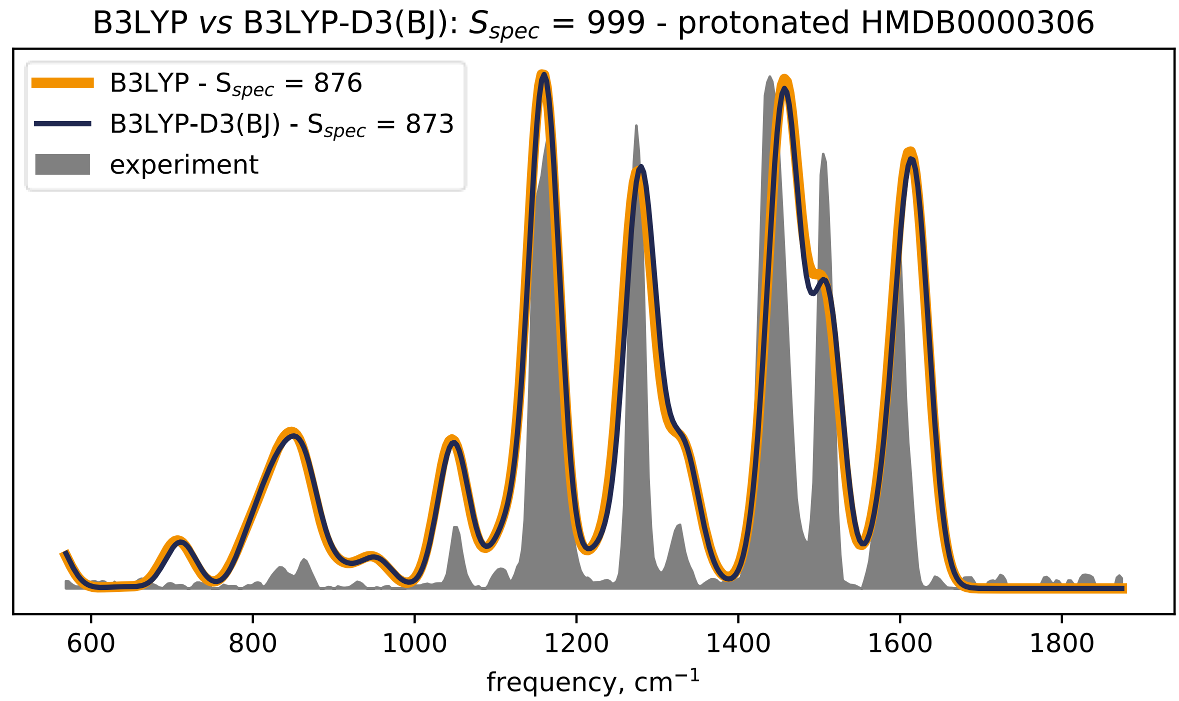
B3LYP vs B3LYP-D3(BJ): S_{spec} = 999 - protonated HMDB0000306
B3LYP - S_{spec} = 876
B3LYP-D3(BJ) - S_{spec} = 873
experiment
600
800
1000
1200
1400
1600
1800
frequency, cm^{-1}

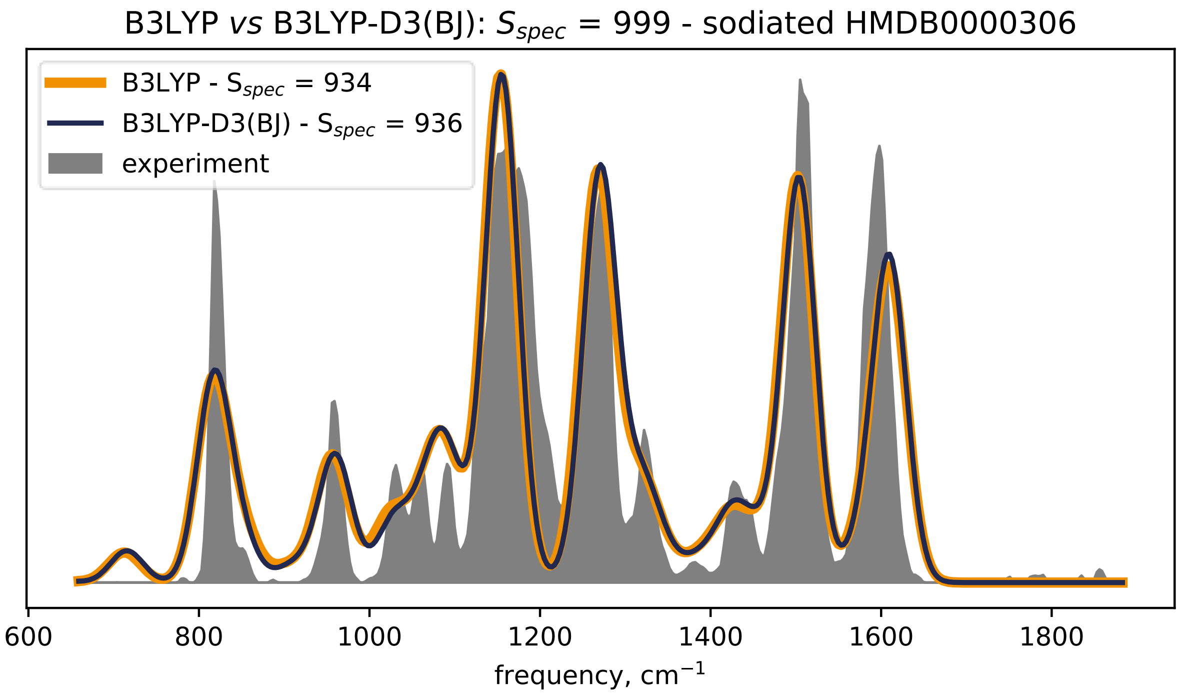
B3LYP vs B3LYP-D3(BJ): S_{spec} = 999 - sodiated HMDB0000306
B3LYP - S_{spec} = 934
B3LYP-D3(BJ) - S_{spec} = 936
experiment
600
800
1000
1200
1400
1600
1800
frequency, cm^{-1}

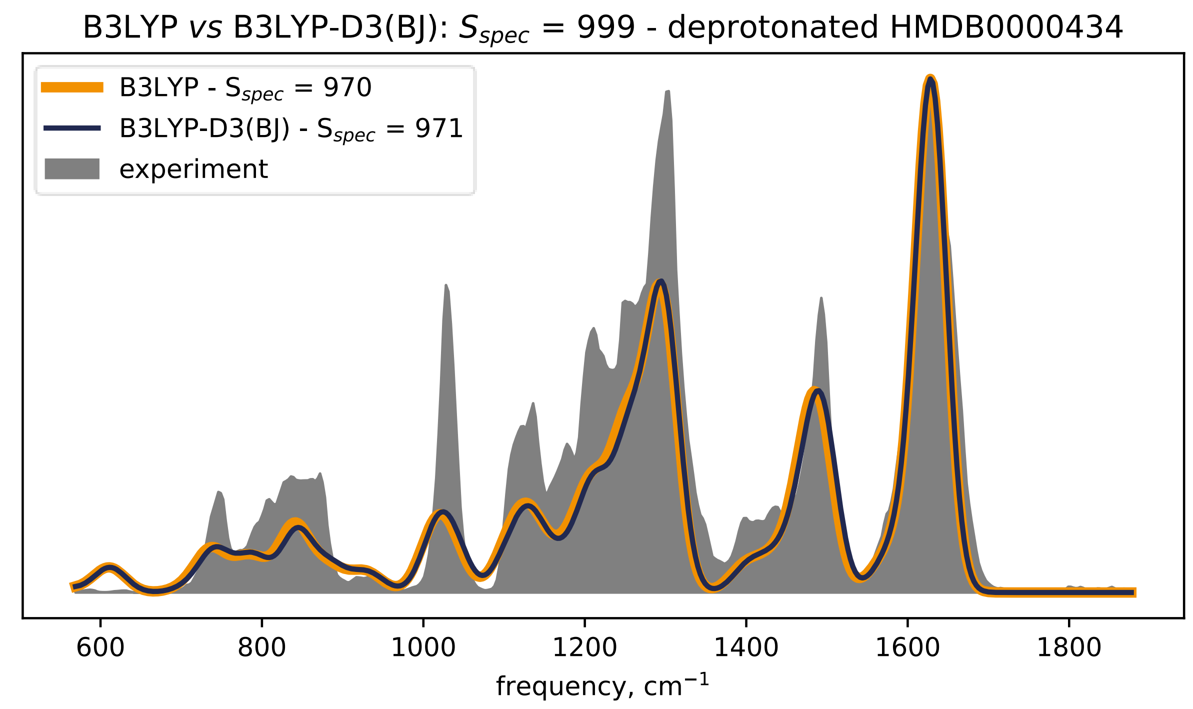

B3LYP vs B3LYP-D3(BJ): S_{spec} = 999 - deprotonated HMDB0000434
B3LYP - S_{spec} = 970
B3LYP-D3(BJ) - S_{spec} = 971
experiment
600
800
1000
1200
1400
1600
1800
frequency, cm^{-1}

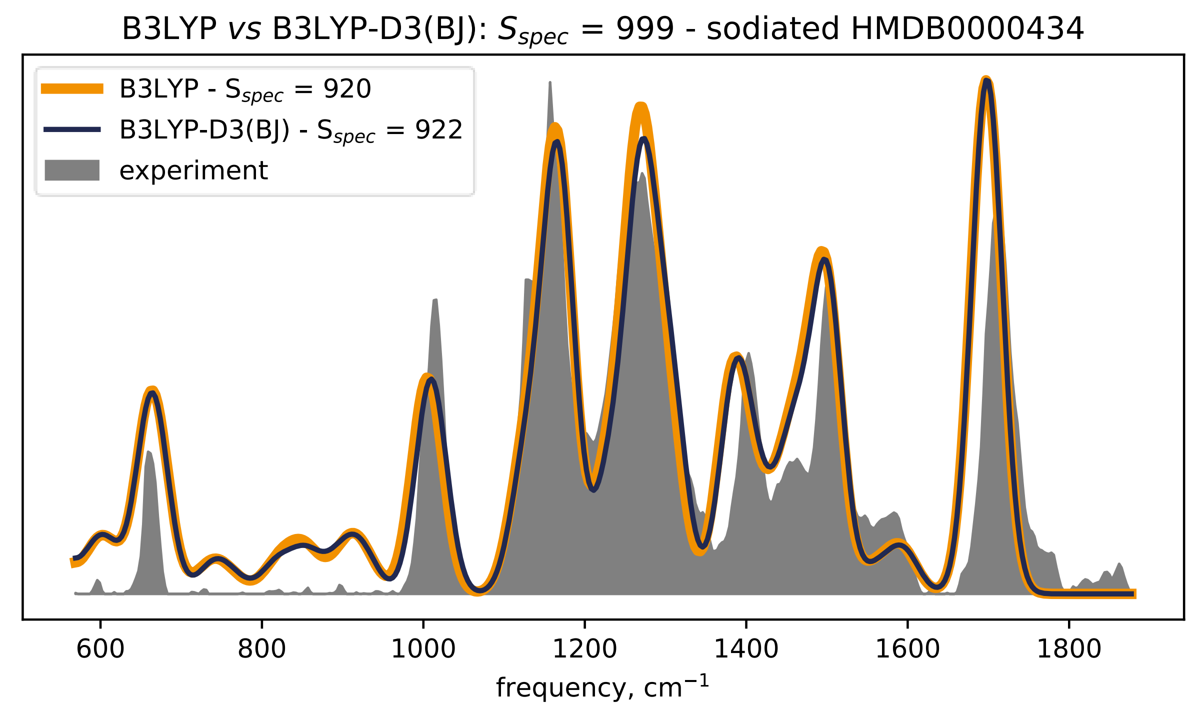

B3LYP vs B3LYP-D3(BJ): S_{spec} = 999 - sodiated HMDB0000434
B3LYP - S_{spec} = 920
B3LYP-D3(BJ) - S_{spec} = 922
experiment
600
800
1000
1200
1400
1600
1800
frequency, cm^{-1}

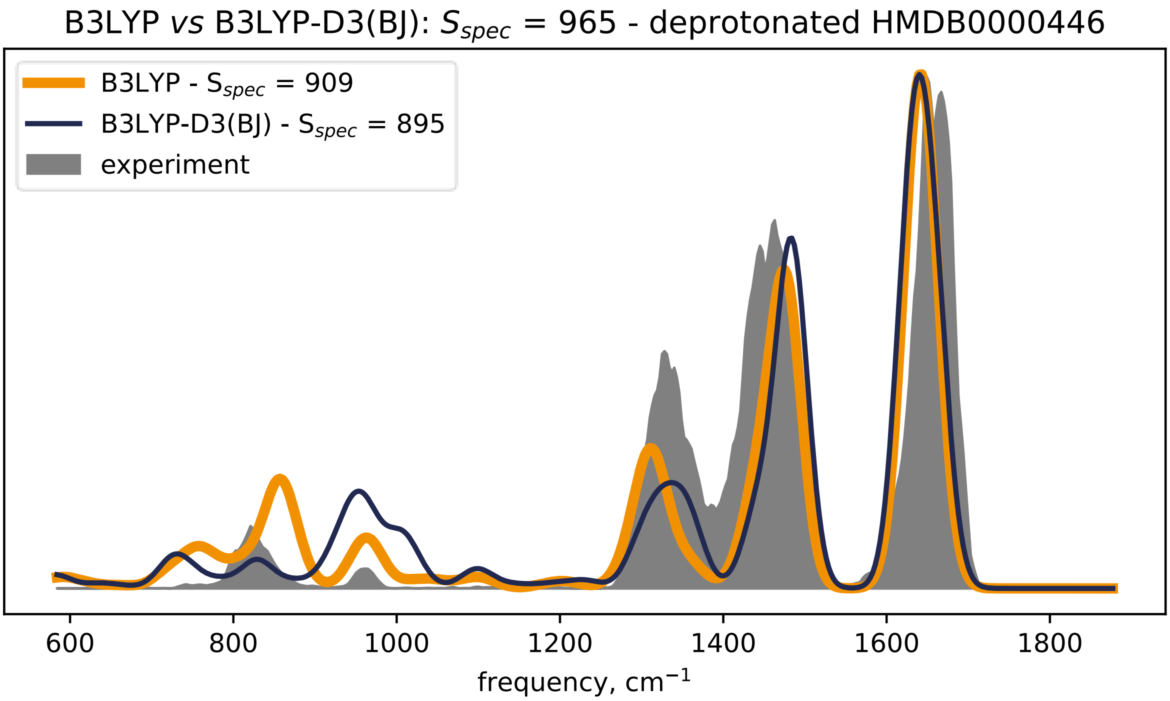
B3LYP vs B3LYP-D3(BJ): S_{spec} = 965 - deprotonated HMDB0000446
B3LYP - S_{spec} = 909
B3LYP-D3(BJ) - S_{spec} = 895
experiment
600
800
1000
1200
1400
1600
1800
frequency, cm^{-1}

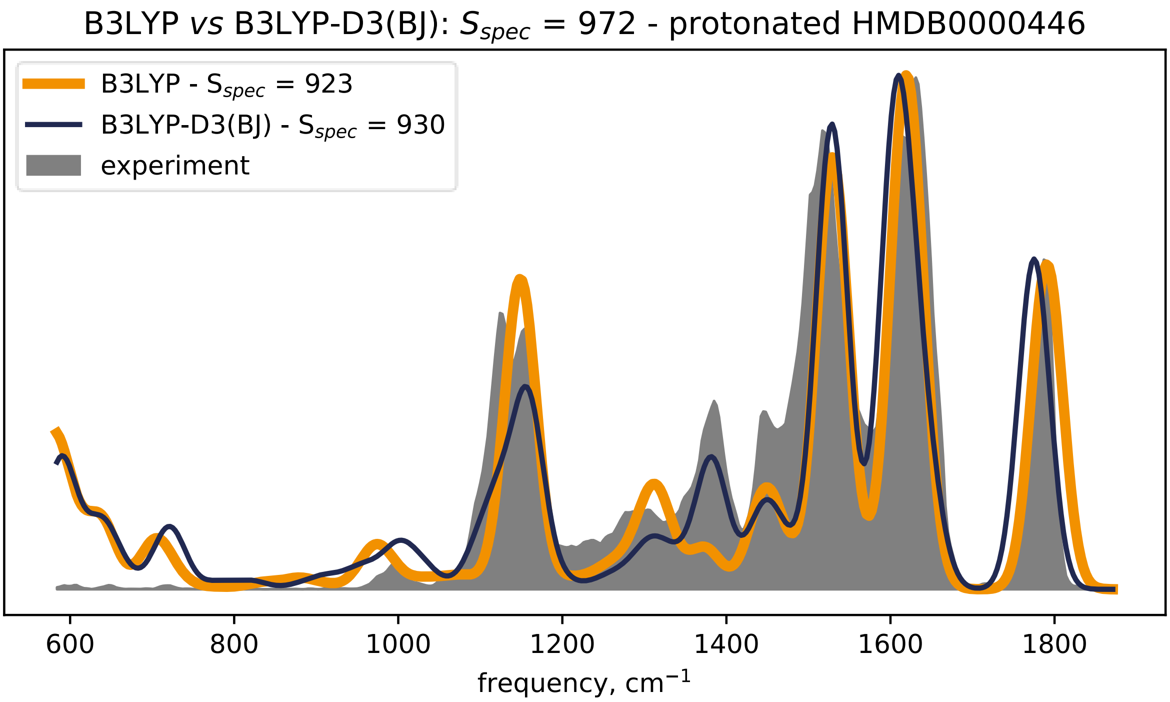
B3LYP vs B3LYP-D3(BJ): S_{spec} = 972 - protonated HMDB0000446
B3LYP - S_{spec} = 923
B3LYP-D3(BJ) - S_{spec} = 930
experiment
600
800
1000
1200
1400
1600
1800
frequency, cm^{-1}

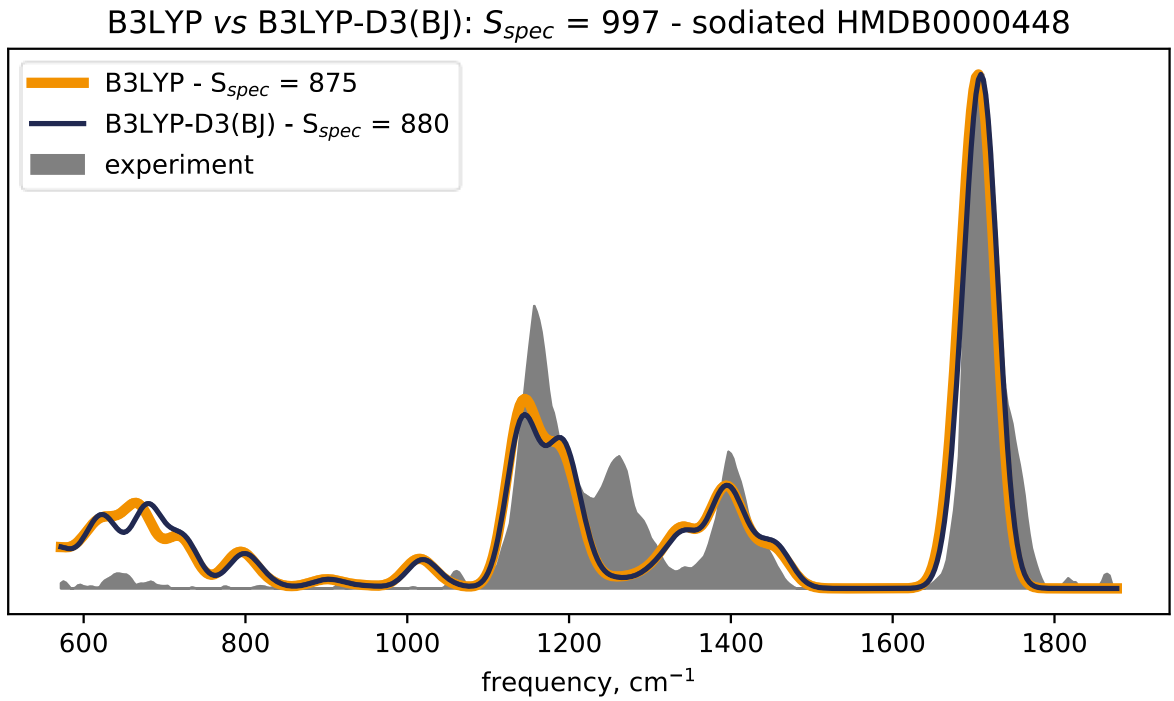
B3LYP vs B3LYP-D3(BJ): S_{spec} = 997 - sodiated HMDB0000448
B3LYP - S_{spec} = 875
B3LYP-D3(BJ) - S_{spec} = 880
experiment
600
800
1000
1200
1400
1600
1800
frequency, cm^{-1}

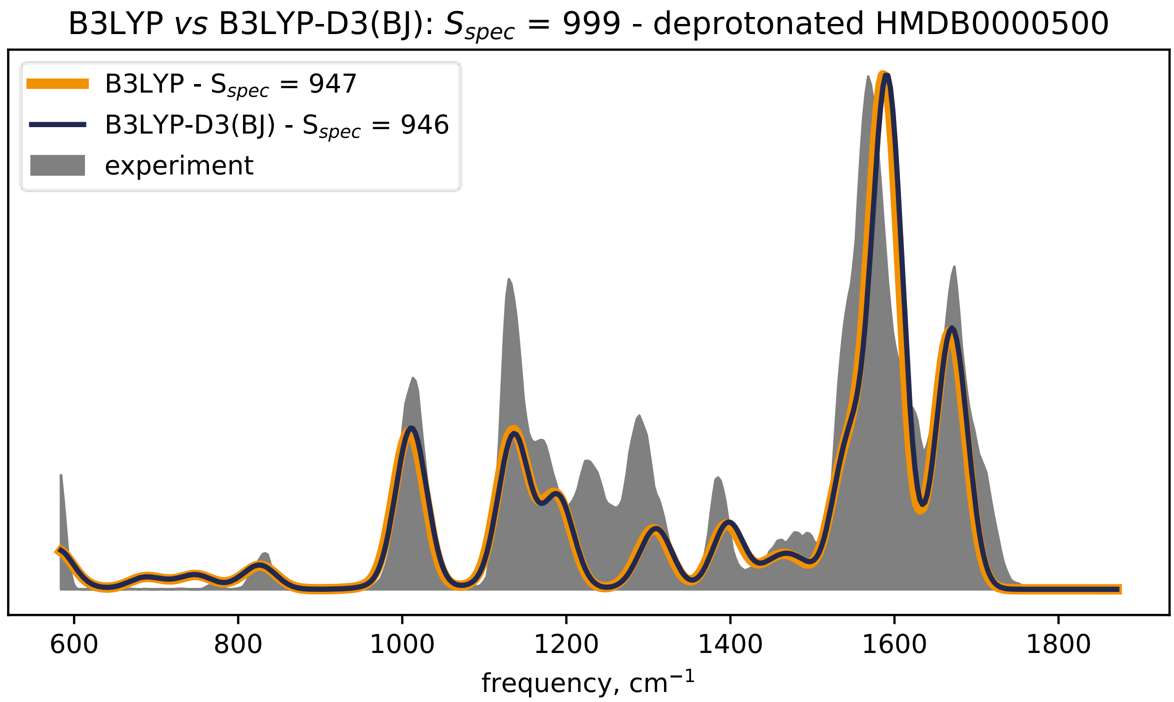
B3LYP vs B3LYP-D3(BJ): S_{spec} = 999 - deprotonated HMDB0000500
B3LYP - S_{spec} = 947
B3LYP-D3(BJ) - S_{spec} = 946
experiment
600
800
1000
1200
1400
1600
1800
frequency, cm^{-1}

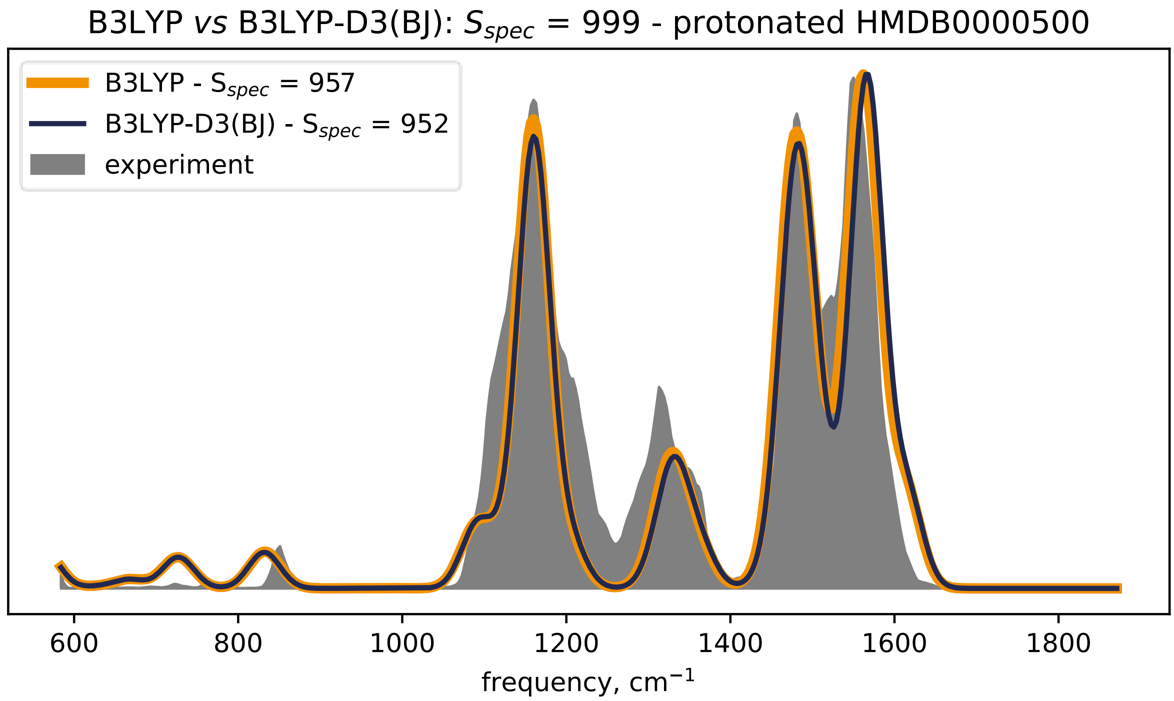
B3LYP vs B3LYP-D3(BJ): S_{spec} = 999 - protonated HMDB0000500
B3LYP - S_{spec} = 957
B3LYP-D3(BJ) - S_{spec} = 952
experiment
600
800
1000
1200
1400
1600
1800
frequency, cm^{-1}

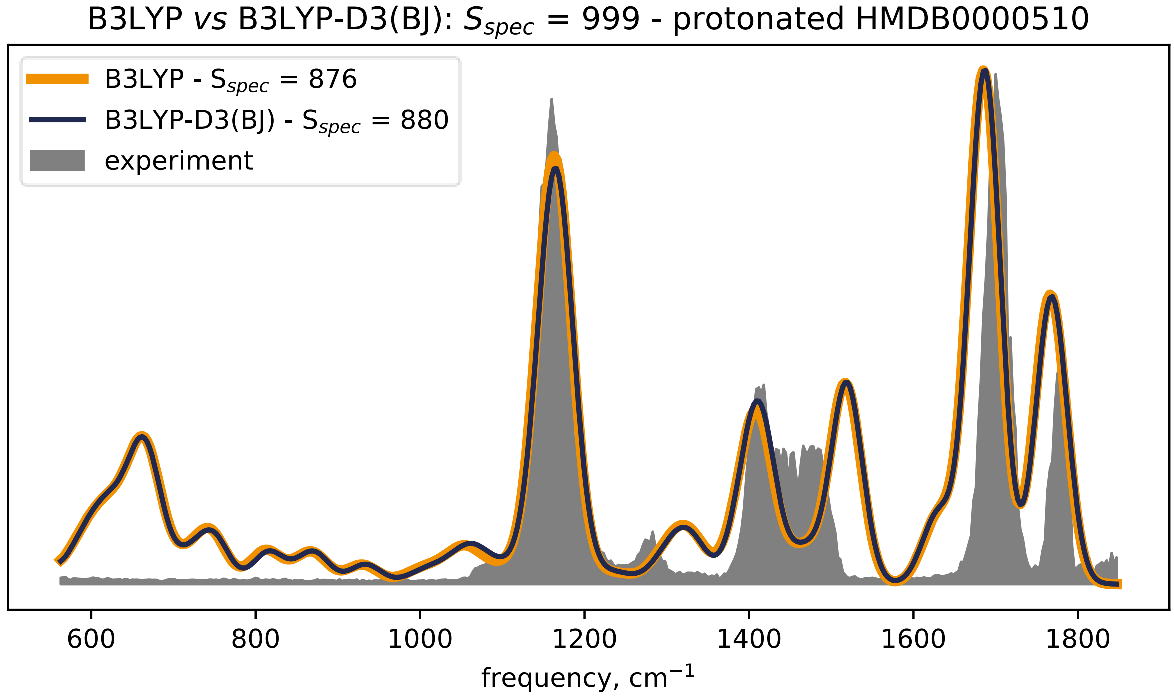
B3LYP vs B3LYP-D3(BJ): S_{spec} = 999 - protonated HMDB0000510
B3LYP - S_{spec} = 876
B3LYP-D3(BJ) - S_{spec} = 880
experiment
600
800
1000
1200
1400
1600
1800
frequency, cm^{-1}

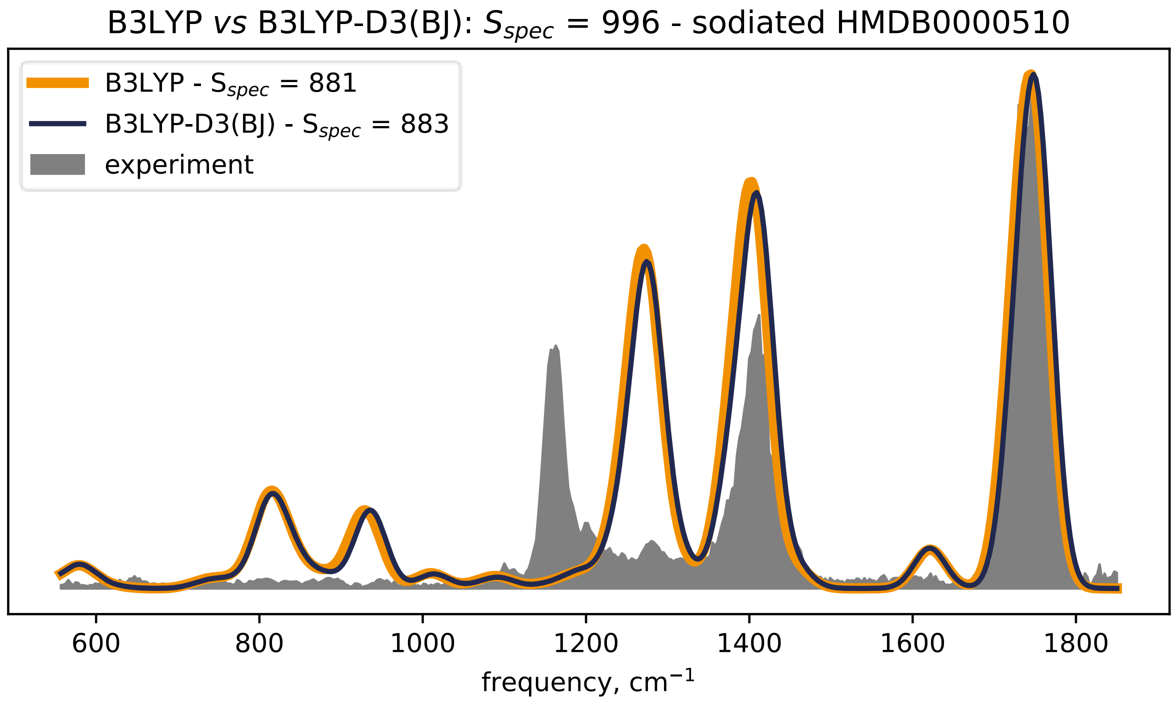
B3LYP vs B3LYP-D3(BJ): S_{spec} = 996 - sodiated HMDB0000510
B3LYP - S_{spec} = 881
B3LYP-D3(BJ) - S_{spec} = 883
experiment
600
800
1000
1200
1400
1600
1800
frequency, cm^{-1}

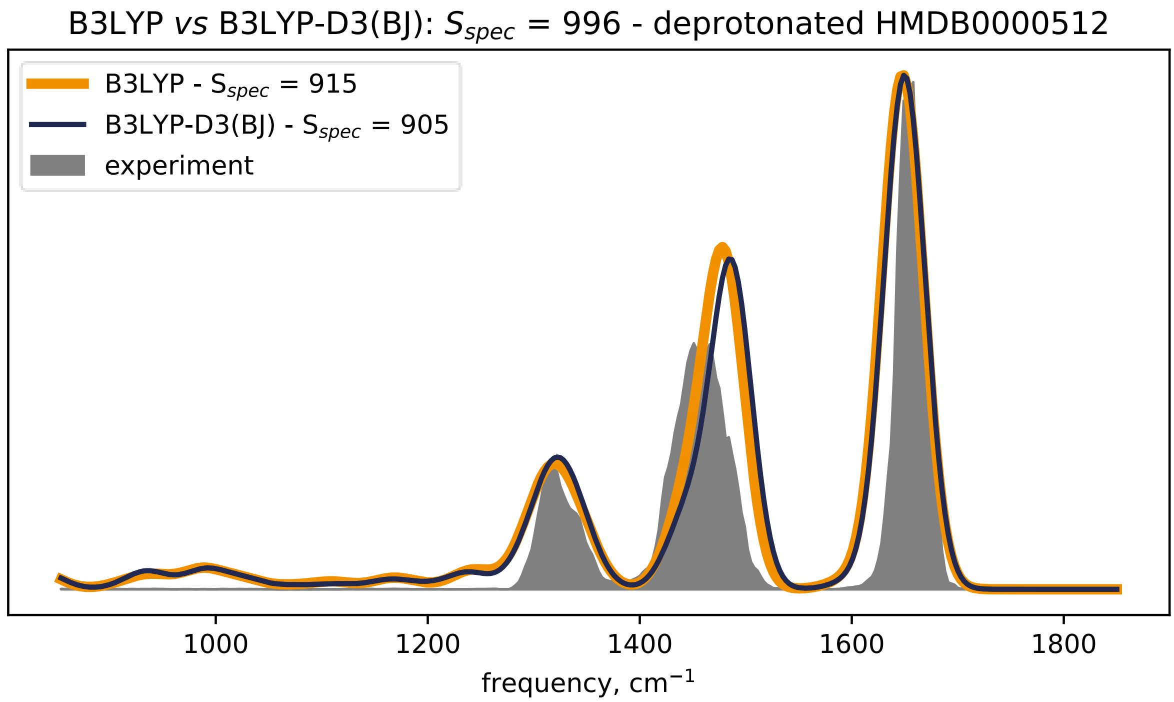
B3LYP vs B3LYP-D3(BJ): S_{spec} = 996 - deprotonated HMDB0000512
B3LYP - S_{spec} = 915
B3LYP-D3(BJ) - S_{spec} = 905
experiment
1000
1200
1400
1600
1800
frequency, cm^{-1}

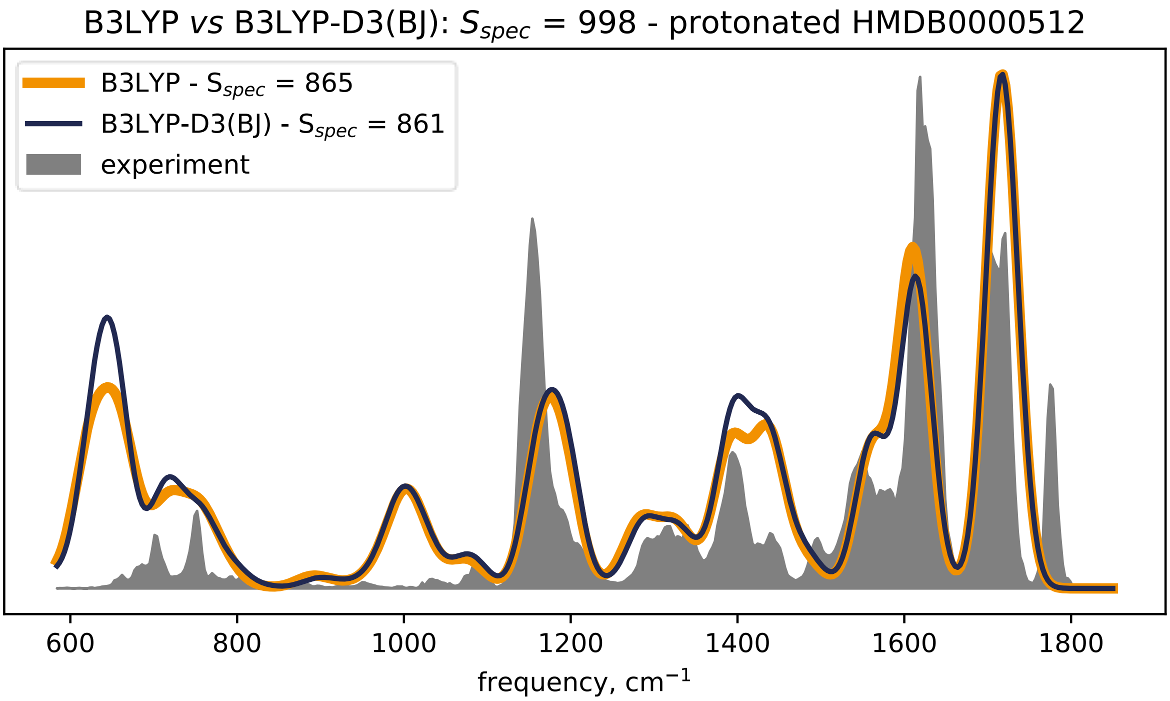

B3LYP vs B3LYP-D3(BJ): S_{spec} = 998 - protonated HMDB0000512
B3LYP - S_{spec} = 865
B3LYP-D3(BJ) - S_{spec} = 861
experiment
600
800
1000
1200
1400
1600
1800
frequency, cm^{-1}

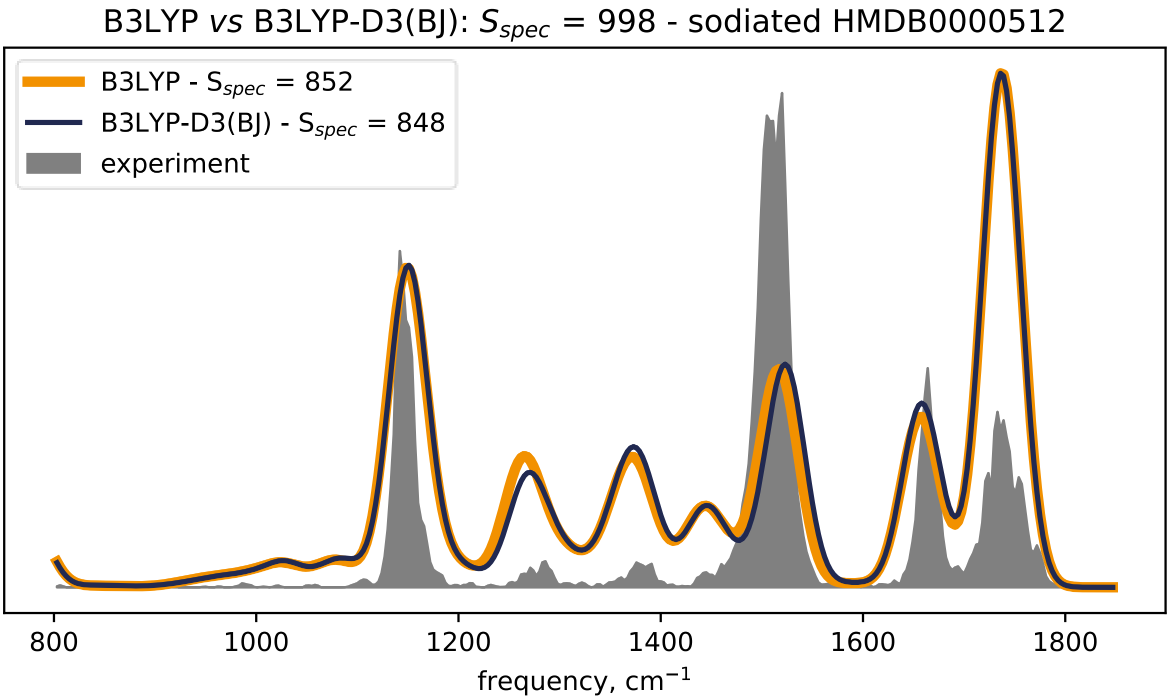

B3LYP vs B3LYP-D3(BJ): S_{spec} = 998 - sodiated HMDB0000512
B3LYP - S_{spec} = 852
B3LYP-D3(BJ) - S_{spec} = 848
experiment
800
1000
1200
1400
1600
1800
frequency, cm^{-1}

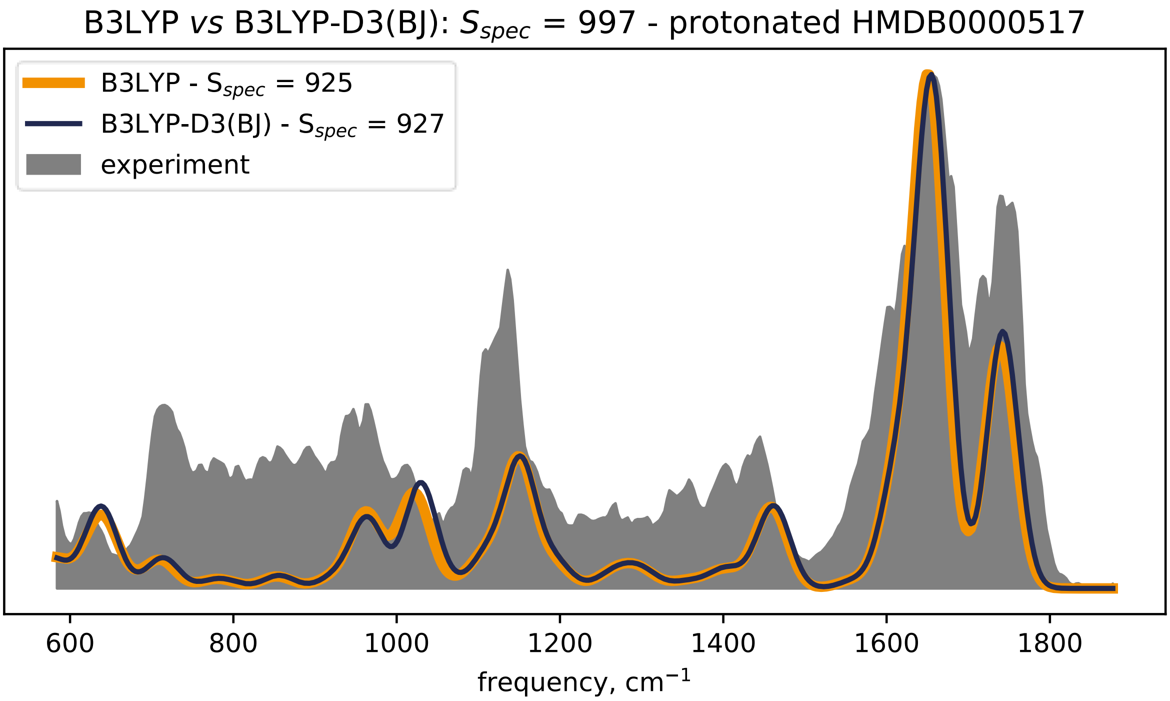
B3LYP vs B3LYP-D3(BJ): S_{spec} = 997 - protonated HMDB0000517
B3LYP - S_{spec} = 925
B3LYP-D3(BJ) - S_{spec} = 927
experiment
600
800
1000
1200
1400
1600
1800
frequency, cm^{-1}

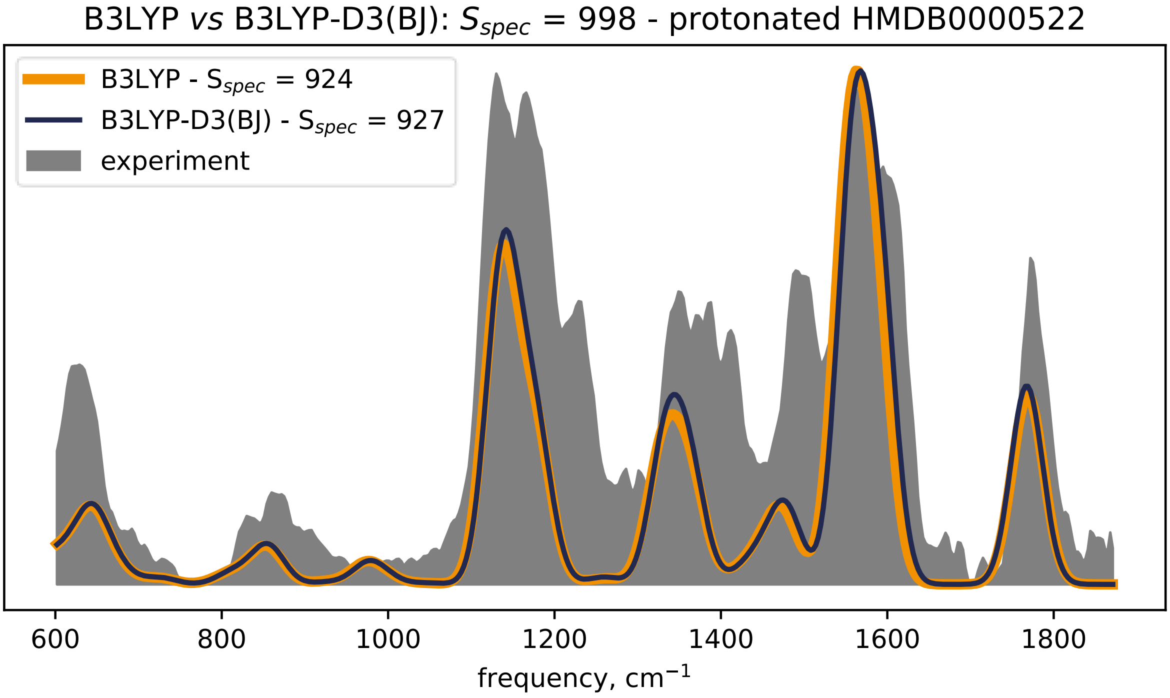
B3LYP vs B3LYP-D3(BJ): S_{spec} = 998 - protonated HMDB0000522
B3LYP - S_{spec} = 924
B3LYP-D3(BJ) - S_{spec} = 927
experiment
600
800
1000
1200
1400
1600
1800
frequency, cm^{-1}

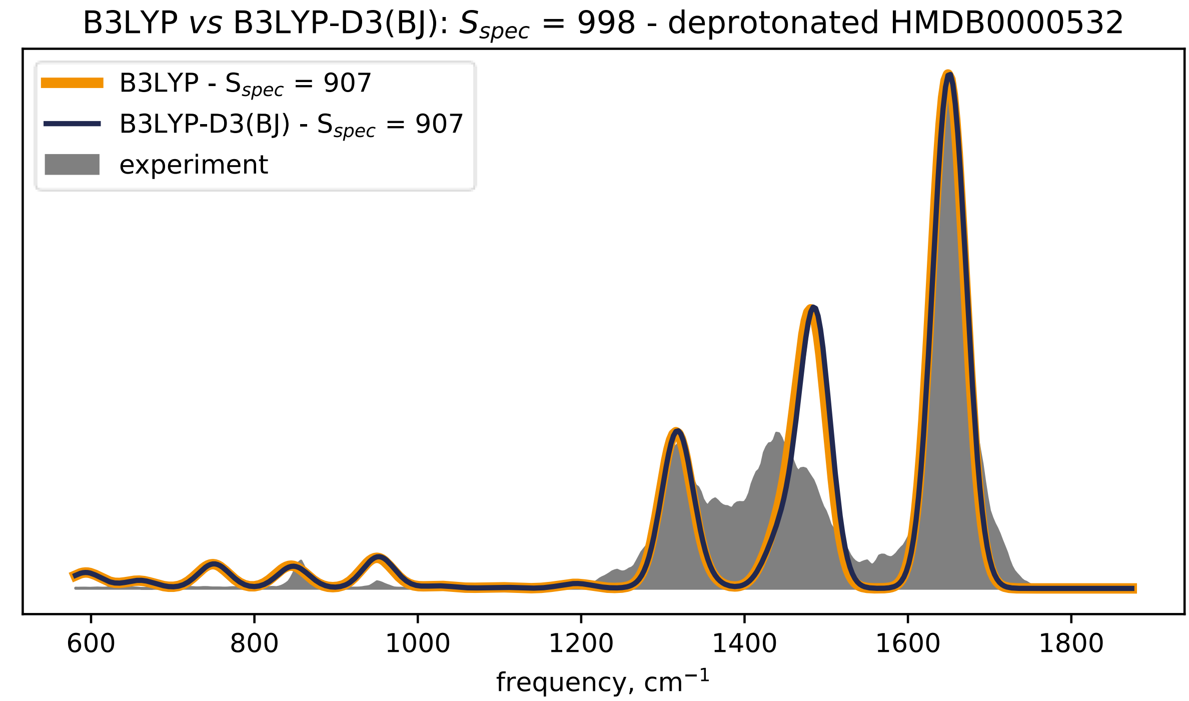
B3LYP vs B3LYP-D3(BJ): S_{spec} = 998 - deprotonated HMDB0000532
B3LYP - S_{spec} = 907
B3LYP-D3(BJ) - S_{spec} = 907
experiment
600
800
1000
1200
1400
1600
1800
frequency, cm^{-1}

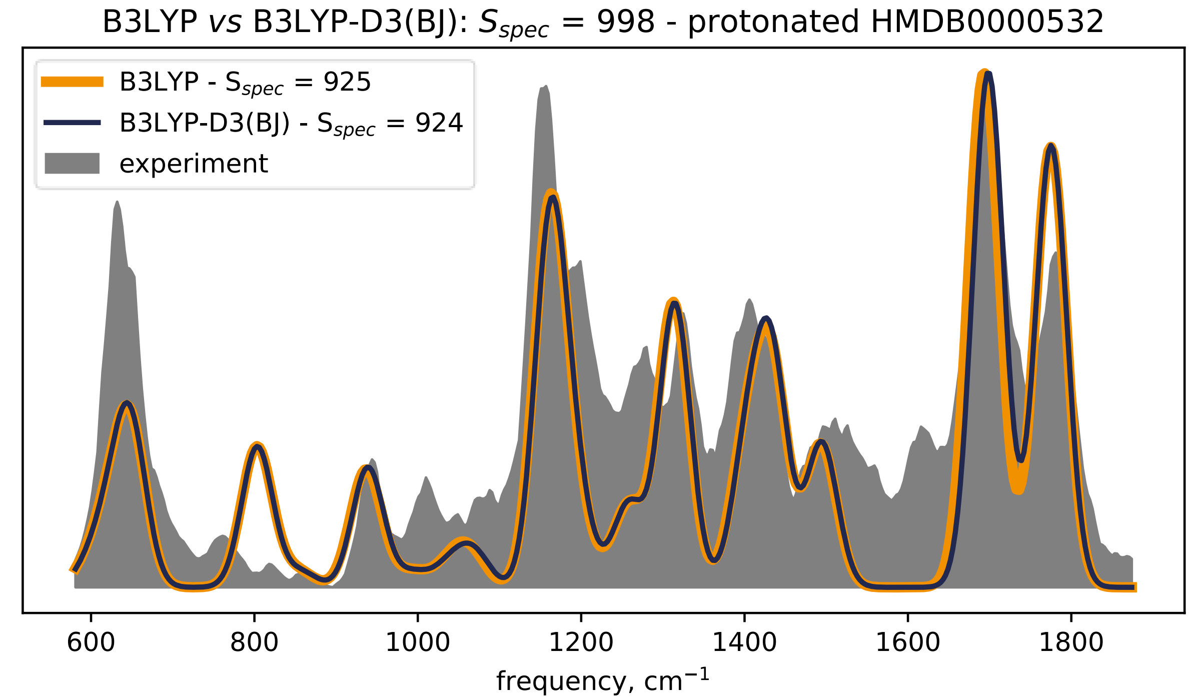
B3LYP vs B3LYP-D3(BJ): S_{spec} = 998 - protonated HMDB0000532
B3LYP - S_{spec} = 925
B3LYP-D3(BJ) - S_{spec} = 924
experiment
600
800
1000
1200
1400
1600
1800
frequency, cm^{-1}

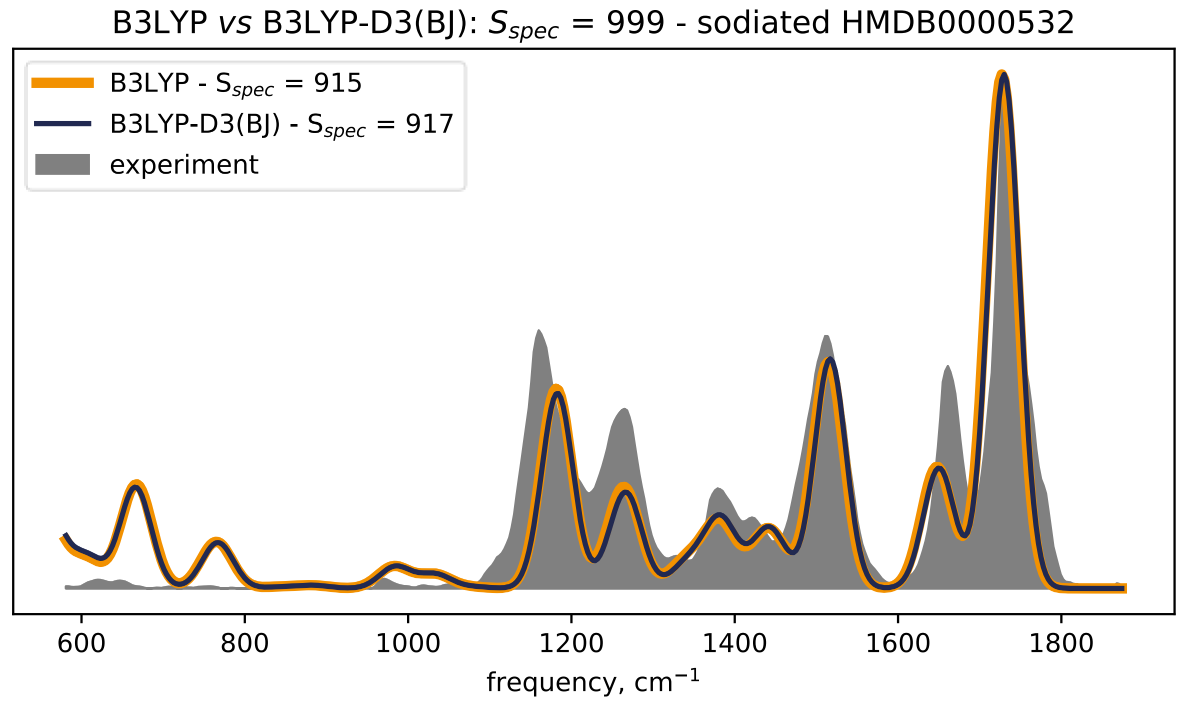
B3LYP vs B3LYP-D3(BJ): S_{spec} = 999 - sodiated HMDB0000532
B3LYP - S_{spec} = 915
B3LYP-D3(BJ) - S_{spec} = 917
experiment
600
800
1000
1200
1400
1600
1800
frequency, cm^{-1}

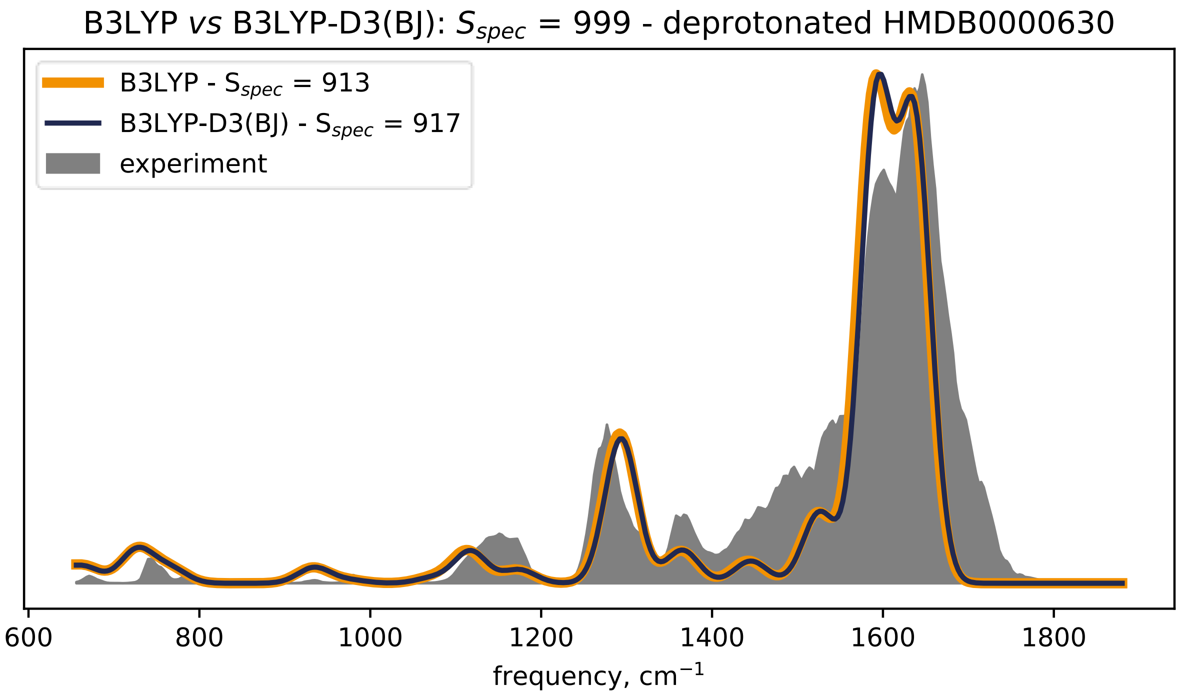
B3LYP vs B3LYP-D3(BJ): S_{spec} = 999 - deprotonated HMDB0000630
B3LYP - S_{spec} = 913
B3LYP-D3(BJ) - S_{spec} = 917
experiment
600
800
1000
1200
1400
1600
1800
frequency, cm^{-1}

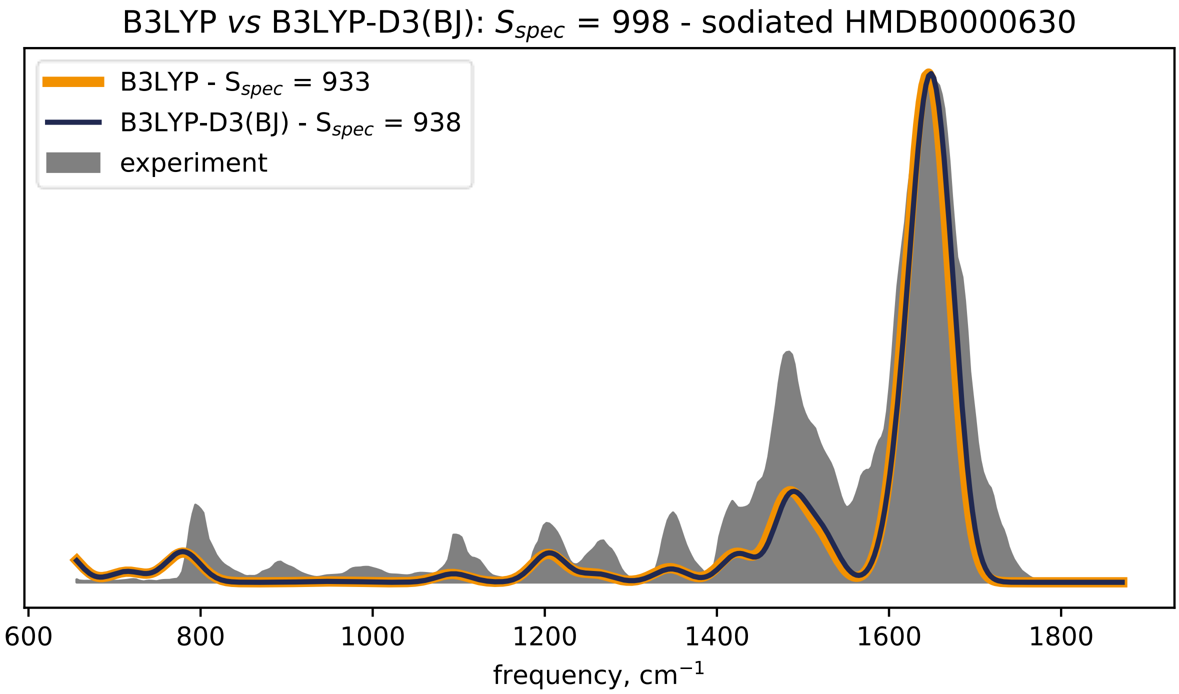
B3LYP vs B3LYP-D3(BJ): S_{spec} = 998 - sodiated HMDB0000630
B3LYP - S_{spec} = 933
B3LYP-D3(BJ) - S_{spec} = 938
experiment
600
800
1000
1200
1400
1600
1800
frequency, cm^{-1}

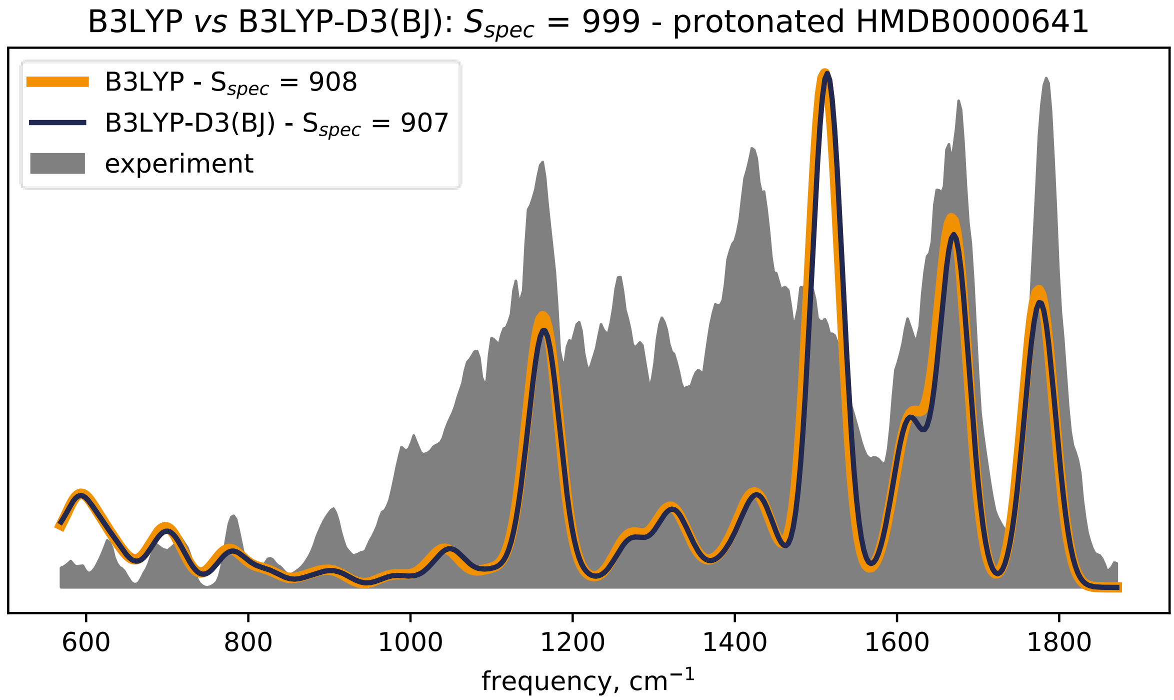
B3LYP vs B3LYP-D3(BJ): S_{spec} = 999 - protonated HMDB0000641
B3LYP - S_{spec} = 908
B3LYP-D3(BJ) - S_{spec} = 907
experiment
600
800
1000
1200
1400
1600
1800
frequency, cm^{-1}

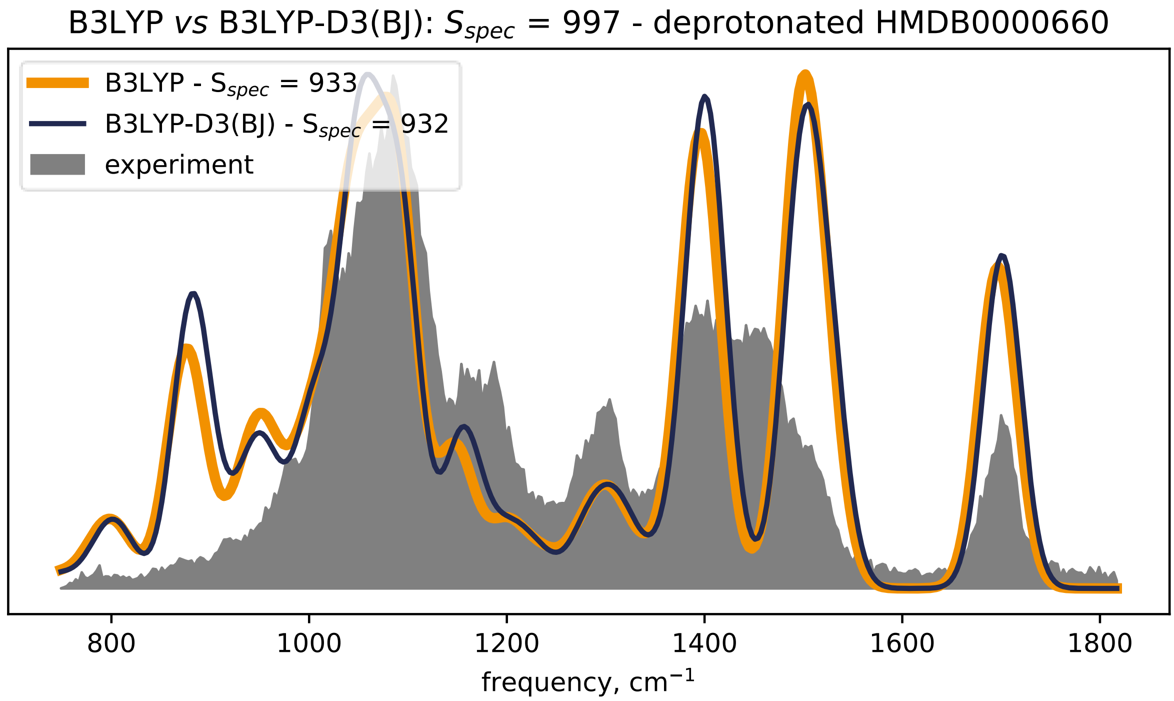

B3LYP vs B3LYP-D3(BJ): S_{spec} = 997 - deprotonated HMDB0000660
B3LYP - S_{spec} = 933
B3LYP-D3(BJ) - S_{spec} = 932
experiment
800
1000
1200
1400
1600
1800
frequency, cm^{-1}

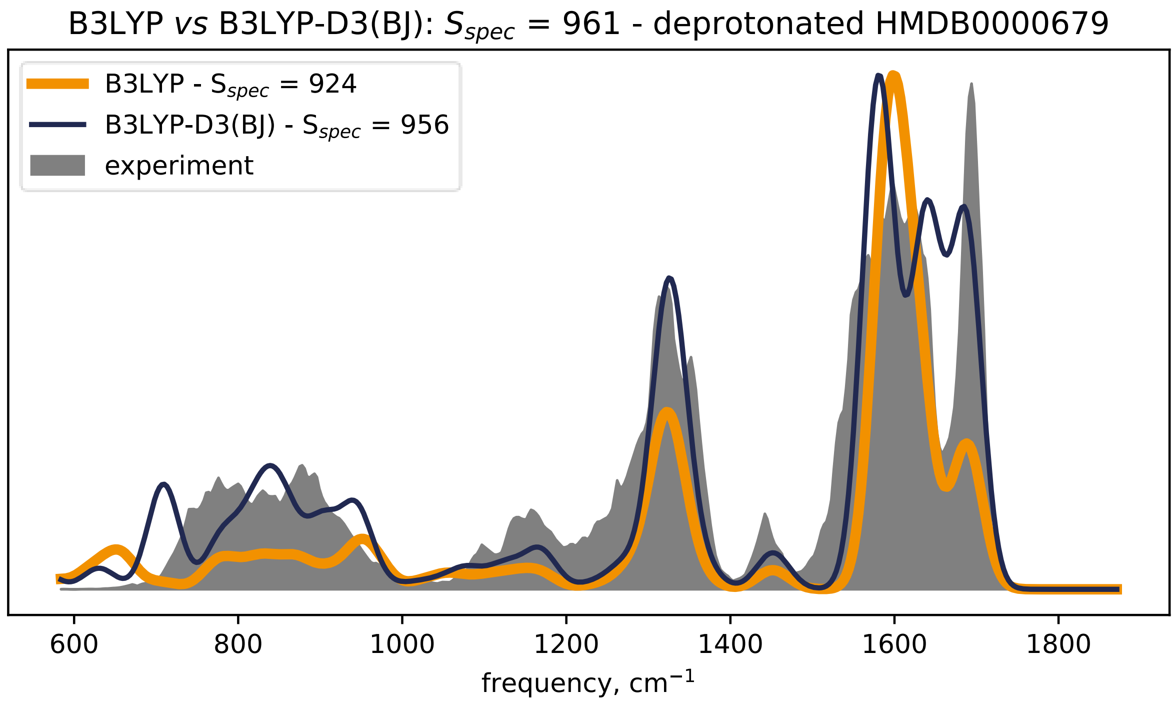

B3LYP vs B3LYP-D3(BJ): S_{spec} = 961 - deprotonated HMDB0000679
B3LYP - S_{spec} = 924
B3LYP-D3(BJ) - S_{spec} = 956
experiment
600
800
1000
1200
1400
1600
1800
frequency, cm^{-1}

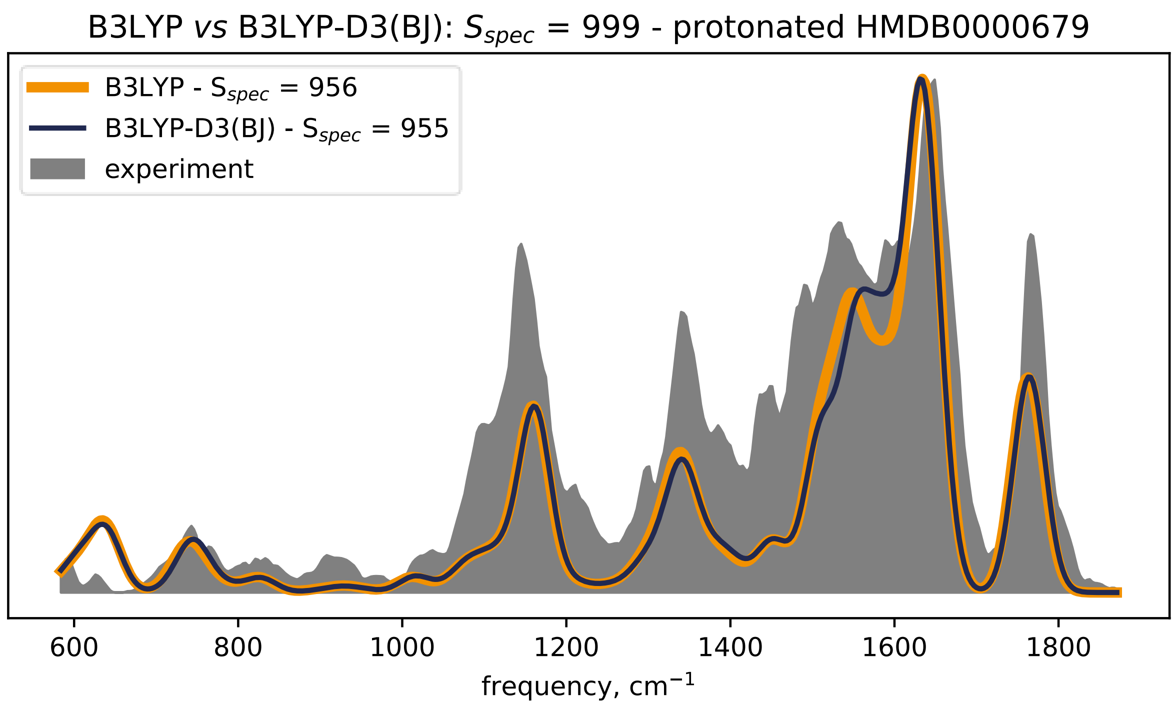
B3LYP vs B3LYP-D3(BJ): S_{spec} = 999 - protonated HMDB0000679
B3LYP - S_{spec} = 956
B3LYP-D3(BJ) - S_{spec} = 955
experiment
600
800
1000
1200
1400
1600
1800
frequency, cm^{-1}

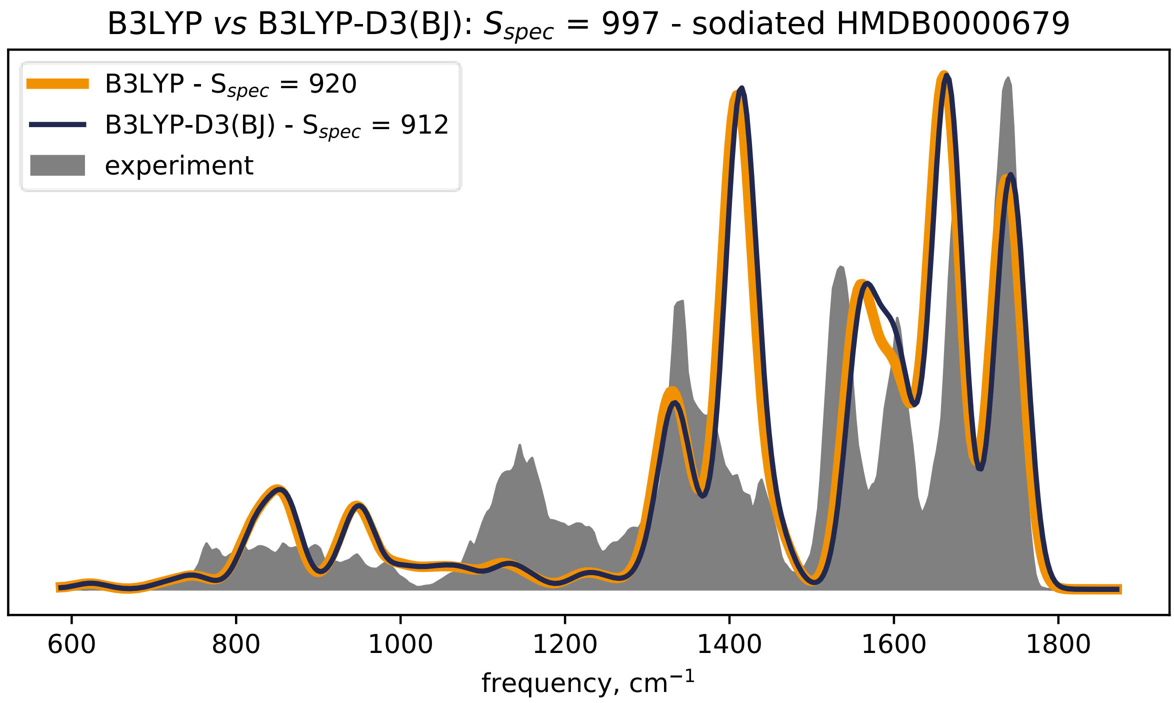
B3LYP vs B3LYP-D3(BJ): S_{spec} = 997 - sodiated HMDB0000679
B3LYP - S_{spec} = 920
B3LYP-D3(BJ) - S_{spec} = 912
experiment
600
800
1000
1200
1400
1600
1800
frequency, cm^{-1}

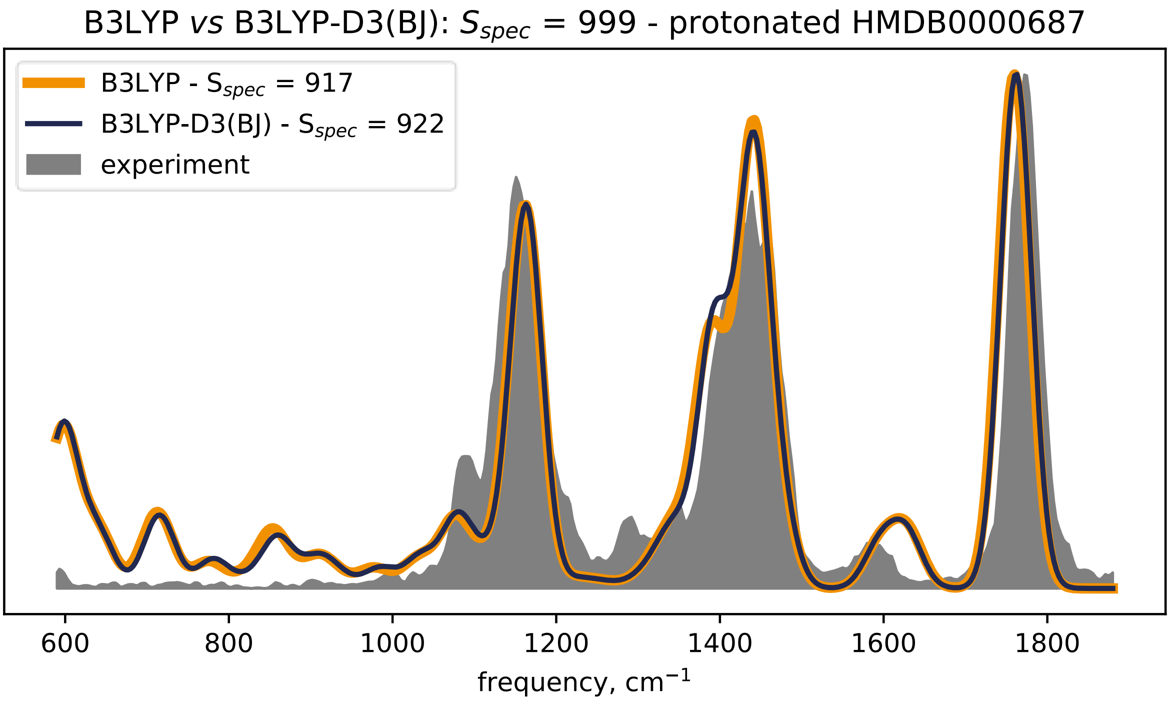
B3LYP vs B3LYP-D3(BJ): S_{spec} = 999 - protonated HMDB0000687
B3LYP - S_{spec} = 917
B3LYP-D3(BJ) - S_{spec} = 922
experiment
600
800
1000
1200
1400
1600
1800
frequency, cm^{-1}

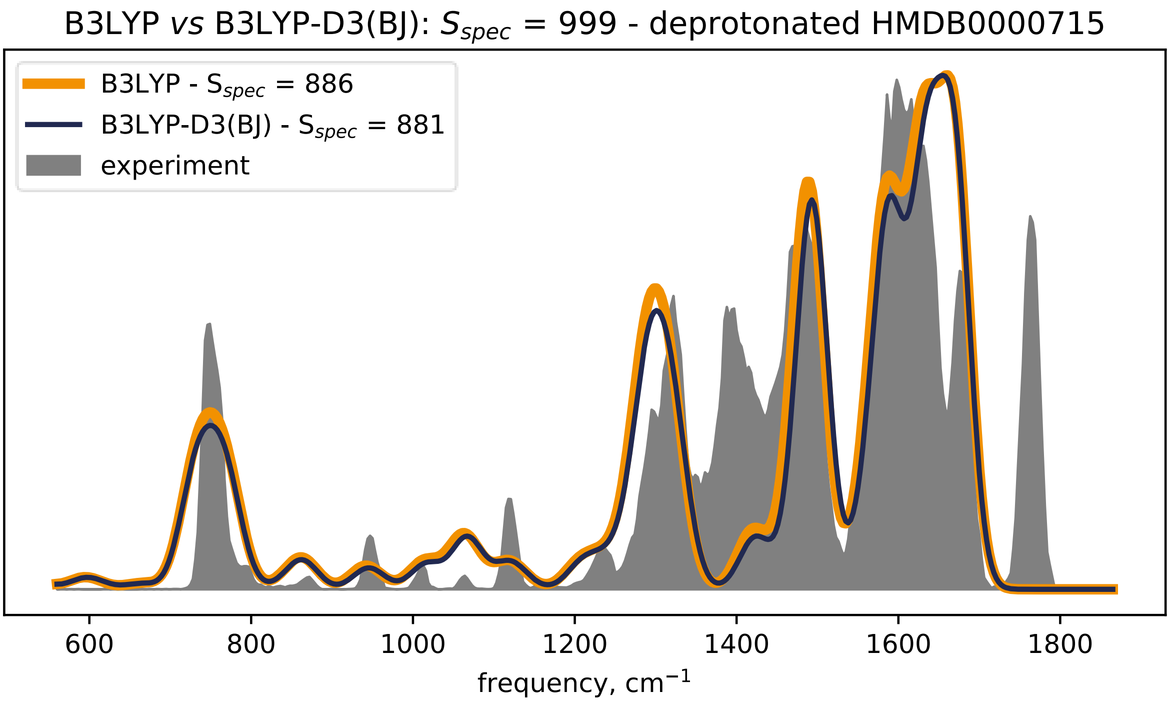
B3LYP vs B3LYP-D3(BJ): S_{spec} = 999 - deprotonated HMDB0000715
B3LYP - S_{spec} = 886
B3LYP-D3(BJ) - S_{spec} = 881
experiment
600
800
1000
1200
1400
1600
1800
frequency, cm^{-1}

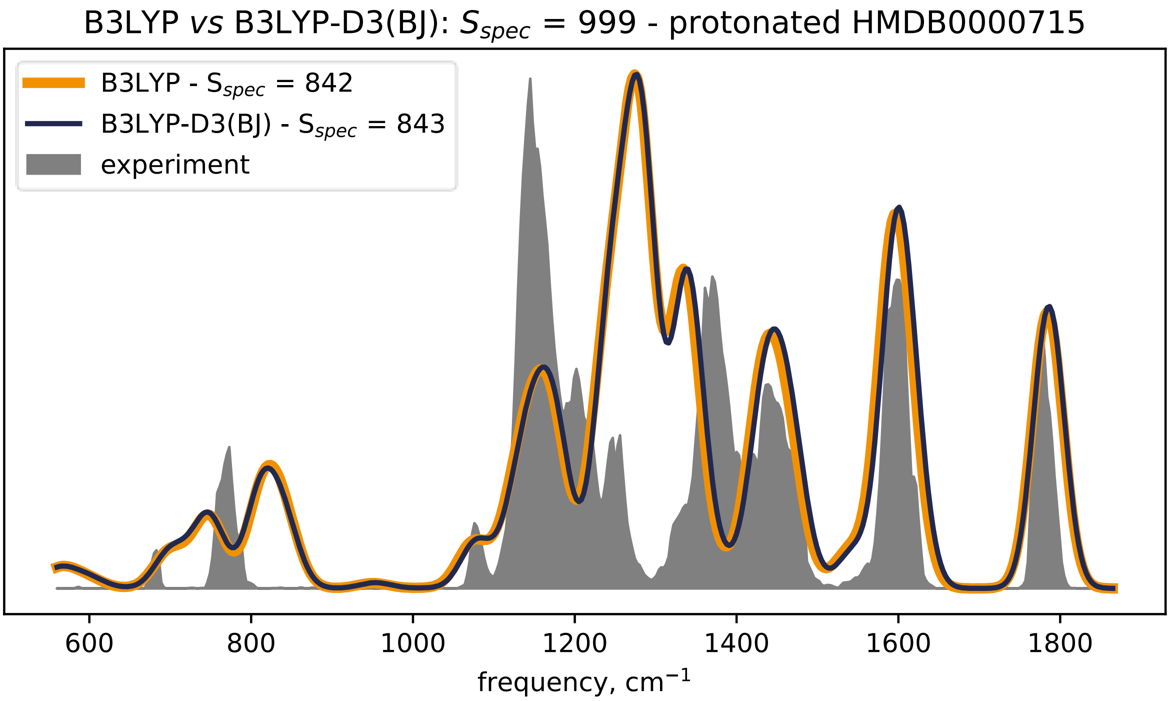
B3LYP vs B3LYP-D3(BJ): S_{spec} = 999 - protonated HMDB0000715
B3LYP - S_{spec} = 842
B3LYP-D3(BJ) - S_{spec} = 843
experiment
600
800
1000
1200
1400
1600
1800
frequency, cm^{-1}

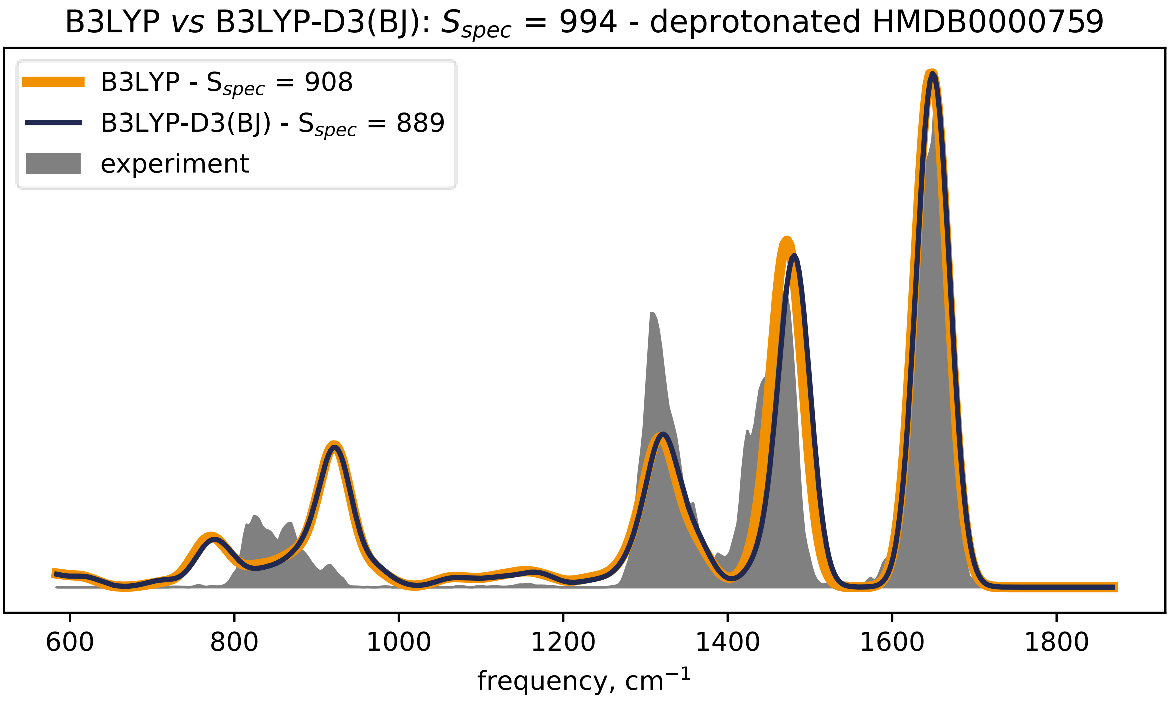
B3LYP vs B3LYP-D3(BJ): S_{spec} = 994 - deprotonated HMDB0000759
B3LYP - S_{spec} = 908
B3LYP-D3(BJ) - S_{spec} = 889
experiment
600
800
1000
1200
1400
1600
1800
frequency, cm^{-1}

B3LYP *vs* B3LYP-D3(BJ): S_{spec} = 980 - deprotonated HMDB0000765

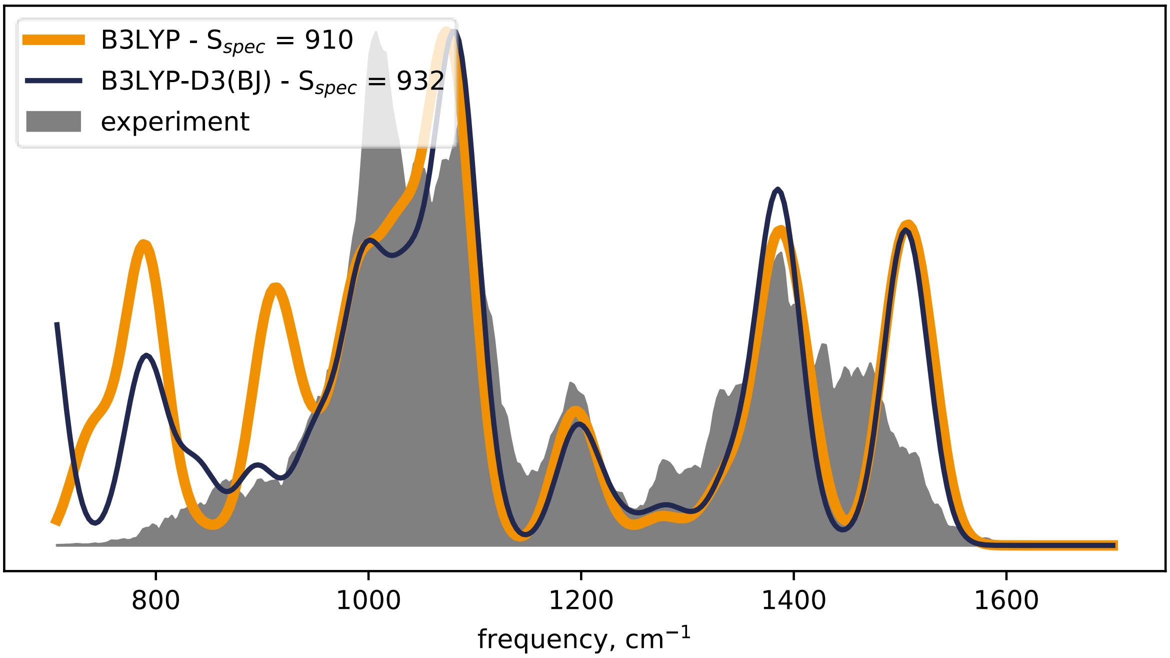

B3LYP *vs* B3LYP-D3(BJ): S_{spec} = 997 - deprotonated HMDB0000821

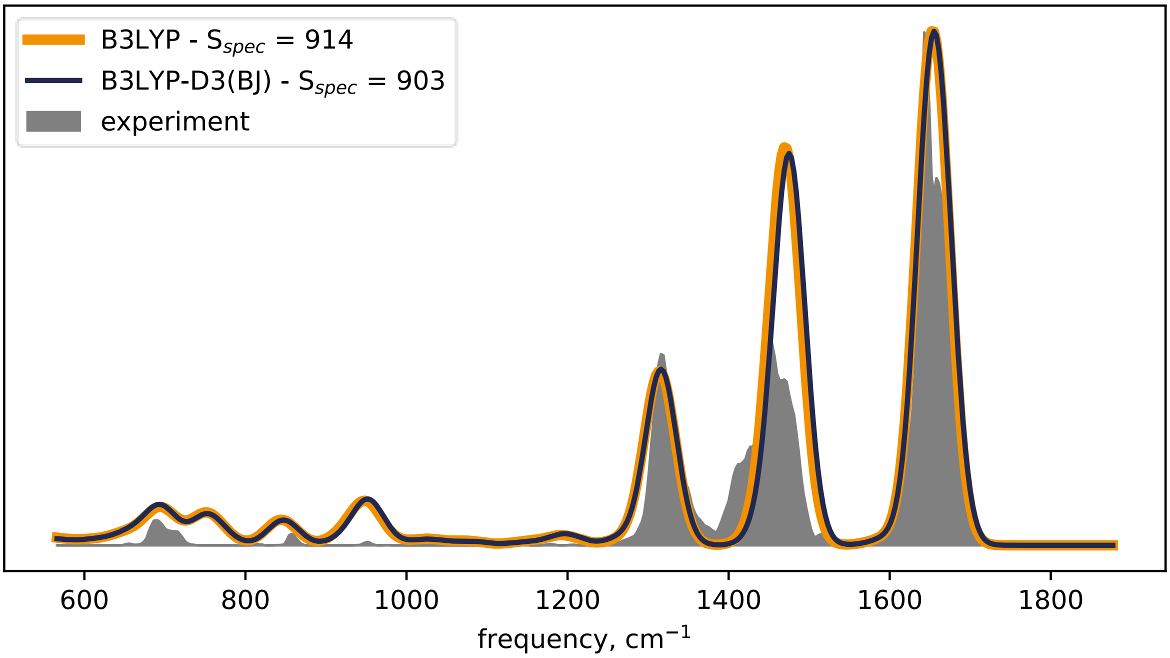

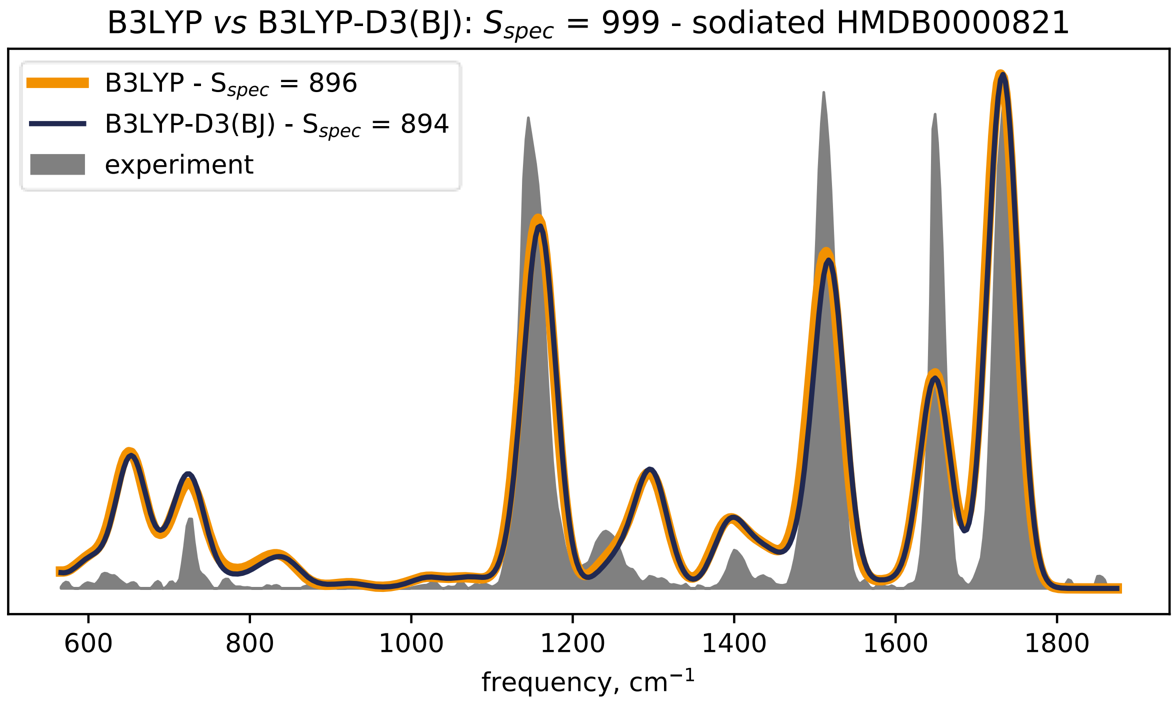
B3LYP vs B3LYP-D3(BJ): S_{spec} = 999 - sodiated HMDB0000821
B3LYP - S_{spec} = 896
B3LYP-D3(BJ) - S_{spec} = 894
experiment
600
800
1000
1200
1400
1600
1800
frequency, cm^{-1}

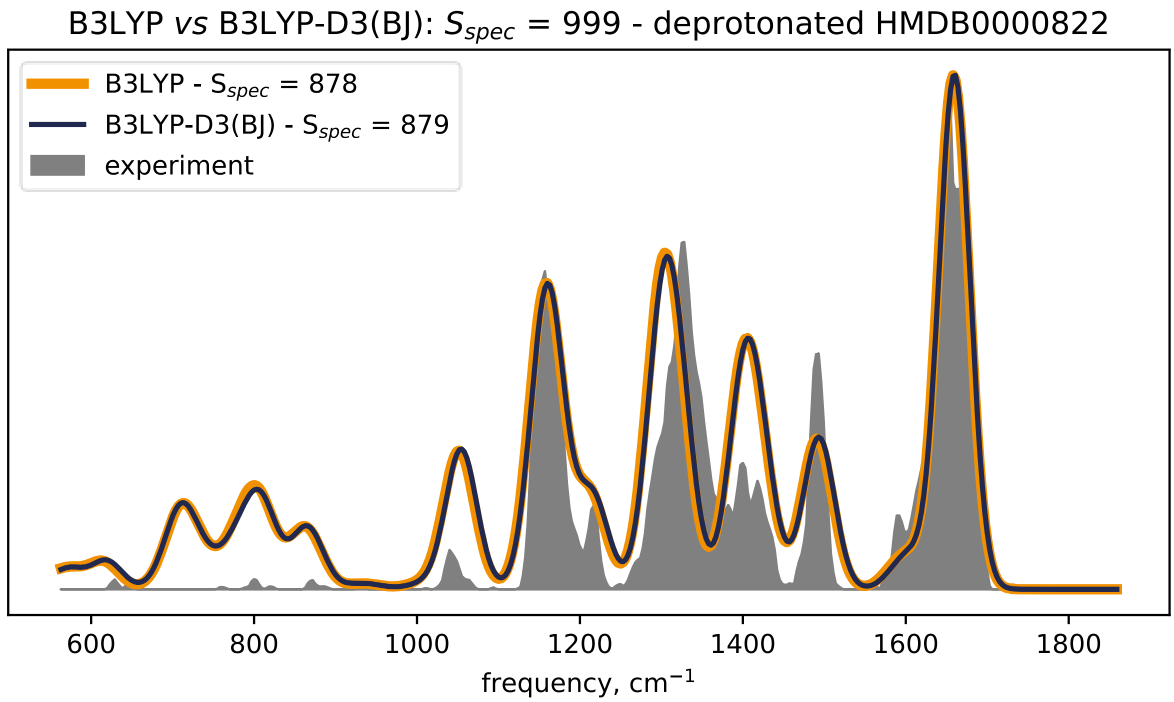
B3LYP vs B3LYP-D3(BJ): S_{spec} = 999 - deprotonated HMDB0000822
B3LYP - S_{spec} = 878
B3LYP-D3(BJ) - S_{spec} = 879
experiment
600
800
1000
1200
1400
1600
1800
frequency, cm^{-1}

B3LYP *vs* B3LYP-D3(BJ): S_{spec} = 971 - protonated HMDB0000828

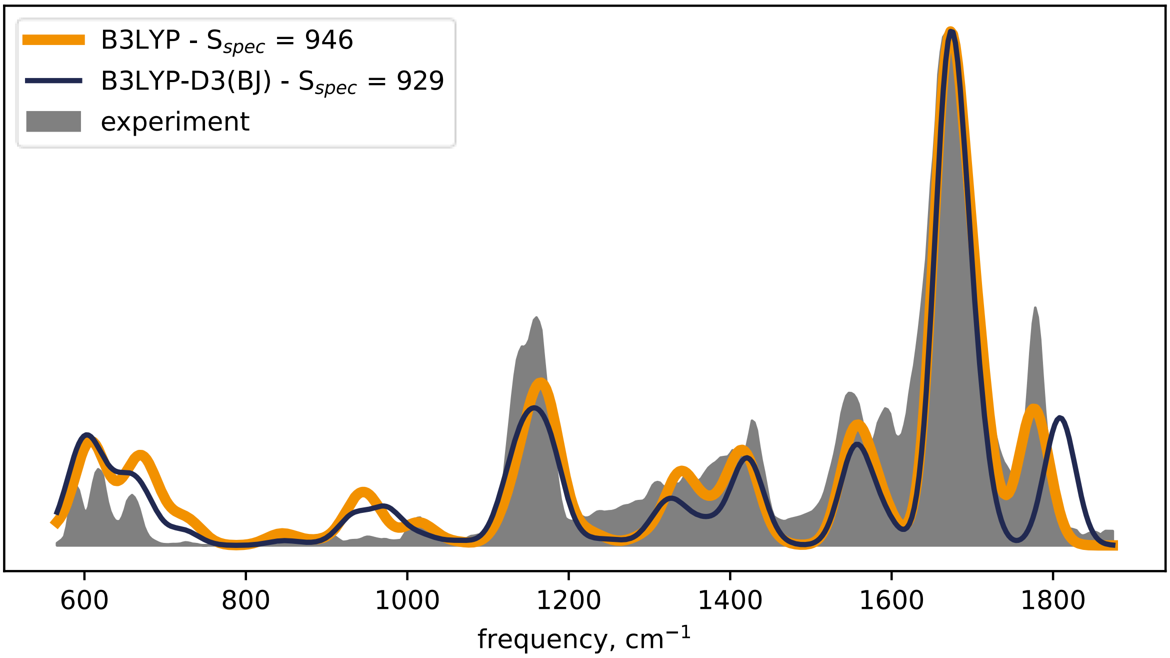

B3LYP *vs* B3LYP-D3(BJ): S_{spec} = 998 - sodiated HMDB0000828

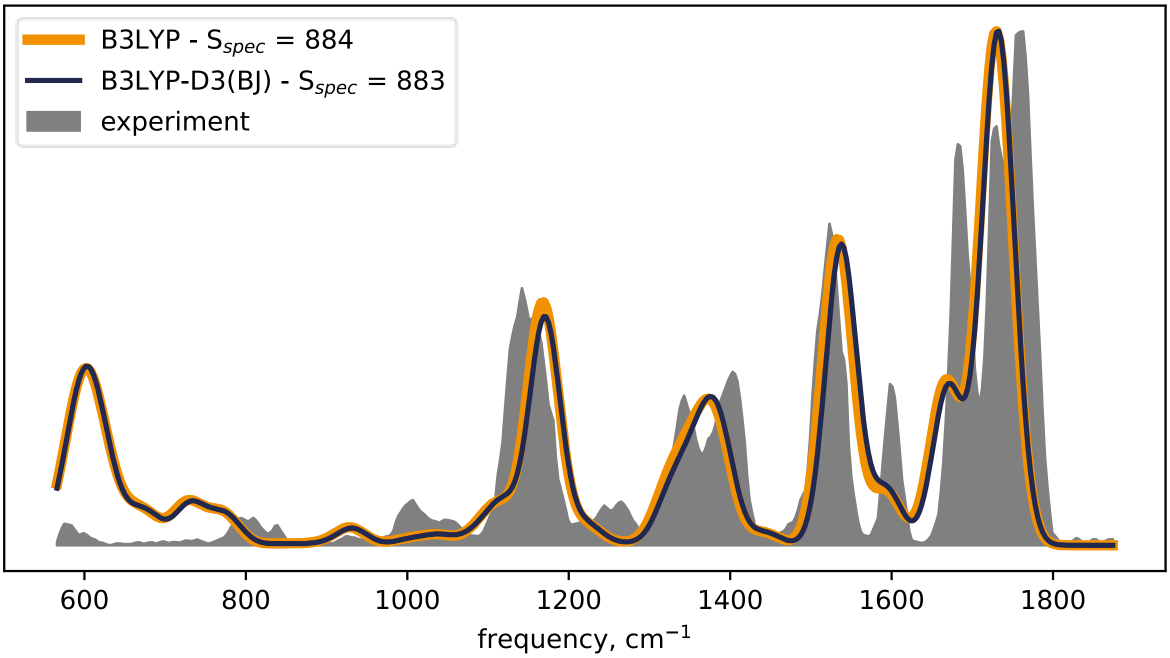

B3LYP *vs* B3LYP-D3(BJ): S_{spec} = 999 - deprotonated HMDB0000842

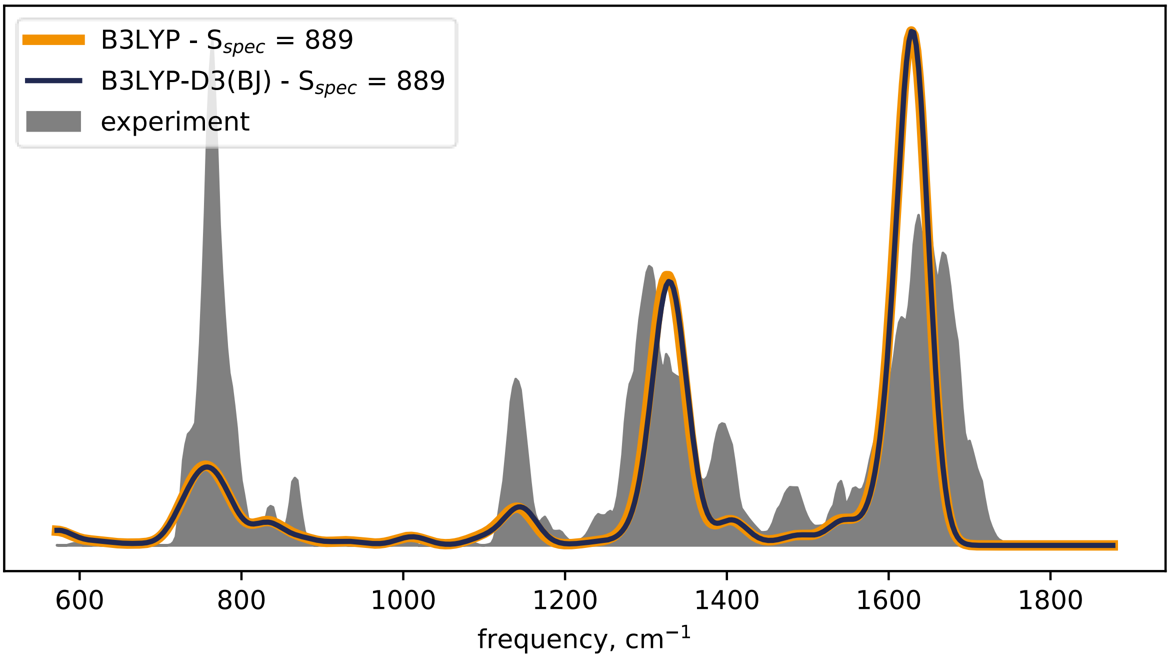

B3LYP *vs* B3LYP-D3(BJ): S_{spec} = 999 - protonated HMDB0000842

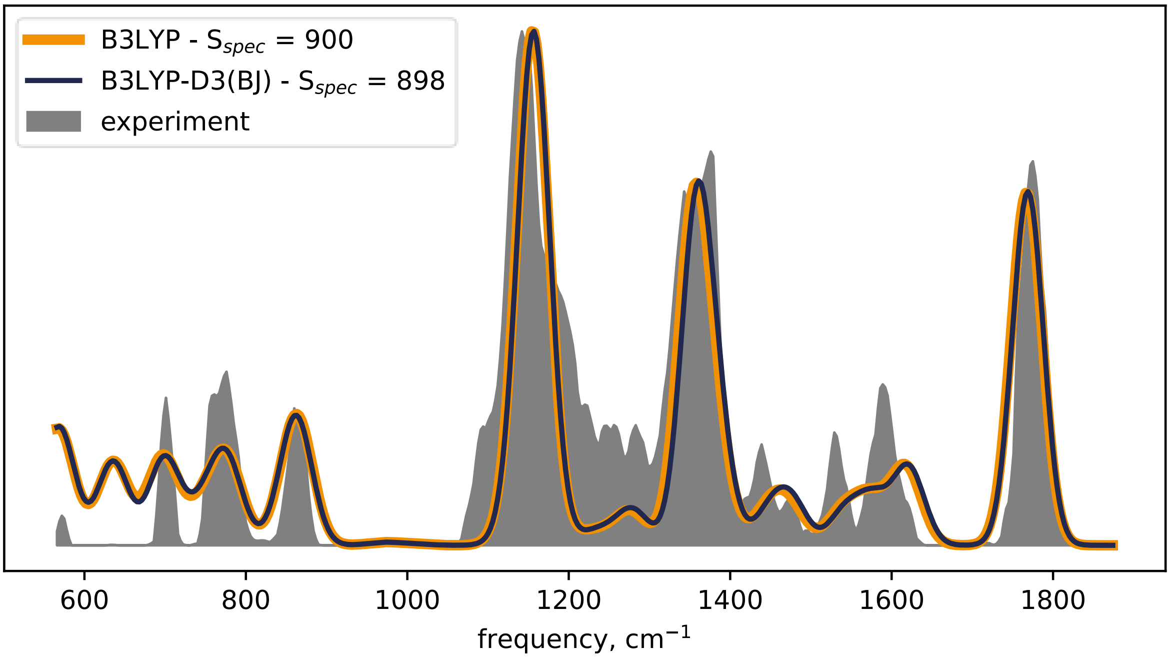

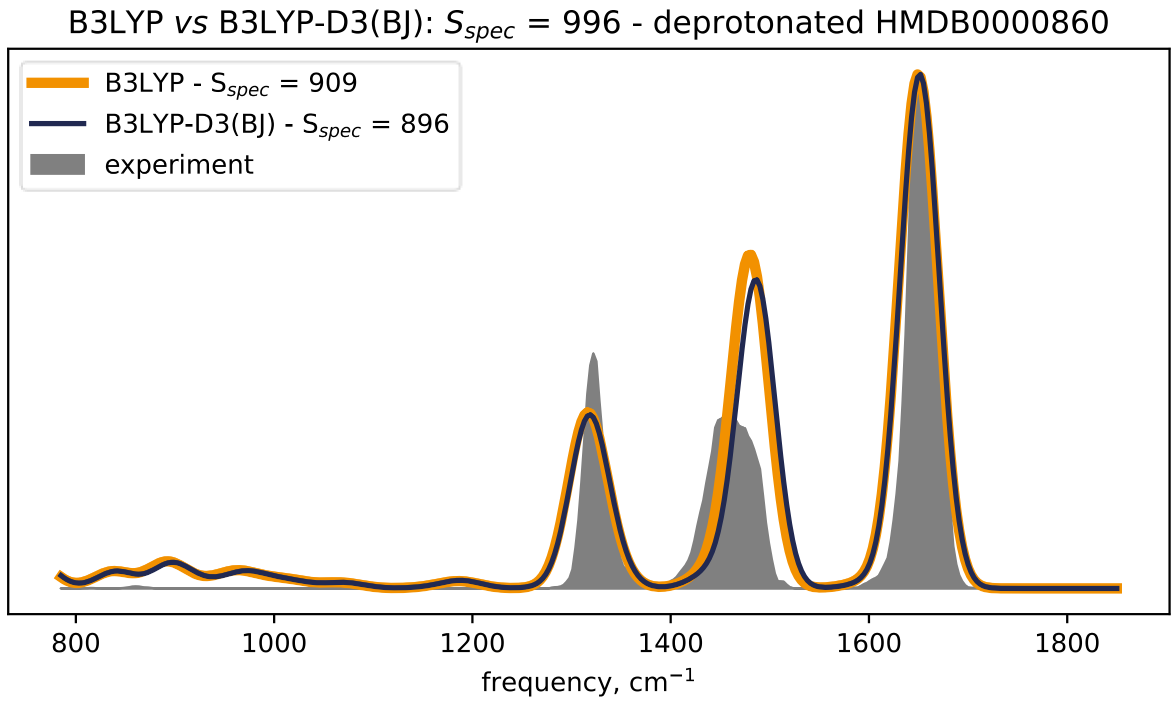

B3LYP vs B3LYP-D3(BJ): S_{spec} = 996 - deprotonated HMDB0000860
B3LYP - S_{spec} = 909
B3LYP-D3(BJ) - S_{spec} = 896
experiment
800
1000
1200
1400
1600
1800
frequency, cm^{-1}

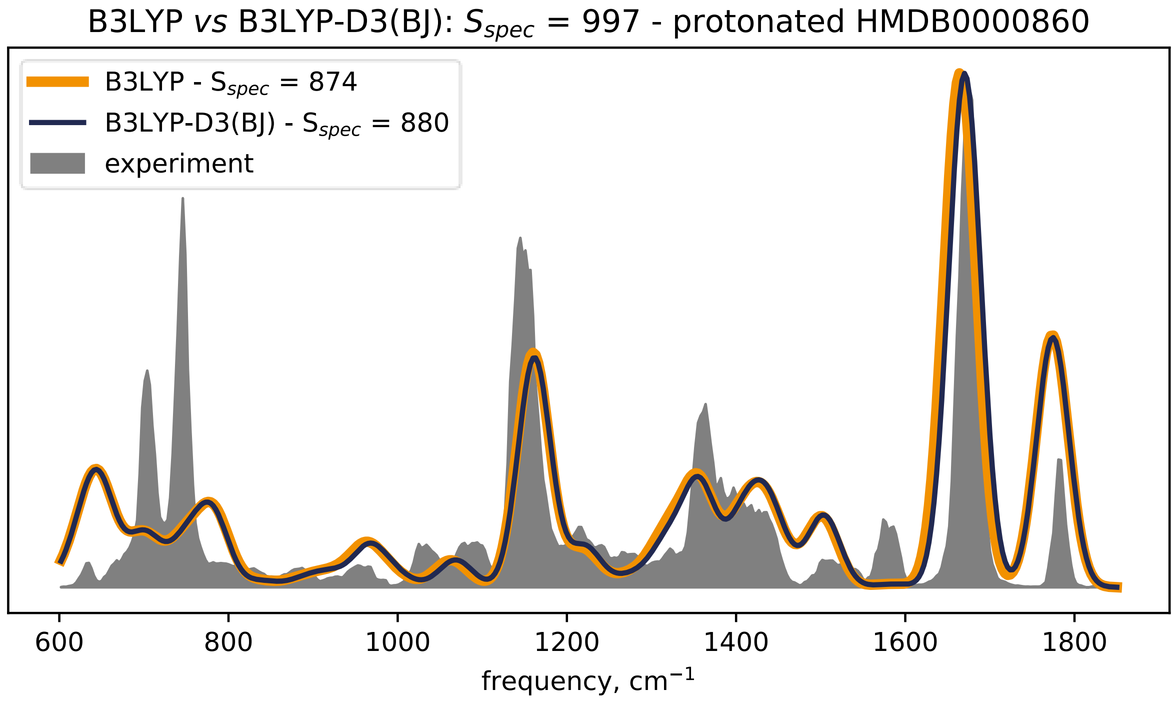

B3LYP vs B3LYP-D3(BJ): S_{spec} = 997 - protonated HMDB0000860
B3LYP - S_{spec} = 874
B3LYP-D3(BJ) - S_{spec} = 880
experiment
600
800
1000
1200
1400
1600
1800
frequency, cm^{-1}

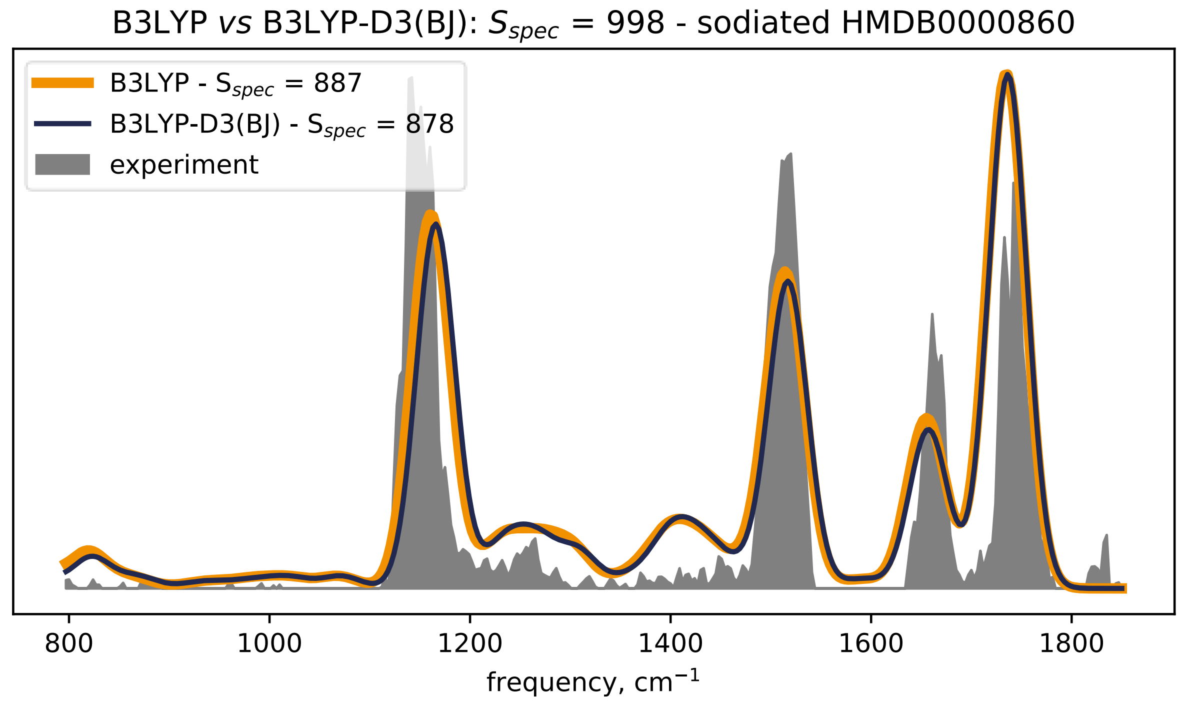
B3LYP vs B3LYP-D3(BJ): S_{spec} = 998 - sodiated HMDB0000860
B3LYP - S_{spec} = 887
B3LYP-D3(BJ) - S_{spec} = 878
experiment
800
1000
1200
1400
1600
1800
frequency, cm^{-1}

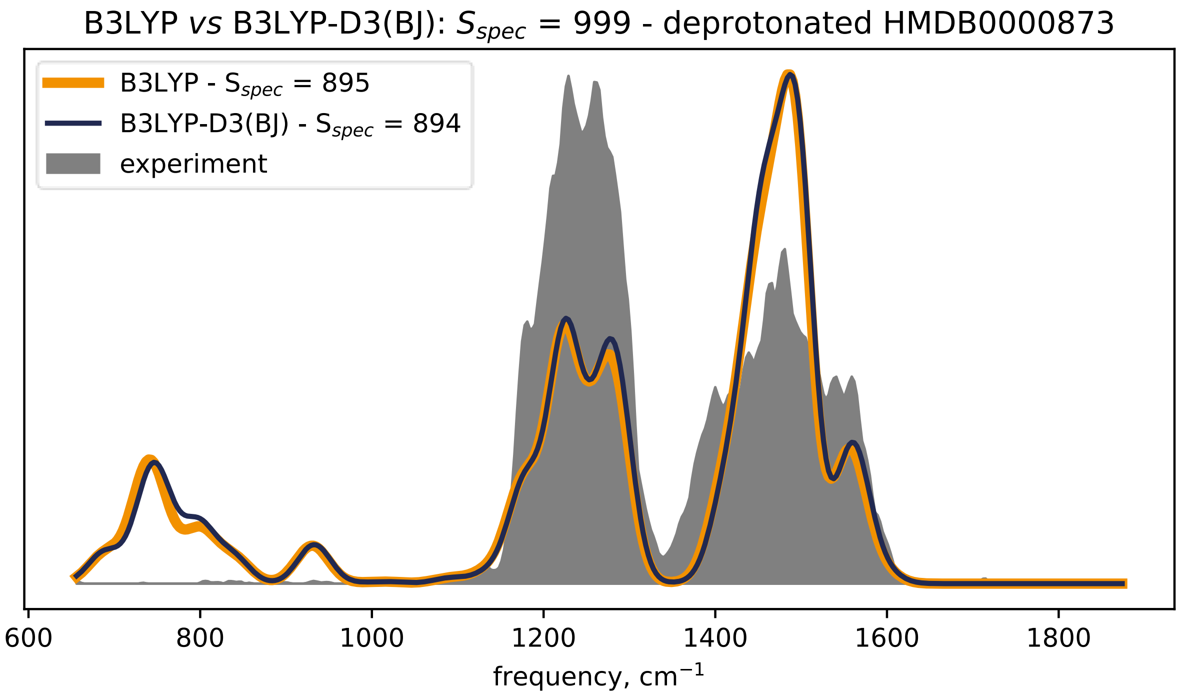
B3LYP vs B3LYP-D3(BJ): S_{spec} = 999 - deprotonated HMDB0000873
B3LYP - S_{spec} = 895
B3LYP-D3(BJ) - S_{spec} = 894
experiment
600
800
1000
1200
1400
1600
1800
frequency, cm^{-1}

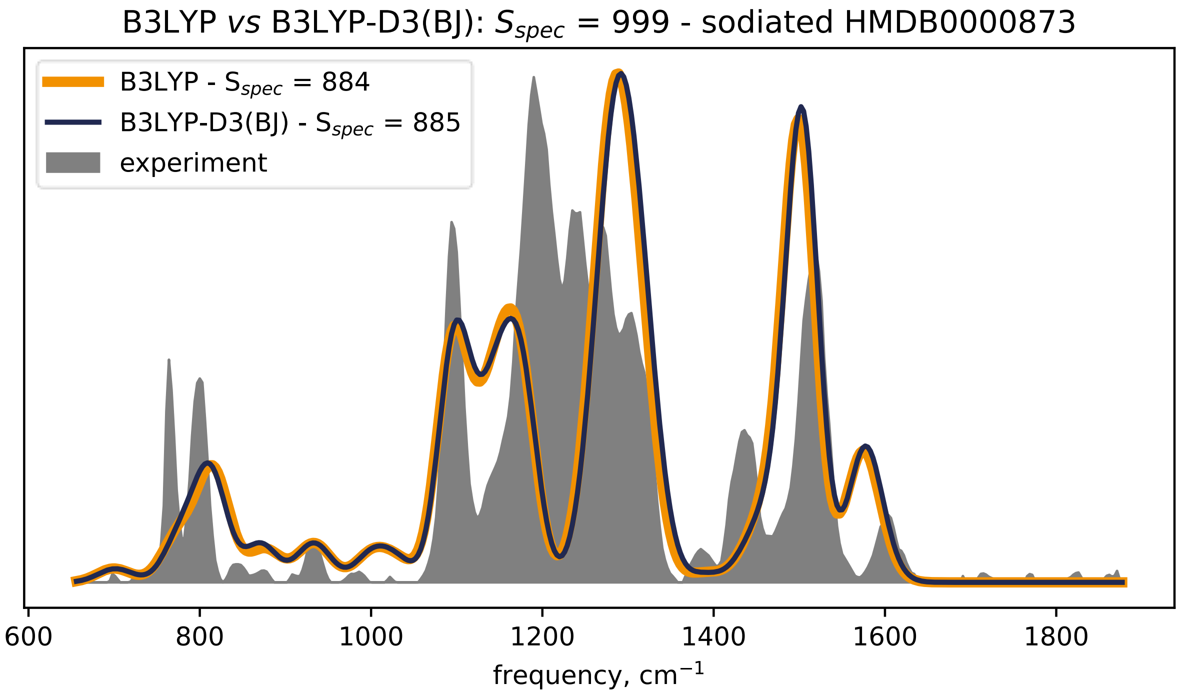
B3LYP vs B3LYP-D3(BJ): S_{spec} = 999 - sodiated HMDB0000873
B3LYP - S_{spec} = 884
B3LYP-D3(BJ) - S_{spec} = 885
experiment
600
800
1000
1200
1400
1600
1800
frequency, cm^{-1}

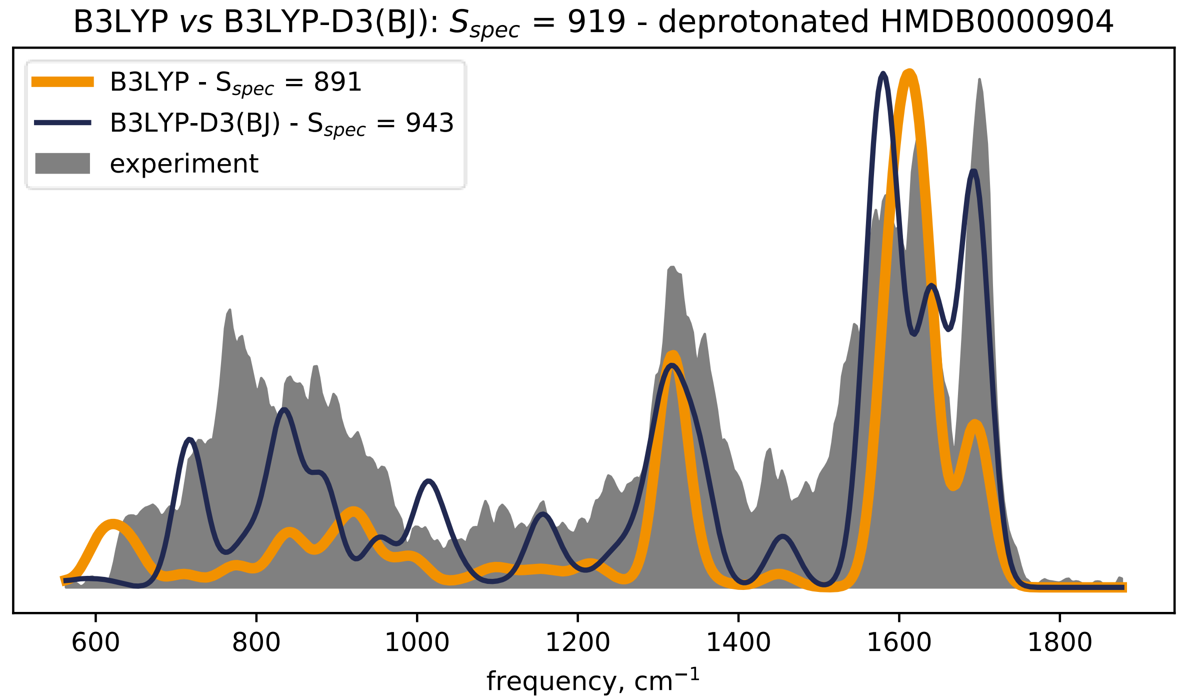
B3LYP vs B3LYP-D3(BJ): S_{spec} = 919 - deprotonated HMDB0000904
B3LYP - S_{spec} = 891
B3LYP-D3(BJ) - S_{spec} = 943
experiment
600
800
1000
1200
1400
1600
1800
frequency, cm^{-1}

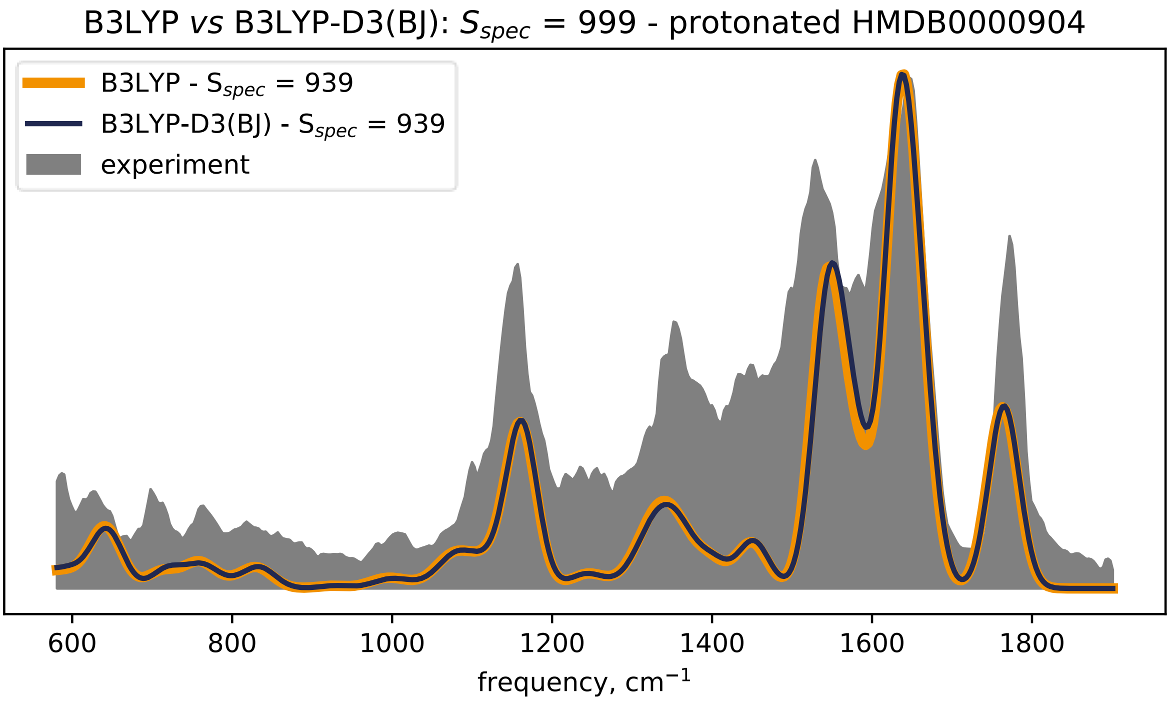
B3LYP vs B3LYP-D3(BJ): S_{spec} = 999 - protonated HMDB0000904
B3LYP - S_{spec} = 939
B3LYP-D3(BJ) - S_{spec} = 939
experiment
600
800
1000
1200
1400
1600
1800
frequency, cm^{-1}

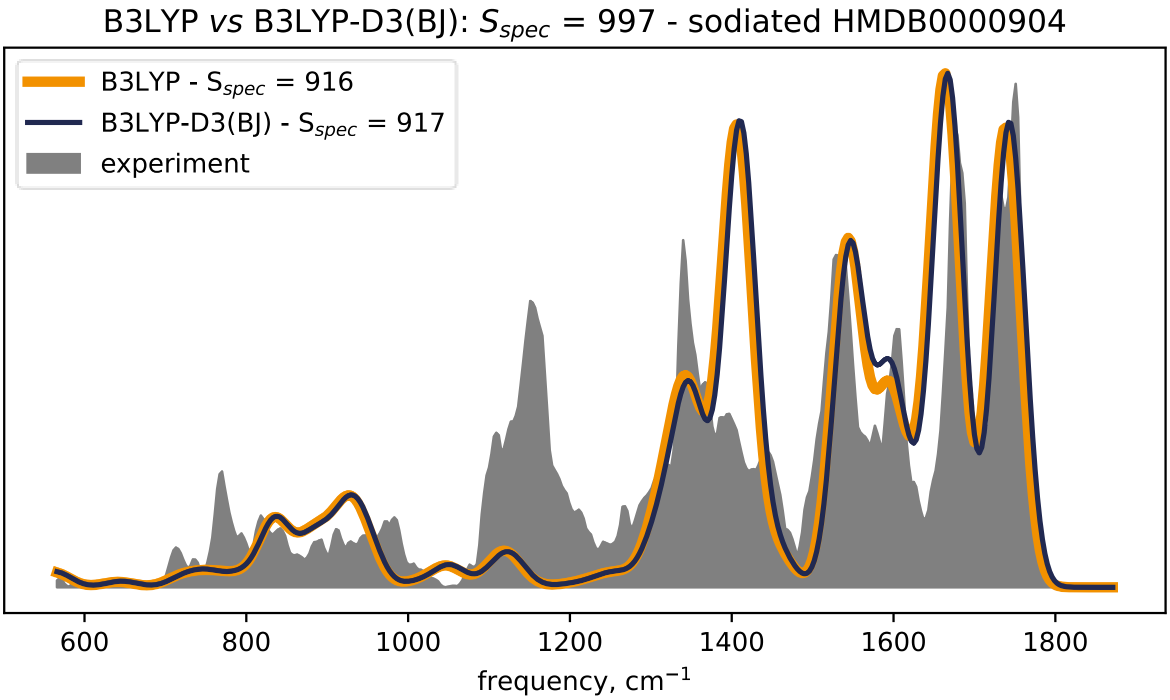
B3LYP vs B3LYP-D3(BJ): S_{spec} = 997 - sodiated HMDB0000904
B3LYP - S_{spec} = 916
B3LYP-D3(BJ) - S_{spec} = 917
experiment
600
800
1000
1200
1400
1600
1800
frequency, cm^{-1}

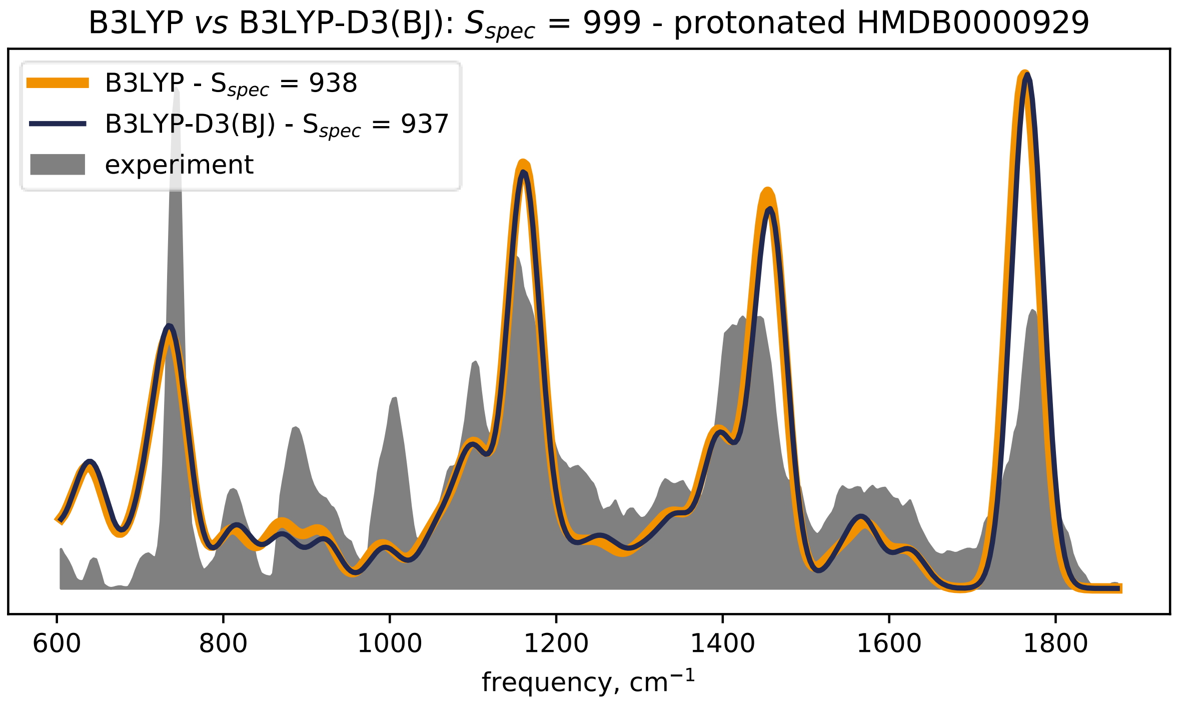

B3LYP vs B3LYP-D3(BJ): S_{spec} = 999 - protonated HMDB0000929
B3LYP - S_{spec} = 938
B3LYP-D3(BJ) - S_{spec} = 937
experiment
600
800
1000
1200
1400
1600
1800
frequency, cm^{-1}

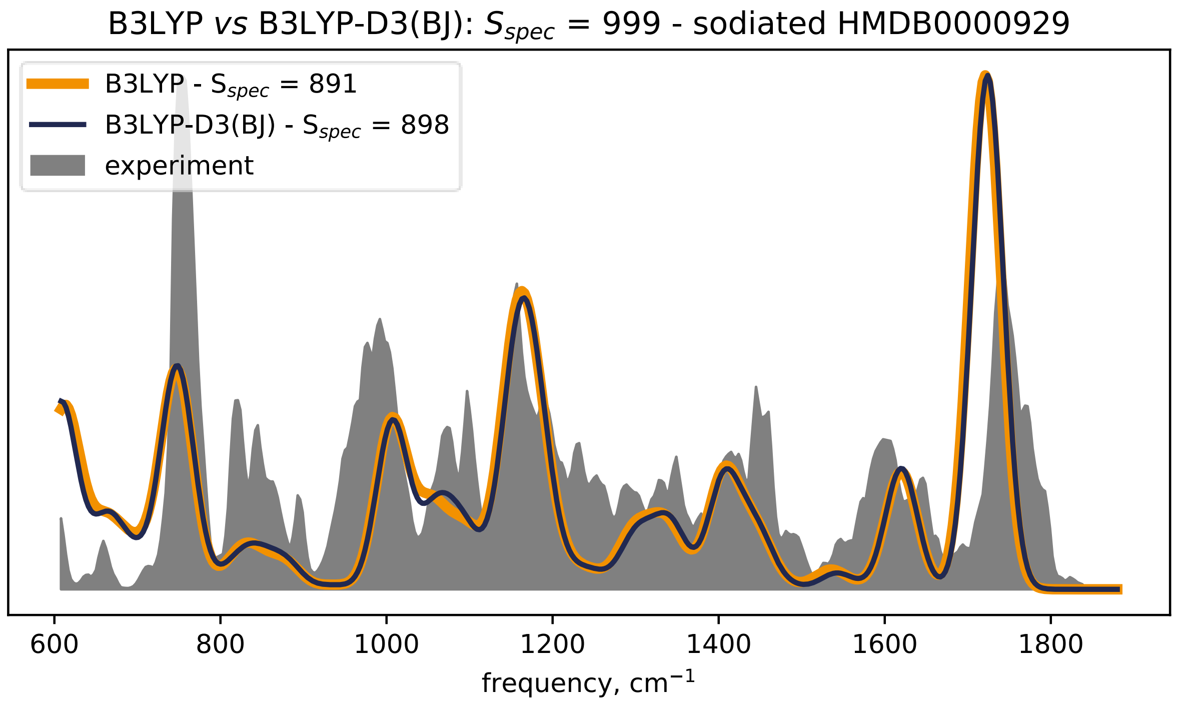

B3LYP vs B3LYP-D3(BJ): S_{spec} = 999 - sodiated HMDB0000929
B3LYP - S_{spec} = 891
B3LYP-D3(BJ) - S_{spec} = 898
experiment
600
800
1000
1200
1400
1600
1800
frequency, cm^{-1}

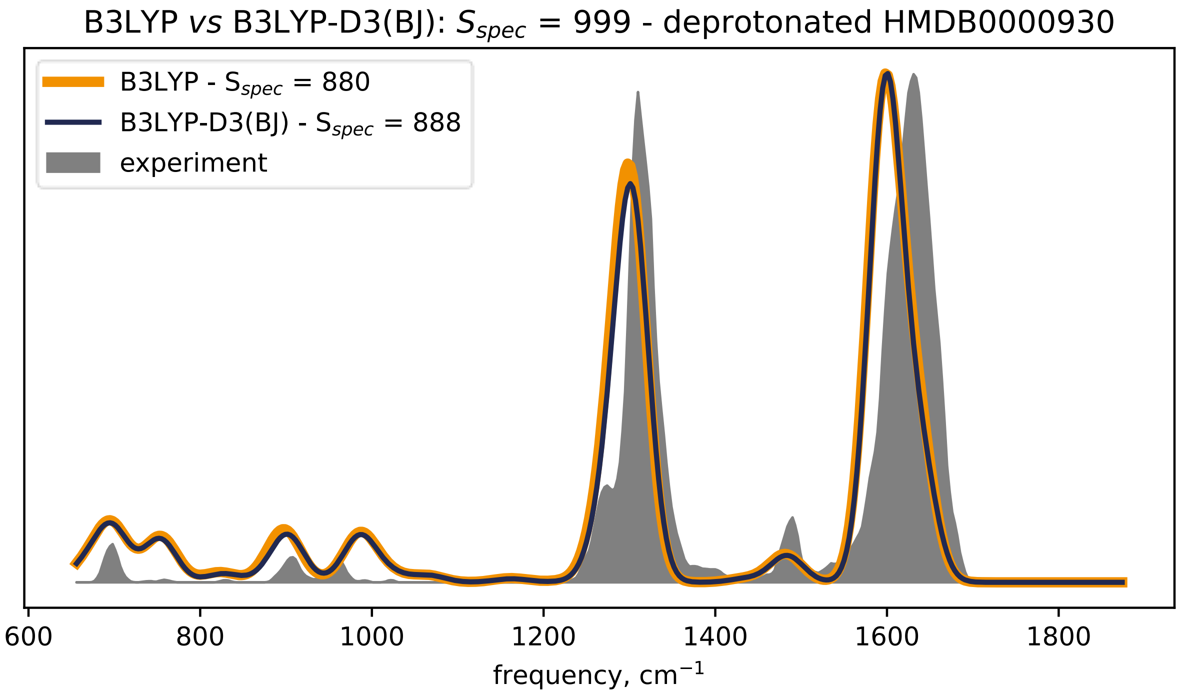
B3LYP vs B3LYP-D3(BJ): S_{spec} = 999 - deprotonated HMDB0000930
B3LYP - S_{spec} = 880
B3LYP-D3(BJ) - S_{spec} = 888
experiment
600
800
1000
1200
1400
1600
1800
frequency, cm^{-1}

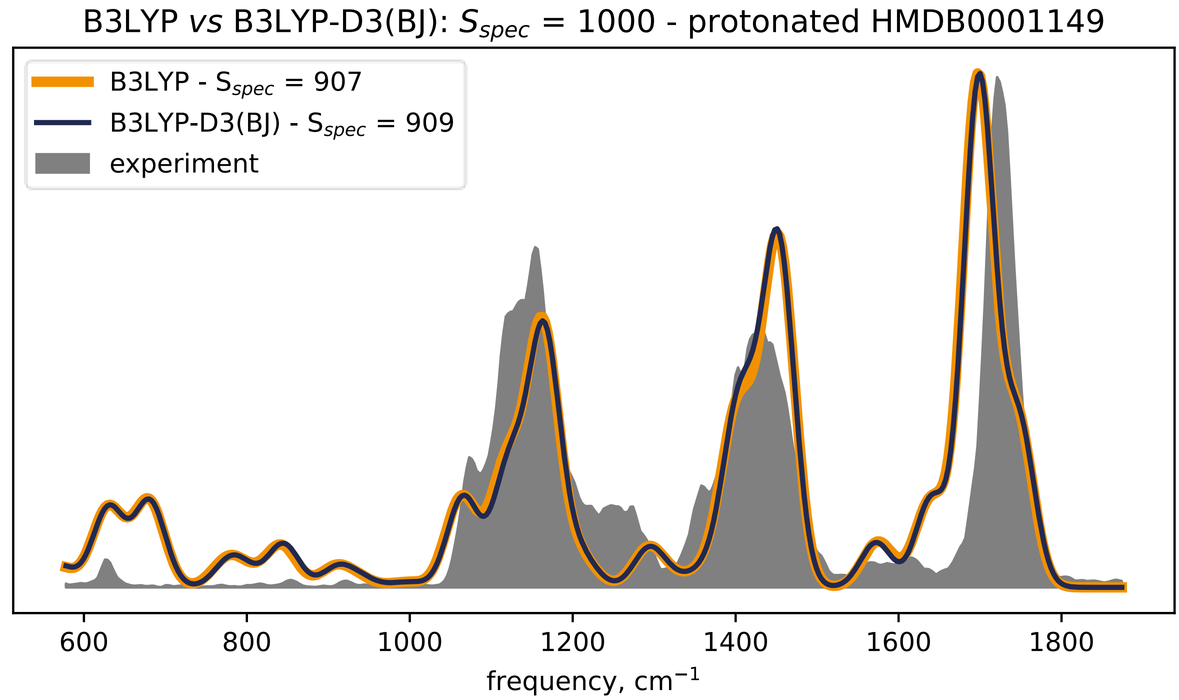
B3LYP vs B3LYP-D3(BJ): S_{spec} = 1000 - protonated HMDB0001149
B3LYP - S_{spec} = 907
B3LYP-D3(BJ) - S_{spec} = 909
experiment
600
800
1000
1200
1400
1600
1800
frequency, cm^{-1}

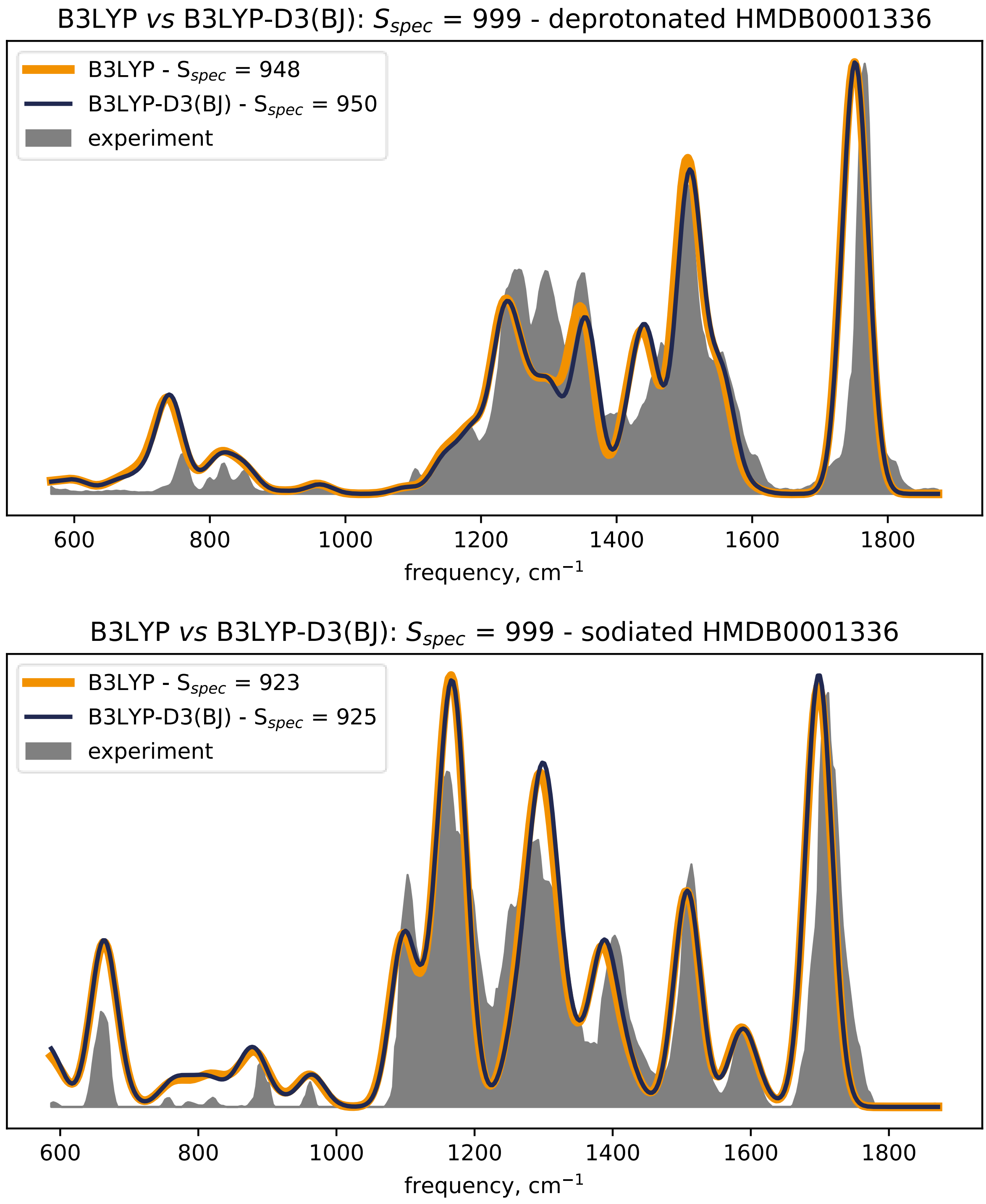

B3LYP vs B3LYP-D3(BJ): S_{spec} = 999 - deprotonated HMDB0001336
B3LYP - S_{spec} = 948
B3LYP-D3(BJ) - S_{spec} = 950
experiment
600
800
1000
1200
1400
1600
1800
frequency, cm^{-1}
B3LYP vs B3LYP-D3(BJ): S_{spec} = 999 - sodiated HMDB0001336
B3LYP - S_{spec} = 923
B3LYP-D3(BJ) - S_{spec} = 925
experiment
600
800
1000
1200
1400
1600
1800
frequency, cm^{-1}

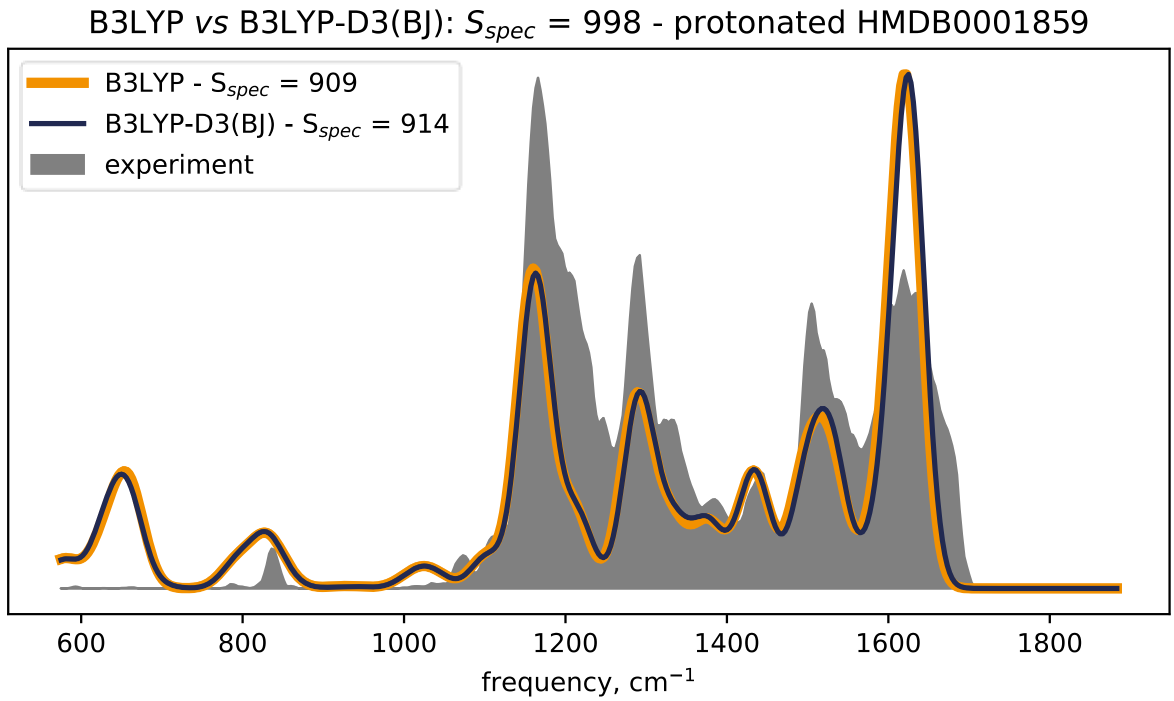
B3LYP vs B3LYP-D3(BJ): S_{spec} = 998 - protonated HMDB0001859
B3LYP - S_{spec} = 909
B3LYP-D3(BJ) - S_{spec} = 914
experiment
600
800
1000
1200
1400
1600
1800
frequency, cm^{-1}

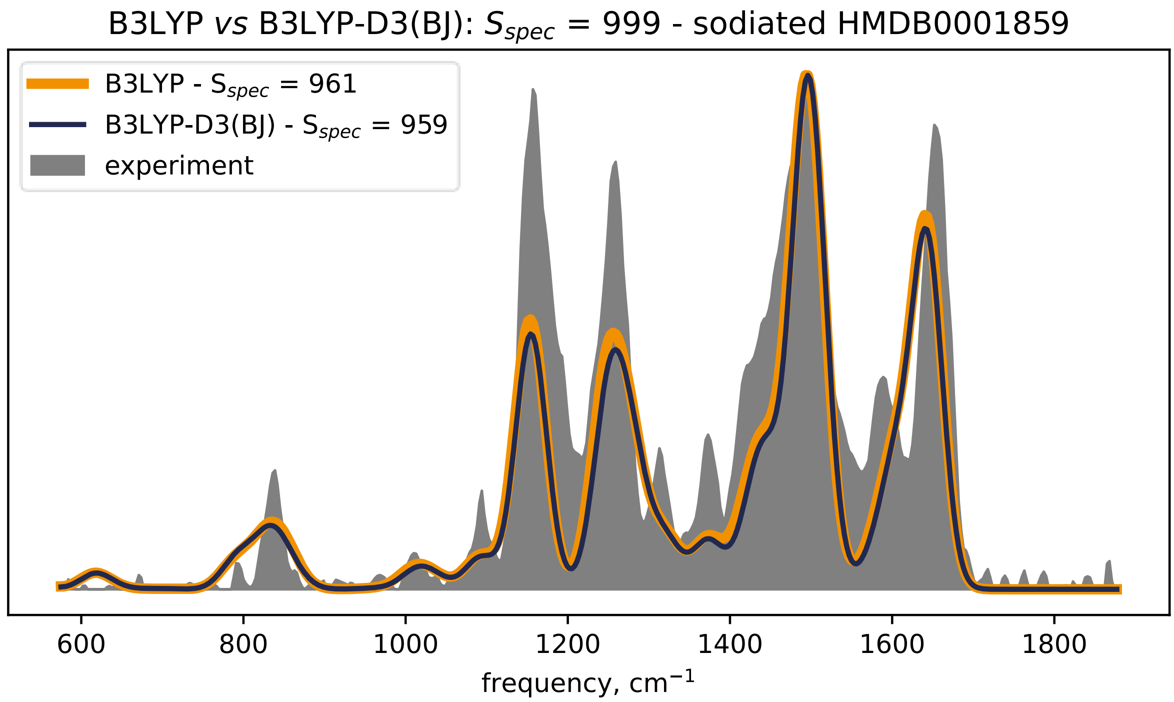
B3LYP vs B3LYP-D3(BJ): S_{spec} = 999 - sodiated HMDB0001859
B3LYP - S_{spec} = 961
B3LYP-D3(BJ) - S_{spec} = 959
experiment
600
800
1000
1200
1400
1600
1800
frequency, cm^{-1}

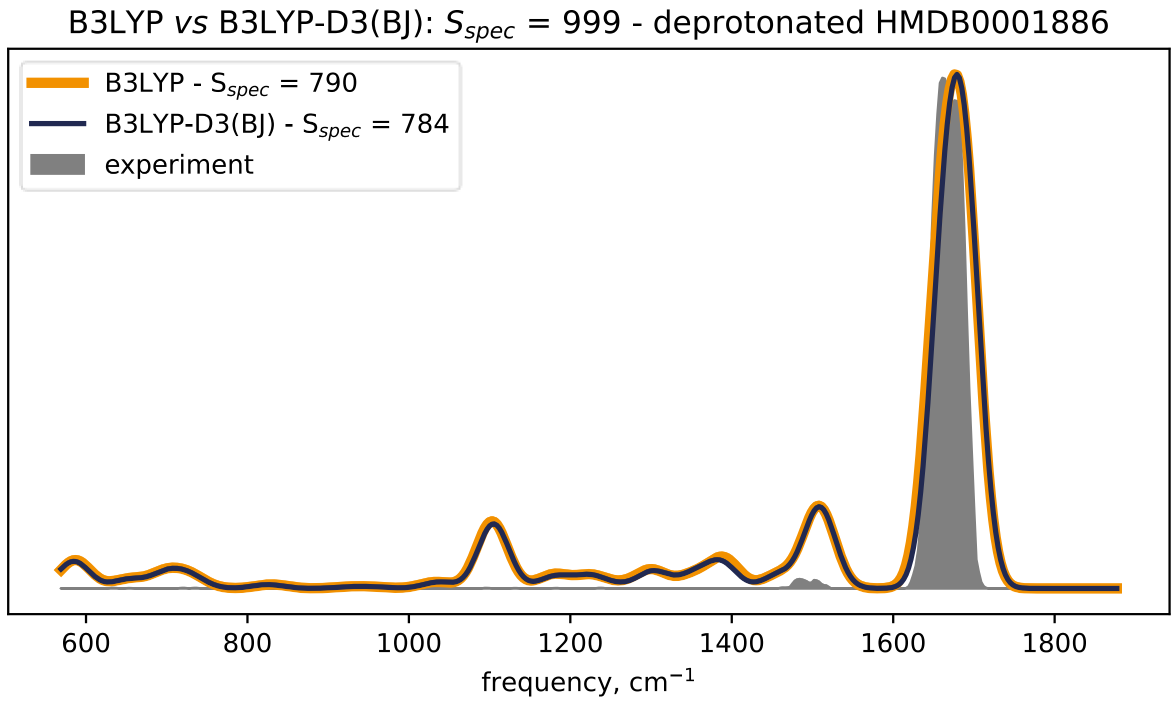
B3LYP vs B3LYP-D3(BJ): S_{spec} = 999 - deprotonated HMDB0001886
B3LYP - S_{spec} = 790
B3LYP-D3(BJ) - S_{spec} = 784
experiment
600
800
1000
1200
1400
1600
1800
frequency, cm^{-1}

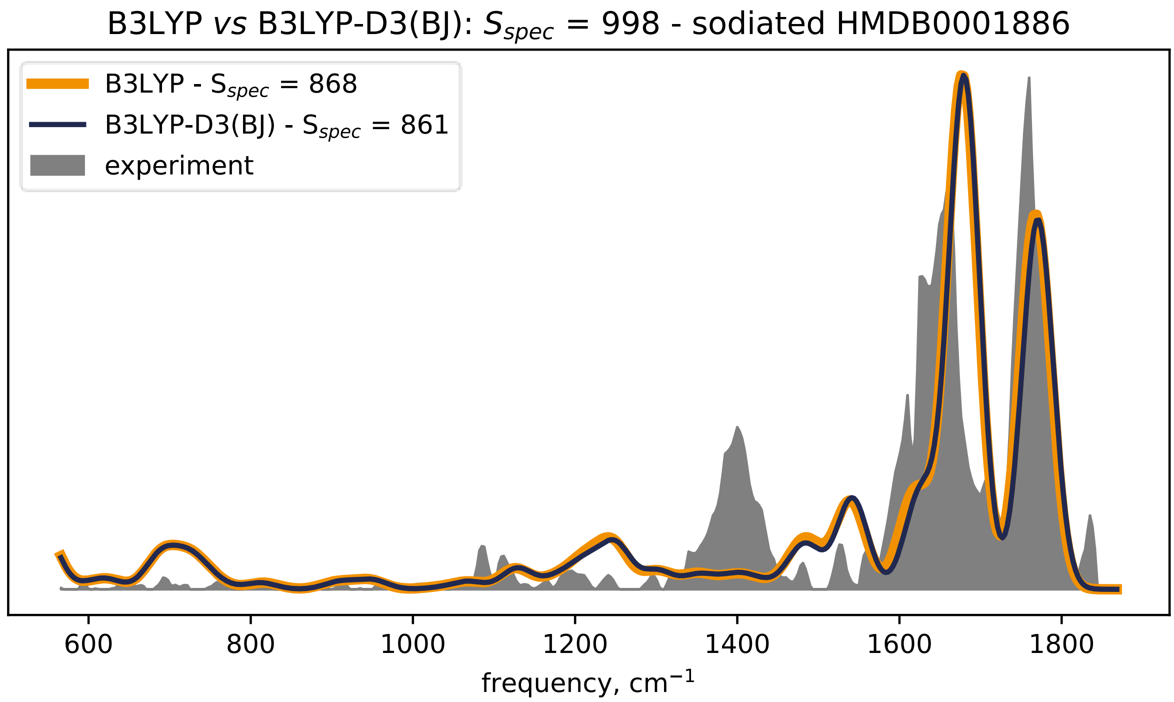
B3LYP vs B3LYP-D3(BJ): S_{spec} = 998 - sodiated HMDB0001886
B3LYP - S_{spec} = 868
B3LYP-D3(BJ) - S_{spec} = 861
experiment
600
800
1000
1200
1400
1600
1800
frequency, cm^{-1}

B3LYP *vs* B3LYP-D3(BJ): S_{spec} = 997 - deprotonated HMDB0001890

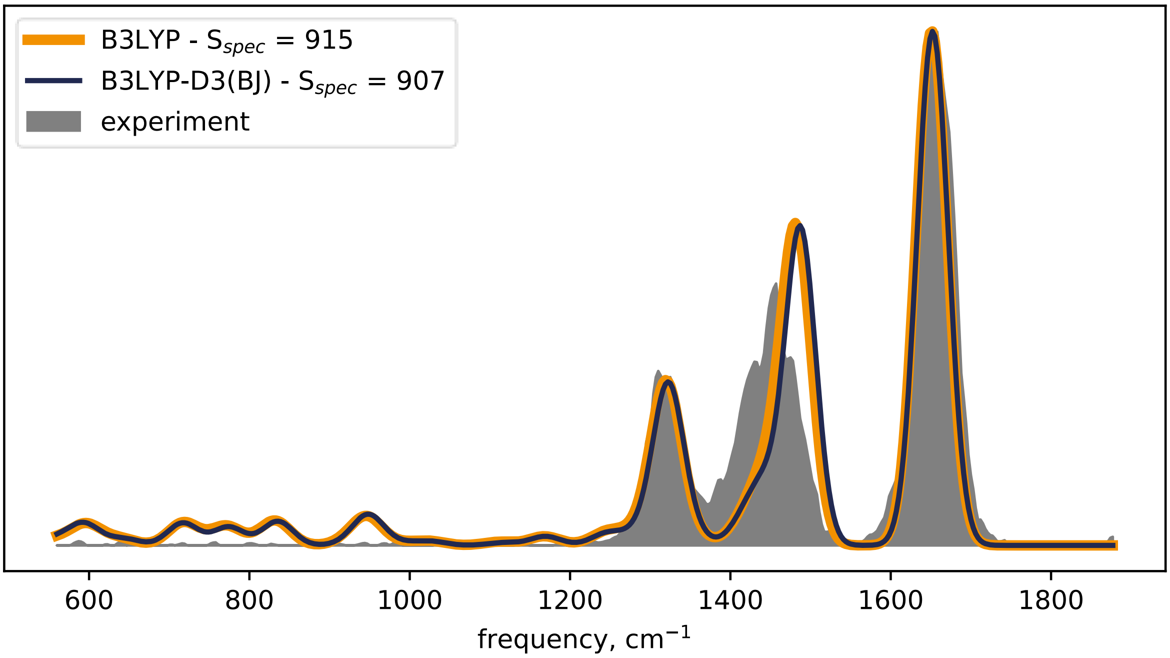

B3LYP *vs* B3LYP-D3(BJ): S_{spec} = 995 - sodiated HMDB0001890

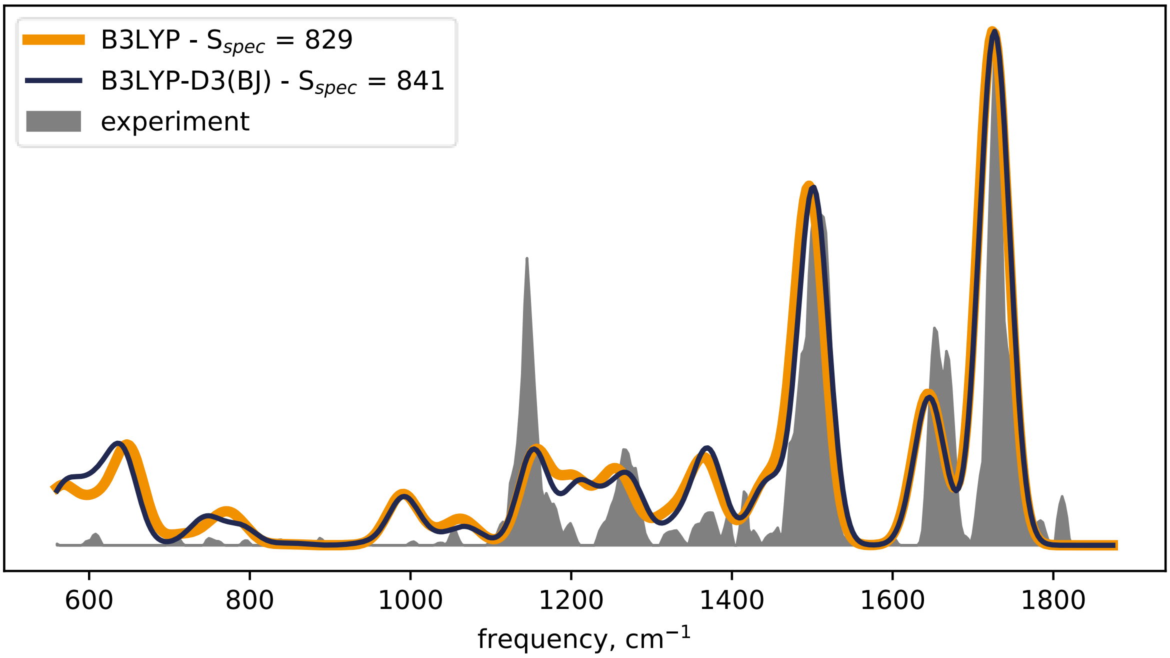

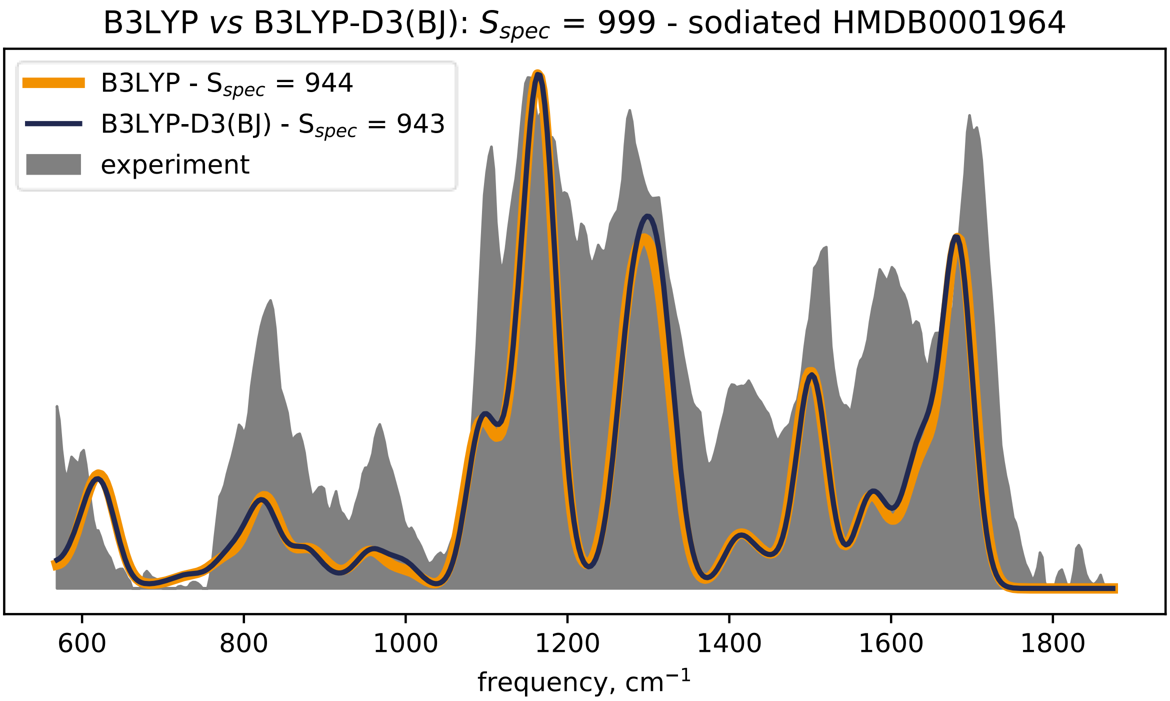
B3LYP vs B3LYP-D3(BJ): S_{spec} = 999 - sodiated HMDB0001964
B3LYP - S_{spec} = 944
B3LYP-D3(BJ) - S_{spec} = 943
experiment
600
800
1000
1200
1400
1600
1800
frequency, cm^{-1}

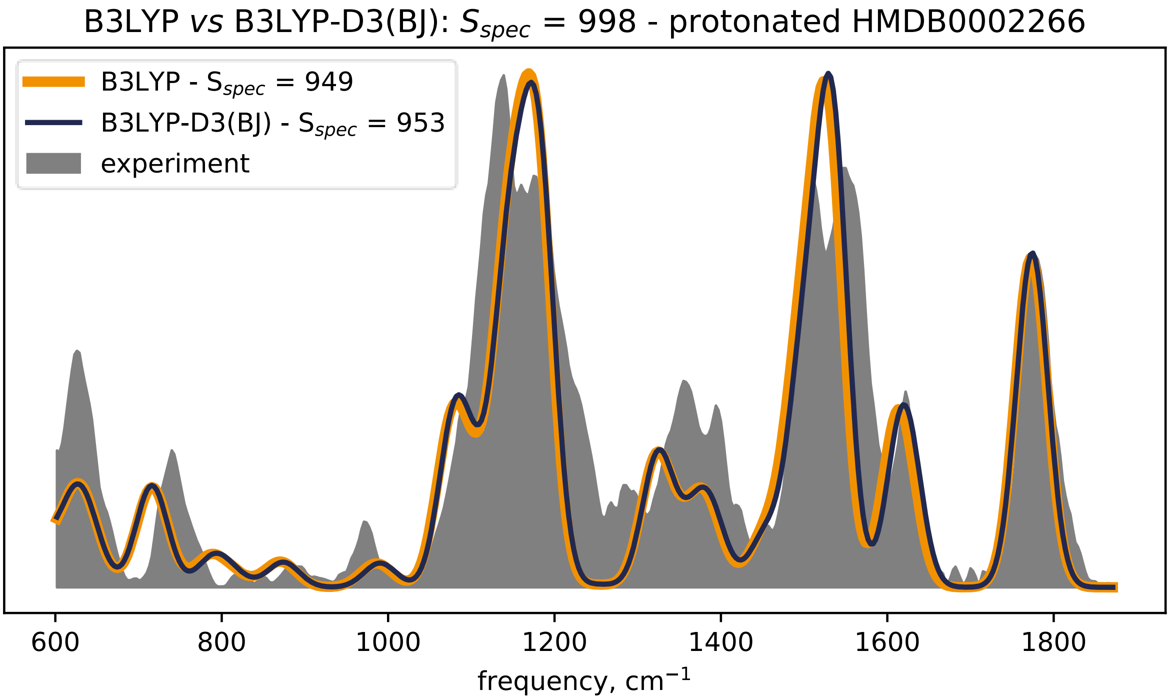
B3LYP vs B3LYP-D3(BJ): S_{spec} = 998 - protonated HMDB0002266
B3LYP - S_{spec} = 949
B3LYP-D3(BJ) - S_{spec} = 953
experiment
600
800
1000
1200
1400
1600
1800
frequency, cm^{-1}

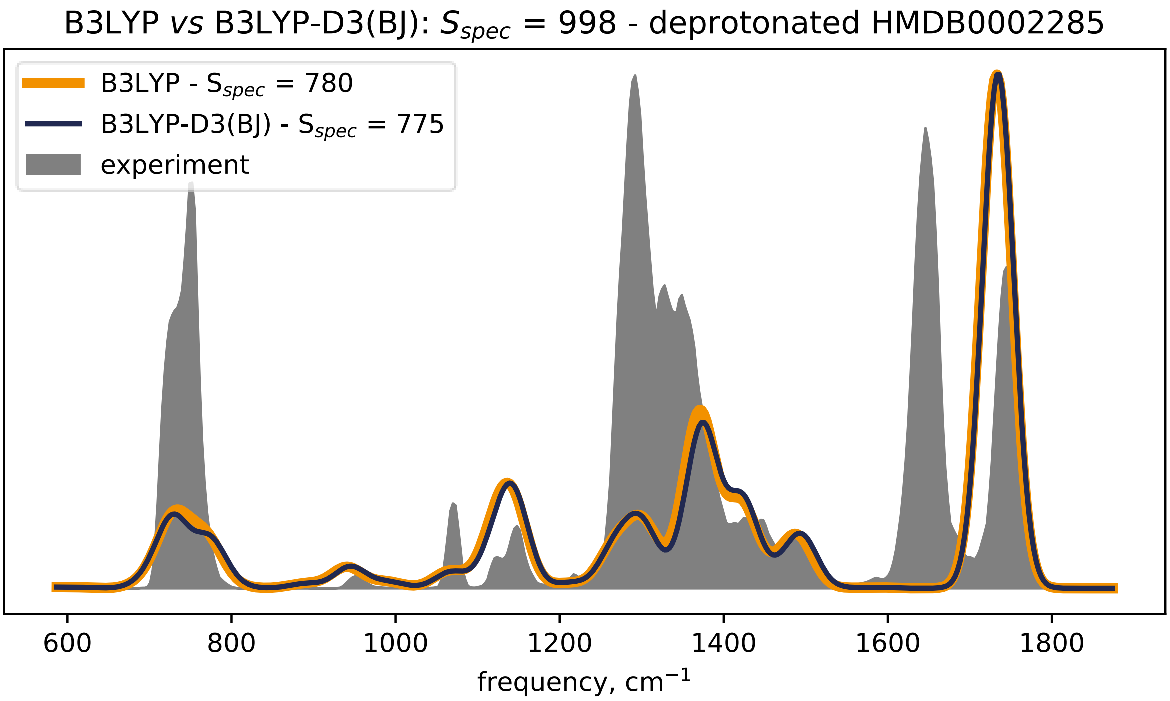

B3LYP vs B3LYP-D3(BJ): S_{spec} = 998 - deprotonated HMDB0002285
B3LYP - S_{spec} = 780
B3LYP-D3(BJ) - S_{spec} = 775
experiment
600
800
1000
1200
1400
1600
1800
frequency, cm^{-1}

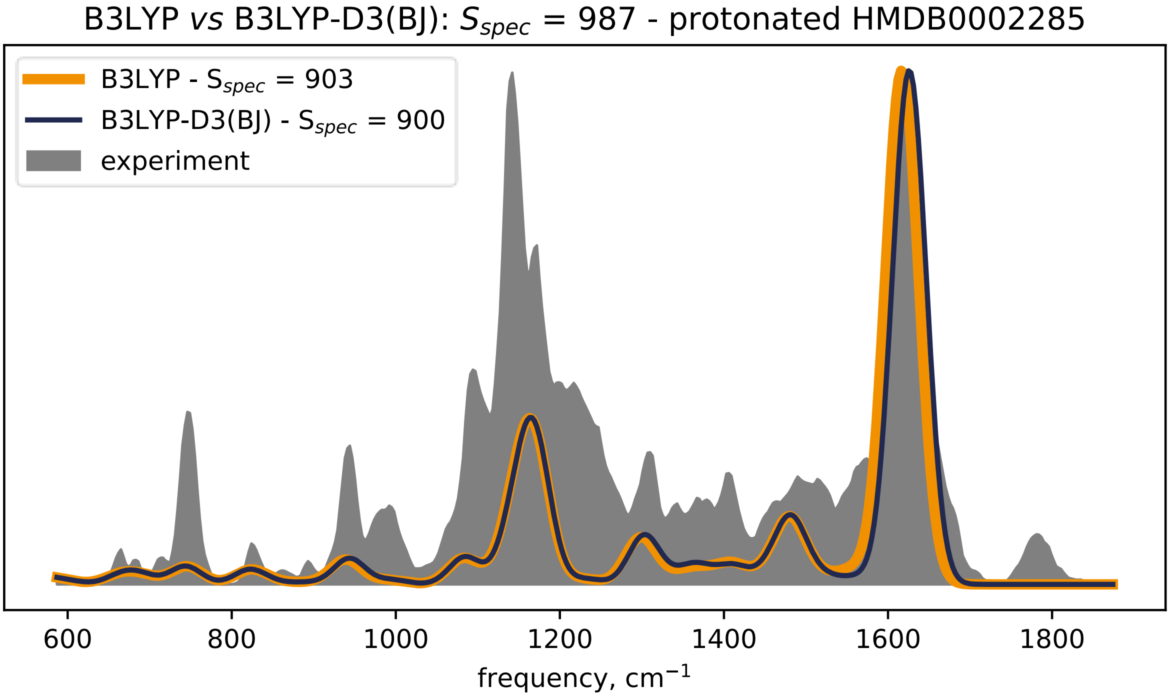

B3LYP vs B3LYP-D3(BJ): S_{spec} = 987 - protonated HMDB0002285
B3LYP - S_{spec} = 903
B3LYP-D3(BJ) - S_{spec} = 900
experiment
600
800
1000
1200
1400
1600
1800
frequency, cm^{-1}

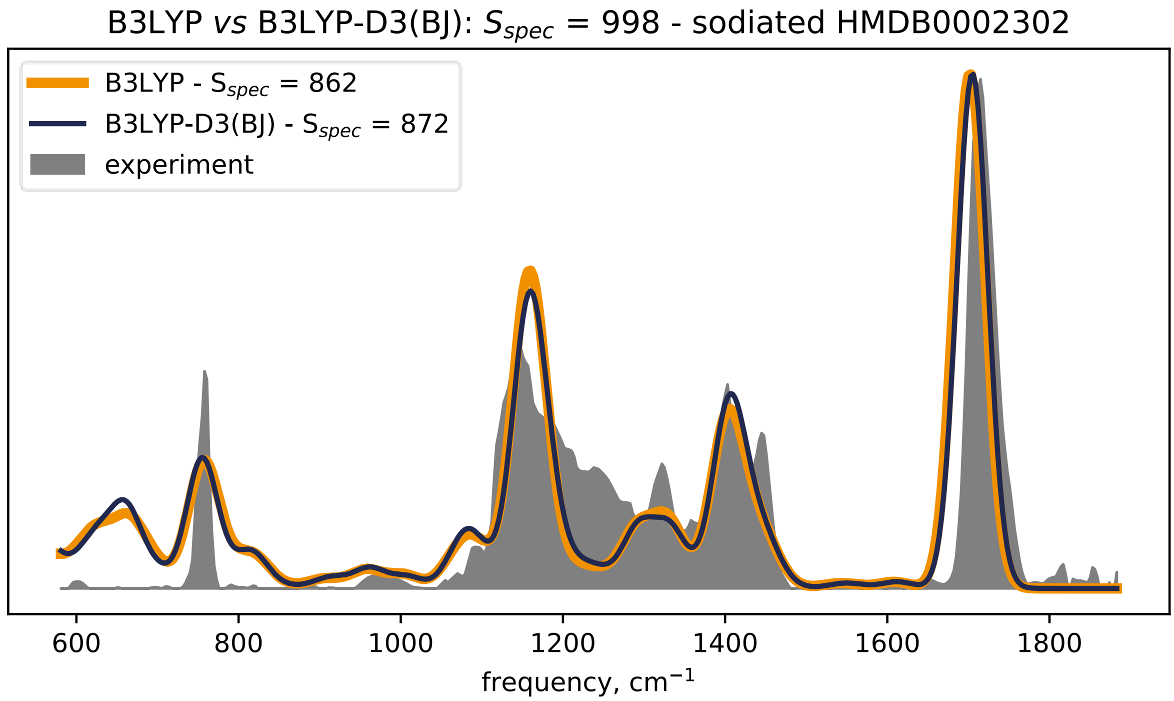
B3LYP vs B3LYP-D3(BJ): S_{spec} = 998 - sodiated HMDB0002302
B3LYP - S_{spec} = 862
B3LYP-D3(BJ) - S_{spec} = 872
experiment
600
800
1000
1200
1400
1600
1800
frequency, cm^{-1}

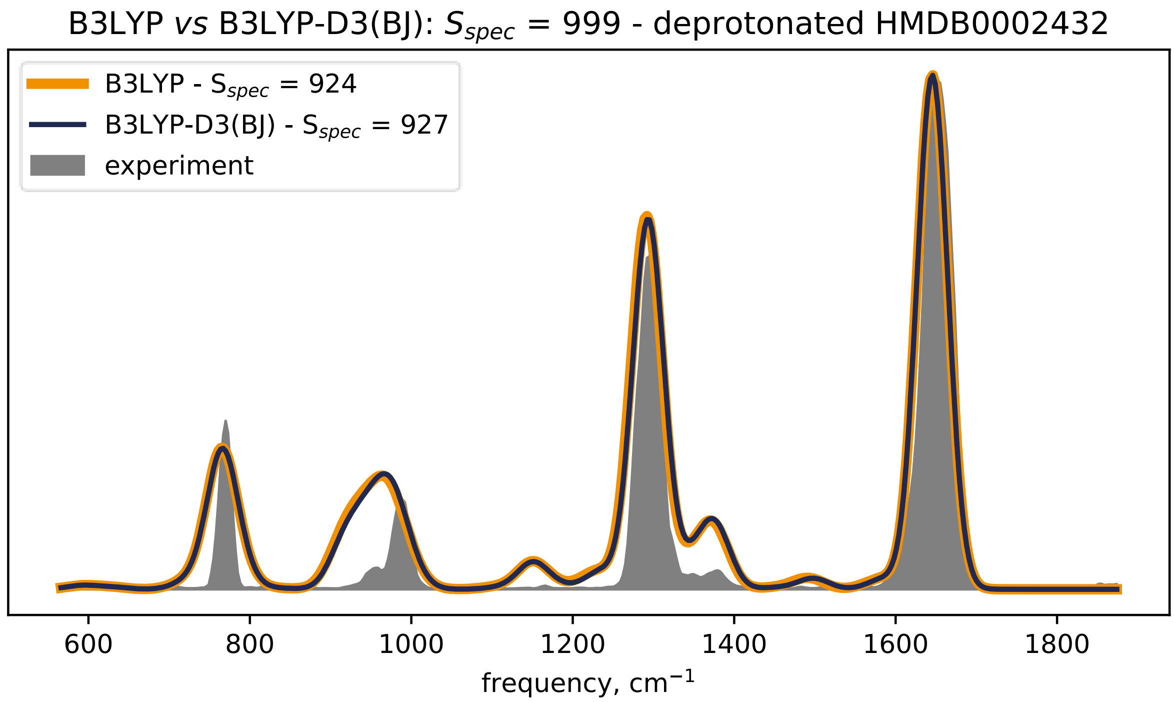
B3LYP vs B3LYP-D3(BJ): S_{spec} = 999 - deprotonated HMDB0002432
B3LYP - S_{spec} = 924
B3LYP-D3(BJ) - S_{spec} = 927
experiment
600
800
1000
1200
1400
1600
1800
frequency, cm^{-1}

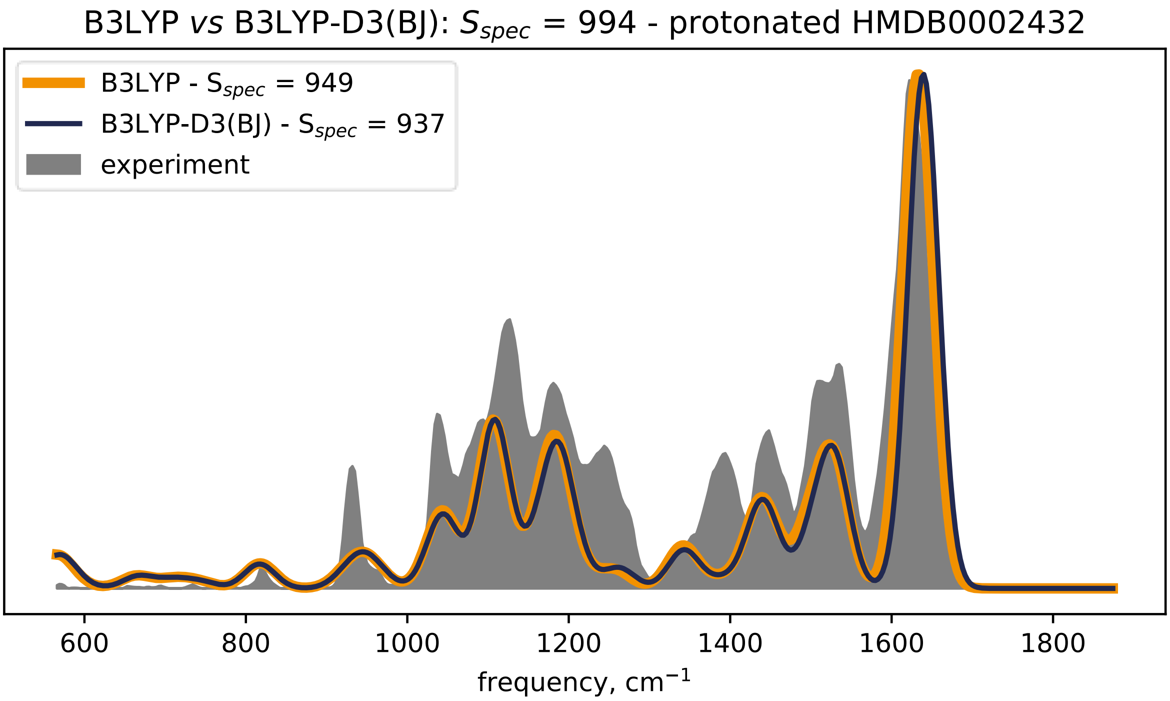
B3LYP vs B3LYP-D3(BJ): Sspec = 994 - protonated HMDB0002432
B3LYP - Sspec = 949
B3LYP-D3(BJ) - Sspec = 937
experiment
600
800
1000
1200
1400
1600
1800
frequency, cm−1

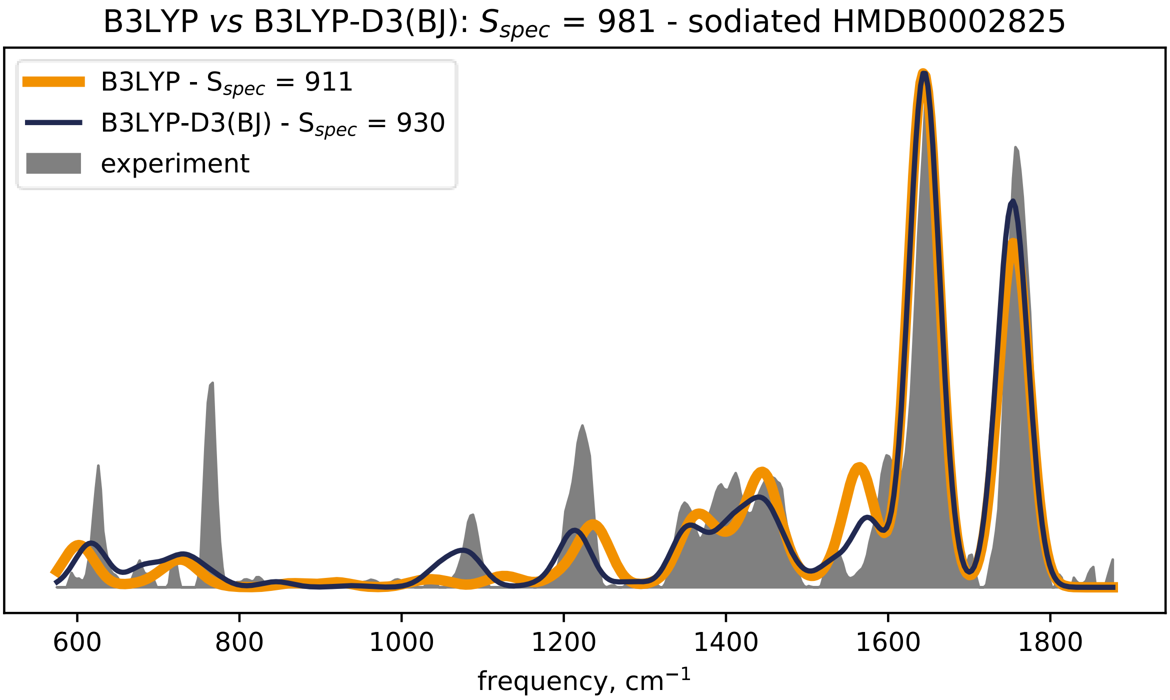
B3LYP vs B3LYP-D3(BJ): Sspec = 981 - sodiated HMDB0002825
B3LYP - Sspec = 911
B3LYP-D3(BJ) - Sspec = 930
experiment
600
800
1000
1200
1400
1600
1800
frequency, cm−1

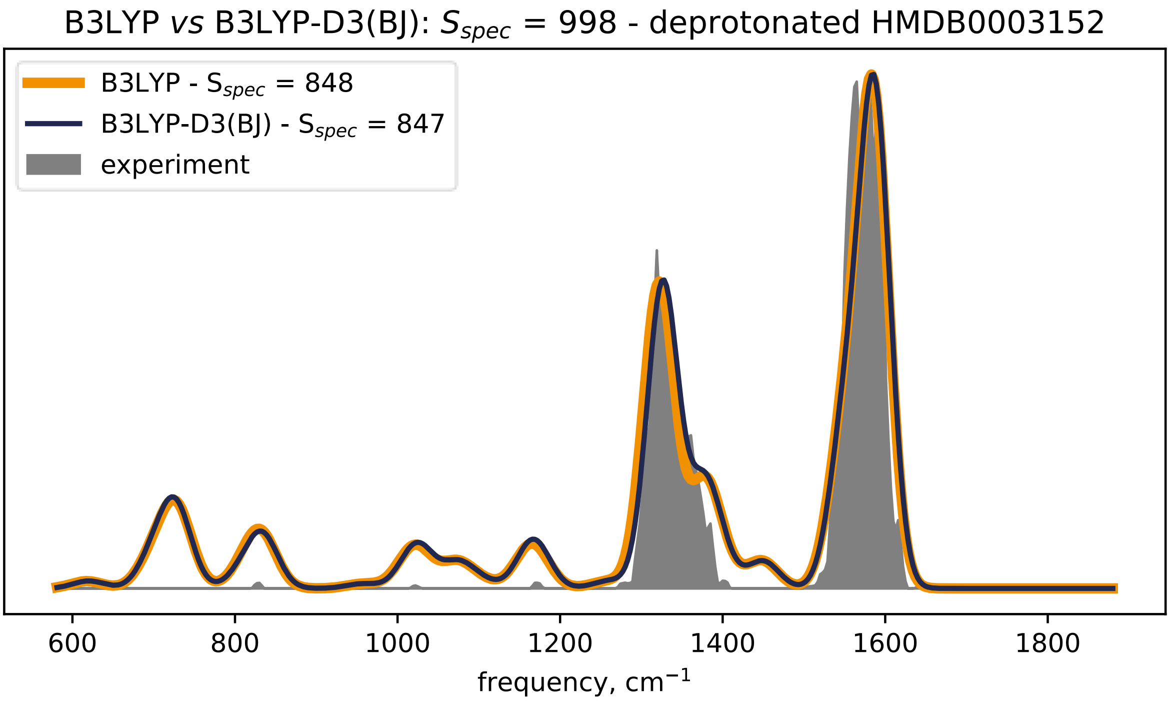
B3LYP vs B3LYP-D3(BJ): S_{spec} = 998 - deprotonated HMDB0003152
B3LYP - S_{spec} = 848
B3LYP-D3(BJ) - S_{spec} = 847
experiment
600
800
1000
1200
1400
1600
1800
frequency, cm^{-1}

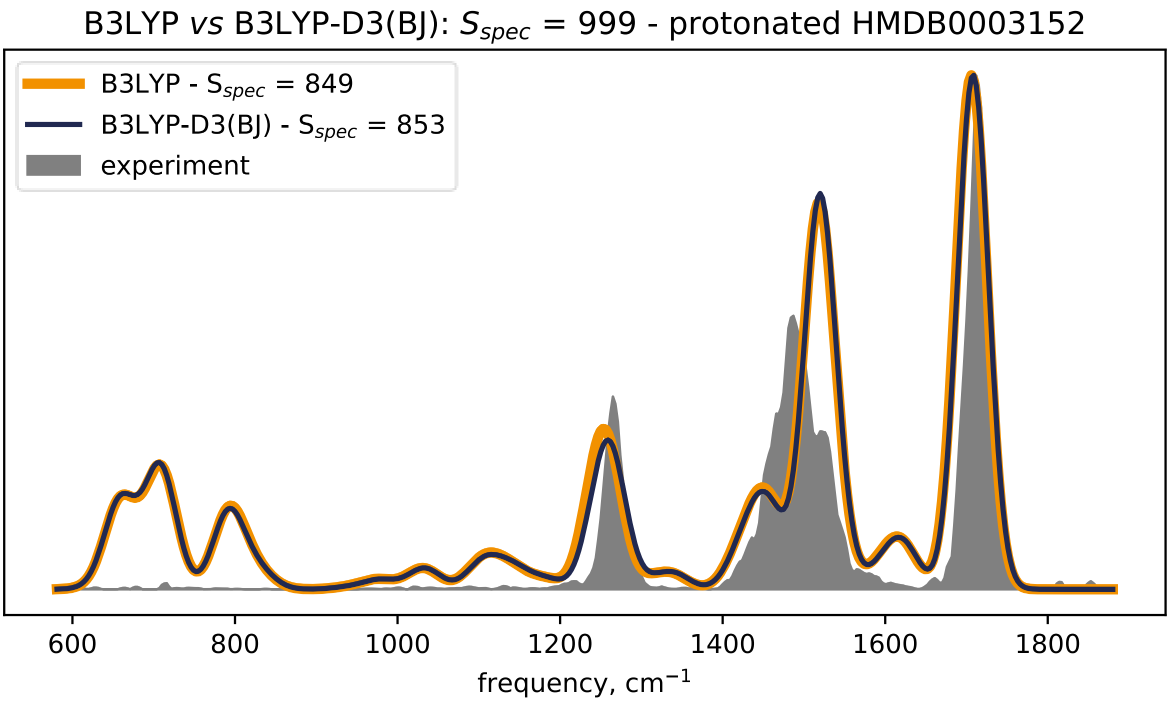
B3LYP vs B3LYP-D3(BJ): S_{spec} = 999 - protonated HMDB0003152
B3LYP - S_{spec} = 849
B3LYP-D3(BJ) - S_{spec} = 853
experiment
600
800
1000
1200
1400
1600
1800
frequency, cm^{-1}

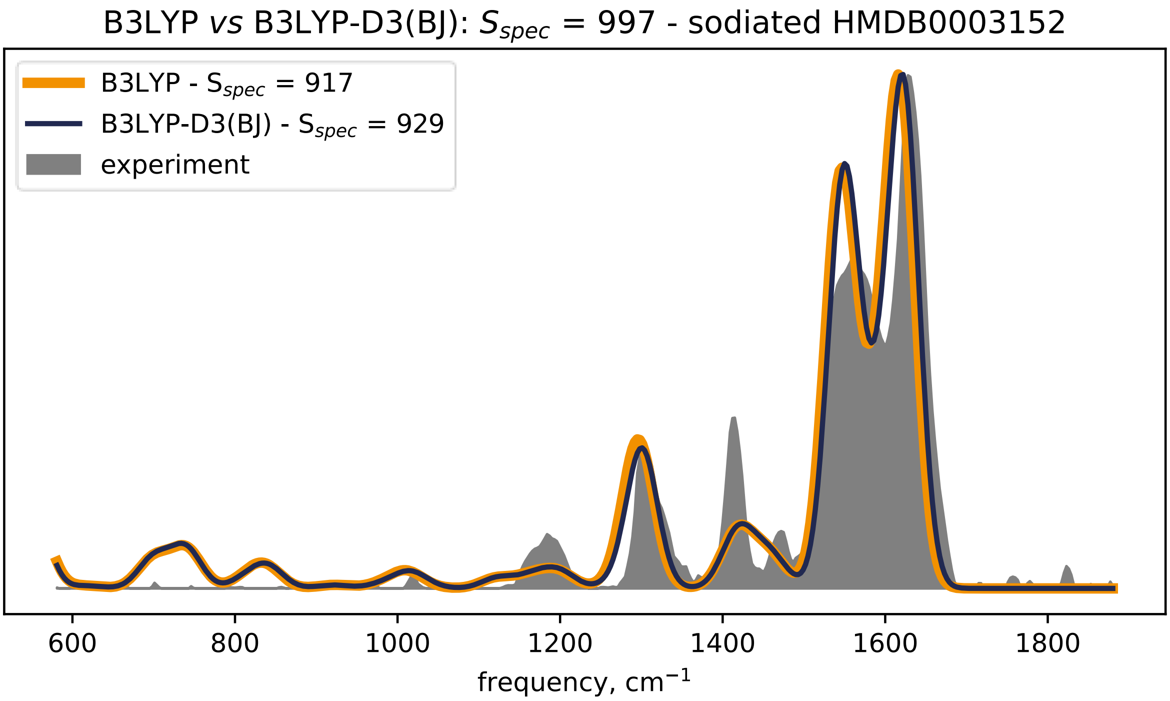

B3LYP vs B3LYP-D3(BJ): S_{spec} = 997 - sodiated HMDB0003152
B3LYP - S_{spec} = 917
B3LYP-D3(BJ) - S_{spec} = 929
experiment
600
800
1000
1200
1400
1600
1800
frequency, cm^{-1}

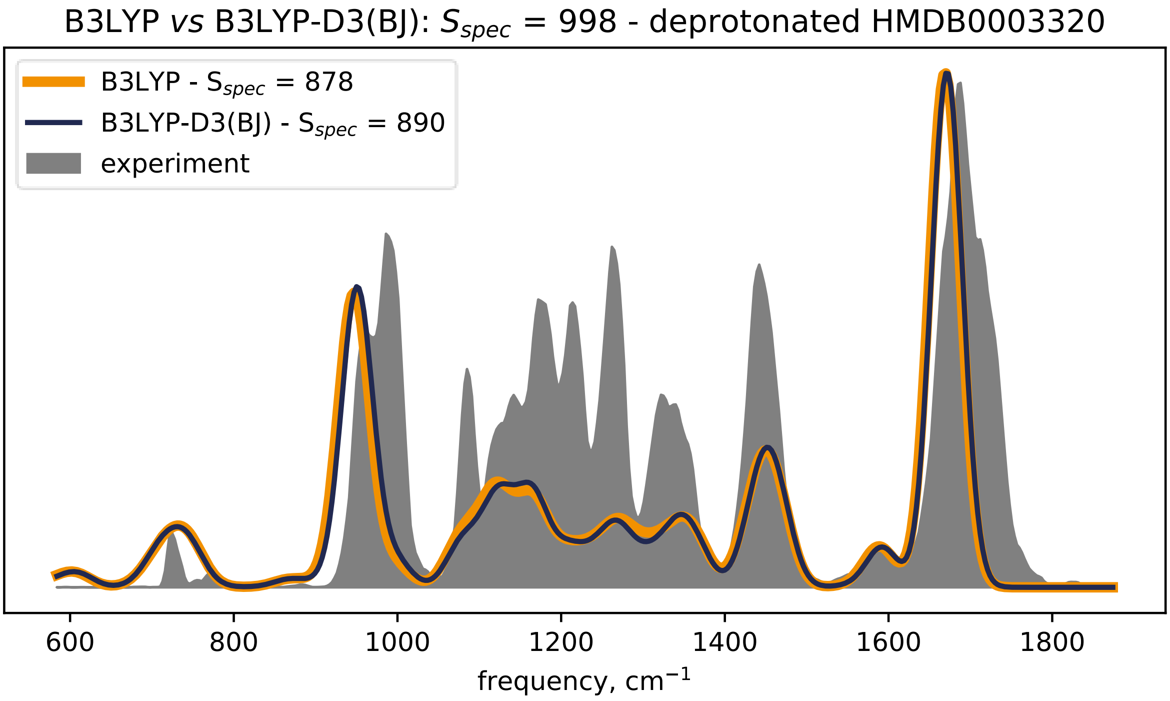

B3LYP vs B3LYP-D3(BJ): S_{spec} = 998 - deprotonated HMDB0003320
B3LYP - S_{spec} = 878
B3LYP-D3(BJ) - S_{spec} = 890
experiment
600
800
1000
1200
1400
1600
1800
frequency, cm^{-1}

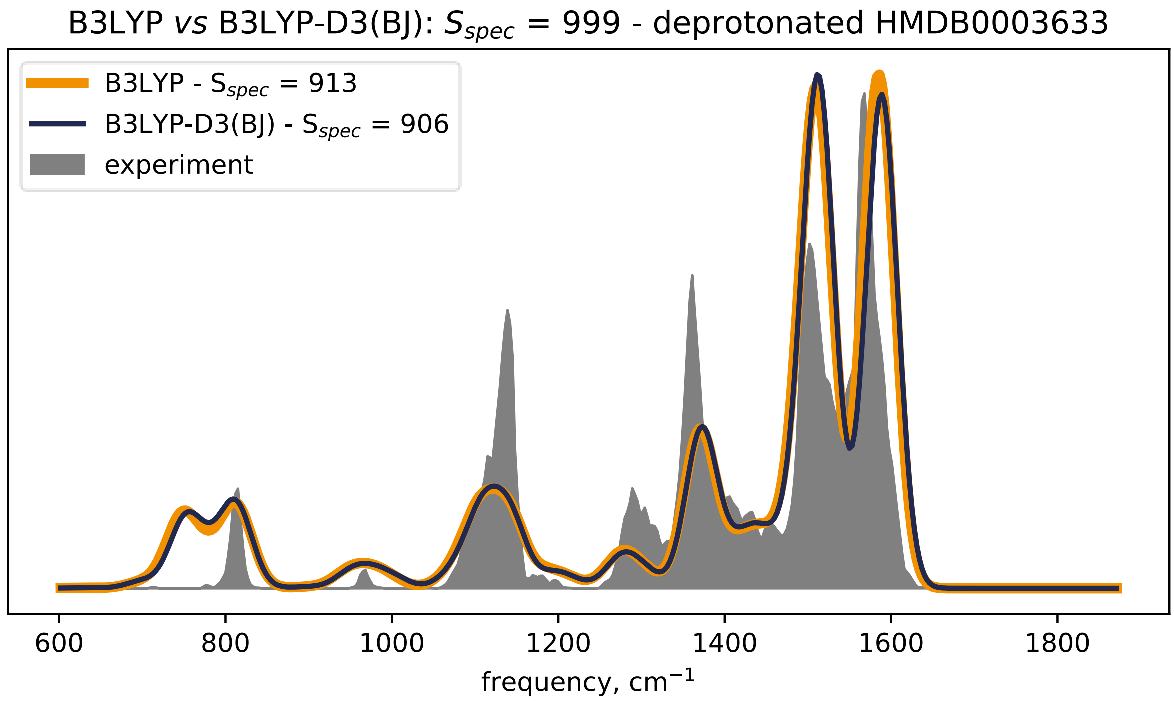

B3LYP *vs* B3LYP-D3(BJ): S_{spec} = 999 - deprotonated HMDB0003633
B3LYP - S_{spec} = 913
B3LYP-D3(BJ) - S_{spec} = 906
experiment
600
800
1000
1200
1400
1600
1800
frequency, cm^{-1}

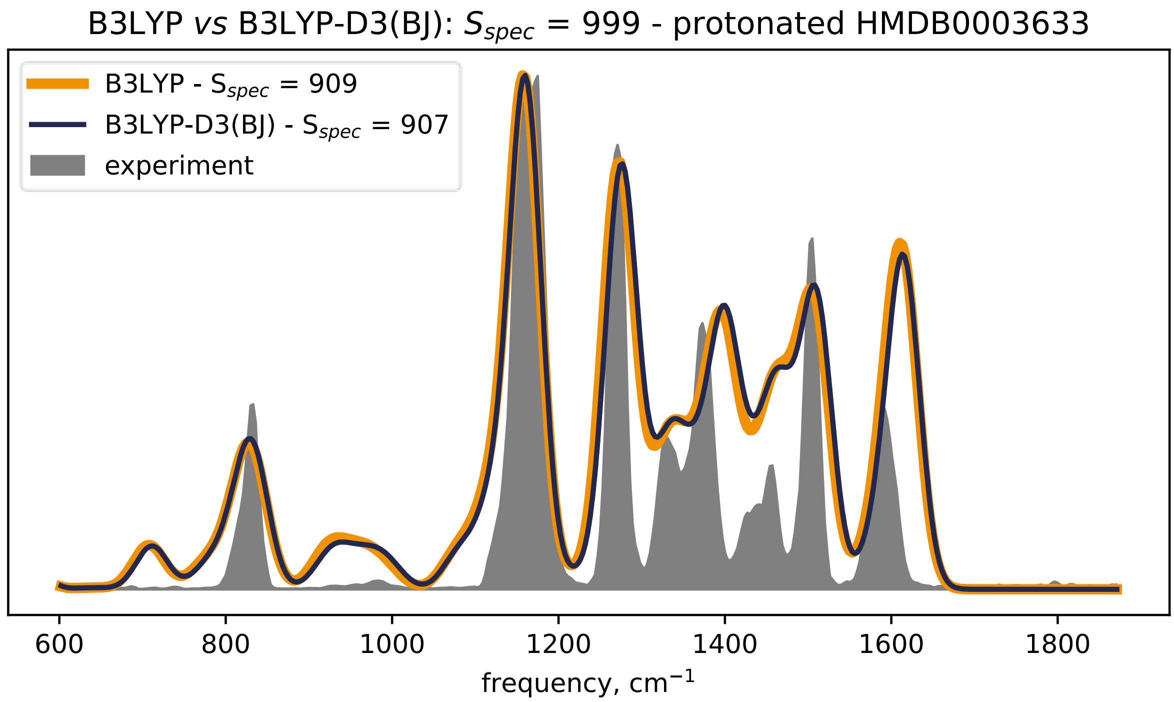

B3LYP *vs* B3LYP-D3(BJ): S_{spec} = 999 - protonated HMDB0003633
B3LYP - S_{spec} = 909
B3LYP-D3(BJ) - S_{spec} = 907
experiment
600
800
1000
1200
1400
1600
1800
frequency, cm^{-1}

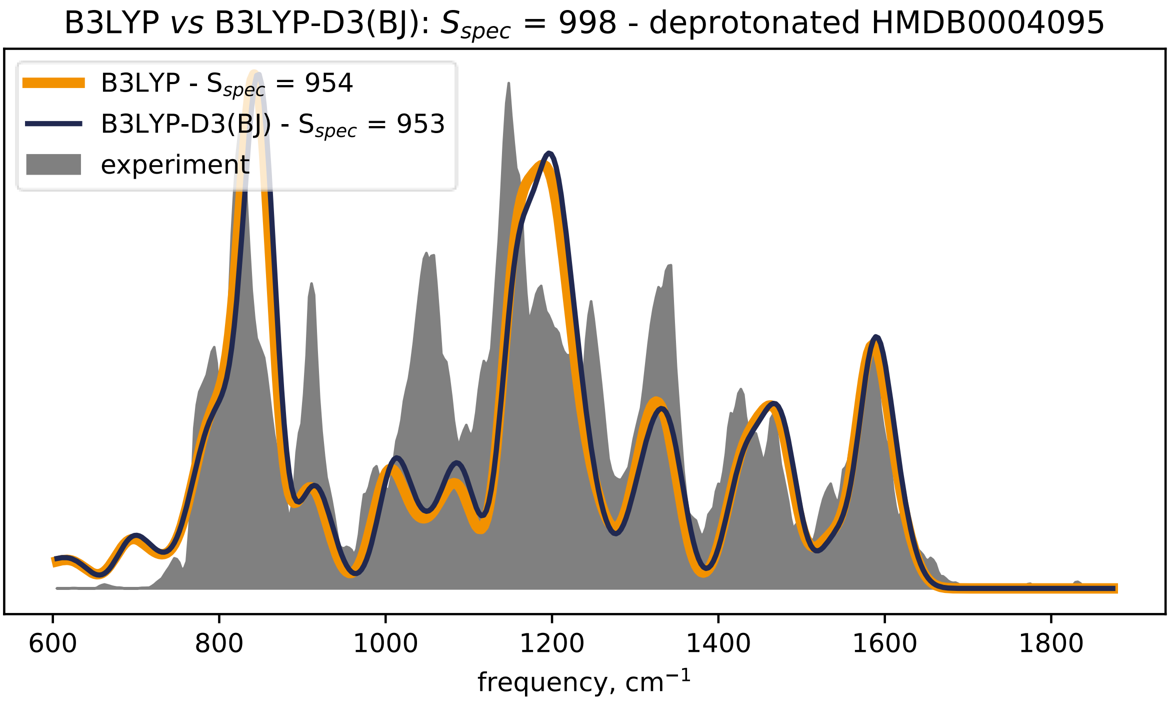
B3LYP vs B3LYP-D3(BJ): S_{spec} = 998 - deprotonated HMDB0004095
B3LYP - S_{spec} = 954
B3LYP-D3(BJ) - S_{spec} = 953
experiment
600
800
1000
1200
1400
1600
1800
frequency, cm^{-1}

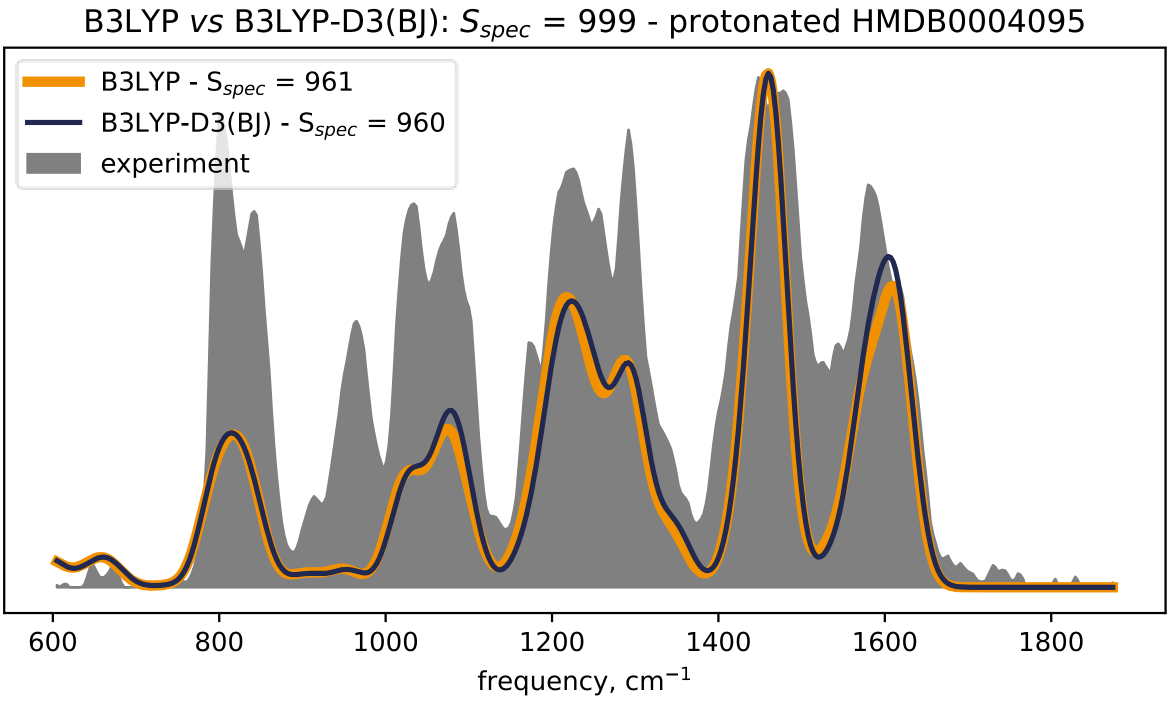
B3LYP vs B3LYP-D3(BJ): S_{spec} = 999 - protonated HMDB0004095
B3LYP - S_{spec} = 961
B3LYP-D3(BJ) - S_{spec} = 960
experiment
600
800
1000
1200
1400
1600
1800
frequency, cm^{-1}

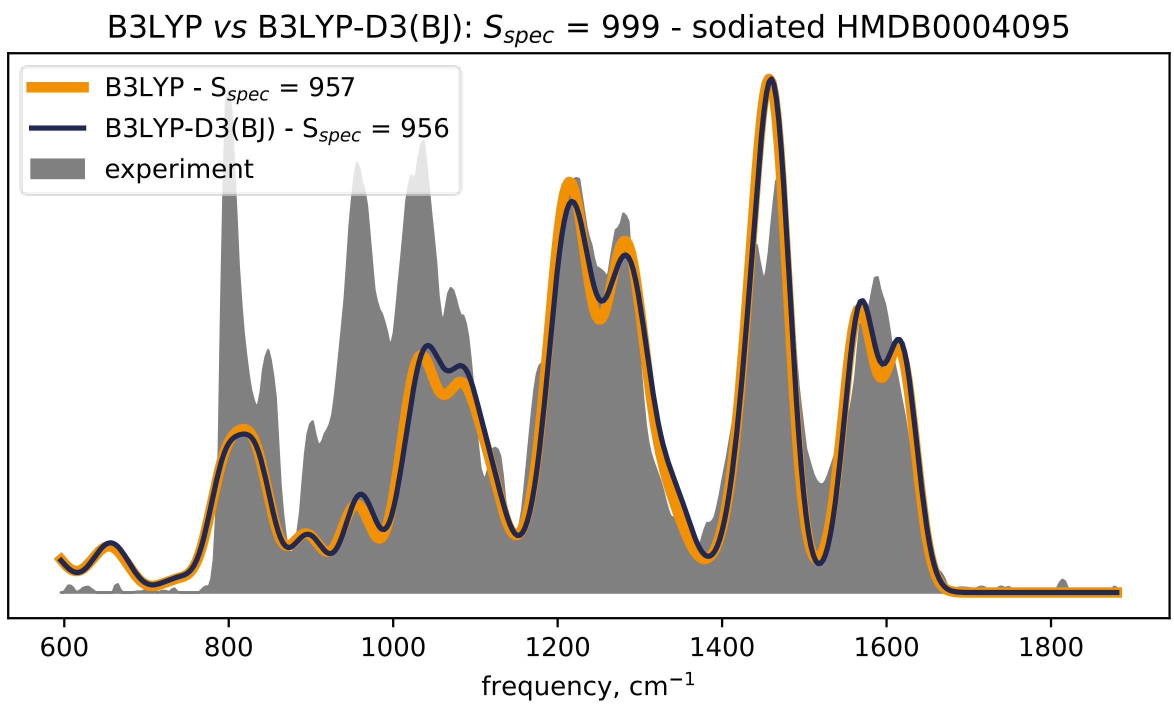

B3LYP vs B3LYP-D3(BJ): S_{spec} = 999 - sodiated HMDB0004095
B3LYP - S_{spec} = 957
B3LYP-D3(BJ) - S_{spec} = 956
experiment
600
800
1000
1200
1400
1600
1800
frequency, cm^{-1}

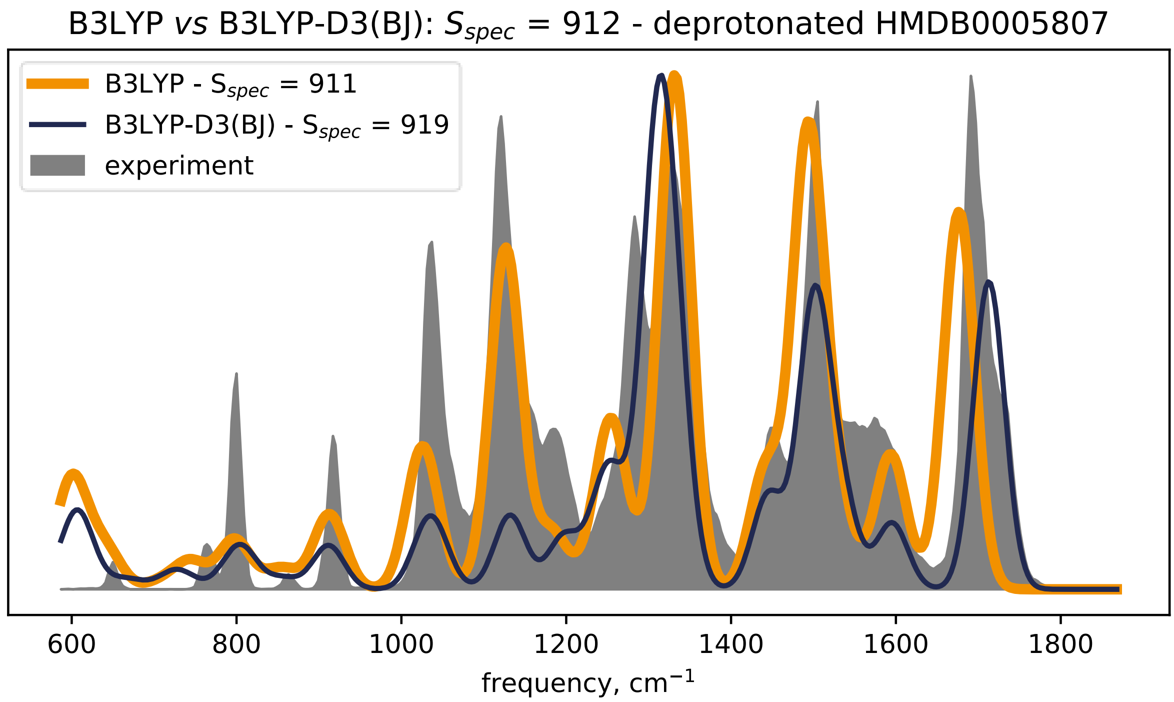

B3LYP vs B3LYP-D3(BJ): S_{spec} = 912 - deprotonated HMDB0005807
B3LYP - S_{spec} = 911
B3LYP-D3(BJ) - S_{spec} = 919
experiment
600
800
1000
1200
1400
1600
1800
frequency, cm^{-1}

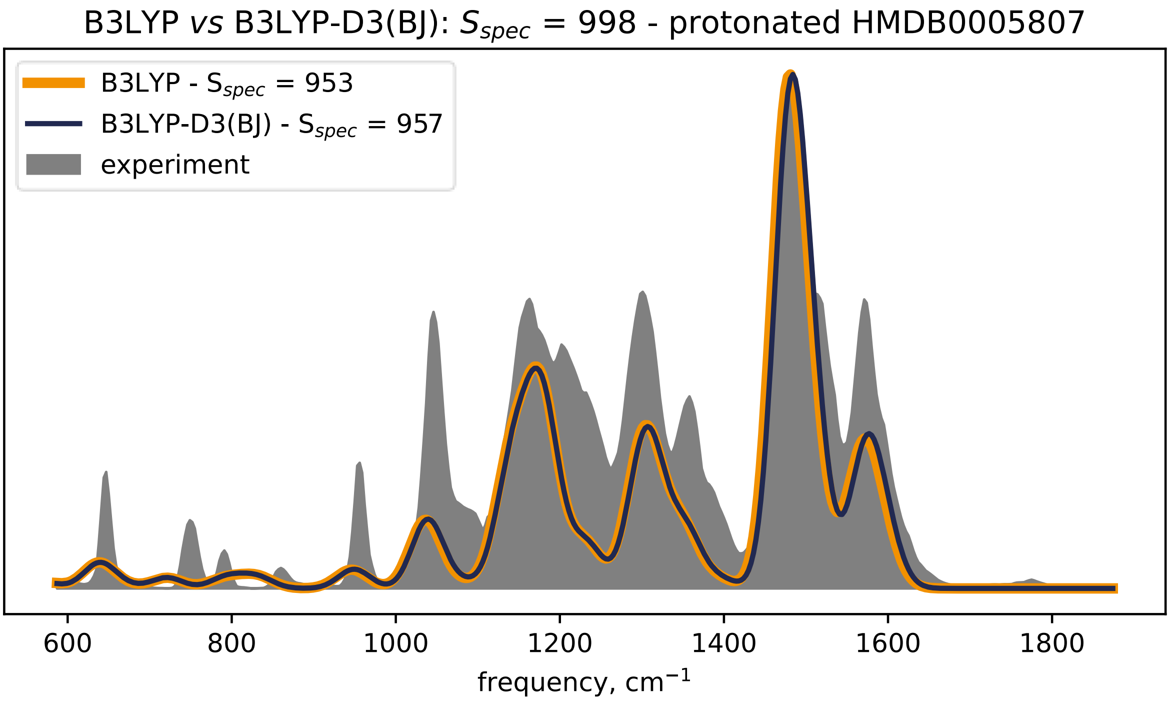
B3LYP vs B3LYP-D3(BJ): S_{spec} = 998 - protonated HMDB0005807
B3LYP - S_{spec} = 953
B3LYP-D3(BJ) - S_{spec} = 957
experiment
600
800
1000
1200
1400
1600
1800
frequency, cm^{-1}

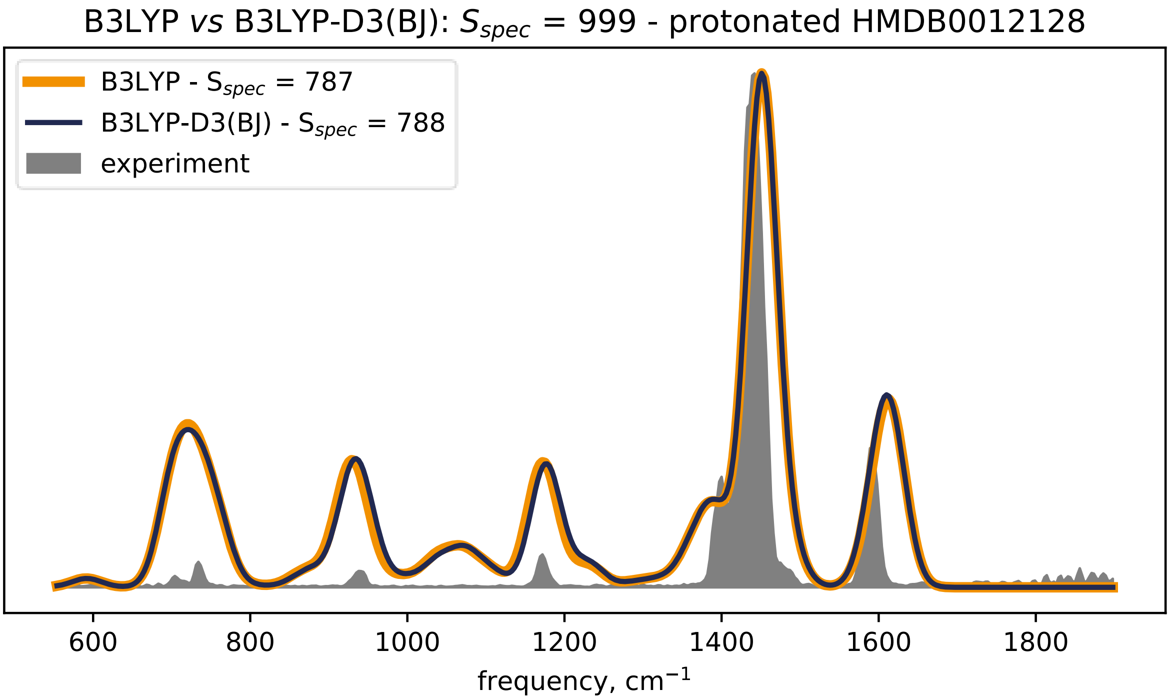
B3LYP vs B3LYP-D3(BJ): S_{spec} = 999 - protonated HMDB0012128
B3LYP - S_{spec} = 787
B3LYP-D3(BJ) - S_{spec} = 788
experiment
600
800
1000
1200
1400
1600
1800
frequency, cm^{-1}

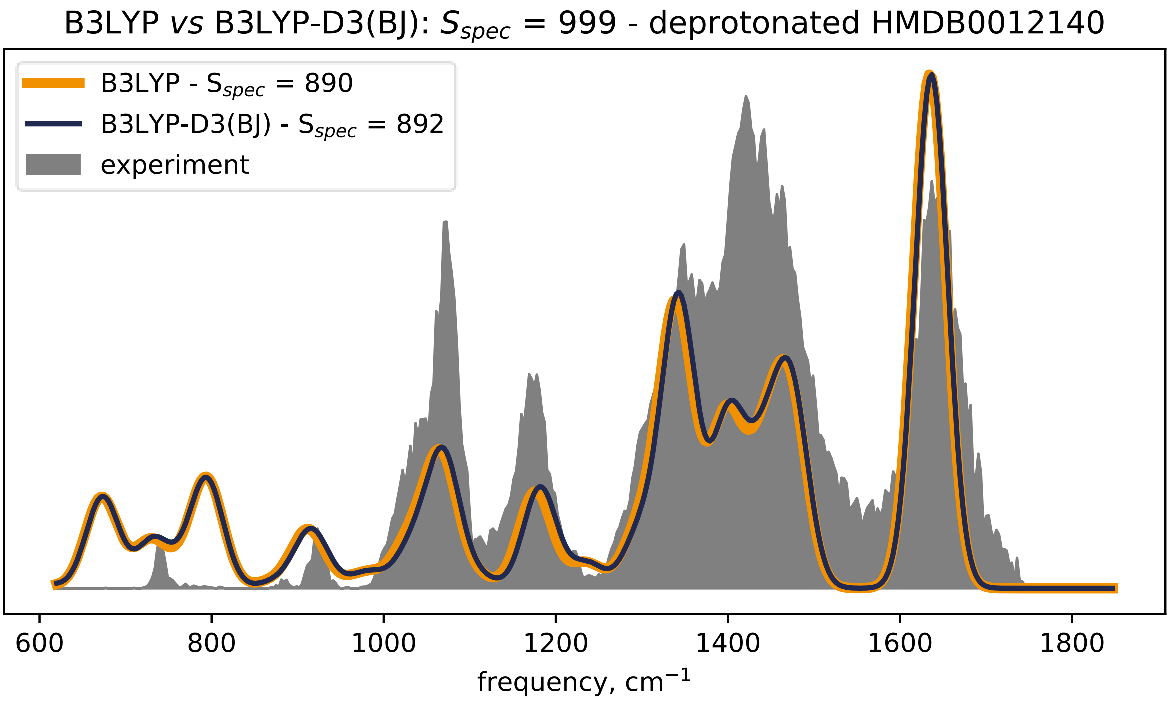
B3LYP vs B3LYP-D3(BJ): S_{spec} = 999 - deprotonated HMDB0012140
B3LYP - S_{spec} = 890
B3LYP-D3(BJ) - S_{spec} = 892
experiment
600
800
1000
1200
1400
1600
1800
frequency, cm^{-1}

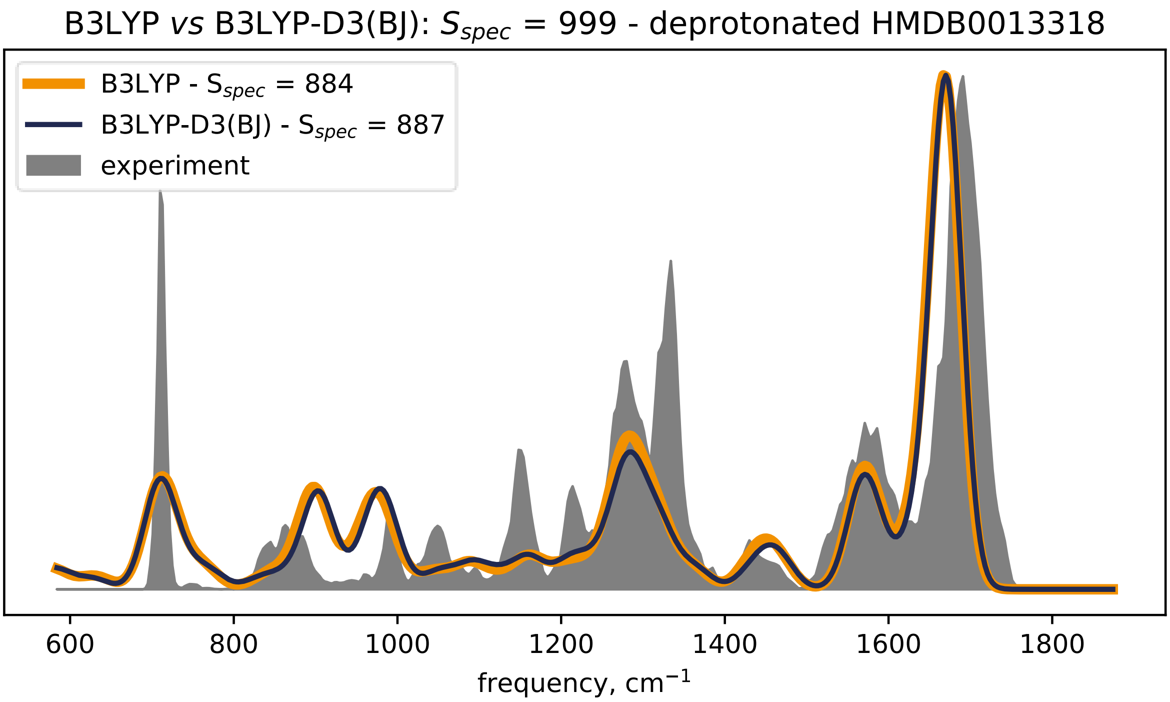
B3LYP vs B3LYP-D3(BJ): S_{spec} = 999 - deprotonated HMDB0013318
B3LYP - S_{spec} = 884
B3LYP-D3(BJ) - S_{spec} = 887
experiment
600
800
1000
1200
1400
1600
1800
frequency, cm^{-1}

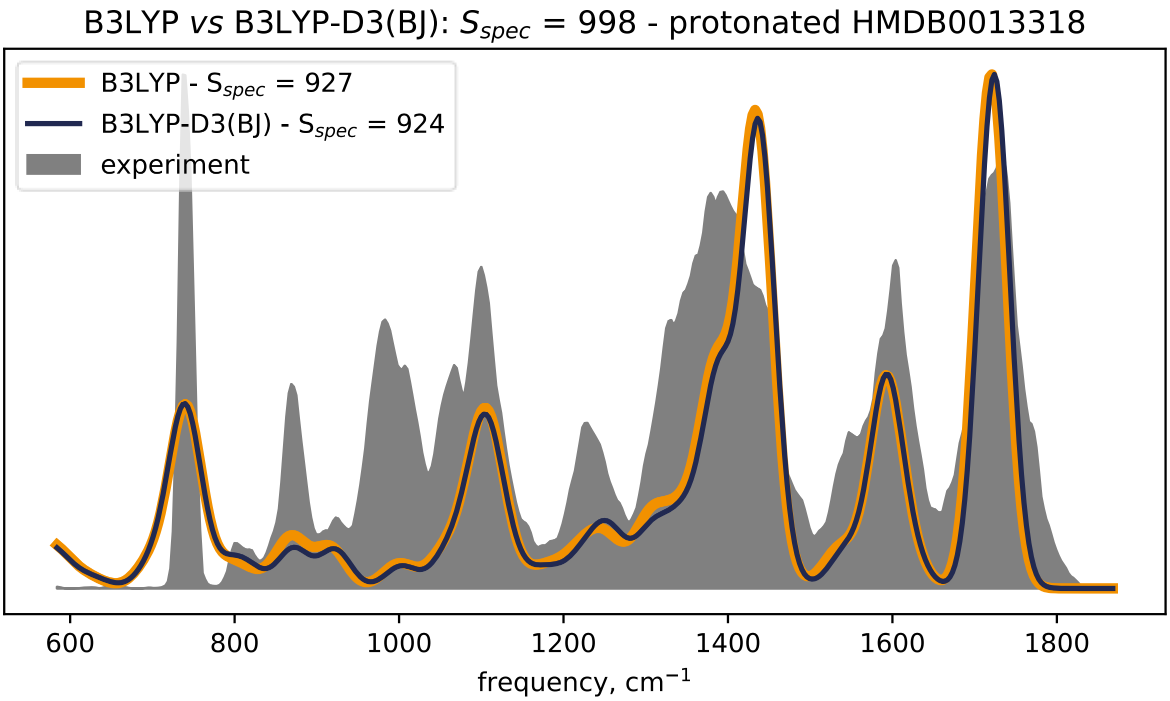
B3LYP vs B3LYP-D3(BJ): S_{spec} = 998 - protonated HMDB0013318
B3LYP - S_{spec} = 927
B3LYP-D3(BJ) - S_{spec} = 924
experiment
600
800
1000
1200
1400
1600
1800
frequency, cm^{-1}

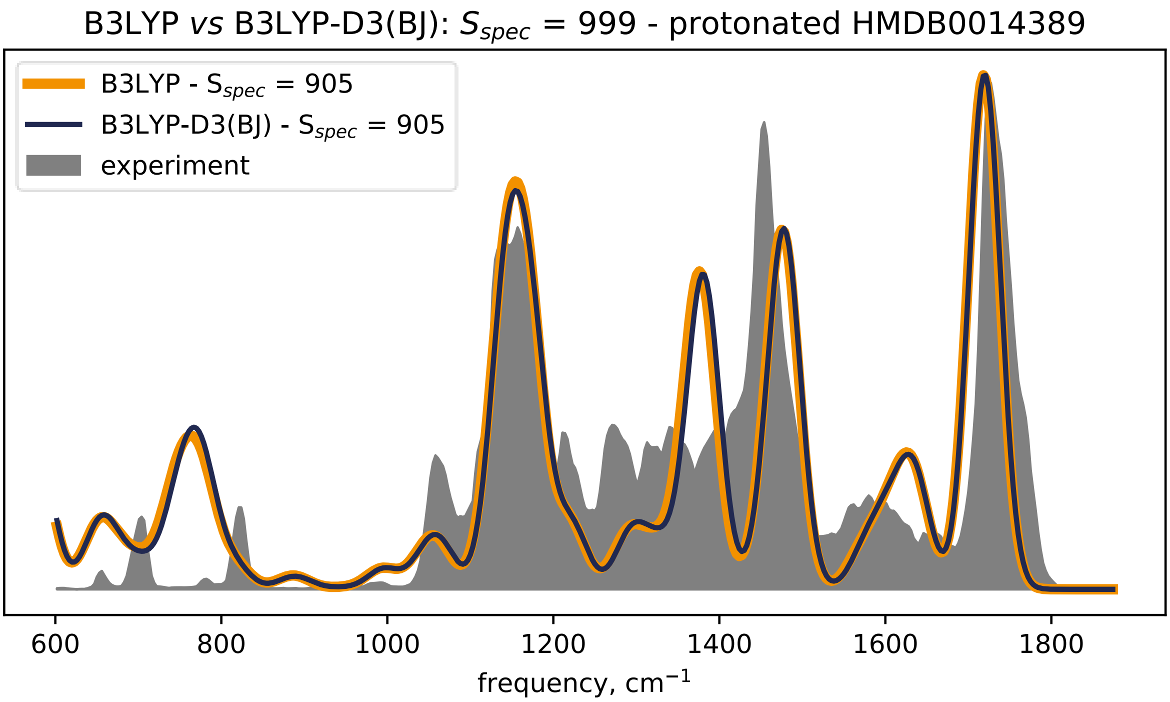
B3LYP vs B3LYP-D3(BJ): S_{spec} = 999 - protonated HMDB0014389
B3LYP - S_{spec} = 905
B3LYP-D3(BJ) - S_{spec} = 905
experiment
600
800
1000
1200
1400
1600
1800
frequency, cm^{-1}

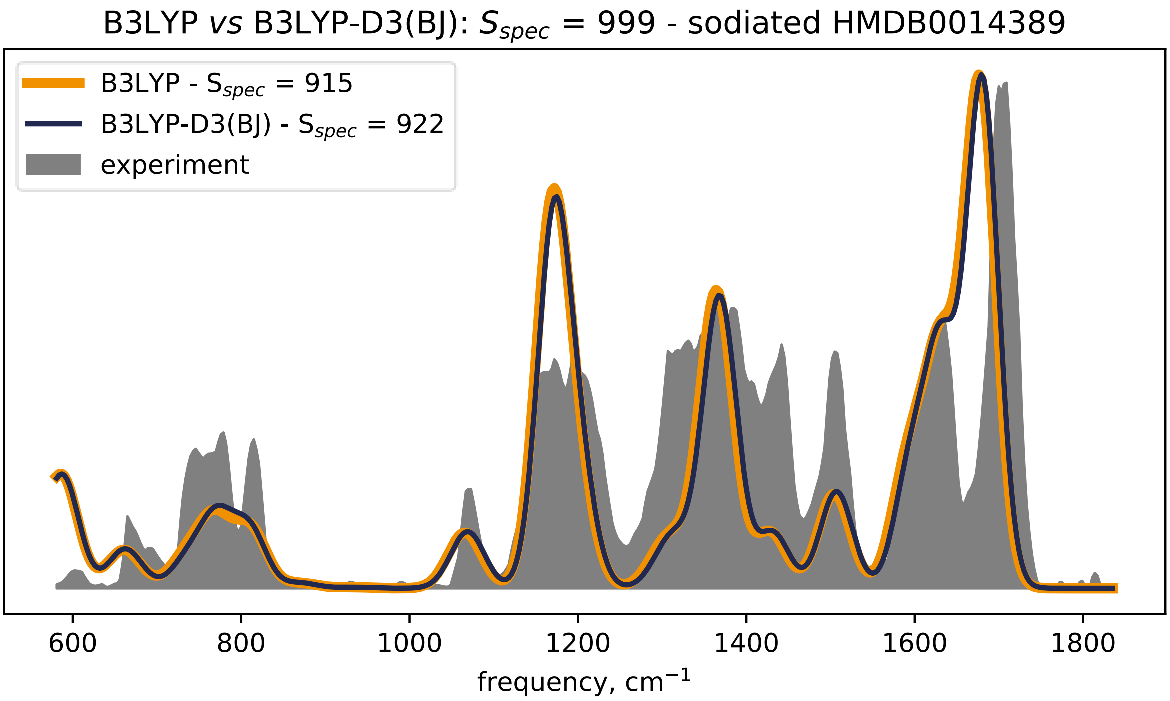
B3LYP *vs* B3LYP-D3(BJ): S_{spec} = 999 - sodiated HMDB0014389
B3LYP - S_{spec} = 915
B3LYP-D3(BJ) - S_{spec} = 922
experiment
600
800
1000
1200
1400
1600
1800
frequency, cm^{-1}

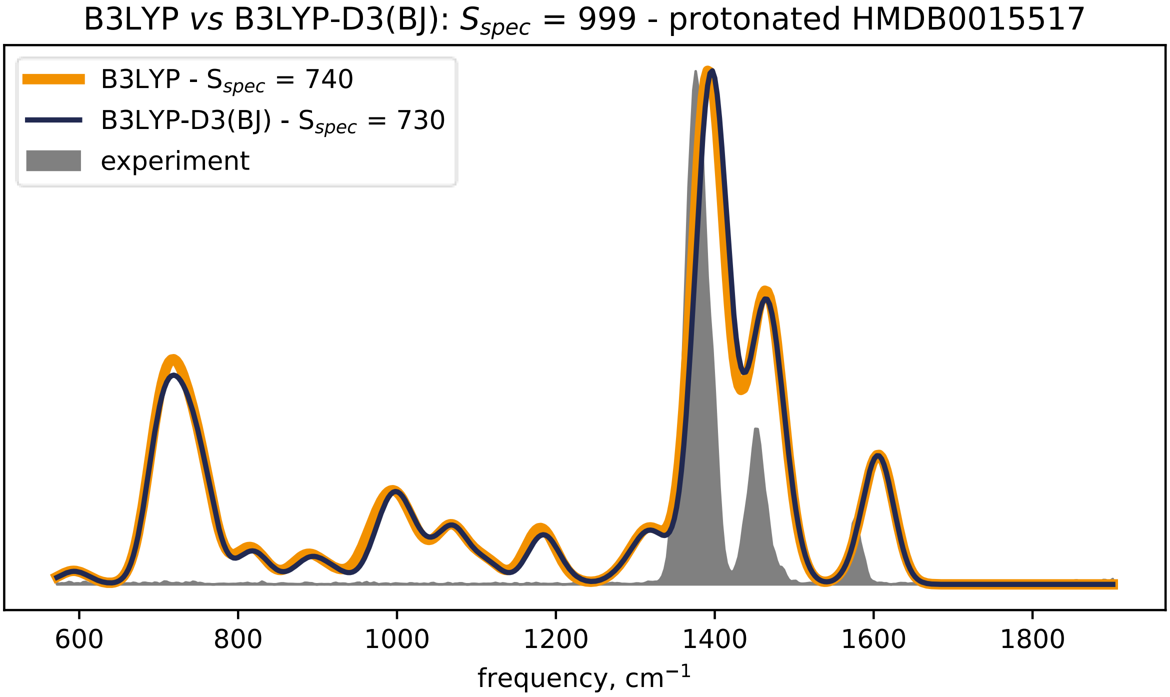
B3LYP *vs* B3LYP-D3(BJ): S_{spec} = 999 - protonated HMDB0015517
B3LYP - S_{spec} = 740
B3LYP-D3(BJ) - S_{spec} = 730
experiment
600
800
1000
1200
1400
1600
1800
frequency, cm^{-1}

B3LYP *vs* B3LYP-D3(BJ): S_{spec} = 999 - protonated HMDB0028850

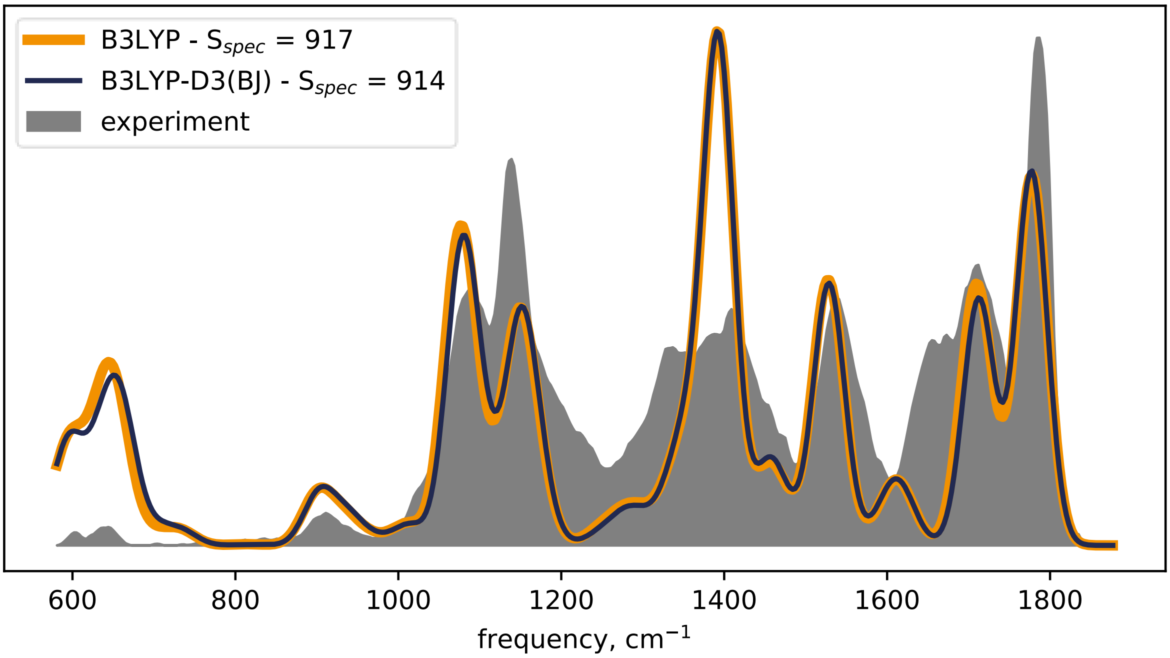

B3LYP *vs* B3LYP-D3(BJ): S_{spec} = 999 - protonated HMDB0031861

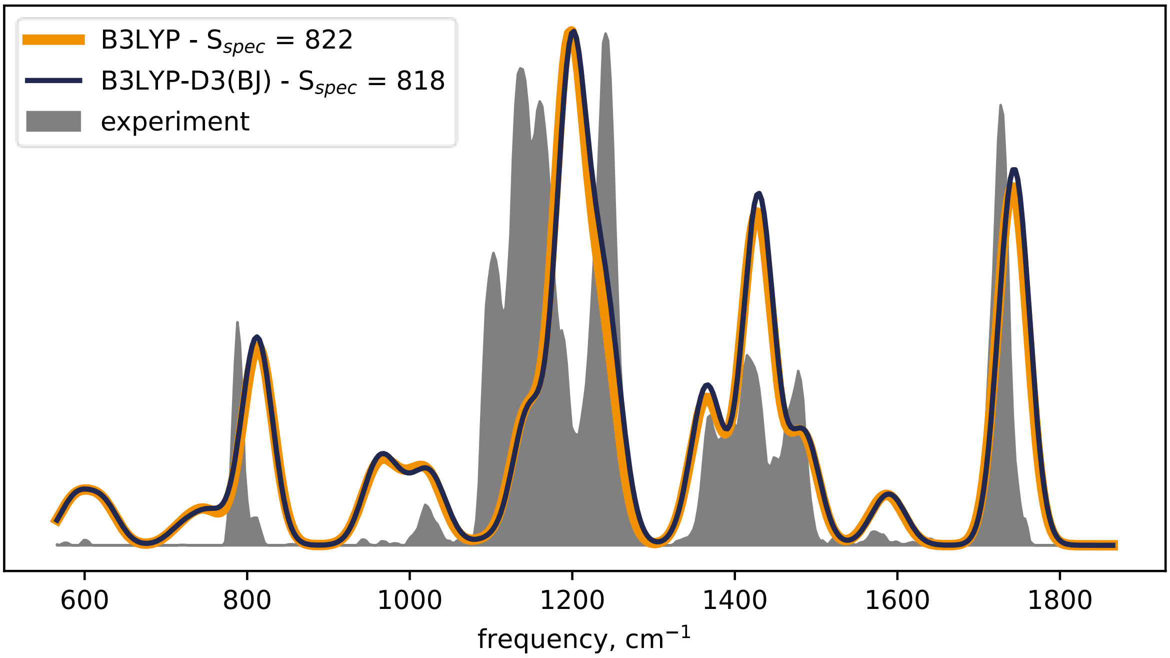

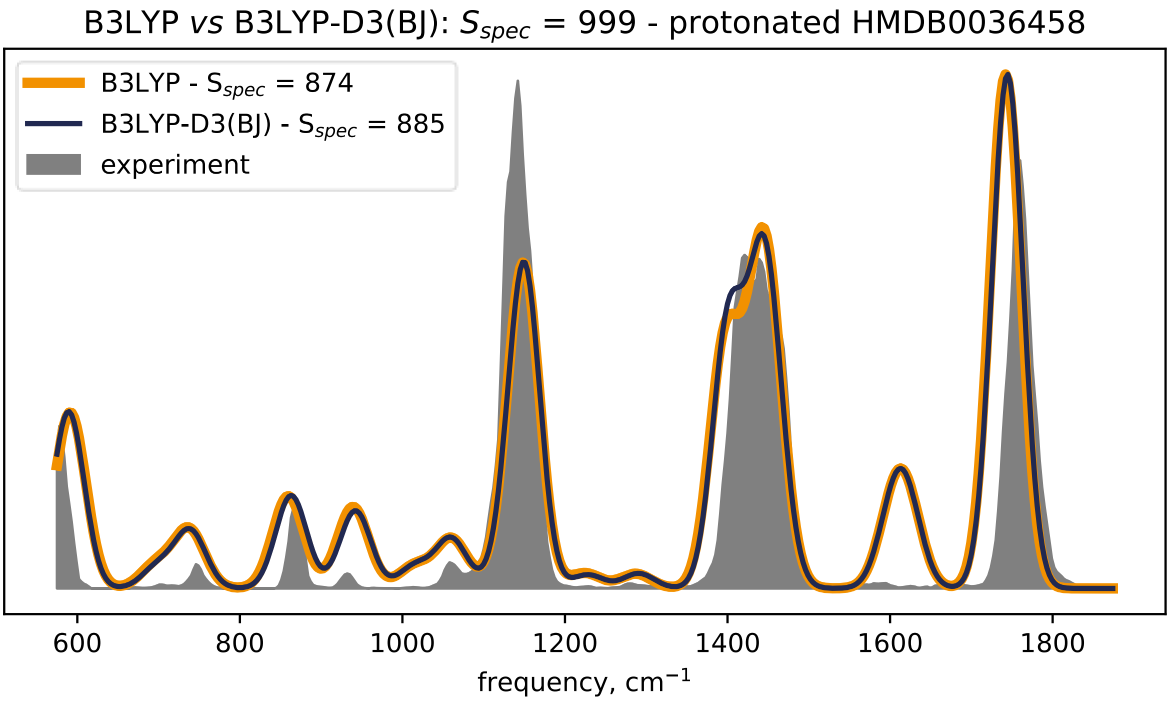
B3LYP vs B3LYP-D3(BJ): S_{spec} = 999 - protonated HMDB0036458
B3LYP - S_{spec} = 874
B3LYP-D3(BJ) - S_{spec} = 885
experiment
600
800
1000
1200
1400
1600
1800
frequency, cm^{-1}

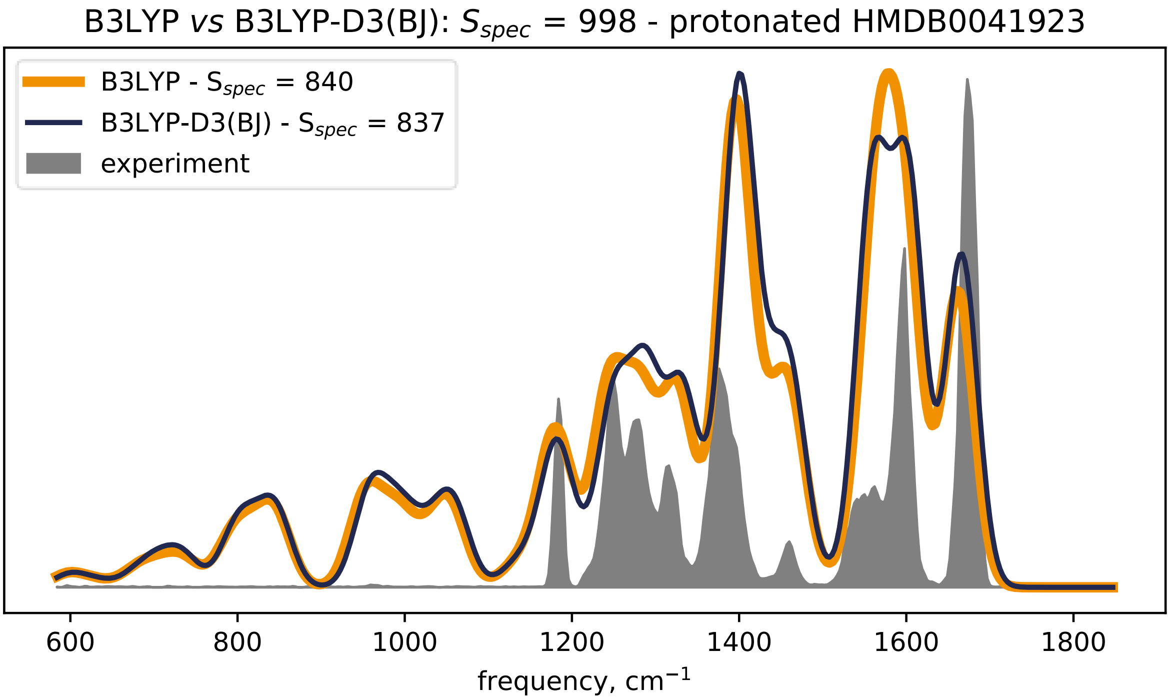
B3LYP vs B3LYP-D3(BJ): S_{spec} = 998 - protonated HMDB0041923
B3LYP - S_{spec} = 840
B3LYP-D3(BJ) - S_{spec} = 837
experiment
600
800
1000
1200
1400
1600
1800
frequency, cm^{-1}

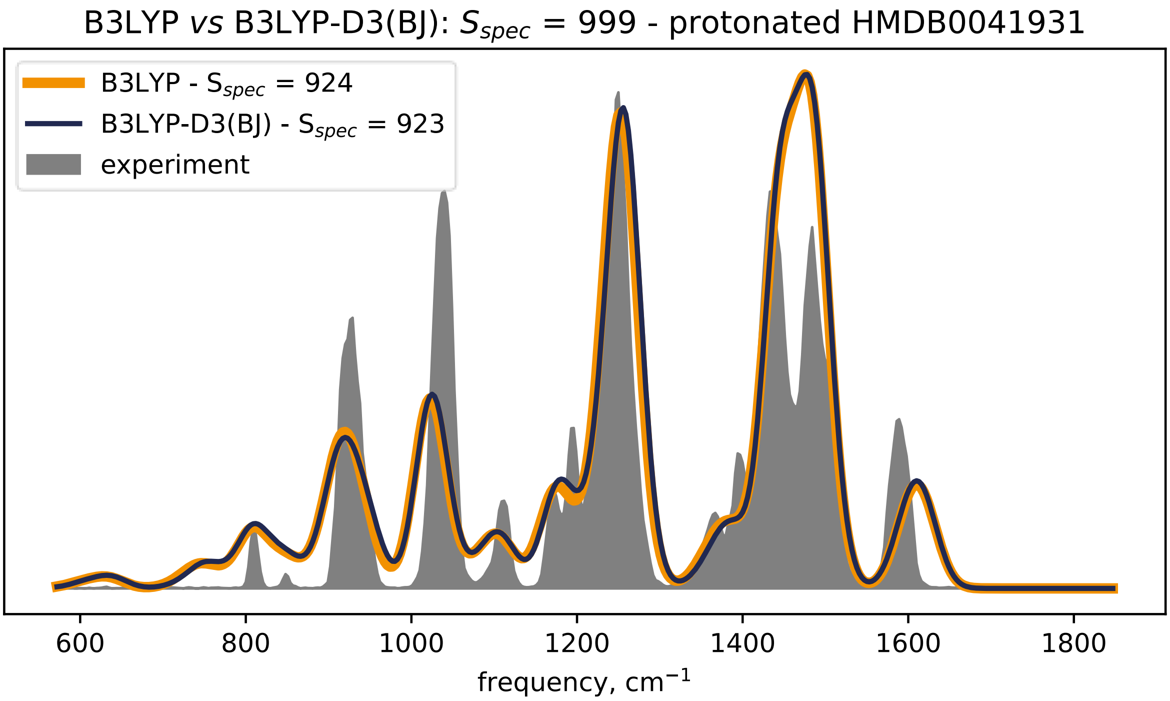
B3LYP vs B3LYP-D3(BJ): S_{spec} = 999 - protonated HMDB0041931
B3LYP - S_{spec} = 924
B3LYP-D3(BJ) - S_{spec} = 923
experiment
600
800
1000
1200
1400
1600
1800
frequency, cm^{-1}

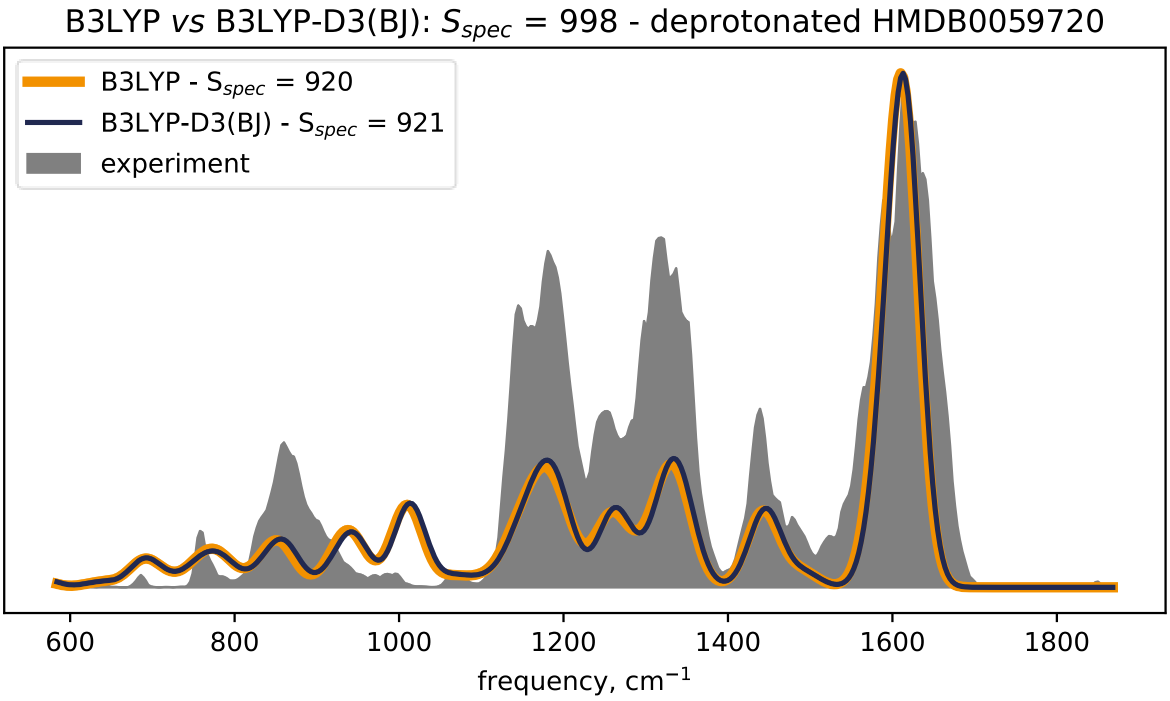
B3LYP vs B3LYP-D3(BJ): S_{spec} = 998 - deprotonated HMDB0059720
B3LYP - S_{spec} = 920
B3LYP-D3(BJ) - S_{spec} = 921
experiment
600
800
1000
1200
1400
1600
1800
frequency, cm^{-1}

B3LYP *vs* B3LYP-D3(BJ): S_{spec} = 999 - protonated HMDB0059720

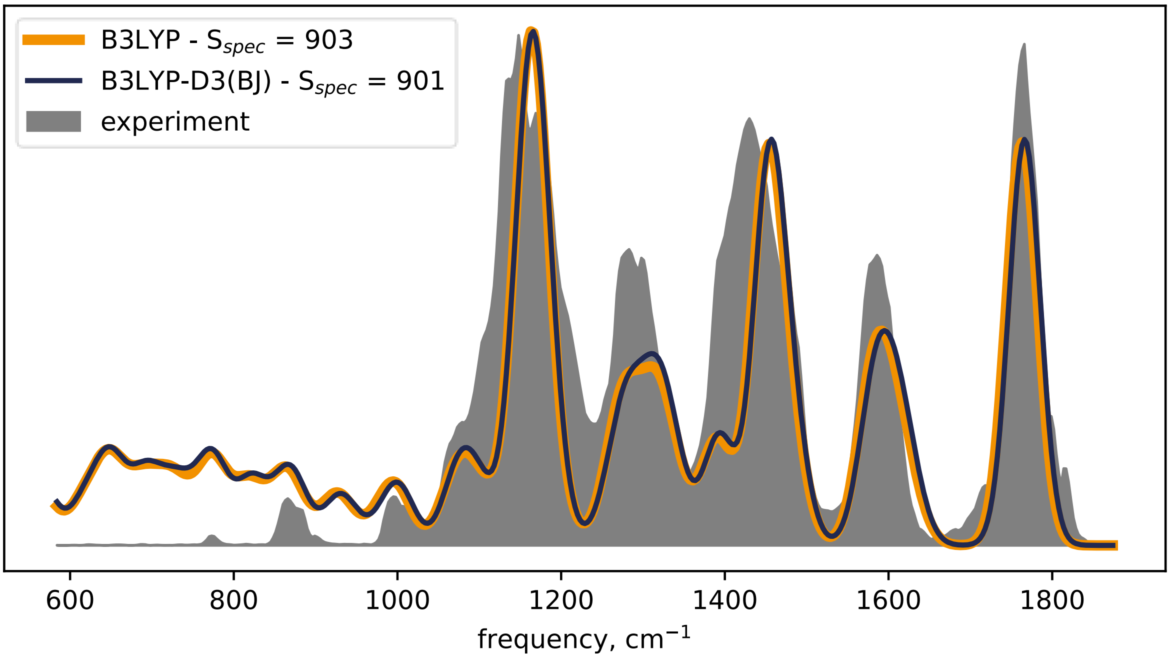

B3LYP *vs* B3LYP-D3(BJ): S_{spec} = 999 - sodiated HMDB0059720

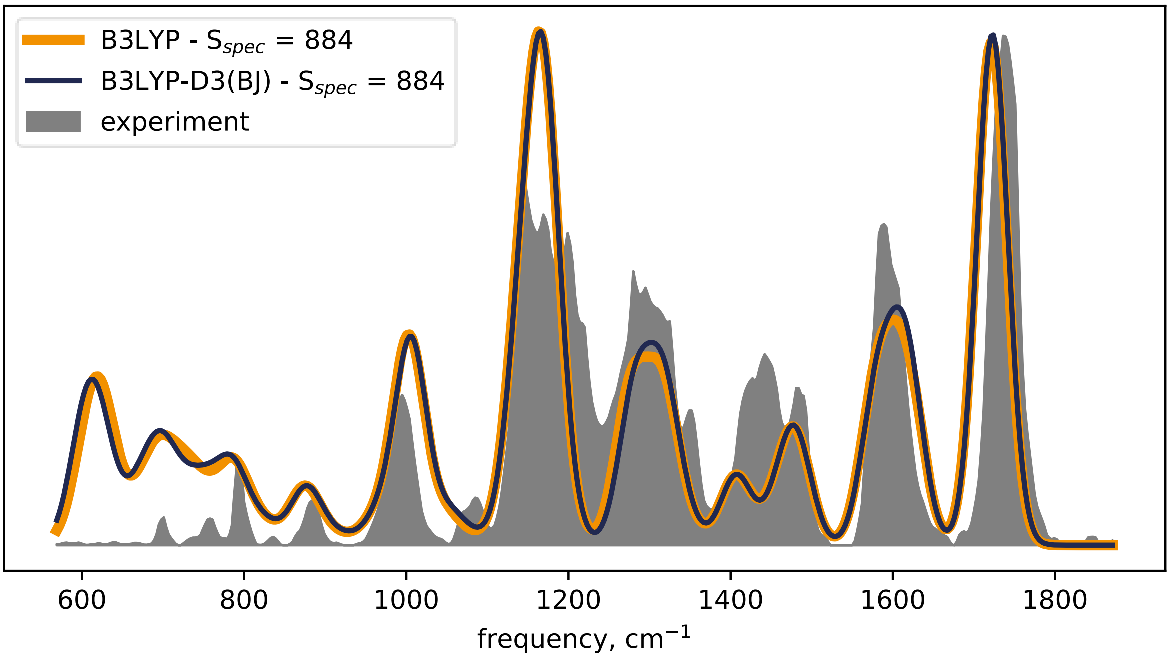

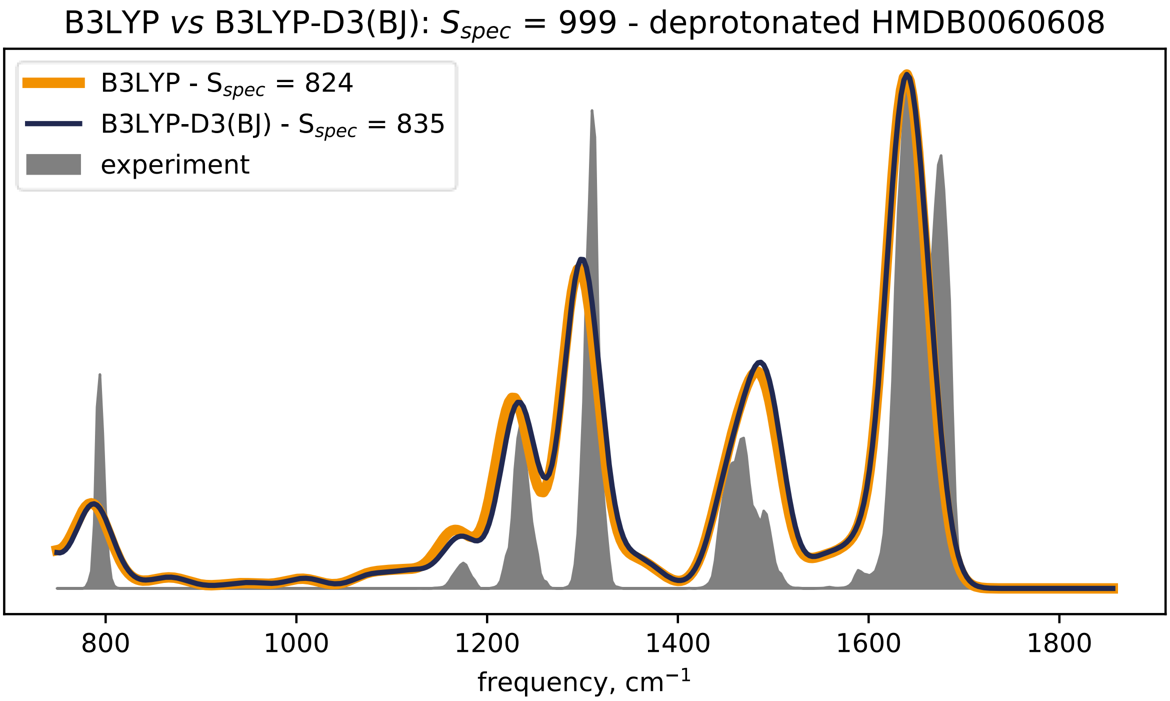

B3LYP vs B3LYP-D3(BJ): S_{spec} = 999 - deprotonated HMDB0060608
B3LYP - S_{spec} = 824
B3LYP-D3(BJ) - S_{spec} = 835
experiment
800
1000
1200
1400
1600
1800
frequency, cm^{-1}

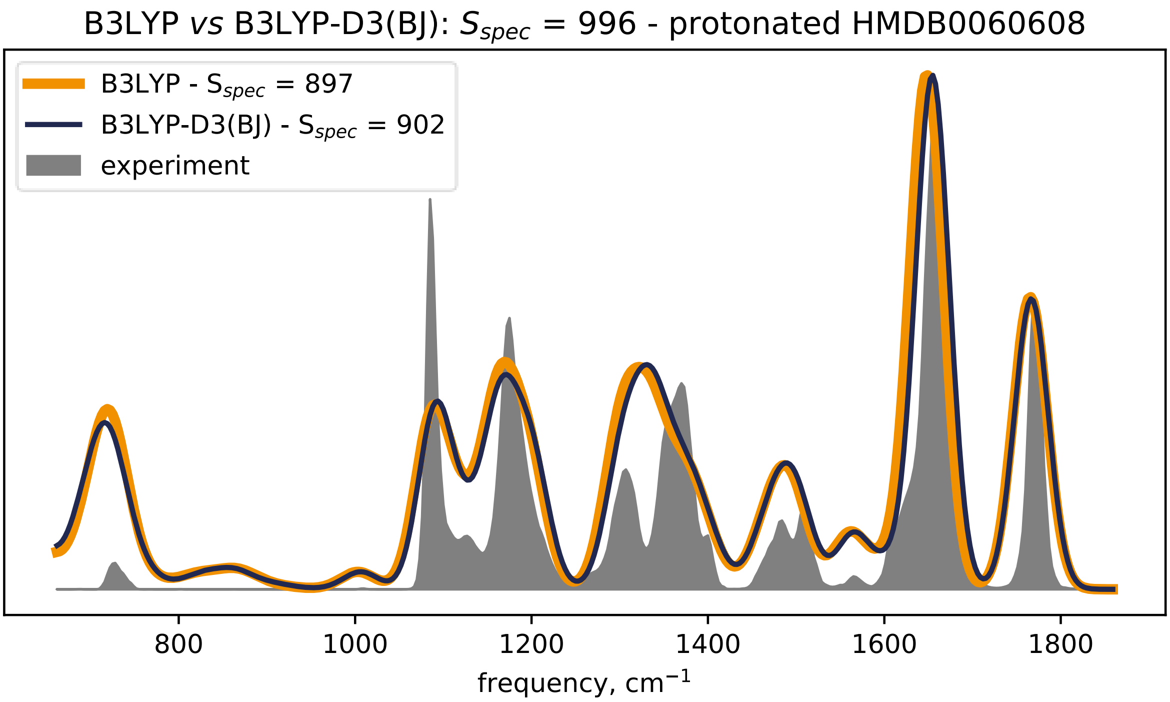

B3LYP vs B3LYP-D3(BJ): S_{spec} = 996 - protonated HMDB0060608
B3LYP - S_{spec} = 897
B3LYP-D3(BJ) - S_{spec} = 902
experiment
800
1000
1200
1400
1600
1800
frequency, cm^{-1}

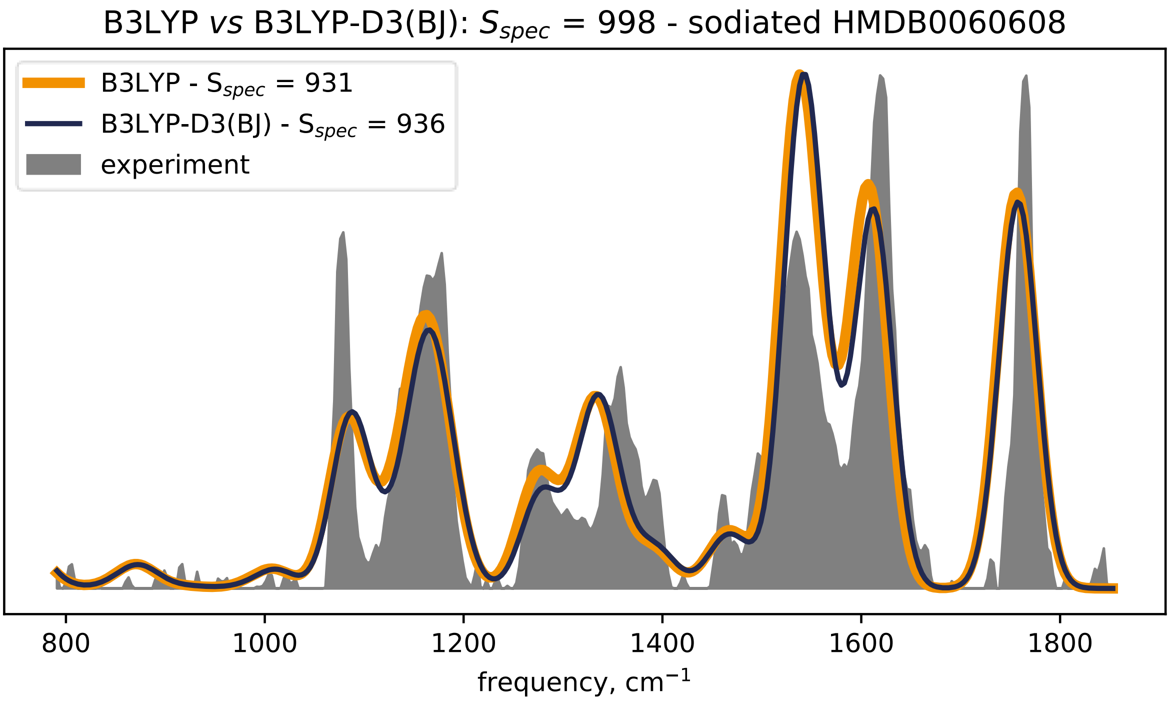
B3LYP vs B3LYP-D3(BJ): S_{spec} = 998 - sodiated HMDB0060608
B3LYP - S_{spec} = 931
B3LYP-D3(BJ) - S_{spec} = 936
experiment
800
1000
1200
1400
1600
1800
frequency, cm^{-1}

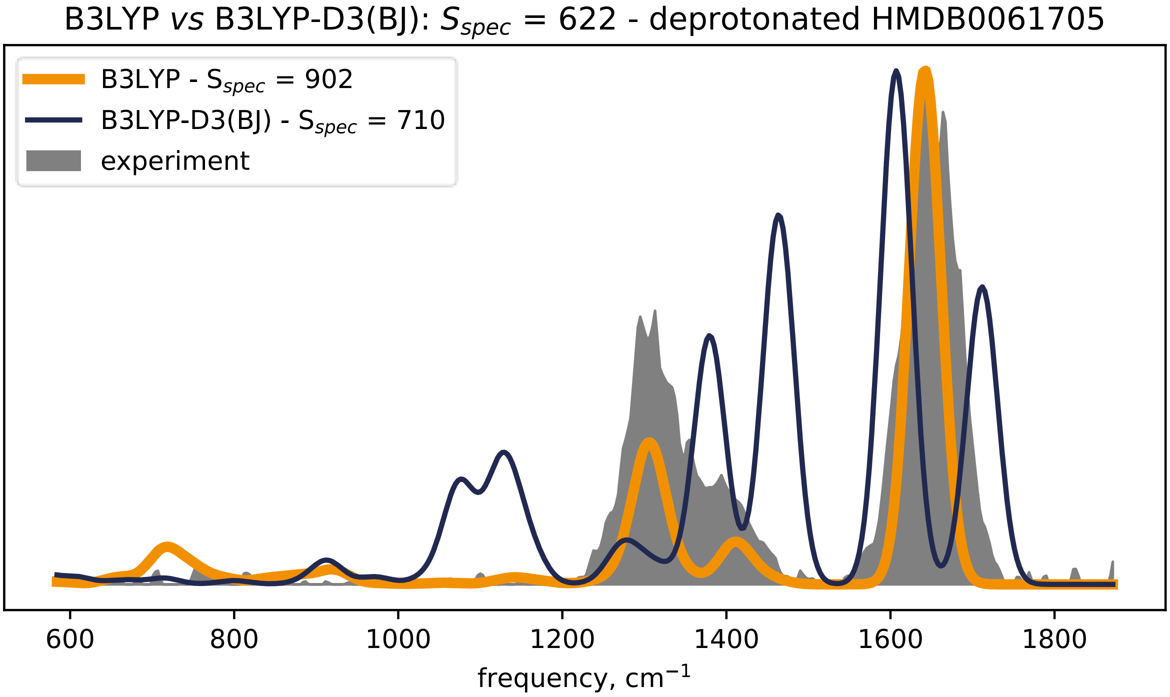
B3LYP vs B3LYP-D3(BJ): S_{spec} = 622 - deprotonated HMDB0061705
B3LYP - S_{spec} = 902
B3LYP-D3(BJ) - S_{spec} = 710
experiment
600
800
1000
1200
1400
1600
1800
frequency, cm^{-1}

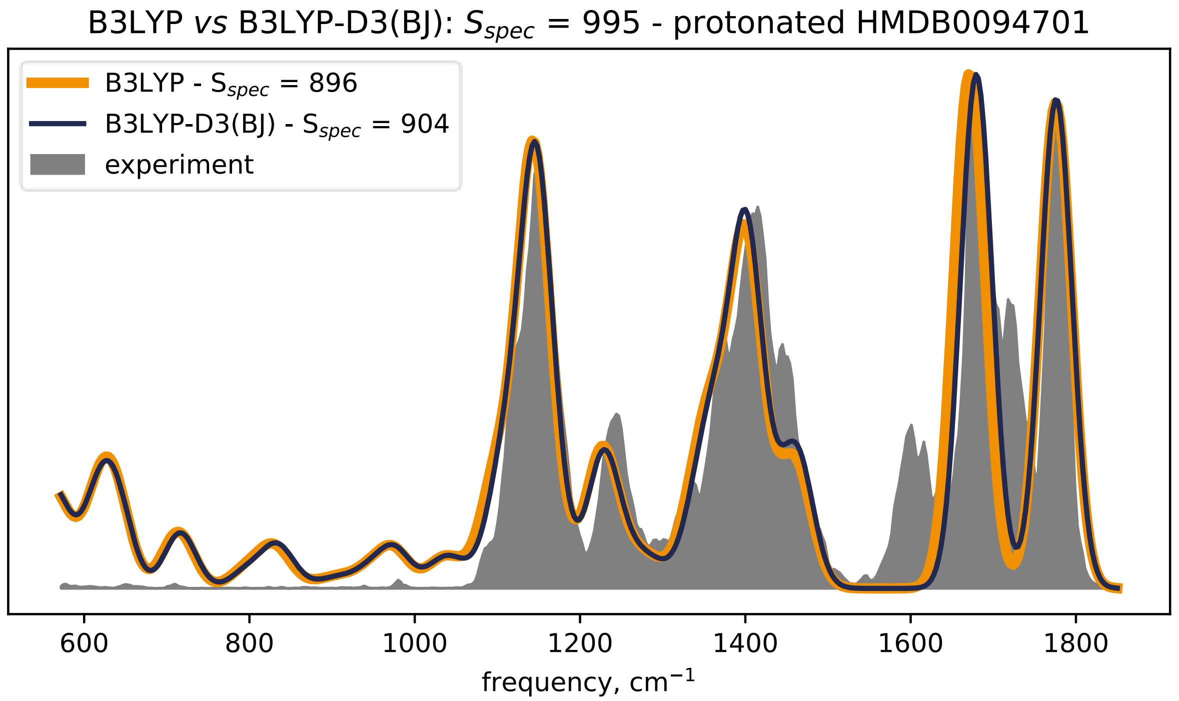

B3LYP *vs* B3LYP-D3(BJ): S_{spec} = 995 - protonated HMDB0094701
B3LYP - S_{spec} = 896
B3LYP-D3(BJ) - S_{spec} = 904
experiment
600
800
1000
1200
1400
1600
1800
frequency, cm^{-1}
